# A Modular Strategy for the Synthesis of Macrocycles and Medium-Sized Rings via Cyclization/Ring Expansion Cascade Reactions

**DOI:** 10.1021/jacs.4c00659

**Published:** 2024-02-19

**Authors:** Illya Zalessky, Jack M. Wootton, Jerry K. F. Tam, Dominic E. Spurling, William C. Glover-Humphreys, James R. Donald, Will E. Orukotan, Lee C. Duff, Ben J. Knapper, Adrian C. Whitwood, Theo F. N. Tanner, Afjal H. Miah, Jason M. Lynam, William P. Unsworth

**Affiliations:** † Department of Chemistry, University of York, York, YO10 5DD U.K.; ‡ GSK, Gunnels Wood Rd, Stevenage, SG1 2NY U.K.

## Abstract

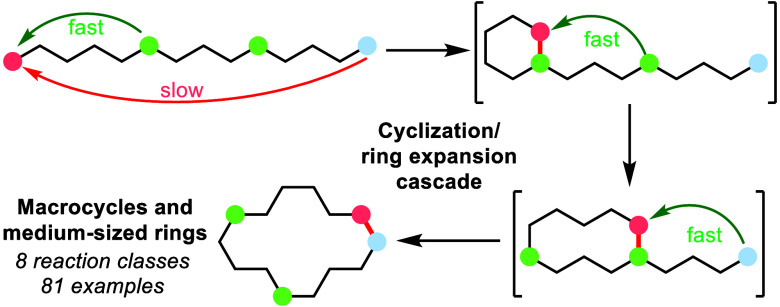

Macrocycles and medium-sized rings are important in many scientific fields and technologies but are hard to make using current methods, especially on a large scale. Outlined herein is a strategy by which functionalized macrocycles and medium-sized rings can be prepared using cyclization/ring expansion (CRE) cascade reactions, without resorting to high dilution conditions. CRE cascade reactions are designed to operate exclusively via kinetically favorable 5–7-membered ring cyclization steps; this means that the problems typically associated with classical end-to-end macrocyclization reactions are avoided. A modular synthetic approach has been developed to facilitate the simple assembly of the requisite linear precursors, which can then be converted into an extremely broad range of functionalized macrocycles and medium-sized rings using one of nine CRE protocols.

## Introduction

Macrocycles (12+ membered rings) are highly important in many scientific fields and technologies. Bioactive macrocycles are found widely in Nature and have vital applications in medicine, e.g., natural product drugs erythromycin (antibiotic) and rapamycin (immunosuppressant), alongside many others.^1^ Macrocycles are also widely used as ligands^2^ and sensors^3^ and have broad utility in self-assembly/supramolecular applications.^4^ Medium-sized rings (8–11-membered rings) are also important, most notably in medicinal chemistry, where they are considered to be privileged but under-explored scaffolds for exploration as new bioactive lead compounds.^5^

**Scheme 1:**
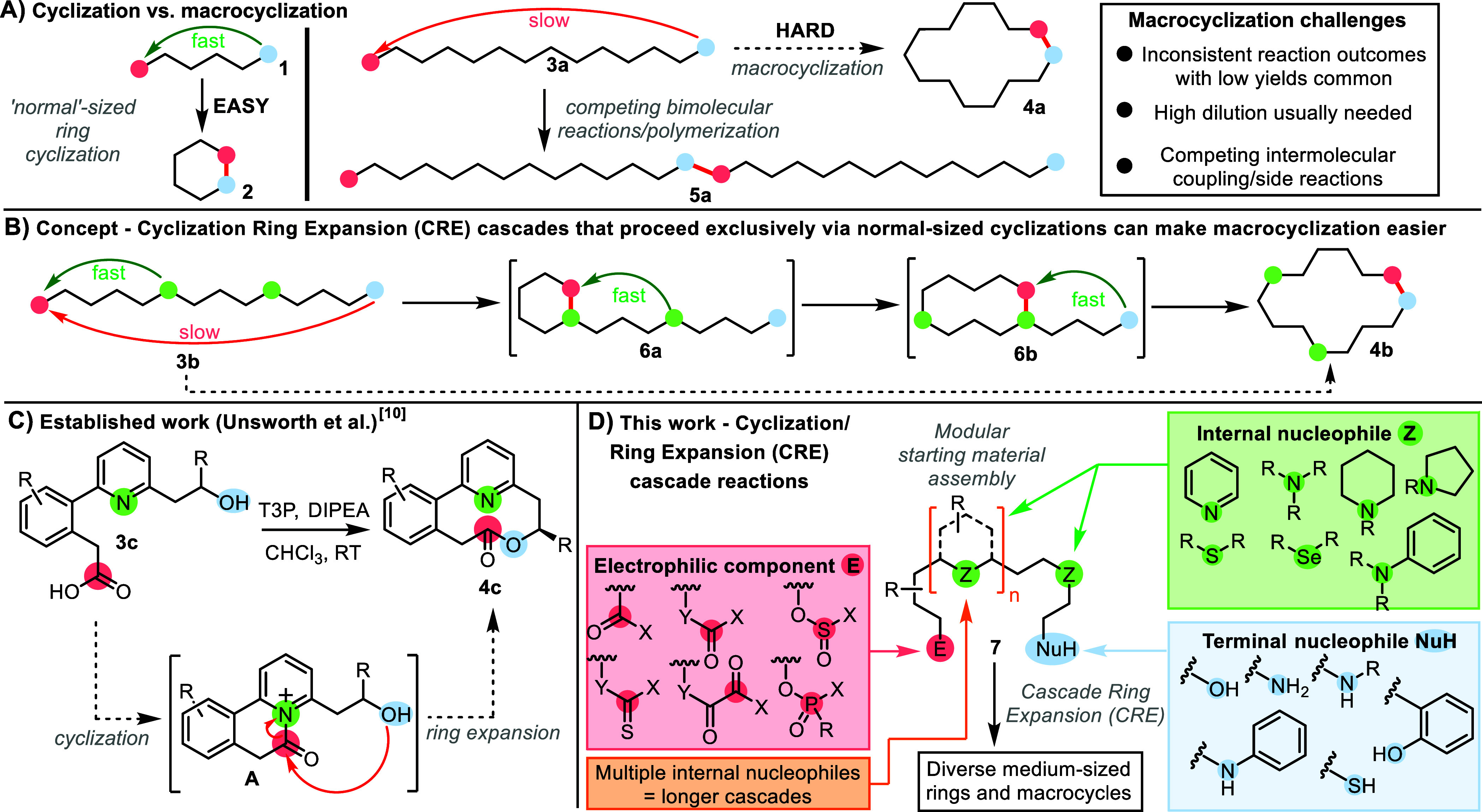
A) Cyclization vs Macrocyclization; B) The CRE Concept; C) Established Work; D) This work—CRE Cascade Reactions

The synthesis of macrocycles and medium-sized rings can be challenging, especially on large scales.^6^ This is largely a result of the well-known difficulty of achieving selective intramolecular coupling via end-to-end cyclization when preparing larger rings; while normal-sized ring (5–7-membered) cyclization reactions of the type **1** → **2** (Scheme 1A) are generally kinetically favorable, “easy” reactions, the analogous cyclization reactions to make larger ring products (**3a** → **4a**) are usually much more difficult, and are often plagued by competing intermolecular reactions (e.g., **3a** → **5a**, Scheme 1A).^7^ High-dilution (or pseudohigh-dilution) approaches are routinely used to limit intermolecular reactions, but rarely prevent them completely, and can negatively impact the practicality and scalability of the synthesis.^6^

This manuscript is focused on the development of a general, modular strategy for the synthesis of macrocycles and medium-sized rings that does not require high dilution conditions, using novel cyclization/ring expansion (CRE) cascade reactions.^8,9^ The concept is illustrated in Scheme 1B. A key design principle followed in all of the new methods described is to ensure that the cascade proceeds exclusively via low-energy “normal-sized” ring cyclic transition states. This is accomplished via the strategic placement of reactive groups within the linear precursor **3b** (depicted in green), to enable a cyclization/ring expansion cascade sequence (e.g., **3b** → **6a** → **6b** → **4b**, Scheme 1B). Thus, by breaking down a difficult, direct end-to-end cyclization of a large ring (i.e., as is required to convert **3a** → **4a**) into smaller, easier steps, a much more kinetically favorable overall cyclization can be facilitated.

This idea was exemplified by our published proof of concept study (Scheme 1C).^10^ In this work, we showed that pyridine-containing linear hydroxy acids of the type **3c** undergo efficient cyclization to form medium-sized ring lactones **4c**, with the pyridine moiety acting as an internal nucleophilic catalyst; following carboxylic acid activation, the reactions proceed via an initial cyclization to form an acylpyridinium reactive intermediate (**3c** → **A**) followed by ring expansion (**A** → **4c**) in situ. Importantly, these reactions do not require high dilution to proceed in a high yield (≈0.1 M concentration typical). This proof-of-concept study confirmed the viability of the CRE approach to synthesize medium ring lactones like **4c** from pyridine-containing hydroxy acids. In this paper, we show that this concept can be applied much more widely (Scheme 1D). Broad variation of the internal nucleophile (**Z**, box 1, green) and terminal nucleophile (**NuH**, box 2, blue) has been demonstrated, with various new CRE reaction systems established. Furthermore, a series of entirely new CRE reaction modes have also been developed by variation of the electrophilic component (**E**, box 3, pink). We also show that the inclusion of up to three internal nucleophilic components can be incorporated into the linear precursor to allow larger macrocyclic products to be prepared via longer cascades for the first time. Taken together, these four points of variation allow access to a remarkably broad array of product classes and chemistries, establishing the CRE approach as a versatile and general strategy for large ring synthesis.^11^

## Results and Discussion

**Scheme 2:**
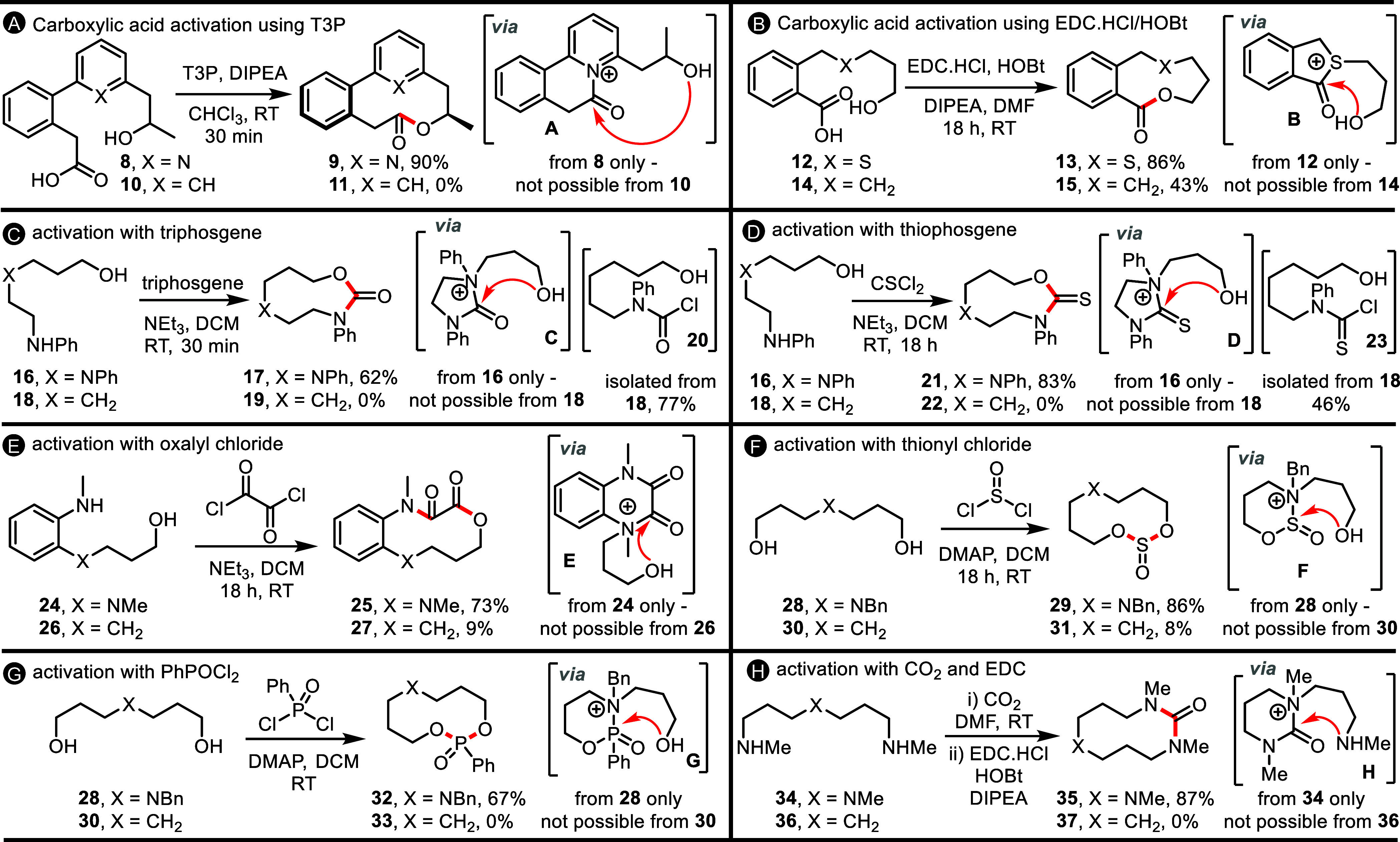
CRE Methods **A**–**H** and Their Control Reactions Full details of all synthetic protocols are included in the Supporting Information.

We started by seeking to establish a series of new CRE cascade reactions. When doing so, confirming that the internal nucleophile component participates in the cascade as designed is a key consideration. This was done using control reactions, as exemplified in Scheme 2A (CRE method **A**). In this CRE reaction, which featured in our proof-of-concept study,^10^ the conversion of hydroxy acid **8** into 10-membered lactone **9** proceeds in 90% isolated yield, affording the single atropisomer shown. The reaction is proposed to proceed via acylpyridinium intermediate **A**, and to support this notion, hydroxy acid **10**—a substrate analogous to **8** but lacking the key pyridine nitrogen group—was reacted under the same reaction conditions. In this control reaction, none of the analogous lactone **11** was obtained, thus confirming the importance of the pyridine nitrogen in enabling the cyclization of **8**, presumably via the CRE cascade proposed.^12^ Similar control reactions were performed for all of the newly discovered CRE reaction classes (CRE method **B**–**I**) reported herein.

First, we established that coupling reagents other than T3P can be used to activate carboxylic acids to promote CRE. The most effective alternative activation method tested was the combination of EDC.HCl and HOBt (CRE method **B**, Scheme 2B). Several examples of the successful use of these conditions feature throughout the manuscript (vide infra), with one of the more unusual cases included in Scheme 2B. In this reaction, sulfide-containing hydroxy acid **12** was reacted with EDC.HCl, HOBt, and NEt(*i*-Pr)_2_ for 18 h at RT in DMF, resulting in the formation of 9-membered ring lactone **13** in 86% yield. The reaction is proposed to proceed via a reactive acyl sulfonium cation **B**.^13^ To support this, substrate **14** (which lacks the internal S atom present in **12**) was prepared and reacted under the same conditions. In this control reaction, the analogous cyclized product (**15**) was formed, but notably, the reaction was slower (see Supporting Information) and proceeded in lower yield (43%). This result suggests that in this case, direct end-to-end cyclization is viable and may compete with the CRE pathway in the formation of sulfide-containing lactone **13**. Nonetheless, the improvement in yield when the sulfur atom is included is clear, suggesting that CRE via intermediate **B** is still likely to be the major pathway. Additional experiments supporting the intermediacy of an acyl sulfonium cation (detection via NMR and MS experiments) are described in the Supporting Information.

We then examined more fundamentally different CRE reaction modes (**C**–**H**). The idea in these variants was that rather than starting with a carboxylic acid derivative, similar cascade reactivity could be accessed by reacting bis-nucleophilic substrates (amines and alcohols) with bis-electrophile reagents; for example, the reaction of amino alcohol **16** with triphosgene (CRE method **C**, Scheme 2C). In this case, the CRE cascade is thought to proceed via initial reaction between triphosgene and the terminal aniline group of **16**, followed by cyclization to form cationic intermediate **C**. Reactive intermediate **C** is then set up to undergo facile ring expansion to form cyclic carbamate product **17**, which was isolated in 62% yield. In contrast, none of the analogous carbamate **19** was obtained when control substrate **18** was tested; the only tractable product was carbamoyl chloride **20**, thus supporting our proposed CRE mechanism. A similar result was obtained using thiophosgene as the reagent; thiocarbamate **21** was obtained in 83% yield from **16**, likely via intermediate **D**, while none of **22** was produced in the control reaction (CRE method **D**, Scheme 2D). CRE methods based on the use of other readily available bis-electrophilic reagents—oxalyl chloride, thionyl chloride, and phenylphosphonic dichloride—were also developed (CRE methods **E**–**G**, Scheme 2E–G). In these cases, the expected medium-sized ring products **25**, **29**, and **32** were obtained from amino alcohol or diol starting materials in good yields, while the respective control reactions afforded very low yields or none of the analogous products (**27**, **31**, and **33**, 0–9%).

Finally, a CRE method was developed based on the activation of carbamic acids formed in situ from the reaction of amines and CO_2_ (CRE method **H**, Scheme 2H). In this reaction, CO_2_ (from dry ice) was bubbled through a solution of triamine **34**, and then activated using a combination of EDC.HCl and HOBt to afford 10-membered ring cyclic urea **35** in 87% yield.^14^ None of the analogous product **37** was obtained in the control reaction from diamine **36**. This method represents a less hazardous alternative to the CRE method **C**, avoiding the use of toxic triphosgene.

**Figure 1:**
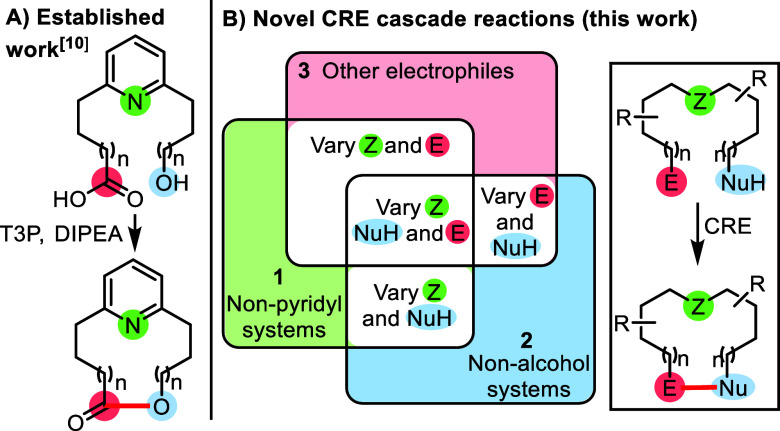
Key to the novel CRE reactions presented in Scheme 3.

**Scheme 3:**
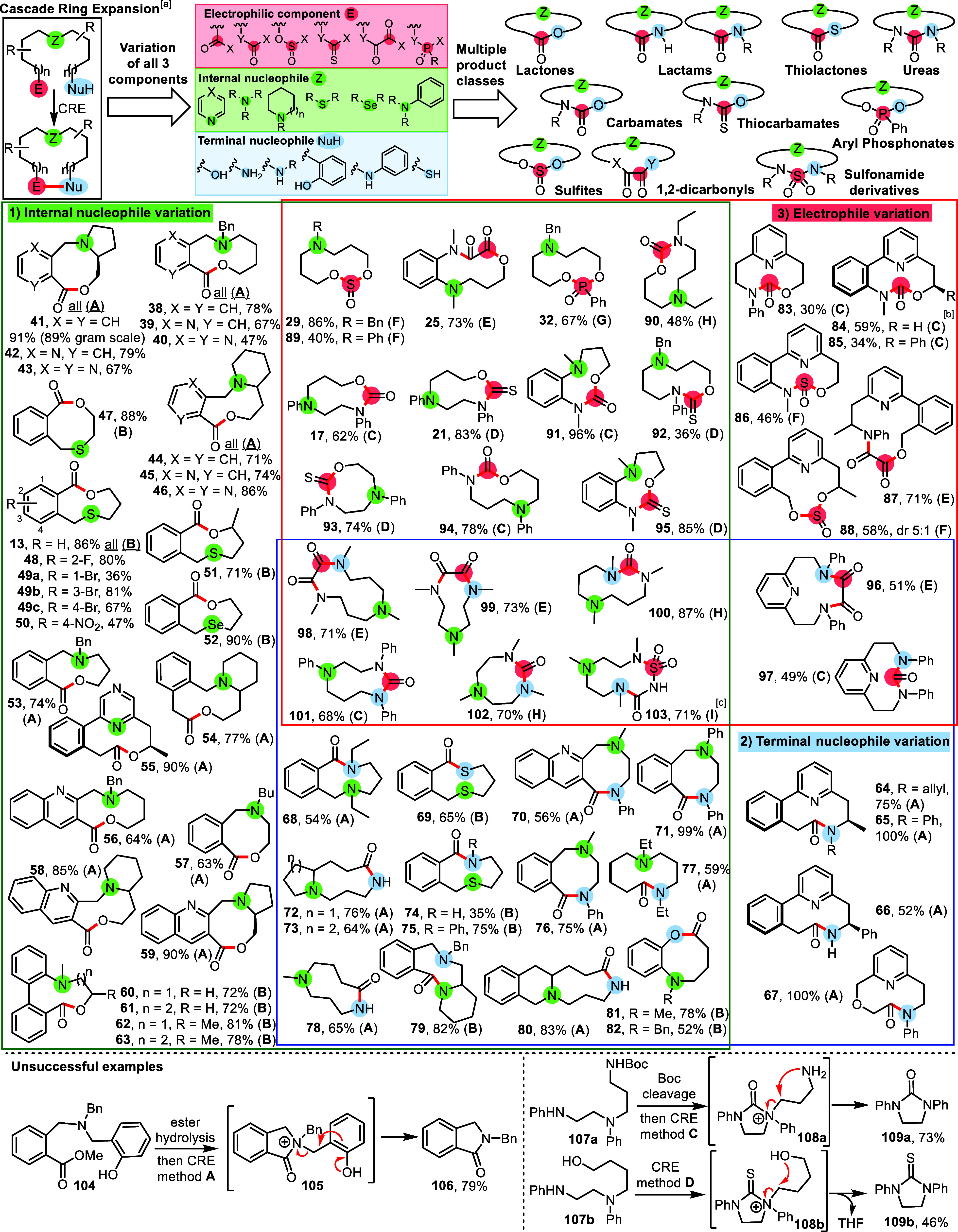
Cyclization/Ring Expansion Cascade Reactions Using Substrates with One Internal Nucleophile: Scope of CRE for Medium-Sized Ring Synthesis CRE methods **A**–**H** are summarized in Scheme 2, with full synthetic details for all reactions included in the Supporting Information. NMR yield measured by comparison to an internal standard as **85** is unstable during chromatography. Conditions for CRE method **I**: Chlorosulfonyl isocyanate (1.2 equiv), NEt_3_ (3.0 equiv), DCM, RT, 18 h.

With eight general CRE reaction types established (CRE methods **A**–**H**, Scheme 2), we turned our attention to exploring their scope (Figure 1 and Scheme 3). The products are arranged within Scheme 3 to highlight the three major points of variation compared with the established CRE of pyridine-containing hydroxy acids (Figure 1A).^10^ Medium-sized ring products prepared from linear starting materials in which the pyridine internal nucleophile has been replaced by other saturated nucleophiles are shown in box 1 (green). Box 2 (blue) features products made by replacing the aliphatic alcohol with other terminal nucleophiles. Box 3 (pink) is focused on variation of the electrophilic component, using CRE methods **C**–**I**. The boxes are arranged in a Venn diagram layout, such that their intersections show products made by varying any two or all three components. In all cases, the newly formed bond(s) are highlighted in red.

A wide range of functionalized lactones **38**–**63** (Scheme 3, box 1, green) were prepared from hydroxy acids using CRE methods **A** and **B** by varying the internal nucleophile highlighted in green.^15^ In these examples, the use of saturated internal nucleophiles (nucleophiles with no bonds to H, e.g., 3° amines) is a key design feature, as it is important that the positively charged reactive intermediate formed following the initial cyclization cannot be neutralized by deprotonation. Wide variation of the internal nucleophile component has been demonstrated, with successful examples including diazines (e.g., **55**), aliphatic tertiary amines (e.g., **38**–**40**, **53**, **57**), cyclic amines (e.g., **41**–**46**, **54**, **58**),^16^ anilines (e.g., **60**–**63**), sulfides (e.g., **47**–**51**), and selenides (**52**). Gram-scale reaction (e.g., **41**) can also be performed with minimal impact of the reaction yield.

CRE method **A** also works well for the synthesis of pyridine-containing lactams (e.g., **64**–**67**, Scheme 3, box 2, blue),^17^ with these substrates formed via the CRE of protecting group-free pyridine containing amino acid derivatives. In the box 1 and 2 intersection, various products are shown in which both the internal and terminal nucleophilic components were varied; these products include various substituted and unsubstituted lactams (e.g., **68**, **70**–**75**), lactones formed from phenols (**81** and **82**), and thiolactone **69**,^18^ with broad variation of the internal nucleophile also demonstrated across this series.

Products obtained by varying the electrophilic component using CRE methods feature **C**–**H** in box 3 (pink). Pyridine-containing products **83**–**88** were produced using CRE methods **C**, **E**, and **F**, including medium-sized cyclic carbamate, sulfite, and 1,2-dicarbonyl derivatives.^19^ The yields were comparatively low in these cases, with these products found to be unstable during column chromatography (e.g., **85**). In general, in this series, superior yields were obtained when switching to nonpyridine examples (e.g., **89**–**95**, box 1 and 3 intersection), where the products made were stable. This section includes products prepared via all of the new CRE methods **C–H**.^20^

The central box features medium-sized ring lactam, urea, and sulfonamide products (**98**–**103**) in which all three components have been varied compared with the archetypical example **3c**, highlighting well how replacing any/all of the components can give rise to diverse product classes. Included with this section is a product prepared via a ninth CRE method, with sulfonamide derivative **103** formed in good yield from the reaction of a methylated triamine starting material with chlorosulfonyl isocyanate (CRE method **I**). As for CRE method **A**–**H**, a control reaction was done for **103**, which confirmed that the analogous product did not form when the internal nucleophile was absent (see Supporting Information). Notably, many of the products produced were isolated as crystalline solids, and in total, X-ray crystallographic data was obtained for 18 of the CRE products synthesized in this manuscript.^21^

The ability to vary all three components interchangeably is central to the CRE concept. In total, 65 diverse medium-sized ring products are featured in Scheme 3. Most were formed in good to excellent yield, with the reactions carried out at a standard concentration (typically ≈0.1 M).^22^ Within these scoping studies, 9 distinct CRE reaction classes in total were examined, and products spanning the full medium-sized ring range (8–12 membered rings) were prepared. Ring closure via the construction of 10 different functional groups has been demonstrated. Wide variation of the internal nucleophile has also been shown as well as explorations of various substituents, annulation with aromatic/aza aromatic rings, and atroposelective examples. Our goal when selecting substrates for this scoping study was to demonstrate the versatility of the CRE approach, and to ensure that all new examples ask a genuine question of the methods.^23^ However, it is notable that with the ability to vary all three major reaction components so widely, the examples chosen still represent only a small fraction of the potential scope of the CRE method.

Three instructive unsuccessful examples are listed at the bottom of Scheme 3. In all cases, the CRE cascade failed, likely due to there being an alternative lower energy pathway by which the positively charged reactive intermediate can be quenched. For example, following ester hydrolysis (from **104**) and carboxylic acid activation using CRE method **A**, it is thought that cationic intermediate **105** formed in the expected manner. But then, rather than undergo ring expansion, **105** is able to fragment to form lactam **106** as shown, enabled by the *ortho* phenol group being conjugated to the nitrogen cation. Similarly, when substrates **107a/b** were reacted using CRE method **C/D**, cationic intermediates (**108a/b**) likely formed as planned, but rearrange to form ureas **109a/b** via intramolecular nucleophilic substitution reactions as shown. Despite these reactions not delivering the desired products, these results do provide additional support for the proposed CRE mechanisms; i.e., the isolation of 5-membered ring products **106** and **109a/b** provides indirect evidence that the proposed 5-membered cationic intermediates did form.

**Scheme 4:**
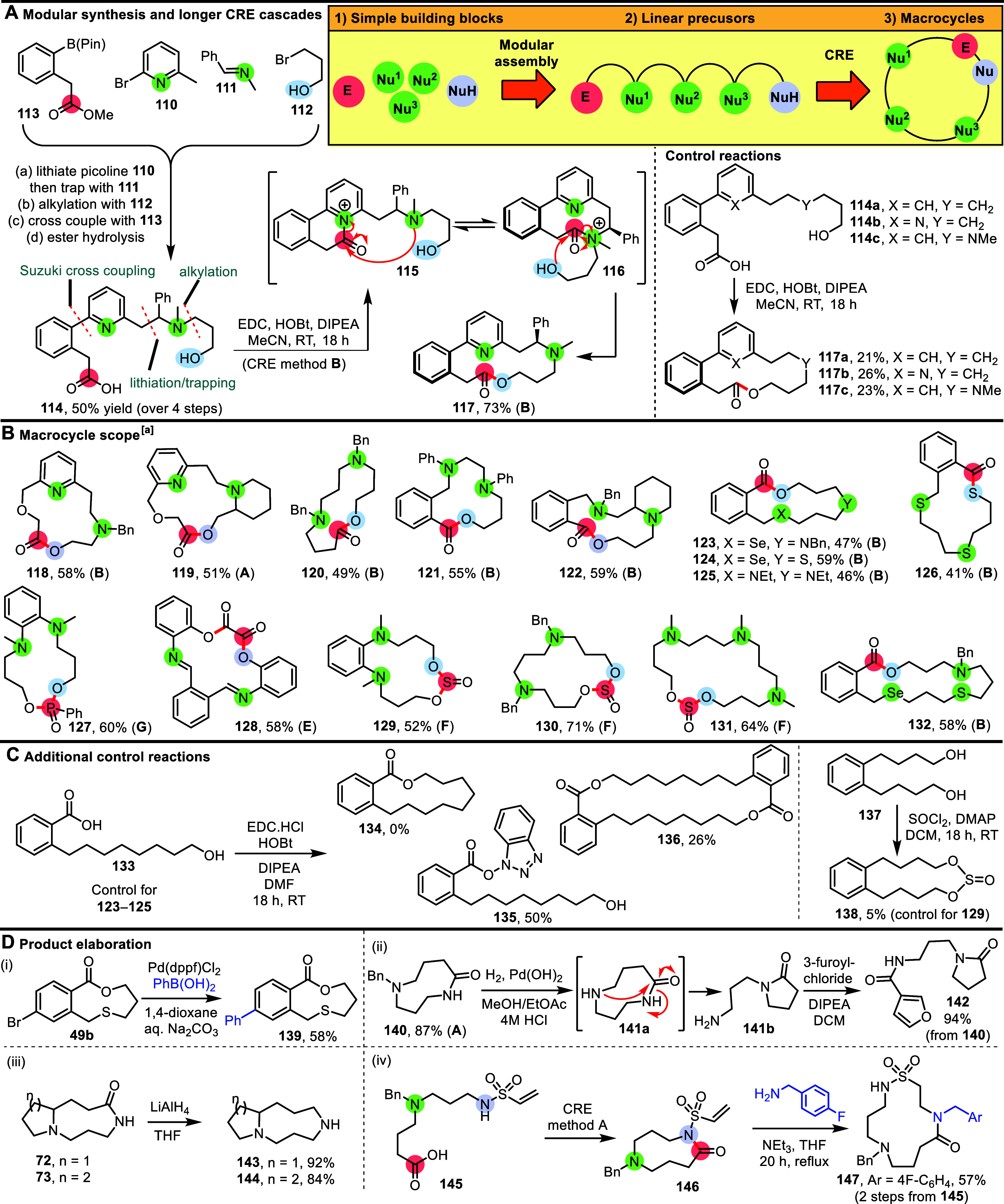
Cyclization/Ring Expansion Cascade Reactions Using Substrates with Two or Three Internal Nucleophiles: Scope of CRE for Macrocycle Synthesis Conditions for CRE methods **A**–**B** and **E**–**G** are summarized in Scheme 2, with full synthetic details for all individual reactions included in the Supporting Information.

The medium-sized ring products included in Scheme 3 were all prepared from linear starting materials containing a single internal nucleophile. However, such substrates are not suitable for the synthesis of macrocyclic products, if we want to ensure that the cascades proceed exclusively via kinetically favorable 5–7-membered cyclic transition states as designed. Thus, to enable larger macrocyclic ring systems to be prepared via CRE, a further extension to the approach is required and is summarized in Scheme 4. The idea is that through the incorporation of more than one internal nucleophile, longer cascade processes can be performed. This was validated by the CRE of linear starting material **114**; in this case, activation of the carboxylic acid (via CRE method **B**) is thought to initiate cyclization (**114** → **115**) and two successive ring expansion reactions (**115** → **116** → **117**), to furnish 14-membered ring lactone **117**, which was isolated in 73% yield. Of additional note, macrocycle **117** was obtained as the single atropisomer shown, with the stereoselectivity thought to be controlled by the benzyl stereogenic center, via point-to-axial chirality transfer.^10,24^ The structure and assigned relative stereochemistry of **117** is supported by X-ray crystallographic data.^21^

To support the proposed CRE mechanism and confirm the importance of both internal nucleophilic components (highlighted in green), three separate control substrates **114a**–**c** were synthesized and tested under the same conditions as those used to prepare **117**. Control substrates **114a**–**c** lack either one or both of the pyridine and tertiary amine groups present in **114**. When each of compounds **114a**–**c** were reacted using the standard CRE method **B** conditions, all were converted into the corresponding macrocyclic lactones **117a**–**c**, but in low yields (21–26%). These results confirm that direct end-to-end macrocyclization to make 14-membered lactones of the type **117** is possible but is relatively inefficient under the conditions tested. Therefore, it is likely that direct end-to-end macrocyclization is only a minor background process during the synthesis of **117**, with the proposed CRE mechanism likely to be the dominant pathway; the approximately 3-fold increase in the yield of **117** compared to any of **117a**–**c** well highlights the improvement afforded by the CRE approach.

The straightforward manner in which starting material **114** was prepared is also noteworthy. Using four simple molecular building blocks **110**–**113**, linear precursor **114** was synthesized in 50% overall yield, using 4 routine synthetic transformations [(i) lithiation of picoline **110**/trapping with imine **111**; (ii) alkylation with **112**; (iii) Suzuki coupling with **113**; (iv) hydrolysis]. Indeed, similar building block approaches were used to assemble most of the starting materials used in this study (see Supporting Information), with the internal nucleophiles often serving as convenient synthetic handles to facilitate the construction of the requisite starting materials. Thus, there is a clear, practical pathway to convert simple molecular building blocks into linear precursors and then into macrocycles (Scheme 4 box); this is important for researchers seeking to use the reported methods or design their own CRE systems.

Substrate scoping studies for starting materials including two or three internal nucleophiles are summarized in Scheme 4B. Macrocyclic lactones **118**–**125** were each prepared using CRE methods **A** and **B** from long-chain carboxylic derivatives, with examples including pyridine, tertiary amine, aniline, sulfide, and selenide internal nucleophiles all demonstrated. The synthesis of macrocyclic thioester **126** is particularly noteworthy, with the CRE based on all sulfur-based nucleophiles. Longer CRE cascade reactions can also be performed that do not rely on carboxylic acid activation, with macrocycles **127**–**131** prepared by using CRE methods **E**–**G**. The last of these (**131**) includes three internal nucleophiles, thus extending the cascade by the addition of another cyclization/ring expansion sequence. The successful synthesis of macrocyclic lactone **132** is especially noteworthy, as it uses four different nucleophilic groups in total in the cascade; this allowed the synthesis of the usual 17-membered ring lactone **132**, in which 4 different heteroatoms have been incorporated.

Further control reactions were performed for these longer chain systems (Scheme 4C). For example, lactones **123**–**125** were prepared in 47–59% yield from linear hydroxy acids, containing combinations of tertiary amine, sulfide, or selenide groups in the linear chain, but when hydroxy acid **133** (which has the same chain length but lacks any of the internal nucleophiles) was reacted under the same conditions, none of the analogous macrocyclic lactone **134** was formed. Instead, HOBt adduct **135** was isolated (indicative of a reduced reaction rate), alongside dimer **136** (indicative of intermolecular coupling). Similarly, while macrocyclic sulfite **129** was isolated in 52% yield, diol **137** (which lacks the internal amine groups) was converted into macrocycle **138** in just a 5% yield under the same conditions.

Finally, the medium-sized ring and macrocyclic products prepared throughout this paper have the potential to be elaborated by additional synthetic transformations (Scheme 4D). Many of the substrates prepared in this paper feature aromatic groups on which it should be simple to incorporate groups to enable cross coupling reactions; as a simple demonstration, lactone **49b** was shown to undergo Suzuki–Miyuara cross coupling with phenyl boronic acid to afford **139** in 58% unoptimized yield (Scheme 4D(i)). Cleavage of *N*-benzyl substituents also has the potential to generate a reactive handle to enable the derivatization of the CRE products. However, the hydrogenolysis of CRE product **140** highlights how the potential for ring contraction reactions can be an unwanted complication (Scheme 4D(ii)); in this case, following hydrogenolysis under acidic conditions, spontaneous ring contraction to a γ-lactam occurred, presumably under thermodynamic control. The resulting amine **141** was subsequently acylated to facilitate isolation of the polar product, with amide **142** isolated in 94% overall yield in 2 steps from **140**. However, medium-sized ring secondary amines are readily accessible via reductive methods; for example, medium-sized ring lactams **72** and **73** were each reduced by LiAlH_4_, to form cyclic secondary amines **143** and **144** in high yield (Scheme 4D(iii)).

It is also possible to take products prepared via CRE and expand the rings further using another class of ring expansion reactions. This is exemplified by the overall conversion of carboxylic acid **145** into macrocycle **147**. This sequence started with the CRE of acid **145** to form 9-memebered ring product **146**, with a sulfonamide nitrogen acting as the terminal nucleophile in this CRE example. The unpurified product **146** was then used directly in a different type of ring expansion reaction, using our published conjugate addition/ring expansion method;^9d^ thus, the reaction of intermediate **146** with 4-fluorobenzylamine led to its smooth conversion into macrocycle **147** in good overall yield from **145**.

## Conclusions

In summary, a series of CRE cascade reactions have been developed for the synthesis of a wide range of functionalized medium-sized rings and macrocyclic products. By operating solely via kinetically favorable 5–7-membered ring cyclization steps and in situ ring expansion, direct end-to-end cyclization is avoided, meaning that the CRE reactions generally proceed in good yield without the use of high-dilution conditions. Very broad scope has been demonstrated for variation of the electrophilic component, the internal nucleophile, and the terminal nucleophile. The proposed CRE cascade mechanism is supported by control reactions in numerous cases, which consistently show the importance of the internal nucleophiles in mediating an efficient overall cyclization. The internal nucleophile groups also provide convenient synthetic handles that allow the requisite linear starting materials to be prepared with relative ease via a modular approach. The practicality and versatility of the series of CRE methods introduced in the manuscript are expected to be of high value in a myriad of scientific fields and technologies that rely on the design and synthesis of functionalized large-ring systems.^1−4^ The modular nature of the methods is expected to be useful when generating libraries to optimize the properties of the large-ring products, and the inherent scalability^25^ of the methods should also be important if translating the methods for use in larger scale or industrial settings.

## Supporting Information Available

Synthetic protocols and characterization data (NMR, IR, MS, R_f_, melting point) for all materials produced in this manuscript. Additional mechanistic studies to support the intermediacy of **B**, X-ray crystallographic information and copies of ^1^H and ^13^C NMR spectra. (PDF)

## ABBREVIATIONS

CRE: Cyclization/ring expansion
DIPEA: N,N-Diisopropylethylamine
T3P: Propylphosphonic anhydride
EDC: 1-Ethyl-3-(3-(dimethylamino)propyl)carbodiimide
HOBt: Hydroxy benzotriazole
DCM: Dichloromethane
DMF: Dimethylformamide
DMAP: 4-Dimethylaminopyridine
Boc: *tert*-Butyloxycarbonyl
