# Supplementary material for: A Modular Strategy for the Synthesis of Macrocycles and Medium-Sized Rings via Cyclization/Ring Expansion Cascade Reactions
Source: J Am Chem Soc. 2024 Feb 19;146(8):5702–11. doi: 10.1021/jacs.4c00659 (PMC10910531; doi:10.1021/jacs.4c00659)
Supplement: Supplementary file 1 — ja4c00659_si_001.pdf [file ja4c00659_si_001.pdf]

Supporting Information

A modular strategy for the synthesis of macrocycles and medium-sized rings via cyclisation/ring expansion cascade reactions.

Illya Zalessky[a], Jack M. Wootton[a], Jerry K. F. Tam[a], Dominic E. Spurling[a#], William C. Glover-Humphreys[a], James R. Donald[a$], Will E. Orukotan[a], Lee C. Duff[a], Ben J. Knapper, Adrian C. Whitwood[a], Theo F. N. Tanner[a], Afjal H. Miah[b], Jason M. Lynam[a] and William P. Unsworth[a]*

AUTHOR ADDRESSES

[a] Department of Chemistry, University of York, York, YO10 5DD (UK).

[b] GSK, Gunnels Wood Rd, Stevenage, SG1 2NY (UK)

PRESENT ADDRESSES

[#]Mr Dominic E. Spurling: School of Chemistry, University of St Andrews, North Haugh, St Andrews, KY16 9ST (UK)

[$]Dr James R. Donald: Pharmenable Therapeutics, Compass House, Vision Park, Histon, Cambridge, CB24 9AD (UK)

Table of Contents

	Page
1) General information	3–5
2) General procedures	6–10
3) Compound characterization data and procedures	11–270
4) Evidence for formation of sulfonium cation intermediates	271–274
5) Computational Chemistry	275–277
6) X-ray structures	278–295
7) ^{1}H and ^{13}C NMR spectra	296–591
8) References	592–594

1) General Information

Unless otherwise stated, all reactions were carried out at RT under an inert (N_2 or Ar) atmosphere in oven-dried glassware. Except where stated all reagents were purchased from commercial sources: Merck (Sigma Aldrich), Alfa Aesar, Acros Organics, Fisher Chemicals, VWR, TCI, Across chemicals and Fluorochem and were used without further purification. Anhydrous CH_2Cl_2, toluene, MeCN, Et_2O and DMF were obtained from an Innovative Technology Inc. PureSolv® solvent purification system. Dry THF was obtained from the SPS laboratory system and used immediately after being dispensed. Dry Et_3N and DIPEA obtained by drying with CaH_2 and then distilling and storing over KOH or 3 Å molecular sieves, 3 Å under Ar. Anhydrous MeOH, DMSO, acetone, ^{t}BuOH, benzene, CCl_4 and ^{n}BuOH was purchased from Sigma Aldrich and used as supplied.

^{1}H NMR spectra were recorded at 400 MHz on Bruker AV400 or Bruker AMX 400/JEOL ECS-400 and at 500 MHz on Bruker DRX500 MHz Ultra ShieldTM spectrometry.^{13}C NMR spectra were recorded at 101 MHz on Bruker AV 400 or Bruker AMX 400 MHz Ultra ShiledTM and 126 MHz on Bruker DRX500 MHz Ultra ShiledTM spectrometry.^{19}F NMR spectra were recorded at 376 MHz on Bruker AV400 or Bruker AMX 400/JEOL ECS-400 spectrometry. ^{31}P NMR spectra were recorded at 162 MHz on Bruker AV400 or Bruker AMX 400/JEOL ECS-400 spectrometry.

All spectroscopic data was acquired at 295 K (25 °C) unless stated otherwise and samples were dissolved in $CDCl_3$ unless specified otherwise.Chemical shifts (δ) are reported in parts per million (ppm), with residual solvent peaks: $CDCl_3$: δ_H = 7.26, $CDCl_3$: δ_C = 77.0, $(CD_3)_2SO$: δ_H = 2.50, δ_C = 39.5, CD_3OD: δ_H = 3.31, δ_C = 49.0, C_6D_6: δ_H = 7.16, δ_C = 128.1, CD_2Cl_2: δ_H = 5.32, δ_C = 53.8, $(CD_3)_2CO$: δ_H = 2.05, δ_C = 206.3, D_2O: δ_H = 4.79, $DCON(CD_3)_2$: δ_H = 8.03, δ_C = 163.2, being used for internal reference. The multiplicity abbreviations used are: s, singlet; d, doublet; t, triplet; q, quartet; p, pentet; m, multiplet; dd, doublet of doublets; dt doublet of triplets; td, triplet of doublets; tt, triplet of triplets; ddd, doublet of doublets of doublets; pd, pentet of doublets; where br indicates a broad signal, and app. indicates an apparent. ^{1}H experiments are reported as: chemical shift in ppm, quoted to the nearest 0.01 ppm, (integration, multiplicity, coupling constant and assignment (where possible)). ^{13}C experiments are reported as: chemical shift in ppm, quoted to the nearest 0.1 ppm, (carbon assignment

(where possible) or multiplicity, coupling constant and assignment (where applicable)).^{19}F experiments are reported as: chemical shift in ppm, quoted to the nearest 0.1 ppm, (multiplicity, coupling constant and assignment (where possible)). ^{31}P experiments are reported as: chemical shift in ppm, quoted to the nearest 0.1 ppm, (multiplicity, and assignment (where possible)).

Assignment of compounds was achieved through use of ^{135}DEPT, COSY, HSQC and HMBC experiments.Spectra were analysed using MestReNova 12.0.3-21384 software and values of coupling constant (*J*) are reported in Hertz (Hz) to the nearest 1 decimal place, i.e., 0.1 Hz. The term "overlapping" is used to describe resonance peak, which is behind another resonance peak, *i.e.*, compound resonance behind the solvent peak or combination of two resonance peaks. The systematic chemical names were generated using the IUPAC name generator tool option is included within the ChemBioFDaw Ultra 19.1 software.

Infrared (IR) spectra were recorded on a PerkinElmer UATR 2 or Pekin Elmer Spectrum 100 spectrometer fitted with a universal Attenuated Total Reflectance (ATR) accessory; date was recorded as a thin film dispersed from either CH_2Cl_2 or $CDCl_3$, neat or solid state by ATR-FTIR. IR-recorded experiments are reported as: IR (method of recorded) ν_{max} (IR absorption maxima) / unit (cm^{-1}) chemical absorption (assignment (where possible)). The intensity of each absorbance bands gives the annotated appearance, and each bond was described as w (weak), m (medium), s (strong), sh (sharp) and with the prefix v (very) and suffix br (broad).

High Resolution Mass Spectra (HRMS) were obtained by the University of York Mass Spectrometry Service, recorded on a Waters XEVO G2-XS TOF, Waters Synapt G2S TOF or Bruker Micro-TOF mass spectrometer, with HRMS mode incorporating a lock-in mass into the mobile phase (leucine enkehalin) or on a Bruker Daltonics, Micro-TOF spectrometer, using Electrospray Ionisation (ESI) or Atmospheric Pressure Chemical Ionisation (APCI), positive or negative generative modes.

Thin Layer Chromatography (TLC) was carried out on Merck silica gel $60F_{254}$ pre-coated aluminium foil sheets and was visualised using UV light (λ = 254 nm, short wavelength) or UV light (λ = 366 nm, long wavelength) and stained with basic aqueous potassium permanganate ($KMnO_4$), ninhydrin or vanillin solution dip. Concentration under reduced pressure or *vacuo*

was performed using a Büchi® Rotavapor® R-210 evaporator with jack and water bath, 29/32 joint, 240V rotary evaporator using a mixture of acetone and dry ice or ice/water as the coolant. Flash column chromatography was conducted using Aldrich technical grade silica gel (SiO_2), 60 Å, 230-400 mesh, 40-63 µm particle size, under a light positive pressure of air, eluting with the specified solvent system.

Melting points were recorded as decomposition temperature range and measured on a Stuart SMP10 or Gallenkamp apparatus using open tubes with no corrections. Before measuring the melting point, in most instances, the solids were purified by recrystallisation after purification by column chromatography, where "(from [solvent])" donating solvent systems were used, e.g. single or multiple.

X-ray crystallography data was collected, solved, and refined by Dr Adrian C. Whitwood in the School of Chemistry at the University of York. Diffraction data were collected at 100 K on an Oxford Diffraction SuperNova diffractometer with Cu-K_α radiation (λ = 1.54184 Å) using a HyPix-6000HE detector. The crystal was cooled with an Oxford Instruments Cryojet. Diffractometer control, data collection, initial unit cell determination, frame integration and unit-cell refinement were carried out with CrysAlisPro, Face-indexed absorption corrections were applied using spherical harmonics, implemented in SCALE3 ABSPACK scaling algorithm within CrysAlisPro. OLEX2 was used for overall structure solution, refinement and preparation of computer graphics and publication data. Within OLEX2, the algorithms used for structure solution were 'Superflip charge-flipping smtbx-flip charge-flipping ShelXT dual-spaceRefinement by full-matrix least-squares used the SHELXL algorithm within OLEX2. All non-hydrogen atoms were refined anisotropically. Hydrogen atoms were placed using a riding model and included in the refinement at calculated positions. CrystalMaker® 10 software was also used to visualise the X-ray structures with their corresponding CCDC deposit number shown.

2) General procedures

To account for minor variations made to procedures/ purification on a case-by-case basis (*e.g.* minor changes to reactions time, concentrations, work-up quantities/solvents etc.), separate methods are reported for all of the individual compounds in manuscript (see Section 3). However, for convenience, a standard general procedure for CRE methods **A**–**H** are described below, that will serve as a good starting point if attempting novel examples.

General CRE method A

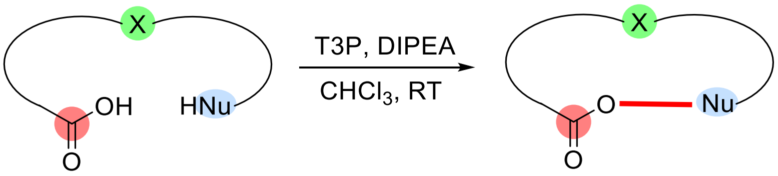

The carboxylic acid starting material (1 mmol) was dissolved in the reaction solvent (chloroform typically, although DMF and acetonitrile can also be used, 10 mL) and DIPEA (1.85 mmol) was added followed by T3P (50% w/v in ethyl acetate, 1.5 mmol) and stirred at room temperature for 1 hours under argon. Water (30 mL) was then added, and the reaction mixture extracted with dichloromethane (3 × 30 mL). The combined organic phases were then dried over $MgSO_4$, filtered, concentrated under vacuum and purified by flash column chromatography. The same method can also be performed using Li carboxylate salts rather than carboxylic acids as the starting material, and if the starting material to be used is an HCl salt, an extra equivalent of DIPEA should be added.

General CRE method B

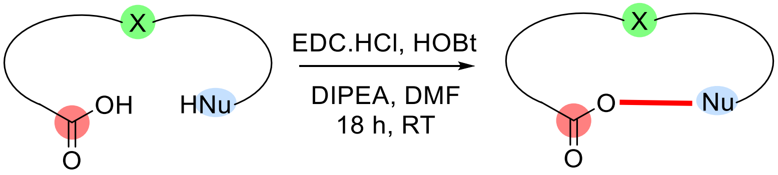

EDC.HCl (1.50 mmol) was added to solution of carboxylic acid (1.00 mmol), HOBt (188 mg, 1.40 mmol) and dry DIPEA (5.00 mmol) in anhydrous DMF (10 mL). The reaction was stirred at RT, for 18 h, then diluted with EtOAc (20 mL), transferred to separating funnel and the organic phase was washed sequentially with H_2O (3 × 20 mL) and sat. brine (2 × 20 mL). The organic phase was dried over $MgSO_4$, filtered and concentrated under reduced pressure and

purified by flash column chromatography. The same method can also be performed using Li carboxylate salts rather than carboxylic acids as the starting material, and if the starting material to be used is an HCl salt, an extra equivalent of DIPEA should be added.

General CRE method C

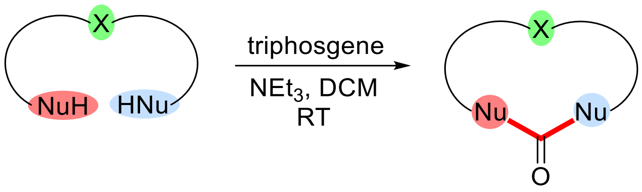

To a solution of di-nucleophile (1.00 mmol) in DCM (10 mL) at RT, triethylamine (1.50 mmol) and triphosgene (0.400 mmol, i.e. corresponding to 1.2 phosgene equivalents) were added sequentially. The resulting mixture was stirred at RT for 30 min, quenched by the addition of water (20 mL), extracted with DCM (2 x 20 mL), dried over $MgSO_4$, concentrated under vacuum and purified by column chromatography.

General CRE method D

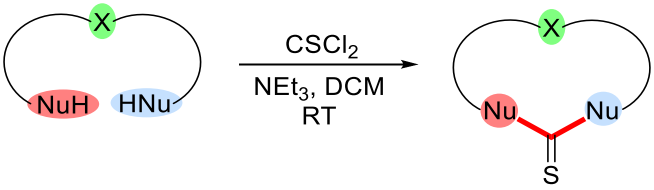

Thiophosgene (1.20 mmol) was added via syringe over a period of 30 sec. to a solution of 3 di-nucleophile (1.00 mmol) and anhydrous Et_3N (3.50 mmol) in anhydrous CH_2Cl_2 (20 mL). The resulting mixture was stirred at RT for 18 h. The mixture was quenched with sat. $NaHCO_{3(aq)}$ (40 mL), before was transferred to separating funnel. The aqueous phase was extracted with CH_2Cl_2 (3 × 30 mL) and combined organic layers were dried over $MgSO_4$, filtered and concentrated under reduced pressure and purified by column chromatography.

General CRE method E

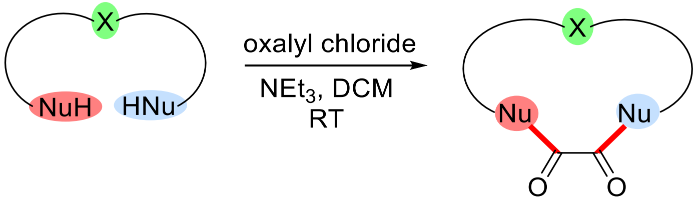

Oxalyl chloride (1.20 mmol) is added in a single portion via syringe to a solution of di-nucleophile (1.00 mmol) and dry Et_3N (500 µL, 3.00 mmol) in anhydrous CH_2Cl_2 (20 mL) under argon at RT. The resulting solution is stirred at RT overnight under Ar, then diluted with DCM (30 mL), washed with water (20 mL) and extracted with CH_2Cl_2 (3 × 30 mL). The combined organic layers were dried over $MgSO_4$, filtered and concentrated under reduced pressure and purified by column chromatography. In cases where there are concerns about the solubility of product in water (*e.g.* when using triamine starting materials) it is also possible to concentrate the reaction mixture and purify by column chromatography directly, skipping the aqueous work-up step.

General CRE method F

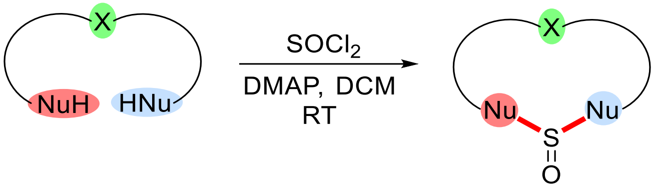

Thionyl chloride (2.00 mmol) was added in a single portion via syringe to a solution of di-nucleophile (1.00 mmol) and DMAP (4.00 mmol) in anhydrous CH_2Cl_2 (24.0 mL) at 0 °C under Ar. The resulting mixture was allowed to warm gradually to RT and stirred for 18 h. The resulting mixture was then concentrated under reduced pressure and purified by flash column chromatography. For an example which includes an aqueous work-up, see the method to prepare compound **89**.

General CRE method G

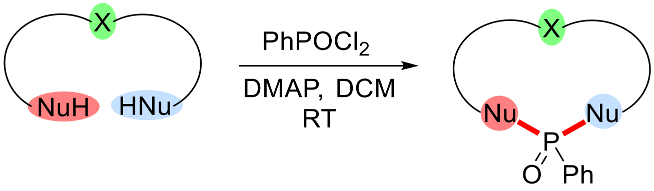

Phenylphosphonic dichloride (1.20 mmol) was added in a single portion via syringe to a solution of di-nucleophile (1.00 mmol) and DMAP (3.00 mmol) in anhydrous CH_2Cl_2 (20 mL) at 0 °C under argon. The resulting mixture was allowed to warm gradually to RT and stirred for 18 h. The resulting mixture was then concentrated under reduced pressure and purified by flash column chromatography.

General CRE method H

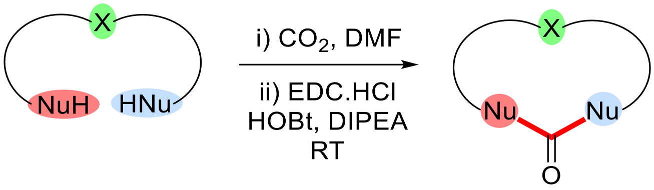

Diamine (1.00 mmol) was added a round bottom flask (250 mL RBF) containing anhydrous DMF (20 mL). The resulting reaction vessel was evacuated under vacuum and backfilled with CO_2 (via balloon) 6 times. Next, EDC.HCl (1.50 mmol), HOBt (405 mg, 1.20 mmol) and dry DIPEA (1.20 mmol) were each added in a single portion at RT. The resulting solution was stirred at RT overnight under a slight positive pressure of CO_2 (balloon) and the progress of the reaction was monitored via TLC. After 18 h, the reaction was diluted with CH_2Cl_2 (25 mL) and poured into a separating funnel. The organic layer was washed sequentially with sat. $NaHCO_{3(aq)}$ (2 × 30 mL) and sat. brine (6 × 30 mL), before it was dried over $MgSO_4$, filtered and concentrated under reduced pressure. The crude product was purified by flash column chromatography. See next page (Figure S1) for a photo of a typical reaction set-up.

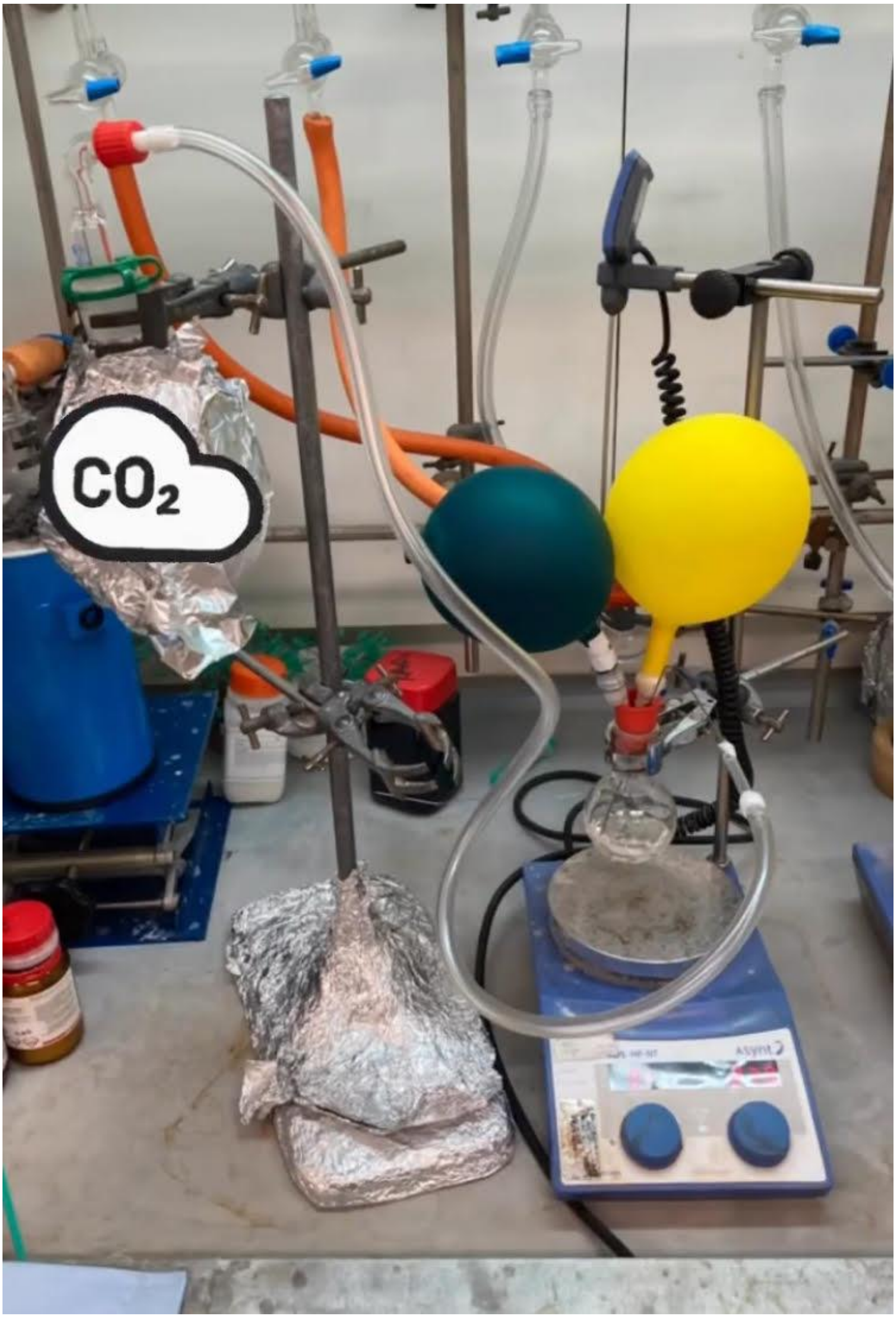

Figure S1 – reaction set up for general CRE method H. The CO_2 source inside the folied-covered flask is regular laboratory grade dry ice, with this system also used so fill the balloons with CO_2.

3) Compound characterization data and procedures

Key to intemediates used to make lactone 9[1]

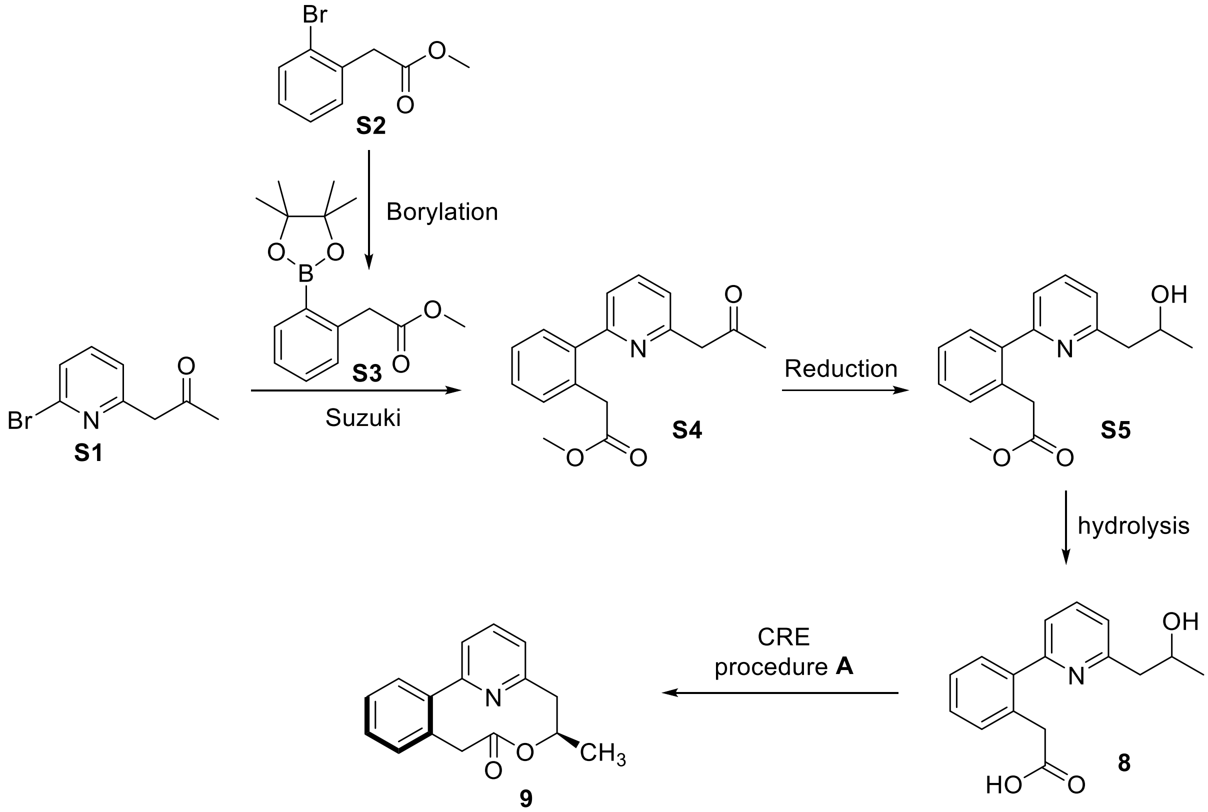

1-(6-Bromopyridin-2-yl)propan-2-one (S1)

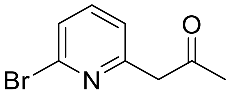

To a stirring solution of diisopropylamine (11.0 mL, 78.6 mmol) in dry THF (200 mL), was added *n*-butyllithium (32.7 mL, 78.6 mmol, 2.4 M solution in hexane) dropwise at −10 °C. The resulting solution was stirred at 0 °C for 30 mins, after which the solution was cooled to −78 °C. 6-Bromo-2-methylpyridine (4.44 mL, 39.3 mmol) was then added dropwise and the solution was stirred for an additional 1 h. *N*-methoxy-*N*-methylacetamide (8.73 mL, 78.6 mmol) was then added and the solution was stirred for a further 2 h. After allowing to warm to r.t., the solution was quenched with water (150 mL), extracted with diethyl ether (3 × 150 mL) and the organic layer was washed with brine (300 mL). The organic layer was dried ($MgSO_4$), filtered and solvent removed *in vacuo* to yield the crude product. Purification *via* flash column chromatography (SiO_2, 7:3 → 3:2 hexane:ethyl acetate) afforded the *title*

compound as a yellow oil (7.00 g, 83%); R_f 0.50 (3:7 ethyl acetate:hexane); ν_{max}/cm^{-1} (thin film) 2976, 1716, 1579, 1554, 1406; δ_H (400 MHz, $CDCl_3$) 7.51 (1H, t, *J* = 7.8 Hz, ArH) 7.38 (1H, d, *J* = 7.8 Hz, ArH), 7.17 (1H, d, *J* = 7.8 Hz, ArH), 3.90 (2H, s, CH_2), 2.24 (3H, s, CH_3); δ_C (100 MHz, $CDCl_3$) 204.0 (CO), 155.6 (ArC), 141.7 (ArC), 139.0 (ArC), 126.5 (ArC), 123.3 (ArC), 52.5 (CH_2), 30.3 (CH_3); HRMS (ESI): calcd. for $C_8H_9{}^{79}BrNO$ 213.9858. Found: $[MH]^+$, 213.9862 (1.7 ppm error).

Methyl 2-(2-bromophenyl)acetate (S2)

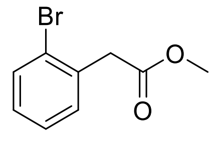

To a stirring solution of 2-(2-bromophenyl)acetic acid (13.2 g, 61.6 mmol) in methanol (130 mL) was added concentrated sulfuric acid (2.50 mL) and the resulting solution was refluxed overnight. After cooling to r.t., the reaction was quenched with water (50 mL) and extracted with diethyl ether (3 × 150 mL). The organic extract was washed with brine (30 mL), dried over anhydrous $MgSO_4$, filtered and concentrated *in vacuo* to afford the *title compound* as a clear oil (13.4 g, 95%); δ_H (400 MHz, $CDCl_3$) 7.56 (1H, d, *J* = 8.4 Hz, ArH), 7.28−7.26 (2H, m, ArH), 7.16–7.12 (1H, m, ArH), 3.79 (2H, s, CH_2), 3.71 (3H, s, CH_3); the data obtained are consistent with those reported in the literature.[2]

Methyl 2-(2-(4,4,5,5-tetramethyl-1,3,2-dioxaborolan-2-yl)phenyl)acetate (S3)

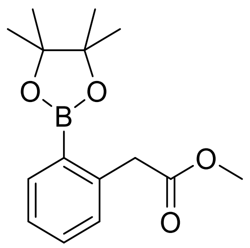

To a stirring solution of methyl 2-(2-bromophenyl)acetate **S2** (12.5 g, 55.0 mmol) in 1,4-dioxane (200 mL), was added bis(pinacalato)diboron (15.3 g, 60.6 mmol), potassium acetate (19.9 g, 203.7 mmol) and $PdCl_2$(dppf).DCM (2.25 g, 3.53 mmol). The reaction mixture was flushed with argon and heated under reflux for 4 h. The reaction mixture was then diluted with ethyl acetate, passed through Celite® (ethyl acetate wash), and concentrated *in vacuo*. Purification *via* silica gel chromatography (DCM → 1:9 ethyl acetate:DCM) yielded the pure product as an off-white solid (8.20 g, 54%); δ_H (400 MHz, $CDCl_3$) 7.83 (dd, *J* = 7.6, 1.5 Hz, 1H,

ArH), 7.40−7.36 (m, 1H, ArH), 7.28−7.24 (m, 1H, ArH), 7.18 (d, *J* = 7.6 Hz, 1H, ArH), 3.97 (s, 2H, CH_2), 3.66 (s, 3H, OCH_3), 1.31 (s, 12H, 4 × CH_3); the data obtained are consistent with those reported in the literature.[3]

Methyl 2-(2-(6-(2-oxopropyl)pyridin-2-yl)phenyl)acetate (S4)

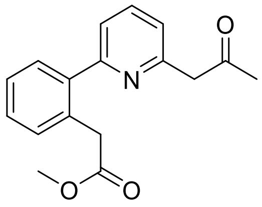

To a microwave vial, methyl 2-(2-(4,4,5,5-tetramethyl-1,3,2-dioxaborolan-2-yl)phenyl)acetate **S3** (1.80 g, 6.52 mmol), potassium triphosphate (2.08 g, 9.78 mmol) and $Pd(PPh_3)_4$ (377 mg, 0.330 mmol) was added and the vial was purged with argon. Dimethylformamide (16 mL) and 1-(6-bromopyridin-2-yl)propan-2-one **S1** (698 mg, 3.26 mmol) were added, and the solution was heated and stirred for 90 min at 150 ^{o}C in a microwave reactor. The solution was then cooled, diluted with ethyl acetate and passed through Celite®, washing with ethyl acetate. The solvent was then evaporated *in vacuo*. Water (30 mL), followed by ethyl acetate (100 mL) were added and both layers were separated. The aqueous layers were extracted with ethyl acetate (3 × 100 mL). The combined organic layers were dried with anhydrous $MgSO_4$, filtered, and concentrated *in vacuo*. Purification *via* flash column chromatography (SiO_2, 3:7→2:3 ethyl acetate: hexane) afforded the *title compound* as a yellow oil (633 mg, 69%); R_f 0.49 (1:1 ethyl acetate:hexane); ν_{max}/cm^{-1} (thin film) 2949, 1726, 1569, 1447, 1161; δ_H (400 MHz, $CDCl_3$) 7.73 (1H, t, *J* = 7.9 Hz, ArH), 7.47–7.45 (1H, m, ArH), 7.40–7.33 (4H, m, ArH), 7.17 (1H, dd, *J* = 7.8, 0.7 Hz, ArH), 3.93 (2H, s, $NCCH_2$), 3.80 (2H, s, C**H₂**CO_2CH_3), 3.56 (3H, s, OCH_3), 2.21 (3H, s, $COCH_3$); δ_C (101 MHz, $CDCl_3$) 205.4 (CO), 172.2 (COO), 159.1 (ArC), 153.9 (ArC), 139.9 (ArC), 137.1 (ArC), 132.4 (ArC), 131.6 (ArC), 129.7 (ArC), 128.5 (ArC), 127.4 (ArC), 122.1 (ArC), 121.9 (ArC), 52.9 (NC**C**H_2), 51.7 (OCH_3), 39.4 (**C**H_2COO), 29.9 (CO**C**H_3); HRMS (ESI): calcd. for $C_{17}H_{18}NO_3$ 284.1275. Found: $[MH]^+$, 284.1281 (2.2 ppm error).[1]

Methyl 2-(2-(6-(2-hydroxypropyl)pyridin-2-yl)phenyl)acetate (S5)

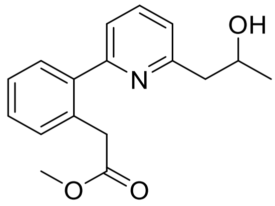

To a stirring solution of methyl 2-(2-(6-(2-oxopropyl)pyridin-2-yl)phenyl)acetate **S4** (96.0 mg, 0.339 mmol) in methanol (5.6 mL), was added sodium borohydride (51.3 mg, 1.36 mmol) and the resulting mixture was stirred at r.t. for 1 h. The solvent was then removed *in vacuo* and water (5 mL) was added. The water was then extracted with ethyl acetate (3 × 10 mL) and the combined organic extracts were washed with brine (10 mL). The organic extract was dried ($MgSO_4$), filtered and concentrated *in vacuo* to yield. Purification *via* flash column chromatography (SiO_2, 1:9 methanol:ethyl acetate) afforded the *title compound* as a colorless oil (89.0 mg, 92%); R_f 0.45 (1:1 ethyl acetate:hexane); ν_{max}/cm^{-1} (thin film) 3405, 2966, 1733, 1590, 1570, 1447, 1372, 1339, 1251, 1212. 1158, 1118, 1083, 1038, 1012; δ_H (400 MHz, $CDCl_3$) 7.70 (1H, t, *J* = 7.8 Hz, ArH), 7.44–7.30 (5H, m, ArH), 7.10 (1H, d, *J* = 7.3 Hz, ArH), 4.55 (1H, br s, OH), 4.32–4.24 (1H, m, C**H**-OH), 3.86 (1H, d, *J* = 16.0 Hz, C**H**H-CO), 3.79 (1H, d, *J* = 16.0 Hz, CH**H**-CO), 3.57 (3H, s, OCH_3), 2.97 (1H, dd, *J* = 14.7, 3.2 Hz, NCC**H**H), 2.88 (1H, dd, *J* = 14.7, 8.7 Hz, NCCH**H**), 1.29 (3H, d, *J* = 6.3 Hz, CH-C**H₃**); δ_C (101 MHz, $CDCl_3$) 172.2 (CO), 159.4 (ArC), 158.5 (ArC), 140.2 (ArC), 137.2 (ArC), 132.2 (ArC), 131.3 (ArC), 129.8 (ArC), 128.5 (ArC), 127.4 (ArC), 121.9 (ArC), 121.8 (ArC), 67.1 (CH-OH), 51.9 (OCH_3), 45.6 (NC**C**H_2), 39.3 (**C**H_2-CO), 23.0 (CH_3); HRMS (ESI): calcd. for $C_{17}H_{20}NO_3$ 286.1438 found: $[MH]^+$, 286.1437 (0.4 ppm error). [1]

2-(2-(6-(2-Hydroxypropyl)pyridin-2-yl)phenyl)acetic acid (8)

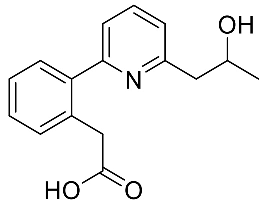

To methyl 2-(2-(6-(2-hydroxypropyl)pyridin-2-yl)phenyl)acetate **S5** (179 mg, 0.631 mmol), was added aqueous LiOH (1.39 mL, 0.694 mmol, 0.5 M) and THF (1.39 mL, 17.1 mmol) and the resulting solution was stirred for 16 h. The resulting solution was then concentrated *in vacuo*. Purification *via* flash column chromatography (SiO_2, 1:9 methanol:ethyl acetate)

afforded the *title compound* **8** as a white solid (99 mg, 57%); R_f 0.26 (1:9 methanol:ethyl acetate); m.p. 122–125 °C; ν_{max}/cm^{-1} (thin film) 3398, 2971, 1719, 1599, 1450, 1119, 1014; δ_H (400 MHz, $CDCl_3$) 7.89 (1H, t, *J* = 7.8 Hz, ArH), 7.51–7.46 (3H, m, ArH), 7.43–7.35 (3H, m, ArH), 4.22–4.14 (1H, m, C**H**-OH), 3.65 (2H, s, C**H₂**CO₂H), 3.06 (1H, dd, *J* = 13.5, 4.3 Hz, NCC**H**H), 2.98 (1H, dd, *J* = 13.5, 8.0 Hz, NCCH**H**), 1.27 (3H, d, *J* = 5.9 Hz, CH-C**H₃**); δ_C (101 MHz, $CDCl_3$) 173.1 (CO), 157.4 (ArC), 156.7 (ArC), 139.4 (ArC), 137.3 (ArC), 132.9 (ArC), 131.5 (ArC), 130.6 (ArC), 129.8 (ArC), 127.7 (ArC), 123.9 (ArC), 122.8 (ArC), 67.8 (OCH), 45.6 (NC**C**H_2), 41.9 (**C**H_2CO), 23.3 (CH_3); HRMS (ESI): calcd. for $C_{16}H_{18}NO_3$ 272.1281 found: $[MH]^+$, 272.1280 (0.6 ppm error).[1]

4-Methyl-4,5-dihydro-6,10-(azeno)benzo[*d*][1]oxacyclododecin-2(1*H*)-one (9)

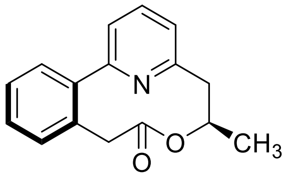

To a stirring solution of 2-(2-(6-(2-hydroxypropyl)pyridin-2-yl)phenyl)acetic acid **8** (44.0 mg, 0.163 mmol) in chloroform (3 mL), was added diisopropylethylamine (50.0 µL, 0.302 mmol), followed by the addition of T3P (0.284 mmol, 156 mg of a 50% solution in ethyl acetate). Upon the addition of T3P, the solution rapidly changed from a colourless to an orange solution. After stirring for 30 mins at r.t., the solution concerntrated and purified *via* flash column chromatography (SiO_2, 1:1 ethyl acetate:hexane) afforded the *title compound* **9** as a pale yellow solid (37 mg, 90%); R_f 0.45 (3:7 ethyl acetate:hexane); m.p. 110−114 °C; ν_{max}/cm^{-1} (thin film) 3063, 2973, 2928, 1720, 1586, 1575, 1450, 1422; δ_H (400 MHz, $CDCl_3$) 7.80–7.78 (1H, m, ArH), 7.69 (1H, t, *J* = 7.6 Hz, ArH), 7.54 (1H, d, *J* = 7.6 Hz, ArH), 7.43–7.40 (3H, m, ArH), 7.04 (1H, dd, *J* = 7.6, 0.5 Hz, ArH), 5.64–5.55 (1H, m, OCH), 3.65 (1H, d, *J* = 14.5 Hz, C**H**HCO), 3.54 (1H, d, *J* = 14.5 Hz, CH**H**CO), 3.12–3.00 (2H, m, NCCH_2), 1.50 (3H, d, *J* = 6.4 Hz, CH_3); δ_C (101 MHz, $CDCl_3$) 174.3 (CO), 155.6 (ArC), 154.9 (ArC), 137.2 (ArC), 135.1 (ArC), 133.9 (ArC), 129.2 (ArC), 127.9 (ArC), 127.7 (ArC), 121.0 (ArC), 118.4 (ArC), 70.4 (OCH), 44.4 (CO-**C**H_2), 40.4 (NC**C**H_2), 21.4 (CH_3); HRMS (ESI): calcd. for $C_{16}H_{16}NO_2$ 254.1176. Found: $[MH]^+$, 254.1173 (1.0 ppm error).[1]

Methyl 2-(3'-(2-oxopropyl)-[1,1'-biphenyl]-2-yl)acetate (S6)

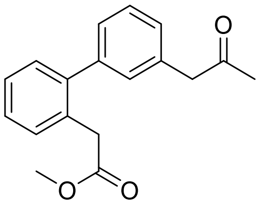

To a microwave vial, methyl 2-(2-(4,4,5,5-tetramethyl-1,3,2-dioxaborolan-2-yl)phenyl)acetate **S3** (556 mg, 2.00 mmol), potassium triphosphate (639 mg, 3.00 mmol) and $Pd(PPh_3)_4$ (115 mg, 0.100 mmol) were added and the vial was purged with argon. Dimethylformamide (8 mL) and 3-bromophenylacetone (215 mg, 1.00 mmol) were added and the resulting solution was then heated and stirred for 90 min at 150 °C in a microwave reactor. The solution was then cooled, diluted with ethyl acetate and passed through Celite®. The combined organics were then washed with water (25 mL) and brine (25 mL). The aqueous layer was then re-extracted with diethyl ether (25 mL), and the organic extract was washed with brine (25 mL). The combined organic layers were dried with anhydrous $MgSO_4$, filtered, and concentrated *in vacuo* to yield the crude product. Purification *via* flash column chromatography (7:3 → 65:35 hexane:ethyl acetate) afforded the *title compound* as a pale yellow oil (203 mg, 72%); R_f 0.31 (7:3 hexane/ethyl acetate); ν_{max}/cm^{-1} (thin film) 3022, 2951, 1724, 1601, 1584, 1497, 1476, 1433, 1356, 1337, 1211, 1156, 1106, 1054, 1002; δ_H (400 MHz, $CDCl_3$) 7.40–7.31 (4H, m, ArH), 7.28–7.26 (1H, m, ArH), 7.23–7.20 (2H, m, ArH), 7.17−7.15 (1H, m, ArH), 3.73 (2H, s, C**H₂**CO₂), 3.62 (3H, s, OC**H₃**), 3.60 (2H, s, C**H₂**CO), 2.18 (3H, s, CC**H₃**); δ_C (101 MHz, $CDCl_3$) 206.4 (CO), 172.4 (COO), 142.2 (ArC), 141.7 (ArC), 134.3 (ArC), 131.8 (ArC), 130.53 (ArC), 130.50 (ArC), 130.3 (ArC), 128.8 (ArC), 128.4 (ArC), 128.1 (ArC), 127.8 (ArC), 127.4 (ArC), 52.1 (OCH_3), 51.1 (**C**$H_2CO_2CH_3$), 38.9 (**C**H_2COCH_3), 29.5 (CO**C**H_3); HRMS (ESI): calcd. for $C_{18}H_{18}NaO_3$ 305.1148. Found: $[MNa]^+$, 305.1145 (1.2 ppm error).[1]

Methyl-2-(3'-(2-hydroxypropyl)-[1,1'-biphenyl]-2-yl)acetate (S7)

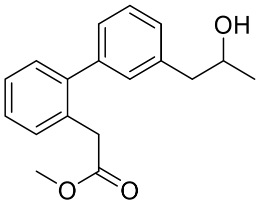

To a stirring solution of methyl 2-(3'-(2-oxopropyl)-[1,1'-biphenyl]-2-yl)acetate **S6** (161 mg, 0.569 mmol) in methanol (5 mL), was added sodium borohydride (86.0 mg, 2.28 mmol) and the resulting solution was stirred at r.t. for 1 hr. The solution was then quenched with saturated ammonium chloride (5 mL) and water (5 mL). The reaction mixture was then extracted with ethyl acetate (3 × 5 mL) and the organic layer was washed with brine (10 mL). The organic extract was then dried with $MgSO_4$, filtered and concentrated *in vacuo* to yield the crude product. Purification *via* flash column chromatography (7:3 → 3:2 hexane:ethyl acetate) afforded the *title compound* as a colorless oil (119 mg, 74%); R_f 0.19 (hexane:ethyl acetate 7:3); ν_{max}/cm^{-1} (thin film) 3423, 2967, 1733, 1435, 1159; δ_H (400 MHz, $CDCl_3$) 7.39–7.33 (4H, m, ArH), 7.31–7.27 (1H, m, ArH), 7.23–7.17 (3H, m, ArH), 4.10–4.03 (1H, m, C**H**OH), 3.65 (2H, d, *J* = 3.7 Hz, C**H₂**CH), 3.59 (3H, s, OC**H₃**), 2.86–2.81 (1H, dd, *J* = 13.4, 4.8 Hz, C**H**HCO), 2.77–2.72 (1H, dd, *J* = 13.4, 7.9 Hz, CH**H**CO), 2.32 (1H, br s, O**H**), 1.28 (3H, d, *J* = 6.4 Hz, CHC**H₃**); δ_C (101 MHz, $CDCl_3$) 172.4 (CO), 142.3 (ArC), 141.4 (ArC), 138.6 (ArC), 131.8 (ArC), 130.5 (ArC), 130.4 (ArC), 130.1 (ArC), 128.4 (ArC), 128.3 (ArC), 127.6 (ArC), 127.3 (ArC), 127.2 (ArC), 68.8 (OCH), 52.0 (OCH_3), 45.8 (NC**C**H₂), 39.2 (**C**H₂CO), 22.8 (CH_3); HRMS (ESI): calcd. for $C_{18}H_{21}O_3$ 285.1485. Found: $[MH]^+$, 285.1483 (1.0 ppm error).[1]

2-(3'-(2-Hydroxypropyl)-[1,1'-biphenyl]-2-yl) acetic acid (10)

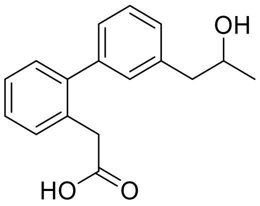

To methyl-2-(3'-(2-hydroxypropyl)-[1,1'-biphenyl]-2-yl)acetate **S7** (119 mg, 0.422 mmol), was added 0.5 M aqueous LiOH (0.930 mL, 0.464 mmol) and THF (0.930 mL) and the resulting solution was stirred for 16 h and then concentrated *in vacuo*. Purification *via* flash column chromatography (9:1 ethyl acetate:methanol) afforded the *title compound* as a colorless oil (92.9 mg, 81%); R_f 0.71 (4:1 ethyl acetate:methanol); ν_{max}/cm^{-1} (thin film) 2969, 2927, 1705,

1601; δ_H (400 MHz, $CDCl_3$) 7.33–7.29 (4H, m, ArH), 7.27–7.24 (1H, m, ArH), 7.16–7.13 (3H, m, ArH), 4.06–3.98 (1H, m, C**H**-OH), 3.55 (2H, s, C**H₂**CO_2H), 2.78 (1H, dd, *J* = 13.7, 5.0 Hz, C**H**H-CH), 2.67 (1H, dd, *J* = 13.7, 8.2 Hz, CH**H**-CH), 1.20 (3H, d, *J* = 5.9 Hz, CH-C**H₃**); δ_C (101 MHz, $CDCl_3$) 176.9 (CO), 142.6 (ArC), 141.2 (ArC), 138.5 (ArC), 131.7 (ArC), 130.7 (ArC), 130.5 (ArC), 130.1 (ArC), 128.6 (ArC), 128.4 (ArC), 127.7 (ArC), 127.4 (ArC), 127.3 (ArC), 69.2 (CH-OH), 45.6 (**C**H_2CH), 39.3 (**C**H_2CO), 22.7 (CH_3); HRMS (ESI): calcd. for $C_{17}H_{18}NaO_3$ 293.1148 found: $[MNa]^+$, 293.1141 (2.3 ppm error).[1]

4-Methyl-4,5-dihydro-6,10-(metheno)benzo[d][1]oxacyclododecin-2(1H)-one (S8)

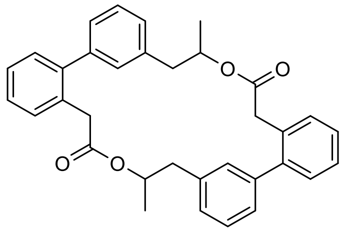

To a solution of 2-(3'-(2-hydroxypropyl)-[1,1'-biphenyl]-2-yl)acetic acid **10** (156 mg, 0.577 mmol) in chloroform (11 mL) was added DIPEA (0.200 mL, 149 mg, 1.15 mmol) and pyridine (0.050 mL, 46 mg, 0.577 mmol) followed by T3P (0.551 mg, 50% in ethyl acetate, 0.866 mmol) and the reaction mixture was stirred at RT for 5 h. The reaction mixture was concentrated *in vacuo* and the crude product was purified by column chromatography (SiO_2, 7:3 hexane:ethyl acetate) to yield the title compound (13 mg, 9%) as a 1:1 mixture of diastereoisomers and as a colourless oil. R_f 0.45 (7:3 hexane:ethyl acetate); δ_H (400 MHz, $CDCl_3$) 7.36–6.98 (16H, m, ArH), 5.18–5.05 (2H, m, CH), 3.64–3.38 (4H, m, CH_2CO_2), 2.90–2.70 (4H, m, C**H₂**CHO), 1.22–1.17 (6H, m, CH_3).[1]

Ethyl 2–{[(3–hydroxypropyl)sulfanyl]methyl}benzoate (S9)

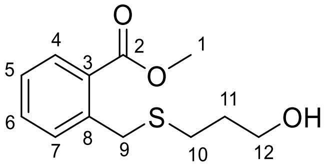

K_2CO_3 (2.53 g, 18.3 mmol) was added to a pale yellow solution of 3-mercapto-1-propanol (2.50 mL, 1.25 mmol) and methyl 2-(bromomethyl)benzoate (4.29 g, 1.88 mmol) in anhydrous DMF (42 mL) at RT. The resulting milky/white suspension was then heated to 60 °C. After 12 h, the reaction was deemed to have gone to completion by TLC. The reaction mixture allowed to cool to RT, before was quenched with H_2O (30 mL). The milky/white suspension was poured

into a separating funnel and the aqueous layer was extracted with EtOAc (3 × 20 mL). The combined organic layers were washed sequentially with H_2O (3 × 20 mL) and sat. brine (2 × 20 mL), before dried over $MgSO_4$, filtered and concentrated under reduced pressure to yield a pale yellow oil (4.21 g). The crude product was purified by flash column chromatography (SiO_2, 70 mm column, eluent: EtOAc:*n*–hexane, 60:40) to afford alcohol **S9** as a pale yellow viscous oil (3.86 g, 86%). R_f = 0.25 (50:50 EtOAc:*n*–hexane); δ_H (400 MHz; $CDCl_3$) 8.01 (1H, dd, *J* 7.7, 1.5, C(4)H), 7.44 (2H, td, *J* 7.7, 1.5, C(7)H), 7.40 – 7.24 (2H, m, C(6/5)H), 4.12 (2H, s, C(9)H_2), 3.90 (3H, s, C(1)H_3), 3.69 (2H, t, *J* 6.1, C(12)H_2), 2.55 (2 H, t, *J* 7.0, C(10)H_2), 1.79 (2H, tt, *J* 7.0, 6.1, C(11)H_2); HRMS (ESI$^+$): m/z calc. for $C_{12}H_{16}NaO_3S$: 263.0712, found: 263.0716 $[M+Na]^+$.

2-(((3-Hydroxypropyl)thio)methyl)benzoic acid (**12**)

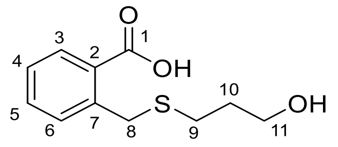

$NaOH_{(aq)}$ (0.5 M, 16 mL) was added dropwise over a period of 2 min to solution of alcohol **S9** (3.86 g, 1.61 mmol) in MeOH (16 mL). A colour change to pale yellow solution was immediately noted and the reaction mixture was stirred at RT for 12 h, at which point the reaction mixture was deemed to have gone to completion by TLC (complete consumption of alcohol **S9** was noted). The reaction mixture was then acidified to pH 2.0 with 1M $HCl_{(aq)}$ (20 mL). The resulting off–white solid was collected by suction filtration and washed with H_2O (3 × 20 mL) and then air dried to yield carboxylic acid **12** as a white solid (2.18 g, 60%). R_f = 0.14 (10:90 MeOH:EtOAc); Melting Point: 110 – 112 °C (from CH_2Cl_2); IR (solid state) ν_{max} / cm^{-1}: 3495br (O–H alcohol), 3470br (O–H alcohol), 2950br (C–H alkyl, O–H carboxylic acid), 2643br (C–H alkyl, O–H carboxylic acid), 1674vs (C=O carboxylic acid), 1597w (CC aromatic), 1574s (CC aromatic), 1486m, 1435m, 1408s, 1301vs (C–O), 1267vs (C–O), 1197w, 1170w, 1140m, 1082m, 1055s, 1024m, 924s, 905m, 841w, 809w, 772s, 771s, 737m, 683m, 658s, 544s, 500m, 475w; δ_H (400 MHz; CD_3OD) 7.90 (1H, dd, *J* 7.5 1.5, C(3)H), 7.45 (1H, app. td, *J* 7.5, 1.5, C(5)H), 7.38 (1H, dd, *J* 7.5, 1.5, C(6)H), 7.32 (1 H, app. td, *J* 7.5, 1.5, C(4)H), 4.14 (2H, s, C(8)H_2), 3.58 (2H, t, *J* 6.3, C(11)H_2), 2.48 (2H, t, *J* 7.4, C(9)H_2), 1.78 – 1.69 (2H, m, C(10)H_2); δ_C (101 MHz; CD_3OD) 170.9 (C, C1), 142.1 (C, C7), 132.7 (CH, C5), 132.2 (CH, C3), 132.2 (CH, C6), 131.5 (C,

C2), 128.0 (CH, C4), 61.5 (CH_2, C11), 35.0 (CH_2, C8), 33.2 (CH_2, C10), 28.9 (CH_2, C9); HRMS (ESI^+): m/z calc. for $C_{11}H_{14}NaO_3S$: 249.0556, found: 249.0558 $[M+Na]^+$.

4,5-Dihydro-3*H*-benzo[*g*][1,5]oxathionin-1(7*H*)-one (13)

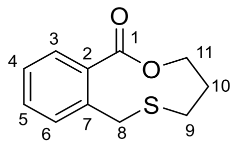

EDC.HCl (294 mg, 1.53 mmol) was added to solution of carboxylic acid **12** (223 mg, 0.988 mmol), HOBt (188 mg, 1.39 mmol) and dry DIPEA (500 µL, 5.21 mmol) in anhydrous DMF (10 mL). A colour change of the reaction mixture over 30 min from clear colorless to pale yellow. After total of 18 h of stirring at RT, the reaction deemed to have to completion by TLC. The resulting mixture was diluted with EtOAc (20 mL), before was transferred to separating funnel and the organic phase was washed sequentially with H_2O (3 × 20 mL) and sat. brine (2 × 20 mL). The organic phase was dried over $MgSO_4$, filtered and concentrated under reduced pressure to yield a white solid (196 mg). The crude product was purified by flash column chromatography (SiO_2, 20 mm column, eluent: EtOAc:*n*–hexane, 50:50) to afford lactone **13** as a white solid (178 mg, 86%). R_f = 0.56 (50:50 EtOAc:*n*–hexane); Melting Point: 163– 168 °C (from *n*–hexane); IR (solid sate) ν_{max} / cm^{-1}: 2963w (C–H alkyl), 2916w (C–H alkyl), 1714vs (C=O aryl ester), 1599m (CC aromatic), 1486w, 1452m, 1431m, 1423m, 1381m, 1350m, 1298s, 1268vs, 1210m, 1195m, 1126vs, 1088m, 1046m, 975s, 962m, 975s, 901w, 890w, 873w, 834w, 810w, 801w, 768s, 707vs, 660m, 579m, 533w, 503w, 482m; δ_H (400 MHz; $CDCl_3$) 7.85 (1H, dd, *J* 7.0, 2.0, C(3)H), 7.38–7.30 (2H, m, C(4+5)H), 7.15 (1H, dd, *J* 7.0, 2.0, C(6)H), 4.62 (2H, t, *J* 5.9, C(11)H_2), 4.13 (2H, s, C(8)H_2), 2.90 – 2.86 (2H, m, C(9)H_2), 2.21 – 2.14 (2H, m, C(10)H_2); δ_C (101 MHz; $CDCl_3$) 168.2 (C, C1), 142.2 (C, C7), 131.7 (C, C2), 131.6 (CH, C5), 131.4 (CH, C3), 130.1 (CH, C6), 127.6 (CH, C4), 66.0 (CH_2, C11), 41.1 (CH_2, C8), 35.3 (CH_2, C9), 30.2 (CH_2, C10); HRMS (ESI^+): m/z calc. for $C_{11}H_{13}O_2S$: 209.0631, found: 209.0631 $[M+H]^+$, m/z calc. for $C_{11}H_{12}NaO_2S$: 231.0450, found : 231.0448 $[M+Na]^+$.

X-ray crystallographic data for compound **13** can be accessed via www.ccdc.cam.ac.uk/data_request/cif (CCDC 2221211)

Methyl 2-(5-hydroxypent-1-yn-1-yl) benzoate (S10)

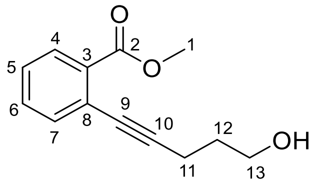

4-Pentyn-1-ol (2.41 mL, 25.9 mmol) was added to solution of methyl 2-iodobenzoate (2.00 mL, 13.6 mmol) in dry Et_3N (45 mL) at RT. After 5 min, CuI (256 mg, 1.36 mmol) and bis(triphenylphosphine)palladium chloride (478 mg, 68.1 µmol) was added to the pale-yellow solution. The resulting reaction mixture was stirred at RT. Upon stirring, the colour of the reaction mixture changed over 4 h from grey-green (after 1 h) to dark brown (after 2 h) and finally black-brown. After a total of 2 h, the reaction was deemed to have gone to completion by TLC. The dark brown reaction mixture was filtered through Celite and washed with EtOAc (20 mL). The resulting filtrate was diluted with EtOAc (20 mL) was poured to into separating funnel and the organic layer was washed sequentially with $HCl_{(aq)}$ (1.0 M, 3 × 40 mL) and brine (1 × 40 mL). The organic layer was dried with $MgSO_4$, filtered and concentrated under reduced pressure to yield a brown/orange oil (3.11 g). The crude product was purified by flash column chromatography (SiO_2, 70 mm column, eluent: EtOAc:*n*–hexane, 50:50) to afford alkyne **S10** as a yellow oil (2.58 g, 87%). R_f = 0.08 (30:70 EtOAc:*n*-hexane); IR (thin film) ν_{max} / cm^{-1}: 3403br (O–H alcohol), 3066w (C–H alkenyl), 2950m (C–H alkyl), 2878 (C–H alkyl), 2230w (CC alkynyl), 1715s (C=O aryl ester), 1597m (CC aromatic), 1556m (CC aromatic), 1485s (CC aromatic), 1447m, 1433m, 1348w, 1293s, 1276s, 1249s, 1190m, 1164w, 1130s, 1083s, 1044m, 986w, 960m, 923m, 847m, 825w, 798w, 756s, 736w, 701s, 655w, 537w, 502w; δ_H (400 MHz; $CDCl_3$) 7.83 (1H, dd, *J* 7.8, 1.6, C(4)H), 7.45 (1H, dd, *J* 7.5, 1.6 C(7)H), 7.36 (1H, dd, *J* 7.7, 1.6, C(5)H), 7.25 (1H, td, *J* 7.7, 1.6, C(6)H), 3.85 (3H, s, $C(1)H_3$), 3.79 (2H, t, *J* 6.0, $C(13)H_2$), 3.05 (1H, br, s, OH), 2.55 (2H, t, *J* 6.5, $C(11)H_2$), 1.82 (2H, p, *J* 6.5 $C(12)H_2$); δ_C (101 MHz; $CDCl_3$) 167.9 (C, C2), 134.2 (CH, C7), 131.7 (CH, C5), 131.5 (C, C8), 130.2 (CH, C4), 127.3 (CH, C6), 124.3 (C, C3), 95.2 (C, C10), 79.7(C, C9), 61.4 (CH_2, C13), 52.2 (CH_3, C1), 31.0 (CH_2, C12), 16.5 (CH_2, C11); HRMS (ESI^+): m/z calc. for $C_{13}H_{15}O_3$: 219.1021, found: 219.1016 $[M+H]^+$, m/z calc. for $C_{13}H_{14}NaO_3$: 241.0842, found: 241.0835 $[M+Na]^+$.

2-(5-Hydroxypentyl)benzoic acid (14).

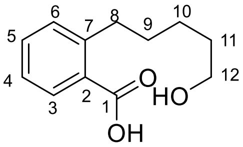

Pd/C (115 mg, Pd 10% on carbon) was added to a round bottom flask, previously purged under N_2 (for 10 min), containing methyl 2-(5-hydroxypent-1-yn-1-yl) benzoate **S10** (1.13 g, 5.18 mmol) at RT. The alkyne was dissolved by addition of EtOAc (26 mL) and the reaction vessel was evacuated under vacuum and then backfilled with H_2 (via balloon) three times, then stirred at RT under an atmosphere of H_2 (balloon) for 4 h. The reaction was then purged with N_2 for 10 min, filtered through Celite and washed with EtOAc (3 × 20 mL). The resulting filtrate was concentrated under reduced pressure to yield the reduced product as a clear colourless oil (1.14 g). The crude product was directly used in the next reaction step without further purification. To this crude product in MeOH (10 mL), $NaOH_{(aq)}$ (2.5 M, 10 mL) was added dropwise over a period of 5 min. A colour change to a cloudy lemon-yellow solution was immediately noted and the resulting suspension was stirred at RT. After 16 h, the reaction mixture was judged to be complete, based on TLC analysis. The pale-yellow solution was then acidified to pH 2.0 with 1M $HCl_{(aq)}$ (10 mL). The resulting white solid was collected by suction filtration and washed with H_2O (3 × 20 mL) and then air dried to yield carboxylic acid **14** as a white solid (760 mg, 73%). R_f = 0.22 (15:85 MeOH:CH_2Cl_2); Melting Point: 90–92 °C (from CH_2Cl_2); IR (solid state) ν_{max} / cm^{-1}: 3458br (O–H alcohol), 2943br (C–H alkyl), 2919mbr (C–H alkyl) 2858br (C–H alkyl), 2802 (C–H alkyl), 2627br (O–H carboxylic acid), 2560w, 2485w, 1687vs (C=O carboxylic acid), 1602w (CC aromatic), 1576w (CC aromatic), 1491w (CC aromatic), 1464w, 1449w, 1418w, 1375w, 1354w, 1310s, 1287m, 1246vs, 1209m, 1192m, 1167w, 1144m, 1110w, 1085m, 1062m, 1045s, 1017m, 983m, 894s, 873w, 835w, 824w, 804w, 769s, 744m, 715s, 685w, 656m, 584m, 558m, 486w; δ_H (400 MHz; CD_3OD) 7.85 (1H, dd, *J* 7.7, 1.5, C(3)H), 7.41 (1H, td, *J* 7.5, 1.5, C(5)H), 7.29 – 7.19 (2 H, m, C(6+4)H), 3.54 (2H, t, *J* 6.6, C(12)H_2), 2.97 (2H, s, C(8)H_2), 1.67 – 1.49 (4H, m, C(11+9)H_2) 1.47 – 1.35 (2H, m, C(10)H_2); δ_H (400 MHz; $CDCl_3$) 8.02 (1H, dd, *J* 8.0, 1.6, C(3)H), 7.46 (1H, td, *J* 8.0, 1.6, C(5)H), 7.31 – 7.25 (2H, m, C(6+4)H), 6.47 (1H, br, s, OH), 3.68 (2H, t, *J* 6.6, C(12)H_2), 3.07 – 2.98 (2H, m, C(8)H_2), 1.72 – 1.58 (4H, m, C(11+9)H_2) 1.55 – 1.40 (2H, m, C(10)H_2); δ_C (101 MHz; CD_3OD) 171.3 (C, C1), 145.5 (C, C7), 132.9 (CH, C5), 132.1 (CH, C6), 131.7 (CH, C3), 131.3 (C, C2), 126.8 (CH, C4),

62.9 (CH_2, C12), 35.4 (CH_2, C8), 33.4 (CH_2, C9), 32.9 (CH_2, C11), 27.0 (CH_2, C10); HRMS (ESI$^+$): m/z calc. for $C_{12}H_{16}NaO_3$: 231.0992, found: 231.0997 $[M+Na]^+$.

4,5,6,7-tetrahydrobenzo[*c*]oxonin-1(3*H*)-one (15) and 8,9,10,11,19,20,21,22-octahydrodibenzo[*c,l*][1,10]dioxacyclooctadecine-5,16(7*H*,18*H*)-dione (S11)

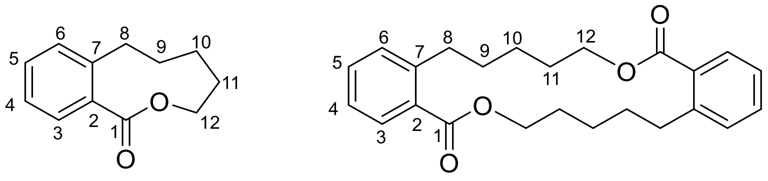

EDC.HCl (290 mg, 1.51 mmol) was added to solution of carboxylic acid **14** (209 mg, 1.00 mmol), HOBt (168 mg, 1.24 mmol) and anhydrous DIPEA (440 µL, 2.50 mmol) in anhydrous DMF (10 mL). A colour change to pale yellow was noted upon after addition of DIPEA via syringe over a period of 30 sec. The resulting mixture was stirred at RT overnight under N_2 and progress of the reaction was monitored by TLC. After total of 18 h, the reaction wasn't judged to be complete, based on TLC analysis which showed carboxylic acid **14** still present. The resulting mixture was diluted with EtOAc (20 mL), before was transferred to separating funnel and the organic phase was washed sequentially with 1M $HCl_{(aq)}$ (2 × 20 mL) and brine (3 × 20 mL). The organic phase was dried over $MgSO_4$, filtered and concentrated under reduced pressure to yield a white solid (303 mg). The crude product was purified by flash column chromatography (SiO_2, 20 mm column, eluent: EtOAc:*n*–hexane, 10:90) to afford lactone **15** as a white solid (82.1 mg, 43%) and dimeric lactone **S11** (21.4 mg, 12%).

Data for **15**: R_f = 0.44 (50:50 EtOAc:*n*–hexane); IR (thin film) ν_{max} / cm^{-1}: 2930w (C–H alkyl), 1714vs (C=O ester), 1604w (CC aromatic), 1451m (CC aromatic), 1287s, 1256s, 1123s, 1088w, 1044w, 979w, 777w, 748w, 704w; δ_H (400 MHz; $CDCl_3$) 7.81 (1H, dd, *J* 7.7, 1.5, C(3)H), 7.38 (1H, td, *J* 7.5, 1.5 C(5)H), 7.28 (1H, td, *J* 7.5 1,5, C(4)H), 7.19 (1H, dd, *J* 7.7, 1.5, C(6)H), 4.55 (2H, t, J 5.7, C(12)H_2), 3.06 (2 H, t, *J* 6.0, C(8)H_2), 1.94 – 1.84 (2H, m, C(11)H_2), 1.76 – 1.62 (2H, m, C(9)H_2), 1.66 – 1.58 (2H, m, C(10)H_2); δ_C (101 MHz; CD_3OD) 171.0 (C, C1), 145.6 (C, C7), 132.0 (CH, C5), 131.4 (C, C2), 131.1 (CH, C3), 130.7 (CH, C6), 126.8 (CH, C4), 67.2 (CH_2, C12), 35.1 (CH_2, C8), 31.6 (CH_2, C9), 27.8 (CH_2, C12), 26.6 (CH_2, C10); HRMS (APCI$^+$): m/z calc. for $C_{12}H_{15}O_2$: 191.1067, found: 191.1061 $[M+H]^+$.

Data for **S11** R_f = 0.35 (50:50 EtOAc:*n*–hexane); IR (thin film) ν_{max} / cm^{-1}: 2937w (C–H alkyl), 1699vs (C=O ester), 1601w (CC aromatic), 1449m (CC aromatic), 1290s, 1272s, 1131s, 1110m, 1040w, 1044w, 948w, 752w, 709w; δ_H (400 MHz; $CDCl_3$) 7.85 (2H, dd, *J* 7.7, 1.5, C(3)H), 7.42 (2H, td, *J* 7.7, 1.5 C(5)H), 7.30 – 7.21 (4H, m, C(4+6)H), 4.36 (4H, t, *J* 5.8, C(12)H_2), 2.96 (4H, t, *J* 6.0, C(8)H_2), 1.86 – 1.75 (4H, m, C(11)H_2), 1.76 – 1.62 (4H, m, C(9)H_2), 1.75 – 1.58 (4H, m, C(10)H_2); δ_C (101 MHz; CD_3OD) 169.4 (C, C1), 143.0 (C, C7), 132.0 (CH, C5), 131.2 (CH, C3), 130.8 (CH, C6), 130.6 (C, C2), 126.1 (CH, C4), 65.8 (CH_2, C12), 35.0 (CH_2, C8), 33.9 (CH_2, C9), 29.4 (CH_2, C12), 27.5 (CH_2, C10); HRMS (APCI$^+$): m/z calc. for $C_{24}H_{29}O_4$: 381.2060, found: 381.2070 $[M+H]^+$.

3-(Phenyl(2-(phenylamino)ethyl)amino)propan-1-ol (16)

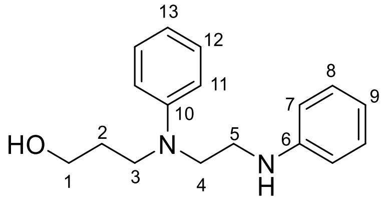

K_2CO_3 (7.64 g, 55.3 mmol) was added pale yellow solution of N^1,N^2-diphenylethane-1,2-diamine (7.04 g, 33.2 mmol) and 3-bromopropan-1-ol (3.07 g, 22.1 mmol) in anhydrous MeCN (74 mL) at RT under Ar. The resulting suspension was then heated to 95 °C, with a colour change to a pale pink suspension observed. After 20 h, the reaction was deemed to have gone to completion by TLC and a colour change to a cloudy beige suspension was observed. The reaction mixture was cooled to RT, before filtered through Celite using a Hirsch funnel and the filtrate residual was washed with EtOAc (3 × 60 mL). The resulting filtrate was concentrated to dryness under reduced pressure to yield crude product as a lilac-coloured oil (10.3 g). The crude product was purified by flash column chromatography (SiO_2, 85 mm column, eluent: EtOAc:*n*–hexane, 30:70 to 40:60) to afford the title compound **16** as a pale yellow oil (2.11 g, 35%). R_f = 0.55 (50:50 EtOAc:n-hexane); IR (neat) ν_{max} / cm^{-1}: 3550wbr (N–H secondary aniline), 3378wbr (O–H alcohol), 3023w (N–H secondary aniline), 2940w (C–H alkyl), 2877w (C–H alkyl), 1597s (CC aromatic/aniline), 1502s (CC aromatic/aniline), 1431w, 1369w, 1320w, 1255w, 1226w, 1195w, 1178w, 1122w, 991m, 925w, 867w, 746s, 692s, 508m; δ_H (400 MHz; $CDCl_3$) 7.32 – 7.22 (2H, m, C(12)H), 7.22 – 7.14 (2H, m, C(8)H), 6.88 – 6.81 (2H, m, C(11)H), 6.78 (1H, tt, *J* 7.3, 1.1, C(13)H), 6.72 (1H, tt, *J* 7.3, 1.1, C(9)H), 6.66 – 6.59 (2H, m,

C(7)H), 3.71 (2H, t, *J* 5.9, C(1)H_2), 3.56 (2H, t, *J* 6.4, C(4)H_2), 3.48 (2H, t, *J* 6.9, C(3)H_2), 3.36 (2H, t, *J* 6.4, C(5)H_2), 1.89 – 1.78 (2H, m, C(2)H_2); δ_C (101 MHz; $CDCl_3$) 147.9 (C, C10), 148.0 (C, C6), 129.6 (CH, C12), 129.5 (CH, C8), 117.8 (CH, C11), 113.9 (2 × CH, C9+13 overlapping), 113.1 (CH, C7), 60.7 (CH_2, C1), 51.2 (CH_2, C4), 48.8 (CH_2, C3), 41.5 (CH_2, C5), 29.9 (CH_2, C2); HRMS (ESI^+): m/z calc. for $C_{17}H_{23}N_2O$: 271.1805, found: 271.1801 $[MH]^+$.

3,6-Diphenyl-1,3,6-oxadiazecan-2-one (17)

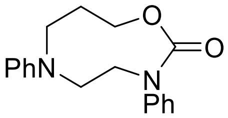

To a solution of 3-(phenyl(2-(phenylamino)ethyl)amino)propan-1-ol **16** (75 mg, 0.277 mmol) in DCM (2.8 mL) at RT, triethylamine (58.0 µL, 0.416 mmol) and triphosgene (32.9 mg, 0.111 mmol) were added sequentially. The resulting mixture was stirred at RT for 30 min, quenched by the addition of water (20 mL), extracted with DCM (2 x 20 mL) and dried of $MgSO_4$ and concentrated under vacuum. Purification by column chromatography (5:1 → 2:1 hexane:ethyl acetate) afforded the title compound **17** as a colourless oil (51 mg, 62%); R_f = 0.60 (1:1 hexane:ethyl acetate); IR (thin film) ν_{max} / cm^{-1} 2958w, 1705s, 1503m, 1256m, 747m, 693s; δ_H (400 MHz; $CDCl_3$) 7.30 – 7.26 (2H, m, Ar-CH), 7.21 – 7.14 (3H, m, Ar-CH), 7.08 (2H, d, *J* 7.8, Ar-CH), 6.70 (1H, t, *J* 7.3, Ar-CH), 6.55 (2H, d, *J* 8.2, Ar-CH), 4.39 (2H, t, *J* 5.7, OCH_2), 3.96 (2H, t, *J* 5.2, NCH_2), 3.73 (2H, t, *J* 5.5, NCH_2), 3.60 – 3.54 (2H, m, NCH_2), 2.06 – 1.98 (2H, m, CH_2); δ_C (101 MHz; $CDCl_3$) 156.3 (C=O), 147.3 (Ar-C), 142.9 (Ar-C), 129.3 (Ar-CH), 128.7 (Ar-CH), 125.8 (Ar-CH), 125.4 (Ar-CH), 116.5 (Ar-CH), 111.9 (Ar-CH), 66.1 (OCH_2), 53.5 (NCH_2), 51.8 (NCH_2), 50.6 (NCH_2), 26.4 (CH_2); HRMS (ESI^+): m/z calc. for $C_{18}H_{21}N_2O_2$: 319.1417, found: 319.1418 $[MH]^+$.

6-(Phenylamino)hexan-1-ol (18)

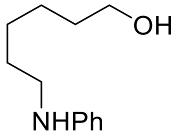

Aniline (3.50 g, 37.5 mmol) and 6-chloro-1-hexanol (0.583 g, 4.27 mmol) were added to a round bottom flask and the resulting mixture was stirred at 120 °C overnight under Ar. After total of 20 h, the reaction mixture was allowed to cool to RT, diluted with CH_2Cl_2 (50 mL) and washed with sat. aq. $NaHCO_3$. The crude product was purified by flash column chromatography (SiO_2, eluent: CH_2Cl_2 → 1:1 hexane: EtOAc) to afford the *title compound* **18** as a pale yellow oil (338 mg, 41%). R_f = 0.29 (1:1 EtOAc:n-hexane); δ_H (400 MHz; $CDCl_3$) 7.18 (2H, dd, *J* 8.6, 7.3, CH), 6.78 – 6.70 (1H, m, C(H), 6.67 (2H, dd, *J* 8.6, 7.3, CH), 3.65 (2H, t, *J* 6.6, CH_2), 3.12 (2H, t, *J* 7.2, CH_2), 1.70 – 1.62 (2H, m, CH_2), 1.62 – 1.54 (2H, m, CH_2), 1.47 – 1.37 (4H, m, 2 × CH_2); HRMS (ESI$^+$): m/z calc. for $C_{12}H_{20}NO$: 194.1539, found: 194.1541 $[MH]^+$. Spectroscopic data match those reported in the literature.[4]

(6-Hydroxyhexyl)(phenyl)carbamic chloride (20)

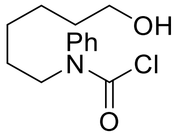

To a solution of 6-(phenylamino)hexan-1-ol **18** (56 mg, 0.290 mmol) in DCM (2.9 mL) at RT, triethylamine (60.6 µL, 0.435 mmol) and triphosgene (34.4 mg, 0.116 mmol) were added sequentially. The resulting mixture was stirred at RT for 30 min, quenched by the addition of water (20 mL), extracted with DCM (2 x 20 mL) and dried of $MgSO_4$ and concentrated under vacuum. Purification by column chromatography (5:1 → 2:1 hexane:ethyl acetate) afforded the title compound **17** as a pale brown liquid (57 mg, 77%); R_f = 0.40 (1:1 hexane:ethyl acetate); IR (thin film) ν_{max} / cm^{-1} 3386br, 2935m, 1736s, 1323m, 698w; δ_H (400 MHz; $CDCl_3$) 7.45 – 7.34 (3H, m, Ar-CH), 7.21 – 7.16 (2H, m, Ar-CH), 3.69 (2H, br t, *J* 6.8, OCH_2), 3.58 (2H, t, *J* 6.6, NCH_2), 1.65 – 1.47 (4H, m, 2 x CH_2), 1.38 – 1.27 (4H, m, 2 x CH_2); δ_C (101 MHz; $CDCl_3$) 149.3 (C=O), 141.9 (Ar-C), 129.6 (Ar-CH), 128.7 (Ar-CH), 128.4 (Ar-CH), 62.7 (OCH_2), 53.1 (NCH_2), 32.6 (CH_2), 27.5 (CH_2), 26.3 (CH_2), 25.4 (CH_2); HRMS (ESI$^+$): m/z calc. for $C_{13}H_{18}{}^{35}ClNO_2$: 278.0918, found: 278.0925 $[MNa]^+$.

3,6-Diphenyl-1,3,6-oxadiazonane-2-thione (21)

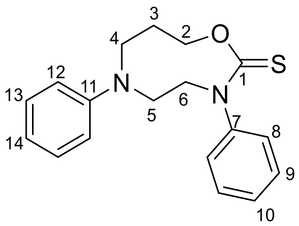

Thiophosgene (180 µL, 2.40 mmol) was added via syringe over a period of 30 seconds to a pale yellow solution of 3-(phenyl(2-(phenylamino)ethyl)amino)propan-1-ol **16** (541 mg, 2.01 mmol), anhydrous Et_3N (976 µL, 7.01 mmol) in anhydrous CH_2Cl_2 (40 mL). An immediate colour change to blood red solution was noted along with liberation of fumes (presumably $HCl_{(g)}$). The resulting mixture was stirred at RT overnight under argon and progress of the reaction was monitored by TLC. After total of 18 h, the reaction was judged to be complete, based on TLC analysis. The resulting mixture was quenched with sat. $NaHCO_{3(aq)}$ (40 mL), before being transferred to separating funnel. The aqueous phase was extracted with CH_2Cl_2 (3 × 30 mL) and combined organic layers were dried over $MgSO_4$, filtered and concentrated under reduced pressure to yield a brown oil (1.06 g). The crude product was purified by flash column chromatography (SiO_2, 30 mm column, eluent: EtOAc:*n*–hexane, 20:50) to afford the title compound **21** as a pale yellow oil (519 mg, 83%). R_f = 0.76 (50:50 EtOAc:n-hexane); IR (thin film) ν_{max} / cm^{-1}: 3039w (C–H alkyl), 2951w (C–H alkyl), 2278w (C-S thiocarbonate), 1596s (CC aromatic/aniline), 157w (CC aromatic/aniline), 1505m, 1494m, 1465m, 1412m, 1359m, 1322m, 1303m, 1261m, 1230m, 1171m, 1117m, 1037m, 987w, 973w, 926w, 860w, 837w, 812w, 745s, 692s, 617w, 570w, 544w, 500w; δ_H (500 MHz; C_6D_6) 7.24 – 7.16 (2H, m, C(8)H), 7.11 – 7.03 (2H, m, C(13)H), 7.01 – 6.94 (3H, m, C(10+12)H), 6.80 (1H, tt, *J* 7.3, 1.1, C(14)H), 6.29 – 6.22 (2H, m, C(9)H), 4.47 (2H, t, *J* 5.7, C(2)H_2), 3.44 (2H, t, *J* 5.0, C(6)H_2), 3.14 (2H, t, *J* 5.0, C(5)H_2), 2.98 – 2.93 (2H, m, C(4)H_2), 1.55 – 1.48 (2H, m, C(3)H_2); δ_C (101 MHz; C_6D_6) 190.7 (C, C1), 147.0 (C, C11), 146.2 (C, C7), 129.7 (CH, C8), 129.1 (CH, C13), 127.7 (CH, C12), 127.3 (CH, C10), 116.5 (CH, C14), 111.6 (CH, C9), 71.5 (CH_2, C2), 54.8 (CH_2, C5), 53.7 (CH_2, C6), 53.0 (CH_2, C4), 26.8 (CH_2, C3); HRMS (ESI^+): m/z calc. for $C_{18}H_{21}N_2OS$: 311.1369, found: 313.1371 $[MH]^+$, m/z calc. for $C_{18}H_{20}N_2NaOS$: 335.189, found: 335.188 $[MNa]^+$.

(6-Hydroxyhexyl)(phenyl)carbamothioic chloride (23)

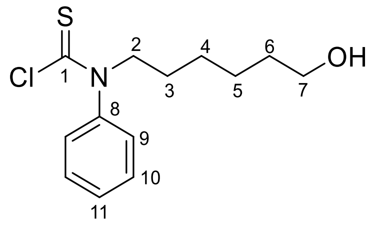

Thiophosgene (53.0 µL, 0.691 mmol) was added via syringe over a period of 35 sec. to a pale brown solution of 6-(phenylamino)hexan-1-ol **18** (113 mg, 0.576 mmol), dry Et_3N (281 µL, 2.02 mmol) in anhydrous CH_2Cl_2 (12 mL). A colour change to dark brown solution was noted along with liberation of fumes ($HCl_{(g)}$). The resulting mixture was stirred at RT overnight under Ar and progress of the reaction was monitored by TLC. After total of 18 h, the reaction was judged to be complete, based on TLC analysis. The resulting mixture was quenched with sat. $NaHCO_{3(aq)}$ (20 mL), before was transferred to separating funnel and layers were separated. The aqueous phase was extracted with CH_2Cl_2 (3 × 30 mL) and combined organic layers were dried over $MgSO_4$, filtered and concentrated under reduced pressure to yield a dark brown oil (363 mg). The crude product was purified by flash column chromatography (SiO_2, 30 mm column, eluent: EtOAc:*n*–hexane, 20:80 to 50:50) to afford the title compound **23** as a pale yellow oil (72 mg, 46%). R_f = 0.32 (50:50 EtOAc:n-hexane); IR (thin film) ν_{max} / cm^{-1}: 3393brw (H–O alcohol), 3060w (C–H alkyl), 2932w (C–H alkyl), 2859w (C–H alkyl), 1699w (C=S), 1673s, 1594w, 1491m, 1458m, 1409s, 1370w, 1244m, 1170w, 1096w, 1073w, 1055w, 1027w, 936w, 913w, 845w, 696w, 632w, 598w, 568w, 556w, 476w; δ_H (400 MHz; $(CD_3)_2CO$) 7.59 – 7.45 (3H, m, C(9+11)H), 7.41 – 7.34 (2H, m, C(10)H), 4.26 – 4.20 (2H, m, C(2)H_2), 3.49 (2H, t, *J* 6.4, C(7)H_2), 1.81 – 1.68 (2H, m, C(6)H_2), 1.55 – 1.42 (2H, m, C(3)H_2), 1.40 – 1.26 (4H, m, C(4+5)H_2); δ_C (126 MHz; $(CD_3)_2CO$) 175.1 (C, C1), 145.7 (C, C8), 130.6 (2 × CH, C9), 129.7 (CH, C11), 127.5 (2 × CH, C10), 62.3 (CH_2, C7), 59.7 (CH_2, C2), 33.5 (CH_2, C6), 27.1 (CH_2, C3), 26.6 (CH_2, C4), 26.3 (CH_2, C5); HRMS (APCI): calcd. for $C_{13}H_{19}{}^{35}ClNOS$, 272.087039. Found: $[MH]^+$, 272.086564 (1.7 ppm error).

3-(Methyl(2-(methylamino)phenyl)amino)propan-1-ol (24)

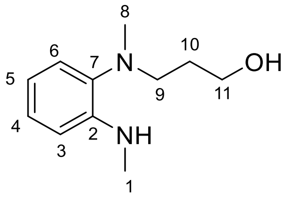

K_2CO_3 (1.30 g, 9.38 mmol) was added to a black brown suspension of N^1,N^2-diphenylethane-1,2-diamine (559 mg, 2.63 mmol), 4-bromobutan-1-ol (565 µL, 6.25 mmol) and KI (2.49 g, 15.0 mmol) in anhydrous MeCN (21.0 mL) at RT under argon. The resulting mixture was then heated to 95 °C under Ar. After 18 h, the reaction was deemed to have gone to completion by TLC. The reaction mixture was allowed to cool to RT, before being filtered through (via filter paper) and filter cake was washed with EtOAc (3 × 20 mL). The resulting filtrate was concentrated under reduced pressure to yield a black brown oil (1.86 g). The crude product was purified by flash column chromatography (SiO_2, 50 mm column, eluent: EtOAc:*n*-hexane, 50:50) to afford alcohol **24** as a brown oil (817 mg, 67%). R_f = 0.44 (EtOAc:*n*–hexane, 50:50); IR (neat) ν_{max} / cm^{-1}: 3372brw, (O–H alcohol/ N–H aniline), 3044w (C–H alkyl), 2936brw (C–H alkyl / N–H aniline), 2868w (C–H alkyl / N–H aniline), 2808w (C–H alkyl / N–H aniline), 1734w, 1598m (CC aromatic), 1509s (CC aromatic), 1476w, 1460w, 1448w, 1425w, 1410w, 1375w, 1323w, 1272w, 1244w, 1202w, 1165m, 1119w, 1056m, 1040s, 956w, 924w, 874w, 840w, 740s, 665w, 601w, 575w, 474w; δ_H (400 MHz; $CDCl_3$) 7.11 – 7.00 (2H, m, C(4+6)H), 6.70 (1H, td, *J* = 7.8, 1.4 Hz, C(5)H), 6.63 (1H, dd, *J* = 7.8, 1.4 Hz, C(3)H), 3.72 (2H, t, *J* = 6.1 Hz, C(11)H_2), 2.97 (2H, t, *J* = 6.7 Hz, C(9)H_2), 2.86 (3H, s, C(1)H_3), 2.62 (3H, s, C(8)H_3), 1.80 – 1.69 (2H, m, C(10)H_2); δ_C (101 MHz; $CDCl_3$) 144.9 (C, C2), 139.2 (C, C7), 125.4 (CH, C4), 120.3 (CH, C6), 116.6 (CH, C5), 110.0 (CH, C3), 61.7 (CH_2, C11), 53.5 (CH_2, C9), 42.0 (CH_3, C8), 30.8 (CH_3, C1), 30.1 (CH_2, C10); HRMS (ESI): calcd. for $C_{11}H_{19}N_2O$, 195.1492. Found: $[MH]^+$, 195.1494 (−1.0 ppm error).

1,8-Dimethyl-5,6,7,8-tetrahydro-1H-benzo[e][1,4,7]oxadiazecine-2,3-dione (25)

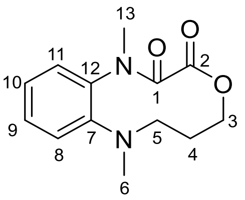

Oxalyl chloride (113 µL, 1.32 mmol) was added in a single portion via syringe to a pale yellow solution of 3-(methyl(2-(methylamino)phenyl)amino)propan-1-ol **24** (233 mg, 1.20 mmol), dry Et_3N (500 µL, 3.60 mmol) in anhydrous CH_2Cl_2 (24.0 mL) under Ar at RT. An immediate colour changed to dark brown solution was noted along with the liberation of grey fumes. The resulting solution was stirred at RT overnight under Ar and the progress of the reaction was monitored by TLC. After total of 18 h, the reaction was deemed to have gone to completion by TLC analysis. The resulting mixture was diluted with EtOAc (30 mL), before being filtered through Celite and washed with EtOAc (3 × 20 mL). The filtrated was concentrated under reduced pressure to yield a brown oil (371 mg). The crude product was purified by flash column chromatography (SiO_2, 20 mm column, eluent: EtOAc:*n*–hexane, 50:50) to afford the title compound **25** as a white solid (217 mg, 73%). R_f = 0.36 (EtOAc:*n*–hexane, 50:50); Melting Point: 134 – 136 °C (from Et_2O:EtOAc:$CHCl_3$); IR (solid state) ν_{max} / cm^{-1}: 2997w (C–H alkyl), 2955w (C–H alkyl), 2927w (C–H alkyl), 2900w, 2846m, 1718s (C=O ester), 1650s (C=O amide), 1594m (CC aromatic), 1501m (CC aromatic), 1456m (CC aromatic), 1436w, 1422w, 1390w, 1375w, 1356w, 1325w, 1302w, 1266w, 1245m, 1210m, 1178s, 1145w, 1124m, 1100s, 1071m, 1049m, 1024w, 986m, 956w, 936w, 921m, 898m, 878w, 827w, 795, 779s, 765s, 735w, 676s, 653w, 593m, 571w, 561s, 507m, 495m, 452m; δ_H (500 MHz; $CDCl_3$) 7.22 – 7.09 (3H, m, C(8+9+10)H), 6.96 (1H, dd, *J* = 7.4, 1.9 Hz, C(11)H), 4.96 (1H, br, s, C(3)**H**H'), 4.10 (1H, br, s, C(3)H**H'**), 3.41 (3H, s, C(6)H_3), 3.23 – 3.14 (2H, m, C(5)H_2), 2.49 (3H, s, C(13)H_3), 2.03 (1H, br, s, C(4)**H**H'), 1.51 (1H, br, s, C(4)H**H'**); δ_C (126 MHz; $CDCl_3$) 161.6 (NCO, C1), 160.7 (OCO, C2), 147.2 (C, C7), 138.6 (C, C12), 127.2 (CH), 125.1 (CH), 123.7 (CH), 122.7 (CH, C11), 64.6 (CH_2, C3), 53.0 (CH_2, C5), 40.9 (CH_3, C13), 35.7 (CH_3, C6), 23.8 (CH_2, C4); HRMS (ESI): calcd. for $C_{13}H_{17}N_2O_3$, 249.1234. Found: $[MH]^+$, 249.1238 (−1.7 ppm error), calcd. for $C_{13}H_{16}N_2NaO_3$, 271.1053. Found: $[MNa]^+$, 271.1055 (−0.5 ppm error).

X-ray crystallographic data for this compound can be accessed via www.ccdc.cam.ac.uk/data_request/cif (CCDC 2223454).

4-(2-(Methylamino)phenyl)but-3-yn-1-ol (S12)

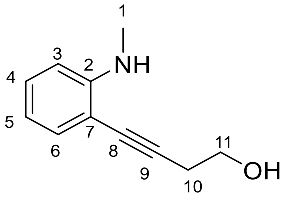

But-3-yn-1-ol (707 µL, 9.34 mmol) was added dropwise over 20 sec via syringe to a pale-yellow solution of *N*-methyl-2-iodoaniline (1.81 g, 7.79 mmol), dry Et_3N (3.26 mL, 23.4 mmol) in anhydrous DMF (26.0 mL) at RT under Ar. After 2 min, CuI (14.8 mg, 77.9 µmol) and bis(triphenylphosphine)palladium chloride (109 mg, 0.156 mmol) in a single portion. The resulting reaction mixture was stirred at RT under Ar and an immediate colour changed to yellow suspension noted. Upon stirring, the colour of the reaction mixture changed over 6 min from colourless solution (after 1 min) to pale orange (after 3 min) and finally black brown. After a total of 18 h, the reaction was deemed to have gone to completion by TLC. The resulting reaction mixture was filtered through Celite, washed with EtOAc (3 × 40 mL). The resulting filtrate was concentrated under reduced pressure to yield a dark brown oil (1.81 g). The crude product was purified by flash column chromatography (SiO_2, 50 mm column, eluent: EtOAc:*n*–hexane, 30:70) to afford alkyne **S12** as a yellow oil (1.20 g, 88%). R_f = 0.11 (30:70 EtOAc:*n*-hexane); IR (neat) ν_{max} / cm^{-1}: 3404brw (O–H alcohol/N–H aniline), 3339brw (C–H alkenyl/O–H alcohol/), 2901m (C–H alkyl), 2824 (C–H alkyl), 1600s (CC aromatic), 1574s (CC aromatic), 1509s (CC aromatic), 1461s, 1425m, 1320s, 1287s, 1167s, 1036s, 744s; δ_H (400 MHz; $CDCl_3$) 7.26 (1H, dd, *J* = 8.1, 1.8 Hz, C(6)H), 7.20 (1H, td, *J* = 8.1, 1.8 Hz, C(4)H), 6.64 – 6.59 (1H, m, C(5)H), 6.58 (1H, d, *J* = 8.1 Hz, C(3)H), 3.79 (2H, t, *J* = 6.2 Hz, C(11)H_2), 2.88 (3H, s, C(1)H_3), 2.71 (2H, t, *J* = 6.4 Hz, C(10)H_2); δ_C (101 MHz; $CDCl_3$) 149.9 (C), 132.0 (CH), 129.6 (CH), 116.3 (CH), 109.1 (CH), 107.8 (C), 92.3 (C), 79.0 (C), 61.3 (CH_2, C11), 30.4 (CH_3, C1), 24.1 (CH_2, C10); HRMS (ESI): calcd. for $C_{11}H_{14}NO$, 176.1070. Found: $[MH]^+$, 176.1072 (−1.1 ppm error), calcd. for $C_{11}H_{13}NNaO$, 198.0889. Found: $[MNa]^+$, 198.0890 (−0.2 ppm error).

4-(2-(Methylamino)phenyl)butan-1-ol (26)

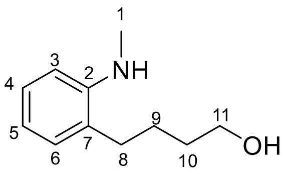

Pd/C (59.8 mg, Pd 10% on carbon) was added to the round bottom, previously purged under N_2 (for 10 min), containing 4-(2-(methylamino)phenyl)but-3-yn-1-ol **S12** (967 mg, 5.62 mmol) at RT. The alkyne was dissolved by addition of anhydouse MeOH (26.0 mL, degassed for 10 min) and the reaction vessel was evacuated under vacuum and then backfilled with H_2 (via balloon) three times, then stirred at RT under slight positive atmosphere of H_2 (via balloon) for 48 h. The reaction was then purged with N_2 for 20 min, filtered through Celite and washed with MeOH (3 × 20 mL). The resulting filtrate was concentrated under reduced pressure to yield crude product as a pale-yellow oil (1.54 g). The crude product was purified by flash column chromatography (SiO_2, 50 mm column, eluent: EtOAc:*n*–hexane, 50:50) to afford the title compound **26** as a pale orange oil (619 mg, 61%). R_f = 0.20 (50:50 EtOAc:*n*-hexane); IR (neat) ν_{max} / cm^{-1}: 3380brw (O–H alcohol/N–H aniline), 3006w, 2933w (C–H alkyl), 2864w, 2813w (C–H alkyl), 1604s (CC aromatic), 1584s (CC aromatic), 1509s (CC aromatic), 1469w, 1462w, 1426w, 1306m, 1262m, 1217w, 1168m, 11127w, 1103w, 1054m, 977w, 927w, 835w, 745s, 666w, 617w; δ_H (400 MHz; $CDCl_3$) 7.18 (1H, td, *J* = 7.7, 1.7 Hz, C(4)H), 7.06 (1H, dd, *J* = 7.4, 1.7 Hz, C(6)H), 6.71 (2H, td, *J* = 7.4, 1.2 Hz, C(5)H_2), 6.65 (1H, dd, *J* = 8.1, 1.2 Hz, C(3)H_2), 3.68 (2H, t, *J* = 6.1 Hz, C(11)H_2), 2.89 (3H, s, C(1)H_3), 2.51 (2H, t, *J* = 7.4 Hz, C(8)H_2), 1.79 – 1.56 (4H, m, C(9+10)H_2); δ_C (101 MHz; $CDCl_3$) 146.6 (C, C2), 128.8 (CH, C3), 127.1 (CH, C4), 126.1 (C, C7), 117.0 (CH, C5), 109.8 (CH, C3), 62.2 (CH_2, C11), 32.2 (CH_2, C10), 30.8 (CH_3, C1), 30.6 (CH_2, C8), 24.6 (CH_2, C9); HRMS (ESI): calcd. for $C_{11}H_{18}NO$, 180.1383. Found: $[MH]^+$, 180.1384 (−0.5 ppm error), calcd. for $C_{11}H_{17}NNaO$, 202.1202. Found: $[MNa]^+$, 202.1204 (−0.8 ppm error).

1-Methyl-5,6,7,8-tetrahydro-1H-benzo[e][1,4]oxazecine-2,3-dione (27)

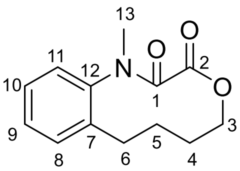

Oxalyl chloride (163 µL, 1.90 mmol) was added in a single portion via syringe to colouless solution of 4-(2-(methylamino)phenyl)butan-1-ol **26** (310 mg, 1.73 mmol), dry Et_3N (724 µL, 5.19 mmol) in anhydrous CH_2Cl_2 (35.0 mL) under Ar at RT. An immediate colour change to pale yellow solution was noted along with the liberation of grey fumes. The resulting solution was stirred at RT overnight under Ar and the progress of the reaction was monitored by TLC. Upon stirring, the colour of the reaction mixture changed to blood red and finally black brown solution. After total of 18 h, the reaction was deemed to have gone to completion by TLC analysis. The resulting mixture was diluted with EtOAc (30 mL), before being filtered through Celite and washed with EtOAc (3 × 20 mL). The filtrated was concentrated under reduced pressure to yield a pale yellow oil (217 mg). The crude product was purified by flash column chromatography (SiO_2, 40 mm column, eluent: EtOAc:*n*–hexane, 40:60) to afford the title compound **27** as a crystalline colurless solid (34.4 mg, 9%). R_f = 0.64 (EtOAc:*n*–hexane, 50:50); Melting Point: 147 – 148 °C (from *n*–hexane:Et_2O); IR (solid state) ν_{max} / cm^{-1}: 2988w, 2954m (C–H alkyl), 2875w, 1737s, (C=O ester), 1657s (C=O amide), 1600w (CC aromatic), 1488m, 1463w, 1444s, 1429w, 1393s, 1374m, 1351w, 1289m, 1219s, 1211s, 1173w, 1159w, 1130w, 1104w, 1081w, 1066m, 1046m, 1035s, 1003w, 961m, 875w, 826m, 807m, 797s, 789s, 765s, 732w, 717s, 666s, 588s, 563m, 529, 511w, 459w; δ_H (400 MHz; $CDCl_3$) 7.37 – 7.30 (1H, m, C(11)H), 7.30 – 7.20 (3H, m, C(8+9+10)H), 4.82 – 4.73 (1H, m, C(3)**H**H’), 3.93 – 3.81 (1H, m, C(3)H**H’**), 3.25 (3H, s, C(13)H_3), 3.22 – 3.12 (1H, m, C(6)**H**H’), 2.78 – 2.67 (1H, m, C(6)H**H’**), 2.26 – 2.10 (1H, m, C(5)**H**H’), 2.08 – 1.94 (1H, m, C(5)H**H’**), 1.62 – 1.46 (1H, m, C(4)**H**H’), 1.36 – 1.25 (1H, m, C(4)H**H’**); δ_C (101 MHz; $CDCl_3$) 162.5 (CO, C2), 162.4 (CO, C1), 140.4 (C, C12), 137.6 (C, C7), 130.0 (CH, C8), 129.2 (CH), 129.1 (CH), 127.8 (CH, C11), 67.5 (CH_2, C3), 36.3 (CH_3, C13), 27.2 (CH_2, C6), 26.8 (CH_2, C5), 22.1 (CH_2, C4); HRMS (ESI): calcd. for $C_{13}H_{16}NO_3$, 234.1125. Found: $[MH]^+$, 234.1130 (−2.2 ppm error), calcd. for $C_{13}H_{15}NNaO_3$, 256.0944. Found: $[MNa]^+$, 256.0945 (−0.3 ppm error).

X-ray crystallographic data for compound **27** can be accessed via www.ccdc.cam.ac.uk/data_request/cif (CCDC 2223696).

3,3'-(Benzylazanediyl)bis(propan-1-ol) (28)

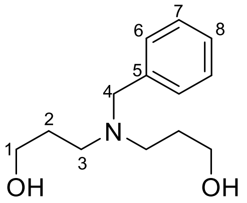

Na_2CO_3 (4.85 g, 45.8 mmol) was added in a single portion to a solution of 3-chloropropan-1-ol (3.37 mL, 40.3 mmol), benzylamine (2.00 mL, 18.3 mmol) and KI (3.04 g, 18.3 mmol) in anhydrous MeCN (61.0 mL) at RT under argon. The resulting suspension was stirred at 110 °C for 20 h under Ar, after which the reaction was deemed to have gone to completion by TLC. The reaction mixture allowed to cool to RT, before was filtered through Celite and washed with EtOAc (30 mL). The resulting filtrate was concentrated under reduced pressure to yield a pale-yellow oil (4.71 g). The crude product was purified by flash column chromatography (SiO_2, 60 mm column, eluent: MeOH:CH_2Cl_2,15:85) to afford the title compound **28** as a pale-yellow oil (3.08 g, 75%). R_f = 0.42 (MeOH:CH_2Cl_2,15:85); IR (neat) ν_{max} / cm^{-1}: 3339brw (O–H alcohol), 2943w (C–H alkyl), 2834w (C–H alkyl), 1666w (CC aromatic), 1495w, 1453m, 1371w, 1216w, 1128w, 1056s, 913w, 736m, 698s, 619w, 539w, 487w; δ_H (400 MHz; $CDCl_3$) 7.39 – 7.23 (5H, m, C(6+7+8)H), 3.69 (4H, t, J = 5.6 Hz, C(1)H_2), 3.60 (2H, s, C(4)H_2), 2.65 (4H, t, J = 6.4 Hz, C(3)H_2), 1.82 – 1.70 (4H, m, C(2)H_2); δ_C (101 MHz; $CDCl_3$) 137.9 (C, C5), 129.4 (2 × CH), 128.6 (2 × CH), 127.5 (CH, C8), 62.4 (2 × CH_2, C1), 58.9 (CH_2, C4), 52.4 (2 × CH_2, C3), 28.6 (2 × CH_2, C2); HRMS (ESI): calcd. for $C_{13}H_{22}NO_2$, 224.1645. Found: $[MH]^+$, 224.1640 (2.3 ppm error).

Benzyl-1,3,2,7-dioxathiazecane 2-oxide (29)

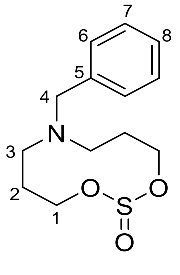

Thionyl chloride (166 µL, 2.29 mmol) was added in a single portion via syringe to a solution of 3,3'-(benzylazanediyl)bis(propan-1-ol) **28** (470 mg, 2.08 mmol), DMAP (890 mg, 7.28 mmol) in anhydrous CH_2Cl_2 (41.6 mL) at 0 °C under argon. An immediate colour changed to pale yellow solution was noted along with the liberation of grey fumes. The resulting mixture was allowed to warm gradually to RT overnight under argon and the progress of the reaction was

monitored *via* TLC, with a colour change to yellow solution observed. After total of 18 h, the reaction was deemed to have gone to completion by TLC analysis. The resulting mixture was concentrated under reduced pressure to yield a pale-yellow solid (901 mg). The crude product was purified by flash column chromatography (SiO_2, 40 mm column, eluent: EtOAc:*n*–hexane, 30:70) to afford the title compound **29** as a colourless oil (481 mg, 86%). R_f = 0.28 (EtOAc:*n*–hexane, 30:70); IR (neat) ν_{max} / cm $^{-1}$: 2931w, 2804w, 1702w, 1599w, 1495w, 1452m, 1372w, 1355w, 1293w, 1244w, 1197m, 1148w, 1071w, 1057w, 1044w, 1029w, 989w, 925m, 904s, 846m, 794w, 766m, 698s, 673w, 618w, 585w, 550w, 486w; δ_H (400 MHz; $CDCl_3$) 7.37 – 7.30 (2H, m, C(6)H), 7.29 – 7.20 (2H, m, C(7)H), 7.20 – 7.10 (1H, m, C(8)H), 4.44 (2H, ddd, *J* = 11.3, 7.5, 4.1 Hz, 2 × C(1)H**H'**), 3.93 (2H, ddd, *J* = 10.1, 5.7, 3.8 Hz, 2 × C(1)**H**H'), 3.45 (2H, s, C(4)H_2), 2.48 (4H, t, *J* = 5.9 Hz, C(3)H_2), 1.80 – 1.62 (4H, m, C(2)H_2); δ_C (101 MHz; $CDCl_3$) 139.6 (C, C5), 128.6 (CH, C6), 128.3 (CH, C7), 126.9 (CH, C8), 62.0 (CH_2, C1), 59.3 (CH_2, C4), 50.7 (CH_2, C3), 26.8 (CH_2, C2); HRMS (ESI): calcd. for $C_{13}H_{20}NO_3S$, 270.1158. Found: $[MH]^+$, 270.1160 (−0.4 ppm error), calcd. for $C_{13}H_{19}NNaO_3S$, 292.0978. Found: $[MNa]^+$, 292.0980 (−0.7 ppm error).

1,3,2-Dioxathiecane 2-oxide (31)

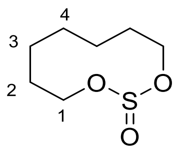

Thionyl chloride (230 µL, 3.17 mmol) was added in a single portion via syringe to a colourless solution of heptane-1,7-diol **30** (380 mg, 2.90 mmol), DMAP (1.23 g, 10.1 mmol) in anhydrous CH_2Cl_2 (58.0 mL) at 0 °C under Ar. The resulting mixture was allowed to warm gradually to RT overnight under Ar and the progress of the reaction was monitored *via* TLC, with a colour change to a pale-yellow solution observed. After a total of 18 h, the reaction was deemed to have gone to completion by TLC analysis and a colour change to creamy orange suspension was noted. The resulting mixture was concentrated under reduced pressure to yield a brown oil (731 mg). The crude product was purified by flash column chromatography (SiO_2, 40 mm column, eluent: EtOAc:*n*–hexane, 10:90 to 50:50) to afford the title compound **31** as a colourless oil (41.5 mg, 8%). R_f = 0.30 (EtOAc:*n*–hexane, 10:90); IR (neat) ν_{max} / cm $^{-1}$: 2929m (C–H alkyl), 1475w, 1456w, 1381w, 1279w, 1201s (S=O sulfite), 1063w, 1003m, 950s, 922m,

887s, 871m, 859s, 840w, 816m, 781w, 696s, 665m, 579w, 518w, 471w; δ_H (400 MHz; $CDCl_3$) 4.42 (2H, ddd, *J* = 11.0, 8.6, 3.5 Hz, 2 × C(1)H**H'**), 4.01 (2H, ddd, *J* = 11.0, 6.2, 3.7 Hz, 2 × C(1)**H**H'), 1.89 – 1.67 (4H, m, C(2)H_2), 1.67 – 1.46 (6H, m, C(3+4)H_2); δ_C (101 MHz; $CDCl_3$) 63.0 (CH_2, C1), 27.0 (CH_2, C2), 24.0 (CH_2, C4), 23.3 (CH_2, C3). HRMS (ESI): calcd. for $C_7H_{14}NaO_3S$, 201.0556. Found: $[MNa]^+$, 201.0559 (−1.6 ppm error).

7-Benzyl-2-phenyl-1,7,2,3-oxazadiphosphecane 2-oxide (32)

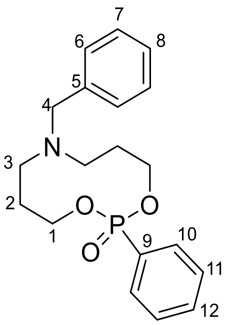

Phenylphosphonic dichloride (133 µL, 0.957 mmol) was added in a single portion via syringe to a solution of 3,3'-(benzylazanediyl)bis(propan-1-ol) **28** (178 mg, 0.797 mmol), DMAP (292 mg, 2.39 mmol) in anhydrous CH_2Cl_2 (16.0 mL) at 0 °C under Ar. The resulting mixture was allowed to warm gradually to RT overnight under Ar and the progress of the reaction was monitored *via* TLC. After total of 18 h, the reaction was deemed to have gone to completion by TLC analysis. The resulting mixture was concentrated under reduced pressure to yield a pale-yellow solid (304 mg). The crude product was purified by flash column chromatography (SiO_2, 30 mm column, eluent: EtOAc:*n*–hexane, 70:30) to afford the title compound **32** as a colourless oil (186 mg, 67%). R_f = 0.20 (EtOAc:*n*–hexane, 30:70); IR (neat) ν_{max} / cm^{-1}: 3060w, 2954w (C–H alkyl), 2803w, 2731w, 1597w (CC aromatic), 1495w (CC aromatic), 1463w, 1452w, 1439w, 1374w, 1296w, 1239s, 1131m, 1088m, 1070m, 1055m, 1018m, 981s, 915w, 894w, 874w, 815m, 743s, 725s, 707s, 695s, 664w, 620w, 558s, 533w, 514m, 472w; δ_H (400 MHz; $CDCl_3$) 7.87 – 7.77 (2H, m, C(11)H), 7.56 – 7.50 (3H, m, C(6+12)H), 7.48 – 7.41 (2H, m, C(10)H), 7.40 – 7.32 (2H, m, C(7)H), 7.28 – 7.22 (1H, m, C(8)H), 4.71 – 4.59 (2H, m, 2 × C(1)H**H'**), 4.14 – 4.01 (2H, m, 2 × C(1)**H**H'), 3.51 (2H, s, C(4)H_2), 2.71 – 2.61 (2H, m, 2 × C(3)H**H'**), 2.61 – 2.51 (2H, m, 2 × C(3)**H**H'), 1.97 – 1.81 (4H, m, C(4)H_2); δ_C (101 MHz; $CDCl_3$) 139.6 (C, C5), 132.0 (d, *J* = 3.6 Hz, CH, C12), 131.0 (d, *J* = 9.6 Hz, CH, C11), 128.9 (CH, C6), 128.6 (CH, C7), 128.1 (d, *J* = 206.3 Hz, C, C9), 127.0 (CH, C8), 64.8 (d, *J* = 6.9 Hz, CH_2, C1), 58.6 (CH_2, C4), 50.2 (CH_2, C3),

27.7 (d, *J* = 3.7 Hz, CH_2, C2); δ_P (162 MHz; $CDCl_3$) 18.2 (s, $PhPO(O)_2$); HRMS (ESI): calcd. for $C_{19}H_{25}NO_3P$, 346.1567. Found: $[MH]^+$, 346.1563 (1.1 ppm error), calcd. for $C_{19}H_{24}NNaO_3P$, 368.1386. Found: $[MNa]^+$, 368.1385 (0.4 ppm error).

Bis(7-chloroheptyl) phenylphosphonate (S13)

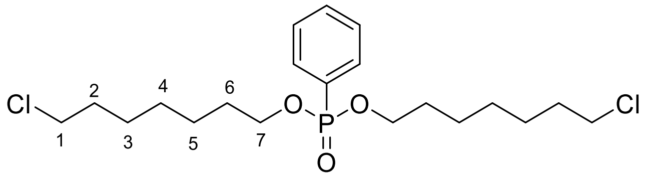

This product was formed as an unexpected side product during the control reaction from diol **30**; medium-sized ring **33** was not obtained, with compound **S13** the only product identified:

Phenylphosphonic dichloride (670 µL, 4.82 mmol) was added in a single portion via syringe to a colourless solution of heptane-1,7-diol **30** (579 mg, 4.38 mmol), DMAP (1.34 g, 11.0 mmol) in anhydrous CH_2Cl_2 (88.0 mL) at 0 °C under Ar. The resulting mixture was allowed to warm gradually to RT overnight under Ar and the progress of the reaction was monitored *via* TLC. After total of 18 h, the reaction was deemed to have gone to completion by TLC analysis, with colour change to pale yellow solution noted. The resulting mixture was concentrated under reduced pressure to yield a pale-yellow oil (847 mg). The crude product was purified by flash column chromatography (SiO_2, 40 mm column, eluent: EtOAc:*n*–hexane, 50:50) to afford the title compound **S13** as a colourless oil (379 mg, 42%). R_f = 0.30 (EtOAc:*n*–hexane, 50:50); IR (neat) ν_{max}/ cm^{-1}: 3468w, 2933w (C–H alkyl), 2858w (C–H alkyl), 1735w, 1594w (CC aromatic), 1464m, 1439m, 1390w, 1249m, 1131s, 989s, 819w, 750m, 727m, 696s, 649m, 564m, 534m; δ_H (400 MHz; $CDCl_3$) 7.84 – 7.74 (2H, m, C(10)H), 7.60 – 7.51 (1H, m, C(11)H), 7.51 – 7.42 (2H, m, C(9)H), 4.12 – 3.93 (4H, m, C(7)H_2), 3.51 (4H, t, *J* = 6.7 Hz, C(1)H_2), 1.78 – 1.59 (8H, m, C(2+6)H_2), 1.46 – 1.23 (12H, m, C(3+4+5)H_2); δ_C (101 MHz; $CDCl_3$) 132.6 (d, *J* = 3.4 Hz, CH, C11), 131.9 (d, *J* = 9.6 Hz, CH, C10), 128.6 (d, *J* = 15.2 Hz, CH, C9), 128.4 (d, *J* = 187.3 Hz, C, C8), 66.1 (d, *J* = 5.8 Hz, CH_2, C7), 45.2 (CH_2, C1), 32.6 (CH_2), 30.4 (d, *J* = 6.6 Hz, CH_2, C6), 28.5 (CH_2), 26.8 (CH_2), 25.5 (CH_2); HRMS (ESI): calcd. for $C_{20}H_{34}{}^{35}Cl_2O_3P$, 423.1617. Found: $[MH]^+$, 423.1623 (−1.4 ppm error), calcd. for $C_{20}H_{33}{}^{35}Cl_2NaO_3P$, 445.1437. Found: $[MNa]^+$, 445.1440 (−0.7 ppm error).

1,3,7-Trimethyl-1,3,7-triazecan-2-one (35)

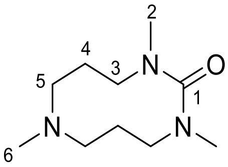

N1,N3-dimethyl-N1-(3-(methylamino)propyl)propane-1,3-diamine **34** (500 µL, 2.50 mmol) was added reaction flask (250 mL RBF) containing anhydrous DMF (50.0 mL). The resulting reaction vessel was evacuated under *vacuo* and backfilled with CO_2 (via balloon) 6 times. Next, EDC.HCl (719 mg, 3.75 mmol), HOBt (405 mg, 3.01 mmol) and dry DIPEA (1.52 mL, 3.14 mmol) were each added in single portion at RT, with a colour change to pale yellow solution noted. The resulting solution was stirred at RT overnight under a slight positive pressure of CO_2 (balloon) and the progress of the reaction was monitored via TLC. After total of 18 h, the reaction was deemed to have gone to completion by TLC analysis and colour change to pale orange solution was observed. The resulting mixture was diluted with CH_2Cl_2 (60 mL) and poured into separating funnel. The organic layer was washed sequentially with sat. $NaHCO_{3(aq)}$ (2 × 80 mL) and sat. brine (6 × 80 mL), before was dried over $MgSO_4$, filtered and concentrated under reduced pressure to yield an orange oil (510 mg). The crude product was purified by flash column chromatography (SiO_2, 40 mm column, eluent: MeOH:EtOAc, 9:1) to afford the title compound **35** as a colourless oil (434 mg, 87%). R_f = 0.04 (MeOH); IR (neat) ν_{max} / cm^{-1} 3479m, 2918m (C–H alkyl), 2846m (C–H alkyl), 2784m (C–H alkyl), 1626s (C=O urea), 1492s, 1466m, 1451m, 1411w, 1388s, 1354s, 1313w, 1290w, 1273m, 1249w, 1231m, 1201m, 1156s, 1121w, 1096m, 1064m, 1045w, 1004w, 982m, 953w, 908w, 869m, 855w, 829w, 812w, 775w, 746m, 708w, 590w, 534w, 493w, 452w; δ_H (400 MHz; $CDCl_3$) 3.34 – 3.27 (4H, m, C(3)H_2), 2.76 (6H, s, C(1)H_3), 2.35 – 2.28 (4H, m, C(5)H_2), 2.11 (3H, s, C(6)H_3), 1.62 – 1.52 (4H, m, C(4)H_3); δ_C (101 MHz; $CDCl_3$) 164.8 (CO, C1), 55.2 (CH_2, C5), 48.2 (CH_2, C3), 42.4 (CH_3, C6), 37.0 (CH_3, C2), 24.8 (CH_2, C4); HRMS (ESI): calcd. for $C_{10}H_{22}N_3O$, 200.1757. Found: $[MH]^+$, 200.1755 (1.1 ppm error), calcd. for $C_{10}H_{21}N_3NaO$, 222.1577. Found: $[MNa]^+$, 222.1576 (0.6 ppm error).

Di-*tert*-butyl heptane-1,7-diyldicarbamate (S14)

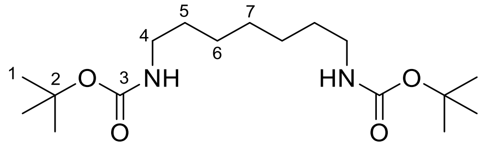

Boc_2O (7.85 g, 36.0 mmol) was added in a single portion to a solution of 1,7-diaminoheptane (2.23 g, 17.1 mmol), Et_3N (9.54 mL, 68.4 mmol) in anhydrous THF (57.0 mL) under Ar at RT. The resulting solution was stirred at RT overnight under Ar at RT and the progress of the reaction was monitored by TLC. After total of 18 h, the reaction was deemed to have gone to completion by TLC analysis. The resulting mixture was quenched with sat. $NH_4Cl_{(aq)}$ (60 mL) and poured into the separating funnel. The aqueous layer was extracted Et_2O (3 × 80 mL), before combined phases were dried over $MgSO_4$, filtered and concentrated under reduced pressure to yield a colourless oil (3.71 g). The crude product was purified by flash column chromatography (SiO_2, 70 mm column, eluent: EtOAc:*n*–hexane, 30:70) to afford the title compound **S14** as a white solid (2.50 g, 44%). R_f = 0.45 (EtOAc:*n*–hexane, 30:70); δ_H (400 MHz; $CDCl_3$) 4.96 (2H, t, *J* = 6.1 Hz, NH), 2.90 – 2.78 (4H, m, C(4)H_2), 1.28 – 1.21 (6H, m, CH_2), 1.20 (16H, s, C(1)H_3), 1.11 – 1.02 (6H, m, CH_2); δ_C (101 MHz; $CDCl_3$) 155.8 (2 × CO, C3), 78.2 (2 × C, C2), 40.1 (2 × CH_2, C4), 29.6 (2 × CH_2), 28.6 (2 × CH_2), 28.1 (2 × CH_3, C1), 26.4 (2 × CH_2); HRMS (ESI): calcd. for $C_{17}H_{34}N_2NaO_4$, 353.2411. Found: $[MNa]^+$, 353.2414 (−1.0 ppm error), calcd. for $C_{17}H_{34}KN_2O_4$, 369.2150. Found: $[MK]^+$, 369.2152 (−0.6 ppm error). The data obtained was consistent with those previously reported.[5]

N1,N7-dimethylheptane-1,7-diamine (36)

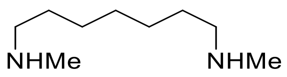

$LiAlH_4$ (2.4 M in THF, 55.0 mL, 129 mmol, 8.5 eq) was added dropwise manner via syringe pump over a period of 10 min to a solution of di-tert-butyl heptane-1,7-diyldicarbamate **S14** (4.27 g, 12.9 mmol) in anhydrous THF (43.0 mL) under Ar at 0 °C. The clear colourless solution was stirred at 0 °C for 1 h and then gradually warmed to RT, before allowed to be stirred overnight at reflux under Ar atmosphere. After total of 18 h, the reaction was deemed to have gone to completion by TLC analysis. The resulting mixture was gradually cool to RT and then further to 0 ° under Ar. The resulting milky white suspension was quenched by the sequential

addition of Et_2O (10 mL), water (15 mL), 10%–15% $NaOH_{(aq)}$ (5 mL) and water (10 mL) [Note: a solid slurry formed and agitation with a spatula was needed to help mix during the quench]. The resulting slurry was then dried over $MgSO_4$ (3.60 g) and stirred at RT for 1 h. The resulting white slurry/solid was filtered through Celite, washed with MeOH (2 × 10 ml) and the solvent was removed in *vacuo* to afford the title compound **36** as a white solid (2.50 g, 44%). IR (solid state) ν_{max} / cm $^{-1}$: 3255br (N–H, secondary amine), 1642w, 1473w, 1084w, 599w; δ_H (400 MHz; D_2O) 2.58 (4H, s, br, CH_2), 2.29 (6H, s, br, CH_2), 1.25 (4H, s, br, CH_2), 0.94 (6H, s, br, CH_2); δ_C (101 MHz; D_2O) 49.1 (CH_2), 48.9 (CH_2), 32.7 (CH_2), 27.7 (CH_2), 25.4 (CH_2); HRMS (ESI): calcd. for $C_9H_{23}N_2$, 159.1856. Found: $[MH]^+$, 159.1856 (−0.3 ppm error). The scptroscopic data are consistent with those previously reported.[6]

Methyl 2-((benzyl(4-hydroxybutyl) amino) methyl) benzoate (S15)

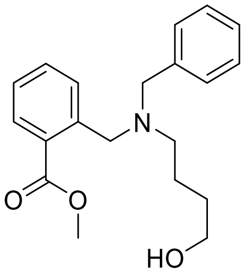

To a stirring solution of potassium carbonate (0.415 g, 3.00 mmol) in acetonitrile (5.00 mL), 4-(benzylamino) butan-1-ol (0.179 g, 1.00 mmol) was added followed by methyl 2-(bromomethyl) benzoate (0.229 g, 1.00 mmol). The reaction mixture was refluxed at 90 °C for 3 hours under argon before filtering through Celite washing with DCM, concentrating under vacuum and purifying by flash column chromatography (10:9:1 ethyl acetate:hexane:triethylamine) to afford the title compound **S15** a clear oil (0.327 g, 99%) R_f = 0.22 (14:5:1 hexane:ethyl acetate:triethylamine); ν_{max}/cm^{-1} (thin film) 3391, 2945, 2803, 1718, 14334, 1265, 1081, 909, 729; δ_H (400 MHz, $CDCl_3$) 7.75 (1H, d, *J* = 7.81 Hz, ArC**H**), 7.66 (1H, d, *J* = 7.8 Hz, ArC**H**), 7.42 (1H, t, *J* = 7.5 Hz), 7.32−7.17 (6H, m, ArC**H**), 3.93 (2H, s, ArC**H**$_2$N), 3.84 (3H, s, ArC**H**$_2$N), 3.54 (2H, s, ArC**H**$_2$N), 3.48 (2H, t, *J* = 6.2 Hz, CH_2C**H**$_2$N), 2.42 (2H, t, *J* = 6.8 Hz, CH_2C**H**$_2$OH), 1.55 (2H, quintet, *J* = 6.6 Hz, CH_2C**H**$_2$$CH_2$), 1.49−1.42 (2H, m, CH_2C**H**$_2$$CH_2$); δ_c (101 MHz, $CDCl_3$) 168.9 (**C**O), 141.1 (Ar**C**), 139.2 (Ar**C**), 131.6 (Ar**C**H), 131.0 (Ar**C**), 130.2 (ArCH), 130.1 (ArCH), 129.1 (Ar**C**H), 128.3 (Ar**C**H), 127.0 (Ar**C**H), 126.8 (Ar**C**H), 62.4 (**C**H$_2$N),

58.5 (Ar**C**H_2N), 56.6 (Ar**C**H_2N), 53.7 (CH_2**C**H_2OH), 52.1 (COO**C**H_3), 30.8 (**C**H_2), 23.4 (**C**H_2); HRMS (ESI) calcd. for $C_{20}H_{26}NO_3$ 328.1913. Found $[MH]^+$ 328.1909 (−1.22 ppm error).

7-Benzyl-3,4,5,6,7,6-hexahydro-1H-benzo[c] [1,6] oxazecin-1-one (38)

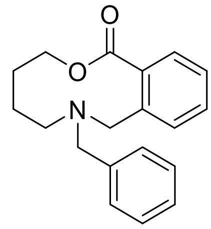

To a stirring solution of methyl 2-((benzyl (4-hydroxybutyl) amino) methyl) benzoate **S15** (0.327 g, 0.998 mmol) in tetrahydrofuran (2.20 mL), aqueous lithium hydroxide (0.5 M) was added (0.323 g, 2.7 mL, 1.35 mmol) and heated for at 50 °C for 2 hours. The solvent was removed under vacuum using dichloromethane (5 × 50 mL) to form an azeotropic mixture, to help ensure that all of the water from the hydrolysis was removed ahead of the next step. The intermediate lithium 2-(((3-hydroxy-3-phenylpropyl) (methyl)amino) methyl) benzoate was dissolved in chloroform (9.98 mL) and DIPEA (0.445 mL, 2.35 mmol) was added followed by T3P 50% w/v in ethyl acetate (0.127 g, 2.00 mmol) and stirred at room temperature for 2 hours under argon. The reaction mixture was then transferred to a separating funnel, brine (50.0 mL) was added and extracted with dichloromethane (2 × 100 mL). The combined organic phases were then dried with sodium sulphate, filtered, concentrated under vacuum and purified via flash column chromatography (15:4:1 hexane:ethyl acetate:triethylamine) to afford the title compound **38** (0.230 g, 78%) as a colourless oil. R_f = 0.49 (15:4:1 hexane:ethyl acetate:triethylamine); ν_{max}/cm^{-1} (thin film) 3063, 2954, 2799, 2251, 1717, 1452, 1294, 1266, 1131, 1091, 908, 725; δ_H (400 MHz, $CDCl_3$) 7.72 (1H, dd, *J* = 7.7 Hz, 1.4 Hz, Ar**H**), 7.39−7.18 (8H, m, Ar**H**), 5.05−2.82 (6H, bm, NC**H**$_2$, NC**H**$_2$Ar, NC**H**$_2$Ar), 2.34−2.13 (2H, m, C**H**$_2$O), 1.89−1.54 (4H, m, C**H**$_2$C**H**$_2$); δ_C (101 MHz, $CDCl_3$) 169.9 (**C**O), 140.4 (Ar**C**), 139.2 (Ar**C**), 134.0 (Ar**C**), 130.5 (Ar**C**H), 130.2 (Ar**C**H), 129.7 (Ar**C**H), 129.6 (Ar**C**H), 128.3 (Ar**C**H) 127.6(Ar**C**H), 127.0 (Ar**C**H), 65.8 (**C**H_2N), 59.8 (**C**H_2O), 57.0 (**C**H_2N), 53.2 (**C**H_2N), 25.4 (**C**H_2), 23.2 (**C**H_2); HRMS (ESI) calcd. for $C_{19}H_{22}NO_2$ 296.1651. Found $[MH]^+$ 296.1643 (−2.70 ppm error).

Methyl 2-(bromomethyl) nicotinate (S16)

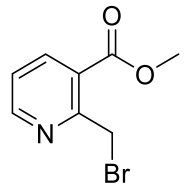

Benzene (13.0 mL) was degassed with argon for 20 minutes then added to a mixture of methyl 2-methylnicotinate (1.00 g, 6.62 mmol), *N*-bromosuccinimide (1.30 g, 7.26 mmol) and azobisisobutyronitrile (0.0540 g, 0.330 mmol) under argon. The reaction mixture was refluxed at 85 °C for 24 hours. The reaction mixture was filtered through Celite washing with DCM, before removing the solvent under vacuum and purification via flash column chromatography (1:1 hexane:ethyl acetate) to afford the title compound as a brown solid (0.289 g, 19%), R_f = 0.48 (1:1 hexane:ethyl acetate); δ_H (400 MHz, $CDCl_3$) 8.66 (1H, dd, *J* = 4.8 Hz, 1.8 Hz, Ar**H**), 8.23 (1H, dd, *J* = 7.8 Hz, 1.8 Hz, Ar**H**), 7.31−7.28 (1H, m, Ar**H**), 4.99 (2H, s, C**H**$_2$Br), 3.93 (3H, s, OC**H**$_3$). Spectroscopic data are consistent with those reported in the literature.[7]

Methyl 2-((benzyl(4-hydroxybutyl) amino) methyl) nicotinate (S17)

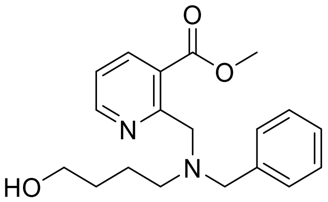

To a stirring solution of potassium carbonate (0.415 g, 3.00 mmol) in acetonitrile (5.00 mL), 4-benzylamino butan-1-ol (0.179 g, 1.00 mmol) was added followed by methyl 2-(bromo methyl) nicotinate **S16** (0.230 g, 1.00 mmol). The reaction mixture was refluxed at 90 °C for 3 hours under argon before filtering through Celite washing with DCM, concentrating under vacuum and purifying by flash column chromatography (15:4:1 dichloromethane:ethyl acetate:triethylamine) to afford the title compound as a clear oil (0.153 g, 47%) R_f = 0.47 (15:4:1 dichloromethane:ethyl acetate:triethylamine); ν_{max}/cm^{-1} (thin film) 3357, 2948, 2864, 2238, 1726, 1575, 1431, 1136, 1086, 1057, 910, 728; δ_H (400 MHz, $CDCl_3$) 8.53−8.51 (1H, m, Ar**H**), 7.94−7.91 (1H, m, Ar**H**), 7.20−7.09 (6H, m, Ar**H**), 4.06 (2H, s, ArC**H**$_2$N), 3.80 (3H, s, OC**H**$_3$), 3.56 (1H, bs, O**H**), 3.50 (2H, s, ArC**H**$_2$N), 3.42 (2H, t, *J* = 6.9 Hz, CH$_2$C**H**$_2$OH), 2.37 (2H, t, *J* = 6.1 Hz, CH$_2$C**H**$_2$N), 1.49–1.43 (2H, m, CH$_2$C**H**$_2$CH$_2$), 1.38–1.32 (2H, m, CH$_2$C**H**$_2$CH$_2$); δ_C (101 MHz, $CDCl_3$) 167.9 (**C**O), 159.9 (Ar**C**), 150.6 (Ar**C**H), 138.5 (Ar**C**), 137.9 (Ar**C**H), 129.3 (Ar**C**H), 128.1

(Ar**C**), 128.0 (Ar**C**H), 127.0 (Ar**C**H), 122.0 (Ar**C**H), 62.1 (**C**H_2OH), 59.2 (Ar**C**H_2N), 58.4 (Ar**C**H_2N), 53.4 (CH_2**C**H_2N), 52.5 (O**C**H_3), 30.9 (**C**H_2), 22.6 (**C**H_2); HRMS (ESI) calcd. for $C_{19}H_{25}N_2O_3$ 329.1865. Found $[MH]^+$, 329.1858 (−2.13 ppm error).

11-Benzyl-7,8,9,10,11,12-hexahydro-5H-pyrido[4,3-c] [1,6] oxazecin-5-one (39)

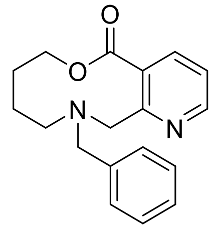

To a stirring solution of methyl 2-((benzyl (4-hydroxy butyl) amino) methyl) nicotinate **S17** (0.153 g, 0.465 mmol) in tetrahydrofuran (1.10 mL), aqueous lithium hydroxide (0.5 M) (1.12 mL, 0.558 mmol) was added and heated for at 50 °C for 3 hours. The solvent was removed under vacuum using dichloromethane (5 × 50.0 mL) to form an azeotropic mixture, to help ensure that all of the water from the hydrolysis was removed ahead of the next step. The intermediate lithium 2-((benzyl(4-oxidobutyl) amino) methyl) nicotinate was dissolved in chloroform (4.65 mL) and DIPEA (0.158 mL, 0.907 mmol) was added followed by T3P 50% w/v in ethyl acetate (0.473 g, 0.744 mmol) and stirred at room temperature for 2 hours under argon. The reaction mixture was then transferred to a separating funnel, brine (50.0 mL) was added and extracted with dichloromethane (2 × 100 mL). The combined organic phases were then dried with sodium sulphate, filtered, concentrated under vacuum and purified via flash column chromatography (8:2 dichloromethane:ethyl acetate) to afford the title compound **39** (92.6 mg, 67%) R_f = 0.67 (8:2 dichloromethane:ethyl acetate); ν_{max}/cm^{-1} (thin film) 3065, 2955, 2802, 1720, 1588, 1453, 1434, 1295, 1201, 1133, 1092, 954, 789, 729; δ_H (400 MHz, $CDCl_3$) 8.52 (1H, dd, *J* = 5.0 Hz, 1.7 Hz, ArC**H**), 7.93 (1H, dd, *J* = 7.7 Hz, 1.7 Hz, ArC**H**), 7.25−7.21 (3H, m, ArC**H**), 7.17−7.13 (3H, m, ArC**H**), 5.15−3.40 (4H, m, 2 × C**H**$_2$N), 3.40−2.98 (2H, m, C**H**$_2$N), 2.33−2.22 (2H, m, C**H**$_2$), 1.75−1.60 (4H, m, 2 × C**H**$_2$); δ_C (101 MHz, $CDCl_3$) 168.6 (**C**O), 160.9 (Ar**C**), 150.1 (Ar**C**H), 138.6 (Ar**C**), 137.3 (Ar**C**H), 129.8 (Ar**C**), 129.5 (Ar**C**H), 128.2 (Ar**C**H), 127.0 (Ar**C**H), 122.2 (Ar**C**H), 66.2 (**C**H_2N), 60.3 (**C**H_2O), 59.2 (**C**H_2N), 52.4 (**C**H_2N), 25.4 (**C**H_2), 23.1 (**C**H_2); HRMS (ESI) calcd. for $C_{18}H_{21}N_2O_2$ 297.1603. Found $[MH]^+$ 297.1588.

Methyl 3-((benzyl(4-hydroxybutyl) amino) methyl) pyrazine-2-carboxylate (S18)

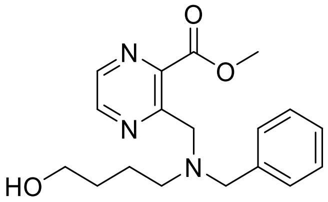

To a stirring solution of potassium carbonate (0.228 g, 1.65 mmol) in acetonitrile (5.50 mL), 4-benzylamino butan-1-ol (0.197 g, 1.10 mmol) was added followed methyl 3-(bromo methyl) pyrazine-2-carboxylate (0.255 g, 1.10 mmol). The reaction mixture was refluxed at 90 °C for 3 hours under argon before filtering through Celite, washing with DCM, concentrating under vacuum and purifying by flash column chromatography (10:9:1 hexane:ethyl acetate:triethylamine) to afford the title compound as a clear oil (0.256 g, 71%). R_f = 0.40 (10:9:1 hexane:ethyl acetate:triethylamine); ν_{max}/cm^{-1} (thin film) 3406, 2949, 2243, 1733, 1301, 1106, 908, 726; δ_H (400 MHz, $CDCl_3$) 8.55−8.54 (1H, m, ArC**H**), 8.45−8.44 (1H, m, ArC**H**), 7.22−7.14 (5H, m, 5 × ArC**H**), 4.09 (2H, s, NC**H**$_2$Ar), 3.94 (s, 3H, OC**H**$_3$), 3.57 (2H, s, NC**H**$_2$Ar), 3.50 (2H, t, *J* = 6.5 Hz, CH_2C**H**$_2$OH), 2.83 (1H, bs, O**H**), 2.48 (2H, t, *J* = 6.5 Hz, CH_2C**H**$_2$N), 1.53 (2H, quintet, *J* = 6.5 Hz CH_2C**H**$_2$$CH_2$), 1.43 (2H, quintet, *J* = 6.5 Hz CH_2C**H**$_2$$CH_2$); δ_c (101 MHz, $CDCl_3$) 166.1 (**C**O), 155.8 (Ar**C**), 145.6 (Ar**C**), 144.8 (Ar**C**H), 142.0 (Ar**C**H), 138.0 (Ar**C**), 129.2 (Ar**C**H), 128.1 (Ar**C**H), 127.1 (Ar**C**H), 62.1 (CH_2**C**H$_2$OH) 58.5 (N**C**H$_2$Ar), 58.1 (N**C**H$_2$Ar), 54.0 (CH_2**C**H$_2$N), 52.9 (O**C**H$_3$), 30.7 (CH_2**C**H$_2$$CH_2$), 22.7 ($CH_2$**C**H$_2$$CH_2$); HRMS (ESI) calcd. for $C_{18}H_{24}N_3O_3$ 330.1818. Found $[MH]^+$ 330.1814 (−1.21 ppm error).

11-Benzyl-7,8,9,10,11,12-hexahydro-5H-pyrazino[2,3-c] [1,6] oxazecin-5-one (40)

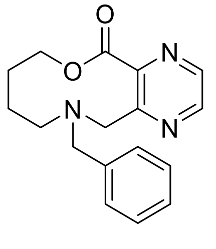

To a stirring solution of methyl 3-((benzyl (4-hydroxy butyl) amino) methyl) pyrazine-2-carboxylate **S18** (0.256 g, 0.776 mmol) in tetrahydrofuran (2.20 mL), aqueous lithium hydroxide (0.5 M) (2.20 mL, 1.01 mmol) was added and heated for at 50 °C for 3 hours. The solvent was removed under vacuum using dichloromethane (5 × 50 mL) to form an azeotropic mixture, to help ensure that all of the water from the hydrolysis was removed ahead of the

next step. The intermediate lithium 3-((benzyl(4-oxidobutyl) amino) methyl) pyrazine-2-carboxylate was dissolved in chloroform (7.76 mL) and DIPEA (0.250 mL, 1.44 mmol) was added followed by T3P 50% w/v in ethyl acetate (0.741 g, 1.16 mmol) and stirred at room temperature for 24 hours under argon. The reaction mixture was then transferred to a separating funnel, brine (50.0 mL) was added and extracted with dichloromethane (2 × 100 mL). The combined organic phases were then dried with sodium sulphate, filtered, concentrated under vacuum and purified via flash column chromatography (10:9:1 hexane:ethyl acetate:triethylamine) to afford the title compound **40** (0.109 g, 47%) R_f = 0.51 (10:9:1 hexane:ethyl acetate:triethylamine); ν_{max}/cm^{-1} (thin film) 2924, 2244, 1737, 1452, 1300, 1110, 724, 699; δ_H (400 MHz, $CDCl_3$) 8.51−8.50 (1H, m, ArC**H**), 8.49−8.47 (1H, m, ArC**H**), 7.23−7.20 (2H, m, 2× ArC**H**), 7.16−7.12 (3H, m, 3 × ArC**H**), 5.17−3.40 (4H, m, 2 × C**H**$_2$N), 3.35−3.20 (2H, m, C**H**$_2$N), 2.33−2.22 (2H, m, C**H**$_2$), 1.75−1.60 (4H, m, 2 × C**H**$_2$); δ_c (101 MHz, $CDCl_3$) 168.8 (**C**O), 156.7 (Ar**C**), 147.7 (Ar**C**H), 144.3 (Ar**C**H), 142.7 (Ar**C**H), 137.8 (Ar**C**), 129.6 (Ar**C**H), 128.3 (Ar**C**H), 127.2 (Ar**C**H), 66.6 (**C**H$_2$N), 60.7 (**C**H$_2$O), 57.8 (**C**H$_2$N), 53.5 (**C**H$_2$N), 25.3 (**C**H$_2$), 23.0 (**C**H$_2$); HRMS (ESI) calcd. for $C_{17}H_{20}N_3O_2$ 298.1556. Found $[MH]^+$ 298.1551 (−1.68 ppm error).

Methyl (*S*)-2-((2-(hydroxymethyl) pyrrolidin-1-yl) methyl) benzoate (S19)

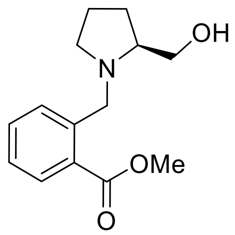

To a stirring solution of potassium carbonate (0.415 g, 3.00 mmol) in acetonitrile (5.00 mL), (S)-pyrrolidin-2-ylmethanol (0.101 g, 0.0990 mL, 1.00 mmol) was added followed by methyl 2-(bromomethyl) benzoate (0.229 g, 1.00 mmol). The reaction mixture was refluxed at 90 °C for 1 hours before filtering through Celite washing with DCM, concentrating under vacuum and purifying by flash column chromatography (1:2 hexane:ethyl acetate → ethyl acetate) to afford the title compound **S19** as a yellow oil (0.197 g, 79%), R_f = 0.20 (ethyl acetate - streaks); ν_{max}/cm^{-1} (thin film) 3505, 2950, 1717, 1269, 1079, 743; δ_H (400 MHz, $CDCl_3$) 7.76 (1H, d, *J* = 7.3 Hz, Ar**H**), 7.47–7.37 (2H, m, 2 × Ar**H**), 7.34–7.28 (1H, m, 2 × Ar**H**), 4.50 (1H, d, *J* = 13.3 Hz, ArC**H**$_a$H$_b$N), 3.90 (3H, s, OC**H**$_3$), 3.72 (1H, dd, *J* = 13.5 Hz, 3.2 Hz, C**H**$_a$H$_b$OH), 3.45 (1H, d, *J* = 13.3 Hz, ArCH$_a$**H**$_b$N), 3.42–3.36 (1H, m, CH$_a$**H**$_b$OH), 2.90 (1H, br s, O**H**), 2.83–2.79 (1H, m, C**H**$_a$H$_b$N),

2.67–2.61 (1H, m, NC**H**), 2.24–2.20 (1H, m, CH_aH_bN), 1.92–1.77 (2H, m, C**H**$_2$), 1.70–1.55 (2H, m, C**H**$_2$); δ_C (101 MHz, $CDCl_3$) 169.2 (**C**O), 140.6 (Ar**C**), 131.4 (Ar**C**H), 130.5 (Ar**C**), 130.2 (Ar**C**H), 129.9 (Ar**C**H), 127.0 (Ar**C**H), 65.3 (N**C**H), 61.9 (**C**H$_2$OH), 57.1 (Ar**C**H$_2$N), 54.9 (**C**H$_2$N), 52.2 (O**C**H$_3$), 27.1 (**C**H$_2$), 23.2 (**C**H$_2$); HRMS (ESI) calcd. for $C_{14}H_{20}NO_3$ 250.1443. Found $[MH]^+$, 250.1440 (−1.20 ppm error).

(S)-2,3,3a,4-Tetrahydro-1H-benzo[f]pyrrolo[2,1-c] [1,4] oxazocin-6(11H)-one (41)

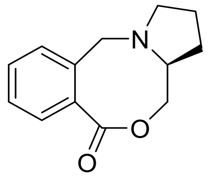

To a stirring solution of methyl (S)-2-((2-(hydroxymethyl) pyrrolidin-1-yl) methyl) benzoate **S19** (0.132 g, 0.529 mmol) in methanol (1.16 mL), aqueous lithium hydroxide (0.5 M) was added (1.16 mL, 0.582 mmol) and heated for at 50 °C for 1 hour. The solvent was removed under vacuum using chloroform (5 × 50.0 mL) to form an azeotropic mixture, to help ensure that all of the water from the hydrolysis was removed ahead of the next step. The intermediate lithium (S)-2-((2-(oxidomethyl) pyrrolidin-1-yl) methyl) benzoate was dissolved in chloroform (5.20 mL) and DIPEA (0.17 mL, 0.979 mmol) was added followed by T3P 50% w/v in ethyl acetate (0.505 g, 0.794 mmol) and stirred at room temperature for 1 hours under argon. The reaction mixture was then transferred to a separating funnel, water (20.0 mL) was added and extracted with dichloromethane (3 × 20.0 mL). The combined organic phases were then dried with magnesium sulphate, filtered, concentrated under vacuum and purified via flash column chromatography (4:1 → 1:1 hexane:ethyl acetate) to afford the title compound **41** (0.105 g, 91%) R_f = 0.45 (ethyl acetate); ν_{max}/cm^{-1} (thin film) 2962, 1715, 1271, 1093, 737; δ_H (400 MHz, $CDCl_3$) 7.34–7.29 (2H, m, 2 × Ar**H**), 7.27–7.23 (1H, m, Ar**H**), 7.10 (1H, d, *J* = 7.8 Hz, Ar**H**), 4.18 (1H, d, *J* = 16.9, ArC**H**$_a$H$_b$N), 3.98–3.95 (1H, OC**H**$_a$H$_b$CH), 3.87–3.85 (1H, OCH$_a$**H**$_b$CH), 3.81 (1H, d, *J* = 16.9, ArCH$_a$**H**$_b$N), 3.19–3.12 (1H, m, NC**H**$_a$H$_b$), 2.80–2.71 (2H, m, NC**H**$_a$H$_b$, N**H**), 2.06–1.68 (4H, m, 2 × C**H**$_2$); δ_C (101 MHz, $CDCl_3$) 173.7 (**C**O), 139.3 (Ar**C**), 130.9 (Ar**C**), 129.8 (Ar**C**H), 128.9 (Ar**C**H), 127.4 (Ar**C**H), 126.0 (Ar**C**H), 68.8 (O**C**H$_2$CH), 64.3 (N**C**H), 56.1 (Ar**C**H$_2$N), 55.2 (N**C**H$_2$), 30.1 (**C**H$_2$), 23.4 (**C**H$_2$); HRMS (ESI) calcd. for $C_{13}H_{16}NO_2$ 218.118. Found $[MH]^+$ 218.1177 (−1.84 ppm error). $[\alpha]_D^{20}$ = 35.7 (c = 1.0, DCM).

Methyl (S)-2-((2-(hydroxymethyl) pyrrolidin-1-yl) methyl) nicotinate (S20)

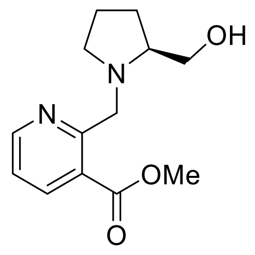

To a stirring solution of potassium carbonate (0.126 g, 0.912 mmol) in acetonitrile (6.90 mL), (S)-pyrrolidin-2-ylmethanol (0.0615 g, 0.608 mmol) was added followed by methyl 2-(bromo methyl) nicotinate (0.140 g, 0.608 mmol). The reaction mixture was refluxed at 90 °C for 3 hours under argon before filtering through Celite washing with DCM, concentrating under vacuum and purifying by flash column chromatography (16:3:1 dichloromethane:ethyl acetate:triethylamine) to afford the title compound as a dark red oil (0.128 g, 84%) R_f = 0.39 (16:3:1 dichloromethane:ethyl acetate:triethylamine); ν_{max}/cm^{-1} (thin film) 3378, 2952, 2872, 2809, 1722, 1574, 1431, 1279, 1083, 756; δ_H (400 MHz, $CDCl_3$) 8.55−8.52 (1H, m, Ar**H**), 7.97−7.94 (1H, m, Ar**H**), 7.20−7.16 (1H, m, Ar**H**), 4.39 (1H, d, *J* = 13.6 Hz, ArC$\mathbf{H}_a$$H_b$N), 3.18 (3H, s, OC$\mathbf{H}_3$), 3.79 (1H, d, *J* = 13.6 Hz, ArC$H_a$$\mathbf{H}_b$N), 3.53−3.48 (1H, m, CHC$\mathbf{H}_a$$H_b$OH), 3.33−3.28 (1H, m, CHC$H_a$$\mathbf{H}_b$OH), 2.79−2.74 (1H, m, CH_2C$\mathbf{H}_a$$H_b$N), 2.65−2.59 (1H, m, NC**H**$(CH_2)_2$), 2.37−2.30 (1H, m, CH_2C$H_a$$\mathbf{H}_b$N), 1.82−1.72 (1H, m, C$\mathbf{H}_a$$H_b$), 1.70−1.50 (1H, m, C$H_a$$\mathbf{H}_b$) 1.70−1.50 (1H, m, C$\mathbf{H}_2$); δ_C (101 MHz, $CDCl_3$) 167.9 (**C**O), 160.1 (Ar**C**), 151.1 (Ar**C**H), 137.9 (Ar**C**H), 126.7 (Ar**C**), 122.0 (Ar**C**H), 66.1 (O**C**H_3), 62.8 (**C**H_2OH), 59.1 (Ar**C**H_2N) 55.3 (**C**H_2N), 52.6 (**C**HN), 27.2 (**C**H_2), 23.3 (**C**H_2); HRMS (ESI) calcd. for $C_{13}H_{19}N_2O_3$ 251.1396. Found $[MH]^+$ 251.1391 (−1.99 ppm error).

(*S*)-7a,8,9,10-Tetrahydro-7H-pyrido[2,3-f] pyrrolo [2,1-c] [1,4] oxazocin-5(12H)-one (42)

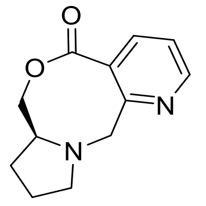

To a stirring solution of methyl (*S*)-2-((2-(hydroxymethyl) pyrrolidin-1-yl) methyl) nicotinate **S20** (0.125 g, 0.500 mmol) in tetrahydrofuran (1.20 mL), aqueous lithium hydroxide (0.5 M) (1.20 mL, 0.600 mmol) was added and heated for at 50 °C for 3 hours. The solvent was removed under vacuum using dichloromethane (5 × 50.0 mL) to form an azeotropic mixture,

to help ensure that all of the water from the hydrolysis was removed ahead of the next step. The intermediate lithium 2-((2-(2-oxidoethyl) piperidin-1-yl) methyl) nicotinate was dissolved in chloroform (5.00 mL) and DIPEA (0.162 mL, 0.925 mmol) was added followed by T3P 50% w/v in ethyl acetate (0.477 g, 0.750 mmol) and stirred at room temperature for 2 hours under argon. The reaction mixture was then transferred to a separating funnel, brine (50.0 mL) was added and extracted with dichloromethane (2 × 100 mL). The combined organic phases were then dried with sodium sulphate, filtered, concentrated under vacuum and purified via flash column chromatography (16:3:1 dichloromethane:ethyl acetate:triethylamine) to afford the title compound **42** (0.0864 g, 79%). R_f = 0.32 (16:3:1 dichloromethane:ethyl acetate:triethylamine); ν_{max}/cm^{-1} (thin film) 3424, 2926, 1708, 1422, 1275, 1094, 738; δ_H (400 MHz, $CDCl_3$) 8.52−8.50 (1H, m, Ar**H**), 7.65 (1H, d, *J* = 7.8 Hz), 7.20 (1H, m, *J* = 7.8 Hz, *J* = 4.6 Hz, Ar**H**), 4.21 (1H, d, *J* = 18.1 Hz, ArC$\mathbf{H}_a$$H_b$N), 4.02 (1H, d, *J* = 12.1 Hz, C$\mathbf{H}_a$$H_b$O), 3.96 (1H, d, *J* = 18.1 Hz, ArC$H_a$$\mathbf{H}_b$N), 3.81−3.77 (1H, m, C$H_a$$\mathbf{H}_b$O), 3.17−3.14 (1H, m, NC$\mathbf{H}_a$$H_b$$CH_2$), 2.84−2.78 (1H, m, NC$H_a$$\mathbf{H}_b$$CH_2$), 2.84−2.78 (1H, m, NC**H**$(CH_2)_2$) 2.07−1.96 (1H, m, C$\mathbf{H}_a$$H_b$), 1.94−1.82 (2H, m, C$\mathbf{H}_2$), 1.80−1.70 (1H, m, C$H_a$$\mathbf{H}_b$); δ_C (101 MHz, $CDCl_3$) 171.1 (**C**O), 159.3 (Ar**C**), 150.4 (Ar**C**H), 137.3 (Ar**C**H), 126.6 (Ar**C**), 122.1 (Ar**C**H), 68.7 (O**C**H_2), 64.3 (**C**H), 58.5 (Ar**C**H_2N), 55.1 (N**C**$H_2$$CH_2$), 29.8 (**C**$H_2$), 23.7 (**C**$H_2$); HRMS (ESI) calcd. for $C_{12}H_{15}N_2O_2$ 219.1134. Found $[MH]^+$ 219.1132 (−0.913 ppm error); $[\alpha]_D^{20}$ = 16.3 (c = 1.0, DCM).

Methyl (*S*)-3-((2-(hydroxymethyl) pyrrolidin-1-yl) methyl) pyrazine-2-carboxylate (S21)

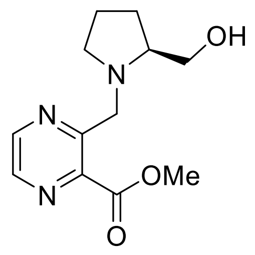

To a stirring solution of potassium carbonate (0.207 g, 1.50 mmol) in acetonitrile (5.00 mL), (S)-pyrrolidin-2-yl methanol (0.101 g, 1.00 mmol) was added followed by 3-(bromo methyl) pyrazine-2-carboxylate (0.231 g, 1.00 mmol). The reaction mixture was refluxed at 90 °C for 3 hours under argon before filtering through Celite washing with DCM, concentrating under vacuum and purifying by flash column chromatography (10:9:1 hexane:ethyl acetate:triethylamine) to afford the title compound an oil (0.198 g, 79%) R_f = 0.16 (10:9:1 hexane:ethyl acetate:triethylamine); ν_{max}/cm^{-1} (thin film) 3421, 2952, 1728, 1400, 1104, 733;

δ_H (400 MHz, $CDCl_3$) 8.44 (1H, d, *J* = 2.6 Hz, Ar**H**), 8.35 (1H, d, *J* = 2.6 Hz, Ar**H**), 4.33 (1H, d, *J* = 14.0 Hz, ArC**H**$_a$H$_b$N), 3.80 (3H, s, OC**H**$_3$), 3.60 (1H, d, *J* = 14.0 Hz, ArCH$_a$**H**$_b$N), 3.43 (1H, dd, *J* = 11.6 Hz, 3.6 Hz, CHC**H**$_a$H$_b$OH), 3.23 (1H, dd, *J* = 11.6 Hz, 3.6 Hz, CHC**H**$_a$H$_b$OH), 3.08 (1H, bs, O**H**), 2.64−2.60 (1H, m, CH$_2$C**H**$_a$H$_b$N), 2.56−2.50 (1H, m, NC**H**(CH$_2$)$_2$), 2.18−2.11 (1H, m, CH$_2$CH$_a$**H**$_b$N), 1. 73−1.63 (1H, m, C**H**$_a$H$_b$), 1.60−1.39 (1H, m, CH$_a$**H**$_b$) 1.60−1.39 (1H, m, C**H**$_2$); δ_c (101 MHz, $CDCl_3$) 165.7 (**C**O), 154.4 (Ar**C**), 144.4 (Ar**C**H), 143.9 (Ar**C**), 141.4 (Ar**C**H), 64.9 (OC**H**$_3$), 61.8 (**C**H$_2$OH), 57.1 (Ar**C**H$_2$N) 54.2 (**C**H$_2$N), 52.3 (**C**HN), 26.1 (**C**H$_2$), 22.4 (**C**H$_2$); HRMS (ESI) calcd. for $C_{12}H_{18}N_3O_3$ 252.1348. Found $[MH]^+$ 252.1348 (0.00 ppm error).

(S)-7a,8,9,10-Tetrahydro-7H-pyrazino[2,3-f] pyrrolo[2,1-c] [1,4] oxazocin-5(12H)-one (43)

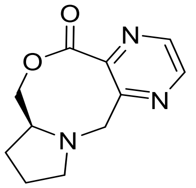

To a stirring solution of methyl (S)-3-((2-(hydroxy methyl) pyrrolidin-1-yl) methyl) pyrazine-2-carboxylate **S21** (0.153 g, 0.607 mmol) in tetrahydrofuran (2.00 mL), aqueous lithium hydroxide (0.5 M) (2.00 mL, 1.02 mmol) was added and heated at 50 °C for 3 hours. The solvent was removed under vacuum using dichloromethane (5 × 50.0 mL) to form an azeotropic mixture, to help ensure that all of the water from the hydrolysis was removed ahead of the next step. The intermediate lithium (S)-3-((2-(oxidomethyl) pyrrolidin-1-yl) methyl) pyrazine-2-carboxylate was dissolved in chloroform (7.9 mL) and DIPEA (0.250 mL, 1.45 mmol) was added followed by T3P 50% w/v in ethyl acetate (0.750 g, 1.18 mmol) and stirred at room temperature for 24 hours under argon. The reaction mixture was transferred to a separating funnel, brine (50.0 mL) was added and extracted with dichloromethane (3 × 100 mL). The combined organic phases were then dried with sodium sulphate, filtered, concentrated under vacuum and purified via flash column chromatography (10:9:1 hexane:ethyl acetate:triethylamine) to afford the title compound **43** (0.0892 g, 67%) R_f = 0.21 (10:9:1 hexane:ethyl acetate:triethylamine); ν_{max}/cm^{-1} (thin film) 3386.7, 2951.7, 1701.5, 1397.4, 1343.5, 1276.7, 1153.4, 858.4, 733.7; δ_H (400 MHz, $CDCl_3$) 8.48−8.47 (2H, m, 2 × ArC**H**), 4.24 (1H, dd , *J* = 18.4 Hz, *J* = 2.1 Hz, ArC**H**$_a$H$_b$N), 4.10−4.05 (1H, m, C**H**$_a$H$_b$O), 3.98 (1H, dd , *J* = 18.4 Hz, *J* = 2.1 Hz, ArCH$_a$**H**$_b$N), 3.79−3.74 (1H, m, CH$_a$**H**$_b$O), 3.17−3.11 (1H, m, NC**H**$_a$H$_b$CH$_2$), 2.91−2.85 (1H, m, NC**H**(CH$_2$)$_2$), 2.84−2.78 (1H, m, NCH$_a$**H**$_b$CH$_2$), 2.08−1.97 (1H, m,

C**H**$_a$H$_b$), 1.92 −1.81 (1H, m, CH$_a$**H**$_b$), 1.92 −1.81 (1H, m, C**H**$_a$H$_b$), 1.78−1.69 (1H, m, CH$_a$**H**$_b$); δ_c (101 MHz, $CDCl_3$) 168.3 (**C**O), 155.0 (Ar**C**), 144.8 (Ar**C**), 144.7 (Ar**C**H), 143.4 (Ar**C**H), 69.3 (O**C**H$_2$), 64.3 (**C**H), 57.0 (Ar**C**H$_2$N), 55.3 (N**C**H$_2$CH$_2$), 30.0 (**C**H$_2$), 22.9 (**C**H$_2$); HRMS (ESI) calcd. for $C_{11}H_{14}N_3O_2$ 220.1086. Found $[MH]^+$ 220.1087 (0.454 ppm error); $[\alpha]_D^{20}$ = −22.0 (c = 1.0, DCM).

Methyl 2-((2-(2-hydroxyethyl) piperidin-1-yl) methyl) benzoate (S22)

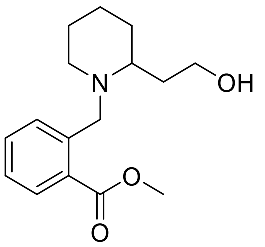

To a stirring solution of potassium carbonate (0.416 g, 3.00 mmol) in acetonitrile (5.00 mL), 2-(piperidin-2-yl) ethan-1-ol (0.129 g, 1.00 mmol) was added followed by methyl 2-(bromomethyl) benzoate (0.229 g, 1.00 mmol). The reaction mixture was refluxed at 90 °C for 1 hours before filtering through Celite washing with DCM, concentrating under vacuum and purifying by flash column chromatography (1:2 hexane:ethyl acetate → ethyl acetate) to afford the title compound as a yellow oil (0.262 g, 94%) R_f = 0.2 (ethyl acetate - streaks); ν_{max}/cm–1 (thin film) 3417, 2932, 1721, 1264, 1085, 739; δ_H (400 MHz, $CDCl_3$); 7.79 (1H, d, *J* = 7.8 Hz, ArC**H**), 7.44–7.40 (2H, m, 2 × ArC**H**), 7.30–7.24 (1H, m, ArC**H**), 4.26 (1H, d, *J* = 13.7, ArC**H$_a$**H$_b$N), 3.94 (1H, d, *J* = 13.7, ArCH$_a$**H$_b$**N), 3.87 (3H, s, OC**H**$_3$), 3.77–3.70 (1H, m, C**H$_a$**H$_b$OH), 3.63–3.55 (1H, m, CH$_a$**H$_b$**OH), 2.91–2.84 (1H, m, C**H$_a$**H$_b$N), 2.81–2.75 (1H, m, C**H**N), 2.33–2.29 (1H, m, CH$_a$**H$_b$**N), 2.05–2.29 (1H, m, C**H$_a$**H$_b$), 1.85−1.78 (1H, m, CH$_a$**H$_b$**), [1.69–1.32 (6H, m, 3 × C**H**$_2$)]; δ_c (101 MHz, $CDCl_3$); 168.6 (**C**O), 140.8 (Ar**C**), 131.7 (Ar**C**H), 130.6 (Ar**C**), 130.4 (Ar**C**H), 130.2 (Ar**C**H), 126.9 (Ar**C**H), 62.1 (**C**H$_2$OH), 58.8 (**C**HN), 55.0 (Ar**C**H$_2$N), 52.2 (O**C**H$_3$), 48.4 (**C**H$_2$N), 31.6 (**C**H$_2$), 27.1 (**C**H$_2$), 22.4 (**C**H$_2$), 21.7 (**C**H$_2$); HRMS (ESI) calcd. for $C_{16}H_{24}NO_3$ 278.1756. Found $[MH]^+$, 278.1745 (1.5 ppm error).

8,8a,9,10,11,12-Hexahydro-7H-benzo[g]pyrido[2,1-d] [1,5] oxazonin-5(14H)-one (44)

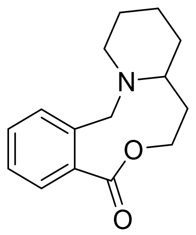

To a stirring solution of methyl 2-((2-(2-hydroxyethyl) piperidin-1-yl) methyl) benzoate (**S22**) (0.245 g, 0.883 mmol) in methanol (1.94 mL), aqueous lithium hydroxide (0.5 M) was added (1.94 mL, 0.971 mmol) and heated for at 50 °C for 1 hours. The solvent was removed under vacuum using chloroform (5 × 50.0 mL) to form an azeotropic mixture, to help ensure that all of the water from the hydrolysis was removed ahead of the next step. The intermediate lithium 2-((2-(2-oxidoethyl) piperidin-1-yl) methyl) benzoate was dissolved in chloroform (8.8 mL) and DIPEA (0.285 mL, 1.63 mmol) was added followed by T3P 50% w/v in ethyl acetate (0.834 g, 1.32 mmol) and stirred at room temperature for 1 hours under argon. The reaction mixture was then transferred to a separating funnel, water (20.0 mL) was added and extracted with dichloromethane (3 × 20.0 mL). The combined organic phases were dried with magnesium sulphate, filtered, concentrated under vacuum and purified via flash column chromatography (3:1 → 1:1 hexane:ethyl acetate) to afford the title compound as a colourless oil (0.154 g, 71%) as a roughly 6:1 mixture of rotamers R_f = 0.50 (ethyl acetate); ν_{max}/cm^{-1} (thin film) 2932, 1727, 1121, 1086, 734; δ_H (400 MHz, $CDCl_3$); 7.55 (1H, d, *J* = 7.3, ArC**H**, both rotamers), 7.33–7.27 (2H, m, 2 × ArC**H**, both rotamers), 7.14 (1H, d, *J* = 7.8 Hz, ArC**H**, both rotamers), 5.32–5.25 (1H, m, $C\mathbf{H_a}H_bO$, major rotamer), 5.15–5.09 (1H, m, $C\mathbf{H_a}H_bO$, minor rotamer), 4.65 (1H, d, *J* = 13.7, $ArC\mathbf{H_a}H_bN$, major rotamer), 4.26 (1H, d, *J* = 14.2, $ArC\mathbf{H_a}H_bN$, minor rotamer), 4.09–4.06 (1H, m, $ArCH_a\mathbf{H_b}N$, minor rotamer), 3.99 (1H, d, *J* = 13.7 $CH_a\mathbf{H_b}O$, major rotamer), 3.40 (1H, d, *J* = 14.2, $ArCH_a\mathbf{H_b}N$, minor rotamer), 2.99−2.00 (1H, m, C**H**N, minor rotamer), 2.74 (1H, d, *J* = 13.7, $ArCH_a\mathbf{H_b}N$, major rotamer), [2.70−2.48 (3H, m, $C\mathbf{H_2}N$, C**H**N), both rotamers], [16H in total 2.16−1.21 (m) both rotamers]; δ_c (101 MHz, $CDCl_3$); data for the major rotamer only 172.0 (**C**O), 143.0 (Ar**C**), 133.5 (Ar**C**), 130.1 (Ar**C**H), 128.9 (Ar**C**H), 128.6 (Ar**C**H), 127.1 (Ar**C**H), 63.1 ($O\mathbf{C}H_2CH_2$), 58.4 (N**C**H), 57.2 ($Ar\mathbf{C}H_2N$), 53.5 ($\mathbf{C}H_2N$), 32.1 ($\mathbf{C}H_2$), 31.2 ($\mathbf{C}H_2$), 25.7 ($\mathbf{C}H_2$), 24.6 ($\mathbf{C}H_2$); HRMS (ESI): calcd. for $C_{15}H_{20}NO_2$, 246.1494. Found $[MH]^+$, 246.1481 (−5.28 ppm error).

Methyl 2-((2-(2-hydroxyethyl) piperidin-1-yl) methyl) nicotinate (S23)

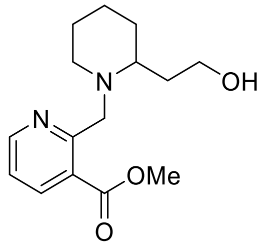

To a stirring solution of potassium carbonate (0.415 g, 3.00 mmol) in acetonitrile (5.00 mL), 2-(piperidin-2-yl) ethan-1-ol (0.129 g, 1.00 mmol) was added followed by methyl 2-(bromo methyl) nicotinate **S16** (0.230 g, 1.00 mmol). The reaction mixture was refluxed at 90 °C for 3 hours under argon before filtering through Celite washing with DCM, concentrating under vacuum and purifying by flash column chromatography (15:4:1 dichloromethane:ethyl acetate:triethylamine) to afford the title compound as a dark red oil (0.153 g, 55%) R_f = 0.47 (15:4:1 dichloromethane:ethyl acetate:triethylamine); ν_{max}/cm^{-1} (thin film) 3365, 2935, 2238, 1725, 15779, 1430, 1283, 1087, 728; δ_H (400 MHz, $CDCl_3$) 8.50 (1H, m, Ar**H**), 7.96 (1H, d, *J* = 7.8 Hz, Ar**H**), 7.14 (1H, m,Ar**H**), 4.74 (1H, bs, O**H**), 4.23 (1H, d, *J* = 14.1 Hz ArC**H**$_a$H$_b$N), 4.02 (1H, d, *J* = 14.1 Hz ArCH$_a$**H**$_b$N), 3.80 (3H, s, OC**H**$_3$), 3.66−3.60 (1H, m, C**H**$_a$H$_b$OH), 3.49−3.43 (1H, m, CH$_a$**H**$_b$OH), 2.76−2.67 (1H, m, NC**H**(CH$_2$)$_2$), 2.76−2.67 (1H, m, NC**H**$_a$H$_b$), 2.31−2.25 (1H, m, NCH$_a$**H**$_b$), 1.97−1.88 (1H, m, C**H**$_a$H$_b$CH$_2$OH), 1.72−1.64 (1H, m, C**H**$_a$H$_b$), 1.53−1.46 (1H, m, C**H**$_a$H$_b$), 1.53−1.46 (1H, m, C**H**$_a$H$_b$), 1.43−1.33 (2H, m, CH$_a$**H**$_b$CH$_2$OH), 1.43−1.33 (1H, m, CH$_a$**H**$_b$), 1.28−1.20 (1H, m, CH$_a$**H**$_b$); δ_C (101 MHz, $CDCl_3$) 167.4 (**C**O), 160.3 (Ar**C**), 150.9 (Ar**C**), 138.1 (ArCH), 127.1 (Ar**C**H), 121.8 (Ar**C**H), 61.5 (**C**H$_2$OH), 58.0 (N**C**H), 57.3 (Ar**C**H$_2$N), 52.4 (O**C**H$_3$), 48.6 (**C**H$_2$N), 31.7 (CH**C**H$_2$CH$_2$), 27.1 (**C**H$_2$), 22.7 (**C**H$_2$), 21.2 (**C**H$_2$); HRMS (ESI) calcd. for $C_{15}H_{23}N_2O_3$ 279.1709. Found [MH]$^+$ 279.1708 (−0.358 ppm error).

8,8a,9,10,11,12-Hexahydro-7H-dipyrido[2,1-d:2',3'-g] [1,5] oxazonin-5 (14H)-one (45)

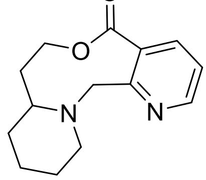

To a stirring solution of methyl 2-((2-(2-hydroxy ethyl) piperidin-1-yl) methyl) nicotinate **S23** (0.129 g, 0.463 mmol) in tetrahydrofuran (1.40 mL), aqueous lithium hydroxide (0.5 M) (1.39

mL, 0.695 mmol) was added and heated for at 50 °C for 3 hours. The solvent was removed under vacuum using dichloromethane (5 × 50.0 mL) to form an azeotropic mixture, to help ensure that all of the water from the hydrolysis was removed ahead of the next step. The intermediate lithium 2-((2-(2-oxidoethyl) piperidin-1-yl) methyl) nicotinate was dissolved in chloroform (4.60 mL) and DIPEA (0.140 mL, 0.851 mmol) was added followed by T3P 50% w/v in ethyl acetate (0.446 g, 0.700 mmol) and stirred at room temperature for 2 hours under argon. The reaction mixture was then transferred to a separating funnel, brine (50 mL) was added and extracted with with dichloromethane (2 × 100 mL). The combined organic phases were then dried with sodium sulphate, filtered, concentrated under vacuum and purified via flash column chromatography (8:2 dichloromethane:ethyl acetate) to afford the title compound (0.0869 g, 76%), which existed as a 4:1 mixture of rotamers in solution in $CDCl_3$. R_f = 0.77 (16:3:1 dichloromethane:ethyl acetate:triethylamine); ν_{max}/cm^{-1} (thin film) 3431, 2930, 1727, 1429, 1282, 1122, 1087, 792, 707; δ_H (400 MHz, $CDCl_3$) 8.48−8.44 (1H, m, Ar**H**), 7.83−7.76 (1H, m, Ar**H**), 7.22−7.17 (1H, m, Ar**H**), 5.27 (1H, t, *J* = 12.2 Hz, C**H**$_a$H$_b$OH) $_{major}$, 5.09 (1H, t, *J* = 11.2 Hz, C**H**$_a$H$_b$OH) $_{minor}$, 4.73−4.68 (1H, m, ArC**H**$_a$H$_b$N) $_{major}$, 4.33−4.29 (1H, m, ArC**H**$_a$H$_b$N) $_{minor}$, 4.09−4.04 (1H, m, CH$_a$**H**$_b$OH) $_{minor}$, 4.01−3.95 (1H, m, CH$_a$**H**$_b$OH) $_{major}$, 3.71−3.57 (1H, m, ArCH$_a$**H**$_b$N) $_{minor}$, 3.08−3.03 (1H ,m, ArCH$_a$**H**$_b$N) $_{major}$, 2.99−2.91 (1H, m, C**H**) $_{minor}$, 2.67−2.61 (1H, m, C**H**) $_{major,}$ 2.67−2.61 (1H, m, C**H**$_a$H$_b$) $_{major}$, 2.52−2.42 (1H, m, C**H**$_a$H$_b$N) $_{both\ rotamers}$, 2.17−2.06 (1H, m C**H**$_a$H$_b$) $_{minor}$, 2.02−1.95 (1H, m, CH$_a$**H**$_b$N) $_{major}$, 1.83−1.07 (7H in total) $_{both\ rotamers}$,[1.82−1.79 (2H, m) $_{minor}$, 1.68−1.56 (2H, m) $_{major}$, 1.50−1.19 (5H, m) $_{both\ rotamers}$, 1.15−1.07 (1H, m) $_{minor}$]; δ_C (101 MHz, $CDCl_3$) 170.4 (**C**O) $_{major}$, 170.1 (**C**O) $_{minor}$, 164.3 (Ar**C**) $_{major}$, 163.5 (Ar**C**) $_{minor}$, 149.6 (Ar**C**H) $_{major}$, 149.4 (Ar**C**H) $_{minor}$, 136.2 (Ar**C**H) $_{major}$, 136.1 (Ar**C**H) $_{minor}$, 129.4 (Ar**C**) $_{major}$, 121.9 (Ar**C**H) $_{major}$, 121.8 (Ar**C**H) $_{minor}$, 66.2 (O**C**H$_2$) $_{minor}$, 63.1 (O**C**H$_2$) $_{major}$, 62.7 (**C**H) $_{minor}$, 59.3 (Ar**C**H$_2$N) $_{major}$, 58.2 (**C**H) $_{major}$, 53.8 (N**C**H$_2$CH$_2$) $_{major}$, 50.1 (Ar**C**H$_2$N) $_{minor}$, 49.2 (N**C**H$_2$CH$_2$) $_{minor}$, 31.7 (**C**H$_2$) $_{major}$, 31.1 (**C**H$_2$) $_{minor}$, 31.0 (**C**H$_2$) $_{major}$, 28.0 (**C**H$_2$) $_{minor}$ 25.8 (**C**H$_2$) $_{minor}$, 25.5 (**C**H$_2$) $_{major}$, 13.4 (**C**H$_2$) $_{major}$, 18.6 (**C**H$_2$) $_{minor}$; HRMS (ESI) calcd. for $C_{14}H_{19}N_2O_2$ 247.1447. Found $[MH]^+$ 247.1441 (−2.43 ppm error).

Methyl 3-((2-(2-hydroxyethyl) piperidin-1-yl) methyl) pyrazine-2-carboxylate (S24)

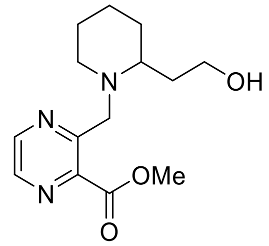

To a stirring solution of potassium carbonate (0.138 g, 1.00 mmol) in acetonitrile (5.00 mL), 2-(piperidin-2-yl) ethan-1-ol (0.089 g, 0.690 mmol) was added followed by 3-(bromo methyl) pyrazine-2-carboxylate (0.160 g, 0.690 mmol). The reaction mixture was refluxed at 90 °C for 3 hours under argon before filtering through Celite washing with DCM, concentrating under vacuum and purifying by flash column chromatography (10:9:1 hexane:ethyl acetate:triethylamine) to afford the title compound an oil (0.112 g, 58%) R_f = 0.17 (10:9:1 hexane:ethyl acetate:triethylamine); ν_{max}/cm^{-1} (thin film) 3394, 2936, 2241, 1732, 1301, 1105, 729; δ_H (400 MHz, $CDCl_3$) 8.53–8.52 (1H, m, Ar**H**), 8.43–8.42 (1H, m, Ar**H**), 4.21 (1H, d, *J* = 14.7 Hz, $ArC\mathbf{H}_aH_bN$), 3.99 (1H, d, *J* = 14.7 Hz, $ArCH_a\mathbf{H}_bN$), 3.90 (3H, s, $OC\mathbf{H}_3$), 3.67–3.61 (1H, m, $C\mathbf{H}_aH_bOH$), 3.53–3.47 (1H, m, $CH_a\mathbf{H}_bOH$), 2.71–2.67 (1H, m $NC\mathbf{H}(CH_2)_2$), 2.71–2.67 (1H, m, $NC\mathbf{H}_aH_b$), 2.25–2.19 (1H, m, $NCH_a\mathbf{H}_b$), 1.93–1.88 (1H, m, $C\mathbf{H}_aH_bCH_2OH$), 1.68–1.62 (1H, m, $C\mathbf{H}_aH_b$), 1.68–1.62 (1H, m, $C\mathbf{H}_aH_b$), 1.54–1.42 (2H, m, $CH_a\mathbf{H}_bCH_2OH$), 1.54–1.42 (1H, m, $C\mathbf{H}_aH_b$), 1.54–1.42 (1H, m, $C\mathbf{H}_aH_b$), 1.37–1.28 (1H, m, $CH_a\mathbf{H}_b$), 1.37–1.28 (1H, m, $CH_a\mathbf{H}_b$), 1.37–1.28 (1H, m, $CH_a\mathbf{H}_b$); δ_C (101 MHz, $CDCl_3$) 166.0 (**C**O), 155.9 (Ar**C**), 150.9 (Ar**C**), 145.3 (Ar**C**H), 144.9 (Ar**C**), 142.0 (Ar**C**H), 61.2 (**C**H_2OH), 58.6 (N**C**H), 56.6 (Ar**C**H_2N), 53.0 (O**C**H_3), 49.2 (**C**H_2N), 31.6 (CH**C**H_2CH_2), 27.7 (**C**H_2), 23.1 (**C**H_2), 21.3 (**C**H_2); HRMS (ESI) calcd. for $C_{14}H_{22}N_3O_3$ 280.1661. Found $[MH]^+$ 280.1652 (−3.2 ppm error).

8,8a,9,10,11,12-Hexahydro-7H-pyrazino[2,3-g] pyrido[2,1-d] [1,5] oxazonin-5(14H)-one (46)

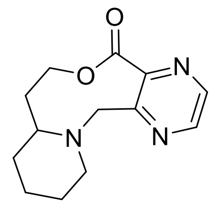

To a stirring solution of methyl 3-((2-(2-hydroxyethyl) piperidin-1-yl) methyl) pyrazine-2-carboxylate **S24** (0.112 g, 0.400 mmol) in tetrahydrofuran (1.84 mL), aqueous lithium hydroxide (0.5 M) (1.84 mL, 0.920 mmol) was added and heated for at 50 °C for 3 hours. The solvent was removed under vacuum using dichloromethane (5 × 50.0 mL) to form an azeotropic mixture, to help ensure that all of the water from the hydrolysis was removed ahead of the next step. The intermediate lithium 3-((2-(2-oxidoethyl) piperidin-1-yl) methyl) pyrazine-2-carboxylate was dissolved in chloroform (4.0 mL) and DIPEA (0.130 mL, 0.600 mmol) was added followed by T3P 50% w/v in ethyl acetate (0.382 g, 0.600 mmol) and stirred at room temperature for 2 hours under argon. The reaction mixture was then transferred to a separating funnel, brine (50 mL) was added and extracted with dichloromethane (2 × 100 mL). The combinedorganic phases were then dried with sodium sulphate, filtered, concentrated under vacuum and purified via flash column chromatography (10:9:1 hexane:ethyl acetate:triethylamine) to afford the title compound **46** (84.6 mg, 86%), which existed as a 4:1 mixture of rotamers when dissolved in $CDCl_3$. R_f = 0.40 (10:9:1 hexane:ethyl acetate:triethylamine); ν_{max}/cm^{-1} (thin film) 2931, 1744, 1454, 1291, 1104, 708; δ_H (400 MHz, $CDCl_3$) 8.45−8.43 (1H, m, Ar**H**)$_{both\ rotamers}$, 8.41−8.39 (1H, m, Ar**H**)$_{both\ rotamers}$, 5.34 (1H, t, *J* = 11.8 Hz, OC**H**$_a$H$_b$CH$_2$)$_{major}$, 5.15 (1H, t, *J* = 11.8 Hz, OC**H**$_a$H$_b$CH$_2$)$_{minor}$, 4.68 (1H, d, *J* = 15.0 Hz, ArC**H**$_a$H$_b$N)$_{major}$, 4.31 (1H, d, *J* = 15.0 Hz, ArC**H**$_a$H$_b$N)$_{minor}$, 4.09 (1H, d, *J* = 11.8 Hz, OCH$_a$**H**$_b$CH$_2$)$_{minor}$, 4.05−3.99 (1H, m, OCH$_a$**H**$_b$CH$_2$)$_{major}$, 3.65 (1H, d, *J* = 15.0 Hz, ArCH$_a$**H**$_b$N)$_{minor}$, 3.06 (1H, d, *J* = 14.7 Hz, ArCH$_a$**H**$_b$N)$_{major}$, 2.99−2.91 (1H, m, C**H**)$_{minor}$ 2.69−2.58 (1H, m, C**H**)$_{major}$, 2.69−2.58 (1H, m, C**H**$_a$H$_b$)$_{major}$, 2.56−2.49 (1H, m, CH$_a$**H**$_b$N)$_{minor}$, 2.42−2.34 (1H, m, C**H**$_a$H$_b$N)$_{both\ rotamers}$, 2.13−2.06 (1H, m, C**H**$_a$H$_b$)$_{minor}$, 1.99 (1H, t, *J* = 10.5 Hz, CH$_a$**H**$_b$N)$_{major}$, 1.80−1.09 (7H in total)$_{both\ rotamers}$ [1.80−1.72 (1H, m)$_{minor}$, 1.69−1.58 (2H, m)$_{major}$, 1.52−1.48 (1H, m)$_{minor}$, 1.44−1.09 (5H, m)$_{both\ rotamers}$]; δ_C (101 MHz, $CDCl_3$) 167.4 (**C**O)$_{major}$, 167.0 (**C**O)$_{minor}$, 160.0 (Ar**C**)$_{minor}$, 159.2(Ar**C**)$_{major}$, 147.8 (Ar**C**)$_{minor}$, 147.4 (Ar**C**)$_{major}$, 143.7 (Ar**C**H)$_{major}$, 143.5

(Ar**C**H)$_{minor}$, 142.4 (Ar**C**H)$_{major}$, 142.3 (Ar**C**H)$_{minor}$, 66.4 (O**C**H$_2$)$_{minor}$, 63.3 (O**C**H$_2$)$_{major}$, 62.6 (N**C**H)$_{minor}$, 58.3 (N**C**H)$_{major}$, 58.1 (Ar**C**H$_2$N)$_{major}$, 54.05 (N**C**H$_2$CH$_2$)$_{major}$, 50.3 (Ar**C**H$_2$N)$_{minor}$, 48.2 (N**C**H$_2$CH$_2$)$_{minor}$, 31.5 (**C**H$_2$)$_{major}$, 31.0 (**C**H$_2$)$_{minor}$, 30.8 (**C**H$_2$)$_{major}$, 28.0 (**C**H$_2$)$_{minor}$, 25.6 (**C**H$_2$)$_{minor}$, 25.4 (**C**H$_2$)$_{major}$, 24.2 (**C**H$_2$)$_{major}$, 18.4 (**C**H$_2$)$_{minor}$; HRMS (ESI) calcd. for $C_{13}H_{18}N_3O_2$ 248.1399. Found $[MH]^+$ 248.1395 (−1.61 ppm error).

Methyl 2-(((2-hydroxyethyl)thio)methyl)benzoate (S25)

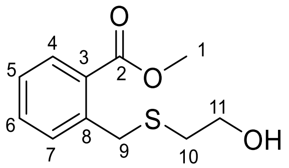

K_2CO_3 (1.26 g, 8.60 mmol) was added to pale yellow solution of 2-hydroxy-1-ethanethiol (300 µL, 4.30 mmol) and methyl 2-(bromomethyl) benzoate (1.53 g, 6.68 mmol) in anhydrous DMF (14 mL) at RT. The resulting turquoise suspension was then heated to 70 ^oC, whereupon a colour change to grey-purple was noted. After 12 h, the reaction was deemed to have gone to completion by TLC analysis. The reaction mixture was allowed to cool to RT and quenched by addition of H_2O (30 mL). The diluted milky/white reaction mixture was transferred into separating funnel containing EtOAc (30 mL) and the aqueous layer was extracted with EtOAc (3 × 20 mL). The combined organic layers were washed sequentially with 1 M $HCl_{(aq)}$ (3 × 10 mL), sat. $NaHCO_{3(aq)}$ (1 × 20 mL) and sat. brine (2 × 20 mL), before being dried over $MgSO_4$, filtered and concentrated under reduced pressure to yield pale yellow oil (2.80 g). The crude product was purified by flash column chromatography (SiO_2, 60 mm column, eluent: EtOAc:*n*–hexane, 40:60) to afford the title compound as a pale yellow oil (1.32 g, 90%). R_f = 0.29 (50:50 EtOAc:*n*–hexane); IR (thin film) ν_{max} / cm^{-1}: 3410vbrm (O–H alcohol), 2999w (C–H alkyl), 2950w (C–H alkyl), 2876w (C–H alkyl), 1714vs (C=O aryl ester), 1600w (CC aromatic), 1576w (CC aromatic), 1488w, 1434m, 1292m, 1262vs, 1190w, 1164w, 1122m, 1077s, 1045s, 1011m, 963w, 891w, 840w, 801w, 767w, 714s, 663m, 580w, 470w; δ_H (400 MHz; $CDCl_3$) 7.92 (1H, dd, *J* 7.7, 1.7, C(4)H), 7.45 (1H, td, *J* 7.7, 1.7, C(7)H), 7.35 – 7.30 (2 H, m, C(4+5)H), 4.14 (2H, s, C(9)H$_2$), 3.92 (3H, s, C(1)H$_3$), 3.70 (2H, t, *J* 5.8, C(11)H$_2$), 2.67 (2H, t, *J* 5.8, C(10)H$_2$); δ_C (101 MHz; $CDCl_3$) 167.9 (C, C2), 140.2 (C, C8), 132.2 (CH, C7), 131.3 (CH, C5), 131.2 (CH, C6), 129.4 (C, C3), 127.4 (CH, C4), 60.5 (CH$_2$, C11), 52.3 (CH$_3$, C1), 35.0 (CH$_2$, C10), 34.1 (CH$_2$, C9); HRMS (ESI$^+$): m/z calc. for $C_{11}H_{14}NaO_3S$: 249.0556, found: 249.0556 $[M+Na]^+$.

2-(((2-Hydroxyethyl)thio)methyl)benzoic acid (S26)

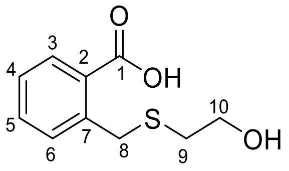

$NaOH_{(aq)}$ (4.0 M, 11 mL) was added dropwise over a period of 5 minutes to solution of alcohol **S25** (400 mg, 2.25 mmol) in MeOH (11 mL). A colour change to pale yellow solution was noted immediately. Upon further addition, the appearance of the reaction mixture changed to a milky white suspension. The resulting clear colourless solution was stirred at RT for 16 h, after which time the reaction was deemed to have gone to completion by TLC (complete consumption of alcohol **S25** was noted). The resulting mixture was then acidified to pH 2.0 with 1M $HCl_{(aq)}$ (20 mL) and poured into separating funnel containing CH_2Cl_2 (20 mL). The aqueous layer was extracted with CH_2Cl_2 (3 × 20 mL), before combined organic phases were dried over $MgSO_4$, filtered and concentrated under reduced pressure to afford carboxylic acid **S26** as a white solid (377 mg, 79%). R_f = 0.27 (10:90 MeOH:CH_2Cl_2 (10 mL) + 3 drops of AcOH); Melting Point: 60 – 63 °C (from *n*–hexane:CH_2Cl_2 , 5:2); IR (solid state) ν_{max} / cm^{-1}: 3273vbrm (O–H alcohol), 3064vbrw (C–H aryl), 2954vbrm (C–H alkyl, O–H carboxylic acid), 2919vbrm (C–H alkyl, O–H carboxylic acid), 2815vbrm (C–H alkyl), 2645m (C–H alkyl), 2519w, 1678vs (C=O aryl carboxylic acid), 1598w (CC aromatic), 1573m (CC aromatic), 1483w, 1447w, 1407s, 1300w, 1290w, 1268vs, 1201w, 1179w, 1164w, 1142w, 1077w, 1049m, 1004m, 967w, 923m, 857w, 836w, 806w, 767m, 730m, 710m, 689w, 660m, 580w, 545w, 479w; δ_H (400 MHz; CD_3OD) 7.90 (1 H, dd, *J* 7.8, 1.5, C(3)H), 7.49 – 7.42 (1 H, m, C(5)H), 7.41 – 7.36 (1 H, m, C(6)H), 7.36 – 7.29 (1 H, m, C(4)H), 4.17 (2 H, s, C(8)H_2), 4.16 (1 H, s, OH), 3.60 (2 H, t, *J* 6.7, C(10)H_2), 2.55 (2 H, t, J 6.7, C(9)H_2); δ_C (101 MHz; CD_3OD) 170.8 (C, C1), 142.1 (C, C7), 132.8 (CH, C5),132.3 (CH, C3), 132.2 (CH, C6), 131.4 (C, C2), 128.1 (CH, C4), 62.3 (CH_2, C10), 35.0 (CH_2, C8), 34.70 (CH_2, C9); HRMS ($APCI^+$): m/z calc. for $C_{10}H_{13}O_3S$: 213.057992, found: 213.058412 $[M+H]^+$.

3,4-Dihydrobenzo[*f*][1,4]oxathiocin-1(6*H*)-one (47)

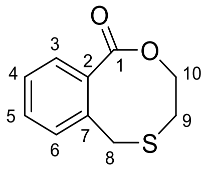

EDC.HCl (332 mg, 1.64 mmol) was added to solution of carboxylic acid **S26** (194 mg, 0.912 mmol), HOBt (191 mg, 1.37 mmol) and anhydrous DIPEA (400 µL, 2.28 mmol) in anhydrous DMF (9 mL). A colour change to pale yellow was noted immediately. After total of 18 h of stirring at RT, the reaction was deemed to have to completion by TLC analysis. The resulting mixture was diluted with EtOAc (30 mL) and poured into separating funnel, where the organic phase was washed sequentially with 1M $HCl_{(aq)}$ (2 × 20 mL), sat. $NaHCO_{3(aq)}$ (1 × 20 mL) and sat. brine (2 × 20 mL). The resulting organic layer was dried over $MgSO_4$, filtered and concentrated under reduced pressure to yield a clear colourless oil (289 mg). The crude product was purified by flash column chromatography (SiO_2, 30 mm column, eluent: EtOAc:*n*–hexane, 20:80) to afford the title compound **47** as a white crystalline solid (156 mg, 88%). R_f = 0.77 (50:50 EtOAc:*n*–hexane); Melting Point: 75 – 77 °C (from *n*–hexane); IR (solid sate) ν_{max} / cm^{-1}: 3072w (C–H aryl), 3031w (C–H aryl), 2961w (C–H alkyl), 2899w (C–H alkyl), 1771m (C=O aryl ester), 1599m (CC aromatic), 1484w (CC aromatic), 1462w, 1445w, 1414w, 1404w, 1361m, 1296m, 1226m, 1203w, 1167w, 1157w, 1113m, 1102m, 1082s, 1047m, 1033m, 995w, 955w, 933w, 893w, 897w, 864w, 839w, 825w, 769m, 755s, 705m, 696m, 671w, 656m, 579w, 542w, 489m, 470w; δ_H (400 MHz; $CDCl_3$) 7.67 (1 H, dd, *J* 7.7, 1.7, C(3)H), 7.39 (1 H, td, *J* 7.7, 1.7, C(5)H), 7.33 (1 H, td, J 7.5, 1.4, C(4)H), 7.12 (1 H, dd, J 7.0, 1.0, C(6)H), 4.70 (2 H, br s, C(10)H_2), 4.07 (2 H, s, C(8)H_2), 3.18 (2 H, s, C(9)H_2); δ_C (101 MHz; $CDCl_3$) 172.6 (C, C1), 142.1 (C, C7), 131.9 (CH, C5), 130.8 (C, C2), 130.2 (CH, C3) 129.8 (CH, C6), 127.8 (CH, C4), 67.5 (CH_2, C10), 39.7 (CH_2, C8), 39.0 (CH_2, C9); HRMS (ESI^+): m/z calc. for $C_{10}H_{10}NaO_2S$: 217.0294, found: 217.0295 $[M+Na]^+$.

X-ray crystallographic data for compound **47** can be accessed via www.ccdc.cam.ac.uk/data_request/cif (CCDC 2221558)

Synthesis of methyl 5-fluoro-2-(((3-hydroxypropyl)thio)methyl)benzoate (S29)

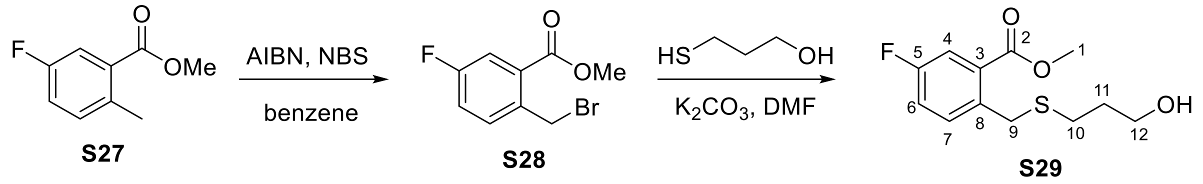

AIBN (46.7 mg, 0.287 mmol) was added to a yellow suspension of methyl ester **S27** (483 mg, 2.87 mmol) and *N*-bromosuccinimide (665 mg, 3.73 mmol) in anhydrous benzene (25 mL) at RT. The resulting suspension was heated to reflux, whereupon a colour changed to pale yellow was noted. After 48 h, the reaction mixture allowed to cool to RT and evaporated to dryness, before diluted with 30 mL of EtOAc. The diluted mixture was transferred to separating funnel, where organic layer was washed sequentially with 1 M $HCl_{(aq)}$ (2 × 30 mL), sat. $NaHCO_{3(aq)}$ (2 × 20 mL) and sat. brine (2 × 20 mL), dried over $MgSO_4$, filtered and concentrated under reduced pressure to afford bromide as orange oil (854 mg). The resulting bromide **S28** was directly used in the next reaction step without further purification.

K_2CO_3 (651 mg, 3.83 mmol) was added to a yellow solution of 3-mercapto-1-propanol (110 µL, 1.91 mmol) and the crude sample of bromide **S28** prepared as above (assumed to be 2.87 mmol of **S28**) in anhydrous DMF (10 mL) at RT. The resulting suspension was then heated to 70 °C, whereupon a colour change to pale yellow was noted. Upon further heating, the colour of the reaction suspension changed to dark brown. After 16 h, the reaction was deemed to have gone to completion by TLC. The reaction mixture was cooled to RT, before diluted with EtOAc (30 mL). The diluted solution was poured into a separating funnel and the organic layer was washed sequentially with H_2O (1 × 30 mL), 1M $HCl_{(aq)}$ (3 × 20 mL), sat. $NaHCO_{3(aq)}$ (3 × 20 mL) and sat. brine (2 × 20 mL), before being dried over $MgSO_4$, filtered and concentrated under reduced pressure to yield brown oil (850 mg). The crude product was purified by flash column chromatography (SiO_2, 50 mm column, eluent: EtOAc:*n*–hexane, 50:50) to the title compound **S29** as a pale yellow oil (600 mg, 81%). R_f = 0.32 (20:80 EtOAc:*n*-hexane);IR (thin film) ν_{max} / cm^{-1}: 3425mvbr (O–H alcohol), 2952w (C–H alkyl), 1724vs (C=O aryl ester), 1611w (CC aromatic), 1583w (CC aromatic), 1495m (CC aromatic), 1436m, 1374w, 1308w, 1268s, 1241m, 1209vs, 1183m, 1124w, 1066m, 1045m, 983m, 911w, 8334w, 793w, 772w, 722w, 676w, 635w, 608w, 481w; δ_H (400 MHz; $CDCl_3$) 7.61 (1H, dd, *J* 9.4, 2.9, C(4)H), 7.33 (1H, dd, *J* 8.3, 5.5, C(7)H), 7.15 (1H, td, *J* 8.3, 2.9, C(6)H), 4.10 (2H, s, C(9)H_2), 3.91 (3H, s, C(1)H_3), 3.71 (2H, t, *J* 6.2, C(12)H_2), 2.55 (2H, t, *J* 7.1, C(10)H_2), 1.84 – 1.77 (2H, m, C(11)H_2); δ_F (376 MHz;

$CDCl_3$) −114.4 (q, *J* = 7.6 Hz, C(5)F); δ_C (101 MHz; $CDCl_3$) 166.7 (d, *J* 2.7, C, C2), 161.2 (d, J 247, C, C5), 136.5 (d, *J* 3.7, C, C8), 132.7 (d, *J* 7.6, CH, C7), 131.0 (d, *J* 7.4, CH, C3), 118.8 (d, *J* 21.1, CH, C6), 118.0 (d, *J* 23.2, CH, C4), 61.4 (CH_2, C12), 52.4 (CH_3, C1), 33.8 (CH_2, C9), 31.80 (CH_2, C11), 28.4 (CH_2, C10); HRMS (ESI^+): m/z calc. for $C_{12}H_{15}FNaO_3S$: 281.0618 found: 281.0614 $[M+Na]^+$.

5-Fluoro-2-(3-hydroxypropylsulfanylmethyl)benzoic acid (S30)

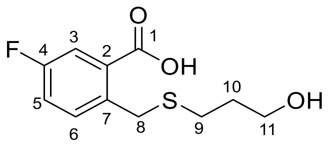

$NaOH_{(aq)}$ (4.0 M, 4.50 mL) was added dropwise over a period of 2 min to solution of ester **S29** (241 mg, 1.01 mmol) in MeOH (3.50 mL). A colour change to yellow solution was noted immediately during dropwise addition. Upon further addition, the appearnce of the reaction mixture changed to pale yellow. The reaction mixture was stirred at RT for 16 h, before the reactions was deemed to have gone to completion by TLC (complete consumption of ester **S29** was noted). The resulting solution was then acidified to pH 2.0 with 1M $HCl_{(aq)}$ (20 mL) and transferred to separating funnel. The aqueous layer was extracted with CH_2Cl_2 (3× 30 mL), the combined organic phases were dried over $MgSO_4$, filtered and concerted under reduced pressure to yield a crude product as a pale yellow oil (265 mg). The crude product was purified by flash column chromatography (SiO_2, 30 mm column, eluent: MeOH:CH_2Cl_2, 10:90) to afford a carboxylic acid **S30** as a white solid (200 mg, 88%). R_f = 0.23 (10:90 MeOH:CH_2Cl_2, (10 mL) + 3 drops of AcOH); Melting Point: 80– 83 °C; IR (solid state) ν_{max} / cm^{-1}: 3473vbrm (O–H alcohol), 2926vbrm C–H alkyl, O–H carboxylic acid), 2923vbrw (C–H alkyl, O–H carboxylic acid), 2793vbrm (C–H alkyl, O–H carboxylic acid), 2565vbrm (C–H alkyl, O–H carboxylic acid),1698vs (C=O aryl carboxylic acid), 1603w (CC aromatic), 1584m (CC aromatic), 1494m (CC aromatic), 1429w, 1417w, 1402w, 1382w, 1335w, 1308m, 1291m, 1259s, 1215vs, 1182s, 1065w, 1037m, 1007s, 938m, 890m, 877m, 813w, 799w, 782w, 752m, 721w, 689w, 666w; δ_H (400 MHz; CD_3OD) 7.58 (1H, dd, *J* = 9.4, 2.9 Hz, C(3)H), 7.41 (1H, td, *J* = 8.5, 5.5 Hz, C(5)H), 7.20 (1H, td, *J* = 8.5, 2.9 Hz, C(6)H), 4.13 (2H, s, C(8)H_2), 3.58 (2H, t, *J* = 6.3 Hz, C(11)H_2), 2.49 (2 H, t, *J* = 7.3 Hz, C(9)H_2), 1.74 (2H, app. p, *J* = 6.3 Hz, C(10)H_2); δ_C (101 MHz; CD_3OD) 170.0 (C, C1), 162.6 (d, *J* = 245.2, C, C4), 138.0 (d, *J* = 3.5, C, C7), 134.0 (d, *J* = 7.7, CH, C6, overlapping peak

with C, C2), 119.1 (d, *J* = 21.4, CH, C5), 118.5 (d, *J* 23.4, CH, C3), 61.5 (CH_2, C11), 34.2 (CH_2, C8), 33.3 (CH_2, C10), 28.9 (CH_2, C9); δ_F (376 MHz; $CDCl_3$) – 116.74 (td, *J* = 10.5, 9.3, 3.4 Hz, C(4)F); HRMS (ESI$^+$): m/z calc. for $C_{11}H_{13}FNaO_3S$: 267.0462 found: 267.0461 $[M+Na]^+$.

10-Fluoro-4,5-dihydro-3*H*-benzo[*g*][1,5]oxathionin-1(7*H*)-one (48)

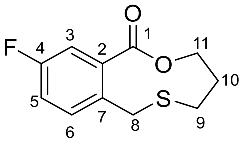

Dry DIPEA (260 µL, 1.49 mmol) was added dropwise over 3 min to pale yellow solution of carboxylic acid **S30** (146 mg, 0.596 mmol) and HOBt (96.5 mg, 0.715 mmol) in anhydrous DMF (6 mL). A colour change to yellow was noted immediately. The resulting mixture was stirred for 2 min at RT, after which time EDC.HCl (173 mg, 0.895 mmol) was added and the resulting mixture stirred at RT. After 18 h, the reaction was deemed to have gone to completion by TLC. The resulting mixture was diluted with EtOAc (20 mL), before was transferred to separating funnel and organic phase was washed sequentially with 1M $HCl_{(aq)}$ (3 × 20 mL), sat. $NaHCO_3$ (3 × 20 mL) and sat. brine (3 × 20 mL). The organic layer was dried over $MgSO_4$, filtered and concentrated under reduced pressure to yield a pale yellow solid (144 mg). The crude product was purified by flash column chromatography (SiO_2, 20 mm column, eluent: EtOAc:*n*–hexane, 30:70) to afford the title compound **48** as a white solid (108 mg, 80%). R_f = 0.65 (50:50 EtOAc:*n*–hexane); Melting Point: 106 – 107 °C (from *n*–hexane); IR (solid state) ν_{max} / cm^{-1}: 2992w (C–H alkyl), 2962w (C–H alkyl), 2924w (C–H alkyl), 1710s (C=O aryl ester), 1673w (CC aromatic), 1605m (CC aromatic), 1585m (CC aromatic), 1495m (CC aromatic), 1453m, 1428vs, 1380w, 1350w, 1306m, 1271vs, 1232m, 1218s, 1196m, 1152w, 1134m, 1126s, 1076m, 1063m, 1045m, 965s, 932m, 886s, 879s, 835m, 817m, 797s, 777m, 762w, 717s, 693m, 672m, 637m, 579s, 525s, 504w, 477s; δ_H (400 MHz; $CDCl_3$) 7.54 (1H, dd, *J* = 8.4, 2.8 Hz, C(3)H), 7.12 (1H, dd, *J* = 8.4, 5.3 Hz, C(5)H), 7.04 (1H, td, *J* = 8.4, 2.8 Hz, C(6)H), 4.63 (2H, br t, *J* = 5.9 Hz, C(11)H_2), 4.09 (2H, s, C(8)H_2), 2.89 – 2.85 (2H, m, C(9)H_2), 2.21 – 2.14 (2H, m, C(10)H_2); δ_C (101 MHz; $CDCl_3$) 166.7 (d, *J* = 2.8 Hz, C, C1), 161.5 (d, *J* = 247.3 Hz, C, C4), 138.0 (d, *J* = 3.4 Hz, C, C7), 133.5 (d, *J* = 7.6 Hz, C, C2), 131.5 (d, *J* = 7.6 Hz, CH, C6), 118.0 (d, *J* = 15.1 Hz, CH, C5), 117.79 (d, *J* = 12.8 Hz, CH, C3), 66.0 (CH_2,C11), 40.1 (CH_2,C8), 34.9 (CH_2,C9), 29.8 (CH_2,C10); δ_F (376 MHz; $CDCl_3$) –114.4 (td, *J* = 8.4, 5.3, C(4)F); HRMS (ESI$^+$): m/z calc. for $C_{11}H_{11}FNaO_2S$: 249.0356 found: 249.0359 $[M+Na]^+$.

X-ray crystallographic data for this compound can be accessed via www.ccdc.cam.ac.uk/data_request/cif (CCDC 2221239)

Methyl 2-bromo-6-(bromomethyl)benzoate (S31)

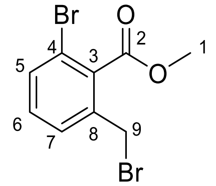

Ph_2O_2 (108 mg, 0.758 mmol) was added to a yellow suspension of NBS (085 mg, 4.55 mmol) and 2-bromo-6-methyl benzoate (0.869 mg, 3.79 mmol) in degassed (for 10 min) anhydrous benzene (19 mL). The resulting sunflower yellow suspension was then heated to 95 °C, whereupon a colour change to pale yellow suspension was noted. After 20 h, the reaction wasn't deemed to have gone to completion by TLC analysis (consumption of stating material wasn't observed). The reaction mixture was allowed to cool to RT, before concentrated under reduced pressure and diluted with EtOAc (30 mL). The diluted mixture was poured into a separating funnel, where organic phase was washed sequentially with 1M $HCl_{(aq)}$ (3 × 20 mL), sat. $NaHCO_{3(aq)}$ (1 × 10 mL) and sat. brine (3 × 10 mL). The organic layer was dried over $MgSO_4$, filtered and concentrated under reduced pressure to yield a pale–yellow oil (1.40 g). The crude product was purified by flash column chromatography (SiO_2, 60 mm column, eluent: EtOAc:*n*–hexane, 10:90) to afford the title compound **S31** as a colourless oil (752 mg, 65%). R_f = 0.40 (20:80 EtOAc:*n*–hexane); IR (thin film) ν_{max} / cm^{-1}: 2951w (C–H alkyl), 1728vs (C=O aryl ester), 1592w (CC aromatic), 1565w (CC aromatic), 1443m (CC aromatic), 1428m, 1278vs, 1214m, 1182m, 1154w, 1116m, 1102m, 1058m, 953w, 888m, 855w, 826w, 790m, 769w, 730m, 698m, 627m, 576m, 557w, 490w; δ_H (400 MHz; $CDCl_3$) 7.54 (1 H, dd, *J* 7.8, 1.1 C(5)H), 7.38 (1 H, dd, *J* 7.8, 1.1, C(7)H), 7.26 (1H, t, *J* 7.8, C(6)H), 4.48 (2 H, s, C(9)H_2), 4.00 (3 H, s, C(1)H_3); δ_C (101 MHz; $CDCl_3$) 167.2 (C, C2), 137.1 (C, C4), 135.4 (C, C3), 132.9 (CH, C5), 131.1 (CH, C6), 129.1 (CH, C7), 120.1 (C, C8), 52.8 (CH_3, C1), 29.6 (CH_2, C9); HRMS (ESI$^+$): m/z calc. for $C_9H_8{}^{79}Br_2NaO_2$: 328.8783, found: 328.8785 $[MNa]^+$.

Methyl 2-bromo-6-(((3-hydroxypropyl)thio)methyl)benzoate (S32)

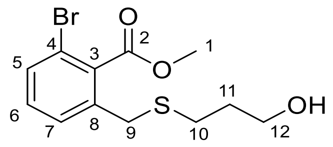

K_2CO_3 (424 mg, 2.46 mmol) was added to a pale yellow solution of 3-mercapto-1-propanol (110 µL, 1.23 mmol) and methyl 2-bromo-6-(bromomethyl)benzoate **S31** (493 mg, 1.60 mmol) in anhydrous DMF (12 mL) at RT. The resulting suspension was then heated to 70 °C, whereupon a colour change to milky/white suspension was noted. Upon further heating, the colour of the reaction suspension changed to cloudy white. After 16 h, the reaction was deemed to have gone to completion by TLC, with a colour change to sunflower yellow was observed. The mixture allowed to cool to RT and quenched by addition of H_2O (30 mL). The resulting milky white suspension was poured into separating funnel containing EtOAc (20 mL) and aqueous layer was extracted with EtOAc (2 × 20 mL). The combined organic layers were washed sequentially with 1M $HCl_{(aq)}$ (2 × 20 mL), sat. $NaHCO_{3(aq)}$ (2 × 20 mL), H_2O (2 × 20 mL) and sat. brine (2 × 20 mL), before being dried over $MgSO_4$, filtered and concentrated under reduced pressure to yield brown oil (622 mg). The crude product was purified by flash column chromatography (SiO_2, 40 mm column, eluent: EtOAc:*n*–hexane, 50:50) to afford the title compound **S32** as a pale yellow oil (490 mg, 96%). R_f = 0.32 (50:50 EtOAc:*n*–hexane); IR (thin film) ν_{max} / cm^{-1}: 3393brm (O–H alcohol), 2949m (C–H alkyl), 2878rm (C–H alkyl) 1726vs (C=O aryl ester), 1591w (CC aromatic), 1562w (CC aromatic), 1440m, 1428m, 1280vs, 1184w, 1134m, 1102m, 1058s, 954w, 914w, 884w, 826w, 793w, 750w, 729m, 696w, 595w, 666w, 562w, 487w; δ_H (400 MHz; $CDCl_3$) 7.48 (1H, dd, *J* 7.9, 1.1, C(5)H), 7.35 (1H, d, *J* 7.9, C(7)H), 7.22 (1H, t, *J* 7.9, C(6)H), 3.96 (3H, s, C(1)H_3), 3.74 (2H, s, C(9)H_2), 3.69 (2H, app. q, *J* 5.5, C(12)H_2), 2.52 (2H, t, *J* 7.1, C(10)H_2), 1.83 – 1.72 (2H, m, C(11)H_2); δ_C (101 MHz; $CDCl_3$) 168.1 (C, C2), 138.4 (C, C4), 135.3 (C, C8), 131.6 (CH, C5), 130.8 (CH, C6), 128.8 (CH, C7), 119.9 (C, C3), 61.4 (CH_2, C12), 52.7 (CH_3, C1), 34.0 (CH_2, C11), 28.2 (CH_2, C10); HRMS (ESI^+): m/z calc. for $C_{12}H_{16}{}^{79}BrO_3S$: 318.9998, found: 318.9995 $[MH]^+$, m/z calc. for $C_{12}H_{15}{}^{79}BrNaO_3S$: 340.9817, found: 340.9815 $[MNa]^+$.

2-Bromo-6-(((3-hydroxypropyl)thio)methyl)benzoic acid (S33)

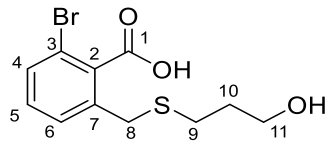

$NaOH_{(aq)}$ (4.0 M, 7.50 mL) was added dropwise over a period of 3 min to solution of alcohol **S32** (490 mg, 1.53 mmol) in MeOH (7.50 mL). A colour change to milky white suspension was noted immediately during dropwise addition. The resulting suspension was then heated to 60 °C, whereupon a colour change to clear colorless solution was noted. After 16 h, the reaction was deemed to have gone to completion by TLC analysis (complete consumption of alcohol **S32** was noted). The reaction mixture was allowed to cool to RT and acidified to pH 2.0 by addition of 1M $HCl_{(aq)}$ (20 mL). The acidified solution was poured into separating funnel and aqueous layer was extracted with CH_2Cl_2 (4 × 20 mL). The combined organic layers were dried over Na_2SO_4, filtered and concentrated under reduced pressure to yield a colourless oil (666 mg). The crude product was purified by flash column chromatography (SiO_2, 30 mm column, eluent: MeOH:CH_2Cl_2, 10:90) to afford the title compound **S33** as a white solid (393 mg, 84%); R_f = 0.48 (20:80 MeOH:CH_2Cl_2, (10 mL) + 3 drops of AcOH); IR (solid state) ν_{max} / cm^{-1}: 3301vbrm (O–H alcohol, O–H carboxylic acid), 2929vbrm (C–H alkyl, O–H carboxylic acid), 2618vbrw (C–H alkyl, O–H carboxylic acid), 2496vbr (C–H alkyl, O–H carboxylic acid), 1695vs (C=O aryl carboxylic acid), 1561s (CC aromatic), 1439s (CC aromatic), 1391m, 1274m, 1181w, 1144w, 1108w, 1040m, 911w, 751w, 678w, 597w, 575w; δ_H (400 MHz; $CDCl_3$) 7.47 (1H, dd, *J* 7.9, 1.1 C(4)H), 7.36 (1H, dd, *J* 7.9, 1.1, C(6)H), 7.21 (1H, t, *J* 7.9, C(5)H), 6.70 (1H, br s, OH), 3.81 (2H, s, C(8)H_2), 3.74 (2H, t, *J* 6.5, C(11)H_2), 2.54 (2H, t, *J* 7.0, C(9)H_2), 1.81 (2H, q, *J* 6.5, C(10)H_2); δ_C (101 MHz; $CDCl_3$) 173.3 (C, C1) 138.6 (C, C3), 137.3 (CH, C7), 131.3 (CH, C4), 129.9 (CH, C5), 129.0 (CH, C6), 118.7 (C, C2), 61.7 (CH_2, C11), 34.0 (CH_2, C8) 31.5 (CH_2, C10), 28.6 (CH_2, C9); HRMS (ESI^+): m/z calc. for $C_{11}H_{13}{}^{79}BrNaO_3S$: 326.9661, found: 326.9664 [MNa]

11-Bromo-4,5-dihydro-3*H*-benzo[*g*][1,5]oxathionin-1(7*H*)-one (49a)

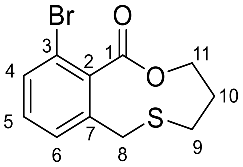

EDC.HCl (462 mg, 2.09 mmol) was added to solution of carboxylic acid **S33** (355 mg, 1.16 mmol), HOBt (239 mg, 1.74 mmol) and anhydrous DIPEA (510 µL, 2.91 mmol) in anhydrous DMF (12 mL). An immediate colour change color to pale yellow observed. After total of 16 h of stirring at RT, the reaction deemed to have to completion by TLC analysis with colour change to brown noted. The resulting mixture was diluted with EtOAc (30 mL). The milky white suspension was poured into separating funnel and the organic phase washed sequentially with 1M $HCl_{(aq)}$ (3 × 10 mL), sat. $NaHCO_{3(aq)}$ (3 × 20 mL), H_2O (3 × 20 mL) and sat. brine (3 × 20 mL). The resulting organic layer was dried over Na_2SO_4, filtered and concentrated under reduced pressure to yield a yellow oil (594 mg). The crude product was purified by flash column chromatography (SiO_2, 30 mm column, eluent: EtOAc:*n*–hexane, 50:50) to afford ester **49a** as a white solid (163 mg, 36%). R_f =0.52 (50:50 EtOAc:*n*–hexane); IR (solid state) ν_{max} / cm^{-1}: 2950w (C–H alkyl), 1729vs (C=O aryl ester), 1588w (CC aromatic), 1453w, 1440w, 1429w, 1415w, 1284m, 1254vs, 1217m, 1198w, 1178w, 1106m, 1071w, 1062m, 1045m, 967m, 867w, 831w, 787m, 770m, 724m, 698m, 637w, 500w; δ_H (400 MHz; $CDCl_3$) 7.54 (1H, dd, *J* 7.9, 1.3, C(4)H), 7.16 (1H, t, *J* 7.9, C(5)H), 7.10 (1H, dd, *J* 7.9, 1.3, C(6)H), 4.59 (2H, t, *J* 5.8, C(11)H_2), 4.00 (2H, s, C(8)H_2), 2.92 – 2.85 (2H, m, C(9)H_2), 2.23 (2H, p, *J* 5.8, C(10)H_2); δ_C (101 MHz; $CDCl_3$) 166.0 (C, C1), 142.1 (C, C7), 133.4 (C, C3), 132.8 (CH, C5), 131.2 (CH, C4), 128.8 (CH, C6), 122.1 (C, C3), 66.8 (CH_2, C11), 40.7 (CH_2, C8), 35.2 (CH_2, C9), 28.7 (CH_2, C10); HRMS (ESI^+): m/z calc. for $C_{11}H_{12}{}^{79}BrO_2S$: 286.9736, found: 286.9738 $[MH]^+$, m/z calc. for $C_{11}H_{11}{}^{79}BrNaO_2S$: 308.9555, found: 308.9558 $[MNa]^+$.

X-ray crystallographic data for this compound can be accessed via www.ccdc.cam.ac.uk/data_request/cif (CCDC 2221227)

Methyl 4-bromo-2-(((3-hydroxypropyl)thio)methyl)benzoate (S34)

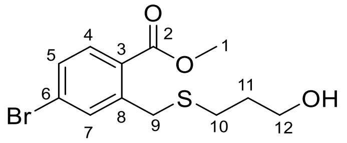

K_2CO_3 (538 mg, 3.89 mmol) was added to a solution of 3-hydroxy-1-propanethiol (170 µL, 2.00 mmol) and methyl 4-bromo-2-(bromomethyl)benzoate (922 mg, 3.00 mmol) in anhydrous DMF (7 mL) at RT. The resulting pale-yellow suspension was heated to 60 °C, whereupon a colour change to beige was noted. Upon further heating, the colour of the reaction suspension changed to yellow. After 18 h, the reaction was deemed to have gone to completion by TLC analysis. The reaction mixture was cooled to RT, before it was quenched by addition of H_2O (10 mL). The quenched solution was poured into a separating funnel. The aqueous layer was extracted with EtOAc (3 × 20 mL). The combined organic layers were washed sequentially with 1 M $HCl_{(aq)}$ (2 × 20 mL) and brine (2 × 20 mL), before was dried over $MgSO_4$, filtered and concentrated under reduced pressure to yield orange oil (1.10 g). The crude product was purified by flash column chromatography (SiO_2, 50 mm column, eluent: EtOAc:*n*–hexane, 45:55) to afford an the title compound as a pale yellow oil (812 mg, 86%). R_f = 0.33 (50:50 EtOAc:*n*–hexane); IR (thin film) ν_{max} / cm^{-1}: 3379mbr (O–H alcohol), 2941mbr (C–H alkyl), 1716v (C=O aryl ester), 1586s (CC aromatic), 1561m (CC aromatic), 1477w (CC aromatic), 1433m, 1388w, 1260s, 1188m, 1127m, 1092s, 1073msh, 963w, 867m, 836w, 809w, 781m, 753m, 723m, 693w, 593w, 560w, 489w; δ_H (400 MHz; $CDCl_3$) 7.78 (1H, d, *J* 8.4, C(4)H), 7.53 (1H, d, *J* 2.0, C(7)H), 7.45 (1H, dd, *J* 8.4, 2.0, C(5)H), 4.08 (2 H, s, C(9)H_2), 3.90 (3H, s, C(1)H_3), 3.72 (2 H, t, *J* 6.0, C(12)H_2), 2.57 (2H, t, *J* 7.1, C(10)H_2), 1.81 (2H, tt, *J* 7.1, 6.0, C(11)H_2); δ_C (101 MHz; $CDCl_3$) 167.2 (C, C2), 142.9 (C, C8), 133.9 (CH, C7), 132.7 (CH, C4), 130.3 (CH, C5), 128.3 (C, C3), 126.7 (C, C6), 61.5 (CH_2, C12), 52.4.0 (CH_3, C1), 34.3 (CH_2, C9), 31.8 (CH_2, C11), 28.6 (CH_2, C10); HRMS (ESI^+): m/z calc. for $C_{12}H_{16}{}^{79}BrO_3S$: 318.9998, found: 318.9996 $[M+H]^+$, m/z calc. for $C_{12}H_{15}{}^{79}BrNaO_3S$: 340.9817, found: 340.9816 $[M+Na]^+$.

4-Bromo-2-(((3-hydroxypropyl)thio)methyl)benzoic acid (S35)

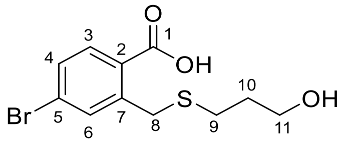

$NaOH_{(aq)}$ (4.0 M, 3.50 mL) was added dropwise over a period of 5 min to solution of alcohol **S34** (666 mg, 2.08 mmol) in MeOH (3.50 mL). A colour change to pale yellow solution was noted immediately during dropwise addition. Upon further addition, the appearance of the reaction mixture changed from a cloudy pale yellow to a milky white suspension and finally to pale-yellow solution. The reaction mixture was stirred at RT for 16 h, before the reactions was deemed to have gone to completion by TLC (complete consumption of alcohol **S34** was noted). The resulting pale-yellow solution was then acidified to pH 2.0 with 1M $HCl_{(aq)}$ (20 mL) at 0 ^{0}C. The resulting white solid was collected by suction filtration and washed with cold H_2O (3 × 20 mL) and then dried under *vacuo* to yield the title compound **S35** as a white solid (526 mg, 82%). R_f = 0.00 (50:50 Et_2O:*n*–hexane); Melting Point: 95 – 96 °C (from cyclohexane:CH_2Cl_2 + MeOH, 1:1 + 2%); IR (solid state) ν_{max} / cm^{-1}: 3368vbrm (O–H alcohol), 2926vbrm (C–H alkyl, O–H carboxylic acid), 2615vbrm (C–H alkyl, O–H carboxylic acid), 2544vbr, 1684vs (C=O aryl carboxylic acid), 1587m (CC aromatic), 1561s (CC aromatic), 1486m, 1410m, 1284m, 1272m, 1298m, 1254s, 1193m, 1139m, 1097m, 1069m, 1025m, 1010m, 917m, 894w, 871s, 847m, 837m, 785m, 769m, 676w, 551w, 474w, 461w; δ_H (400 MHz; CD_3OD) 7.81 (1H, d, *J* 8.1, C(3)H), 7.60 (1H, d, *J* 2.2, C(6)H), 7.50 (1H, dd, *J* 8.1, 2.2, C(4)H), 4.12 (2H, s, C(8)H_2), 3.59 (2 H, t, *J* 6.2, C(11)H_2), 2.50 (H, t, *J* 7.3, C(9)H_2), 1.74 (2H, tt, *J* 7.3, 6.2, C(10)H_2); δ_C (101 MHz; CD_3OD) 169.9 (C, C1) 144.8 (C, C7), 134.9 (CH, C6), 133.9 (CH, C3), 131.1 (CH, C4), 130.5 (CH, C6), 127.0 (C, C5), 61.5 (CH_2, C11), 34.6 (CH_2, C8) 33.3 (CH_2, C10), 29.0 (CH_2, C9); HRMS (ESI^+): m/z calc. for $C_{11}H_{14}{}^{79}BrO_3S$: 304.9842, found: 304.9831 $[M+H]^+$, m/z calc. for $C_{11}H_{13}{}^{79}BrNaO_3S$: 326.9661, found: 326.9649 $[M+Na]^+$, m/z calc. for $C_{11}H_{13}{}^{79}BrNa_2O_3S$: 348.9480, found: 348.9472 $[M-H+2Na]^+$.

9-Bromo-4,5-dihydro-3*H*-benzo[*g*][1,5]oxathionin-1(7*H*)-one (49b)

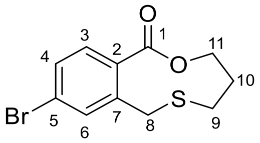

Dry DIPEA (280 µL, 1.61 mmol) was added dropwise over 1 minute to solution of carboxylic acid **S35** (196 mg, 0.642 mmol) and HOBt (108 mg, 0.799 mmol) in anhydrous DMF (6 mL). A colour change to pale yellow was noted. The resulting mixture was stirred for 5 min at RT, after which time EDC.HCl (185 mg, 0.963 mmol) was added and a colour change to pink/brown was observed. The resulting mixture was stirred at RT overnight under N_2 and progress of the reaction was monitored by TLC. After 18 h, the reaction was deemed to have gone to completion by TLC, with the reaction mixture changed to pale yellow colour. The resulting mixture was diluted with EtOAc (20 mL), before was transferred to separating funnel and organic phase was washed sequentially with 1 M $HCl_{(aq)}$ (3 × 20 mL), sat. $NaHCO_3$ (3 × 20 mL) and sat. brine (3 × 20 mL). The organic phase was dried over $MgSO_4$, filtered and concentrated under reduced pressure to yield a white solid (302 mg). The crude product was purified by flash column chromatography (SiO_2, 15 mm column, eluent: EtOAc:*n*–hexane, 20:80) to afford the title compound **49b** as a white solid (150 mg, 81%). R_f = 0.64 (50:50 EtOAc:*n*–hexane); Melting Point: 129 – 130 °C (from *n*–hexane); IR (solid state) ν_{max} / cm^{-1}: 2940w (C–H alkyl), 2957w (C–H alkyl), 2915w (C–H alkyl), 1715vs (C=O aryl ester), 1587vs (CC aromatic), 1557m (CC aromatic), 1477w (CC aromatic), 1455m, 1431wsh, 1423msh,1393m, 1350wsh, 1280ssh, 1266vs, 1213w, 1197w, 1121s, 1094s, 1038w, 977m, 900w, 888m, 868w, 843s, 814w, 792m, 776m, 717w, 695m, 682w, 639w, 594w, 559w, 517w, 499w; δ_H (400 MHz; $CDCl_3$) 7.71 (1H, d, *J* 8.2, C(3)H), 7.47 (1H, dd, *J* 8.2, 2.0, C(4)H), 7.33 (1H, d, *J* 2.0, C(6)H), 4.62 (2H, br t, *J* 6.5, C(11)H_2), 4.07 (2H, s, C(8)H_2), 2.91 – 2.84 (2H, m, C(9)H_2), 2.22 – 2.12 (2 H, m, C(10)H_2); δ_C (101 MHz; $CDCl_3$) 167.3 (C, C1) 144.2 (C, C7), 133.0 (CH, C6), 132.8 (CH, C3), 130.7 (C, C2), 130.6 (CH, C4), 126.0 (C, C5), 66.1 (CH_2, C11), 40.6 (CH_2, C8) 35.3 (CH_2, C9), 30.1 (CH_2, C10); HRMS (ESI$^+$): m/z calc. for $C_{11}H_{12}{}^{79}BrO_2S$: 286.9736, found: 286.9729 $[M+H]^+$, m/z calc. for $C_{11}H_{11}{}^{79}BrNaO_2S$: 308.9555, found: 308.9549 $[M+Na]^+$, m/z calc. for $C_{22}H_{22}{}^{79}Br_2NaO_4S_2$: 594.9218, found: 594.9212 $[2M+Na]^+$.

X-ray crystallographic data for this compound can be accessed via www.ccdc.cam.ac.uk/data_request/cif (CCDC 2221214)

Methyl 3-bromo-2-(bromomethyl)benzoate (S36)

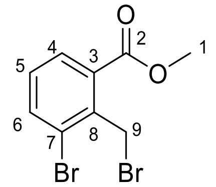

AIBN (78.1 mg, 0.355 mmol) was added to a yellow suspension of methyl 3-bromo-2-methylbenzoate (812 mg, 3.55 mmol) and *N*-bromosuccinimide (870 mg, 4.61 mmol) in anhydrous benzene (12 mL, degasses for 15 min) at RT. The resulting suspension was heated to 95 °C, whereupon a colour changed to pale yellow was noted. After 12 h, the reaction deemed to have gone to completion by TLC. The resulting mixture was allowed to cool to RT, before concentrated under reduced pressure. The yellow solution was diluted with EtOAc (30 mL) and poured into separating funnel. The organic layer was washed sequentially with 1M $HCl_{(aq)}$ (1 × 30 mL), sat. $NaHCO_3$ (1 × 30 mL), H_2O (1 × 30 mL) and sat. brine (1 × 20 mL), dried over Na_2SO_4, filtered and concentrated under reduced pressure to yield a pale yellow oil (1.44 g). The crude product was purified by flash column chromatography (SiO_2, 40 mm column, eluent: EtOAc: *n*–hexane,10:90) to afford the title compound **S36** as a pale yellow oil (1.05 g, 96%). R_f = 0.46 (EtAOc:*n*–hexane, 20:80); IR (thin film) ν_{max} / cm^{-1}: 2951w (C–H alkyl), 1719vs (C=O aryl ester), 1586w (CC aromatic), 1563w (CC aromatic), 1433m, 1290m, 1258vs, 1221s, 1162w, 1111s, 1088m, 967m, 876m, 837w, 806m, 758s, 725m, 704m, 610s, 541w, 487w; δ_H (400 MHz; $CDCl_3$) 7.89 (1H, dd, *J* 7.8, 1.5, C(4)H), 7.77 (1H, dd, *J* 7.8, 1.5, C(6)H), 7.23 (1H, t, J 7.8, C(5)H), 5.13 (2H, s, C(9)H_2), 3.96 (3H, s, C(1)H_3); δ_C (101 MHz; $CDCl_3$) 166.5 (C, C2), 138.0 (C, C8) 137.2 (CH, C6), 131.7 (C, C3), 130.5 (CH, C4), 129.6 (CH, C5), 127.2 (C, C7), 52.8 (CH_3, C1), 30.2 (CH_2, C9); HRMS (ESI^+): m/z calc. for $C_9H_8{}^{79}Br_2NaO_2$: 328.8783, found: 328.8780 $[MNa]^+$.

Methyl 3-bromo-2-(((3-hydroxypropyl)thio)methyl)benzoate (S37)

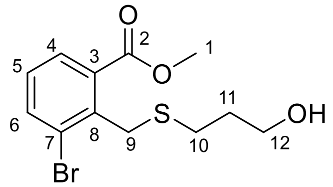

K_2CO_3 (511 mg, 3.14 mmol) was added to a pale yellow solution of 3-mercapto-1-propanothiol (110 µL, 1.57 mmol) and methyl 3-bromo-2-(bromomethyl)benzoate **S36** (727 mg, 2.86 mmol) in anhydrous DMF (8 mL) at RT. The resulting suspension was then heated to 70 °C,

whereupon a colour change to milky white was noted. Upon further heating, the colour of the reaction suspension changed to yellow. After 16 h, the reaction was deemed to have gone to completion by TLC. The reaction mixture was cooled to RT, before diluted with EtOAc (30 mL). The diluted solution was poured into a separating funnel and the organic layer was washed sequentially with H_2O (1 × 20 mL), 1M $HCl_{(aq)}$ (2 × 20 mL), sat. $NaHCO_{3(aq)}$ (1 × 20 mL) and sat. brine (2 × 20 mL), before dried over $MgSO_4$, filtered and concentrated under reduced pressure to yield pale orange oil (800 mg). The crude product was purified by flash column chromatography (SiO_2, 30 mm column, eluent: EtOAc:*n*–hexane, 50:50) to afford the title compound **S37** as a pale yellow oil (716 mg, 95%). R_f = 0.24 (60:40 EtOAc:*n*–hexane); IR (thin film) ν_{max} / cm^{-1}: 3418vbrm (O–H alcohol), 2950w (C–H alkyl), 1720vs (C=O aryl ester), 1587w (CC aromatic), 1561w (CC aromatic), 1432s, 1259vs, 1224m, 1126m, 1090m, 1046m, 967m, 910m, 806w, 763m, 733s, 705vs, 647w, 465w; δ_H (400 MHz; $CDCl_3$) 7.78 (1H, dd, *J* 7.9, 1.4, C(4)H), 7.71 (1H, dd, *J* 7.9, 1.4, C(6)H), 7.16 (1H, t, *J* 7.9, C(5)H), 4.36 (2H, s, C(9)H_2), 3.92 (3H, s, C(1)H_3), 3.71 (2H, q, *J* 6.9, C(12)H_2), 2.65 (2 H, t, *J* 7.0, C(10)H_2), 1.88 – 1.79 (2 H, m, C(11)H_2), 1.64 (1H, br, s, OH); δ_C (101 MHz; $CDCl_3$) 167.6 (C, C2), 139.8 (C, C8), 136.5 (CH, C6), 132.3 (C, C3), 130.0 (CH, C4), 128.1 (CH, C5), 126.5 (C, C7), 61.5 (CH_2, C12), 52.7 (CH_3, C1), 33.2 (CH_2, C9), 32.1 (CH_2, C11), 29.0 (CH_2, C10); HRMS (ESI^+): m/z calc. for $C_{12}H_{15}{}^{79}BrNaO_3S$: 340.9817, found: 340.9816 $[MNa]^+$.

3-Bromo-2-(((3-hydroxypropyl)thio)methyl)benzoic acid (S38)

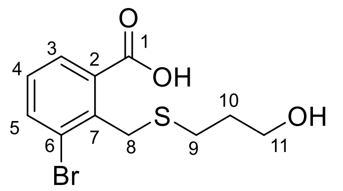

$NaOH_{(aq)}$ (4.0 M, 5.50 mL) was added dropwise over a period of 2 min to solution of methyl 3-bromo-2-(((3-hydroxypropyl)thio)methyl)benzoate **S37** (344 mg, 1.08 mmol) in MeOH (5.50 mL). A colour change to milky white suspension was noted immediately during dropwise addition. Upon further addition, the appearance of the reaction mixture changed over 10 min from pale yellow to a clear colourless and finally cloudy white. The reaction mixture was stirred at RT for 16 h, after which the reaction was deemed to have gone to completion by TLC (complete consumption of ester **S37** was noted) and was acidified to pH 2.0 by addition

of 1M $HCl_{(aq)}$ (20 mL). The acidified solution was poured into separating funnel and aqueous layer was extracted with CH_2Cl_2 (3 × 20 mL). The combined organic layers were dried over $MgSO_4$, filtered and concentrated under reduced pressure to yield a pale-yellow viscous oil (444 mg). The crude product was purified by flash column chromatography (SiO_2, 30 mm column, eluent: MeOH:CH_2Cl_2, 10:90) to afford the title compound **S38** as a white solid (285 mg, 87%). R_f = 0.09 (5:95 MeOH:CH_2Cl_2 (10 mL) + 3 drops of AcOH); Melting Point: 72 – 75 °C (from MeOH:CH_2Cl_2, 1:4); IR (solid state) ν_{max} / cm^{-1}: 3356vbrm (O–H alcohol), 2958vbrm (C–H alkyl, O–H carboxylic acid), 2925vbrm (C–H alkyl, O–H carboxylic acid), 2881m (C–H alkyl), 2653m (C–H alkyl), 2536 (C–H alkyl), 1681vs (C=O aryl carboxylic acid), 1585w (CC aromatic), 1560w (CC aromatic), 1444m, 1431m, 1403s, 1290m, 1266vs, 1226m, 1186m, 1140m, 1123w, 1094w, 1069w, 1045w, 1027w, 940m, 906s, 840w, 807m, 761s, 742m, 685vs, 650w, 612w, 575w, 509w, 499w, 463w; δ_H (400 MHz; $CDCl_3$) 7.90 (1H, dd, *J* 7.9, 1.5, C(3)H), 7.75 (1H, dd, *J* 7.9, 1.5, C(5)H), 7.17 (1H, t, *J* 7.9, C(4)H), 6.30 (2H, br s, 2 × OH), 4.41 (2H, s, C(8)H_2), 3.77 (2H, t, *J* 6.3, C(11)H_2), 2.68 (2H, t, *J* 7.1, C(9)H_2), 1.89 (2H, app. q, *J* 6.3, C(10)H_2); δ_C (101 MHz; $CDCl_3$) 171.0 (C, C1), 140.2 (C, C7), 137.3 (CH, C5), 131.7 (CH, C2), 130.7 (CH, C3), 128.2 (CH, C4), 127.0 (C, C6), 61.7 (CH_2, C11), 33.2 (CH_2, C8), 31.9 (CH_2, C10), 29.1 (CH_2, C9); HRMS (ESI$^+$): m/z calc. for $C_{11}H_{13}{}^{79}BrNaO_3S$: 326.9661, found: 326.9656 $[MNa]^+$.

8-Bromo-4,5-dihydro-3*H*-benzo[*g*][*1,5*]oxathionin-1(7*H*)-one (49c)

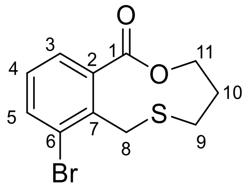

EDC.HCl (110 mg, 0.528 mmol) was added to solution of carboxylic acid **S38** (108 mg, 0.352 mmol), HOBt (61.8 mg, 0.422 mmol) and anhydrous DIPEA (150 µL, 0.880 mmol) in anhydrous DMF (3.52 mL). An immediate colour change colour from pale yellow to pale pink. After total of 16 h of stirring at RT, the reaction deemed to have to completion by TLC, with colour change to dark brown noted. The resulting mixture was diluted with EtOAc (20 mL), before was transferred to separating funnel and the organic phase was washed sequentially with 1 M $HCl_{(aq)}$ (2 × 10 mL), sat. $NaHCO_{3(aq)}$ (3 × 20 mL), H_2O (2 × 20 mL) and sat. brine (2 × 20 mL). The organic layer was dried over $MgSO_4$, filtered and concentrated under reduced pressure to

yield a white solid (129 mg). The crude product was purified by flash column chromatography (SiO_2, 20 mm column, eluent: EtOAc:*n*–hexane, 50:50) to afford the title compound **49c** as a white solid (67.3 mg, 67%). R_f = 0.55 (50:50 EtOAc:*n*–hexane); Melting Point: 80 – 82 °C (from CH_2Cl_2: $CHCl_3$, 1:1); IR (solid sate) ν_{max} / cm^{-1}: 2961w (C–H alkyl), 2922w (C–H alkyl), 1713vs (C=O aryl ester), 1588w (CC aromatic), 1660w (CC aromatic), 1447m, 1431m, 1383m, 1353w, 1278vs, 1244m, 1212s, 1189w, 1132m, 1097m, 1077m, 1036w, 972s, 895w, 880m, 831w, 814s, 791w, 770s, 729w, 703s, 692m, 640w, 598w, 545w, 513w; δ_H (400 MHz; $CDCl_3$) 7.73 (1H, dd, *J* 7.9, 1.5, C(3)H), 7.65 (1H, dd, *J* 7.9, 1.5, C(5)H), 7.16 (1H, t, *J* 7.9, C(4)H), 4.60 (2 H, t, *J* 5.9, C(11)H_2), 4.41 (2H, s, C(8)H_2), 2.93 – 2.86 (2 H, m, C(9)H_2), 2.23 – 2.16 (2H, m, C(10)H_2); δ_C (101 MHz; $CDCl_3$) 167.4 (C, C1), 140.7 (C, C7), 136.0 (CH, C5), 134.4 (C, C2), 130.3 (CH, C3), 128.4 (CH, C4), 125.1 (C, C6), 66.4 (CH_2, C11), 38.6 (CH_2, C8), 35.0 (CH_2, C9), 29.2 (CH_2, C10); HRMS (ESI$^+$): m/z calc. for $C_{11}H_{12}{}^{79}BrO_2S$: 286.9736, found: 286.9734 $[MH]^+$, m/z calc. for $C_{11}H_{11}{}^{79}BrNaO_2S$: 308.9555, found: 308.9556 $[MNa]^+$.

X-ray crystallographic data for this compound can be accessed via www.ccdc.cam.ac.uk/data_request/cif (CCDC 2221221).

Methyl 2-(((3-hydroxypropyl)thio)methyl)-3-nitrobenzoate (S39)

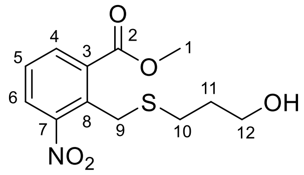

K_2CO_3 (369 mg, 2.31 mmol) was added to a pale-yellow solution of 3-mercaptopropan-1-ol (90.0 µL, 1.27 mmol) and methyl 2-(bromomethyl)-3-nitrobenzoate (317 mg, 1.16 mmol) in anhydrous DMF (4 mL) at RT. The resulting suspension was then heated to 70 °C, whereupon a colour change to orange/brown noted. After 16 h, the reaction was deemed to have gone to completion by TLC. The reaction mixture allowed to cool to RT and quenched by addition of H_2O (30 mL). The resulting milky white suspension was poured into separating funnel containing EtOAc (20 mL) and phases where separated. The pale yellow aqueous layer was extracted with EtOAc (3 × 20 mL), before the combined organic layers were washed sequentially with 1M $HCl_{(aq)}$ (2 × 20 mL), sat. $NaHCO_{3(aq)}$ (2 × 20 mL), H_2O (2 × 20 mL) and sat. brine (2 × 20 mL) and dried over $MgSO_4$, filtered and concentrated under reduced pressure

to yield brown oil (368 mg). The crude product was purified by flash column chromatography (SiO_2, 30 mm column, eluent: EtOAc:*n*–hexane, 50:50) to afford the title compound **S39** as a pale yellow oil (246 mg, 74%). R_f = 0.26 (50:50 EtOAc:*n*–hexane); IR (thin film) ν_{max} / cm^{-1}: 3387brm (O–H alcohol), 3086w (C–H alkenyl), 2952w (C–H alkyl), 2879w (C–H alkyl), 1722s (C=O aryl ester), 1604w (CC aromatic), 1575w (CC aromatic), 1529vs (N–O aryl), 1434m, 1356s (N–O aryl), 1267s, 1196m, 1087w, 1047m, 981w, 904w, 863w, 823w, 801w, 770m, 722m, 704s, 590w, 525w, 487w; δ_H (400 MHz; $CDCl_3$) 7.98 (1H, dd, *J* = 8.0, 1.4 Hz, C(4)H), 7.84 (1H, dd, *J* = 8.0, 1.4 Hz, C(6)H), 7.44 (1 H, t, *J* = 7.9 Hz, C(5)H), 4.33 (2H, s, C(9)H_2), 3.95 (3H, s, C(1)H_3), 3.67 (2 H, t, *J* = 6.1 Hz, C(12)H_2), 2.56 (2H, t, *J* = 7.1 Hz, C(10)H_2), 1.75 (2H, tt, *J* = 7.1, 6.1 Hz, C(11)H_2), 1.62 (1H, br s, OH); δ_C (101 MHz; $CDCl_3$) 167.1 (C, C2) 151.3 (C, C7), 135.0 (C, C8), 134.2 (CH, C4), 133.2 (C, C3),127.8 (CH, C6), 127.3 (CH, C5), 61.5 (CH_2, C12), 53.1 (CH_3, C1), 32.0 (CH_2, C11), 29.1 (CH_2, C10), 28.1 (CH_2, C9); HRMS (ESI): m/z calc. for $C_{12}H_{15}NNaO_5S$: 308.0563. Found: $[MNa]^+$, 308.0565 (–0.7 ppm error).

2-(((3-Hydroxypropyl)thio)methyl)-3-nitrobenzoic acid (S40)

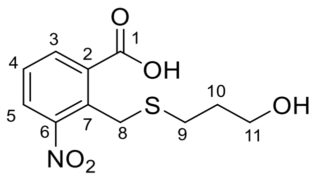

$NaOH_{(aq)}$ (6.0 M, 6.50 mL) was added dropwise over a period of 3 min to solution of methyl 2-(((3-hydroxypropyl)thio)methyl)-3-nitrobenzoate **S39** (490 mg, 1.53 mmol) in MeOH (6.50 mL). A colour change to milky white suspension was noted immediately during dropwise addition. The resulting suspension stirred at RT for a total of 16 h, whereupon a colour change from pale yellow to fluorescent yellow was observed. After that time the reaction was deemed to have gone to completion by TLC analysis (complete consumption of **S39** was noted). The reaction mixture was acidified to pH 2.0 with 1 M $HCl_{(aq)}$ (20 mL). The acidified cloudy/white suspension was poured into separating funnel and aqueous layer was extracted with CH_2Cl_2 (4 × 20 mL), before combined organic layers were dried over $MgSO_4$, filtered and concentrated under reduced pressure to yield an orange oil (202 mg). The crude product was purified by flash column chromatography (SiO_2, 30 mm column, eluent: MeOH:CH_2Cl_2, 5:95) to afford carboxylic acid **56** as a white solid (88.1 mg. 50%). R_f = 0.28 (10:90 MeOH:CH_2Cl_2, (10 mL) + 3 drops of AcOH); δ_H (400 MHz; CD_3OD) 7.81 – 7.71 (2H, m, CH, C(3+5)H), 7.44 – 7.35

(1H, m, CH, C(4)H), 4.38 (2H d, J = 4.6 Hz, CH_2, C(8)H_2), 3.53 (2H, t, J = 5.8 Hz, CH_2, C(11)H_2), 2.54 – 2.45 (2H, m CH_2, C(9)H_2), 1.74 – 1.61 (2H, m, CH_2, C(10)H_2); δ_C (101 MHz; CD_3OD) 176.1 (C, C1), 151.9 (C, C6), 143.9 (C, C2), 133.0 (CH, C3), 132.9 (C, C7), 128.4 (CH, C4), 125.7 (CH, C5), 61.6 (CH_2, C11), 33.5 (CH_2, C10), 29.6 (CH_2, C9), 29.4 (CH_2, C8); HRMS (ESI^-): m/z calc. for $C_{11}H_{12}NO_5S$: 270.0442, found: 270.0441 $[M-H]^-$, m/z calc. for $C_{11}H_{11}NaNO_5S$: 292.0261, found: 292.0276 $[M+Na-2H]^-$.

8-Nitro-4,5-dihydro-3H-benzo[g][1,5]oxathionin-1(7H)-one (50)

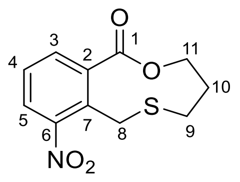

EDC.HCl (246 mg, 0.487 mmol) was added to a fluorescent green solution of 2-(((3-hydroxypropyl)thio)methyl)-3-nitrobenzoic acid **S40** (88.1 mg, 0.325 mmol), HOBt (63.6 mg, 0.390 mmol) and anhydrous DIPEA (140 µL, 0.813 mmol) in anhydrous DMF (3 mL) at RT. A colour change to pale yellow was noted immediately upon addition of DIPEA via syringe over a period of 30 sec. The resulting pale brown solution mixture was stirred for 24 h under Ar, after which time the reaction was deemed to have gone completion by TLC, with a colour change back to pale yellow was noted. The reaction mixture was diluted with EtOAc (20 mL) and was transferred separating funnel. The resulting organic layer was washed sequentially with 1 M $HCl_{(aq)}$ (3 × 20 mL), sat. $NaHCO_3$ (3 × 20 mL), H_2O (3 × 20 mL) and brine (3 × 20 mL), before being dried over $MgSO_4$, filtered and concentrated under reduced pressure to yield a pale-yellow oil (183 mg). The crude product was purified by flash column chromatography (SiO_2, 30 mm column, eluent: EtOAc:*n*–hexane, 30:70) to afford the title compound **50** as a white solid (38.9 mg, 47%). R_f = 0.51 (1:1 EtOAc:*n*–hexane); Melting Point: 110 – 112 °C (from CH_2Cl_2); IR (solid state) ν_{max} / cm^{-1}: 2961w (C–H alkyl), 2917w (C–H alkyl), 1720s (C=O aryl ester), 1605w (CC aromatic), 1572w (CC aromatic), 1525s (N–O stretch), 1460m, 1432w, 1352s (N–O stretch), 1289s, 1213m, 1117s, 1082m, 1041w, 975w, 919w, 885w, 844w, 822w, 789w, 749s, 721m, 702vs, 667w, 648w, 592w, 486w; δ_H (400 MHz; $CDCl_3$) 8.00 (1H, dd, J = 7.9, 1.5 Hz, C(3)H), 7.76 (1H, t, J = 7.9, 1.5 Hz, C(5)H), 7.45 (1H, dd, J = 7.9 Hz, C(4)H), 4.66 (2H, t, J 5.9 Hz, C(11)H_2), 4.22 (2H, s, C(8)H_2), 2.95 – 2.88 (2H, m, C(9)H_2), 2.27 – 2.16 (2H, m, C(10)H_2); δ_C (101 MHz; $CDCl_3$) 166.4 (C, C1), 151.1 (C, C6), 135.7 (C, C7), 134.9 (C, C2), 134.6 (CH, C3),

128.0 (CH, C4), 126.4 (CH, C5), 66.7 (CH_2, C11), 35.2 (CH_2, C9),33.4 (CH_2, C8), 29.1 (CH_2, C10); HRMS (ESI$^+$): m/z calc. for $C_{11}H_{12}NO_4S$: 254.0482, found: 254.0480 $[M+H]^+$, m/z calc. for $C_{11}H_{12}NNaO_4S$: 276.0301, found: 276.0299 $[M+Na]^+$.

Methyl 2-(((3-oxopropyl)thio)methyl)benzoate (S41)

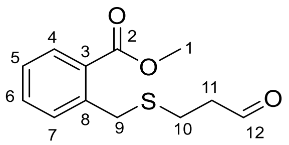

DMSO (610 µL, 8.53 mmol) was added dropwise over 10 min (via syringe pump) to a pale-yellow solution of oxalyl chloride (340 µL, 3.91 mmol) in anhydrous CH_2Cl_2 (6 mL) at –78 °C. The resulting solution was stirred for 20 min at –78 °C, after which time, a solution of ethyl 2-{[(3-hydroxypropyl)sulfanyl]methyl}benzoate (alcohol **S9**, 860 mg, 3.56 mmol) in anhydrous CH_2Cl_2 (6 mL) was added dropwise over 20 min (via syringe pump). The reaction mixture was stirred at –78 °C for an additional 20 min, before anhydrous Et_3N (1.98 mL, 0.013 mmol) was added dropwise over 10 min (via syringe pump). The resulting solution was stirred at –78 °C and gradually allowed to warm to RT. A colour change to milky/white was observed and the reaction mixture was stirred at RT for 18 h, before the reaction mixture was deemed to have gone to completion by TLC. The pale-yellow solution was then quenched with H_2O (20 mL) and poured into a separating funnel containing CH_2Cl_2 (20 mL). The aqueous layer was extracted with CH_2Cl_2 (3 × 20 mL), before the combined organic phases were dried over $MgSO_4$, filtered and concentrated under reduced pressure to yield an orange viscose oil (1.24 g). The crude product was purified by flash column chromatography (SiO_2, 60 mm column, eluent: EtOAc:*n*–hexane, 10:90 to 40:60) to afford aldehyde **S41** as a pale yellow oil (411 mg, 48%). R_f = 0.51 (EtOAc:*n*–hexane, 50:50); IR (thin film) ν_{max} / cm^{-1}: 2999w (C–H alkenyl), 2951w (C–H alkyl), 2834w (C–H alkyl), 2834w (C–H alkyl), 2729w (H–CO aldehyde), 1714vs (C=O), 1599w (CC aromatic), 1576w (CC aromatic), 1488w (CC aromatic), 1433w, 1388w, 1332w, 1292w, 1261vs, 1191m, 1164w, 1122m, 1077m, 1046w, 964w, 890w, 841w, 804w, 768w, 715s, 663w, 622w, 518w, 478w; δ_H (400 MHz; $CDCl_3$) 9.71 (1H, s, HCO), 7.92 (1H, dd, *J* 7.7, 1.5, C(4)H), 7.45 (1H, td, *J* 7.7, 1.5, C(7)H), 7.40 – 7.28 (2H, m, C(5+6)H), 4.15 (2H, s, C(9)H_2), 3.90 (3H, s, C(1)H_3), 2.77 – 2.62 (4H, m, C(10+11)H_2); δ_C (101 MHz; $CDCl_3$) 200.7 (C, C12), 167.9 (C, C2), 140.2 (C, C8), 131.9 (CH, C7), 131.2 (CH, C3), 131.0 (CH, C5), 129.4 (C, C3),

128.2 (CH, C6), 52.2 (CH_3, C1), 43.5 (CH_2, C11), 34.6 (CH_2, C9), 24.0 (CH_2, C10); HRMS (ESI^+): *m/z* calc. for $C_{12}H_{14}NaO_3S$: 261.0556, found: 261.0559 $[M+Na]^+$.

Methyl 2-(((3-hydroxybutyl)thio)methyl)benzoate (S42)

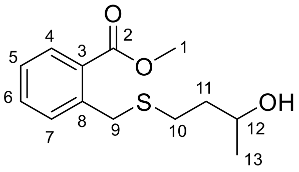

$AlMe_3$ (1.04 mL, 2.0 M in *n*–hexane, 2.07 mmol) was added dropwise over 5 min to a solution of aldehyde **S41** (411 mg, 1.73 mmol) in anhydrous CH_2Cl_2 (17 mL) at –78 °C. The resulting pale-yellow solution was stirred at –78 °C for 1 h, before allowed to be warmed to 0 °C for 1 h and then stirred for additional 1 h at RT. After a total of 3 h, the reaction was deemed to have gone to completion by TLC. The resulting pale-yellow solution was quenched by addition of H_2O (20 mL), instantaneous effervescence and fizzing was noted. The quenched reaction solution was poured into separating funnel containing CH_2Cl_2 (20 mL) and aqueous layer was extracted with CH_2Cl_2 (3 × 20 mL). The combined organic layers were dried over $MgSO_4$, filtered and concentrated under reduced pressure to yield a pale-yellow oil (667 mg). The crude product was purified by flash column chromatography (SiO_2, 40 mm column, eluent: hexane:EtOAc, 50:50) to afford secondary alcohol **S42** as a pale yellow oil (215 mg, 46%). R_f = 0.51 (EtAOc:*n*–hexane, 50:50); IR (thin film) ν_{max} / cm^{-1}: 3408br (O–H alcohol), 3067w (C–H alkyl), 3024w (C–H alkyl), 2964w (C–H alkyl), 2952w (C–H alkyl), 2927w (C–H alkyl), 1716s (C=O aryl ester), 1600w (CC aromatic), 1576w (CC aromatic), 1488w (CC aromatic), 1447w, 1434m, 1292w, 1262vs, 1190w, 1164w, 1121m, 1077s, 1046w, 949w, 965w, 906w, 878w, 842w, 801w, 766w, 754w, 715s, 663w, 580w, 469w; δ_H (400 MHz; $CDCl_3$) 7.90 (1H, dd, *J* 7.7, 1.5, C(4)H), 7.44 (1H, td, *J* 7.7, 1.5, C(7)H), 7.37 – 7.28 (2H, m, C(5+6)H), 4.13 (2H, s, C(9)H_2), 3.91 (3H, s, C(1)H_3), 3.90 – 3.87 (1H, br m, C(12)H), 2.55 (2H, t, *J* 7.2 C(10)H_2), 1.77 (1H, br d, *J* 4.4, OH), 1.72 – 1.64 (2 H, m, C(11)H_2), 1.17 (3H, d, *J* 6.2, C(13)H_3); δ_C (101 MHz; $CDCl_3$) 168.1 (C, C2), 140.6 (C, C8), 132.0 (CH, C4), 131.3 (CH, C6), 131.2 (CH, C7), 129.7 (C, C3), 127.3 (CH, C5), 62.8 (CH_2, C1), 52.4 (CH_3, C12), 38.2 (CH_2, C12), 34.7 (CH_2, C9), 28.5 (CH_2, C10), 23.6 (CH_3, C13); HRMS (ESI^+): *m/z* calc. for $C_{13}H_{18}NaO_3S$: 277.0869, found: 277.0868 $[M+Na]^+$.

2-(((3-Hydroxybutyl)thio)methyl)benzoic acid (S43)

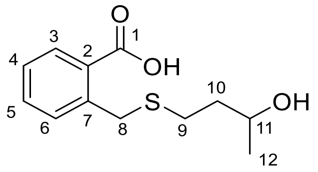

$NaOH_{(aq)}$ (4.0 M, 8 mL) was added dropwise over a period of 1 min to solution of methyl 2-(((3-hydroxybutyl)thio)methyl)benzoate **S42** (215 mg, 0.802 mmol) in methanol (8 mL). The resulting pale-yellow solution was heated to 50 °C for a total of 18 h, after which the reaction mixture was deemed to have gone to completion by TLC and was acidified to pH 2.0 by addition of 1 M $HCl_{(aq)}$ (20 mL). The acidified mixture was poured into separating funnel and aqueous layer was extracted with CH_2Cl_2 (4 × 30 mL). The combined organic layers were dried over $MgSO_4$, filtered and concentrated under reduced pressure to yield a pale-yellow oil (302 mg). The crude product was purified by flash column chromatography (SiO_2, 30 mm column, eluent: MeOH:CH_2Cl_2, 10:90) to afford carboxylic acid **S43** as a pale-yellow oil (157 mg, 82%). R_f = 0.41 (10:90 MeOH:EtOAc (10 mL) + 0.35% of AcOH); IR (neat) ν_{max} / cm^{-1}: 3410vbrm (O–H alcohol,), 2968m (C–H alkyl), 2928m (C–H alkyl), 2631brm (O–H carboxylic acid), 2503brm (O–H carboxylic acid), 1689s (C=O aryl carboxylic acid), 1600w (CC aromatic), 1576w (CC aromatic), 1490w, 1448w, 1401w, 1374m, 1295m, 12400s, 1164w, 1122m, 1073w, 1045m, 929w, 906w, 838w, 766m, 713s, 645m, 609w, 582w, 552w, 487w; δ_H (400 MHz; $CDCl_3$) 7.98 (1H, dd, *J* 7.5 1.5, C(3)H), 7.54 (2H, br s, 2 × OH),7.43 (1H, td, *J* 7.5, 1.5, C(5)H), 7.37 – 7.22 (2H, m, C(4+5)H), 4.22 – 4.09 (2H, m, C(8)H_2), 3.99 – 3.86 (1H, m, C(11)H), 2.55 (2H, t, *J* 7.3, C(9)H_2), 1.83 – 1.60 (2H, m, C(10)H_2), 1.16 (3H, d, *J* 6.2, C(12)H_3); δ_C (101 MHz; $CDCl_3$) 171.8 (C, C1), 141.2 (C, C7), 132.5 (CH, C3), 131.9 (CH, C5), 131.2 (CH, C6), 128.9 (C, C2), 127.1(CH, C4), 67.3 (CH, C11), 38.0 (CH_2, C10), 34.5 (CH_2, C8), 28.4 (CH_2, C9), 23.0 (CH_3, C12); HRMS (ESI^+): m/z calc. for $C_{12}H_{16}NaO_3S$: 263.0712, found: 263.0711 $[M+Na]^+$.

3-Methyl-4,5-dihydro-3*H*-benzo[*g*][1,5]oxathionin-1(7*H*)-one (51)

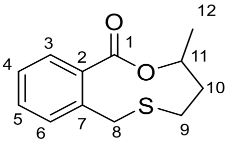

EDC.HCl (227 mg, 0.990 mmol) was added to clear colorless solution of 2-(((3-hydroxybutyl)thio)methyl)benzoic acid **S43** (157 mg, 0.660 mmol), HOBt (174 mg, 0.787 mmol) and dry DIPEA (290 µL, 1.64 mmol) in anhydrous DMF (7 mL). A colour change to pale

yellow was immediately noted and the reaction was stirred at RT for 16 h, before the reaction was deemed to have to completion by TLC. The resulting mixture was diluted with EtOAc (20 mL) and poured into separating funnel. The diluted milky/white suspension was washed sequentially with 1 M $HCl_{(aq)}$ (2 × 10 mL), sat. $NaHCO_{3(aq)}$ (3 × 20 mL) and sat. brine (3 × 20 mL). The organic layer was dried over $MgSO_4$, filtered and concentrated under reduced pressure to yield a pale-yellow oil (208 mg). The crude product was purified by flash column chromatography (SiO_2, 30 mm column, eluent: EtOAc:*n*–hexane, 20:80) to afford the title compund **51** as a colorless oil (105 mg, 71%). R_f = 0.52 (50:50 EtOAc:*n*–hexane); IR (thin film) ν_{max} / cm^{-1}: 2979 (C–H alkyl), 2935w (C–H alkyl), 1712s (C=O aryl ester), 1600m (CC aromatic), 1449m (CC aromatic), 1420w, 1385w, 1348w, 1296m, 1265vs, 1219w, 1117s, 1086w, 1038s, 895w, 864w, 840w, 795w, 761w, 730w, 710s, 700w, 569w, 481w, δ_H (400 MHz; $CDCl_3$) 7.83 (1H, dd, *J* 6.9, 2.1, C(3)H), 7.39 – 7.29 (2H, m, C(4+5)H), 7.15 (1H, dd, *J* 6.9, 2.1, C(6)H), 5.56 – 5.46 (1H, m, C(11H), 4.10 (2H, s, C(8)H_2), 2.96 – 2.80 (2H, m, C(9)H_2), 2.52 – 1.87 (2H, m, C(10)H_2), 1.41 (3H, d, *J* 6.7, C(12)H_3); δ_C (101 MHz; $CDCl_3$) 168.1 (C, C1), 141.8 (C, C7), 131.9 (C, C2), 131.4 (CH, C5), 131.0 (CH, C3), 129.9 (CH, C6), 127.5 (CH, C4), 71.3 (CH_2, C11), 40.0 (CH_2, C8), 34.5 (CH_2, C9), 30.9 (CH_2, C10), 18.9 (CH_3, C12); HRMS (ESI^+): m/z calc. for $C_{12}H_{15}O_2S$: 223.0787, found: 223.0785 $[M+H]^+$, m/z calc. for $C_{12}H_{14}NaO_2S$: 245.0607, found : 245.0605 $[M+Na]^+$.

Synthesis of methyl 2-(((3-hydroxypropyl)selanyl)methyl)benzoate (S45)

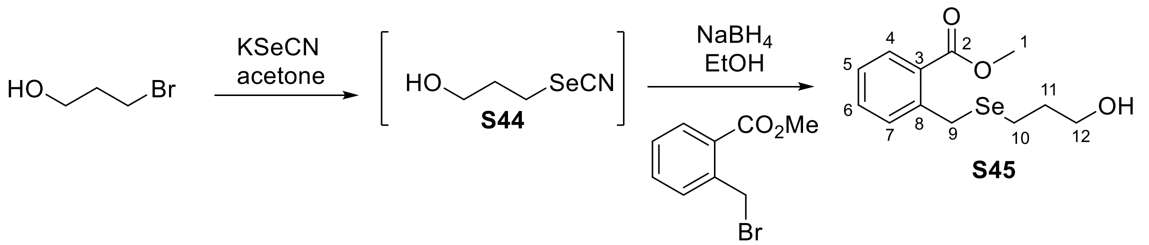

KSeCN (0.812 mg, 5.53 mmol) was added to a pale-yellow solution of 3-bromopropan-1-ol (769 mg, 5.53 mmol) in anhydrous acetone (28 mL) at RT under argon. The resulting pale-yellow suspension was then heated to 75 °C. After a total of 16 h, the reaction was deemed to have gone to completion by TLC. The reaction mixture was cooled to RT, filtered and the filtrate cake was washed with acetone (3 × 20 mL). The reaction mixture was concentrated under reduced pressure to yield 3-selenocyanatopropan-1-ol **S44** as a pale-yellow oil (1.51 g). This crude product was directly used in the next reaction step without further purification.

$NaBH_4$ (0.868 g, 22.9 mmol) was added to a pale yellow solution of 3-selenocyanatopropan-1-ol **S44** (1.51 g of the crude material noted above) and methyl 2-(bromomethyl)benzoate (2.52 g, 13.8 mmol) in anhydrous EtOH (46 mL) at 0 °C. A colour change to yellow and effervescence (liberation of H_2) was observed. The resulting milky white suspension was stirred at 0 °C for 2 h, before being warmed to RT, with a colour change to clear colourless solution was noted. After a total of 16 h, the reaction was deemed to have gone to completion by TLC. The resulting reaction mixture was quenched by addition of H_2O (40 mL) and allowed to stirrer at RT for 2 min, before being concentrated under reduced pressure. The concentrated mixture was diluted with EtOAc (30 mL) and transferred into a separating funnel. The organic layer was washed with H_2O (4 × 20 mL), before being dried over $MgSO_4$, filtered and concentrated under reduced pressure to yield a pale-yellow liquid (1.30 g). The crude product was purified by flash column chromatography (SiO_2, 50 mm column, eluent: EtOAc:*n*–hexane, 30:70) to afford a selenide **S45** as a clear colourless oil (932 mg, 59%). R_f = 0.28 (40:60 EtOAc:*n*–hexane); IR (neat) ν_{max} / cm^{-1}: 3405wbr (O–H alcohol), 3079w (C–H aryl), 2946w (C–H alkyl), 1713vs (C=O aryl ester), 1604w (CC aromatic), 1575 (CC aromatic), 1435m, 1297w, 1262vs, 1189w, 1114m, 1074m, 750vs, 708m, 665w; δ_H (400 MHz; $CDCl_3$) 7.90 (1 H, dd, *J* 8.1 1.5, C(4)H), 7.42 – 7.37 (1 H, m, C(7)H), 7.26 (2 H, ddd, *J* 8.1 6.7 1.5, C(5+6)H), 4.16 (2 H, s, C(9)H_2), 3.88 (3 H, s, C(1)H_3), 3.65 (2 H, t, *J* 6.1, C(12)H_2), 2.60 (2 H, t, *J* 7.2, C(10)H_2), 2.07 (1 H, br s, OH),1.84 (2 H, tt, *J* 7.2 6.1, C(11)H_2); δ_C (101 MHz; $CDCl_3$) 167.8 (C, C1), 142.2 (C, C8), 132.0 (CH, C7), 131.4 (CH, C4), 130.9 (CH, C6), 128.7 (C, C3), 126.9 (CH, C5), 62.3 (CH_2, C12), 52.2 (CH_3, C1), 32.8 (CH_2, C11), 25.8 (CH_2, C9), 20.7 (CH_2, C10); HRMS (ESI^+): m/z calc. for $C_{12}H_{17}O_3{}^{80}Se$: 289.0337, found: 289.0339 $[M+H]^+$, m/z calc. for $C_{12}H_{16}NaO_3{}^{80}Se$: 311.0157, found : 311.0155 $[M+Na]^+$.

2-(((3-Hydroxypropyl)selanyl)methyl)benzoic acid (S46)

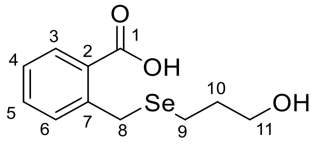

$NaOH_{(aq)}$ (4.0 M, 12 mL) was added dropwise over a period of 5 min to a clear colourless solution of methyl 2-(((3-hydroxypropyl)selanyl)methyl)benzoate **S45** (672 mg, 2.44 mmol) in methanol (12 mL) at RT. A colour change to milky/wite suspension was immediately noted and the reaction mixture was stirred at 60 °C for 18 h, at which point the reaction mixture

was deemed to have gone to completion by TLC (complete consumption of ester **S45** was noted). The reaction mixture was then acidified to pH 2.0 with 1 M $HCl_{(aq)}$ (35 mL). The resulting white solid was collected by suction filtration and washed with H_2O (3 × 40 mL) and then air dried to yield a carboxylic acid **S46** as a white solid (561 mg, 84%). R_f = 0.77 (50:90 MeOH:EtOAc); Melting Point: 121– 123.5 °C (from MeOH:CH_2Cl_2 , 1:10); IR (solid state) ν_{max} / cm^{-1}: 3390vbrm (O–H alcohol), 3327vbrw (C–H aryl), 3068vbrw (C–H alkyl, O–H carboxylic acid), 2935vbrm (C–H alkyl, O–H carboxylic acid), 2657vbrm (C–H alkyl), 2520w (C–H alkyl), 1676vs (C=O aryl carboxylic acid), 1601w (CC aromatic), 1575w (CC aromatic), 1493w, 1449m, 1407m, 1267s, 1130m, 1051m, 921m, 878m, 763s, 706s, 659s, 539m; δ_H (400 MHz; CD_3OD) 7.92 (1 H, dd, *J* 7.7 1.5, C(3)H), 7.43 (1 H, td, *J* 7.7 1.5, C(5)H), 7.34 – 7.26 (2 H, m, C(4+6)H), 4.89 (2 H, s, OH), 4.21 (2 H, s, C(8)H_2), 3.57 (2 H, t, *J* 6.3, C(11)H_2), 2.57 (2 H, t, J 7.4, C(9)H_2), 1.87 – 1.74 (1 H, s, C(10)H_2); δ_C (101 MHz; CD_3OD) 170.7 (C, C1), 144.0 (C, C7), 132.9 (CH, C5), 132.5 (CH, C3), 132.0 (CH, C6), 130.7 (C, C2), 127.7 (CH, C4), 64.4 (CH_2, C11), 34.3 (CH_2, C10), 26.1 (CH_2, C8), 20.9 (CH_2, C9); HRMS (ESI^-): m/z calc. for $C_{11}H_{13}O_3{}^{80}Se$: 273.0035, found: 273.0026 $[M-H]^-$.

4,5-Dihydro-3*H*-benzo[*g*][1,5]oxaselenonin-1(7*H*)-one (52)

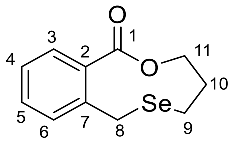

EDC.HCl (211 mg, 1.10 mmol) was added to a clear colourless solution of carboxylic acid **S46** (174 mg, 0.735 mmol), HOBt (119 mg, 1.10 mmol) and dry DIPEA (320 µL, 1.84 mmol) in anhydrous DMF (7.50 mL). A colour change to a pale yellow was immediately noted and the reaction mixture was stirred at RT for 18 h of stirring at RT, before reaction was deemed to have to completion by TLC. The resulting mixture was diluted with EtOAc (30 mL) and transferred into separating funnel and the organic phase was washed sequentially with 1 M $HCl_{(aq)}$ (3 × 30 mL), sat. $NaHCO_3$ (3 × 30 mL) and brine (4 × 30 mL). The organic layer was dried over $MgSO_4$, filtered and concentrated under reduced pressure to yield a white solid (179 mg). The crude product was purified by flash column chromatography (SiO_2, 20 mm column, eluent: EtOAc:*n*–hexane, 20:80) to afford selenide **52** as a white solid (168 mg, 90%). R_f = 0.57 (40:60 EtOAc:*n*–hexane); IR (solid sate) ν_{max} / cm^{-1}: 2963w (C–H alkyl), 2914w (C–H alkyl),

1712vs (C=O aryl lactone), 1596w (CC aromatic), 1483w, 1451w, 1430w, 1382w, 1350w, 1263m, 1294m, 1195w, 1183w, 1142w, 1083w, 1043m, 971m, 889w, 864w, 846w, 788m, 702m, 659w, 626w, 497w, 463w; δ_H (400 MHz; $CDCl_3$) 7.89 (1 H, dd, *J* 7.5, 1.8, C(3)H), 7.35 (1 H, td, *J* 7.5, 1.8, C(5)H), 7.31 (1 H, td, *J* 7.5, 1.8, C(4)H), 7.16 (1 H, dd, *J* 7.5, 1.8, C(6)H), 4.62 (2H, t, *J* 5.9, C(11)H_2), 4.13 (2H, s, C(8)H_2), 2.90 – 2.86 (2H, m, C(9)H_2), 2.21 – 2.14 (2H, m, C(10)H_2); δ_C (126 MHz; $CDCl_3$) 168.2 (C, C1), 143.1 (C, C7), 131.7 (CH, C5), 131.7 (CH, C3), 131.5 (C, C2), 130.0 (CH, C6), 127.4 (CH, C4), 66.1 (CH_2, C11), 31.4 (CH_2, C8), 30.7 (CH_2, C10), 25.7 (CH_2, C9); HRMS (ESI^+): m/z calc. for $C_{11}H_{13}O_2{}^{80}Se$: 257.0075, found: 257.0077 $[M+H]^+$, m/z calc. for $C_{11}H_{12}NaO_2{}^{80}Se$: 278.9895, found : 2 $[M+Na]^+$.

X-ray crystallographic data for compound **52** can be accessed via www.ccdc.cam.ac.uk/data_request/cif (CCDC 2235961)

Methyl 2-((benzyl(3-hydroxypropyl) amino) methyl) benzoate (S47)

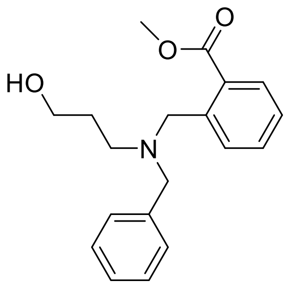

To a stirring solution of potassium carbonate (0.415 g, 3.00 mmol) in acetonitrile (5.00 mL), (23-(benzylamino) propan-1-ol (0.165 g, 1.00 mmol) was added followed by methyl 2-(bromomethyl) benzoate (0.230 g, 1.00 mmol). The reaction mixture was refluxed at 90 °C for 3 hours under argon before filtering through Celite washing with DCM, concentrating under vacuum and purifying by flash column chromatography (8:2 hexane:ethyl acetate) to afford the title compound as a clear oil (0.272 g, 87%) R_f = 0.54 (8:2 hexane:diethyl ether); ν_{max}/cm^{-1} (thin film) 3402, 2949, 2242, 1718, 1434, 1264, 1130, 1078, 909, 729, 699; δ_H (400 MHz, $CDCl_3$) 7.79 (1H, d, *J* = 7.7 Hz, Ar**H**), 7.57 (1H, d, *J* = 7.8 Hz, Ar**H**), 7.44 (1H, t, *J* = 7.5 Hz, Ar**H**), 7.30−7.28 (4H, m, Ar**H**), 7.27−7.20 (2H, m, Ar**H**), 3.93 (2H, s, ArC**H**$_2$N), 3.89 (1H, bs, O**H**), 3.84 (3H, s, OC**H**$_3$), 3.57 (2H, s, ArC**H**$_2$N), 3.55 (2H, t, *J* = 5.4 Hz, CH_2C**H**$_2$OH), 2.57 (2H, t, *J* = 6.3 Hz, NC**H**$_2$$CH_2$), 1.70 (2H, quintet, *J* = 6.0 Hz, CH_2C**H**$_2$$CH_2$); δ_c (101 MHz, $CDCl_3$) 168.5 (**C**O), 140.5 (Ar**C**), 138.5 (Ar**C**), 131.8 (Ar**C**H), 131.0 (Ar**C**), 130.3 (Ar**C**H), 129.3 (Ar**C**H), 128.4 (Ar**C**H), 127.3 (Ar**C**H), 127.0 (Ar**C**H), 62.8 (CH_2**C**H_2OH), 58.8 (Ar**C**H_2N), 56.5 (Ar**C**H_2N), 52.3 (CH_2C**H**$_2$N), 52.2

(CH_3O), 28.7 (CH_2**CH_2**CH_2); HRMS (ESI) calcd. for $C_{19}H_{24}NO_3$ 314.1756. Found $[MH]^+$ 314.1745 (−3.51 ppm error).

4-Benzyl-4,5,6,7-tetrahydrobenzo[g] [1,5] oxazonin-1(3H)-one (53)

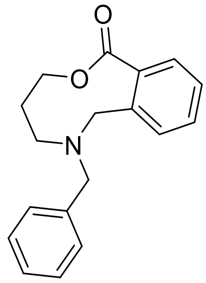

To a stirring solution of methyl methyl 2-((benzyl(3-hydroxypropyl) amino) methyl) benzoate **S47** (0.272 g, 0.869 mmol) in tetrahydrofuran (8.70 mL), aqueous lithium hydroxide (0.5 M) (2.00 mL, 1.04 mmol) was added and heated for at 50 °C for 3 hours. The solvent was removed under vacuum using dichloromethane (5 × 50.0 mL) to form an azeotropic mixture, to help ensure that all of the water from the hydrolysis was removed ahead of the next step. The intermediate lithium 2-((benzyl(3-oxidopropyl) amino) methyl) benzoate was dissolved in chloroform (8.70 mL) and DIPEA (0.280 mL, 1.61 mmol) was added followed by T3P 50% w/v in ethyl acetate (0.830 g, 1.30 mmol) and stirred at room temperature for 3 hours under argon. The reaction mixture was then transferred to a separating funnel, brine (50 mL) was added and extracted with dichloromethane (2 × 100 mL). The combined organic phases were then dried with sodium sulphate, filtered, concentrated under vacuum and purified via flash column chromatography (8:2 hexane:ethyl acetate) to afford the title compound **53** (0.181 g, 74%) R_f = 0.52 (8:2 hexane:ethyl acetate); ν_{max}/cm^{-1} (thin film) 2956, 2803, 2251, 1719, 1605, 1450, 1352, 1265, 1239, 1136, 1067, 989, 909, 767, 727, 699; δ_H (400 MHz, $CDCl_3$) 7.70 (1H, dd, *J* = 6.9 Hz, 2.1 Hz, Ar**H**), 7.37−7.31 (2H, m, 2 × Ar**H**), 7.30−7.25 (2H, m, 2 × Ar**H**), 7.21−7.18 (4H, m, 4 × Ar**H**), 4.96−3.80 (3H, m, C**H_2**O & C**H_a**H_bN), 3.75−3.16 (3H, CH_a**H_b**N, ArC**H_2**N), 2.67 (2H, s, ArC**H_2**N), 2.04−1.60 (CH_2C**H_2**CH_2); δ_c (101 MHz, $CDCl_3$) 172.1 (**C**O), 142.5 (Ar**C**), 138.4 (Ar**C**), 133.7 (Ar**C**), 130.5 (Ar**C**H), 129.5 (Ar**C**H), 129.4 (Ar**C**H), 129.0 (Ar**C**H), 128.3 (Ar**C**H), 127.4 (Ar**C**H), 127.1 (Ar**C**H), 66.9 (**C**H_2O), 59.92 (Ar**C**H_2N), 55.91 (Ar**C**H_2N), 51.7 (CH_2**C**H_2N), 25.4 (CH_2**C**$H_2$$CH_2$); HRMS (ESI) calcd. for $C_{18}H_{20}NO_2$ 282.1494. Found $[MH]^+$ 282.1485 (−3.19 ppm error).

Methyl 2-(2-((2-(2-hydroxyethyl) piperidin-1-yl) methyl) phenyl) acetate (S48)

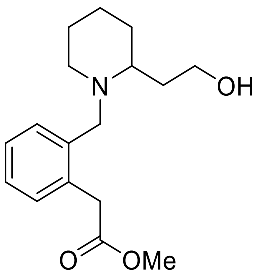

To a stirring solution of potassium carbonate (0.415 g, 3.00 mmol) in acetonitrile (5 mL), 2-(piperidin-2-yl) ethan-1-ol (0.129 g, 1.00 mmol) was added followed by methyl 2-(2-(bromomethyl) phenyl) acetate (0.243 g, 1.00 mmol). The reaction mixture was refluxed at 90 °C for 3 hours under argon before filtering through Celite washing with DCM, concentrating under vacuum and purifying by flash column chromatography (10:9:1 hexane:ethyl acetate:triethylamine) to afford the title compound **S48** as a colurless oil (0.151 g, 52%) R_f = 0.46 (10:9:1 hexane:ethyl acetate:triethylamine); ν_{max}/cm^{-1} (thin film) 3396, 2933, 2252, 1734, 1425, 1157, 730; δ_H (400 MHz, $CDCl_3$) 7.30−7.27 (1H, m, ArC**H**), 7.22−7.18 (3H, m, ArC**H**), 4.04 (1H, d, *J* = 13.4 Hz, ArC**H**$_a$H$_b$N), 3.82−3.59 (5H, m, ArC**H**$_2$COOMe, C**H**$_2$OH, O**H**), 3.66 (3H, s, OC**H**$_3$), 3.42 (1H, d, *J* = 13.4 Hz, ArCH$_a$**H**$_b$N), 2.80−2.74 (1H, m, C**H**$_a$H$_b$N), 2.65 (1H, quintet, *J* = 5.2 Hz, C**H**), 2.20-2.14 (1H, m, CH$_a$**H**$_b$N), 2.00 (1H, quintet, *J* = 6.4 Hz, C**H**$_a$H$_b$CH$_2$OH), 1.80−1.73 (1H, m, C**H**$_a$H$_b$), 1.69−1.61 (1H, m, CH$_a$**H**$_b$CH$_2$OH), 1.69−1.61 (1H, m, C**H**$_a$H$_b$'), 1.57−1.49 (1H, m, CH$_a$**H**$_b$'), 1.46−1.30 (2H, m, C**H**$_2$), 1.46−1.30 (1H, m, CH$_a$**H**$_b$); δ_c (101 MHz, $CDCl_3$) 172.4 (**C**O), 137.7 (Ar**C**), 133.5 (Ar**C**), 131.1 (Ar**C**H), 130.4 (Ar**C**H), 127.4 (Ar**C**H), 127.3 (Ar**C**H), 61.5 (**C**H$_2$OH), 59.0 (**C**H), 55.8 (Ar**C**H$_2$N), 52.1 (O**C**H$_3$), 49.1 (Ar**C**H$_2$COOMe), 38.4 (**C**H$_2$N), 32.1 (CH**C**H$_2$CH$_2$OH), 28.1 (**C**H$_2$), 23.3 (**C**H$_2$), 22.2 (**C**H$_2$); HRMS (ESI) calcd. for $C_{17}H_{26}NO_3$ 292.1913. Found $[MH]^+$ 292.1910 (−1.03 ppm error).

5,9,9a,10,11,12,13,15-Octahydro-6H,8H-benzo[g]pyrido[2,1-d] [1,5] oxazecin-4-one (54)

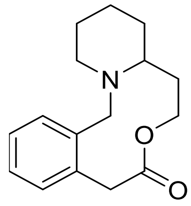

To a stirring solution of methyl 2-(2-((2-(2-hydroxyethyl) piperidin-1-yl) methyl) phenyl) acetate **S48** (0.150 g, 0.515 mmol) in tetrahydrofuran (1.34 mL), aqueous lithium hydroxide (0.5 M) (1.34 mL, 0.670 mmol) was added and heated for at 50 °C for 3 hours. The solvent was removed under vacuum using dichloromethane (5 × 50.0 mL) to form an azeotropic mixture, to help ensure that all of the water from the hydrolysis was removed ahead of the next step. The intermediate lithium 2-(2-((2-(2-oxidoethyl) piperidin-1-yl) methyl) phenyl) acetate was dissolved in chloroform (5.15 mL) and DIPEA (0.166 mL, 0.953 mmol) was added followed by T3P 50% w/v in ethyl acetate (0.492 g, 0.773 mmol) and stirred at room temperature for 24 hours under argon. The reaction mixture was then transferred to a separating funnel, brine (50.0 mL) was added and extracted with dichloromethane (2 × 100 mL). The combined organic phases were then dried with sodium sulphate, filtered, concentrated under vacuum and purified via flash column chromatography (10:9:1 hexane:ethyl acetate:triethylamine) to afford the title compound **54** (0.103 g, 77%) R_f = 0.63 (10:9:1 hexane:ethyl acetate:triethylamine); ν_{max}/cm^{-1} (thin film) 2930, 2249, 1728, 1452, 1372, 1233, 1044, 1101, 1045, 728; δ_H (400 MHz, $CDCl_3$) 7.25−7.18 (3H, m, 3 × Ar**H**), 7.12−7.10 (1H, m, Ar**H**), 4.62−4.57 (1H, m, C**H**$_a$H$_b$O), 3.98 (1H, d, *J* = 13.7 Hz, ArC**H**$_a$H$_b$N) 3.85−3.78 (1H, m, CH$_a$**H**$_b$O), 3.64 (1H, d, *J* = 13.7 Hz, ArC**H**$_a$H$_b$N), 3.63 (1H, d, *J* = 16.6 Hz, ArC**H**$_a$H$_b$CO), 3.52 (1H, d, *J* = 16.6 Hz, ArCH$_a$**H**$_b$CO), 2.89−2.82 (1H, m, C**H**$_a$H$_b$N), 2.72−2.68 (1H, m, C**H**), 2.38 (1H, dt, *J* = 13.9 Hz, 4.1Hz, CH$_a$**H**$_b$N), 2.34−2.24 (1H, m CHC**H**$_a$H$_b$CH$_2$O), 1.88−1.79 (1H, m CHCH$_a$**H**$_b$CH$_2$O), 1.67−1.45 (1H, m, C**H**$_a$H$_b$), 1.67−1.45 (2H, m, C**H**$_2$), 1.35−1.29 (1H, m, CH$_a$**H**$_b$), 1.27−1.20 (2H, m, C**H**$_2$); δ_C (101 MHz, $CDCl_3$) 170.4 (**C**O), 138.0 (Ar**C**), 135.1 (Ar**C**), 131.5 (Ar**C**H), 130.3 (Ar**C**H), 127.5 (Ar**C**H), 126.9 (Ar**C**H), 61.7 (O**C**H$_2$), 57.1 (Ar**C**H$_2$N), 52.8 (**C**H), 46.0 (**C**H$_2$N), 41.5 (Ar**C**H$_2$CO), 26.8 (**C**H$_2$), 25.7 (**C**H$_2$), 21.4 (**C**H$_2$), 20.8 (**C**H$_2$); HRMS (ESI) calcd. for $C_{16}H_{22}NO_2$ 260.1651. Found $[MH]^+$ 260.1643 (−3.8 ppm error).

Key for intermediates used to make lactone 55

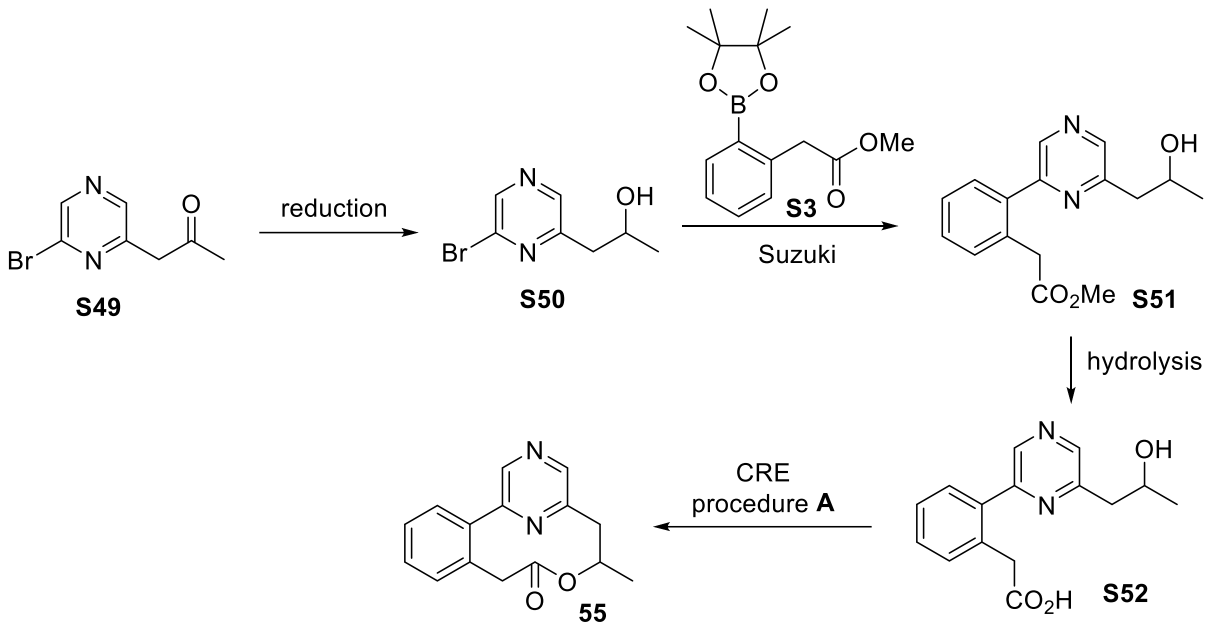

1-(6-Bromopyrazin-2-yl)propan-2-one (S49)

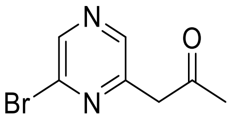

N,N-Diisopropylamine (282 µL, 2.00 mmol) was dissolved in THF (5 mL) and cooled to 0 ^{o}C before *n*-BuLi (1.6 M solution in hexanes, 1.25 mL, 2.00 mmol) was added dropwise and stirred for 30 mins. The LDA solution was then cooled to −78 °C, where a solution of 2-bromo-6-methylpyrazine (173 µL, 1.00 mmol) in THF (1.0 mL), was added dropwise and stirred for 30 mins. *N*-Methoxy-*N*-methylacetamide (213 µL, 2.00 mmol) was added and stirred for a further 30 mins at −78 °C before slowly warming to RT. The solution was then quenched with sat. $NH_4Cl_{(aq)}$ (20 mL) and extracted with ethyl acetate (3 × 50 mL) and washed with brine (10 mL). The combined organic extracts were dried over $MgSO_4$, filtered and removed *in vacuo*. Purification by flash column chromatography (SiO_2, 50% ethyl acetate in hexanes→ethyl acetate) afforded the *title compound* (as a 10:1 mixture of keto:enol tautomers) as a colourless oil (174 mg, 81%); R_F 0.59 (ethyl acetate); ν_{max}/cm^{-1} (thin film) 1722, 1639, 1563, 1511; δ_H (400 MHz, $CDCl_3$) 12.1 (1H, s, O**H**, enol), 8.58 (1H, s, C**H**, keto), 8.42 (1H, s, C**H**, keto), 8.28 (1H, s, C**H**, enol), 8.15 (1H, s, C**H**, enol), 5.35 (1H, s, C**H**COH, enol), 3.95 (2H, s, C**H₂**CO, keto), 2.29 (3H, s, C**H₃**, keto), 2.05 (3H, s, C**H₃**, enol); δ_C (101 MHz, $CDCl_3$) data for ketone tautomer only: 203.0 (**C**O), 151.0 (**C**N), 145.6 (**C**HN), 143.4 (**C**HN), 140.1 (**C**Br), 49.3 (**C**H_2), 30.3

(CH_3); HRMS (ESI): calcd. for $C_7H_7{}^{79}BrN_2O$, 236.9634. Found: $[MNa]^+$, 236.9630 (1.6 ppm error).

1-(6-Bromopyrazin-2-yl)propan-2-ol (S50)

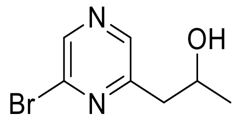

2-(6-Bromopyrazin-2-yl)-1-phenylethan-1-ol **S49** (160 mg, 0.744 mmol) was dissolved in methanol (7.4 mL) and cooled to 0 °C. Sodium borohydride (85 mg, 2.23 mmol) was added portionwise and stirred at RT for 1 h. Upon completion, the solvent was removed *in vacuo*. Purification by flash column chromatography (SiO_2, diethyl ether) afforded the *title compound* as a yellow oil (152 mg, 94%); R_F 0.50 (ethyl acetate); ν_{max}/cm^{-1} (thin film) 3378, 2968, 2928, 1561, 1508; δ_H (400 MHz, $CDCl_3$) 8.54 (1H, s, C**H**), 8.40 (1H, s, C**H**), 4.33–4.20 (1H, m, C**H**OH), 2.93 (1H, dd, *J* = 14.5, 3.8 Hz, C**H**H'CHOH), 2.86 (1H, dd, *J* = 14.5, 8.4 Hz, CH**H'**CHOH), 2.75 (1H, d, *J* = 3.8 Hz, O**H**), 1.30 (3H, d, *J* = 6.5 Hz, C**H₃**); δ_C (101 MHz, $CDCl_3$) 156.1 (**C**CH_2), 145.2 (**C**H), 143.1 (**C**H), 140.0 (**C**Br), 66.9 (**C**HOH), 43.2 (**C**H_2), 23.3 (**C**H_3); HRMS (ESI): calcd. for $C_7H_9{}^{79}BrN_2NaO$, 238.9790. Found: $[MNa]^+$, 239.9788 (1.0 ppm error).

Methyl 2-(2-(6-(2-hydroxypropyl)pyrazin-2-yl)phenyl)acetate (S51)

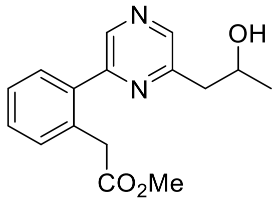

1-(6-Bromopyrazin-2-yl)propan-2-ol **S50** (135 mg, 0.622 mmol), methyl 2-(2-(4,4,5,5-tetramethyl-1,3,2-dioxaborolan-2-yl)phenyl)acetate **S3** (258 mg, 0.933 mmol), potassium phosphate (264 mg, 1.24 mmol) and $PdCl_2(dppf).CH_2Cl_2$ (26.0 mg, 12.5 µmol) were dissolved in THF (6.2 mL) under a nitrogen atmosphere. H_2O (56.0 µL, 3.11 mmol) was added and the solution heated, at reflux, to 80 °C for 18 h. Upon completion, the solvent was removed *in vacuo*. Purification by flash column chromatography (SiO_2, 33% diethyl ether in hexanes→diethyl ether) afforded the *title compound* as a colourless oil (97 mg, 55%); R_F 0.10 (50% ethyl acetate in hexanes); ν_{max}/cm^{-1} (thin film) 3406, 2967, 1735, 1529; δ_H (400 MHz, $CDCl_3$) 8.59 (1H, s, C**H**N), 8.41 (1H, s, C**H**N), 7.48–7.33 (4H, m, 4 × C**H**), 4.33–4.23 (1H, m,

CHOH), 3.83 (1H, d, J = 16.0 Hz, CHH′CO_2Me), 3.75 (1H, d, J = 16.0 Hz, CH**H′**CO_2Me), 3.55 (3H, s, OC**H₃**), 3.46 (1H, br s, O**H**), 2.99 (1H, dd, J = 14.5, 3.1 Hz, C**H**H′CH), 2.88 (1H, dd, J = 14.5, 9.2 Hz, CH**H′**CH), 1.30 (3H, d, J = 6.1 Hz, CHC**H₃**); δ_C (101 MHz, $CDCl_3$) 172.2 (**C**O_2Me), 153.8 (**C**N), 153.6 (**C**N), 143.0 (**C**HN), 142.5 (**C**HN), 136.6 (**C**), 133.0 (**C**), 132.0 (**C**H), 129.9 (**C**H), 129.5 (**C**H), 127.8 (**C**H), 67.0 (**C**HOH), 52.1 (O**C**H_3), 43.5 (**C**H_2CHOH), 39.5 (**C**H_2CO_2Me), 23.3 (**C**H_3); HRMS (ESI): calcd. for $C_{16}H_{18}N_2NaO_3$, 309.1210. Found: $[MNa]^+$, 309.1202 (2.4 ppm error).

2-(2-(6-(2-Hydroxypropyl)pyrazin-2-yl)phenyl)acetic acid (S52)

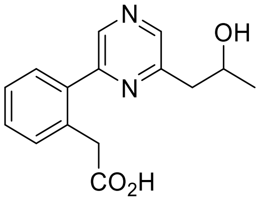

Methyl 2-(2-(6-(2-hydroxypropyl)pyrazin-2-yl)phenyl)acetate **S51** (90.0 mg, 0.314 mmol) was dissolved in THF (1.0 mL) and $LiOH_{(aq)}$ (0.5 M, 0.94 mL, 0.472 mmol) was added and stirred for 18 h. Upon completion, the solvent was removed *in vacuo*. Purification by flash column chromatography (SiO_2, diethyl ether→20% methanol in diethyl ether) afforded the *title compound* as a colourless oil (74 mg, 87%); R_F 0.21 (20% methanol in diethyl ether); ν_{max}/cm^{-1} (thin film) 3360, 2967, 2926, 1711, 1579, 1530; δ_H (400 MHz, $CDCl_3$) 8.63 (1H, s, C**H**N), 8.45 (1H, s, C**H**N), 7.46–7.32 (4H, m, 4 × C**H**), 4.21–4.11 (1H, m, C**H**OH), 3.63 (1H, d, J = 15.3 Hz, C**H**H′CO_2Me), 3.54 (1H, d, J = 15.3 Hz, CH**H′**CO_2Me), 2.95 (1H, dd, J = 13.7, 3.8 Hz, C**H**H′CHOH), 2.86 (1H, dd, J = 13.7, 8.4 Hz, CH**H′**CHOH), 1.21 (3H, d, J = 6.1 Hz, C**H₃**); δ_C (101 MHz, $CDCl_3$) 166.3 (**C**O_2H), 153.1 (**C**N), 152.5 (**C**N), 143.5 (**C**HN), 142.9 (**C**HN), 135.6 (**C**), 134.1 (**C**), 132.2 (**C**H), 130.0 (**C**H), 127.6 (**C**H), 127.5 (**C**H), 67.2 (**C**HOH), 43.1 (**C**H_2CH), 41.2 (**C**H_2CO_2H), 23.2 (**C**H_3); HRMS (ESI): calcd. for $C_{15}H_{16}N_2NaO_3$, 295.1053. Found: $[MNa]^+$, 295.1054 (−0.2 ppm error).

4-Methyl-4,5-dihydro-6,10-(azeno)benzo[*i*][1]oxa[6]azacyclododecin-2(1*H*)-one (55)

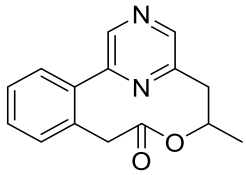

2-(2-(6-(2-Hydroxypropyl)pyrazin-2-yl)phenyl)acetic acid **S52** (65 mg, 0.239 mmol) was dissolved in $CHCl_3$ (2.4 mL), where DIPEA (75 µL, 0.430 mmol) and T3P (50% in ethyl acetate, 228 mg, 0.359 mmol) were added sequentially and stirred for 30 mins. Upon completion, the solvent was removed *in vacuo*. Purification by flash column chromatography (SiO_2, 50% diethyl ether in hexanes) afforded the *title compound* as a colourless oil (55 mg, 90%); R_F 0.55 (ethyl acetate); ν_{max}/cm^{-1} (thin film) 2975, 2929, 1723, 1546; δ_H (400 MHz, $CDCl_3$) 8.81 (1H, s, C**H**N), 8.34 (1H, s, C**H**N), 7.85–7.78 (1H, m, C**H**), 7.47–7.38 (3H, m, 3 × C**H**), 5.60–5.48 (1H, m, C**H**O), 3.58 (1H, d, *J* = 15.3 Hz, C**H**H′CO_2), 3.54 (1H, d, *J* = 15.3 Hz, CH**H′**CO_2), 3.11 (1H, dd, *J* = 17.6, 3.8 Hz, C**H**H′CH), 3.01 (1H, dd, *J* = 17.6, 9.9 Hz, CH**H′**CH), 1.50 (3H, d, *J* = 6.1 Hz, C**H₃**); δ_C (101 MHz, $CDCl_3$) 174.1 (**C**O_2CH), 150.7 (**C**N), 150.0 (**C**N), 141.6 (**C**HN), 139.5 (**C**HN), 135.6 (**C**), 134.4 (**C**), 134.3 (**C**H), 130.2 (**C**H), 127.9 (**C**H), 127.6 (**C**H), 70.3 (**C**HO), 44.1 (**C**H_2CH), 37.9 (**C**H_2CO_2), 21.3 (**C**H_3); HRMS (ESI): calcd. for $C_{15}H_{14}N_2NaO_2$, 277.0947. Found: $[MNa]^+$, 277.0953 (−2.0 ppm error).

Methyl 3-((benzyl(4-hydroxybutyl) amino) methyl) isoquinoline-4-carboxylate (S53)

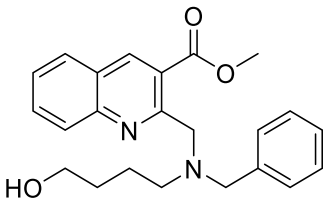

To a stirring solution of potassium carbonate (0.160 g, 1.16 mmol) in acetonitrile (3.90 mL), (4-(benzylamino) butan-1-ol (0.139 g, 0.773 mmol) was added followed by methyl 2-(bromomethyl) quinoline-3-carboxylate **S55** (0.216 g, 0.773 mmol). The reaction mixture was refluxed at 90 °C for 3 hours under argon before filtering through Celite washing with DCM, concentrating under vacuum and purifying by flash column chromatography (10:9:1 hexane:ethyl acetate:triethylamine) to afford the title compound as a pale yellow oil (0.271 g, 93%) R_f = 0.40 (10:9:1 hexane:ethyl acetate:triethylamine); ν_{max}/cm^{-1} (thin film) 3383, 2944, 1727, 1439, 1247, 1067, 757; δ_H (400 MHz, $CDCl_3$) 8.43−8.40 (1H, m, Ar**H**), 8.08−8.04 (1H, m, Ar**H**), 7.77−7.64 (2H, m, 2× Ar**H**), 7.49−7.42 (1H, m, Ar**H**), 7.20−7.15 (4H, m, Ar**H**), 7.15−7.10 (1H, m, Ar**H**), 4.25 (2H, s, ArC**H₂**N), 3.89 (3H, s, OC**H₃**), 3.72 (1H, bs, O**H**), 3.56 (2H, s, ArC**H₂**N), 3.45 (2H, t, *J* = 6.4 Hz, CH_2C**H₂**OH), 2.45−2.40 (2H, m, CH_2C**H₂**N), 1.53−1.46 (2H, m, CH_2C**H₂**CH_2), 1.41−1.33 (2H, m, CH_2C**H₂**CH_2); δ_c (101 MHz, $CDCl_3$) 168.1 (**C**O), 159.2 (Ar**C**), 147.6 (Ar**C**), 138.8 (Ar**C**), 138.5 (Ar**C**H), 131.4 (Ar**C**H), 129.3 (Ar**C**H), 128.9 (Ar**C**H), 128.3 (Ar**C**H), 120.1 (Ar**C**H),

127.2 (Ar**C**H), 126.9 (Ar**C**H), 126.2 (Ar**C**), 125.9 (Ar**C**), 62.1 (**C**H_2OH), 60.3 (Ar**C**H_2N), 58.1 (Ar**C**H_2N), 53.2 (CH_2**C**H_2N), 52.6 (O**C**H_3), 30.8 (**C**H_2), 22.3 (**C**H_2); HRMS (ESI) calcd. for $C_{23}H_{27}N_2O_3$ 379.2022. Found $[MH]^+$ 379.2010 (−3.16 ppm error).

7-Benzyl-3,4,5,6,7,6-hexahydro-1H- [1,6] oxazecino [4,3-b] quinolin-1-one (56)

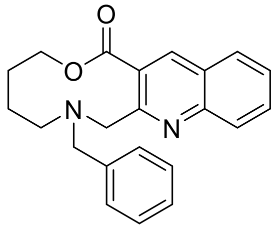

To a stirring solution of methyl 3-((benzyl (4-hydroxy butyl) amino) methyl) isoquinoline-4-carboxylate **S53** (0.271 g, 0.715 mmol) in tetrahydrofuran (1.90 mL), aqueous lithium hydroxide (0.5 M) (1.90 mL, 0.930 mmol) was added and heated for at 50 °C for 3 hours. The solvent was removed under vacuum using dichloromethane (5 × 50.0 mL) to form an azeotropic mixture, to help ensure that all of the water from the hydrolysis was removed ahead of the next step. The intermediate lithium 3-((benzyl(4-oxidobutyl) amino) methyl) isoquinoline-4-carboxylate was dissolved in chloroform (7.32 mL) and DIPEA (0.231 mL, 1.32 mmol) was added followed by T3P 50% w/v in ethyl acetate (0.683 g, 1.07 mmol) and stirred at room temperature for 24 hours under argon. The reaction mixture was then transferred to a separating funnel, brine (50.0 mL) was added and extracted with dichloromethane (2 × 100 mL). The combined organic phases were then dried with sodium sulphate, filtered, concentrated under vacuum and purified via flash column chromatography (10:9:1 hexane:ethyl acetate:triethylamine) to afford the title compound (0.158 g, 64%) R_f = 0.61 (10:9:1 hexane:ethyl acetate:triethylamine); ν_{max}/cm^{-1} (thin film) 2954, 2251, 1719, 1236, 1212, 1058, 729, 699; δ_H (400 MHz, $CDCl_3$) 8.45 (1H, s, Ar**H**), 8.07 (1H, d, *J* = 8.1 Hz, Ar**H**), 7.82 (1H, d, *J* = 8.1 Hz, Ar**H**), 7.72 (1H, t, *J* = 8.1 Hz, Ar**H**), 7.51 (1H, t, *J* = 8.1 Hz, Ar**H**), 7.24−7.17 (4H, m, 4 × Ar**H**), 7.13 (1H, tt, *J* = 6.7 Hz, 1.6 Hz, Ar**H**), 5.31−4.47 (2H, m, C**H**$_2$O), 3.91−2.94 (4H, m, 2 × ArC**H**$_2$N), 2.40−2.25 (2H, m, C**H**$_2$N), 1.93−1.60 (4H, m, 2 × C**H**$_2$); δ_c (101 MHz, $CDCl_3$) 168.5 (**C**O), 160.1 (Ar**C**), 127.7 (Ar**C**), 138.6 (Ar**C**), 138.2 (Ar**C**H), 131.0 (Ar**C**H), 129.5 (Ar**C**H), 128.9 (Ar**C**H), 128.4 (Ar**C**H), 128.2 (Ar**C**H), 127.6 (Ar**C**), 127.1 (Ar**C**H), 127.0 (Ar**C**H), 126.7 (Ar**C**), 66.5 (O**C**H_2), 60.3 (Ar**C**H_2N), 59.6 (Ar**C**H_2N), 53.4 (**C**H_2N), 25.5 (**C**H_2), 23.2 (**C**H_2); HRMS (ESI) calcd. for $C_{22}H_{23}N_2O_2$ 347.1760. Found $[MH]^+$ 347.1756 (−1.2 ppm error).

Methyl 2-((butyl(2-hydroxyethyl) amino) methyl) benzoate (S54)

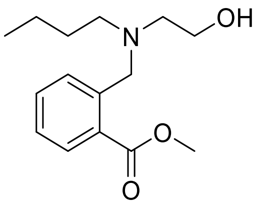

To a stirring solution of potassium carbonate (0.416 g, 3.00 mmol) in acetonitrile (5.00 mL), 2-(butylamino) ethan-1-ol (0.151 g, 1.30 mmol) was added followed by methyl 2-(bromomethyl) benzoate (0.229 g, 1.00 mmol). The reaction mixture was refluxed at 90 °C for 1 hours before filtering through Celite washing with DCM, concentrating under vacuum and purifying by flash column chromatography (1:2 hexane:ethyl acetate → ethyl acetate) to afford the title compound as a yellow oil (0.225 g, 85%) R_f = 0.30 (ethyl acetate - streak); ν_{max}/cm^{-1} (thin film) 3443, 2953, 1719, 1267, 1044, 740; δ_H (400 MHz, $CDCl_3$) 7.69 (1H, d, *J* = 7.8 Hz, ArC**H**), 7.44–7.37 (2H, m, 2 × ArC**H**), 7.30–7.26 (1H, m, ArC**H**), 3.87 (5H, s, OC**H**$_2$, OC**H**$_3$), 3.52 (2H, t, *J* = 5.1, C**H**$_2$OH), 2.57 (2H, t, *J* = 5.1, C**H**$_2$N), 2.39–2.35 (2H, m, C**H**$_2$N), 1.42–1.34 (2H, m, C**H**$_2$), 1.24–1.14 (2H, m, C**H**$_2$), 0.81 (3H, t, *J* = 7.3, C**H**$_3$CH$_2$); δ_c (101 MHz, $CDCl_3$) 169.4 (**C**O), 140.4 (Ar**C**), 135.4 (Ar**C**H), 131.3 (Ar**C**), 130.5 (Ar**C**H), 129.9 (Ar**C**H), 127.1 (Ar**C**H), 50.0 (C**H**$_2$OH), 57.1 (Ar**C**H$_2$N), 55.8 (**C**H$_2$N), 54.0 (**C**H$_2$N), 52.3 (O**C**H$_3$), 28.6 (**C**H$_2$), 20.6 (**C**H$_2$), 14.1 (**C**H$_3$); HRMS (ESI) calcd. for $C_{15}H_{24}NO_3$ 266.1756. Found $[MH]^+$ 266.1745 (−4.13 ppm error).

5-Butyl-3,4,5,4-tetrahydro-1H-benzo[f] [1,4] oxazocin-1-one (57)

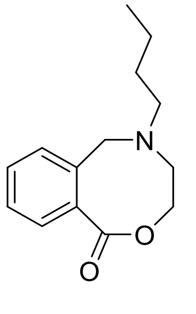

To a stirring solution of methyl 2-((butyl(2-hydroxyethyl) amino) methyl) benzoate **S54** (0.176 g, 0.663 mmol) in methanol (1.46 mL), aqueous lithium hydroxide (0.5 M) was added (1.46 mL, 0.730 mmol) and heated for at 50 °C for 1 hours. The solvent was removed under vacuum using chloroform (5 × 50.0 mL) to form an azeotropic mixture, to help ensure that all of the water from the hydrolysis was removed ahead of the next step. The intermediate lithium 2-

((butyl(2-oxidoethyl) amino) methyl) benzoate was dissolved in chloroform (6.60 mL) and DIPEA (0.214 mL, 1.23 mmol) was added followed by T3P 50% w/v in ethyl acetate (0.633 g, 0.995 mmol) and stirred at room temperature for 1 hours under argon. The reaction mixture was then transferred to a separating funnel, water (20.0 mL) was added and extracted with dichloromethane (3 × 20 mL). The combined organic phases were then dried with magnesium sulphate, filtered, concentrated under vacuum and purified via flash column chromatography (3:1 → 1:1 hexane:ethyl acetate) to afford the title compound **57** as a colourless oil (0.097 g, 63%); ν_{max}/cm^{-1} (thin film) 2956, 1714, 1276, 1112, 736; δ_H (400 MHz, $CDCl_3$) 7.38−7.36 (1H, m, ArC**H**), 7.34−7.32 (1H, m, ArC**H**), 7.28−7.24 (1H, m, ArC**H**), 7.11 (1H, d, *J* = 7.8 Hz, ArC**H**) 4.00 (2H, t, *J* = 5.5, OC**H**$_2$CH$_2$), 3.83 (2H, s, ArC**H**$_2$N), 2.86 (2H, t, *J* = 5.5, C**H**$_2$N), 2.64 (2H, t, *J* = 7.5, C**H**$_2$N), 1.52−1.45 (2H, m, C**H**$_2$), 1.32−1.22 (2H, m, C**H**$_2$), 0.87 (3H, t, *J* = 7.3, CH$_2$C**H**$_3$); δ_C (101 MHz, $CDCl_3$) 173.6 (**C**O), 139.8 (Ar**C**), 130.1 (Ar**C** & Ar**C**H), 129.7 (Ar**C**H), 127.4 (Ar**C**H), 126.3 (Ar**C**H), 65.9 (**C**H$_2$O), 58.3 (Ar**C**H$_2$N), 57.6 (**C**H$_2$N), 56.8 (**C**H$_2$N), 29.3 (**C**H$_2$), 20.4 (**C**H$_2$), 14.1 (**C**H$_3$); HRMS (ESI) calcd. for $C_{14}H_{20}NO_2$ 234.1494. Found $[MH]^+$ 234.1487 (−2.99 ppm error).

Methyl 2-(bromomethyl) quinoline-3-carboxylate (S55)

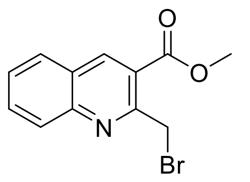

Benzene was degassed for 20 minutes using argon before adding it to a mixture of methyl 2-methylquinoline-3-carboxylate (**6-135**) (0.614 g, 3.05 mmol), azobisisobutyronitrile (0.025 g, 0.153 mmol) and N-bromosuccinimide (0.710 g, 3.97 mmol) and refluxed at 85 °C for 24 hours. The reaction mixture was filtered through Celite and washed with dichloromethane, concentrated under vacuum and purified by flash column chromatography (98:2 toluene:ethyl acetate) to afford the title compound (0.335 g, 39%) R_f = 0.45 (98:2 toluene:ethyl acetate); ν_{max}/cm^{-1} (thin film) 2950, 1719, 1437, 1252, 1204, 1062, 802, 756, 594; δ_H (400 MHz, $CDCl_3$) 8.68 (1H, s, Ar**H**), 7.97 (1H, d, *J* = 8.5 Hz, Ar**H**), 7.74 (1H, d, *J* = 8.5 Hz, Ar**H**), 7.70 (1H, t, *J* = 8.5 Hz, Ar**H**), 7.48 (1H, t, *J* = 8.5 Hz, Ar**H**), 5.12 (2H, s, ArC**H**$_2$Br), 3.93 (3H, s, OC**H**$_3$); δ_C (101 MHz, $CDCl_3$) 165.9 (**C**O), 156.1 (Ar**C**), 148.4 (Ar**C**), 141.1 (ArCH), 132.2 (Ar**C**H), 129.2 (Ar**C**H), 128.6 (Ar**C**H), 128.0 (Ar**C**H), 126.6 (Ar**C**), 122.5 (Ar**C**), 52.8 (O**C**H$_3$), 33.8

(Ar**C**H_2Br), HRMS (ESI) calcd. for $C_{12}H_{11}BrNO_2$ 279.9973. Found $[MH]^+$ 279.9964 (−3.22 ppm error).

Methyl 2-((2-(2-hydroxyethyl) piperidin-1-yl) methyl) quinoline-3-carboxylate (S56)

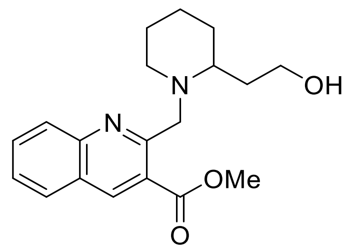

To a stirring solution of potassium carbonate (0.131 g, 0.945 mmol) in acetonitrile (3.15 mL), 2-(piperidin-2-yl) ethan-1-ol (0.082 g, 0.630 mmol) was added followed by methyl 2-(bromomethyl) quinoline-3-carboxylate **S55** (0.177 g, 0.630 mmol). The reaction mixture was refluxed at 90 °C for 3 hours under argon before filtering through Celite washing with DCM, concentrating under vacuum and purifying by flash column chromatography (10:9:1 hexane:ethyl acetate:triethylamine) to afford the title compound **S56** as an oil (0.186 g, 90%) R_f = 0.22 (10:9:1 hexane:ethyl acetate:triethylamine); ν_{max}/cm^{-1} (thin film) 3375, 2936, 1726, 1445, 1247, 1065, 763; δ_H (400 MHz, $CDCl_3$) 8.46 (1H, s, Ar**H**), 8.02 (1H, d, *J* = 8.5 Hz, Ar**H**), 7.72 (1H, d, *J* = 8.1 Hz, Ar**H**), 7.68−7.63 (1H, m, Ar**H**), 7.46−7.40 (1H, m, Ar**H**), 4.58 (1H, s, O**H**), 4.26 (1H, d, *J* = 14.2 Hz, ArC$\mathbf{H}_a$$H_b$N), 4.21 (1H, d, *J* = 14.2 Hz, ArC$H_a$$\mathbf{H}_b$N), 3.97 (3H, s, OC$\mathbf{H}_3$), 3.70−3.63 (1H, m, C$\mathbf{H}_a$$H_b$OH), 3.54−3.48 (1H, m, C$H_a$$\mathbf{H}_b$OH), 2.81−2.72 (1H, m, C**H**), 2.81−2.72 (1H, m, CH_2C$\mathbf{H}_a$$H_b$N), 2.37−2.33 (1H, m, CH_2C$H_a$$\mathbf{H}_b$N), 2.07−1.98 (1H, m, C$\mathbf{H}_a$$H_b$$CH_2$OH), 1.77−1.69 (1H, m, C$\mathbf{H}_a$$H_b$), 1.55−1.48 (1H, m, C$\mathbf{H}_a$$H_b$), 1.55−1.48 (1H, m, C$\mathbf{H}_a$$H_b$), 1.45−1.37 (1H, m, C$H_a$$\mathbf{H}_b$$CH_2$OH), 1.45−1.37 (1H, m, C$H_a$$\mathbf{H}_b$), 1.30−1.23 (1H, m, C$H_a$$\mathbf{H}_b$), 1.30−1.23 (1H, m, C$H_a$$\mathbf{H}_b$); δ_C (101 MHz, $CDCl_3$) 167.6 (**C**O), 159.3 (Ar**C**), 147.8 (Ar**C**), 139.2 (Ar**C**H), 131.4 (Ar**C**H), 128.9 (Ar**C**H), 128.3 (Ar**C**H), 127.1 (Ar**C**H), 126.1 (Ar**C**), 124.9 (Ar**C**), 61.7 (O**C**H_2), 58.7 (Ar**C**H_2N), 57.3 (N**C**H), 52.5 (O**C**H_3), 48.1 (CH_2**C**H_2N), 31.1 (**C**H_2), 27.1 (**C**H_2), 22.9 (**C**H_2), 20.7 (**C**H_2); HRMS (ESI) calcd. for $C_{19}H_{25}N_2O_3$ 329.1865. Found $[MH]^+$ 329.1863 (−0.608 ppm error).

2,3,4,4a,5,4-Hexahydro-1H-pyrido [2',1':4,5] [1,5] oxazonino[7,6-b] quinolin-8(15H)-one (58)

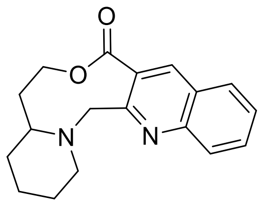

To a stirring solution of methyl 2-((2-(2-hydroxyethyl) piperidin-1-yl) methyl) quinoline-3-carboxylate **S56** (0.186 g, 0.565 mmol) in tetrahydrofuran (1.47 mL), aqueous lithium hydroxide (0.5 M) (1.47 mL, 0.735 mmol) was added and heated for at 50 °C for 3 hours. The solvent was removed under vacuum using dichloromethane (5 × 50 mL) to form an azeotropic mixture, to help ensure that all of the water from the hydrolysis was removed ahead of the next step. The intermediate lithium 2-((2-(2-oxidoethyl) piperidin-1-yl) methyl) quinoline-3-carboxylate was dissolved in chloroform (2.83 mL) and DIPEA (0.182 mL, 1.05 mmol) was added followed by T3P 50% w/v in ethyl acetate (0.539 g, 0.848 mmol) and stirred at room temperature for 24 hours under argon. The reaction mixture was then transferred to a separating funnel, brine (50.0 mL) was added and extracted with dichloromethane (2 × 100 mL). The combined organic phases were then dried with sodium sulphate, filtered, concentrated under vacuum and purified via flash column chromatography (10:9:1 hexane:ethyl acetate:triethylamine) to afford the title compound **58** (0.1414 g, 85%). In solution in $CDCl_3$, the product exists as a 4:1 mixture of rotamers. R_f = 0.64 (10:9:1 hexane:ethyl acetate:triethylamine); ν_{max}/cm^{-1} (thin film) 2933, 2252, 1725, 1204, 1059, 909, 727; δ_H (400 MHz, $CDCl_3$) 8.21 (1H, d, *J* = 3.8 Hz, Ar**H**) $_{major}$, 9.20 (1H, d, *J* = 3.4 Hz, Ar**H**) $_{minor}$, 7.95 (1H, dd, *J* = 8.6 Hz, *J* = 3.4 Hz, Ar**H**) $_{both\ rotamers}$, 7.73-7.70 (1H, m, Ar**H**) $_{both\ rotamers}$, 7.65−7.60 (1H, m, Ar**H**) $_{both\ rotamers}$, 7.44−7.39 (1H, m, Ar**H**) $_{both\ rotamers}$, 5.29 (1H, td, *J* = 11.8 Hz, *J* = 3.0 Hz, $OC\mathbf{H}_aH_bCH_2$) $_{major}$, 5.10 (1H, t, *J* = 11.8 Hz, $OC\mathbf{H}_aH_bCH_2$) $_{minor}$, 4.79 (1H, dd, *J* = 14.3 Hz, *J* = 3.5 Hz, $ArC\mathbf{H}_aH_bN$) $_{major}$, 4.39 (1H, dd, *J* = 14.3 Hz, $ArC\mathbf{H}_aH_bN$) $_{minor}$, 4.04−3.98 (1H, m, $OCH_a\mathbf{H}_bCH_2$) $_{minor}$, 3.96−3.92 (1H, m, $OCH_a\mathbf{H}_bCH_2$) $_{major}$, 3.75 (1H, dd, *J* = 15.1 Hz, *J* = 3.3 Hz, $ArCH_a\mathbf{H}_bN$) $_{minor}$, 3.14 (1H, dd, *J* = 14.3 Hz, *J* = 3.5 Hz, $ArCH_a\mathbf{H}_bN$) $_{major}$, 2.98−2.88 (1H, m, C**H**N)$_{minor}$, 2.66−2.57 (1H, m, C**H**N)$_{major}$, 2.66−2.57 (1H, m, $CHC\mathbf{H}_aH_bCH_2$), 2.51−2.41 (1H, m, $CH_2C\mathbf{H}_aH_bN$) $_{both\ rotamers}$, 2.41−2.36 (1H, m, $CH_2CH_a\mathbf{H}_bN$) $_{minor}$, 2.11−2.03 (1H, m, $C\mathbf{H}_aH_b$)$_{minor}$, 2.00−1.92 (1H, m, $CH_2CH_a\mathbf{H}_bN$) $_{major}$, 1.81−1.08 (7H in total, $C\mathbf{H}_2$) $_{both\ rotamers}$ [1.81−1.67 (2H, m) $_{minor}$, 1.67−1.54 (2H, m) $_{major}$, 1.41−1.11 (5H, m) $_{both\ rotamers}$, 1.08−0.99 (1H, m)$_{major}$; δ_C (101 MHz, $CDCl_3$) 170.3

(**C**O) $_{major}$, 169.8 (**C**O) $_{minor}$, 163.2 (Ar**C**) $_{minor}$, 162.3 (Ar**C**) $_{major}$, 147.3 (Ar**C**) $_{major}$, 147.2 (Ar**C**) $_{minor}$, 137.0 (Ar**C**H) $_{major}$, 136.7 (Ar**C**H) $_{minor}$, 130.8 (Ar**C**H) $_{major}$, 130.7 (Ar**C**H) $_{minor}$, 128.8 (Ar**C**H) $_{major}$, 128.7 (Ar**C**H) $_{minor}$, 128.33 (Ar**C**H) $_{major}$, 128.29 (Ar**C**H) $_{minor}$, 127.6 (Ar**C**) $_{minor}$, 127.4 (Ar**C**) $_{major}$, 126.8 (Ar**C**H) $_{major}$, 126.7 (Ar**C**H) $_{minor}$, 126.5 (Ar**C**) $_{major}$, 66.0 (O**C**H$_2$) $_{minor}$, 62.9 (O**C**H$_2$) $_{major}$, 62.6 (N**C**H) $_{minor}$, 59.5 (N**C**H$_2$) $_{major}$, 58.3 (**C**HN) $_{major}$, 53.8 (N**C**H$_2$) $_{major}$, 49.9 (N**C**H$_2$) $_{minor}$, 49.4 (N**C**H$_2$) $_{minor}$, 31.7 (**C**H$_2$) $_{major}$, 31.0 (**C**H$_2$) $_{minor}$, 30.9 (**C**H$_2$) $_{major}$, 28.1 (**C**H$_2$) $_{major}$, 25.8 (**C**H$_2$) $_{minor}$, 25.5 (**C**H$_2$) $_{major}$, 24.4 (**C**H$_2$) $_{major}$, 18.8 (**C**H$_2$) $_{minor}$; HRMS (ESI) calcd. for $C_{18}H_{21}N_2O_2$ 297.1603. Found [MH]$^+$ 297.1597 (−2.02 ppm error).

Methyl (*S*)-2-((2-(hydroxymethyl) pyrrolidin-1-yl) methyl) quinoline-3-carboxylate (S57)

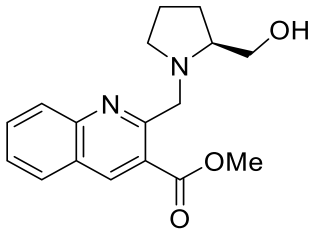

To a stirring solution of potassium carbonate (0.123 g, 0.891 mmol) in acetonitrile (2.98 mL), (S)-pyrrolidin-2-ylmethanol (0.060 g, 0.594 mmol) was added followed by methyl 2-(bromomethyl) quinoline-3-carboxylate **S55** (0.166 g, 0.594 mmol). The reaction mixture was refluxed at 90 °C for 3 hours under argon before filtering through Celite washing with DCM, concentrating under vacuum and purifying by flash column chromatography (10:9:1 hexane:ethyl acetate:triethylamine) to afford the title compound an oil (0.112 g, 63%). R_f = 0.30 (10:9:1 hexane:ethyl acetate:triethylamine); ν_{max}/cm^{-1} (thin film) 3455, 2959, 1722, 1439, 1248, 1064, 757, 757; δ_H (400 MHz, CDCl$_3$) 8.53 (1H, s, Ar**H**), 8.07 (1H, d, *J* = 8.5 Hz, Ar**H**), 7.83 (1H, d, *J* = 8.1 Hz, Ar**H**), 7.76 (1H, m, Ar**H**), 7.57−7.53 (1H, m, Ar**H**), 4.62 (1H, d, *J* = 13.9 Hz, ArC**H**$_a$H$_b$N), 4.03 (1H, d, *J* = 13.9 Hz, ArC**H**$_a$H$_b$N), 3.95 (3H, s, OC**H**$_3$), 3.57 (1H, dd, *J* = 11.6 Hz, *J* = 3.2 Hz, C**H**$_a$H$_b$OH), 3.37 (1H, dd, *J* = 11.6 Hz, *J* = 3.2 Hz, CH$_a$**H**$_b$OH), 2.86−2.81 (1H, m, C**H**$_a$H$_b$N), 2.76−2.71 (1H, m, CH$_a$**H**$_b$N), 2.51−2.44 (1H, m, NC**H**), 1.19−1.82 (1H, m, C**H**$_a$H$_b$), 1.79−1.59 (1H, m, CH$_a$**H**$_b$), 1.79−1.59 (2H, m, C**H**$_2$); δ_C (101 MHz, CDCl$_3$) 168.3 (**C**O), 159.0 (Ar**C**), 147.8 (Ar**C**), 138.9 (Ar**C**H), 131.7 (Ar**C**H), 129.1 (Ar**C**H), 128.7 (Ar**C**H), 127.2 (Ar**C**H), 126.2 (Ar**C**), 124.6 (Ar**C**), 66.3 (O**C**H$_3$), 62.8 (Ar**C**H$_2$N), 60.2 (N**C**H$_2$), 52.7 (**C**H), 27.2 (**C**H$_2$), 23.4 (**C**H$_2$); HRMS (ESI) calcd. for $C_{17}H_{21}N_2O_3$ 301.1552. Found [MH]$^+$ 301.1548 (−1.33 ppm error).

(*S*)-2,3,3a,4-Tetrahydro-1H-pyrrolo [2',1':3,4] [1,4] oxazocino [6,7-b] quinolin-6(13H)-one (59)

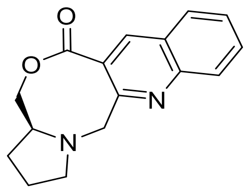

To a stirring solution of methyl (S)-2-((2-(hydroxy methyl) pyrrolidin-1-yl) methyl) quinoline-3-carboxylate **S57** (0.133 g, 0.444 mmol) in tetrahydrofuran (1.15 mL), aqueous lithium hydroxide (0.5 M) (1.15 mL, 0.577 mmol) was added and heated for at 50 °C for 3 hours. The solvent was removed under vacuum using dichloromethane (5 × 50.0 mL) to form an azeotropic mixture, to help ensure that all of the water from the hydrolysis was removed ahead of the next step. The intermediate lithium (S)-2-((2-(oxidomethyl) pyrrolidin-1-yl) methyl) quinoline-3-carboxylate was dissolved in chloroform (2.22 mL) and DIPEA (0.143 mL, 0.822 mmol) was added followed by T3P 50% w/v in ethyl acetate (0.424 g, 0.666 mmol) and stirred at room temperature for 24 hours under argon. The reaction mixture was then transferred to a separating funnel, brine (50.0 mL) was added and extracted with dichloromethane (2 × 100 mL). The combined organic phases were then dried with sodium sulphate, filtered, concentrated under vacuum and purified via flash column chromatography (10:9:1 hexane:ethyl acetate:triethylamine) to afford the title compound **59** (0.107 g, 90%) R_f = 0.35 (10:9:1 hexane:ethyl acetate:triethylamine); ν_{max}/cm^{-1} (thin film) 2960, 2247, 1708, 1491. 1178, 915, 728; δ_H (400 MHz, $CDCl_3$) 8.15−8.13 (1H, m, Ar**H**), 7.92−7.89 (1H, m, Ar**H**), 7.74−7.71 (1H, m, Ar**H**), 7.67−7.61 (1H, m, Ar**H**), 7.46−7.40 (1H, m, Ar**H**), 4.31 (1H, d, *J* = 18.2 Hz, ArC**H**$_a$H$_b$N), 4.13 (1H, d, *J* = 18.2 Hz, ArCH$_a$**H**$_b$N), 4.04−3.98 (1H, m, OC**H**$_a$H$_b$CH), 3.79−3.73 (1H, m, OCH$_a$**H**$_b$CH), 3.16−3.10 (1H, m, CH$_2$C**H**$_a$H$_b$N), 2.90−2.84 (1H, m, NC**H**), 2.82−2.75 (1H, m, CH$_2$CH$_a$**H**$_b$N), 2.03−1.92 (1H, m, C**H**$_a$H$_b$), 1.87−1.78 (1H, m, CH$_a$**H**$_b$), 1.87−1.78 (1H, m, C**H**$_a$H$_b$), 1.76−1.68 (1H, m, CH$_a$**H**$_b$); δ_c (101 MHz, $CDCl_3$) 170.9 (**C**O), 159.9 (Ar**C**), 147.8 (Ar**C**), 137.6 (ArCH), 131.1 (Ar**C**H), 128.4 (ArCH), 128.2 (Ar**C**H), 126.9 (ArCH), 126.6 (Ar**C**), 125.7 (Ar**C**), 69.6 (O**C**H$_2$), 64.3 (**C**H), 59.1 (Ar**C**H$_2$N), 55.1 (CH$_2$**C**H$_2$N), 29.9 (**C**H$_2$), 24.0 (**C**H$_2$); HRMS (ESI) calcd. for $C_{16}H_{17}N_2O_2$ 269.1290. Found $[MH]^+$ 269.1286 (−1.49 ppm error); $[\alpha]_D^{20}$ = 33.62 (c = 1.0, DCM).

***N*-(2-(*tert*-Butyldimethylsiloxy)-ethyl)-2-bromoaniline (S58)**

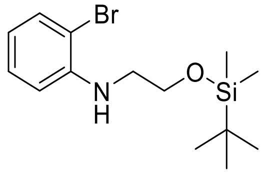

Sodium hydride (60% dispersion in mineral oil, 0.800 g, 20.0 mmol) was added to dry THF (5.2 mL). 2-Bromoaniline (1.72 g, 10.0 mmol) was dissolved in dry THF (1.7 mL) and then added dropwise to the sodium hydride suspension. This was stirred for 10 minutes before adding 2-bromo-1-*tert*-butyldimethylsiloxy-ethane (2.20 mL, 2.40 g, 10.0 mmol). The reaction mixture was then stirred at room temperature for 18 h. The reaction was quenched with sat. aq. ammonium chloride (20 mL). Deionized water (20 mL) was then added, and the product was extracted with diethyl ether (2 x 50 mL). The combined organic layers were dried over magnesium sulfate, filtered and concentrated *in vacuo*. The crude product was purified by column chromatography (SiO_2, 20:1 hexane:diethyl ether) to yield the title compound (3.11 g, 94%) as a yellow oil. R_f 0.70 (20:1 hexane:diethyl ether); δ_H (300 MHz, $CDCl_3$) 7.43 (1H, dd, *J* = 8.0, 1.5 Hz, ArH), 7.18 (1H, ddd, *J* = 8.0, 8.0, 1.5 Hz, ArH), 6.65 (1H, dd, *J* = 8.0, 1.5 Hz, ArH), 6.58 (1H, ddd, *J* = 8.0, 8.0, 1.5 Hz, ArH), 3.88 (2H, t, *J* = 5.5 Hz, OCH_2), 3.27 (2H, t, *J* = 5.5 Hz, NCH_2), 0.93 (9H, s, $C(CH_3)_3$), 0.10 (6H, s, $Si(CH_3)_2$). Characterisation data matched those reported in the literature.[8]

***N*-Methyl-*N*-(2-(*tert*-butyldimethylsiloxy)-ethyl)-2-bromoaniline (S59)**

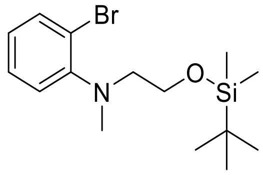

A round-bottom flask was charged with trifluoroacetic acid (4.0 mL) and then cooled to 0 °C. Sodium borohydride (243 mg, 6.42 mmol) was added over 5 minutes, then the mixture was warmed to room temperature. Paraformaldehyde (151 mg, 5.03 mmol) was added followed by *N*-(2-(*tert*-butyldimethylsiloxy)-ethyl)-2-bromoaniline **S58** (163 mg, 0.493 mmol) dissolved in DCM (2.0 mL). The reaction mixture was stirred at room temperature for 1 h. The mixture was then poured into 2 M aq. NaOH (50 mL) that had been cooled to 0 °C. The product was extracted with DCM (2 x 20 mL) and the combined organic layers were dried over magnesium

sulfate, filtered and concentrated *in vacuo* to yield the title compound (150 mg, 89%) as a yellow oil. This product was used without further purification. δ_H (300 MHz, $CDCl_3$) 7.56 (1H, d, *J* = 8.0 Hz, ArH), 7.29–7.25 (2H, m, ArH), 6.97–6.88 (1H, m, ArH), 3.85 (2H, t, *J* = 6.0 Hz, OCH_2), 3.30–3.17 (2H, m, NCH_2), 2.92 (3H, s, NCH_3), 0.87 (9H, s, $C(CH_3)_3$), 0.03 (6H, s, $Si(CH_3)_2$). Characterisation data matched those reported in the literature.[9]

***N*-(2-Hydroxyethyl)-2-bromoaniline (S60)**

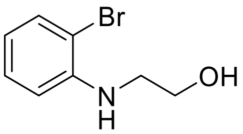

N-(2-(*tert*-Butyldimethylsiloxy)-ethyl)-2-bromoaniline **S58** (857 mg, 2.58 mmol) was dissolved in dry THF (4.7 mL). A solution of TBAF in THF (3.90 mL, 1 M, 3.90 mmol) was added dropwise and the reaction mixture was stirred for 1 h at room temperature. The mixture was then concentrated *in vacuo* and purified by column chromatography (SiO_2, 7:3 hexane:ethyl acetate) to yield the title compound (491 mg, 87%) as a yellow oil. R_f 0.31 (7:3 hexane:ethyl acetate); δ_H (300 MHz, $CDCl_3$) 7.44 (1H, dd, *J* = 8.0, 1.5 Hz, ArH), 7.19 (1H, ddd, *J* = 8.0, 8.0, 1.5 Hz, ArH), 6.69 (1H, dd, *J* = 8.0, 1.5 Hz, ArH), 6.60 (1H, ddd, *J* = 8.0, 8.0, 1.5 Hz, ArH), 3.88 (2H, t, *J* = 5.0 Hz, OCH_2), 3.37 (2H, t, *J* = 5.0 Hz, NCH_2), 1.79 (1H, br s, NH). Characterisation data matched those reported in the literature.[9]

***N*-(2-Hydroxyethyl)-*N*-methyl-2-bromoaniline (S61)**

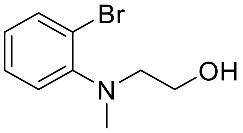

N-(2-Hydroxyethyl)-2-bromoaniline **S60** (1.61 g, 7.50 mmol) was dissolved in acetonitrile (42 mL) and then cooled to 0 °C. Acetic acid (4.30 mL, 4.50 g, 75.0 mmol), sodium cyanoborohydride (2.36 g, 37.5 mmol) and formaldehyde (37 wt.% in water, 11.2 mL, 150 mmol) were added and the mixture was stirred at room temperature for 20 h. Sat. aq. ammonium chloride (50 mL) was added to the reaction mixture and this was concentrated *in vacuo* to remove the organic solvent. Sat. aq. sodium bicarbonate (70 mL) was added, and the product was extracted with DCM (2 x 50 mL). The combined organic layers were dried over magnesium sulfate, filtered and concentrated *in vacuo*. The crude product was purified by column chromatography (SiO_2, 3:1 hexane:ethyl acetate) to yield title compound **S61** (1.47 g,

85%) as a yellow oil. R*f* 0.22 (3:1 hexane:ethyl acetate); δ_H (300 MHz, $CDCl_3$) 7.59 (1H, dd, *J* = 8.0, 1.5 Hz, ArH), 7.33–7.27 (1H, m, ArH), 7.19 (1H, dd, *J* = 8.0, 1.5 Hz, ArH), 6.99 (1H, ddd, *J* = 8.0, 8.0, 1.5 Hz, ArH), 3.68 (2H, td, *J* = 5.5 Hz, 5.5 Hz, OCH_2), 3.17 (2H, t, *J* = 5.5 Hz, NCH_2), 2.77–2.73 (4H, m, NCH_3 and OH). Characterisation data matched those reported in the literature.[9]

Methyl 2'-((2-hydroxyethyl)(methyl)amino)-[1,1'-biphenyl]-2-carboxylate (S62)

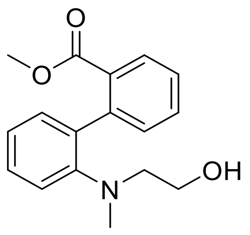

A dry round-bottom flask was charged with *N*-(2-hydroxyethyl)-*N*-methyl-2-bromoaniline **S61** (1.31 g, 5.69 mmol), methyl 2-(4,4,5,5-tetramethyl-1,3,2-dioxaborolan-2-yl) benzoate (3.75 g, 14.3 mmol), bis(triphenylphosphine)palladium(II) dichloride (0.400 g, 0.570 mmol) and potassium carbonate (3.15 g, 22.8 mmol). Dimethylacetamide (32 mL) and deionised water (6.3 mL) were added, and the reaction mixture was stirred at 80 °C for 24 h. Deionised water (150 mL) was added, and the product was extracted with ethyl acetate (2 x 150 mL). The combined organic layers were dried over magnesium sulfate, filtered and concentrated *in vacuo*. The crude product was purified by column chromatography (SiO_2, 4:1 toluene:ethyl acetate) to yield a mixture of impurity and title compound (1.23 g, 58% from ^1H NMR spectrum) as a yellow oil which was used without further purification. R*f* 0.26 (4:1 toluene:ethyl acetate); δ_H (300 MHz, $CDCl_3$) 7.86 (1H, d, *J* = 8.0 Hz, ArH), 7.58 (1H, ddd, *J* = 8.0, 8.0, 1.5 Hz, ArH), 7.45–7.13 (6H, m, ArH), 3.67 (3H, s, CH_3O), 3.50 (2H, t, *J* = 5.0 Hz, C**H**$_2$OH), 2.81 (2H, t, *J* = 5.0 Hz, NCH_2), 2.54 (3H, s, NCH_3); δ_C (75 MHz, $CDCl_3$) 169.0 (**C**O_2CH_3), 151.1 (ArC), 140.9 (ArC), 136.3 (ArC), 131.9 (ArC), 131.1 (ArC), 131.0 (ArC), 130.4 (ArC), 129.2 (ArC), 128.6 (ArC), 127.2 (ArC), 123.6 (ArC), 119.9 (ArC), 58.5 (NCH_2), 55.7 (CH_2OH), 51.9 (CH_3O), 39.9 (NCH_3); ν_{max}/cm^{-1} (thin film) 3380 (OH), 2950 (CH), 1720 (CO), 1598, 1507, 1482, 1446, 1281, 1128, 1083, 1047, 748, 694; HRMS (ESI): calcd. for $C_{17}H_{20}NO_3$, 286.1438. Found: $[MH]^+$, 286.1436 (0.3 error ppm).

2'-((2-Hydroxyethyl)(methyl)amino)-[1,1'-biphenyl]-2-carboxylic acid (S63)

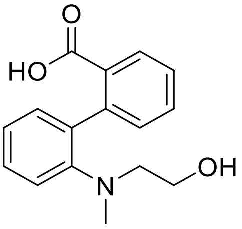

Methyl 2'-((2-hydroxyethyl)(methyl)amino)-[1,1'-biphenyl]-2-carboxylate **S62** (131 mg, 0.459 mmol) was dissolved in THF (1.4 mL) and then 0.5 M aq. LiOH (2.5 mL) was added, and the reaction mixture was heated to reflux for 24 h. This reaction was not performed under an inert atmosphere. The reaction mixture was concentrated *in vacuo* and then purified by column chromatography (SiO_2, 9:1 ethyl acetate:methanol) to yield the title compound (78 mg, 62%) as a yellow solid. R_f 0.57 (9:1 ethyl acetate:methanol); ν_{max}/cm^{-1} (thin film) 3379 (OH), 2956 (CH), 1702 (CO), 1596, 1481, 1449, 1401, 1259, 1046, 774, 746; δ_H (300 MHz, $CDCl_3$) 7.84 (1H, d, *J* = 7.5 Hz, ArH), 7.57 (1H, ddd, *J* = 7.5, 7.5, 1.0, ArH), 7.44–7.30 (3H, m, ArH), 7.24–7.09 (3H, m, ArH), 3.61–3.43 (2H, m, OCH_2), 2.89–2.84 (2H, m, NCH_2), 2.50 (3H, s, NCH_3); δ_C (75 MHz, $CDCl_3$) 171.8 (CO_2H), 149.8 (ArC), 139.7 (ArC), 135.9 (ArC), 132.0 (ArC), 131.7 (ArC), 131.3 (ArC), 131.2 (ArC), 130.0 (ArC), 128.8 (ArC), 127.5 (ArC), 124.2 (ArC), 119.8 (ArC), 59.1 (OCH_2), 58.2 (NCH_2), 40.1 (NCH_3); HRMS (ESI): calcd. for $C_{16}H_{18}NO_3$, 272.1281. Found: $[MH]^+$, 272.1272 (3.3 ppm error).

5-Methyl-6,7-dihydrodibenzo[*e*,*g*][1,4]oxazonin-9(5*H*)-one (60)

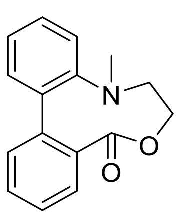

A round-bottom flask was charged with 2'-((2-hydroxyethyl)(methyl)amino)-[1,1'-biphenyl]-2-carboxylic acid **S63** (74 mg, 0.273 mmol) and DMF (3.1 mL). To this was added DIPEA (0.24 mL, 181 mg, 1.40 mmol), EDC·HCl (82 mg, 0.428 mmol) and HOBt (58 mg, 0.429 mmol). The resulting mixture was stirred at room temperature for 24 h. The reaction mixture was diluted with ethyl acetate (25 mL) and then washed with brine (3 x 25 mL). The combined aqueous layers were then extracted with ethyl acetate (2 x 25 mL). The combined organic layers were then dried over magnesium sulfate, filtered and concentrated *in vacuo*. The crude product

was purified by column chromatography (SiO_2, 4:1 hexane:ethyl acetate) to yield the title compound **60** (49 mg, 72%) as a colourless oil. R*f* 0.36 (4:1 hexane:ethyl acetate); δ_H (300 MHz, $CDCl_3$) 7.94 (1H, dd, *J* = 8.0, 1.5 Hz, ArH), 7.62–7.55 (2H, m, ArH), 7.51–7.31 (5H, m, ArH), 4.18 (2H, t, *J* = 6.5 Hz, OCH_2), 3.33 (2H, t, *J* = 6.5 Hz, NCH_2), 2.42 (3H, s, NCH_3); δ_C (75 MHz, $CDCl_3$) 168.1 (COO), 148.0 (ArC), 138.7 (ArC), 138.3 (ArC), 132.0 (ArC), 131.9 (ArC), 131.0 (ArC), 130.5 (ArC), 129.8 (ArC), 128.8 (ArC), 127.3 (ArC), 126.7 (ArC), 124.0 (ArC), 64.4 (OCH_2), 48.9 (NCH_2), 44.0 (NCH_3); ν_{max}/cm^{-1} (thin film) 2928 (CH), 1705 (CO), 1599, 1485, 1443, 1364, 1297, 1285, 1244, 1120, 1105, 1089, 1049, 1005, 901820, 782, 745, 692; HRMS (ESI): calcd. for $C_{16}H_{16}NO_2$, 254.1176. Found: $[MH]^+$, 254.1175 (−0.2 error ppm).

***N*-(3-Hydroxypropyl)-2-bromoaniline (S64)**

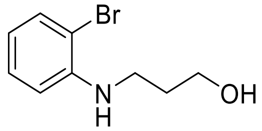

Sodium hydride (60% dispersion in mineral oil, 0.480 g, 12.0 mmol) was added to dry THF (3.1 mL). 2-Bromoaniline (1.03 g, 6.00 mmol) was dissolved in dry THF (1.0 mL) and then added dropwise to the sodium hydride suspension. This was stirred for 10 minutes before adding (3-bromopropoxy)-*tert*-butyldimethylsilane (1.40 mL, 1.52 g, 6.01 mmol). The reaction mixture was then stirred at room temperature for 18 h. The reaction was quenched with sat. aq. ammonium chloride (10 mL). Deionised water (20 mL) was added, and the product was extracted with diethyl ether (2 x 50 mL). The combined organic layers were dried over magnesium sulfate, filtered and concentrated *in vacuo*. The crude product was purified by column chromatography (SiO_2, 20:1 hexane:diethyl ether) to yield a mixture of *N*-(3-(*tert*-butyldimethylsiloxy)-propyl)-2-bromoaniline and (3-bromopropoxy)-*tert*-butyldimethylsilane (1.884 g, 3:1 ratio) as a yellow oil which was used without further purification. [R*f* 0.69 (20:1 hexane:diethyl ether); δ_H (300 MHz, $CDCl_3$) NMR signals for the desired product *N*-(3-(*tert*-butyldimethylsiloxy)-propyl)-2-bromoaniline only: 7.42 (1H, dd, *J* = 8.0, 1.5 Hz, ArH), 7.18 (1H, ddd, *J* = 8.0, 8.0, 1.5 Hz, ArH), 6.69 (1H, dd, *J* = 8.0, 1.5 Hz, ArH), 6.57 (1H, ddd, *J* = 8.0, 8.0, 1.5 Hz, ArH), 3.78 (2H, t, *J* = 5.0 Hz, OCH_2), 3.30 (2H, t, *J* = 7.0 Hz, NCH_2), 1.93–1.85 (2H, m, $NCH_2C\mathbf{H}_2$), 0.92 (9H, s, $C(CH_3)_3$), 0.09 (6H, s, $Si(CH_3)_2$). Characterisation data matched those reported in the literature.[10] Next, a dry round-bottom flask was charged with the impure sample of *N*-(3-(*tert*-butyldimethylsiloxy)-propyl)-2-bromoaniline (1.88 g) and THF (10 mL). A

solution of TBAF in THF (7.20 mL, 1 M, 7.20 mmol) was added dropwise and the reaction mixture was stirred at room temperature for 2 h. The mixture was then concentrated *in vacuo* and purified by column chromatography (SiO_2, 7:3 hexane:ethyl acetate) to yield the title compound **S64** (0.985 g, 71% over 2 steps) as a yellow oil. R_f 0.34 (7:3 hexane:ethyl acetate); δ_H (300 MHz, $CDCl_3$) 7.43 (1H, dd, *J* = 8.0, 1.5 Hz, ArH), 7.19 (1H, ddd *J* = 8.0, 8.0, 1.5 Hz, ArH), 6.69 (1H, dd, *J* = 8.0, 1.5 Hz, ArH), 6.58 (1H, ddd, *J* = 8.0, 8.0, 1.5 Hz, ArH), 3.85 (2H, t, *J* = 6.0 Hz, OCH_2), 3.34 (2H, t, *J* = 6.5 Hz, NCH_2), 1.99–1.91 (2H, m, $NCH_2C\mathbf{H}_2$); δ_C (75 MHz, $CDCl_3$) 145.1 (ArC), 132.4 (ArC), 128.5 (ArC), 117.8 (ArC), 111.4 (ArC), 109.9 (ArC), 61.2 (OCH_2), 41.4 (NCH_2), 31.7 ($NCH_2\mathbf{C}H_2$); ν_{max}/cm^{-1} (thin film) 3367 (OH), 2932 (CH), 1595, 1506, 1456, 1429, 1318, 1288, 1067, 1017, 925, 738, 661; HRMS (ESI): calcd. for $C_9H_{13}{}^{79}BrNO$, 230.0175. Found: $[MH]^+$, 230.0175 (0.3 error ppm).

***N*-(3-Hydroxypropyl)-*N*-methyl-2-bromoaniline (S65)**

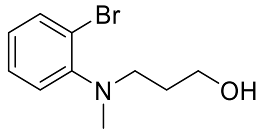

N-(3-Hydroxypropyl)-2-bromoaniline **S64** (0.913 g, 3.97 mmol) was dissolved in acetonitrile (22.8 mL) and then cooled to 0 °C. Acetic acid (2.3 mL, 2.46 g, 41.0 mmol), sodium cyanoborohydride (1.29 g, 20.5 mmol), and formaldehyde (37 wt.% in water, 6.1 mL, 82.4 mmol) were added and the mixture was stirred at room temperature for 20 h. Sat. aq. ammonium chloride (60 mL) was added and the resulting mixture was concentrated *in vacuo* to remove the organic solvent. Sat. aq. sodium bicarbonate (70 mL) was added, and the product was extracted with DCM (2 x 50 mL). The combined organic layers were dried over magnesium sulfate, filtered and concentrated *in vacuo*. The crude product was purified by column chromatography (SiO_2, 7:3 hexane:ethyl acetate) to yield the title compound **S65** (0.905 g, 90%) as a clear oil. R_f 0.34 (7:3 hexane:ethyl acetate); ν_{max}/cm^{-1} (thin film) 3317 (OH), 2946 (CH), 1585, 1475, 1458, 1438, 1420, 1173, 1115, 1058, 1025, 927, 758, 724, 654; δ_H (300 MHz, $CDCl_3$) 7.59 (1H, d, *J* = 8.0 Hz, ArH), 7.30 (1H, t, *J* = 8.0 Hz, ArH), 7.16 (1H, d, *J* = 8.0 Hz, ArH), 6.97 (1H, t, *J* = 8.0 Hz, ArH), 3.82 (2H, t, *J* = 5.5 Hz, OCH_2), 3.19 (2H, t, *J* = 6.0 Hz, NCH_2), 2.75 (3H, s, NCH_3), 1.88–1.80 (2H, m, $NCH_2C\mathbf{H}_2$); δ_C (75 MHz, $CDCl_3$) 151.3 (ArC), 133.8 (ArC), 128.3 (ArC), 125.4 (ArC), 122.3 (ArC), 120.9 (ArC), 63.3 (OCH_2), 55.5 (NCH_2), 42.6 (NCH_3), 28.8

($NCH_2\mathbf{C}H_2$); HRMS (ESI): calcd. for $C_{10}H_{15}{}^{79}BrNO^+$, 244.0332. Found: $[MH]^+$, 244.0327 (1.6 error ppm).

Methyl 2'-((3-hydroxypropyl)(methyl)amino)-[1,1'-biphenyl]-2-carboxylate (S66)

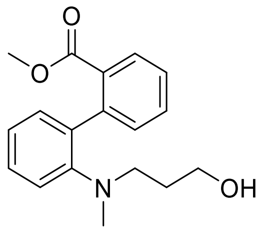

A dry round-bottom flask was charged with *N*-(3-hydroxypropyl)-*N*-methyl-2-bromoaniline **S65** (466 mg, 1.91 mmol), methyl 2-(4,4,5,5-tetramethyl-1,3,2-dioxaborolan-2-yl) benzoate (1.31 g, 5.00 mmol), bis(triphenylphosphine)palladium(II) dichloride (140 mg, 0.199 mmol) and potassium carbonate (1.11 g, 8.03 mmol). Dimethylacetamide (11 mL) and deionised water (2.2 mL) were added, and the reaction mixture was stirred at 80 °C for 24 h. Deionised water (60 mL) was added, and the product was extracted with ethyl acetate (3 x 40 mL). The combined organic layers were dried over magnesium sulfate, filtered and concentrated *in vacuo*. The crude product was purified by column chromatography (SiO_2, 4:1 toluene:ethyl acetate) to yield a mixture of impurity and title compound **S66** (382 mg, 52% from ^{1}H NMR spectrum) as a yellow oil which was used without further purification. R_f 0.25 (4:1 toluene:ethyl acetate); ν_{max}/cm^{-1} (thin film) 3408 (OH), 2949 (CH), 1717 (CO), 1597, 1506, 1480, 1430, 1372, 1279, 1124, 1051, 914, 744; δ_H (300 MHz, $CDCl_3$) 7.86 (1H, dd, *J* = 7.5, 1.5 Hz, ArH), 7.55 (1H, ddd, *J* = 7.5, 7.5, 1.5 Hz, ArH), 7.42–7.06 (6H, m, ArH), 3.63 (3H, s, OCH_3), 3.31–3.25 (2H, m, OCH_2), 2.78–2.67 (2H, m, NCH_2), 2.55 (3H, s, NCH_3), 1.57–1.48 (2H, m, $NCH_2C\mathbf{H}_2$); δ_C (75 MHz, $CDCl_3$) 168.5 ($\mathbf{C}O_2CH_3$), 151.4 (ArC), 141.5 (ArC), 136.1 (ArC), 131.7 (ArC), 131.1 (ArC), 131.0 (ArC), 130.3 (ArC), 129.7 (ArC), 129.2 (ArC), 128.5 (ArC), 127.0 (ArC), 123.0 (ArC), 61.41 (OCH_2), 54.4 (NCH_2), 51.8 (OCH_3), 39.7 (NCH_3), 29.6 ($NCH_2\mathbf{C}H_2$); HRMS (ESI): calcd. for $C_{18}H_{22}NO_3^+$, 300.1594. Found $[MH]^+$, 300.1587 (1.2 error ppm).

2'-((3-Hydroxypropyl)(methyl)amino)-[1,1'-biphenyl]-2-carboxylic acid (S67)

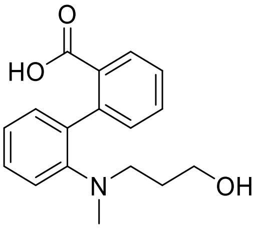

Methyl 2'-((3-hydroxypropyl)(methyl)amino)-[1,1'-biphenyl]-2-carboxylate **S66** (75 mg, 0.251 mmol) was dissolved in THF (0.7 mL) and then 0.5 M aq. LiOH (1.3 mL) was added, and the reaction mixture was heated to reflux for 24 h. This reaction was not carried out under an inert atmosphere. The reaction mixture was concentrated *in vacuo* and then purified by column chromatography (SiO_2, ethyl acetate→9:1 ethyl acetate:methanol) to yield the title compound **S67** (43 mg, 60%) as a white solid. R_f 0.13 (ethyl acetate); δ_H (300 MHz, $CDCl_3$) 7.84 (1H, dd, *J* = 7.5, 1.5 Hz, ArH), 7.54 (1H, ddd, *J* = 7.5, 7.5, 1.5 Hz, ArH), 7.45–7.26 (3H, m, ArH), 7.19–7.12 (3H, m, ArH), 3.32 (2H, t, *J* = 6.0 Hz, OCH_2), 2.89–2.73 (2H, m, NCH_2), 2.58 (3H, s, NCH_3), 1.60–1.49 (2H, m, $NCH_2C\mathbf{H}_2$); δ_C (75 MHz, $CDCl_3$) 171.3 (CO_2H), 149.3 (ArC), 139.3 (ArC), 136.1 (ArC), 132.7 (ArC), 131.8 (ArC), 131.5 (2ArC), 130.1 (ArC), 128.8 (ArC), 127.5 (ArC), 124.2 (ArC), 119.5 (ArC), 60.5 (OCH_2), 53.7 (NCH_2), 39.6 (NCH_3), 29.3 ($NCH_2\mathbf{C}H_2$); ν_{max}/cm^{-1} (thin film) 3379 (OH), 2934 (CH), 1695 (CO), 1595, 1478, 1444, 1254, 1132, 1051, 909, 767, 743, 647. HRMS (ESI): calcd. for $C_{17}H_{20}NO_3$, 286.1438. Found $[MH]^+$, 286.1436 (–0.1 error ppm)

5-Methyl-5,6,7,8-tetrahydro-10*H*-dibenzo[*f*,*h*][1,5]oxazecin-10-one (61)

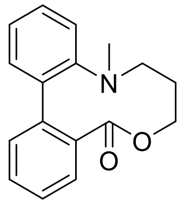

A round-bottom flask was charged with 2'-((3-hydroxypropyl)(methyl)amino)-[1,1'-biphenyl]-2-carboxylic acid **S67** (42 mg, 0.147 mmol) and DMF (1.7 mL). To this was added DIPEA (0.14 mL, 0.798 mmol), EDC·HCl (46 mg, 0.240 mmol) and HOBt (32 mg, 0.237 mmol). The resulting mixture was stirred at room temperature for 24 h. The reaction mixture was diluted with ethyl acetate (25 mL) and then washed with brine (3 x 25 mL). The combined aqueous layers were then extracted with ethyl acetate (2 x 25 mL). The combined organic layers were then dried over magnesium sulfate, filtered and concentrated *in vacuo*. The crude product was purified

by column chromatography (SiO_2, 4:1 hexane:ethyl acetate) to yield the title compound **61** (29 mg, 72%) as a white solid. R_f 0.50 (4:1 hexane:ethyl acetate); ν_{max}/cm^{-1} (thin film) 2956 (CH), 1708 (CO), 1597, 1485, 1445, 1358, 1289, 1271, 1248, 1127, 1104, 1076, 1047, 1013, 988, 908, 772, 741, 694; δ_H (300 MHz, $CDCl_3$) 7.67 (1H, dd, *J* = 7.5, 1.5 Hz, ArH), 7.57–7.49 (2H, m, ArH), 7.44–7.33 (3H, m, ArH), 7.24 (1H, ddd, *J* = 7.5, 7.5, 1.5 Hz, ArH), 7.09 (1H, dd, *J* = 7.5, 1.5 Hz, ArH), 4.78–4.70 (1H, m, OC**H**H'), 4.42–4.34 (1H, m, OCH**H'**), 3.41–3.31 (1H, m, NC**H**H'), 3.21–3.12 (1H, m, NCH**H'**), 2.19 (3H, s, NCH_3), 1.87–1.66 (2H, m, $NCH_2C\mathbf{H}_2$); δ_C (75 MHz, $CDCl_3$) 169.2 (COO), 149.6 (ArC), 137.3 (ArC), 136.4 (ArC), 132.4 (ArC), 131.0 (ArC), 130.7 (ArC), 130.6 (ArC), 128.6 (ArC), 128.3 (ArC), 127.1 (ArC), 124.9 (ArC), 122.2 (ArC), 63.0 (OCH_2), 52.0 (NCH_2), 42.6 (NCH_3), 24.6 ($NCH_2\mathbf{C}H_2$); HRMS (ESI): calcd. for $C_{17}H_{18}NO_2^+$, 268.1332. Found: $[MH]^+$, 268.1335 (−0.4 error ppm).

Methyl 2'-((2-hydroxypropyl)(methyl)amino)-[1,1'-biphenyl]-2-carboxylate (S68)

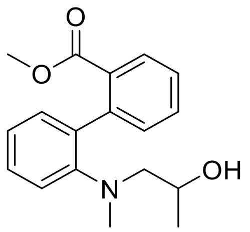

A round-bottom flask was charged with oxalyl chloride (0.040 mL, 62 mg, 0.488 mmol) and dry DCM (1.3 mL) before being cooled to −78 °C. A solution of DMSO (0.070 mL, 74 mg, 0.947 mmol) in dry DCM (0.64 mL) was then added dropwise and the resulting mixture was stirred for 5 minutes at −78 °C. A solution of methyl 2'-((2-hydroxyethyl)(methyl)amino)-[1,1'-biphenyl]-2-carboxylate **S62** (77 mg, 0.270 mmol) in dry DCM (3.8 mL) was then added dropwise and the resulting mixture was stirred for 15 minutes at −78 °C before adding triethylamine (0.320 mL, 233 mg, 2.30 mmol) dropwise. This was stirred for 5 minutes at −78 °C and then warmed to room temperature and stirred for 18 h. Deionised water (15 mL) was added, and the product was extracted with DCM (2 x 15 mL). The combined organic layers were dried over magnesium sulfate, filtered and concentrated *in vacuo* to yield crude methyl 2'-(methyl(2-oxoethyl)amino)-[1,1'-biphenyl]-2-carboxylate (93 mg) as a yellow oil, which was used without further purification. Next, a round-bottom flask charged with the unpurified sample of methyl 2'-(methyl(2-oxoethyl)amino)-[1,1'-biphenyl]-2-carboxylate (93 mg, 0.328 mmol) and dry Et_2O (0.60 mL) was cooled to −78 °C. A solution of MeLi in Et_2O (3.60 mL, 0.1

M, 0.360 mmol) was added dropwise and the resulting mixture was stirred for 0.5 h at −78 °C, then 0.5 h at room temperature. Deionised water (10 mL) was added and the product was extracted with DCM (2 x 10 mL). The combined organic layers were dried over magnesium sulfate, filtered and concentrated *in vacuo*. The crude product was purified by column chromatography (SiO_2, 4:1 hexane:ethyl acetate) to yield the title compound **S68** (21 mg, 26% over 2 steps) as a 1:1 mixture of diastereoisomers and as a yellow oil. R_f 0.16 (4:1 hexane:ethyl acetate); ν_{max}/cm^{-1} (thin film) 3465 (OH), 2944 (CH), 1720 (CO), 1597, 1498, 1480, 1444, 1291, 1127, 1084, 1052, 970, 747; δ_H (300 MHz, $CDCl_3$) 7.93–7.84 (2H, m, ArH), 7.61–7.54 (2H, m, ArH), 7.45–7.31 (6H, m, ArH), 7.23–7.13 (6H, m, ArH), 3.77 (2H, m, C**H**OH both diastereoisomers), 3.69 and 3.64 (3H, s, OCH_3), 2.73–2.41 (10H, m NCH_3, NCH_2 both diastereoisomers), 0.98 and 0.92 (3H, d, *J* = 6.0 Hz, CHC**H**$_3$); δ_C (75 MHz, $CDCl_3$) 168.7 (**C**O_2CH_3 both diastereoisomers), 151.0 (ArC), 141.0 (ArC), 140.9 (ArC), 136.8 (ArC), 131.8 (ArC), 131.7 (ArC), 131.2 (ArC), 131.0 (ArC), 130.4 (ArC), 130.3 (ArC), 130.0 (ArC), 129.7 (ArC), 128.6 (ArC), 128.5 (ArC), 127.3 (ArC), 127.2 (ArC), 124.0 (ArC), 123.5 (ArC), 120.6 (ArC), 120.0 (ArC), 65.0 and 64.3 (NCH_2), 63.9 and 63.4 (C**H**OH), 51.9 (OCH_3 both diastereoisomers), 41.8 and 40.2 (NCH_3), 20.2 and 19.9 (CH**C**H$_3$); HRMS (ESI): calcd. for $C_{18}H_{21}NO_3^+$, 300.1594. Found $[MH]^+$, 300.1592 (–0.4 error ppm).

2'-((2-Hydroxypropyl)(methyl)amino)-[1,1'-biphenyl]-2-carboxylic acid (S69)

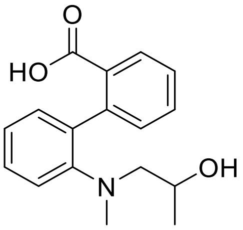

Methyl 2'-((2-hydroxypropyl)(methyl)amino)-[1,1'-biphenyl]-2-carboxylate **S68** (201 mg, 0.671 mmol) was dissolved in THF (1.5 mL), then 0.5 M aq. LiOH (2.6 mL) was added, and the reaction mixture was heated to reflux for 24 h. This reaction was not carried out under an inert atmosphere. The reaction mixture was concentrated *in vacuo* and then purified by column chromatography (SiO_2, ethyl acetate→9:1 ethyl acetate:methanol) to yield the title compound (89 mg, 46%) as a 1:1 mixture of diastereoisomers and as a yellow solid. R_f 0.22 (ethyl acetate); ν_{max}/cm^{-1} (thin film) 3390 (OH), 2969 (CH), 1687 (CO), 1595, 1480, 1446, 1393, 1271, 1138, 1055, 971, 726, 646; δ_H (300 MHz, $CDCl_3$) 7.80–7.71 (2H, m, ArH), 7.54–7.44 (2H,

m, ArH), 7.37–7.21 (6H, m, ArH), 7.17–7.00 (6H, m, ArH), 3.83–3.61 (2H, m, C**H**OH both diastereoisomers), 2.80–2.33 (10H, m, NCH_2, NCH_3 both diastereoisomers), 0.96–0.81 (6H, m, CHC**H**$_3$ both diastereoisomers); δ_C (75 MHz, $CDCl_3$) 173.5 (CO_2H both diastereoisomers), 151.4 (ArC), 150.3 (ArC), 140.5 (ArC), 140.1 (ArC), 136.6 (ArC), 136.3 (ArC), 133.3 (ArC), 131.1 (ArC), 130.9 (ArC), 130.0 (ArC), 129.7 (ArC), 128.3 (ArC), 128.2 (ArC), 127.1 (ArC), 123.3 (ArC), 123.2 (ArC), 119.8 (ArC), 119.5 (ArC), 64.7 and 63.7 (CHOH), 63.8 (NCH_2 both diastereoisomers), 41.8 and 40.3 (NCH_3), 20.2 (CH**C**H_3 both diastereoisomers); HRMS (ESI): calcd. for $C_{17}H_{20}NO_3^+$, 286.1438. Found $[MH]^+$, 286.1440 (-0.5 error ppm).

5,7-Dimethyl-6,7-dihydrodibenzo[e,g][1,4]oxazonin-9(5H)-one (62)

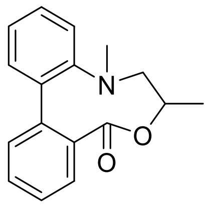

A round-bottom flask was charged with 2'-((2-hydroxypropyl)(methyl)amino)-[1,1'-biphenyl]-2-carboxylic acid **S69** (63 mg, 0.221 mmol) and DMF (2.6 mL). To this was added DIPEA (0.210 mL, 1.20 mmol), EDC·HCl (67 mg, 0.350 mmol) and HOBt (47 mg, 0.348 mmol). The resulting mixture was stirred at room temperature for 24 h. The reaction mixture was diluted with ethyl acetate (20 mL) and then washed with brine (3 x 20 mL). The combined aqueous layers were then extracted with ethyl acetate (2 x 20 mL). The combined organic layers were then dried over magnesium sulfate, filtered and concentrated *in vacuo*. The crude product was purified by column chromatography (SiO_2, 9:1 hexane:ethyl acetate) to yield the title compound **62** (48 mg, 81%) as a single diastereoisomer as a clear oil. R_f 0.31 (9:1 hexane:ethyl acetate); ν_{max}/cm^{-1} (thin film) 2931 (CH), 1701 (CO), 1599, 1483, 1443, 1336, 1287, 1247, 1117, 1104, 1050, 966, 913, 770, 742, 689, 662; δ_H (300 MHz, $CDCl_3$) 7.91 (1H, dd, *J* = 7.5, 1.5 Hz, ArH), 7.62–7.54 (2H, m, ArH), 7.50–7.31 (5H, m, ArH), 4.73–4.62 (1H, m, OCH), 3.36 (1H, dd, *J* = 11.5, 7.5 Hz, NC**H**H'), 3.17 (1H, dd, *J* = 11.5, 5.5 Hz, NCH**H'**), 2.42 (3H, s, NCH_3), 1.16 (3H, d, *J* = 6.5 Hz, CHC**H**$_3$); δ_C (75 MHz, $CDCl_3$) 168.6 (COO), 148.7 (ArC), 138.4 (ArC), 137.8 (ArC), 132.5 (ArC), 131.6 (ArC), 131.1 (ArC), 130.4 (ArC), 129.7 (ArC), 128.7 (ArC), 127.4 (ArC), 126.8 (ArC), 124.8 (ArC), 73.8 (OCH), 55.8 (NCH_2), 44.3 (NCH_3), 20.1 (CH**C**H_3); HRMS (ESI): calcd. for $C_{17}H_{18}NO_2$, 268.1332. Found $[MH]^+$, 268.1334 (–1.3 error ppm).

Methyl 2'-((3-hydroxybutyl)(methyl)amino)-[1,1'-biphenyl]-2-carboxylate (S70)

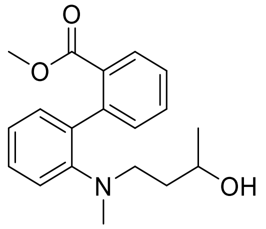

A round-bottom flask was charged with oxalyl chloride (0.050 mL, 75 mg, 0.591 mmol) and dry DCM (1.6 mL) before being cooled to −78 °C. A solution of DMSO (0.090 mL, 94 mg, 1.20 mmol) in dry DCM (0.81 mL) was then added dropwise and the resulting mixture was stirred for 5 minutes at −78 °C. A solution of methyl 2'-((3-hydroxypropyl)(methyl)amino)-[1,1'-biphenyl]-2-carboxylate **S66** (98 mg, 0.327 mmol) in dry DCM (4.6 mL) was then added dropwise and the resulting mixture was stirred for 15 minutes at −78 °C before adding triethylamine (0.390 mL, 283 mg, 2.78 mmol) dropwise. This was stirred for 5 minutes at −78 °C and then warmed to room temperature and stirred for 18 h. Deionised water (15 mL) was added, and the product was extracted with DCM (2 x 15 mL). The combined organic layers were dried over magnesium sulfate, filtered and concentrated *in vacuo* to yield crude methyl 2'-(methyl(3-oxopropyl)amino)-[1,1'-biphenyl]-2-carboxylate (104 mg) as a yellow oil, which was used without further purification. Next, a round-bottom flask charged with the unpurified sample of 2'-(methyl(3-oxopropyl)amino)-[1,1'-biphenyl]-2-carboxylate (104 mg) and dry Et_2O (0.56 mL) was cooled to −78 °C. A solution of methyl litium in Et_2O (4.50 mL, 0.1 M, 0.450 mmol) was added dropwise and the resulting mixture was stirred for 0.5 h at −78 °C, then 0.5 h at room temperature. Deionised water (10 mL) was added and the product was extracted with DCM (2 x 10 mL). The combined organic layers were dried over magnesium sulfate, filtered and concentrated *in vacuo*. The crude product was purified by column chromatography (SiO_2, 4:1 hexane:ethyl acetate) to yield the title compound (30 mg, 29% over 2 steps) as a 1:1 mixture of diastereoisomers and as a colourless oil. R_f 0.10 (4:1 hexane:ethyl acetate); ν_{max}/cm^{-1} (thin film) 3421 (OH), 2925 (CH), 1719 (CO), 1597, 1498, 1481, 1445, 1374, 1290, 1261, 1184, 1125, 1084, 938, 745, 712; δ_H (300 MHz, $CDCl_3$) 7.91–7.88 (2H, m, ArH), 7.59–7.56 (2H, m, ArH), 7.44–7.32 (6H, m, ArH), 7.16–7.08 (6H, m, ArH), 6.67–3.65 (6H, m, OCH_3 both diastereoisomers), 3.49–3.39 (2H, m, C**H**OH both diastereoisomers), 2.86–2.69 (4H, m, NCH_2 both diastereoisomers), 2.58–2.56 (6H, m, NCH_3 both diastereoisomers), 1.49–1.38 (4H, m, NCH_2C**H_2** both diastereoisomers), 1.01–0.98 (6H,

m, CHC**H**$_3$ both diastereoisomers); δ_C (75 MHz, $CDCl_3$) 168.4 and 168.3 (**C**O_2CH_3) 151.3 (ArC), 141.6 (ArC), 136.2 (ArC), 134.9 (ArC), 133.5 (ArC), 131.7 (ArC), 131.6 (ArC), 131.1 (ArC), 131.0 (ArC), 130.4 (ArC), 130.3 (ArC), 129.9 (ArC), 129.8 (ArC), 129.2 (ArC), 128.5 (ArC), 128.4 (ArC), 127.0 (ArC), 126.9 (ArC), 123.1 (ArC), 123.0 (ArC), 119.6 (ArC), 119.5 (ArC), 119.4 (ArC), 112.9 (ArC), 67.1 and 66.9 (CHOH) 54.5 and 54.3 (NCH_2), 51.8 (OCH_3 both diastereoisomers), 39.9 and 39.8 (NCH_3), 35.8 and 35.7 (NCH_2**C**H_2), 23.4 and 23.3 (CH**C**H_3); HRMS (ESI): calcd. for $C_{19}H_{24}NO_3^+$, 314.1751. Found $[MH]^+$, 314.1751 (0.1 error ppm).

2'-((3-Hydroxybutyl)(methyl)amino)-[1,1'-biphenyl]-2-carboxylic acid (S71)

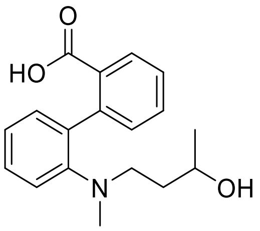

Methyl 2'-((3-hydroxybutyl)(methyl)amino)-[1,1'-biphenyl]-2-carboxylate **S70** (30 mg, 0.096 mmol) was dissolved in THF (0.22 mL), then 0.5 M aq. LiOH (0.4 mL) was added, and the reaction mixture was heated to reflux for 24 h. This reaction was not carried out under an inert atmosphere. The reaction mixture was concentrated *in vacuo* and then purified by column chromatography (SiO_2, ethyl acetate→9:1 ethyl acetate:methanol) to yield the title compound (15 mg, 50%) as a 1:1 mixture of diastereoisomers and as a white solid. R_f 0.24 (ethyl acetate); ν_{max}/cm^{-1} (thin film) 3368 (OH), 2926 (CH), 1698 (CO), 1596, 1479, 1446, 1377, 1262, 1130, 1082, 910, 744, 647; δ_H (300 MHz, $CDCl_3$) 7.87–7.82 (2H, m, ArH), 7.58–7.52 (2H, m, ArH), 7.48–7.33 (4H, m, ArH), 7.29–7.25 (2H, m, ArH), 7.20–7.14 (6H, m, ArH), 3.56–3.44 (2H, m, C**H**OH both diastereoisomers), 2.98–2.72 (4H, m, NCH_2 both diastereoisomers), 2.66–2.59 (6H, m, NCH_3 both diastereoisomers), 1.56–1.31 (4H, m, NCH_2C**H**$_2$ both diastereoisomers), 1.03–0.96 (6H, m, CHC**H**$_3$ both diastereoisomers); δ_C (75 MHz, $CDCl_3$) 170.9 (CO_2H both diastereoisomers), 148.8 (ArC), 138.7 (ArC), 136.1 (ArC), 131.0 (ArC), 133.2 (ArC), 132.2 (ArC), 131.7 (ArC), 131.6 (ArC), 131.4 (ArC), 130.2 (ArC), 130.1 (ArC), 129.0 (ArC), 128.9 (ArC), 127.7 (ArC), 124.5 (ArC), 124.4 (ArC), 66.2 (CHOH both diastereoisomers), 53.9 and 53.6 (NCH_2), 39.8 and 39.5 (NCH_3), 35.7 and 35.4 (NCH_2**C**H_2), 23.5 (CH**C**H_3 both diastereoisomers); HRMS (ESI): calcd. for $C_{18}H_{22}NO_3$, 300.1594. Found $[MH]^+$, 300.1596 (–0.8 error ppm).

5,8-Dimethyl-5,6,7,8-tetrahydro-10H-dibenzo[f,h][1,5]oxazecin-10-one (63)

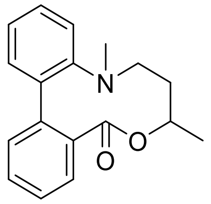

A round-bottom flask was charged with 2'-((3-hydroxybutyl)(methyl)amino)-[1,1'-biphenyl]-2-carboxylic acid **S71** (15 mg, 0.050 mmol) and DMF (0.58 mL). To this was added DIPEA (0.050 mL, 0.271 mmol), EDC·HCl (15 mg, 0.078 mmol) and HOBt (11 mg, 0.081 mmol). The resulting mixture was stirred at room temperature for 24 h. The reaction mixture was diluted with ethyl acetate (5 mL) and then washed with brine (3 x 5 mL). The combined aqueous layers were then extracted with ethyl acetate (2 x 5 mL). The combined organic layers were then dried over magnesium sulfate, filtered and concentrated *in vacuo*. The crude product was purified by column chromatography (SiO_2, 9:1 hexane:ethyl acetate) to yield the title compound (11 mg, 78%) as a 1.1:1 (A:B) mixture of diastereoisomers and as a white solid. R_f 0.36 (9:1 hexane:ethyl acetate); δ_H (400 MHz, $CDCl_3$) 7.82 (1H, d, *J* = 8.0 Hz, ArH), 7.58–7.29 (12H, m, ArH), 7.21–7.13 (2H, m, ArH), 7.00 (1H, d, *J* = 8.0 Hz, ArH), 5.66–5.56 (1H, m, OCH diastereoisomer A), 5.53–5.44 (1H, m, OCH diastereoisomer B), 3.42–3.28 (2H, m, NCH_2 diastereoisomer A), 3.20–3.13 (2H, m, NCH_2 diastereoisomer B), 2.22–2.15 (6H, m, NCH_3 both diastereoisomers), 1.96–1.72 (2H, m, $NCH_2C\mathbf{H_2}$ diastereoisomer A), 1.56–1.42 (2H, m, $NCH_2C\mathbf{H_2}$ diastereoisomer B), 1.39 (3H, d, *J* = 6.5 Hz, $CHC\mathbf{H_3}$ diastereoisomer B), 1.04 (3H, d, *J* = 6.5 Hz, $CHC\mathbf{H_3}$ diastereoisomer A); δ_C (101 MHz, $CDCl_3$) 169.0 (COO both diastereoisomers), 152.2 (ArC), 147.3 (ArC), 137.6 (ArC), 137.4 (ArC), 137.0 (ArC), 135.1 (ArC), 133.9 (ArC), 131.7 (ArC), 131.6 (ArC), 131.3 (ArC), 131.0 (ArC), 130.4 (ArC), 130.3 (ArC), 129.8 (ArC), 129.0 (ArC), 128.7 (ArC), 128.3 (ArC), 127.4 (ArC), 126.9 (ArC), 126.0 (ArC), 125.4 (ArC), 124.5 (ArC), 123.1 (ArC), 121.4 (ArC), 70.1 (OCH diastereoisomer A), 69.9 (OCH diastereoisomer B), 52.7 (NCH_2 diastereoisomer A), 50.4 (NCH_2 diastereoisomer B), 44.2 (NCH_3 diastereoisomer B), 40.6 (NCH_3 diastereoisomer A), 32.3 ($NCH_2\mathbf{C}H_2$ diastereoisomer B), 31.8 ($NCH_2\mathbf{C}H_2$ diastereoisomer A), 21.0 ($CH\mathbf{C}H_3$ diastereoisomer B), 20.8 ($CH\mathbf{C}H_3$ diastereoisomer A); ν_{max}/cm^{-1} (thin film) 2927 (CH), 1721 (CO), 1703 (CO), 1597, 1485, 1445, 1289, 1273, 1125, 1077, 1051, 963, 742, 695; HRMS (ESI): calcd. for $C_{18}H_{20}NO_2^+$, 282.1489. Found $[MH]^+$, 282.1488 (–0.2 error ppm).

Intermediates used to make lactams 64 and 65

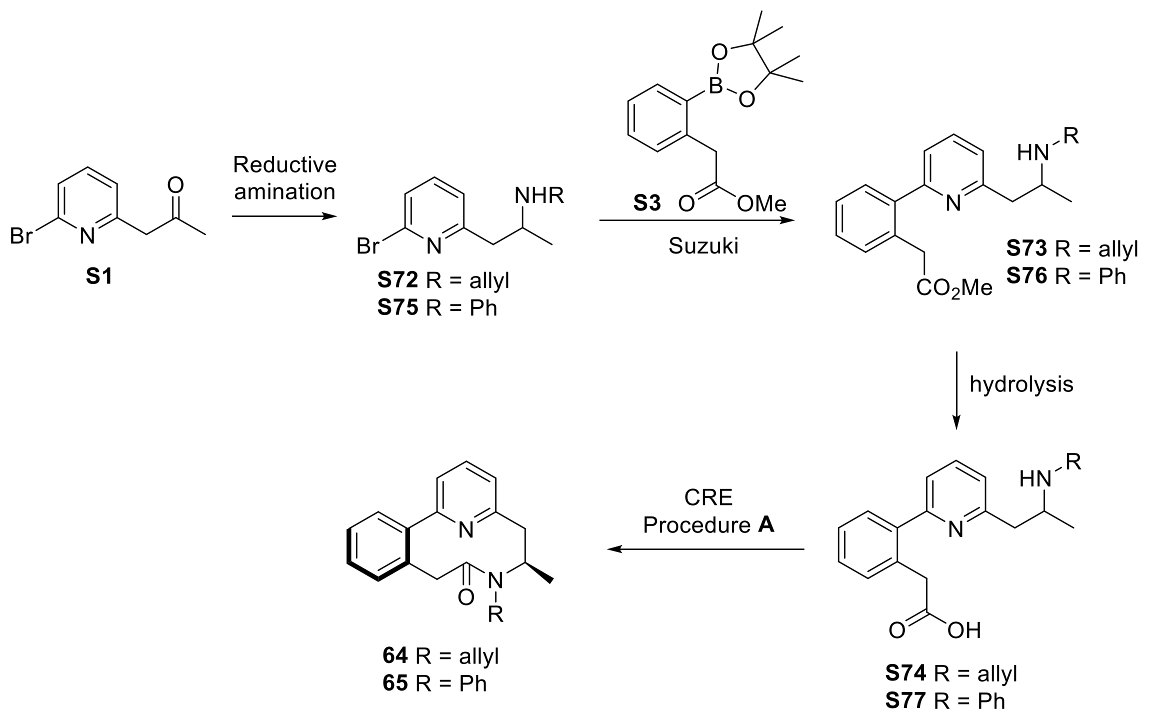

N-(1-(6-bromopyridin-2-yl)propan-2-yl)prop-2-en-1-amine (S72)

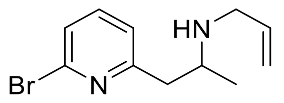

To a solution of 1-(6-bromopyridin-2-yl)propan-2-one **S1** (270 mg, 1.26 mmol) in dichloroethane (4.5 mL) at r.t., allylamine (72 mg, 1.261 mmol), acetic acid (0.095 mL, 1.26 mmol) and sodium triacetoxyborohydride (401 mg, 1.89 mmol) were added sequentially. The reaction mixture was stirred at r.t. overnight. It was then quenched with 1 M NaOH and extracted with ethyl acetate (3 × 30 mL). The combined organic layers were dried over anhydrous $MgSO_4$, filtered and concentrated *in vacuo*. Purification *via* flash column chromatography (SiO_2, 2:3 ethyl acetate:hexane → 4:1 ethyl acetate:methanol) afforded the *title compound* as a yellow oil (112 mg, 33%); R_f 0.15 (ethyl acetate:methanol 85:15); ν_{max}/cm^{-1} (thin film) 3287, 3075, 2967, 1643, 1581, 1552, 1404, 1375, 1201, 1159, 1089; δ_H (400 MHz, $CDCl_3$) 7.42 (1H, t, *J* = 7.6 Hz, ArH), 7.28 (1H, d, *J* = 7.6 Hz, ArH), 7.08 (1H, d, *J* = 7.6 Hz, ArH), 5.89−5.79 (1H, m, C**H**=CH$_2$), 5.16−5.04 (2H, m, CH=C**H$_2$**), 3.33−3.28 (1H, m, C**H**H-NH), 3.22−3.15 (2H, m, CH**H**-NH and C**H**-CH$_3$), 2.90 (1H, dd, *J* = 13.7, 6.9 Hz, NC-C**H**H), 2.73 (1H, dd, *J* = 14.2, 6.1 Hz, NC-CH**H**), 1.05 (3H, d, *J* = 6.9 Hz, CH$_3$); δ_C (101 MHz, $CDCl_3$) 161.1 (ArC), 138.6 (ArC), 135.9 (**C**H=CH$_2$), 125.6 (ArC), 122.7 (ArC), 116.5 (CH=**C**H$_2$), 52.6 (CH-NH), 49.4 (NH-CH$_2$),

44.4 (NC-CH_2), 19.7 (CH_3); HRMS (ESI): calcd. for $C_{11}H_{16}{}^{79}BrN_2$ 255.0491. Found: $[MH]^+$, 255.0494 (−1.1 ppm error).

Methyl 2-(2-(6-(2-(allylamino)propyl)pyridin-2-yl)phenyl)acetate (S73)

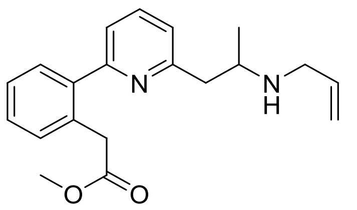

To a microwave vial, methyl 2-(2-(4,4,5,5-tetramethyl-1,3,2-dioxaborolan-2-yl)phenyl)acetate **S3** (131 mg, 0.475 mmol), potassium triphosphate (152 mg, 0.714 mmol) and $Pd(PPh_3)_4$ (27.5 mg, 0.0238 mmol) was added and the vial was purged with argon. Dimethylformamide (1.5 mL) and *N*-(1-(6-bromopyridin-2-yl)propan-2-yl)prop-2-en-1-amine **S72** (60.7 mg, 0.285 mmol) were added, and the solution was heated and stirred for 90 min at 150 °C in a microwave reactor. The solution was then cooled, diluted with ethyl acetate and passed through Celite®. The solvent was then evaporated *in vacuo*. Water (10 mL), followed by ethyl acetate (20 mL) were added and both layers were separated. The aqueous layers were extracted with ethyl acetate (3 × 20 mL). The combined organic layers were dried with anhydrous $MgSO_4$, filtered, and concentrated *in vacuo*. Purification *via* flash column chromatography (SiO_2, 1:4 ethyl acetate:hexane → 3:1 ethyl acetate:methanol) afforded the *title compound* as a yellow oil (40.4 mg, 52%); R_f 0.21 (7:3 ethyl acetate:methanol); ν_{max}/cm^{-1} (thin film) 2951, 1735, 1569, 1447, 1339, 1252, 1212, 1160; δ_H (400 MHz, $CDCl_3$) 7.70 (1H, t, *J* = 7.8 Hz, ArH), 7.43−7.33 (5H, m, ArH), 7.31 (1H, d, *J* = 7.3 Hz, ArH), 7.13 (1H, d, *J* = 7.3 Hz, ArH), 5.89−5.81 (1H, m, C**H**=CH_2), 5.17−5.06 (2H, m, CH=C**H₂**), 3.82 (2H, s, CH_2-CO), 3.57 (3H, s, COO-CH_3), 3.48−3.42 (1H, m, C**H**H-NH), 3.37−3.26 (2H, m, CH**H**-NH and C**H**-CH_3), 3.10 (1H, dd, *J* = 14.2, 7.3 Hz, NC-C**H**H), 2.90 (1H, dd, *J* = 14.2, 5.9 Hz, NC-C**H**H), 1.21 (3H, d, *J* = 6.4 Hz, CH_3); δ_C (101 MHz, $CDCl_3$) 172.2 (CO), 158.9 (ArC), 158.5 (ArC), 137.1 (ArC), 134.7 (**C**H=CH_2), 132.2 (ArC), 131.2 (ArC), 129.8 (ArC), 128.6 (ArC), 127.4 (ArC), 122.1 (ArC), 121.8 (ArC), 117.6 (CH=**C**H_2), 52.9 (**C**H-CH_3), 51.9 (COO-**C**H_3), 48.9 (NH-CH_2), 43.8 (NC-**C**H_2), 39.0 (**C**H_2-CO), 19.1 (CH_3); HRMS (ESI): calcd. for $C_{20}H_{25}N_2O_2$ 325.1911. Found: $[MH]^+$, 325.1903 (2.2 ppm error).

2-(2-(6-(2-(Allylamino)propyl)pyridin-2-yl)phenyl)acetic acid (S74)

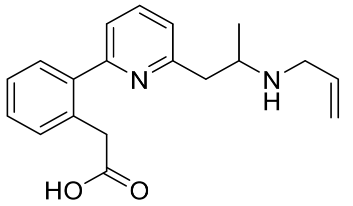

To methyl 2-(2-(6-(2-(allylamino)propyl)pyridin-2-yl)phenyl)acetate **S73** (35.2 mg, 0.109 mmol), was added 0.5 M aqueous LiOH (0.3 mL) and THF (0.3 mL) and the resulting solution was stirred for 16 h. The resulting solution was then removed *in vacuo* to afford the crude *title compound* as a colorless oil, which was used in the next step without further purification.

3-Allyl-4-methyl-4,5-dihydro-1*H*-6,10-(azeno)benzo[*d*][1]azacyclododecin-2(3*H*)-one (64)

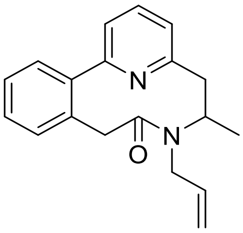

To a stirring solution of unpurified 2-(2-(6-(2-(allylamino)propyl)pyridin-2-yl)phenyl)acetic acid **S74** (33.8 mg, 0.109 mmol) in chloroform (2.6 mL), was added diisopropylethylamine (35.1 µL, 0.202 mmol), followed by the addition of T3P (50% solution in ethyl acetate, 104 mg, 0.164 mmol). After stirring for 30 mins at r.t., the solution was taken directly to purification with no work-up. Purification *via* flash column chromatography (SiO_2, 3:7 → 2:3 ethyl acetate:hexane) afforded the *title compound* **64** as a colorless oil (24.0 mg, 75% over 2 steps from **S73**); ν_{max}/cm^{-1} (thin film) 3067, 3011, 2966, 2929, 1625, 1586, 1578, 1565, 1430, 1397, 1319, 1301, 1266, 1210, 1180, 1157, 1121, 1095, 1048; δ_H (400 MHz, $CDCl_3$) 7.89−7.86 (1H, m, ArH), 7.64 (1H, t, *J* = 7.9 Hz, ArH), 7.55 (1H, d, *J* = 8.3 Hz, ArH), 7.42−7.34 (3H, m, ArH), 6.94 (1H, d, *J* = 7.6 Hz, ArH), 5.73−5.63 (1H, m, C**H**=CH_2), 5.16−5.06 (3H, m, C**H**H-CO, C**H**-CH_3 and CH=C**H**H), 4.99−4.95 (1H, m, CH=CH**H**), 3.83−3.78 (1H, m, NC**H**H), 3.52−3.46 (1H, m, NCH**H**), 3.32 (1H, d, *J* = 13.3 Hz, CH**H**-CO), 3.05−3.03 (2H, m, NC-CH_2), 1.47 (3H, d, *J* = 6.9 Hz, CH_3); δ_C (101 MHz, $CDCl_3$) 174.2 (CO), 156.5 (ArC), 155.6 (ArC), 137.5 (ArC), 137.2 (**C**H=CH_2), 135.9 (ArC), 135.8 (ArC), 135.5 (ArC), 129.1 (ArC), 127.2 (ArC), 126.7 (ArC), 120.1 (ArC), 117.3 (ArC),

115.3 (CH=**C**H_2), 53.5 (NCH), 43.2 (NCH_2), 42.7 (**C**H_2-CO), 39.7 (NC**C**H_2), 20.2 (CH_3); HRMS (ESI): calcd. for $C_{19}H_{20}N_2NaO$ 315.1468. Found: $[MNa]^+$, 315.1464 (0.3 ppm error).

***N*-(1-(6-bromopyridin-2-yl)propan-2-yl)aniline (S75)**

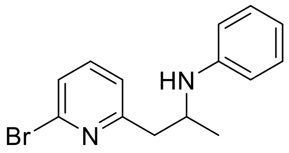

To a solution of 1-(6-bromopyridin-2-yl)propan-2-one **S1** (383 mg, 1.79 mmol) in dichloroethane (6.3 mL) at r.t., aniline (166 mg, 1.79 mmol), acetic acid (0.102 mL, 1.79 mmol) and sodium triacetoxyborohydride (568 mg, 2.68 mmol) were added sequentially. The reaction mixture was stirred at r.t. overnight. It was then quenched with 1 M NaOH and extracted with ethyl acetate (3 × 30 mL). The combined organic layers were dried over anhydrous $MgSO_4$, filtered and concentrated *in vacuo*. Purification *via* flash column chromatography (SiO_2, 1:9 → 1:4 ethyl acetate:hexane) afforded the *title compound* as a yellow oil (256 mg, 49%); R_f 0.49 (4:1 hexane:ethyl acetate); ν_{max}/cm^{-1} (thin film) 3369, 3050, 3020, 2964, 2924, 1600, 1580, 1551, 1503, 1431, 1403, 1376, 1316, 1291, 1254, 1227, 1198, 1179, 1157, 1122, 1089, 1059, 1028; δ_H (400 MHz, $CDCl_3$) 7.41 (1H, t, *J* = 7.6 Hz, ArH), 7.30 (1H, d, *J* = 7.6 Hz, ArH), 7.19−7.15 (2H, m, ArH), 7.09 (1H, d, *J* = 6.9 Hz, ArH), 6.68 (1H, t, *J* = 7.3 Hz, ArH), 6.64−6.62 (2H, m, ArH), 3.96−3.91 (2H, m, CH-NH), 3.00 (1H, dd, *J* = 13.3, 6.5 Hz, NC-C**H**H), 3.08 (1H, dd, *J* = 13.3, 5.7 Hz, NC-CH**H**), 1.22 (3H, d, *J* = 6.9 Hz, CH_3); δ_C (101 MHz, $CDCl_3$) 160.8 (ArC), 147.1 (ArC), 141.3 (ArC), 138.5 (ArC), 129.2 (ArC), 125.6 (ArC), 122.6 (ArC), 122.5 (ArC), 117.1 (ArC), 113.2 (ArC), 48.8 (CH-NH), 44.2 ($NCCH_2$), 20.6 (CH_3); HRMS (ESI): calcd. for $C_{14}H_{16}{}^{79}BrN_2$ 291.0491. Found: $[MH]^+$, 291.0486 (1.8 ppm error).

Methyl 2-(2-(6-(2-(phenylamino)propyl)pyridin-2-yl)phenyl)acetate (S76)

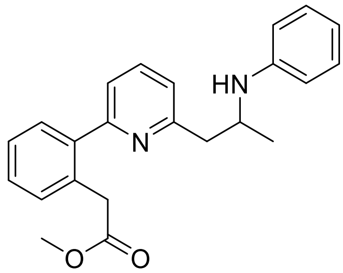

To a microwave vial, methyl 2-(2-(4,4,5,5-tetramethyl-1,3,2-dioxaborolan-2-yl)phenyl)acetate **S3** (247 mg, 0.892 mmol), potassium triphosphate (284 mg, 1.34 mmol) and $Pd(PPh_3)_4$ (51.5 mg, 0.0446 mmol) was added and the vial was purged with argon. Dimethylformamide (2.3 mL) and *N*-(1-(6-bromopyridin-2-yl)propan-2-yl)aniline **S75** (130 mg, 0.446 mmol) were added, and the solution was heated and stirred for 90 min at 150 ^{o}C in a microwave reactor. The solution was then cooled, diluted with ethyl acetate and passed through Celite®. The solvent was then evaporated *in vacuo*. Water (10 mL), followed by ethyl acetate (30 mL) were added and both layers were separated. The aqueous layers were extracted with ethyl acetate (3 × 20 mL). The combined organic layers were dried with anhydrous $MgSO_4$, filtered, and concentrated *in vacuo*. Purification *via* flash column chromatography (SiO_2, 1:9 → 1:4 ethyl acetate:hexane) afforded the *title compound* as a yellow oil (86.1 mg, 54%); R_f 0.52 (4:1 hexane:ethyl acetate); ν_{max}/cm^{-1} (thin film) 3338, 2970, 2932, 2883, 1466, 1408, 1378, 1340, 1306, 1160, 1128, 1107; δ_H (400 MHz, $CDCl_3$) 7.66 (1H, t, *J* = 7.6 Hz, ArH), 7.48−7.38 (4H, m, ArH), 7.32 (1H, d, *J* = 7.6 Hz, ArH), 7.19−7.10 (3H, m, ArH), 6.71−6.61 (3H, m, ArH), 3.99−3.94 (1H, m, C**H**-NH), 3.88 (2H, s, CH_2CO), 3.61 (3H, s, OCH_3), 3.07 (1H, dd, *J* = 13.7, 6.9 Hz, NCC**H**H), 2.98 (1H, dd, *J* = 13.7, 5.7 Hz, NCCH**H**), 1.26 (3H, d, *J* = 6.1 Hz, CH_3); δ_C (101 MHz, $CDCl_3$) 172.4 (CO), 158.7 (ArC), 158.6 (ArC), 147.4 (ArC), 140.3 (ArC), 136.8 (ArC), 132.4 (ArC), 131.5 (ArC), 129.9 (ArC), 129.1 (ArC), 128.4 (ArC), 127.4 (ArC), 121.6 (ArC), 121.5 (ArC), 116.7 (ArC), 113.0 (ArC), 51.8 (OCH_3), 48.9 (CH-NH), 44.9 (NC**C**H_2), 39.4 (**C**H_2-CO), 20.7 (CH_3); HRMS (ESI): calcd. for $C_{23}H_{25}N_2O_2$ 361.1911. Found: $[MH]^+$, 361.1908 (0.8 ppm error).

2-(2-(6-(2-(Phenylamino)propyl)pyridin-2-yl)phenyl)acetic acid (S77)

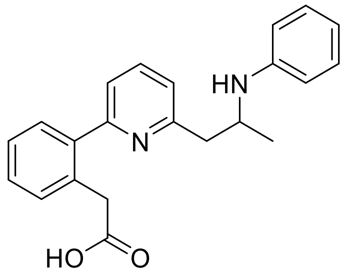

To methyl 2-(2-(6-(2-(phenylamino)propyl)pyridin-2-yl)phenyl)acetate **S76** (73.6 mg, 0.204 mmol), was added 0.5 M aqueous LiOH (0.6 mL) and THF (0.6 mL) and the resulting solution was stirred for 16 h. The resulting solution was then removed *in vacuo*. Purification by column chromatography (SiO_2, 1:1 ethyl acetate:hexane → ethyl acetate) afforded the *title compound* as a pale orange oil (59.0 mg, 83%); R_f 0.18 (7:3 hexane:ethyl acetate); ν_{max}/cm^{-1} (thin film) 3355, 3052, 2968, 2453, 1720, 1599, 1578, 1497, 1450, 1377, 1316, 1259, 1144, 1100, 1013; δ_H (400 MHz, $CDCl_3$) 7.89 (1H, t, *J* = 7.6 Hz, ArH), 7.56−7.37 (6H, m, ArH), 7.13−7.09 (2H, m, ArH), 6.65 (1H, d, *J* = 7.3 Hz, ArH), 6.53−6.51 (2H, m, ArH), 4.01−3.92 (1H, m, C**H**-NH), 3.63 (2H, s, CH_2CO), 3.17−3.07 (2H, m, $NCCH_2$), 1.29 (3H, d, *J* = 6.9 Hz, CH_3); δ_C (101 MHz, $CDCl_3$) 172.8 (CO), 157.7 (ArC), 156.8 (ArC), 146.8 (ArC), 139.4 (ArC), 137.3 (ArC), 132.9 (ArC), 131.5 (ArC), 130.7 (ArC), 129.9 (ArC), 129.3 (ArC), 128.4 (ArC), 127.8 (ArC), 123.4 (ArC), 122.8 (ArC), 117.4 (ArC), 113.2 (ArC), 49.2 (CH-NH), 43.5 (NC**C**H_2), 42.0 (**C**H_2-CO), 21.2 (CH_3); HRMS (ESI): calcd. for $C_{22}H_{23}N_2O_2$ 347.1754. Found: $[MH]^+$, 347.1748 (1.9 ppm error).

4-Methyl-3-phenyl-4,5-dihydro-1*H*-6,10-(azeno)benzo[*d*][1]azacyclododecin-2(3*H*)-one (65)

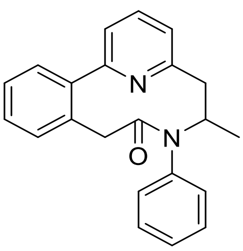

To a stirring solution of 2-(2-(6-(2-(phenylamino)propyl)pyridin-2-yl)phenyl)acetic acid **S77** (33.3 mg, 0.0961 mmol) in chloroform (1.6 mL), was added diisopropylethylamine (0.0309 mL, 0.178 mmol), followed by the addition of T3P (50% solution in ethyl acetate, 91.6 mg, 0.144 mmol). After stirring for 30 mins at r.t., the solution was taken directly to purification

with no work-up. Purification *via* flash column chromatography (SiO_2, 1:1 ethyl acetate:hexane) afforded an inseparable mixture of isomers of the *title compound* **65** as a white solid (quantitative yield) as a 4:1 mixture of rotamers; R_f 0.35 (1:1 ethyl acetate:hexane); m.p. 172−175 °C; ν_{max}/cm^{-1} (thin film) 3058, 3021, 2969, 2930, 2901, 1634, 1586, 1577, 1565, 1492, 1422, 1335, 1314, 1303, 1226, 1128, 1087, 1073, 1059, 1048, 1018, 1003; δ_H (400 MHz, $CDCl_3$; 7.93**–**6.72 (24H, m, ArH, both rotamers), 5.53−5.43 (1H, m, CHN, minor rotamer), 5.41−5.32 (1H, m, CHN, major rotamer), 5.29 (1H, d, *J* = 13.3 Hz, C**H**H-CO, major), 3.45 (1H, d, *J* = 13.3 Hz, CH**H**-CO, major), 3.39 (1H, d, *J* = 14.9 Hz, C**H**H-CO, minor), 3.20 (1H, d, *J* = 14.9 Hz, CH**H**-CO, minor), 3.17−2.98 (2H, m, $NCCH_2$, minor), 2.95−2.93 (2H, m, $NCCH_2$, major), 1.29 (3H, d, *J* = 6.9 Hz, CH_3, major), 1.16 (3H, d, *J* = 6.9 Hz, CH_3, minor); δ_C (101 MHz, $CDCl_3$) 176.4 (CO, minor), 174.8 (CO, major), 156.8 (ArC), 156.6 (ArC), 155.5 (ArC), 140.2 (ArC), 138.2 (ArC), 137.4 (ArC), 137.3 (ArC), 135.7 (ArC), 135.5 (ArC), 133.7 (ArC), 130.5 (ArC), 129.2 (ArC), 129.1 (ArC), 128.8 (ArC), 128.4 (ArC), 127.2 (ArC), 127.0 (ArC), 126.8 (ArC), 126.6 (ArC), 121.1 (ArC), 120.1 (ArC), 119.3 ((ArC) 117.1 (ArC), 53.3 (CHN, major), 49.7 (CHN, minor), 43.7 (CH_2CO, minor), 43.4 (CH_2CO, major), 40.0 (NC**C**H_2, minor), 39.8 (NC**C**H_2, major), 21.2 (CH_3, major), 19.7 (CH_3, minor); HRMS (ESI): calcd. for $C_{22}H_{21}N_2O$ 329.1642. Found: $[MH]^+$, 329.1648 (1.9 ppm error).

Intermediates used to make lactam 65

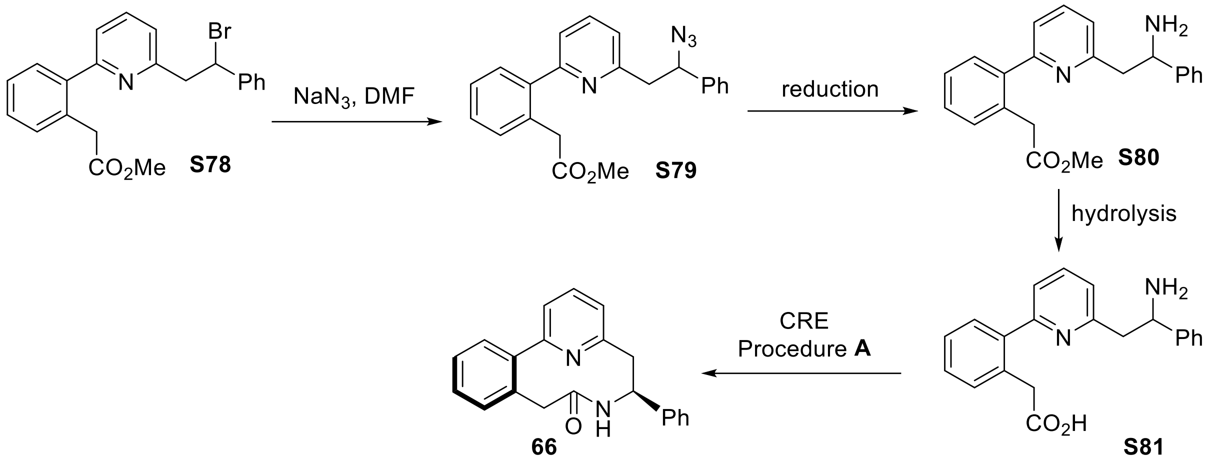

Methyl 2-(2-(6-(2-bromo-2-phenylethyl)pyridin-2-yl)phenyl)acetate (S78)

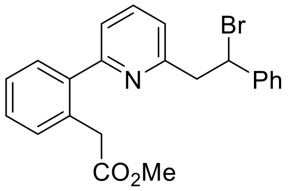

Methyl 2-(2-(6-(2-hydroxy-2-phenylethyl)pyridin-2-yl)phenyl)acetate **S5** (800 mg, 2.31 mmol) and tetrabromomethane (841 mg, 2.54 mmol) were dissolved in CH_2Cl_2 (5 mL) and cooled to 0 °C. Triphenylphosphine (665 mg, 2.54 mmol) was added portionwise and the solution allowed to warm to RT over 30 mins where the solvent was removed *in vacuo*. Purification by flash column chromatography (SiO_2, 50% ethyl acetate in hexane) afforded the *title compound* **S78** as a yellow oil (896 mg, 95%); R_F 0.58 (50% ethyl acetate in hexane); ν_{max}/cm^{-1} (thin film) 3062, 3030, 2949, 1735, 1584; δ_H (400 MHz, $CDCl_3$) 7.71–7.00 (12H, m, 12 × C**H**), 5.56 (1H, dd, *J* = 9.2, 6.9 Hz, C**H**Br), 3.83 (2H, s, C**H₂**CO₂Me), 3.78–3.60 (2H, m, C**H₂**CHBr), 3.59 (3H, s, OC**H₃**); δ_C (101 MHz, $CDCl_3$) 172.4 (**C**O₂Me), 159.3 (**C**N), 157.0 (**C**N), 141.7 (**C**H), 138.6 (**C**), 136.9 (**C**), 132.4 (**C**), 131.6 (**C**H), 129.6 (**C**H), 128.71 (**C**H), 128.65 (**C**H), 128.4 (**C**H), 127.5 (**C**H), 126.4 (**C**H), 123.0 (**C**H), 122.2 (**C**H), 53.8 (O**C**H₃), 51.9 (**C**HBr), 47.6 (**C**H₂CHBr), 39.2 (**C**H₂CO₂Me); HRMS (ESI): calcd. for $C_{22}H_{21}{}^{79}BrNO_2$, 410.0750. Found: $[MH]^+$, 410.0744 (1.6 ppm error).

Methyl 2-(2-(6-(2-azido-2-phenylethyl)pyridin-2-yl)phenyl)acetate (S79)

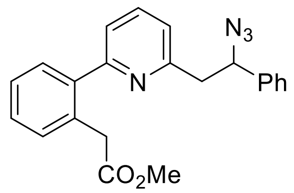

Methyl 2-(2-(6-(2-bromo-2-phenylethyl)pyridin-2-yl)phenyl)acetate **S78** (350 mg, 0.856 mmol) was dissolved in anhydrous DMF (2.8 mL) and NaN_3 (278 mg, 4.28 mmol) added, under a nitrogen atmosphere, and heater to 100 °C for 1 h. The solution was cooled, diluted with ethyl acetate (50 mL), washed with water (3 × 10 mL) and brine (10 mL). The combined organics were dried over $MgSO_4$, filtered and removed *in vacuo*. Purification by flash column chromatography (SiO_2, 50% ethyl acetate in hexanes) afforded the *title compound* **S79** as a yellow oil (225 mg, 71%); R_F 0.60 (50% ethyl acetate in hexane); ν_{max}/cm^{-1} (thin film) 3029, 2949, 2097, 1737, 1583; δ_H (400 MHz, $CDCl_3$) 7.92–6.94 (12H, m, 12 × C**H**), 5.08 (1H, dd, *J* = 8.7, 5.5 Hz, C**H**N_3), 3.83 (2H, s, C**H₂**CO_2Me), 3.58 (3H, s, OC**H₃**), 3.32–3.08 (2H, m, C**H₂**CHN_3); δ_C (101 MHz, $CDCl_3$) 172.3 (**C**O_2Me), 159.2 (**C**N), 156.8 (**C**N), 140.3 (**C**), 139.4 (**C**), 138.7 (**C**), 136.9 (**C**H), 131.6 (**C**H), 129.9 (**C**H), 128.8 (**C**H), 128.6 (**C**H), 128.3 (**C**H), 127.5 (**C**H), 126.9 (**C**H), 122.3 (**C**H), 122.1 (**C**H), 65.7 (**C**N_3), 51.9 (O**C**H_3), 45.0 (**C**H_2CHN_3), 39.3 (**C**H_2CO_2Me); HRMS (ESI): calcd. for $C_{22}H_{21}N_4O_2$, 373.1659. Found: $[MH]^+$, 373.1656 (0.7 ppm error).

Methyl 2-(2-(6-(2-amino-2-phenylethyl)pyridin-2-yl)phenyl)acetate (S80)

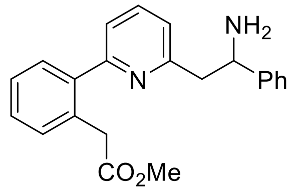

Methyl 2-(2-(6-(2-azido-2-phenylethyl)pyridin-2-yl)phenyl)acetate **S79** (175 mg, 0.470 mmol) was dissolved in methanol (5 mL) and placed under a nitrogen atmosphere. Palladium on carbon (47.0 mg, Pd 10% on carbon), was then added and the vessel backfilled with hydrogen (via balloon) several times, then stirred at RT under a slight positive pressure of hydrogen (balloon) for 4 h. The reaction was then purged with argon, filtered through Celite, washed with methanol where the solvent was removed *in vacuo*. Purification by flash column chromatography (SiO_2, 50% diethyl ether in hexanes → 20% methanol in ethyl acetate) afforded the *title compound* **S80** as a yellow oil (89 mg, 55%); R_F 0.25 (20% methanol in ethyl

acetate); $ν_{max}$/cm^{-1} (thin film) 3027, 2923, 1733, 1570, 1553; $δ_H$ (400 MHz, $CDCl_3$) 7.63 (1H, t, *J* = 8.4 Hz, C**H**), 7.47–7.21 (10H, m, 10 × C**H**), 7.02 (1H, d, *J* = 7.6 Hz, C**H**), 4.50 (1H, dd, *J* = 5.3, 9.2 Hz, C**H**NH_2), 3.89 (1H, d, *J* = 16.8 Hz, C**H**H'CO_2CH_3), 3.83 (1H, d, *J* = 16.8 Hz, CH**H'**CO_2CH_3), 3.57 (3H, s, CO_2C**H$_3$**), 3.18 (1H, dd, *J* = 5.3, 13.7 Hz, C**H**H'CHNH_2), 3.10 (1H, dd, *J* = 9.2, 13.7 Hz, CH**H'**CHNH_2), 2.21 (2H, br s, N**H$_2$**); $δ_C$ (101 MHz, $CDCl_3$) 172.3 (**C**O_2CH_3), 159.0 (**C**N), 158.5 (**C**N), 145.3 (**C**), 140.4 (**C**), 136.8 (**C**H), 132.4 (**C**), 131.5 (**C**H), 129.9 (**C**H), 128.5 (**C**H), 128.4 (2 × C**H**), 127.4 (**C**H), 127.1 (**C**H), 126.5 (2 × C**H**), 122.1 (**C**H), 121.7 (**C**H), 56.0 (**C**HNH_2), 51.9 (CO_2**C**H_3), 47.9 (**C**$H_2CO_2CH_3$), 39.3 (**C**H_2CHNH_2); HRMS (ESI): calcd. for $C_{22}H_{23}N_2O_2$, 347.1754. Found: $[MH]^+$, 347.1754 (0.1 ppm error).

4-Phenyl-4,5-dihydro-1H-6,10-(azeno)benzo[d][1]azacyclododecin-2(3H)-one (66)

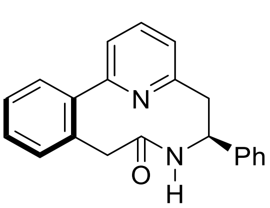

To a stirring solution of ester **S80** (55.0 mg, 0.159 mmol) in THF (0.95 mL) was added $LiOH_{(aq)}$ (0.5 M, 0.95 mL, 0.476 mmol). The resulting solution was stirred at r.t. for 18 h, where upon completion the solvent was removed *in vacuo*. The crude salt was passed through a silica plug and eluted with methanol to remove the excess lithium salts and the crude carboxylic acid **S81** was used without further purification. The crude material was dissolved in chloroform (159 mL), where diisopropylethylamine (50.0 µL, 0.286 mmol) and T3P (50% solution in ethyl acetate, 151 mg, 0.238 mmol) was added sequentially. Upon the addition of T3P, the solution rapidly changed from a colourless to an orange solution. After stirring for 30 mins at r.t., the solution was taken directly to purification with no work-up. Purification by flash column chromatography (SiO_2, ethyl acetate → 10% methanol in ethyl acetate → 20% methanol in ethyl acetate) afforded the *title compound* **66** (as a 4:1 mixture of rotamers) as a yellow oil (36 mg, 52%); R_F 0.58 (20% methanol in ethyl acetate); $ν_{max}$/cm^{-1} (thin film) 3288, 2853, 2925, 1646, 1577, 1452; $δ_H$ (400 MHz, $CDCl_3$) 7.99–6.95 (24H, 12 × C**H**, both rotamers), 5.60 (1H, td, *J* = 11.5, 2.8 Hz, C**H**NH, major rotamer), 5.55–5.41 (1H, m, N**H**, major), 5.33–5.25 (1H, m, N**H**, minor rotamer), 4.92 (1H, d, *J* = 13.3 Hz, C**H**NH, minor), 3.86 (1H, d, *J* = 15.1 Hz, C**H**H'CONH, major), 3.72 (1H, d, *J* = 15.1 Hz, CH**H'**CONH, major), 3.69–2.94 (6H, m, C**H$_2$**CONH, minor and C**H$_2$**CH, both); $δ_C$ (101 MHz, $CDCl_3$) data for major rotamer only: 176.4 (**C**ONH), 156.4 (**C**N),

155.3 (**C**N), 141.2 (**C**), 137.4 (**C**H), 136.2 (**C**), 135.5 (**C**), 129.4 (**C**H), 128.9 (**C**H), 128.7 (**C**H), 127.5 (**C**H), 126.8 (**C**H), 126.4 (**C**H), 126.2 (**C**H), 120.5 (**C**H), 117.4 (**C**H), 57.3 (**C**HNH), 42.4 (**C**H_2CONH), 29.4 (**C**H_2CHNH); HRMS (ESI): calcd. for $C_{21}H_{18}N_2NaO$, 337.1311. Found: $[MNa]^+$, 337.1310 (0.3 ppm error).[1]

***tert*-Butyl 2-((6-chloropyridin-2-yl)methoxy)acetate (S81)**

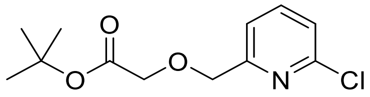

To a stirring solution of sodium hydride (2.20 g, 55.1 mmol, 60% suspension in mineral oil) in dry THF (50 mL), (6-chloropyridin-2-yl)methanol (5.42 mL, 50.0 mmol) was added and stirred at 0 °C for 2 hours under argon. *tert*-Butyl bromoacetate (8.85 mL, 59.9 mmol) was then added to the stirring mixture, and the reaction was warmed to room temperature and stirred for a further 18 hours. The reaction mixture was quenched with sat. aq. ammonium chloride (30 mL) and extracted with ethyl acetate (3 × 50 mL). The combined organic phases were then dried with sodium sulphate, filtered, concentrated under vacuum and purified via flash column chromatography (9:1 hexane:ethyl acetate) to afford the title compound **S81** as a colourless oil (7.37 g, 57%). R_f 0.44 (7:3 hexane:ethyl acetate); ν_{max}/cm^{-1} (thin film) 2979, 2933, 1743, 1586, 1564, 1414, 1368, 1229, 1131, 846, 787, 694; δ_H (400 MHz, $CDCl_3$) 7.65 (1H, dd, *J* = 8.0, 8.0 Hz, Ar**H**), 7.46 (1H, d, *J* = 8.0 Hz, Ar**H**), 7.21 (1H, d, *J* = 8.0 Hz, Ar**H**), 4.68 (2H, s, ArC**H_2**), 4.08 (2H, s, COC**H_2**), 1.46 (9H, s, CC**H_3**); δ_C (101 MHz, $CDCl_3$) 169.1 (**C**O), 159.0 (Ar**C**), 150.5 (Ar**C**), 139.4 (Ar**C**), 123.0 (Ar**C**), 119.8 (Ar**C**), 81.9 (**C**CH_3), 73.2 (Ar**C**H_2), 68.6 (CO**C**H_2), 28.1 (**C**H_3); HRMS (ESI) calcd. for $C_{12}H_{17}{}^{35}ClNO_3$ 258.0891. Found $[MH]^+$ 258.0895 (−0.7 ppm error); calcd. for $C_{12}H_{17}{}^{35}ClNNaO_3$ 280.0711. found $[MNa]^+$ 280.0708 (0.4 ppm error).

***tert*-Butyl 2-((6-vinylpyridin-2-yl)methoxy)acetate (S82)**

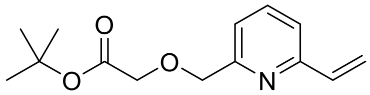

To an argon purged solution of 1:10 ethanol:1,4-dioxane (10 mL), compound **S81** (2.51 g, 9.74 mmol), palladium acetate (82.0 mg, 0.365 mmol), SPhos (403 mg, 0.981 mmol) and potassium phosphate (6.21 g, 29.3 mmol) were added at room temperature. The solution was heated to 100 °C and vinylboronic acid pinacol ester (3.30 mL, 19.5 mmol) was added. The reaction was stirred at 100 °C for 16 h. The reaction was cooled to room temperature, filtered through

Celite and washed with DCM. The filtrate was diluted with H_2O (50 mL) and extracted with DCM (3 × 50 mL). The combined organic phases were then washed with brine (50 mL), dried with sodium sulphate, filtered, concentrated under vacuum and purified via flash column chromatography (9:1 hexane:diethyl ether) to afford the title compound **S82** as a colourless oil (1.91 g, 79%). R_f 0.17 (7:3 hexane:diethyl ether); $ν_{max}/cm^{-1}$ (thin film) 2979, 2933, 1745, 1583, 1572, 1455, 1368, 1226, 1129, 989, 928, 846, 810, 749, 581. $δ_H$ (400 MHz, $CDCl_3$) 7.66 (1H, dd, *J* = 8.0, 8.0 Hz, Ar**H**), 7.39 (1H, d, *J* = 8.0 Hz, Ar**H**), 7.26 (1H, d, *J* = 8.0 Hz, Ar**H**), 6.80 (1H, dd, *J* = 17.5, 11.0 Hz, C**H**CH_2), 6.17 (1H, dd, *J* = 17.5, 1.5 Hz, CHC**H**$_2$), 5.47 (1H, dd, *J* = 11.0, 1.5 Hz, CHC**H**$_2$), 4.73 (2H, s, ArC**H**$_2$), 4.10 (2H, s, COC**H**$_2$), 1.49 (9H, s, C**H**$_3$); $δ_C$ (101 MHz, $CDCl_3$) 169.5 (CO), 157.7 (Ar**C**), 155.3 (Ar**C**), 137.3 (Ar**C**), 137.1 (**C**HCH_2), 120.4 (Ar**C**), 120.0 (ArC), 118.5 (CH**C**H$_2$), 81.9 (**C**CH_3), 74.3 (Ar**C**H$_2$), 68.7 (CO**C**H$_2$), 28.3 (**C**H$_3$); HRMS (ESI) calcd. for $C_{14}H_{19}NNaO_3$ 272.1257. found $[MNa]^+$ 272.1263 (–4.3 ppm error).

***tert*-Butyl 2-((6-(2-(phenylamino)ethyl)pyridin-2-yl)methoxy)acetate (S83)**

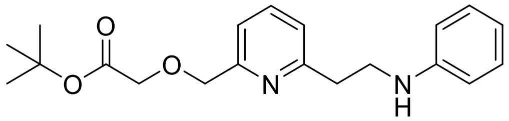

To a stirring solution of alkene **S82** (122 mg, 0.490 mmol) in acetonitrile (1 mL), aniline (0.05 mL, 0.549 mmol) and scandium triflate (26 mg, 52.8 µmol) were added and stirred at 70 °C for 24 hours. The reaction mixture was concentrated under vacuum and purified via flash column chromatography (9:1 hexane:ethyl acetate) to afford the title compound **S83** as a yellow oil (147 mg, 88%). R_f 0.33 (12:7:1 hexane:ethyl acetate:triethylamine). $ν_{max}/cm^{-1}$ (thin film) 3395, 3053, 2978, 2932, 1744, 1602, 1578, 1507, 1458, 1368, 1319, 1229, 1130, 991, 748, 693, 509; $δ_H$ (400 MHz, $CDCl_3$) 7.61 (1H, dd, *J* = 7.5, 7.5 Hz, Ar**H**), 7.36 (1H, d, *J* = 7.5 Hz, Ar**H**), 7.12-7.19 (2H, m, Ar**H**), 7.06 (1H, d, *J* = 7.5 Hz, Ar**H**), 6.65-6.70 (1H, m, Ar**H**), 6.59-6.64 (2H, m, Ar**H**), 4.72 (2H, s, ArC**H**$_2$O), 4.10 (2H, s, COC**H**$_2$), 3.50 (2H, t, *J* = 6.5 Hz, NHC**H**$_2$), 3.06 (2H, t, *J* = 6.5 Hz, NHCH_2C**H**$_2$), 1.48 (9H, s, C**H**$_3$); $δ_C$ (101 MHz, $CDCl_3$) 169.5 (**C**O), 159.2 (Ar**C**), 157.5 (Ar**C**), 148.3 (Ar**C**), 137.3 (Ar**C**), 129.3 (Ar**C**), 122.2 (Ar**C**), 119.4 (Ar**C**), 117.4 (Ar**C**), 113.1 (Ar**C**), 81.9 (**C**CH_3), 74.2 (Ar**C**H$_2$O), 68.6 (CO**C**H$_2$), 43.7 (NH**C**H$_2$), 37.3 (NHCH_2**C**H$_2$), 28.2 (**C**H$_3$); HRMS (ESI) calc. for $C_{20}H_{27}N_2O_3$ 343.2016. found $[MH]^+$ 343.2025 (−2.9 ppm error); calc. for $C_{20}H_{26}N_2NaO_3$ 365.1836. found $[MNa]^+$ 365.1845 (−1.7 ppm error).

Lactam (67)

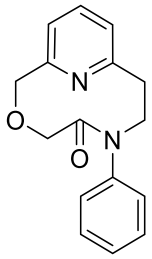

To a stirring solution of ester **S83** (99 mg, 0.289 mmol) in Et_2O (1 mL), 4N HCl in dioxane (1.5 mL, 6.000 mmol) was added and stirred at room temperature for 16 hours. The solvent was removed under vacuum using Et_2O (3 × 2 mL) to form an azeotropic mixture, to help ensure that all of the water from the hydrolysis was removed ahead of the next step. The intermediate amino acid was dissolved in chloroform (2.89 mL) and DIPEA (0.31 mL, 1.780 mmol) was added followed by T3P 50% w/v in ethyl acetate (289 mg, 0.454 mmol) and stirred at room temperature for 16 h under argon. The reaction mixture was diluted with H_2O (20 mL) and extracted with DCM (3 × 20 mL). The combined organic phases were then washed with H_2O (50 mL), dried with sodium sulphate, filtered, concentrated under vacuum and purified via flash column chromatography (18:1:1 hexane:ethyl acetate:triethylamine) to afford the title compound **67** as a colourless film (78 mg, 100%). R_f 0.16 (18:1:1 hexane:ethyl acetate:triethylamine); $\mathbf{v_{max}}/cm^{-1}$ (thin film) 3061, 2926, 2854, 1768, 1595, 1495, 1459, 1385, 1357, 1310, 1283, 1206, 1164, 1076, 989, 905, 809, 757, 700; δ_H (400 MHz, $CDCl_3$) 7.65 (1H, dd, *J* = 7.5, 7.5 Hz, Ar**H**), 7.49-7.59 (2H, m, Ar**H**), 7.42 (2H, dd, *J* = 7.5, 7.5 Hz, Ar**H**), 7.32 (1H, dd, *J* = 7.5, 7.5 Hz, Ar**H**), 7.19 (1H, d, *J* = 7.5 Hz, Ar**H**), 7.14 (1H, d, *J* = 7.5 Hz, Ar**H**), 4.72 (1H, d, *J* = 4.5 Hz, OC**H**HAr), 4.58-4.73 (1H, m, NC**H**HC), 4.38 (1H, d, *J* = 4.5 Hz, OCH**H**Ar), 4.08-4.24 (1H, m, COC**H**H), 3.98 (1H, d, *J* = 13.5 Hz, COCH**H**), 3.24-3.41 (2H, m, $NC\mathbf{H}_2CH_2$, $NCH_2C\mathbf{H}_2$), 2.77-2.86 (1H, m, $NCH_2C\mathbf{H}_2$); δ_C (101 MHz, $CDCl_3$) 173.5 (**C**O), 158.7 (Ar**C**), 156.6 (Ar**C**), 143.2 (Ar**C**), 138.6 (Ar**C**), 129.7 (Ar**C**), 127.3 (Ar**C**), 126.1 (Ar**C**), 122.8 (Ar**C**), 120.9 (Ar**C**), 76.1 (O**C**H_2Ar), 71.5 (CO**C**H_2), 50.2 (N**C**H_2CH_2), 35.2 (NCH_2**C**H_2); HRMS (ESI) calcd. for $C_{16}H_{17}N_2O_2$ 269.1285. found $[MH]^+$ 269.1279 (+0.60 ppm error); calcd. for $C_{16}H_{16}N_2NaO_2$ 291.1104. found $[MNa]^+$ 291.1096 (+2.0 ppm error); calcd. for $C_{16}H_{16}KN_2O_2$ 307.0843. found $[MK]^+$ 307.0842 (+0.9 ppm error).

Methyl 2-((ethyl (3-(ethyl amino) propyl) amino) methyl) benzoate (S84)

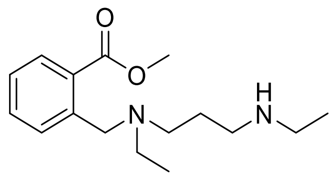

To a stirring solution of N,N'-diethyl-1,3-propanediamine (0.260 g, 2.00 mmol) in acetonitrile (5.00 mL) under argon, potassium carbonate (0.690 g, 5.00 mmol) was added. Methyl 2-bromomethylbenzoate (0.229 g, 1.00 mmol) was then added and the reaction was heated at reflux at 90 °C for 3 hours under argon. The reaction was cooled to room temperature before it was filtered through Celite, washed with acetonitrile, concentrated under vacuum and purified via flash column chromatography (17:2:1 ethyl acetate:methanol:triethylamine) to afford the title compound **S84** as a colourless oil (114 mg, 41%); R_f = 0.33 (17:2:1 ethyl acetate:methanol:triethylamine); ν_{max}/cm^{-1} (thin film) 2968, 2807, 1722, 1434, 1371, 1262, 1190, 1127, 1082, 1045, 967, 737; δ_H (400 MHz, $CDCl_3$) 7.72 (1H, d, *J* = 7.7 Hz, Ar**H**), 7.54 (1H, d, *J* = 7.7 Hz, Ar**H**), 7.41 (1H, td, *J* = 7.7, 1.4 Hz, Ar**H**), 7.26 (1H, t, *J* = 7.7 Hz, Ar**H**), 3.86 (3H, s, OC**H**$_3$), 3.82 (2H, s, ArC**H**$_2$N), 2.60−2.54 (4H, m, 2 × NHC**H**$_2$CH$_3$), 2.53−2.48 (2H, m, NC**H**$_2$CH$_2$), 2.44 (2H, t, *J* = 6.9 Hz, NHC**H**$_2$CH$_2$), 1.61 (2H, q, *J* = 6.9 Hz, CH$_2$C**H**$_2$CH$_2$), 1.06 (3H, t, *J* = 7.2 Hz, NCH$_2$C**H**$_3$), 0.99 (3H, t, *J* = 7.1 Hz, NHCH$_2$C**H**$_3$); δ_C (101 MHz, $CDCl_3$) 169.9 (**C**O), 141.7 (Ar**C**), 131.3 (Ar**C**H), 131.0 (Ar**C**), 129.9 (Ar**C**H), 129.8 (Ar**C**H), 126.6 (Ar**C**H), 56.6 (Ar**C**H$_2$N), 52.0 (COO**C**H$_3$), 51.3 (CH$_2$**C**H$_2$NH), 48.3 (CH$_3$**C**H$_2$N), 47.4 (CH$_2$**C**H$_2$N), 44.2 (CH$_3$**C**H$_2$NH), 27.2 (CH$_2$C**H**$_2$CH$_2$), 15.3 (**C**H$_3$CH$_2$N), 11.5 (**C**H$_3$CH$_2$NH); HRMS (ESI): calcd. for $C_{16}H_{27}N_2O_2$ 279.2072. Found [MH]$^+$ 279.2065 (−2.5 ppm error).

2,4-Diethyl-2,3,4,5,6,7-hexahydro-1H-2,4-benzodiazonin-1-one (68)

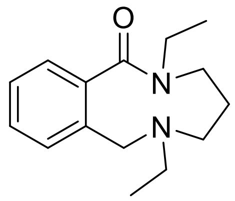

Methyl 2-((ethyl(3-(ethylamino) propyl) amino) methyl) benzoate (**S84**) (0.114 g, 0.411 mmol) and lithium hydroxide (0.600 mmol, 1.20 mL of 0.500 M aq. LiOH) were stirred at room temperature in tetrahydrofuran (0.600 mL) for 24 hours, the solvent was then removed under

vacuum. The intermediate lithium 2-((ethyl(3-(ethylamino) propyl) amino) methyl) benzoate was dissolved in chloroform (9.00 mL) before DIPEA (0.132 mL, 0.760 mmol) was added dropwise and the mixture stirred until a homogeneous solution was observed (30 minutes). Propane phosphonic acid anhydride (T3P) in ethyl acetate 50% w/v (0.412 g, 0.620 mmol) was then added dropwise and the reaction was stirred overnight at room temperature. The solvent was then removed under vacuum and the reaction mixture purified via flash column chromatography (13:6:1 hexane:diethyl ether:triethylamine) to afford the title compound **68** as a dark orange oil (54.8 mg, 54%); R_f = 0.24 (13:6:1 hexane:diethyl ether:triethylamine); ν_{max}/cm^{-1} (thin film) 2965, 2930, 1631, 1420, 1368, 1170, 1061, 959, 848, 765, 730; δ_H (400 MHz, $CDCl_3$) 7.25–7.12 (4H, m, ArC**H**), 4.13–4.04 (1H, m, CONC**H**$_a$H$_b$CH$_3$), 4.00 (1H, d, *J* = 13.6 Hz, ArC**H**$_a$H$_b$N), 3.34 (1H, d, *J* = 13.6 Hz, ArCH$_a$**H**$_b$N), 3.27–3.12 (2H, m, NC**H**$_2$CH$_2$), 2.95–2.85 (1H, m, CONCH$_a$**H**$_b$CH$_3$), 2.67–2.54 (3H, m, NC**H**$_2$CH$_3$ & NC**H**$_a$H$_b$CH$_3$), 2.43 (1H, ddd, *J* = 14.4 Hz, 12.3 Hz, 2.5 Hz, NCH$_a$**H**$_b$CH$_3$), 1.80–1.69 (1H, m, CH$_2$C**H**$_a$H$_b$CH$_2$), 1.21 (3H, t, *J* = 8.0 Hz, CONCH$_2$C**H**$_3$), 1.07 (3H, t, *J* = 8.0 Hz, NCH$_2$C**H**$_3$), 0.99–0.92 (1H, m, CH$_2$CH$_a$**H**$_b$CH$_2$); δ_C (101 MHz, $CDCl_3$) 172.9 (**C**O), 138.4 (Ar**C**), 138.3 (Ar**C**), 129.2 (Ar**C**H), 128.1 (Ar**C**H), 127.3 (Ar**C**H), 126.4 (Ar**C**H), 57.8 (Ar**C**H$_2$N), 52.4 (N**C**H$_2$CH$_2$), 49.4 (N**C**H$_2$CH$_3$), 48.4 (N**C**H$_2$CH$_2$), 38.5 (CON**C**H$_2$CH$_3$), 24.3 (CH$_2$**C**H$_2$CH$_2$), 13.1 (CONCH$_2$**C**H$_3$), 12.1 (NCH$_2$**C**H$_3$); HRMS (ESI): calcd. for $C_{15}H_{23}N_2O$. Found 247.1810 $[MH]^+$, 247.1800 (–4.06 ppm error).

Methyl 2-(((3-mercaptopropyl)thio)methyl)benzoate (S85)

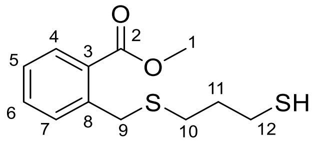

K_2CO_3 (840 mg, 6.12 mmol) was added to a pale-yellow solution of 1,3-propanedithiol (615 µL, 6.12 mmol) and methyl 2-bromomethylbenzoate (935 mg, 4.20 mmol) in anhydrous DMF (14 mL) at RT. The resulting milky–pink suspension was stirred at RT for 18 h, after which the reaction was deemed to have gone to completion by TLC, with a colour change to milky white noted. The reaction mixture was then quenched by addition of H_2O (30 mL) and resulting solution poured into a separating funnel. The aqueous layer was extracted with EtOAc (3 × 20 mL), before the combined organic phases were washed sequentially with 1 M $HCl_{(aq)}$ (2 × 20 mL) and brine (3 × 20 mL). The resulting organic layer was dried over $MgSO_4$, filtered and concentrated under reduced pressure to yield pale yellow oil (1.30 g). The crude product was

purified by flash column chromatography (SiO_2, 50 mm column, eluent: EtOAc:*n*–hexane, 10:90) to afford the title compound **S85** as a pale yellow oil (667 mg, 62%). R_f = 0.34 (20:80 EtOAc:*n*–hexane); IR (thin film) ν_{max} / cm^{-1}: 2943w (C–H alkyl), 2842w (C–H alkyl), 2574w (S–H thiol), 1716vs (C=O aryl ester), 1599w (CC aromatic), 1576 (CC aromatic), 1488w (CC aromatic), 1433m, 1292w, 1260vs, 1189w, 1163w, 1119m, 1076m, 1046w, 964w, 889w, 839w 801w, 767m, 714s, 663w, 580w, 476w; δ_H (400 MHz; $CDCl_3$) 7.91 (1H, dd, *J* 7.7, 1.5 C(4)H), 7.44 (1H, td, *J* 7.7, 1.5, C(7)H), 7.35 – 7.24 (2H, m, C(5+6)H), 4.11 (2H, s, C(9)H_2), 3.91 (3H, s, C(1)H_3), 2.63 – 2.50 (4H, m, C(10+12)H_2), 1.91 – 1.77 (2H, m, C(11)H_2)1.30 (1H, t, *J* 8.1, SH); δ_C (101 MHz; $CDCl_3$) 167.7 (C, C2) 140.5 (C, C8), 131.8 (CH, C7), 131.1 (CH, C4), 131.0 (CH, C6), 129.4 (C, C3), 127.0 (CH, C5), 52.1 (CH_3, C1), 34.4 (CH_2, C9), 33.0 (CH_2, C11), 29.9 (CH_2, C12), 23.3 (CH_2, C10); HRMS (ESI^+): m/z calc. for $C_{12}H_{16}NaO_3S_2$: 279.0484, found: 279.0481 $[MNa]^+$.

2-(((3-Mercaptopropyl)thio)methyl)benzoic acid (S86)

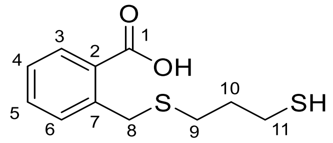

$NaOH_{(aq)}$ (4.0 M, 13.5 mL, degassed for 10 min) was added dropwise over a period of 2 min to a pale-yellow solution of methyl 2-(((3-mercaptopropyl)thio)methyl)benzoate **S85** (667 mg, 2.60 mmol) in anhydrous MeOH (13.5 mL, degassed for 15 min) at RT under Ar. A colour change to milky white suspension was noted immediately during dropwise addition. The resulting suspension was then heated to 40 °C, whereupon a colour change to clear colorless solution observed. After a total of 16 h, the reaction was deemed to have gone to completion by TLC analysis (complete consumption of methyl 2-(((3-mercaptopropyl)thio)methyl)benzoate was noted). The resulting pale yellow mixture was allowed to cool to RT and then acidified to pH 2.0 with 1 M $HCl_{(aq)}$ (30 mL). The resulting acidified solution was poured into separating funnel containing CH_2Cl_2 (20 mL). The aqueous layer was extracted with CH_2Cl_2 (3 × 20 mL), before combined organic phases were dried over Na_2SO_4, filtered and concentrated under reduced pressure to afford the title compound **S86** as a white solid (666 mg, 98%). R_f = 0.45 (15:85 MeOH:CH_2Cl_2); Melting Point: 72–74 °C (from *n*–hexane:CH_2Cl_2, 9:1); IR (solid state) ν_{max} / cm^{-1}: 2955vbrm (O–H alcohol, O–H carboxylic acid), 2916vbrm (C–H alkyl, O–H carboxylic acid), 2641br (C–H alkyl, O–H carboxylic acid), 1674vs (C=O aryl carboxylic acid), 1596w (CC aromatic), 1576m (CC aromatic), 1489w, 1448w,

1439w, 1425w, 1404w, 1298m, 1270s, 1198w, 1165w, 1139w, 1114w, 1080m, 1052w, 1027w, 968w, 906m, 873w, 842w, 808w, 763m, 708s, 677m, 658s, 580w, 537m, 497w; δ_H (400 MHz; $CDCl_3$) 8.08 (1H, dd, *J* 7.7, 1.5 C(3)H), 7.51 (1H, td, *J* 7.7, 1.5, C(6)H), 7.43 – 7.30 (2H, m, C(4+5)H), 4.18 (2H, s, C(8)H_2), 2.67 – 2.54 (4H, m, C(9+11)H_2), 1.93 – 1.81 (2H, m, C(10)H_2), 1.32 (1H, t, *J* 8.1, SH); δ_C (101 MHz; $CDCl_3$) 173.1 (C, C1) 141.8 (C, C7), 133.0 (CH, C6), 132.4 (CH, C3), 131.4 (CH, C5), 128.2 (C, C2), 127.4 (CH, C4), 34.6 (CH_2, C8), 33.2 (CH_2, C10), 30.2 (CH_2, C11), 23.5 (CH_2, C9); HRMS (ESI^+): m/z calc. for $C_{11}H_{14}NaO_2S_2$: 265.0327, found: 265.0329 $[MNa]^+$.

Synthesis of 4,5-dihydro-3H-benzo[*g*][1,5]dithionin-1(7*H*)-one (69) and 8,9,19,20-tetrahydro-7*H*,18*H*-dibenzo[*g,p*][1,5,10,14]tetrathiacyclooctadecine-5,16(11*H*,22*H*)-dione (S87)

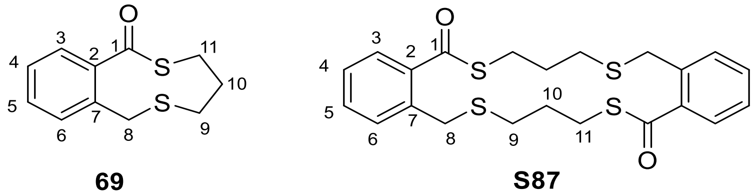

EDC.HCl (194 mg, 0.958 mmol) was added to solution of 2-(((3-mercaptopropyl)thio)methyl)benzoic acid **S86** (155 mg, 0.639 mmol), HOBt (116 mg, 0.766 mmol) and anhydrous DIPEA (280 µL, 1.60 mmol) in anhydrous DMF (12 mL). An immediate colour change colour to pale yellow observed. After total of 16 h of stirring at RT, the reaction deemed to have to completion by TLC analysis with colour change to fluorescent yellow noted. The resulting mixture was diluted with EtOAc (30 mL). The milky/white suspension was transferred to separating funnel and the organic phase was washed sequentially with 1M $HCl_{(aq)}$ (3 × 10 mL), sat. $NaHCO_{3(aq)}$ (3 × 20 mL), H_2O (3 × 20 mL) and sat. brine (3 × 20 mL). The organic layer was dried over Na_2SO_4, filtered and concentrated under reduced pressure to yield a pale-yellow oil (253 mg). The crude product was purified by flash column chromatography (SiO_2, 30 mm column, eluent: EtOAc:*n*–hexane, 10:90) to afford the title compound **69** as a clear colourless oil (91.0 mg, 65%) and a small amount of macrocyclic head-to-tail dimer **S87** as a white solid (20.5 mg, 14%).

Data for **69:** R_f = 0.42 (20:80 EtOAc:*n*–hexane); IR (solid state) ν_{max} / cm^{-1}: 3068w (C–H alkyl), 2921w (C–H alkyl), 2857w (C–H alkyl), 1726s (C=O aryl thioester), 1604w (CC aromatic), 1586w (CC aromatic), 1489w, 1452m, 1378w, 1237m, 1130m, 1008m, 938w, 874w, 764w,

716w; δ_H (400 MHz; $CDCl_3$) 7.44 (1H, td, *J* = 7.6, 1.4 Hz, C(3)H), 7.33 (1H, td, *J* = 7.6, 1.4, C(5)H), 7.22 (1H, dd, *J* 7.6, 1.4, C(4)H), 7.17 (1H, dd, *J* 7.6, 1.4, C(6)H), 3.73 (2H, s, br, C(8)H_2), 3.11 – 3.03 (2H, m, C(11)H_2), 2.92 (2H, s br, C(9)H_2), 1.90 – 1.80 (2H, m, C(10)H_2); δ_C (101 MHz; $CDCl_3$) 203.0 (C, C1) 137.6 (C, C7), 136.7 (C, C2), 131.0 (CH, C3), 129.9 (CH, C4), 127.9 (CH, C5), 125.3 (CH, C6), 37.0 (CH_2, C8) 34.2 (CH_2, C11), 33.3 (CH_2, C9), 30.7 (CH_2, C10); HRMS (ESI^+): m/z calc. for $C_{11}H_{12}NaO_2S_2$: 247.0222, found: 247.0222 $[MNa]^+$

X-ray crystallographic data for this compound can be accessed via www.ccdc.cam.ac.uk/data_request/cif (CCDC 2236987).

Data for macrocylic dimer **S87:** R_f = 0.34 (20:80 EtOAc:*n*–hexane); IR (solid state) ν_{max} / cm^{-1}: 2923w (C–H alkyl), 2854w (C–H alkyl), 1727m (C=O aryl thioester), 1612w (CC aromatic), 1593w (CC aromatic), 1493w, 1457w, 1413w, 1380w, 1336w, 1288w, 1230w, 1186m, 1157s, 1087w, 1059w, 1010m, 961w, 933w, 887w, 779w, 754w, 702m, 613w, 570w, 481w; δ_H (400 MHz; $CDCl_3$) 7.65 (2H, dd, *J* 7.6, 1.5, C(3)H), 7.47 (2H, dd, *J* = 7.6, 1.5 Hz, C(6)H), 7.44 (2H, td, *J* = 7.6, 1.5 Hz, C(5)H),7.30 (2H, td, *J* 7.6, 1.5, C(4)H), 4.04 (4H, s, C(8)H_2), 3.10 (4H, t, *J* 7.1, C(11)H_2), 2.54 (4H, t, *J* 7.2, C(9)H_2), 2.06 – 1.91 (4H, m, C(10)H_2); δ_C (101 MHz; $CDCl_3$) 195.0 (C, C1) 138.6 (C, C7), 136.8 (C, C2), 131.8 (CH, C5), 131.5 (CH, C3), 128.1 (CH, C6), 127.2 (CH, C4), 32.4 (CH_2, C8), 31.1 (CH_2, C11) 29.2 (CH_2, C10), 29.1 (CH_2, C9); HRMS (ESI^+): m/z calc. for $C_{22}H_{24}NaO_2S_4$: 471.0551, found: 471.0558 $[MNa]^+$

X-ray crystallographic data for compound **S87** can be accessed via www.ccdc.cam.ac.uk/data_request/cif (CCDC 2236988).

Methyl 2-((methyl(2-(phenylamino) ethyl) amino) methyl) quinoline-3-carboxylate (S88)

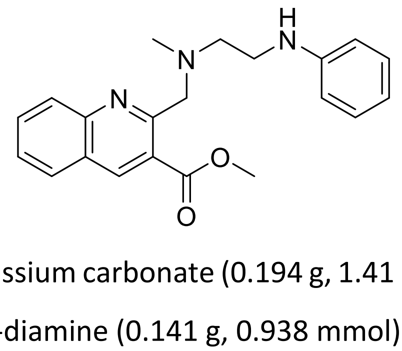

To a stirring suspension of potassium carbonate (0.194 g, 1.41 mmol) in acetonitrile (4.70 mL), N-methyl-N-phenylethane-1,2-diamine (0.141 g, 0.938 mmol) was added followed by methyl 2-(bromomethyl) quinoline-3-carboxylate **S55** (0.222 g, 0.938 mmol). The reaction mixture was stirred at room temperature for 5 hours under argon before filtering through Celite

washing with DCM, concentrating under vacuum and purifying by flash column chromatography (19:1 toluene:methanol) to afford the title compound **S88** as a yellow oil (0.113 g, 35%) R_f = 0.43 streek (ethyl acetate); ν_{max}/cm^{-1} (thin film) 3399, 2955, 2850, 1724, 1603, 1507, 1247, 1065, 751, 697; δ_H (400 MHz, $CDCl_3$) 8.46 (1H, s, Ar**H**), 8.10 (1H, d, *J* = 8.3 Hz, Ar**H**), 7.82 (1H, d, *J* = 8.3 Hz, Ar**H**), 7.76-7.75 (1H, m, Ar**H**), 7.56−7.53 (1H, m, Ar**H**), 7.17−7.13 (2H, m, 2 × Ar**H**), 6.67−6.62 (3H, m, 3 × Ar**H**), 4.35 (1H, s, N**H**), 4.15 (2H, s, ArC**H**$_2$), 4.01 (3H, s, OC**H**$_3$), 3.06 (2H, t, *J* = 5.6 Hz, NC**H**$_2$CH$_2$), 2.65 (2H, t, *J* = 5.6 Hz, NC**H**$_2$CH$_2$), 2.27 (3H, s, C**H**$_3$N); δ_C (101 MHz, $CDCl_3$) 169.0 (**C**O), 158.5 (Ar**C**), 148.7 (Ar**C**), 147.7 (Ar**C**), 138.4 (Ar**C**H), 131.3 (Ar**C**H), 129.2 (Ar**C**H), 129.1 (Ar**C**H), 128.4 (Ar**C**H), 127.2 (Ar**C**H), 126.4 (Ar**C**), 126.2 (Ar**C**), 116.9 (Ar**C**H), 112.7 (Ar**C**H), 64.1 (Ar**C**H$_2$N), 55.7 (N**C**H$_2$), 52.9 (N**C**H$_3$), 41.6 (O**C**H$_3$), 40.7 (NH**C**H$_2$); HRMS (ESI) calcd. for $C_{21}H_{24}N_3O_2$ 350.1869. Found $[MH]^+$ 350.1854 (−4.28 ppm error).

5-Methyl-2-phenyl-3,4,5,4-tetrahydro- [1,4] diazocino[6,7-b] quinolin-1(2H)-one (70)

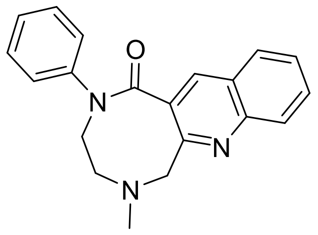

To a stirring solution of methyl 2-((methyl (2-(phenylamino) ethyl) amino) methyl) quinoline-3-carboxylate (**S88**) (0.113 g, 0.324 mmol) in tetrahydrofuran (0.842 mL), aqueous lithium hydroxide (0.5 M) (0.842 mL, 0.421 mmol) was added and heated for at 50 °C for 3 hours. The solvent was removed under vacuum using dichloromethane (5 × 50.0 mL) to form an azeotropic mixture, to help ensure that all of the water from the hydrolysis was removed ahead of the next step. The intermediate lithium (2-(((3-carboxylatoquinolin-2-yl) methyl) (methyl) amino) ethyl) (phenyl) amide was dissolved in chloroform (3.24 mL) and DIPEA (0.105 mL, 0.599 mmol) was added followed by T3P 50% w/v in ethyl acetate (0.309 g, 0.487 mmol) and stirred at room temperature for 24 hours under argon. The reaction mixture was then transferred to a separating funnel, brine (50.0 mL) was added and extracted with dichloromethane (2 × 100 mL). The combined organic phases were then dried with sodium sulphate, filtered, concentrated under vacuum and purified via flash column chromatography (10:9:1 hexane:ethyl acetate:triethylamine) to afford the title compound **70** (0.058 g, 56%) R_f

= 0.29 (10:9:1 hexane:ethyl acetate:triethylamine); ν_{max}/cm^{-1} (thin film) 2944, 2802, 2241, 1642, 1491, 1397, 1192, 1062, 921, 792, 700, 728; δ_H (400 MHz, $CDCl_3$); 8.39 (1H, s, Ar**H**), 8.03 (1H, d, *J* = 8.4 Hz, Ar**H**), 7.82 (1H, d, *J* = 8.1 Hz, Ar**H**), 7.72 (1H, t, *J* = 7.1 Hz, Ar**H**), 7.51 (1H, t, *J* = 7.6 Hz, Ar**H**), 7.46-7.40 (4H, m, 4 × Ar**H**), 7.31 (1H, t, *J* = 7.1 Hz, Ar**H**), 4.32 (1H, d, *J* = 16.1 Hz, ArC**H**$_a$H$_b$N), 4.08 (1H, d, *J* = 16.1 Hz, ArCH$_a$**H**$_b$N), 3.76 (1H, t, *J* = 14.0 Hz, ArC**H**$_a$H$_b$N), 3.51 (1H, d, *J* = 14.0 Hz, ArCH$_a$**H**$_b$N), 2.85 (1H, td, *J* = 12.6 Hz, *J* = 3.4 Hz, CH$_a$**H**$_b$NCH$_3$), 2.65 (3H, s, NC**H**$_3$), 2.53 (1H, d, *J* = 12.6 Hz, CH$_a$**H**$_b$NCH$_3$); δ_C (101 MHz, $CDCl_3$) 170.7 (**C**O) 157.2 (Ar**C**), 148.0 (Ar**C**), 141.8 (Ar**C**), 138.6 (Ar**C**H), 130.9 (Ar**C**H), 129.5 (Ar**C**H), 129.4 (Ar**C**), 128.7 (Ar**C**H), 128.1 (Ar**C**H), 127.6 (Ar**C**H), 127.3 (Ar**C**H), 126.9 (Ar**C**H), 126.7 (Ar**C**), 63.4 (Ar**C**H$_2$N), 55.9 (**C**H$_2$NCH$_3$), 50.9 (**C**H$_2$NAr), 47.2 (N**C**H$_3$); HRMS (ESI) calcd. for $C_{20}H_{20}N_3O$ 318.1606. Found $[MH]^+$ 318.1609 (0.943 ppm error).

2-((Phenyl(2-(phenylamino)ethyl)amino)methyl)benzoic acid (S89)

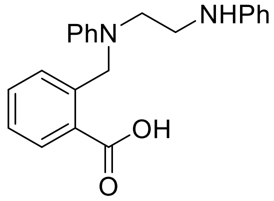

To a stirring solution of 2-formylbenzoic acid (1.00 g, 6.69 mmol) in DCM (60 mL), N,N'-diphenylethylenediamine (2.119 g, 9.98 mmol) and conc. Sulfuric acid (2 drops) were added and stirred at room temperature for 7 hours. Sodium triacetoxyborohydride (2.110 g, 10.0 mmol) was added to the mixture and stirred at room temperature for 18 hours. The reaction mixture was concentrated under vacuum and purified via flash column chromatography (1:1 hexane:diethyl ether) to afford the title compound as a white solid (1.332 g, 57%). R_f 0.28 (1:1 hexane:diethyl ether). M.P. 140 – 143 °C; ν_{max} (thin film)/cm^{-1} 3432, 2946, 2876, 2650, 1691, 1596, 1573, 1505, 1304, 1270, 1252, 1221, 1123, 989, 928, 742, 735, 691, 511; δ_H (400 MHz, $CDCl_3$) 8.15 (1H, dd, *J* = 7.5, 1.5 Hz, Ar**H**), 7.42 (1H, td, *J* = 7.5, 1.5 Hz, Ar**H**), 7.36 (1H, td, *J* = 7.5, 1.5 Hz, Ar**H**), 7.25 – 7.19 (3H, m, Ar**H**), 7.18 – 7.12 (3H, m, Ar**H**), 6.90 – 6.83 (3H, m, Ar**H**), 6.71 (1H, td, *J* = 7.5, 1.0 Hz, Ar**H**), 6.57 (2H, d, *J* = 8.0 Hz, Ar**H**), 4.84 (2H, s, ArC**H**$_2$), 3.60 (2H, t, *J* = 6.5 Hz, NHPhCH$_2$C**H**$_2$), 3.40 (2H, t, *J* = 6.5 Hz, NHPhC**H**$_2$); δ_C (101 MHz, $CDCl_3$) 171.5 (**C**OOH), 147.5 (Ar**C**), 147.4 (Ar**C**), 139.3 (Ar**C**), 133.1 (Ar**C**), 132.8 (Ar**C**), 129.7 (Ar**C**), 129.5 (Ar**C**), 128.7 (Ar**C**), 127.7 (Ar**C**), 120.1 (Ar**C**), 118.1 (Ar**C**), 115.7 (Ar**C**), 113.3 (Ar**C**), 56.2 (Ar**C**H$_2$), 51.6

(NHPhCH$_2$**C**H$_2$), 41.5 (NHPhCH$_2$**C**H$_2$). HRMS (ESI$^+$) $C_{22}H_{23}N_2O_2$ (MH$^+$) theoretical 347.1754; measured 347.1753 (+1.0 ppm error); $C_{22}H_{22}N_2NaO_2$ (MNa$^+$) theoretical 369.1573; measured 369.1575 (−0.6 ppm error); $C_{22}H_{22}KN_2O_2$ (MK$^+$) theoretical 385.1313; measured 385.1308 (−2.0 ppm error).

2,5-Diphenyl-3,4,5,6-tetrahydrobenzo[*f*][1,4]diazocin-1(2*H*)-one (71)

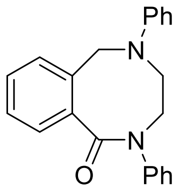

To a stirring solution of 2-((phenyl(2-(phenylamino)ethyl)amino)methyl)benzoic acid **S89** (95 mg, 0.274 mmol) in chloroform (2.7 mL), was added diisopropylethylamine (88.4 µL, 0.508 mmol), followed by the addition of T3P (50% solution in ethyl acetate, 262 mg, 0.411 mmol). After stirring for 1 h at r.t., the solution was diluted with DCM (25 mL), washed with water (20 mL), dried ober $MgSO_4$ and concentrated. Purification *via* flash column chromatography (SiO_2, 5:1 → 2:1 hexane:ethyl acetate) afforded the *title compound* **71** as a colourless oil (90 mg, 99%); R_f = 0.15 (2:1 hexane:ethyl acetate); ν_{max}/cm^{-1} (thin film) 1638s, 1596m, 1493m, 1395s, 1219w, 732m; δ_H (400 MHz, CDCl$_3$) 7.60 (1H, d, *J* = 6.9 Hz, Ar**H**), 7.45−7.17 (10H, m, Ar**H**), 6.91 (2H, d, *J* = 8.2 Hz, Ar**H**), 6.85 (1H, app t, *J* = 7.3, Ar**H**), 4.75 (1H, d, *J* = 17.0, C**H**H'Ar), 4.66 (1H, d, *J* = 17.0, CH**H'**Ar), 4.10–4.02 (1H, m, NCH$_2$), 3.77–3.59 (2H, m, NCH$_2$), 3.27–3.21 (1H, m, NCH$_2$); δ_c (101 MHz, CDCl$_3$) 171.6 (**C**O), 150.6 (Ar**C**), 141.7 (Ar**C**), 137.8 (Ar**C**), 134.7 (Ar**C**), 130.4 (Ar**C**H), 130.3 (Ar**C**H), 129.5 (Ar**C**H), 129.4 (Ar**C**H), 127.9 (Ar**C**H) 127.8 (Ar**C**H), 127.7 (Ar**C**H), 127.2 (Ar**C**H), 119.6 (Ar**C**H), 116.0 (Ar**C**H), 56.5 (**C**H$_2$N), 51.6 (**C**H$_2$N), 48.8 (**C**H$_2$N); HRMS (ESI): calcd for $C_{22}H_{21}N_2O$ 329.1648 & $C_{22}H_{20}N_2NaO$. 351.1468. Found [MH]$^+$, 329.1648 (−3.29 ppm error) & [MNa]$^+$, 351.1468.

Benzyl 2-(3-(*tert*-butoxy)-3-oxopropyl) pyrrolidine-1-carboxylate (S90)

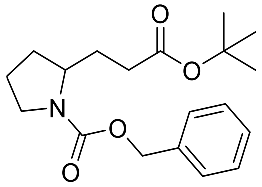

To a 250 mL round bottom flask containing ((benzyloxy)carbonyl) proline (2.89 g, 12.0 mmol), Ir[df(CF_3)ppy]$_2$(dtbby)PF_6 (0.135 g, 0.120 mmol) and potassium phosphate (2.51 g, 14.4 mmol) was added to DMF (30.0 mL) and degassed for 10 minutes using argon. Separately *tert*-butyl acrylate (3.00 mL) was degassed for 10 minutes using argon. A portion of the degassed *tert*-butyl acrylate (1.54 g, 1.76 mL, 12.0 mmol) was then added to the 250 mL round bottom flask topped with a septum wrapped with parafilm several times. The reaction mixture was irradiated with blue LED light for 63 hours whilst stirring (setup as shown below). The reaction was quenched in saturated $NaHCO_3$ aqueous solution (150 mL) and extracted in Et_2O (3 × 100.0 mL). The organic phases were collected and dried with sodium sulphate and the solvent removed under vacuum. The reaction mixture was purified via flash column chromatography (8:2 hexane:ethyl acetate) to afford the title compound **S90** as a pale yellow oil (2.30 g, 57%); R_f = 0.23 (8:2 hexane:ethyl acetate); ν_{max}/cm^{-1} (thin film) 2930, 1700, 1279, 1159; δ_H (400 MHz, $CDCl_3$) 7-39−7.26 (5H, m, 5 × Ar**H**), 5.12 (2H, s, OC**H**$_2$Ar), 3.88 (1H, s, C**H**N), 3.49−3.34 (2H, m, C**H**$_2$N), 2.88−2.14 (2H, m, CH_2C**H**$_2$CO), 2.02−1.77 (4H, m, 2× C**H**$_2$), 1.69−1.60 (2H, m, C**H**$_2$), 1.41 (9H, s, (C**H**$_3$)$_3$C); δ_C (101 MHz, $CDCl_3$) 172.8 (**C**O), 155.2 (Ar**C**H), 137.1 (Ar**C**), 128.5 (ArCH), 128.0 (Ar**C**H), 80.3 (O**C**CH$_3$)$_3$, 66.7 (OC**H**$_2$Ar), 57.2 (N**C**H), 46.5 (**C**H$_2$N), 32.6 (**C**H$_2$CO), 30.7 (**C**H$_2$), 29.7 (**C**H$_2$), 28.2 ((**C**H$_3$)$_3$C), 23.9 (**C**H$_2$), 23.1 (**C**H$_2$); HRMS (ESI): calcd for $C_{19}H_{28}NO_4$ 334.2018 & $C_{19}H_{28}NNaO_4$. 356.1838. Found [MH]$^+$, 334.2007 (−3.29 ppm error) & [MNa]$^+$, 356.1830 (−2.24 ppm error).

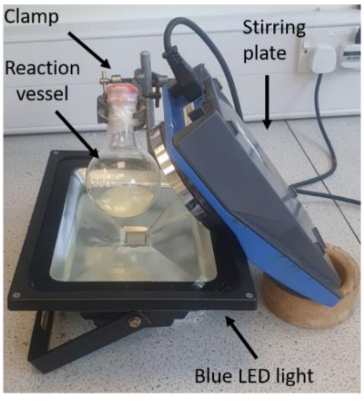

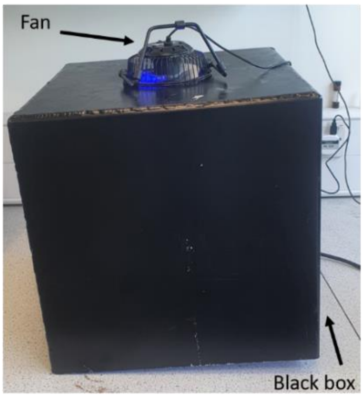

***tert*-Butyl 3-(1-(3-((*tert*-butoxycarbonyl) amino) propyl) pyrrolidin-2-yl) propanoate (S91)**

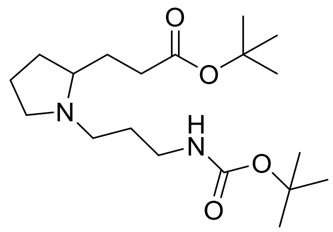

Palladium on carbon (0.200 g) was added to benzyl 2-(3-(*tert*-butoxy)-3-oxopropyl) pyrrolidine-1-carboxylate (**S90**) (2.01 g, 6.03 mmol) in ethanol (60.0 mL) under argon. The solution was evacuated and refilled with hydrogen (× 3) then stirred at room temperature overnight whilst excess hydrogen refills the reaction vessel. The mixture was filtered through Celite and washed with ethanol (100 mL) and the solvent was removed under vacuum using acetonitrile (3 × 15.0 mL) to make an azeotropic mixture. The mixture was redissolved in acetonitrile (30.0 mL) then potassium carbonate (1.67 g, 12.1 mmol) and 3-(Boc-amino) propyl bromide (1.44 g, 6.03 mmol) was added and refluxed overnight under argon. The reaction mixture was diluted with EtOAc (250 mL) and washed with saturated $NaHCO_3$ aqueous solution (200 mL) then back extracted with EtOAc (2 × 150 mL). The organic phases were collected and dried using sodium sulphate, filtered and the solvent removed under vacuum. The reaction mixture was purified via flash column chromatography (75:20:5 hexane:ethyl acetate:triethylamine) to afford the title compound **S91** as a yellow oil (1.85 g, 86%); ν_{max}/cm^{-1} (thin film) 3365, 2972, 2796, 1709, 1515, 1454, 1366, 1249, 1148, 848, 732; δ_H (400 MHz, $CDCl_3$) 5.35 (1H, s, N**H**), 3.23−3.16 (1H, m, C**H**$_a$H$_b$N), 3.13−3.08 (1H, m, C**H**$_a$H$_b$N), 3.08−3.03 (1H, m, CH$_a$**H**$_b$N), 2.83−2.72 (1H, m, C**H**$_a$H$_b$N), 2.27−2.07 (5H, m, CH$_a$**H**$_b$N, C**H**$_2$CO, NC**H**, C**H**$_a$H$_b$), 2.04−1.97 (1H, m, CH$_a$**H**$_b$N), 1.93−1.79 (2H, m, CH$_2$C**H**$_2$CH$_2$), 1.70−1.58 (5H, m, CHC**H**$_2$CH$_2$, CH$_2$C**H**$_2$CH$_2$, CH$_a$**H**$_b$), 1.39 (9H, s, (C**H**$_3$)$_3$O), 1.38 (9H, s, (C**H**$_3$)$_3$O); δ_C (101 MHz, $CDCl_3$) 173.2 (CH$_2$**C**OOC(CH$_3$)$_3$), 156.2 (NH**C**OOC(CH$_3$)$_3$), 80.2 (CH$_2$COO**C**(CH$_3$)$_3$), 78.8 (NHCOO**C**(CH$_3$)$_3$), 64.1 (**C**H), 53.9 (**C**H$_2$N), 52.7 (**C**H$_2$N), 39.9 (**C**H$_2$NH), 32.3 (**C**H$_2$COO), 29.9 (CH$_2$**C**H$_2$CH), 29.1 (CH**C**H$_2$CH$_2$), 28.5 ((**C**H$_3$)$_3$O), 28.3 (CH$_2$**C**H$_2$CH$_2$), 28.2 ((**C**H$_3$)$_3$O); HRMS (ESI): calcd. for $C_{19}H_{37}N_2O_4$ 357.2753. Found $[MH]^+$ 357.2754 (0.280 ppm error).

Decahydro-9H-pyrrolo[1,2-e] [1,5] diazonin-9-one (72)

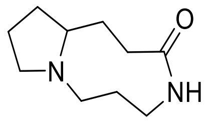

To a stirring solution of *tert*-butyl 3-(1-(3-((*tert*-butoxycarbonyl) amino) propyl) pyrrolidin-2-yl) propanoate (**S91**) (1.83 g, 5.15 mmol) in diethyl ether (13.0 mL), a solution of 4.0 M hydrochloric acid in 1,4 dioxane (26.0 mL, 104.0 mmol) was added dropwise and the reaction was left stirring at room temperature for 4 hours. The reaction mixture was concentrated under vacuum using chloroform (3 × 5.00 mL) to make an azeotropic solution. The intermediate carboxylic acid was then dissolved in chloroform (129.0 mL) and DIPEA (8.97 mL, 51.5 mmol) under argon, until all the reagents dissolved (30 minutes). T3P (50% w/v in ethyl acetate, 4.91 g, 7.72 mmol) was then added and the reaction left to stir over night at room temperature. The solvent was removed under vacuum and the reaction mixture was purified via flash column chromatography (50:45:5 dichloromethane:hexane:triethylamine) forming the title compound **72** as a colourless solid (0.715 g, 76%). In solution in $CDCl_3$, compound **72** exists as a ≈3:1 mixture of rotamers; R_f = 0.22 (70:25:5 dichloromethane: hexane:triethylamine); ν_{max}/cm^{-1} (thin film) 3281, 2944, 2791, 1641, 1467, 1444, 1365, 1293, 1170; δ_H (400 MHz, $CDCl_3$) rotameric mixture, 3:1 ratio, 6.65 (1H, s, N**H**)$_{minor\ rotamer}$, 5.91 (1H, s, N**H**)$_{major\ rotamer}$, 3.98–3.88 (1H, m, C**H**$_a$H$_b$N)$_{minor}$, 3.81–3.71 (1H, m, C**H**$_a$H$_b$N)$_{major}$, 3.23–2.98 [4H, m (CH$_a$**H**$_b$N)$_{both\ rotamers}$, (C**H**$_a$H$_b$N)$_{both\ rotamers}$], 2.78–2.66 (3H, m, (C**H**N)$_{major}$, (C**H**$_a$H$_b$N)$_{both\ rotamers}$), 2.56–2.52 (2H, m, (C**H**$_a$H$_b$)$_{both\ rotamers}$, (C**H**$_a$H$_b$N)$_{minor}$, 2.44–2.39 (2H, m, (C**H**N)$_{minor}$, (CH$_a$**H**$_b$N)$_{major}$), 2.26–2.10 (4H, m, CH$_a$**H**$_b$N)$_{both\ rotamers}$, (C**H**$_2$)$_{minor}$) 2.07–1.27 [18H, m, C**H**$_2$]$_{both\ rotamers}$; δ_C (101 MHz, $CDCl_3$) Data for the major rotamer only 179.4 (**C**O), 61.9 (**C**H), 53.3 (**C**H$_2$N), 49.0 (**C**H$_2$N), 41.1 (**C**H$_2$NH), 31.1 (**C**H$_2$CO), 29.7 (NCH$_2$**C**H$_2$CH$_2$N), 28.9 (CH**C**H$_2$CH$_2$), 25.3 (CH**C**H$_2$CH$_2$), 22.8 (CH$_2$**C**H$_2$CH$_2$); HRMS (ESI): calcd. for $C_{10}H_{18}N_2O$ 183.1497. Found $[MH]^+$ 183.1492 (−2.73 ppm error).

1-[(Benzyloxy) carbonyl] piperidine-2-carboxylic acid (S92)

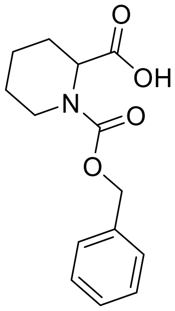

To a stirring solution of piperidine-2-carboxylic acid (10.0 g, 77.4 mmol) in water (155 mL) and 1,4 dioxane (155 mL), sodium carbonate (18.9 g, 178 mmol) was added. Benzoyl chloroformate (15.8 g, 92.9 mmol) was then added dropwise and the reaction was left to stir over night at room temperature. The reaction was concentrated under vacuum using dichloromethane to make an azeotropic mixture. 10% HCl was added until the reaction mixture reached pH = 2. tested by pH indicator sticks. The solution was extracted in dichloromethane (200 mL × 3), the organic phases were combined, dried with sodium sulphate, filtered and the solvent removed under vacuum. To afford the title compound as a colourless viscous oil (19.3 g, 95%); ν_{max}/cm^{-1} (thin film) 2941, 2861, 2542, 1704, 1671, 1423, 1353, 1256, 1165, 1042, 864, 740, 697, 603; δ_H (400 MHz, $CDCl_3$) 11.19 (1H, bs, COO**H**), 7.36−7.31 (5H, m, ArC**H**), 5.20−5.12 (2H, m, ArC**H**$_2$O), 5.03−4.89 (1H, m, C**H**), 4.17−4.02 (1H, m, C**H**$_a$H$_b$N), 3.16−2.96 (1H, m, CH$_a$**H**$_b$N), 2.32−2.20 (1H, m, C**H**$_a$H$_b$), 1.75−1.60 (1H, m, CH$_a$**H**$_b$), 1.75−1.60 (1H, m, C**H**$_a$H$_b$), 1.75−1.60 (1H, m, C**H**$_a$H$_b$), 1.49−1.26 (1H, m, CH$_a$**H**$_b$), 1.49−1.26 (1H, m, CH$_a$**H**$_b$); δ_c (101 MHz, $CDCl_3$) 177.1 & 177.0 (**C**OOH), 156.9 & 156.3 (**C**O), 136.5 (Ar**C**), 128.6 (Ar**C**H), 128.2 (Ar**C**H), 127.9 (Ar**C**H), 67.7 & 67.3 (Ar**C**H$_2$O), 54.5 & 54.4 (**C**H), 40.0 & 40.3 (**C**H$_2$), 26.8 & 26.7 (**C**H$_2$), 24.8 & 24.6 (**C**H$_2$), 20.8 & 20.7 (**C**H$_2$); HRMS (ESI) calcd. for $C_{14}H_{17}NNaO_4$ 286.1055 & $C_{14}H_{17}NKO_4$ 302.0795. Found $[MNa]^+$ 286.1057 (0.70 ppm error) & $[MK]^+$ 302.0781 (−4.63 ppm error).

Benzyl 2-(3-*tert*-butoxy-3-oxopropyl) piperidine-1-carboxylate (S93)

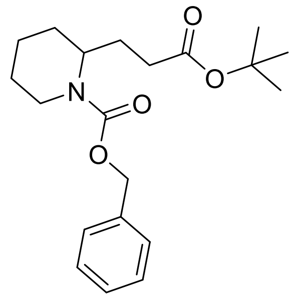

To a 250 mL round bottom flask of 1-[(benzyloxy)carbonyl] piperidine-2-carboxylic acid (**S92**) (2.77 g, 8.00 mmol), 4CzIPN (0.063 g, 0.08 mmol) and dipotassium phosphate (1.67 g, 9.6 mmol) was added to DMF (15.0 mL) and degassed for 15 minutes using argon. Separately, *tert*-butyl acrylate (3.00 mL) was degassed for 15 minutes using argon. A portion of the degassed *tert*-butyl acrylate (1.03 g ,8.00 mmol) was then added to the 250 mL round bottom flask topped with a septum wrapped with parafilm several times. The reaction mixture was irradiated with blue LED light for 72 hours whilst stirring, using the same set up as shown below. The reaction was quenched in saturated $NaHCO_3$ aqueous solution (100 mL) and extracted in Et_2O (3 × 50.0 mL). The organic layers were collected and washed with saturated NaCl aqueous solution (2 × 100 mL) and water (2 × 100 mL). The organic phases were collected and dried with sodium sulphate and the solvent removed under vacuum. Purification via flash column chromatography (15:4:1 hexane:ethyl acetate:triethylamine) afforded the title compound **S93** as a clear oil (1.85 g, 67%); R_f = 0.42 (15:4:1 hexane:ethyl acetate:triethylamine); ν_{max}/cm^{-1} (thin film) 2935, 2864, 1726, 1693, 1422, 1350, 1256, 1150, 847, 731, 696; δ_H (400 MHz, $CDCl_3$) 7.30−7.11 (5H, m, Ar**H**), 5.11 (2H, s, ArC**H**$_2$O), 4.36−4.23 (1H, bm, C**H$_a$**H$_b$N), 2.83 (1H, t, *J* = 12.0 Hz), 2.26−2.13 (2H, m, C**H$_2$**CO), 2.11−2.00 (1H, m, C**H**$_a$H$_b$), 1.70−1.48 (1H, m, CH$_a$**H**$_b$) 1.70−1.48 (1H, m, C**H**$_a$H$_b$), 1.70−1.48 (1H, m, C**H**$_2$), 1.70−1.48 (1H, m, C**H**$_2$), 1.70−1.48 (1H, m, C**H**$_2$), 1.41 (9H, s, OC(C**H**$_3$)$_3$); δ_c (101 MHz, $CDCl_3$) 172.9 & 172.4 (N**C**OO), 155.6 & 155.4 (CH$_2$**C**OO), 137.9 & 137.1 (Ar**C**) 128.12 & 128.6 (Ar**C**H), 128.3 & 128.0 (Ar**C**H), 127.9 & 125.4 (Ar**C**H), 80.6 & 80.3 (O**C**(CH$_3$)$_3$), 67.0 (Ar**C**H$_2$O), 50.6 (**C**HN), 39.9 (C**H**$_2$N), 32.5 (**C**H$_2$COO),28.9 (**C**H$_2$), 28.2 (OC(**C**H$_3$)$_3$), 25.6 (**C**H$_2$), 25.1 (**C**H$_2$), 19.1 (**C**H$_2$); HRMS (ESI) calcd. for $C_{20}H_{29}NNaO_4$ 370.1994. Found [MNa]$^+$, 370.1988 (−1.62 ppm error).

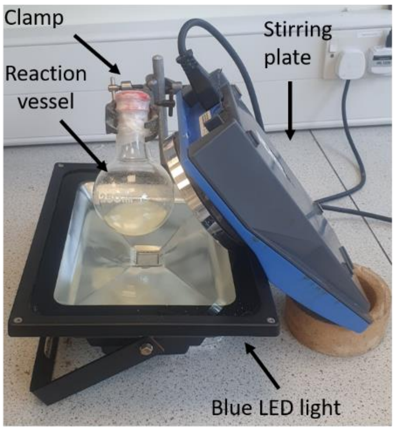

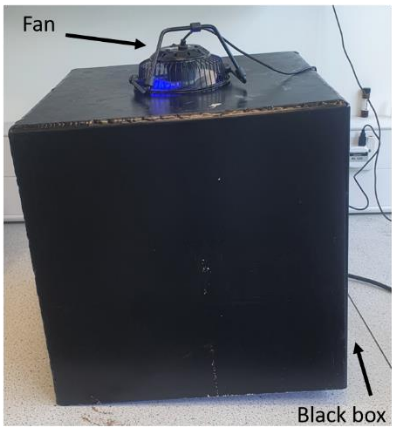

tert-Butyl 3-(1-(3-((tert-butoxycarbonyl) amino) propyl) piperidin-2-yl) propanoate (S94)

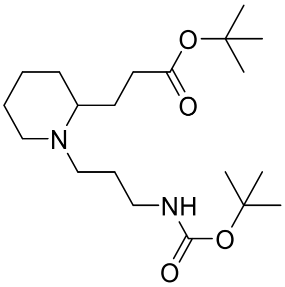

Palladium on carbon (0.215 g) was added to benzyl 2-(3-*tert*-butoxy-3-oxopropyl) piperidine-1-carboxylate (**S93**) (2.15 g, 6.19 mmol) in ethanol (62.0 mL) under argon. The solution was evacuated and refilled with hydrogen (× 3) then stirred at room temperature overnight whilst excess hydrogen refills the reaction vessel. The mixture was filtered through Celite and washed with ethanol (100 mL) and the solvent was removed under vacuum using acetonitrile (3 × 15.0 mL) to make an azeotropic mixture. The mixture was redissolved in acetonitrile (31.0 mL) then potassium carbonate (1.71 g, 12.4 mmol) and 3-(Boc-amino) propyl bromide (1.48 g, 6.19 mmol) was added and refluxed overnight under argon. The reaction mixture was diluted with EtOAc (250 mL) and washed with saturated $NaHCO_3$ aqueous solution (200 mL) then back extracted with EtOAc (2 × 150 mL). The organic phases were collected and dried using sodium sulphate, filtered and the solvent removed under vacuum. The reaction mixture was purified via flash column chromatography (75:20:5 hexane:ethyl acetate:triethylamine) to afford the title compound **S94** as a pale yellow oil (1.38 g, 60%). In solution in $CDCl_3$, the compound exists as a ≈4:1 mixture of rotamers; R_f = 0.24 (75:20:5 hexane:ethyl acetate:triethylamine); ν_{max}/cm^{-1} (thin film) 3369, 2976, 2932, 1713, 1513, 1454, 1366, 1248, 1152, 1046, 849, 780, 608; δ_H (400 MHz, $CDCl_3$) 5.61 (bs, 1H, N**H**), 4.20–4.16 (1H, m, C**H**$_a$H$_b$N)$_{minor}$, 4.10–4.03 (1H, m, CH$_a$**H**$_b$N)$_{minor}$, 3.16–3.06 (2H, m, C**H**$_2$N)$_{both\ rotamers}$, 2.79–2.74

(1H, m, C$H_a$$H_b$N)$_{major}$, 2.70−2.67 (1H, m, C$H_a$$H_b$N)$_{major}$, 2.32−2.09 [(5H, m, C**H**N, CH_a**H_b**N, CH_a**H_b**N, C**H_2**COO)$_{major\ rotamers}$, (1H, m, C**H**N)$_{minor}$], 1.99−1.98 (2H, m, CH_2N)$_{minor}$, 1.83−1.18 [(10H, m C**H_2**)$_{both\ rotamers}$ 1.83−1.76 (m), 1.70−1.48 (m), 1.31−1.18 (m), 1.40−1.38 (18H, m COOC(C**H_3**)$_3$, NHCOOC(C**H_3**)$_3$)$_{both\ rotamers}$; δ_C (101 MHz, $CDCl_3$) only the major rotamer detailed 173.4 (**C**O), 156.2 (**C**O), 80.2 (O**C**(CH_3)$_3$, 78.7 (O**C**(CH_3)$_3$, 59.5 (**C**H), 52.1 (**C**H_2N), 50.6 (**C**H_2N), 40.3 (**C**H_2NH), 31.8 (**C**H_2COO), 29.7 (**C**H_2), 28.5 (OC(**C**H_3)$_3$), 28.4 (**C**H_2), 28.2 (OC(**C**H_3)$_3$), 26.0 (**C**H_2), 25.3 (**C**H_2), 22.8 (**C**H_2); HRMS (ESI): calcd. for $C_{20}H_{38}N_2O_4$ 371.2910. Found $[MH]^+$ 371.2904 (−1.62 ppm error).

Decahydropyrido[1,2-e] [1,5] diazonin-3(2H)-one (73)

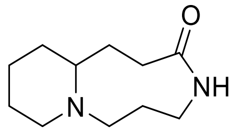

To a stirring solution of *tert*-butyl 3-(1-(3-((*tert*-butoxycarbonyl) amino) propyl) pyrrolidin-2-yl) propanoate (**S94**) (1.38 g, 3.73 mmol) in diethyl ether (9.30 mL), a solution of 4.0 M hydrochloric acid in 1,4 dioxane (18.7 mL, 74.8 mmol) was added dropwise and the reaction was left stirring at room temperature for 4 hours. The reaction mixture was concentrated under vacuum using chloroform (3 × 5.00 mL) to make an azeotropic solution. The intermediate amino acid salt was dissolved in chloroform (93.0 mL) and DIPEA (6.49 mL, 37.3 mmol) under argon, until all the reagents dissolved into solution (30 minutes). T3P (50% w/v in ethyl acetate, 3.56 g, 5.59 mmol) was then added, and the reaction left to stir over night at room temperature. The solvent was removed under vacuum and the reaction mixture was purified via flash column chromatography (70:25:5 dichloromethane:hexane:triethylamine) to form the title compound **73** as a colourless solid (0.466 g, 64%), R_f = 0.32 (70:25:5 dichloromethane:hexane:triethylamine); ν_{max}/cm^{-1} (thin film) 3189, 3060, 2925, 2841, 2796, 1654, 1439, 1398, 1180, 1027, 950, 832, 580, 482; δ_H (400 MHz, $CDCl_3$) 6.43 (1H, s, N**H**), 3.96−3.69 (1H, m, C**H_a**H_bN), 3.20−2.92 (1H, m, CH_a**H_b**N), 2.92−2.59 (2H, m, C**H_2**N), 2.37−2.24 (1H, m, C**H**N), 2.05−1.85 (4H, m, C**H_2**CO, C**H_2**N), 1.85−1.12 (10H, m, 5 × C**H_2**); δ_C (101 MHz, $CDCl_3$) 179.7 (**C**O), 59.5 (**C**HN), 52.9 (**C**H_2N), 46.2 (**C**H_2N), 40.1 (**C**H_2NH), 32.4 (**C**H_2CO), 30.6 (**C**H_2), 28.1 (**C**H_2), 26.1 (**C**H_2), 24.8 (**C**H_2), 24.4 (**C**H_2); HRMS (ESI): calcd. for $C_{11}H_{21}N_2O$ 197.1654. Found $[MH]^+$ 197.1651 (−1.52 ppm error).

Methyl 2-(((3-azidopropyl)thio)methyl)benzoate (S95)

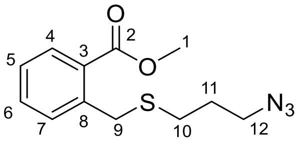

NaN_3 (342 mg, 5.30 mmol) was added to a colourless solution of methyl 2-(((3-bromopropyl)thio)methyl)benzoate **S98** (1.06 g, 3.51 mmol) in anhydrous DMF (12 mL) at RT under Ar. The resulting suspension was then heated to 70 °C, whereupon an immediate colour change to pale yellow was noted. Upon further heating, the colour of the reaction suspension changed to milky white. After 20 h, the reaction was deemed to have gone to completion by TLC analysis, with a colour change to pale yellow creamy suspension noted. The reaction mixture was cooled to RT, before being quenched with addition of H_2O (30 mL). The resulting milky/white suspension was poured into a separating funnel containing EtOAc (30 mL). The aqueous layer was extracted with EtOAc (3 × 20 mL), before the combined organic phases were washed with sat. brine (3 × 20 mL). The organic layer was dried over $MgSO_4$, filtered and concentrated under reduced pressure to yield orange oil (1.09 g). The crude product was purified by flash column chromatography (SiO_2, 40 mm column, eluent: EtOAc:*n*–hexane, 10:90) to afford azide **S95** as a colourless oil (1.07 g, 80%). R_f = 0.23 (10:90 EtOAc:*n*–hexane); IR (neat) ν_{max} / cm^{-1}: 2953w (C–H alkyl), 2515w, 2094s (N=N=N azide), 1717s (C=O aryl tester), 1600w (CC aromatic), 1576w (CC aromatic), 1488w, 1447w, 1434w, 1292w, 1260s, 1190w, 1164w, 1121m, 1077s, 1046w, 966w, 893w, 801w, 765m, 715s, 663w, 580w, 556w, 467w; δ_H (400 MHz; $CDCl_3$) 7.92 (1H, dd, *J* = 7.6, 1.4 Hz, C(4)H), 7.45 (1H, td, *J* = 7.6, 1.6 Hz, C(6)H), 7.37 – 7.26 (2H, m, C(5+7)H), 4.12 (2H, s, C(9)H_2), 3.91 (3H, s, C(1)H_3), 3.35 (2H, t, *J* = 6.6 Hz, C(12)H_2), 2.51 (2H, t, *J* = 7.1 Hz, C(10)H_2), 1.86 – 1.72 (2H, m, C(11)H_2); δ_C (101 MHz; $CDCl_3$) 167.8 (C, C1), 140.5 (C, C8), 131.9 (CH, C6), 131.2 (CH, C4), 131.0 (CH, C7),129.5 (C, C3), 127.2 (CH, C5), 52.2 (CH_3, C1), 50.1 (CH_2, C12), 34.6 (CH_2, C9), 28.7 (CH_2, C10), 28.6 (CH_2, C11); HRMS (ESI): calcd. for $C_{12}H_{15}N_3NaO_2S$, 288.0777 Found: $[MNa]^+$, 288.0777 (–0.1 ppm error).

2-(((3-Aminopropyl)thio)methyl)benzoic acid (S97)

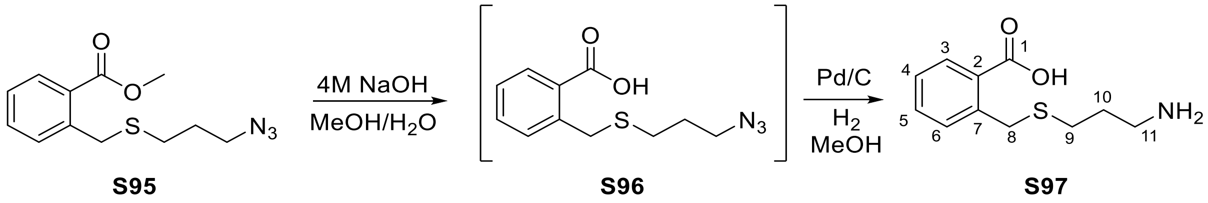

$NaOH_{(aq)}$ (4.0 M, 18.0 mL) was added dropwise over a period of 15 min to a colourless solution of methyl 2-(((3-azidopropyl)thio)methyl)benzoate (1.01 g, 3.51 mmol) **S95** in MeOH (18.0 mL) at RT. A colour change to milky white suspension was noted immediately during dropwise addition. The resulting suspension was then heated to 60 °C, whereupon a colour change to a colourless solution was observed. After a total of 24 h, the reaction was deemed to have gone to completion by TLC analysis (complete consumption of methyl 2-(((3-azidopropyl)thio)methyl)benzoate **S95** was noted). The colourless reaction mixture was allowed to cool to RT and then acidified to pH 2.0 with 1 M $HCl_{(aq)}$ (30 mL). The resulting acidified solution was poured into separating funnel. The aqueous layer was extracted with CH_2Cl_2 (3 × 20 mL), before combined organic phases were dried over Na_2SO_4, filtered and concentrated under reduced pressure to afford a carboxylic acid **S96** as a white solid. The crude product was directly used in the next reaction step without further purification. Carboxylic acid **S96** (873 mg) was dissolved in MeOH (24.0 mL, degassed for 15 min) and placed under an Ar atmosphere. Palladium on carbon (56.0 mg, Pd 10% on carbon) was then added and the reaction vessel was evacuated and backfilled with H_2 (via balloon) three times, then stirred at RT under a slight positive pressure of H_2 (balloon) for 24 h. The reaction was then purged with argon, filtered through Celite, washed with MeOH (2× 10 ml) and the solvent was removed *in vacuo*. Purification by flash column chromatography (SiO_2, MeOH:EtOAc,70:30) afforded the title compound **S97** as a white solid (202 mg, 38%); R_f = 0.28 (70:30 MeOH:EtOAc); Melting Point: 110 – 112 °C; IR (solid state) ν_{max} / cm^{-1}: 3064vbrm (O–H alcohol/carboxylic acid/NH), 2922m (C–H alkyl), 2757m (C–H alkyl, O–H alcohol/carboxylic acid), 2214w, 1628w (C=O aryl carboxylic acid), 1600w (CC aromatic), 1579w (CC aromatic), 1524s, 1445w, 1412w, 1376s, 1331w, 1237w, 1150w, 1087w, 957w, 855w, 818w, 797w, 767w, 746w, 725s, 687w, 661m, 566w, 482w; δ_H (400 MHz; CD_3OD) 7.52 – 7.44 (1H, m, C(3)H), 7.37 – 7.31 (1H, m, C(5)H), 7.30 – 7.17 (2H, m, C(4+6)H), 4.09 – 4.03 (2H, m, C(8)H_2), 2.99 – 2.89 (2H, m, C(11)H_2), 2.53 – 2.43 (2H, m, C(9)H_2), 1.93 – 1.81 (2H, m, C(10)H_2);

δ_C (126 MHz; CD_3OD) 178.0 (C, C1), 142.2 (C, C7), 137.2 (C, C2), 131.3 (CH, C5), 129.5 (CH, C3), 128.7 (CH, C6), 127.7 (CH, C4), 39.6 (CH_2, C11), 33.8 (CH_2, C8), 28.8 (CH_2, C10), 28.2 (CH_2, C9); HRMS (ESI): calcd. for $C_{11}H_{16}NO_2S$, 226.0896. Found: $[MH]^+$, 226.0896 (–0.1 ppm error), calcd. for $C_{11}H_{15}NNaO_2S$, 248.0716. Found: $[MNa]^+$, 248.0717 (–0.4 ppm error).

3,4,5,6-Tetrahydrobenzo[g][1,5]thiazonin-7(1H)-one (74)

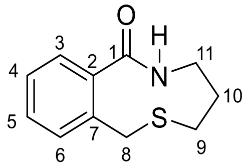

EDC.HCl (343 mg, 1.78 mmol) was added to clear colorless solution of 2-(((3-aminopropyl)thio)methyl)benzoic acid **S97** (177 mg, 0.784 mmol), HOBt (127 mg, 0.941 mmol) and dry DIPEA (350 µL, 1.96 mmol) in anhydrous DMF (7.84 mL) at RT. An immediate colour change to pale yellow was observed. After total of 18 h of stirring at RT under Ar, the reaction was deemed to have gone completion by TLC analysis. The reaction mixture was diluted with EtOAc (30 mL) and poured into separating funnel. The diluted solution was washed sequentially with 1 M $HCl_{(aq)}$ (3 × 20 mL), sat. $NaHCO_{3(aq)}$ (3 × 20 mL) and sat. brine (4 × 20 mL), before being dried over $MgSO_4$, filtered and concentrated under reduced pressure to yield a pale-yellow solid (161 mg). The crude product was purified by flash column chromatography (SiO_2, 20 mm column, eluent: EtOAc) to afford the title compound **73** as a white solid (56.4 mg, 35%). In solution in CD_2Cl_2, this compound exists as a >20:1 mixture of rotamers. R_f = 0.27 (EtOAc); Melting Point: 125 – 125 °C (from *n*–hexane:CH_2Cl_2); IR (solid state) ν_{max} / cm^{-1}: 3276w, 3172w (C–H alkyl), 3045w (C–H alkyl), 2921m (C–H alkyl), 2899w (C–H alkyl), 2838w, 1643s (C=O aryl amide), 1601w (CC aromatic), 1575w (CC aromatic), 1467w, 1448w, 1405s, 1348m, 1279w, 1232w, 1215w, 1148w, 1068w, 946w, 875w, 843m, 769s, 761s, 721m, 683m, 657w, 615m, 602m, 476m; NMR data for the major rotamer only: δ_H (400 MHz; CD_2Cl_2) 7.37 (1H, td, *J* = 7.6, 1.5 Hz, C(3)H), 7.30 (1H, td, *J* = 7.6, 1.5 Hz, C(6)H), 7.23 (1H, dd, J = 7.6, 1.5 Hz, C(4)H), 7.16 (1H, dd, *J* = 7.6, 1.5 Hz, C(6)H), 5.90 (1H, br, s, NH), 4.07 (1H, d, *J* = 14.8 Hz, C(8)**H**H'), 3.28 (1H, d, *J* = 14.7 Hz, C(8)H**H'**), 3.21 – 3.01 (3H, m, C(11)H_2 + C(9)H**H'**), 2.83 – 2.71 (1H, m, C(9)**H**H'), 1.99 – 1.86 (1H, m, C(10)H**H'**), 1.49 – 1.35 (1H, m, C(10)**H**H'); δ_C (126 MHz; CD_2Cl_2) 173.4 (C, C1), 137.3 (C, C7), 135.3 (C, C2), 130.2 (CH, C3), 129.8 (CH, C4), 127.8 (CH, C5), 126.6 (CH, C6), 42.0 (CH_2, C8), 39.5 (CH_2, C11), 35.3 (CH_2,

C9), 31.2 (CH_2, C10); HRMS (ESI): calcd. for $C_{11}H_{14}NOS$, 208.0791. Found: $[MH]^+$, 208.0795 (–2.2 ppm error), calcd. for $C_{11}H_{13}NNaOS$, 230.0610. Found: $[MNa]^+$, 230.0615 (–2.1 ppm error).

X-ray crystallographic data for compound **74** can be accessed via www.ccdc.cam.ac.uk/data_request/cif (CCDC 2236990)

Methyl 2-(((3-bromopropyl)thio)methyl)benzoate (S98)

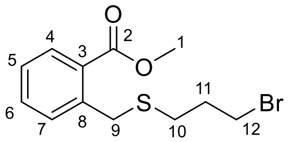

PPh_3 (1.48 g, 4.63 mmol) was added slowly in equal portions over a period of 1 min to a solution of alcohol **12** (618 mg, 2.57 mmol) and CBr_4 (1.72 g, 4.63 mmol) in anhydrous CH_2Cl_2 (26 mL) at 0 °C. A colour change to sunflower yellow was immediately noted. The mixture stirred for 2 h at 0 °C and then allowed to warm to RT to stir for an additional 16 h, with a colour change to orange observed. After total of 18 h, the reaction was deemed to have gone to completion by TLC. The reaction mixture was concentrated under reduced pressure to dryness to yield crude product as a brown/orange oil (2.01g). The crude product was purified by flash column chromatography (SiO_2, 50 mm column, eluent: EtOAc:*n*–hexane, 10:90) to afford the title compound **S98** as a clear colorless oil (720 mg 86%). R_f = 0.57 (50:50 EtOAc:*n*-hexane); IR (thin film) ν_{max} / cm^{-1}: 2949m (C–H alkyl), 1716s (C=O aryl ester), 1600w (CC aromatic), 1576w (CC aromatic), 1488m (CC aromatic), 1433w, 1292m, 1261vs, 1190m, 1164w, 1120m, 1076s, 1046w, 964w, 840w, 801w, 767m, 714s, 663w, 580w, 561w; δ_H (400 MHz; $CDCl_3$) 7.91 (1H, dd, *J* 7.7, 1.5, C(4)H), 7.45 (1H, td, *J* 7.7, 1.5, C(7)H), 7.40 – 7.28 (2H, m, C(5+6)H), 4.13 (2H, s, C(9)H_2), 3.91 (3H, s, C(1)H_3), 3.46 (2H, t, *J* 6.5, C(12)H_2), 2.58 (2H, t, *J* 6.6, C(10)H_2), 2.14 – 1.98 (2 H, m, C(11)H_2); δ_C (101 MHz; $CDCl_3$) 167.7 (C, C2) 140.4 (C, C8), 131.9 (CH, C7), 131.2 (CH, C4), 131.0 (CH, C6), 129.4 (C, C3), 127.1 (CH, C5), 52.2 (CH_3, C1), 34.4 (CH_2, C9) 32.3 (CH_2, C12), 32.1 (CH_2, C11), 29.9 (CH_2, C10); HRMS (ESI^+): m/z calc. for $C_{12}H_{15}{}^{79}BrNaO_2S$: 324.9868, found: 324.9870 $[MNa]^+$.

Methyl 2-(((3-(phenylamino)propyl)thio)methyl)benzoate (S99)

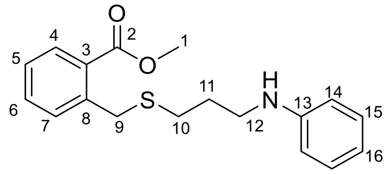

Aniline (550 µL, 5.79 mmol) was added dropwise over 1 min to a pale-yellow solution of bromide **S98** (1.17 g, 3.86 mmol) in anhydrous MeCN (39 mL) at RT under Ar, along with KI (108 mg, 0.386 mmol) and K_2CO_3 (1.07 g, 7.72 mmol). The resulting beige suspension was heated at 90 °C for a total of 18 h, after which time the reaction was deemed to have gone to completion by TLC. The reaction mixture allowed to cool to RT and diluted with 40 mL of EtOAc. The diluted milky white suspension was poured into to separating funnel, where organic layer was washed with sat. brine (4 × 30 mL), before being dried over $MgSO_4$, filtered and concentrated under reduced pressure to yield a pale brown liquid (1.63 g). The crude product was purified by flash column chromatography (SiO_2, 50 mm column, eluent: EtOAc:*n*–hexane, 20:80) to afford an aniline **S99** as a pale yellow oil (731 mg, 60%). R_f = 0.45 (50:50 EtOAc:*n*–hexane); IR (neat) ν_{max} / cm^{-1}: 3416wbr (N–H aryl secondary amine), 2945w (C–H alkyl), 1716s (C=O aryl ester), 1601s (CC aromatic), 1506s (CC aromatic), 1433m, 1297m, 1260s, 1180w, 1122m, 1077m, 1046w, 965w, 747s, 715s, 692s, 664w, 509w; δ_H (400 MHz; $CDCl_3$) 7.99 (1H, dd, *J* 8.1 1.5, C(4)H), 7.47 – 7.37 (1H, m, C(7)H), 7.36 – 7.27 (2H, m, C(5+6)H), 7.22 – 7.11 (2H, m, C(15)H), 6.69 (1H, tt, *J* 7.3, 1.1, C(16)H), 6.62 – 6.54 (2H, m, C(14)H), 4.12 (2H, s, C(9)H_2), 3.91 (3H, s, C(1)H_3), 3.19 (2H, t, *J* 6.7, C(12)H_2), 2.53 (2H, t, *J* 7.1, C(10)H_2), 1.84 (2H, p, *J* 6.7, C(11)H_2); δ_C (101 MHz; $CDCl_3$) 167.9 (C, C1), 148.2 (C, C13), 140.6 (C, C8), 131.9 (CH, C7), 131.2 (CH, C6),131.0 (CH, C4), 129.6 (C, C3), 129.3 (CH, C15), 127.1 (CH, C5), 117.2 (CH, C16), 112.7 (CH, C14), 52.2 (CH_3, C1), 42.6 (CH_2, C12), 34.6 (CH_2, C9), 29.3 (CH_2, C10), 28.8 (CH_2, C11); HRMS (ESI$^+$): m/z calc. for $C_{18}H_{22}NO_2S$: 316.1366, found: 316.1363 $[M+H]^+$, m/z calc. for $C_{18}H_{21}NNaO_2S$: 338.1185, found : 338.1184 $[M+Na]^+$.

2-(((3-(Phenylamino)propyl)thio)methyl)benzoic acid (S100)

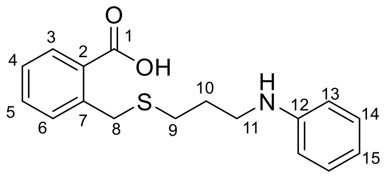

$NaOH_{(aq)}$ (4.0 M, 10.0 mL) was added dropwise over a period of 5 min to a pale-yellow solution of ester **S99** (614 mg, 1.95 mmol) in MeOH (10 mL). A colour change to milky-white suspension was noted immediately during dropwise addition. The resulting suspension was stirred at 60 °C for 16 h, after which the reaction was deemed to have gone to completion by TLC analysis (complete consumption of **S99** noted), with a colour change to pale yellow solution observed. The resulting mixture was acidified to pH 2.0 with 1 M $HCl_{(aq)}$ (30 mL) and poured into separating funnel containing CH_2Cl_2 (20 mL). The aqueous layer was extracted with CH_2Cl_2 (3 × 20 mL), before combined organic phases were dried over $MgSO_4$, filtered and concentrated under reduced pressure to afford a carboxylic acid **S100** as a dark brown/green viscous oil (530 mg, 90%). R_f = 0.02 (50:50 EtOAc:*n*–hexane); IR (neat) ν_{max} / cm^{-1}: 3409wbr (N–H aryl secondary amine), 2925wvbr (C–H alkyl/ O–H carboxylic acid), 2657wvbr (O–H carboxylic acid), 1688m (C=O aryl carboxylic acid), 1601s (CC aromatic), 1575w (CC aromatic), 1505s (CC aromatic), 1374w, 1258m, 1129w, 1075w, 907m, 726vs, 692vs, 582m, 509w; δ_H (400 MHz; $CDCl_3$) 8.06 (1H, br, s, OH), 8.03 (1H, dd, *J* 8.0 1.5, C(3)H), 7.47 (1H, td, *J* 7.5 1.5, C(6)H), 7.37 – 7.31 (2H, m, C(4+5)H), 7.23 – 7.14 (2H, m, C(14)H), 6.82 (1H, tt, *J* 7.5 1.4, C(15)H), 6.79 – 6.75 (2H, m, C(13)H), 4.17 (2H, s, $C(8)H_2$), 3.90 (1H, s, NH), 3.24 (2H, t, *J* 7.1, $C(11)H_2$), 2.54 (2H, t, *J* 7.1, $C(9)H_2$), 1.92 (2H, p, *J* 7.1, $C(10)H_2$); δ_C (101 MHz; $CDCl_3$) 172.3 (C, C1), 145.2 (C, C12), 140.8 (C, C7), 132.3 (CH, C6), 131.8 (CH, C3), 131.1 (CH, C5), 129.4 (C, C2), 129.3 (CH, C14), 127.1 (CH, C4), 120.1 (CH, C15), 115.3 (CH, C13), 44.7 (CH_2, C11), 34.4 (CH_2, C8), 29.1 (CH_2, C9), 27.8 (CH_2, C10); HRMS (ESI^+): m/z calc. for $C_{17}H_{20}NO_2S$: 302.1209, found: 302.1207 $[M+H]^+$, m/z calc. for $C_{17}H_{19}NNaO_2S$: 324.1029, found : 324.1025 $[M+Na]^+$.

6-Phenyl-3,4,5,6-tetrahydrobenzo[*g*][1,5]thiazonin-7(1*H*)-one (75)

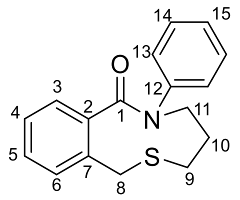

EDC.HCl (74.1 mg, 0.222 mmol) was added to a pale-yellow solution of carboxylic acid **S100** (44.6 mg, 0.148 mmol), HOBt (51.3 mg, 0.178 mmol) and anhydrous DIPEA (100 µL, 4.44 mmol) in anhydrous DMF (3 mL) at RT under Ar, with a colour change to orange/brown noted. The resulting mixture was stirred at RT for 24 h, after which time the reaction was deemed to have gone completion by TLC. The reaction mixture was diluted with EtOAc (30 mL) and transferred into separating funnel. The resulting milky/white organic layer was washed sequentially with 1 M $HCl_{(aq)}$ (3 × 20 mL), sat. $NaHCO_3$ (3 × 20 mL) and brine (3 × 20 mL), before being dried over $MgSO_4$, filtered and concentrated under reduced pressure to yield a clear colourless oil (143 mg). The crude product was purified by flash column chromatography (SiO_2, 20 mm column, eluent: acetone:*n*–hexane, 20:80) to afford lactam **75** as a white solid (31.5 mg, 75%). R_f = 0.50 (40:60 EtOAc:*n*–hexane); IR (solid state) ν_{max} / cm^{-1}: 3064w (C–H alkyl), 3012w (C–H alkyl), 2924w (C–H alkyl), 1643vs (C=O aryl lactam), 1593s (CC aromatic), 1493 (CC aromatic), 1460w, 1449w, 1398s, 1275m, 1162m, 1074w, 1011w, 752vs, 707m, 664m; δ_H (500 MHz; $CDCl_3$) 7.52 – 7.29 (8H, m, C(3–6 and 13–14)H), 7.23 (1H, d, *J* 7.7, C(15)H), 4.19 (1H, d, 14.7, C(8)**H**H’), 3.89 – 3.66 (2H, m, C(11)H_2), 3.52 (1H, d, 14.7, C(8)H**H’**), 2.97–2.90 (1H, m, C(9)**H**H’), 2.75–2.65 (1H, m, C(9)H**H’**), 1.98 – 1.27 (2H, m, C(10)H_2); δ_C (126 MHz; $CDCl_3$) 171.5 (C, C1), 139.1 (C, C7), 136.6 (C, C12), 136.3 (C, C2), 130.1 (CH, C5), 129.7 (CH, C3), 129.5 (CH, C14), 127.9 (CH, C6),127.3 (CH, C4), 126.7 (CH, C13), 126.5 (CH, C15), 49.1 (CH_2, C11), 39.4 (CH_2, C8), 35.0 (CH_2, C9), 28.9 (CH_2, C10); HRMS (ESI^+): m/z calc. for $C_{17}H_{18}NOS$: 284.1104, found: 284.1104 $[M+H]^+$, m/z calc. for $C_{17}H_{17}NNaO_2S$: 306.0923, found : 306.0929 $[M+Na]^+$.

X-ray crystallographic data for compound **75** can be accessed via www.ccdc.cam.ac.uk/data_request/cif (CCDC 2235962)

Methyl 2-((methyl(2-(phenylamino) ethyl) amino) methyl) benzoate (S101)

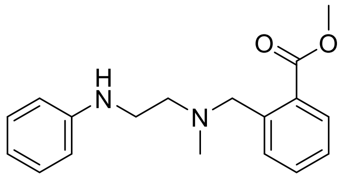

To a stirring solution of potassium carbonate (0.104 g, 0.75 mmol) in acetonitrile (7.50 mL), N-methyl-N-phenylethane-1,2-diamine (0.225 g, 1.50 mmol) was added followed by methyl 2-(bromomethyl) benzoate (0.115 g, 0.500 mmol). The reaction mixture was stirred at room temperature for 5 hours under argon before filtering through Celite washing with DCM, concentrating under vacuum and purifying by flash column chromatography (hexane:ethyl acetate 1:1) to afford the title compound **S101** as a yellow oil (0.149 g, 99%) R_f = 0.59 streek (1:1 hexane:ethyl acetate); ν_{max}/cm^{-1} (thin film) 3391, 2949, 2798, 1717, 1602, 1505, 1431, 1273, 1249, 1082, 741; δ_H (400 MHz, $CDCl_3$) 7.77 (1H, dd, *J* = 7.5 Hz, *J* = 1.4 Hz, Ar**H**), 7.47−7.38 (2H, m, Ar**H**), 7.35(1H, td, *J* = 7.5 Hz, 1.6 Hz, Ar**H**), 7.23−7.19 (2H, m, Ar**H**), 6.74−6.68 (3H, m, Ar**H**), 3.95 (3H, s, OC**H**$_3$), 3.85 (2H, s, NC**H**$_2$Ar), 3.14 (2H, t, *J* = 5.9 Hz, NC**H**$_2$CH$_2$N), 2.65 (2H, t, *J* = 5.9 Hz, NCH$_2$C**H**$_2$N), 2.21 (3H, s, NC**H**$_3$); δ_c (101 MHz, $CDCl_3$) 170.0 (**C**O), 148.9 (Ar**C**), 139.8 (Ar**C**), 130.1 (Ar**C**), 131.2 (Ar**C**H), 130.3 (Ar**C**H), 129.9 (Ar**C**H), 129.3 (Ar**C**H), 127.4 (Ar**C**H), 117.0 (Ar**C**H), 112.9 (Ar**C**H), 61.1 (Ar**C**H$_2$N), 55.8 (**C**H$_2$N), 52.4 (**C**H$_3$O), 41.5 (**C**H$_2$NH), 40.9 (N**C**H$_3$); HRMS (ESI) calcd. for $C_{18}H_{23}N_2O_2$ 299.1760. Found $[MH]^+$ 299.1751 (−3.01 ppm error).

5-Methyl-2-phenyl-3,4,5,4-tetrahydrobenzo[f] [1,4] diazocin-1(2H)-one (76)

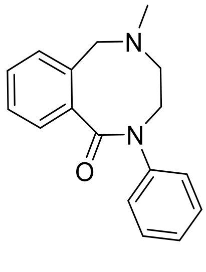

To a stirring solution of methyl methyl 2-((methyl(2-(phenylamino) ethyl) amino) methyl) benzoate (**S101**) (0.129 g, 0.433 mmol) in tetrahydrofuran (1.07 mL), aqueous lithium hydroxide (0.5 M) (1.07 mL, 0.563 mmol) was added and heated for at 50 °C for 3 hours. The solvent was removed under vacuum using dichloromethane (5 × 50.0 mL) to form an azeotropic mixture, to help ensure that all of the water from the hydrolysis was removed

ahead of the next step. The intermediate lithium (2-((2-carboxylatobenzyl) (methyl) amino) ethyl) (phenyl) amide was dissolved in chloroform (4.3 mL) and DIPEA (0.135 mL, 0.800 mmol) was added followed by T3P 50% w/v in ethyl acetate (0.420 g, 0.650 mmol) and stirred at room temperature for 3 hours under argon. The reaction mixture was then transferred to a separating funnel, brine (50.0 mL) was added and extracted with dichloromethane (2 × 100 mL). The combined organic phases were then dried with sodium sulphate, filtered, concentrated under vacuum and purified via flash column chromatography (10:9:1 hexane:ethylacetate:triethylamine) to afford the title compound **76** (0.0860 g, 75%) R_f = 0.30 (10:9:1 hexane:ethylacetate:triethylamine; ν_{max}/cm^{-1} (thin film) 2946, 2240, 1638, 1401, 1029, 727, 697; δ_H (400 MHz, $CDCl_3$) 7.52 (1H, dd, *J* = 7.5 Hz, 1.4 Hz, Ar**H**), 7.45−7.40 (2H, m, 2× Ar**H**), 7.39−7.36 (3H, m, 3 × Ar**H**), 7.33 (1H, dd, *J* = 7.5 Hz, 1.4 Hz, Ar**H**), 7.31−7.28 (1H, m, Ar**H**), 7.27−7.24 (1H, m, Ar**H**), 3.99 (1H, d, *J* = 14.5 Hz, ArC**H**$_a$H$_b$N), 3.88−3.81 (1H, m, C**H**$_a$H$_b$N), 3.67 (1H, d, *J* = 14.5 Hz, ArCH$_a$**H**$_b$N), 3.55−3.49 (1H, m, CH$_a$**H**$_b$N), 2.77−2.71 (1H, m, C**H**$_a$H$_b$N), 2.65−2.59 (1H, m, CH$_a$**H**$_b$N), 2.50 (3H, s, C**H**$_3$N); δ_C (101 MHz, $CDCl_3$) 171.7 (**C**O), 142.2 (Ar**C**), 136.9 (Ar**C**), 135.1 (Ar**C**), 130.4 (Ar**C**H), 129.6 (Ar**C**H), 129.5 (Ar**C**H), 129.0 (Ar**C**H), 127.9 (Ar**C**H), 127.8 (Ar**C**H), 127.2 (Ar**C**H), 59.5 (Ar**C**H$_2$N), 55.9 (**C**H$_2$N), 50.8 (**C**H$_2$N), 46.9 (N**C**H$_3$); HRMS (ESI) calcd. for $C_{17}H_{19}N_2O$ 267.1497. Found $[MH]^+$ 267.1491 (−2.3 ppm error).

Methyl 5-(ethyl(3-(ethylamino)propyl)amino)pentanoate (S102)

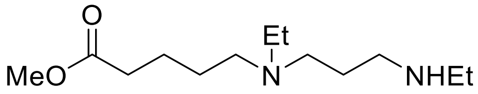

To a stirring mixture of N,N'-diethyl-1,3-propanediamine (0.260 g, 0.318 mL, 2.00 mmol) and potassium carbonate (0.691 g, 5.00 mmol) in acetonitrile (5.00 mL), 5-bromovaleric acid methyl ester (195 mg, 0.143 mL, 1.00 mmol) was added and heated at 60 °C for 4 hours. The reaction mixture was filtered through a pad of Celite®, washing with acetonitrile (50.0 mL), concentrated under vacuum and purified via flash column chromatography (55:40:5 ethyl acetate:methanol:triethylamine) to afford the title compound (0.203 mg, 83%) as a colourless oil, R_f = 0.29 (55:40:5 ethyl acetate:methanol:triethylamine); δ_H (400 MHz, $CDCl_3$) 3.65 (3H, s, OC**H**$_3$), 2.78−2.67 (4H, m, NC**H**$_2$), 2.55−2.46 (4H, m, NC**H**$_2$), 2.45−2.38 (2H, t, *J* = 7.4 Hz, NC**H**$_2$), 2.32 (2H, t, *J* = 7.4 Hz, MeO$_2$CC**H**$_2$), 1.77−1.66 (2H, m, C**H**$_2$), 1.65−1.55 (2H, tt, J = 7.4, 7.4 Hz, C**H**$_2$), 1.50−1.40 (2H, m, C**H**$_2$), 1.19-1.13 (3H, m, C**H**$_3$), 1.04-0.98 (3H, m, C**H**$_3$).

1,5-Diethyl-1,5-diazecan-6-one (77)

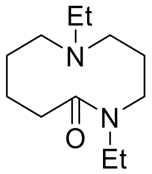

To a stirring solution of methyl 5-(ethyl(3-(ethylamino)propyl)amino)pentanoate (**S102**) (0.093 g, 0.381 mmol) in tetrahydrofuran (1.14 mL), aqueous lithium hydroxide (0.5 M) was added (1.14 mL, 0.571 mmol) and stirred at rt for 48 hours. The solvent was removed under vacuum, the intermediate lithium 5-(ethyl(3-(ethylamino)propyl)amino)pentanoate was dissolved in chloroform (9.50 mL) and DIPEA (0.123 mL, 0.704 mmol) was added followed by T3P 50% w/v in ethyl acetate (0.363 g, 0.571 mmol) and stirred at room temperature for 1 hour under argon. Silica (~0.5 g) was added to the reaction mixture, it was concentrated under vacuum and purified via flash column chromatography (65:30:5 hexane:diethylether:triethylamine) to afford the title compound (0.048 g, 59%) as a colourless oil, R_f = 0.23 (65:30:5 hexane:diethylether:triethylamine); δ_H (400 MHz, $CDCl_3$) 0.99 (3H, t, *J* = 7.0 Hz, CH_3), 1.10 (3H, t, *J* = 7.0 Hz, CH_3), 1.54–1.62 (3H, m, very broad), 1.68–3.10 (9H, m, very broad), 3.40–4.10 (3H, m, very broad); δ_C (101 MHz, $CDCl_3$) 12.2 (CH_3), 12.9 (CH_3), 23.0 (CH_2), 24.7 (CH_2), 27.1 (CH_2), 30.1 (CH_2), 39.5 (CH_2), 44.6 (CH_2), 45.3 (CH_2), 47.8 (CH_2), 54.8 (CH_2), 174.5 (C=O); HRMS (ESI) calcd. for $C_{12}H_{25}N_2O$ 213.1961. Found $[MH]^+$ 213.1963 (−1.40 ppm error).

***tert*-Butyl 4-([(benzyloxy)carbonyl] (methyl)amino) butanoate (S103)**

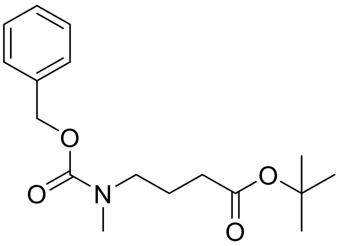

To a 250 mL round bottom flask of N-[(benzyloxy)carbonyl]-N-methylglycine (2.70 g, 12.0 mmol), 4CzIPN (0.095 g, 0.120 mmol) and dipotassium phosphate (2.51 g, 14.4 mmol) were added to DMF (30.0 mL) and degassed for 15 minutes using argon. Separately, *tert*-butyl acrylate (3.00 mL) was degassed again for 15 minutes using argon. *tert*-Butyl acrylate (1.54 g,

12.0 mmol) was then added to the 250 mL round bottom flask topped with a septum wrapped with parafilm several times. The reaction mixture was irradiated with blue LED light for 72 hours whilst stirring (setup pictured below). The reaction was quenched in saturated $NaHCO_3$ aqueous solution (100 mL) and extracted in Et_2O (3 × 50.0 mL). The organic layers were collected and washed with saturated NaCl aqueous solution (2 × 100 mL) and water (2 × 100 mL). The organic phases were collected dried with sodium sulphate, the solvent removed under vacuum and purification via flash column chromatography (15:4:1 hexane:ethyl acetate:triethylamine) afforded the title compound **S103** as a clear oil (2.10 g, 57%). In solution in $CDCl_3$, the compound exists as 2:1 mixture of rotamers; R_f = 0.32 (15:4:1 hexane:ethyl acetate:triethylamine); ν_{max}/cm^{-1} (thin film) 2977, 2934, 1724, 1702, 1455, 1366, 1145, 1055, 846, 768, 752, 698; δ_H (400 MHz, $CDCl_3$) 7.35−7.25 (5H, m, ArC**H**, both rotamers), 5.10 (s, 2H ArC**H**$_2$O, both rotamers), 3.30−3.26 (2H, m, NC**H**$_{2,}$ both rotamers), 2.89 (3H, s, NC**H**$_3$, major rotamer), 2.88 (3H, s, NC**H**$_3$, minor rotamer), 2.24−2.15 (2H, m, C**H**$_2$COO, both rotamers), 1.83−1.75 (2H, m, CH_2C**H**$_2$$CH_{2,}$ both rotamers), 1.43 (9H, s, OC(C**H**$_3$)$_3$, minor rotamer), 1.42 (9H, s, OC(C**H**$_3$)$_{3,}$, major rotamer); δ_c (101 MHz, $CDCl_3$) rotamers observed in a ≈1:1 ratio 174.4 & 172.4 (CH_3**C**OO), 156.4 & 156.2 (O**C**ON), 137.0 (Ar**C**), 128.6 (ArC**H**), 128.0 (ArC**H**), 127.9 (ArC**H**), 80.8 & 80.5 (O**C**(CH_3)$_3$), 67.1 (Ar**C**H_2O), 48.5 & 48.1 (N**C**H_2), 43.2 (**C**H_2CO), 34.8 (**C**H_2), 34.1 (**C**H_2), 33.1 & 32.6 (N**C**H_3), 28.2 & 28.1 (OC(**C**H_3)$_3$), 23.4 & 23.0 (CH_2**C**$H_2$$CH_2$); HRMS (ESI): calcd. for $C_{17}H_{26}NO_4$ 308.1862. Found $[MH]^+$ 308.1854 (−2.60 ppm error).

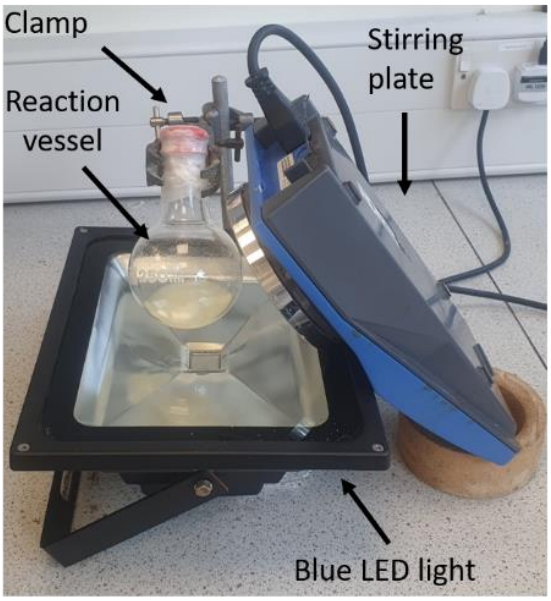

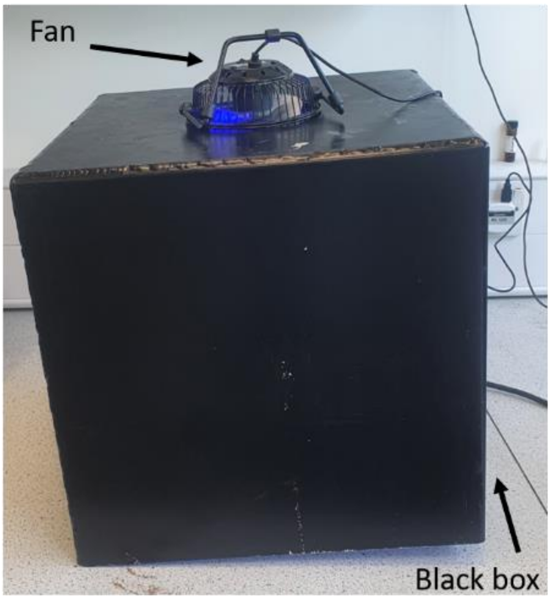

***tert*-Butyl 4-[(3-[(tert-butoxycarbonyl) amino] propyl) (methyl) amino] butanoate (S104)**

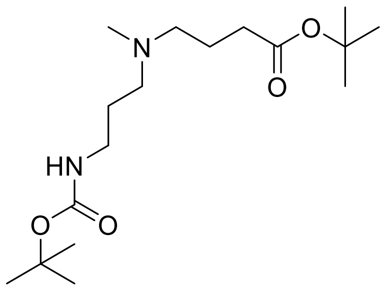

Palladium on carbon (0.202 g) was added to *tert*-butyl 4-[(benzyloxy)carbonyl] (methyl) amino) butanoate (**S103**) (2.02 g, 6.89 mmol) in ethanol (60.0 mL) under argon. The solution was evacuated and refilled with hydrogen (× 3) then stirred at room temperature overnight whilst excess hydrogen refills the reaction vessel. The reaction mixture was filtered through Celite and washed with ethanol (100 mL) and the solvent was removed under vacuum using acetonitrile (3 × 15.0 mL) to make an azeotropic mixture. The resulting crude product [*tert*-butyl 4-(methylamino) butanoate**]** was then dissolved in acetonitrile (30.0 mL) then potassium carbonate (1.91 g, 13.8 mmol) and 3-(Boc-amino) propyl bromide (1.64 g, 6.89 mmol) were added, and the mixture was refluxed overnight under argon. The reaction mixture was diluted with EtOAc (250.0 mL) and washed with saturated $NaHCO_3$ aqueous solution (200 mL) was then back extracted with EtOAc (150 mL × 2). The organic phases were collected, dried using sodium sulphate, filtered, the solvent removed under vacuum and purified via flash column chromatography (15:4:1 hexane:ethyl acetate:triethylamine) to afford the title compound **S104** as a clear viscous oil (0.432 g, 19%); ν_{max}/cm^{-1} (thin film) 3369, 2976, 2934, 2796, 1710, 1514, 1456, 1366, 1249, 1154, 1047, 847, 733; δ_H (400 MHz, $CDCl_3$) 5.44 (1H, bs, N**H**), 3.13−3.05 (2H, bm, C**H**$_2$NH), 2.33−2.27 (2H, m, C**H**$_2$N), 2.27−2.21 (2H, m, C**H**$_2$N), 2.19− 2.12 (2H, m, C**H**$_2$CO), 2.12−2.07 (3H, m, C**H**$_3$N), 1.71−1.61 (2H, m, C**H**$_2$), 1.57−1.48 (2H, m, C**H**$_2$), 1.35 (18H, bs, OC(C**H**$_3$)$_3$); δ_c (101 MHz, $CDCl_3$) 173.0 (CH_2**C**OO), 156.1 (NH**C**OO), 80.0 (O**C**(CH_3)$_3$), 78.7 ((O**C**(CH_3)$_3$), 56.9 (**C**H_2N), 56.5 (**C**H_2N), 41.8 (**C**H_3N), 39.9 (**C**H_2NH), 33.3 (**C**H_2COO), 28.5 (OC(**C**H_3)$_3$), 28.1 ((OC(**C**H_3)$_3$), 26.8 (**C**H_2), 22.7 (**C**H_2); HRMS (ESI): calcd. for $C_{17}H_{35}N_2O_4$ 331.2597. Found $[MH]^+$ 331.2595 (−0.60 ppm error).

1-Methyl-1,5-diazonan-4-one (78)

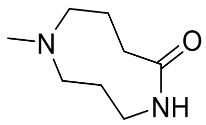

To a stirring solution of *tert*-butyl 4-[(3-[(*tert*-butoxycarbonyl) amino] propyl) (methyl) amino] butanoate (**S104**) (0.250 g, 0.757 mmol) in Et_2O (2.00 mL), HCl in 1,4-dioxane 4.00 M solution (3.80 mL) was added dropwise and left to stir for 4 hours at room temperature. The reaction mixture was concentrated under vacuum using chloroform (3 × 10.0 mL) to help remove all of the solvent via an azeotropic mixture. Chloroform was then added (19.0 mL), followed by DIPEA (1.32 mL, 7.60 mmol) and stirred until all the reagents dissolved into solution (30 minutes). T3P (50% w/v in ethyl acetate, 0.720 g, 1.14 mmol) was then added, and the reaction was left to stir over night at room temperature. The solvent was then removed under vacuum and the reaction mixture purified via flash column chromatography (10:9:1 dichloromethane:hexane:triethylamine) to afforded the title compound **78** as a white solid, (76.8 mg, 65%). In solution in $CDCl_3$, the compound exists as 4:1 mixture of rotamers; R_f = 0.28 (10:9:1 dichloromethane:hexane:triethylamine); ν_{max}/cm^{-1} (thin film) 3286, 2936, 2786, 1634, 1544, 1450, 1353, 1341, 1288, 1180, 1146, 1068, 969, 732, 580; δ_H (400 MHz, $CDCl_3$) 6.72–6.49$_{minor}$ (0.2H, bs, N**H**), 6.13–5.84$_{major}$ (0.8H, bs N**H**), 3.94–3.80 (bm, 0.25H, C**H**$_2$NH), 3.49–3.37 (bm, 1.5H, CH_2NH), 2.99–2.89 (bm, 0.25H, C**H**$_a$H$_b$NH), 2.50–2.40 (1H, m, CH$_a$**H**$_b$N), 2.50–2.40 (2H, m, C**H**$_2$N), 2.50–2.40 (2H, m, C**H**$_2$), 2.23 (3H, s, C**H**$_3$N), 1.86–1.79 (2H, m, C**H**$_2$CO), 1.49 (2H, pen, *J* = 6.5 Hz, C**H**$_2$); δ_c (101 MHz, $CDCl_3$) 179.8$_{minor}$ (**C**O), 178.6$_{major}$ (**C**O), 56.2$_{minor}$ (**C**H$_2$N), 54.8$_{major}$ (**C**H$_2$N), 52.2$_{minor}$ (**C**H$_2$N), 50.3 (**C**H$_2$N), 43.5$_{minor}$ (**C**H$_3$N), 42.3$_{major}$ (**C**H$_3$N), 41.5$_{minor}$ (**C**H$_2$N), 40.5$_{major}$ (**C**H$_2$NCO), 37.1$_{minor}$ (**C**H$_2$NCO), 28.5$_{major}$ (**C**H$_2$), 27.8$_{major}$ (**C**H$_2$), 24.8$_{major}$ (**C**H$_2$CO), 24.5$_{minor}$ (**C**H$_2$), 24.4$_{minor}$ (**C**H$_2$); HRMS (ESI): calcd. for $C_8H_{17}N_2O$ 157.1341. Found $[MH]^+$ 157.1336 (–3.18 ppm error).

***tert*-Butyl 2-((benzylamino)methyl)piperidine-1-carboxylate (S105)**

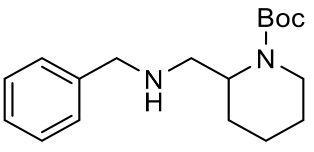

To a solution of 1-Boc-2-aminomethylpiperidine (1.00 mL, 1.01 g, 4.71 mmol) in methanol (25 mL) was added benzaldehyde (0.570 mL, 595 mg, 5.60 mmol) and the resulting solution was

stirred at RT for 6 h. Sodium borohydride (533 mg, 14.1 mmol) was added in portions and then stirred at RT for 30 mins. The reaction mixture was concentrated *in vacuo*. Water (25 mL) was added and the product was extracted with DCM (3 x 25 mL). The combined organic layers were dried over $MgSO_4$, filtered and concentrated *in vacuo*. The crude product was purified by column chromatography (SiO_2, 13:6:1 hexane:ethyl acetate:triethylamine) to yield the title compound (1.15 g, 80%) as a yellow oil. R_f 0.52 (13:6:1 hexane:ethyl acetate:triethylamine); δ_H (400 MHz, $CDCl_3$) 7.37–7.21 (5H, m, ArH), 4.42–4.31 (1H, m, NCH), 4.02–3.92 (1H, m, NC**H**H'), 3.85 (1H, d, *J* = 13.5 Hz, PhC**H**H'), 3.78 (1H, d, *J* = 13.5 Hz, PhCH**H'**), 2.90 (1H, dd, *J* = 12.0, 8.5 Hz, NC**H**H'), 2.74–2.60 (2H, m, 2 x NCH**H'**), 1.71–1.36 (15H, m, $C(CH_3)_3$, CHH'). Characterisation data matched those reported in the literature.[11]

tert-Butyl 2-((benzyl(2-(methoxycarbonyl)benzyl)amino)methyl)piperidine-1-carboxylate (S106)

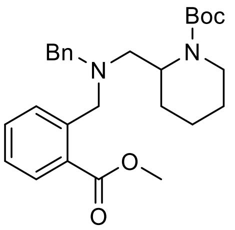

tert-Butyl 2-((benzylamino)methyl)piperidine-1-carboxylate **S105** (687 mg, 2.26 mmol) was dissolved in DCM (4.6 mL) and to this was added methyl 2-(bromomethyl)benzoate (641 mg, 2.80 mmol) and potassium carbonate (567 mg, 4.10 mmol). The resulting mixture was stirred at RT for 22 h. The reaction mixture was diluted with DCM (20 mL), washed with water (25 mL) and brine (2 x 25 mL). The organic layer was dried over $MgSO_4$, filtered and concentrated *in vacuo*. The crude product was purified by column chromatography (SiO_2, 9:1 hexane:ethyl acetate) to yield the title compound **S106** (770 mg, 74%) as a yellow oil. R_f 0.29 (9:1 hexane:ethyl acetate); ν_{max}/cm^{-1} (thin film) 2932, 1723, 1686, 1450, 1415, 1364, 1267, 1172, 1148, 1076, 1045, 741, 700; δ_H (400 MHz, $CDCl_3$) 7.80–7.72 (2H, m, ArH), 7.46 (1H, t, *J* = 7.5 Hz, ArH), 7.36–7.19 (6H, m, ArH), 4.59–4.26 (1H, m, CHH'), 4.17–4.05 (1H, m, CHH'), 3.96–3.77 (5H, m, OCH_3, CHH'), 3.71–3.36 (2H, m, CHH'), 2.67–2.29 (3H, m, CH, CHH'), 1.79–1.18 (14H, m, $C(CH_3)_3$, CHH'), 0.94–0.79 (1H, m, CHH'); δ_C (101 MHz, $CDCl_3$) 168.5 (**C**O_2Me), 155.0 (NCO_2), 141.2 (ArC), 139.3 (ArC), 131.5 (ArC), 130.8 (ArC), 130.3 (ArC), 129.9 (ArC), 129.2 (ArC), 128.1 (ArC), 126.9 (ArC), 126.6 (ArC), 79.2 (**C**$(CH_3)_3$), 58.7 (Ar**C**H_2), 56.3 (Ar**C**H_2), 52.6 (NCH_2), 52.0

(OCH_3), 39.3 (CH), 29.7 (CH_2), 28.6 (C(**C**H_3)$_3$), 26.2 (CH_2), 25.3 (CH_2), 18.7 (CH_2); HRMS (ESI); calcd. for $C_{27}H_{37}N_2O_4$, 453.2784. Found: $[MH]^+$, 453.2745 (0.5 error ppm).

Methyl 2-((benzyl(piperidin-2-ylmethyl)amino)methyl)benzoate (S107)

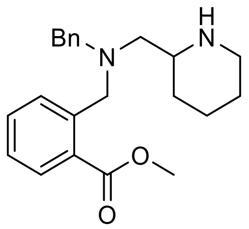

tert-Butyl 2-((benzyl(2-(methoxycarbonyl)benzyl)amino)methyl)piperidine-1-carboxylate **S106** (870 mg, 1.92 mmol) was dissolved in 1,4-dioxane (7.3 mL) and methanol (7.3 mL). To this was added HCl (7.30 mL, 4 M in dioxane, 29.2 mmol) and the reaction was stirred at RT for 18 h. The solution was diluted with ethyl acetate (100 mL) and neutralised using 2 M $NaOH_{(aq)}$. The aqueous layer was extracted with ethyl acetate (3 x 100 mL) and the combined organic layers were dried over $MgSO_4$, filtered and concentrated *in vacuo*. The crude product was purified by column chromatography (SiO_2, 19:1 ethyl acetate:triethylamine) to yield the title compound **S107** (616 mg, 92%) as a yellow oil. R_f 0.45 (19:1 ethyl acetate:triethylamine); ν_{max}/cm^{-1} (thin film) 2929, 2799, 1721, 1601, 1494, 1434, 1265, 1125, 1080, 967, 739, 698; δ_H (400 MHz, $CDCl_3$) 7.76 (1H, dd, *J* = 7.5, 1.5 Hz, ArH), 7.64 (1H, d, *J* = 8.0 Hz, ArH), 7.46 (1H, td, *J* = 7.5, 1.5 Hz, ArH), 7.31–7.19 (6H, m, ArH), 4.06 (1H, d, *J* = 14.5 Hz, ArC**H**H'), 3.89 (3H, s, CH_3), 3.83 (1H, d, *J* = 14.5 Hz, ArCH**H'**), 3.62 (1H, d, *J* = 13.5 Hz, PhC**H**H'), 3.48 (1H, d, *J* = 13.5 Hz, PhCH**H'**), 2.99–2.93 (1H, m, HNC**H**H'), 2.54–2.28 (5H, m, HNCH**H'**, NC**H**H'CH, NCH**H'**CH, CH, NH), 1.74–1.66 (1H, m, CHH'), 1.55–1.48 (2H, m, CHH'), 1.45–1.34 (1H, m, CHH'), 1.25–1.16 (1H, m, CHH'), 1.00–0.88 (1H, m, CHH'); δ_C (101 MHz, $CDCl_3$) 168.7 (CO_2Me), 141.0 (ArC), 139.1 (ArC), 131.5 (ArC), 130.9 (ArC), 130.2 (ArC), 130.0 (ArC), 129.0 (ArC), 128.2 (ArC), 127.0 (ArC), 126.8 (ArC), 61.6 (N**C**H_2CH), 59.9 (Ph**C**H_2), 57.3 (Ar**C**H_2), 54.6 (CH), 52.0 (CH_3), 46.8 ($HNCH_2$), 30.8 (CH_2), 26.3 (CH_2), 24.8 (CH_2); HRMS (ESI): calcd. for $C_{22}H_{29}N_2O_2$, 353.2224. Found: $[MH]^+$, 353.2222 (0.4 error ppm).

2-((Benzyl(piperidin-2-ylmethyl)amino)methyl)benzoic acid (S108)

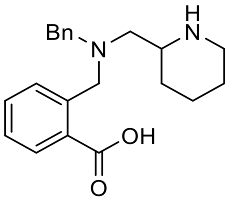

To a solution of methyl 2-((benzyl(piperidin-2-ylmethyl)amino)methyl)benzoate **S107** (78 mg, 0.220 mmol) in THF (0.44 mL) was added lithium hydroxide (0.48 mL, 0.5 M in water, 0.24 mmol) and the mixture was heated to reflux for 22 h. The reaction mixture was concentrated *in vacuo* and purified by column chromatography (SiO_2, 15:4:1 ethyl acetate:methanol:triethylamine) to yield the title compound **S108** (67 mg, 90%) as a white solid. R_f 0.25 (15:4:1 ethyl acetate:methanol:triethylamine); m.p. 147–152 °C; ν_{max}/cm^{-1} (thin film) 3397, 2942, 2811, 1619, 1582, 1549, 1447, 1390, 909, 750, 729, 661; δ_H (400 MHz, $CDCl_3$) 7.64 (1H, dd, *J* = 7.0, 2.0 Hz, ArH), 7.31–7.12 (8H, m, ArH), 4.32 (1H, d, *J* = 12.5 Hz, ArCHH'), 3.67 (1H, d, *J* = 14.0 Hz, ArCHH'), 3.59–3.32 (3H, m, 2 x ArCHH', CHH'), 3.22–3.01 (3H, m, CHH', CH), 2.73 (1H, t, *J* = 12.0 Hz, CHH'), 2.40–2.32 (1H, m, CHH'), 1.86–1.53 (5H, m, CHH', NH), 1.40–1.30 (1H, m, CHH'); δ_C (101 MHz, $CDCl_3$) 176.9 (CO_2H), 139.4 (ArC), 136.3 (ArC), 135.1 (ArC), 130.7 (ArC), 129.9 (ArC), 128.8 (ArC), 128.4 (ArC), 128.2 (ArC), 127.7 (ArC), 127.2 (ArC), 58.2 ($ArCH_2$), 56.7 ($ArCH_2$), 53.6 (CH), 45.7 (CH_2), 43.4 (CH_2), 26.6 (CH_2), 22.4 (CH_2), 22.1 (CH_2); HRMS (ESI); calcd. for $C_{21}H_{27}N_2O_2$, 339.2067. Found: $[MH]^+$, 339.2063 (1.2 error ppm).

6-Benzyl-5,6,7,7a,8,9,10,11-octahydro-13H-benzo[f]pyrido[1,2-a][1,4]diazocin-13-one (79)

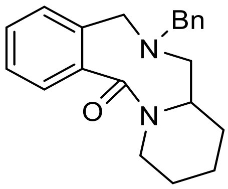

A round-bottom flask was charged with 2-((benzyl(piperidin-2-ylmethyl)amino)methyl)benzoic acid **S108** (65 mg, 0.192 mmol) and DMF (1.9 mL). To this was added DIPEA (0.170 mL, 1.00 mmol), EDC·HCl (58 mg, 0.303 mmol) and HOBt (41 mg, 0.303 mmol). The resulting mixture was stirred at room temperature for 18 h. The reaction mixture was diluted with ethyl acetate (20 mL) and then washed with brine (3 x 20 mL). The combined aqueous layers were then extracted with ethyl acetate (2 x 20 mL). The combined organic layers were then dried over magnesium sulfate, filtered and concentrated *in vacuo*. The crude

product was purified by column chromatography (SiO_2, 15:4:1 hexane:ethyl acetate:triethylamine) to yield the title compound **79** (50 mg, 82%) as a colourless oil. R_f 0.38 (15:4:1 hexane:ethyl acetate:triethylamine); ν_{max}/cm^{-1} (thin film) 2925, 2853, 1624, 1450, 1408, 1370, 1323, 1278, 1132, 1098, 1018, 909, 730, 699, 646; δ_H (400 MHz, $CDCl_3$) 7.43–7.30 (5H, m, ArH), 7.28–7.21 (3H, m, ArH), 6.99–6.94 (1H, m, ArH), 4.47 (1H, d, *J* = 13.5 Hz, C**H**H'NCO), 4.01 (1H, d, *J* = 16.5 Hz, Ar^{1}C**H**H'), 3.94–3.88 (1H, m, NCH), 3.84 (1H, d, *J* = 13.5 Hz, Ar^{2}C**H**H'), 3.73 (1H, d, *J* = 13.5 Hz, Ar^{2}CH**H'**), 3.64 (1H, d, *J* = 16.5 Hz, Ar^{1}CH**H'**), 3.13 (1H, td, *J* = 12.0, 1.5 Hz, NC**H**H'CH), 2.57 (1H, td, *J* = 13.5, 3.5 Hz, CH**H'**NCO), 2.32 (1H, dd, *J* = 12.0, 3.5 Hz, NCH**H'**CH), 1.75–1.68 (1H, m, CHH'), 1.65–1.52 (2H, m, CHH'), 1.49–1.29 (3H, m, CHH'); δ_C (101 MHz, $CDCl_3$) 173.5 (CO), 138.7 (ArC), 138.1 (ArC), 135.0 (ArC), 129.1 (2 x ArC), 128.8 (ArC), 128.5 (ArC), 127.4 (ArC), 127.0 (ArC), 126.8 (ArC), 63.3 ($ArCH_2$), 59.1 ($ArCH_2$), 57.0 (N**C**H_2CH), 51.5 (CH), 37.8 (**C**H_2NCO), 27.3 (CH_2), 24.5 (CH_2), 19.1 (CH_2); HRMS (ESI); calcd. for $C_{21}H_{25}N_2O^+$, 321.1961. Found: $[MH]^+$, 321.1960 (0.6 error ppm).

2-[(Benzyloxy)carbonyl]-1,2,3,4-tetrahydroisoquinoline-3-carboxylic acid (S109)

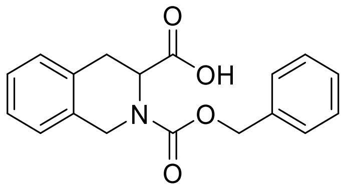

To a stirring solution of 1,2,3,4-tetrahydroisoquinoline-3-carboxylic acid (5.00 g, 28.2 mmol) in THF (50.0 mL) and deionised water (150 mL), solid sodium hydroxide (2.26 g, 56.4 mmol) was added, and the reaction mixture cooled in an ice bath. Benzoyl chloroformate (5.77 g, 33.8 mmol) was added dropwise over an hour and left stirring at room temperature overnight. The reaction mixture was cooled in an ice bath and 10.0% HCl (approximately 70.0 mL) solution was added dropwise until the reaction mixture reached pH ≈ 2 tested by pH indicator sticks. The solution was extracted in ethyl acetate (80.0 mL × 3) and the combined organic phases were washed with brine (100 mL × 3). The aqueous phase was then back extracted with ethyl acetate (100 mL × 2). The organic phases were combined, dried with sodium sulphate, filtered and the solvent removed under vacuum. The reaction mixture was purified via flash column chromatography (8:1 dichloromethane:methanol) to form the title compound **S109** as a very viscous orange oil (5.71 g, 65%). In solution in $CDCl_3$, the compound exists as a ≈4:3 mixture, evident in its ^{13}C NMR data; R_f = 0.27 (8:1 dichloromethane:methanol); ν_{max}/cm^{-1} (thin film) 3439, 3031, 2968, 2579, 1697, 1419, 1318,

1220, 1122, 747, 698; δ_H (400 MHz, $CDCl_3$) 10.80 (1H, bs, COO**H**), 7.39–7.06 (m, 9H, Ar**H**), 5.26–5.19 (2H, m, OC**H**$_2$), 5.26–5.19 (0.5H, m, C**H**), 4.96 (0.5H, t, *J* = 5.1 Hz, C**H**), 4.82–4.76 (1H, m, C**H**$_a$H$_b$N), 4.66–4.54 (1H, m, CH$_a$**H**$_b$N), 3.30–3.14 (2H, m, C**H**$_2$CH); δ_C (101 MHz, $CDCl_3$) rotameric ratio 3:4 176.9$_{minor}$ & 176.7$_{major}$ (N**C**O), 156.5$_{major}$ & 155.7$_{minor}$ (**C**OOH), 136.4$_{major}$ & 136.3$_{minor}$ (Ar**C**), 133.0$_{minor}$ & 132.3$_{major}$ (Ar**C**), 131.5 (Ar**C**), 128.7$_{major}$ & 128.6$_{minor}$ (Ar**C**H), 128.4 (ArCH), 128.3 (ArCH), 128.2$_{major}$ & 128.1$_{minor}$ (Ar**C**H), 127.1 (Ar**C**H), 127.2$_{minor}$ & 127.0$_{major}$ (ArCH), 126.6$_{minor}$ & 126.4$_{major}$ (Ar**C**H), 68.0$_{major}$ & 67.9$_{minor}$ (O**C**H$_2$), 53.6$_{minor}$ & 53.1$_{major}$ (**C**H), 44.6$_{minor}$ & 44.4$_{major}$ (**C**H$_2$N), 31.3$_{minor}$ & 30.9$_{major}$ (**C**H$_2$); HRMS (ESI): calcd. for $C_{18}H_{18}NO_4$ 334.1055. Found [MNa]$^+$ 334.1051 (−0.32 ppm error).

Benzyl 3-(3-*tert*-butoxy-3-oxopropyl)-3,4-dihydroisoquinoline-2(1H)-carboxylate (S110)

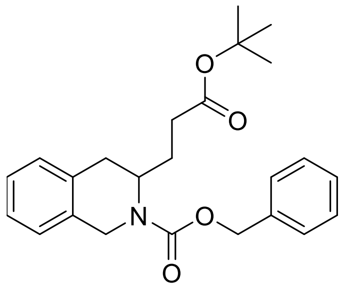

To a 250 mL round bottom flask Isolating 2-[(benzyloxy)carbonyl]-1,2,3,4-tetrahydroisoquinoline-3-carboxylic acid **S109** (3.74 g, 12.0 mmol), IrdF(CF)$_3$ppy)$_2$(dtbbpy)]PF$_6$ (0.135 g, 0.120 mmol) and dipotassium phosphate (2.51 g, 14.4 mmol) was added to DMF (30.0 mL) and degassed for 15 minutes using argon. Separately *tert*-butyl acrylate (3.00 mL) was degassed again for 15 minutes using argon. *tert*-Butyl acrylate (12.0 mmol, 1.54 g) was then added to the 250 mL round bottom flask topped with a septum wrapped with parafilm several times. The reaction mixture was irradiated with blue LED light for 72 hours whilst stirring, setup as shown below. The reaction was quenched in saturated $NaHCO_3$ aqueous solution (100 mL) and extracted in Et_2O (3 × 50.0 mL). The organic layers were collected and washed with saturated NaCl aqueous solution (2 × 100 mL) and water (2 × 100 mL). The organic phases were collected and dried with sodium sulphate and the solvent removed under vacuum. The reaction mixture was purified via flash column chromatography (8:2 hexane:ethyl acetate) to afford the title compound **S110** as a clear yellow oil (2.36 g, 50%); R_f = 0.36 (8:2 hexane:ethyl acetate); ν_{max}/cm^{-1} (thin film) 2976, 2933, 1725, 1695, 1416, 1242, 1144, 846, 749, 697; δ_H (400 MHz, $CDCl_3$) rotameric ratio 1:1 7.41–7.29 (5H, m, Ar**H**),

7.19−7.02 (4H, m, Ar**H**), 5.18 (2H, s, OC**H**$_2$Ar), 5.05−4.87 (1H, m, ArC**H**$_a$H$_b$N), 4.68−4.51 (1H, m, C**H**N), 4.34−4.21 (1H, m, CH$_a$**H$_b$**), 3.15−3.06 (1H, m, C**H$_a$**H$_b$CO), 2.69−2.60 (1H, m, CH$_a$**H$_b$**CO), 2.34−2.15 (2H, m, ArC**H**$_2$CH), 1.83−1.70 (1H, m, C**H$_a$**H$_b$), 1.69−1.53 (1H, m, CH$_a$**H$_b$**), 1.43 (9H, s, OC(C**H**$_3$)$_3$); δ_C (101 MHz, CDCl$_3$) rotomeric ratio 1:1 172.5 & 172.4 (N**C**O), 155.8 & 155.7 (O**C**O), 136.80 (Ar**C**), 132.6 & 132.4 (Ar**C**), 132.2 & 132.1 (Ar**C**), 129.5 & 129.3 (Ar**C**H), 128.7 (Ar**C**H), 128.2 (Ar**C**H), 128.1 (Ar**C**H), 126.8 (Ar**C**H), 126.4 (Ar**C**H), 126.3 & 126.1 (Ar**C**H), 80.5 (O**C**(CH$_3$)$_3$), 67.4 (Ar**C**H$_2$O), 49.5 & 49.2 (N**C**H), 42.6 & 42.5 (**C**H$_2$N), 33.5 & 33.1 (**C**H$_2$), 32.5 & 32.3 (**C**H$_2$), 28.2 (OC(**C**H$_3$)$_3$), 27.1 (**C**H$_2$); HRMS (ESI): calcd. for $C_{24}H_{29}NNaO_4$ 418.1994. Found [MNa]$^+$ 418.1994 (0.00 ppm error).

The same product was also made via the procedure above, but IrdF(CF)$_3$ppy)$_2$(dtbbpy)]PF$_6$ was replaced with 2,4,5,4-tetrakis(carbazol-9-yl)-1,3-dicyanobenze (4CzIPN) (0.095 g, 0.120 mmol) to afford the title compound as a clear yellow oil (2.42 g, 50%).

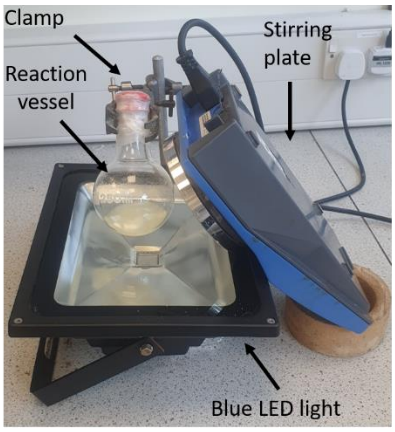

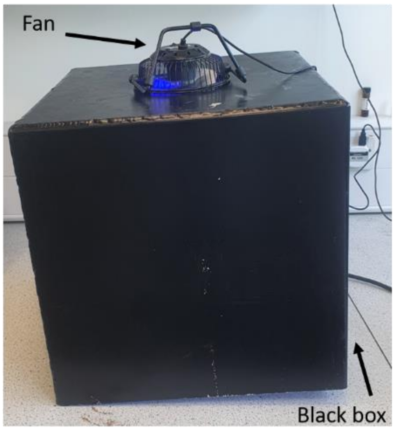

***tert*-Butyl 3-(2-(3-[(*tert*-butoxycarbonyl) amino] propyl)-1,2,3,4-tetrahydroisoquinolin-3-yl) propanoate (S111)**

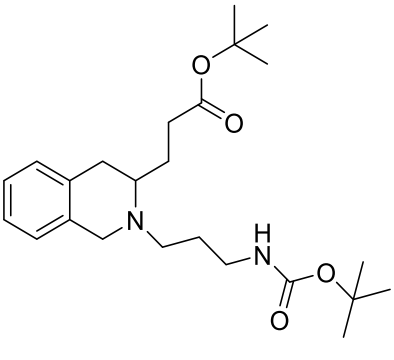

Palladium on carbon (0.230 g) was added to benzyl 3-(3-*tert*-butoxy-3-oxopropyl)-3,4-dihydroisoquinoline-2(1H)-carboxylate **S110** (2.98 g, 7.54 mmol) in ethanol (30.0 mL) under argon. The solution was evacuated and refilled with hydrogen (× 3) then stirred at room

temperature overnight whilst excess hydrogen refills the reaction vessel. The mixture was filtered through Celite and washed with ethanol (100 mL) and the solvent was removed under vacuum using acetonitrile (3 × 15.0 mL) to make an azeotropic mixture. The mixture was redissolved in acetonitrile (30.0 mL) then potassium carbonate (2.08 g, 15.1 mmol) and 3-(Boc-amino) propyl bromide (1.80 g, 7.54 mmol) was added and refluxed overnight under argon. The reaction mixture was diluted with EtOAc (250 mL) and washed with saturated $NaHCO_3$ aqueous solution (200 mL) then back extracted with EtOAc (2 × 150 mL). The organic phases were collected and dried using sodium sulphate, filtered and the solvent removed under vacuum. The reaction mixture was purified via flash column chromatography (15:2:1 hexane:ethyl acetate:triethylamine) to afford the title compound **S111** as a colourless oil (1.23 g, 39%); R_f = 0.53 (15:2:1 hexane:ethyl acetate:triethylamine); ν_{max}/cm^{-1} (thin film) 3369, 2976, 2932, 2249, 1709, 1504, 1454, 1391, 1365, 1248, 1149, 735; δ_H (400 MHz, $CDCl_3$) 7.11–6.96 (4H, m, ArC**H**), 5.46 (1H, bs, N**H**), 3.80–3.67 (2H, m, NC**H**$_2$), 3.22–3.13 (2H, m, C**H**$_2$NH), 2.99–2.89 (1H, m, C**H**N), 2.99–2.89 (1H, m, ArC**H$_a$**H$_b$), 2.65–2.48 (1H, m, ArCH$_a$**H$_b$**), 2.65–2.48 (2H, m, NC**H**$_2$), 2.29 (2H, t, *J* = 7.65 Hz, C**H**$_2$CO), 1.88–1.79 (1H, m, CHC**H$_a$**H$_b$), 1.69–1.61 (2H, m, CH$_2$C**H**$_2$CH$_2$), 1.59–1.50 (1H, m, CHCH$_a$**H$_b$**), 1.41 (9H, s, OC(C**H**$_3$)$_3$), 1.39 (9H, bs, OC(C**H**$_3$)$_3$); δ_C (101 MHz, $CDCl_3$) 173.1 (CH$_2$**C**O), 156.1 (NH**C**O), 134.0 (Ar**C**), 133.6 (Ar**C**), 129.3 (Ar**C**H), 126.8 (Ar**C**H), 126.2 (Ar**C**H), 125.9 (Ar**C**H), 80.3 (O**C**(CH$_3$)$_3$), 78.8 (O**C**(CH$_3$)$_3$), 56.3 (**C**HN), 50.4 (Ar**C**H$_2$N) 50.1 (**C**H$_2$N), 39.9 (**C**H$_2$NH), 32.6 (**C**H$_2$CO), 30.2 (ArC**H**$_2$), 28.5 (OC(**C**H$_3$)$_3$), 28.2 (OC(**C**H$_3$)$_3$), 27.1 (**C**H$_2$), 24.8 (**C**H$_2$); HRMS (ESI): calcd. for $C_{24}H_{39}N_2O_4$ 419.2910. Found $[MH]^+$ 419.2912 (0.48 ppm error).

1,4,5,6,7,9,14,14a-octahydro [1,5] diazonino[1,9-b] isoquinolin-3(2H)-one (80)

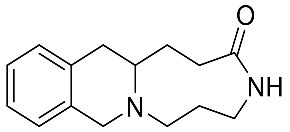

To a stirring solution of *tert*-butyl 3-(2-(3-[(*tert*-butoxycarbonyl) amino] propyl)-1,2,3,4-tetrahydroisoquinolin-3-yl) propanoate (**S111**) (0.155 g, 0.370 mmol) in diethyl ether (1.00 mL), a solution of 4.0 M hydrochloric acid in 1,4 dioxane (1.85 mL, 7.2 mmol) was added dropwise and the reaction was left stirring at room temperature for 3 hours. The reaction mixture was concentrated under vacuum using chloroform (3 × 10 mL) to make an azeotropic solution. The intermediate 3-[2-(3-aminopropyl)-1,2,3,4-tetrahydroisoquinolin-3-yl]

propanoic acid was dissolved in chloroform (13 mL) and DIPEA (0.644 mL, 3.70 mmol) under argon, until all the reagents dissolved into solution (30 minutes). T3P (50% w/v in ethyl acetate, 0.353 g, 0.56 mmol) was then added and the reaction left to stir over night at room temperature. The solvent was removed under vacuum and the reaction mixture was purified via flash column chromatography (70:25:5 dichloromethane:hexane:triethylamine) forming the title compound **80** as a clear oil (75.1 mg, 83%); R_f = 0.47 (70:25:5 dichloromethane:hexane:triethylamine); ν_{max}/cm^{-1} (thin film) 3281, 3206, 2928, 2841, 2225 1646, 1473, 1449 1127, 908, 725; δ_H (400 MHz, $CDCl_3$) significant rotameric broadening of NMR signals was observed 7.12−7.00 (4H, m, Ar**H**), 6.36 (1H, t, *J* = 6.68 Hz, CON**H**), 4.08−3.85 (1H, bm, C**H**$_a$H$_b$N), 3.79−3.69 (2H, bs, ArC**H**$_2$N), 3.48−3.23 (1H, bm, C**H**$_a$H$_b$N), 2.98 (1H, q, *J* = 7.11 Hz, C**H**N), 2.57 (2H, d, *J* = 7.06 Hz, ArC**H**$_2$CH), 2.50−2.28 (2H, m, C**H**$_2$CO), 2.28−2.16 (1H, m, C**H**$_a$H$_b$N), 1.79−1.66 (1H, m, C**H$_a$H$_b$**N), 1.57−1.44 (2H, bm, C**H**$_2$); δ_C (101 MHz, $CDCl_3$) 179.2 (**C**O), 135.1 (Ar**C**), 134.9 (Ar**C**), 128.9 & 128.7 (ArCH), 126.8 (Ar**C**H), 126.2 (Ar**C**H), 125.9 (Ar**C**H), 57.0 (**C**HN), 53.9 (Ar**C**H$_2$N), 41.6 (**C**H$_2$N), 40.1 (**C**H$_2$NH), 31.7 (**C**H$_2$CO), 30.5 (Ar**C**H$_2$CH), 28.1 (C**H**$_2$), 25.7 (C**H**$_2$); HRMS (ESI): calcd for $C_{15}H_{21}N_2O$ 245.1654. Found $[MH]^+$ 245.1646 (−3.26 ppm error).

Ethyl 4-((2-methoxyphenyl)amino)butanoate (S112)

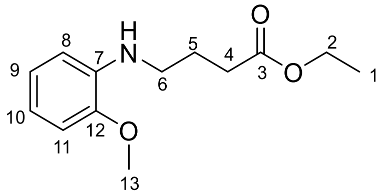

2-Methoxyaniline (2.05 mL, 18.2 mmol) was added via syringe over a period of 30 seconds to a pale brown suspension of ethyl 4-bromobutanoate (1.10 mL, 6.99 mmol) and anhydrous $NaOAc_{(s)}$ (860 mg, 10.5 mmol) in *n*-Butanol (24.0 mL) at RT. An immediate colour change to an orange solution was noted. The resulting solution was stirred at 70 °C overnight under Ar and progress of the reaction was monitored by TLC. After total of 18 h, the reaction was judged to be complete, based on TLC analysis. The resulting mixture was diluted with H_2O (30 mL), before transferred into separating funnel. The aqueous phase was extracted with Et_2O (3 × 40 mL), before the combined organic phases were washed sequentially with H_2O (1 × 30 mL) and sat. brine (3 × 20 mL). The combined organic layers were dried over $MgSO_4$, filtered

and concentrated under reduced pressure to yield a brown oil (3.92 g). The crude product was purified by flash column chromatography (SiO_2, 70 mm column, eluent: EtOAc:*n*–hexane, 30:70) to afford ester **S112** as a pale yellow oil (1.47 g, 89%). R_f = 0.58 (EtOAc:*n*–hexane, 30:70); IR (neat) ν_{max} / cm^{-1}: 3420wbr (N–H aniline), 2939w (C–H alkyl), 2835w (C–H alkyl), 1729s (C=O ester), 1602s (CC aromatic), 1513s (CC aromatic), 1456m, 1431m, 1373m, 1346w, 1301w, 1245s, 1220s, 1175s, 1125m, 1095w, 1048w, 1026s, 901w, 858w, 775w, 734s, 633w, 582w, 458w; δ_H (400 MHz; $CDCl_3$) 6.89 (1H, td, *J* = 7.8, 1.4 Hz, C(10)H), 6.78 (1H, dd, *J* = 7.8, 1.4 Hz, C(8)H), 6.70 – 6.60 (2H, m, C(9+11)H), 4.27 (1H, br, s, NH), 4.16 (2H, q, *J* = 7.2 Hz, C(2)H_2), 3.85 (3H, s, C(13)H_3), 3.21 (2H, t, *J* = 7.0 Hz, C(6)H_2), 2.45 (2H, t, *J* = 7.3 Hz, C(4)H_2), 2.05 – 1.95 (2H, m, C(5)H_2), 1.28 (2H, t, *J* = 7.2 Hz, C(1)H_3); δ_C (101 MHz; $CDCl_3$) 173.4 (C, C3), 146.8 (C, C12), 138.1 (C, C7), 121.3 (CH, C10), 116.4 (CH, C9), 109.7 (CH, C11), 109.4 (CH, C8), 60.5 (CH_2, C2), 55.4 (CH_3, C13), 43.0 (CH_2, C6), 32.0 (CH_2, C4), 24.8 (CH_2, C5), 14.3 (CH_3, C1); HRMS (ESI): calcd. for $C_{13}H_{20}NO_3$, 238.1438. Found: $[MH]^+$, 238.1441 (−1.5 ppm error), calcd. for $C_{13}H_{19}NNaO_3$, 260.1257. Found: $[MNa]^+$, 260.1262 (−2.0 ppm error).

Ethyl 4-((2-methoxyphenyl)(methyl)amino)butanoate (S113)

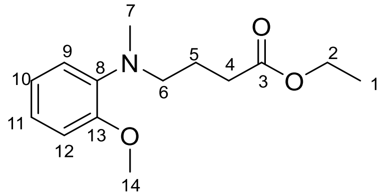

MeI (350 µL, 5.67 mmol) was added to a pale yellow suspension of ethyl 4-((2-methoxyphenyl)amino)butanoate **S112** (1.35 g, 5.67 mmol) and NaOAc (465 mg, 5.67 mmol) in anhydrous THF (19.0 mL) under Ar at RT. The resulting fluorescent yellow suspension was stirred at 50 °C for 16 h under Ar, after which time the reaction was deemed to have gone to completion by TLC. The reaction mixture was quenched with 30 mL of H_2O and poured into separating funnel. The resulting aqueous phase was extracted with Et_2O (3 × 30 mL), before the combined organic phases were washed sequentially with H_2O (1 × 30 mL) and sat. brine (3 × 20 mL). The combined organic layers were dried over $MgSO_4$, filtered and concentrated under reduced pressure to yield a yellow oil (2.11 g). The crude product was purified by flash column chromatography (SiO_2, 60 mm column, eluent: EtOAc:*n*–hexane, 20:80 to 30:70) to afford the title compound **S113** as a pale yellow liquid (207 mg, 14%). R_f = 0.35 (30:70

EtOAc:*n*–hexane); IR (neat) ν_{max} / cm^{-1}: 2940w (C–H alkyl), 2835w (C–H alkyl), 1731s (C=O ester), 1594m (CC aromatic), 1500s (CC aromatic), 1456m, 1420m, 1372m, 1347w, 1299w 1236s, 1179s, 1162s, 1115m, 1100m, 1054m, 1027s, 958w, 915w, 856w, 803w, 742s, 596w, 527w, 486w; δ_H (400 MHz; $CDCl_3$) 7.00 – 6.80 (4H, m, C(9 + 10 + 11 + 12), 4.11 (2H, q, *J* = 7.1 Hz, C(2)H_2), 3.85 (3H, s, C(14)H_3), 3.10 – 3.02 (2H, m, C(6)H_2), 2.77 (3H, s, C(7)H_3), 2.33 (2H, t, *J* = 7.5 Hz, C(4)H_2), 1.92 – 1.79 (2H, m, C(5)H_2), 1.23 (3H, t, *J* = 7.1 Hz, C(1)H_3); δ_C (101 MHz; $CDCl_3$) 173.7 (C, C3), 152.7 (C, C8), 141.5 (C, C13), 122.5 (CH, C11), 120.8 (CH, C10), 119.4 (CH, C9), 111.3 (CH, C12), 60.3 (CH_2, C2), 55.4 (CH_3, C14), 54.6 (CH_2, C6), 40.2 (CH_3, C7), 32.0 (CH_2, C4), 22.4 (CH_2, C5), 14.3 (CH_3, C1); HRMS (ESI): calcd. for $C_{14}H_{22}NO_3$, 252.1594. Found: $[MH]^+$, 252.1597 (−1.3 ppm error).

Synthesis of 6-methyl-3,4,5,6-tetrahydro-2H-benzo[b][1,4]oxazocin-2-one (81)

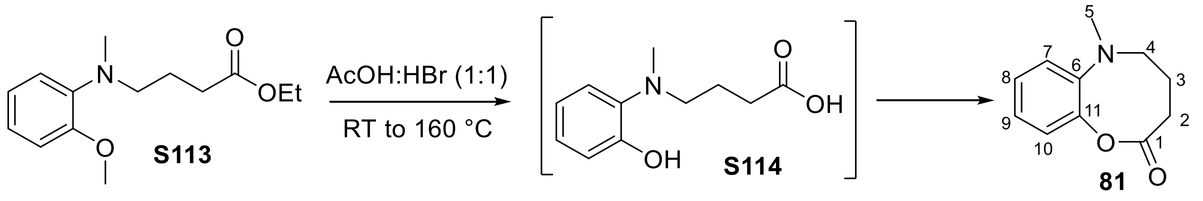

Hydrogen bromide solution (48% in H_2O, 3.0 mL) was added dropwise over a period of 3 min via syring to a solution of ethyl 4-((2-methoxyphenyl)(methyl)amino)butanoate **S113** (250 mg, 1.05 mmol) in AcOH (3.0 mL) at RT under Ar. A colour change to beige suspension was immediately noted along with liberation of fumes and the reaction mixture was stirred at 160 °C for 6 h, at which point the reaction mixture was deemed to have gone to completion by TLC (the appearance 4-((2-methoxyphenyl)(methyl)amino)butanoate **S114** was noted). The resulting reaction mixture was allowed to cool to RT and then concentrated under reduced pressure to yield brown liquid. The concentrated solution was azeotroped with toluene (3 × 30 mL, remove H_2O) [water bath set at 70°C] to afford crude product which was directly used in the next reaction step without further purification. Next, EDC.HCl (705 mg, 3.70 mmol) was added to a pale orange solution of carboxylic acid **S114**, HOBt (355 mg, 2.63 mmol) and dry DIPEA (3.75 mL, 21.2 mmol) in anhydrous DMF (21.0 mL). A colour change to a lilac was immediately noted and the reaction mixture was stirred at RT for 18 h of stirring at RT under Ar, before reaction was deemed to have to completion by TLC. The resulting mixture was diluted with EtOAc (30 mL) and transferred into separating funnel. The organic phase was

washed sequentially with sat. $NaHCO_{3(aq)}$ (2 × 30 mL) and sat. brine (4 × 30 mL), before was dried over $MgSO_4$, filtered and concentrated under reduced pressure to yield an orange oil (328 mg). The crude product was purified by flash column chromatography (SiO_2, 30 mm column, eluent: EtOAc:*n*–hexane, 30:70) to afford lactone **81** as a colourless oil (157 mg, 78%, over two steps). R_f = 0.40 (30:70 EtOAc:n-hexane); IR (thin film) ν_{max} / cm^{-1}: 2945w (C–H alkyl), 2859w (C–H alkyl), 2805w (C–H alkyl), 1748s (C=O aryl ester), 1606m (CC aromatic), 1493s, 1447m, 1364w, 1341m, 1285w, 1256m, 1226m, 1197m, 1169s, 1158s, 1146s, 1104m, 1085m, 1059m, 1036w, 1007s, 982w, 914w, 881w, 832w, 799w, 746s, 706w, 649w, 560w, 541w, 513w, 465w; δ_H (400 MHz; $CDCl_3$) 7.14 – 7.03 (3H, m, C(8 + 9 + 10)H), 7.01 – 6.95 (1H, m, C(7)H), 3.20 – 3.12 (2H, m, C(4)H_2), 2.78 (3H, s, C(5)H_3), 2.51 (2H, t, *J* = 7.0, 6.4 Hz, C(2)H_2), 2.00 – 1.90 (2H, m, C(3)H_2); δ_C (101 MHz; $CDCl_3$) 174.2 (C, C1), 148.4 (C, C6), 141.8 (C, C11), 125.4 (CH), 123.5 (CH), 121.0 (CH), 119.9 (CH), 55.2 (CH_2, C4), 41.4 (CH_3, C5), 32.8 (CH_2, C2), 26.4 (CH_2, C3); HRMS (ESI): calcd. for $C_{11}H_{14}NO_2$, 192.1019. Found: $[MH]^+$, 192.1023 (−2.2 ppm error), calcd. for $C_{11}H_{13}NNaO_2$, 214.0838. Found: $[MNa]^+$, 214.0842 (−1.8 ppm error), calcd. for $C_{22}H_{26}N_2NaO_4$, 405.1785. Found: $[M+M+Na]^+$, 405.1789 (−1.1 ppm error).

2-[(*tert*-Butyldimethylsilyl)oxy]aniline (S115)

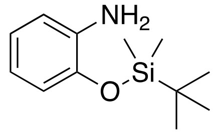

To a solution of 2-aminophenol (10.9 g, 100 mmol) in anhydrous DMF (53.0 mL), TBSCl (16.5 g, 110 mmol) and imidazole (10.2 g, 150 mmol) was added sequentially in a single portion under RT under Ar. The mixture was stirred at RT under Ar overnight, after 18 h, the reaction was deemed to have gone to completion by TLC. The resulting reaction mixture was quenched with sat. $NH_4Cl_{(aq)}$ solution and transfer into a separating funnel. The aqueous layer was extracted with EtOAc (3 × 30 mL), before combined organic layers were collected and washed sequentially with H_2O (2 × 30 mL) and sat. brine (2 × 30 mL). The resulting organic phase was dried over Na_2SO_4, filtrated and concentrated under reduced pressure to yield the crude product as a brown oil (4.01 g). The crude product was purified by flash column chromatography (SiO_2, 60 mm column: eluent:EtOAc:*n*-hexane,10:90) to afford the title compound **S115** as a pale yellow oil (2.66 g, 56%). δ_H (400 MHz; $CDCl_3$) 6.84 – 6.75 (m, 1H),

6.74 (s, 2H), 6.68 – 6.59 (m, 1H), 3.71 (br, s, 2H, NH), 1.03 (s, 9H), 0.25 (s, 6H); δ_C (101 MHz; $CDCl_3$) 143.0 (C), 138.3 (C), 122.0 (CH), 118.6 (CH), 118.5 (CH), 115.8 (CH), 26.0 ($3 \times CH_3$), 18.4 (C), -4.1 ($2 \times CH_3$); HRMS (ESI): calcd. for $C_{12}H_{22}NOSi$, 224.1465. Found: $[MH]^+$, 224.1466 (—0.6 ppm error). The spectroscopic data obtained match those previously reported.[12]

***N*-Benzyl-2-(*tert*-butyldimethylsilyloxy)aniline (S116)**

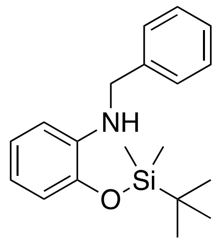

Benzaldehyde (1.21 mL, 11.9 mmol) was added in a single portion via syringe to a solution of 2-[(*tert*-butyldimethylsilyl)oxy]aniline **S115** (2.66 g, 11.9) and $MgSO_4$ (287 mg, 2.39 mmol) in in anhydrous MeOH (40.0 mL) at RT under Ar, with colour changed to bright yellow solution noted. The resulting reaction was stirred at 90 °C for 4 h under Ar, after that time mixture allowed to cool to RT. Next, $NaBH_4$ (903 mg, 23.9 mmol) was added cautiously to the resulting mixture at 0°C under, fizzing, bubbling and effervescent (liberation of H_2) was observed. The resulting milky white suspension was stirred at RT under Ar for 2 h. The resulting mixture was quenched by gradual addition of H_2O (50 mL) and poured into separating funnel. The aqueous layer was extracted with EtOAc (3×20 mL), before combined organic phases were dried over Na_2SO_4, filtered and concentrated under reduced pressure to yield a brown oil (3.76 g). The crude product was purified by flash column chromatography (SiO_2, 60 mm column, eluent: EtOAc:*n*-hexane, 5:95) to afford the title compound **S116** as a pale yellow oil (1.42 g, 38%). δ_H (400 MHz; $CDCl_3$) 7.45 – 7.38 (m, 2H), 7.41 – 7.26 (m, 2H), 6.92 – 6.85 (m, 1H), 6.83 – 6.78 (m, 1H), 6.68 – 6.58 (m, 1H), 4.54 (s, 1H), 4.40 (s, 2H), 1.04 (s, 9H), 0.30 (s, 6H); δ_C (101 MHz; $CDCl_3$) 142.6, 140.3, 139.8, 128.7, 127.4, 127.2, 122.2, 117.6, 116.7, 110.9, 48.2, 26.0, 18.4, -4.1; HRMS (ESI): calcd. for $C_{19}H_{28}NOSi$, 314.1935. Found: $[MH]^+$, 314.1934 (0.1 ppm error). The spectroscopic data obtained match those previously reported.[13]

***tert*-Butyl 3-(benzyl(2-hydroxyphenyl)amino)propanoate (S117)**

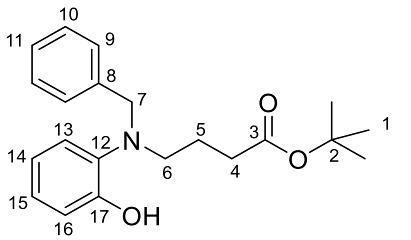

K_2CO_3 (1.29 g, 9.30 mmol) was added in a single portion to a colouless solution of N-benzyl-2-(tert-butyldimethylsilyloxy)aniline **S116** (1.88 g, 8.29 mmol), *tert*-butyl 4-bromobutanoate (817 µL, 4.61mmol) and TBAI (115 mg, 0.310 mmol) in anhydrous DMF (31.0 mL) at RT under Ar. The resulting pale green suspension was then heated to 80 °C under Ar. After 48 h, the reaction was deemed to have gone to completion by TLC. The reaction mixture allowed to cool to RT, before was diluted with EtOAc (30 mL). The diluted reaction mixture was poured into separating funnel and organic layer was washed with sat. brine (3 × 30 mL). The resulting organic phase was collected and dried over MgSO4, filtered and concentrated under reduced pressure to yield a brown oil (1.36 g). The crude product was purified by flash column chromatography (SiO_2, 40 mm column, eluent: EtOAc:*n*-hexane, 20:80) to afford the title compound **S117** as a colourless oil (680 mg, 67%). R_f = 0.25 (EtOAc:*n*-hexane, 20:80); IR (neat) ν_{max} / cm^{-1}: 3387wbr (O–H phenol), 2979w (C–H alkyl), 2935w (C–H alkyl), 2846w (C–H alkyl), 1755 (C=O ester), 1600m (CC aromatic), 1492s (CC aromatic), 1250m, 1149s, 910m, 730s, 698m; δ_H (400 MHz; $CDCl_3$) 7.35 – 7.24 (m, 4H), 7.23 – 7.19 (m, 3H), 7.14 (dd, *J* = 7.8, 1.6 Hz, 1H), 7.09 – 7.04 (m, 1H), 6.91 (dd, *J* = 8.1, 1.5 Hz, 1H), 6.87 – 6.82 (m, 1H), 3.95 (s, 2H), 2.96 – 2.87 (m, 1H), 2.14 (t, *J* = 7.3 Hz, 2H), 1.71 – 1.59 (m, 2H), 1.38 (9H, s, C(1)H_3); δ_C (101 MHz; $CDCl_3$) 172.6 (CO, C3), 153.0 (C), 137.5 (C), 137.5 (C), 136.7 (C), 129.3 (CH), 128.5 (CH), 127.6 (CH), 126.9 (CH), 123.5 (CH), 120.0 (CH), 114.1 (CH), 80.4 (C, C2), 60.9 (CH_2), 53.3 (CH_2), 33.1 (CH_2) 28.1 (CH_3, C1), 22.9 (CH_2); HRMS (ESI): calcd. for $C_{21}H_{28}NO_3$, 342.2064. Found: $[MH]^+$, 342.2072 (−2.3 ppm error), calcd. for $C_{21}H_{27}NNaO_3$, 364.1883. Found: $[MNa]^+$, 364.1892 (−2.5 ppm error).

Synthesis of 5-benzyl-4,5-dihydrobenzo[b][1,4]oxazepin-2(3H)-one (82)

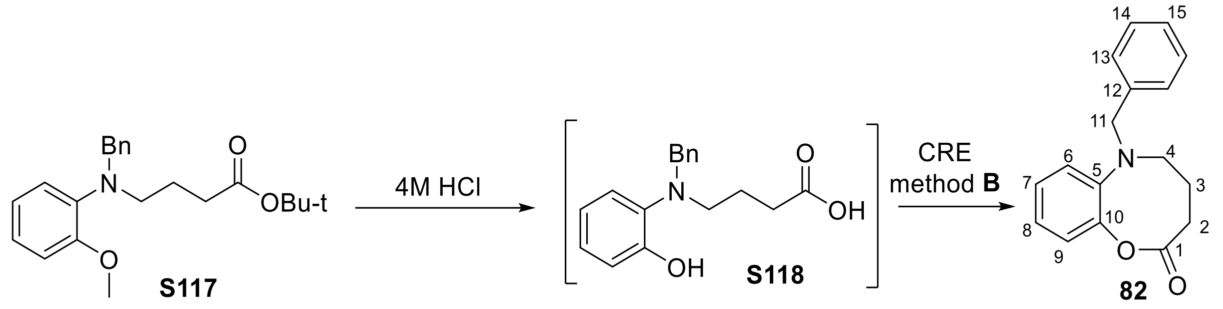

Hydrogen chloride solution (4.0 M in 1,4 dioxane, 9.0 mL) was added dropwise over a period of 1 min via syringe to a pale yellow solution of *tert*-butyl 3-(benzyl(2-hydroxyphenyl)amino)propanoate **S117** (153 mg, 0.447 mmol) in anhydrous Et_2O (5.0 mL) at RT under Ar. A colour change to yellow solution was immediately noted along with the liberation of fumes and the reaction mixture was stirred at RT under Ar for 4 h, at which point the reaction mixture was deemed to have gone to completion by TLC with colour changed to cloudy white suspension was observed. The resulting reaction mixture was concentrated and azeotroped with toluene (3 × 30 mL) to afford crude carboxylic acid **S118** product as a pale pink emulsion which was directly used in the next reaction step without further purification. Next, EDC.HCl (214 mg, 1.12 mmol) was added to a pale-yellow solution of carboxylic acid **S118**, HOBt (90.6 mg, 0.671 mmol) and dry DIPEA (1.20 mL, 6.71 mmol) in anhydrous DMF (9.0 mL). A colour change to a brown solution was noted, after the reaction mixture was stirred at RT for 18 h under Ar. The resulting mixture was diluted with EtOAc (30 mL) and transferred into separating funnel. The resulting organic layer was washed sequentially with sat. $NaHCO_{3(aq)}$ (3 × 20 mL) and sat. brine (4 × 10 mL), before was dried over $MgSO_4$, filtered and concentrated under reduced pressure to yield an orange oil (340 mg). The crude product was purified by flash column chromatography (SiO_2, 30 mm column, eluent: Et_2O:*n*–hexane, 30:70) to afford the title compound **82** as a colourless oil (61.9 mg, 52%, over two steps). R_f = 0.59 (30:70 EtOAc:n-hexane); IR (thin film) ν_{max} / cm^{-1}: 2931w (C–H alkyl), 1750s (C=O ester), 1723s, 1604w (CC aromatic), 1586w (CC aromatic), 1491m, 1450m, 1146s, 1062m, 968m, 748w, 698m; δ_H (400 MHz; $CDCl_3$) 7.45 – 6.98 (9H, m, C(6+7+8+9+13+14+15)H), 4.22 (2H,s, C(11)H_2), 3.09 (2H, t, *J* = 5.9 Hz, C(4)H_2), 2.51 – 2.39 (2H, m, C(2)H_2), 1.87 – 1.77 (2H, m, C(3)H_2); δ_C (101 MHz; $CDCl_3$) 173.2 (CO, C1), 149.3 (C, C10), 142.1 (C, C5), 138.2 (C, C12), 128.6 (2 × CH, C14), 128.5 (2 × CH, C13), 127.4 (CH), 125.9 (CH), 125.7 (CH), 124.5 (CH), 119.8 (CH), 59.9 (CH_2, C11), 54.0 (CH_2, C4), 32.4 (CH_2, C2), 26.9 (CH_2, C3); HRMS (ESI): calcd. for $C_{17}H_{18}NO_2$,

268.1332. Found: $[MH]^+$, 268.1331 (0.2 ppm error), calcd. for $C_{17}H_{17}NNaO_2$, 290.1151. Found: $[MNa]^+$, 290.1151 (0.1 ppm error).

2-(6-Vinylpyridin-2-yl)ethan-1-ol (S119)

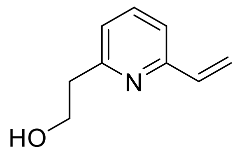

To an argon purged mixture of 1:10 ethanol:1,4-dioxane (2 mL), 2-(6-bromopyridin-2-yl)ethan-1-ol (211 mg, 1.04 mmol), palladium acetate (22.0 mg, 98.0 µmol), SPhos (178 mg, 0.434 mmol) and potassium phosphate (662 mg, 3.12 mmol) were added at room temperature. The solution was heated to 100 °C and vinylboronic acid pinacol ester (0.36 mL, 2.12 mmol) was added. The reaction was stirred at 100 °C for 16 h. The reaction was cooled to room temperature, filtered through Celite and washed with DCM. The filtrate was diluted with H_2O (50 mL) and extracted with DCM (3 × 50 mL). The combined organic phases were then washed with brine (50 mL), dried with sodium sulphate, filtered, concentrated under vacuum and purified via flash column chromatography (3:2 hexane:ethyl acetate) to afford the title compound **S119** as a brown oil (132 mg, 84%). R_f 0.35 (2:3 hexane:ethyl acetate); ν_{max}/cm^{-1} (thin film) 3340, 2928, 1571, 1455, 1248, 1156, 1047, 989, 925, 811, 751; δ_H (400 MHz, $CDCl_3$) 7.54 (1H, dd, *J* = 7.5, 7.5 Hz, Ar**H**), 7.15 (1H, d, *J* = 7.5 Hz, Ar**H**), 6.99 (1H, d, *J* = 7.5 Hz, Ar**H**), 6.73 (1H, dd, *J* = 17.5, 11.0 Hz, ArC**H**CH_2), 6.11 (1H, dd, *J* = 17.5, 1.5 Hz, ArCHC**H**$_2$), 5.42 (1H, dd, *J* = 10.5, 1.5 Hz, ArCHC**H**$_2$); δ_C (101 MHz, $CDCl_3$) 160.5 (Ar**C**), 154.7 (Ar**C**), 137.2 (Ar**C**), 136.6 (Ar**C**HCH_2), 122.2 (Ar**C**), 119.2 (Ar**C**), 118.3 (ArCH**C**H$_2$), 61.7 (**C**H$_2$OH), 38.6 (**C**H$_2$CH$_2$OH); HRMS (ESI) calcd. for $C_9H_{12}NO$ 150.0913. Found $[MH]^+$ 150.0916 (−1.3 ppm error); calcd. for $C_9H_{11}NNaO$ 172.0733. found $[MNa]^+$ 172.0735 (–2.4 ppm error).

2-(6-(2-(Phenylamino)ethyl)pyridin-2-yl)ethan-1-ol (S120)

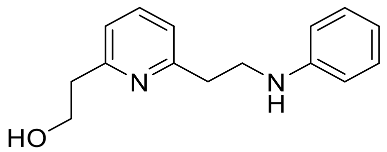

To a stirring solution of 2-(6-vinylpyridin-2-yl)ethan-1-ol **S119** (125 mg, 0.838 mmol) in acetonitrile (1 mL), aniline (0.09 mL, 0.988 mmol) and scandium triflate (43 mg, 87.4 µmol)

were added and stirred at 70 °C for 24 hours. The reaction mixture was concentrated under vacuum and purified via flash column chromatography (2:3 hexane:ethyl acetate) to afford the title compound **S120** as a yellow oil (168 mg, 67%). R_f 0.18 (2:3 hexane:ethyl acetate); ν_{max}/cm^{-1} (thin film) 3368, 2930, 1602, 1576, 1508, 1459, 1260, 1032, 750, 694, 640, 510; δ_H (400 MHz, $CDCl_3$) 7.54 (1H, t, *J* = 7.5 Hz, Ar**H**), 7.23 – 7.13 (2H, m, Ar**H**), 7.03 (1H, t, *J* = 8.0 Hz, Ar**H**), 7.03 (1H, t, *J* = 8.0 Hz, Ar**H**), 6.70 (1H, t, *J* = 7.5 Hz, Ar**H**), 6.66 – 6.59 (2H, t, *J* = 7.5 Hz, Ar**H**), 4.22 (1H, br s, O**H**), 4.03 (2H, t, *J* = 5.5 Hz, C**H**$_2$OH), 3.51 (2H, t, *J* = 6.5 Hz, C**H**$_2$NH), 3.06 (2H, t, *J* = 6.5 Hz, C**H**$_2$CH$_2$NH), 3.01 (2H, t, *J* = 5.5 Hz, C**H**$_2$CH$_2$OH); δ_C (101 MHz, $CDCl_3$) 160.4 (Ar**C**), 158.9 (Ar**C**), 148.1 (Ar**C**), 137.3 (Ar**C**), 129.3 (Ar**C**), 121.2 (Ar**C**), 121.1 (Ar**C**), 117.4 (Ar**C**), 112.9 (Ar**C**), 61.9 (**C**H$_2$OH), 43.6 (**C**H$_2$NH), 38.9 (**C**H$_2$CH$_2$OH), 37.5 (**C**H$_2$CH$_2$NH); HRMS (ESI) calcd. for $C_{15}H_{19}N_2O$ 243.1492. Found $[MH]^+$ 243.1491 (1.4 ppm error).

Cyclic carbamate (83)

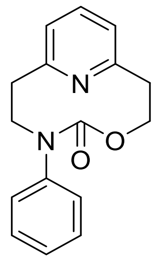

To a stirring solution of 2-(6-(2-(phenylamino)ethyl)pyridin-2-yl)ethan-1-ol **S120** (59.9 mg, 0.247 mmol) and triethylamine (0.172 mL, 1.23 mmol) in DCM (2.47 mL), triphosgene (29.3 mg, 98.8 µmol) was added and stirred at room temperature for 24 hours. The reaction mixture was diluted with H_2O (10 mL), extracted with DCM (3 × 10 mL). The combined organic phases were then dried with sodium sulphate, filtered, concentrated under vacuum and purified via flash column chromatography (7:3 hexane:ethyl acetate) to afford the title compound as a colourless film (20.1 mg, 30%). R_f 0.26 (7:3 hexane:ethyl acetate); ν_{max}/cm^{-1} (thin film) 2954, 1697, 1592, 1571, 1493, 1460, 1386, 1282, 1254, 1106, 987, 776, 754, 703, 590; **δ_H** (400 MHz, $CDCl_3$) 7.88 (2H, s (Br), Ar**H**), 7.61 (1H, t, *J* = 7.5 Hz, Ar**H**), 7.42 (2H, t, *J* = 8.0, 8.0 Hz, Ar**H**), 7.30 (1H, t, *J* = 7.5 Hz, Ar**H**), 7.08 (2H, d, *J* = 7.5 Hz, Ar**H**), 4.75 (1H, td, *J* = 11.5, 2.5 Hz, C**H$_2$),** 4.29 (1H, s(Br), C**H$_2$**), 4.08 (1H, ddd, *J* = 11.5, 5.0, 2.0 Hz, C**H**$_2$), 3.49 (1H, s(Br), C**H**$_2$), 3.26 (1H, td, *J* = 12.5, 5.0 Hz, C**H**$_2$), 3.20 – 3.07 (1H, m, C**H**$_2$), 2.76 (1H, d, *J* = 13.5 Hz, C**H$_2$**), 2.65 (1H, dt, *J* = 13.0, 3.0 Hz, C**H$_2$**); δ_C (101 MHz, $CDCl_3$) 159.3 (**C**O or Ar**C**), 158.6 (**C**O or Ar**C**), 158.3 (**C**O or Ar**C**),

142.5 (ArC), 138.5 (2 × ArC), 128.8 (ArC), 128.3 (ArC), 126.6 (ArC), 120.6 (ArC), 120.1 (ArC), 66.9 (CH_2), 52.9 (CH_2), 38.1 (CH_2), 34.5 (CH_2); HRMS (ESI) calcd. for $C_{16}H_{17}N_2O_2$ 269.1285. Found $[MH]^+$ 269.1287 (–2.7 ppm error); calcd. for $C_{16}H_{16}N_2NaO_2$ 291.1104. Found $[MNa]^+$ 291.1110 (–3.8 ppm error); calcd. for $C_{16}H_{16}KN_2O_2$ 307.0843. Found $[MK]^+$ 307.0845 (–0.7 ppm error).

Ethyl 2-(6-bromopyridin-2-yl)acetate (S121)

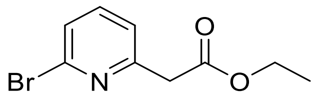

N,N-Diisopropylamine (0.840 mL, 607 mg, 6.00 mmol) was dissolved in THF (15 mL) and cooled to 0 °C before *n*-BuLi (2.40 mL, 2.5 M in hexanes, 6.00 mmol) was added dropwise and stirred for 30 mins. The mixture was cooled to –78 °C before adding 2-bromo-6-methylpyridine (0.340 mL, 516 mg, 3.00 mmol) dropwise and stirring for 30 mins. Ethyl chloroformate (0.140 mL, 163 mg, 1.50 mmol) was added dropwise and stirred for 30 mins before warming to RT. The reaction was quenched with sat. aq. NH_4Cl (10 mL) and extracted with ethyl acetate (3 x 40 mL). The combined organics were washed with brine (10 mL), dried over $MgSO_4$, filtered and concentrated *in vacuo*. The crude product was purified by column chromatography (SiO_2, 6.5:3.5 hexane:ethyl acetate) to yield the title compound **S121** (337 mg, 92%) as a yellow oil. R_f 0.38 (6.5:3.5 hexane:ethyl acetate); δ_H (400 MHz, $CDCl_3$) 7.53 (1H, t, *J* = 8.0 Hz, ArH), 7.40 (1H, d, *J* = 8.0 Hz, ArH), 7.29 (1H, d, *J* = 8.0 Hz), 4.19 (2H, q, *J* = 7.0 Hz, OCH_2), 3.83 (2H, s, CH_2COO), 1.27 (3H, t, *J* = 7.0 Hz). Spectroscopic data matched those reported in the literature.[1]

2-(6-Bromopyridin-2-yl)ethan-1-ol (S122)

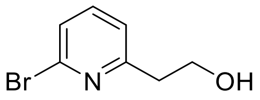

Ethyl 2-(6-bromopyridin-2-yl)acetate **S121** (337 mg, 1.38 mmol) was dissolved in THF (14 mL) and cooled to 0 °C before adding DIBAL-H (3.00 mL, 1.0 M in THF, 3.00 mmol) dropwise. The solution was warmed to RT and stirred for 18 h. The reaction mixture was concentrated *in vacuo* and then purified by column chromatography (SiO_2, diethyl ether) to yield the title compound (218 mg, 78%) as a yellow oil. R_f 0.44 (diethyl ether); δ_H (400 MHz, $CDCl_3$) 7.48 (1H,

t, J = 8.0 Hz, ArH), 7.35 (1H, d, J = 8.0 Hz, ArH), 7.15 (1H, d, J = 8.0 Hz, ArH), 4.01 (2H, t, J = 5.5 Hz, C**H**$_2$OH), 3.12 (1H, br s, OH), 3.01 (2H, t, J = 5.5 Hz, C**H**$_2$CH$_2$OH). Spectroscopic data matched those reported in the literature.[1]

_N_-Methyl-2-(4,4,5,5-tetramethyl-1,3,2-dioxaborolan-2-yl)aniline (S123)

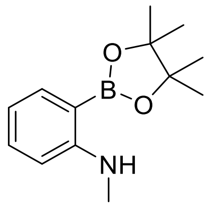

To a solution of 2-(4,4,5,5-tetramethyl-1,3,2-dioxaborolan-2-yl)aniline (1.64 g, 7.50 mmol) in ethanol (34 mL) was added 1-(hydroxymethyl)benzotriazole (1.12 g, 7.50 mmol) and the mixture was stirred at RT for 18 h. The reaction mixture was concentrated *in vacuo* and the product was triturated with hexane to yield crude *N*-((1H-benzo[d][1,2,3]triazol-1-yl)methyl)-2-(4,4,5,5-tetramethyl-1,3,2-dioxaborolan-2-yl)aniline (2.31 g) which was used in the next step without further purification.

To a solution of *N*-((1H-benzo[d][1,2,3]triazol-1-yl)methyl)-2-(4,4,5,5-tetramethyl-1,3,2-dioxaborolan-2-yl)aniline (2.31 g) in THF (19 mL) was added sodium borohydride (250 mg, 6.60 mmol) and the mixture was refluxed for 1 h. The reaction was quenched with water (100 mL) and extracted with ethyl acetate (3 x 100 mL). The combined organic layers were washed with brine (100 mL), dried over $MgSO_4$, filtered and concentrated *in vacuo*. The crude product was purified by column chromatography (4:1 hexane:ethyl acetate) to yield the title compound (1.28 g, 85% over 2-steps) as a white solid. R$_f$ 0.73 (4:1 hexane:ethyl acetate); δ_H (400 MHz, $CDCl_3$) 7.65 (1H, dd, J = 7.5, 2.0 Hz, ArH), 7.34 (1H, td, J = 7.5, 2.0 Hz, ArH), 6.64 (1H, t, J = 7.5 Hz, ArH), 6.57 (1H, d, J = 7.5 Hz, ArH), 5.80 (1H, br s, NH), 2.87 (3H, s, NCH$_3$), 1.35 (12H, s, C(CH$_3$)$_2$). Characterisation data for **S123** matched those reported in the literature.[14]

2-(6-(2-(Methylamino)phenyl)pyridin-2-yl)ethan-1-ol (S124)

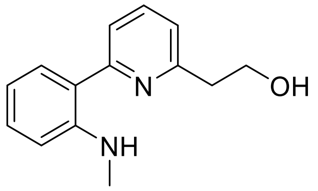

2-(6-Bromopyridin-2-yl)ethan-1-ol **S122** (81 mg, 0.401 mmol) was dissolved in 1,2-dimethoxyethane (3.3 mL). To this was added *N*-methyl-2-(4,4,5,5-tetramethyl-1,3,2-dioxaborolan-2-yl)aniline **S123** (140 mg, 0.601 mmol), caesium carbonate (0.700 mL, 2.1 M in water, 1.40 mmol) and tetrakis(triphenylphosphine)palladium (23 mg, 0.020 mmol) and the reaction mixture was stirred at 80 °C for 18 h. The reaction mixture was concentrated *in vacuo* and then ethyl acetate (10 mL) was added and washed with water (10 mL) and brine (10 mL). The combined aqueous layers were extracted with ethyl acetate (2 x 10 mL). The combined organic layers were dried over $MgSO_4$, filtered and concentrated *in vacuo*. The crude product was purified by column chromatography (SiO_2, 1:1 hexane: ethyl acetate) to yield the title compound **S124** (79 mg, 87%) as a brown oil. R_f 0.28 (1:1 hexane:ethyl acetate); ν_{max}/cm^{-1} (thin film) 3310, 2923, 1606, 1588, 1567, 1519, 1476, 1324, 1223, 1171, 1046, 750; δ_H (400 MHz, $CDCl_3$) 7.68 (1H, t, *J* = 8.0 Hz, ArH), 7.50 (2H, m, ArH), 7.35–7.30 (1H, m, ArH), 7.05 (1H, d, *J* = 7.5 Hz, ArH), 6.80–6.75 (1H, m, ArH), 4.03 (2H, t, *J* = 6.0 Hz, C**H**$_2$OH), 3.06 (2H, t, *J* = 6.0 Hz, C**H**$_2$CH$_2$OH), 2.92 (3H, s, CH_3); δ_C (101 MHz, $CDCl_3$) 159.1 (ArC), 157.8 (ArC), 148.2 (ArC), 137.6 (ArC), 130.4 (ArC), 129.6 (ArC), 122.1 (ArC), 120.7 (ArC), 120.5 (ArC), 116.0 (ArC), 110.9 (ArC), 61.8 (CH_2OH), 40.0 (**C**H$_2$CH$_2$OH), 30.1 (CH_3); HRMS (ESI); calcd. for $C_{14}H_{17}N_2O$, 229.1335. Found: $[MH]^+$, 229.1335 (0.3 error ppm).

1-Methyl-4,5-dihydro-6,10-(azeno)benzo[d][1]oxa[3]azacyclododecin-2(1H)-one (84)

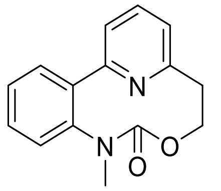

To a solution of 2-(6-(2-(methylamino)phenyl)pyridin-2-yl)ethan-1-ol **S124** (114 mg, 0.500 mmol) and triethylamine (0.350 mL, 253 mg, 2.50 mmol) in DCM (5.0 mL) was added triphosgene (59 mg, 0.200 mmol) and the reaction mixture was stirred at RT for 18 h. The reaction was quenched with sat. aq. $NaHCO_3$ (20 mL) and extracted with DCM (3 x 20 mL).

The combined organic layers were dried over $MgSO_4$, filtered and concentrated *in vacuo*. The crude product was purified by column chromatography (SiO_2, 10:9:1 ethyl acetate:hexane:triethylamine) to yield the title compound **84** (75 mg, 59%) as a yellow oil. R_f 0.56 (10:9:1 ethyl acetate:hexane:triethylamine); ν_{max}/cm^{-1} (thin film) 2958, 1698, 1576, 1451, 1425, 1375, 1330, 1140, 1119, 1016, 754; δ_H (400 MHz, $CDCl_3$) 7.88 (1H, d, *J* = 7.5 Hz, ArH), 7.68 (1H, t, *J* = 8.0 Hz, ArH), 7.51 (1H, d, *J* = 8.0 Hz), 7.46–7.37 (3H, m, ArH), 7.07 (1H, d, *J* = 7.5 Hz, ArH), 5.27 (1H, td, *J* = 12.0, 2.5 Hz, OC**H**H'), 4.34 (1H, ddd, *J* = 12.0, 4.5, 1.5 Hz, OCH**H'**), 3.30 (1H, ddd, *J* = 17.0, 12.5, 4.5, ArC**H**H'), 2.96 (1H, dt, 17.0, 2.5 Hz, ArCH**H'**), 2.75 (3H, s, CH_3); δ_C (101 MHz, $CDCl_3$) 161.7 (CO), 157.0 (ArC), 153.3 (ArC), 145.4 (ArC), 137.5 (ArC), 133.0 (ArC), 131.3 (ArC), 130.5 (ArC), 128.2 (ArC), 127.7 (ArC), 120.7 (ArC), 118.4 (ArC), 66.1 (OCH_2), 39.0 (CH_3), 36.1 ($ArCH_2$); HRMS (ESI); calcd. for $C_{15}H_{15}N_2O_2$, 255.1128. Found: $[MH]^+$, 255.1129 (–0.5 error ppm).

2-(6-Bromopyridin-2-yl)-1-phenylethan-1-ol (S125)

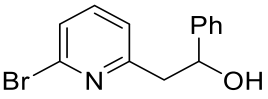

N,*N*-Diisopropylamine (3.67 mL, 26.2 mmol) was dissolved in THF (60 mL) and cooled to 0 ^oC before *n*-BuLi (1.6 M solution in hexanes, 16.4 mL, 26.2 mmol) was added dropwise and stirred for 30 mins. The LDA solution was then cooled to −78 °C, where 2-bromo-6-methylpyridine (1.48 mL, 13.1 mmol) was added dropwise and stirred for 30 mins. Benzaldehyde (2.67 mL, 26.2 mmol) was added and stirred for a further 30 mins at −78 °C before slowly warming to RT. The solution was then quenched with sat. $NH_4Cl_{(aq)}$ (20 mL) and extracted with ethyl acetate (3 × 50 mL) and washed with brine (10 mL). The combined organic extracts were dried over MgSO4, filtered and removed *in vacuo*. Purification by flash column chromatography (SiO_2, 25% ethyl acetate in hexane → ethyl acetate) afforded the title compound **S125** as a yellow oil (3.02 g, 83%); R_f 0.67 (50% ethyl acetate in hexanes); ν_{max}/cm^{-1} (thin film) 3370, 3062, 3030, 2924, 1584,1553, 1437; δ_H (400 MHz, $CDCl_3$) 7.44 (1H, t, J = 7.6 Hz, C**H**), 7.41–7.22 (6H, m, 6 × C**H**), 7.05 (1H, d, J = 7.6 Hz, C**H**), 5.19–5.10 (1H, m, C**H**OH), 4.20–4.12 (1H, m, O**H**), 3.18–3.04 (2H, m, C**H**$_2$CHOH); δ_C (101 MHz, $CDCl_3$) 164.7 (**C**N), 147.4 (**C**Br), 145.1 (**C**), 142.8 (**C**H), 132.3 (**C**H), 131.4 (**C**H), 129.9 (**C**H), 129.6 (**C**H), 126.6 (**C**H), 77.0 (**C**HOH),

49.9 (**C**H_2CHOH); HRMS (ESI): calcd. for $C_{13}H_{12}{}^{79}BrNNaO$, 299.9994. Found: $[MNa]^+$, 299.9994 (0.3 ppm error). Spectroscopic data matched those reported in the literature.[1]

2-(6-(2-(Methylamino)phenyl)pyridin-2-yl)-1-phenylethan-1-ol (S126)

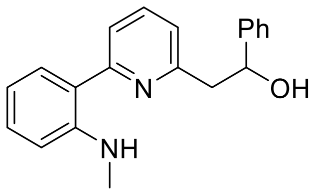

2-(6-Bromopyridin-2-yl)-1-phenylethan-1-ol **S125** (526 mg, 1.89 mmol) was dissolved in 1,2-dimethoxyethane (16 mL). To this was added *N*-methyl-2-(4,4,5,5-tetramethyl-1,3,2-dioxaborolan-2-yl)aniline **S123** (663 mg, 2.84 mmol), caesium carbonate (3.20 mL, 2.1 M in water, 6.62 mmol) and tetrakis(triphenylphosphine)palladium (110 mg, 0.095 mmol) and the reaction mixture was stirred at 80 °C for 18 h. The reaction mixture was concentrated *in vacuo* and then ethyl acetate (50 mL) was added and washed with water (50 mL) and brine (50 mL). The combined aqueous layers were extracted with ethyl acetate (2 x 50 mL). The combined organic layers were dried over $MgSO_4$, filtered and concentrated *in vacuo*. The crude product was purified by column chromatography (SiO_2, 4:1 hexane:ethyl acetate) to yield the title compound **S126** (192 mg, 33%) as a yellow oil. R_f 0.25 (4:1 hexane:ethyl acetate); ν_{max}/cm^{-1} (thin film) 3327, 2877, 2812, 1604, 1587, 1566, 1519, 1454, 1322, 1249, 1170, 1038, 909, 799, 746, 698, 628; δ_H (400 MHz, $CDCl_3$) 7.69 (1H, t, *J* = 8.0 Hz, ArH), 7.53–7.49 (2H, m, ArH), 7.45–7.28 (6H, m, ArH), 7.02 (1H, d, *J* = 8.0 Hz, ArH), 6.82–6.76 (2H, m, ArH), 5.20 (1H, dd, *J* = 7.5, 5.0 Hz, CH), 3.26–3.21 (2H, m, CH_2), 2.93 (3H, s, CH_3); δ_C (101 MHz, $CDCl_3$) 159.1 (ArC), 157.4 (ArC), 148.1 (ArC), 143.9 (ArC), 137.9 (ArC), 130.5 (ArC), 129.8 (ArC), 128.6 (ArC), 128.5 (ArC), 127.6 (ArC), 125.9 (ArC), 121.3 (ArC), 121.0 (ArC), 116.2 (ArC), 111.1 (ArC), 73.7 (CH), 46.6 (CH_2), 30.3 (CH_3); HRMS (ESI); calcd. for $C_{20}H_{21}N_2O$, 305.1648. Found: $[MH]^+$, 305.1648 (0.2 error ppm).

1-Methyl-4-phenyl-4,5-dihydro-6,10-(azeno)benzo[*d*][1]oxa[3]azacyclododecin-2(1*H*)-one (85)

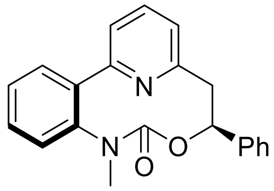

To a solution of 2-(6-(2-(methylamino)phenyl)pyridin-2-yl)-1-phenylethan-1-ol **S126** (103 mg, 0.338 mmol) and triethylamine (0.240 mL, 171 mg, 1.69 mmol) in DCM (3.4 mL) was added triphosgene (40 mg, 0.135 mmol) and the reaction mixture was stirred at RT for 18 h. The reaction was quenched with sat. aq. $NaHCO_3$ (10 mL) and extracted with DCM (3 x 10 mL). The combined organic layers were dried over $MgSO_4$, filtered and concentrated *in vacuo* to yield the crude product (104 mg). Compound **85** was unstable to column chromatography and could not be purified; therefore, to this mixture 1,3,5-trimethoxybenzene (57 mg, 0.338 mmol) was added to determine the yield of the title compound by comparison to this internal standard (34%). The ^{1}H NMR signals corresponding to the product can be found at: δ_H (400 MHz, $CDCl_3$) 7.94–7.89 (1H, m, ArH), 7.74 (1H, t, *J* = 8.0 Hz, ArH), 7.63–7.28 (9H, m, ArH), 7.12 (1H, d, *J* = 8.0 Hz, ArH), 6.33 (1H, dd, *J* = 11.0, 2.5 Hz, CH), 3.44–3.21 (2H, m, CH_2), 2.89 (3H, s, CH_3). HRMS (ESI); calcd. for $C_{21}H_{19}N_2O_2$, 331.1443. Found: $[MH]^+$, 331.1441 (–0.5 error ppm).

1-Methyl-4,5-dihydro-1H-6,10-(azeno)benzo[d][1]oxa[2]thia[3]azacyclododecine 2-oxide (86)

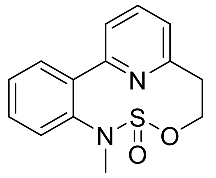

To a solution of 2-(6-(2-(methylamino)phenyl)pyridin-2-yl)ethan-1-ol **S124** (114 mg, 0.500 mmol) and DMAP (305 mg, 2.50 mmol) in DCM (5.0 mL) was added thionyl chloride (0.040mL, 71 mg, 0.600 mmol) and the reaction mixture was stirred at RT for 18 h. The reaction was concentrated *in vacuo* and then purified by column chromatography (SiO_2, 11:8:1 hexane:ethyl acetate:triethylamine) to yield the title compound **86** (63 mg, 46%) as a brown oil. R*f* 0.67 (11:8:1 hexane:ethyl acetate:triethylamine); ν_{max}/cm^{-1} (thin film) 2921, 2813, 1605, 1587, 1566, 1519, 1559, 1326, 1251, 1205, 1172, 963, 872, 803, 748, 699; δ_H (400 MHz, $CDCl_3$)

7.62 (1H, t, *J* = 8.0 Hz, ArH), 7.53–7.47 (2H, m, ArH), 7.32 (1H, td, *J* = 7.5, 1.5 Hz, ArH), 6.99 (1H, d, *J* = 7.5 Hz, ArH), 6.77–6.72 (2H, m, ArH), 4.42–4.28 (2H, m, OCH_2), 3.13 (2H, t, *J* = 6.5 Hz, $ArCH_2$), 2.92 (3H, s, CH_3); δ_C (101 MHz, $CDCl_3$) 159.6 (ArC), 155.1 (ArC), 148.6 (ArC), 137.5 (ArC), 130.5 (ArC), 129.5 (ArC), 121.4 (ArC), 120.7 (ArC), 120.6 (ArC), 115.7 (ArC), 110.9 (ArC), 61.3 (OCH_2), 37.9 ($ArCH_2$), 30.0 (CH_3); HRMS (ESI); calcd. for $C_{14}H_{15}N_2O_2S^+$, 275.0849. Found: $[MH]^+$, 275.0850 (–0.3 error ppm).

(2-(6-(2-(Phenylamino)propyl)pyridin-2-yl)phenyl)methanol (S127)

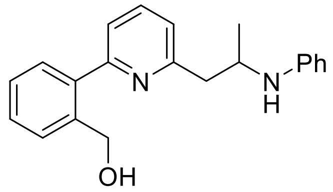

To a stirring solution of 2-(hydroxymethyl)benzene boronic acid (1.28 g, 8.45 mmol), tetrakis(triphenylphosphine)-palladium(0) (0.330 g, 0.280 mmol) and potassium carbonate (1.56 g, 11.3 mmol) in 1,4-dioxane (20.0 mL) and water (5.00 mL), *N*-(1-(6-bromopyridin-2-yl)propan-2-yl)aniline (1.64 g, 5.63 mmol) was added and heated at 80 °C for 18 hours under nitrogen. The reaction mixture was cooled to room temperature and the solvent removed under vacuum. The crude residue was dissolved in ethyl acetate (25 mL) and water (25 mL). The phases were separated and the aqueous washed with ethyl acetate (3 x 25 mL). The combined organic phases were dried with magnesium sulphate, filtered, concentrated under vacuum and purified via flash column chromatography (50% to 70% diethyl ether:hexane) to afford the title compound **S127** as a light brown solid (1.72 g, 5.41 mmol, 96%) R_f = 0.34 (7:3 diethyl ether:hexane). ν_{max}/cm^{-1} (thin film)3330, 3056, 2963, 2867, 1601, 1568, 1499, 1449, 1320, 1258, 1202, 1155, 1013, 959, 749, 694; δ_H (400 MHz, $CDCl_3$) 7.76 (1H, t, *J* = 7.8 Hz, Ar**H**), 7.58−7.49 (2H, m, Ar**H**), 7.48−7.39 (3H, m, Ar**H**), 7.22 (1H, d, *J* = 7.8 Hz, Ar**H**), 7.16 (2H, t, *J* = 8.0 Hz Ar**H**), 6.66 (1H, t, *J* = 7.3 Hz Ar**H**), 6.62 (2H, d, *J* = 8.6 Hz Ar**H**), 6.53 (1H, bs, CH_2O**H**), 4.50 (2H, s, C**H**$_2$OH); 4.05−3.94 (1H, m, CH_2C**H**), 3.80 (1H, bs, CHN**H**), 3.12 (1H, dd, *J* = 13.5, 7.2 Hz, C**H**$_2$CH), 3.12 (1H, dd, *J* = 13.5, 6.2 Hz, C**H**$_2$CH), 1.26 (3H, d, *J* = 6.3 Hz, C**H**$_2$CH); δ_c (101 MHz, $CDCl_3$) 158.8 (Ar**C**), 158.2 (Ar**C**), 147.2 (Ar**C**), 140.4 (Ar**C**), 140.2 (Ar**C**), 138.0 (Ar**C**H), 131.2 (Ar**C**H), 130.3 (Ar**C**H), 129.4 (Ar**C**H), 128.3 (Ar**C**H) 128.3 (Ar**C**H), 122.4 (Ar**C**H), 121.8 (Ar**C**H) 117.2 (Ar**C**H), 113.3 (Ar**C**H), 64.7 (**C**H_2OH), 49.3 (CH_2**C**H), 45.4 (**C**H_2CH), 21.0 (**C**H_3CH); HRMS (ESI) calcd. for $C_{21}H_{22}N_2O$ 318.1732. Found $[MH]^+$ 319.1805 (–0.40 ppm error).

Macrocycle (87)

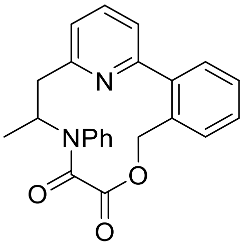

Oxalyl chloride (14.5 µL, 0.166 mmol) was added in a single portion via syringe to a solution of (2-(6-(2-(phenylamino)propyl)pyridin-2-yl)phenyl)methanol **S127** (48 mg, 0.151 mmol), dry Et_3N (105 µL, 0.755 mmol) in anhydrous CH_2Cl_2 (1.5 mL) under Ar at RT. The resulting solution was stirred at RT for 3 h, after which time the reaction was deemed to have gone to completion by TLC analysis. The resulting mixture was diluted with DCM (20 mL), washed with water (10 mL) and concentrated *in vacuo*. Purification by column chromatography (2:1 hexane:ethyl acetate) afforded the macrocycle **87** as a pale yellow oil (40 mg, 71%); R_f 0.52 (ethyl acetate); δ_H (400 MHz, $CDCl_3$) 7.79 (1H, app t, *J* = 7.8, Ar**H**), 7.60–7.38 (8H, m, Ar**H**), 7.06 (1H, d, *J* = 7.8 Hz, Ar**H**), 6.69–6.91 (1H, br m, Ar**H**), 5.53–5.21 (2H, br m, OC**H₂**), 2.94 (1H, dd, *J* = 16.2, 2.8 Hz, ArC**H**H'), 1.45 (3H, d, *J* = 6.4 Hz, C**H₃**), 1.32–1.15 (1H, br m, C**H**CH_3); δ_C (101 MHz, $CDCl_3$) 164.2 (**C**O), 163.0 (**C**O), 159.3 (Ar**C**), 156.6 (Ar**C**), 137.7 (Ar**C**H), 134.6 (Ar**C**), 132.3 (Ar**C**), 130.5 (Ar**C**H), 130.0 (Ar**C**), 129.7 (Ar**C**H), 129.4 (Ar**C**H), 129.4 (Ar**C**H), 129.2 (Ar**C**H), 128.9 (Ar**C**H) 122.7 (Ar**C**H), 122.6 (Ar**C**H), 122.3 (Ar**C**H), 77.3 (O**C**H_2), 41.1 (**C**HCH_3), 29.8 (**C**H_2Ar), 20.5 (**C**H_3); HRMS (ESI): calcd. for $C_{23}H_{20}N_2NaO_3$, 395.1373. Found: $[MNa]^+$, 395.1366 (–1.7 ppm error).

1-(6-(2-(Hydroxymethyl)phenyl)pyridin-2-yl)propan-2-one (S128)

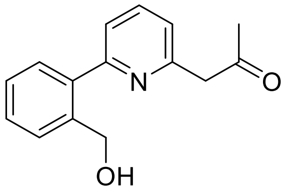

To a stirring solution of 2-(hydroxymethyl)benzene boronic acid (0.114 g, 0.750 mmol), tetrakis(triphenylphosphine)-palladium(0) (0.0290 g, 0.0250 mmol) and potassium carbonate (0.138 g, 0.998 mmol) in 1,4-dioxane (4.00 mL) and water (1.00 mL), 1-(6-bromopyridine-2-yl)propan-2-one (0.107 g, 0.500 mmol) was added and heated at 80 °C for 18 hours under nitrogen. The reaction mixture was cooled to room temperature and the solvent removed

under vacuum. The crude residue was dissolved in ethyl acetate (10 mL) and water (10 mL). The phases were separated and the aqueous washed with ethyl acetate (3 x 10 mL). The combined organic phases were dried with magnesium sulphate, filtered, concentrated under vacuum and purified via flash column chromatography (1:1 hexane:ethyl acetate) to afford the title compound **S128** as a yellow oil (0.116 g, 0.481 mmol, 96%) R_f = 0.15 (1:1 hexane:ethyl acetate). ν_{max}/cm^{-1} (thin film) 3331, 3068, 2957, 2868, 1715, 1591, 1567, 1448, 1357, 1202, 1160, 1014, 958, 825, 763; δ_H (400 MHz, $CDCl_3$) 7.83 (1H, t, *J* = 7.8 Hz, Ar**H**), 7.56–7.50 (2H, m, Ar**H**), 7.49–7.46 (1H, m, Ar**H**), 7.45–7.38 (2H, m, Ar**H**), 7.29–7.23 (1H, m, Ar**H**), 6.02 (1H, bs, CH_2O**H**), 4.46 (2H, s, C**H**$_2$OH), 3.99 (2H, s, C**H**$_2$CO), 2.27 (3H, s, C**H**$_3$CO); δ_c (101 MHz, $CDCl_3$) 204.7 (**C**O), 159.3 (Ar**C**), 153.3 (Ar**C**), 140.3 (Ar**C**), 139.8 (Ar**C**), 138.2 (Ar**C**H), 131.3 (Ar**C**H), 130.3 (Ar**C**H), 129.5 (Ar**C**H), 128.3 (Ar**C**H) 123.0 (Ar**C**H), 122.4 (Ar**C**H), 64.7 (**C**H_2OH), 52.9 (**C**H_2CO), 30.3 (**C**H_3CO); HRMS (ESI) calcd. for $C_{15}H_{15}NO_2$ 241.1103. Found $[MH]^+$ 242.1176 (2.60 ppm error).

1-(6-(2-(hydroxymethyl)phenyl)pyridin-2-yl)propan-2-ol (S129)

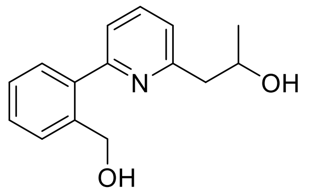

To a stirring solution of 1-(6-(2-(hydroxymethyl)phenyl)pyridin-2-yl)propan-2-one **S128** (1.00 g, 4.14 mmol) in dry methanol (50.0 mL), sodium borohydride (0.627 mg, 16.6 mmol) was added and the resulting reaction mixture stirred at room temperature for 1 hour. The solvent was removed under vacuum and the crude residue dissolved in ethyl acetate (50 mL) and water (50 mL). The phases were separated and the aqueous washed with ethyl acetate (3 x 50 mL). The combined organic phases were washed with brine, dried with magnesium sulphate, filtered and concentrated under vacuum. The crude product was purified via flash column chromatography (4:1 hexane:ethyl acetate) to afford the title compound **S129** as a yellow oil (1.004 g, 4.13 mmol, 99%); R_f = 0.25 (4:1 hexane:ethyl acetate); ν_{max}/cm^{-1} (thin film) 3308, 2965, 2927, 2872, 1592, 1569, 1448, 1201, 1120, 1010, 942, 806, 762, 634; δ_H (400 MHz, $CDCl_3$) 7.77 (1H, t, *J* = 7.4 Hz, Ar**H**), 7.55–7.44 (3H, m, Ar**H**), 7.44–7.37 (2H, m, Ar**H**), 7.24 (1H, d, *J* = 7.7 Hz, Ar**H**), 4.47 (2H, s, C**H**$_2$OH), 4.26 (1H, m, C**H**OH), 3.04–2.91 (2H, m, C**H**$_2$CH), 1.28 (3H, d, *J* = 6.3 Hz, C**H**$_3$CH); δ_c (101 MHz, $CDCl_3$) 158.9 (Ar**C**), 158.2 (Ar**C**), 140.2 (Ar**C**), 140.1

(Ar**C**), 138.0 (Ar**C**H), 131.1 (Ar**C**H), 130.3 (Ar**C**H), 129.3 (Ar**C**H), 128.3 (Ar**C**H) 122.8 (Ar**C**H), 121.9 (Ar**C**H), 67.8 (**C**HOH), 64.6 (**C**H_2OH), 47.5 (**C**H_2CH), 23.5 (**C**H_3CH); HRMS (ESI) calcd. for $C_{15}H_{17}NO_2$ 243.1259. Found $[MH]^+$ 244.1332 (0.40 ppm error).

5-Methyl-5,6-dihydro-1*H*-7,11-(azeno)benzo[*e*][1,3]dioxa[2]thiacyclotridecine 3-oxide (88)

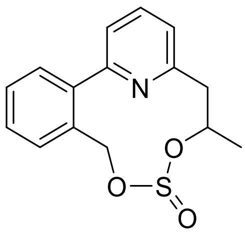

To a solution of 1-(6-(2-(hydroxymethyl)phenyl)pyridin-2-yl)propan-2-ol **S129** (135 mg, 0.555 mmol) and DMAP (340 mg, 2.78 mmol) in DCM (5.6 mL) was added thionyl chloride (0.050 mL, 79 mg, 0.666 mmol) and the reaction mixture was stirred at RT for 18h. The reaction mixture was concentrated *in vacuo* and purified by column chromatography (SiO_2, 1:1 hexane:ethyl acetate) to yield the title compound (93 mg, 58%) as a 5:1 (A:B) mixture of diastereoisomers as a white solid. R*f* 0.72 (1:1 hexane:ethyl acetate); m.p. 40–44 °C; ν_{max}/cm^{-1} (thin film) 2972, 2930, 1589, 1572, 1450, 1378, 1187, 1101, 1036, 908, 879, 838, 760, 728; δ_H (400 MHz, $CDCl_3$) 7.72–7.60 (4H, m, ArH), 7.56–7.36 (8H, m, ArH), 7.11–7.07 (2H, m, ArH), 6.00–5.90 (1H, m, CH isomer B), 5.48–5.38 (1H, m, CH isomer A), 5.17 (1H, d, *J* = 9.5 Hz, C**H**H'O isomer A), 5.07 (1H, d, *J* = 8.5 Hz, C**H**H'O isomer B), 4.77 (1H, d, *J* = 8.5 Hz, CH**H'**O isomer B), 4.69 (1H, d, *J* = 9.5 Hz, CH**H'**O isomer A), 3.27–3.13 (4H, m, ArC**H**$_2$CH both isomers), 1.56–1.51 (6H, m, CH_3 both isomers); δ_C (101 MHz, $CDCl_3$) 157.0 (ArC isomer A, ArC isomer B), 156.0 (ArC isomer B), 155.8 (ArC isomer A), 141.6 (ArC isomer A), 140.8 (ArC isomer B), 137.0 (ArC isomer A, ArC isomer B), 133.8 (ArC isomer A), 133.5 (ArC isomer B), 132.8 (ArC isomer B), 132.5 (ArC isomer A), 129.5 (ArC isomer A), 129.0 (ArC isomer A), 128.9 (ArC isomer B), 128.8 (ArC isomer A), 128.7 (ArC isomer B), 121.4 (ArC isomer A), 121.3 (ArC isomer B), 120.3 (ArC isomer A), 120.2 (ArC isomer B), 69.8 (CH isomer A), 68.9 (CH isomer B), 66.4 (CH_2O isomer B), 61.4 (CH_2O isomer A), 42.4 (Ar**C**H$_2$CH isomer B), 41.9 (Ar**C**H$_2$CH isomer A), 23.5 (CH_3 isomer B), 22.0 (CH_3 isomer A); HRMS (ESI); calcd. for $C_{15}H_{16}NO_3S$, 290.0845. Found: $[MH]^+$, 290.0844 (0.6 error ppm).

3,3'-(Phenylazanediyl)bis(propan-1-ol) (S130)

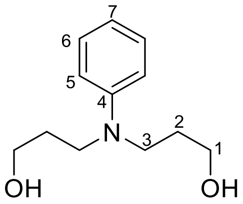

$CaCO_3$ (6.48 g, 64.4 mmol) was added in a single portion to a brown solution of 3-chloropropan-1-ol (18.3 g, 193 mmol) and aniline (3.00 g, 32.2 mmol) in H_2O (108 mL) at RT. The resulting creamy pale-yellow suspension was then heated to 120 °C, after 26 h, the reaction was deemed to have gone to completion by TLC. The reaction mixture allowed to cool to RT and base was added until solution was at pH 10, by adding 1 M $NaOH_{(aq)}$ (30 mL). The basic aqueous solution was then poured into a separating funnel and resulting aqueous phase was extracted with CH_2Cl_2 (3 × 30 mL). The combined organic layers were collected dried over $MgSO_4$, filtered and concentrated under reduced pressure to yield a brown oil (6.10 g). The crude product was purified by flash column chromatography (SiO_2, 70 mm column, eluent: EtOAc:*n*-hexane, 80:20 to 100:0) to afford the title compound **S130** as a colourless viscous oil (6.07 g, 90%); R_f = 0.16 (EtOAc:*n*–hexane, 80:20); IR (neat) ν_{max} / cm $^{-1}$: 3318brw (O–H alcohol), 3061w, 3006w, 2938w (C–H alkyl), 2877w (C–H alkyl), 1921w, 1597s (CC aromatic), 1571w (CC aromatic), 1504s (CC aromatic), 1476w, 1463, 1451w, 1395w, 1367m, 1285w, 1216w, 1193w, 1050s, 1038s, 990w, 980w, 909m, 863w, 744s, 693s, 666w, 511w, 461w; δ_H (500 MHz; $CDCl_3$) 7.28 – 7.20 (2H, m, C(6)H), 6.79 – 6.76 (2H, m, C(5)H), 6.75 – 6.71 (1H, m, C(7)H), 3.69 (4H, t, *J* = 6.0 Hz, C(1)H_2), 3.63 – 3.51 (2H, br, m, OH), 3.42 (4H, t, *J* = 7.1 Hz, C(3)H_2), 1.87 – 1.78 (4H, m, C(2)H_2); δ_C (126 MHz; $CDCl_3$) 148.2 (C, C4), 129.3 (2 × CH, C6), 116.7 (CH, C7), 113.3 (2 × CH, C5), 60.4 (CH_2, C1), 48.4 (CH_2, C3), 29.9 (CH_2, C2); HRMS (ESI): calcd. for $C_{12}H_{20}NO_2$, 210.1489. Found: $[MH]^+$, 210.1486 (1.3 ppm error).

7-Phenyl-1,3,2,7-dioxathiazecane 2-oxide (89)

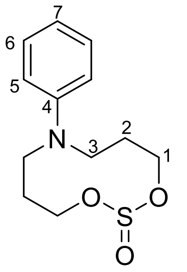

Thionyl chloride (104 µL, 1.43 mmol) was added in a single portion via syringe to colourless solution of 3,3'-(phenylazanediyl)bis(propan-1-ol) **S130** (250 mg, 1.19 mmol), DMAP (509 mg, 4.17 mmol) in anhydrous CH_2Cl_2 (24.0 mL) at 0 °C under Ar. An immediate colour changed to mustard yellow suspension was noted along with the liberation of grey fumes. The resulting mixture was allowed to warm gradually to RT overnight under Ar and the progress of the reaction was monitored by TLC, with colour changed to pale orange solution was observed. After total of 18 h, the reaction was deemed to have gone to completion by TLC analysis. The resulting mixture was quenched with sat. $NaHCO_{3(aq)}$ (30 mL) and poured into separating funnel. The aqueous solution was extracted with CH_2Cl_2 (3 × 30 mL), before combined organic layers were collected dried over $MgSO_4$, filtered and concentrated under reduced pressure to yield a yellow solid (879 mg). The crude product was purified by flash column chromatography (SiO_2, 40 mm column, eluent: EtOAc:*n*–hexane, 20:80) to afford the title compound **89** as a colourless oil (121 mg, 40%); R_f = 0.23 (EtOAc:*n*–hexane, 20:80); IR (neat) ν_{max} / cm^{-1}: 3060w, 2953w, 2849w, 1599s (CC aromatic), 1575w (CC aromatic), 1502s (CC aromatic), 1473w, 1459w, 1431w, 1385w, 1362w, 1326w, 1269w, 1196s (S=O), 1172s (S=O), 1124w, 1092w, 1067w, 1029w, 9892, 955w, 904s, 854w, 823w, 791w, 747m, 735m, 715w, 693, 588w, 621w, 544w, 470w; δ_H (400 MHz; $CDCl_3$) 7.29 – 7.21 (2H, m, C(6)H), 6.82 – 6.75 (3H, m, C(5+7)H), 4.48 (2H, ddd, *J* = 11.0, 7.4, 3.3 Hz, C(1)H_2), 3.94 (2H, ddd, *J* = 11.0, 7.4, 3.3 Hz, C(1)H_2), 3.54 – 3.35 (4H, m, C(3)H_2), 2.19 – 1.96 (4H, m, C(2)H_2); δ_C (101 MHz; $CDCl_3$) 149.7 (C, C4), 129.2 (2 × CH, C6), 118.3 (CH, C7), 115.0 (2 × CH, C5), 61.1 (2 × CH_2, C1), 52.1 (2 × CH_2, C3), 28.5 (2 × CH_2, C2); HRMS (ESI): calcd. for $C_{12}H_{18}NO_3S$, 256.1002. Found: $[MH]^+$, 256.1006 (−1.7 ppm error), calcd. for $C_{12}H_{17}NNaO_3S$, 278.0821. Found: $[MNa]^+$, 278.0825 (−1.2 ppm error), calcd. for $C_{12}H_{17}KNO_3S$, 294.0561. Found: $[MK]^+$, 294.0561 (−0.2 ppm error).

3-(Ethyl(3-(ethylamino) propyl) amino) propan-1-ol (S131)

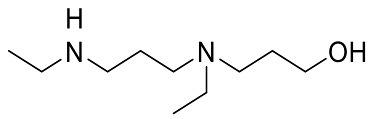

3-Bromopropanol (904 μL, 1.31 g, 10.0 mmol) was added to a mixture of N, N′-diethyl-1,3-propanediamine (3.18 mL, 2.61 g, 20.0 mmol) and potassium carbonate (6.91 g, 50.0 mmol) in acetonitrile (50 mL) and the mixture was heated at 60 °C for 4 hours. After cooling to room temperature, the mixture was filtered through Celite, washing with MeCN (50 mL) and the filtrate concentrated under reduce pressure. The residue was purified by silica gel flash column chromatography, eluting with CH_2Cl_2:MeOH:Et_3N (50:45:5), to give the product **S131** as a colourless oil (1.31 g, 70%), R_f = 0.29 (CH_2Cl_2:MeOH:Et_3N 50:45:5); ν_{max}/cm^{-1} (thin film) 3271, 2965, 2934, 2811, 1461, 1376, 1295, 1117, 1043, 922, 800, 735; δ_H (400 MHz, $CDCl_3$) 0.90−0.94 (3H, m, C**H**$_3$CH$_2$N), 0.96−1.01 (3H, m, C**H**$_3$CH$_2$N), 1.60−1.52 (4H, m, 2× CH$_2$C**H**$_2$CH$_2$), 2.43−2.33 (4H, m, 2× CH$_3$C**H**$_2$N), 2.55−2.48 (6H, m, 3× C**H**$_2$N), 3.44 (1H, bs, N**H**), 3.64−3.61 (2H, m, C**H**$_2$OH); δ_C (101 MHz, $CDCl_3$) 15.1 (**C**H$_3$CH$_2$N), 11.5 (**C**H$_3$CH$_2$N), 27.2 (CH$_2$**C**H$_2$CH$_2$), 28.0 (CH$_2$**C**H$_2$CH$_2$), 44.1 (**C**H$_2$N), 47.2 (**C**H$_2$N), 48.1 (**C**H$_2$N), 51.7 (**C**H$_2$N), 53.6 (**C**H$_2$N), 63.7 (CH$_2$**C**H$_2$OH); HRMS (ESI) calcd. for $C_{10}H_{25}N_2O$ 189.1967. Found 189.1966 [MH]$^+$ (−0.53 ppm error).

3,7-Diethyl-1,3,7-oxadiazecan-2-one (90)

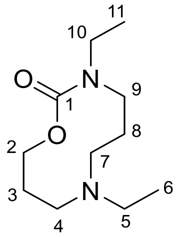

3-(Ethyl(3-(ethylamino)propyl)amino)propan-1-ol **S131** (237 mg, 1.26 mmol) was added reaction flask (250 mL RBF) containing anhydrous DMF (23.0 mL). The resulting reaction vessel was evacuated under *vacuo* and backfilled with CO_2 (via balloon) 6 times. Next, EDC.HCl (209 mg, 1.34 mmol), HOBt (182 mg, 1.34 mmol) and dry DIPEA (546 µL, 3.14 mmol) were each added in single portion at RT, with a colour change to pale yellow solution noted. The resulting solution was stirred at RT overnight under a slight positive pressure of CO_2 (using the reaction set up depicted for 'general CRE method H' on pages S6–7) and the progress of the reaction was monitored via TLC. After total of 18 h, the reaction was deemed to have gone to

completion by TLC analysis and colour change to orange solution was observed. The resulting mixture was diluted with CH_2Cl_2 (30 mL) and poured into separating funnel. The organic layer was washed with sat. brine (6 × 30 mL), dried over $MgSO_4$, filtered and concentrated under reduced pressure to yield a yellow oil (154 mg). The crude product was purified by flash column chromatography (SiO_2, 20 mm column, eluent: MeOH:EtOAc, 80:20) to afford the title compound **90** as a colourless oil (91.7 mg, 48%); R_f = 0.20 (MeOH:EtOAc, 90:10); IR (neat) ν_{max} / cm^{-1}: 2967m (C–H alkyl), 2793w (C–H alkyl), 1693s (C=O carbamate),1476m, 1455m, 1420s, 1364m, 1292m, 1244s, 1219m, 1184w, 1161m, 1141w, 1076m, 1035m, 987w, 973w, 952w, 909w, 852w, 780w, 749s, 613w, 531w; δ_H (400 MHz; $CDCl_3$) 4.32 – 4.25 (2H, m, C(2)H_2), 3.45 – 3.38 (2H, m, C(9)H_2), 3.27 (2H, q, *J* = 7.1 Hz, C(10)H_2), 2.47 – 2.38 (4H, m, C(4+7)H_2), 2.34 (2H, q, *J* = 7.2 Hz, C(5)H_2), 1.80 – 1.71 (2H, m, C(3)H_2), 1.52 – 1.43 (2H, m, C(8)H_2), 1.11 (3H, t, *J* = 7.1 Hz, C(11)H_3), 0.98 (3H, t, *J* = 7.1 Hz, C(6)H_3); δ_C (101 MHz; $CDCl_3$) 157.8 (CO, C1), 65.7 (CH_2, C2), 53.1 (CH_2, C7), 50.2 (CH_2, C4), 48.4 (CH_2, C10), 46.0 (CH_2, C9), 41.5 (CH_2, C5), 26.8 (CH_2, C3), 25.6 (CH_2, C8), 13.8 (CH_3, C11), 12.1 (CH_3, C6); HRMS (ESI): calcd. for $C_{11}H_{23}N_2O_2$, 215.1754. Found: $[MH]^+$, 215.1753 (0.6 ppm error).

1,7-Dimethyl-4,5,6,7-tetrahydrobenzo[d][1,3,6]oxadiazonin-2(1H)-one (91)

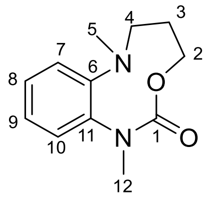

Triphosgene (227 mg, 0.766 mmol) was added in a single portion to a pale yellow solution of 3-(methyl(2-(methylamino)phenyl)amino)propan-1-ol **24** (372 mg, 1.90 mmol), anhydrous Et_3N (795 μL, 5.70 mmol) in anhydrous CH_2Cl_2 (38.0 mL) under Ar at RT. An immediate a colour change to a yellow solution was noted along with the liberation of fumes. The resulting solution was stirred at RT overnight under Ar. After 18 h, the reaction was deemed to have gone to completion by TLC analysis. The resulting mixture was quenched with sat. $NaHCO_{3(aq)}$ (30 mL), before transferred into separating funnel. The aqueous phase was extracted with CH_2Cl_2 (3 × 30 mL) and combined organic layers were collected before dried over $MgSO_4$, filtered and concentrated under reduced pressure to yield a pale-yellow oil (1.16 g). The crude product was purified by flash column chromatography (SiO_2, 20 mm column, eluent: EtOAc:*n*–hexane, 50:50) to afford carbamate **91** as a colourless oil (400 mg, 96%); R_f = 0.43

(EtOAc:*n*–hexane, 50:50); IR (thin film) ν_{max} / cm ^{1}: 3009w (C–H alkyl), 2955w (C–H alkyl), 2897w, 2843w, 2804w, 1701s (C=O carbamate), 1597m (CC aromatic), 1493s (CC aromatic) 1462w, 1421w, 1383w, 1372w, 1359m, 1338m, 1299m, 1278m, 1248m, 1216w, 1175w, 1153m, 1120w, 1080w, 1047w, 1023m, 968w, 919w, 900w, 827w, 804w, 745s, 706w, 666s, 652m, 587m, 566w, 547m, 530w, 506w, 478w; δ_H (400 MHz; $CDCl_3$) 7.27 – 7.20 (1H, m, C(7)H), 7.24 – 7.09 (3H, m, C(8 + 9 + 10)H), 4.60 – 4.50 (1H, m, C(2)**H**H'), 3.79 – 3.69 (1H, m, C(2)H**H'**), 3.18 – 3.14 (1H, m, C(4)**H**H'), 3.13 (3H, s, C(12)H_3), 2.94 – 2.83 (1H, m, C(4)**H**H'), 2.50 (3H, s, C(5)H_3), 1.61 – 1.40 (2H, m, C(3)H_2); δ_C (101 MHz; $CDCl_3$) 159.6 (CO, C1), 148.2 (C, C6), 144.2 (C, C11), 126.7 (CH), 126.5 (CH), 125.4 (CH), 124.4 (CH), 66.5 (CH_2, C2), 55.3 (CH_2, C4), 47.4 (CH_3, C5), 37.5 (CH_3, C12), 27.0 (CH_2, C3); HRMS (ESI): calcd. for $C_{12}H_{17}N_2O_2$, 221.1285. Found: $[MH]^+$, 221.1291 (−2.9 ppm error), calcd. for $C_{12}H_{16}N_2NaO_2$, 243.1104. Found: $[MNa]^+$, 243.1109 (−1.9 ppm error).

3-(Phenylamino)propan-1-ol (S132)

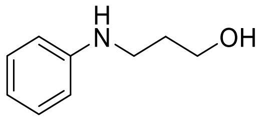

To a stirring mixture of phenyl iodide (15.0 mL, 134.0 mmol) and copper (I) iodide (2.56 g, 13.4 mmol), potassium hydroxide (15.04 g, 267.1 mmol) was added and stirred at room temperature for 10 minutes under argon. 3-Aminopropanol (30.76 mL, 402.2 mmol) was then added to the reaction mixture and stirred at room temperature for 16 hours under argon. The reaction mixture was diluted with water (150 mL), extracted with DCM (3 × 100 mL). The combined organic phases were then washed with brine (50 mL), dried with sodium sulphate, filtered, concentrated under vacuum to afford the title compound **S132** as a brown oil (18.03 g, 89%). R_f 0.17 (7:3 hexane:ethyl acetate); ν_{max}/cm^{-1} (thin film) 3359, 2939, 1603, 1508, 1320, 1261, 1061, 750, 694, 507; δ_H (400 MHz, $CDCl_3$) 7.22 – 7.16 (2H, m, Ar**H**), 6.77 – 6.71 (1H, m, Ar**H**), 6.71 – 6.65 (1H, m, Ar**H**), 3.82 (2H, t, *J* = 6.0 Hz, C**H**$_2$OH), 3.29 (2H, t, *J* = 6.5 Hz, C**H**$_2$NH), 1.90 (2H, tt, *J* = 6.5, 6.5 Hz, C**H**$_2$$CH_2$OH); δ_C (101 MHz, $CDCl_3$) 148.1 (Ar**C**), 129.4 (Ar**C**), 118.1 (Ar**C**), 113.5 (Ar**C**), 61.8 (**C**H_2OH), 42.4 (**C**H_2NH), 31.9 (**C**$H_2$$CH_2$OH); HRMS (ESI) calcd. for $C_9H_{14}NO$ 152.1070. Found $[MH]^+$ 152.1067 (1.9 ppm error).

***tert*-Butyl (3-hydroxypropyl)(phenyl)carbamate (S133)**

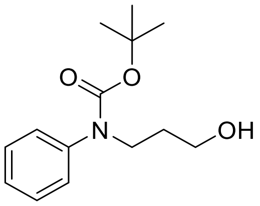

To a stirring solution of **S132** (3.212 g, 21.2 mmol) and di-*tert*-butyl decarbonate (5.099 g, 23.4 mmol) in DCM (200 mL), triethylamine (3.26 mL, 23.4 mmol) was added and stirred at 60 °C under argon for 72 h. The reaction was concentrated under vacuum and purified via flash column chromatography (7:3 hexane:ethyl acetate) to afford the title compound **S133** as a yellow oil (4.050 g, 69 %). R_f 0.53 (1:1 hexane:ethyl acetate); ν_{max}/cm^{-1} (thin film) 3440, 2979, 1673, 1598, 1496, 1455, 1393, 1367, 1301, 1266, 1163, 1072, 1007, 868, 734, 697; δ_H (400 MHz, $CDCl_3$) 7.30 (2H, t, *J* = 8.0 Hz, Ar**H**), 7.18 (1H, tt, *J* = 7.5, 1.5 Hz, Ar**H**), 7.11 (2H, d, *J* = 8.0 Hz, Ar**H**), 3.75 (2H, t, *J* = 6.5 Hz, C**H**$_2$OH), 3.62 (2H, t, *J* = 6.0 Hz, C**H**$_2$NH), 1.65 (2H, tt, *J* = 6.0, 6.0 Hz, C**H**$_2$CH$_2$OH), 1.37 (9H, s, **C**H$_3$); δ_C (101 MHz, $CDCl_3$) 155.8 (**C**O), 142.0 (Ar**C**), 128.8 (Ar**C**), 127.2 (Ar**C**), 126.3 (Ar**C**), 8.05 (**C**CH$_3$), 58.7 (**C**H$_2$NH), 46.2 (**C**H$_2$OH), 30.8 (**C**H$_2$CH$_2$OH), 28.2 (**C**H$_3$); **HRMS** (ESI) calcd. for $C_{14}H_{21}NNaO_3$ 274.1414. Found $[MNa]^+$ 274.1416 (−1.2 ppm error).

***tert*-Butyl (3-oxopropyl)(phenyl)carbamate (S134)**

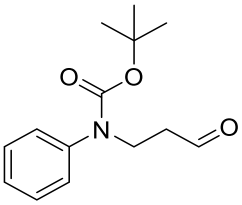

To a stirring solution of **S133** (2.99 g, 11.9 mmol) in DCM (120 mL), Dess–Martin periodinane (5.59 g, 13.2 mmol) was added and stirred at room temperature for 3 h. The reaction was quenched with sat. aq. sodium thiosulphate (100 mL) and sat. aq. sodium hydrogen carbonate (100 mL), then extracted with diethyl ether (3 × 100 mL). The organic phases were combined and washed with brine (50 mL), dried with sodium sulphate, filtered, concentrated under vacuum and purified via flash column chromatography (7:3 hexane:ethyl acetate) to afford the title compound **S134** as a yellow oil (2.882 g, 97 %). R_f 0.46 (7:3 hexane:ethyl acetate); ν_{max}/cm^{-1} (thin film) 2977, 2731, 1690, 1598, 1496, 1455, 1390, 1366, 1303, 1251, 1166, 1146, 759, 697; δ_H (400 MHz, $CDCl_3$) 9.75 (1H, t, *J* = 2.0 Hz, C**H**O), 7.30 – 7.37 (2H, m, Ar**H**), 7.25 – 7.19 (1H, m, Ar**H**), 7.15 (2H, d, *J* = 8.0, Ar**H**), 3.99 (2H, t, *J* = 7.0 Hz, NC**H**$_2$), 2.69 (2H, td, *J* = 7.0,

2.0 Hz, NCH_2CH_2), 1.40 (9H, s, CH_3); δ_C (101 MHz, $CDCl_3$) 200.8 (**C**HO), 154.7 (**C**O), 142.0 (Ar**C**), 129.1 (Ar**C**), 127.3 (Ar**C**), 126.6 (Ar**C**), 80.8 (**C**CH_3), 44.2 (N**C**H_2), 43.3 (NCH_2**C**H_2), 28.4 (**C**H_3); HRMS (ESI) calcd. for $C_{14}H_{19}NNaO_3$ 272.1257. found $[MNa]^+$ 272.1262 (–3.2 ppm error).

***tert*-Butyl (3-(benzyl(3-hydroxypropyl)amino)propyl)(phenyl)carbamate (S135)**

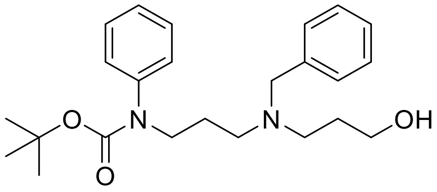

To a stirring solution of **S134** (587 mg, 2.40 mmol) and benzyl aminopropanol (0.460 mL, 2.80 mmol) in DCM (20 mL), sodium triacetoxyborohydride (745 mg, 3.50 mmol) was added and stirred at room temperature for 16 h. The reaction was neutralised with sat. aq. sodium hydrogen carbonate (50 mL), extracted with DCM (3 × 50 mL). The organic phases were combined and dried with sodium sulphate, filtered, concentrated under vacuum and purified via flash column chromatography (ethyl acetate) to afford the title compound **S135** as a yellow oil (451 mg, 48%). R_f 0.33 (ethyl acetate); ν_{max}/cm^{-1} (thin film) 3406, 2928, 1694, 1597, 1495, 1454, 1391, 1366, 1299, 1251, 1171, 1149, 1072, 858, 7335, 697; δ_H (400 MHz, $CDCl_3$) 7.35 – 7.22 (7H, m, Ar**H**), 7.21 – 7.15 (1H, m, Ar**H**), 7.07 (2H, d, *J* = 8.0 Hz, Ar**H**), 3.71 (2H, t, *J* = 5.5 Hz, C**H**$_2$OH), 3.62 (2H, t, *J* = 7.0 Hz, NPhC**H**$_2$), 3.54 (2H, s, ArC**H**$_2$), 2.62 (2H, t, *J* = 5.5 Hz, C**H**$_2CH_2CH_2OH$), 2.44 (2H, t, *J* = 7.5 Hz, $NPhCH_2CH_2C$**H**$_2$), 1.75 (2H, tt, *J* = 7.5, 7.5 Hz, $NPhCH_2$C**H**$_2$), 1.70 (2H, tt, *J* = 5.5, 5.5 Hz, C**H**$_2CH_2OH$), 1.39 (9H, s, C**H**$_3$); δ_C (101 MHz, $CDCl_3$) 154.8 (**C**O), 142.4 (Ar**C**), 129.3 (Ar**C**), 128.9 (Ar**C**), 128.6 (Ar**C**), 127.4 (Ar**C**), 127.2 (Ar**C**), 126.2 (Ar**C**), 80.3 (**C**CH_3), 63.7 (**C**H_2OH), 58.9 (Ph**C**H_2), 53.7 (**C**$H_2CH_2CH_2OH$), 51.3 ($NPhCH_2CH_2$**C**H_2), 48.2 (NPh**C**H_2), 28.4 (**C**H_3), 28.2 (**C**H_2CH_2OH), 25.8 ($NPhCH_2$**C**H_2); HRMS (ESI) calcd. for $C_{24}H_{35}N_2O_3$ 399.2642. Found $[MH]^+$ 399.2650 (−2.3 ppm error).

3-(Benzyl(3-(phenylamino)propyl)amino)propan-1-ol (S136)

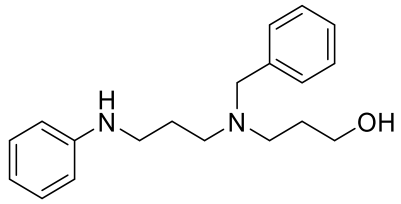

To a stirring solution of **S135** (54 mg, 0.1 mmol) in diethyl ether (3 mL), 4N aq. HCl in dioxane (0.31 mL, 1.2 mmol) was added and stirred at room temperature for 16 hours. The reaction was neutralised with sodium hydrogen carbonate (20 mL), extracted with diethyl ether (3 × 20 mL). The combined organic phases were then dried with sodium sulphate, filtered, concentrated under vacuum and purified via flash column chromatography (ethyl acetate) to afford the title compound **S136** as a colourless film (36 mg, 89%). R_f 0.15 (ethyl acetate); ν_{max}/cm^{-1} (thin film) 3342, 3027, 2931, 2829, 1602, 1506, 1453, 1319, 1265, 1067, 732, 693, 510; δ_H (400 MHz, $CDCl_3$) 7.38 – 7.26 (5H, m, Ar**H**), 7.19 – 7.12 (2H, m, Ar**H**), 6.68 (1H, d, *J* = 7.5 Hz, Ar**H**), 6.53 (2H, d, *J* = 8.0 Hz, Ar**H**), 3.75 (2H, t, *J* = 5.0 Hz, C**H**$_2$OH), 3.59 (2H, s, ArC**H**$_2$), 3.12 (2H, t, *J* = 6.5 Hz, NHC**H**$_2$), 2.68 (2H, t, *J* = 6.0 Hz, C**H**$_2$CH$_2$CH$_2$OH), 2.55 (2H, t, *J* = 7.5 Hz, NHCH$_2$CH$_2$C**H**$_2$), 1.83 (2H, tt, *J* = 7.0 Hz, NHCH$_2$C**H**$_2$), 1.77 (2H, tt, *J* = 5.5 Hz, C**H**$_2$CH$_2$OH); δ_C (101 MHz, $CDCl_3$) 148.3 (Ar**C**), 138.3 (Ar**C**), 129.4 (Ar**C**), 129.3 (Ar**C**), 128.7 (Ar**C**), 127.5 (Ar**C**), 117.2 (Ar**C**), 112.8 (Ar**C**), 64.1 (**C**H$_2$OH), 59.0 (Ph**C**H$_2$), 54.2 (**C**H$_2$CH$_2$CH$_2$OH), 51.4 (NHCH$_2$CH$_2$**C**H$_2$), 41.9 (NH**C**H$_2$), 28.1 (**C**H$_2$CH$_2$OH), 26.4 (NHCH$_2$**C**H$_2$); HRMS (ESI) calcd. for $C_{19}H_{27}N_2O$ 299.2118. Found $[MH]^+$ 299.2119 (−0.6 ppm error).

7-Benzyl-3-phenyl-1,3,7-oxadiazecane-2-thione (92)

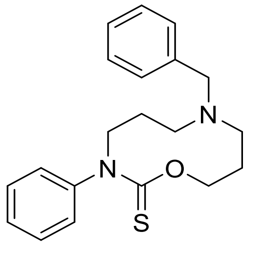

To a stirring solution of **S136** (69.4 mg, 0.2 mmol) and DMAP (143 mg, 1.2 mmol) in dry DCM (2.33 mL), thiophosgene (21 µL, 0.3 mmol) was added and stirred at room temperature for 16 hours under argon. The reaction was diluted with water (10 mL), extracted with DCM (3 × 20 mL). The organic phases were combined, dried with sodium sulphate, filtered, concentrated under vacuum and purified via flash column chromatography (1:1 hexane:ethyl acetate) to

afford the title compound **92** as a colourless film (28.8 mg, 36%). R_f 0.85 (1:1 hexane:ethyl acetate); ν_{max}/cm^{-1} (thin film) 2923, 2797, 1597, 1494, 1457, 1415, 1302, 1286, 1203, 1189, 1065, 1021, 909, 798, 725, 694, 578; δ_H (400 MHz, $CDCl_3$) 7.49 – 7.41 (4H, m, Ar**H**), 7.40 – 7.32 (3H, m, Ar**H**), 7.30 – 7.24 (3H, m, Ar**H**), 4.73 (2H, s (Br), OC**H**$_2$), 4.16 (2H, s (Br), NPhC**H**$_2$), 3.59 (2H, s, PhC**H**$_2$), 2.62 – 2.50 (4H, m, BnN(C**H**$_2$)$_2$), 1.95 – 1.82 (2H, m, OCH$_2$C**H**$_2$), 1.75 – 1.66 (2H, m, NPhCH$_2$C**H**$_2$); δ_C (101 MHz, $CDCl_3$) 190.3 (**C**S), 144.8 (Ar**C**), 140.3 (Ar**C**), 129.7 (Ar**C**), 128.6 (Ar**C**), 128.5 (Ar**C**), 128.2 (Ar**C**), 128.0 (Ar**C**), 127.0 (Ar**C**), 71.8 (O**C**H$_2$), 60.0 (Ph**C**H$_2$), 53.5 (OCH$_2$CH$_2$**C**H$_2$), 50.1 (NPh**C**H$_2$), 47.9 (NPhCH$_2$CH$_2$**C**H$_2$), 26.9 (NPhCH$_2$**C**H$_2$), 26.4 (OCH$_2$**C**H$_2$); HRMS (ESI) calcd. for $C_{20}H_{25}N_2OS$ 341.1682. Found $[MH]^+$ 341.1692 (−3.1 ppm error).

2-(Phenyl(2-(phenylamino)ethyl)amino)ethan-1-ol (S137)

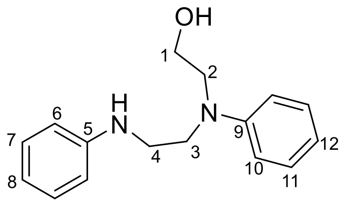

K_2CO_3 (2.44 g, 17.6 mmol) was added to a colourless solution of N1,N2-diphenylethane-1,2-diamine (2.25 g, 10.4 mmol) and 2-bromoethan-1-ol (881 mg, 7.05 mmol) in anhydrous MeCN (35.3 mL) at RT under Ar. The resulting pale yellow suspension was then heated to 90 °C under Ar. After 18 h, the reaction was deemed to have gone to completion by TLC, with a colour change to light pink suspension noted. The reaction mixture allowed to cool to RT, before being filtered through Celite and washed with EtOAc (3 × 30 mL). The resulting filtrate was concentrated under reduced pressure to yield a cherry red oil (3.30 g). The crude product was then purified by flash column chromatography (SiO_2, 65 mm column, eluent: EtOAc:*n*-hexane, 40:60) to afford alcohol **S137** as a pale yellow oil (158 mg, 9%); R_f = 0.37 (50:50 EtOAc:*n*–hexane); IR (neat) ν_{max} / cm^{-1}: 3364brw (N–H aniline/ O–H alcohol), 3023w (C–H alkyl), 2877w (C–H alkyl), 1726w, 1597s (CC aromatic), 1501s (CC aromatic), 1352w, 1322w, 1254w, 1215w, 1179w, 1123w, 1034m, 992m, 867w, 744s, 691s, 666w, 506m; δ_H (500 MHz; $CDCl_3$) 7.32 – 7.25 (2H, m, C(11)H), 7.25 – 7.17 (2H, m, C(7)H), 6.85 – 6.79 (2H, m, C(10)H), 6.82 – 6.74 (2H, m, C(8+12)H), 6.69 – 6.62 (2H, m, C(6)H), 3.77 (2H, t, *J* = 5.5 Hz, C(1)H$_2$), 3.61 (2H, t, *J* = 6.1 Hz, C(3)H$_2$), 3.52 (2H, t, *J* = 5.5 Hz, C(2)H$_2$), 3.39 (2H, t, *J* = 6.1 Hz, C(4)H$_2$), 3.20 (2H, s, OH+NH); δ_C (126 MHz; $CDCl_3$) 148.2 (C, C9), 148.0 (C, C5), 129.5 (2 × CH, C11), 129.4 (2 × CH, C7), 118.1 (CH, C8), 117.5 (CH, C12), 113.4 (2 × CH, C6), 113.3 (2 × CH, C10), 60.5 (CH$_2$, C1),

54.7 (CH_2, C2), 51.6 (CH_2, C3), 42.1 (CH_2, C4); HRMS (ESI): calcd. for $C_{16}H_{21}N_2O$, 257.1648. Found: $[MH]^+$, 257.1648 (−0.0 ppm error), calcd. for $C_{16}H_{20}NaN_2O$, 279.1468. Found: $[MNa]^+$, 279.1468 (−0.1 ppm error).

3,6-Diphenyl-1,3,6-oxadiazocane-2-thione (93)

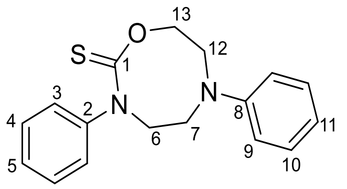

Thiophosgene (40.0 µL, 0.524mmol) was added via syringe over a period of 20 sec to a pale yellow solution of 2-(phenyl(2-(phenylamino)ethyl)amino)ethan-1-ol **S137** (112 mg, 0.437mmol), anhydrous Et_3N (213 µL, 1.53 mmol) in anhydrous CH_2Cl_2 (9.0 mL) under Ar at RT. An immediate colour change to dark purple was noted along with liberation of grey fumes ($HCl_{(g)}$). The resulting black solution was stirred at RT overnight under Ar and progress of the reaction was monitored by TLC. After total of 18 h, the reaction was judged to be complete, based on TLC analysis. The resulting mixture was quenched with sat. $NaHCO_{3(aq)}$ (20 mL), before transferred into separating funnel. The aqueous phase was extracted with CH_2Cl_2 (3 × 30 mL) and combined organic layers were dried over $MgSO_4$, filtered and concentrated under reduced pressure to yield a brown oil (360 mg). The crude product was purified by flash column chromatography (SiO_2, 30 mm column, eluent: EtOAc:n-hexane, 20:80) to afford the title compound **93** as a white solid (97 mg, 74%); R_f = 0.60 (50:50 EtOAc:*n*–hexane); Melting Point: 144 – 146 °C (from EtOAc:n-hexane); IR (solid state) ν_{max} / cm^{-1}: 2990w (C–H alkyl), 2948w (C–H alkyl), 1684w, 1595s (CC aromatic), 1574w (CC aromatic), 1491m, 1477s, 1452m, 1441m, 1410s, 1390w, 1369m, 1355m, 1332m, 1305s, 1268w, 1239m, 1208m, 1197m, 1183w, 1153s, 1112w, 1071m, 1057s, 1046m, 1022s, 998s, 967w, 947w, 878w, 861m, 807w, 755s, 722w, 713w, 694s, 660m, 621w, 579m, 564w, 544w, 523m, 485m, 454w; δ_H (400 MHz; Acetone-d_6) 7.47 – 7.40 (2H, m, C(3)H), 7.39 – 7.31 (3H, m, C(4+5)H), 7.27 – 7.18 (2H, m, C(10)H), 6.91 – 6.84 (2H, m, C(9)H), 6.75 – 6.67 (1H, m, C(11)H), 4.78 (2H, t, *J* = 5.1 Hz, C(13)H_2), 4.20 (2H, br, s, C(6 or 7)H_2) 3.87 – 3.80 (4H, m, C(6 or 7+12)H_2); δ_C (101 MHz; Acetone-d_6) 194.8 (CS, C1), 148.1 (C, C8), 146.4 (C, C2), 130.3 (2 × CH, C10), 130.1 (2 × CH, C3), 128.5 (CH, C5), 127.9 (2 × CH, C4), 118.0 (CH, C11), 113.6 (2 × CH, C9), 73.7 (CH_2, C13), 54.7 (CH_2), 52.3 (CH_2), 51.1 (CH_2); HRMS (ESI): calcd. for $C_{17}H_{19}N_2OS$,299.1213. Found: $[MH]^+$, 257.1648

(−0.1 ppm error), calcd. for $C_{17}H_{18}N_2NaOS$, 321.1032. Found: $[MNa]^+$, 321.1025 (2.2 ppm error), calcd. for $C_{17}H_{18}KN_2OS$, 337.0771. Found: $[MK]^+$, 337.0772 (−0.2 ppm error).

X-ray crystallographic data for this compound can be accessed via www.ccdc.cam.ac.uk/data_request/cif (CCDC 2221454)

3-(Phenyl(3-(phenylamino)propyl)amino)propan-1-ol (S138)

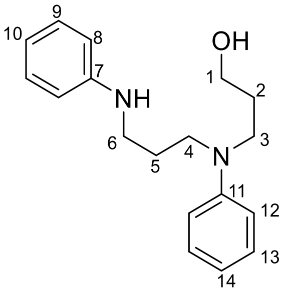

K_2CO_3 (2.29 g, 16.6 mmol) was added to a pale-yellow solution of N^1,N^3-diphenylpropane-1,3-diamine (1.88 g, 8.29 mmol) and 3-bromopropan-1-ol (500 µL, 5.53 mmol) in anhydrous MeCN (19.0 mL) at RT under Ar. The resulting dark brown suspension was then heated to 95 °C under Ar. After 18 h, the reaction was deemed to have gone to completion by TLC. The reaction mixture allowed to cool to RT, before was quenched with H2O (30 mL). The diluted reaction mixture was poured into separating funnel and the aqueous layer was extracted with Et_2O (3 × 30 mL). The combined organic layers were washed sat. brine (3 × 30 mL). The resulting layer was collected and dried over MgSO4, filtered and concentrated under reduced pressure to yield a brown oil (2.50 g). The crude product was purified by flash column chromatography (SiO2, 50 mm column, eluent: EtOAc:*n*-hexane, 50:50) to afford alcohol **S138** as a pale yellow oil (1.97 g, 84%); R_f = 0.34 (EtOAc:*n*–hexane, 50:50); IR (neat) ν_{max} / cm^{-1}: 3385brw (O–H alcohol), 2941w (C–H alkyl), 2875w (C–H alkyl), 1729w, 1599s (CC aromatic), 1504s (CC aromatic), 1476w, 1431w, 1372w, 1318w, 1249m, 1217m, 1179w, 1042m, 990w, 917w, 867w, 745s, 692s, 683w, 608w, 509m; δ_H (400 MHz; $CDCl_3$) 7.38 – 7.33 (2H, m, C(13)H), 7.32 – 7.27 (2H, m, C(9)H), 6.89 – 6.78 (4H, m, C(8+12)H), 6.74 – 6.68 (2H, m, C(10+14)H), 3.73 (2H, t, *J* = 6.0 Hz, C(1)H_2), 3.53 – 3.43 (4H, m, C(3+4)H_2), 3.23 (2H, t, *J* = 6.8 Hz, C(6)H_2), 2.01 – 1.93 (2H, m, C(5)H_2), 1.92 – 1.85 (2H, m, C(2)H_2); δ_C (101 MHz; $CDCl_3$) 148.2 (C, C11), 148.0 (C, C7), 129.2 (2 × CH, C13), 129.3 (2 × CH, C9), 117.4 (CH, C10), 116.5 (CH, C14), 113.0 (2 × CH, C12), 112.9 (2 × CH, C8), 60.5 (CH_2, C1), 49.0 (CH_2, C4), 48.2 (CH_2, C3), 41.8 (CH_2, C6), 29.8 (CH_2, C2), 26.9 (CH_2, C5); HRMS (ESI): calcd. for $C_{18}H_{25}N_2O$, 285.1961. Found: $[MH]^+$, 285.1962 (−0.1 ppm error).

3,7-Diphenyl-1,3,7-oxadiazecan-2-one (94)

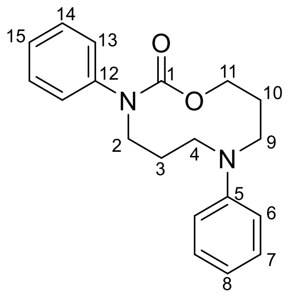

Triphosgene (240 mg, 0.807 mmol) was added in a single portion to a pale yellow solution of 3-(phenyl(3-(phenylamino)propyl)amino)propan-1-ol **S138** (574 mg, 2.02mmol), dry Et_3N (986 µL, 7.10 mmol) in anhydrous CH_2Cl_2 (40.0 mL) under Ar at RT. An immediate colour change to orange solution was noted along with liberation of fumes ($HCl_{(g)}$). The resulting solution was stirred at RT overnight under Ar. After 18 h, the reaction was judged to be complete, based on TLC analysis with colour changed to a pale brown solution was noted. The resulting mixture was quenched with sat. $NaHCO_{3(aq)}$ (30 mL), before transferred into separating funnel. The aqueous phase was extracted with CH_2Cl_2 (3 × 40 mL) and combined organic layers were dried over $MgSO_4$, filtered and concentrated under reduced pressure to yield a yellow oil (1.37 g). The crude product was purified by flash column chromatography (SiO_2, 30 mm column, eluent: EtOAc:*n*–hexane, 35:65) to afford carbamate **94** as a colourless oil (487 mg, 78%); R_f = 0.53 (EtOAc:*n*–hexane, 50:50); IR (neat) ν_{max} / cm^{-1}: 2957w (C–H alkyl), 2830w (C–H alkyl), 1699s (CO carbamate) 1598s (CC aromatic), 1495s, 1463m, 1452m, 1407m, 1364w, 1342w, 1331w, 1282w, 1247m, 1231m, 1208m, 1147m, 1123w 1065m, 1027w, 1005w, 991w, 870w, 764s, 709s, 694s, 665w, 650w, 618w, 595w, 541w, 521w, 4780w; δ_H (400 MHz; $CDCl_3$) 7.37 – 7.24 (4H, m, C(7+14)H), 7.24 – 7.16 (1H, m, C(15)H), 7.12 – 7.05 (2H, m, C(13)H) 6.96 – 6.85 (3H, m, C(6+8)H), 4.58 – 4.50 (2H, m, C(11)H_2), 3.93 – 3.86 (2H, m, C(2)H_2), 3.52 – 3.42 (4H, m, C(4+9)H_2), 2.20 – 2.11 (2H, m, C(10)H_2), 1.89 – 1.79 (2H, m, C(3)H_2); δ_C (101 MHz; $CDCl_3$) 156.0 (CO, C1), 150.6 (C, C5), 142.0 (C, C12), 128.9 (2 × CH, C7), 128.8 (2 × CH, C14), 126.9 (2 × CH, C13), 126.1 (CH, C15), 118.4 (CH, C8), 116.0 (2 × CH, C6), 66.3 (CH_2, C11), 58.9 (CH_2, C4), 50.2 (CH_2, C9), 49.5 (CH_2, C2), 29.0 (CH_2, C10), 26.4 (CH_2, C3); HRMS (ESI): calcd. for $C_{19}H_{23}N_2O_2$, 311.1754. Found: $[MH]^+$, 311.1758 (−1.4 ppm error), calcd. for $C_{19}H_{22}N_2NaO_2$, 333.1573. Found: $[MNa]^+$, 333.1575 (−0.5 ppm error), calcd. for $C_{19}H_{22}N_2KO_2$, 349.1313. Found: $[MK]^+$, 349.1315 (−0.7 ppm error).

1,7-Dimethyl-4,5,6,7-tetrahydrobenzo[d][1,3,6]oxadiazonine-2(1H)-thione (95)

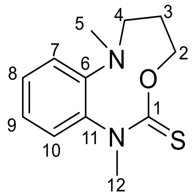

Thiophosgene (108 µL, 1.41 mmol) was added in a single portion via syringe to a pale yellow solution of 3-(methyl(2-(methylamino)phenyl)amino)propan-1-ol **24** (228 mg, 1.18 mmol), anhydrous Et_3N (574 µL, 4.11 mmol) in anhydrous CH_2Cl_2 (24.0 mL) under Ar at RT. An immediate a colour of the reaction mixture changed over 2 min from yellow to dark green and finally black brown was noted along with the liberation of fumes. The resulting solution was stirred at RT overnight under Ar. After total of 18 h, the reaction was deemed to have gone to completion by TLC analysis. The resulting mixture was quenched with sat. $NaHCO_{3(aq)}$ (30 mL), before transferred into separating funnel. The aqueous phase was extracted with CH_2Cl_2 (3 × 30 mL) and combined organic layers were collected, dried over $MgSO_4$, filtered and concentrated under reduced pressure to yield a brown oil (423 mg). The crude product was purified by flash column chromatography (SiO_2, 35 mm column, eluent: EtOAc:*n*–hexane, 40:60) to afford thiocarbamate **95** as an orange oil (238 mg, 85%); R_f = 0.61 (EtOAc:*n*–hexane, 50:50); IR (thin film) ν_{max} / cm^{-1}: 3040w (C–H alkyl), 2953brw (C–H alkyl), 1734w, 1598m (CC aromatic), 1596s (C=S thiocarbamate), 1571w (CC aromatic),1505s (CC aromatic), 1495s, 1466s,1412m, 1360m, 1322m, 1303m, 1265m,1230s, 1206s, 1173s, 1117m, 1066m, 1017m, 987w, 926w, 860w, 837w, 746s, 732s, 692s, 617w, 570w, 544w, 513w, 473w; δ_H (400 MHz; $CDCl_3$) 7.42 – 7.07 (4H, m, C(7 + 9 + 10)), [5.14 – 5.04 (m), 4.69 – 4.42 (m), 4.19 – 4.09 (m), 4.07 – 3.98 (m), 3.98 – 3.89 (m), 3.86 – 3.71 (m), 3.61 (s, 3H), 3.51 – 3.40 (m), 3.27 – 3.17 (m), 3.09 – 2.97 (m), 2.56 (s, 3H), 2.28 – 2.04 (m), 1.83 – 1.70 (m), 1.64 – 1.46 (m), 1.39 – 0.97 (m)] = 12H (3 × CH_2 + 2 × CH_3). Note, due to the ^{1}H NMR spectrum suffering from severe rotameric broadening, the ^{1}H NMR signals in this region could not be confidently assigned. The ^{13}C NMR data proved to be much more informative in confirming the assigned structure; δ_C (101 MHz; $CDCl_3$) 194.2 (CS), 147.6 (C), 144.5 (C), 127.8 (CH), 126.7 (CH), 125.4 (CH), 124.9 (CH), 72.6 (CH_2), 55.6 (CH_2), 46.6 (CH_3), 42.7 (CH_3), 27.0 (CH_2); HRMS (APCI): calcd. for $C_{12}H_{17}N_2OS$, 237.105611. Found: $[MH]^+$, 237.104996 (−2.6 ppm error).

2,6-Divinylpyridine (S139)

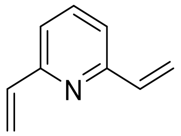

To a stirring solution of indium (III) chloride (185 mg, 0.8 mmol) in dry THF (10 mL), vinylmagnesium bromide (3.40 mL, 3.4 mmol, 1M solution in hexane) was added and stirred at –78 °C for 30 minutes under argon. The reaction was warmed to room temperature and stirred for a further 30 minutes. The reaction mixture was added to a solution of 2,6-dibromopyridine (237 mg, 1.0 mmol) and tetrakis(triphenylphosphine)palladium(0) (153 mg, 0.1 mmol) in dry THF (10 mL) and the reaction was heated to 60 °C for 1 hour. The reaction mixture was cooled to room temperature and quenched with sat. aq. sodium hydrogen carbonate (10 mL) and extracted with diethyl ether (3 × 50 mL). The combined organic phases were then washed with water (50 mL), brine (50 mL), dried with sodium sulphate, filtered, concentrated under vacuum and purified via flash column chromatography (49:1 hexane:diethyl ether) to afford the title compound **S139** as a colourless oil (77 mg, 59%). R_f 0.12 (49:1 hexane:diethyl ether); ν_{max}/cm^{-1} (thin film) 3094, 3056, 3016, 1862, 1573, 1565, 1449, 1159, 987, 923, 824, 744; δ_H (400 MHz, $CDCl_3$) 7.61 (1H, t, *J* = 8.0 Hz, Ar**H**), 7.22 (2H, d, *J* = 7.5 Hz, Ar**H**), 6.83 (2H, dd, *J* = 10.5, 1.0 Hz, CH_2C**H**), 6.25 (2H, dd, *J* = 17.5, 1.5 Hz, C**H**$_2$CH), 5.48 (2H, dd, *J* = 10.5, 1.5 Hz, C**H**$_2$CH); δ_C (101 MHz, $CDCl_3$) 155.5 (Ar**C**), 137.2 (CH_2**C**H), 137.0 (Ar**C**), 120.1 (Ar**C**), 118.4 (**C**H_2CH); HRMS (APCI) calcd. for $C_9H_{10}N$ 132.0808. Found $[MH]^+$ 132.0802 (3.5 ppm error). The spectroscopic data match those reported in the literature.[15]

***N*,*N*'-(Pyridine-2,6-diylbis(ethane-2,1-diyl))dianiline (S140)**

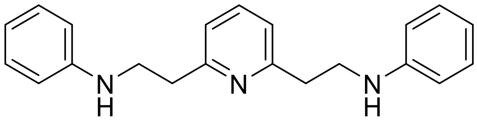

To a stirring solution of 2,6-divinylpyridine **S139** (279 mg, 2.10 mmol) in acetonitrile (2 mL), aniline (0.460 mL, 5.00 mmol) and scandium triflate (229 mg, 0.500 mmol) were added and stirred at 70 °C for 3 days. The reaction mixture was concentrated under vacuum and purified via flash column chromatography (4:1 hexane:ethyl acetate) to afford the title compound **S140** as a yellow oil (578 mg, 86%); R_f 0.08 (4:1 hexane:ethyl acetate); ν_{max}/cm^{-1} (thin film) 3401, 3051, 3021, 2925, 1602, 1506, 1456, 1320, 1260, 1179, 991, 748, 692, 508; δ_H (400 MHz, $CDCl_3$) 7.56 (1H, t, *J* = 7.5 Hz, Ar**H**), 7.21 (4H, m, Ar**H**), 7.06 (2H, d, *J* = 7.5 Hz, Ar**H**), 6.74 (2H, t, *J* = 7.5 Hz, Ar**H**), 6.66 (4H, d, *J* = 8.0 Hz, Ar**H**), 4.27 (2H, s(Br), N**H**), 3.58 (4H, t, *J* = 6.5 Hz, NHC**H**$_2$),

3.12 (4H, t, J = 6.5 Hz, NHC**H**$_2$); δc (101 MHz, $CDCl_3$) 159.5 (Ar**C**), 148.3 (Ar**C**), 137.1 (Ar**C**), 129.3 (Ar**C**), 121.1 (Ar**C**), 117.4 (Ar**C**), 113.0 (Ar**C**), 43.7 (NH**C**H$_2$), 37.4 (Ar**C**H$_2$); HRMS (ESI) calcd. for $C_{21}H_{24}N_3$ 318.1965. Found $[MH]^+$ 318.1966 (2.1 ppm error).

Medium-sized ring (96)

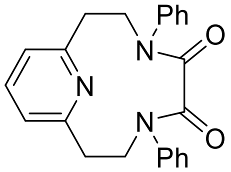

Oxalyl chloride (15.2 µL, 0.174 mmol) was added in a single portion via syringe to a solution of *N*,*N*'-(pyridine-2,6-diylbis(ethane-2,1-diyl))dianiline **S140** (50 mg, 0.158 mmol), dry Et_3N (110 µL, 0.788 mmol) in anhydrous CH_2Cl_2 (1.6 mL) under Ar at RT. The resulting solution was stirred at RT for 30 min, after which time the reaction was deemed to have gone to completion by TLC analysis. The resulting mixture was diluted with DCM (20 mL), washed with water (10 mL) and concentrated *in vacuo*. Purification by column chromatography (20:1 ethyl acetate:triethyl amine) afforded medium-sized ring **96** as a white solid (30 mg, 51%); R_f 0.60 (20:1 ethyl acetate:triethyl amine); M.p. 68–70 °C; ν_{max}/cm^{-1} (thin film) 1650s, 1494m, 1263w, 910w, 729m, 697s; δ_H (400 MHz, $CDCl_3$) 7.66 (2H, d, J = 7.8, Ar**H**), 7.50–7.25 (7H, m, Ar**H**), 6.98 (1H, d, J = 7.8 Hz, Ar**H**), 6.77 (2H, d, J = 7.8, Ar**H**), 6.72 (1H, d, J = 7.8 Hz, Ar**H**), 4.71–4.61 (2H, br m, NC**H₂**), 4.05–3.99 (2H, m, NC**H₂**), 3.22–3.17 (2H, m, ArC**H₂**), 2.66–2.61 (2H, br NC**H₂**); δ_C (101 MHz, $CDCl_3$) 165.1 (**C**O), 164.9 (**C**O), 158.8 (Ar**C**), 157.1 (Ar**C**), 139.8 (Ar**C**), 138.8 (Ar**C**), 137.1 (Ar**C**H), 130.0 (Ar**C**H), 129.7 (Ar**C**H), 129.2 (Ar**C**H), 129.0 (Ar**C**H), 128.1 (Ar**C**H), 127.5 (Ar**C**H), 121.8 (Ar**C**H) 121.5 (Ar**C**H), 50.4 (N**C**H$_2$), 47.3 (N**C**H$_2$), 36.8 (**C**H$_2$Ar), 35.2 (**C**H$_2$Ar); HRMS (APCI): calcd. for $C_{23}H_{22}N_3O_2$, 372.171843. Found: $[MH]^+$, 373.170653 (–3.2 ppm error).

Medium-sized ring (97)

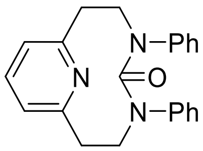

To a solution of N,N'-(pyridine-2,6-diylbis(ethane-2,1-diyl))dianiline **S140** (64 mg, 0.202 mmol) in DCM (2.0 mL) at RT, triethylamine (42.2 µL, 0.303 mmol) and triphosgene (23.9 mg, 0.0807 mmol) were added sequentially. The resulting mixture was stirred at RT for 18 h, quenched by the addition of water (20 mL), extracted with DCM (2 x 20 mL), dried over $MgSO_4$ and concentrated under vacuum. Purification by column chromatography (5:1 → 2:1 hexane:ethyl acetate) afforded the title compound **97** as a white solid (34 mg, 49%); R_f = 0.15 (1:1 hexane:diethyl ether); M.p. 66–68 °C; IR (thin film) ν_{max} / cm^{-1} 1646s, 1460s, 1326m, 1162m, 1003w, 744m, 694s. Note: both the ^1H and ^{13}C NMR spectra obtained for this product showed severe broadening of their NMR signals. δ_H (400 MHz, $CDCl_3$) 7.66 (1H, app t, *J* = 7.4, Ar**H**), 7.33−7.19 (8H, br m, Ar**H**), 4.30–3.60 (4H, br m, 2 x CH_2), 3.35–2.70 (4H, br m, 2 x CH_2); δ_C (101 MHz, $CDCl_3$) 160.4 (**C**O), 157.6 (Ar**C**), 144.6 (Ar**C**), 137.6 (Ar**C**), 124.2 (Ar**C**H), 123.5–122.5 (br m, Ar**C**H), 120.5 (Ar**C**H), 50.0 (N**C**H_2), 36.9 (**C**H_2Ar); HRMS (APCI): m/z calc. for $C_{22}H_{22}N_3O$: 344.175455, found: 344.175739 $[MH]^+$.

1,4,8-trimethyl-1,4,8-triazacycloundecane-2,3-dione (98)

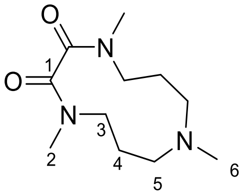

Oxalyl chloride (188 µL, 2.19 mmol) was added in a single portion via syringe to a colouless solution of N1,N3-dimethyl-N1-(3-(methylamino)propyl)propane-1,3-diamine (400 µL, 1.99 mmol), dry Et_3N (695 µL, 4.99 mmol) in anhydrous CH_2Cl_2 (40.0 mL) under Ar at RT. The resulting solution was stirred at RT overnight under Ar and the progress of the reaction was monitored by TLC. After total of 18 h, the reaction was deemed to have gone to completion by TLC analysis. The resulting mixture was concentrated under *vacuo* to yield a brown oil (730 mg). The crude product was purified by flash column chromatography (SiO_2, 40 mm column, eluent: MeOH:EtOAc, 0:100 to 30:70) to afford the title compound **98** as a white solid (321

mg, 71%); R_f = 0.08 (EtOAc); M.p. 135 – 136 °C (from *n*–hexane:Et_2O); IR (solid state) ν_{max} / cm^{-1}: 2947w, 2881w (C–H alkyl), 2803w, 2776w, 2240w, 1615s (C=O amide), 1532w, 1509w, 1452m, 1424m, 1401m, 1354m, 1314w, 1253w, 1263w, 1237m, 1218m, 1198w, 1132m, 1060m, 1031w, 1021m, 985w, 969w, 953w, 922w, 887w, 843w, 787w, 746s, 664m, 645w, 633m, 565w; δ_H (400 MHz; $CDCl_3$) 4.00 (2H, ddd, *J* = 14.6, 11.6, 2.8 Hz, 2 × C(3)H**H'**), 3.13 (2H, ddd, *J* = 14.6, 3.5, 3.5 Hz, 2 × C(3)**H**H'), 2.92 (6H, s, C(2)H_3), 2.36 (4H, dd, *J* = 6.7, 5.3 Hz, C(5)H_2), 1.94 (3H, s, C(6)H_3), 1.84 – 1.69 (2H, m, 2 × C(4)H**H'**), 1.47 – 1.34 (2H, m, 2 × C(4)**H**H'); δ_C (101 MHz; $CDCl_3$) 165.4 (CO, C1), 57.8 (CH_2, C5), 48.2 (CH_2, C3), 37.8 (CH_3, C6), 31.5 (CH_3, C2), 22.4 (CH_2, C4); HRMS (ESI): calcd. for $C_{11}H_{22}N_3O_2$, 228.1707. Found: $[MH]^+$, 228.1706 (0.1 ppm error), calcd. for $C_{11}H_{21}N_3NaO_2$, 250.1526. Found: $[MNa]^+$, 250.1525 (0.4 ppm error), calcd. for $C_{11}H_{21}KN_3O_2$, 266.1265. Found: $[MK]^+$, 266.1265 (0.0 ppm error).

X-ray crystallographic data for compound **98** can be accessed via www.ccdc.cam.ac.uk/data_request/cif (CCDC 2232114).

1,4,7-Trimethyl-1,4,7-triazonane-2,3-dione (99)

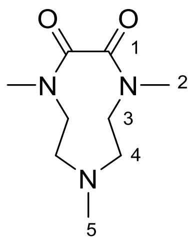

Oxalyl chloride (226 µL, 2.64 mmol) was added in a single portion via syringe to a solution of N[1],N[2]-dimethyl-N[1]-(2-(methylamino)ethyl)ethane-1,2-diamine (400 µL, 2.40 mmol), dry Et_3N (1.17 mL, 8.40 mmol) in anhydrous CH_2Cl_2 (48.0 mL) under Ar at RT. The resulting solution was stirred at RT overnight under Ar. After total of 18 h, the reaction was deemed to have gone to completion by TLC analysis. The resulting mixture was concentrated under *vacuo* to yield a brown oil (730 mg). The crude product was purified by flash column chromatography (SiO_2, 40 mm column, eluent: MeOH:EtOAc, 30:70) to afford the title compound **99** as a pale yellow oil (350 mg, 73%); R_f = 0.19 (MeOH:EtOAc, 30:70); IR (neat) ν_{max} / cm^{-1}: 3496brw, 2941w (C–H alkyl), 2805w, 1627s (C=O amide), 1511w, 1453m, 1420m, 1400m, 1359m, 1317w, 1277w, 1232m, 1203m, 1146m, 1128m, 1083w, 1067w, 1040w, 1003w, 946w, 979w, 946w, 866w, 784m, 742w, 675w, 646w, 497w; δ_H (400 MHz; $CDCl_3$) 3.56 (2H, ddd, *J* = 15.6, 9.8, 3.3 Hz, 2 × C(3)H**H'**), 3.25 – 3.13 (2H, m, 2 × C(3)**H**H'), 2.92 (6H, s, C(2)H_3), 2.70 (2H, ddd, *J* = 14.6, 9.7,

3.2 Hz, 2 × C(4)H**H'**), 2.59 (2H, dd, *J* = 14.3, 3.6 Hz, 2 × C(4)**H**H'), 2.46 (3H, s, C(5)H_3); δ_C (101 MHz; $CDCl_3$) 166.1 (CO, C1), 55.4 (CH_2, C4), 51.3 (CH_2, C3), 47.9 (CH_3, C5), 31.8 (CH_3, C2); HRMS (ESI): calcd. for $C_9H_{18}N_3O_2$, 200.1394. Found: $[MH]^+$, 200.1396 (−1.2 ppm error), calcd. for $C_9H_{17}N_3NaO_2$, 222.1213. Found: $[MNa]^+$, 222.1213 (−0.2 ppm error), calcd. for $C_9H_{17}KN_3O_2$, 238.0952. Found: $[MK]^+$, 238.0957 (−2.1 ppm error).

1,3,7-Trimethyl-1,3,7-triazecan-2-one (100)

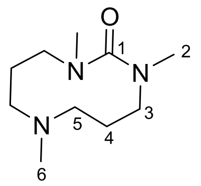

N^1,N^3-dimethyl-N^1-(3-(methylamino)propyl)propane-1,3-diamine (500 μL, 2.50 mmol) was added reaction flask (250 mL RBF) containing anhydrous DMF (50 mL). The resulting reaction vessel was evacuated under *vacuo* and backfilled with CO_2 (via balloon) 6 times. Next, EDC.HCl (719 mg, 3.75 mmol), HOBt (405 mg, 3.01 mmol) and dry DIPEA (1.52 mL, 3.14 mmol) were each added in single portion at RT, with a colour change to pale yellow solution noted. The resulting solution was stirred at RT overnight under a slight positive pressure of CO_2 (using the set up depicted for general CRE method H on pages S6–7) and the progress of the reaction was monitored via TLC. After total of 18 h, the reaction was deemed to have gone to completion by TLC analysis and colour change to pale orange solution was observed. The resulting mixture was diluted with CH_2Cl_2 (60 mL) and poured into separating funnel. The organic layer was washed sequentially with sat. $NaHCO_{3(aq)}$ (2 × 80 mL) and sat. brine (6 × 80 mL), before was dried over $MgSO_4$, filtered and concentrated under reduced pressure to yield an orange oil (510 mg). The crude product was purified by flash column chromatography (SiO_2, 40 mm column, eluent: MeOH:EtOAc, 90:10) to afford the title compound **100** as a colourless oil (434 mg, 87%); R_f = 0.04 (MeOH); IR (neat) ν_{max} / cm^{-1}: 3479m, 2918m (C–H alkyl), 2846m (C–H alkyl), 2784m (C–H alkyl), 1626s (C=O urea), 1492s, 1466m, 1451m, 1411w, 1388s, 1354s, 1313w, 1290w, 1273m, 1249w, 1231m, 1201m, 1156s, 1121w, 1096m, 1064m, 1045w, 1004w, 982m, 953w, 908w, 869m, 855w, 829w, 812w, 775w, 746m, 708w, 590w, 534w, 493w, 452w; δ_H (400 MHz; $CDCl_3$) 3.34 – 3.27 (4H, m, C(3)H_2), 2.76 (6H, s, C(1)H_3), 2.35 – 2.28 (4H, m, C(5)H_2), 2.11 (3H, s, C(6)H_3), 1.62 – 1.52 (4H, m, C(4)H_3); δ_C (101 MHz; $CDCl_3$) 164.8 (CO, C1), 55.2 (CH_2, C5), 48.2 (CH_2, C3), 42.4 (CH_3, C6), 37.0 (CH_3, C2), 24.8

(CH$_2$, C4); HRMS (ESI): calcd. for $C_{10}H_{22}N_3O$, 200.1757. Found: $[MH]^+$, 200.1755 (1.1 ppm error), calcd. for $C_{10}H_{21}N_3NaO$, 222.1577. Found: $[MNa]^+$, 222.1576 (0.6 ppm error).

***N*-phenyl-3-(phenylamino)-*N*-(2-(phenylamino)ethyl)propenamide (S141)**

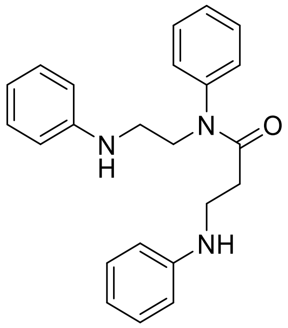

To a solution of N,N'-diphenylethylenediamine (1.03 g, 4.85 mmol) and 3-(phenylamino)propionic acid (160 mg, 0.969 mmol) in DCM (20 mL), DIPEA (0.340 mL, 1.94 mmol) was added followed by T3P 50% w/v in ethyl acetate (925 mg, 1.453 mmol). The resulting mixture was stirred at RT for 18 h, quenched by the addition of water (50 mL), extracted with DCM (2 x 50 mL) and concentrated under vacuum. Purification by column chromatography (10:1 → 2:1 → 1:1 hexane:ethyl acetate) afforded the title compound **S141** as a pale yellow oil (254 mg, 73%); R_f = 0.50 (1:1 hexane:ethyl acetate); IR (thin film) ν_{max} / cm^{-1} 3375brm 1639m, 1600s, 1493s, 1260m, 1026w, 747s, 692s; δ_H (400 MHz, CDCl$_3$) 7.38−7.30 (3H, m, Ar**H**), 7.19−7.03 (6H, m, Ar**H**), 6.73−6.64 (2H, m, Ar**H**), 6.56 (2H, d, *J* = 7.8 Hz, Ar**H**), 6.50 (2H, d, *J* = 7.8 Hz, Ar**H**), 3.97 (2H, t, *J* = 6.0, CH$_2$NPhCO), 3.39 (2H, t, *J* = 6.4, CH$_2$NHPh), 3.26 (2H, t, *J* = 6.0, CH$_2$NHPh), 2.34 (2H, t, *J* = 6.4, CH$_2$CO); δ_c (101 MHz, CDCl$_3$) 172.9 (**C**O), 148.2 (Ar**C**), 147.9 (Ar**C**), 142.0 (Ar**C**), 130.1 (Ar**C**H), 129.4 (Ar**C**H), 129.3 (Ar**C**H), 128.4 (Ar**C**H), 128.3 (Ar**C**H), 117.6 (Ar**C**H), 117.3 (Ar**C**H), 113.2 (Ar**C**H) 112.5 (Ar**C**H), 48.7 (N**C**H$_2$), 42.5 (N**C**H$_2$), 40.0 (N**C**H$_2$), 33.9 (**C**H$_2$CO); HRMS (ESI$^+$): m/z calc. for $C_{23}H_{26}N_3O$: 360.2076, found: 360.2070 $[MH]^+$ (–1.7 ppm error).

N1,N3-diphenyl-N1-(2-(phenylamino)ethyl)propane-1,3-diamine (S142)

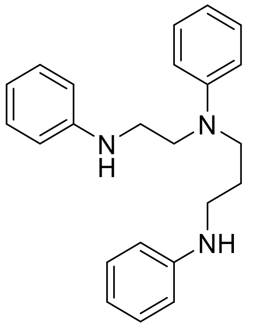

To a solution of *N*-phenyl-3-(phenylamino)-*N*-(2-(phenylamino)ethyl)propenamide (**S141**) (250 mg, 0.696 mmol) in THF (20 mL) at RT, $LiAlH_4$ powder (39.5 mg, 1.04 mmol) was added in a single portion. The resulting mixture was heated to reflux and stirred for 2 h, before cooling to 0 °C. Once cooled, the reaction was quenched by the sequential addition of water (40 µL), 15% aq. NaOH solution (40 µL) and more water (120 µL). This mixture was warmed to RT and stirred for 15 min, before the addition of $MgSO_4$. This mixture was stirred for a further 15 min, then filtered and concentrated *in vacuo*. Purification by column chromatography (2:1 hexane:ethyl acetate) afforded the title compound **S142** as a yellow oil (166 mg, 69%); R_f = 0.80 (1:1 hexane:ethyl acetate); IR (thin film) ν_{max} / cm^{-1} 3404br 1597s, 1500s,1316w, 743s; δ_H (400 MHz, $CDCl_3$) 7.32−7.16 (6H, m, Ar**H**), 6.82−6.56 (9H, m, Ar**H**), 6.73−6.64 (2H, m, Ar**H**), 3.54 (2H, t, *J* = 6.4, NCH_2), 3.45 (2H, t, *J* = 7.3, NCH_2), 3.35 (2H, t, *J* = 6.4, NCH_2), 3.16 (2H, t, *J* = 6.8, NCH_2), 1.94–1.87 (2H, m, $CH_2C\mathbf{H_2}CH_2$); δ_C (101 MHz, $CDCl_3$) 148.3 (Ar**C**), 148.2 (Ar**C**), 148.0 (Ar**C**), 129.6 (Ar**C**H), 129.5 (Ar**C**H), 129.4 (Ar**C**H), 117.8 (Ar**C**H), 117.6 (Ar**C**H), 117.2 (Ar**C**H), 113.3 (Ar**C**H), 113.03 (Ar**C**H) 112.95 (Ar**C**H), 50.7 (N**C**H_2), 49.3 (N**C**H_2), 41.9 (N**C**H_2), 41.5 (N**C**H_2), 27.2 (**C**H_2); HRMS (ESI^+): m/z calc. for $C_{23}H_{28}N_3$: 346.2280, found: 346.2278 $[MH]^+$ (–0.1 ppm error).

1,3,6-Triphenyl-1,3,6-triazonan-2-one (101)

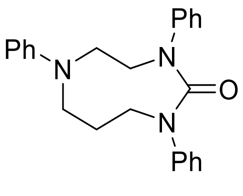

To a solution of N1,N3-diphenyl-N1-(2-(phenylamino)ethyl)propane-1,3-diamine **S142** (38 mg, 0.110 mmol) in DCM (1.1 mL) at RT, triethylamine (23.0 µL, 0.165 mmol) and triphosgene (13.1 mg, 0.0440 mmol) were added sequentially. The resulting mixture was stirred at RT for

2 h, quenched by the addition of water (10 mL), extracted with DCM (2 x 10 mL) and concentrated under vacuum. Purification by column chromatography (5:1 → 1:1 hexane:diethyl ether) afforded the title compound **101** as a white solid (28 mg, 68%); R_f = 0.25 (1:1 hexane:diethyl ether); M.p. 87–89 °C; IR (thin film) ν_{max} / cm^{-1} 1686s, 1597m, 1504s, 1312w, 749s, 652m; δ_H (400 MHz, d-6 DMSO) 7.39–6.95 (12H, br m, Ar**H**), 6.71–6.61 (3H, br m, Ar**H**), 3.87–3.82 (2H, br m, NCH_2), 3.62–3.58 (2H, br m, NCH_2), 3.56–3.51 (2H, br m, NCH_2), 3.49–3.44 (2H, br m, NCH_2), 1.82–1.75 (2H, br m, $CH_2C\mathbf{H_2}CH_2$); δ_C (100 MHz, DMSO d-6) 160.1 (**C**O), 148.4 (Ar**C**), 145.5 (Ar**C**), 140.7 (Ar**C**), 129.9 (Ar**C**H), 129.5 (Ar**C**H), 129.4 (Ar**C**H), 124.7 (Ar**C**H), 122.7 (Ar**C**H), 119.0 (Ar**C**H), 118.3 (Ar**C**H), 116.8 (Ar**C**H) 112.8 (Ar**C**H), 55.3 (N**C**H_2), 53.2 (N**C**H_2), 49.6 (N**C**H_2), 48.6 (N**C**H_2), 25.7 (**C**H_2); HRMS (ESI$^+$): m/z calc. for $C_{24}H_{25}N_3NaO$: 394.1888, found: 394.1890 [MNa]$^+$ (1.0 ppm error).

1,3,6-Trimethyl-1,3,6-triazocan-2-one (102)

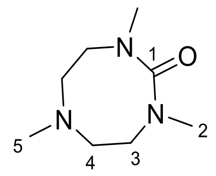

N^1,N^2-dimethyl-N^1-(2-(methylamino)ethyl)ethane-1,2-diamine (900 µL, 5.99 mmol) was added reaction flask (500 mL RBF) containing anhydrous DMF (120 mL). The resulting reaction vessel was evacuated under *vacuo* and backfilled with CO_2 (via balloon) 6 times. Next, EDC.HCl (1.72 g, 8.98 mmol), HOBt (972 mg, 7.19 mmol) and dry DIPEA (3.65 mL, 21.0 mmol) were each added in single portion at RT, with a colour change to pale yellow solution noted. The resulting solution was stirred at RT overnight under a slight positive pressure of CO_2 (balloon) and the progress of the reaction was monitored via TLC. After total of 18 h, the reaction was deemed to have gone to completion by TLC analysis and colour change to pale orange solution was observed. The resulting mixture was diluted with CH_2Cl_2 (100 mL) and poured into separating funnel. The organic layer was washed sequentially with sat. $NaHCO_{3(aq)}$ (2 × 120 mL) and sat. brine (10 × 120 mL), before was dried over $MgSO_4$, filtered and concentrated under reduced pressure to yield a pale yellow liquid (5.70 g). The crude product was purified by flash column chromatography (SiO_2, 50 mm column, eluent: MeOH:EtOAc, 90:10 to 100:0) to afford the title compound **102** as a colourless oil (720 mg, 70%); R_f = 0.15 (MeOH, 100); IR (neat) ν_{max} / cm $^{-1}$: 3486brw, 2936m (C–H alkyl), 2878m (C–H alkyl), 2850m (C–H alkyl),

2800m(C–H alkyl), 2219w, 1632 (C=O urea), 1494s, 1453s, 1428m, 1412m, 1399s, 1384s, 1329m, 1351w, 1304w, 1267w, 1221s, 1166m, 1145w, 1127m, 1107w, 1084w, 1038m, 1004w, 978w, 926w, 890w, 858w, 789w, 761w, 705w, 643w, 618w, 594m, 580w, 537w, 503w; δ_H (400 MHz; $CDCl_3$) 3.29 – 3.21 (4H, m, C(3)H_2), 2.70 (6H, s, C(2)H_3), 2.66 – 2.55 (4H, m, C(4)H_2), 2.33 (3H, s, C(5)H_3); δ_C (101 MHz; $CDCl_3$) 164.0 (CO, C1), 54.9 (2 × CH_2, C4), 54.1 (2 × CH_2, C3), 47.0 (2 × CH_3, C2), 37.1 (CH_3, C5); HRMS (ESI): calcd. for $C_8H_{18}N_3O$, 172.1444. Found: $[MH]^+$, 172.1446 (–0.9 ppm error), calcd. for $C_8H_{17}N_3NaO$, 194.1264. Found: $[MNa]^+$, 194.1264 (0.1 ppm error).

4,7,10-Trimethyl-1,2,4,7,10-thiatetrazecan-3-one 1,1-dioxide (103)

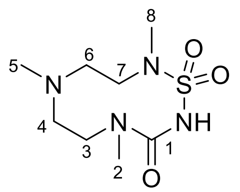

Chlorosulfonyl isocyanate (380 µL, 4.31 mmol) was added in a single portion via syringe to a clear solution of N[1],N[2]-dimethyl-N[1]-(2-(methylamino)ethyl)ethane-1,2-diamine (600 µL, 3.59 mmol) and dry Et_3N (1.50 mL, 11.0 mmol) in anhydrous CH_2Cl_2 (72.0 mL) at 0 °C under Ar. The resulting mixture was allowed to warm gradually to RT overnight under Ar, with a colour change to a pale yellow solution observed. After a total of 18 h, the reaction was deemed to have gone to completion by TLC analysis. The resulting mixture was quenched with sat. $NaHCO_{3(aq)}$ (80 mL) and poured into a separating funnel. The aqueous layer was extracted EtOAc (3 × 100 mL) and combined organic layers were washed sequentially with $NH_4Cl_{(aq)}$ (3 × 80 mL) and sat. brine (3 × 80 mL), before being dried over $MgSO_4$, filtered and concentrated under reduced pressure to yield an orange oil (871 mg). The crude product was purified by flash column chromatography (SiO_2, 40 mm column, eluent: EtOAc:MeOH, 90:10 to 100:0) to afford the title compound **103** as a colourless oil (642 mg, 71%); R_f = 0.29 (MeOH); IR (neat) ν_{max} / cm^{-1}: 3439w (N–H sulfonamide), 2940w (C–H alkyl), 2796w, 2246w, 1688w, 1574s (C=O urea), 1455m, 1392w, 1236m, 1149w, 1112m, 1067w, 1037w, 998w, 966w, 905s, 831w, 724s, 689m, 645m, 607m, 529w, 480w; δ_H (400 MHz, $CDCl_3$) 4.88 (2H, s, br, C(3)H_2), 3.48 (2H, s, br, C(7)H_2), 3.21 (2H, s, br, C(4)H_2), 2.87 (3H, s, C(8)H_3), 2.83 (3H, s, C(2)H_3), 2.71 (2H, s, br, C(6)H_2), 2.43 (3H, s, C(5)H_3); δ_C (101 MHz, $CDCl_3$, at 50 °C) 160.1 (CO, C1), 57.0 (CH_2, C6), 54.4 (CH_2,

C3), 49.0 (CH_2, C4), 48.2 (CH_2, C7), 43.8 (CH_3, C5), 39.2 (CH_3, C8), 34.6 (CH_3, C8); HRMS (ESI): calcd. for $C_8H_{19}N_4O_3S$, 251.1172. Found: $[MH]^+$, 251.1166 (2.5 ppm error), calcd. for $C_8H_{18}N_4NaO_3S$, 251.1172. Found: $[MNa]^+$, 251.1166 (1.8 ppm error).

Control reaction for 103: unsuccessful synthesis of 4,8-dimethyl-1,5,4,6,8-oxathiatriazecan-7-one 5,5-dioxide

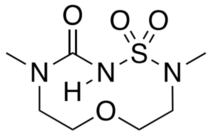

Chlorosulfonyl isocyanate (431 µL, 4.95 mmol) was added in a single portion via syringe to a clear solution of 1,5-bis(methylamino)-3-oxapentane (500 µL, 3.29 mmol) and dry Et_3N (2.30 mL, 16.5 mmol) in anhydrous CH_2Cl_2 (66 mL) at 0 °C under Ar. The resulting mixture was allowed to warm gradually to RT overnight under Ar and the progress of the reaction was monitored *via* TLC, with a colour change to a pale-yellow solution observed. After stirring for 18 h, the reaction was deemed to have gone to completion based on the full consumption of 1,5-bis(methylamino)-3-oxapentane when alaysed by TLC and mass spectrometry analysis. The resulting mixture was quenched with sat. $NaHCO_{3(aq)}$ (80 mL) and poured into a separating funnel. The aqueous layer was extracted EtOAc (3 × 100 mL) and combined organic layers were washed sequentially with $NH_4Cl_{(aq)}$ (3 × 80 mL) and sat. brine (3 × 80 mL), before being dried over $MgSO_4$, filtered and concentrated under reduced pressure. When analysed by TLC, and by ^1H NMR spectroscopy a complex mixture of multiple spots was apparent, with no evidence that 8-dimethyl-1,5,4,6,8-oxathiatriazecan-7-one 5,5-dioxide has formed.

Methyl 2-((benzyl (2-hydroxybenzyl amino) methyl) benzoate (104)

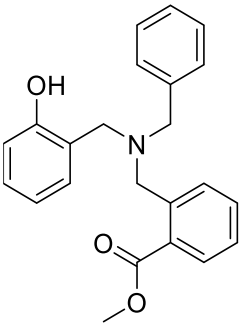

To a stirring solution of potassium carbonate (0.415 g, 3.00 mmol) in acetonitrile (5.00 mL), 2-((benzylamino) methyl) phenol (0.2133 g, 1.00 mmol) was added followed by methyl 2-(bromomethyl) benzoate (0.230 g, 1.00 mmol). The reaction mixture was refluxed at 90 °C for

3 hours under argon before filtering through Celite washing with DCM, concentrating under vacuum and purifying by flash column chromatography (8:2 hexane:diethyl ether) to afford the title compound **104** as a colourless oil (0.2552 g, 71%) R_f = 0.27 (8:2 hexane:diethyl ether) ν_{max}/cm^{-1} (thin film) 3029, 2956, 2838, 2255, 1719, 1588, 1488, 1434, 1453, 1254, 1079, 909, 729; δ_H (400 MHz, $CDCl_3$) 10.68 (1H, bs, O**H**), 7.90 (1H, d, *J* = 7.4 Hz, *J* = 1.2 Hz, Ar**H**), 7.52−7.46 (2H, m, Ar**H**), 7.39−7.28 (6H, m, Ar**H**), 7.17 (1H, td, *J* = 8.0 Hz, 1.5 Hz, Ar**H**), 7.04 (1H, dd, *J* = 7.4 Hz, 1.2 Hz), 6.86 (1H, dd, *J* = 8.0 Hz, 1.5 Hz, Ar**H**), 6.81 (1H, td, *J* = 7.4 Hz, 1.2 Hz, Ar**H**), 4.05 (2H, s, ArC**H**$_2$N), 3.90 (3H, s, C**H**$_3$O), 3.77 (2H, s, ArC**H**$_2$N), 3.71 (2H, s, ArC**H**$_2$N); δ_c (101 MHz, $CDCl_3$) 168.0 (**C**O), 157.6 (Ar**C**), 138.8 (Ar**C**), 136.6 (Ar**C**), 132.3 (Ar**C**H), 131.5 (Ar**C**H), 131.0 (Ar**C**H), 130.9 (Ar**C**H), 130.1 (Ar**C**H), 129.2 (Ar**C**H), 128.9 (Ar**C**H), 128.7 (Ar**C**H), 127.8 (Ar**C**H), 127.7 (Ar**C**H), 122.2 (Ar**C**), 119.4 (Ar**C**H), 116.2 (Ar**C**H), 58.4 (ArC**H**$_2$N), 56.8 (ArC**H**$_2$N), 55.4 (ArC**H**$_2$N), 52.3 (OC**H**$_3$); HRMS (ESI) calcd. for $C_{23}H_{24}NO_3$ 362.1756. Found $[MH]^+$ 362.1748 (−2.21 ppm error).

2-Benzylisoindolin-1-one (106)

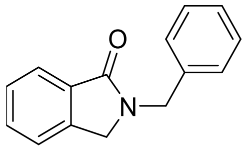

To a stirring solution of ester **104** (100 mg, 0.277 mmol) in methanol (10 mL) and water (5 mL), aqueous lithium hydroxide (0.5 M) (1.27 mL, 0.637mmol) was added and heated for at 50 °C for 3 hours. The solvent was removed under vacuum using dichloromethane (5 × 50.0 mL) to help remove the water via an azeotropic mixture. The intermediate lithium 2-((benzyl(2-oxidobenzyl) amino) methyl) benzoate was then dissolved in chloroform (2.80 mL) and DIPEA (0.090 mL, 0.513 mmol) was added followed by T3P 50% w/v in ethyl acetate (0.220 g, 0.693 mmol) and stirred at room temperature for 24 hours under argon. The reaction mixture was washed in dichloromethane (2 × 100 mL) and brine (50.0 mL) and the combined organic phases were dried with sodium sulphate, filtered, concentrated under vacuum and purified via flash column chromatography (6:4 hexane:ethyl acetate) to afford the title compound **106** (49.0 mg, 79%) R_f = 0.25 (6:4 hexane:ethyl acetate); ν_{max}/cm^{-1} (thin film) 3481, 3031, 2915, 1674, 1452, 733, 701; δ_H (400 MHz, $CDCl_3$) 7.88 (1H, d, *J* = 6.8 Hz, Ar**H**), 7.52−7.48 (1H, td, *J* = 7.3 Hz, *J* = 1.3 Hz, Ar**H**), 7.46−7.42 (1H, m, Ar**H**), 7.36 (1H, d, *J* = 7.3 Hz, Ar**H**), 7.34−7.31 (1H, m, Ar**H**), 7.30−7.29 (2H, m, Ar**H**) 7.29−7.24 (1H, m, Ar**H**), 4.79 (2H, s, NC**H**$_2$Ar),

4.24 (2H, s, ArC**H**$_2$N); δ_C (101 MHz, $CDCl_3$) 168.6 (**C**O), 141.3 (Ar**C**), 137.1 (Ar**C**), 132.7 (Ar**C**), 131.5 (Ar**C**H), 128.9 (Ar**C**H), 128.3 (Ar**C**H), 128.1 (Ar**C**H), 127.8 (Ar**C**H), 124.0 (Ar**C**H), 122.9 (Ar**C**H), 49.5, (N**C**H$_2$Ar), 46.5 (Ar**C**H$_2$N); HRMS (ESI) calcd. for $C_{15}H_{14}NO$ 224.1075. Found $[MH]^+$ 224.1074 (−0.446 ppm error). The NMR data are consistent with published literature data.[16]

tert-Butyl (3-(phenyl(2-(phenylamino)ethyl)amino)propyl)carbamate (107a)

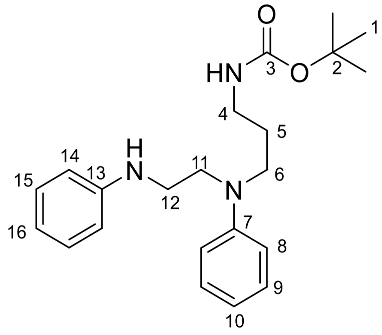

K_2CO_3 (2.44 g, 17.3 mmol) was added to a pale-yellow solution of N^1,N^2-diphenylethane-1,2-diamine (1.87 g, 8.80 mmol) and _tert_-butyl (3-bromopropyl)carbamate (500 µL, 5.53 mmol) in anhydrous MeCN (44.0 mL) at RT under Ar. The resulting dark brown suspension was then heated to 95 °C under Ar. After 18 h, the reaction was deemed to have gone to completion by TLC. The reaction mixture allowed to cool to RT and diluted with EtOAc (30 mL) and poured into a separating funnel. The diluted solution was washed with sat. $NaHCO_{3(aq)}$ (1 × 30 mL) and resulting aqueous phase was extracted with EtOAc (3 × 30 mL), before the combined organic phases were collected dried over $MgSO_4$, filtered and concentrated under reduced pressure to yield a pale-yellow solid (3.72 g). The crude product was purified by flash column chromatography (SiO_2, 70 mm column, eluent: EtOAc:_n_-hexane, 20:80) to afford product **107a** as a pale yellow oil (317 mg, 19%); R_f = 0.23 (EtOAc:_n_–hexane, 20:80); IR (neat) ν_{max} / cm^{-1}: 3405brw (N–H analine/carbamate), 2974w (C–H alkyl), 1693s (C=O carbamate), 1598s (CC aromatic), 1503s (CC aromatic), 1391w, 1365m, 1320w, 1249m, 1210w, 1164s, 1072w, 1036w, 992w, 910w, 868m, 745s, 692s, 647w, 507m; δ_H (400 MHz; $CDCl_3$) 7.29 – 7.14 (4H, m, C(8+9)H), 6.79 – 6.70 (5H, m, C(10+15+16)H), 6.67 – 6.58 (2H, m, C(14)H), 4.59 (1H, br, s, NH carbamate), 3.85 (1H, br, s, NH aniline), 3.52 (2H, t, J = 6.4 Hz, C(11)H_2), 3.39 – 3.30 (4H, m, C(6+12)H_2), 3.21 – 3.11 (2H, m, C(4)H_2), 1.82 – 1.70 (2H, m, C(5)H_2), 1.45 (9H, s, C(1)H_3); δ_C (101 MHz; $CDCl_3$) 156.1 (CO, C3), 148.1 (C, C7), 148.0 (C, C13), 129.5 (2 × CH, C9), 129.4 (2 × CH, C8), 117.7 (CH, C10), 117.1 (CH, C16), 113.2 (2 × CH, C15), 113.0 (2 × CH, C14), 79.4 (C,

C2), 50.60 (CH_2, C11), 49.0 (CH_2, C6), 41.4 (CH_2, C12), 38.6 (CH_2, C4), 28.5 (2 × CH_3, C1), 27.8 (CH_2, C5); HRMS (ESI): calcd. for $C_{22}H_{32}N_3O_2$, 370.2489. Found: $[MH]^+$, 370.2499 (−2.7 ppm error), calcd. for $C_{22}H_{31}N_3NaO_2$, 392.2308. Found: $[MNa]^+$, 392.2319 (−2.7 ppm error), calcd. for $C_{22}H_{31}KN_3O_2$, 408.2048. Found: $[MK]^+$, 408.2060 (−3.1 ppm error).

1,3-Diphenylimidazolidine-2-thione (109a)

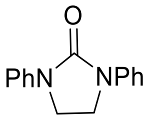

Hydrogen chloride solution (4.0 M in 1,4-dioxane, 12.0 mL) was added dropwise over a period of 30 sec via syringe to a solution of tert-butyl (3-(phenyl(2-(phenylamino)ethyl)amino)propyl)carbamate **107a** (110 mg, 0.296 mmol) in anhydrous Et_2O (6.0 mL) at RT under Ar. A white solid was immediately noted along with a colour change to creamy white suspension and the reaction mixture was stirred at RT for 4 h under Ar, at which point the reaction mixture was deemed to have gone to completion by TLC (formation of tert-butyl (3-(phenyl(2-(phenylamino)ethyl)amino)propyl)carbamate TFA salt). The resulting mixture was concentrated under *vacuo* to yield a brown solid. Next, the crude mixture was redissolved in anhydrous CH_2Cl_2 to obtain dark orange emulsion. Then Et_3N (410 µL, 2.96 mmol) was added dropwise over a period of 1 min via syringe, whereupon a liberation grey fume and colour changed to pale yellow solution was observed. Triphosgene (87.8 mg, 0.296 mmol) was added in a single portion to a corresponding mixture, with a colour change to a pale-yellow solution was immediately noted and the reaction mixture was stirred at RT for 18 h of stirring at RT under Ar, before reaction was deemed to have to completion by TLC. The resulting mixture was concentrated under reduced pressure to yield a white solid (154 mg). The crude product was purified by flash column chromatography (SiO_2, 30 mm column, eluent: EtOAc:*n*–hexane, 30:70 to 50:50) to afford urea **109a** as a white solid (51.2 mg, 73%, over two steps). δ_H (400 MHz; $CDCl_3$) 7.64 – 7.54 (m, 4H), 7.43 – 7.32 (m, 4H), 7.10 (tt, *J* = 7.5, 1.1 Hz, 2H), 3.99 (s, 4H); δ_C (101 MHz; $CDCl_3$) 140.2 (CO), 129.0 (4 × CH), 123.2 (2 × CH), 122.7 (2 ×C), 118.2 (4 × CH), 42.1 (2 × CH_2). The spectroscopic data are consistent with those previously reported.[17]

4-(Phenyl(2-(phenylamino)ethyl)amino)butan-1-ol (107b)

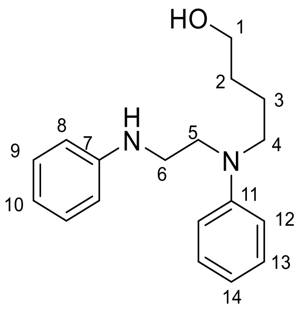

K_2CO_3 (911 mg, 6.60 mmol) was added to a pale yellow solution of N1,N2-diphenylethane-1,2-diamine (559 mg, 2.63 mmol) and 4-bromobutan-1-ol (336 mg, 2.20 mmol) in anhydrous MeCN (44.0 mL) at RT under Ar. The resulting milky white suspension was then heated to 95 °C under Ar for 18 h, after which time the reaction was deemed to have gone to completion by TLC. The reaction mixture was allowed to cool to RT, before being filtered through Celite and washed with EtOAc (3 × 30 mL). The resulting filtrate was concentrated under reduced pressure to yield a beige oil (1.09 g). The crude product was purified by flash column chromatography (SiO_2, 50 mm column, eluent: EtOAc:*n*-hexane, 50:50) to afford the title compound **107b** as a colourless oil (37.0 mg, 6%). R_f = 0.41 (50:50 EtOAc:*n*–hexane); IR (neat) ν_{max} / cm^{-1}: / 3550w (N–H aniline), 3378brw (O–H alcohol), 3023w, 2940w, 2877w; 1934w, 1727w, 1597s (CC aromatic), 1502s (CC aromatic), 1431w, 1369w, 1321w, 1255w, 1226w, 1195w, 1178w, 1122w, 1042m, 991w, 925w, 867w, 756s, 692s, 508m; δ_H (400 MHz; $CDCl_3$) 7.28 – 7.14 (4H, m, C(9+13)H), 6.81 – 6.68 (4H, m, C(10+12+14)H), 6.66 – 6.59 (2H, m, C(8)H), 3.65 (2H, t, *J* = 6.2 Hz, C(1)H_2), 3.54 (2H, t, *J* = 6.5 Hz, C(5)H_2), 3.39 – 3.30 (4H, m, C(4+6)H_2), 1.72 – 1.52 (4H, m, C(2+3)H_2); δ_C (101 MHz; $CDCl_3$) 148.3 (C, C11), 148.1 (C, C7), 129.5 (2 × CH, C13), 129.5 (2 × CH, C9), 117.8 (CH, C14), 117.1 (CH, C10), 113.3 (2 × CH, C12), 113.1 (2 × CH, C8), 62.8 (CH_2, C1), 51.5 (CH_2, C4), 50.6 (CH_2, C5), 41.5 (CH_2, C6), 30.3 (CH_2, C2), 23.7 (CH_2, C3); HRMS (ESI): calcd. for $C_{18}H_{25}N_2O$, 285.1961. Found: $[MH]^+$, 285.1964 (−1.0 ppm error).

1,3-Diphenylimidazolidine-2-thione (109b)

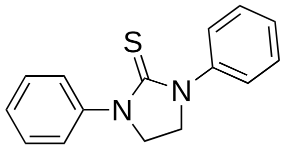

Thiophosgene (13.0 µL, 0.176 mmol) was added via syringe over a period of 30 sec to a pale brown solution of 4-(phenyl(2-(phenylamino)ethyl)amino)butan-1-ol **107b** (41.6 mg, 0.146 mmol), anhydrous Et_3N (71.3 µL, 0.511 mmol) in anhydrous CH_2Cl_2 (3.0 mL) under Ar at RT. An immediate colour change to dark brown solution was noted along with liberation of grey fumes ($HCl_{(g)}$). The resulting solution was stirred at RT overnight under Ar and progress of the reaction was monitored by TLC. After total of 18 h, the reaction was judged to be complete, based on TLC analysis. The resulting mixture was concentrated under reduced pressure and purified by flash column chromatography (SiO_2, 20 mm column, eluent: EtOAc, 100%) to afford thiourea **109b** as a white solid (17.1 mg, 46%). R_f = 0.41 (50:50 EtOAc:*n*–hexane); δ_H (400 MHz; $CDCl_3$) 7.64 – 7.56 (m, 4H), 7.43 – 7.33 (m, 4H), 7.10 (td, *J* = 7.6, 7.0, 1.2 Hz, 2H), 3.99 (s, 4H); The spectroscopic data are consistent with those previously reported.[18]

2-(6-Bromopyridin-2-yl)-*N*-methyl-1-phenylethan-1-amine (S143)

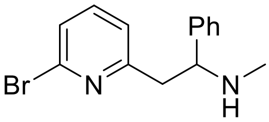

N,N-Diisopropylamine (4.35 mL, 35.1 mmol) was dissolved in THF (175 mL) and cooled to 0 ^oC before n-BuLi (2.5 M solution in hexanes, 14.0 mL, 35.1 mmol) was added dropwise and stirred for 30 mins. The LDA solution was then cooled to −78 °C, where 2-bromo-6-methylpyridine **110** (1.98 mL, 17.5 mmol) was added dropwise and stirred for 1 h. *N*-Benzylidenemethylamine **111** (4.35 mL, 35.1 mmol) was added and stirred for a further 2 h at −78 °C before slowly warming to RT. The solution was then quenched with sat. aq. NH_4Cl (150 mL) and extracted with ethyl acetate (3 × 100 mL) and washed with brine (150 mL). The combined organic extracts were dried over $MgSO_4$, filtered and removed in vacuo. Purification by flash column chromatography (SiO_2, 5% methanol in ethyl acetate) afforded the title compound **S143** as a yellow oil (3.82 g, 81%); R_f. 0.23 (5% methanol in ethyl acetate); δ_H (400 MHz, $CDCl_3$) 7.35–7.27 (7H, m, ArH), 6.86 (1H, d, *J* = 7.0 Hz, Ar**H**), 3.98 (1H, dd, *J* = 8.4, 6.1 Hz, CH_2C**H**NMe), 3.12 (1H, dd, *J* = 13.7, 6.1 Hz, CC**H**H'CHPh), 3.01 (1H, dd, *J* = 13.7, 8.4 Hz,

CCHH'CHPh), 2.25 (3H, s, NHC**H₃**), 1.68–1.65 (1H, br s, N**H**). Spectroscopic data matched those reported in the literature.[1]

3-{[2-(6-Bromopyridin-2-yl)-1-phenylethyl](methyl)amino}propan-1-ol (S144)

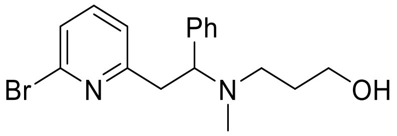

2-(6-Bromopyridin-2-yl)-*N*-methyl-1-phenylethan-1-amine **S143** (1.00 g, 3.45 mmol), 3-iodo-1-propanol **112** (660 µL, 6.89 mmol) and potassium carbonate (953 mg, 6.90 mmol) were dissolved in acetonitrile (7 mL). After stirring at 85 °C for 18 h the reaction mixture was diluted with water (5 mL) and extracted with ethyl acetate (3 × 5 mL). The combined organic layers were dried with anhydrous $MgSO_4$, filtered, and then purified by flash column chromatography (SiO_2, 5% methanol in ethyl acetate) affording the *title compound* **S144** as a colourless oil (900 mg, 75%); R_f. 0.23 (5% methanol in ethyl acetate); ν_{max}/cm^{-1} (neat) 3369, 3029, 2943, 2850, 2237, 1583, 1553, 1435, 1406, 1175, 1156, 1116, 1068, 1033; δ_H (400 MHz, $CDCl_3$) 7.37–7.24 (7H, m, Ar**H**), 6.92 (1H, d, *J* = 7.0 Hz, Ar**H**), 4.19 (1H, t, *J* = 7.0 Hz, PhC**H**N), 3.71–3.68 (2H, m, C**H₂**OH), 3.55–3.50 (1H, dd, *J* = 13.7, 7.4 Hz, C**H**H'CHPh), 3.20–3.14 (1H, dd, *J* = 13.7 8.0 Hz, CH**H**'CHPh), 2.73–2.67 (1H, ddd, *J* = 12.1, 7.9, 4.3 Hz, NC**H**H'CH_2), 2.62–2.56 (1H, ddd, *J* = 12.1, 7.0, 4.2 Hz, NCH**H**'CH_2), 2.31 (3H, s, C**H₃**N), 1.74–1.63 (2H, m, C**H₂**); δ_C (101 MHz, $CDCl_3$) 160.8 (Ar**C**), 141.4 (Ar**C**), 138.5 (Ar**C**), 137.9 (Ar**C**H), 128.8 (Ar**C**H), 128.1 (Ar**C**H), 127.5 (Ar**C**H), 125.6 (Ar**C**H), 122.7 (Ar**C**H), 69.1 (CH_2**C**HN), 64.0 (**C**H_2OH), 54.6 (C**C**H_2CH), 40.4 (N**C**H_2), 37.5 (**C**H_3N), 27.8 (**C**H_2); HRMS (ESI): calcd. for $C_{17}H_{22}{}^{79}BrN_2O$, 349.0910. Found: $[MH]^+$, 349.0906 (1.1 ppm error)].

Methyl 2-[2-(6-{2-[(3-hydroxypropyl)(methyl)amino]-2-phenylethyl}pyridin-2-yl)phenyl]acetate (S144)

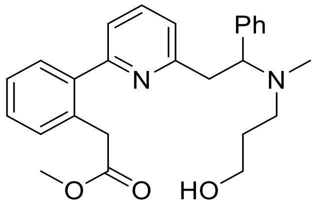

3-{[2-(6-Bromopyridin-2-yl)-1-phenylethyl](methyl)amino}propan-1-ol **S144** (683 mg, 1.96 mmol), methyl 2-(2-(4,4,5,5-tetramethyl-1,3,2-dioxaborolan-2-yl)phenyl) acetate **113** (813 mg, 2.94 mmol), potassium phosphate (833 mg, 3.92 mmol) and $PdCl_2$(dppf)·CH_2Cl_2 (80.0 mg, 89.0 µmol) were charged into an round bottom flask purged with nitrogen. THF (20 mL) and de-ionised water (178 µL, 9.81 mmol) were added and heated to 80 °C, at reflux, for 18 h. Upon completion the solution was cooled to room temperature, diluted with water (15 mL), extracted with ethyl acetate (3 × 20 mL) and washed with sat. brine (10 mL). The combined organic extracts were dried over $MgSO_4$, filtered, and removed *in vacuo*. Purification by flash column chromatography (SiO_2, 10% methanol in ethyl acetate) afforded the *title compound* as a brown oil (713 mg, 87%); R_f. 0.38 (10% methanol in ethyl acetate); ν_{max}/cm^{-1} (neat) 3367, 3061, 3025, 2950, 2852, 1736, 1587, 1570, 1496, 1448, 1339, 1254, 1211, 1159, 1071; δ_H (400 MHz, $CDCl_3$) 7.54 (1H, t, *J* = 7.8 Hz, Ar**H**), 7.41–7.21 (10H, m, Ar**H**), 6.91 (1H, d, *J* = 7.6 Hz, Ar**H**), 4.22 (1H, t, *J* = 8.0 Hz, NC**H**Ph), 3.85–3.74 (2H, m, C**H₂**CO₂), 3.64 (2H, t, *J* = 5.1 Hz, C**H₂**OH), 3.57–3.51 (4H, m, CO₂C**H₃** and C**H**H'CHN), 3.25–3.19 (1H, dd, *J* = 13.6, 8.0 Hz, CH**H**'CHN), 2.70–2.57 (2H, m, NC**H₂**), 2.30 (1H, s, NC**H₃**), 1.70–1.60 (2H, m, C**H₂**); δ_C (101 MHz, $CDCl_3$) 172.5 (**C**O₂), 159.0 (Ar**C**), 158.6 (Ar**C**), 140.7 (Ar**C**), 138.2 (Ar**C**), 136.7 (Ar**C**H), 132.4 (Ar**C**), 131.4 (Ar**C**H), 130.1 (Ar**C**H), 129.1 (Ar**C**H), 128.5 (Ar**C**H), 128.2 (Ar**C**H), 127.5 (Ar**C**H), 126.8 (Ar**C**H) 122.1 (Ar**C**H), 121.7 (Ar**C**H), 69.4 (N**C**HPh), 64.3 (**C**H₂OH), 55.1 (N**C**H₂), 52.0 (CO₂**C**H₃), 40.7 (**C**H₂CHN), 39.2 (C**H₂**CO₂), 37.4 (N**C**H₃), 27.8 (**C**H₂); HRMS (ESI): calcd. for $C_{26}H_{31}N_2O_3$, 419.2329. Found: $[MH]^+$, 419.2326 (0.7 ppm error)].

[2-(6-{2-[(3-Hydroxypropyl)(methyl)amino]-2-phenylethyl}pyridin-2-yl)phenyl]acetic acid (114)

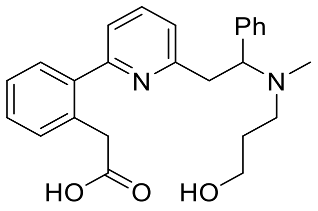

Methyl 2-[2-(6-{2-[(3-hydroxypropyl)(methyl)amino]-2-phenylethyl}pyridin-2-yl)phenyl]acetate **S144** (713 mg, 1.70 mmol) was dissolved in aqueous lithium hydroxide solution (0.5 M, 17 mL, 8.52 mmol) and THF (17 mL). The resulting bi-phasic solution was vigorously stirred for 18 h at RT. Upon completion, the solvent was removed *in vacuo*. Purification by flash column chromatography (SiO_2, 50% methanol in ethyl acetate) afforded the *title compound* as a brown powder (687 mg, 96%); m.p. 73–76 °C; R_f 0.20 (50% methanol in ethyl acetate); ν_{max}/cm^{-1} (neat) 3323, 3061, 3028, 2943, 2244, 1717, 1571, 1493, 1453, 1375, 1158, 1065; δ_H (400 MHz, $CDCl_3$) 7.36 (1H, s, Ar**H**), 7.47–7.45 (1H, d, *J* = 7.6 Hz, Ar**H**), 7.38–7.17 (9H, m, Ar**H**), 6.96 (1H, s, Ar**H**), 4.07–4.03 (1H, t, *J* = 7.0 Hz, NC**H**Ph), 3.72–3.59 (2H, br, C**H₂**CO₂), 3.55–3.48 (3H, m, C**H₂**OH and C**H**H′CHN), 3.20 (1H, br, CH**H**′CHN), 2.58 (2H, t, *J* = 7.0 Hz, NC**H₂**), 2.28 (3H, s, NC**H₃**), 1.75–1.53 (2H, m, C**H₂**); δ_C (101 MHz, $CDCl_3$) 175.4 (**C**O₂), 157.9 (Ar**C**), 157.6 (Ar**C**), 138.4 (Ar**C**), 138.2 (Ar**C**), 136.4 (Ar**C**H), 134.1 (Ar**C**), 131.3 (Ar**C**H), 130.0 (Ar**C**H), 129.1 (Ar**C**H), 129.0 (Ar**C**H), 128.3 (Ar**C**H), 127.9 (Ar**C**H), 127.1 (Ar**C**H), 123.0 (Ar**C**H), 122.6 (Ar**C**H), 69.4 (N**C**HPh), 62.7 (**C**H₂OH), 53.9 (N**C**H₂), 42.1 (**C**H₂CHN), 39.7 (C**H₂**CO₂), 37.3 (N**C**H₃), 27.6 (**C**H₂); HRMS (ESI): calcd. for $C_{25}H_{29}N_2O_3$, 405.2173. Found: $[MH]^+$, 405.2176 (−0.8 ppm error)].

14-Methyl-15-phenyl-10-oxa-14,21-diazatricyclo[15.3.1.0²,⁷]henicosa-1(21),2,4,6,17,19-hexaen-9-one (117)

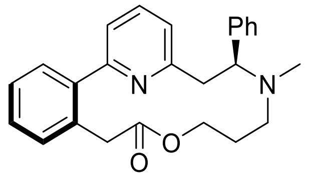

To a stirring solution of [2-(6-{2-[(3-hydroxypropyl)(methyl)amino]-2-phenylethyl}pyridin-2-yl)phenyl]acetic acid **114** (202 mg, 0.52 mmol) in acetonitrile (5 mL), was added diisopropylethylamine (430 µL, 2.47 mmol), followed by the addition of EDC·HCl (144 mg, 0.75

mmol) and HOBt (101 mg, 0.75 mmol). After stirring for 18 h at RT, the reaction mixture was directly concentrated *in vacuo*. Purification by flash column chromatography (SiO_2, 50% ethyl acetate in hexanes) afforded the *title compound* as a red oil (142 mg, 73%); R_f 0.59 (ethyl acetate); ν_{max}/cm^{-1} (neat) 3069, 3025, 2941, 2851, 1735, 1590, 1569, 1450, 1360, 1341, 1289, 1213, 1173, 1085, 1008; δ_H (400 MHz, $CDCl_3$) 7.71 (1H, t, *J* = 7.7 Hz, Ar**H**), 7.51 (1H, dd, *J* = 6.9, 2.1 Hz, Ar**H**), 7.42–7.26 (9H, m, Ar**H**), 7.18 (1H, d, *J* = 7.7 Hz, Ar**H**), 4.48–4.40 (2H, m, NC**H**Ph and ArC**H**H'CO_2), 4.00 (1H, d, *J* = 17.5 Hz, ArCH**H'**CO_2), 3.80–3.75 (1H, m, OC**H**H'CH_2), 3.69–3.62 (2H, m, OCH**H'**CH_2 and ArC**H**H'CH), 3.10–3.05 (1H, dd, *J* = 15.2, 4.5 Hz, ArCH**H'**CH), 2.80–2.73 (1H, m, NC**H**H'CH_2), 2.28–2.20 (4H, m, NCH**H'**CH_2 and NC**H₃**), 1.66–1.60 (2H, m, CH_2C**H₂**CH_2); δ_C (101 MHz, $CDCl_3$) 172.0 (**C**O_2), 160.0 (Ar**C**), 158.9 (Ar**C**), 140.2 (Ar**C**), 138.0 (Ar**C**H), 137.0 (Ar**C**H), 133.1 (Ar**C**), 132.3 (Ar**C**H), 130.2 (Ar**C**H), 128.6 (Ar**C**H), 128.4 (Ar**C**H), 128.0 (Ar**C**H), 127.7 (Ar**C**H), 127.3 (Ar**C**H), 121.5 (Ar**C**H), 120.6 (Ar**C**H), 67.4 (N**C**HPh), 61.3 (CO_2**C**H_2CH_2), 47.5 (N**C**H_2), 40.6 (2 overlapping signals, Ar**C**H_2CH and Ar**C**H_2CO_2), 37.6 (N**C**H_3), 24.9 (CH_2**C**H_2CH_2); HRMS (ESI): calcd. for $C_{25}H_{27}N_2O_2$, 387.2067. Found: $[MH]^+$, 387.2065 (0.5 ppm error)].

X-ray crystallographic data for compound **117** can be accessed via www.ccdc.cam.ac.uk/data_request/cif (CCDC 2004423)

6-(3-Bromophenyl)hex-5-yn-1-ol (S145)

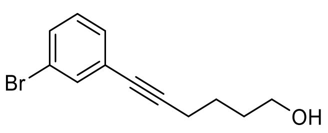

To a suspension of bis(triphenylphosphine)palladium(II) dichloride (7 mg, 0.010 mmol) and copper(I) iodide (6 mg, 0.030 mmol) in degassed triethylamine (1.0 mL) was added 3-bromoiodobenzene (0.130 mL, 283 mg, 1.00 mmol) followed by 5-hexyn-1-ol (0.130 mL, 118 mg, 1.20 mmol). The reaction mixture was stirred at 60 °C for 2h. Diethyl ether (10 mL) was added and the resulting suspension was filtered through Celite (washing with diethyl ether). The filtrate was concentrated *in vacuo* and then purified by column chromatography (SiO_2, 4:1 hexane:ethyl acetate) to yield the title compound **S145** (251 mg, 99%) as a colourless oil. R_f 0.17 (4:1 hexane:ethyl acetate); ν_{max}/cm^{-1} (thin film) 3326, 2939, 2865, 2230, 1590, 1555, 1473, 1404, 1065, 995, 879, 780, 729, 681; δ_H (400 MHz, $CDCl_3$) 7.52 (1H, t, *J* = 2.0 Hz, ArH),

7.39–7.35 (1H, d, *J* = 8.0 Hz, ArH), 7.31–7.27 (1H, d, *J* = 8.0 Hz, ArH), 7.11 (1H, t, *J* = 8.0 Hz, ArH), 3.66 (2H, t, *J* = 6.0 Hz, C**H**$_2$OH), 2.57 (1H, s, OH), 2.42 (2H, t, *J* = 6.5 Hz, C≡CCH$_2$), 1.75–1.60 (4H, m, CH$_2$C**H**$_2$C**H**$_2$CH$_2$); δ$_C$ (101 MHz, CDCl$_3$) 134.3 (ArC), 130.7 (ArC), 130.1 (ArC), 129.7 (ArC), 125.9 (ArC), 122.0 (ArC), 91.6 (ArC≡**C**), 79.6 (Ar**C**≡C), 62.2 (CH$_2$OH), 31.8 (CH$_2$), 24.9 (CH$_2$), 19.2 (C≡C**C**H$_2$); HRMS (ESI) calcd. for $C_{12}H_{13}{}^{79}BrONa$, 275.0042. Found: $[MNa]^+$, 275.0029 (4.8 error ppm).

Methyl 2-(3'-(6-hydroxyhex-1-yn-1-yl)-[1,1'-biphenyl]-2-yl)acetate (S146)

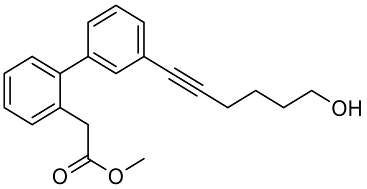

A dry round bottom flask was charged with 6-(3-bromophenyl)hex-5-yn-1-ol **S145** (100 mg, 0.395 mmol), methyl 2-(2-(4,4,5,5-tetramethyl-1,3,2-dioxaborolan-2-yl)phenyl)acetate **S3** (173 mg, 0.593 mmol) and tetrakis(triphenylphosphine)palladium (23 mg, 0.0198 mmol). To this was added caesium carbonate (0.660 mL, 2.1 M in water, 1.38 mmol) and 1,2-dimethoxyethane (3.3 mL) and the reaction mixture was heated to 80 °C for 18 h. The reaction mixture was concentrated *in vacuo* and redissolved in ethyl acetate (20 mL). The organic layer was washed with water (20 mL) and brine (20 mL) and the combined aqueous layers were extracted with ethyl acetate (2 x 20 mL). The combined organic layers were dried over MgSO$_4$, filtered and concentrated *in vacuo*. The crude product was purified by column chromatography (SiO$_2$, 7:3 hexane:ethyl acetate) to yield the title compound (97 mg, 76%) as a colourless oil. R$_f$ 0.27 (7:3 hexane:ethyl acetate); δ$_H$ (400 MHz, CDCl$_3$) 7.41–7.30 (6H, m, ArH), 7.27–7.20 (2H, m, ArH), 3.71 (2H, t, *J* = 6.0 Hz, OCH$_2$), 3.64 (3H, s, CH$_3$), 3.59 (2H, s, C**H**$_2$CO$_2$Me), 2.47 (2H, t, *J* = 6.5 Hz, C≡CCH$_2$), 1.81–1.65 (4H, m, 2 x CH$_2$); δ$_C$ (101 MHz, CDCl$_3$) 172.4 (CO$_2$), 141.7 (ArC), 141.1 (ArC), 132.4 (ArC), 131.7 (ArC), 130.4 (ArC), 130.3 (ArC), 130.1 (ArC), 128.5 (ArC), 128.2 (ArC), 127.8 (ArC), 127.3 (ArC), 123.9 (ArC), 90.3 (ArC≡**C**), 80.8 (Ar**C**≡C), 62.3 (OCH$_2$), 52.0 (CH$_3$), 38.7 (**C**H$_2$CO$_2$Me), 31.9 (CH$_2$), 25.0 (CH$_2$), 19.2 (C≡C**C**H$_2$); ν_{max}/cm^{-1} (thin film) 3393, 2945, 1736, 1596, 1474, 1434, 1338, 1252, 1209, 1159, 1062, 1003, 900, 800, 760, 703; HRMS (ESI); calcd. for $C_{21}H_{23}O_3^+$, 323.1642. Found: $[MH]^+$, 323.1644 (–0.7 error ppm).

Methyl 2-(3'-(6-hydroxyhexyl)-[1,1'-biphenyl]-2-yl)acetate (S147)

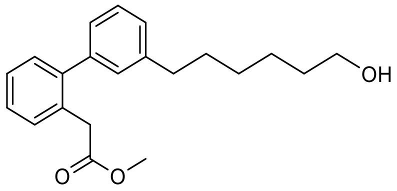

To a solution of methyl 2-(3'-(6-hydroxyhex-1-yn-1-yl)-[1,1'-biphenyl]-2-yl)acetate **S146** (94 mg, 0.292 mmol) in ethanol (1.9 mL) was added 10% palladium on carbon (9 mg). the reaction mixture was stirred under a hydrogen atmosphere at RT for 16 h. The suspension was filtered through Celite and the filtrate concentrated *in vacuo* to yield the title compound **S147** (92 mg, 97%) as a colourless oil. ν_{max}/cm^{-1} (thin film) 3369, 2929, 2856, 1736, 1600, 1476, 1434, 1338, 1249, 1211, 1157, 1053, 1005, 906, 796, 756, 709; δ_H (400 MHz, $CDCl_3$) 7.39–7.28 (5H, m, ArH), 7.20–7.12 (3H, m, ArH), 3.66–3.60 (7H, m, OCH_2, C**H**$_2CO_2C$**H**$_3$), 2.67 (2H, t, *J* = 8.0 Hz, $ArCH_2$), 1.72–1.54 (4H, m, 2 x CH_2), 1.44–1.36 (4H, m, 2 x CH_2); δ_C (101 MHz, $CDCl_3$) 172.6 (CO_2), 142.7 (2 x ArC), 141.0 (ArC), 131.8 (ArC), 130.4 (ArC), 130.2 (ArC), 129.4 (ArC), 128.2 (ArC), 127.5 (ArC), 127.3 (ArC), 127.2 (ArC), 126.6 (ArC), 62.9 (OCH_2), 52.0 (CH_3), 38.9 (**C**H_2CO_2), 35.9 ($ArCH_2$), 32.7 (CH_2), 31.4 (CH_2), 29.1 (CH_2), 25.7 (CH_2); HRMS (ESI); calcd. for $C_{21}H_{27}O_3$, 327.1955. Found: $[MH]^+$, 327.1961 (–1.8 error ppm).

2-(3'-(6-Hydroxyhexyl)-[1,1'-biphenyl]-2-yl)acetic acid (114a)

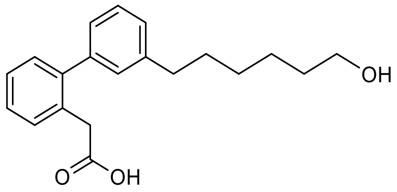

To a solution of methyl 2-(3'-(6-hydroxyhexyl)-[1,1'-biphenyl]-2-yl)acetate **S147** (216 mg, 0.662 mmol) in THF (1.3 mL) was added lithium hydroxide (1.5 mL, 0.5 M in water, 0.728 mmol) and the reaction mixture was heated to reflux for 18 h. The reaction mixture was concentrated *in vacuo* and purified by column chromatography (SiO_2, 10:9:1 ethyl acetate:hexane:acetic acid) to yield the title compound **114a** (182 mg, 88%) as a yellow oil. R_f 0.58 (10:9:1 ethyl acetate:hexane:acetic acid); ν_{max}/cm^{-1} (thin film) 3420, 2931, 2856, 1709, 1477, 1413, 1228, 1053, 797, 759, 709; δ_H (400 MHz, $CDCl_3$) 7.37–7.27 (5H, m, ArH), 7.19–7.10 (3H, m, ArH), 3.66–3.59 (4H, m, OCH_2, C**H**$_2CO_2H$), 2.64 (2H, t, *J* = 7.5 Hz, $ArCH_2$), 1.67 (2H, p, *J*

= 7.5 Hz, CH_2), 1.55 (2H, p, *J* = 6.5 Hz, CH_2), 1.43–1.30 (4H, m, 2 x CH_2); δ_C (101 MHz, $CDCl_3$) 176.4 (CO_2H), 142.9 (ArC), 142.7 (ArC), 141.1 (ArC), 131.6 (ArC), 130.7 (ArC), 130.3 (ArC), 129.6 (ArC), 128.3 (ArC), 127.6 (ArC), 127.5 (ArC), 127.4 (ArC), 126.5 (ArC), 62.9 (OCH_2), 38.8 (**C**H_2CO_2H), 35.5 ($ArCH_2$), 32.1 (CH_2), 31.0 (CH_2), 28.5 (CH_2), 25.4 (CH_2); HRMS (ESI); calcd. for $C_{20}H_{24}O_3Na^+$, 335.1618. Found: $[MNa]^+$, 335.1623 (–1.6 error ppm).

5-Oxa-1(1,3),2(1,2)-dibenzenacycloundecaphan-4-one (117a)

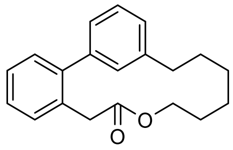

A round-bottom flask was charged with 2-(3'-(6-hydroxyhexyl)-[1,1'-biphenyl]-2-yl)acetic acid **114a** (96 mg, 0.307 mmol) and MeCN (3.1 mL). To this was added DIPEA (0.270 mL, 1.54 mmol), EDC·HCl (88 mg, 0.461 mmol) and HOBt (62 mg, 0.461 mmol) and the resulting mixture was stirred at room temperature for 18 h. The reaction mixture was concentrated *in vacuo* and purified by column chromatography (SiO_2, 9:1 hexane:ethyl acetate) to yield the title compound (19 mg, 21%) as a white solid, along with macrocyclic dimer **S148** (29 mg, 32%, see below). Data for **117a**: R_f 0.57 (9:1 hexane:ethyl acetate); m.p. 75–79 °C; δ_H (400 MHz, $CDCl_3$) 7.43–7.31 (4H, m, ArH), 7.30–7.26 (1H, m, ArH), 7.22–7.18 (1H, m, ArH), 7.14–7.08 (2H ,m, ArH), 4.16–4.12 (2H, m, OCH_2), 3.56 (2H, s, CH_2CO_2), 2.74–2.70 (2H, m, $ArCH_2$), 1.78–1.71 (2H, m, CH_2), 1.66–1.58 (2H, m, CH_2), 1.44–1.28 (4H, m, CH_2); δ_C (101 MHz, $CDCl_3$) 172.3 (CO_2), 142.9 (ArC), 142.1 (ArC), 141.2 (ArC), 131.9 (ArC), 131.2 (ArC), 130.1 (ArC), 129.8 (ArC), 128.6 (ArC), 127.9 (ArC), 127.6 (ArC), 127.3 (ArC), 126.3 (ArC), 65.9 (OCH_2), 39.2 (**C**H_2CO_2), 34.5 ($ArCH_2$), 28.9 (CH_2), 27.4 (CH_2), 27.3 (CH_2), 26.6 (CH_2); v_{max}/cm^{-1} (thin film) 3022, 2924, 2857, 1734, 1599, 1476, 1421, 1331, 1206, 1152, 1046, 977, 796, 755, 709; HRMS (ESI); calcd. for $C_{20}H_{23}O_2^+$, 295.1693. Found: $[MH]^+$, 295.1692 (0.0 error ppm).

5,16-Dioxa-1,12(1,3),2,13(1,2)-tetrabenzenacyclodocosaphane-4,15-dione (S148)

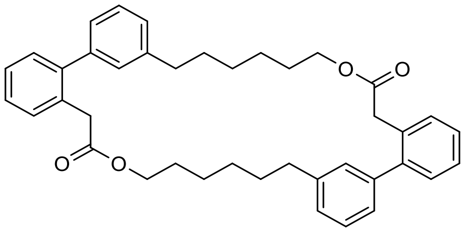

Following the above procedure, the title compound (29 mg, 32%) was also obtained, as a white solid. R_f 0.33 (9:1 hexane:ethyl acetate); m.p. 130–134 °C; δ_H (400 MHz, $CDCl_3$) 7.38–7.26 (10H, m, ArH), 7.16–7.09 (6H, m, ArH), 3.99 (4H, t, *J* = 6.5 Hz, OCH_2), 3.60 (4H, s, CH_2CO_2), 2.61 (4H, t, *J* = 7.5 Hz, $ArCH_2$), 1.66–1.49 (8H, m, CH_2), 1.38–1.23 (8H, m, CH_2); δ_C (101 MHz, $CDCl_3$) 172.2 (CO_2), 142.7 (ArC), 142.4 (ArC), 141.2 (ArC), 132.0 (ArC), 130.8 (ArC), 130.2 (ArC), 129.5 (ArC), 128.4 (ArC), 127.6 (ArC), 127.3 (2 x ArC), 126.7 (ArC), 65.0 (OCH_2), 39.2 (**C**H_2CO_2), 36.0 ($ArCH_2$), 31.5 (CH_2), 29.1 (CH_2), 28.7 (CH_2), 26.0 (CH_2); v_{max}/cm^{-1} (thin film) 3032, 2928, 2856, 1734, 1600, 1475, 1421, 1334, 1245, 1207, 1156, 984, 910, 796, 756, 709; HRMS (ESI); calcd. for $C_{40}H_{44}O_4Na^+$, 611.3132. Found $[MNa]^+$, 611.3130 (0.6 error ppm).

6-(6-Bromopyridin-2-yl)hex-5-yn-1-ol (S149)

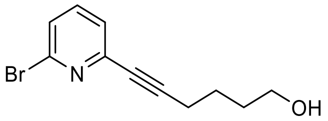

To a solution of 2,6-dibromopyridine (1.18 g, 5.00 mmol) in THF (20 mL) was added 5-hexyne-1-ol (0.550 mL, 491 mg, 5.00 mmol), triethylamine (4.90 mL, 3.54 g, 35.0 mmol), copper(I) iodide (95 mg, 0.500 mmol) and tetrakis(triphenylphosphine)palladium (289 mg, 0.250 mmol) and the reaction mixture was stirred at RT for 18 h. The reaction mixture was concentrated *in vacuo*, redissolved in DCM (50 mL) and washed with water (50 mL) and brine (50 mL). the organic layer was dried over $MgSO_4$, filtered and concentrated *in vacuo*. The crude product was purified by column chromatography (SiO_2, 7:3 hexane:ethyl acetate) to yield the title compound **S149** (684 mg, 54%) as a yellow oil. R_f 0.21 (7:3 hexane:ethyl acetate); δ_H (400 MHz, $CDCl_3$) 7.45 (1H, t, *J* = 7.5 Hz, ArH), 7.36 (1H, d, *J* = 7.5 Hz, ArH), 7.29 (1H, d, *J* = 7.5 Hz, ArH), 3.70–3.64 (2H, m, OCH_2), 2.49–2.43 (2H, m, $ArCH_2$), 1.76–1.65 (4H, m, 2 x CH_2); δc (101 MHz, $CDCl_3$) 144.3 (ArC), 141.5 (ArC), 138.4 (ArC), 127.1 (ArC), 125.8 (ArC), 92.8 (ArC≡**C**), 79.6 (Ar**C**≡C), 62.2 (OCH_2), 31.9 (CH_2), 24.6 (CH_2), 19.2 (C≡**C**CH_2); v_{max}/cm^{-1} (thin film) 3352, 2937,

2865, 2229, 1571, 1543, 1428, 1288, 1157, 1122, 1062, 980, 791, 728, 658; HRMS (ESI); calcd. for $C_{11}H_{13}{}^{79}BrNO^+$, 254.0175. Found: $[MH]^+$, 254.0173 (0.6 error ppm).

Methyl 2-(2-(6-(6-hydroxyhex-1-yn-1-yl)pyridin-2-yl)phenyl)acetate (S150)

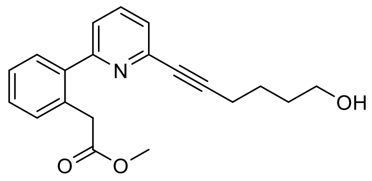

A dry round bottom flask was charged with 6-(6-bromopyridin-2-yl)hex-5-yn-1-ol **S149** (684 mg, 2.69 mmol), methyl 2-(2-(4,4,5,5-tetramethyl-1,3,2-dioxaborolan-2-yl)phenyl)acetate **S3** (1.18 g, 4.04 mmol) and tetrakis(triphenylphosphine)palladium (156 mg, 0.135 mmol). To this was added caesium carbonate (4.50 mL, 2.1 M in water, 9.42 mmol) and 1,2-dimethoxyethane (22 mL) and the reaction mixture was heated to 80 °C for 18 h. The reaction mixture was concentrated *in vacuo* and redissolved in ethyl acetate (50 mL). The organic layer was washed with water (50 mL) and brine (50 mL) and the combined aqueous layers were extracted with ethyl acetate (2 x 50 mL). The combined organic layers were dried over $MgSO_4$, filtered and concentrated *in vacuo*. The crude product was purified by column chromatography (SiO_2, 1:1 hexane:ethyl acetate) to yield the title compound **S150** (573 mg, 66%) as a colourless oil. R_f 0.28 (1:1 hexane:ethyl acetate); δ_H (400 MHz, $CDCl_3$) 7.68 (1H, t, J = 8.0 Hz, ArH), 7.43–7.31 (6H, m, ArH), 3.79 (2H, s, CH_2CO_2), 3.65 (2H, t, J = 6.0 Hz, OCH_2), 3.60 (3H, s, CH_3), 2.47 (2H, t, J = 6.5 Hz, C≡CCH_2), 1.77–1.64 (4H, m, 2 x CH_2); δ_C (101 MHz, $CDCl_3$) 172.4 (**C**O_2Me), 159.6 (ArC), 143.11 (ArC), 140.2 (ArC), 136.7 (ArC), 132.5 (ArC), 131.3 (ArC), 129.9 (ArC), 128.7 (ArC), 127.5 (ArC), 125.1 (ArC), 122.8 (ArC), 90.6 (ArC≡**C**), 81.0 (Ar**C**≡C), 62.3 (OCH_2), 52.0 (CH_3), 39.2 (**C**H_2CO_2Me), 31.9 (CH_2), 24.7 (CH_2), 19.2 (C≡C**C**H_2); ν_{max}/cm^{-1} (thin film) 3380, 2946, 2229, 1733, 1562, 1441, 1338, 1251, 1212, 1159, 1062, 1007, 816, 763; HRMS (ESI); calcd. for $C_{20}H_{22}NO_3^+$, 324.1594. Found: $[MH]^+$, 324.1599 (–1.6 error ppm).

Methyl 2-(2-(6-(6-hydroxyhexyl)pyridin-2-yl)phenyl)acetate (S151)

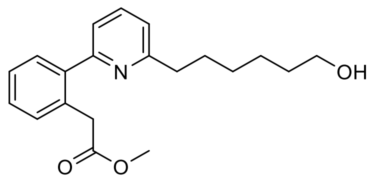

To a solution of methyl 2-(2-(6-(6-hydroxyhex-1-yn-1-yl)pyridin-2-yl)phenyl)acetate **S150** (537 mg, 1.66 mmol) in ethanol (11 mL) was added 10% palladium on carbon (54 mg). The reaction mixture was stirred under a hydrogen atmosphere at RT for 18 h. The reaction mixture was filtered through Celite and the filtrate concentrated *in vacuo* to yield the title compound **S151** (506 mg, 93%) as a yellow oil. ν_{max}/cm^{-1} (thin film) 3370, 2929, 2857, 1734, 1589, 1570, 1446, 1339, 1249, 1212, 1159, 1055, 1004, 813, 760; δ_H (400 MHz, $CDCl_3$) 7.67 (1H, t, *J* = 7.5 Hz, ArH), 7.46–7.42 (1H, m, ArH), 7.39–7.33 (3H, m, ArH), 7.29–7.26 (1H, m, ArH), 7.10 (1H, d, *J* = 7.5 Hz, ArH), 3.86 (2H, s, CH_2CO_2), 3.63–3.56 (5H, m, OCH_2, CH_3), 2.83 (2H, t, *J* = 8.0 Hz, $ArCH_2$), 1.81–1.72 (2H, m, CH_2), 1.60–1.52 (2H, m, CH_2), 1.45–1.37 (4H, m, 2 x CH_2); 172.5 (**C**O_2Me), 161.7 (ArC), 158.9 (ArC), 140.6 (ArC), 137.0 (ArC), 132.6 (ArC), 131.5 (ArC), 130.0 (ArC), 128.6 (ArC), 127.5 (ArC), 121.3 (ArC), 120.9 (ArC), 62.9 (OCH_2), 51.9 (CH_3), 39.4 (**C**H_2CO_2Me), 38.3 ($ArCH_2$), 32.7 (CH_2), 29.9 (CH_2), 29.2 (CH_2), 25.7 (CH_2); HRMS (ESI); calcd. for $C_{20}H_{26}NO_3$, 328.1907. Found: $[MH]^+$, 328.1907 (–0.1 error ppm).

2-(2-(6-(6-Hydroxyhexyl)pyridin-2-yl)phenyl)acetic acid (114b)

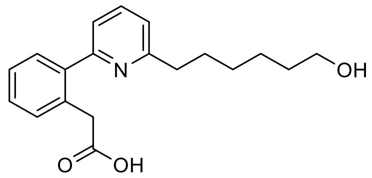

To a solution of methyl 2-(2-(6-(6-hydroxyhexyl)pyridin-2-yl)phenyl)acetate **S151** (493 mg, 1.50 mmol) in THF (3.0 mL) was added lithium hydroxide (3.30 mL, 0.5 M in water, 1.65 mmol) and the mixture was heated to reflux for 18 h. The reaction mixture was concentrated *in vacuo* and purified by column chromatography (SiO_2, 99:1 ethyl acetate:acetic acid) to yield the title compound **114b** (391 mg, 83%) as a colourless oil. R_f 0.42 (99:1 ethyl acetate:acetic acid); ν_{max}/cm^{-1} (thin film) 3416, 2930, 2857, 1720, 1598, 1578, 1500, 1449, 1256, 1145, 1053, 1014, 816, 764, 726, 697; δ_H (400 MHz, $CDCl_3$) 7.90 (1H, t, *J* = 8.0 Hz, ArH), 7.56–7.48 (3H, m, ArH), 7.46–7.37 (2H ,m, ArH), 7.31 (1H, d, *J* = 7.5 Hz, ArH), 3.67 (2H, s, C**H**$_2CO_2$H), 3.62 (2H, t, *J* = 6.5 Hz, OCH_2), 2.91 (2H, t, *J* = 8.0 Hz, $ArCH_2$), 1.78–1.69 (2H, m, CH_2), 1.60–1.51 (2H, m, CH_2), 1.44–1.37 (4H, m, 2 x CH_2); δ_C (101 MHz, $CDCl_3$) 173.0 (CO_2H), 161.0 (ArC), 157.1 (ArC), 139.7 (ArC), 137.6 (ArC), 133.1 (ArC), 131.7 (ArC), 130.7 (ArC), 130.0 (ArC), 127.9 (ArC), 122.6 (ArC), 122.5 (ArC), 62.8 (OCH_2), 42.2 (**C**H_2CO_2H), 36.5 ($ArCH_2$), 32.5 (CH_2), 30.3 (CH_2), 29.0 (CH_2), 25.4 (CH_2); HRMS (ESI); calcd. for $C_{19}H_{24}NO_3$, 314.1751. Found: $[MH]^+$, 314.1750 (0.2 error ppm).

5-Oxa-1(2,6)-pyridina-2(1,2)-benzenacycloundecaphan-4-one (117b)

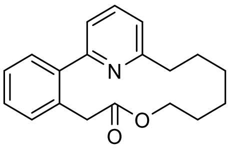

A round-bottom flask was charged with 2-(2-(6-(6-hydroxyhexyl)pyridin-2-yl)phenyl)acetic acid **114b** (391 mg, 1.25 mmol) and MeCN (12.5 mL). To this was added DIPEA (1.10 mL, 6.25 mmol), EDC·HCl (360 mg, 1.88 mmol) and HOBt (254 mg, 1.88 mmol) and the resulting mixture was stirred at room temperature for 18 h. The reaction mixture was concentrated *in vacuo* and purified by column chromatography (SiO_2, 4:1 hexane:ethyl acetate) to yield the title compound **117b** (97 mg, 26%) as a colourless oil, along with the macrocyclic dimer **S152** (see below, 35 mg, 9%). R_f 0.51 (4:1 hexane:ethyl acetate); δ_H (400 MHz, $CDCl_3$) 7.68 (1H, t, *J* = 8.0 Hz, ArH), 7.50–7.46 (1H ,m, ArH), 7.41–7.30 (4H, m, ArH), 7.11 (1H, d, *J* = 8.0 Hz, ArH), 4.08–4.04 (2H, m, OCH_2), 4.02 (2H, s, CH_2CO_2), 2.94–2.88 (2H, m, $ArCH_2$), 1.92–1.84 (2H, m, CH_2), 1.70–1.64 (2H, m, CH_2), 1.52–1.43 (2H, m, CH_2), 1.35–1.26 (2H, m, CH_2); δ_C (101 MHz, $CDCl_3$) 171.7 (CO_2), 161.3 (ArC), 159.4 (ArC), 140.4 (ArC), 136.9 (ArC), 132.4 (ArC), 132.3 (ArC), 130.1 (ArC), 128.3 (ArC), 127.5 (ArC), 121.0 (ArC), 120.8 (ArC), 63.9 (OCH_2), 39.6 (C**H**$_2$CO$_2$), 36.6 ($ArCH_2$), 27.7 (CH_2), 26.8 (CH_2), 25.7 (CH_2), 24.7 (CH_2); ν_{max}/cm^{-1} (thin film) 2926, 2857, 1737, 1588, 1570, 1445, 1344, 1208, 1152, 1039, 989, 909, 811, 757, 727, 699, 630; HRMS (ESI); calcd. for $C_{19}H_{22}NO_2^+$, 296.1645. Found: $[MH]^+$, 296.1644 (0.2 error ppm).

5,16-Dioxa-1,12(2,6)-dipyridina-2,13(1,2)-dibenzenacyclodocosaphane-4,15-dione (S152)

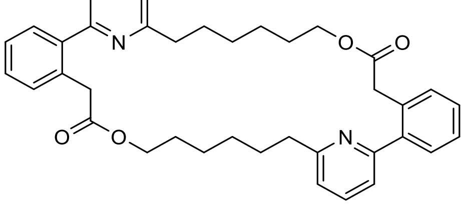

Following the above procedure, the title compound **S152** (35 mg, 9%) was also obtained, as a white solid. R_f 0.17 (4:1 hexane:ethyl acetate); m.p. 125–128 °C; ν_{max}/cm^{-1} (thin film) 2928, 2857, 1729, 1589, 1570, 1446, 1418, 1339, 1208, 1155, 1041, 990, 909, 812, 758, 728, 630; δ_H (400 MHz, $CDCl_3$) 7.66 (2H, t, *J* = 8.0 Hz, ArH), 7.45–7.41 (2H, m, ArH), 7.39–7.34 (6H, m, ArH), 7.30–7.26 (2H, m, ArH), 7.06 (2H, d, *J* = 8.0 Hz, ArH), 3.96 (4H, t, *J* = 7.0 Hz, OCH_2), 3.93 (4H, s, CH_2CO_2), 2.82–2.76 (4H, m, $ArCH_2$), 1.79–1.70 (4H, m, CH_2), 1.58–1.49 (4H, m, CH_2), 1.40–1.28

(8H, m, CH_2); 172.1 (CO_2), 161.3 (ArC), 159.3 (ArC), 140.6 (ArC), 137.0 (ArC), 132.9 (ArC), 132.0 (ArC), 129.9 (ArC), 128.5 (ArC), 127.5 (ArC), 121.2 (ArC), 120.7 (ArC), 64.7 (OCH_2), 39.4 (CH_2CO_2), 38.4 ($ArCH_2$), 29.8 (CH_2), 29.2 (CH_2), 28.6 (CH_2), 26.0 (CH_2); HRMS (ESI); calcd. for $C_{38}H_{43}N_2O_2$, 591.3217. Found: $[MH]^+$, 591.3221 (–0.6 error ppm).

X-ray crystallographic data for compound **S152** can be accessed via www.ccdc.cam.ac.uk/data_request/cif (CCDC 2267184).

2-(3-Bromophenyl)-*N*-(3-hydroxypropyl)-*N*-methylacetamide (S153)

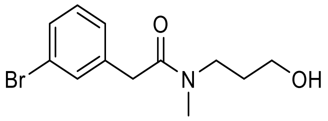

A solution of 3-bromophenylacetic acid (1.08 g, 5.00 mmol) in DCM (6.7 mL) was cooled to 0 °C before dropwise addition of thionyl chloride (0.550 mL, 892 mg, 7.50 mmol) and *N*,*N*-dimethylformamide (1 drop) and the reaction mixture was warmed to RT and stirred for 3 h. The reaction mixture was concentrated *in vacuo* and then redissolved in DCM (6.7 mL) before cooling to 0 °C and adding 3-methylamino-1-propanol (1.10 mL, 1.07 g, 12.0 mmol). The reaction mixture was warmed to RT and stirred for 2h. The reaction was quenched with water (40 mL) and extracted with DCM (3 x 40 mL). the combine organic layers were dried over $MgSO_4$, filtered and concentrated *in vacuo*. The crude product was purified by column chromatography (SiO_2, ethyl acetate) to yield the title compound **S153** (1.27 g, 89%) as a 4:1 mixture of rotamers and as a colourless oil. R_f 0.29 (ethyl acetate); ν_{max}/cm^{-1} (thin film) 3393, 2938, 2869, 1619, 1567, 1473, 1428, 1403, 1261, 1166, 1071, 943, 857, 7634, 683, 602; δ_H (400 MHz, $CDCl_3$) 7.42–7.32 (4H, m, 2 x ArH rotamer A, 2 x ArH rotamer B), 7.20–7.13 (4H, m, 2 x ArH rotamer A, 2 x ArH rotamer B), 3.72 (2H, s, $ArCH_2$ rotamer B), 3.68 (2H, s, $ArCH_2$ rotamer A), 3.59 (2H, t, *J* = 5.5 Hz, OCH_2 rotamer B), 3.52 (2H, t, *J* = 6.0 Hz, NCH_2 rotamer A), 3.45 (2H, t, *J* = 5.5 Hz, OCH_2 rotamer A), 3.41 (2H, t, *J* = 6.5 Hz, NCH_2 rotamer B), 2.98 (3H, s, NCH_3 rotamer A), 2.91 (3H, s, NCH_3 rotamer B), 1.75–1.65 (4H, m, $OCH_2C\mathbf{H}_2$ both rotamers); δ_C (101 MHz, $CDCl_3$) 171.8 (NCO rotamer A), 170.6 (NCO rotamer B), 137.9 (ArC rotamer B), 136.9 (ArC rotamer A), 132.0 (ArC rotamer B), 131.9 (ArC rotamer A), 130.3 (ArC rotamer A), 130.2 (ArC rotamer A), 130.1 (ArC rotamer B), 129.9 (ArC rotamer B), 127.8 (ArC rotamer B), 127.6 (ArC rotamer A), 122.7 (ArC rotamer A), 122.5 (ArC rotamer B), 58.5 (OCH_2 rotamer B), 58.1 (OCH_2 rotamer A), 46.9 (NCH_2 rotamer B), 44.4 (NCH_2 rotamer A), 40.4 ($ArCH_2$ rotamer

A), 39.8 ($ArCH_2$ rotamer B), 35.9 (NCH_3 rotamer A), 33.5 (NCH_3 rotamer B), 30.9 (OCH_2**C**H_2 rotamer B), 29.5 (OCH_2**C**H_2 rotamer A); HRMS (ESI); calcd. for $C_{12}H_{17}{}^{79}BrNO_2$, 286.0437. Found: $[MH]^+$, 286.0438 (–0.3 error ppm).

2-(3'-(2-((3-Hydroxypropyl)(methyl)amino)ethyl)-[1,1'-biphenyl]-2-yl)acetic acid (114c)

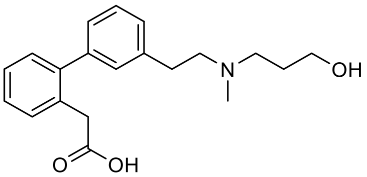

Lithium aluminium hydride (5.00 mL, 2.4 M in THF, 11.9 mmol) was diluted with THF (50 mL) and cooled to 0 °C. To this was added a solution of 2-(3-bromophenyl)-*N*-(3-hydroxypropyl)-*N*-methylacetamide **S153** (1.13 g, 3.95 mmol) in THF (20 mL) dropwise and the reaction mixture was heated to reflux for 2 h. The reaction was quenched with ethyl acetate followed by 4 M aq. NaOH (50 mL) and sat. aq. Rochelle salt (10 mL). The aqueous layer was extracted with ethyl acetate (3 x 50 mL) and the combined organic layers were dried over $MgSO_4$, filtered and concentrated *in vacuo*. The crude product was purified by column chromatography (SiO_2, 19:1 ethyl acetate:triethylamine) to yield mixture of 3-((3-bromophenethyl)(methyl)amino)propan-1-ol and 3-(methyl(phenethyl)amino)propan-1-ol (606 mg) which was used in the next step without further purification.

Next, to a dry round bottom flask was charged with the impure 3-((3-bromophenethyl)(methyl)amino)propan-1-ol (606 mg), methyl 2-(2-(4,4,5,5-tetramethyl-1,3,2-dioxaborolan-2-yl)phenyl)acetate (824 mg, 2.82 mmol) and tetrakis(triphenylphosphine)palladium (108 mg, 0.094 mmol) were added. To this was added caesium carbonate (3.10 mL, 2.1 M in water, 6.59 mmol) and 1,2-dimethoxyethane (16 mL) and the reaction mixture was heated to 80 °C for 18 h. The reaction mixture was concentrated *in vacuo* and redissolved in ethyl acetate (30 mL). The organic layer was washed with water (30 mL) and brine (30 mL) and the combined aqueous layers were extracted with ethyl acetate (2 x 30 mL). The combined organic layers were dried over $MgSO_4$, filtered and concentrated *in vacuo*. The crude product was purified by column chromatography (SiO_2, 19: ethyl acetate:triethylamine) to yield a mixture of methyl 2-(3'-(2-((3-hydroxypropyl)(methyl)amino)ethyl)-[1,1'-biphenyl]-2-yl)acetate and 3-

(methyl(phenethyl)amino)propan-1-ol (396 mg) which was used in the next step without further purification.

To a solution of the impure methyl 2-(3'-(2-((3-hydroxypropyl)(methyl)amino)ethyl)-[1,1'-biphenyl]-2-yl)acetate (396 mg) in THF (1.9 mL) was added lithium hydroxide (3.30 mL, 0.5 M in water, 1.66 mmol) and the reaction mixture was heated to reflux for 18 h. The reaction mixture was concentrated *in vacuo*. The crude product was purified by column chromatography (SiO_2, 10:9:1 methanol:ethyl acetate:triethylamine) to yield the title compound **114c** (167 mg, 13% over 3-steps) as a colourless oil. R_f 0.16 (10:9:1 methanol:ethyl acetate:triethylamine); δ_H (400 MHz, $CDCl_3$) 7.37–7.33 (1H, m, ArH), 7.29–7.17 (6H, m, ArH), 7.08–7.04 (1H, m, ArH), 3.59 (2H, t, *J* = 5.5 Hz, OCH_2), 3.46 (2H, s, C**H**$_2$$CO_2H$), 3.02–2.80 (6H, m, $ArCH_2$, 2 x NCH_2), 2.47 (3H, s, CH_3), 1.79–1.70 (2H, m, OCH_2C**H**$_2$); δ_C (101 MHz, $CDCl_3$) 177.5 (CO_2H), 142.1 (ArC), 141.9 (ArC), 137.4 (ArC), 134.8 (ArC), 131.1 (ArC), 130.0 (ArC), 129.8 (ArC), 128.6 (ArC), 127.7 (ArC), 127.5 (ArC), 127.3 (ArC), 126.5 (ArC), 60.3 (OCH_2), 57.6 (NCH_2), 54.6 (NCH_2), 41.8 (**C**H_2CO_2H), 40.3 (CH_3), 31.4 ($ArCH_2$), 27.3 (OCH_2**C**H_2); ν_{max}/cm^{-1} (thin film) 3312, 2923, 1579, 1475, 1423, 1366, 1260, 1062, 907, 800, 757, 726, 643; HRMS (ESI); calcd. for $C_{20}H_{26}NO_3$, 328.1907. Found: $[MH]^+$, 328.1906 (0.5 error ppm).

5-Methyl-9-oxa-5-aza-1(1,2),2(1,3)-dibenzenacycloundecaphan-10-one (117c)

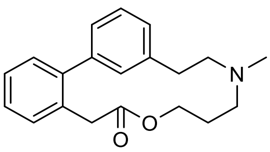

A round-bottom flask was charged with 2-(3'-(2-((3-hydroxypropyl)(methyl)amino)ethyl)-[1,1'-biphenyl]-2-yl)acetic acid **114c** (167 mg, 0.510 mmol) and MeCN (5.1 mL). To this was added DIPEA (0.440 mL, 2.55 mmol), EDC·HCl (147 mg, 0.765 mmol) and HOBt (103 mg, 0.765 mmol) and the resulting mixture was stirred at room temperature for 18 h. The reaction mixture was concentrated *in vacuo* and purified by column chromatography (SiO_2, 10:9:1 hexane:ethyl acetate:triethylamine) to yield the title compound (37 mg, 23%) as a colourless oil, along with macrocyclic dimer **S154** (see below, 32 mg, 20%). R_f 0.60 (10:9:1 hexane:ethyl acetate:triethylamine); ν_{max}/cm^{-1} (thin film) 2923, 2847, 2788, 1732, 1600, 1475, 1421, 1355, 1332, 1209, 1158, 1039, 908, 792, 755, 708, 625; δ_H (400 MHz, $CDCl_3$) 7.39–7.29 (5H, m, ArH), 7.27–7.25 (1H, m, ArH), 7.21–7.18 (1H, m, ArH), 7.16–7.13 (1H, m, ArH), 3.96–3.92 (2H, m,

OCH_2), 3.67 (2H, s, CH_2CO_2), 2.86–2.75 (4H, m, $ArCH_2$, NCH_2), 2.29 (2H, t, *J* = 6.5 Hz, NCH_2), 2.22 (3H, s, NCH_3), 1.69–1.61 (2H, m, CH_2); δ_C (101 MHz, $CDCl_3$) 171.9 (CO_2), 142.6 (ArC), 141.4 (ArC), 141.3 (ArC), 132.2 (ArC), 131.3 (ArC), 130.3 (ArC), 129.7 (ArC), 128.4 (ArC), 127.5 (3 x ArC), 126.6 (ArC), 62.8 (OCH_2), 58.3 (NCH_2), 53.8 (NCH_2), 42.5 (NCH_3), 39.9 (**C**H_2CO_2), 35.0 ($ArCH_2$), 26.2 (CH_2); HRMS (ESI); calcd. for $C_{20}H_{24}NO_2^+$, 310.1802. Found: $[MH]^+$, 310.1809 (–2.3 error ppm).

9,20-Dimethyl-5,16-dioxa-9,20-diaza-1,12(1,2),2,13(1,3)-tetrabenzenacyclodocosaphane-4,15-dione (S154)

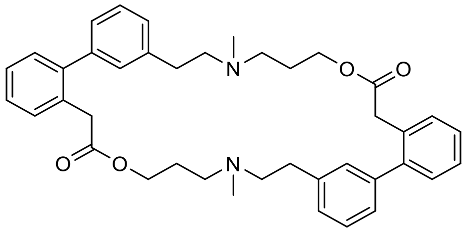

Following the above procedure, the title compound **S154** (32 mg, 20%) was also obtained, as a white solid. R_f 0.30 (10:9:1 hexane:ethyl acetate:triethylamine); m.p. 91–94 °C; δ_H (400 MHz, $CDCl_3$) 7.39–7.25 (10H, m, ArH), 7.17–7.10 (6H, m, ArH), 4.04 (4H, t, *J* = 6.5 Hz, OCH_2), 3.59 (4H, s, CH_2CO_2), 2.77–2.71 (4H, m, $ArCH_2$), 2.63–2.56 (4H, m, NCH_2), 2.39–2.33 (4H, m, NCH_2), 2.26 (6H, s, NCH_3), 1.72 (4H, p, *J* = 6.5 Hz, CH_2); δ_C (101 MHz, $CDCl_3$) 172.1 (CO_2), 142.6 (ArC), 141.3 (ArC), 140.4 (ArC), 132.0 (ArC), 130.7 (ArC), 130.2 (ArC), 129.7 (ArC), 128.4 (ArC), 127.7 (ArC), 127.6 (ArC), 127.3 (ArC), 126.9 (ArC), 63.2 (OCH_2), 59.5 (NCH_2), 53.9 (NCH_2), 42.2 (NCH_3), 39.2 (**C**H_2CO_2), 33.8 ($ArCH_2$), 26.6 (CH_2); ν_{max}/cm^{-1} (thin film) 2950, 2847, 2792, 1731, 1600, 1475, 1380, 1335, 1245, 1207, 1155, 1038, 909, 798, 756, 729, 709, 645; HRMS (ESI); calcd. for $C_{40}H_{47}N_2O_4^+$, 619.3530. Found: $[MH]^+$, 619.3524 (1.1 error ppm).

tert-Butyl 2-((6-(2-(benzyl(2-hydroxyethyl)amino)ethyl)pyridin-2-yl)methoxy)acetate (S155)

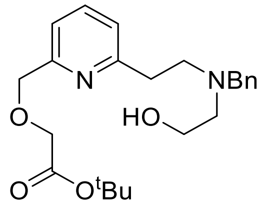

To a stirring solution of *tert*-butyl 2-((6-vinylpyridin-2-yl)methoxy)acetate **S82** (182 mg, 731 µmol) in acetonitrile (2 mL), 2-(benzylamino)ethanol (0.12 mL, 845 µmol) and scandium triflate (78 mg, 158 µmol) were added and stirred at 70 °C for 24 hours. The reaction mixture was concentrated under vacuum and purified via flash column chromatography (19:1 ethyl acetate:triethyl amine) to afford the title compound **S155** as a yellow oil (236 mg, 81%). R_f 0.28 (19:1 ethyl acetate:triethyl amine); v_{max} (thin film)/cm^{-1} 3354, 2978, 2934, 2815, 2247, 1746, 1594, 1578, 1456, 1368, 1229, 1132, 1055, 911, 846, 729, 698; ^{1}H NMR (400 MHz, $CDCl_3$) δ 7.55 (1H, dd, *J* = 7.5, 7.5 Hz, Ar**H**), 7.38 (1H, d, *J* = 7.5 Hz, Ar**H**), 7.166 – 7.10 (3H, m, Ar**H**), 6.98 – 6.93 (2H, m, Ar**H**), 6.89 (1H, d, *J* = 7.5 Hz, Ar**H**), 4.72 (2H, s, ArC**H**$_2$O), 4.11 (2H, s, COC**H**$_2$), 3.60 – 3.53 (4H, m, PhC**H**$_2$ + C**H**$_2$OH), 2.92 – 2.87 (2H, m, C**H**$_2$), 2.86 – 2.80 (2H, m, C**H**$_2$), 2.74 – 2.69 (2H, m, C**H**$_2$), 1.48 (9H, s, C**H**$_3$); ^{13}C NMR (101 MHz, $CDCl_3$) δ 169.5 (**C**O), 159.9 (Ar**C**), 157.2 (Ar**C**), 139.2 (Ar**C**), 137.3 (Ar**C**), 128.7 (Ar**C**), 128.2 (Ar**C**), 126.9 (Ar**C**), 112.2 (Ar**C**), 119.0 (Ar**C**), 81.8 (**C**(CH_3)$_3$), 74.0 (Ar**C**H_2O), 68.7 (CO**C**H_2), 59.7 (**C**H_2OH), 59.2 (Ph**C**H_2), 56.1 (**C**H_2CH_2OH), 53.8 ($ArCH_2$**C**H_2), 36.0 (Ar**C**H_2CH_2), 28.2 (**C**H_3); HRMS (ESI$^+$) calcd. for $C_{23}H_{33}N_2O_4$, 401.2235 Found: [MH]$^+$, 401.2432 (0.5 ppm error).

9-Benzyl-3,6-dioxa-9-aza-1(2,6)-pyridinacycloundecaphan-5-one (118)

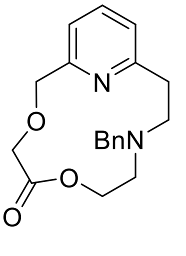

To a stirring solution of **S155** (87 mg, 217 µmol) in diethyl ether (2 mL), 4M HCl in dioxane (1.10 mL, 4.40 mmol) was added and the mixture was stirred at room temperature for 16 hours. The solvent was removed under vacuum using Et_2O (3 × 2 mL) to form an azeotropic mixture, to help ensure that all of the water from the hydrolysis was removed ahead of the

next step. The intermediate carboxylic acid was dissolved in $CHCl_3$ (2.17 mL) and DIPEA (0.37 mL, 2.12 mmol) was added followed by T3P (50% w/v in ethyl acetate, 278 mg, 437 μmol) and stirred at room temperature for 16 hours under argon. The reaction mixture was diluted with H_2O (10 mL) and extracted with DCM (3 × 10 mL). The combined organic phases were then washed with H_2O (10 mL), dried with sodium sulphate, filtered, concentrated under vacuum and purified via flash column chromatography (16:3:1 hexane:ethyl acetate:triethyl amine) to afford the title compound as a yellow film (41 mg, 58%); R_f 0.51 (12:7:1 hexane:ethyl acetate:triethyl amine); ν_{max} (thin film)/cm^{-1} 3061, 3026, 2919, 2797, 2149, 1753, 1648, 1595, 1576, 1455, 1361, 1276, 1188, 1109, 809, 734, 699; ^{1}H NMR (400 MHz, $CDCl_3$) δ 7.54 (1H, dd, *J* = 7.5, 7.5 Hz, Ar**H**), 7.25 – 7.18 (4H, m, Ar**H**), 7.19 – 7.08 (2H, m, Ar**H**), 6.92 – 6.86 (1H, m, Ar**H**), 4.72 (2H, s, ArC**H**$_2$O), 4.31 (2H, s, COC**H**$_2$), 3.99 – 3.92 (2H, m, COOC**H**$_2$), 3.60 (2H, s, PhC**H**$_2$), 3.04 – 2.97 (2H, m, C**H**$_2$), 2.91 – 2.84 (2H, m, C**H**$_2$), 2.64 – 2.57 (2H, m, C**H**$_2$); ^{13}C NMR (101 MHz, $CDCl_3$) δ 170.8 (**C**O), 160.7 (Ar**C**), 155.5 (Ar**C**), 139.5 (Ar**C**), 137.0 (Ar**C**), 129.0 (Ar**C**), 128.2 (Ar**C**), 127.0 (Ar**C**), 123.4 (Ar**C**), 121.9 (Ar**C**), 75.9 (Ar**C**H$_2$O), 68.2 (CO**C**H$_2$), 64.3 (COO**C**H$_2$), 59.2 (Ph**C**H$_2$), 54.9 (**C**H$_2$), 52.7 (**C**H$_2$), 36.9 (**C**H$_2$); HRMS (ESI$^+$) calcd. For $C_{19}H_{25}N_2O_4$, 345.1809; Found: [MH]$^+$, 345.1806 (0.8 ppm error).

***tert*-Butyl 2-((6-(2-(2-(hydroxymethyl)piperidin-1-yl)ethyl)pyridin-2-yl)methoxy)acetate (S156)**

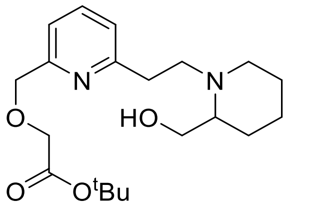

To a stirring solution of *tert*-butyl 2-((6-vinylpyridin-2-yl)methoxy)acetate **S82** (137 mg, 551 μmol) in acetonitrile (2 mL), 2-piperidine methanol (71 mg, 616 μmol) and scandium triflate (31 mg, 63.0 μmol) were added and stirred at 70 °C for 24 hours. The reaction mixture was purified via flash column chromatography (19:1 ethyl acetate:triethyl amine) to afford the title compound **S156** as a yellow oil (88 mg, 44%). R_f 0.21 (19:1 ethyl acetate:triethyl amine). ν_{max} (thin film)/cm^{-1} 3353, 2931, 2857, 1746, 1594, 1578, 1457, 1368, 1130, 1041, 846. ^{1}H NMR (400 MHz, $CDCl_3$) δ 7.57 (1H, dd, *J* = 7.5, 7.5 Hz, Ar**H**), 7.32 (1H, d, *J* = 7.5 Hz, Ar**H**), 7.01 (1H, d, *J* = 7.5 Hz, Ar**H**), 4.67 (2H, s, ArC**H**$_2$O), 4.06 (2H, s, COC**H**$_2$), 3.74 (1H, dd, *J* = 11.5, 4.0 Hz,

C**H**$_2$OH), 3.44 (2H, dd + s (Br), *J* = 11.5, 4.0 Hz, C**H**$_2$OH + O**H**), 3.19 (1H, dt, *J* = 13.0, 7.5 Hz, ArCH$_2$C**H**$_2$), 3.00 – 2.82 (3H, m, C**H**$_2$), 2.82 – 2.72 (1H, m, C**H**$_2$), 2.39 (1H, dtt, *J* = 8.0, 8.0, 4.0 Hz, C**H**), 2.31 – 2.23 (1H, m, C**H**$_2$), 1.69 – 1.17 (6H, m, C**H**$_2$), 1.44 (9H, s, C**H**$_3$). ^{13}C NMR (101 MHz, CDCl$_3$) δ 169.5 (**C**O), 159.8 (Ar**C**), 157.5 (Ar**C**), 137.1 (Ar**C**), 122.2 (Ar**C**), 119.1 (Ar**C**), 81.7 (**C**(CH$_3$)$_3$), 74.0 (Ar**C**H$_2$O), 68.6 (CO**C**H$_2$), 62.9 (**C**H$_2$), 61.0 (**C**H), 52.5 (N**C**H$_2$), 51.3 (**C**H$_2$), 34.7 (**C**H$_2$), 28.2 (**C**H$_3$), 27.6 (**C**H$_2$), 24.4 (C**H**$_2$), 23.7 (**C**H$_2$). HRMS (ESI$^+$) calcd. for $C_{20}H_{33}N_2O_4$ 365.2435; Found: [MH]$^+$, 365.2430 (1.2 ppm error).

6,9-Dioxa-1(2,6)-pyridina-4(1,2)-piperidinacyclodecaphan-7-one (119)

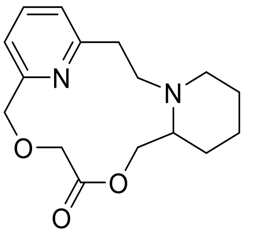

To a stirring solution of *tert*-butyl 2-((6-(2-(2-(hydroxymethyl)piperidin-1-yl)ethyl)pyridin-2-yl)methoxy)acetate **S156** (78 mg, 214 µmol) in diethyl ether (2 mL), 4M HCl in dioxane (0.55 mL, 2.20 mmol) was added and the mixture was stirred at room temperature for 16 hours. The solvent was removed under vacuum using Et$_2$O (3 × 2 mL) to form an azeotropic mixture, to help ensure that all of the water from the hydrolysis was removed ahead of the next step. The intermediate carboxylic acid was dissolved in DMF (1.56 mL) and DIPEA (0.57 mL, 3.27 mmol) was added followed by EDCI (81 mg, 423 µmol), HOBt (41 mg, 303 µmol) and stirred at room temperature for 16 hours under argon. The reaction mixture was diluted with H$_2$O (10 mL) and extracted with DCM (3 × 10 mL). The combined organic phases were then washed with H$_2$O (10 mL), dried with sodium sulphate, filtered, concentrated under vacuum and purified via flash column chromatography (12:7:1 hexane:ethyl acetate:triethyl amine) to afford the title compound **119** as a colourless oil (32 mg, 51%). R_f 0.31 (12:7:1 hexane:ethyl acetate:triethyl amine); ν_{max} (thin film)/cm^{-1} 3363, 2932, 2856, 2792, 1748, 1592, 1576, 1457, 1256, 1195, 1110, 807; ^{1}H NMR (400 MHz, CDCl$_3$) δ 7.57 (1H, dd, *J* = 7.5, 7.5 Hz, Ar**H**), 7.14 (1H, d, *J* = 7.5 Hz, Ar**H**), 7.06 (1H, d, *J* = 7.5 Hz, Ar**H**), 4.77 (1H, d, *J* = 2.0 Hz, ArC**H**$_2$O), 4.27 – 4.16 (2H, m, C**H**$_2$), 4.04 – 3.94 (2H, m, C**H**$_2$), 3.41 (1H, ddd, *J* = 13.0, 8.5, 5.0 Hz, C**H**$_2$), 3.11 – 3.03 (1H, m, C**H**$_2$), 2.98 – 2.78 (2H, m, C**H**$_2$), 2.54 (1H, s (Br), C**H**), 2.33 – 2.20 (1H, m, C**H**$_2$), 1.70–1.19 (6H, m, C**H**$_2$); ^{13}C NMR (101 MHz, CDCl$_3$) δ 170.7 (**C**O), 160.6 (Ar**C**), 155.8 (Ar**C**), 137.1 (Ar**C**), 123.2 (Ar**C**), 121.3 (Ar**C**), 74.3 (**C**H$_2$), 67.7 (**C**H$_2$), 66.7 (**C**H$_2$), 58.5 (**C**H), 51.9 (**C**H$_2$),

51.7 (**C**H_2), 35.2 (**C**H_2), 28.7 (**C**H_2), 24.6 (**C**H_2), 23.8 (**C**H_2); HRMS (ESI$^+$) Calcd. for $C_{16}H_{23}N_2O_3$ 291.1703; Found: $[MH]^+$ 291.1725 (–4.1 ppm error).

Methyl 4-[benzyl(3-hydroxypropyl)amino]butanoate (S157)

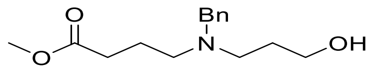

Methyl 4-bromobutyrate (0.69 mL, 5.54 mmol) and 3-(benzylamino)propan-1-ol (1.32 mL, 8.29 mmol) were dissolved in acetonitrile (55 mL) and potassium carbonate (1.53 g, 11.05 mmol) was added and the solution heated, at reflux, to 85 ^{o}C for 3 h. Upon completion the solution was diluted with water (50 mL) before being extracted with ethyl acetate (3 × 50 mL) and washed with sat. aq. brine (40 mL). The combined organic extracts were dried over $MgSO_4$, filtered and removed *in vacuo*. Purification by flash column chromatography (SiO_2, ethyl acetate) afforded the *title compound* **S157** as a colourless oil (572 mg, 39%); R_f 0.30 (ethyl acetate); ν_{max}/cm^{-1} (neat) 3424, 2950, 2813, 1736; δ_H (400 MHz, $CDCl_3$) 7.33–7.26 (5H, m, Ph), 3.73 (2H, t, *J* = 3.7 Hz, C**H₂**OH), 3.63 (1H, s, OC**H₃**), 3.58 (2H, s, NC**H₂**Ph), 2.67 (2H, t, *J* = 2.7 Hz, C**H₂**CO_2), 2.60 (2H, t, *J* = 2.5, NC**H₂**CH_2), 2.29 (2H, t, *J* = 2.3, NC**H₂**CH_2), 1.88–1.81 (2H, m, CH_2C**H₂**CH_2), 1.76–1.68 (2H, m, CH_2C**H₂**CH_2); δ_C (101 MHz, $CDCl_3$) 173.6 (**C**O_2Me), 138.0 (**C**), 129.2 (**C**H), 128.6 (**C**H), 127.4 (**C**H), 64.2 (**C**H_3O), 58.8 (**C**H_2OH), 54.0 (**C**H_2N), 52.9 (**C**H_2N), 51.7 (**C**H_2N), 31.8 (CH_2**C**H_2CO_2), 28.1 (CH_2**C**H_2), 22.0 (CH_2**C**H_2); HRMS (ESI): calcd for $C_{15}H_{24}NO_3$ 266.1751. Found: $[MNa]^+$, 266.1744 (2.6 ppm error).

Methyl 4-[benzyl(3-bromopropyl)amino]butanoate (S158)

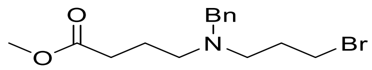

Methyl 4-[benzyl(3-hydroxypropyl)amino]butanoate **S157** (572 mg, 2.17 mmol) and carbon tetrabromide (787 mg, 2.37 mmol) were dissolved in CH_2Cl_2 (8.6 mL) and cooled to 0 °C before triphenylphosphine (622 mg, 2.37 mmol) was added portion-wise. After stirring for 1 h at RT, the reaction mixture was concentrated directly and then purified by flash column chromatography (SiO_2, 30% ethyl acetate in hexanes → 40% ethyl acetate in hexanes) affording the *title compound* **S158** as a yellow oil (582 mg, 72%); R_f 0.81 (50% ethyl acetate in hexanes); ν_{max}/cm^{-1} (neat) 2950, 2806, 1734, 1494, 1452, 1436, 1365, 1256, 1199, 1170, 1125, 1074, 1028; δ_H (400 MHz, $CDCl_3$) 7.27–7.20 (5H, m, Ph), 3.60 (3H, s, C**H₃**O), 3.52 (2H, s, C**H₂**Ph),

3.40 (2H, t, *J* = 6.8 Hz, C**H₂**Br), 2.54 (2H, t, *J* = 6.7 Hz, NC**H₂**), 2.42 (2H, t, *J* = 6.9 Hz, NC**H₂**), 2.30 (2H, t, *J* = 7.3 Hz, CO_2C**H₂**), 1.99–1.94 (2H, m, C**H₂**), 1.81–1.76 (2H, m, C**H₂**); δ_C (101 MHz, $CDCl_3$) 174.4 (**C**O), 139.5 (Ar**C**), 129.2 (Ar**C**), 128.6 (Ar**C**), 127.4 (Ar**C**), 59.0 (**C**H_2Ph), 53.2 (**C**H_2N), 52.2 (**C**H_2N), 51.9 (**C**H_3O), 32.2 (**C**H_2), 31.9 (**C**H_2), 30.8 (**C**H_2), 22.7 (**C**H_2); HRMS (ESI): calcd. for $C_{15}H_{23}{}^{79}BrNO_2$, 328.0907. Found: $[MH]^+$, 328.0899 (2.3 ppm error)].

Methyl 4-[benzyl({3-[benzyl(3-hydroxypropyl)amino]propyl})amino]butanoate (S159)

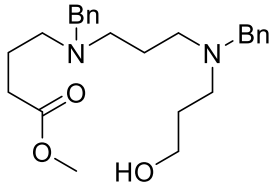

Methyl 4-[benzyl(3-bromopropyl)amino]butanoate **S158** (1.21 g, 3.71 mmol), 3-(benzylamino)propan-1-ol (885 µL, 5.56 mmol) and potassium carbonate (1.03 g, 7.42 mmol) were dissolved in acetonitrile (37 mL). After stirring at 85 °C for 3 h the reaction mixture was concentrated directly and then purified by flash column chromatography (SiO_2, 5% methanol in ethyl acetate) affording the *title compound* **S159** as a yellow oil (710 mg, 45%); R_f. 0.53 (10% methanol in ethyl acetate); ν_{max}/cm^{-1} (neat) 3417, 3027, 2948, 2803, 1735, 1602, 1494, 1452, 1366, 1170, 1071, 1028; δ_H (400 MHz, $CDCl_3$) 7.33–7.23 (10H, m, 2 × Ph), 3.73–3.70 (2H, m, C**H₂**OH), 3.64 (3H, s, OC**H₃**), 3.56 (2H, s, C**H₂**Ph), 3.51 (2H, s, C**H₂**Ph), 2.65–2.62 (2H, t, *J* = 5.9 Hz, NC**H₂**), 2.47–2.38 (6H, m, 3 × NC**H₂**), 2.31–2.27 (2H, t, *J* = 7.5 Hz, OCC**H₂**), 1.75–1.65 (6H, m, 3 × C**H₂**); δ_C (101 MHz, $CDCl_3$) 174.2 (**C**O), 139.6 (Ar**C**), 138.3 (Ar**C**), 129.1 (Ar**C**), 128.8 (Ar**C**), 128.4 (Ar**C**), 128.1 (Ar**C**), 127.2 (Ar**C**), 126.8 (Ar**C**), 64.0 (**C**H_2OH), 58.9 (**C**H_2Ph), 58.6 (**C**H_2Ph), 54.0 (**C**H_2N), 52.8 (**C**H_2N), 51.9 (**C**H_2N), 51.7 (**C**H_2N), 51.4 (CO**C**H_3), 31.7 (**C**H_2), 28.0 (**C**H_2), 24.3 (**C**H_2), 22.4 (**C**H_2); HRMS (ESI): calcd. for $C_{25}H_{37}N_2O_3$, 413.2799. Found: $[MH]^+$, 413.2795 (1.0 ppm error)].

4-[Benzyl({3-[benzyl(3-hydroxypropyl)amino]propyl})amino]butanoic acid (S160)

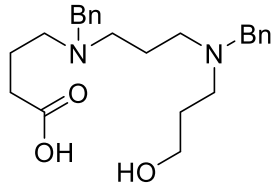

Methyl 4-[benzyl({3-[benzyl(3 hydroxypropyl)amino]propyl})amino]butanoate **S159** (710 mg, 1.72 mmol) was dissolved in aqueous lithium hydroxide solution (0.5 M, 12 mL, 6.03 mmol) and THF (12 mL). The resulting bi-phasic solution was vigorously stirred for 18 h. Upon completion, the solvent was removed *in vacuo*. The crude material was then passed through a silica plug and eluted with 50% methanol in ethyl acetate to afford the *title compound* **S160** as a colourless oil (272 mg, 40%); R_f. 0.17 (20% methanol in ethyl acetate); ν_{max}/cm^{-1} (neat) 2943, 2811, 1575, 1494, 1453, 1407, 1072; δ_H (400 MHz, $CDCl_3$) 7.31–7.21 (10H, m, 2 × Ph), 3.69–3.64 (4H, m, C**H₂**Ph and HOC**H₂**), 3.54 (2H, s, C**H₂**Ph), 2.62–2.55 (4H, m, 2 × C**H₂**N), 2.48–2.45 (2H, m, C**H₂**N), 2.38 (2H, t, *J* = 7.1 Hz, C**H₂**N), 2.26 (2H, t, *J* = 6.5 Hz, C**H₂**CO_2H), 1.79–1.68 (6H, m, 3 × C**H₂**); δ_C (101 MHz, $CDCl_3$) 179.3 (**C**O_2H), 137.9 (Ar**C**), 136.4 (Ar**C**), 129.7 (Ar**C**H), 129.4 (Ar**C**H), 128.6 (Ar**C**H), 128.5 (Ar**C**H), 127.8 (Ar**C**H), 127.4 (Ar**C**H), 62.6 (**C**H_2OH), 58.9 (**C**H_2Ph), 58.3 (**C**H_2Ph), 53.6 (**C**H_2N), 52.8 (**C**H_2N), 51.5 (**C**H_2N), 51.2 (**C**H_2N), 35.4 (**C**H_2), 31.0 (**C**H_2), 28.3 (**C**H_2), 23.4 (**C**H_2); HRMS (ESI): calcd. for $C_{24}H_{35}N_2O_3$, 299.2624. Found: $[MH]^+$, 299.2640 (0.5 ppm error)].

5,9-Dibenzyl-1-oxa-5,9-diazacyclotridecan-13-one (120)

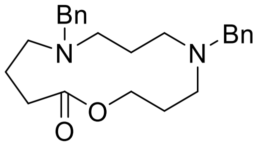

To a stirring solution of 4-[benzyl({3-[benzyl(3-hydroxypropyl)amino]propyl})amino]butanoic acid **S160** (186 mg, 0.47 mmol) in acetonitrile (5 mL), was added diisopropylethylamine (435 µL, 2.50 mmol), followed by the addition of EDC·HCl (144 mg, 0.75 mmol) and HOBt (101 mg, 0.75 mmol). After stirring for 18 h at RT, the reaction mixture was concentrated directly and then purified by flash column chromatography (SiO_2, 50% ethyl acetate in hexanes) to afford the *title compound* **120** as a colourless oil (87 mg, 49%); R_f. 0.40 (50% ethyl acetate in hexanes); ν_{max}/cm^{-1} (neat) 3027, 2928, 2796, 1728, 1602, 1494, 1452, 1372, 1356, 1340, 1231, 1208, 1171, 1119, 1070, 1028; δ_H (400 MHz, $CDCl_3$) 7.29–7.20 (10H, m, 2 × Ph), 4.23–4.20 (2H,

m, OC**H₂**), 3.49 (2H, s, C**H₂**Ph), 3.47 (2H, s, C**H₂**Ph), 2.65–2.62 (2H, t, *J* = 6.3 Hz, NC**H₂**), 2.51–2.44 (4H, m, 2 × NC**H₂**), 2.35–2.31 (4H, m, NC**H₂** and C**H₂**CO_2), 1.19–1.68 (4H, m, 2 × C**H₂**), 1.59–1.53 (2H, m, C**H₂**); δ_C (101 MHz, $CDCl_3$) 174.3 (**C**O_2), 140.1 (Ar**C**), 139.8 (Ar**C**), 129.2 (Ar**C**H), 129.0 (Ar**C**H), 128.3 (Ar**C**H), 128.2 (Ar**C**H), 127.0 (Ar**C**H), 126.9 (Ar**C**H), 62.1 (**C**H_2O), 59.4 (**C**H_2Ph), 59.2 (**C**H_2Ph), 52.7 (**C**H_2N), 52.6 (**C**H_2N), 52.2 (**C**H_2N), 49.7 (**C**H_2N), 31.9 (**C**H_2CO_2), 26.6 (**C**H_2), 26.4 (**C**H_2), 23.3 (**C**H_2); HRMS (ESI): calcd. for $C_{24}H_{33}N_2O_2$, 381.2537. Found: $[MH]^+$, 381.2530 (1.7 ppm error)]

Methyl 2-(((2-((3-hydroxypropyl)(phenyl)amino)ethyl)(phenyl)amino)methyl)benzoate (S161)

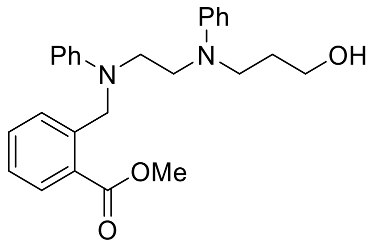

To a stirring solution of potassium carbonate (0.444 g, 3.21 mmol) in acetonitrile (10.7 mL), 3-(phenyl(2-(phenylamino)ethyl)amino)propan-1-ol **16** (0.139 g, 0.773 mmol) was added followed by methyl 2-(bromomethyl) benzoate (0.245 g, 1.07 mmol). The reaction mixture was heated at reflux at 85 °C for 18 h under argon before filtering through Celite washing with DCM, concentrating under vacuum and purifying by flash column chromatography (5:1 → 2:1 hexane:ethyl acetate) to afford the title compound **S161** as a colourless oil (0.338 g, 75%). R_f = 0.65 (1:1 hexane:ethyl acetate); ν_{max}/cm^{-1} (thin film) 3408, 2949, 1715, 1596, 1503, 1253, 741, 692; δ_H (400 MHz, $CDCl_3$) 8.01 (1H, d, *J* = 7.3, Ar**H**), 7.42–7.15 (7H, m, Ar**H**), 6.76–6.62 (6H, m, Ar**H**), 4.94 (2H, s, ArC**H₂**), 3.89 (3H, s, OC**H₃**), 3.70 (2H, t, *J* = 5.7, C**H₂**), 3.62–3.58 (4H, 2 x C**H₂**), 3.45 (2H, t, *J* = 6.9, C**H₂**), 1.86–1.79 (2H, m, CH_2C**H₂**CH_2); δ_C (101 MHz, $CDCl_3$) 167.8 (**C**O), 148.2 (Ar**C**), 147.7 (Ar**C**), 140.8 (Ar**C**), 132.7 (Ar**C**H), 131.4 (Ar**C**H), 129.6 (Ar**C**H), 129.5 (ArCH), 128.6 (Ar**C**), 127.3 (Ar**C**H), 126.8 (Ar**C**H), 116.7 (Ar**C**H), 116.6 (Ar**C**H), 112.7 (Ar**C**H) 111.9 (Ar**C**H), 60.8 (**C**H_2), 53.6 (**C**H_2), 52.1 (O**C**H_3), 48.5 (**C**H_2), 48.4 (**C**H_2), 48.3 (**C**H_2), 30.2 (**C**H_2); HRMS (ESI) calcd. for $C_{26}H_{30}N_2O_3$ 419.2329. Found $[MH]^+$ 419.2329 (0 ppm error).

Macrocycle (121)

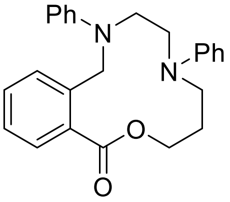

To a stirring solution of methyl 2-(((2-((3-hydroxypropyl)(phenyl)amino)ethyl)(phenyl)amino)methyl)benzoate **S161** (55 mg, 0.131 mmol) in methanol (0.6 mL), aqueous lithium hydroxide (0.5 M) (0.29 mL, 0.144 mmol) was added and heated for at 60 °C for 3 hours. The solvent was removed under vacuum using dichloromethane (3 × 10 mL) to form an azeotropic mixture, to help ensure that all of the water from the hydrolysis was removed ahead of the next step. The intermediate lithium carboxylate salt (0.131 mmol, assuming full conversion) was then disolved in DMF (1.3 mL) and diisopropylethylamine (110 µL, 0.655 mmol) was added, followed by the addition of EDC·HCl (37.7 mg, 0.197 mmol) and HOBt (26.6 mg, 0.197 mmol). After stirring for 18 h at RT, the reaction mixture was quenched by the addition of water (15 mL), extracted with DCM (3 × 20 mL), dried over $MgSO_4$ and concentrated under vacuum. Purification by flash column chromatography (SiO_2, 3:1 → 1:1 hexane:ethyl acetate) afforded the *title compound* **121** as a colourless oil (28 mg, 55%). In solution in $CDCl_3$, compound **121** exists as a 10:4:1 (A:B:C) mixture of rotamers. $R_{f.}$ 0.60 (1:1 hexane:ethyl acetate); ν_{max}/cm^{-1} (neat) 2923, 1713, 1596, 1503, 1259, 736; δ_H (400 MHz, $CDCl_3$) 7.96 (1H, d, *J* = 7.6, Ar**H**, rotamer C), 7.93 (1H, d, *J* = 7.6, Ar**H**, rotamer B), 7.60 (1H, d, *J* = 7.6, Ar**H**, rotamer A), 7.41–6.58 (13H, m, 13 x Ar**H**, all rotamers), 4.91 (2H, s, ArC**H₂**, rotamer C), 4.86 (2H, s, ArC**H₂**, Ar**H**, rotamer B), 4.75 (2H, s, ArC**H₂**, rotamer A), 4.46 (2H, t, *J* = 5.3, C**H₂**, rotamer A), 4.26–4.24 (2H, m, C**H₂**, rotamers B and C), 3.62–3.34 (6H, m, 3 x C**H₂**, all rotamers), 2.10–1.87 (2H, m, CH_2C**H₂**CH_2, all rotamers); δ_C (101 MHz, $CDCl_3$) data for rotamers A and B only: 169.5 (C=O, rotamer A), 167.6 (C=O, rotamer B) 149.8 (ArC, rotamer A), 149.2 (ArC, rotamer A), 148.1 (ArC, rotamer B) 148.0 (ArC, rotamer B) 140.1 (ArC, rotamer B) 138.6 (ArC, rotamer A) 132.8 (ArC, rotamer A), 132.5 (ArC, rotamer B), 131.5 (ArCH, rotamer B) 131.2 (ArCH, rotamer A), 130.5 (ArCH), 129.7 (ArCH), 129.6 (ArCH), 129.2 (ArCH), 128.8 (ArCH), 127.7 (ArCH), 127.5 (ArCH), 127.0 (ArCH), 118.1 (ArCH, rotamer A), 117.6 (ArCH, rotamer A), 116.9 (ArCH, rotamer B), 116.8 (ArCH, rotamer B), 115.2 (ArCH, rotamer A), 113.6 (ArCH, rotamer A), 112.6 (ArCH, rotamer B), 111.9 (ArCH, rotamer

B), 64.6 (OCH_2, rotamer A), 63.2 (OCH_2, rotamer B), 53.7 (NCH_2, rotamer B), 52.7 (NCH_2, rotamer A), 51.0 (NCH_2, rotamer A), 49.5 (NCH_2, rotamer A), 48.6 (NCH_2, rotamer B), 46.8 (NCH_2, rotamer A), 27.6 (CH_2, rotamer A), 26.8 (CH_2, rotamer B); HRMS (ESI): calcd. for $C_{25}H_{27}N_2O_2$, 387.2067. Found: $[MH]^+$, 387.2081 (–3.4 ppm error)

Methyl 2-((benzyl((1-(3-hydroxypropyl)piperidin-2-yl)methyl)amino)methyl)benzoate (S162)

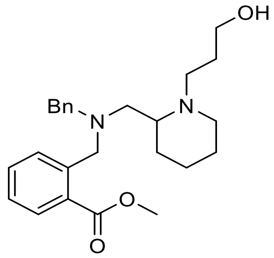

Methyl 2-((benzyl(piperidin-2-ylmethyl)amino)methyl)benzoate **S107** (255 mg, 0.723 mmol) was dissolved in acetonitrile (1.4 mL) and to this was added potassium carbonate (100 mg, 0.724 mmol) and 3-bromo-1-propanol (0.070 mL, 100 mg, 0.719 mmol). The reaction mixture was heated to reflux for 20 h. The solid material was filtered off, washed with ethyl acetate and the filtrate was concentrated *in vacuo*. The crude product was purified by column chromatography (SiO_2, 19:1 diethyl ether:triethylamine) to yield the title compound **S162** (222 mg, 75%) as a colourless oil. R_f 0.33 (19:1 diethyl ether:triethylamine); δ_H (400 MHz, $CDCl_3$) 7.76 (1H, d, J = 7.5 Hz, ArH), 7.70 (1H, d, J = 8.0 Hz, ArH), 7.46 (1H, t, J = 7.5 Hz, ArH), 7.31–7.17 (6H, m, ArH), 3.95–3.81 (5H, m, $ArCH_2$, CH_3), 3.64–3.61 (4H, m, $ArCH_2$, C**HH'**), 2.96–2.86 (1H, m, CHH'), 2.75–2.59 (2H, m, CHH'), 2.53–2.45 (1H, m, NCH), 2.42–2.31 (2H, m, CHH'), 2.16–2.08 (1H, m, CHH'), 1.73–1.57 (2H, m, CHH'), 1.48–1.13 (6H, m, CHH'); δ_C (101 MHz, $CDCl_3$) 168.3 (CO), 140.8 (ArC), 138.9 (ArC), 131.4 (ArC), 130.8 (ArC), 130.4 (ArC), 129.9 (ArC), 129.3 (ArC), 128.1 (ArC), 127.0 (ArC), 126.7 (ArC), 64.1 (CH_2), 59.8 ($ArCH_2$), 58.5 (NCH), 57.2 ($ArCH_2$), 55.1 (CH_2), 54.1 (CH_2), 51.9 (CH_3), 50.4 (CH_2), 30.3 (CH_2), 27.3 (CH_2), 24.9 (CH_2), 21.9 (CH_2); ν_{max}/cm^{-1} (thin film) 3397, 2928, 2852, 1721, 1450, 1433, 1363, 1266, 1128, 1079, 1046, 967, 913, 740, 700; HRMS (ESI); calcd. for $C_{25}H_{35}N_2O_3^+$, 411.2642. Found: $[MH]^+$, 411.2642 (0.1 error ppm).

2-((Benzyl((1-(3-hydroxypropyl)piperidin-2-yl)methyl)amino)methyl)benzoic acid (S163)

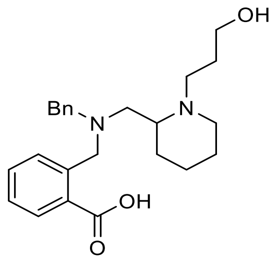

To a solution of methyl 2-((benzyl((1-(3-hydroxypropyl)piperidin-2-yl)methyl)amino)methyl)benzoate **S162** (220 mg, 0.536 mmol) in THF (1.1 mL) was added lithium hydroxide (1.72 mL, 0.5 M in water, 0.860 mmol) and the mixture was refluxed for 22 h. The reaction mixture was concentrated *in vacuo* and purified by column chromatography (SiO_2, 15:4:1 ethyl acetate:methanol:triethylamine) to yield the title compound **S163** (140 mg, 65%) as a white solid. R_f 0.28 (15:4:1 ethyl acetate:methanol:triethylamine); m.p. 51–55 °C; δ_H (400 MHz, $CDCl_3$) 7.68 (1H, d, *J* = 7.5 Hz, ArH), 7.23–7.05 (8H, m, ArH), 4.82 (1H, d, *J* = 12.0 Hz, CHH'), 3.76–3.46 (5H, m, NCH, CHH'), 2.96 (1H, t, *J* = 12.0 Hz, CHH'), 2.86 (1H, d, *J* = 11.0 Hz, CHH'), 2.73 (1H, d, *J* = 12.5 Hz, CHH'), 2.63 (1H, t, *J* = 13.0 Hz), 2.50–2.36 (2H, m, CHH'), 1.96 (1H, d, *J* = 14.0 Hz, CHH'), 1.81–1.59 (2H, m, CHH'), 1.52–1.20 (6H, m, CHH'); δ_C (101 MHz, $CDCl_3$) 176.7 (CO), 139.9 (ArC), 137.3 (ArC), 135.3 (ArC), 130.5 (ArC), 130.3 (ArC), 129.0 (ArC), 128.1 (ArC), 127.9 (ArC), 127.6 (ArC), 127.3 (ArC), 58.9 (2 x CH_2), 58.3 (NCH), 57.9 (CH_2), 54.0 (CH_2), 47.7 (CH_2), 40.3 (CH_2), 26.9 (CH_2), 22.4 (CH_2), 21.8 (CH_2), 17.9 (CH_2); ν_{max}/cm^{-1} (thin film) 3247, 2941, 1584, 1562, 1440, 1371, 1065, 922, 753, 726, 703; HRMS (ESI); calcd. for $C_{24}H_{33}N_2O_3^+$, 397.2486. Found $[MH]^+$, 397.2484 (0.3 error ppm).

16-Benzyl-1,2,3,4,7,8,15,16,17,17a-decahydro-6H,10H-benzo[j]pyrido[1,2-e][1]oxa[5,8]diazacyclododecin-10-one (122)

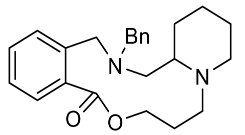

A round-bottom flask was charged with 2-((benzyl((1-(3-hydroxypropyl)piperidin-2-yl)methyl)amino)methyl)benzoic acid **S163** (66 mg, 0.166 mmol) and DMF (1.7 mL). To this was added DIPEA (0.160 mL, 0.921 mmol), EDC·HCl (52 mg, 0.271 mmol) and HOBt (36 mg, 0.266 mmol). The resulting mixture was stirred at room temperature for 18 h. The reaction mixture was diluted with ethyl acetate (20 mL) and then washed with brine (3 x 20 mL). The

combined aqueous layers were then extracted with ethyl acetate (2 x 20 mL). The combined organic layers were then dried over magnesium sulfate, filtered and concentrated *in vacuo*. The crude product was purified by column chromatography (SiO_2, 15:4:1 ethyl acetate:hexane:triethylamine) to yield the title compound **122** (38 mg, 59%) as a colourless oil. R_f 0.50 (15:4:1 ethyl acetate:hexane:triethylamine); δ_H (400 MHz, $CDCl_3$) 7.62 (1H, dd, *J* = 7.5, 1.5 Hz, ArH), 7.40–7.30 (2H, m, ArH), 7.26–7.15 (6H, m, ArH), 4.89 (1H, dt, *J* = 11.0, 3.5 Hz, OC**H**H'), 4.23 (1H, d, *J* = 12.5 Hz, ArC**H**H'), 3.93 (1H, t, *J* = 11.0 Hz, OCH**H'**), 3.58 (1H, d, *J* = 12.5 Hz, ArCH**H'**), 3.44 (1H, d, *J* = 12.5 Hz, PhC**H**H'), 3.19 (1H, ddd, *J* = 14.0, 11.0, 3.0 Hz, NC**H**H'C_2H_4O), 3.00 (1H, d, *J* = 12.5 Hz, PhCH**H'**), 2.94–2.84 (1H, m, NCH**H'**C_2H_4O), 2.73 (1H, dd, *J* = 14.5, 7.0 Hz, NC**H**H'CH), 2.68–2.62 (1H, m, NC**H**H'), 2.29–2.15 (3H, m, NCH**H'**CH, NCH**H'**, CHH'), 2.05–1.97 (1H, m, NCH), 1.72–1.61 (1H, m, CHH'), 1.54–1.44 (2H, m, CHH'), 1.40–1.22 (2H, m, CHH'), 1.02–0.81 (2H, m, CHH'); δ_C (101 MHz, $CDCl_3$) 170.4 (CO), 138.2 (ArC), 137.3 (ArC), 133.6 (ArC), 131.2 (ArC), 130.6 (ArC), 130.2 (ArC), 129.9 (ArC), 128.0 (ArC), 127.7 (ArC), 127.1 (ArC), 64.0 (N**C**H_2CH), 63.5 (OCH_2), 61.9 ($ArCH_2$), 59.7 ($PhCH_2$), 56.9 (NCH), 52.0 (NCH_2), 50.2 (N**C**$H_2CH_2CH_2O$), 31.8 (CH_2), 25.7 (CH_2), 24.3 (CH_2), 22.4 (CH_2); ν_{max}/cm^{-1} (thin film) 2929, 2792, 1715, 1451, 1380, 1288, 1124, 1090, 1047, 907, 729, 700; HRMS (ESI); calcd. for $C_{24}H_{31}N_2O_2^+$, 379.2380. Found $[MH]^+$, 379.2375 (1.3 error ppm).

Methyl 2-(((3-bromopropyl)selanyl)methyl)benzoate (S164)

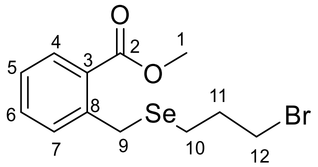

PPh_3 (3.29 g, 12.6 mmol) was added portionwise over a period of 5 min to a solution of methyl 2-(((3-hydroxypropyl)selanyl)methyl)benzoate **S45** (3.01 g, 10.5 mmol) and CBr_4 (4.18 g, 12.6 mmol) in anhydrous CH_2Cl_2 (26 mL) at 0 °C. A colour change to yellow was immediately noted. The mixture stirred for 1 h at 0 °C and then allowed to warm to RT and stirred for additional 16 h, whilst a colour change to brown-orange was observed. The reaction mixture was concentrated under reduced pressure, but not to dryness, to yield a brown/orange oil, which was diluted with EtOAc (30 mL). The resulting solid (Ph_3PO) was filtered, and the filter cake was washed with EtOAc (3 × 30 mL). The resulting filtrated was poured into separating funnel where organic layer was washed sequentially with sat. $NaHCO_3$ (3 × 20 mL) and brine (3 × 30

mL). The organic layer was dried over $MgSO_4$, filtered and concentrated under reduced pressure to yield an orange oil (8.89 g). The crude product was purified by flash column chromatography (SiO_2, 70 mm column, eluent: Et_2O:n-hexane, 10:90) to afford the title compound **S164** as a pale yellow oil (3.21 g 87%); R_f = 0.63 (40:60 EtOAc:n-hexane); IR (neat) ν_{max} / cm^{-1}: 2948w (C-H alkyl), 1714s (C=O aryl ester), 1599w (CC aromatic), 1575w (CC aromatic), 1488m (CC aromatic), 1433w, 1291m, 1260s, 1189m, 1112m, 1074m, 1045m, 965w, 839w, 751m, 707m, 664w, 614w, 558w; δ_H (400 MHz; $CDCl_3$) 7.93 (1H, dd, *J* 7.5, 1.3, C(4)H), 7.42 (1H, td, *J* 7.5, 1.3, C(6)H), 7.32 – 7.256 (2H, m, C(5+7)H), 4.19 (2H, s, C(9)H_2), 3.91 (3H, s, C(1)H_3), 3.45 (2H, t, *J* 6.5, C(12)H_2), 2.65 (2H, t, *J* 7.1, C(10)H_2), 2.16 – 2.07 (2H, m, C(11)H_2); δ_C (126 MHz; $CDCl_3$) 167.4 (C, C2), 142.9 (C, C8), 131.9 (CH, C7), 131.2 (CH, C4), 130.8 (C, C3), 128.6 (CH, C6), 126.8 (CH, C5), 52.0 (CH_3, C1), 33.3 (CH_2, C12), 33.0 (CH_2, C11), 25.7 (CH_2, C9), 22.1 (CH_2, C10); HRMS (ESI$^+$): m/z calc. for $C_{12}H_{16}{}^{79}BrO_2{}^{80}Se$: 350.9493, found: 350.9478 $[MH]^+$, m/z calc. for $C_{12}H_{15}{}^{79}BrNaO_2{}^{80}Se$: 372.9313, found : 372.9290 $[MNa]^+$.

Methyl 2-(((3-(benzyl(3-hydroxypropyl)amino)propyl)selanyl)methyl)benzoate (S165)

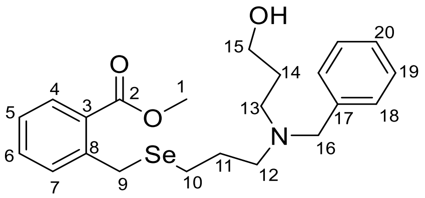

K_2CO_3 (393 mg, 2.84 mmol) was added to a pale-yellow solution of 3-(benzylamino)propan-1-ol (1.32 mL, 8.33 mmol) and methyl 2-(((3-bromopropyl)selanyl)methyl)benzoate **S164** (496 mg, 1.42 mmol) in anhydrous MeCN (29.0 mL) at RT under Ar. The resulting milky white suspension was stirred at 95 °C for 18 h under Ar, after which the reaction was deemed to have gone to completion by TLC, with a colour change to pale yellow suspension noted. The reaction mixture was then quenched by addition of H_2O (30 mL) and resulting solution poured into a separating funnel. The aqueous layer was extracted with EtOAc (3 × 30 mL), before the combined organic phases were washed with sat. brine (3 × 20 mL). The resulting organic layer was dried over $MgSO_4$, filtered and concentrated under reduced pressure to yield a pale-yellow liquid (622 mg). The crude product was purified by flash column chromatography (SiO_2, 40 mm column, eluent: EtOAc:n-hexane, 50:50 to 60:50) to afford the title compound **S165** as a pale yellow oil (266 mg 35%); R_f = 0.19 (50:50 EtOAc:n-hexane); IR (neat) ν_{max} / cm^{-1}:

3394brw (O–H alcohol), 2947w (C–H alkyl), 2809w (C–H alkyl),1716s (C=O aryl ester), 1599w (CC aromatic), 1578w (CC aromatic), 1434m (CC aromatic), 1261s, 1189w, 1113s, 1073s, 756m, 699s, 665w, 616w; δ_H (500 MHz; $CDCl_3$) 7.91 (1H, dd, *J* = 7.7, 1.5 Hz, C(4)H), 7.39 (1H, td, *J* = 7.7, 1.5 Hz, C(6)H), 7.34 – 7.29 (2H, m, C(7+20)H), 7.29 – 7.25 (4H, m, C(18+19)H), 7.25 – 7.21 (1H, m, C(5)H), 4.15 (2H, s, C(9)H_2), 3.89 (3H, s, C(1)H_3) 3.70 (2H, t, *J* = 5.7, 5.3 Hz, C(15)H_2), 3.53 (2H, s, C(16)H_2), 2.61 (2H, t, *J* = 5.8 Hz, C(13)H_2), 2.52 – 2.43 (4H, m, C(10+12)H_2), 1.85 – 1.75 (2H, m, C(11)H_2), 1.74 – 1.66 (2H, m, C(14)H_2); δ_C (126 MHz; $CDCl_3$) 167.8 (C, C2), 142.4 (C, C8), 138.4 (C, C17), 132.0 (CH, C6), 131.5 (CH, C4), 131.0 (CH, C7), 129.3 (2 × CH, C(18)H), 129.0 (C, C3), 128.6 (2 × CH, C(19)H), 127.4 (CH, C20), 126.9 (CH, C5), 64.3(CH_2, C15), 59.0 (CH_2, C16), 54.2 (CH_2, C13), 53.9 (CH_2, C12), 52.2 (CH_2, C1), 28.25 (CH_2, C14), 27.5 (CH_2, C11), 26.1 (CH_2, C8), 22.0 (CH_2, C10); HRMS (ESI): calcd. for $C_{22}H_{30}NO_3{}^{80}Se$, 436.1385. Found: $[MH]^+$, 436.1372 (3.3 ppm error).

6-Benzyl-4,5,6,7,8,9-hexahydro-3H-benzo[k][1]oxa[9]selena[5]azacyclotridecin-1(11H)-one (123)

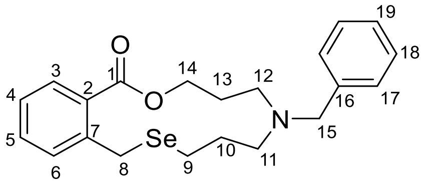

$NaOH_{(aq)}$ (4.0 M, 5.0 mL) was added dropwise over a period of 1 min via syringe to a pale yellow solution of methyl 2-(((3-(benzyl(3-hydroxypropyl)amino)propyl)selanyl)methyl)benzoate **S165** (216 mg, 0.497 mmol) in MeOH (5.0 mL) at RT. A colour change to milky/wite suspension was immediately noted and the reaction mixture was stirred at 60 °C for 18 h, at which point the reaction mixture was deemed to have gone to completion by TLC (complete consumption of **S165** was noted). The colourless reaction mixture was allowed to cool to RT and then acidified to pH 2.0 with 1 M $HCl_{(aq)}$ (10 mL). The acidified solution was concentrated under reduced pressure to dryness and the resulting white solid was azeotroped with toluene (3 × 20 mL, to remove H_2O) to afford a crude product which dissolved in anhydrous DMF (10.0 mL) and used directly in the next reaction step without further purification. EDC.HCl (173 mg, 0.902 mmol) was added to this crude product mixture (a pale-yellow suspension), followed by HOBt (101 mg, 0.750 mmol) and dry DIPEA (871 µL, 5.10 mmol). A colour change to a cloudy yellow was immediately noted

and the reaction mixture was stirred at RT for 18 h of stirring at RT under Ar. The resulting mixture was then diluted with EtOAc (30 mL) and transferred into a separating funnel and the organic phase was washed with sat. brine (4 × 30 mL). The organic layer was dried over $MgSO_4$, filtered and concentrated under reduced pressure to yield a colourless oil (215 mg). The crude product was purified by flash column chromatography (SiO_2, 30 mm column, eluent: EtOAc:*n*–hexane, 20:80) to afford macrocycle **122** as a colourless oil (85.7 mg, 47%, over two steps); R_f = 0.71 (EtOAc:n-hexane, 50:50); IR (thin film) ν_{max} / cm^{-1}: 3061w (C–H alkyl), 3026w (C–H alkyl), 2942w (C–H alkyl), 2800w (C–H alkyl), 1713s (C=O aryl ester), 1600w (CC aromatic), 1574w (CC aromatic), 1493w (CC aromatic), 1449m, 1369w, 1351w, 1291w, 1262s, 1186w, 1145w, 1114m, 1084w, 1069m, 1042w, 1028w, 940w, 923w, 862w, 831w, 803w, 758m, 735m, 713m, 698s, 663w, 611w, 575w, 500w, 466w; δ_H (500 MHz; $CDCl_3$) 7.74 (1H, dd, *J* = 7.7, 1.5 Hz, C(3)H), 7.42 (1H, td, J = 7.7, 1.5 Hz, C(5)H), 7.34 (1H, dd, *J* = 7.7, 1.54 Hz, C(6)H), 7.32 – 7.28 (4H, m, C(17+18)H), 7.27 – 7.20 (2H, m, C(4+19)H), 4.59 – 4.53 (2H, m, C(14)H_2), 4.28 (2H, s, C(8)H_2), 3.48 (2H, s, C(15)H_2), 2.64 – 2.55 (4H, m, C(9+12)H_2), 2.36 – 2.31 (2H, m, C(11)H_2), 2.01 – 1.92 (2H, m, C(10)H_2), 1.62 – 1.53 (2H, m, C(13)H_2); δ_C (126 MHz; $CDCl_3$) 169.0 (C, C1), 142.7 (C, C7), 139.7 (C, C16), 132.0 (CH, C5),131.4(CH, C3), 130.4 (C, C2), 129.9 (CH, C6), 129.0 (2 × CH, C17), 128.4 (2 × CH, C18), 127.1 (CH, C19), 126.4 (CH, C4), 62.6 (CH_2, C14), 59.2 (CH_2, C15), 54.4 (CH_2, C12), 49.6 (CH_2, C11), 30.3 (CH_2, C13), 26.1 (CH_2, C10), 24.4 (CH_2, C8), 24.0 (CH_2, C9); HRMS (ESI): calcd. for $C_{21}H_{26}NO_2{}^{80}Se$, 404.1123. Found: $[MH]^+$, 404.1121 (0.9 ppm error).

Methyl 2-(((3-((3-hydroxypropyl)thio)propyl)selanyl)methyl)benzoate (S166)

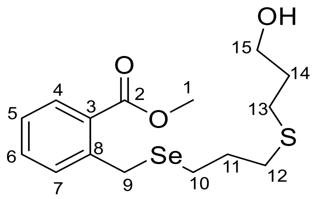

K_2CO_3 (583 mg, 4.22 mmol) was added to a pale-yellow solution of 3-mercapto-1-propanol (221 µL, 3.17 mmol) and methyl 2-(((3-bromopropyl)selanyl)methyl)benzoate **S164** (739 mg, 2.11 mmol) in anhydrous DMF (21 mL) at RT. The resulting milky/white suspension was then heated to 70 °C. After 18 h, the reaction was deemed to have gone to completion by TLC, with a colour change to yellow suspension noted. The reaction mixture allowed to cool to RT,

before was quenched with H_2O (30 mL). The diluted milky/white suspension was poured into separating funnel and the aqueous layer was extracted with EtOAc (3 × 20 mL). The combined organic layers were washed sequentially with 1 M $HCl_{(aq)}$ (3 × 30 mL), sat. $NaHCO_3$ (3 × 30 mL) and sat. brine (4 × 30 mL). The resulting layer was collected and dried over $MgSO_4$, filtered and concentrated under reduced pressure to yield a pale yellow oil (1.15 g). The crude product was purified by flash column chromatography (SiO_2, 50 mm column, eluent: EtOAc:*n*-hexane, 50:50) to afford the title compound **S166** as a pale yellow liquid (719 mg, 94%); R_f = 0.43 (EtOAc:n-hexane, 50:50); IR (neat) ν_{max} / cm^{-1}: 3428wbr (O–H alcohol), 3009w (C–H aryl), 2948w (C–H alkyl), 1715vs (C=O aryl ester), 1601w (CC aromatic), 1578 (CC aromatic), 1493w, 1435m, 1262m, 1113m, 1074m, 750s, 708m, 665w; δ_H (500 MHz; $CDCl_3$) 7.92 (1H, dd, *J* 7.6, 1.6, C(4)H), 7.42 (1H, td, *J* 7.6, 1.6, C(6)H), 7.33 – 7.25 (2H, m, C(5+7)H), 4.18 (2H, s, C(9)H_2), 3.91 (3H, s, C(1)H_3), 3.74 (2H, app. q, *J* 5.9, C(15)H_2), 2.67 – 2.53 (6H, m, C(10+12+13)H_2), 1.93 – 1.77 (4H, m, C(11+12)H_2), 1.64 (1H, s, br, OH); δ_C (126 MHz; $CDCl_3$) 167.8 (C, C2), 142.2 (C, C8), 132.0 (CH, C7), 131.4 (CH, C4), 130.9 (CH, C6), 128.8 (C, C3), 126.8 (CH, C5), 61.7 (CH_2, C15), 52.2 (CH_3, C1), 32.1 (CH_2, C14), 31.9 (CH_2, C11), 30.1 (CH_2, C12), 28.8 (CH_2, C13), 25.9 (CH_2, C9), 23.2 (CH_2, C10); HRMS (ESI^+): m/z calc. for $C_{15}H_{23}O_3S^{80}Se$: 363.0528, found: 363.0526 $[MH]^+$, m/z calc. for $C_{15}H_{22}NaO_3S^{80}Se$: 385.0347, found : 385.0345 $[MNa]^+$.

2-(((3-((3-Hydroxypropyl)thio)propyl)selanyl)methyl)benzoic acid (S167)

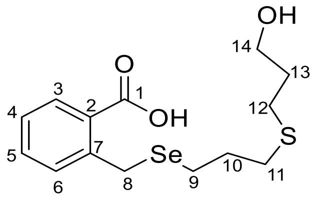

$NaOH_{(aq)}$ (4.0 M, 12.5 mL) was added dropwise over a period of 1 min to a clear colourless solution of methyl 2-(((3-((3-hydroxypropyl)thio)propyl)selanyl)methyl)benzoate **S166** (446 mg, 1.23 mmol) in MeOH (12.5 mL) at RT. A colour change to milky/wite suspension was immediately noted and the reaction mixture was stirred at 60 °C for 16 h. The transparent colourless reaction mixture was allowed to cool to RT and then acidified to pH 1.0 with 1 M $HCl_{(aq)}$ (30 mL). The resulting acidified solution was poured into separating funnel and aqueous layer was extracted with CH_2Cl_2 (3 × 20 mL). The combined organic phases were dried over $MgSO_4$, filtered and concentrated under reduced pressure to afford carboxylic acid **S167** as a colourless oil (418 mg, 98%); R_f = 0.28 (15:85 MeOH:CH_2Cl_2); IR (neat) ν_{max} / cm^{-1}: 3353vbrm

(O–H alcohol/ carboxylic acid), 3068vbrw (C–H aryl, O–H alcohol/ carboxylic acid), 2930vbrm (C–H alkyl, O–H carboxylic acid), 2627w (C–H alkyl, O–H alcohol/ carboxylic acid), 2534w (O–H carboxylic acid) 1686vs (C=O aryl carboxylic acid), 1599w (CC aromatic), 1574w (CC aromatic), 1489w (CC aromatic), 1415w, 1250s, 1044m, 953s, 757s, 707vs, 649m; δ_H (500 MHz; CD_2Cl_2) 8.07 (1H, dd, *J* = 8.3, 1.5 Hz, C(3)H), 7.51 (1H, td, *J* = 7.6, 1.5 Hz, C(5)H), 7.39 – 7.33 (2H, m, C(4+6)H), 7.18 (2H, br, s, OH), 4.28 (2H, s, C(8)H_2), 3.80 (2H, t, *J* = 6.1 Hz, C(14)H_2), 2.71 – 2.57 (6H, m, C(9+11+12)H_2), 1.99 – 1.83 (4H, m, C(10+13)H_2); δ_C (126 MHz; CD_2Cl_2) 171.7 (C, C1), 143.4 (C, C7), 133.0 (CH, C6), 132.2 (CH, C5), 131.5 (CH, C3), 128.4 (C, C2), 127.2 (CH, C4),61.9 (CH_2, C14), 32.4 (CH_2, C11), 32.1 (CH_2, C12), 30.7 (CH_2, C10), 28.8 (CH_2, C13), 25.9 (CH_2, C8), 23.5 (CH_2, C9); HRMS (ESI): calcd. for $C_{14}H_{21}O_3S^{80}Se$, 349.0371. Found: $[MH]^+$, 349.0370 (0.4 ppm error), calcd. for $C_{14}H_{20}NaO_3S^{80}Se$, 371.0191. Found: $[MNa]^+$, 371.0190 (0.0 ppm error).

4,5,8,9-Tetrahydro-3H,7H-benzo[k][1]oxa[5]thia[9]selenacyclotridecin-1(11H)-one (124)

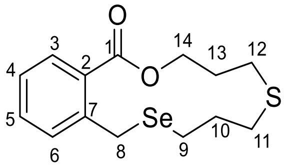

EDC.HCl (342 mg, 1.79 mmol) was added to a colouless solution of 2-(((3-((3-hydroxypropyl)thio)propyl)selanyl)methyl)benzoic acid **S167** (414 mg, 1.19 mmol), HOBt (193 mg, 1.43 mmol) and dry DIPEA (520 µL, 2.98 mmol) in anhydrous DMF (23.8 mL) at RT under Ar, with a colour change to pale yellow noted. The resulting mixture was stirred at RT for 24 h under Ar, after which time the reaction was deemed to have gone completion by TLC. The reaction mixture was diluted with EtOAc (40 mL) and transferred into separating funnel. The resulting yellow organic layer was washed sequentially with 1 M $HCl_{(aq)}$ (2 × 20 mL), sat. $NaHCO_{3(aq)}$ (3 × 20 mL) and brine (3 × 20 mL), before being dried over $MgSO_4$, filtered and concentrated under reduced pressure to yield a pale yellow oil (240 mg). The crude product was purified by flash column chromatography (SiO_2, 30 mm column, eluent: 60 EtOAc:*n*–hexane, 20:80) to afford the title compound **124** as a colourless oil (230 mg, 59%); R_f = 0.64 (50:50 EtOAc:*n*–hexane); IR (thin film) ν_{max} / cm^{-1}: 2953w (C–H alkyl), 2861w (C–H alkyl), 1713s (C=O aryl lactam), 1604w (CC aromatic), 1449w, 1378w, 1293w, 1261s, 1115m, 1074w, 767w; δ_H (500 MHz; C_6D_6) 7.81 (1H, dd, *J* = 7.7, 1.4 Hz, C(3)H), 7.18 (1H, dd, *J* = 7.7, 1.4 Hz, C(6)H), 7.02 (1H, td, *J* = 7.7, 1.4 Hz, C(5)H), 6.88 (1H, td, J = 7.7, 1.4 Hz, C(4)H), 4.22 – 4.16

(2H, m, C(14)H_2), 4.13 (2H, s, C(8)H_2) 2.41 (2H, t, *J* = 7.2 Hz, C(12)H_2), 2.31 (2H, t, *J* = 6.9 Hz, C(9)H_2), 2.29 – 2.25 (2H, m, C(11)H_2), 1.73 – 1.60 (4H, m, C(10+13)H_2); δ_C (126 MHz; C_6D_6) 168.1 (C, C1), 140.8 (C, C7), 132.0 (CH, C6), 131.9 (CH, C5), 131.2 (C, C2) , 130.8 (CH, C3), 126.7 (CH,C4), 63.6 (CH, C14), 31.1 (CH_2, C11), 30.6 (CH_2, C10), 29.7 (CH_2, C13), 28.5 (CH_2, C12), 24.4 (CH_2, C8), 24.1 (CH_2, C9); HRMS (ESI): calcd. for $C_{14}H_{18}NaO_2S^{80}Se$, 353.0085. Found: $[MNa]^+$, 353.0083 (0.6 ppm error).

Methyl 2-((ethyl(3-(ethyl(3-hydroxypropyl) amino) propyl) amino) methyl) benzoate (S168)

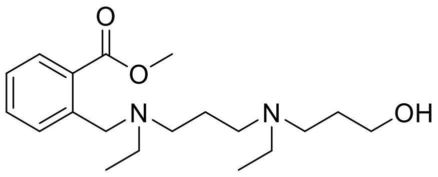

To a stirring solution of potassium carbonate (0.415 g, 3.00 mmol) in acetonitrile (5.00 mL), 3-(ethyl(3-(ethylamino) propyl) amino) propan-1-ol **S131** (0.188 g, 1.00 mmol) was added followed by methyl 2-(bromomethyl) benzoate (0.230 g, 1.00 mmol). The reaction mixture was refluxed at 90 °C for 3 hours under argon before filtering through Celite washing with DCM, concentrating under vacuum and purifying by flash column chromatography (hexane:methanol:triethylamine 17:2:1) to afford the title compound as a clear oil (0.238 g, 71%) R_f = 0.20 (hexane:methanol:triethylamine 17:2:1) ν_{max}/cm^{-1} (thin film) 2950, 2814, 2238, 1721, 1449, 1372, 1268, 1128, 1081, 910, 729; δ_H (400 MHz, $CDCl_3$) 7.56 (1H, d, *J* = 7.6 Hz, Ar**H**), 7.41 (1H, d, *J* = 7.6 Hz, Ar**H**), 7.27 (1H, t, *J* = 7.6 Hz, Ar**H**), 7.11 (1H, t, *J* = 7.6 Hz, Ar**H**), 5.09 (1H, bs, O**H**), 3.72 (3H, s, OC**H**$_3$), 3.70 (2H, s, ArC**H**$_2$N), 3.59 (2H, t, *J* = 5.2 Hz, C**H**$_2$OH), 2.44 (2H, t, *J* = 5.8 Hz, C**H**$_2$N), 2.39−2.31 (4H, m, 2× NC**H**$_2$CH$_3$), 2.29−2.22 (4H, m, C**H**$_2$N), 1.49 (2H, quintet, *J* = 5.8 Hz, CH$_2$C**H**$_2$CH$_2$), 1.46−1.40 (2H, m, CH$_2$C**H**$_2$CH$_2$), 0.88−0.83 (4H, m, C**H**$_3$CH$_2$N); δ_c (101 MHz, $CDCl_3$) 168.8 (**C**O), 141.5 (Ar**C**), 131.1 (Ar**C**H), 131.0 (Ar**C**), 129.8 (Ar**C**H), 129.6 (Ar**C**H), 126.5 (Ar**C**H), 64.0 (**C**H$_2$OH), 56.6 (Ar**C**H$_2$), 53.9 (**C**H$_2$N), 51.8 (**C**H$_3$O), 51.7 (**C**H$_2$N), 51.2(**C**H$_2$N), 47.5 (CH$_3$**C**H$_2$N), 47.3 (CH$_3$**C**H$_2$N), 27.9 (CH$_2$**C**H$_2$CH$_2$), 24.2 (CH$_2$**C**H$_2$CH$_2$), 11.5 (2 × **C**H$_3$CH$_2$N); HRMS (ESI) calcd. for $C_{19}H_{33}N_2O_3$ 337.2491. Found $[MH]^+$ 337.2478 (−3.87 ppm error).

6,10-Diethyl-4,5,6,7,8,9,10,11-octahydrobenzo[k][1] oxa [5,9] diazacyclotridecin-1(3H)-one (125)

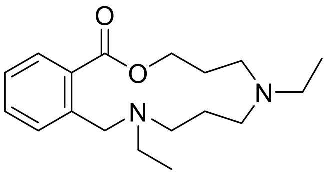

To a stirring solution of methyl 2-((ethyl(3-(ethyl(3-hydroxypropyl) amino) propyl) amino) methyl) benzoate **S168** (0.112 g, 0.332 mmol) in tetrahydrofuran (0.8 mL), aqueous lithium hydroxide (0.5 M) (0.800 mL, 0.400 mmol) was added and heated for at 50 °C for 3 hours. The solvent was removed under vacuum using dichloromethane (5 × 50.0 mL) to form an azeotropic mixture, to help ensure that all of the water from the hydrolysis was removed ahead of the next step. The intermediate lithium 2-((ethyl (3-(ethyl (3-oxidopropyl) amino) propyl) amino) methyl) benzoate was dissolved in acetonitrile (3.3 mL) and DIPEA (0.310 mL, 1.80 mmol) was added followed by HOBt (0.0680 g, 0.500 mmol) and EDCI (0.0780 g, 0.500 mmol) and left to stir at room temperature for 18 hours under argon. The reaction mixture was then transferred to a separating funnel, brine (50.0 mL) was added and extracted with diethyl ether (3 × 50 mL). The combined organic phases were then dried with sodium sulphate, filtered, concentrated under vacuum and purified via flash column chromatography (hexane:dichloromethane:triethylamine 10:9:1) to afford the title compound **125** (46.4 mg, 46%) R_f = 0.54 (hexane:dichloromethane:triethylamine 10:9:1); ν_{max}/cm^{-1} (thin film) 2965, 2801, 1723, 1457, 1375, 1271, 1127, 1085, 787, 743; δ_H (400 MHz, $CDCl_3$) 7.57−7.55 (1H, m, Ar**H**), 7.36 (1H, td, *J* = 7.6 Hz, 1.5 Hz, Ar**H**), 7.29−7.25 (2H, m, 2 × Ar**H**), 4.42 (2H, t, *J* = 5.5 Hz, OC**H**$_2$CH$_2$), 3.81 (2H, s, ArC**H**$_2$N), 2.61 (2H, t, *J* = 6.5 Hz, CH$_2$C**H**$_2$N), 2.47−2.39 (8H, m, C**H**$_2$N), 1.87 (2H, quintet, *J* = 6.4 Hz, CH$_2$C**H**$_2$CH$_2$), 1.49 (2H, quintet, *J* = 6.4 Hz, CH$_2$C**H**$_2$CH$_2$), 0.98 (3H, t, *J* = 7.0 Hz, C**H**$_3$CH$_2$O), 0.93 (3H, t, *J* = 7.3 Hz, C**H**$_3$CH$_2$O); δ_C (101 MHz, $CDCl_3$) 169.9 (**C**O), 139.0 (Ar**C**), 133.2 (Ar**C**), 131.4 (Ar**C**H), 130.3 (Ar**C**H), 129.1 (Ar**C**H), 127.0 (Ar**C**H), 63.4 (O**C**H$_2$CH$_2$)), 55.4 (Ar**C**H$_2$N), 51.2 (CH$_2$**C**H$_2$N), 51.0 (**C**H$_2$N), 49.2 (**C**H$_2$N), 45.6(**C**H$_2$N), 27.0 (CH$_2$**C**H$_2$CH$_2$), 23.6 (CH$_2$**C**H$_2$CH$_2$), 12.4 (**C**H$_3$CH$_2$N), 9.8 (**C**H$_3$CH$_2$N); HRMS (ESI) calcd. for $C_{18}H_{29}N_2O_2$, 305.2229. Found $[MH]^+$ 305.2223 (−1.97 ppm error).

Methyl 2-(((3-((3-mercaptopropyl)thio)propyl)thio)methyl)benzoate (S169)

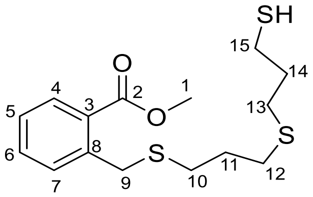

K_2CO_3 (415 mg, 3.01 mmol) was added to a colourless solution of 1,3-propanedithiol (230 µL, 2.25 mmol) and methyl 2-(((3-bromopropyl)thio)methyl)benzoate **S98** (455 mg, 1.50 mmol) in anhydrous DMF (15.0 mL) at RT. The resulting milky–pink suspension was stirred at RT for 18 h, with a colour change to milky white noted. The reaction mixture was then quenched by the addition of H_2O (30 mL) and resulting solution poured into a separating funnel. The aqueous layer was extracted with EtOAc (4 × 30 mL), before the combined organic phases were washed sat. brine (3 × 20 mL). The resulting organic layer was dried over $MgSO_4$, filtered and concentrated under reduced pressure to yield a pale yellow oil (1.07 g). The crude product was purified by flash column chromatography (SiO_2, 40 mm column, eluent: EtOAc:*n*–hexane, 10:90) to afford thiol **S169** as colouless oil (467 mg, 94%); R_f = 0.58 (50:50 EtOAc:*n*–hexane); IR (neat) ν_{max} / cm^{-1}: 2935w (C–H alkyl), 2557brw (S–H thiol), 1717vs (C=O aryl ester), 1600w (CC aromatic), 1488w (CC aromatic), 1434m, 1293w, 1263s, 1121m, 1077m, 1046, 967w, 749s, 716s, 666m, 580w; δ_H (400 MHz; $CDCl_3$) 7.91 (1H, dd, *J* = 7.7, 1.4 Hz, C(4)H), 7.44 (1H, td, *J* = 7.7, 1.4 Hz, C(6)H), 7.37 – 7.28 (2H, m, C(5+7)H), 4.11 (2H, s, C(9)H_2), 3.91 (3H, s, C(1)H_3), 2.66 – 2.50 (8H, m, C(10+12+13+15)H_2), 1.90 – 1.76 (4H, m, C(11+14)H_2), 1.36 (1H, t, *J* = 8.0 Hz, SH); δ_C (101 MHz; $CDCl_3$) 167.9 (C, C2), 140.7 (C, C8), 131.9 (CH, C7), 131.3 (CH, C4), 131.0 (CH, C6), 129.6 (C, C3), 127.2 (CH, C5), 52.3 (CH_3, C1), 34.7 (CH_2, C9), 33.4 (CH_2, C14), 30.9 (CH_2, C12), 30.7 (CH_2, C10), 30.4 (CH_2, C13), 29.2 (CH_2, C11), 23.5 (CH_2, C15); HRMS (ESI): calcd. for $C_{15}H_{23}O_2S_3$, 331.0855. Found: $[MH]^+$, 331.0852 (0.8 ppm error), calcd. for $C_{15}H_{22}NaO_2S_3$, 353.0674. Found: $[MNa]^+$, 353.0673 (0.5 ppm error).

2-(((3-((3-Mercaptopropyl)thio)propyl)thio)methyl)benzoic acid (S170)

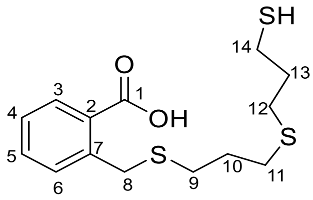

$NaOH_{(aq)}$ (4.0 M, 7.05 mL, degassed for 10 min) was added dropwise over a period of 10 min to a colourless solution of methyl 2-(((3-((3-mercaptopropyl)thio)propyl)thio)methyl)benzoate **S169** (466 mg, 1.41 mmol) in MeOH (7.05 mL, degassed for 15 min) at RT under Ar. A colour change to milky white suspension was noted immediately following the dropwise addition. The resulting suspension was then heated to 60 °C under Ar, whereupon a colour change to a colourless solution was observed. After a total of 18 h, the reaction was deemed to have gone to completion by TLC analysis (complete consumption of ester **S169** was noted). The colourless reaction mixture was allowed to cool to RT and then acidified to pH 2.0 with 1 M $HCl_{(aq)}$ (30 mL). The resulting acidified solution was poured into separating funnel. The aqueous layer was extracted with CH_2Cl_2 (4 × 20 mL), before combined organic phases were dried over Na_2SO_4, filtered and concentrated under reduced pressure to afford a carboxylic acid **S170** as a colourless oil (309 mg, 69%); R_f = 0.58 (EtOAc); IR (neat) ν_{max} / cm^{-1}: 3079w (C–H alkyl), 2917w (C–H alkyl/ O–H carboxylic acid), 2651brw (C–H alkyl/ O–H carboxylic acid), 2534 (O–H carboxylic acid/ S–H thiol),1684vs (C=O carboxylic acid), 1600w (CC aromatic), 1575w (CC aromatic), 1490w, 1445w, 1406w, 1296m, 1265s, 1198w, 1164w, 1132w, 1047w, 923w, 839w, 802w, 764s, 713s, 655m, 583w, 555w, 491w; δ_H (400 MHz; $CDCl_3$) 8.07 (1H, dd, *J* = 7.6, 1.5 Hz, C(3)H), 7.51 (1H, td, *J* = 7.6, 1.5 Hz, C(5)H), 7.43 – 7.30 (2H, m, C(5+7)H), 4.19 (2H, d, *J* = 6.4 Hz, C(8)H_2), 2.84 – 2.44 (8H, m, C(9+11+12+14)H_2), 2.04 – 1.73 (4H, m, C(10+13)H_2), 1.36 (1H, t, *J* = 8.1 Hz, SH); δ_C (101 MHz; $CDCl_3$) 173.2 (C, C1), 141.8 (C, C7), 133.0 (CH, C6), 132.3 (CH, C3), 131.4 (CH, C5), 128.3 (C, C2), 127.4 (CH, C4), 34.7 (CH_2, C8), 33.4 (CH_2, C13), 30.9 (CH_2, C9), 30.8 (CH_2, C11), 30.4 (CH_2, C12), 29.2 (CH_2, C10), 23.5 (CH_2, C14); HRMS (ESI): calcd. for $C_{14}H_{20}NaO_2S_3$, 339.0518. Found: $[MNa]^+$, 339.0511 (2.1 ppm error).

4,5,8,9-Tetrahydro-3H,7H-benzo[k][1,5,9]trithiacyclotridecin-1(11H)-one (126)

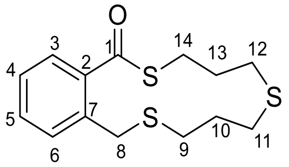

EDC.HCl (167 mg, 0.873 mmol) was added to a pale yellow solution of 2-(((3-((3-mercaptopropyl)thio)propyl)thio)methyl)benzoic acid **S170** (184 mg, 0.582 mmol), HOBt (94.3 mg, 0.698 mmol) and dry DIPEA (250 µL, 1.46 mmol) in anhydrous DMF (5.82 mL) at RT. An immediate colour change to yellow was observed. After total of 18 h of stirring at RT under Ar, the reaction was deemed to have gone completion by TLC analysis. The reaction mixture was diluted with EtOAc (30 mL) and poured into separating funnel. The diluted solution was washed with sat. brine (4 × 30 mL), before being dried over $MgSO_4$, filtered and concentrated under reduced pressure to yield a colourless oil (246 mg). The crude product was purified by flash column chromatography (SiO_2, 30 mm column, eluent: EtOAc: *n*–hexane,1:9) to afford the title compound **126** as a pale-yellow oil (71.9 mg, 41%); R_f = 0.58 (50:50 EtOAc:*n*–hexane); IR (neat) ν_{max} / cm^{-1}: 2921m (C–H alkyl), 2853w (C–H alkyl), 1731w, 1659vs (C=O aryl ester), 1597w (CC aromatic), 1571w (CC aromatic), 1480w, 1443m, 1343w, 1300w, 1207s, 1189s, 1042m, 952w, 913vs, 885m, 806w, 762s, 682m, 650m, 587w, 549w, 508w; δ_H (400 MHz; CD_6D_6) 7.48 (1H, dd, *J* = 7.7, 1.4 Hz, C(3)H), 7.09 (1H, dd, *J* = 7.7, 1.4 Hz, C(6)H), 6.93 (1H, td, *J* = 7.7, 1.4 Hz, C(5)H), 6.82 (1H, td, *J* = 7.7, 1.4 Hz, C(4)H), 3.92 (2H, s, C(8)H_2), 2.85 – 2.71 (2H, m, C(14)H_2), 2.62 – 2.51 (2H, m, C(12)H_2), 2.39 – 2.32 (2H, m, C(11)H_2), 2.27 – 2.17 (2H, m, C(9)H_2), 1.87 – 1.75 (2H, m, C(13)H_2), 1.73 – 1.63 (2H, m, C(10)H_2); δ_C (126 MHz; CD_6D_6) 194.6 (C, C1), 139.7 (C, C7), 136.5 (C, C2), 131.6 (CH, C6), 131.3 (CH, C5), 127.7 (CH, C3), 127.2 (CH, C4), 32.9 (CH_2, C8), 31.9 (CH_2, C9), 31.3 (CH_2, C13), 31.0 (CH_2, C11), 30.7 (CH_2, C12), 30.7 (CH_2, C10), 29.0 (CH_2, C14); HRMS (ESI): calcd. for $C_{14}H_{18}NaOS_3$, 321.0412. Found: $[MNa]^+$, 321.0411 (0.2 ppm error).

3,3'-(1,2-Phenylenebis(methylazanediyl))bis(propan-1-ol) (S171)

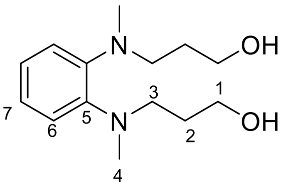

K_2CO_3 (3.55 g, 25.7 mmol) was added to a black brown suspension of N^1,N^2-diphenylethane-1,2-diamine (1.01 g, 7.34 mmol), 4-bromobutan-1-ol (1.99 mL, 22.1 mmol) and KI (3.05 g, 18.3 mmol) in anhydrous MeCN (25.0 mL) at RT under Ar. The resulting mixture was then heated to 95 °C under Ar. After 18 h, the reaction was deemed to have gone to completion by TLC. The reaction mixture was allowed to cool to RT, before being filtered through Celite and washed with EtOAc (3 × 30 mL). The resulting filtrate was concentrated under reduced pressure to yield a dark brown oil (3.71 g). The crude product was purified by flash column chromatography (SiO_2, 30 mm column, eluent: EtOAc:*n*-hexane, 90:10 to 100:0) to afford the title compound **S171** as a pale yellow viscous oil (973 mg, 53%). R_f = 0.08 (EtOAc); IR (neat) ν_{max} / cm $^{-1}$: 3326w, (O–H alcohol), 2942w (C–H alkyl), 2846w (C–H alkyl), 1590w (CC aromatic), 1493s, 1453w, 1382w, 1274w, 1211w, 1173w, 1122w, 1054s, 954w, 928w, 747s, 708w, 665w, 556w, 487w; δ_H (400 MHz; $CDCl_3$) 7.12 – 7.07 (2H, m, C(7)H), 7.06 – 7.02 (2H, m, C(6)H), 4.68 (2H, s, br, OH), 3.66 (4H, t, *J* = 5.9 Hz, C(1)H_2), 3.00 (4H, t, *J* = 6.8 Hz, C(3)H_2), 2.65 (6H, s, C(4)H_3), 1.72 – 1.63 (4H, m, C(2)H_2); δ_C (101 MHz; $CDCl_3$) 147.9 (2 × C, C5), 124.4 (2 × CH, C6), 121.4 (2 × CH, C7), 60.0 (2 × CH_2, C1), 53.4 (2 × CH_2, C3), 41.5 (2 × CH_3, C4), 29.3 (2 × CH_2, C2); HRMS (ESI): calcd. for $C_{14}H_{25}N_2O_2$, 253.1911. Found: $[MH]^+$, 253.1908 (0.9 ppm error).

1,11-Dimethyl-6-phenyl-1,2,3,4,8,9,10,11-octahydrobenzo[h][1,3]dioxa[7,10]diaza[2]phosphacyclotridecine 6-oxide (127)

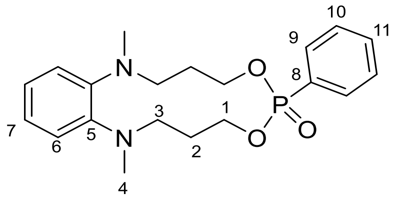

Phenylphosphonic dichloride (269 µL, 1.957 mmol) was added in a single portion via syringe to a solution of 3,3'-(1,2-phenylenebis(methylazanediyl))bis(propan-1-ol) **S171** (374 mg, 1.48 mmol), DMAP (633 mg, 5.18 mmol) in anhydrous CH_2Cl_2 (30.0 mL) at 0 °C under Ar. The resulting mixture was allowed to warm gradually to RT overnight under Ar and the progress

of the reaction was monitored *via* TLC. After total of 18 h, the reaction was deemed to have gone to completion by TLC analysis. The resulting mixture was concentrated under reduced pressure to yield a pale-yellow solid (985 mg). The crude product was purified by flash column chromatography (SiO_2, 30 mm column, eluent: EtOAc:*n*–hexane, 90:10 to 100:0) to afford the title compound **127** as a colourless viscous oil (333 mg, 60%). R_f = 0.42 (EtOAc); IR (neat) ν_{max} / cm $^{-1}$: 2955w (C–H alkyl), 1738w, 1591w (CC aromatic), 1494m, 1450w, 1439w,1239s, 1185m, 1131s, 1086w, 1043s, 985s, 886w, 856w, 817w, 791w, 744s, 711m, 694s, 664m, 560s; δ_H (400 MHz; $CDCl_3$) 7.77 – 7.68 (2H, m, C(10)H), 7.54 – 7.44 (1H, m, C(11)H), 7.44 – 7.36 (2H, m, C(9)H), 6.95 (4H, s, C(6+7)H), 4.31 – 4.19 (2H, m, 2 × C(1)H**H'**), 4.09 – 3.97 (2H, m, 2 × C(1)**H**H'), 3.57 – 3.46 (2H, m, 2 × C(3)H**H'**), 3.33 – 3.20 (2H, m, 2 × C(3)**H**H'), 2.76 (6H, s, C(4)H_3), 2.03 – 1.91 (2H, m, 2 × C(2)H**H'**), 1.88 – 1.76 (2H, m, 2 × C(2)**H**H'); δ_C (101 MHz; $CDCl_3$) 145.2 (2 × C, C5), 132.2 (d, *J* = 3.1 Hz, CH, C11), 131.2 (d, *J* = 9.7 Hz, CH, C10), 128.4 (d, *J* = 192.2 Hz, C, C8), 128.34 (d, *J* = 15.2 Hz, CH, C9), 122.3 (2 × CH, C6), 119.6 (2 × CH, C7), 64.1 (d, *J* = 6.5 Hz, 2 × CH_2, C1), 49.1 (2 × CH_2, C3), 39.9 (2 × CH_3, C4), 27.41 (d, *J* = 6.3 Hz, 2 × CH_2, C2); δ_P (162 MHz; $CDCl_3$) 18.3 (s, $PhPO(O)_2$); HRMS (ESI): calcd. for $C_{20}H_{28}N_2O_3P$, 375.1832. Found: $[MH]^+$, 375.1826 (1.6 ppm error).

***N*,*N*'-Bis(salicylidene)-*o*-phenylenediamine (S172)**

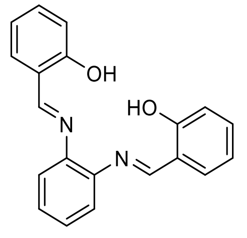

To a solution of salicylaldehyde (2.10 mL, 2.44 g, 20.0 mmol) in ethanol (74 mL) was added a solution of *o*-phenylenediamine (1.08 g, 10.0 mmol) in ethanol (50 mL) and the resulting solution was refluxed for 8 h. The mixture was concentrated *in vacuo* and the solid was washed with ethanol to yield the title compound (2.14 g, 68%) as an orange solid. δ_H (400 MHz, $CDCl_3$) 13.05 (2H, s, OH), 8.65 (2H, s, NCH), 7.41–7.33 (6H, m, ArH), 7.26–7.23 (2H, m, ArH), 7.06 (2H, d, *J* = 8.0 Hz, ArH), 6.94 (2H, t, *J* = 7.5 Hz, ArH). Characterisation data matched those reported in the literature.[19]

(13*E*,19*E*)-Tribenzo[e,i,m][1,4]dioxa[8,11]diazacyclotetradecine-6,7-dione (128)

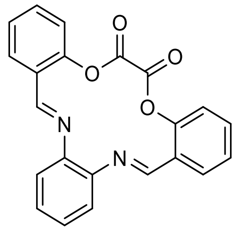

To a solution of *N*,*N*'-bis(salicylidene)-*o*-phenylenediamine **S172** (316 mg, 1.00 mmol) and triethylamine (0.700 mL, 506 mg, 5.00 mmol) in DCM (10 mL) was added oxalyl chloride (0.100 mL, 140 mg, 1.10 mmol) and the mixture was stirred at RT for 18 h. The reaction mixture was concentrated *in vacuo* and purified by silica plug filtration (ethyl acetate) to yield the title compound **128** (216 mg, 58%) as a brown solid. δ_H (400 MHz, $CDCl_3$) 8.50 (2H, s, NCH), 7.61 (2H, dd, *J* = 7.5, 1.5 Hz, ArH), 7.47 (2H, td, *J* = 7.5, 1.5 Hz, ArH), 7.36 (2H, td, *J* = 7.5, 1.5 Hz, ArH), 7.28–7.25 (2H, m, ArH), 7.23–7.16 (4H, m, ArH); δ_C (101 MHz, $CDCl_3$) 155.8 (NCH), 155.1 (CO), 148.2 (ArC), 144.9 (ArC), 133.9 (ArC), 131.8 (ArC), 128.1 (ArC), 127.2 (ArC), 127.0 (ArC), 123.1 (ArC), 117.4 (ArC); ν_{max}/cm^{-1} (thin film) 2879, 1789, 1765, 1620, 1572, 1482, 1374, 1279, 1180, 1130, 906, 831, 756, 724, 647; HRMS (ESI); calcd. for $C_{22}H_{15}N_2O_4^+$, 371.1026. Found: $[MH]^+$, 371.1010 (4.4 error ppm).

1,11-Dimethyl-1,2,3,4,8,9,10,11-octahydrobenzo[h][1,3]dioxa[2]thia[7,10]diazacyclotridecine 6-oxide (129)

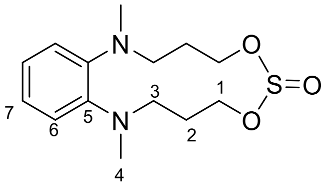

Thionyl chloride (133 µL, 1.83 mmol) was added in a single portion via syringe to a pale yellow solution of 3,3'-(1,2-phenylenebis(methylazanediyl))bis(propan-1-ol) **S171** (355 mg, 1.41 mmol), DMAP (601 mg, 4.92 mmol) in anhydrous CH_2Cl_2 (28.0 mL) at 0 °C under Ar. The resulting mixture was allowed to warm gradually to RT overnight under Ar and the progress of the reaction was monitored *via* TLC, with a colour change to a pale brown solution observed. After a total of 18 h, the reaction was deemed to have gone to completion by TLC analysis and a colour change to yellow was noted. The resulting mixture was concentrated under reduced pressure to yield a brown oil (412 mg). The crude product was purified by flash column chromatography (SiO_2, 40 mm column, eluent: EtOAc:*n*–hexane, 50:50) to afford the

title compound **129** as a colourless oil (219 mg, 52%). R_f = 0.73 (EtOAc); IR (neat) ν_{max} / cm^{-1}: 2945m (C–H alkyl), 2800w (C–H alkyl), 1591w (CC aromatic), 1494s, 1453m, 1375w, 1301w, 1267w, 1200s (S=O sulfite), 1121w, 1060m, 1016w, 922s, 900s, 835w, 813s, 734s, 701s, 605w, 583w, 509w; δ_H (400 MHz; $CDCl_3$) 6.97 (4H, s, C(6+7)H), 4.22 – 4.09 (2H, m, 2 × C(1)H**H'**), 4.07 – 3.93 (2H, m, 2 × C(1)**H**H'), 3.41 – 3.28 (2H, m, 2 × C(3)H**H'**), 3.25 – 3.14 (2H, m, 2 × C(3)**H**H'), 2.74 (6H, s, C(4)H_3), 1.98 – 1.77 (4H, m, C(2)H_2); δ_C (101 MHz; $CDCl_3$) 145.6 (2 × C, C5), 122.8 (2 × CH, C6), 120.2 (2 × CH, C7), 60.5 (2 × CH_2, C1), 50.0 (2 × CH_2, C3), 40.5 (2 × CH_3, C4), 26.1 (2 × CH_2, C2); HRMS (ESI): calcd. for $C_{14}H_{23}N_2O_3S$, 299.1424. Found: $[MH]^+$, 299.1418 (2.1 ppm error).

***N*^1,*N*^3-dibenzylpropane-1,3-diamine (S173)**

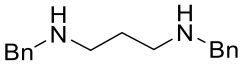

To a stirring solution of 1,3-diaminopropane (5.00 mL, 59.9 mmol) in DCM (150 mL), sodium sulfate (18.7 g, 132 mmol) and benzaldehyde (13.5 mL, 132 mmol) were added and stirred at room temperature for 16 hours. The reaction mixture was filtered, concentrated under vacuum and redissolved in EtOH (100 mL). Sodium borohydride (6.79 g, 179 mmol) was added to the stirring solution at 0 °C in portions and stirred at room temperature for 16 hours. The reaction was diluted with H_2O (100 mL), extracted with ethyl acetate (3 × 100 mL), the combined organic phase washed with brine (50 mL), dried with sodium sulfate, filtered, concentrated under vacuum to afford the title compound **S173** as a colourless oil (13.9 g, 91%). R_f 0.13 (2:3 hexane:ethyl acetate); ν_{max} (thin film)/cm^{-1} 3062, 3027, 2925, 2814, 1495, 1453, 1358, 1113, 1028, 732, 696, 595, 463; ^{1}H NMR (400 MHz, $CDCl_3$) δ 7.40 – 7.24 (10H, m, Ar**H**), 3.81 (4H, s, PhC**H**$_2$), 2.74 (4H, t, *J* = 7.0 Hz, NHC**H**$_2$CH$_2$), 1.76 (2H, t, *J* = 7.0 Hz, NHCH$_2$C**H**$_2$), 1.65 (2H, s (Br), N**H**); ^{13}C NMR (101 MHz, $CDCl_3$) δ 140.5 (Ar**C**), 128.4 (Ar**C**), 128.2 (Ar**C**), 126.9 (Ar**C**), 54.2 (Ph**C**H$_2$), 48.1 (NH**C**H$_2$CH$_2$), 30.2 (NHCH$_2$**C**H$_2$); HRMS (ESI$^+$) Calcd. for $C_{17}H_{23}N_2$ 255.1856; Found [MH$^+$] 255.1855 (0.1 ppm error).

N^1,N^3-dibenzyl-N^1,N^3-bis(3-((*tert*-butyldimethylsilyl)oxy)propyl)propane-1,3-diamine (S174)

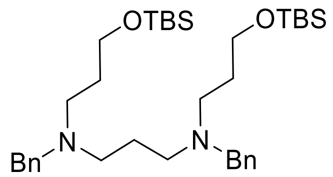

To a stirring solution of **S173** (299 mg, 1.18 mmol) in MeCN (12 mL), (3-bromopropoxy)-*tert*-butyldimethylsilane (308 mg, 1.22 mmol) and potassium carbonate (489 mg, 3.54 mmol) were added and stirred at 90 °C for 16 hours. The reaction mixture was diluted with H_2O (30 mL), extracted with ethyl acetate (3 × 30 mL), the combined organic phase dried with sodium sulfate, filtered, concentrated under vacuum and purified via flash column chromatography (3:2 hexane:ethyl acetate) to afford the title compound **S174** as a colourless oil (169 mg, 24%). R_f 0.80 (3:2 hexane:ethyl acetate). ν_{max} (thin film)/cm^{-1} 3028, 2952, 2929, 2857, 2800, 1974, 1495, 1472, 1361, 1254, 1096, 965, 835, 775, 733, 697. ^{1}H NMR (400 MHz, $CDCl_3$) δ 7.32 – 7.19 (10H, m, Ar**H**), 3.59 (4H, t, *J* = 6.5 Hz, C**H**$_2$O), 3.52 (4H, s, PhC**H**$_2$), 2.46 (4H, t, *J* = 7.5 Hz, NC**H**$_2$CH$_2$), 2.41 (4H, t, *J* = 7.5 Hz, NC**H**$_2$CH$_2$), 1.71 – 1.60 (6H, m, NCH$_2$C**H**$_2$), 0.87 (18H, s, C(C**H**$_3$)$_3$), 0.02 (12H, s, Si(C**H**$_3$)$_2$). ^{13}C NMR (101 MHz, $CDCl_3$) δ 140.1 (Ar**C**), 129.0 (Ar**C**), 128.2 (Ar**C**), 126.8 (Ar**C**), 61.7 (O**C**H$_2$), 58.8 (Ph**C**H$_2$), 52.2 (N**C**H$_2$), 50.6 (N**C**H$_2$), 30.4 (OCH$_2$**C**H$_2$), 26.1 (C(**C**H$_3$)$_3$), 24.6 (NCH$_2$**C**H$_2$), 18.4 (**C**(CH$_3$)$_3$), -5.2 (Si(**C**H$_3$)$_2$). HRMS (ESI$^+$) $C_{35}H_{63}N_2O_2Si_2$ (MH$^+$) theoretical 599.4423; measured 599.4451 (–3.6 ppm error).

3,3'-(Propane-1,3-diylbis(benzylazanediyl))bis(propan-1-ol) (S175)

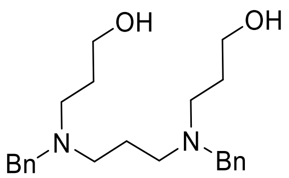

To a stirring solution of N^1,N^3-dibenzyl-N^1,N^3-bis(3-((*tert*-butyldimethylsilyl)oxy)propyl)propane-1,3-diamine **S174** (165 mg, 275 µmol) in THF (2 mL), TBAF (0.61 mL, 610 µmol, 1M solution in THF) was added and stirred at 0 °C for 1 hour. The reaction mixture was warmed to room temperature and stirred for a further 3 hours. The reaction mixture was purified via flash column chromatography (19:1 ethyl acetate:triethyl amine) to afford the title compound **S175** as a colourless oil (50.9 mg, 50%). R_f 0.19 (19:1 ethyl

acetate:triethyl amine); ν_{max} (thin film)/cm^{-1} 3321, 3027, 2926, 2806, 1452, 1369, 1138, 1058, 911, 732, 698; ^{1}H NMR (400 MHz, $CDCl_3$) δ 7.34 – 7.19 (10H, m, Ar**H**), 5.02 (2H, s (Br), O**H**), 3.68 (4H, t, *J* = 5.0 Hz, C**H**$_2$OH), 3.51 (4H, s, PhC**H**$_2$), 2.60 (4H, t, *J* = 5.5 Hz, NC**H**$_2$), 2.39 (4H, t, *J* = 7.5 Hz, NC**H**$_2$), 1.71 – 1.60 (6H, m, NCH$_2$C**H**$_2$), 1.81 – 1.63 (6H, m, NCH$_2$C**H**$_2$); ^{13}C NMR (101 MHz, $CDCl_3$) δ 138.3 (Ar**C**), 129.3 (Ar**C**), 128.5 (Ar**C**), 127.3 (Ar**C**), 63.9 (**C**H$_2$OH), 59.0 (Ph**C**H$_2$), 53.9 (N**C**H$_2$), 51.9 (N**C**H$_2$), 28.1 (**C**H$_2$CH$_2$OH), 24.3 (NCH$_2$**C**H$_2$); HRMS (ESI$^+$) Calcd. for $C_{23}H_{35}N_2O_2$ 371.2693; Found: [MH]$^+$, 371.2695 (–1.1 ppm error).

7,11-Dibenzyl-1,3-dioxa-2-thia-7,11-diazacyclotetradecane 2-oxide (130)

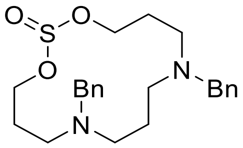

To a stirring solution of **S175** (50.9 mg, 137 μmol) in DCM (1.37 mL), DMAP (84.3 mg, 690 μmol) and thionyl chloride (12.0 μL, 165 μmol) was added and stirred at room temperature for 24 hours. The reaction mixture was purified via flash column chromatography (18:1:1 hexane:ethyl acetate:triethyl amine) to afford the title compound **130** as a colourless film (40.9 mg, 71%). R_f 0.36 (18:1:1 hexane:ethyl acetate:triethyl amine). ν_{max} (thin film)/cm^{-1} 3027, 2930, 2800, 1700, 1494, 1452, 1369, 1204, 909, 731, 698. ^{1}H NMR (400 MHz, $CDCl_3$) δ 7.43 – 7.13 (10H, m, Ar**H**), 4.24 – 4.16 (2H, m, C**H**$_2$O), 4.09 – 4.02 (2H, m, C**H**$_2$O), 3.56 – 3.44 (4H, m, PhC**H**$_2$), 2.63 – 2.49 (4H, m, NC**H**$_2$), 2.48 – 2.38 (4H, m, NC**H**$_2$), 2.00 – 1.78 (4H, m, OCH$_2$C**H**$_2$), 1.70 – 1.57 (2H, m, NCH$_2$C**H**$_2$). ^{13}C NMR (101 MHz, $CDCl_3$) δ 139.9 (Ar**C**), 128.8 (Ar**C**), 128.3 (Ar**C**), 127.0 (Ar**C**), 59.8 (**C**H$_2$O), 59.1 (Ph**C**H$_2$), 51.5 (N**C**H$_2$), 50.2 (N**C**H$_2$), 27.5 (**C**H$_2$CH$_2$OH), 26.2 (NCH$_2$**C**H$_2$). HRMS (ESI$^+$) Calcd. for $C_{23}H_{33}N_2O_3S$ 417.2206; Found: [MH]$^+$, 417.2200 (0.7 ppm error).

3,3'-(((Methylazanediyl)bis(propane-3,1-diyl))bis(methylazanediyl))bis(propan-1-ol) (S176)

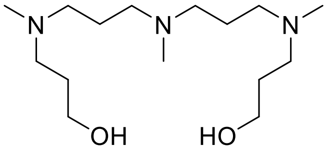

To a solution of *N*,*N*-bis[3-(methylamino)propyl]methylamine (0.200 mL, 173 mg, 1.00 mmol) in DCM (5 mL) was added potassium carbonate (415 mg, 3.00 mmol) followed by 3-bromo-1-propanol (0.200 mL, 306 mg, 2.20 mmol) and the reaction mixture was stirred at room temperature for 18 h. The solids were filtered off and washed with DCM and the filtrate

concentrated *in vacuo*. The crude product was purified by column chromatography (SiO_2, 19:1 methanol:triethylamine) to yield the title compound **S176** (165 mg, 57%) as a colourless oil. R_f 0.23 (19:1 MeOH:triethylamine); ν_{max}/cm^{-1} (thin film) 3329, 2945, 2842, 2791, 1461, 1372, 1318, 1213, 1163, 1057, 923, 831, 730; δ_H (400 MHz, $CDCl_3$) 5.35 (2H, s, OH), 3.64 (4H, t, *J* = 5.5 Hz, C**H**$_2$OH), 2.47 (4H, t, *J* = 6.0 Hz, NCH_2), 2.32–2.21 (8H, m, NCH_2), 2.14 (6H, s, NCH_3), 2.10 (3H, s, NCH_3), 1.62–1.50 (8H, m, CH_2); δ_C (101 MHz, $CDCl_3$) 63.7 (CH_2OH), 57.6 (NCH_2), 56.0 (NCH_2), 55.4 (NCH_2), 42.0 (NCH_3), 41.9 (NCH_3), 27.9 (CH_2), 24.7 (CH_2); HRMS (ESI); calcd. for $C_{15}H_{36}N_3O_2^+$, 290.2802: Found $[MH]^+$, 290.2810 (–2.9 error ppm).

7,11,15-Trimethyl-1,3-dioxa-2-thia-7,11,15-triazacyclooctadecane 2-oxide (131)

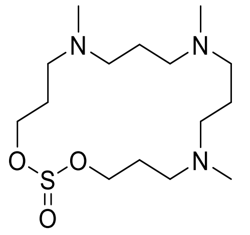

To a solution of 3,3'-(((methylazanediyl)bis(propane-3,1-diyl))bis(methylazanediyl))bis(propan-1-ol) **S176** (165 mg, 0.570 mmol) and DMAP (348 mg, 2.85 mmol) in DCM (5.7 mL) was added thionyl chloride (0.050 mL, 81 mg, 0.684 mmol) and the reaction mixture was stirred at RT for 18 h. The reaction mixture was concentrated *in vacuo* and purified by column chromatography (SiO_2, 19:1 methanol:triethylamine) to yield the title compound **131** (122 mg, 64%) as a yellow oil. R_f 0.38 (19:1 methanol:triethylamine); ν_{max}/cm^{-1} (thin film) 2947, 2840, 2789, 1647, 1461, 1276, 1317, 1205, 1079, 912, 843, 785, 705; δ_H (400 MHz, $CDCl_3$) 4.12–3.96 (4H, m, OCH_2), 2.41–2.28 (12H, m, 3 x NCH_2), 2.20 (3H, s, NCH_3), 2.13 (6H, s, NCH_3), 1.82–1.73 (4H, m, CH_2), 1.63–1.54 (4H, m, CH_2); δ_C (101 MHz, $CDCl_3$) 60.6 (OCH_2), 55.6 (NCH_2), 54.8 (NCH_2), 52.7 (NCH_2), 43.0 (NCH_3), 42.5 (NCH_3), 27.4 (CH_2), 24.9 (CH_2); HRMS (ESI); calcd. for $C_{15}H_{34}N_3O_3S^+$, 336. 2315. Found: $[MH]^+$, 336.2324 (–2.6 error ppm).

Methyl 2-(((3-((3-bromopropyl)thio)propyl)selanyl)methyl)benzoate (S177)

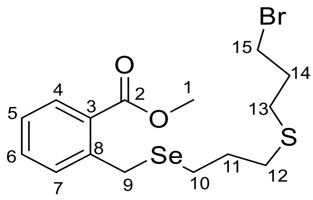

PPh_3 (1.04 g, 3.96 mmol) was added slowly in equal portions over a period of 2 min to a solution of methyl 2-(((3-((3-hydroxypropyl)thio)propyl)selanyl)methyl)benzoate **S166** (1.19 g, 3.30 mmol) and CBr_4 (1.31 g, 3.96 mmol) in anhydrous CH_2Cl_2 (33 mL) at 0 °C. A colour change to pale yellow solution was immediately noted. The mixture stirred for 2 h at 0 °C and then allowed to warm to RT and stirred for additional 16 h. After total of 18 h, the reaction was deemed to have gone to completion by TLC. The reaction mixture was concentrated under reduced pressure not to dryness to yield a brown/orange oil, which was diluted with Et_2O (30 mL). The resulting white solid (Ph_3PO) was filtered, and the filter cake was washed with Et_2O (3 × 20 mL). The resulting filtrate was concentrated under reduced pressure to yield a crude product as an orange oil (1.72 g). The crude product was purified by flash column chromatography (SiO_2, 50 mm column, eluent: Et_2O:n-hexane, 10:90) to afford the title compound **S177** as a colourless oil (798 mg, 57%). R_f = 0.078 (50:50 EtOAc:n-hexane); IR (neat) ν_{max} / cm^{-1}: 2947w (C-H alkyl), 2841w (C-H alkyl), 1715s (C=O aryl ester), 1599w (CC aromatic), 1575w (CC aromatic), 1487m (CC aromatic), 1433w, 1292w, 1260s, 1189m, 1164w, 1112m, 1074s, 1045m, 964w, 840w, 791w, 758s, 707m, 664w, 616w, 562w, 512w, 462w; δ_H (400 MHz; $CDCl_3$) 7.93 (1H, dd, *J* 7.5, 1.3, C(4)H), 7.42 (1H, td, *J* 7.5, 1.3, C(6)H), 7.33 – 7.23 (2H, m, C(5+7)H), 4.18 (2H, s, C(9)H_2), 3.91 (3H, s, C(1)H_3), 3.50 (2H, t, *J* 6.4, C(15)H_2), 2.66 – 2.58 (4H, m, C(10+12)H_2), 2.62 – 2.51 (2H, m, C(13)H_2), 2.14 – 2.03 (2H, m, C(14)H_2), 1.93 – 1.82 (2H, m, C(11)H_2); δ_C (126 MHz; $CDCl_3$) 167.5 (C, C2), 142.1 (C, C8), 131.8 (CH, C7), 131.2 (CH, C4), 130.7 (CH, C6), 128.7 (C, C3), 126.7 (CH, C5), 52.0 (CH_3, C1), 32.2 (CH_2, C15), 32.1 (CH_2, C14), 31.8 (CH_2, C12), 30.1 (CH_2, C13), 30.0 (CH_2, C11), 25.8 (CH_2, C9), 23.0 (CH_2, C10); HRMS (ESI$^+$): Calcd. for $C_{15}H_{22}{}^{79}BrO_2S^{80}Se$: 424.9684, found: 424.9679 $[MH]^+$, calcd. for $C_{15}H_{21}{}^{79}BrNaO_2S^{80}Se$: 446.9503, found : 446.9501 $[MNa]^+$, calc. for $C_{15}H_{21}{}^{79}BrKO_2S^{80}Se$: 462.9242, found : 462.9257 $[MK]^+$.

Methyl 2-(((3-((3-(benzyl(3-hydroxypropyl)amino)propyl)thio)propyl)selanyl)methyl)benzoate (S178)

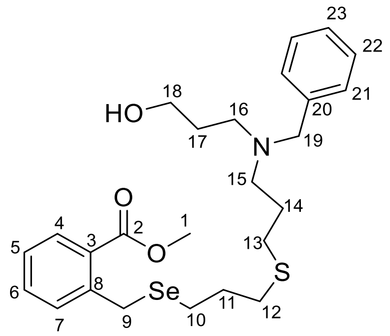

K_2CO_3 (534 mg, 3.86 mmol) was added pale yellow solution of 3-(benzylamino)propan-1-ol (478 mg, 2.89 mmol) and methyl 2-(((3-((3-bromopropyl)thio)propyl)selanyl)methyl)benzoate **S177** (818 mg, 1.93 mmol) in anhydrous MeCN (19 mL) at RT. The resulting suspension was then heated to 70 °C, whereupon a colour change to milky/white was noted. After 16 h, the reaction was deemed to have gone to completion by TLC, whereupon a colour change to cloudy yellow suspension was observed. The reaction mixture was cooled to RT, before filtered through Celite using Hirsch funnel and the filtrate residual was washed with EtOAc (3 × 20 mL). The resulting solution was concentrated to dryness under reduced pressure to yield crude product as a pale yellow oil (1.23 g). The crude product was purified by flash column chromatography (SiO_2, 50 mm column, eluent: EtOAc:*n*–hexane, 90:10 to 100:0) to afford the title compound **S178** as a colourless oil (554 mg, 56%). R_f = 0.22 (80:20 EtOAc:n-hexane); IR (neat) ν_{max} / cm^{-1}: 3420mvbr (O–H alcohol), 2938w (C–H alkyl), 2835w (C–H alkyl), 1716s (C=O aryl ester), 1599w (CC aromatic), 1578w (CC aromatic), 1493m (CC aromatic), 1434m, 1262vs, 1189m, 1112m, 1073s, 967w, 753w, 741s, 665m, 616w; δ_H (400 MHz; $CDCl_3$) 7.91 (1H, dd, *J* 7.6, 1.6, C(4)H), 7.400 (1H, td, *J* 7.6, 1.6, C(6)H), 7.36 – 7.23 (7H, m, C(5+7+21+22+23)H), 4.17 (2H, s, C(9)H_2), 3.90 (3H, s, C(1)H_3), 3.72 (2H, t, *J* 6.5, C(18)H_2), 3.57 (2H, s, C(19)H_2), 2.65 (2H, t, *J* 6.5, C(16)H_2), 2.59 (2H, t, *J* 7.2, C(15)H_2), 2.55 – 2.48 (4H, m, C(10+12)H_2), 2.43 (2H, t, *J* 7.3, C(13)H_2), 1.91 – 1.70 (6H, m, C(11+14+17)H_2); δ_C (126 MHz; $CDCl_3$) 167.8 (C, C2), 142.4 (C, C8), 138.4 (C, C20), 132.1 (CH, C7), 131.5 (CH, C4), 131.0 (CH, C6), 129.3 (CH, C21), 128.9 (C, C3), 128.6 (CH, C22), 127.4 (CH, C23), 126.9 (CH, C5), 64.3 (CH_2, C18), 59.1 (CH_2, C19), 54.3 (CH_2, C16), 53.0 (CH_2, C15), 52.2 (CH_3, C1), 32.2 (CH_2, C12), 30.2 (CH_2, C17), 30.1 (CH_2, C11), 28.2 (CH_2, C13), 26.9

(CH$_2$, C14), 26.0 (CH$_2$, C9), 23.2 (CH$_2$, C10); HRMS (ESI$^+$): m/z calc. for $C_{25}H_{36}NO_3S^{80}Se$: 510.1576, found: 510.1580 [MH]$^+$.

2-(((3-((3-(Benzyl(3-hydroxypropyl)amino)propyl)thio)propyl)selanyl)methyl)benzoic acid (S179)

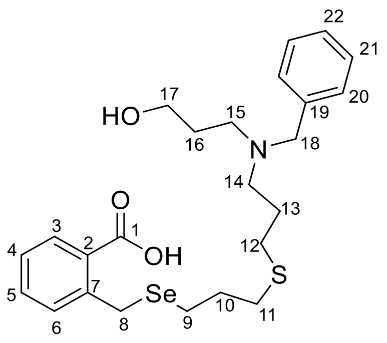

NaOH$_{(aq)}$ (4.0 M, 11 mL) was added dropwise over a period of 2 min to a clear colourless solution of 2-(((3-((3-(benzyl(3-hydroxypropyl)amino)propyl)thio)propyl)selanyl)methyl)benzoate **S178** (550 mg, 1.08 mmol) in MeOH (11 mL). A colour change to milky/white suspension was noted immediately during the dropwise addition. The resulting suspension was then heated to 60 °C under Ar for 16 h. The reaction mixture was then acidified to pH 2.0 with 1 M HCl$_{(aq)}$ (20 mL). The acidified cloudy/white suspension was poured into a separating funnel and aqueous layer was extracted with EtOAc (3 × 20 mL), before the combined organic layers were dried over $MgSO_4$, filtered and concentrated under reduced pressure to yield a yellow viscous oil. The crude product was purified by flash column chromatography (SiO_2, 30 mm column, eluent: MeOH: EtOAc, 10:90) to afford the title compound **S179** as a pale yellow oil (458 mg, 86%). R_f = 0.17 (10:90 MeOH:EtOAc); IR (neat) ν_{max} / cm^{-1}: 3342vbrm (O–H alcohol, O–H carboxylic), 3068vbrm (C–H alkyl, O–H carboxylic acid), 2938vbrm (C–H alkyl), 2586w (C–H alkyl), 1704vs (C=O aryl carboxylic acid), 1599w (CC aromatic), 1578w, (CC aromatic) 1450w, 1374w, 1219m, 1116w, 1067m, 917w, 745s, 700s, 646m, 526w; δ_H (500 MHz; CD_3OD) 7.62 (1H, d, *J* 7.4, C(4)H), 7.52 (2H, dd, *J* 7.3, 2.3, C(21)H), 7.44 – 7.38 (3H, m, C(20+22)H), 7.31 (1H, dd, *J* 7.4, 1.4, C(6)H), 7.26 (1H, t, *J* 7.4, C(4)H), 7.18 (1H, td, *J* 7.4, 1.4, C(5)H), 4.24 (2H, s, br, C(18)H$_2$), 4.19 (2H, s, C(8)H$_2$), 3.62 (2H, t, *J* 5.8, C(17)H$_2$), 3.11 – 3.03 (4H, m, C(14+15)H$_2$), 2.57 (2H, t, *J* 6.9, C(9)H$_2$), 2.54 – 2.48 (4H, m, C(11+12)H$_2$), 2.07 – 1.98 (2H, m, C(16)H$_2$), 1.97 – 1.88 (2H, m, C(13)H$_2$), 1.87 – 1.79 (2H, m, C(10)H$_2$); δ_C (126 MHz; $CDCl_3$) 169.8 (C, C1), 141.9 (C, C7), 132.1

(C, C19), 131.6 (CH, C6), 131.5 (CH, C20), 131.5 (CH, C3), 130.3 (CH, C5),129.8 (C, C2), 129.5 (CH, C21), 128.6 (CH, C22), 126.9 (CH, C4), 59.3 (CH_2, C17), 57.3 (CH_2, C18), 51.6 (CH_2, C14), 51.2 (CH_2, C15), 31.2 (CH_2, C11), 30.4 (CH_2, C10), 28.8 (CH_2, C13), 26.8 (CH_2, C16), 26.7 (CH_2, C12), 25.4 (CH_2, C10), 23.7 (CH_2, C8), 23.4 (CH_2, C9); HRMS (ESI$^+$): m/z calc. for $C_{24}H_{34}NO_3S^{80}Se$: 496.1419, found: 496.1401 $[MH]^+$.

6-Benzyl-4,5,6,7,8,9,12,13-octahydro-3H,11H-benzo[o][1]oxa[9]thia[13]selena[5]azacycloheptadecin-1(15H)-one (132)

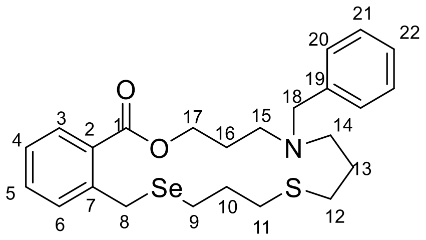

EDC.HCl (266 mg, 1.39 mmol) was added to solution of 2-(((3-((3-(benzyl(3-hydroxypropyl)amino)propyl)thio)propyl)selanyl)methyl)benzoic acid **S179** (458 mg, 0.924 mmol), HOBt (150 mg, 1.11 mmol) and anhydrous DIPEA (805 µL, 4.62 mmol) in anhydrous DMF (18 mL). A colour change of the reaction mixture over 25 min from clear colourless to pale yellow solution. After total of 18 h of stirring at RT, the reaction deemed to have to completion by TLC. The resulting mixture was diluted with EtOAc (30 mL), before was transferred to separating funnel and the organic phase was washed sequentially with H_2O (3 × 20 mL) and sat. brine (3 × 20 mL). The resulting organic layer was dried over $MgSO_4$, filtered and concentrated under reduced pressure to yield an orange oil (441 mg). The crude product was purified by flash column chromatography (SiO_2, 30 mm column, eluent: EtOAc:*n*–hexane, 30:70) to afford macrocycle **132** as a colourless viscous oil (259 mg, 59%). R_f = 0.56 (50:50 EtOAc:*n*–hexane); IR (neat) ν_{max} / cm^{-1}: 3064w (C–H alkyl), 3026w (C–H alkyl), 2932w (C–H alkyl), 2803w (C–H alkyl), 1712vs (C=O aryl ester), 1599m (CC aromatic), 1575w (CC aromatic), 1492w, 1150m, 1291w, 1257vs, 1189w, 1148w, 1112m, 1070m, 1044w, 1028w, 964w, 926w, 758m, 733w, 699w, 665w, 618w, 468w; δ_H (500 MHz; C_6D_6) 7.85 (1H, dd, *J* = 7.0, 2.0 Hz, C(3)H), 7.28 (2H, dd, *J* = 7.3, 2.0 Hz, C(20)H), 7.21 – 7.15 (2H, m, C(5+6)H), 7.12 – 7.08 (1H, m, C(22)H), 7.04 – 6.96 (2H, m, C(21)H), 6.95 – 6.87 (1H, m, C(4)H), 4.27 (2H, t, *J* = 6.6 Hz, C(17)H_2), 4.26 (2H, s, C(8)H_2), 3.27 (2H, s, C(18)H_2), 2.43 (2H, t, *J* = 6.8 Hz, C(9)H_2), 2.38 – 2.33 (4H, m, C(11+14)H_2), 2.32 – 2.25 (4H, m, C(12+15)H_2), 1.73 – 1.63 (4H, m, C(13+16)H_2), 1.66 – 1.55 (2H, m, C(10)H_2); δ_C (126 MHz; C_6D_6) 167.4 (C, C1), 142.7 (C, C7), 140.3 (C, C19), 131.7 (CH,

C5), 131.6 (CH, C3), 131.0 (CH, C6), 130.4 (C, C2), 129.2 (CH, C20), 128.6 (CH, C21), 127.2 (CH, C22), 126.7 (CH, C4), 63.4 (CH_2, C17), 59.6 (CH_2, C8), 53.1 (CH_2, C14), 50.3 (CH_2, C15), 31.3 (CH_2, C13), 30.7 (CH_2, C12), 29.9 (CH_2, C11), 28.2 (CH_2, C16), 27.0 (CH_2, C10), 25.7 (CH_2, C18), 23.9 (CH_2, C9); HRMS (ESI^+): m/z calc. for $C_{24}H_{32}O_2S^{80}Se$: 478.1313, found: 478.1319 $[MH]^+$.

Methyl 2-(8-hydroxyoct-1-yn-1-yl)benzoate (S180)

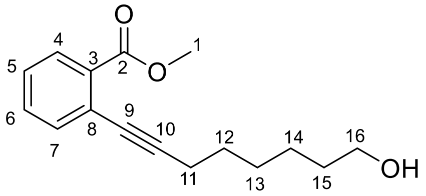

Oct-7-yn-1-ol (890 mg, 7.05 mmol) was added to solution of methyl 2-iodobenzoate (1.68 g, 6.41 mmol) in dry Et_3N (21 mL) at RT. After 5 min, CuI (122 mg, 0.641 mmol) and bis(triphenylphosphine)palladium chloride (225 mg, 0.321 mol) was added to the pale-yellow solution. The resulting reaction mixture was stirred at RT. Upon stirring, the colour of the reaction mixture changed over 4 h from grey green (after 1 h) to dark brown (after 2 h) and finally black brown. After a total of 2 h, the reaction was deemed to have gone to completion by TLC. The dark brown reaction mixture was filtered through Celite, washed with EtOAc (20 mL). The resulting filtrate was diluted with EtOAc (20 mL) was poured to into separating funnel and the organic layer was washes sequentially with $HCl_{(aq)}$ (1.0 M, 3 × 40 mL) and brine (1 × 40 mL). The organic layer was dried with $MgSO_4$, filtered and concentrated under reduced pressure to yield a brown/orange oil (2.74 g). The crude product was purified by flash column chromatography (SiO_2, 65 mm column, eluent: EtOAc:*n*–hexane, 50:50) to afford the title compound **S180** as a yellow liquid (1.79 g, 98%); R_f = 0.08 (30:70 EtOAc:*n*-hexane); IR (neat) ν_{max} / cm^{-1}: 3379mvbr (O–H alcohol), 2933m (C–H alkyl), 2859 (C–H alkyl), 2228w (CC alkynyl), 1719vs (C=O aryl ester), 1597m (CC aromatic), 1557m (CC aromatic), 1485m, 1447w, 1433m, 1293m, 1276m, 1249s, 1190w, 1163w, 1130m, 1055m, 1043m, 964w, 824w, 798w, 754s, 701m, 666w, 655w, 538w; δ_H (500 MHz; $CDCl_3$) 7.88 (1H, dd, *J* 7.7, 1.4, C(4)H), 7.50 (1H, dd, *J* 7.7, 1.4, C(7)H), 7.42 (1H, td, *J* 7.7, 1.4, C(5)H), 7.30 (1H, td, *J* 7.7, 1.4, C(6)H), 3.91 (3H, s, C(1)H_3), 3.68 – 3.64 (2H, m, C(16)H_2), 2.49 (2H, t, *J* 7.0, C(11)H_2), 1.72 –1.58 (4H, m, C(12+14)H_2), 1.57 –1.50 (2H, m, C(15)H_2), 1.46 –1.38 (2H, m, C(13)H_2); δ_C (126 MHz; $CDCl_3$) 167.1 (C, C2), 134.4 (CH, C7), 132.0 (C, C8), 131.6 (CH, C5), 130.3 (CH, C4), 127.3 (CH, C6),

124.6 (C, C3), 96.0 (C, C10), 79.5 (C, C9), 63.0 (CH_2, C16), 52.2 (CH_3, C1), 32.8 (CH_2, C12), 28.7 (CH_2, C14), 25.4 (CH_2, C13), 19.8 (CH_2, C11); HRMS (ESI$^+$): m/z calc. for $C_{16}H_{21}O_3$: 261.1485, found: 261.1486 $[MH]^+$, m/z calc. for $C_{16}H_{20}NaO_3$: 283.1305, found: 283.1305 $[MNa]^+$, m/z calc. for $C_{16}H_{20}KO_3$: 299.1044, found: 299.1044 $[MK]^+$.

Methyl 2-(8-hydroxyoctyl)benzoate (S181)

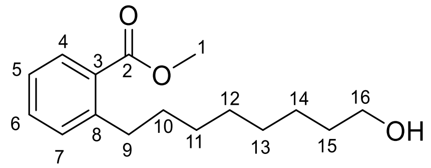

Pd/C (68.5 mg, Pd 10% on carbon) was added to a round bottom flask, previously purged under N_2 (for 10 min), containing alkyne **S180** (1.68 g, 6.43 mmol) at RT. The alkyne was dissolved by addition of MeOH (32 mL, purged with argon for 10 min) and the reaction vessel was evacuated under vacuum and then backfilled with H_2 (via balloon) three times, then stirred at RT under an atmosphere of H_2 (balloon) for 4 h. The reaction was then purged with Ar for 10 min, filtered through Celite and washed with EtOAc (3 $\times$ 20 mL). The resulting filtrate was concentrated under reduced pressure to yield a clear colourless oil (1.69 g). The crude product was purified was purified by flash column chromatography (SiO_2, 50 mm column, eluent: EtOAc:*n*–hexane, 40:60) to afford the title compound **S181** as a colourless viscous oil (1.67 g, 98%); R_f = 0.26 (50:50 EtOAc:*n*–hexane); IR (thin film) ν_{max} / cm^{-1}: 3399mbr (O–H alcohol), 2928m (C–H alkyl), 2855m (C–H alkyl), 1722vs (C=O aryl ester), 1602w (CC aromatic), 1575w (CC aromatic), 1488w (CC aromatic) 1434m, 1373w, 1291w, 1250s, 1190w, 1164w, 1132w, 1096m, 1072m, 1046m, 967w, 881w, 843w, 799w, 751m, 710m, 664w, 634w, 607w, 582w; δ_H (400 MHz; $CDCl_3$) 7.84 (1H, dd, *J* 7.7, 1.5, C(4)H), 7.40 (1H, dd, *J* 7.7, 1.5, C(7)H), 7.27 – 7.19 (2H, m, C(5+6)H), 3.88 (3H, s, C(1)H_3), 3.63 (2H, t, *J* 6.6, C(16)H_2), 2.96 – 2.90 (2H, m, *J* 7.0, C(9)H_2), 1.63 – 1.51 (4H, m, C(10+15)H_2), 1.40 – 1.32 (8H, m, C(11+12+13+14)H_2); δ_C (126 MHz; $CDCl_3$) 168.4 (C, C2), 144.8 (C, C8), 131.9 (CH, C7), 131.0 (CH, C6), 130.7 (CH, C4), 129.6 (C, C3), 125.80 (CH, C5), 63.2 (CH_2, C16), 52.0 (CH_3, C1), 34.6 (CH_2, C9), 32.9 (CH_2, C14), 31.9 (CH_2, C10), 29.8 (CH_2, C13), 29.5 (CH_2, C12), 29.4 (CH_2, C11), 25.8 (CH_2, C15); HRMS (ESI$^+$): m/z calc. for $C_{16}H_{24}NaO_3$: 287.1618, found: 287.1619 $[MNa]^+$.

2-(8-Hydroxyoctyl)benzoic acid (133)

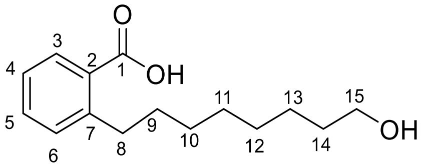

$NaOH_{(aq)}$ (4.0 M, 26 mL) was added dropwise over a period of 5 min to clear colourless solution of methyl 2-(8-hydroxyoctyl)benzoate **S181** (1.39 g, 5.24 mmol) in MeOH (26 mL). A colour change to cloudy pale-yellow suspension was immediately noted, upon further addition another colour change to milky/white suspension was observed. The resulting suspension was then heated to 60 °C under Ar for 16 h. After that time the reaction was deemed to have gone to completion by TLC analysis, with a colour change to colourless solution. The reaction mixture was then acidified to pH 2.0 with 1M $HCl_{(aq)}$ (20 mL). The acidified cloudy/white suspension was poured into separating funnel and aqueous layer was extracted with CH_2Cl_2 (3 × 30 mL), before combined organic layers were dried over $MgSO_4$, filtered and concentrated under reduced pressure to yield title compound **133** as a colourless viscous oil (1.21 g, 92%); R_f = 0.77 (50:50 MeOH:EtOAc:); IR (neat) ν_{max} / cm^{-1}: 3383wbr (O–H alcohol), 2927wbr (C–H alkyl), 2855wbr (C–H alkyl), 2637mbr (O–H carboxylic acid), 1689vs (C=O carboxylic acid), 1602w (CC aromatic), 1575w (CC aromatic), 1488w (CC aromatic), 1455w, 1403w, 1297m, 1263m, 1164w, 1139w, 1098w, 1070w, 1049w, 937w, 839w, 804w, 738s, 706m, 649m, 550w; δ_H (500 MHz; CD_2Cl_2) 7.98 (1H, dd, *J* 7.7, 1.5, C(3)H), 7.69 (2H, s, br, OH), 7.45 (1H, td, *J* 7.7, 1.5, C(5)H), 7.30 – 7.23 (2H, m, C(4+6)H), 3.65 (2H, t, *J* 6.7, C(15)H_2), 3.04 – 2.97 (2H, m, C(8)H_2), 1.66 – 1.53 (4H, m, C(9+10)H_2) 1.44 – 1.29 (8H, m, C(11+12+13+14)H_2); δ_C (126 MHz; CD_2Cl_2) 172.6 (C, C1), 146.0 (C, C7), 132.9 (CH, C5), 131.7 (CH, C3), 131.5 (CH, C6), 129.2 (C, C2), 126.2 (CH, C4), 63.1 (CH_2, C15), 34.9 (CH_2, C8), 32.9 (CH_2, C13), 32.2 (CH_2, C9), 30.0 (CH_2, C10), 29.7 (CH_2, C12), 29.6 (CH_2, C11), 26.1 (CH_2, C14); HRMS (ESI^+): m/z calc. for $C_{15}H_{22}NaO_3$: 273.1461, found: 273.1466 $[MNa]^+$.

Synthesis of 1H-benzo[d][1,2,3]triazol-1-yl 2-(8-hydroxyoctyl)benzoate (135) and 7,8,9,10,11,12,13,14,21,22,23,24,25,26,27,28-hexadecahydro-5H,19H-dibenzo[c,o][1,13]dioxacyclotetracosine-5,19-dione (136)

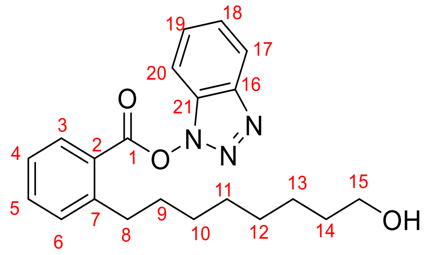

135

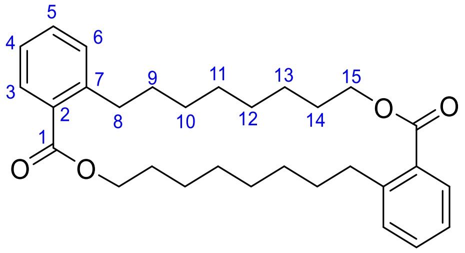

136

EDC.HCl (612 mg, 3.19 mmol) was added to colourless solution of 2-(8-hydroxyoctyl)benzoic acid **133** (532 mg, 2.13 mmol), HOBt (345 mg, 2.56 mmol) and dry DIPEA (930 µL, 5.33 mmol) in anhydrous DMF (21 mL). A colour change to pale yellow was noted after addition of DIPEA via syringe over a period of 30 sec. The resulting mixture was stirred at RT overnight under Ar and progress of the reaction was monitored by TLC. After total of 18 h, the reaction was judged to be complete, based on TLC analysis. The resulting mixture was diluted with EtOAc (40 mL), before was transferred to separating funnel and the organic phase was washed sequentially with 1 M $HCl_{(aq)}$ (3 × 20 mL), sat. $NaHCO_{3\ (aq)}$ (3 × 20 mL) and sat. brine (3 × 20 mL). The resulting organic layer was dried over $MgSO_4$, filtered and concentrated under reduced pressure to yield a white solid (838 mg). The crude product was purified by flash column chromatography (SiO_2, 20 mm column, eluent: EtOAc:*n*–hexane, 50:50) to afford HOBt adduct **135** (392 mg, 50%) as a colourless viscous oil and dimer **136** as a white solid, (127 mg, 26%)

Data for **135.** R_f = 0.67 (50:50 EtOAc:*n*–hexane); IR (neat) ν_{max} / cm^{-1}: 3381sbr (O–H alcohol), 2927m (C–H alkyl), 2855w, (C–H alkyl), 1797m (C=O ester), 1601w (CC aromatic), 1574ww (CC aromatic), 1486m (CC aromatic), 1445w, 1375w, 1281w, 1268w, 1155w, 1217s, 1088m, 1053m, 963s, 917m, 810m, 781m, 739s, 698m, 664m, 637m, 606w, 500w; δ_H (500 MHz; $CDCl_3$) 8.29 (1H, dd, *J* 8.1, 1.4, C(3)H), 8.08 (1H, dd, *J* 8.1, 1.4, C(6)H), 7.61 (1H, td, *J* 8.1, 1.4, C(5)H), 7.58 – 7.51 (1H, m, C(4)H), 7.50 – 7.36 (5H, m, C(17+18+19+20+21)H), 3.57 (2H, t, *J* 6.7, C(15)H_2), 3.01 – 2.94 (2H, m, C(8)H_2), 1.65 – 1.56 (2H, m, C(9)H_2), 1.55 – 1.45 (2H, m, C(14)H_2), 1.38 – 1.21 (8H, m, C(10+11+12+13)H_2); δ_C (126 MHz; $CDCl_3$) 162.8 (C, C1), 147.6 (C, C7), 143.6 (C, C16), 134.7 (CH, C4), 131.7 (CH, C3), 131.5 (CH, C6), 128.9 (C, C2), 128.8 (CH,

C5), 126.5 (CH, C19), 124.9 (CH, C18), 123.3 (C, C21), 120.6 (CH, C17), 108.4 (CH, C20), 62.9 (CH_2, C15), 34.6 (CH_2, C8), 32.8 (CH_2, C14), 31.6 (CH_2, C9), 29.5 (CH_2, C11), 29.3 (CH_2, C12), 29.3 (CH_2, C10), 25.7 (CH_2, C13); HRMS (ESI$^+$): m/z calc. for $C_{21}H_{26}N_3O_3$: 368.1969, found: 368.1970 $[MH]^+$, m/z calc. for $C_{21}H_{25}NaN_3O_3$: 390.1788, found: 390.1786 $[MNa]^+$, m/z calc. for $C_{21}H_{25}KN_3O_3$: 406.1527, found: 406.1527 $[MK]^+$;

Data for **136.** R_f = 0.22 (50:50 EtOAc:*n*–hexane); Melting Point: 110– 112 °C (from *n*-hexane); IR (solid state) ν_{max} / cm $^{-1}$: 2920w (C–H alkyl), 2848m (C–H alkyl), 1699vs (C=O aryl ester), 1601w (CC aromatic), 1485m (CC aromatic), 1449m, 1463m, 1382w, 1308w, 1285m, 1269s, 1260m, 1246m, 1134m, 1100s, 1067m, 1047m, 991w, 964w, 934w, 867w, 802w, 754s, 723w, 707s, 659w, 601w, 534w, 511w; δ_H (500 MHz; $CDCl_3$) 7.78 (2H, dd, *J* 7.7, 1.5, C(3)H), 7.38 (2H, td, *J* 7.7, 1.5 C(5)H), 7.26 – 7.19 (4H, m, C(4+6)H), 4.32 (4H, t, *J* 6.4, C(15)H_2), 2.92 – 2.85 (4H, m, C(8)H_2), 1.79 – 1.70 (4H, m, C(14)H_2), 1.60 – 1.51 (4H, m, C(9)H_2), 1.47 – 1.28 (16H, m, C(10+11+12+13)H_2); δ_C (126 MHz; $CDCl_3$) 169.2 (C, C1), 143.5 (C, C7), 131.6 (CH, C5), 131.0 (CH, C6), 130.9 (C, C2), 130.7 (CH, C3), 125.9 (CH, C4), 65.4 (CH_2, C15), 35.0 (CH_2, C8), 32.8 (CH_2, C9), 30.3 (CH_2, C10), 29.9 (CH_2, C11), 29.8 (CH_2, C12), 29.1 (CH_2, C14), 26.6 (CH_2, C13); HRMS (ESI$^+$): m/z calc. for $C_{30}H_{41}O_4$: 465.2999, found: 465.2970 $[MH]^+$, m/z calc. for $C_{30}H_{40}NaO_4$: 487.2819, found: 487.2831 $[MNa]^+$.

X-ray crystallographic data for compound **136** can be accessed via www.ccdc.cam.ac.uk/data_request/cif (CCDC 2237214).

4,4'-(1,2-Phenylene)bis(but-3-yn-1-ol) (S182)

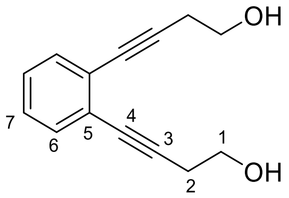

But-3-yn-1-ol (361 µL, 4.80 mmol) was added to solution of methyl 1,2-diiodobenzene (250 µL, 1.91 mmol) in anhydrous Et_3N (19.0 mL, degassed for 15 min) at RT under Ar. After 10 min, CuI (10.9 mg, 0.057 mmol) and bis(triphenylphosphine)palladium chloride (67.0 mg, 0.096 mmol) was added to the pale-yellow solution. The resulting reaction mixture was stirred at RT under Ar. After a total of 24 h, the reaction was deemed to have gone to completion by

TLC. The dark brown reaction mixture was filtered through Celite and washed with EtOAc (3 × 40 mL). The resulting filtrate was concentrated under *vacuo* to yield an orange oil (871 mg). The crude product was purified by flash column chromatography (SiO_2, 40 mm column, eluent: EtOAc:*n*–hexane, 70:30 to 100:0) to afford the title compound **S182** as a pale yellow oil (403 mg, 98%). R_f = 0.22 (70:30 EtOAc:*n*-hexane); IR (neat) ν_{max} / cm^{-1}: 3341brm (O–H alcohol), 3064w (C–H alkyl), 2898w (C–H alkyl), 2887w (C–H alkyl), 2238w (CC alkynyl), 1727w, 1480m, 1443w, 1265w, 1038s, 846w, 757s, 733s; δ_H (400 MHz; $CDCl_3$) 7.35 (2H, dd, *J* = 5.8, 3.4 Hz, 2 × C(6)H), 7.15 (2H, dd, *J* = 5.8, 3.4 Hz, 2 × C(7)H), 3.76 (4H, t, *J* = 6.2 Hz, C(1)H_2), 3.69 (2H, s, br, OH), 2.66 (4H, t, *J* = 6.1 Hz, C(2)H_2); δ_C (101 MHz; $CDCl_3$) 131.7 (2 × CH, C6), 127.6 (2 × CH, C7), 125.8 (2 × C, C5), 90.9 (2 × C, C3), 81.3 (2 × C, C4), 60.7 (2 × CH_2, C1), 23.8 (2 × CH_2, C2); HRMS (ESI): calcd. for $C_{14}H_{15}O_2$, 215.1067. Found: $[MH]^+$, 215.1064 (1.0 ppm error), calcd. for $C_{14}H_{14}NaO_2$, 237.0886. Found: $[MNa]^+$, 237.0883 (1.4 ppm error), calcd. for $C_{14}H_{14}KO_2$, 253.0625. Found: $[MNa]^+$, 253.0618 (3.1 ppm error).

4,4'-(1,2-Phenylene)bis(butan-1-ol) (137)

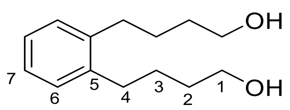

A round bottom flask containing 4,4'-(1,2-phenylene)bis(but-3-yn-1-ol) **S182** (448 mg, 2.09 mmol) was purged with argon for 10 min, before Pd/C (22.2 mg, Pd 10% on carbon) was added, and the flask purged with argon for an additional 10 min . Methanol (21.0 mL, degassed with argon for 10 min) was then added, and the reaction vessel was evacuated under vacuum and then backfilled with H_2 (via balloon) three times, then stirred at RT under slight positive atmosphere of H_2 (via balloon) for 72 h. The reaction was then purged with Ar for 10 min, filtered through Celite and washed with MeOH (3 × 20 mL). The resulting filtrate was concentrated under reduced pressure to yield crude product as a colourless oil (510 mg). The crude product was purified by flash column chromatography (SiO_2, 40 mm column, eluent: EtOAc:*n*–hexane, 95:5 to 100:0) to afford the title compound **137** as a colourless viscous oil (373 mg, 80%). R_f = 0.39 (EtOAc); IR (neat) ν_{max} / cm^{-1}: 3338brw (O–H alcohol), 2935w (C–H alkyl), 2864w (C–H alkyl),1726w, 1490w, 1451w, 1374w, 1243w, 1114w, 1045s, 980w, 934w, 749s, 666w, 608w, 464w; δ_H (400 MHz; $CDCl_3$) 7.19 – 7.09 (4H, m, C(6+7)H), 3.66 – 3.58 (4H, m, C(1)H_2), 3.37 (2H, s, OH), 2.69 – 2.60 (4H, m, C(4)H_2), 1.72 – 1.61 (8H, m,

C(2+3)H$_2$); δ$_C$ (101 MHz; CDCl$_3$) 140.1 (2 × C, C5), 129.2 (2 × CH, C6), 125.9 (2 × CH, C7), 62.2 (2 × CH$_2$, C1), 32.5 (2 × CH$_2$, C3), 32.3 (2 × CH$_2$, C4), 27.5 (2 × CH$_2$, C2); HRMS (ESI): calcd. for $C_{14}H_{23}O_2$, 223.1693. Found: [MH]$^+$, 223.1693 (0.0 ppm error), calcd. for $C_{14}H_{22}NaO_2$, 245.1512. Found: [MNa]$^+$, 245.1512 (−0.0 ppm error), calcd. for $C_{14}H_{22}KO_2$, 261.1251. Found: [MK]$^+$, 261.1239 (4.9 ppm error).

1,2,3,4,8,9,10,11-Octahydrobenzo[h][1,3]dioxa[2]thiacyclotridecine 6-oxide (138)

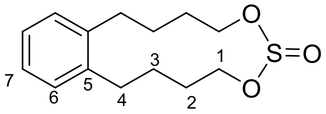

Thionyl chloride (109 µL, 1.50 mmol) was added in a single portion via syringe to a colourless solution of 4,4'-(1,2-phenylene)bis(butan-1-ol) **137** (257 mg, 1.16 mmol), DMAP (496 mg, 4.06 mmol) in anhydrous CH_2Cl_2 (23.0 mL) at 0 °C under Ar. The resulting mixture was allowed to warm gradually to RT overnight under Ar and the progress of the reaction was monitored *via* TLC, with a colour change to a pale yellow solution observed. After a total of 18 h, the reaction was deemed to have gone to completion by TLC analysis and a colour change to a yellow suspension was noted. The resulting mixture was concentrated under reduced pressure to yield a yellow oil (876 mg). The crude product was purified by flash column chromatography (SiO_2, 40 mm column, eluent: EtOAc:*n*–hexane, 10:90 to 20:80) to afford the title compound **138** as a pale yellow oil (15.5 mg, 5%); R$_f$ = 0.27 (10:90 EtOAc:*n*-hexane); IR (neat) ν$_{max}$ / cm^{-1}: 2939m (C–H alkyl), 2866w (C–H alkyl), 1489w (CC aromatic), 1463w, 1200s (S=O sulfite), 1089w, 1054w, 1029w, 924w, 886s, 865m, 851w, 826s, 774w, 738s, 702s, 613w, 584m, 500w, 472w; *δ*$_H$ (400 MHz; CDCl$_3$) 7.16 (4H, s, C(6+7)H), 4.40 – 4.31 (2H, m, 2 × C(1)H**H'**), 4.14 – 4.04 (2H, m, 2 × C(1)**H**H'), 2.81 – 2.69 (2H, m, 2 × C(4)H**H'**), 2.69 – 2.56 (2H, m, 2 × C(4)**H**H') 1.95 – 1.83 (4H, m, 2 × C(2)H$_2$), 1.83 – 1.71 (4H, m, 2 × C(3)H$_2$); δ$_C$ (101 MHz; CDCl$_3$) 140.2 (2 × C, C5), 129.9 (2 × CH, C6), 126.3 (2 × CH, C7), 61.8 (2 × CH$_2$, C1), 31.9 (2 × CH$_2$, C4), 28.2 (2 × CH$_2$, C2), 27.6 (2 × CH$_2$, C3); HRMS (ESI): calcd. for $C_{14}H_{20}NaO_3S$, 291.1025. Found: [MH]$^+$, 291.1023 (0.8 ppm error).

9-Phenyl-4,5-dihydro-3*H*-benzo[*g*][1,5]oxathionin-1(7*H*)-one (139)

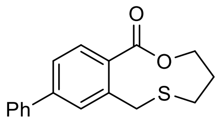

To a solution of 9-bromo-4,5-dihydro-3*H*-benzo[*g*][1,5]oxathionin-1(7*H*)-one **49b** (19 mg, 0.0662 mmol) in degassed 1,4-dioxane (1.1 mL) was added phenylboronic acid (16 mg, 0.132 mmol), sodium carbonate (21 mg, 0.199 mmol), water (0.51 mL) and Pd(dppf)Cl_2·DCM (3 mg, 3.31 µmol) and the resulting solution was stirred at 50 °C for 18 h. Water (10 mL) was added and the mixture was extracted with DCM (3 x 10 mL). The combined organic layers were dried over $MgSO_4$, filtered and concentrated *in vacuo*. The crude product was purified by column chromatography (SiO_2, 8:2 hexane:ethyl acetate) to yield the title compound **139** (11 mg, 58%) as a white solid. R_f 0.42 (8:2 hexane:ethyl acetate); δ_H (400 MHz, $CDCl_3$) 7.95 (1H, d, *J* = 8.0 Hz, ArH), 7.62–7.53 (3H, m, ArH), 7.49–7.43 (2H, m, ArH), 7.41–7.36 (2H, m, ArH), 4.71–4.61 (2H, m, OCH_2), 4.22 (2H, s, $ArCH_2$), 2.94–2.89 (2H, m, SC**H**$_2$CH$_2$), 2.24–2.17 (2H, m, SCH$_2$C**H**$_2$); δ_C (101 MHz, $CDCl_3$) 168.0 (CO), 144.5 (ArC), 142.8 (ArC), 140.0 (ArC), 132.0 (ArC), 130.4 (ArC), 129.0 (ArC), 128.9 (ArC), 128.2 (ArC), 127.4 (ArC), 126.2 (ArC), 66.0 (OCH_2), 41.3 ($ArCH_2$), 35.3 (S**C**H$_2$CH$_2$), 30.4 (SCH$_2$**C**H$_2$); ν_{max}/cm^{-1} (thin film) 3031, 2956, 1714, 1607, 1483, 1455, 1422, 1397, 1377, 1287, 1265, 1212, 1195, 1129, 1091, 975, 908, 844, 759, 698; HRMS (ESI); calcd. for $C_{17}H_{16}O_2SNa^+$, 307.0763. Found: $[MNa]^+$, 307.0765 (–0.5 error ppm).

Methyl 2-((butylthio)methyl)benzoate (S183)

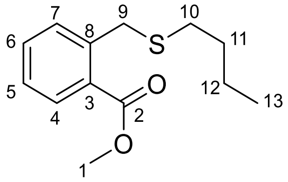

K_2CO_3 (1.04 g, 7.42 mmol) was added to a clear colorless solution of butane-1-thiol (840 µL, 7.42 mmol) and methyl 2-(bromomethyl)benzoate (880 mg, 3.71 mmol) in anhydrous DMF (12 mL) at RT. The resulting grey/purple suspension was then heated to 70 °C, whereupon a colour change to rose pink was noted. Upon further heating, the colour of the reaction suspension changed to peace orange. After 12 h, the reaction was deemed to have gone to completion by TLC. The reaction mixture was cooled to RT, before diluted with H_2O (30 mL) and poured into a separating funnel containing EtOAc (20 mL). The phases where separated and the aqueous layer was extracted with EtOAc (4 × 20 mL). The combined organic layers

were washed sequentially with 1.0 M $HCl_{(aq)}$ (2 × 20 mL), 1.0 M $NaOH_{(aq)}$ (2 × 20 mL), sat. $NaHCO_{3(aq)}$ (2 × 20 mL) and sat. brine (1 × 20 mL), before being dried over $MgSO_4$, filtered and concentrated under reduced pressure to yield a pale orange oil (1.02 g). The crude product was purified by flash column chromatography (SiO_2, 50 mm column, eluent: EtOAc:*n*–hexane, 10:90) to afford the title compound **S183** as a pale yellow oil (817 mg, 92%); R_f = 0.67 (20:80 EtOAc:*n*–hexane);IR (thin film) ν_{max}/cm^{-1}: 2954m (C–H alkyl), 2930m (C–H alkyl), 2872w (C–H alkyl), 1719s (C=O aryl ester), 1600w (CC aromatic), 1576w (CC aromatic), 1488m (CC aromatic), 1434w, 1379w, 1291w, 1260s, 1190m, 1164w, 1122m, 1077s, 1046w, 966w, 9120w, 839w, 767m, 714m, 663m, 580w, 474w; δ_H (400 MHz; $CDCl_3$) 7.90 (1H, dd, *J* = 7.7, 1.5 Hz, C(4)H), 7.43 (1H, td, *J* = 7.7, 1.5 Hz, C(6)H), 7.36 – 7.27 (2H, m, C(5+7)H), 4.11 (2H, s, C(9)H_2), 3.91 (3H, s, C(1)H_3), 2.42 (2H, t, *J* = 7.5 Hz, C(10)H_2), 1.58 – 1.46 (2H, m, C(11)H_2), 1.44 – 1.29 (2H, m, C(12)H_2), 0.87 (3H, t, *J* = 7.3 Hz, C(13)H_3); δ_C (101 MHz; $CDCl_3$) 167.9 (C, C2), 140.8 (C, C8), 131.7 (CH, C7), 132.1 (CH, C4), 130.9 (CH, C6), 129.6 (C, C3), 126.9 (CH, C5), 52.1 (CH_3, C1), 34.5 (CH_2, C9), 31.5 (CH_2, C10), 31.4 (CH_2, C11), 22.0 (CH_2, C12), 13.7 (CH_3, C13); HRMS (ESI): m/z calcd. for $C_{13}H_{18}NaO_2S$: 261.0920 found: 261.0912 $[MNa]^+$.

2-((Butylthio)methyl)benzoic acid (S184)

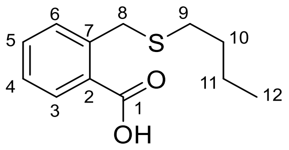

$NaOH_{(aq)}$ (6.0 M, 17 mL) was added dropwise over a period of 3 min to solution of methyl 2-((butylthio)methyl)benzoate **S183** (816 mg, 3.42 mmol) in MeOH (17 mL) at RT. A colour change to milky/white suspension was immediately noted. The resulting mixture was then heated to 70 °C, whereupon a colour change to pale yellow was noted. After 48 h, the reaction mixture was deemed to have gone to completion by TLC (complete consumption of methyl 2-((butylthio)methyl)benzoate **S183** was noted). The reaction mixture was then cooled to 0 °C, before acidified to pH 2.0 with 3.0 M $HCl_{(aq)}$ (60 mL). The resulting white solid was collected by suction filtration and washed with cold H_2O (4 × 20 mL) and then air dried to yield title compound **S184** as a white solid (691 mg, 90%); R_f = 0.55 (15:85, MeOH:CH_2Cl_2);melting point: 77 – 78 °C (from H_2O); IR (solid state) ν_{max}/cm^{-1}: 3496m (O–H alcohol), 2955m (C–H alkyl, O–H carboxylic acid), 2916w (C–H alkyl, O–H carboxylic acid), 2857m (C–H alkyl, O–H carboxylic acid), 2642m (C–H alkyl, O–H carboxylic acid), 1675s (C=O aryl carboxylic acid), 1597w (CC

aromatic),1575s (CC aromatic), 1491w, 1466w, 1448w, 1426w, 1405m, 1300m, 1272s, 1220w, 1198w, 1168w, 1141w, 1080m, 1052w, 966w, 906m, 867w, 841w, 766m, 749m, 724w, 708s, 681w; δ_H (400 MHz; CD_3OD) 7.89 (1H, dd, *J* = 7.7, 1.5 Hz, C(3)H), 7.45 (1H, td, *J* =7.7, 1.5 Hz, C(5)H), 7.37 (1H, d, *J* = 7.7 Hz, C(5)H) , 7.32 (1H, td, *J* = 7.6, 1.6 Hz, C(4)H), 4.13 (2H, s, C(8)H_2), 2.40 (2H, t, *J* = 7.3 Hz, C(9)H_2), 1.55 – 1.42 (2H, m, C(10)H_2), 1.42 – 1.27 (2H, m, C(11)H_2), 0.87 (3H, t, *J* = 7.3 Hz, C(12)H_3); δ_C (101 MHz; $CDCl_3$) 173.1 (CO, C1), 142.0 (C, C7), 132.9 (CH, C6), 132.3 (CH, C5), 131.3 (CH, C3), 128.3 (C, C2), 127.2 (CH, C4), 34.7 (CH_2, C8), 31.7 (CH_2, C), 31.6 (CH_2, C), 22.1 (CH_2, C), 13.8 (CH_3, C12); HRMS (ESI): m/z calcd. for $C_{12}H_{16}NaO_2S$: 247.0763, found: 247.0767 $[MNa]^+$.

***tert*-Butyl (3-(benzylamino)propyl)carbamate (S185)**

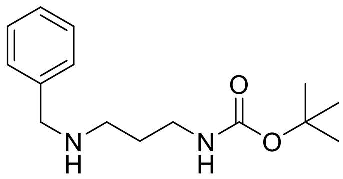

A round-bottomed flask was charged with methanol (20.0 mL), benzaldehyde (1.79 mL, 17.6 mmol) and *tert*-butyl *N*-(3-aminopropyl)carbamate (2.02 mL, 11.6 mmol). The solution was heated to reflux for 3 days before being cooled to room temperature. Sodium borohydride (666 mg, 17.6 mmol) was then added portion-wise and the solution stirred for 1.5 h. The reaction was quenched with ammonium chloride solution (100 mL) and extracted with ethyl acetate (60 mL) and DCM (100 mL). The organic layer was dried over magnesium sulfate, filtered, and concentrated *in vacuo* to give the product. Purification by flash column chromatography (SiO_2, 100% hexane → 9:1 hexane : ethyl acetate → 8:2 hexane : ethyl acetate) afforded the title compound **S185** as a colourless oil (2.76 g, 90%); R_f = 0.50 (20:1 ethyl acetate : triethylamine); ν_{max}/cm^{-1} (thin film): 3335, 2976, 2931, 1691, 1512, 1453, 1365, 1272, 1250, 1167, 735, 698; δ_H (400 MHz, $CDCl_3$) 7.14 – 7.32 (5H, m, 5 x Ar**H**), 5.50 (1H, br s, N**H**CO), 3.71 (2H, s, PhC**H₂**N), 3.16 (2H, q, *J* = 6.0 Hz, C**H₂**NHBoc), 2.64 (2H, t, *J* = 6.5 Hz, C**H₂**NHBn), 1.61 (2H, p, *J* = 6.5 Hz, C**H₂**CH_2NHBoc), 1.41 (9H, s, tert-butyl); δ_C (100 MHz, $CDCl_3$), 156.1 (**C**O), 140.2 (Ar**C**), 128.3 (2 x Ar**C**H), 128.1 (2 x Ar**C**H), 126.9 (Ar**C**CH_2N), 78.7 (**C**$(CH_3)_3$), 53.9 (Ph**C**H_2N), 47.2 (**C**H_2NHBn), 39.3 (**C**H_2NHBoc), 29.6 (**C**H_2CH_2NHBoc), 28.4 (tert-butyl); HRMS (ESI^+): calcd. for $C_{15}H_{25}N_2O_2$, 265.1911. Found: $[MH]^+$, 265.1914 (−1.5 ppm error).

Methyl 4-(benzyl(3-((*tert*-butoxycarbonyl)amino)propyl)amino)butanoate (S186)

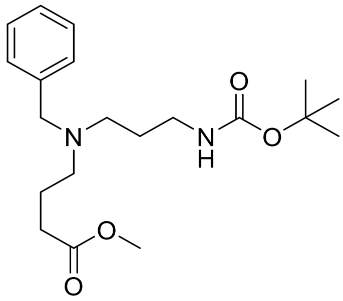

tert-Butyl (3-(benzylamino)propyl)carbamate **S185** (2.12 g, 8.01 mmol) was dissolved in dry acetonitrile (35.0 mL), after which potassium carbonate (1.67 g, 12.1 mmol) and sodium iodide (245 mg, 1.63 mmol) were added. Methyl 4-bromobutyrate (1.10 mL, 8.81 mmol) was then added and the solution heated to reflux for 16 hours. The solution was cooled to room temperature and diluted with ethyl acetate (80 mL), washed with sodium bicarbonate solution (80 mL) and back-extracted with ethyl acetate (80 mL). The organic phases were combined and dried over magnesium sulfate, filtered and concentrated *in vacuo*. The product was purified by column chromatography (SiO_2, 9:1 hexane : ethyl acetate → 7:3 ethyl acetate : hexane) to yield the title product **S186** as a colourless oil (2.27 g, 78%); R_f = 0.27 (1:1 ethyl acetate : hexane); ν_{max}/cm^{-1} (thin film): 3375, 2950, 2807,1737, 1713, 1512, 1453, 1365, 1249, 1171, 736, 700; δ_H (400 MHz, $CDCl_3$) 7.21 – 7.34 (5H, m, Ar**H**), 5.28 (1H, br s, N**H**), 3.63 (3H, s, OC**H₃**), 3.51 (2H, s, PhC**H₂**N), 3.13 (2H, q, *J* = 6.0 Hz, C**H₂**NHBoc), 2.46 (2H, t, *J* = 6.5 Hz, C**H₂**CH_2CH_2NHBoc), 2.41 (2H, t, *J* = 7.0 Hz, C**H₂**$CH_2CH_2CO_2CH_3$), 2.31 (2H, t, *J* = 7.5 Hz, C**H₂**CO_2CH_3), 1.79 (2H, p, *J* = 7.0 Hz, C**H₂**$CH_2CO_2CH_3$), 1.62 (2H, p, *J* = 6.5 Hz, C**H₂**CH_2NHBoc), 1.43 (9H, s, OC(C**H₃**)₃); δ_C (101 MHz, $CDCl_3$) 174.2 (**C**O_2CH_3), 156.1 (**C**OC$(CH_3)_3$), 139.4 (Ar**C**CH_2N), 129.0 (2 x Ar**C**H), 128.4 (2 x Ar**C**H), 127.1 (Ar**C**H), 78.9 (**C**$(CH_3)_3$), 58.8 (**C**H_2PhN), 53.0 (**C**$H_2CH_2CH_2CO_2CH_3$), 52.2 (**C**$H_2CH_2CH_2$NHBoc), 51.6 (O**C**H_3), 39.7 (**C**H_2NHBoc), 31.8 (**C**$H_2CO_2CH_3$), 28.6 (C(**C**H_3)₃), 26.7 (**C**H_2CH_2NHBoc), 22.4 (**C**$H_2CH_2CO_2CH_3$); HRMS (ESI^+): calcd. for $C_{20}H_{33}N_2O_4$, 365.2435. Found $[MH]^+$ 365.2439 (−1.3 ppm error).

1-Benzyl-1,5-diazonan-6-one (140)

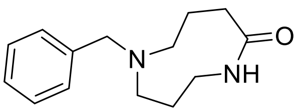

To a stirring solution of **S186** (999 mg, 2.60 mmol) in dichloromethane (20 mL), 4N hydrochloric acid in dioxane (12.30 mL, 49.2 mmol) was added and stirred at room temperature for 24 hours. The reaction was then concentrated under vacuum, azeotroping with diethyl ether to ensure dryness. The residue was redissolved in dichloromethane (49 mL), before the addition of diisopropyl ethylamine (2.14 mL, 12.3 mmol) and T3P (1.76 mL, 3.0 mmol). The solution was stirred at RT for 16 hours under an argon atmosphere. The reaction was diluted with water (100 mL), extracted with DCM (3 × 100 mL), the combined organic layers dried with sodium sulfate, filtered, concentrated under vacuum. The crude was purified via flash column chromatography (dichloromethane 3:2 diethyl ether → ethyl acetate 9:1 methanol) to afford the title compound **140** as a white solid (494 mg, 2.1 mmol, 87%). R_f 0.32 (19:1 ethyl acetate:triethyl amine). ν_{max} (thin film)/cm^{-1} 3284, 3206, 2931, 2796, 2229, 1646, 1452, 1373, 1340, 1291, 1134, 908, 723, 698. ^{1}H NMR (400 MHz, $CDCl_3$) δ 7.30 – 7.14 (5H, m, ArH), 6.73 (1H, m, NH), 3.54 (2H, s, $PhCH_2$), 3.58 – 3.43 (2H, s (Br), $NHCH_2$), 2.56 – 2.38 (6H, m, NCH_2 + NCH_2 + $COCH_2$), 1.79 (2H, tt, *J* = 6.5 Hz, $CH_2CH_2CH_2$), 1.44 (2H, tt, *J* = 5.5 Hz, $CH_2CH_2CH_2$). ^{13}C NMR (400 MHz, $CDCl_3$) δ 178.3 (CO), 139.5 (ArC), 128.8 (ArC), 128.2 (ArC), 126.8 (ArC), 59.4 ($PhCH_2$), 51.6 (NCH_2), 47.9 (NCH_2), 40.1 ($NHCH_2$), 28.6 ($CH_2CH_2CH_2$), 27.6 ($COCH_2$), 24.5 ($CH_2CH_2CH_2$). HRMS (ESI$^+$) $C_{14}H_{21}N_2O$ (MH$^+$) theoretical 233.1648; measured 233.1647 (-0.4 ppm error); $C_{14}H_{20}N_2NaO$ (MNa$^+$) theoretical 255.1468; measured 255.1464 (-0.1 ppm error); $C_{14}H_{20}KN_2O$ (MK$^+$) theoretical 271.1207; measured 271.1198 (–0.7 ppm error).

***N*-(3-(2-Oxopyrrolidin-1-yl)propyl)furan-3-carboxamide (142)**

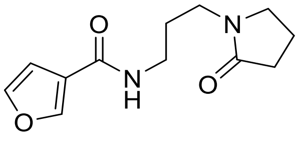

A solution of 1-benzyl-1,5-diazonan-6-one **140** (1.00 g, 4.3 mmol), palladium hydroxide (59.6 mg, 2.6 mol%) in methanol (0.50 mL) and ethyl acetate (2.50 mL) was degassed under vacuum, after which the reaction was placed under a hydrogen atmosphere. 4N HCl in dioxane (1.60 mL, 6.4 mmol) was added to the reaction mixture and stirred at room

temperature for 16 hours. The reaction mixture was then filtered through celite, washed with methanol. The filtrate was concentrated under vacuum and the residue (predominantly 1-(3-aminopropyl)pyrrolidin-2-one **141b**) was redissolved in dichloromethane (4.00 mL).

Next, a solution of 3-furoyl chloride was prepared as described: To a stirring suspension of 3-Furoic acid (581 mg, 5.2 mmol) in dichloromethane (4 mL), oxalyl chloride (1.30 mL, 15.4 mmol) and DMF (1 drop) was added and stirred at room temperature for 30 minutes. The solvent was removed under vacuum to afford a yellow solid. The solid was redissolved in dichloromethane (4.00 mL) and added directly to the solution of **141b** described above. Diisopropyl ethylamine (2.20 mL, 12.6 mmol) was added and the reaction was stirred at room temperature for 16 hours. The reaction mixture was diluted with water (100 mL), the aqueous layer was extracted with dichloromethane (3 × 100 mL). The combined organic layer was dried with sodium sulfate, filtered and concentrated in vacuo and purified via flash column chromatography to afford the title compound **142** as a yellow oil (963 mg, 4.1 mmol, 94%). R_f 0.12 (19:1 ethyl acetate:methanol). ν_{max} (thin film)/cm^{-1} 3301, 3129, 2934, 2872, 1635, 1587, 1537, 1437, 1319, 1292, 1266, 1202, 1160, 1083, 1018, 875, 828, 756, 602. ^{1}H NMR (400 MHz, $CDCl_3$) δ 7.95 (1H, s, ArH), 7.60 (1H, s (Br), NH), 7.33 (1H, s, ArH), 6.73 (1H, s, ArH), 3.37 – 3.29 (4H, m, NCH_2 + $COCH_2$), 3.25 (2H, dt, *J* = 6.0, 6.0 Hz, $PhCH_2$), 2.36 (2H, t, *J* = 8.0 Hz, NCH_2), 1.98 (2H, tt, *J* = 7.5, 7.5 Hz, $COCH_2CH_2$), 1.67 (2H, tt, *J* = 6.0, 6.0 Hz, $NHCH_2CH_2$). ^{13}C NMR (400 MHz, $CDCl_3$) δ 176.2 (CO), 162.7 (CO), 144.9 (ArC), 143.5 (ArC), 122.8 (ArC), 108.5 (ArC), 47.4 ($COCH_2$), 39.5 (NCH_2), 35.1 ($NHCH_2$), 30.9 (NCH_2), 26.1 ($NHCH_2CH_2$), 17.8 ($COCH_2CH_2$). HRMS (ESI$^+$) $C_{12}H_{16}N_2NaO_3$ (MNa^+) theoretical 259.1053; measured 259.1053 (–1.2 ppm error).

Dodecahydro-1*H*-pyrrolo[1,2-*e*][1,5]diazonine (143)

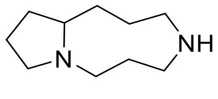

$LiAlH_4$ (2.4 M in THF, 2.13 mL, 5.10 mmol) was added dropwise to a solution of **72** (465 mg, 2.55 mmol) in THF (25.5 mL) at 0 °C and the resulting mixture heated at reflux for 4 h. The reaction was cooled to 0 °C and H_2O (194 μL), NaOH (15% in H_2O, 194 μL) and then H_2O (581 μL) were added. The mixture was warmed to rt, filtered through Celite® washing with THF, and the filtrate concentrated under reduced pressure. The residue was dissolved in Et_2O (5 mL) and then concentrated under reduced pressure (repeated 3 x) to give amine **143** as a

pale-yellow oil (397 mg, 92%); R_f ~ 0 (CH_2Cl_2/hexane/Et_3N 70:25:5); IR (thin film) ν_{max} / cm^{-1}: 3331br, 2910m, 1357w, 1141m, 924m, 727s, 639w; δ_H (400 MHz; $CDCl_3$) 3.05–2.92 (2H, m), 2.87–2.80 (1H, m), 2.79–2.70 (1H, m), 2.66–2.55 (2H, m), 2.51–2.35 (2H, m), 2.21–2.14 (1H, m), 1.83–1.70 (3H, m), 1.65–1.22 (7H, m); δ_C (101 MHz; $CDCl_3$) 64.0 (NCH), 57.9 (NCH_2), 56.9 (NCH_2), 50.6 (NCH_2), 50.5 (NCH_2), 35.5 (CH_2), 32.4 (CH_2), 28.4 (CH_2), 26.6 (CH_2), 23.6 (CH_2); HRMS (ESI^+) Calcd. for $C_{10}H_{21}N_2$ 169.1699; Found [MH^+] 169.1700 (–0.1 ppm error).

Dodecahydropyrido[1,2-*e*][1,5]diazonine (144)

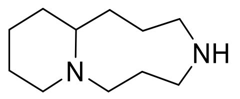

$LiAlH_4$ (2.4 M in THF, 1.76 mL, 4.23 mmol) was added dropwise to a solution of **73** (415 mg, 2.11 mmol) in THF at 0 °C and the resulting mixture heated at reflux for 4 h. The reaction was cooled to 0 °C and H_2O (160 μL), NaOH (15% in H_2O, 160 μL) and then H_2O (480 μL) were added. The mixture was warmed to rt, filtered through Celite® washing with THF, and the filtrate concentrated under reduced pressure. The residue was dissolved in Et_2O (5 mL) and then concentrated under reduced pressure (repeated 3 x) to give amine **144** as a pale-yellow oil (323 mg, 84%); R_f ~ 0 (CH_2Cl_2/Et_3N 19:1); IR (thin film) ν_{max} / cm^{-1}: 3346br, 2925s, 1334m, 1304m, 1270s, 1127m, 1000w; δ_H (400 MHz; $CDCl_3$) 3.08–2.95 (1H, br m, NH), 2.87–2.76 (3H, m), 2.72–2.65 (1H, m), 2.55–2.45 (1H, m), 2.24–2.00 (3H, m), 1.93–1.75 (2H, m), 1.66–1.59 (1H, m), 1.43–1.25 (8H, m), 1.25–1.10 (2H, m); δ_C (101 MHz; $CDCl_3$) 61.2 (NCH), 55.1 (NCH_2), 49.7 (NCH_2, broad), 48.8 (NCH_2), 44.0 (NCH_2), 31.0 (CH_2), 29.5 (CH_2), 28.0 (CH_2), 25.5 (CH_2. broad), 24.9 (CH_2), 21.5 (CH_2, broad); HRMS (ESI^+) Calcd. for $C_{11}H_{23}N_2$ 183.1856; Found [MH^+] 183.1858 (–1.4 ppm error).

Methyl 4-(benzyl(3-(vinylsulfonamido)propyl)amino)butanoate (S187)

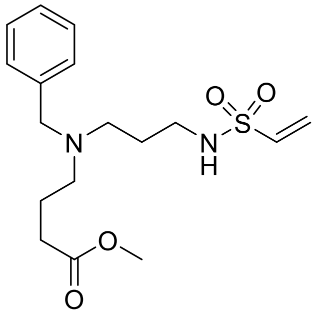

Methyl 4-(benzyl(3-((tert-butoxycarbonyl)amino)propyl)amino)butanoate **S186** (2.67 g, 7.33 mmol) was dissolved in 1,4-dioxane (45.0 mL) and 4 M HCl in 1,4-dioxane (15.0 mL, 432 mmol) was added. The solution was stirred at room temperature for 1.5 days, after which the solution was concentrated *in vacuo* to yield the salt product. This salt was dissolved in anhydrous DCM (45.0 mL) at room temperature and the solution was cooled to 0 °C using an ice bath. Triethylamine (10.1 mL, 72.6 mmol) was added dropwise, followed by 2-chloroethane-1-sulfonyl chloride (1.05 mL, 10.8 mmol). The solution was allowed to gradually warm to room temperature while being stirred for 4 days. The solution was diluted with DCM (45 mL) and washed with brine (2 x 45 mL). The aqueous layer was then washed with DCM (2 x 45 mL). The organic layers were then combined, dried over magnesium sulfate, filtered and concentrated *in vacuo* to give the crude product. This was purified by column chromatography (SiO_2, 1:1 ethyl acetate : hexane) to yield the title compound **S187** as a pale yellow oil (1.44 g, 56%). R_f = 0.24 (1:1 ethyl acetate : hexane); ν_{max}/cm^{-1} (thin film): 3291, 2951, 2815, 1732, 1453, 1436, 1326, 1256, 1146, 1076, 967, 733, 700, 658, 546; δ_H (400 MHz, $CDCl_3$) 7.20 – 7.37 (5H, m, 5 x Ar**H**), 6.37 (1H, dd, *J* = 16.5, 9.5 Hz, C**H**S), 6.15 (1H, d, *J* = 16.5 Hz, **H**C=CHS), 5.87 (1H, d, *J* = 9.5 Hz, **H**C=CHS), 3.64 (3H, s, OC**H₃**), 3.51 (2H, s, PhC**H₂**N), 3.00 (2H, t, *J* = 6.0 Hz, C**H₂**NHS), 2.52 (2H, t, *J* = 6.0 Hz, C**H₂**CH_2CH_2NHS), 2.40 – 2.47 (2H, m, C**H₂**$CH_2CH_2CO_2CH_3$), 2.30 (2H, t, *J* = 7.5 Hz, C**H₂**CO_2CH_3), 1.82 (2H, p, *J* = 7.5 Hz, C**H₂**$CH_2CO_2CH_3$), 1.69 (2H, p, *J* = 6.0 Hz, C**H₂**CH_2NHS); δ_C (100 MHz, $CDCl_3$) 173.9 (**C**O_2CH_3), 138.5 (Ar**C**CH_2N), 135.8 (H_2C=**C**HS), 129.2 (2 x Ar**C**H), 128.5 (2 x Ar**C**H), 127.3 (Ar**C**H), 126.3 (H_2**C**=CHS), 58.7 (Ph**C**H_2N), 52.8 (**C**$H_2CH_2CH_2CO_2CH_3$), 52.4 (**C**$H_2CH_2CH_2$NHS), 51.6 (O**C**H_3), 42.8 (**C**H_2NHS), 31.7 (**C**$H_2CO_2CH_3$), 25.7 (**C**H_2CH_2NHS), 22.0 (**C**$H_2CH_2CO_2CH_3$); HRMS (ESI$^+$): calcd. for $C_{17}H_{27}N_2O_4S$, 355.1686. Found $[MH]^+$ 355.1685 (0.2 ppm error).

Methyl 4-(benzyl(3-(vinylsulfonamido)propyl)amino)butanoic acid (145)

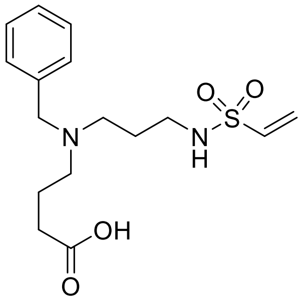

Methyl 4-(benzyl(3-(vinylsulfonamido)propyl)amino)butanoate **S187** (1.41 g, 4.00 mmol) was dissolved in THF (50.0 mL) and water (50.0 mL), and lithium hydroxide (1.01 g, 42.2 mmol) was added and the solution stirred at RT for 2 h. The solution was concentrated *in vacuo* to give the crude product. This was purified by column chromatography (SiO_2, 9:1 ethyl acetate : methanol → 7:3 ethyl acetate : methanol) to yield the title compound **145** as a colourless oil (1.08 g, 79%). R_f = 0.20 (1:1 ethyl acetate : methanol); ν_{max}/cm^{-1} (thin film): 2941, 1574, 1322, 1148, 1083, 968, 907, 725, 700, 647, 547; δ_H (400 MHz, $CDCl_3$) 8.71 – 10.04 (1H, br s, O**H**), 7.27 – 7.39 (5H, m, 5 x Ar**H**), 6.48 (1H, dd, *J* = 16.5, 10.0 Hz, C**H**S), 6.11 (1H, d, *J* = 16.5 Hz, **H**C=CHS), 5.84 (1H, d, *J* = 10.0 Hz, **H**C=CHS), 3.84 (2H, s, PhC**H₂**N), 2.97 (2H, t, *J* = 6.5 Hz, C**H₂**NHS), 2.79 (2H, t, *J* = 7.0 Hz, C**H₂**CH_2CH_2NHS), 2.75 (2H, t, *J* = 6.5 Hz, C**H₂**$CH_2CH_2CO_2H$), 2.30 (2H, t, *J* = 6.0 Hz, C**H₂**CO_2H), 1.75 – 1.91 (4H, m, C**H₂**CH_2CO_2H,C**H₂**CH_2NHS); δ_C (100 MHz, $CDCl_3$) 178.4 (**C**O_2H), 136.0 (H_2C=**C**HS), 133.7 (Ar**C**CH_2N), 130.3 (2 x Ar**C**H), 129.0 (2 x Ar**C**H), 128.7 (Ar**C**H), 126.2 (H_2**C**=CHS), 57.7 (Ph**C**H_2N), 53.6 (**C**$H_2CH_2CH_2CO_2H$), 50.9 (**C**$H_2CH_2CH_2$NHS), 41.1 (**C**H_2NHS), 35.1 (**C**H_2CO_2H), 25.1 (**C**H_2CH_2NHS), 21.2 (**C**$H_2CH_2CO_2H$); HRMS (ESI^+): calcd. for $C_{16}H_{24}NaN_2O_4S$, 363.1349. Found $[MNa]^+$ 363.1353 (—1.0 ppm error).

6-Benzyl-11-(4-fluorobenzyl)-1-thia-2,6,11-triazacyclotridecan-10-one 1,1-dioxide (147)

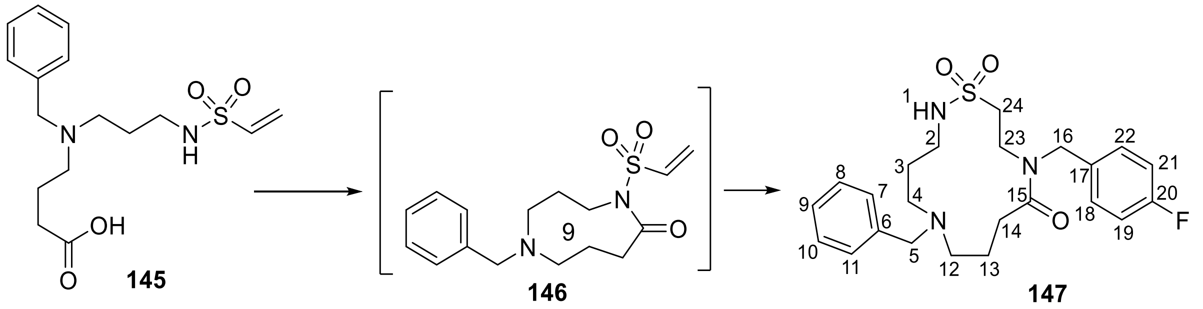

To a round-bottomed flask methyl 4-(benzyl(3-(vinylsulfonamido)propyl)amino)butanoic acid **145** (224 mg, 0.655 mmol) was dissolved in chloroform (16.0 mL) and DIPEA (229 µL, 1.32 mmol), was added dropwise. T3P (50% solution in ethyl acetate, 0.669 mL, 1.05 mmol) was added dropwise and the solution stirred at room temperature for 20 hours. The reaction mixture was diluted with DCM (30 mL) and washed with $NaHCO_3$ (2 x 30 mL). The organic layer was dried, filtered and concentrated *in vacuo* to yield the crude product (1-benzyl-5-(ethenesulfonyl)-1,5-diazonan-6-one **146**) as an orange oil [191 mg, 90%, HRMS (ESI$^+$): calcd. for $C_{16}H_{23}N_2O_3S$, 323.1424. Found $[MH]^+$ 323.1420 (1.2 ppm error] that was used directly in the next step without further purification. The unpurified 1-benzyl-5-(ethenesulfonyl)-1,5-diazonan-6-one (191 mg, 0.594 mmol) was dissolved in THF (2.0 mL), and triethylamine (0.25 mL, 1.80 mmol) was added, followed by 4-fluorobenzylamine (0.10 mL, 0.890 mmol) in a single portion. The reaction mixture was then heated at reflux for 20 h, before the solvent was removed *in vacuo*. Purification by flash column chromatography (SiO_2, 1:1 ethyl acetate : hexane → 100% ethyl acetate) afforded macrocycle **147** as a colourless oil (166 mg, 57% over 2 steps from **145**), which exists in solution in $CDCl_3$ as a 5:2 mixture of rotamers; R_f = 0.50 (100% ethyl acetate); ν_{max}/cm^{-1} (thin film): 3205, 2934, 2811, 1631, 1452, 1414, 1323, 1222, 1139, 1097, 911, 727, 702; δ_H (400 MHz, $CDCl_3$), 7.41 (4H, d, *J* = 4.0 Hz, H-7,8,10,11), 7.21 – 7.36 (1H, m, H-9), 6.94 – 7.02 (2H, m, H-19,21), 6.84 – 6.90 (2H, m, H-18,22), 6.47 (1H, d, *J* = 11.5 Hz, H-1), 4.42 (1H, d, *J* = 17.5 Hz, H-16), 4.32 (1H, d, *J* = 17.5 Hz, H-16'), 4.03 – 4.25 (1H, m, H-5 [overlapping]), 4.03 – 4.25 (2H, m, H-23 [overlapping]), 3.33 – 3.44 (1H, m, H-2), 2.91 – 3.26 (1H, m, H-2' [overlapping]), 2.91 – 3.26 (2H, m, H-24 [overlapping]), 2.91 – 3.26 (1H, m, H-4 [overlapping]), 2.74 (1H, d, *J* = 12.5 Hz, H-5'), 2.54 – 2.63 (1H, m, H-12), 2.31 – 2.44 (1H, m, H-13), 2.12 – 2.27 (1H, m, H-3 [overlapping]), 2.12 – 2.27 (1H, m, H-4' [overlapping]), 2.08 (1H, d, *J* = 13.0 Hz, H-12'), 1.97 (2H, t, *J* = 4.5 Hz, H-14), 1.66 – 1.76 (1H, m, H-3'), 1.37 – 1.48

(1H, m, H-13'), full data for minor rotamer: 7.91 (1H, br t, J = 4.5 Hz, H-1), 7.27 – 7.36 (5H, m, 5 x ArH), 7.21 – 7.27 (2H, m, H-18,22), 6.84 – 6.90 (2H, m, H-19,21), 4.57 (2H, s, H-16), 3.78 (2H, t, J = 4.0 Hz, H-23), 3.51 (2H, s, H-5), 3.08 (2H, t, J = 6.0 Hz, H-24 [overlapping]), 2.91 – 3.04 (2H, m, H-2), 2.54 – 2.63 (2H, m, H-12 [overlapping]), 2.54 – 2.63 (2H, m, H-14 [overlapping]), 2.51 (2H, t, J = 5.5 Hz, H-4), 1.84 (2H, p, J = 6.5 Hz, H-13), 1.62 (2H, p, J = 5.5 Hz, H-3); δ_C (100 MHz, $CDCl_3$) data for the major rotamer 176.2 (C-15), 162.1 (C-20, $^1J_{CF}$ = 246.4 Hz), 138.2 (C-6), 132.6 (C-17, $^4J_{CF}$ = 2.9 Hz), 130.6 (C-7,11), 128.7 (C-8,10), 127.61 (C-18,22, $^3J_{CF}$ = 7.8 Hz), 127.53 – 127.69 (C-9), 115.9 (C-19,21, $^2J_{CF}$ = 21.2 Hz), 58.5 (C-5), 56.3 (C-12), 55.0 (C-4), 54.2 (C-16), 46.2 (C-24), 45.6 (C-23), 44.8 (C-2), 32.3 (C-14), 24.8 (C-3), 20.8 (C-13); data for the minor rotamer: 172.7 (C-15), 162.3 (C-20, $^1J_{CF}$ = 246.3 Hz), 137.3 (C-6), 133.2 (C-17, $^4J_{CF}$ = 3.5 Hz), 130.1 (C-18,22, $^3J_{CF}$ = 8.3 Hz), 129.8 (C-7,11), 128.6 (C-8,10), 127.61 (C-9), 115.7 (C-19,21, $^2J_{CF}$ = 21.8 Hz), 59.5 (C-5), 54.0 (C-4), 51.9 (C-12), 48.9 (C-24), 48.2 (C-16), 43.3 (C-2), 42.5 (C-23), 29.9 (C-14), 24.1 (C-3), 22.6 (C-13); δ_F (282 MHz, $CDCl_3$), major rotamer: −114.72 (1F, m, ArF), minor rotamer −114.24 (1F, m, ArF); HRMS (ESI$^+$): calcd. for $C_{23}H_{31}FN_3O_3S$, 448.2065. Found: $[MH]^+$, 448.2052 (−2.8 ppm error).

Methyl 2-((2-(hydroxymethyl)azetidin-1-yl)methyl)benzoate (S188)

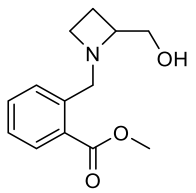

To a solution of *tert*-butyl 2-(hydroxymethyl)azetidine-1-carboxylate (187 mg, 1.00 mmol) in DCM (5.0 mL) was added trifluoroacetic acid (1.1 mL, 1.71 g, 15.0 mmol) and the resulting solution was stirred at room temperature for 1.5 h. The reaction mixture was concentrated *in vacuo* and toluene (3 x 10 mL) was added and concentrated *in vacuo* to form an azeotropic mixture to remove excess trifluoroacetic acid. The resulting colourless oil was dissolved in dry DCM (5.0 mL) and to this was added potassium carbonate (276 mg, 2.00 mmol) followed by methyl 2-(bromomethyl)benzoate (115 mg, 0.500 mmol) and the reaction mixture was heated to reflux for 18 h. The solids were filtered and washed with DCM and the filtrate was concentrated *in vacuo*. The crude product was purified by column chromatography (SiO_2, 50:49:1 ethyl acetate:hexane:triethylamine) to yield the title compound **S188** (107 mg, 91%) as a yellow oil. R_f 0.23 (50:49:1 ethyl acetate:hexane:triethylamine); δ_H (400 MHz, $CDCl_3$) 7.78

(1H, dd, J = 8.0, 1.5 Hz, ArH), 7.40 (1H, td, J = 7.5, 1.5 Hz, ArH), 7.36–7.31 (1H, m, ArH), 7.27 (1H, td, J = 7.5, 1.5 Hz, ArH), 4.19 (1H, d, J = 13.5 Hz, ArC**H**H'), 3.87 (3H, s, CH_3), 3.67 (1H, d, J = 13.5 Hz, ArCH**H'**), 3.47–3.37 (2H, m, NCH, OC**H**H'), 3.35–3.18 (3H, m, OCH**H'**, NC**H**H', OH), 2.90–2.82 (1H, m, NCH**H'**), 2.16–2.04 (1H, m, NCH_2C**H**H'), 1.92–1.83 (1H, m, NCH_2CH**H'**); δ_C (101 MHz, $CDCl_3$) 168.7 (CO), 139.6 (ArC), 131.7 (ArC), 130.3 (ArC), 130.2 (ArC), 129.9 (ArC), 127.1 (ArC), 66.8 (NCH), 62.9 (OCH_2), 60.1 ($ArCH_2$), 52.1 (CH_3), 51.8 (NCH_2), 18.6 (NCH_2**C**H_2); ν_{max}/cm^{-1} (thin film) 3423, 2951, 2841, 1717, 1601, 1578, 1434, 1263, 1198, 1128, 1078, 1041, 974, 785, 740, 667; HRMS (ESI); calcd. for $C_{13}H_{18}NO_3^+$, 236.1281. Found: $[MH]^+$, 236.1282 (–0.4 error ppm).

1,2,2a,3-tetrahydroazeto[2,1-*c*]benzo[*f*][1,4]oxazocin-5(10*H*)-one (S189)

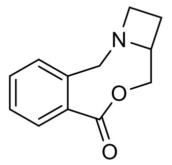

To a solution of methyl 2-((2-(hydroxymethyl)azetidin-1-yl)methyl)benzoate (107 mg, 0.455 mmol) in THF (1.4 mL), was added aq. lithium hydroxide (1.00 mL, 0.5 M, 0.500 mmol) and the reaction mixture was heated to 70 °C for 6 h. The reaction mixture was concentrated *in vacuo* and DCM (10 x 10 mL) was added and concentrated *in vacuo* to form and azeotropic mixture to remove excess water. The resulting white solid was dissolved in dry $CHCl_3$ and to this was added DIPEA (0.160 mL, 118 mg, 0.910 mmol) followed by T3P (0.400 mL, 50% in ethyl acetate, 0.683 mmol) and the reaction mixture was stirred at room temperature for 18h. After this time the reaction mixture was concentrated *in vacuo*, however no evidence of the desired product was observed by TLC, ^{1}H NMR or HRMS.

4) Evidence for formation of sulfonium cation intermediates

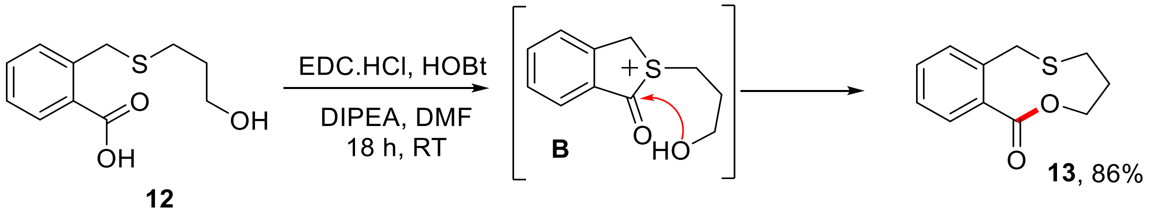

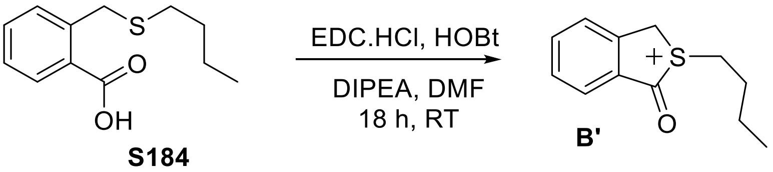

To obtain additional supporting evidence that the formation of sulfonium cation **B** is reasonable during the conversion of **12** into **13**, an analogous sulfide-containing substrate **S184** was made and tested using the same reaction conditions. The idea was that sulfonium cation **B’** would form, but without the terminal alcohol nucleophile to allow ring expansion, intermediate **B’** could then be detected by NMR and mass spectrometry (MS).

Thus, carboxylic acid **S184** was prepared (see section 3 above for details of its synthesis) and then reacted under the standard conditions (**general CRE method B**) with EDC.HCl and HOBt and the five-membered-ring acyl sulfonium cation **B’** was observed by APCI$^+$ MS; a peak at m/z of 207.084693 was observed which corresponds to the mass of cationic intermediate **B’** $[C_{12}H_{15}OS]^+$. The peak at m/z 174.1609 is likely associated with the reagent EDC.HCl.

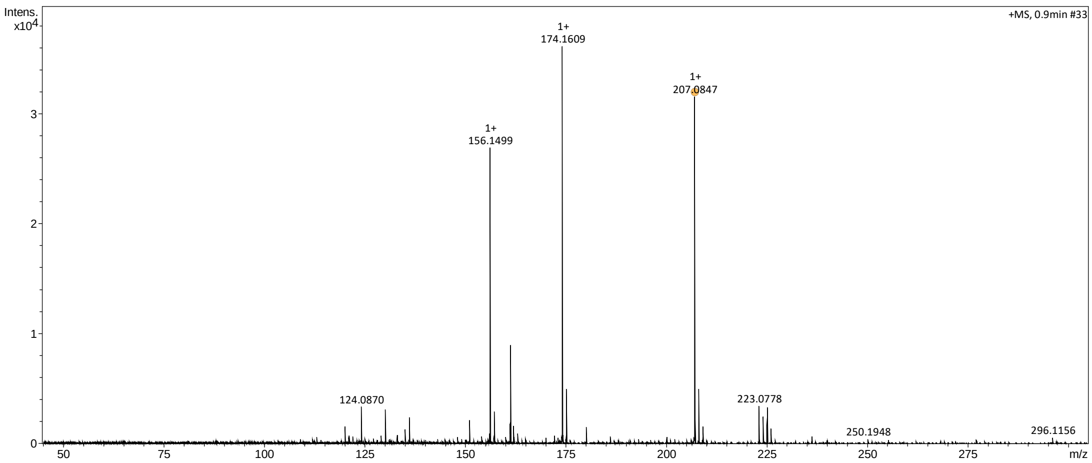

Meas. m/z	#	Ion Formula	m/z	err [ppm]	err [mDa]	mSigma	Mean err [ppm]
207.084693	1	C12H15OS	207.083813	-4.2	-0.9	9.9	-1.8

We also sought to obtain evidence for the formation of intermediate **B'** using ^{13}C NMR spectroscopy. Thus, carboxylic acid **S184** was reacted under the standard conditions in an NMR tubes using DMF-d_7 as solvent and monitored by ^{13}C NMR. The NMR spectrum below was taken after reaction for 1 h, with the major carbonyl resonance corresponding to the carbonyl of HOBt adduct **S185**. Leaving the mixture for a longer time period (up to 10 days) resulted in no significant change.

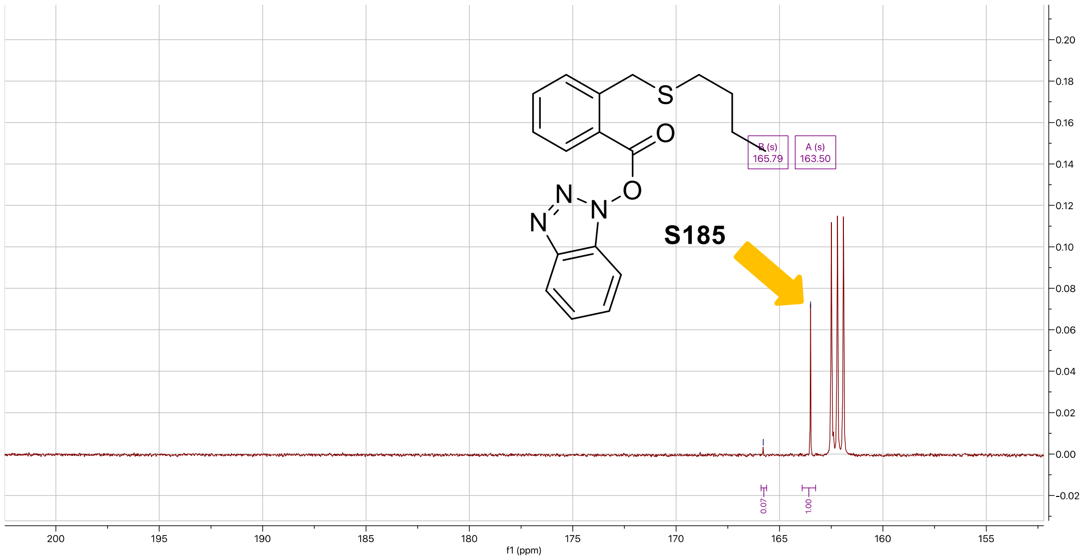

While sulfonium cation **B'** was not detected, we postulated that under the reaction conditions, with excess HOBt present and no chance to rearrange, cation **B** may exist in equilibrium with **S185**.

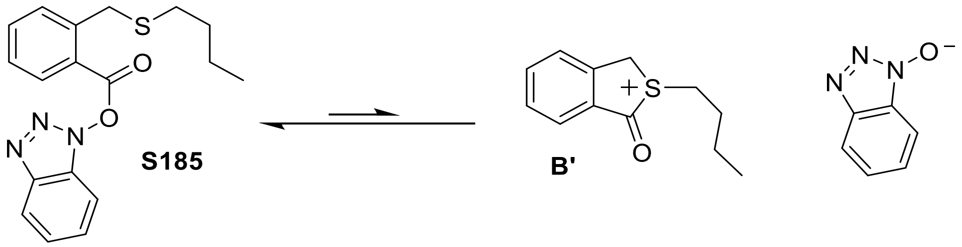

Therefore, the experiment was repeated, but with the addition of sodium tetrakis[3,5-bis(trifluoromethyl)phenyl]borate (NaBARF). The hope was that this non-coordinating anion would help to stabilise **B'** and push the equilibrium more in favour of the right-hand side such that sulfonium cation **B'** could be observed. This worked well; after the addition of NaBARF, a new carbonyl signal at 172.4 ppm was observed in the ^{13}C NMR spectrum, which we propose

corresponds to cationic intermediate **B'**, presumably associated with a BARF anion. A corresponding reduction in the intensity of the peak for HOBt adduct **S185** was also observed.

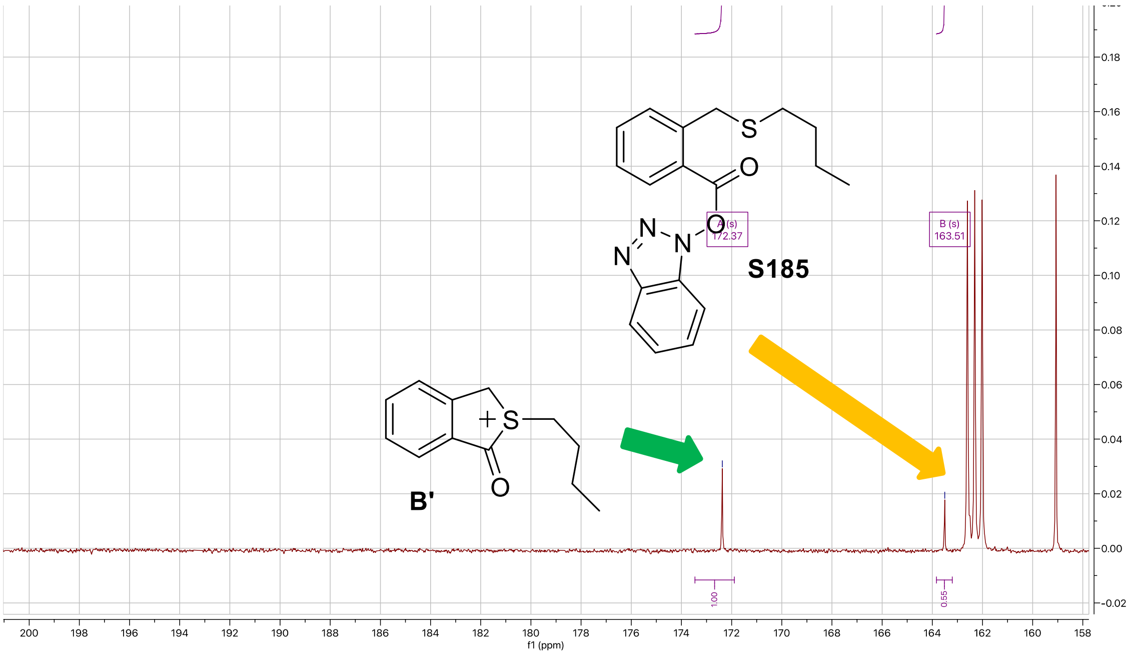

In situ synthesis of 2-butyl-1-oxo-2,3-dihydro-1*H*-benzo[*c*]thiophen-2-ium (B')

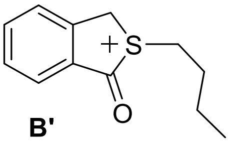

B'

EDC.HCl (212 mg, 0.990 mmol) was added to a clear colourless solution of 2-((butylthio)methyl)benzoic acid **S184** (148 mg, 0.660 mmol), HOBt (139 mg, 0.792 mmol) and anhydrous DIPEA (290 µL, 1.65 mmol) in anhydrous DMF-d_7 (7 mL) at RT in a standard NMR tube. A colour change to pale yellow was noted immediately upon the addition of DIPEA via syringe over a period of 20 min. The resulting mixture was stirred at RT for a total of 1 h under Ar, after which time tetrakis(3,5-bis(trifluoromethyl)phenyl)borate (BARF, 80.1 mg) was added to reaction mixture. After 2 h, the reaction mixture was directly analysed by ^{13}C NMR, with a new carbonyl signal consistent with formation of **B'** observed: δ_C (101 MHz; $DCON(CD_3)_2$) 173.1 (C=O). The reaction was also analysed by HRMS (APCI): m/z calcd. for $C_{12}H_{15}OS$: 207.083813, found: 207.084693 $[M]^+$.

These ^{13}C NMR data for were in line with those previously reported in the literature for structurally similar 5-membered ring cyclic sulfonium cations.[20],[21]

5) Computational Chemistry

The structures **A** and **A'** were loaded in PCModel,[22] and a conformational analysis was performed using the Molecular Mechanics Force Field (MMFF94) level of theory.[23–27] The structures within 3.5 kcal mol^{-1} of the lowest energy conformation were kept and the geometry of each structure was optimised using the Gaussian 16, Revision C.02 package,[28] at the B3LYP/6-31G* level of theory.[29-33] The lowest energy structure was then reoptimised with tight convergence criteria followed by frequency calculations, which confirmed the structures were minima on the potential energy surface due to the absence of imaginary frequencies. The relative energies of these structures are given in the scheme below.

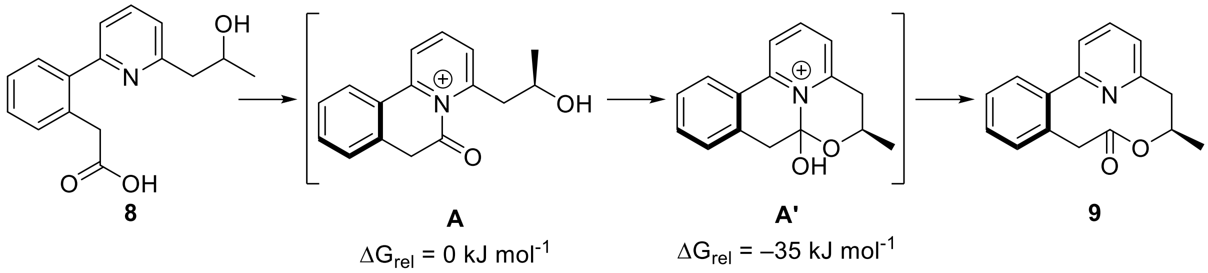

Energies and xyz coordinates for A

B3LYP/6-31G*

SCF Done: E(RB3LYP) = -824.327219460

Zero-point correction= 0.289556
Thermal correction to Gibbs Free Energy= 0.245571

C	-4.55019900	-0.98106500	0.35202400
C	-4.51675700	0.39979700	0.13880500
C	-3.29947600	1.05114500	-0.01851200
C	-2.09147900	0.32866800	0.03873100
C	-2.13806300	-1.06586300	0.23134700
C	-3.36203400	-1.71130000	0.39006800
H	-5.50093100	-1.49128700	0.47122900
H	-5.44038500	0.96679900	0.08477600
H	-3.29724900	2.11717500	-0.21633800
H	-3.38713800	-2.78740400	0.53560400
C	-0.79509100	1.01119000	-0.07830600
C	-0.65124200	2.38476000	0.11801700
C	0.59071600	2.98914200	-0.00976500
H	-1.51690700	2.97071400	0.39544200

C	1.59278600	0.84821900	-0.51386400
C	1.70753600	2.21668400	-0.32001800
H	0.69383600	4.05780500	0.15087300
H	2.69107200	2.66461300	-0.38783200
N	0.34020200	0.27806600	-0.39333200
C	-0.83508100	-1.82978200	0.25461100
H	-0.40040900	-1.82610600	1.26741300
H	-0.95804800	-2.86912800	-0.05681000
C	0.14975400	-1.18524600	-0.67255600
O	0.74854400	-1.70348900	-1.56677800
C	2.83322300	0.02276700	-0.75468700
H	2.71698400	-0.64085000	-1.61066000
H	3.63752700	0.72778600	-0.98145200
C	3.26122300	-0.78985300	0.48772500
H	2.50759500	-1.57218300	0.68310000
C	4.61515900	-1.45904500	0.26739900
H	5.38798300	-0.70854100	0.07042600
H	4.90828100	-2.02769300	1.15786300
H	4.58063800	-2.15960500	-0.57359900
O	3.27948000	0.14402900	1.56293200
H	3.70571300	-0.27417900	2.32794300

Energies and xyz coordinates for A’

B3LYP/6-31G*

SCF Done: E(RB3LYP) = -824.345931253

Zero-point correction= 0.291505
Thermal correction to Gibbs Free Energy= 0.250797

C	4.19312900	-1.19353300	-0.05997200
C	4.24471200	0.14254500	0.34512900
C	3.08528800	0.91061600	0.36231700
C	1.85541700	0.35309100	-0.03368100
C	1.80512300	-1.00360600	-0.42162600
C	2.97455900	-1.76356200	-0.43158000
H	5.09689000	-1.79485100	-0.07182400
H	5.18605200	0.58314600	0.65739500
H	3.13886300	1.93626900	0.71118600
H	2.93051200	-2.80666400	-0.73228000
C	0.62906000	1.16965500	-0.06808200

C	0.65951600	2.56437300	-0.13161600
C	-0.52188800	3.29520900	-0.14162900
H	1.61443700	3.06934900	-0.19426900
C	-1.77006700	1.23634900	-0.07810100
C	-1.74107100	2.62624100	-0.10752400
H	-0.49142800	4.37946800	-0.18392800
H	-2.68067300	3.16769200	-0.11746500
N	-0.59617200	0.54589200	-0.06272800
C	0.47097700	-1.58163100	-0.81218400
H	0.21294500	-1.34632400	-1.85229900
H	0.45378100	-2.67074400	-0.71251800
C	-0.62663600	-1.03045600	0.08419900
C	-3.08834500	0.49722400	-0.10863300
H	-3.81263300	1.05053400	0.49837700
H	-3.45749100	0.52018000	-1.14389000
C	-2.98409600	-0.95398700	0.34450700
H	-2.79632600	-1.01416200	1.42076200
C	-4.17935800	-1.80668100	-0.03482700
H	-4.02478300	-2.83849900	0.29153600
H	-5.08351500	-1.42412500	0.44975000
H	-4.33381800	-1.80207900	-1.11867700
O	-1.82795600	-1.49250800	-0.35352500
O	-0.46007800	-1.29116200	1.43413200
H	0.48673100	-1.33214700	1.65219900

6) X-ray structures

4,5-Dihydro-3*H*-benzo[*g*][1,5]oxathionin-1(7*H*)-one (13)

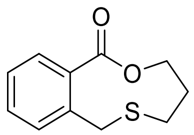

Datablock wpu2006 - ellipsoid plot

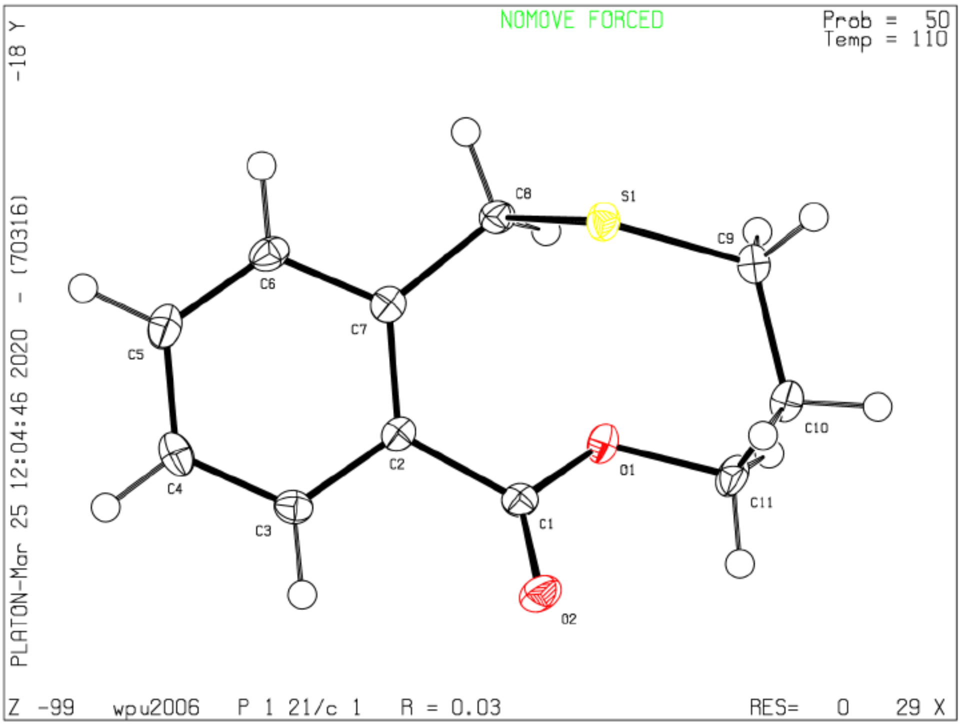

X-ray crystallographic data for this compound can be accessed via www.ccdc.cam.ac.uk/data_request/cif (CCDC 2221211)

1,8-Dimethyl-5,6,7,8-tetrahydro-1H-benzo[e][1,4,7]oxadiazecine-2,3-dione (25)

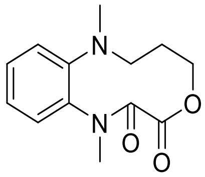

Datablock wpu22001 - ellipsoid plot

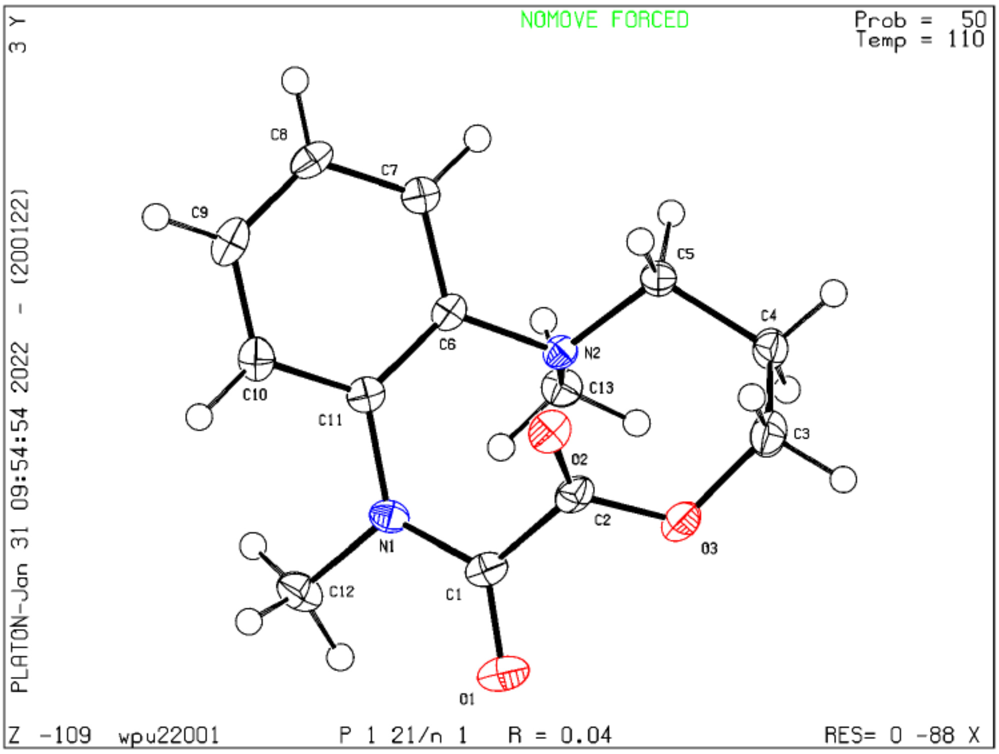

X-ray crystallographic data for this compound can be accessed via www.ccdc.cam.ac.uk/data_request/cif (CCDC 2223454).

1-Methyl-5,6,7,8-tetrahydro-1H-benzo[e][1,4]oxazecine-2,3-dione (27)

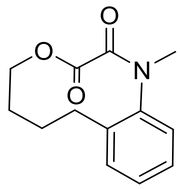

Datablock wpu22002 - ellipsoid plot

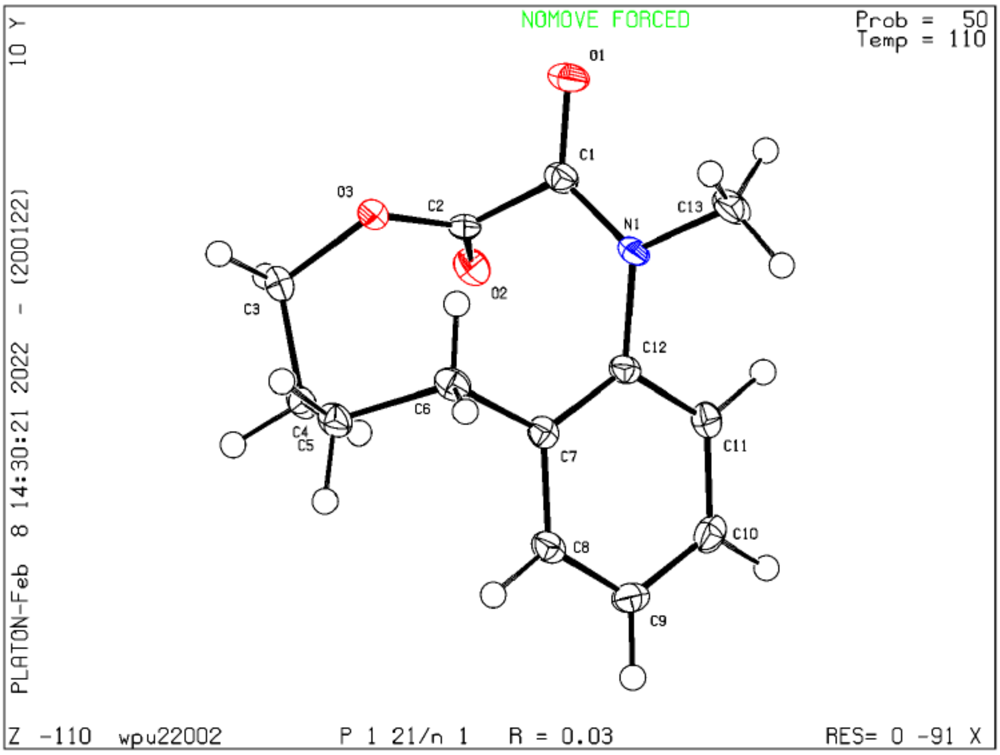

X-ray crystallographic data for this compound can be accessed via www.ccdc.cam.ac.uk/data_request/cif (CCDC 2223696).

3,4-Dihydrobenzo[*f*][1,4]oxathiocin-1(6*H*)-one (47)

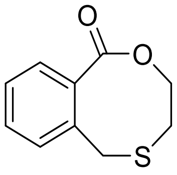

Datablock exp_57 - ellipsoid plot

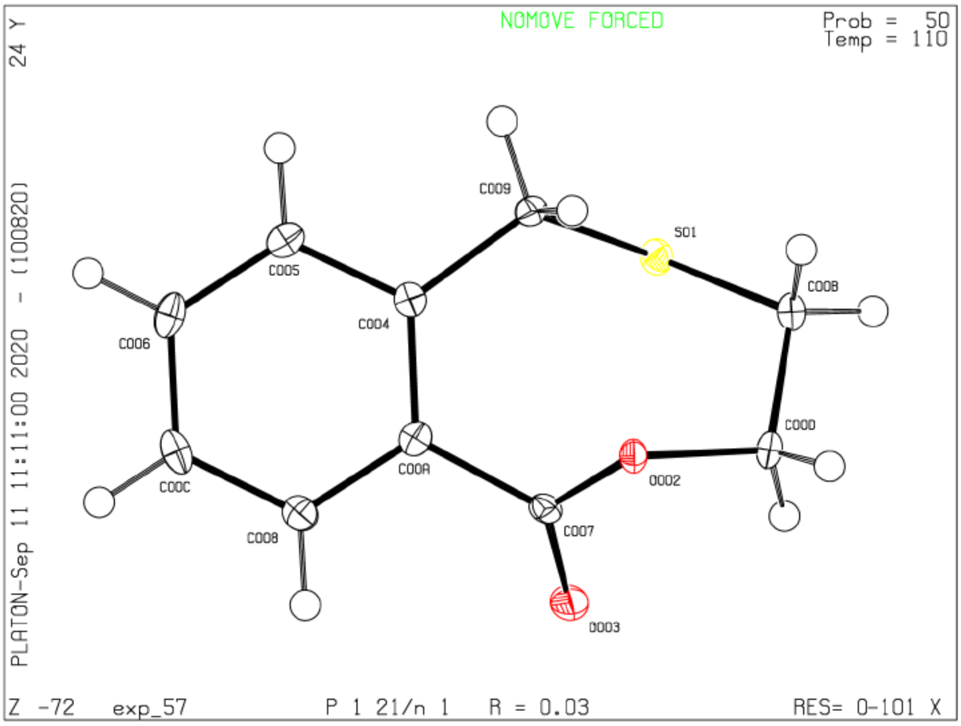

X-ray crystallographic data for this compound can be accessed via www.ccdc.cam.ac.uk/data_request/cif (CCDC 2221558)

10-Fluoro-4,5-dihydro-3*H*-benzo[*g*][1,5]oxathionin-1(7*H*)-one (48)

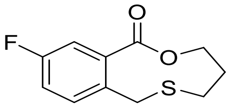

Datablock wpu2010 - ellipsoid plot

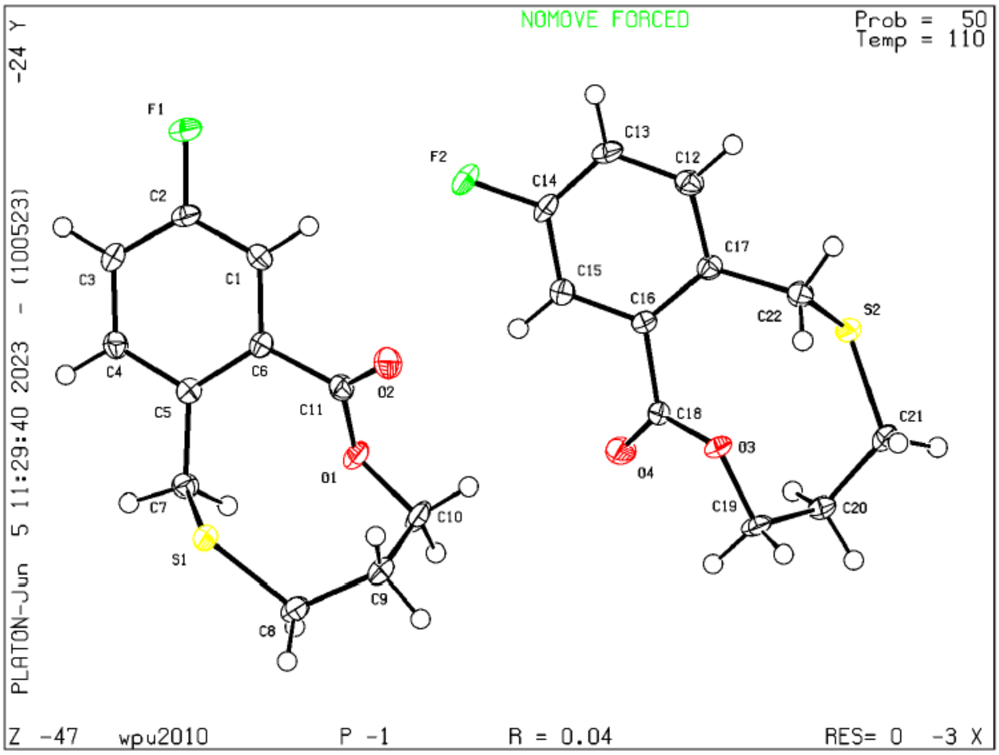

X-ray crystallographic data for this compound can be accessed via www.ccdc.cam.ac.uk/data_request/cif (CCDC 2221239)

11-Bromo-4,5-dihydro-3*H*-benzo[*g*][1,5]oxathionin-1(7*H*)-one (49a)

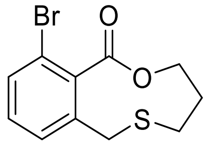

Datablock wpu2011 - ellipsoid plot

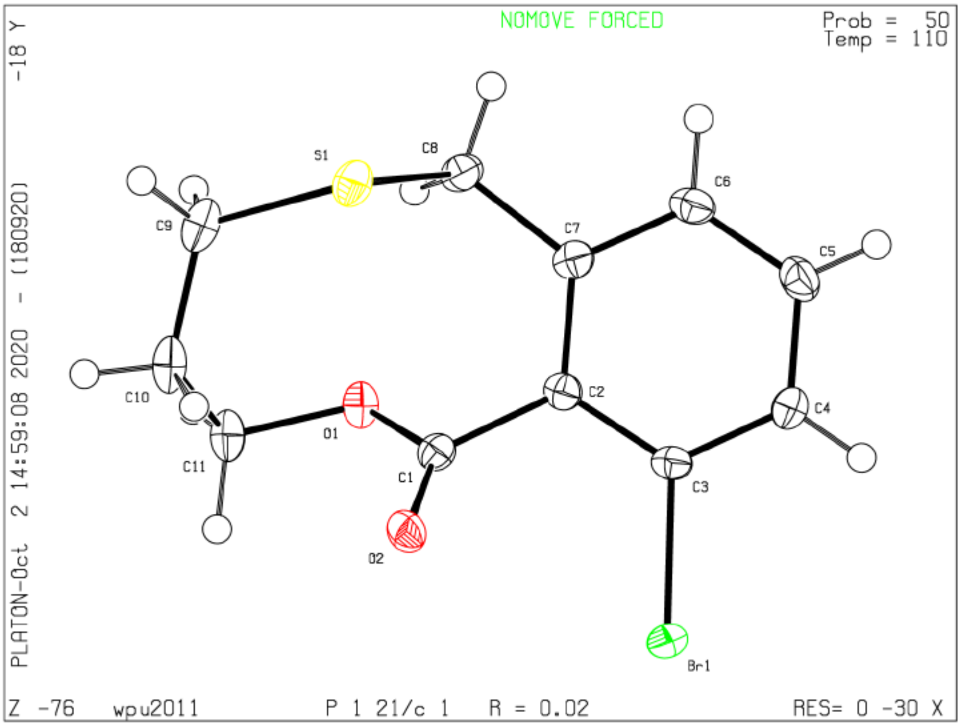

X-ray crystallographic data for this compound can be accessed via www.ccdc.cam.ac.uk/data_request/cif (CCDC 2221227)

9-Bromo-4,5-dihydro-3*H*-benzo[*g*][1,5]oxathionin-1(7*H*)-one (49b)

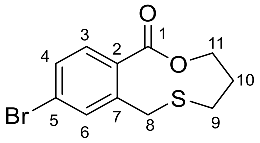

Datablock wpu2005 - ellipsoid plot

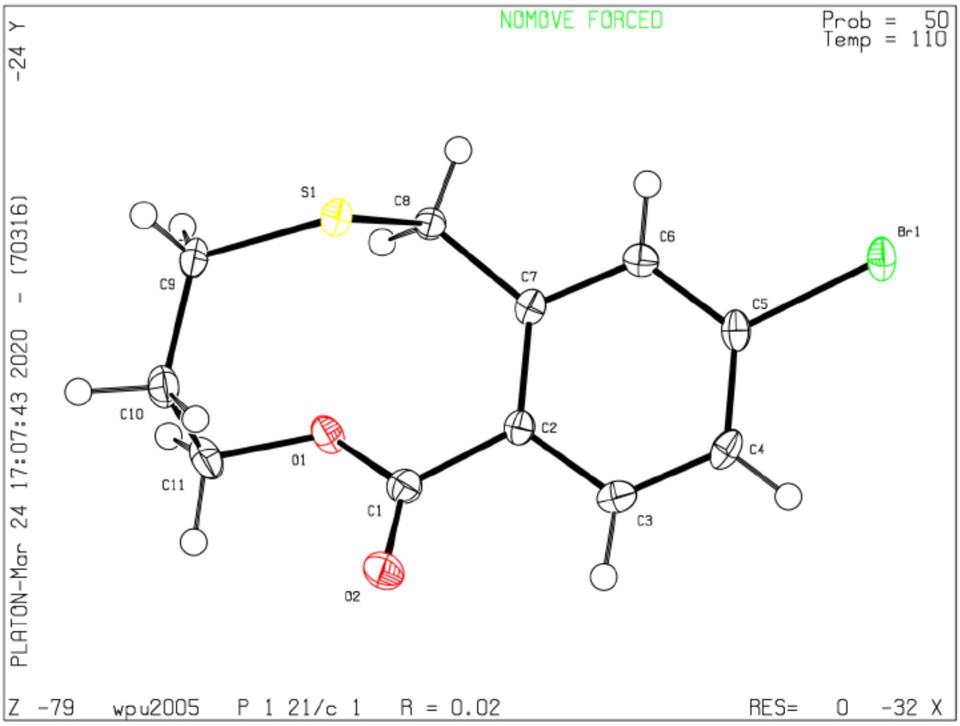

X-ray crystallographic data for this compound can be accessed via www.ccdc.cam.ac.uk/data_request/cif (CCDC 2221214)

8-Bromo-4,5-dihydro-3*H*-benzo[*g*][*1,5*]oxathionin-1(7*H*)-one (49c)

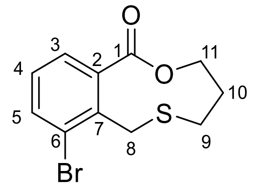

Datablock wpu2007 - ellipsoid plot

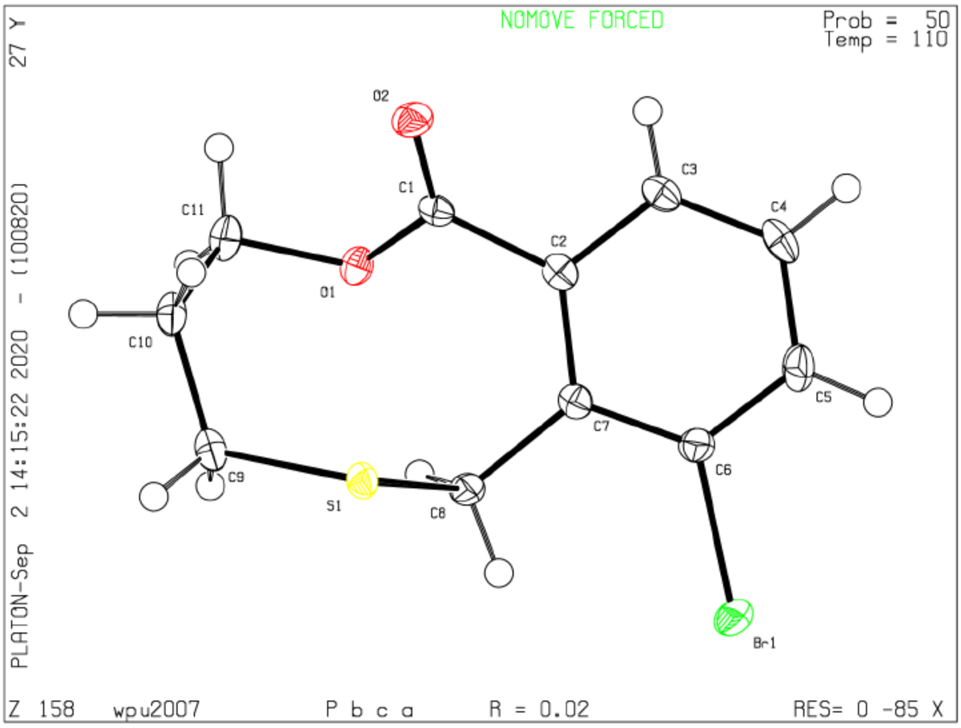

X-ray crystallographic data for this compound can be accessed via www.ccdc.cam.ac.uk/data_request/cif (CCDC 2221221).

4,5-Dihydro-3*H*-benzo[*g*][1,5]oxaselenonin-1(7*H*)-one (52)

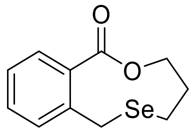

Datablock: wpu21006 - ellipsoid plot

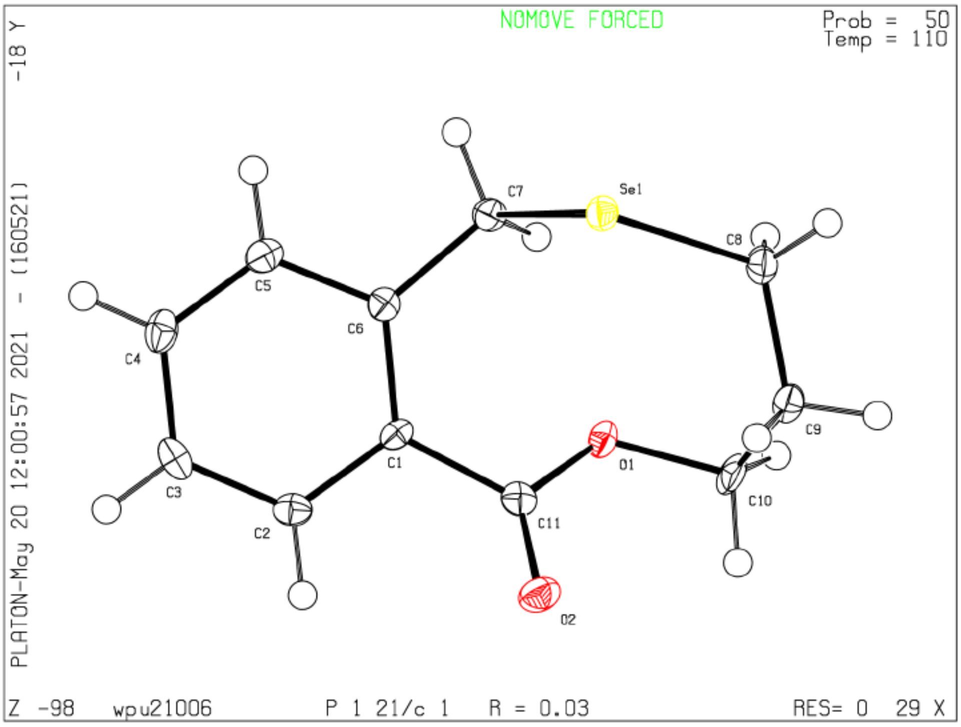

X-ray crystallographic data for this compound can be accessed via www.ccdc.cam.ac.uk/data_request/cif (CCDC 2235961)

4,5-Dihydro-3H-benzo[*g*][1,5]dithionin-1(7*H*)-one (69)

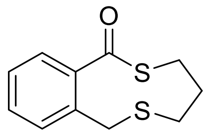

Datablock wpu2015a - ellipsoid plot

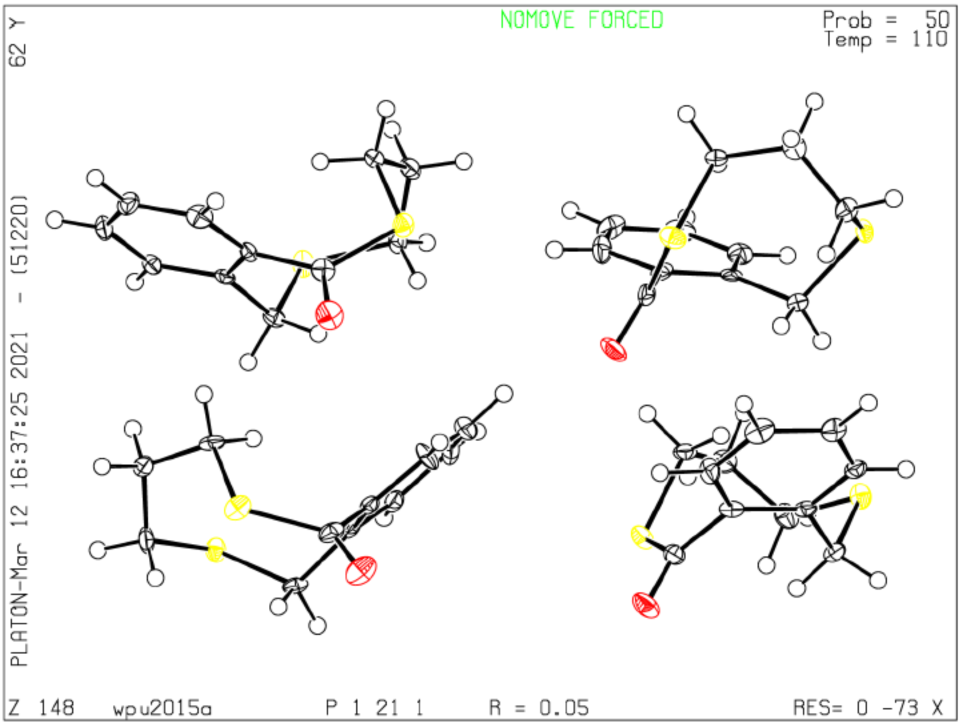

X-ray crystallographic data for this compound can be accessed via www.ccdc.cam.ac.uk/data_request/cif (CCDC 2236987)

8,9,19,20-Tetrahydro-7*H*,18*H*-dibenzo[*g,p*][1,5,10,14]tetrathiacyclooctadecine-5,16(11*H*,22*H*)-dione (S87)

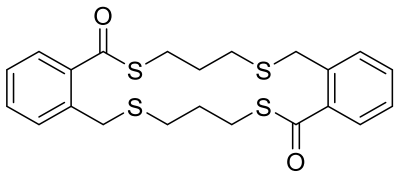

Datablock: wpu2014 - ellipsoid plot

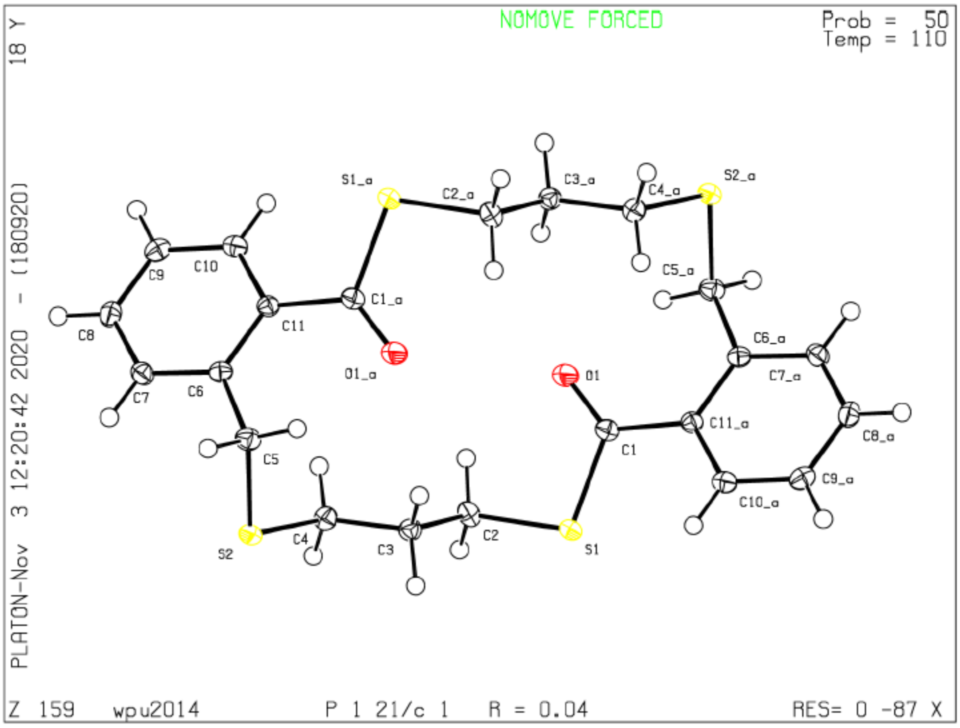

X-ray crystallographic data for this compound can be accessed via www.ccdc.cam.ac.uk/data_request/cif (CCDC 2236988).

3,4,5,6-Tetrahydrobenzo[g][1,5]thiazonin-7(1H)-one (74)

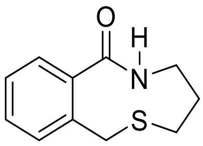

Datablock: wpu21002 - ellipsoid plot

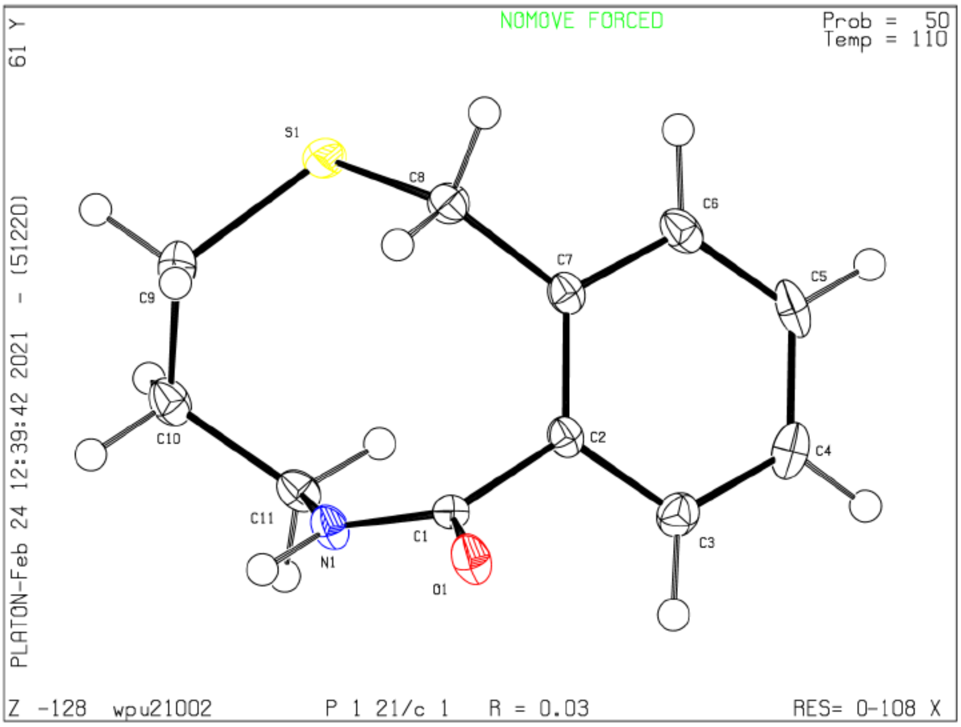

X-ray crystallographic data for this compound can be accessed via www.ccdc.cam.ac.uk/data_request/cif (CCDC 2236990)

6-Phenyl-3,4,5,6-tetrahydrobenzo[*g*][1,5]thiazonin-7(1*H*)-one (75)

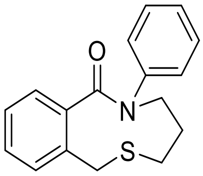

Datablock wpu21005 - ellipsoid plot

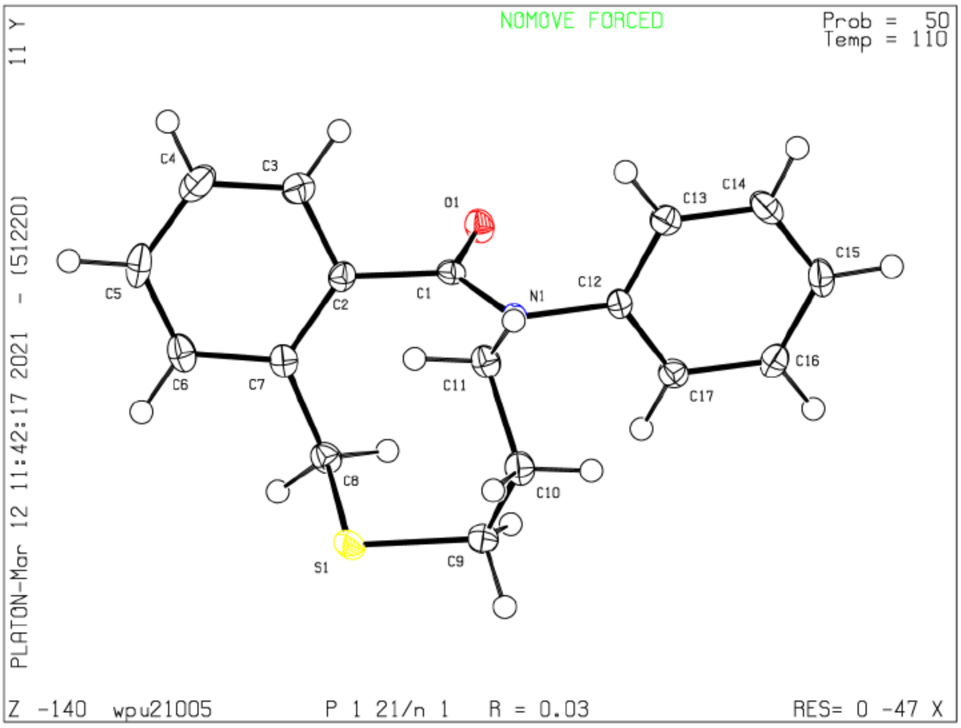

X-ray crystallographic data for this compound can be accessed via www.ccdc.cam.ac.uk/data_request/cif (CCDC 2235962)

3,6-Diphenyl-1,3,6-oxadiazocane-2-thione (93)

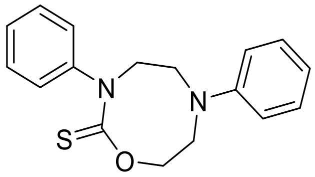

Datablock wpu21023 - ellipsoid plot

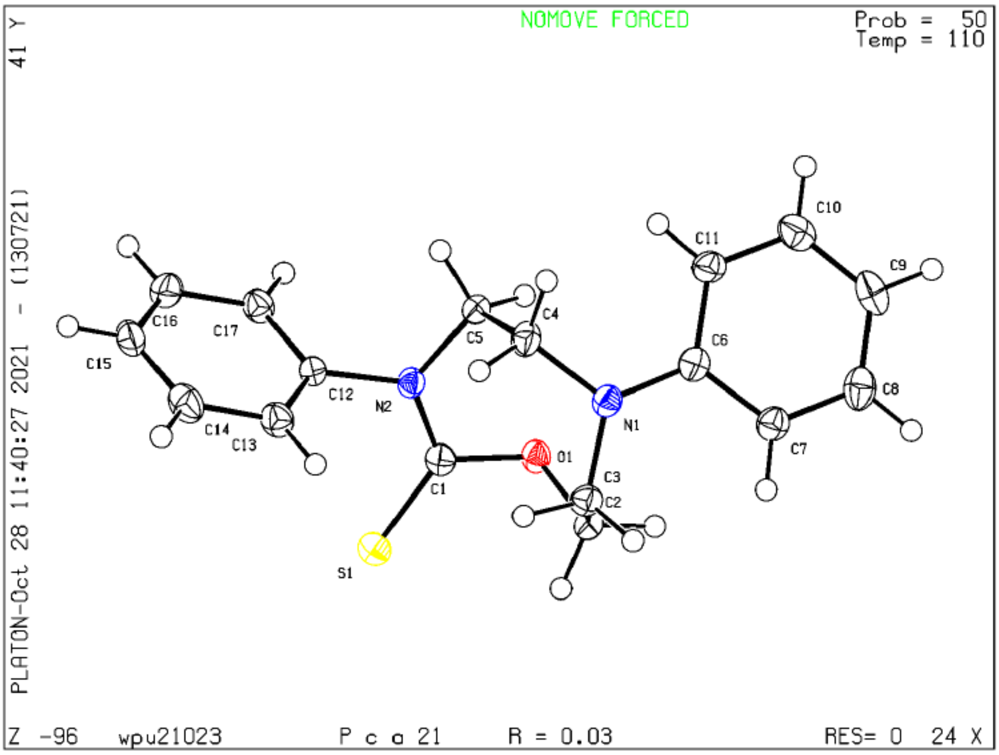

X-ray crystallographic data for this compound can be accessed via www.ccdc.cam.ac.uk/data_request/cif (CCDC 2221454)

14-Methyl-15-phenyl-10-oxa-14,21-diazatricyclo[15.3.1.$0^{2,7}$]henicosa-1(21),2,4,6,17,19-hexaen-9-one (117)

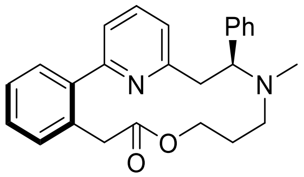

Datablock wpu2001 - ellipsoid plot

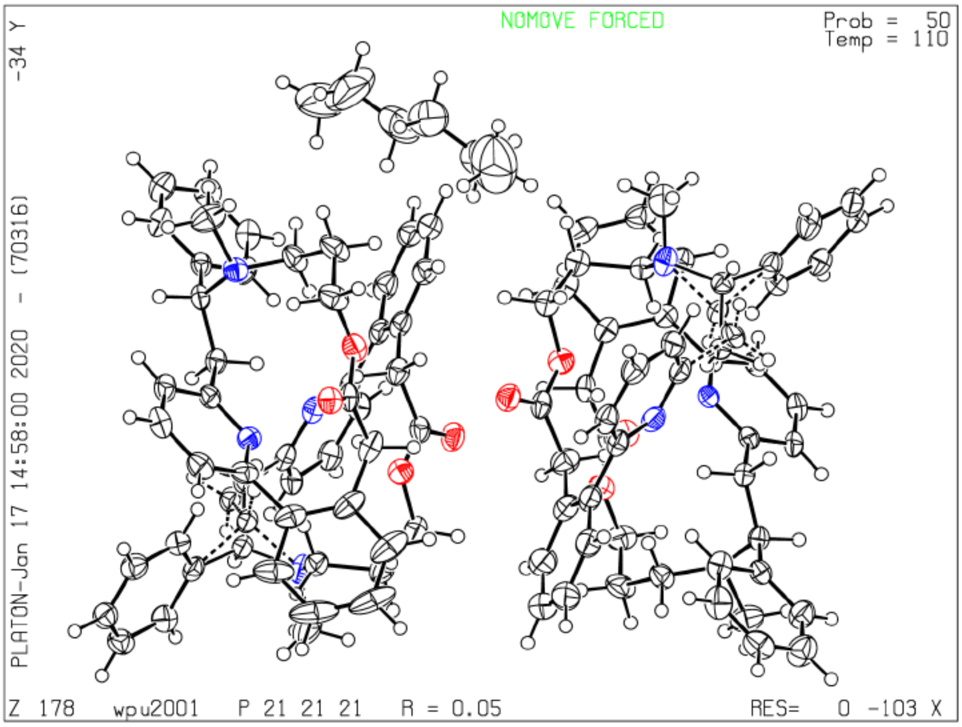

X-ray crystallographic data for this compound can be accessed via www.ccdc.cam.ac.uk/data_request/cif (CCDC 2004423)

5,16-Dioxa-1,12(2,6)-dipyridina-2,13(1,2)-dibenzenacyclodocosaphane-4,15-dione (S152)

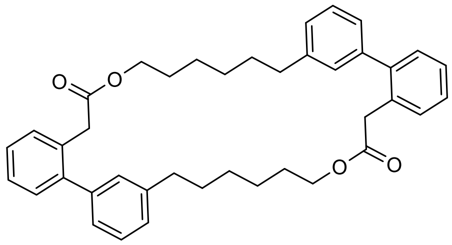

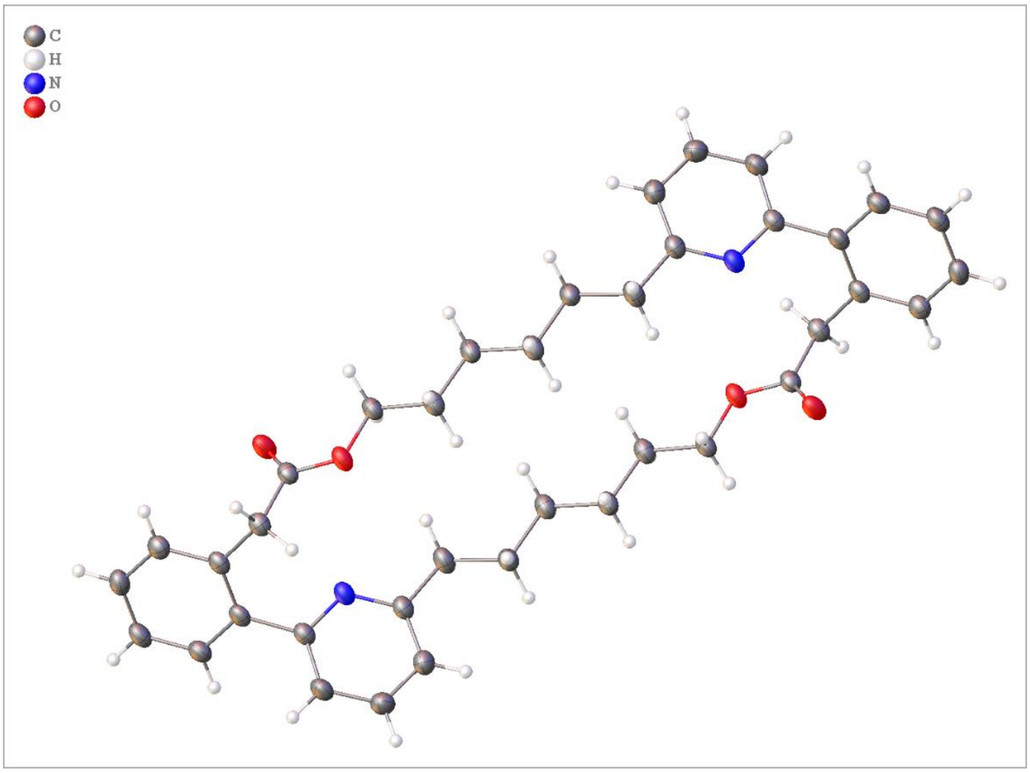

X-ray crystallographic data for compound **S152** can be accessed via www.ccdc.cam.ac.uk/data_request/cif (CCDC 2267184).

7,8,9,10,11,12,13,14,21,22,23,24,25,26,27,28-hexadecahydro-5H,19H-dibenzo[c,o][1,13]dioxacyclotetracosine-5,19-dione (136)

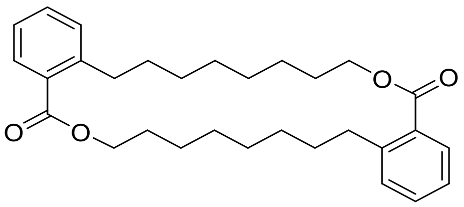

Datablock wpu21007 - ellipsoid plot

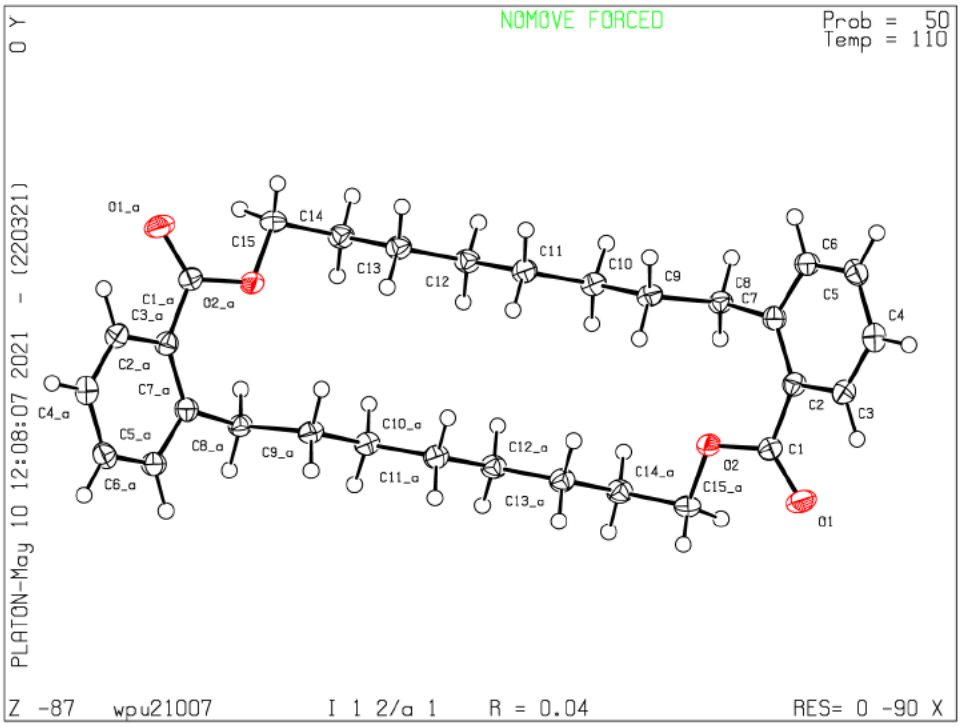

X-ray crystallographic data for compound **136** can be accessed via www.ccdc.cam.ac.uk/data_request/cif (CCDC 2237214).

1-Benzyl-1,5-diazonan-6-one (140)

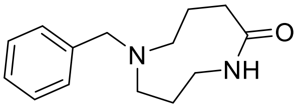

Datablock wpu23010 - ellipsoid plot

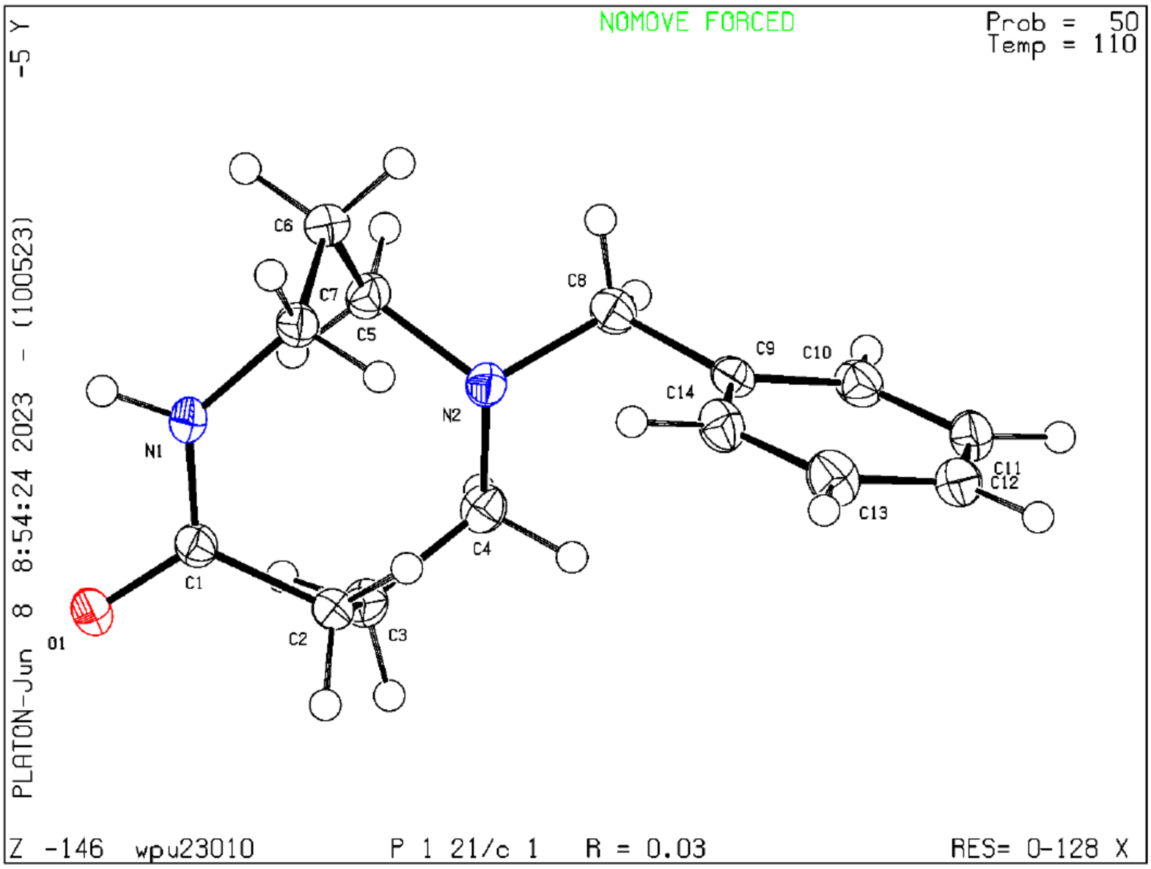

X-ray crystallographic data for compound **140** can be accessed via www.ccdc.cam.ac.uk/data_request/cif (CCDC 2320967).

7) ^{1}H and ^{13}C NMR spectra

Methyl 2-(2-bromophenyl)acetate (S2)

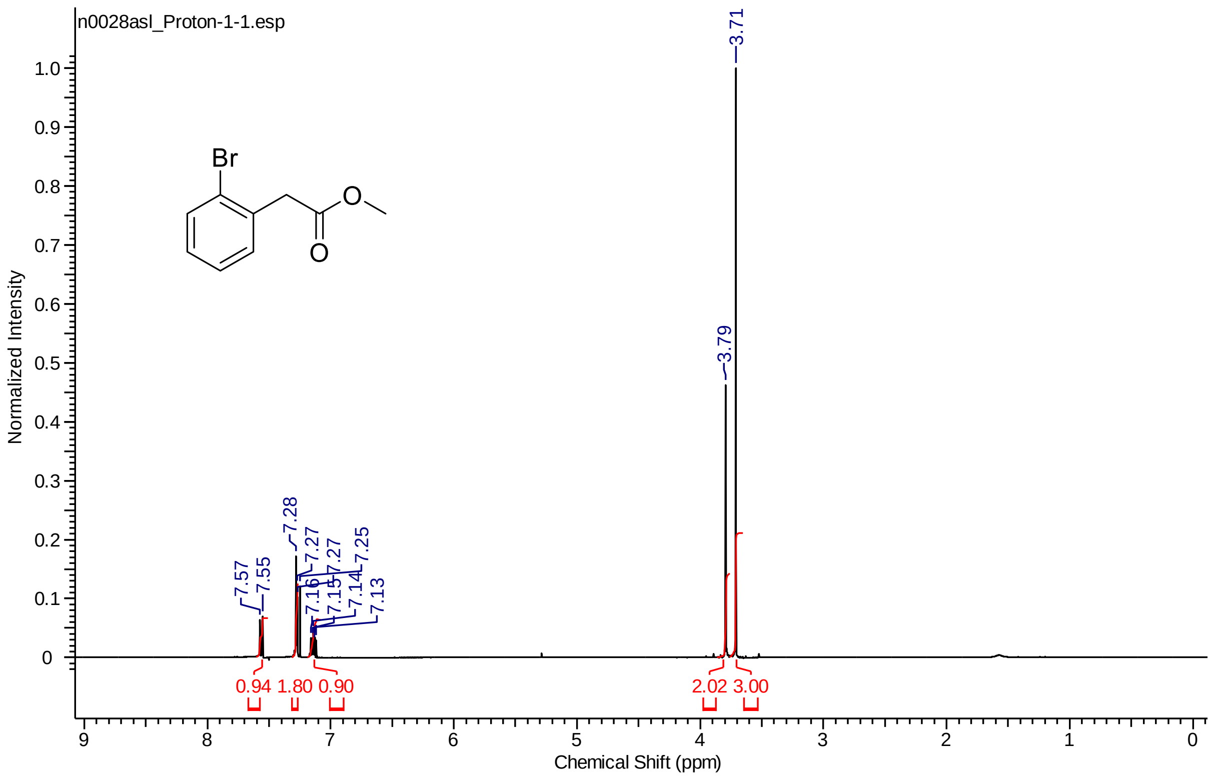

Methyl 2-(2-(4,4,5,5-tetramethyl-1,3,2-dioxaborolan-2-yl)phenyl)acetate (S3)

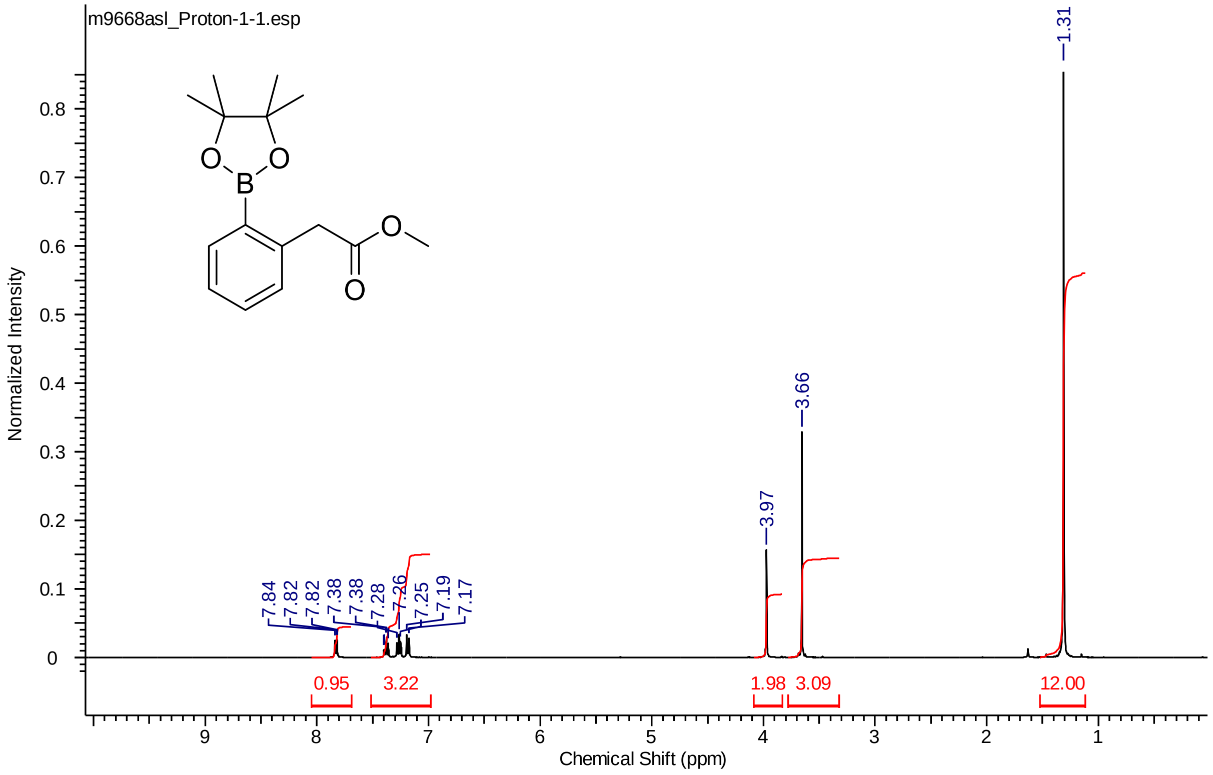

Methyl 2-(2-(6-(2-oxopropyl)pyridin-2-yl)phenyl)acetate (S4)

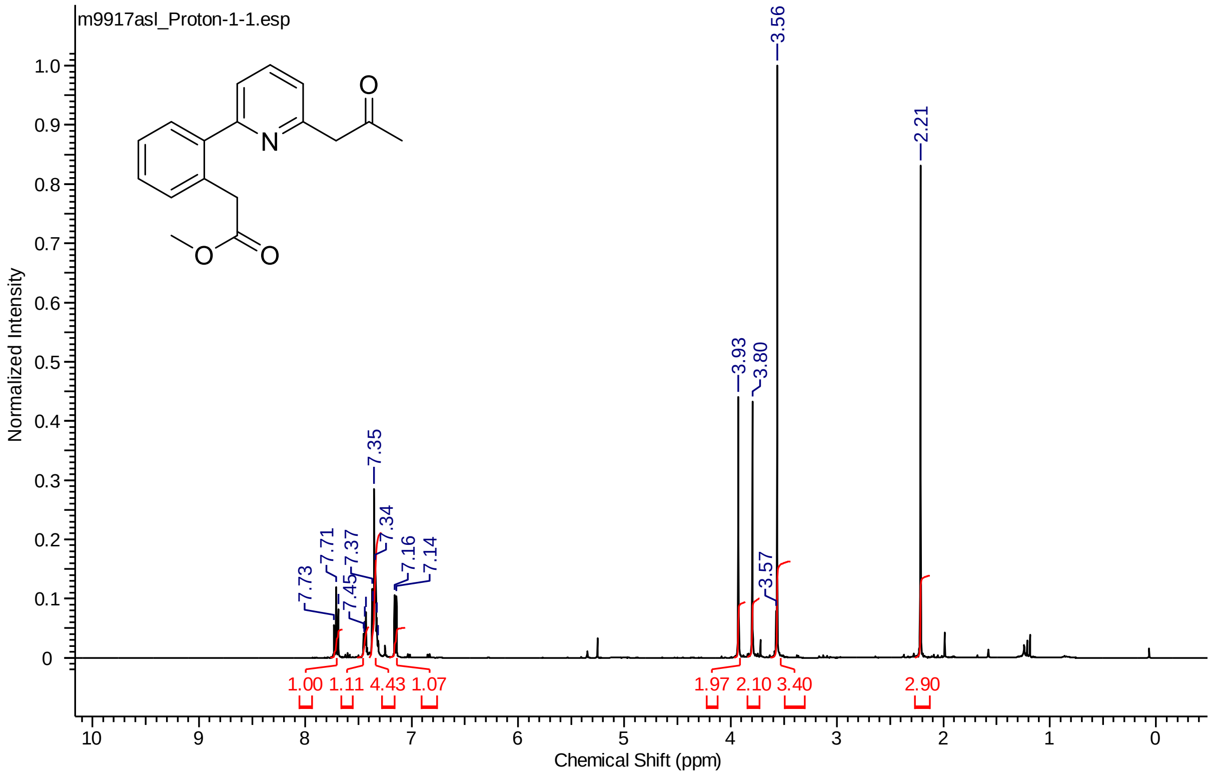

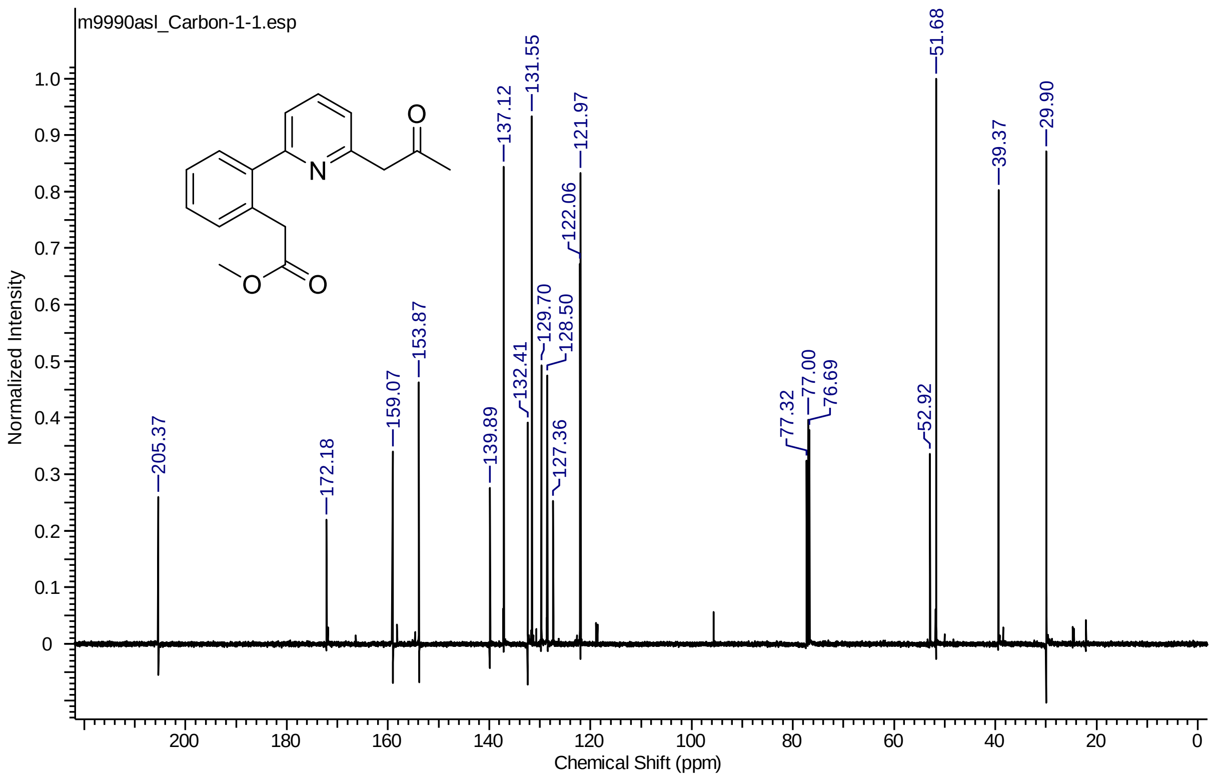

Methyl 2-(2-(6-(2-hydroxypropyl)pyridin-2-yl)phenyl)acetate (S5)

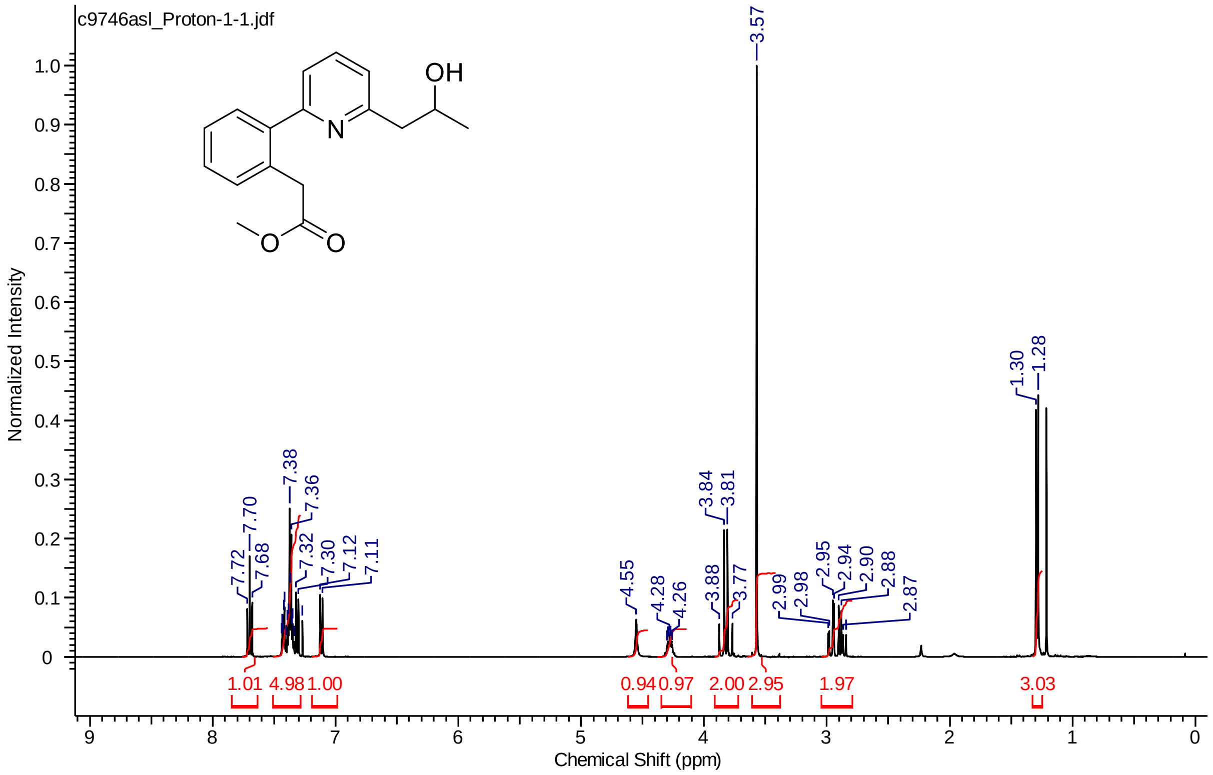

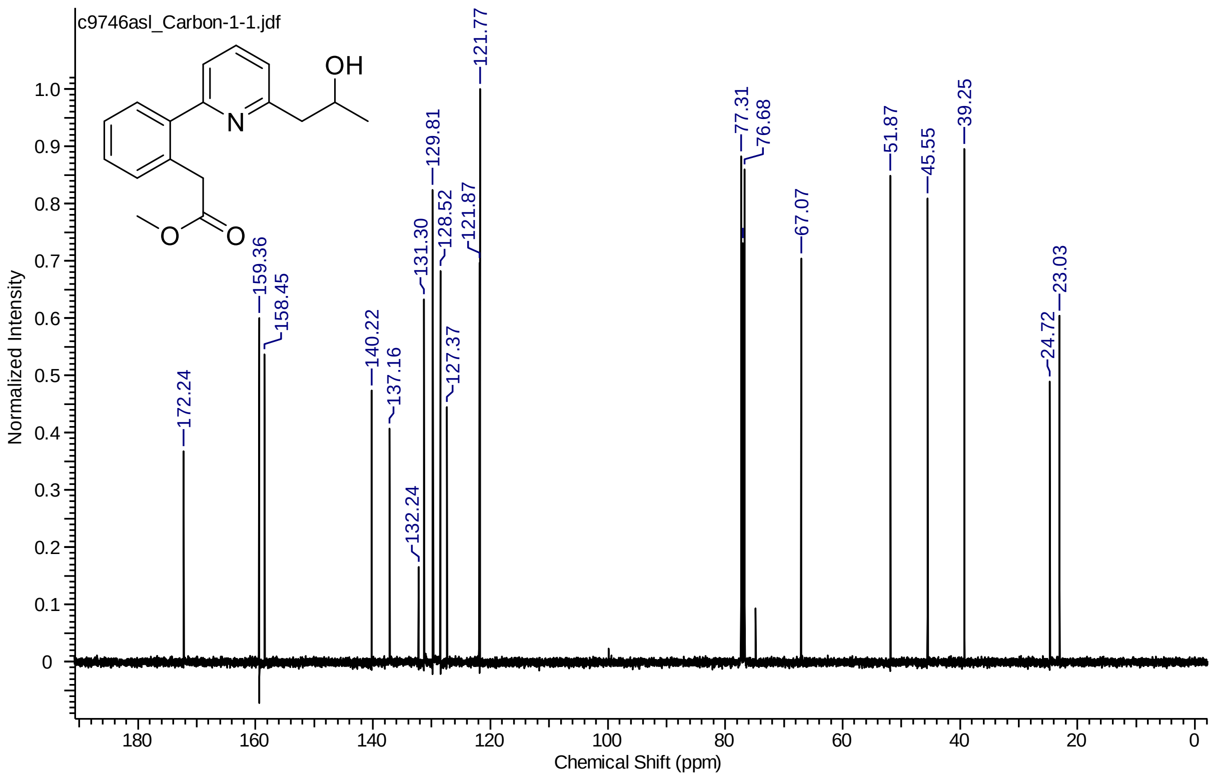

2-(2-(6-(2-Hydroxypropyl)pyridin-2-yl)phenyl)acetic acid (8)

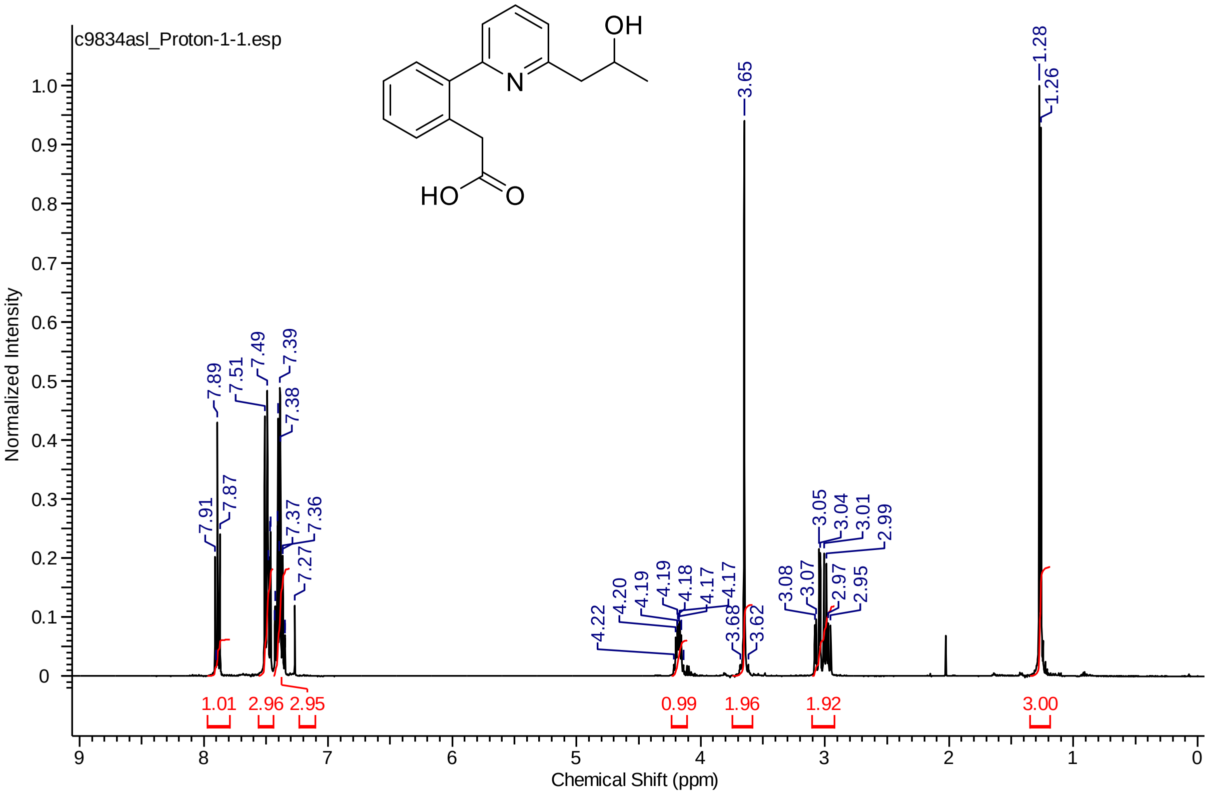

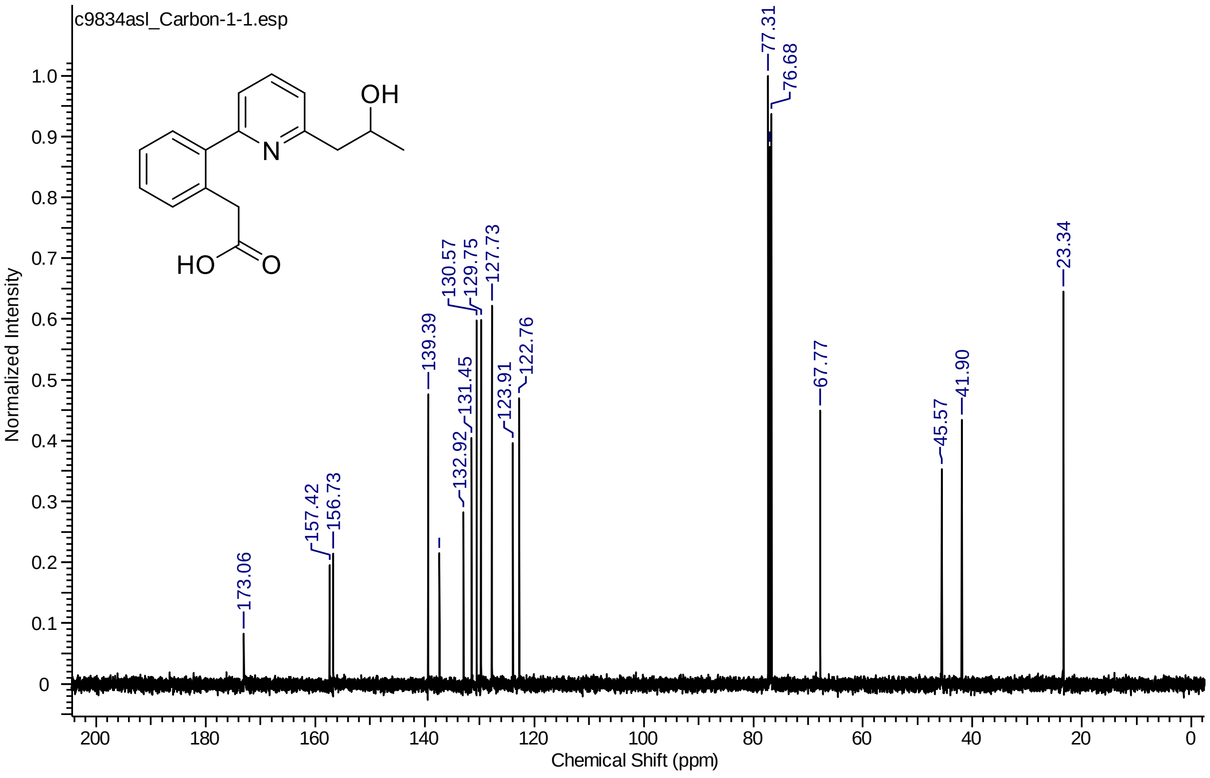

4-Methyl-4,5-dihydro-6,10-(azeno)benzo[*d*][1]oxacyclododecin-2(1*H*)-one (9)

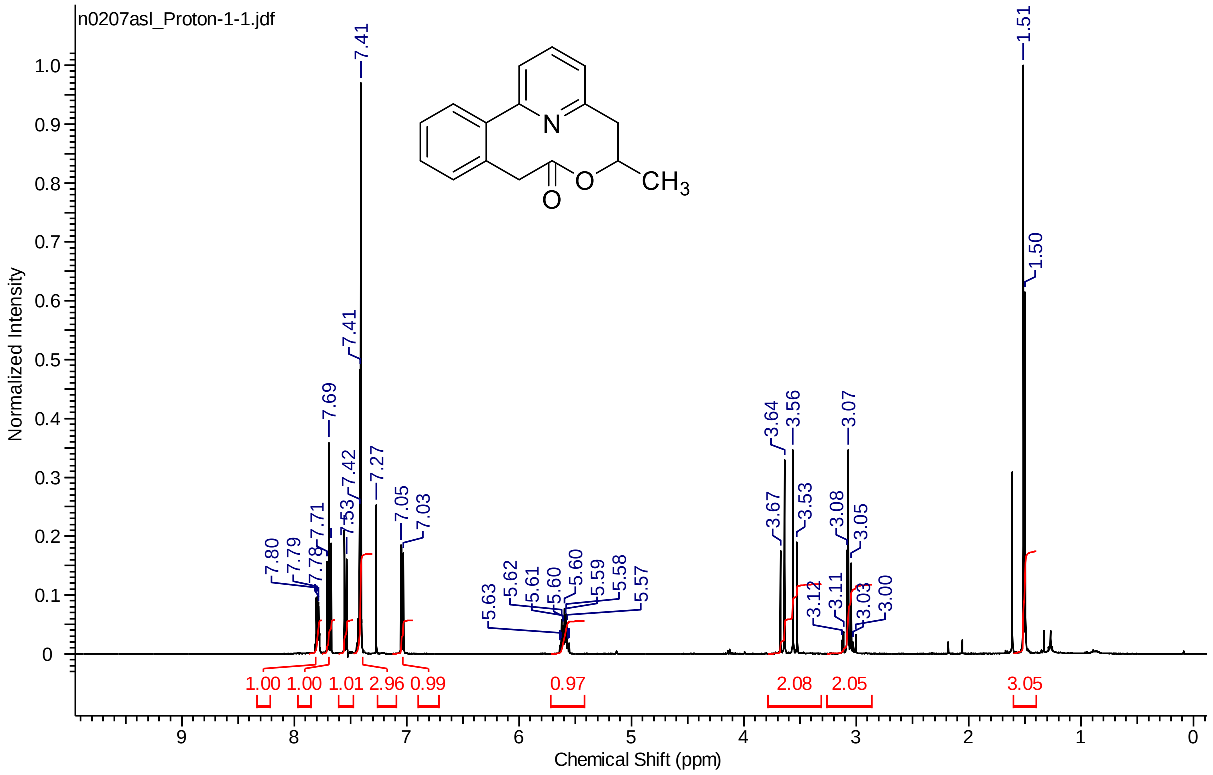

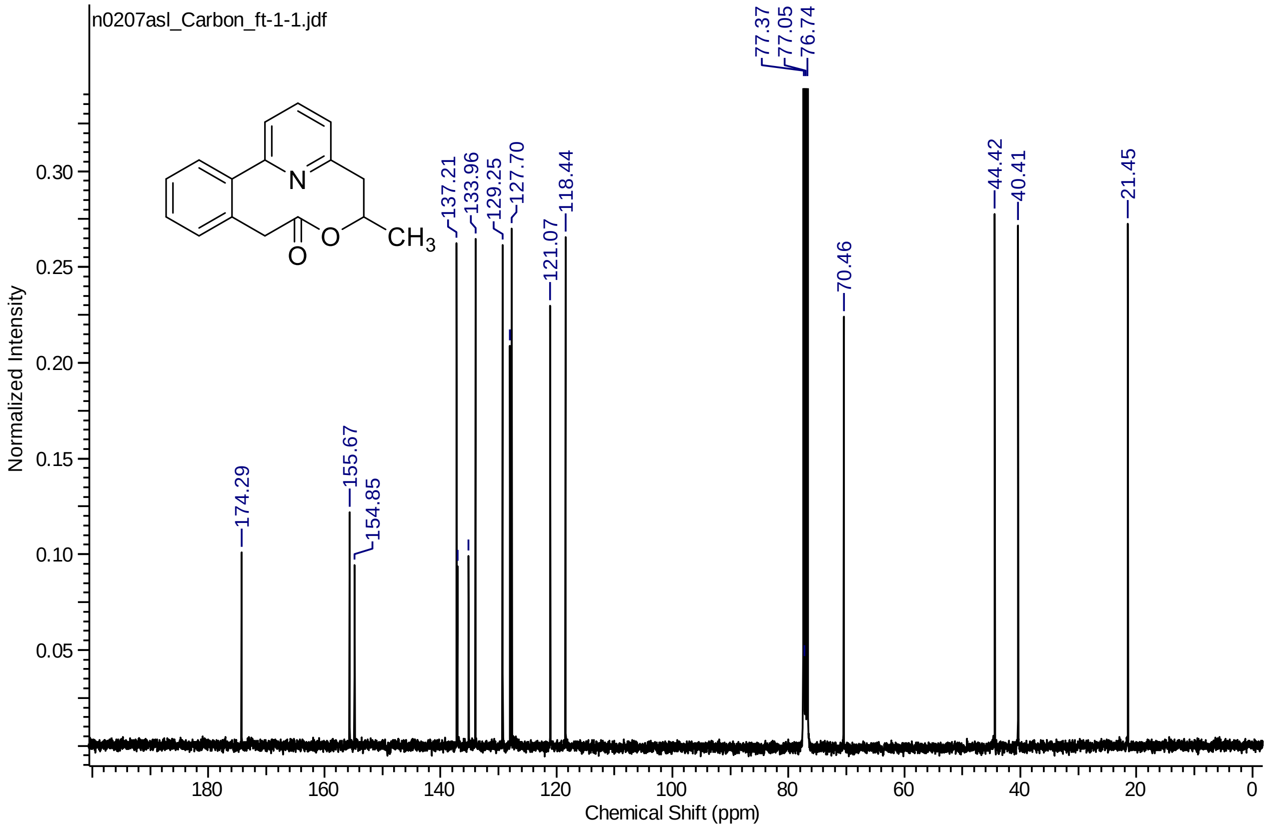

Methyl 2-(3'-(2-oxopropyl)-[1,1'-biphenyl]-2-yl)acetate (S6)

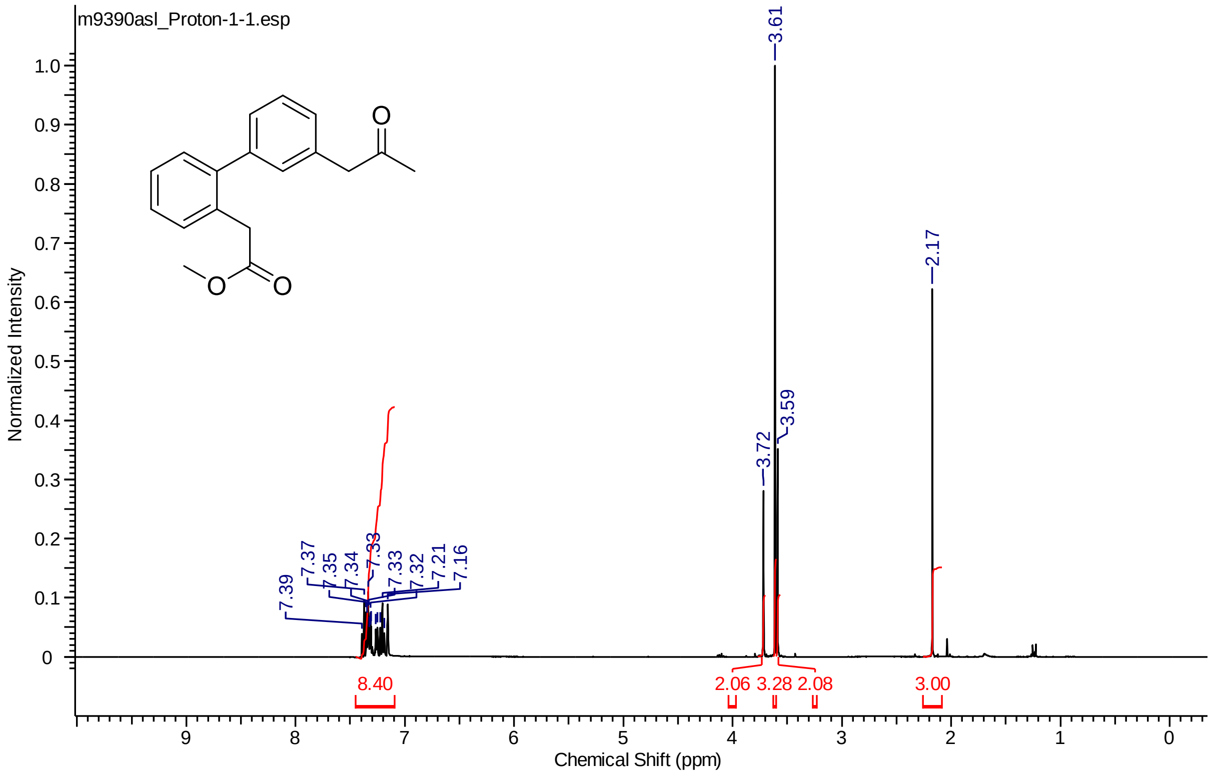

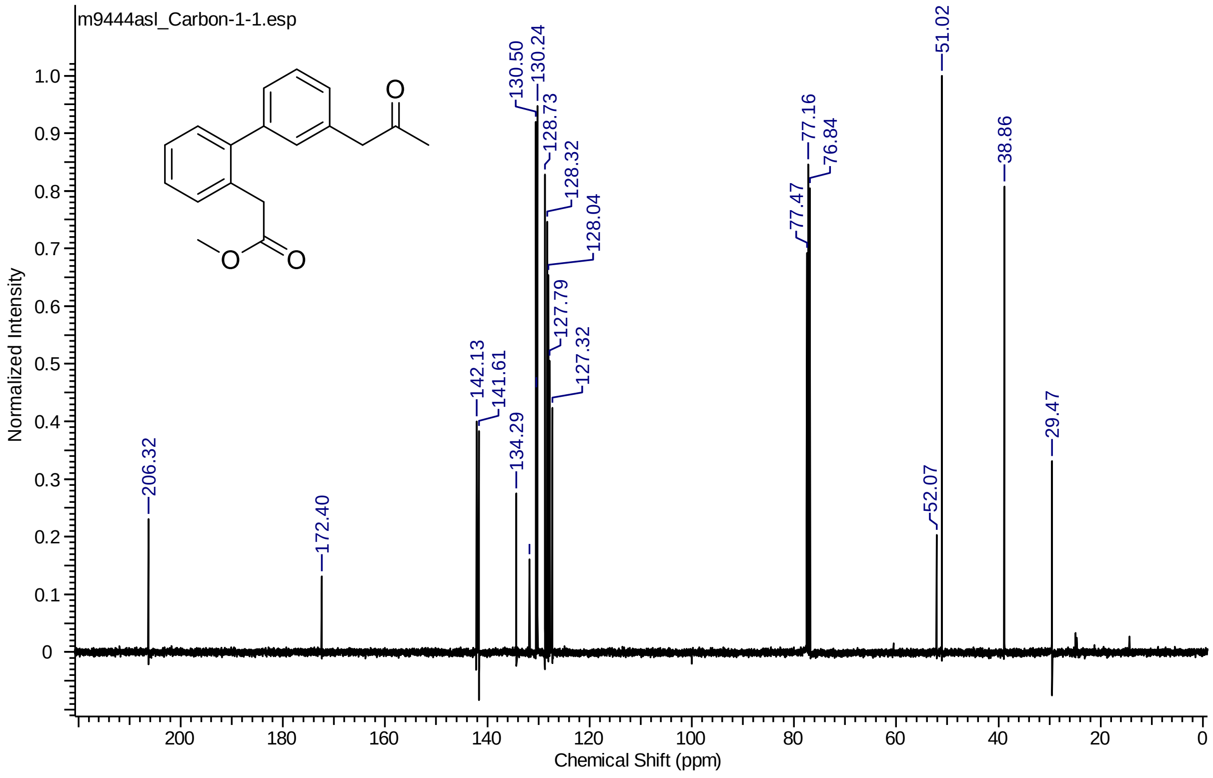

Methyl-2-(3'-(2-hydroxypropyl)-[1,1'-biphenyl]-2-yl)acetate (S7)

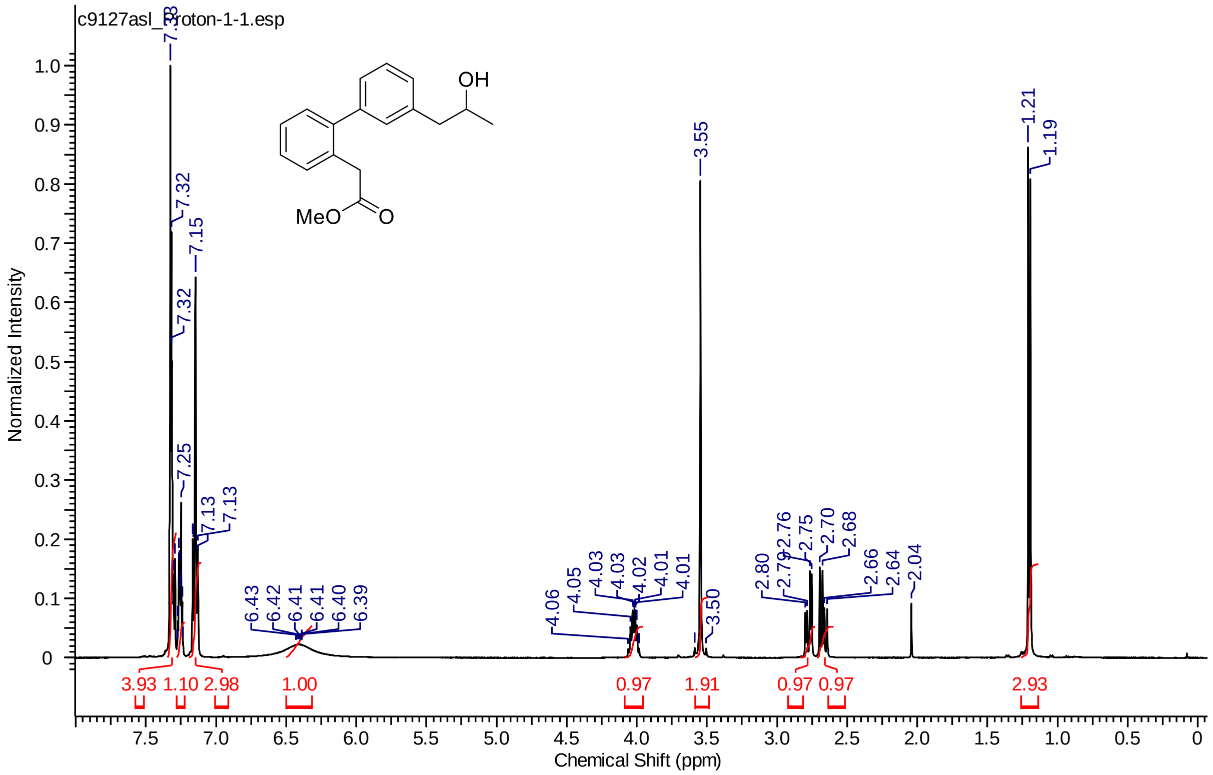

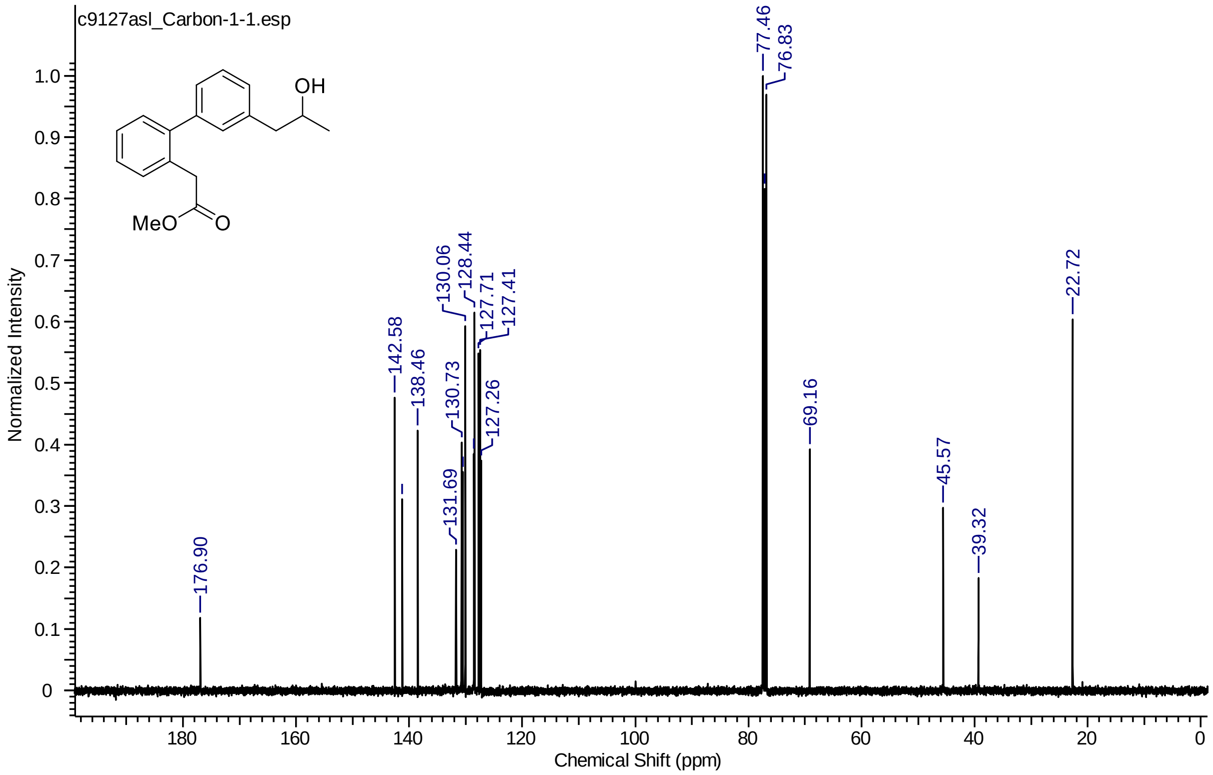

2-(3'-(2-Hydroxypropyl)-[1,1'-biphenyl]-2-yl) acetic acid (10)

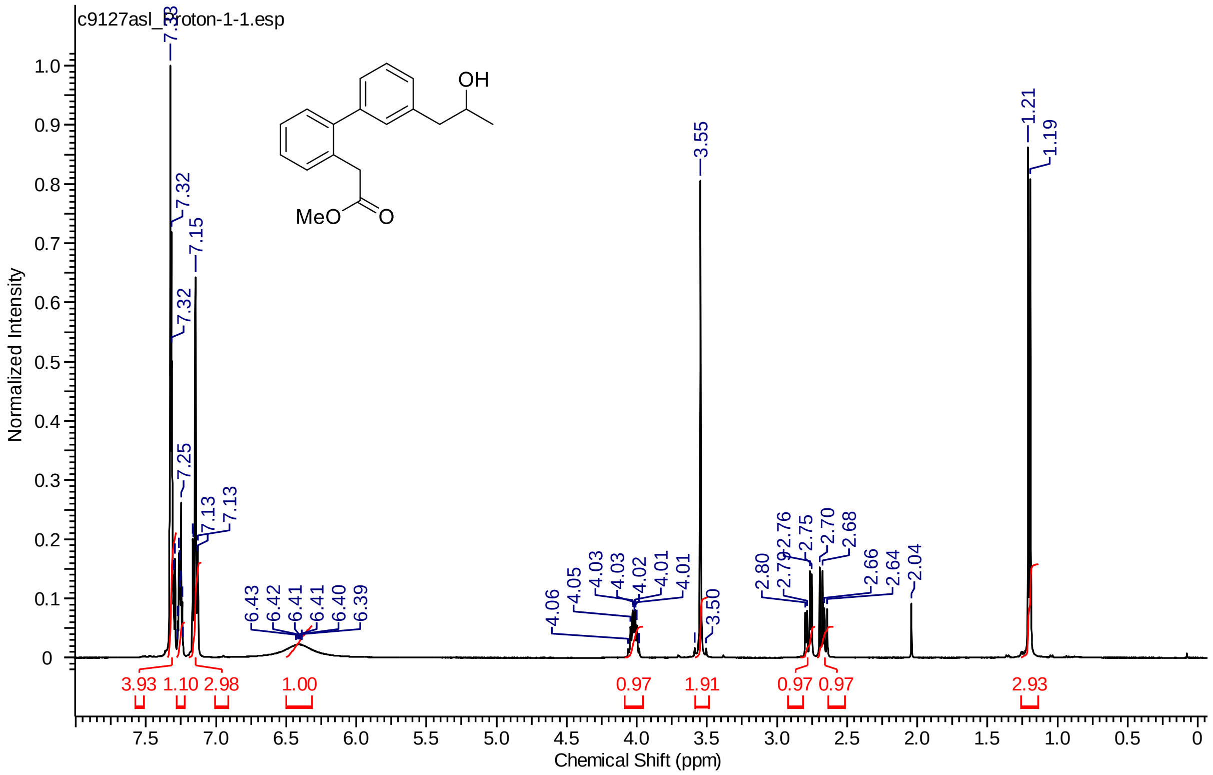

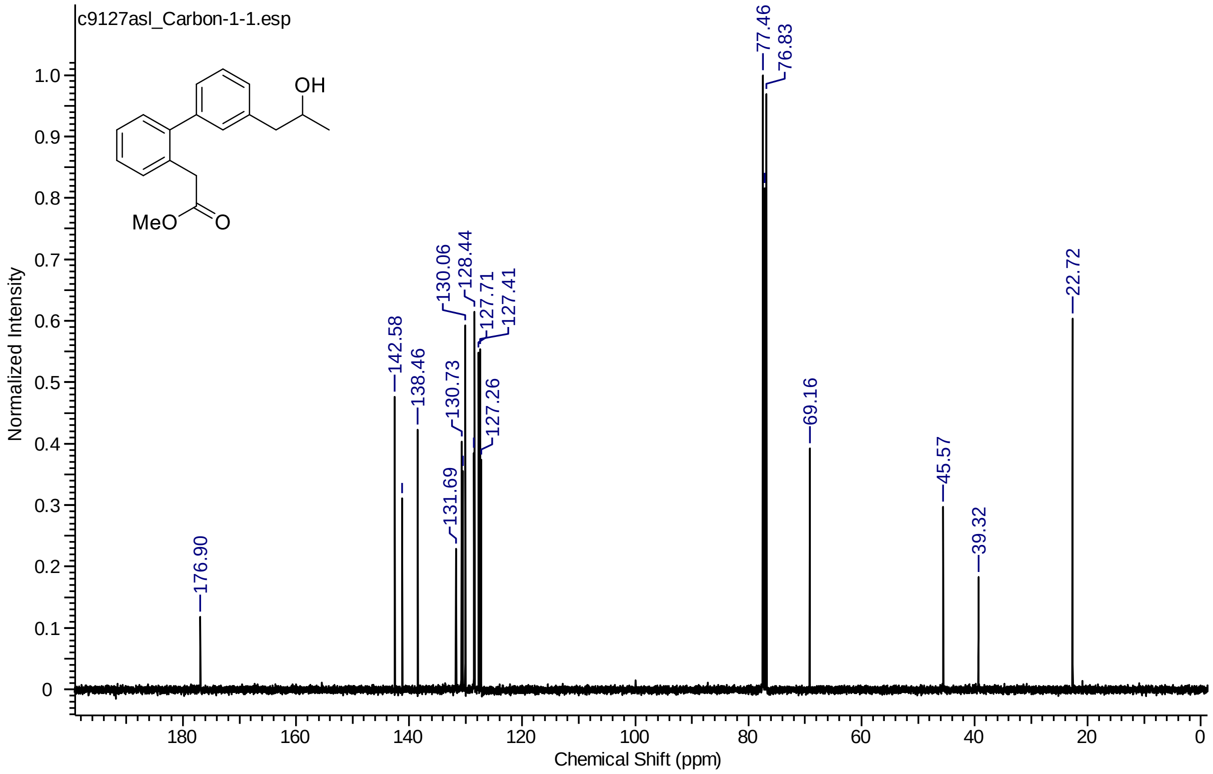

4-Methyl-4,5-dihydro-6,10-(metheno)benzo[d][1]oxacyclododecin-2(1H)-one (S8)

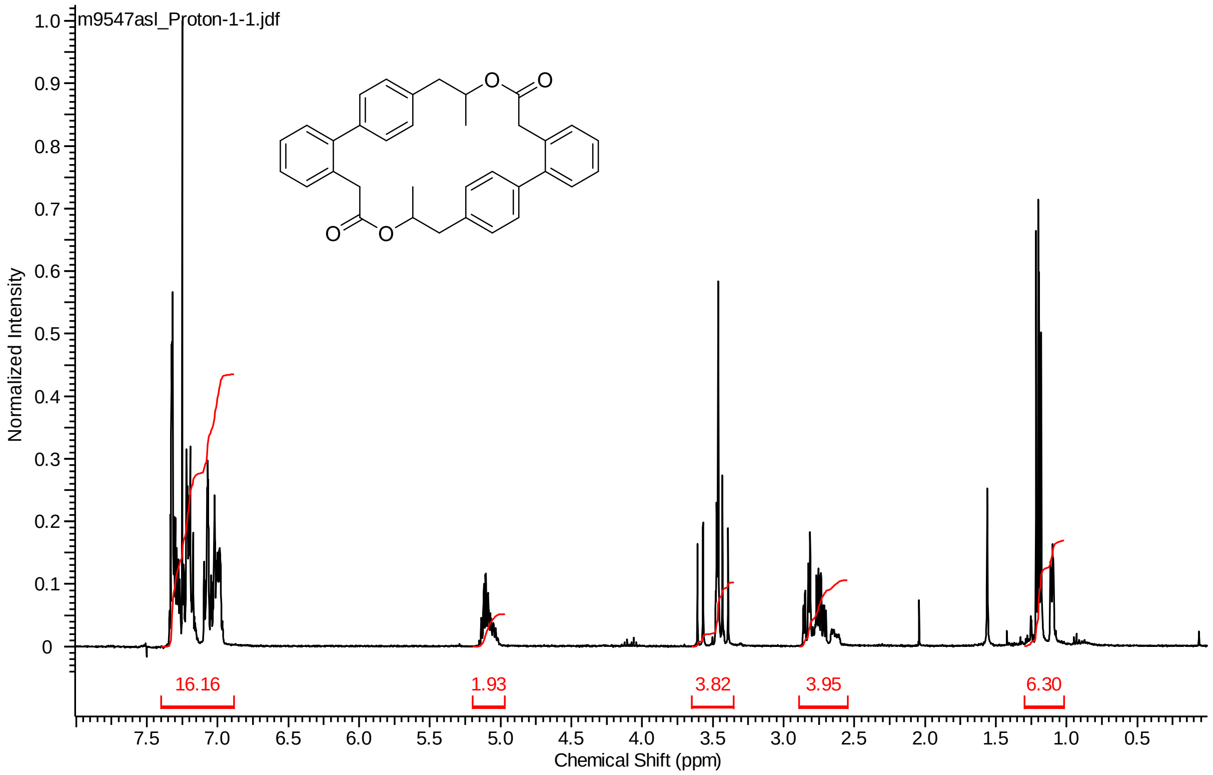

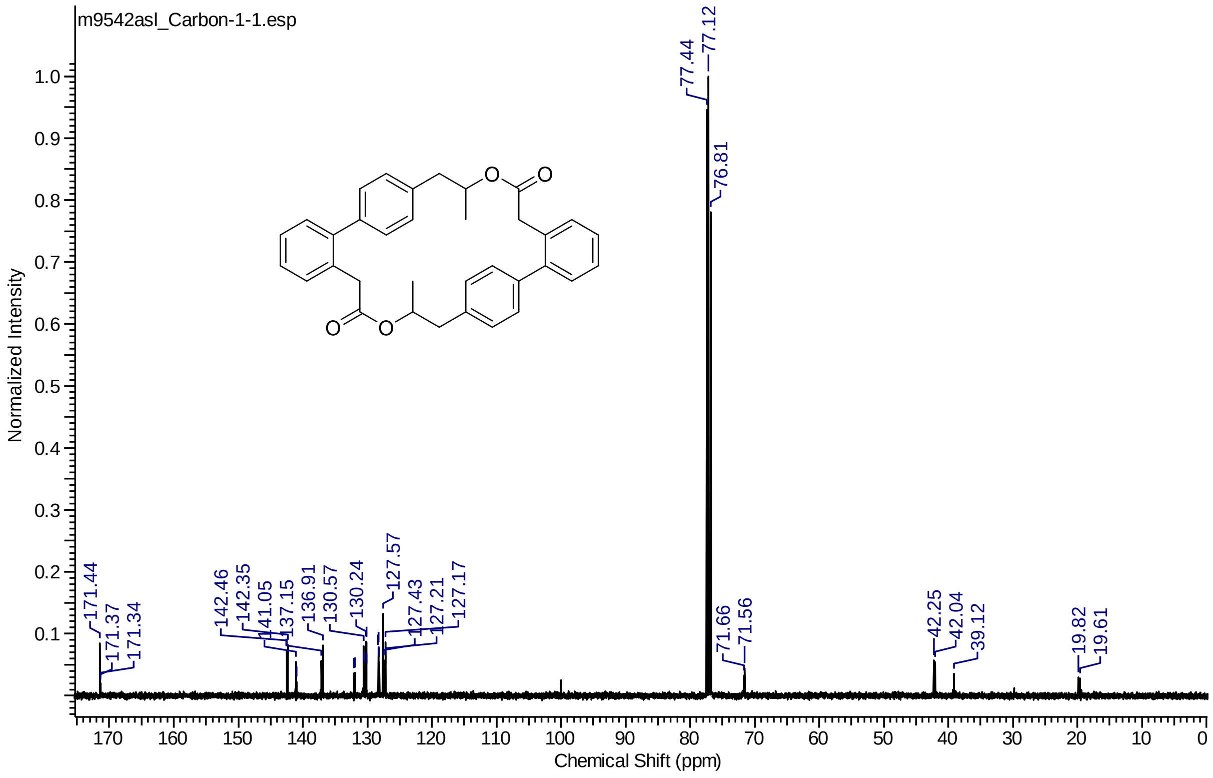

2-(((3-Hydroxypropyl)thio)methyl)benzoic acid (12)

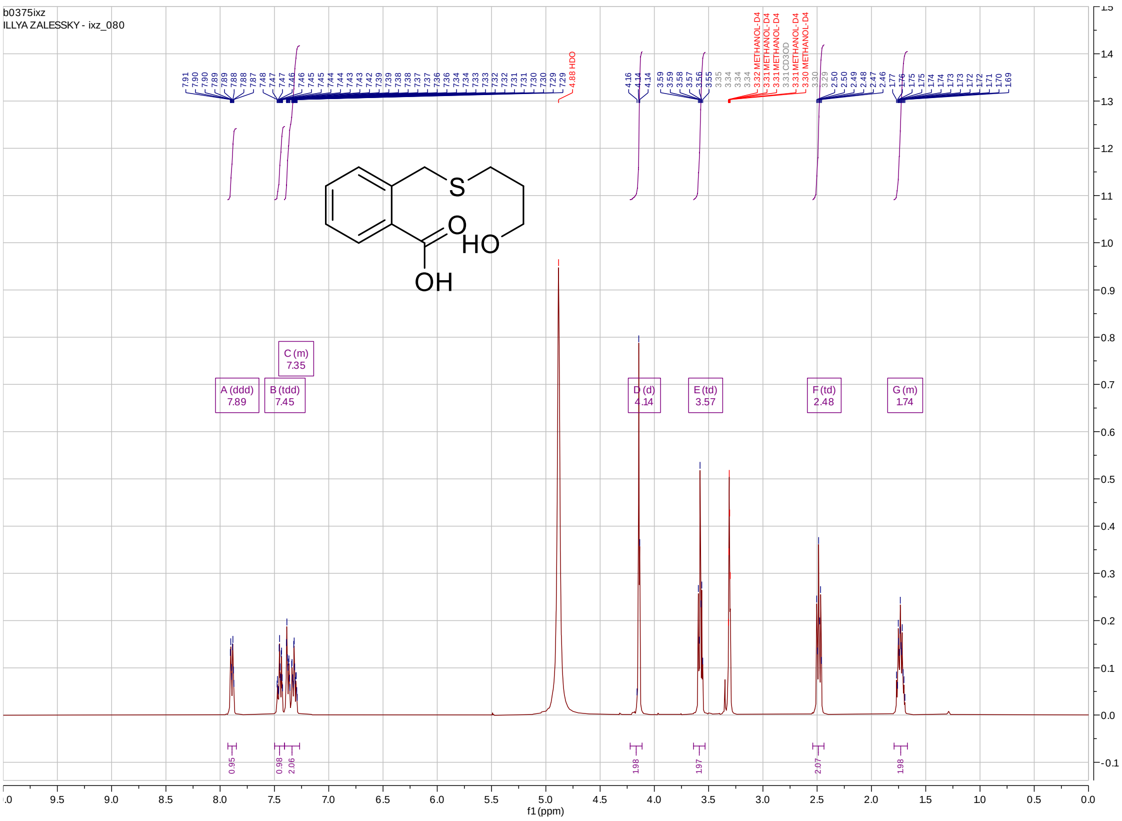

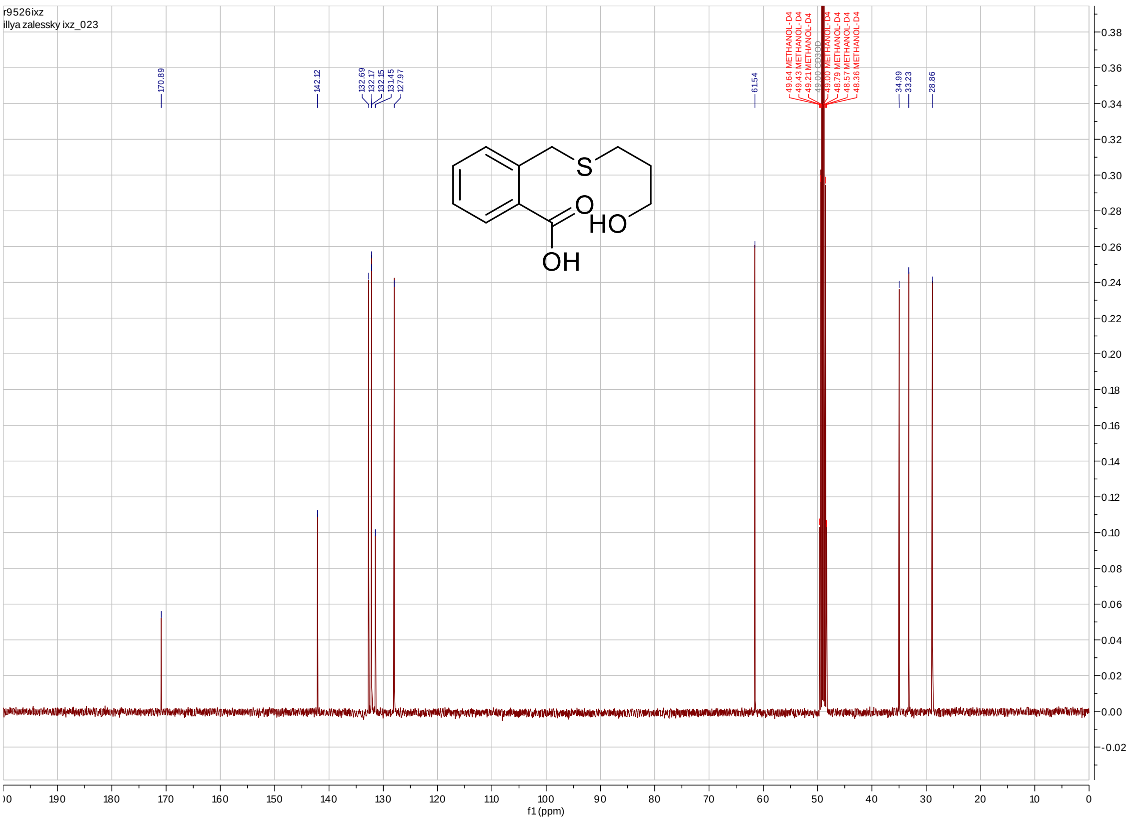

4,5-Dihydro-3*H*-benzo[*g*][1,5]oxathionin-1(7*H*)-one (13)

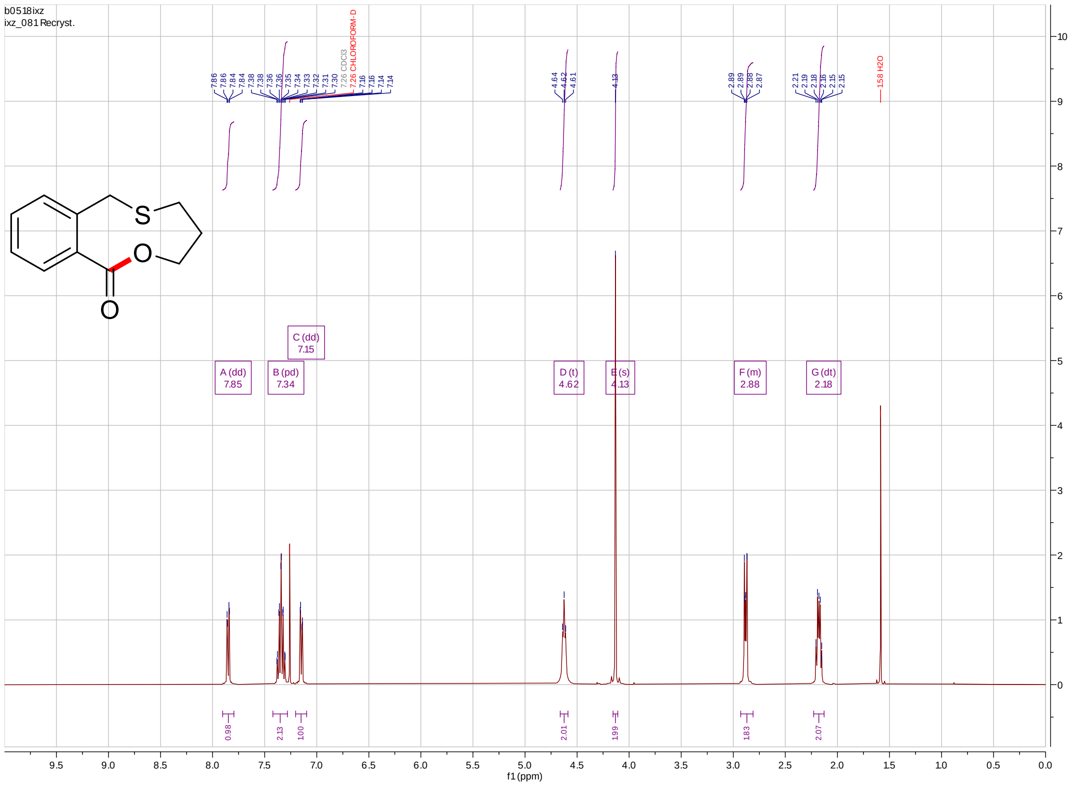

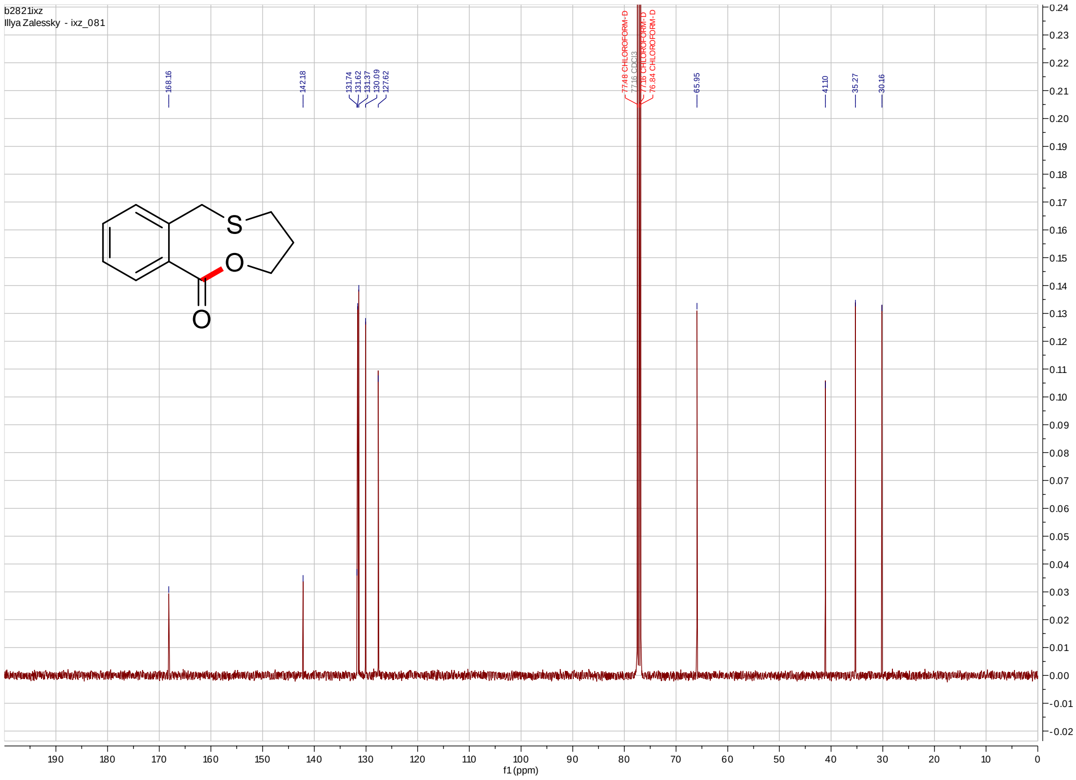

2-(5-Hydroxypentyl)benzoic acid (14).

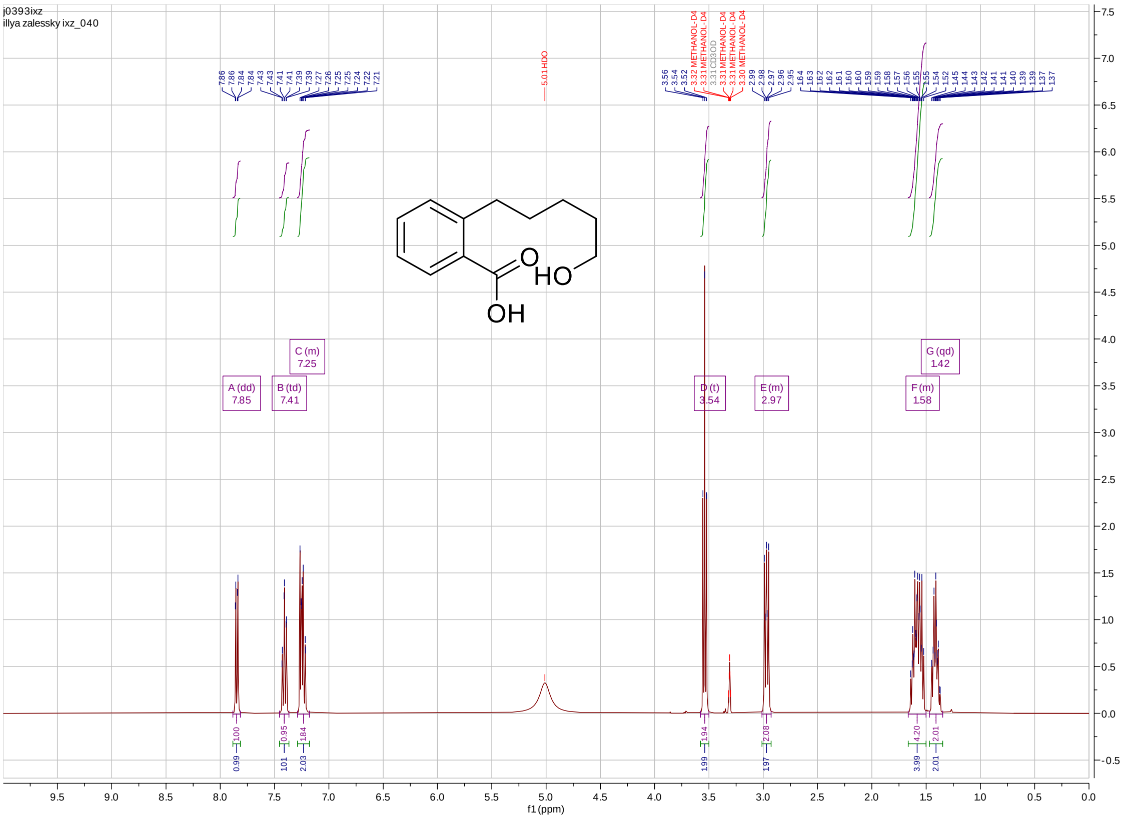

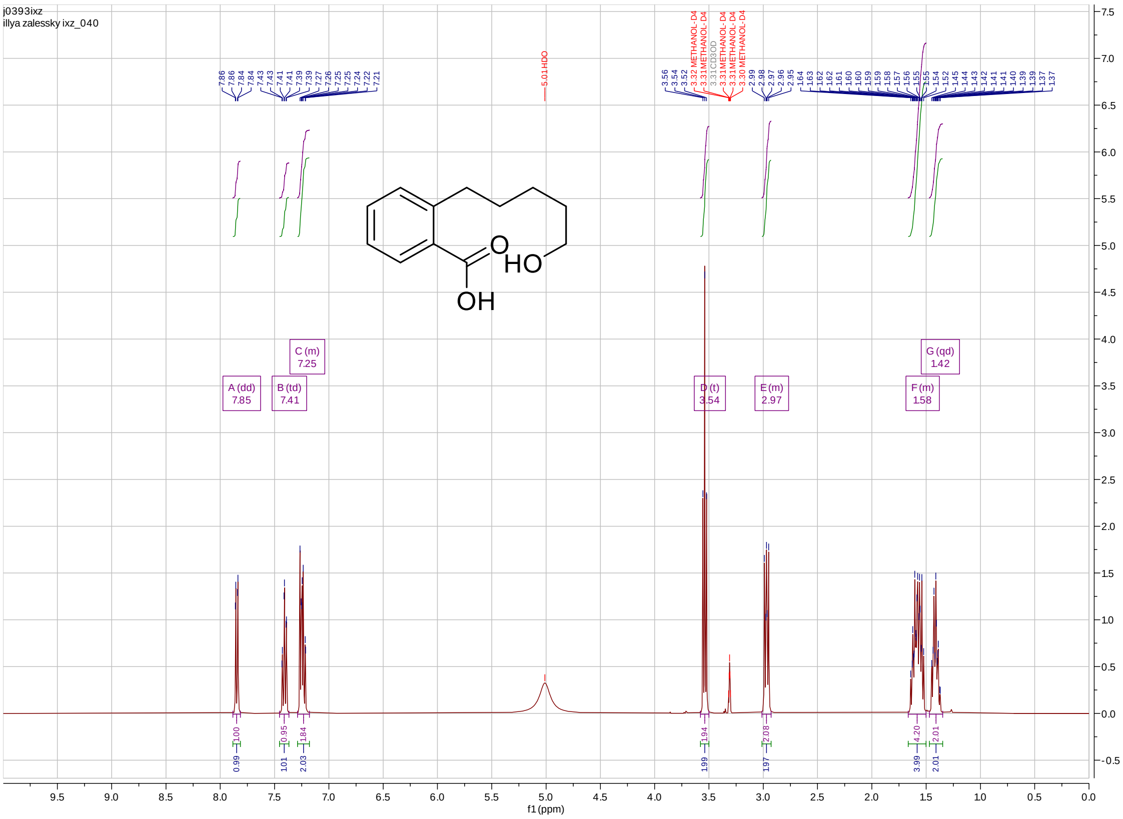

4,5,6,7-Tetrahydrobenzo[*c*]oxonin-1(3*H*)-one (15)

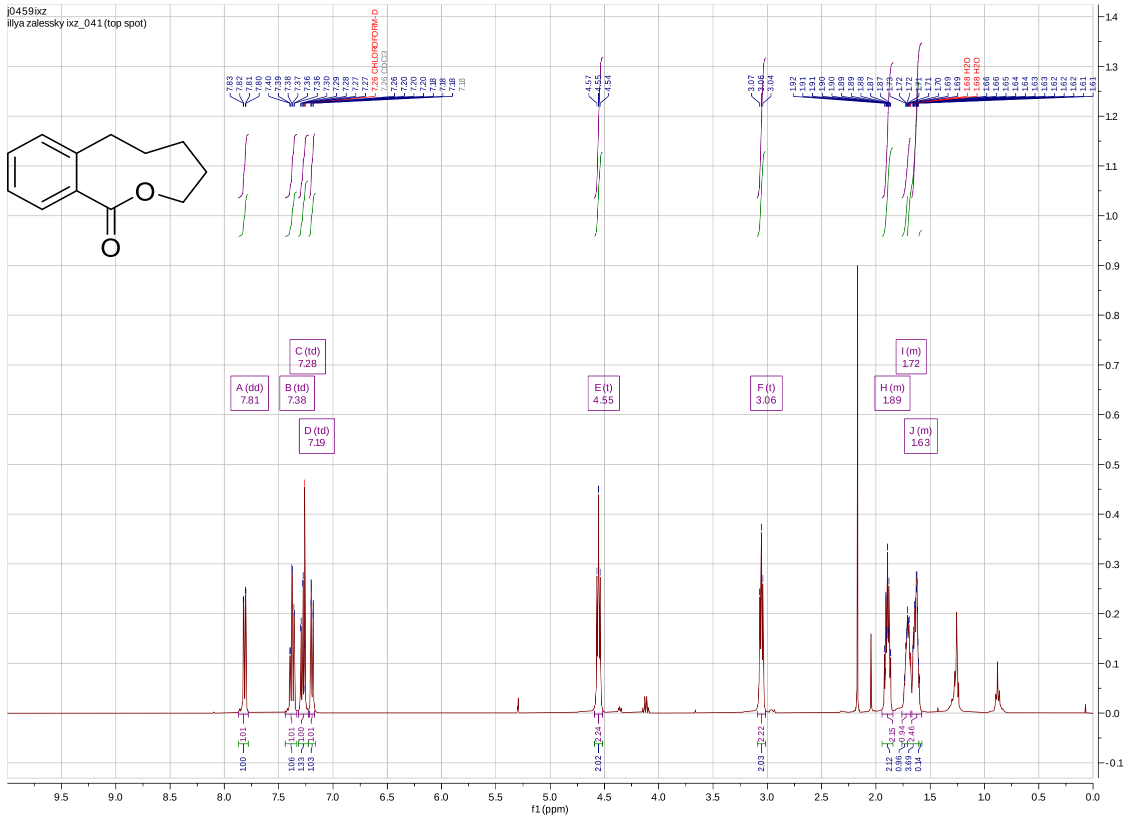

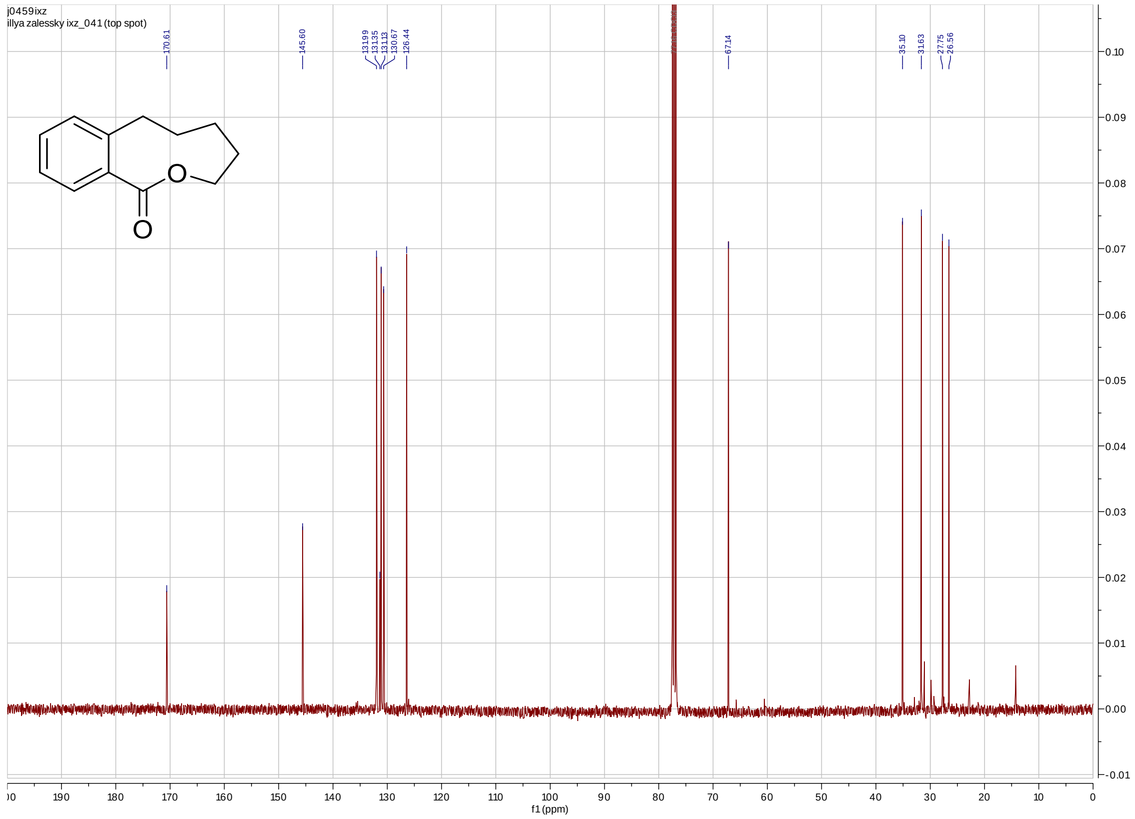

8,9,10,11,19,20,21,22-Octahydrodibenzo[*c*,*l*][1,10]dioxacyclooctadecine-5,16(7*H*,18*H*)-dione (S11)

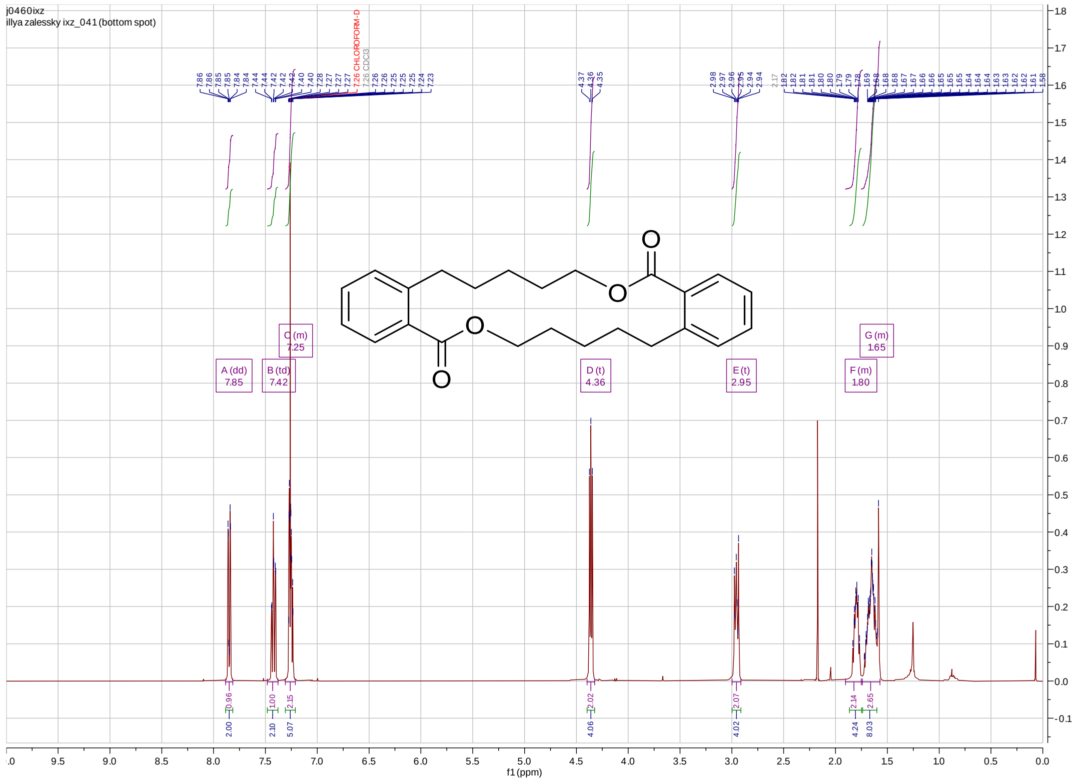

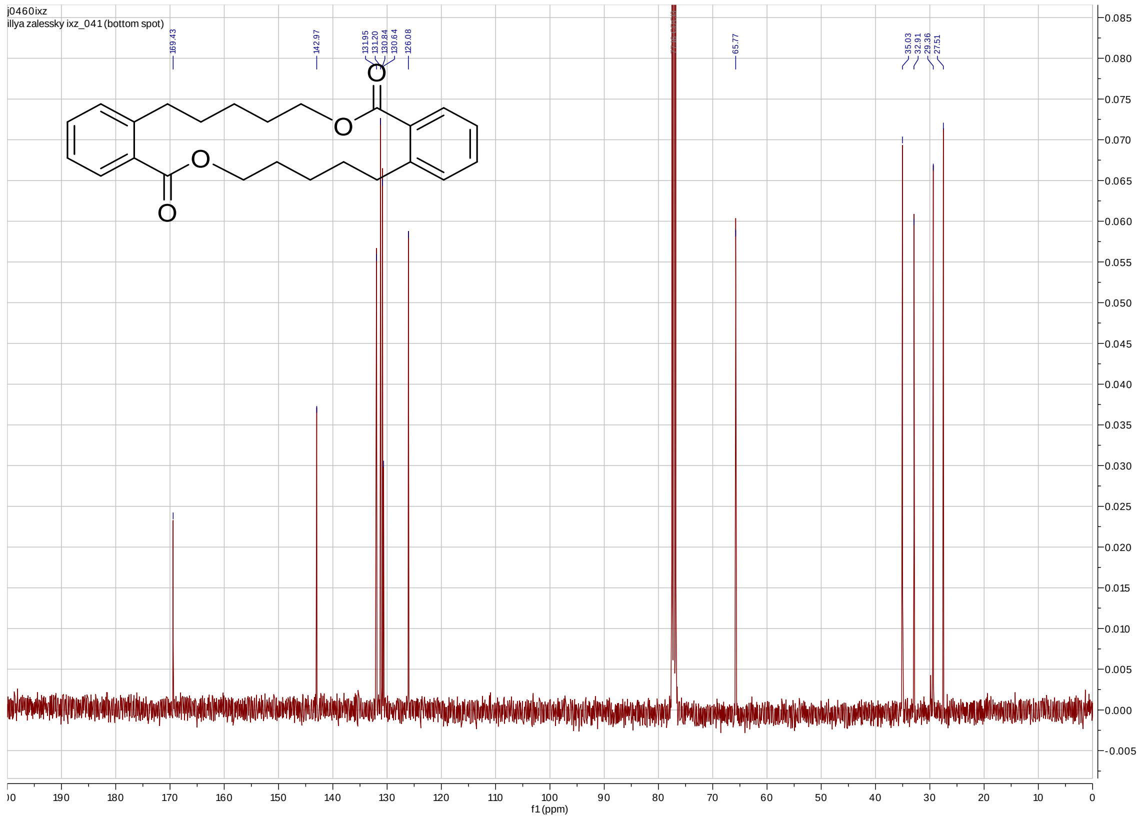

3-(Phenyl(2-(phenylamino)ethyl)amino)propan-1-ol (16)

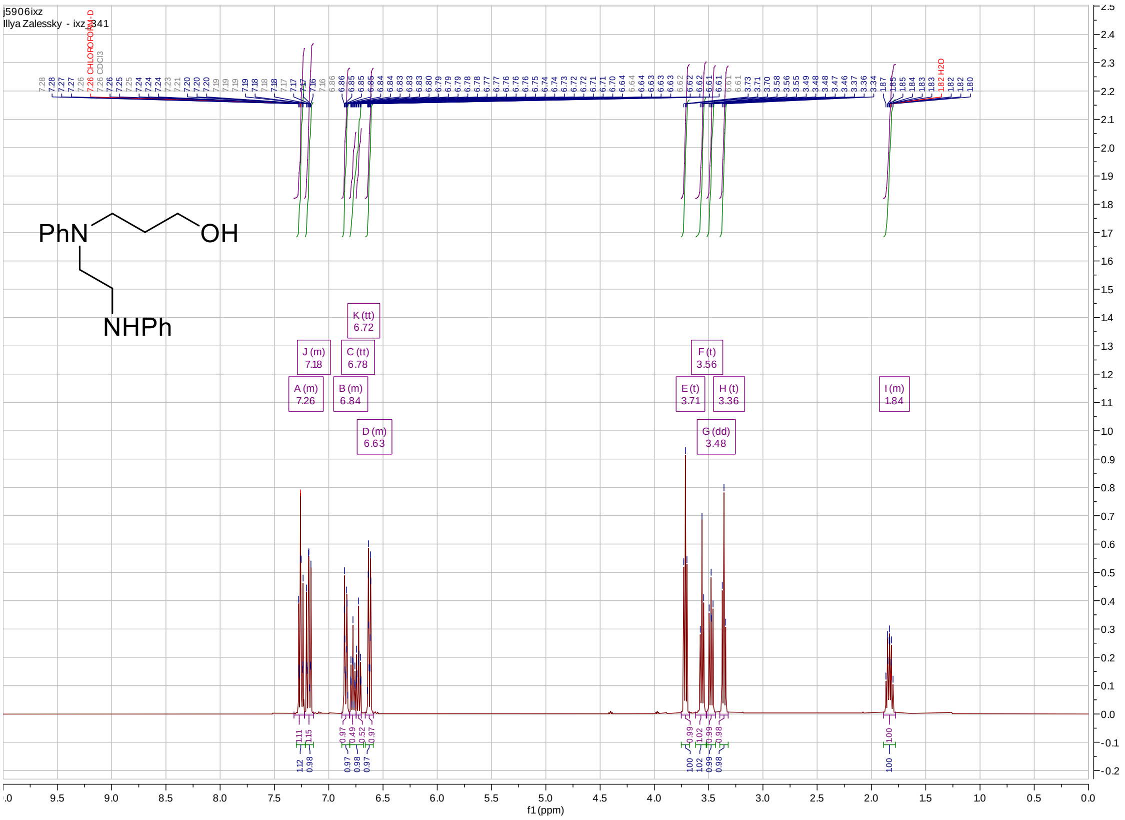

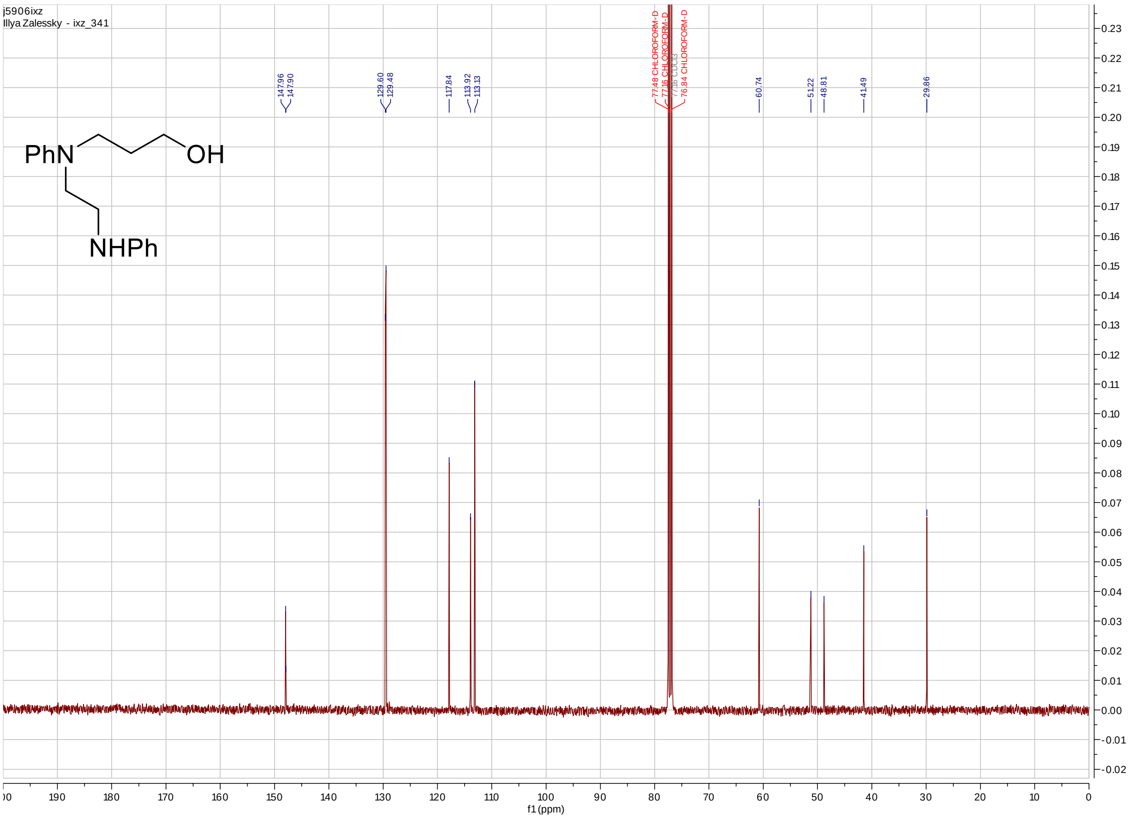

3,6-Diphenyl-1,3,6-oxadiazecan-2-one (17)

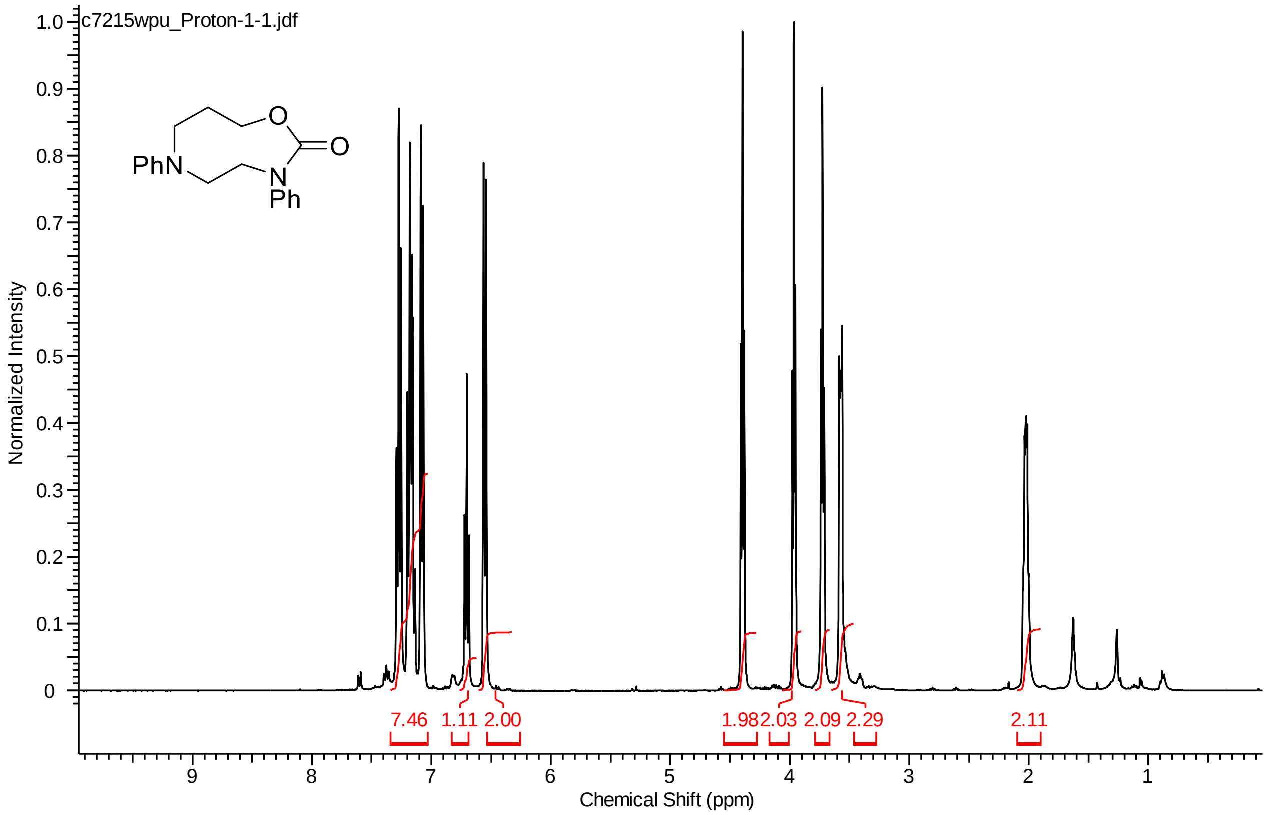

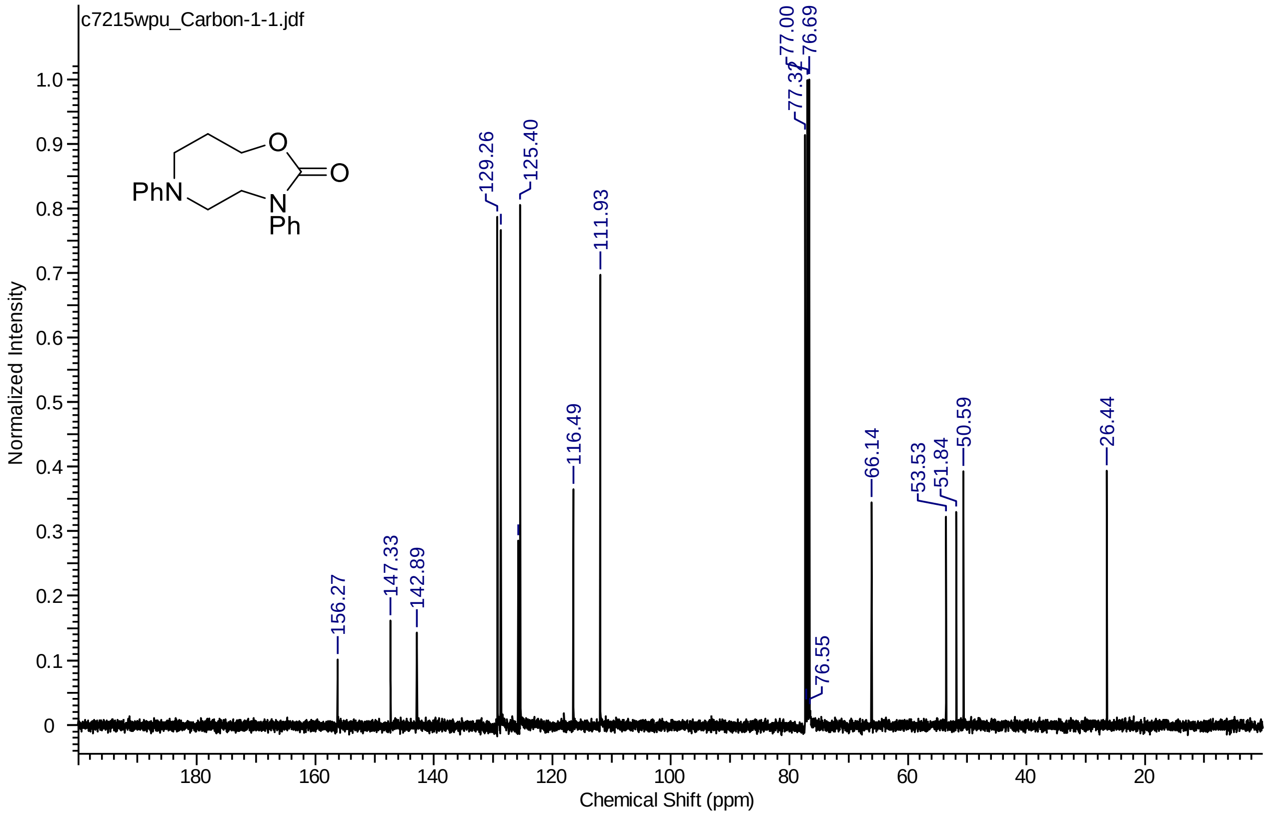

6-(Phenylamino)hexan-1-ol (18)

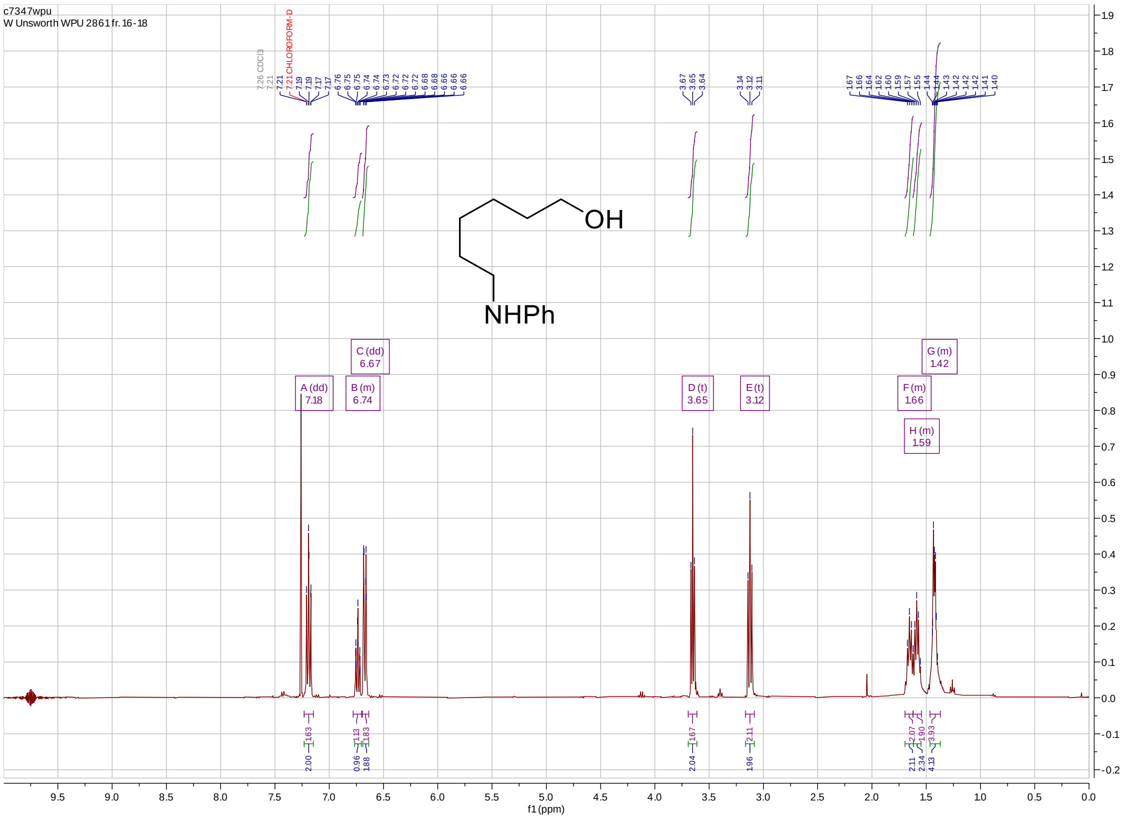

(6-Hydroxyhexyl)(phenyl)carbamic chloride (20)

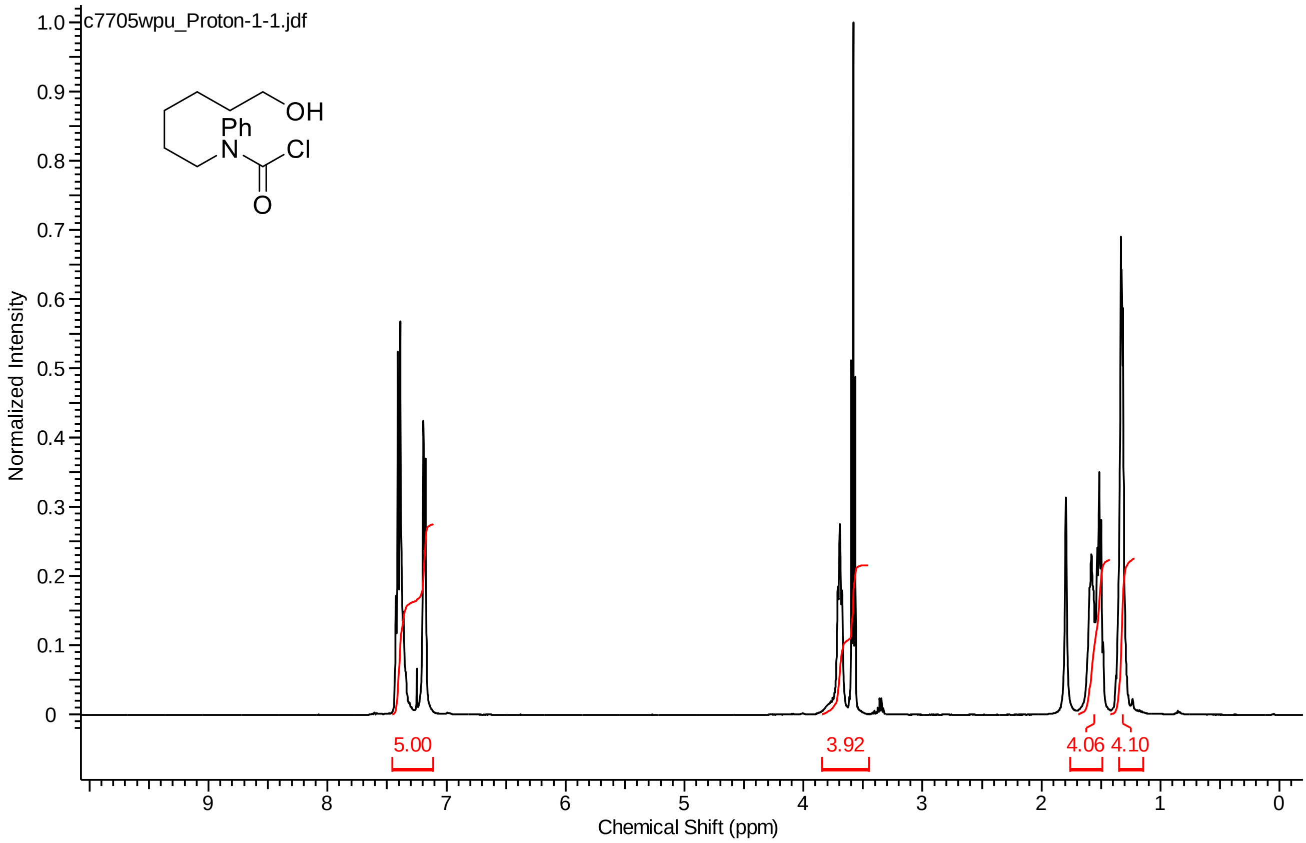

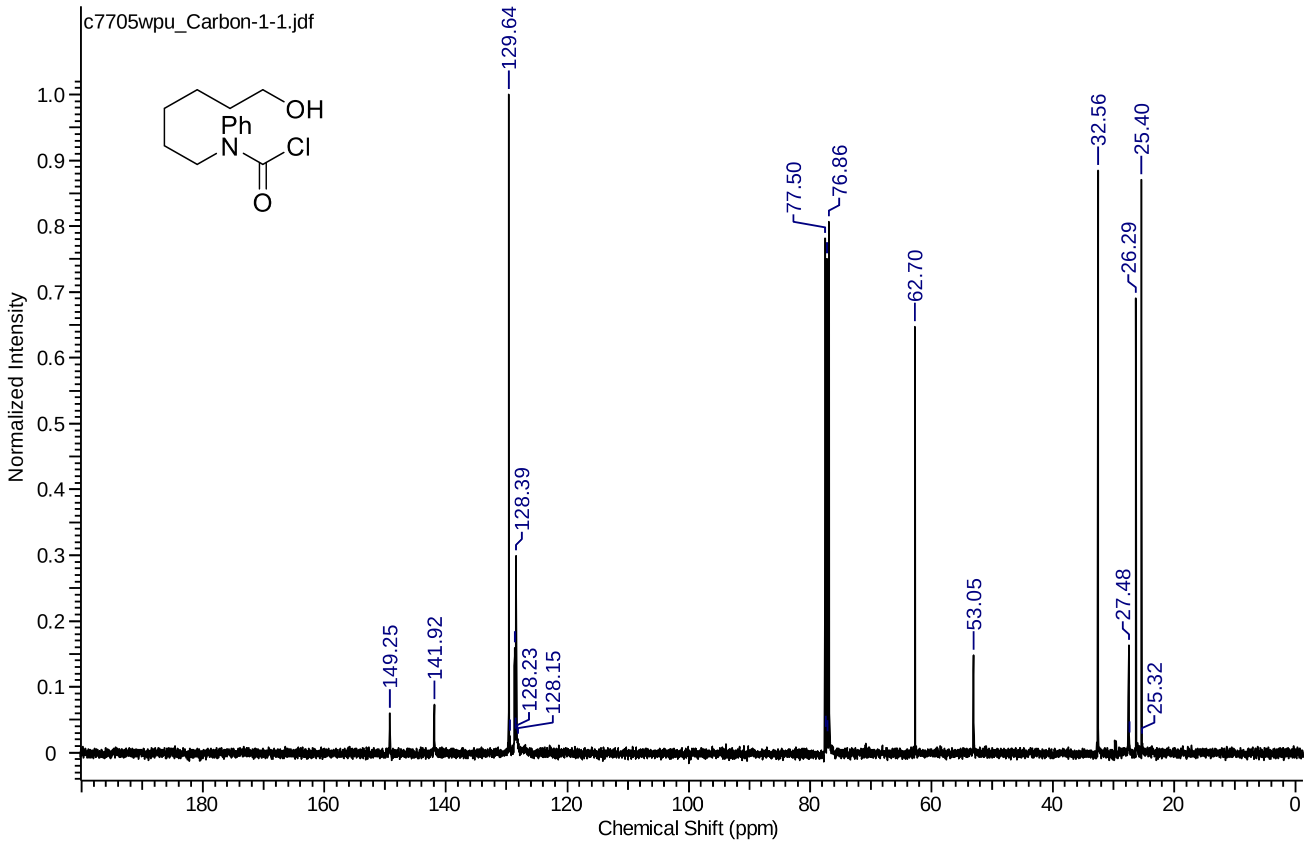

3,6-Diphenyl-1,3,6-oxadiazonane-2-thione (21)

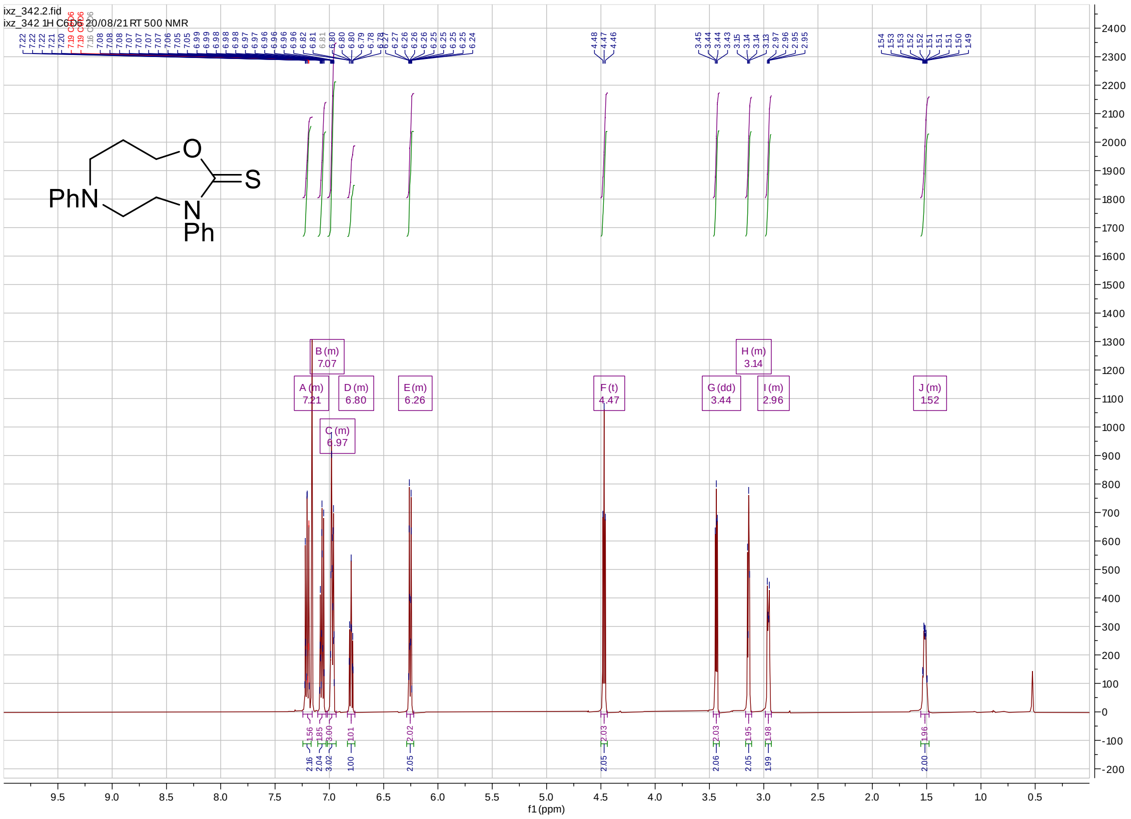

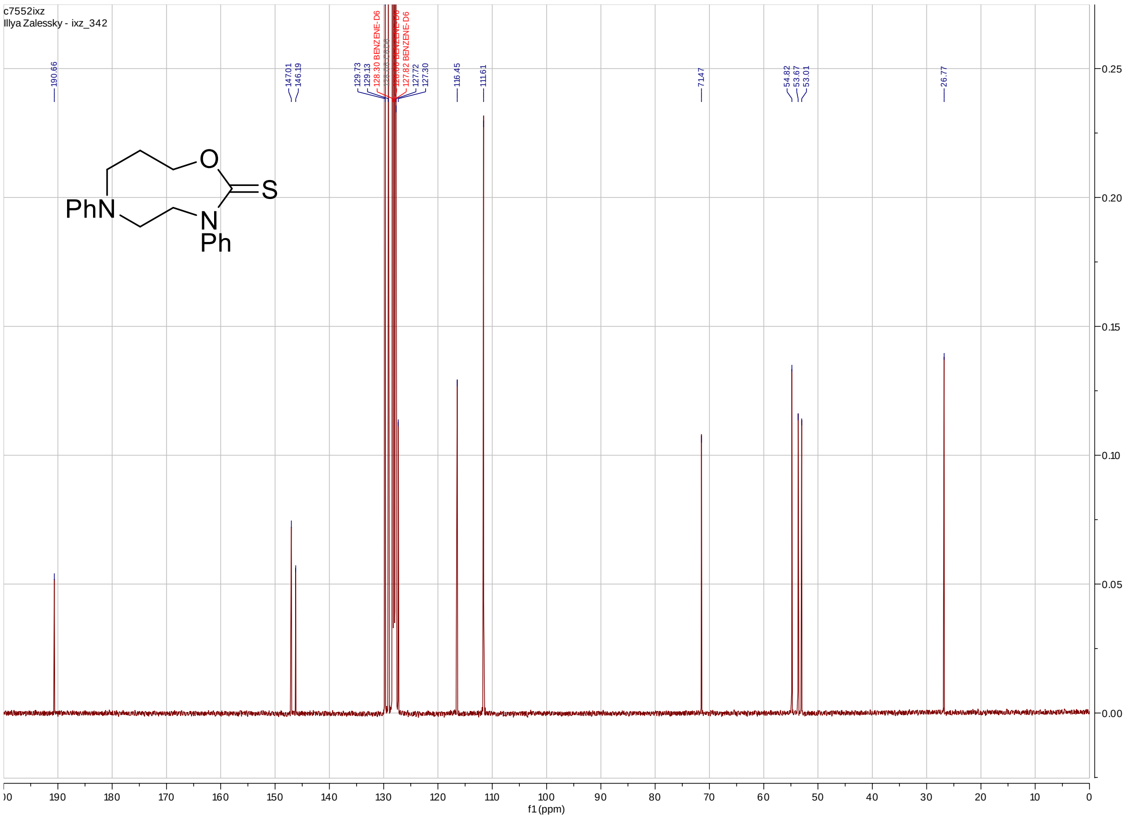

(6-Hydroxyhexyl)(phenyl)carbamothioic chloride (23)

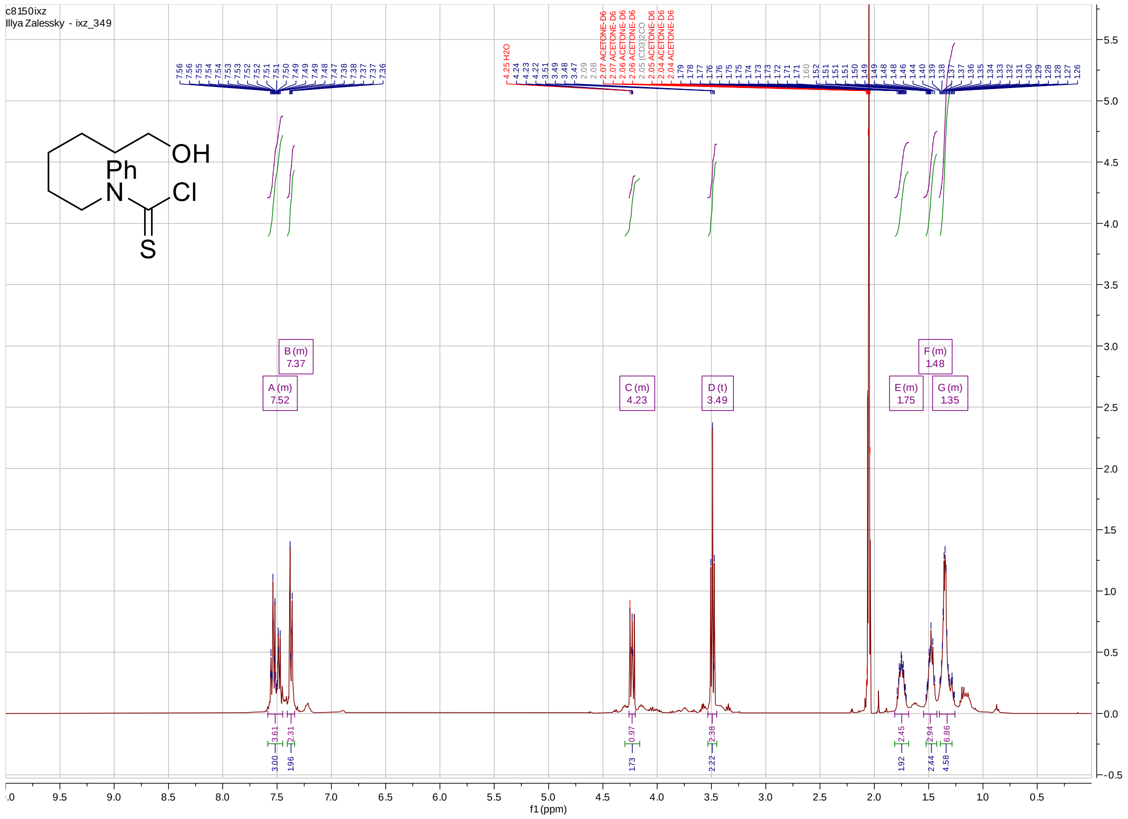

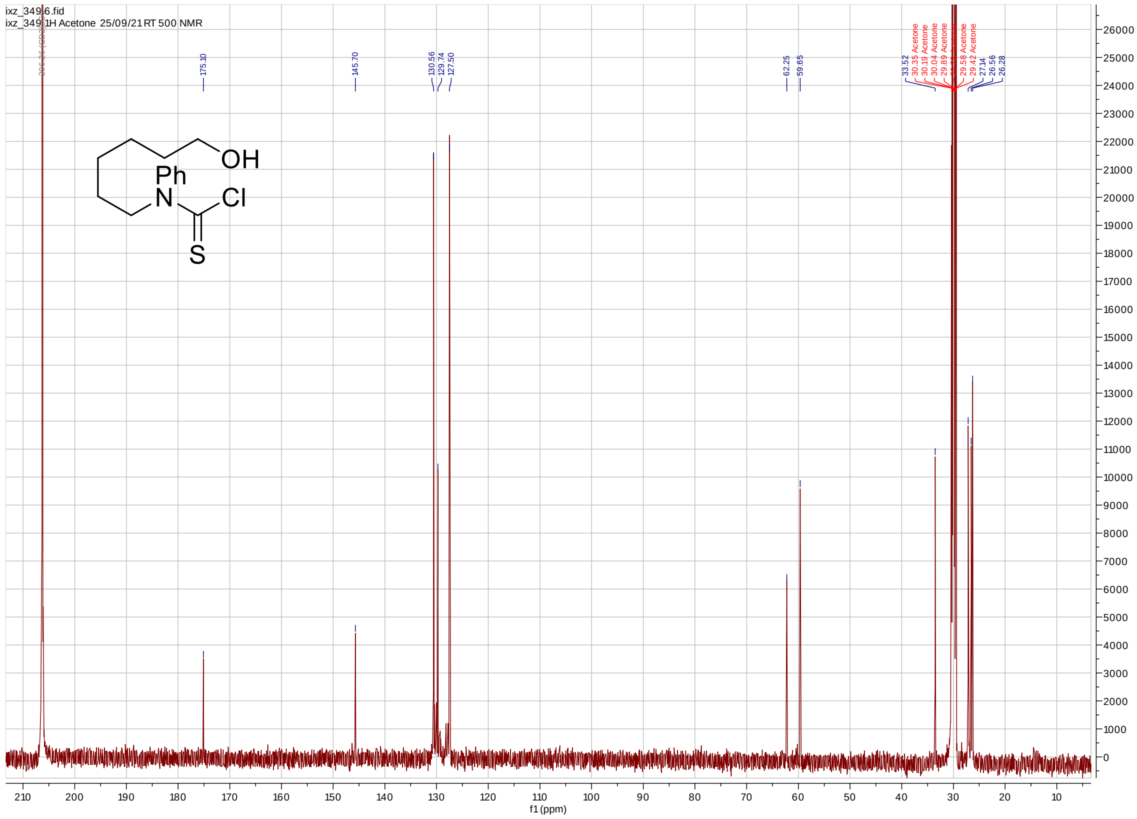

3-(Methyl(2-(methylamino)phenyl)amino)propan-1-ol (24)

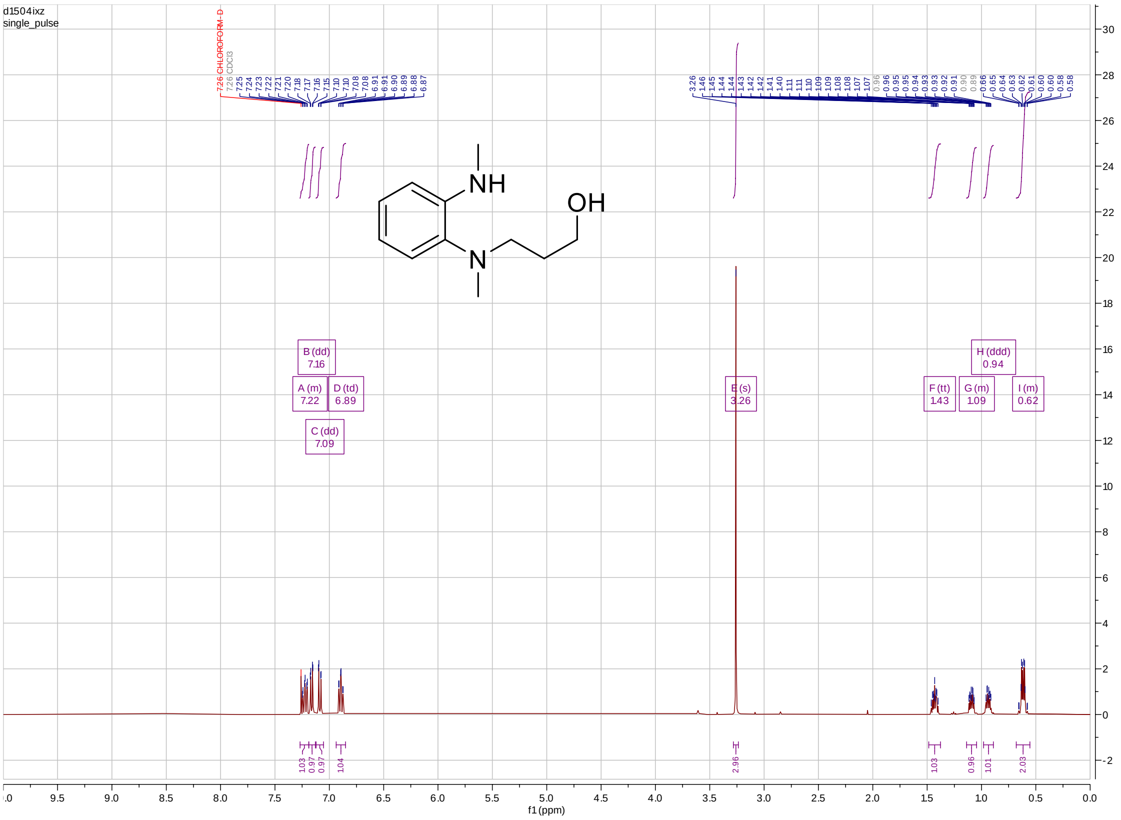

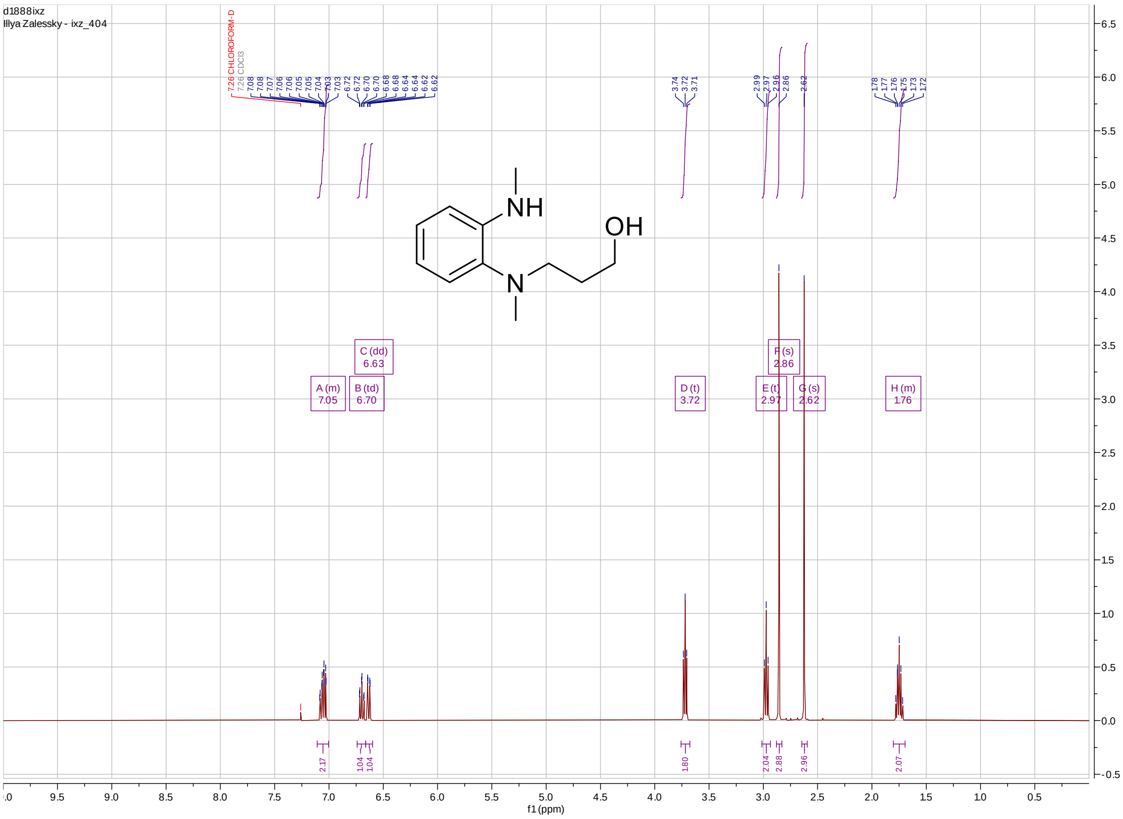

1,8-Dimethyl-5,6,7,8-tetrahydro-1H-benzo[e][1,4,7]oxadiazecine-2,3-dione (25)

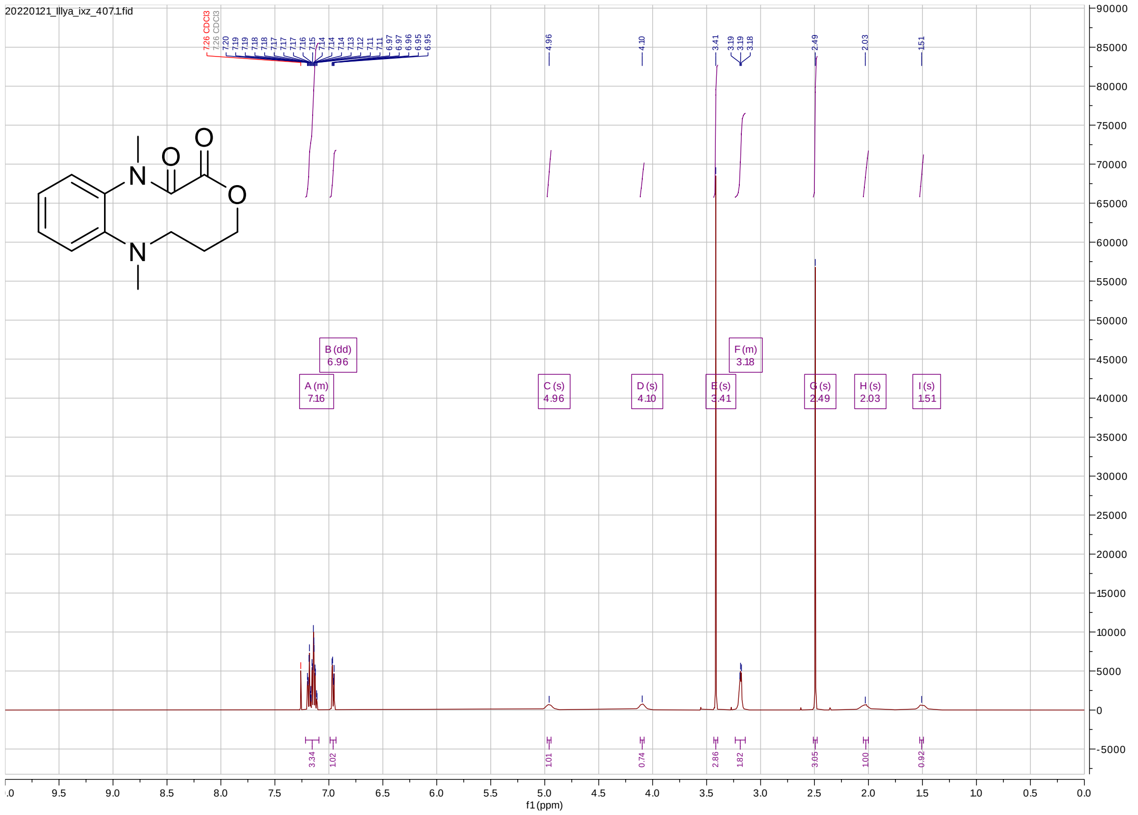

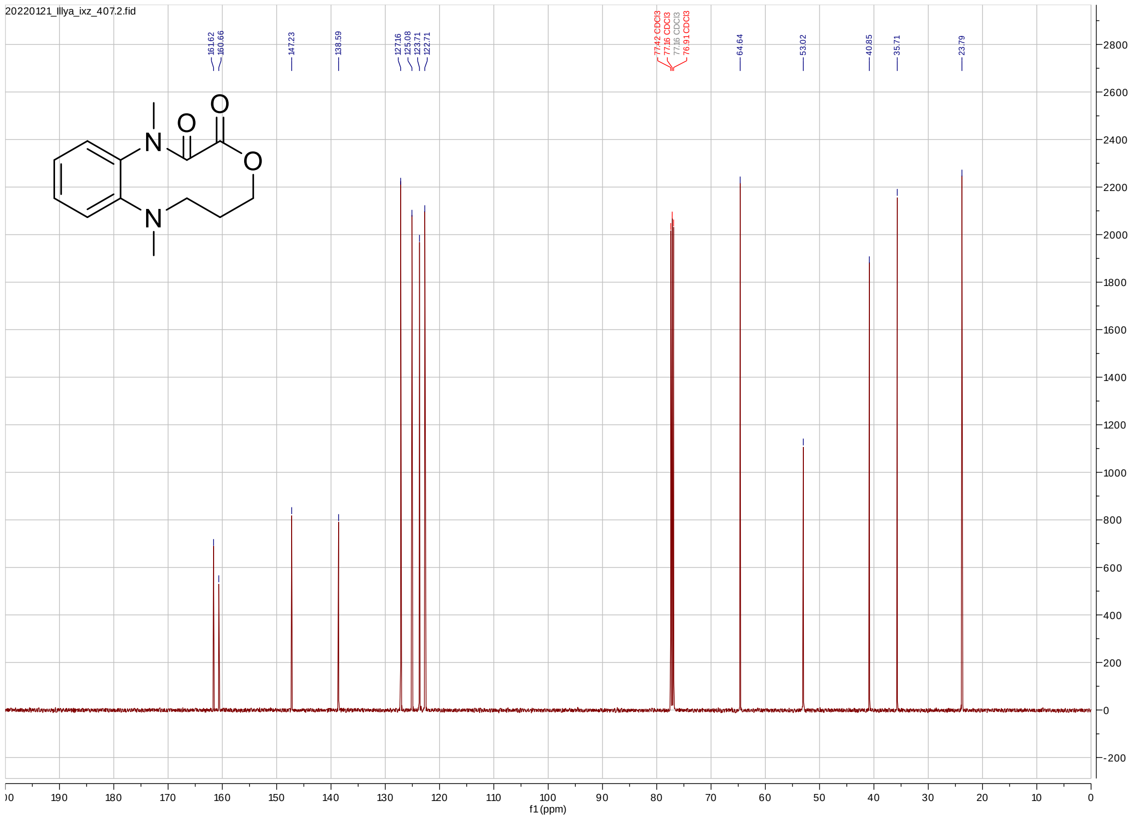

4-(2-(Methylamino)phenyl)but-3-yn-1-ol (S12)

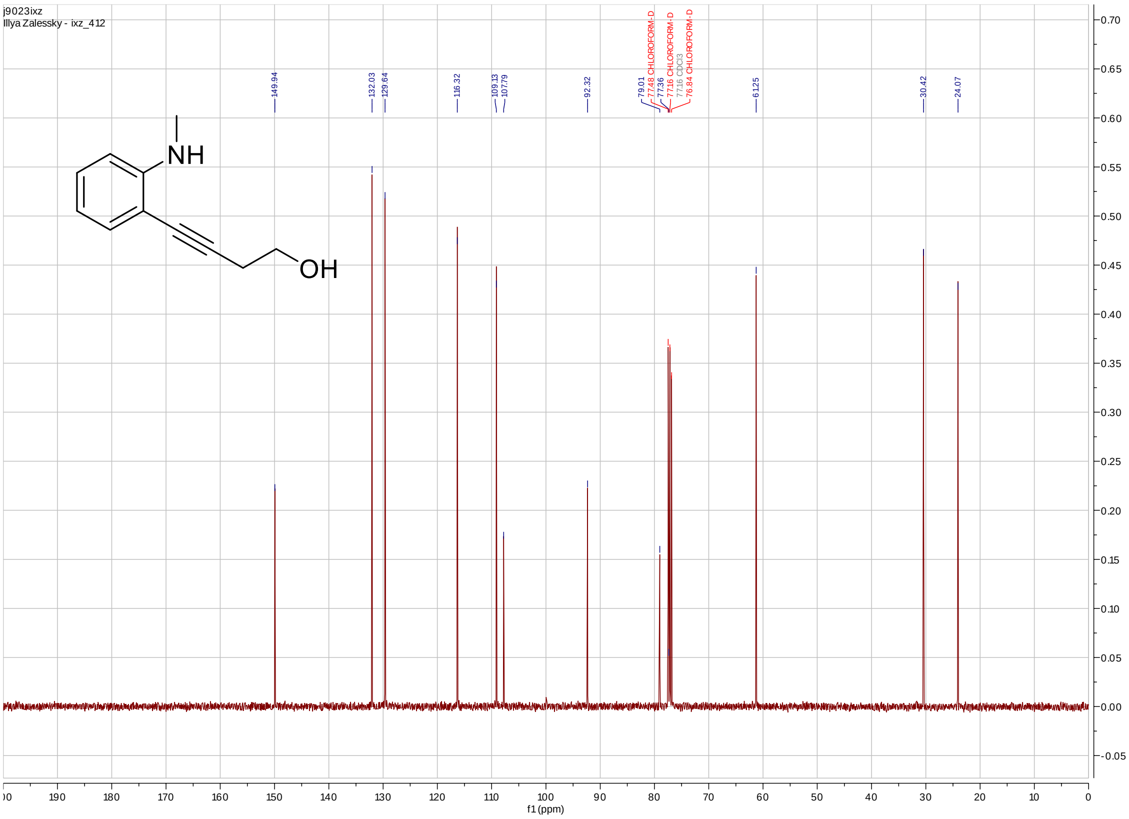

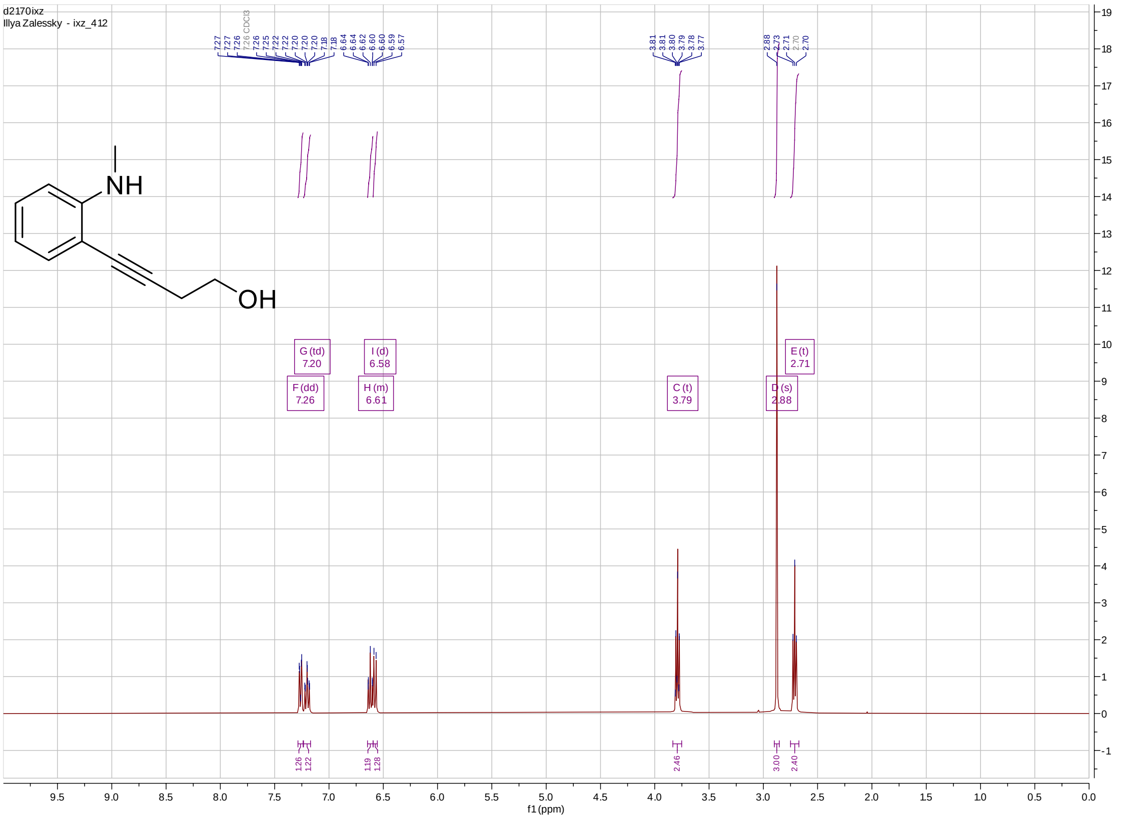

4-(2-(Methylamino)phenyl)butan-1-ol (26)

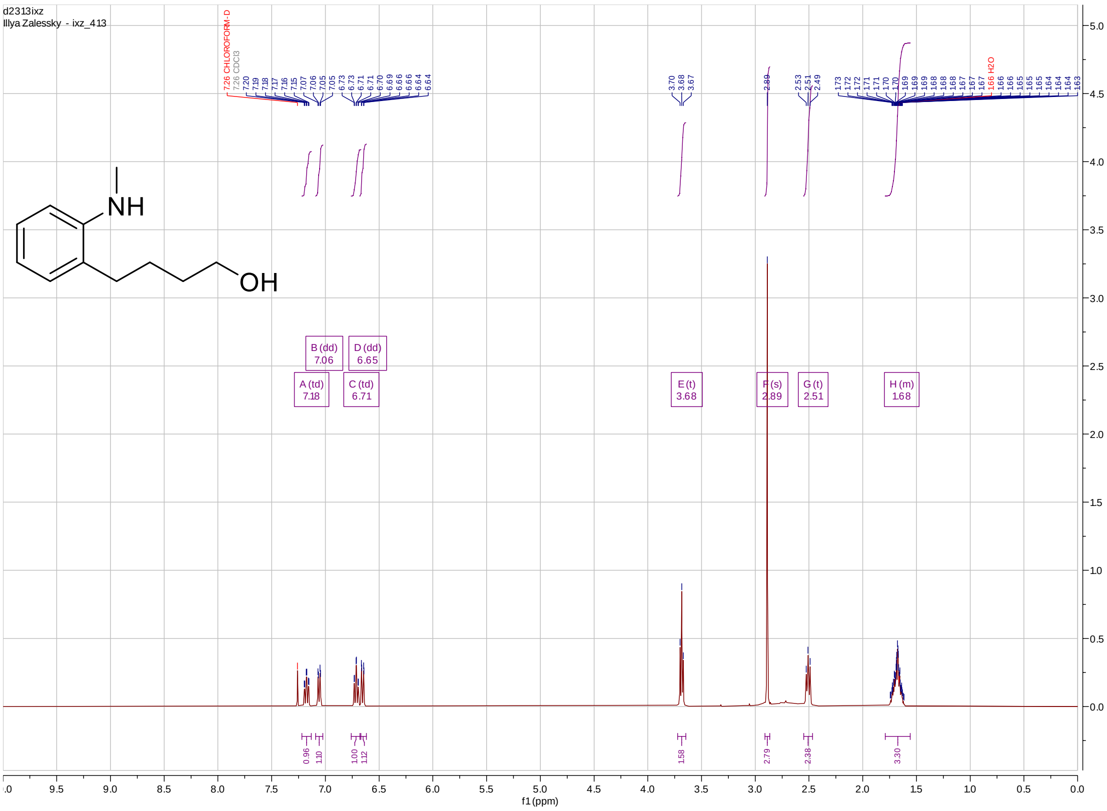

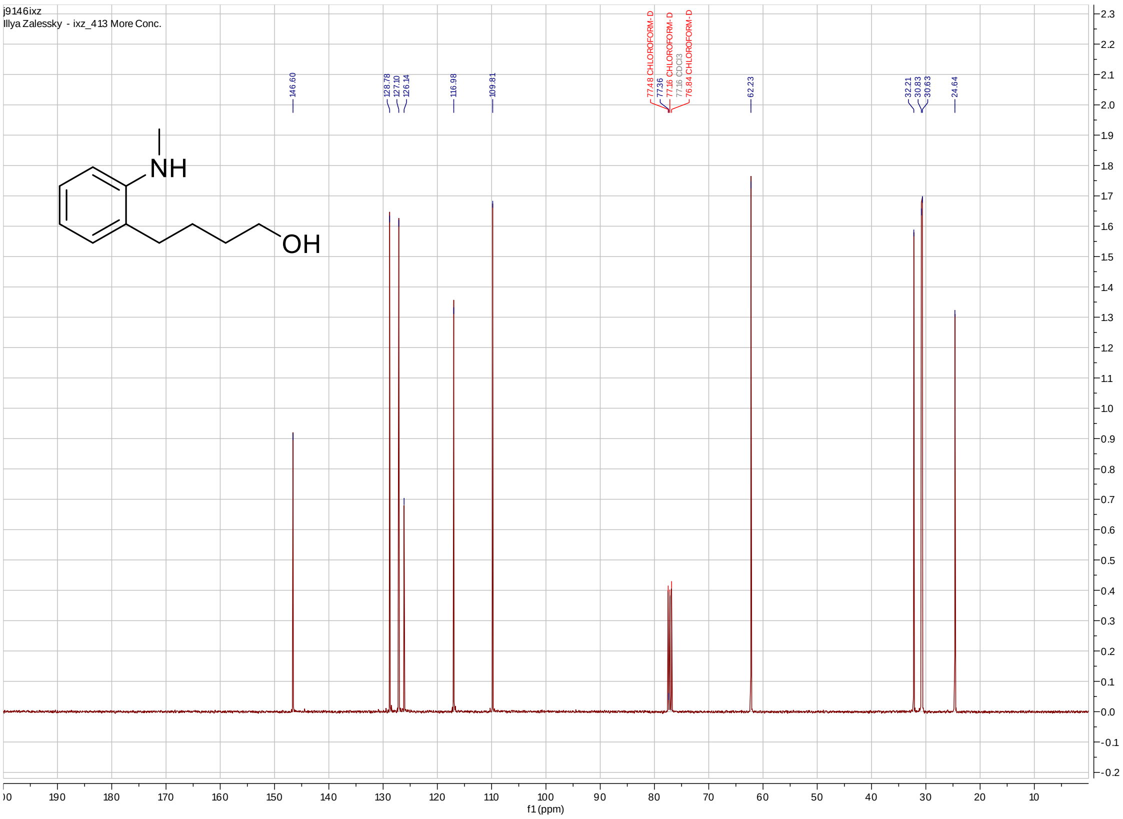

1-Methyl-5,6,7,8-tetrahydro-1H-benzo[e][1,4]oxazecine-2,3-dione (27)

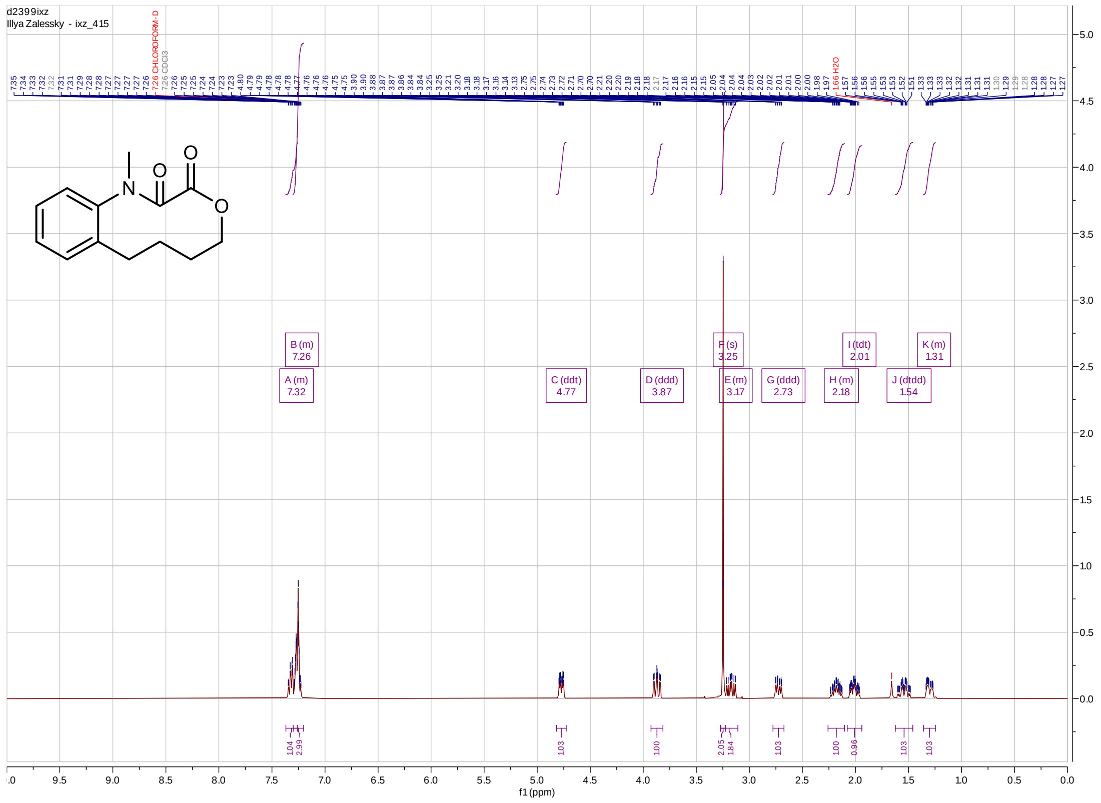

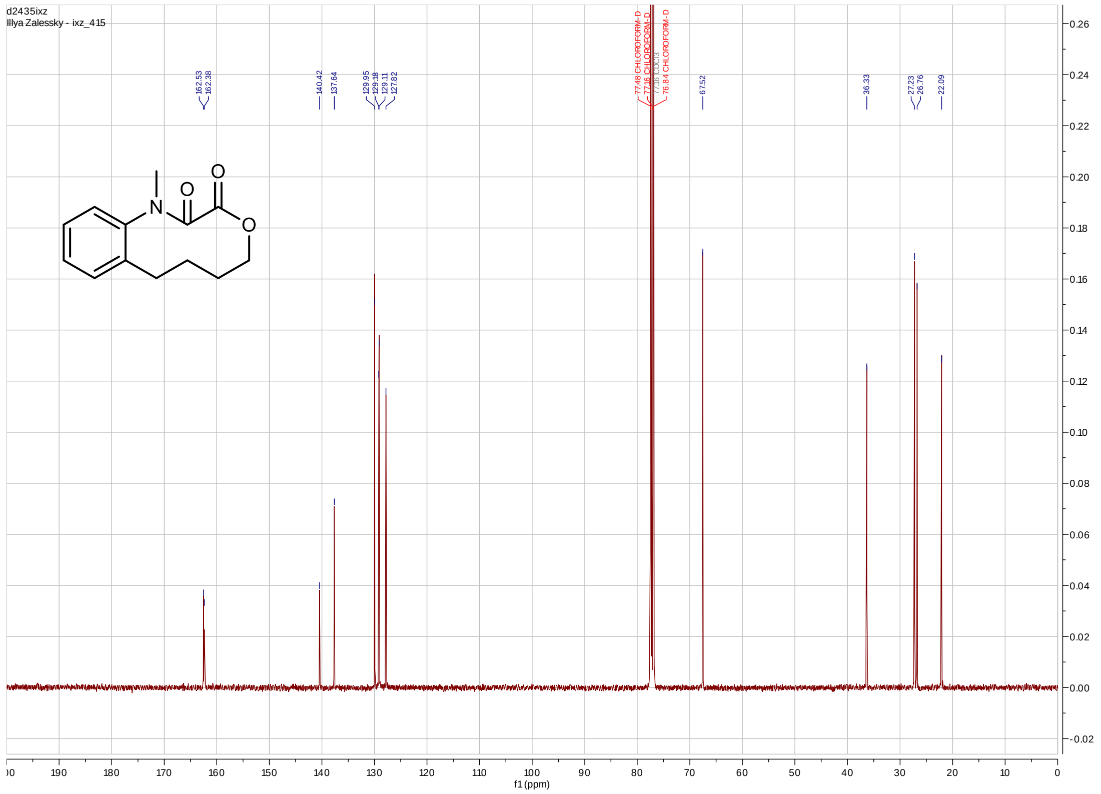

3,3'-(Benzylazanediyl)bis(propan-1-ol) (28)

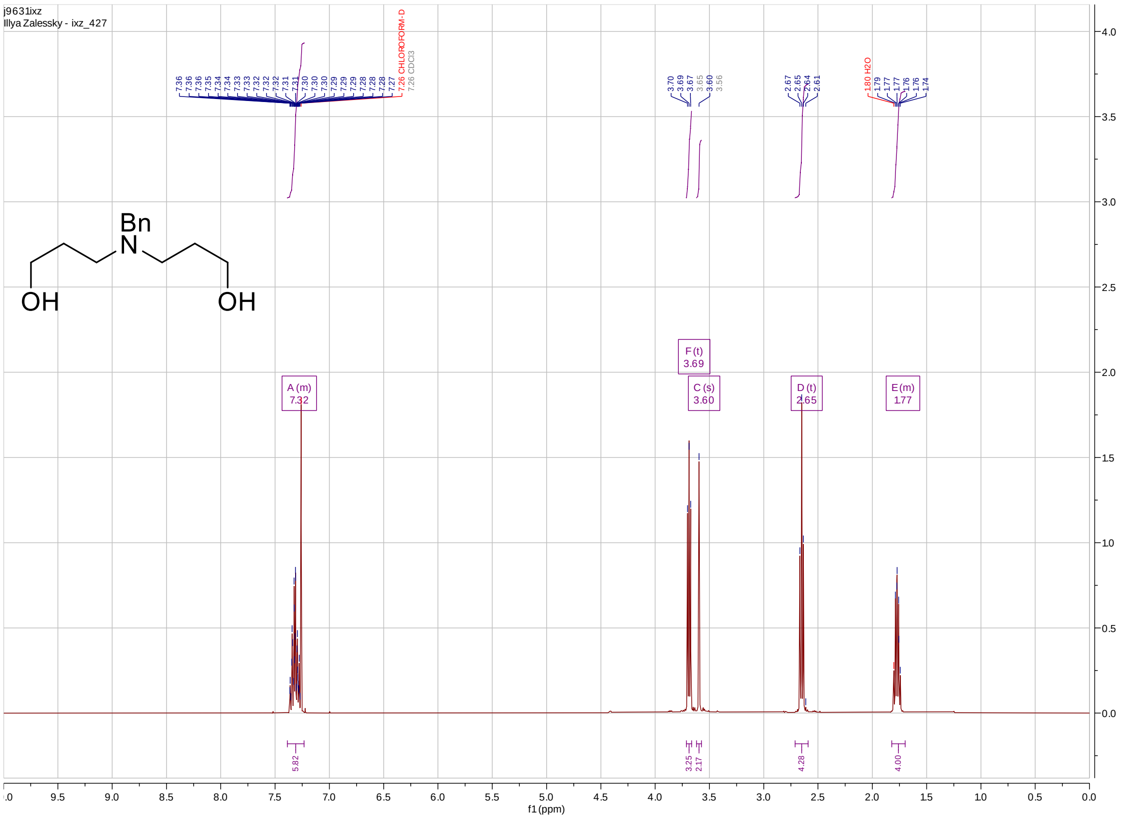

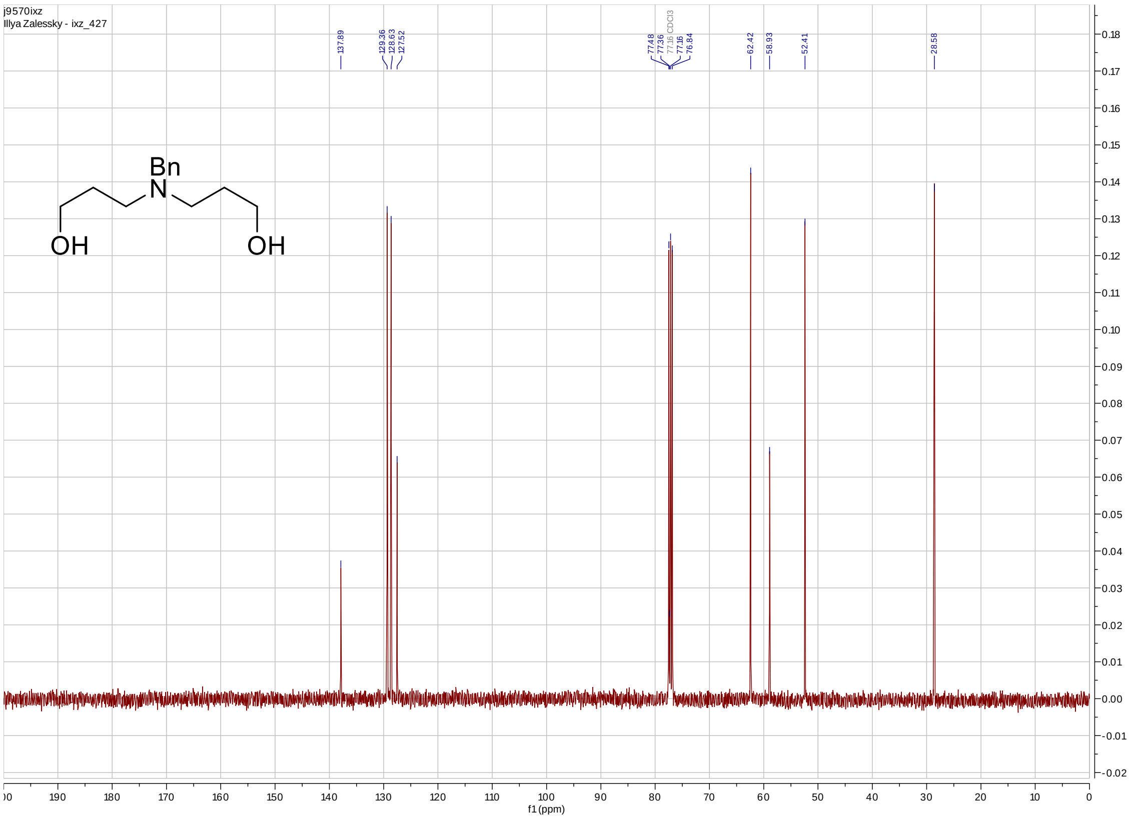

Benzyl-1,3,2,7-dioxathiazecane 2-oxide (29)

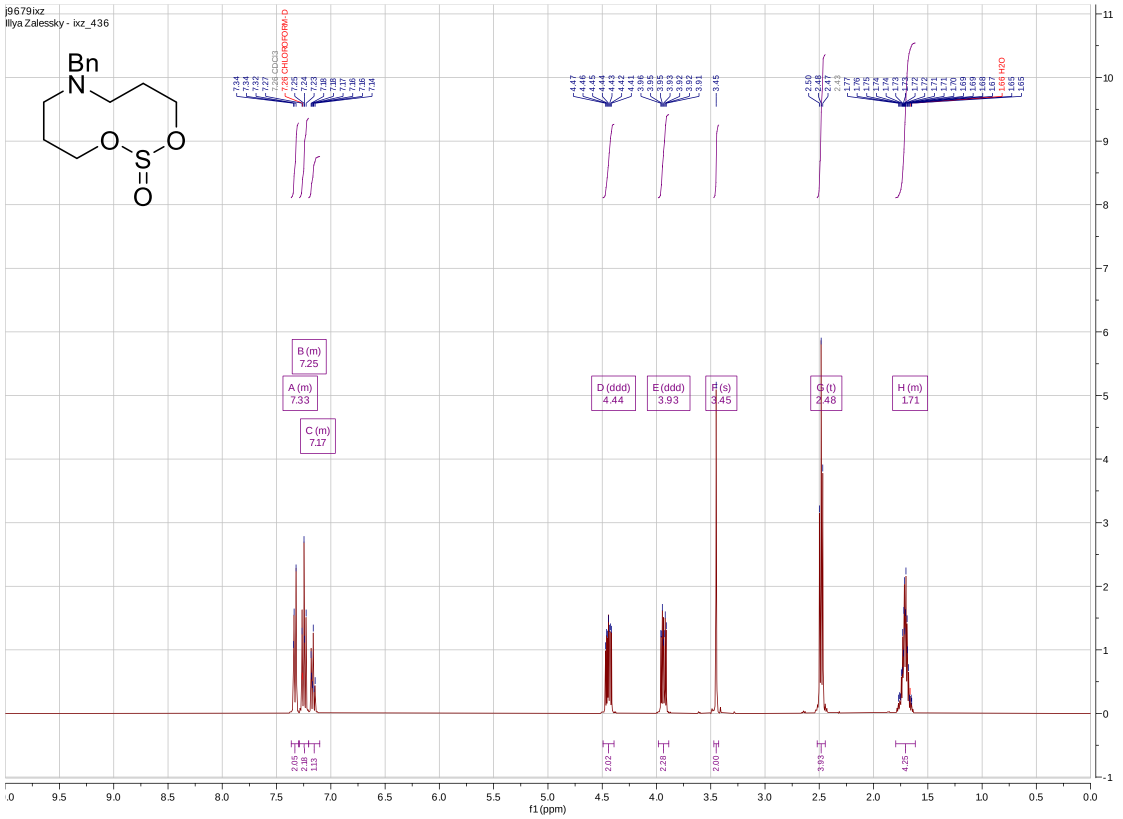

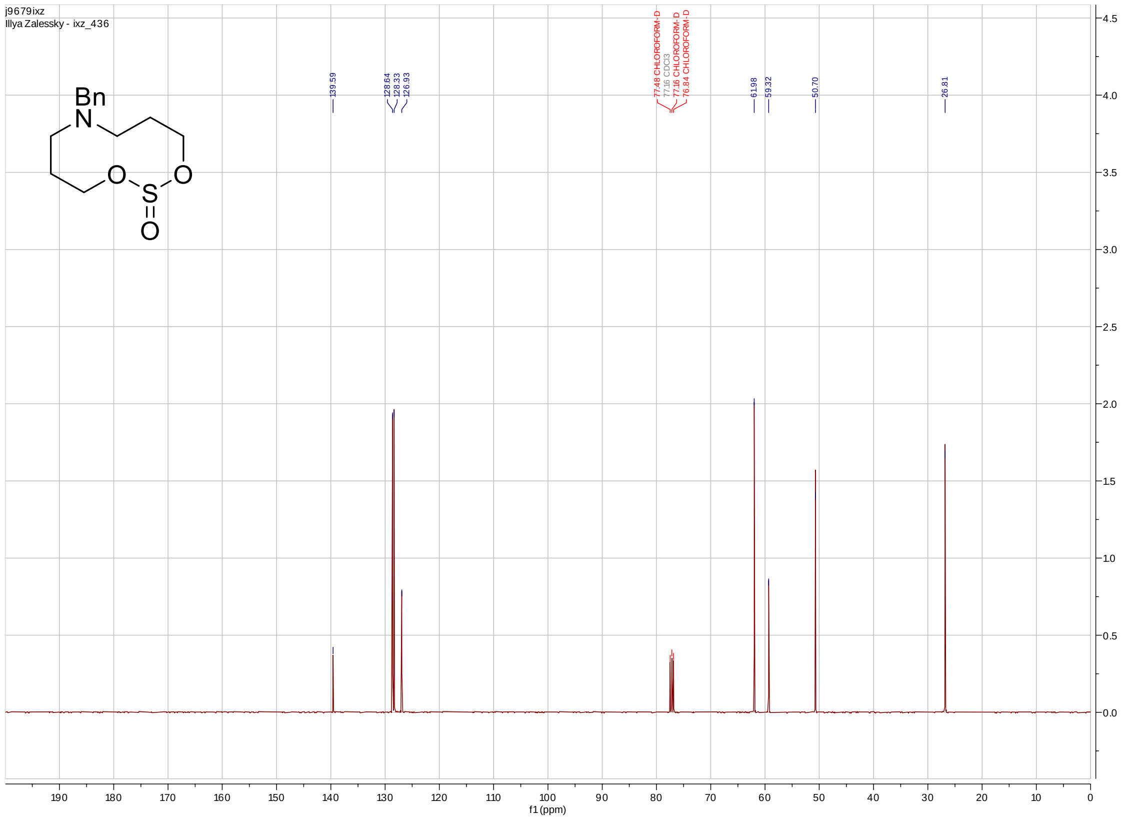

1,3,2-Dioxathiecane 2-oxide (31)

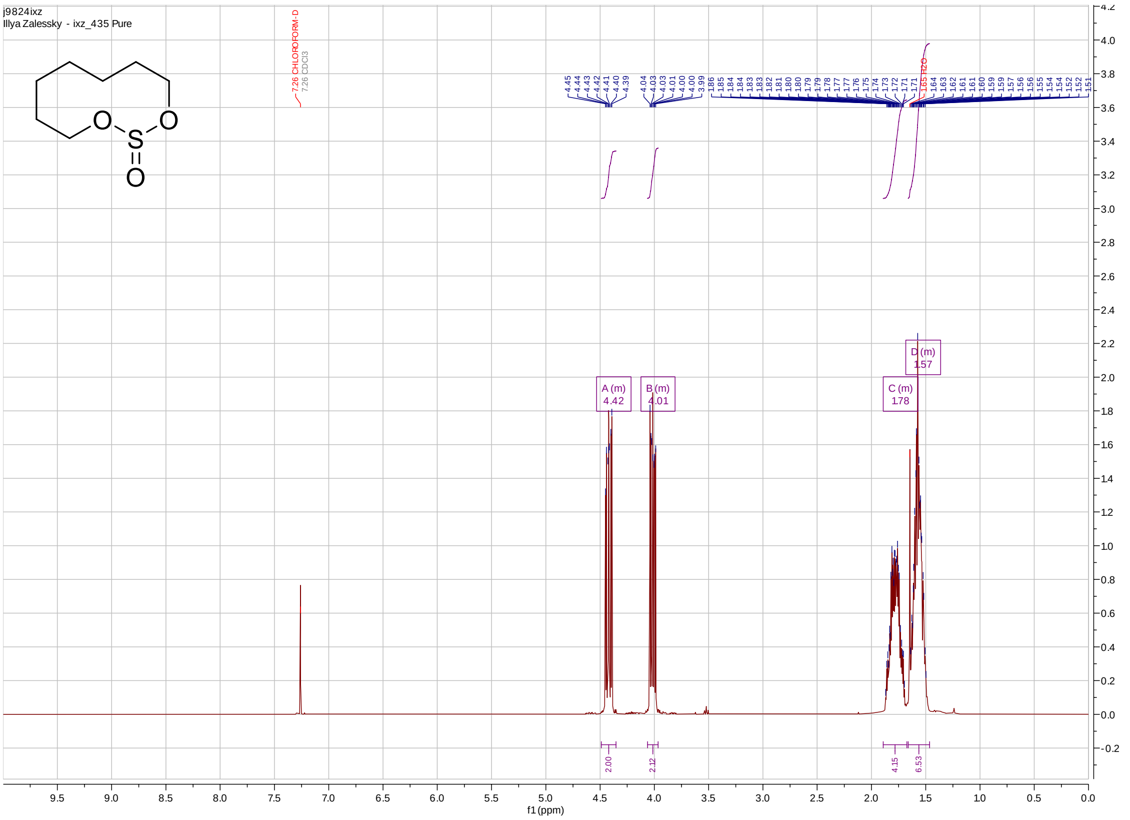

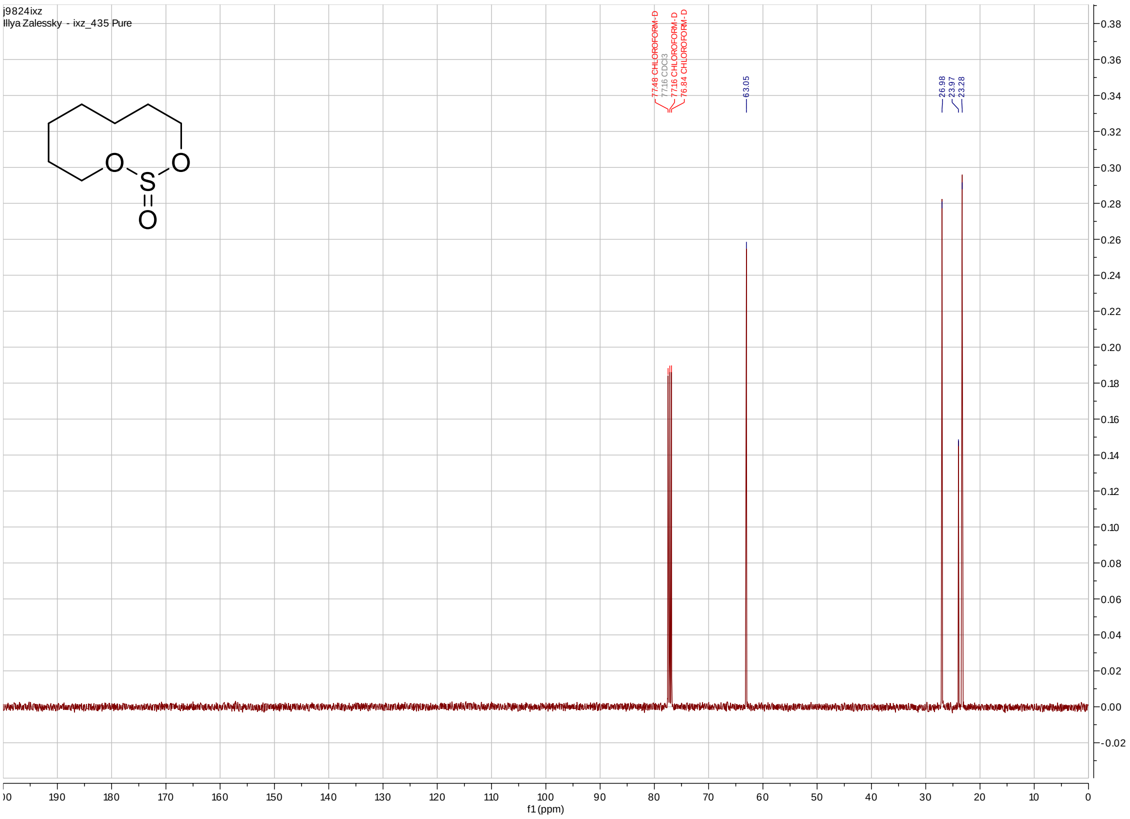

7-Benzyl-2-phenyl-1,7,2,3-oxazadiphosphecane 2-oxide (32)

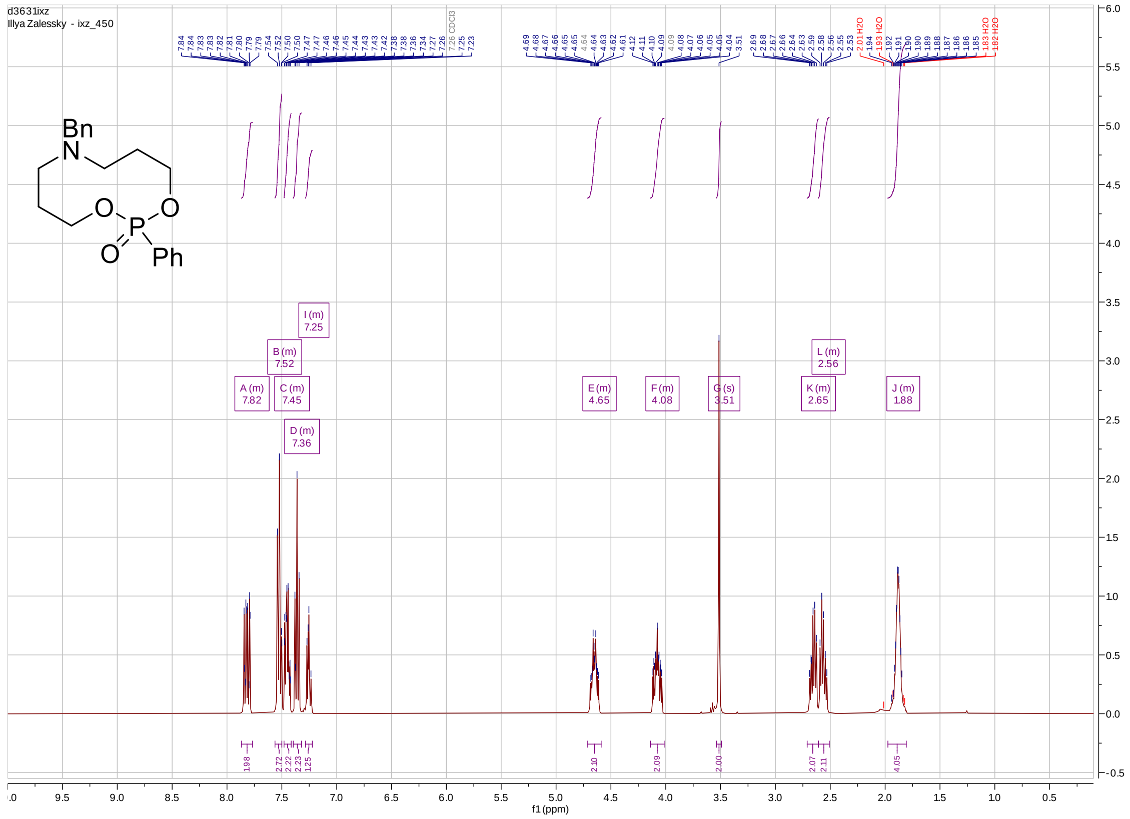

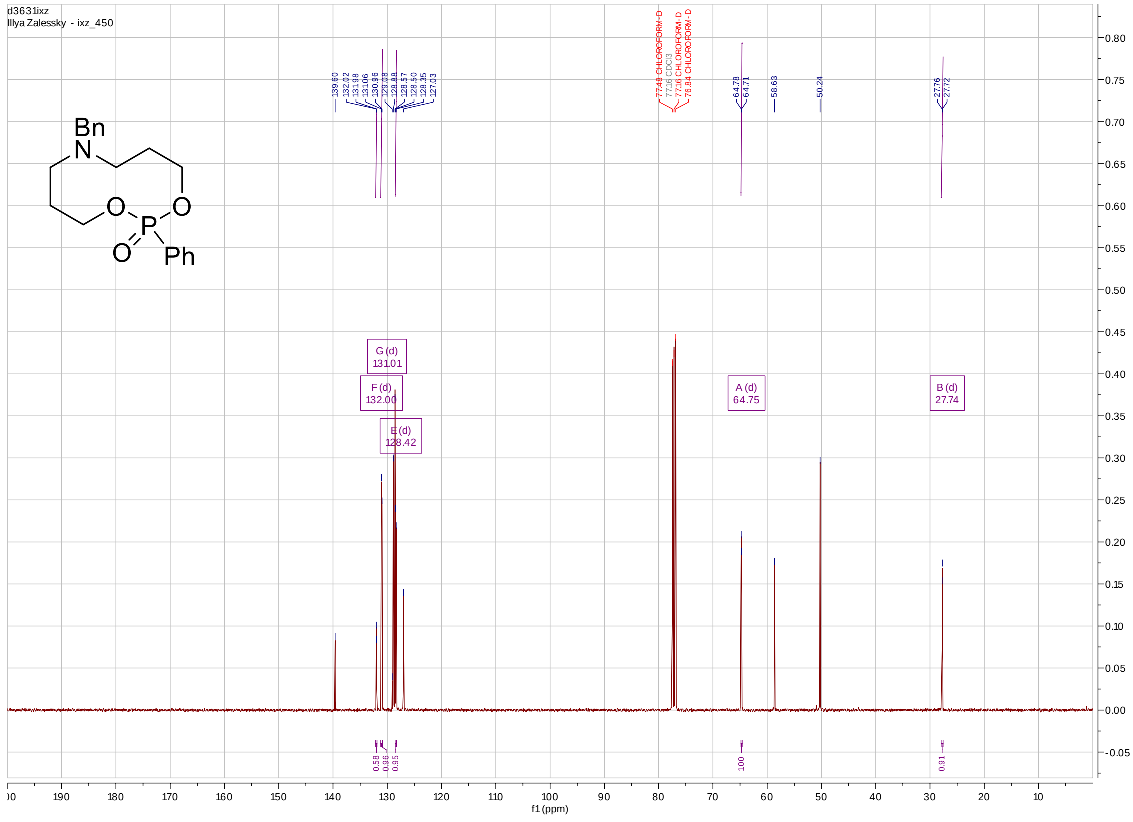

7-Benzyl-2-phenyl-1,7,2,3-oxazadiphosphecane 2-oxide (32) continued

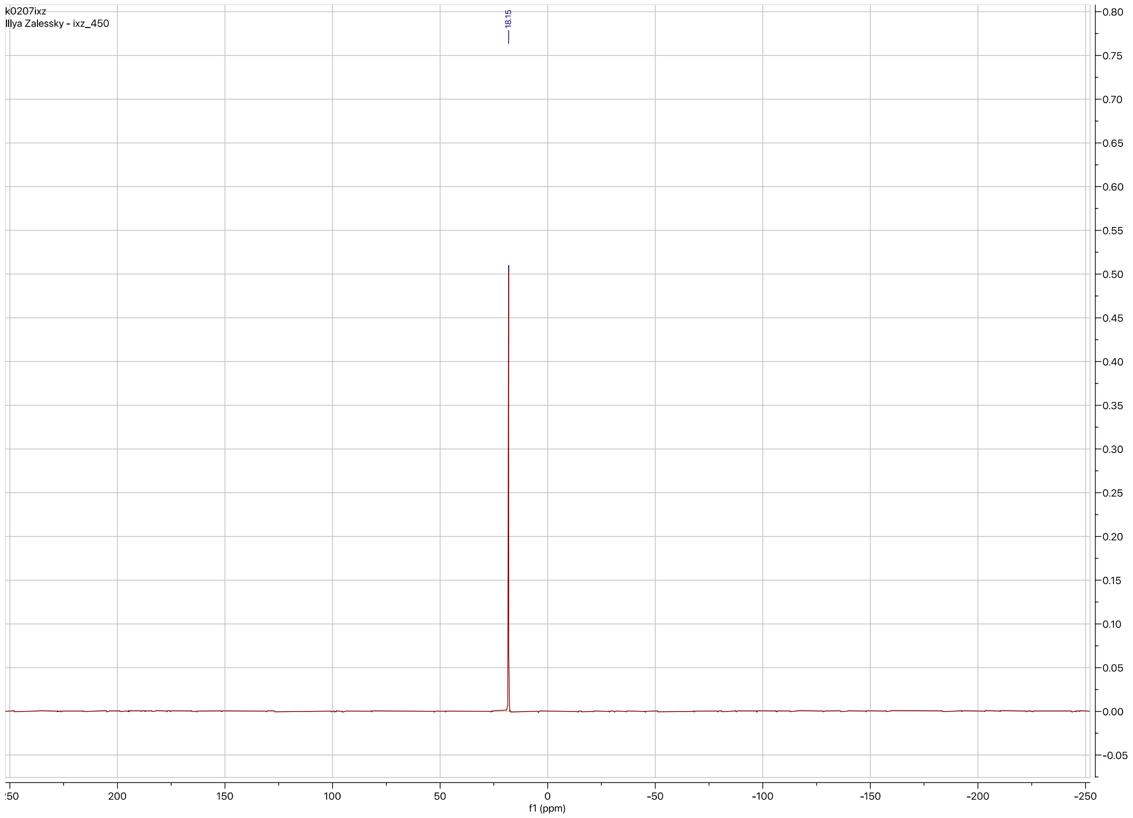

Bis(7-chloroheptyl) phenylphosphonate (S13)

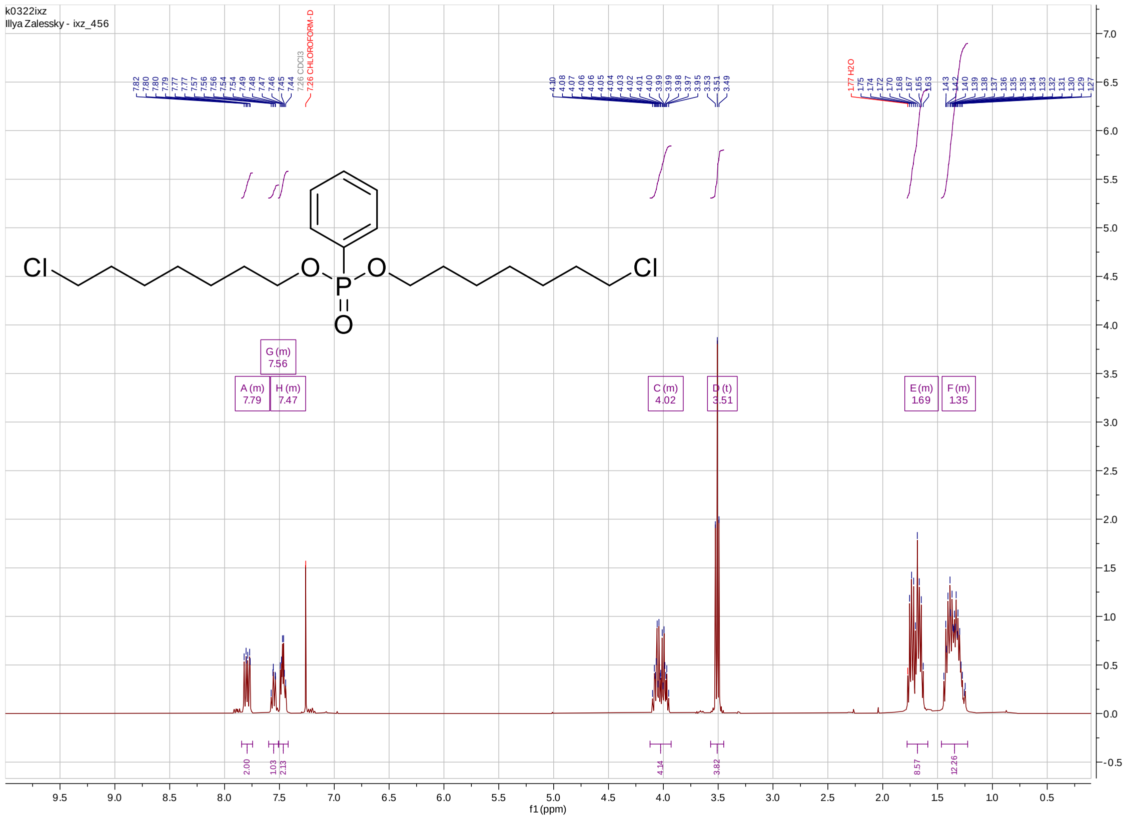

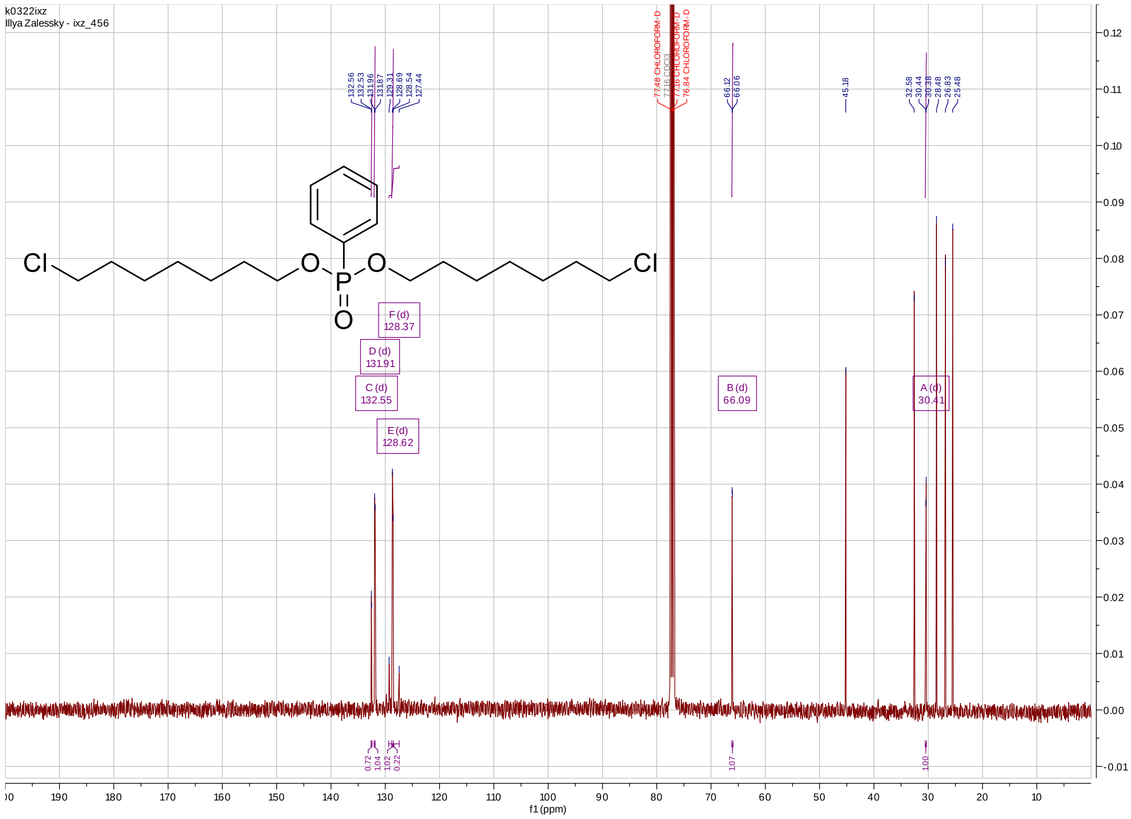

1,3,7-Trimethyl-1,3,7-triazecan-2-one (35)

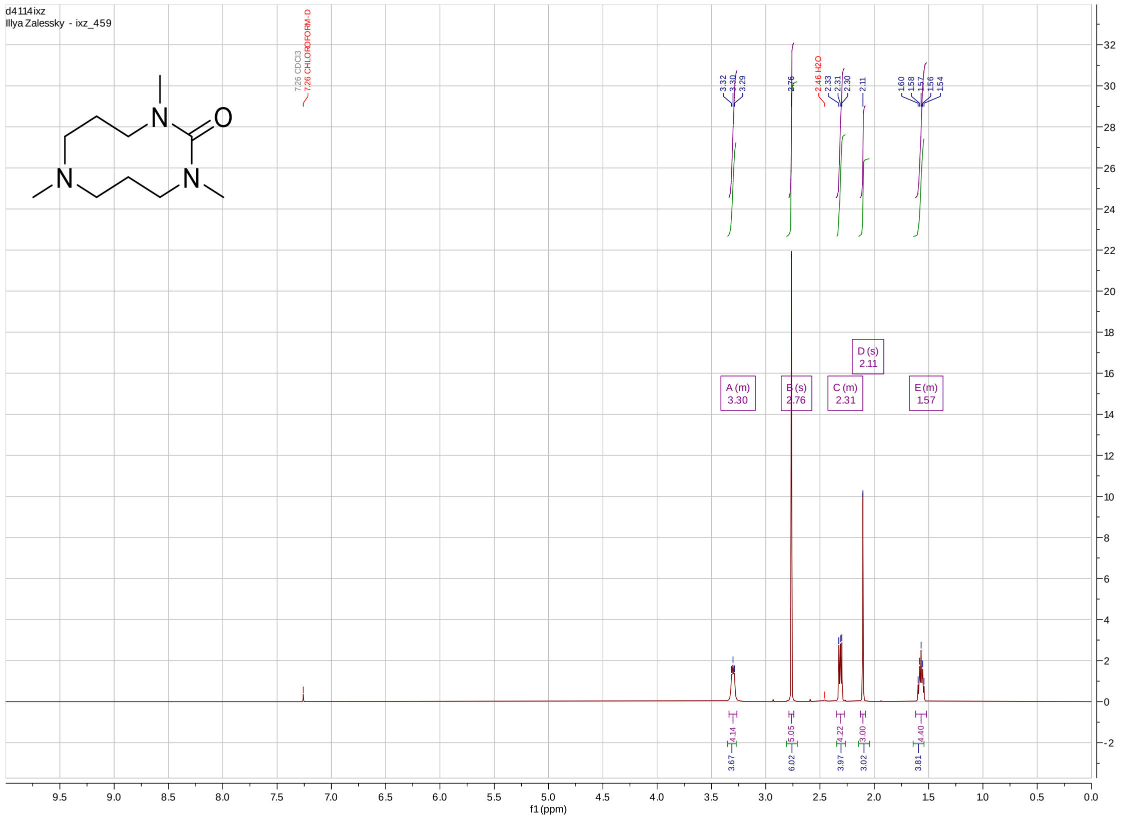

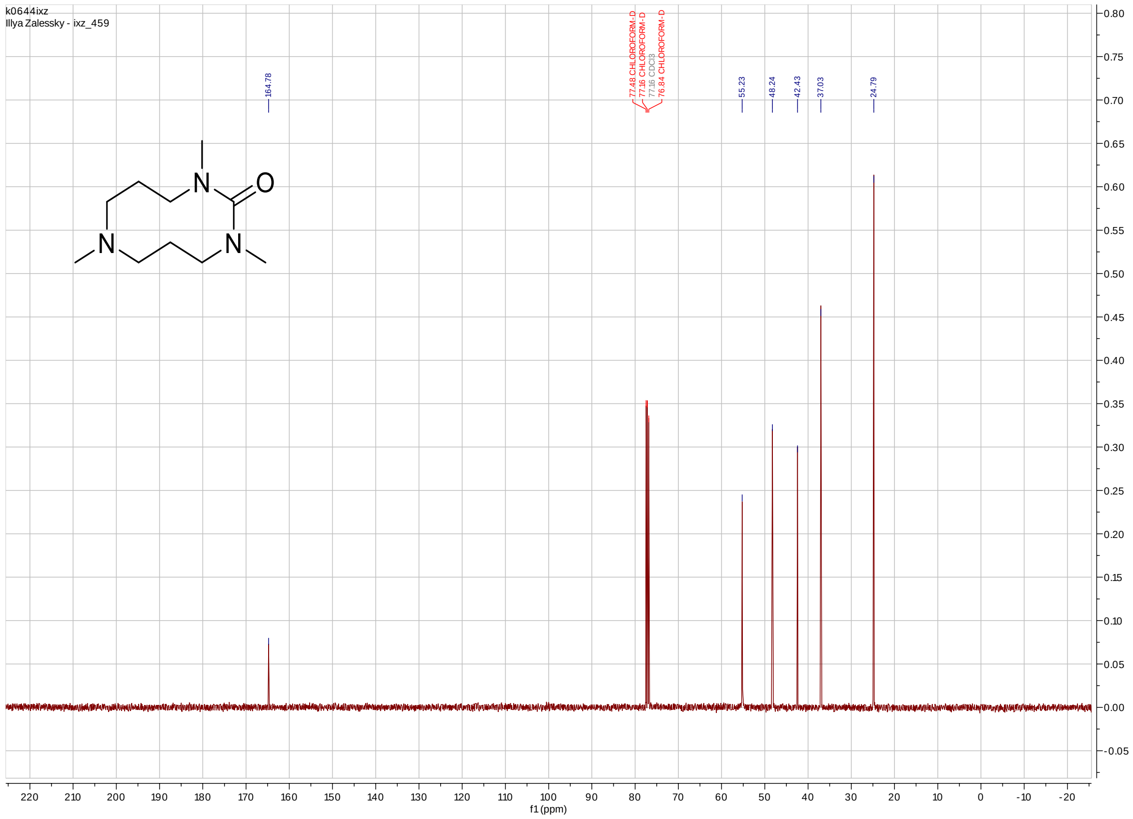

Di-*tert*-butyl heptane-1,7-diyldicarbamate (S14)

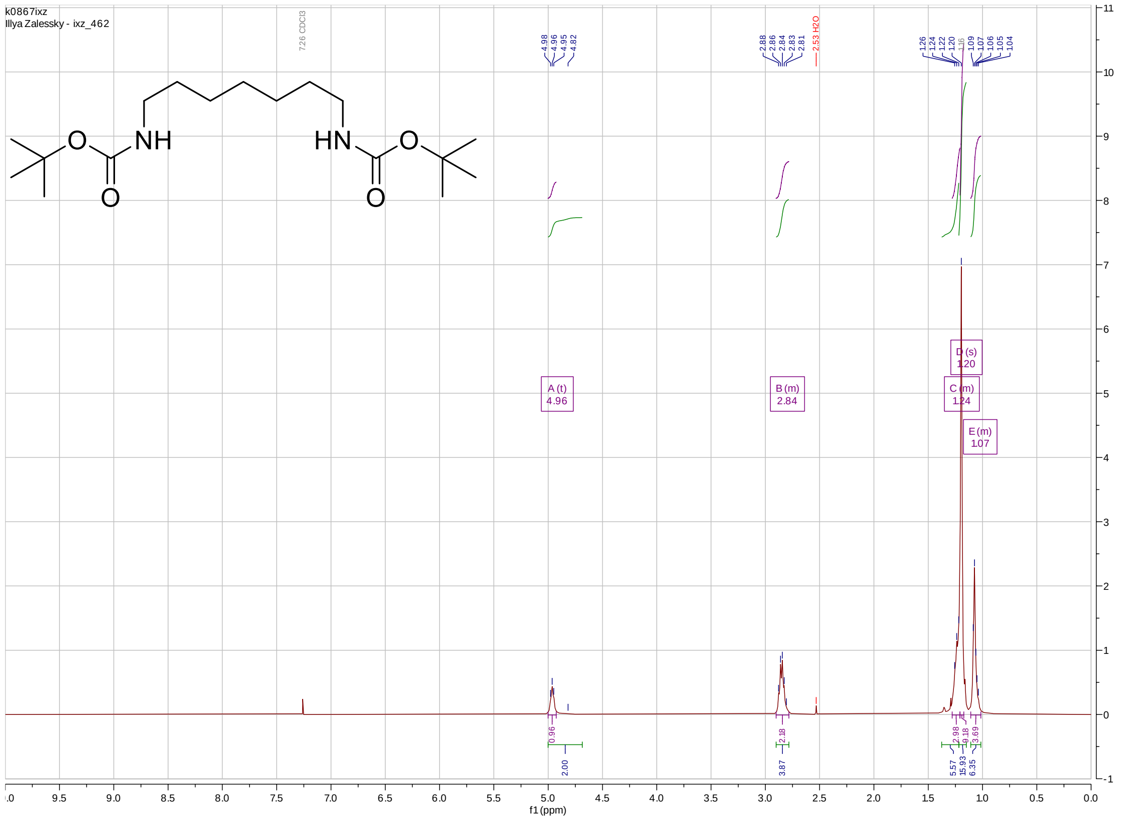

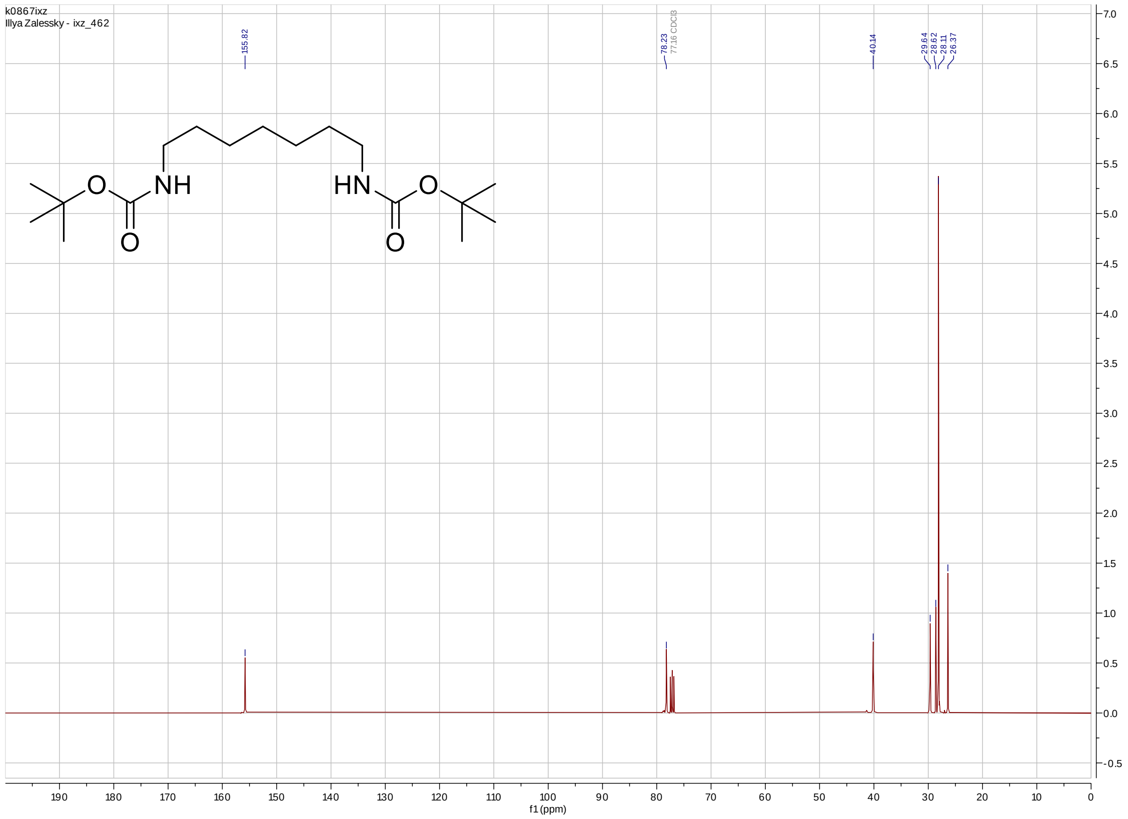

N1,N7-dimethylheptane-1,7-diamine (36)

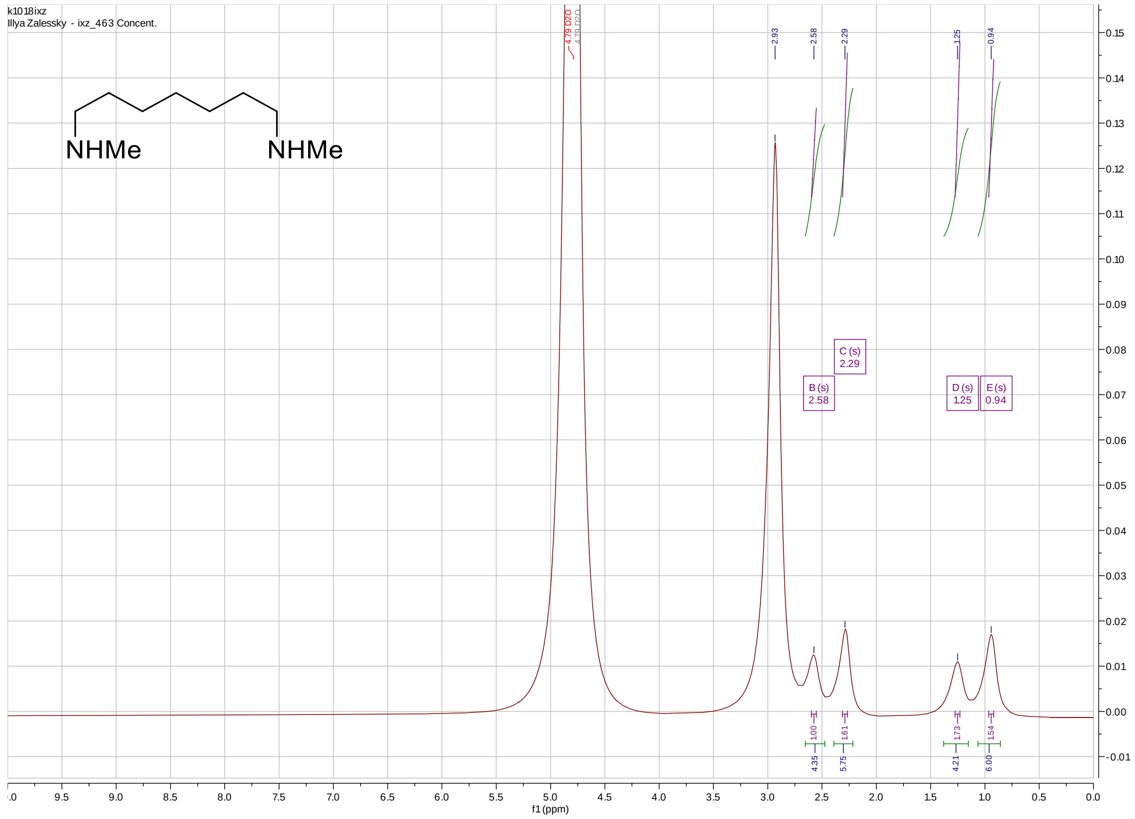

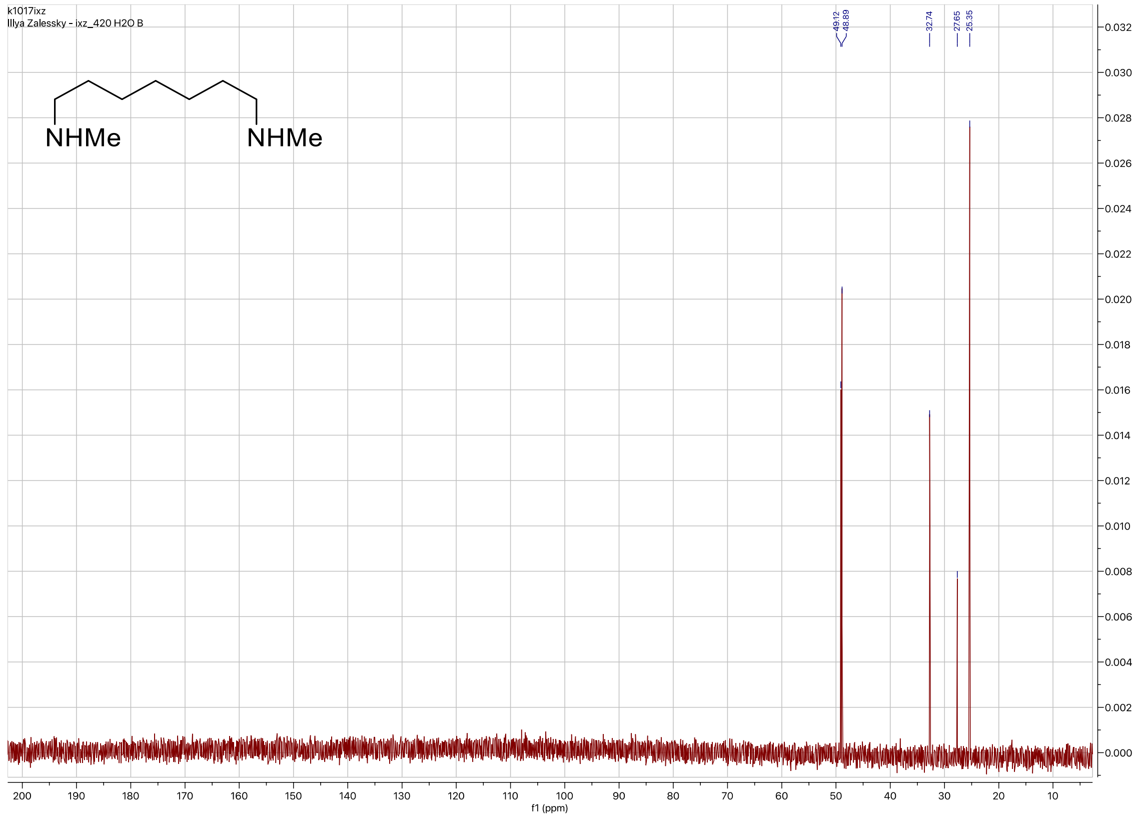

Methyl 2-((benzyl(4-hydroxybutyl) amino) methyl) benzoate (S15)

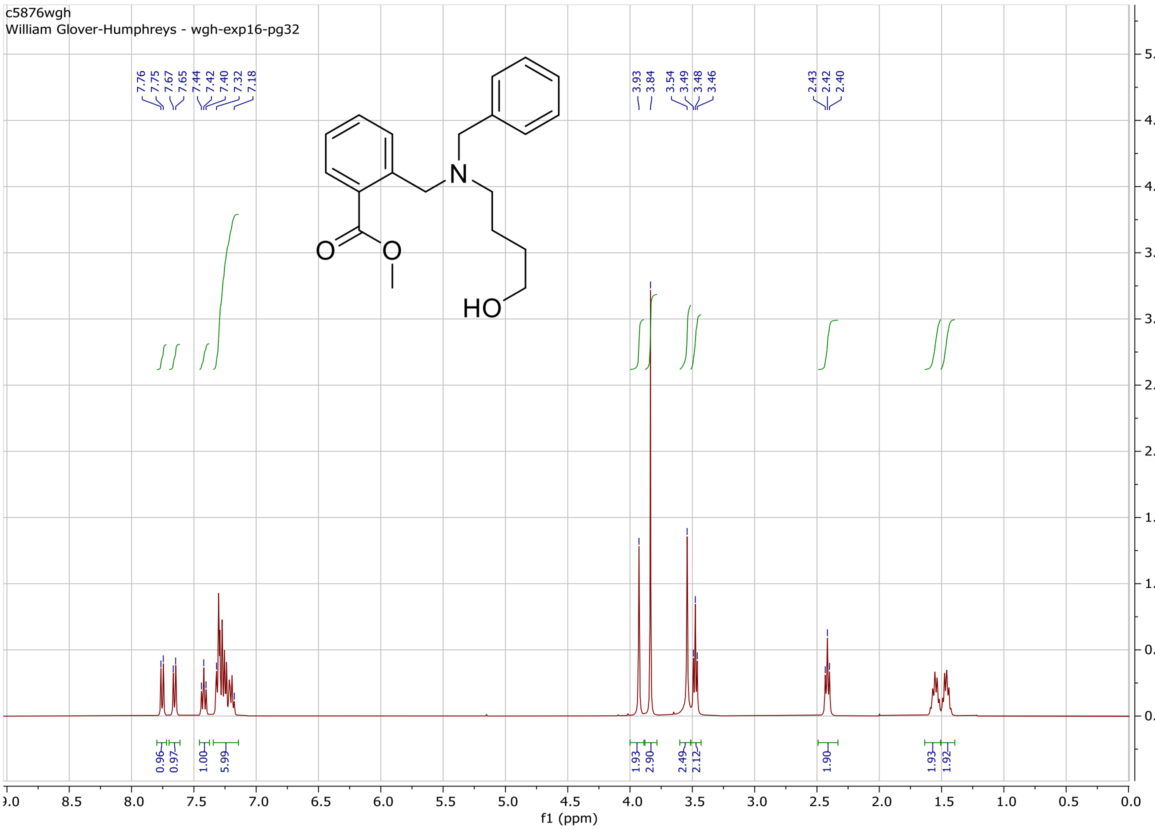

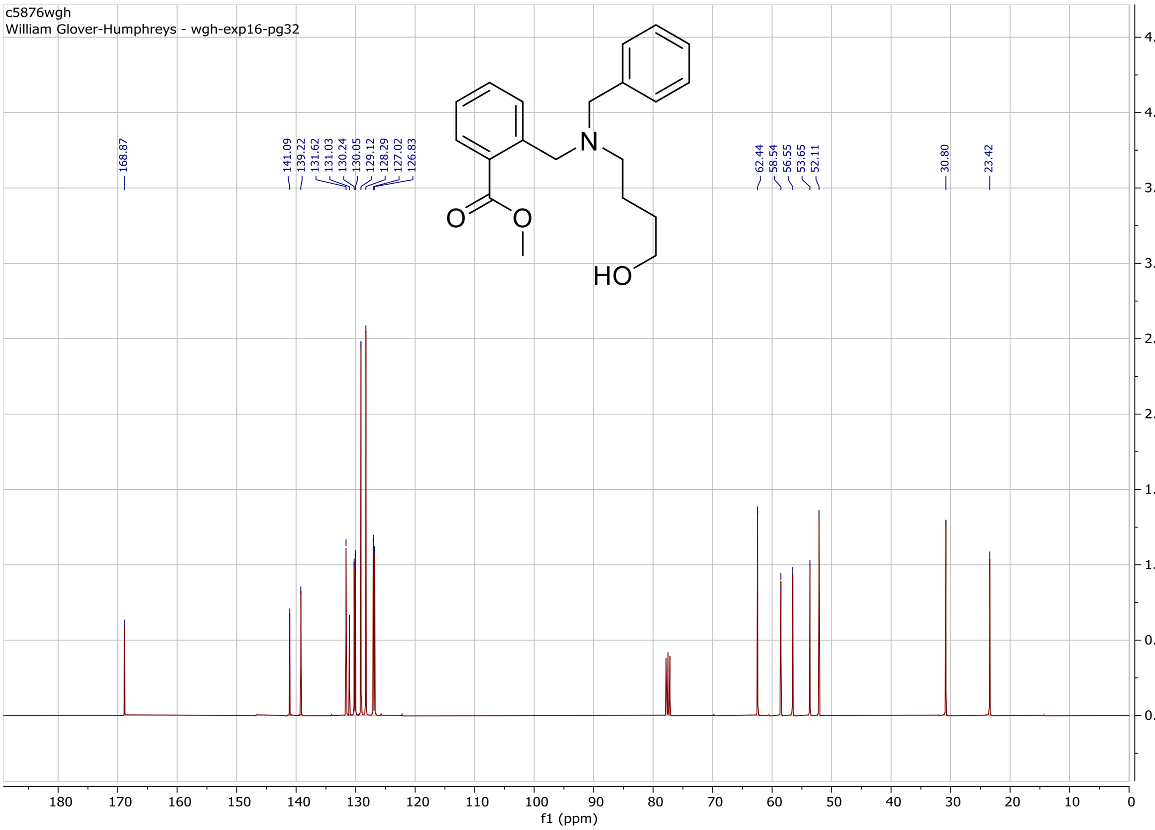

7-Benzyl-3,4,5,6,7,6-hexahydro-1H-benzo[c] [1,6] oxazecin-1-one (38)

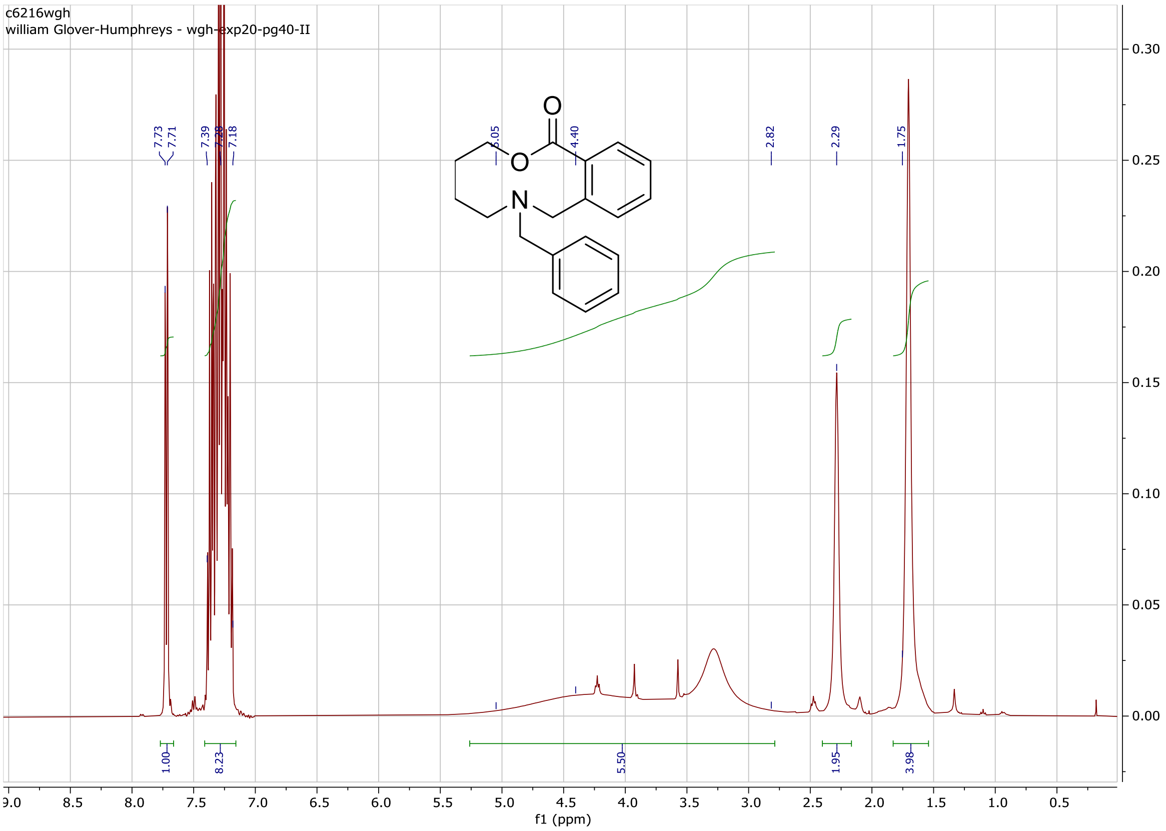

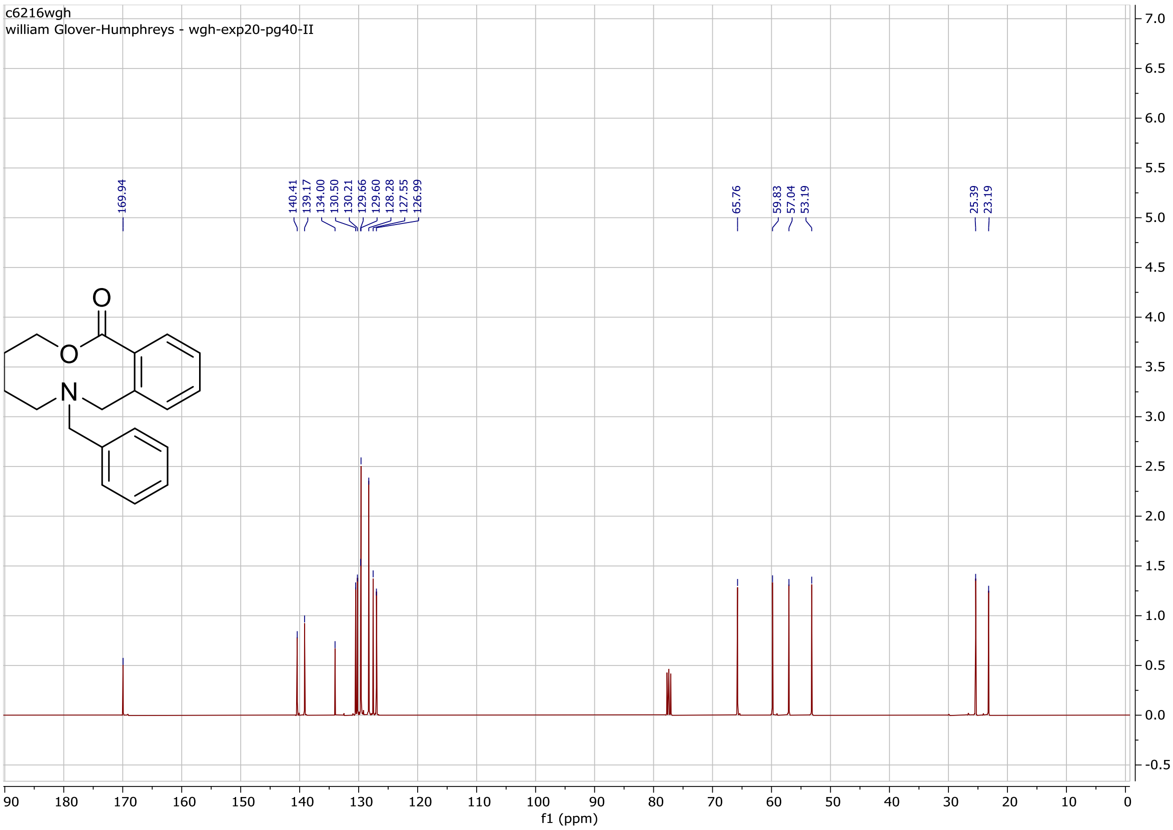

Methyl 2-((benzyl(4-hydroxybutyl) amino) methyl) nicotinate (S16)

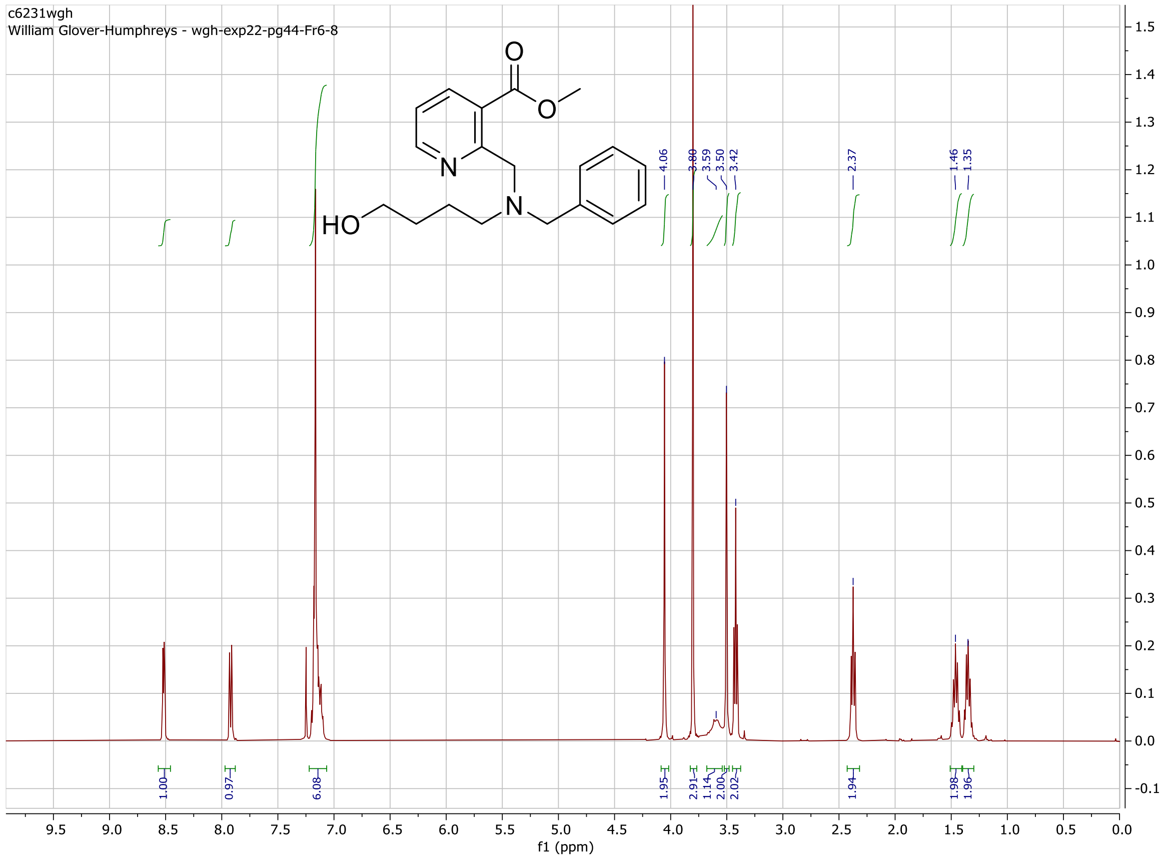

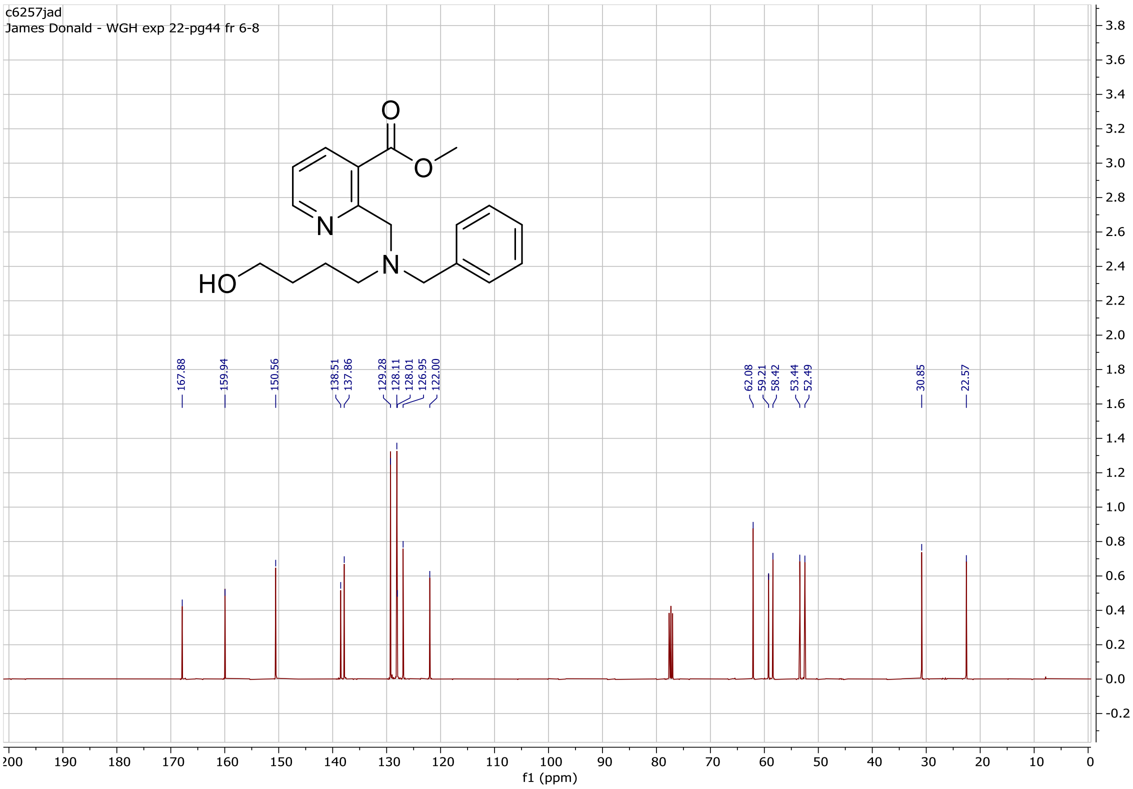

11-Benzyl-7,8,9,10,11,12-hexahydro-5H-pyrido[4,3-c] [1,6] oxazecin-5-one (39)

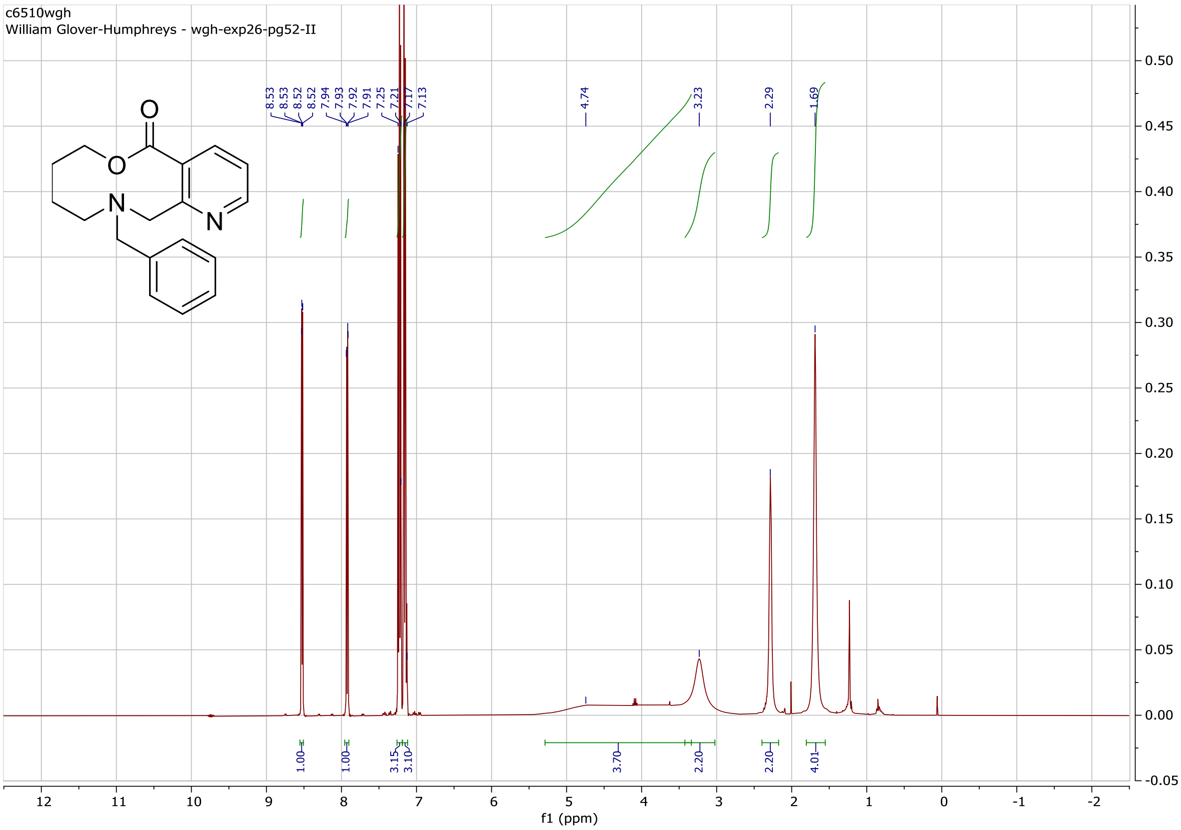

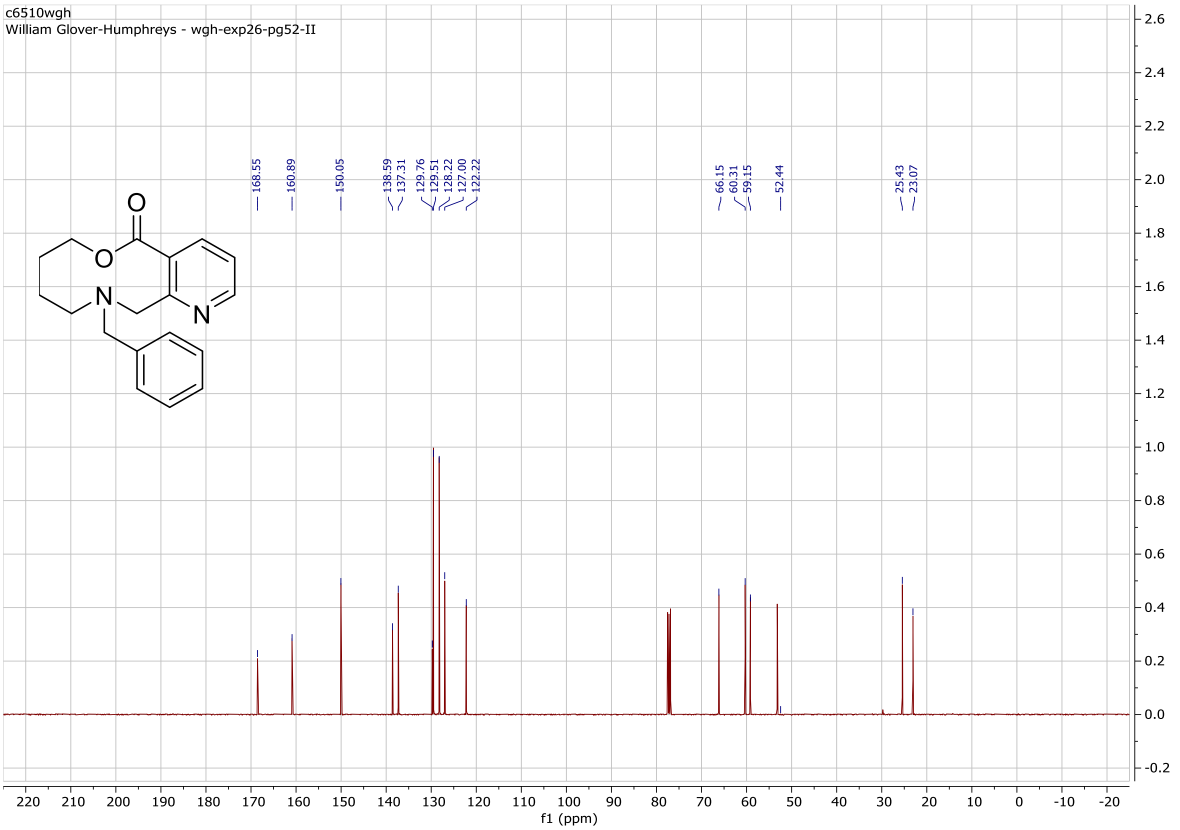

Methyl 3-((benzyl(4-hydroxybutyl) amino) methyl) pyrazine-2-carboxylate (S18)

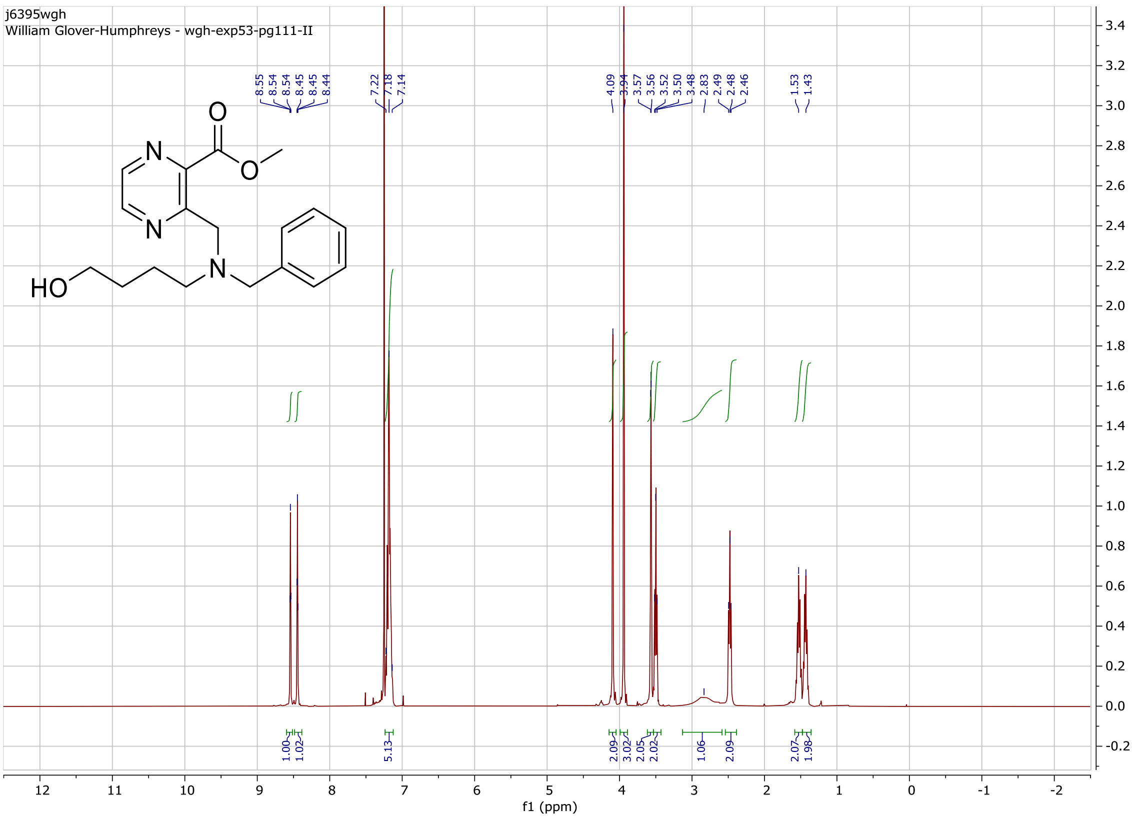

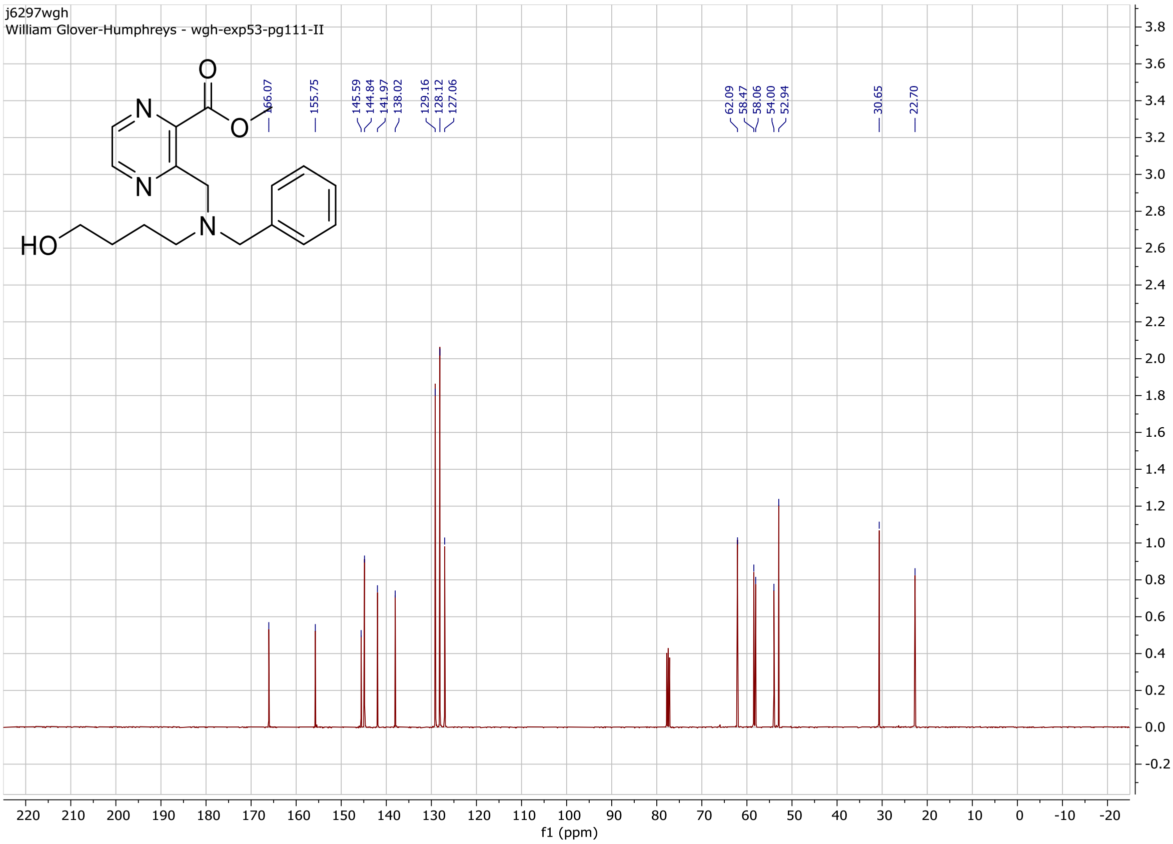

11-Benzyl-7,8,9,10,11,12-hexahydro-5H-pyrazino[2,3-c] [1,6] oxazecin-5-one (40)

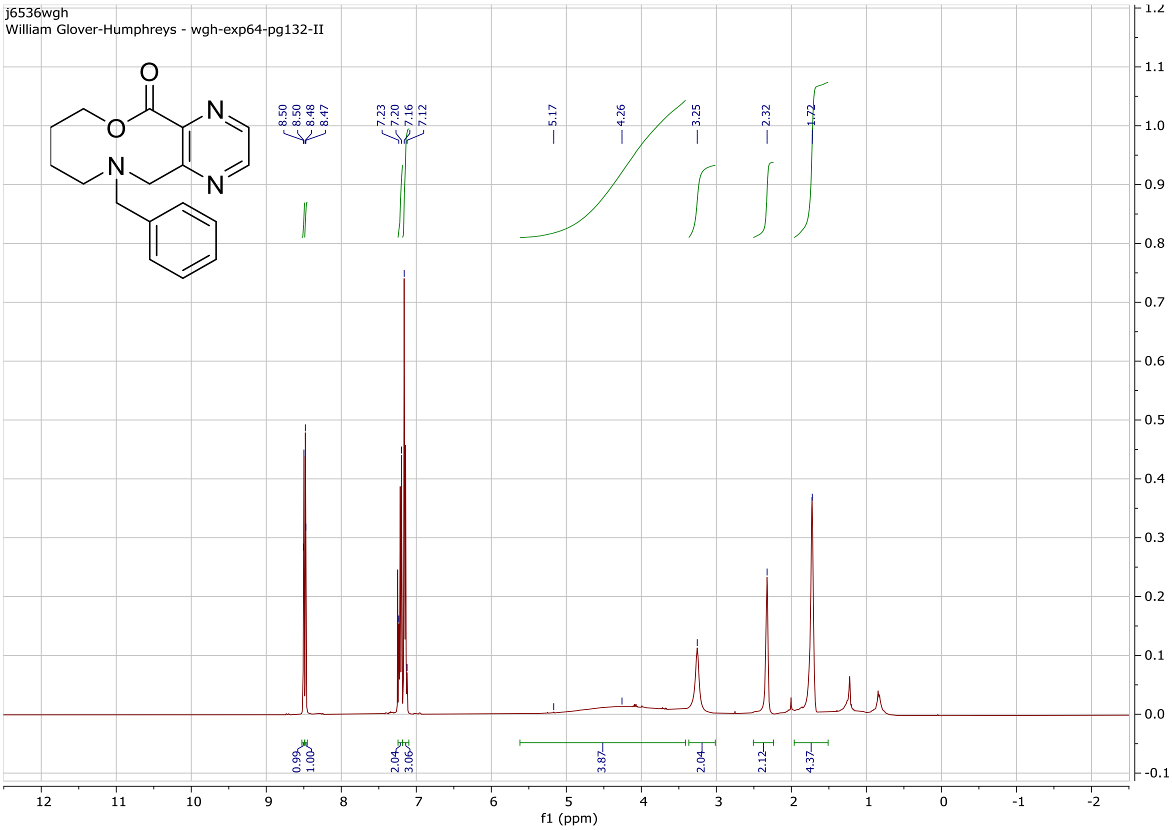

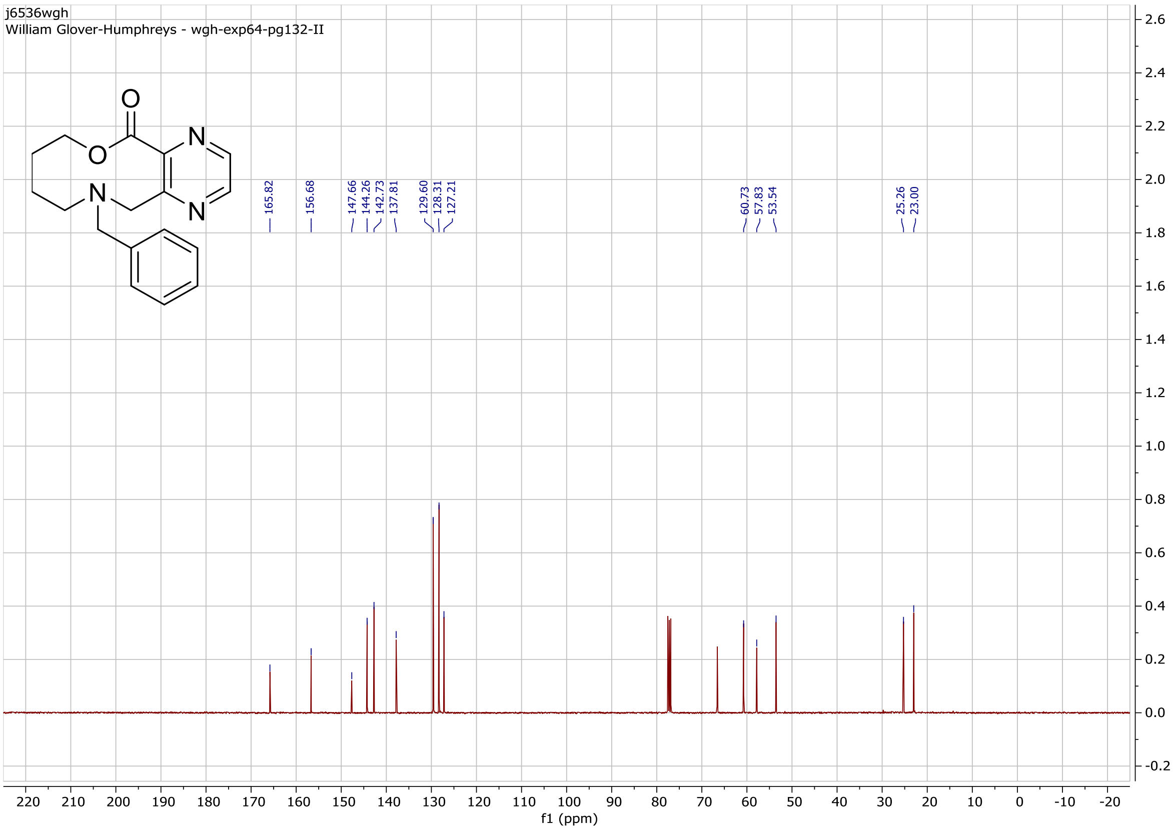

Methyl (S)-2-((2-(hydroxymethyl) pyrrolidin-1-yl) methyl) benzoate (S19)

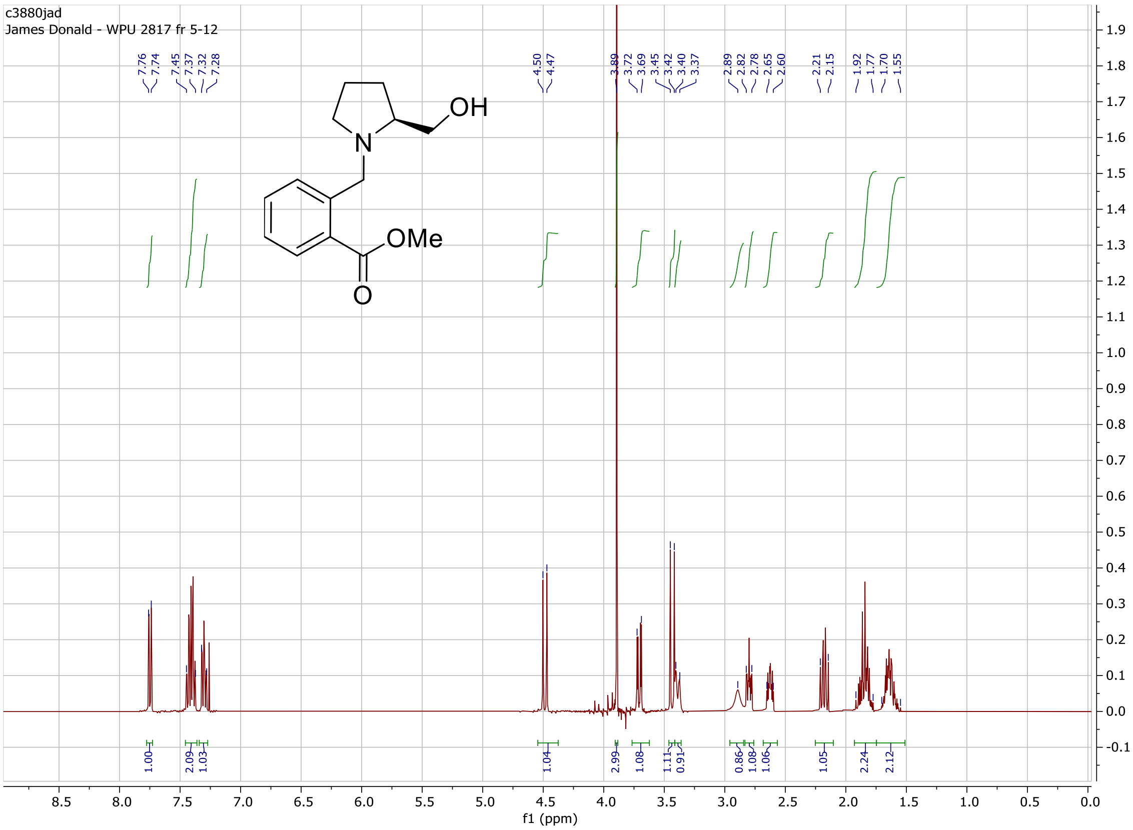

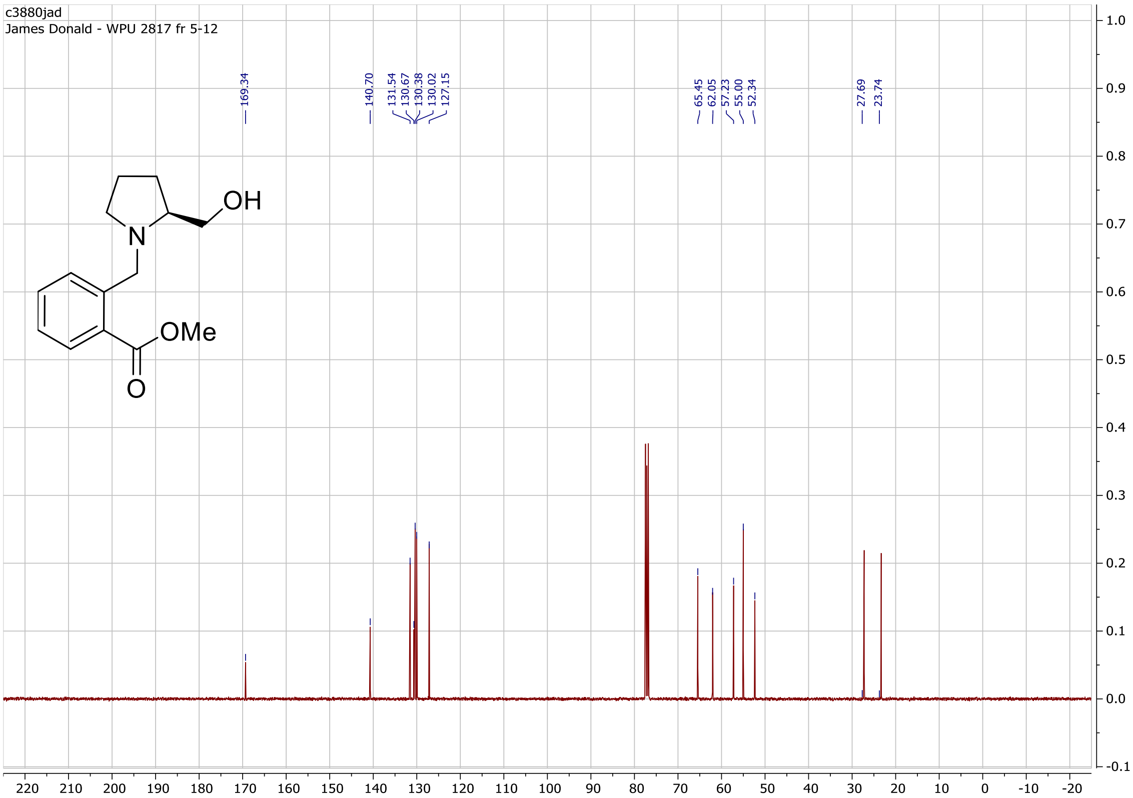

(S)-2,3,3a,4-Tetrahydro-1H-benzo[f]pyrrolo[2,1-c] [1,4] oxazocin-6(11H)-one (41)

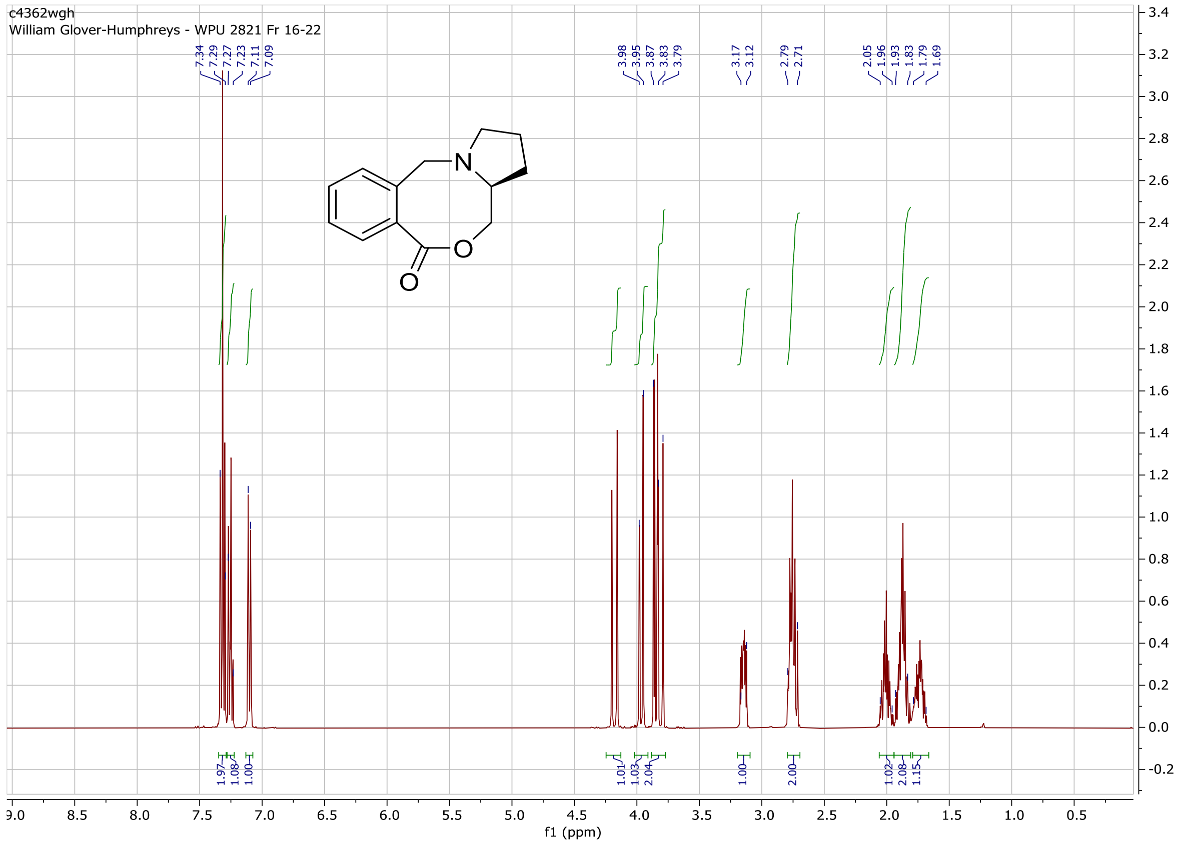

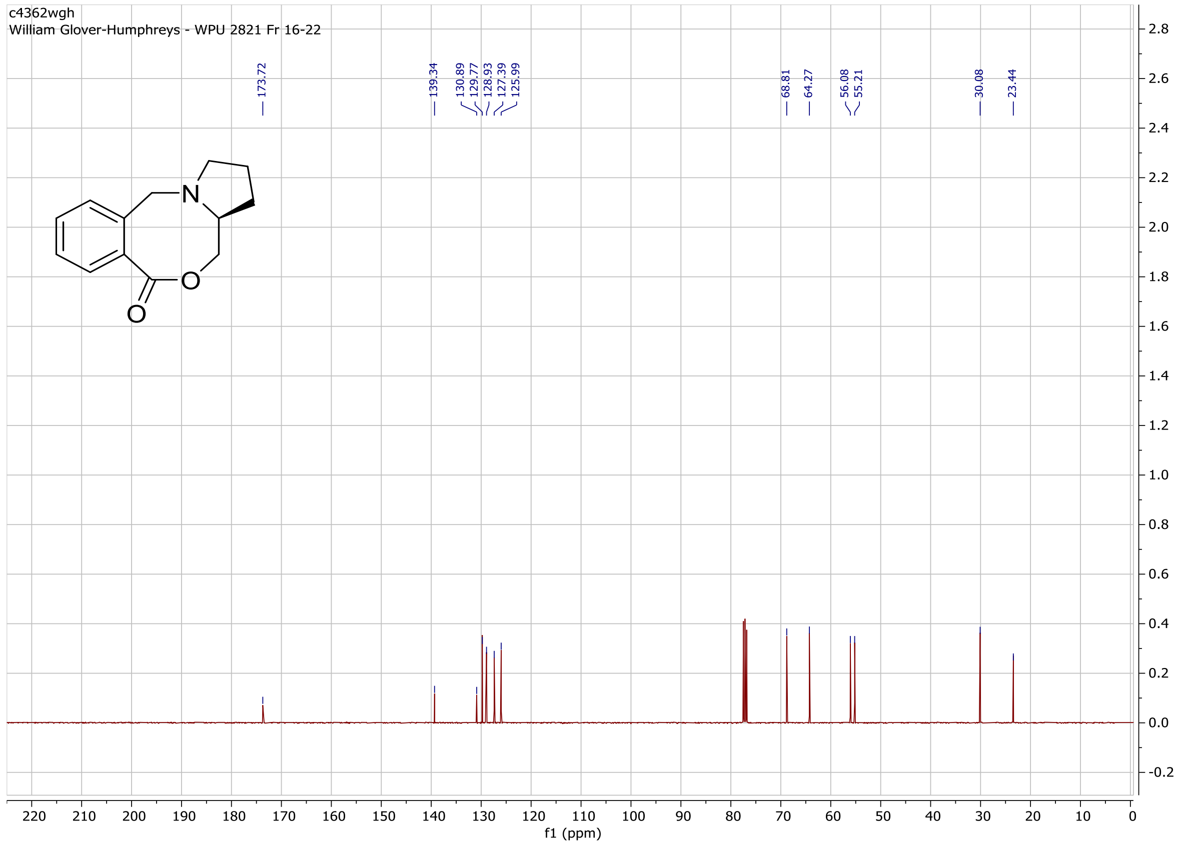

Methyl (S)-2-((2-(hydroxymethyl) pyrrolidin-1-yl) methyl) nicotinate (S20)

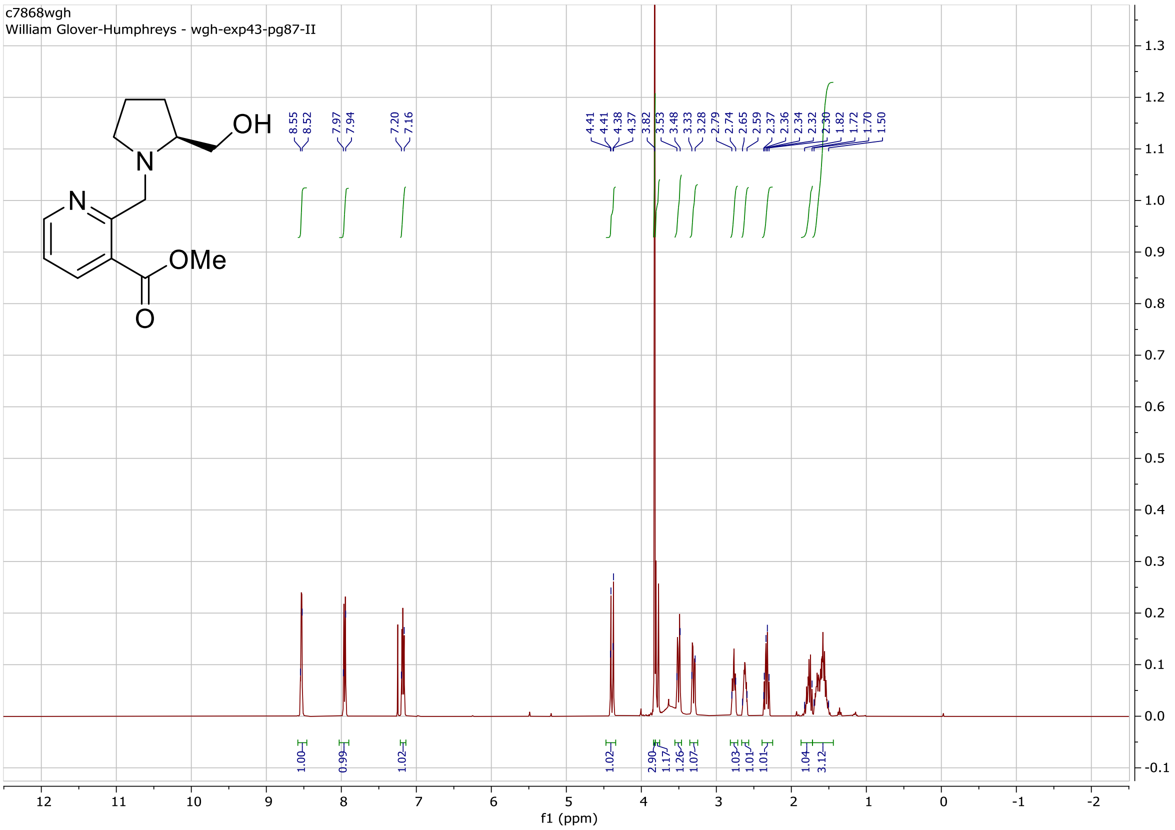

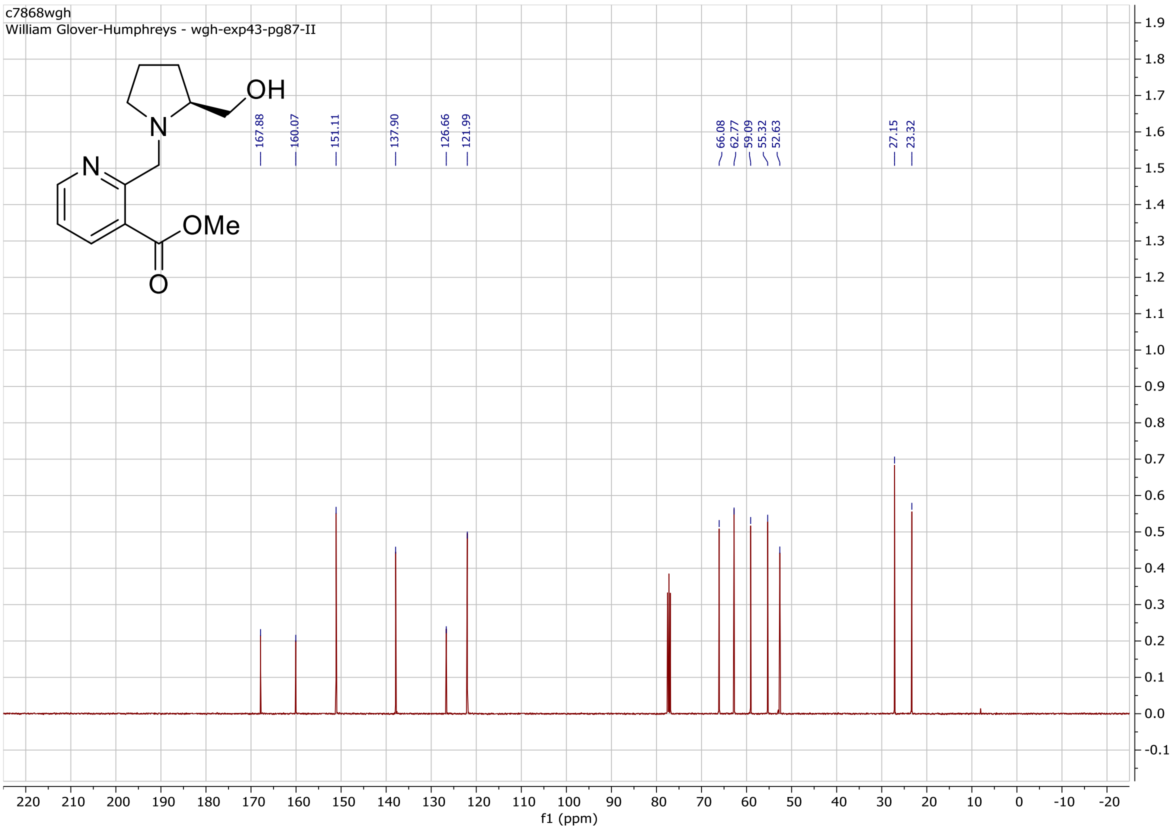

(*S*)-7a,8,9,10-Tetrahydro-7H-pyrido[2,3-f] pyrrolo [2,1-c] [1,4] oxazocin-5(12H)-one (42)

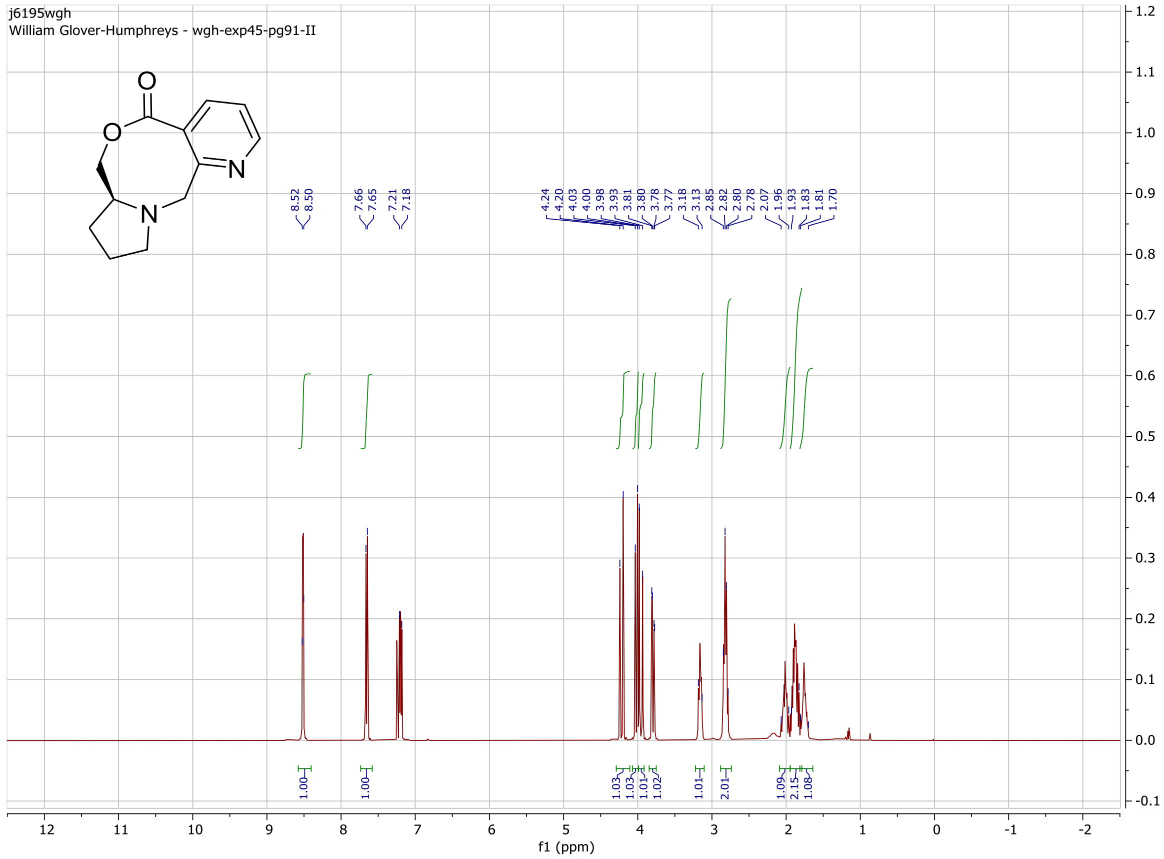

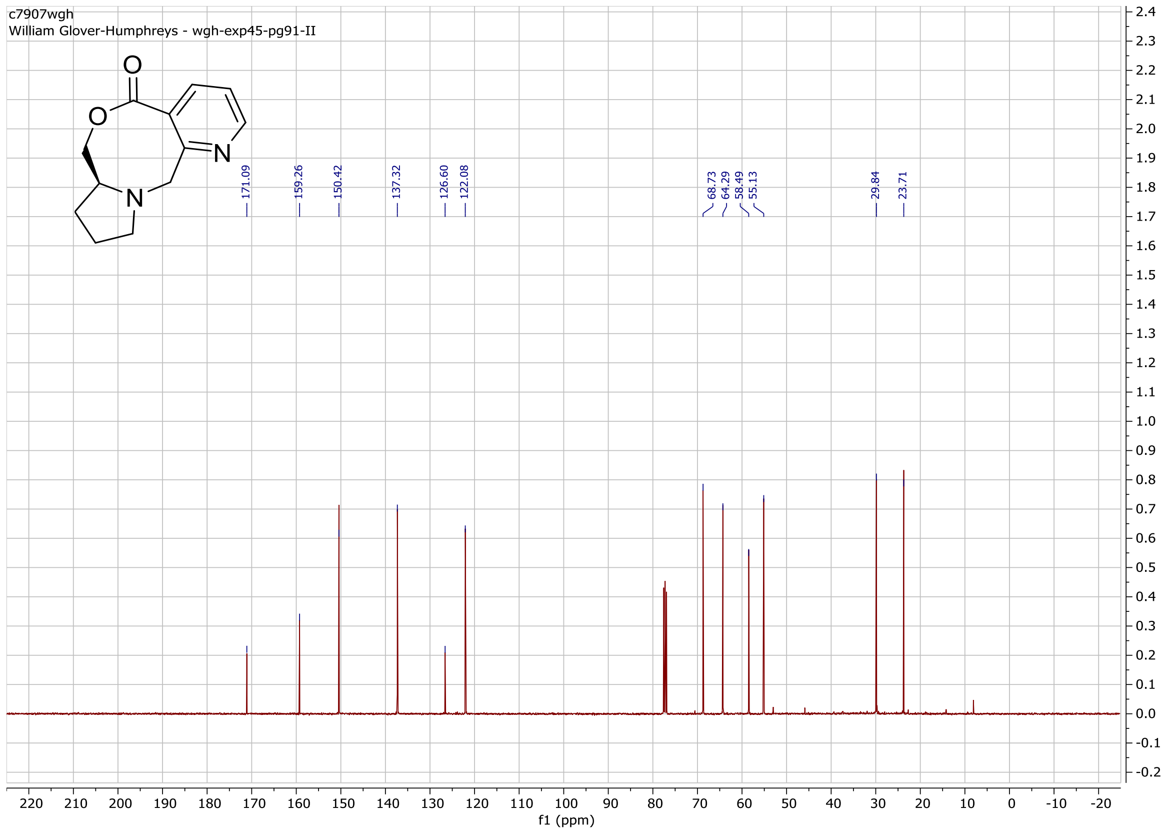

Methyl (*S*)-3-((2-(hydroxymethyl) pyrrolidin-1-yl) methyl) pyrazine-2-carboxylate (S21)

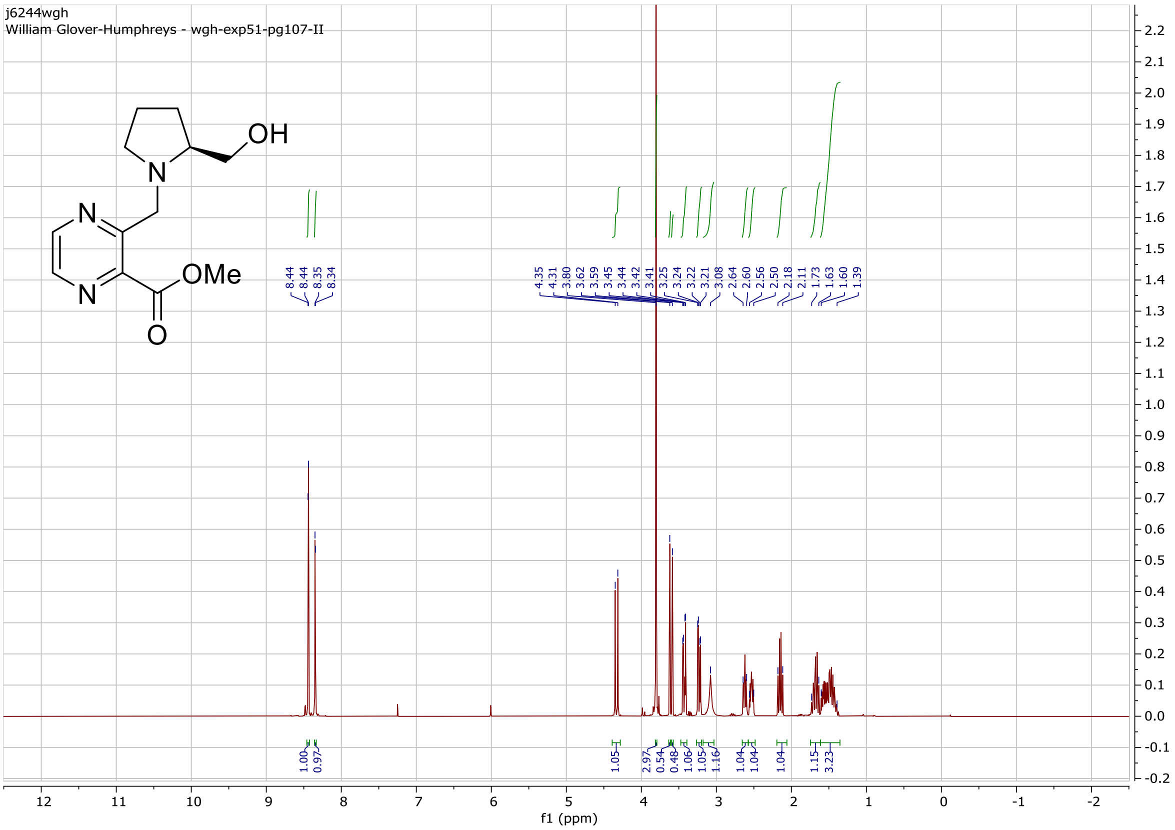

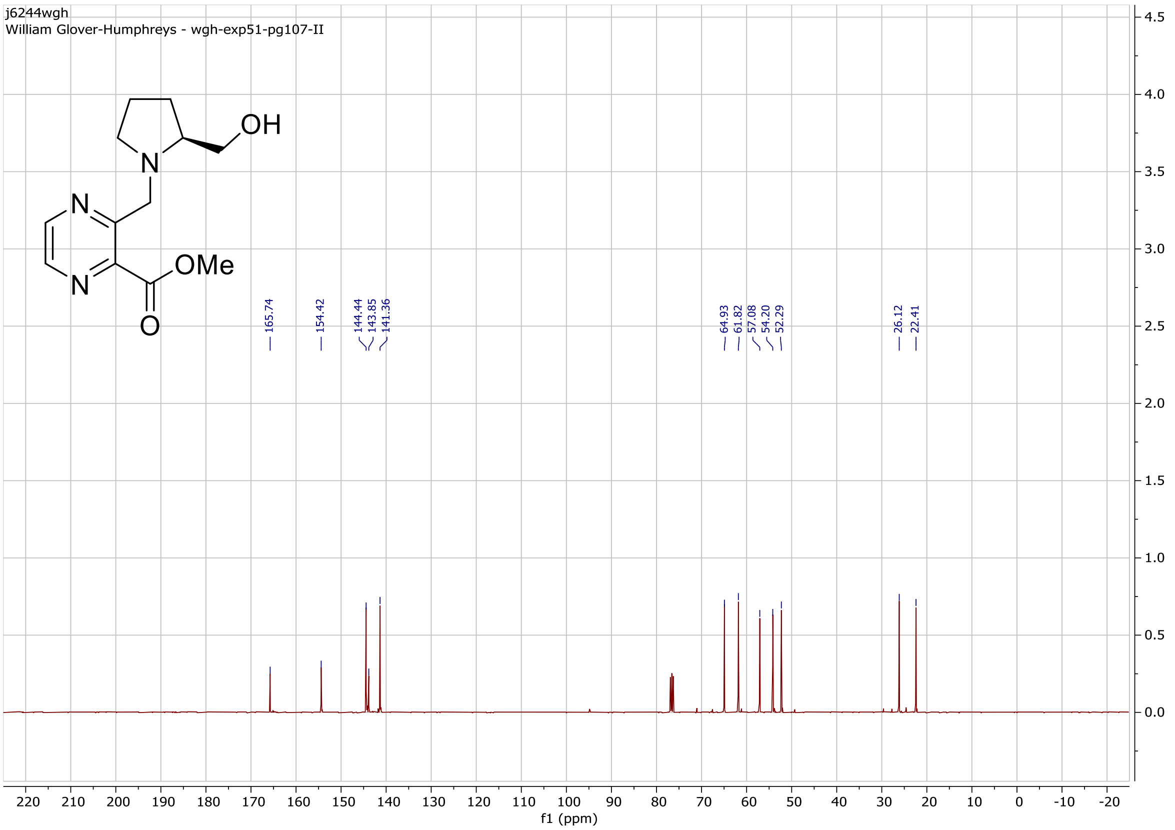

(S)-7a,8,9,10-Tetrahydro-7H-pyrazino[2,3-f] pyrrolo[2,1-c] [1,4] oxazocin-5(12H)-one (43)

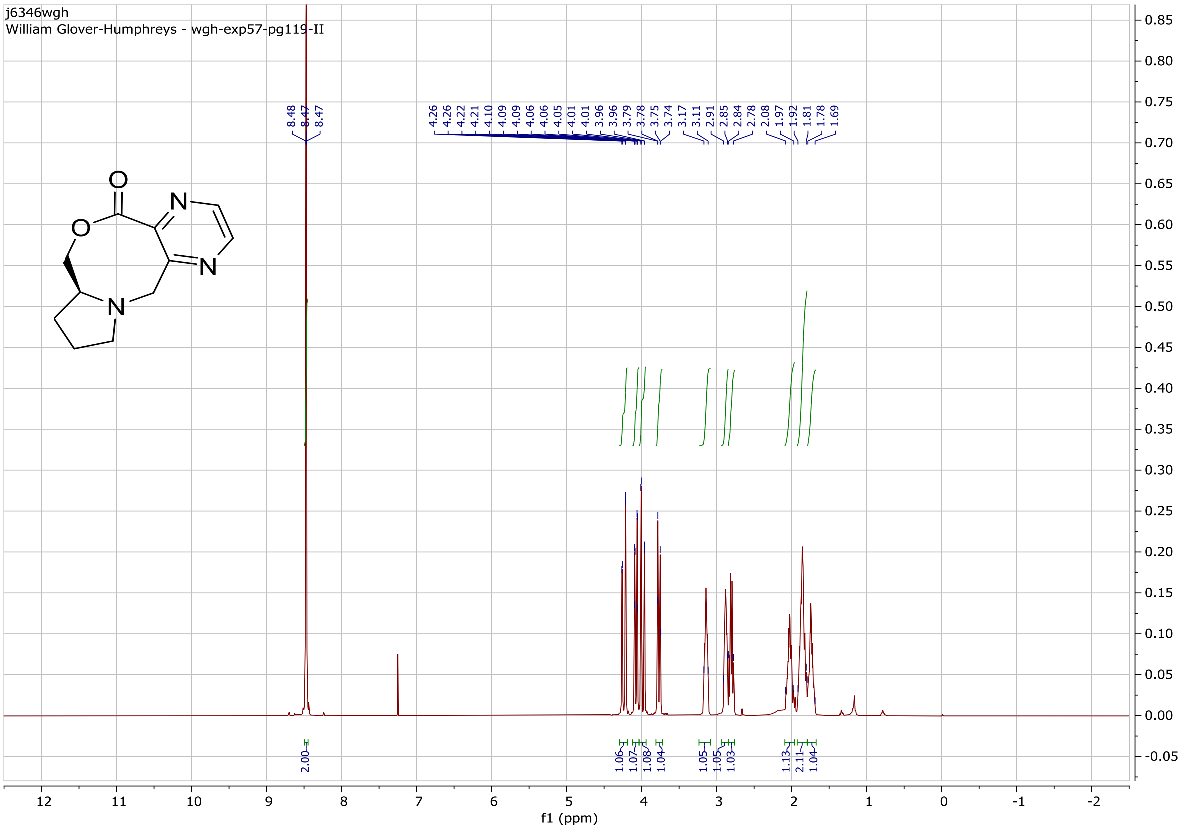

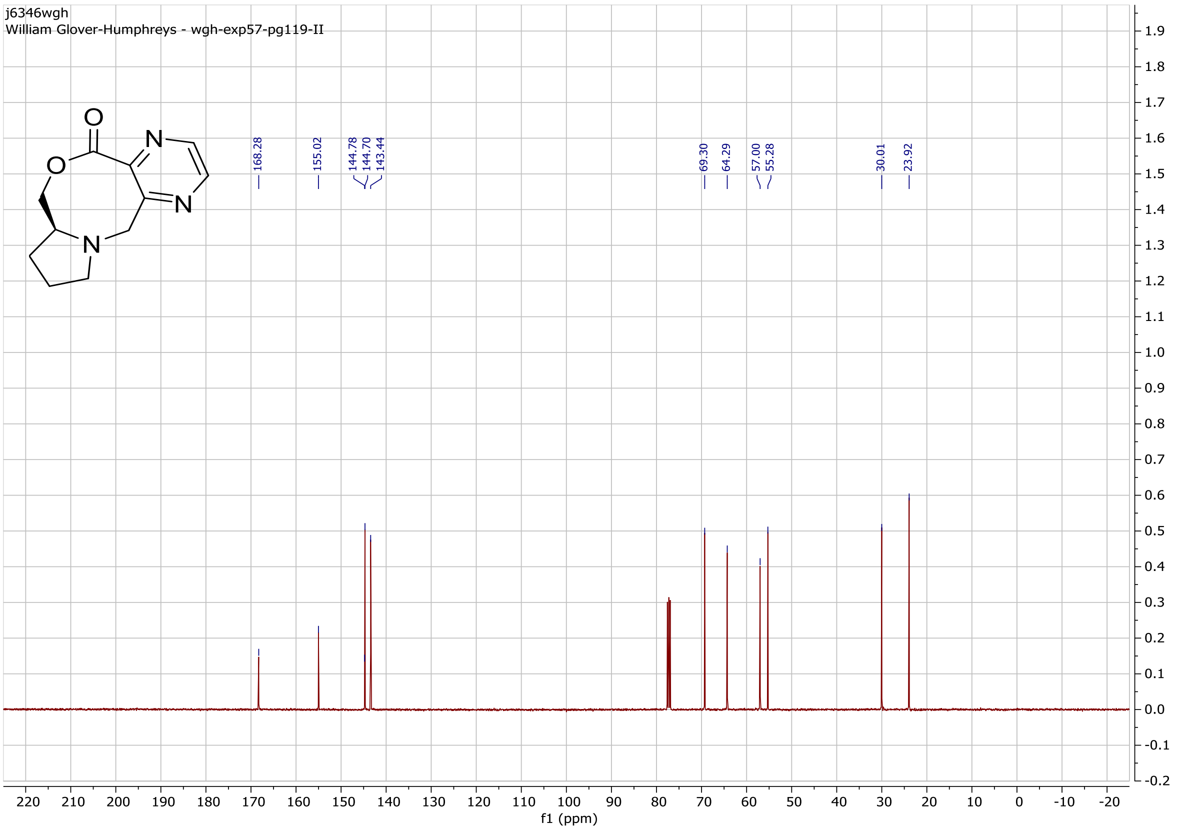

Methyl 2-((2-(2-hydroxyethyl) piperidin-1-yl) methyl) benzoate (S22)

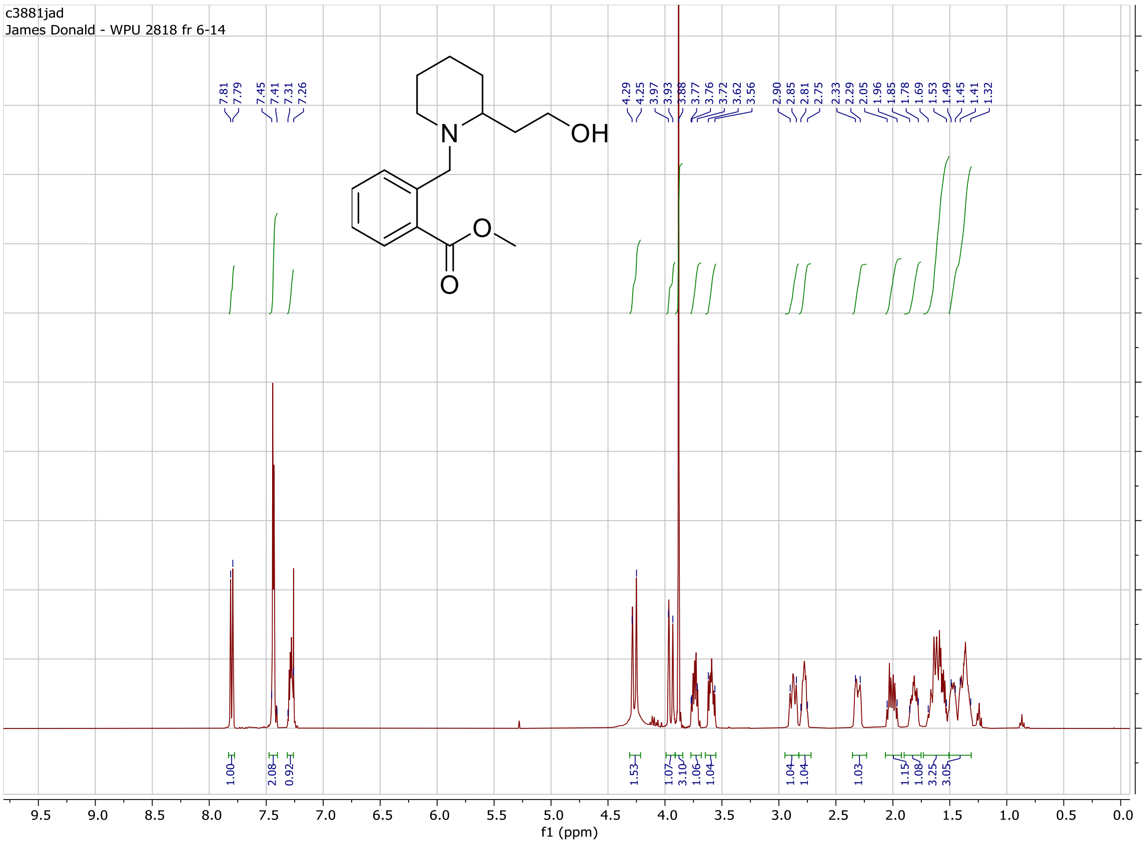

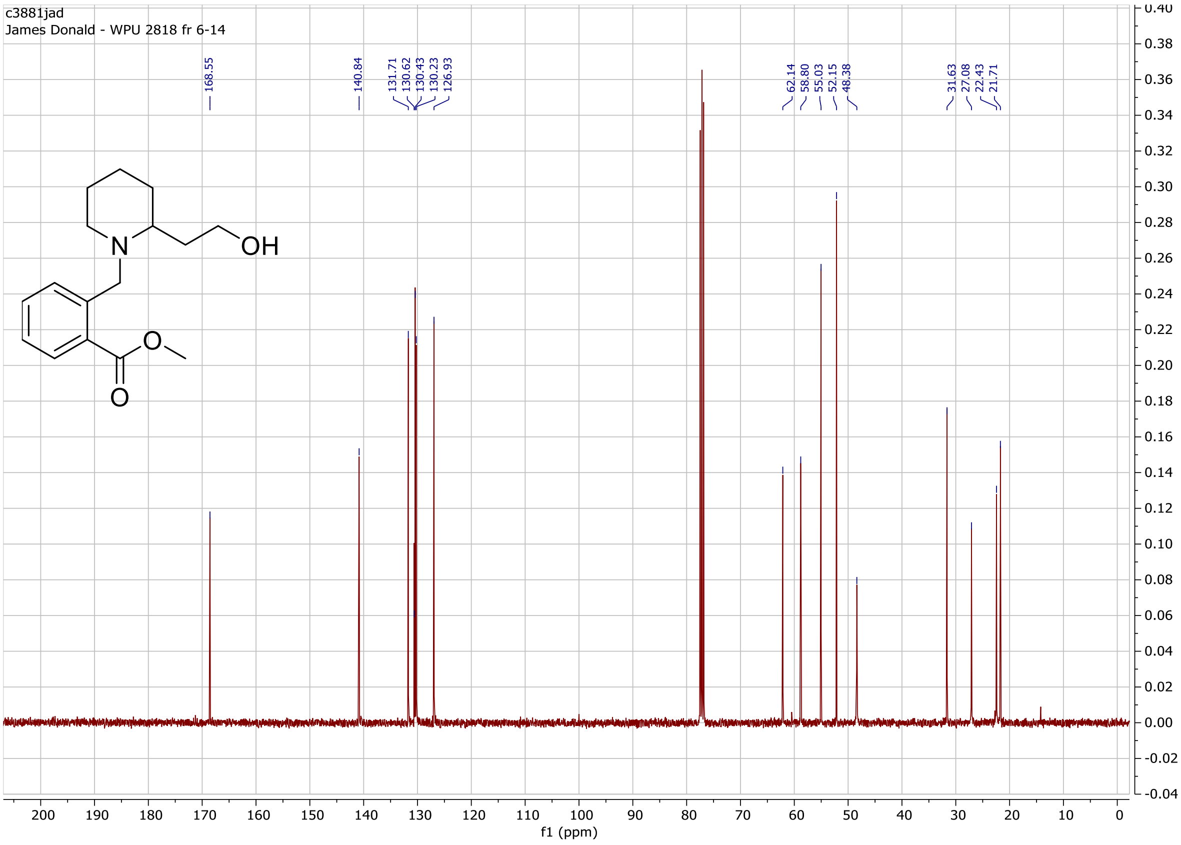

8,8a,9,10,11,12-Hexahydro-7H-benzo[g]pyrido[2,1-d] [1,5] oxazonin-5(14H)-one (44)

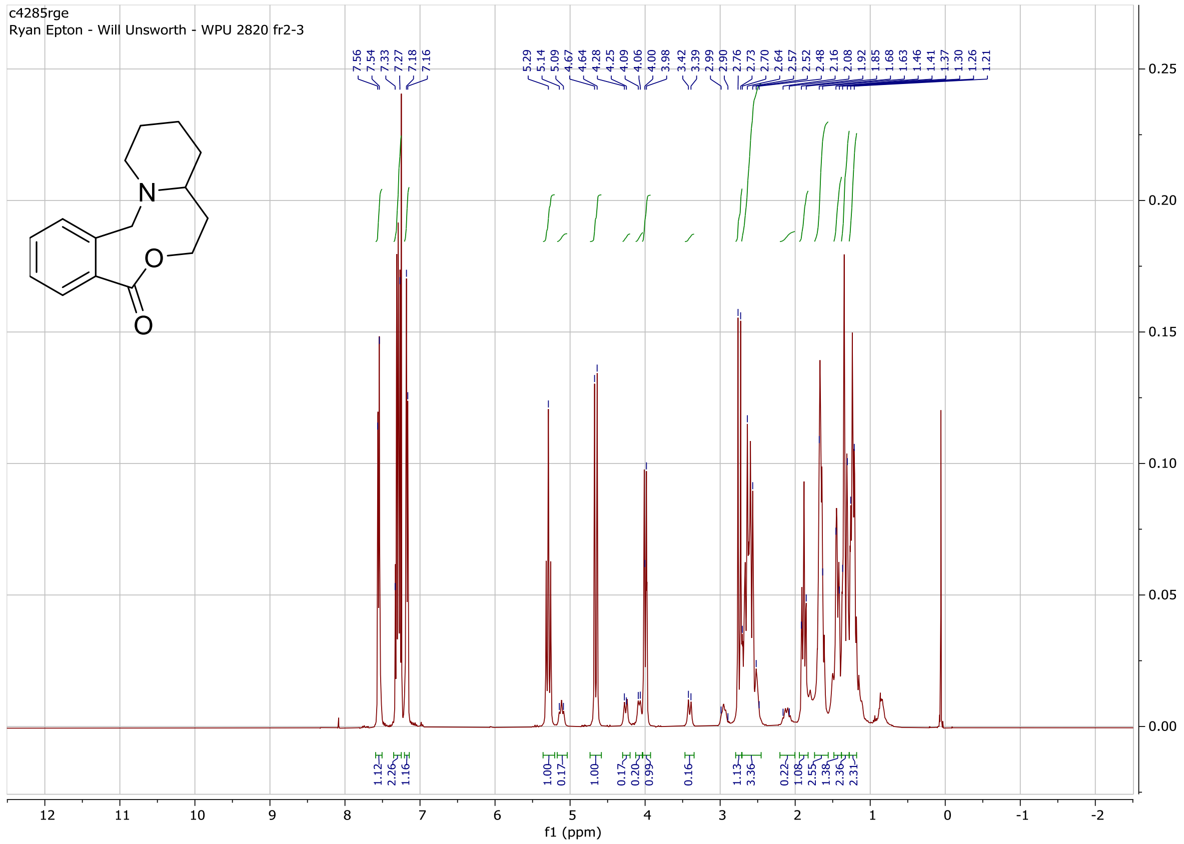

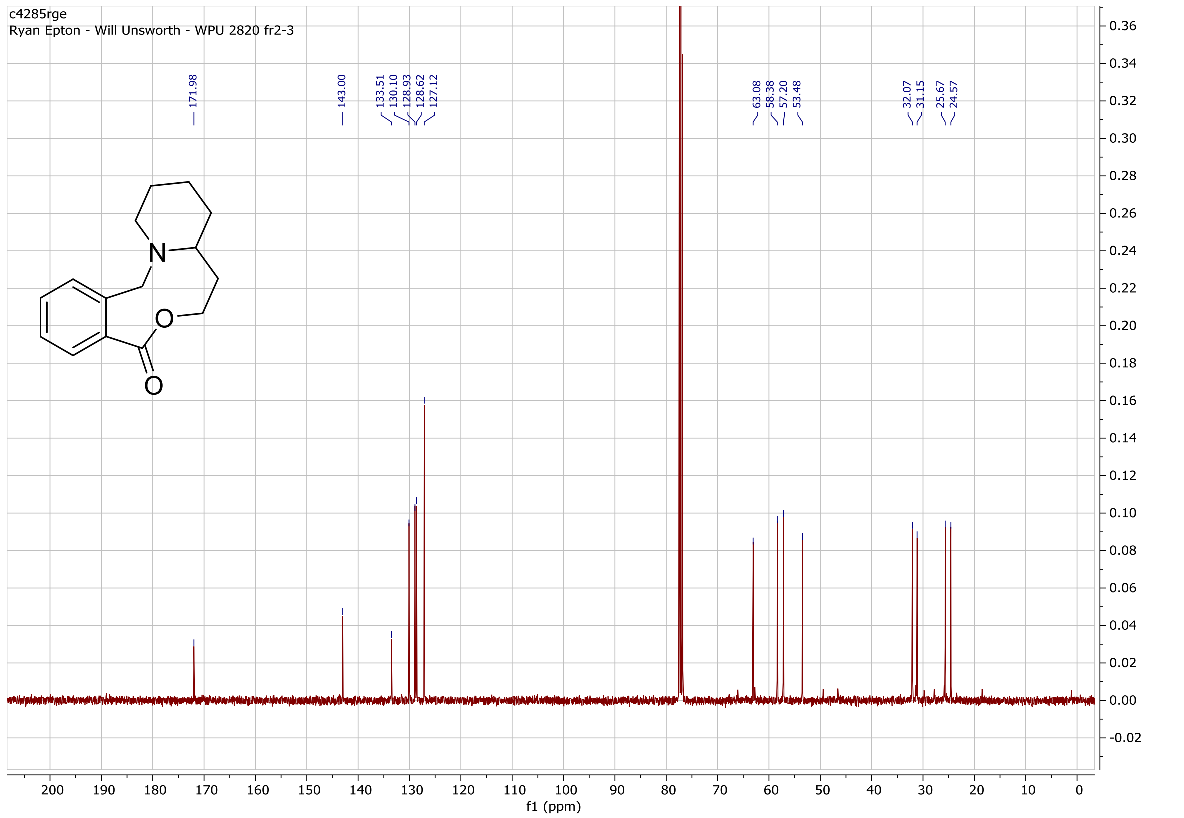

Methyl 2-((2-(2-hydroxyethyl) piperidin-1-yl) methyl) nicotinate (S23)

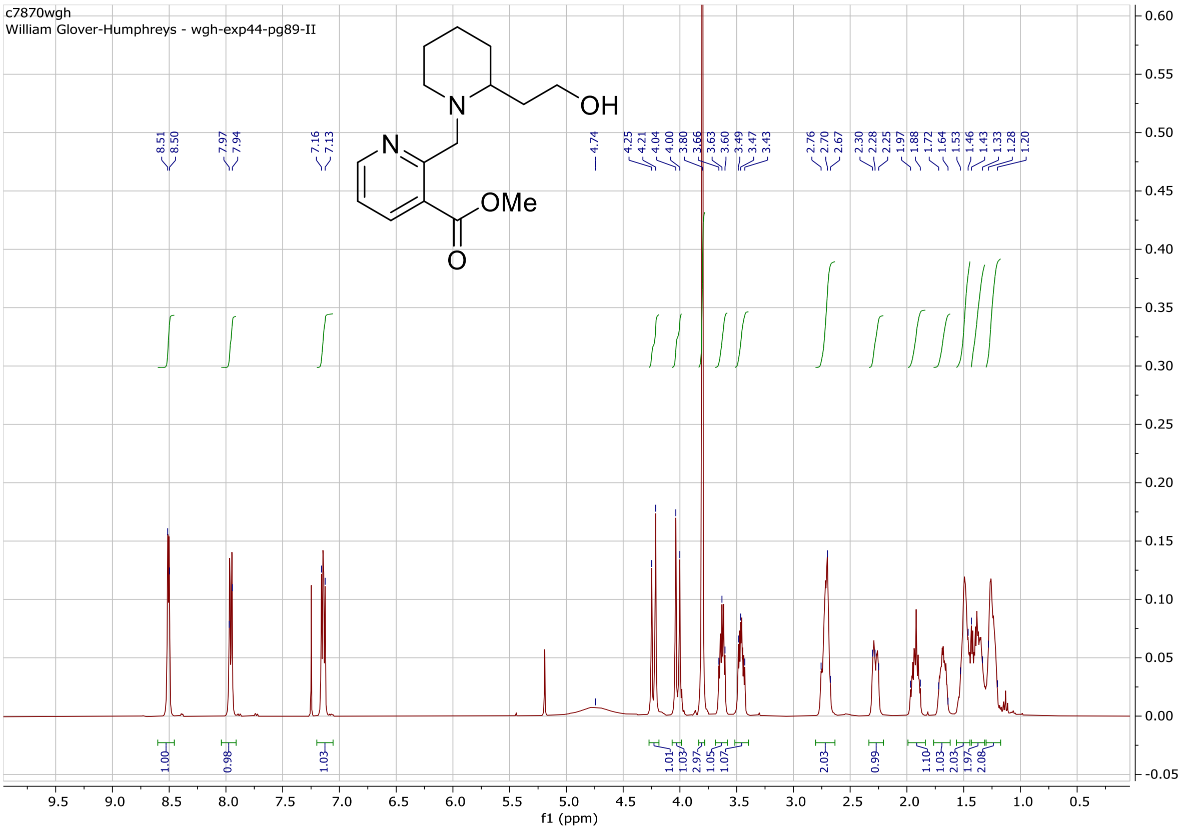

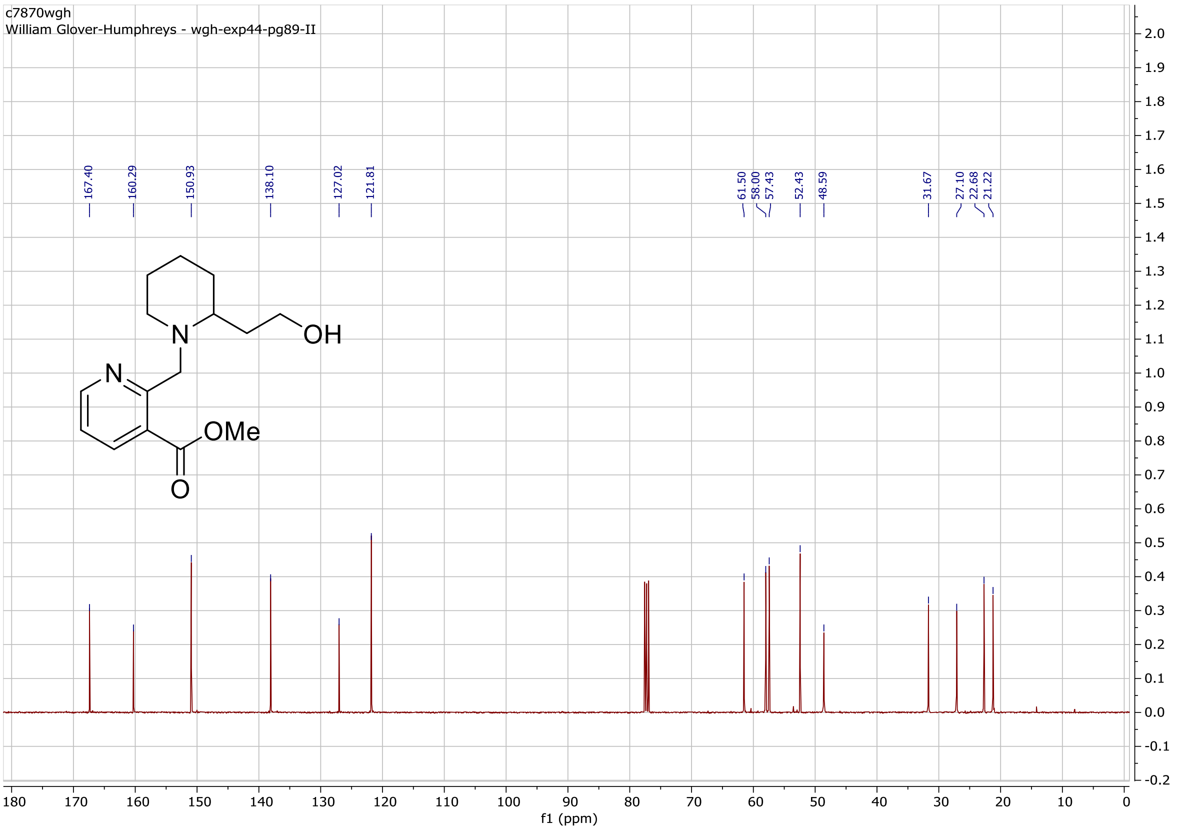

8,8a,9,10,11,12-Hexahydro-7H-dipyrido[2,1-d:2',3'-g] [1,5] oxazonin-5 (14H)-one (45)

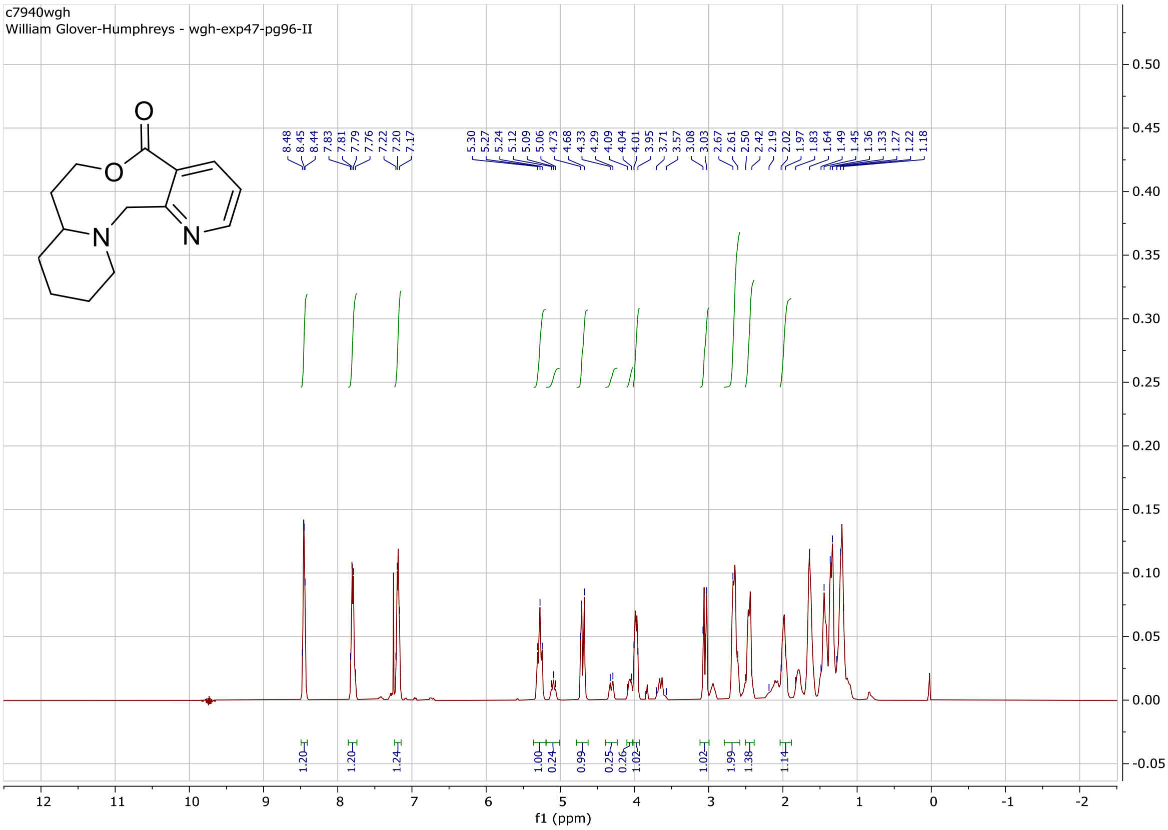

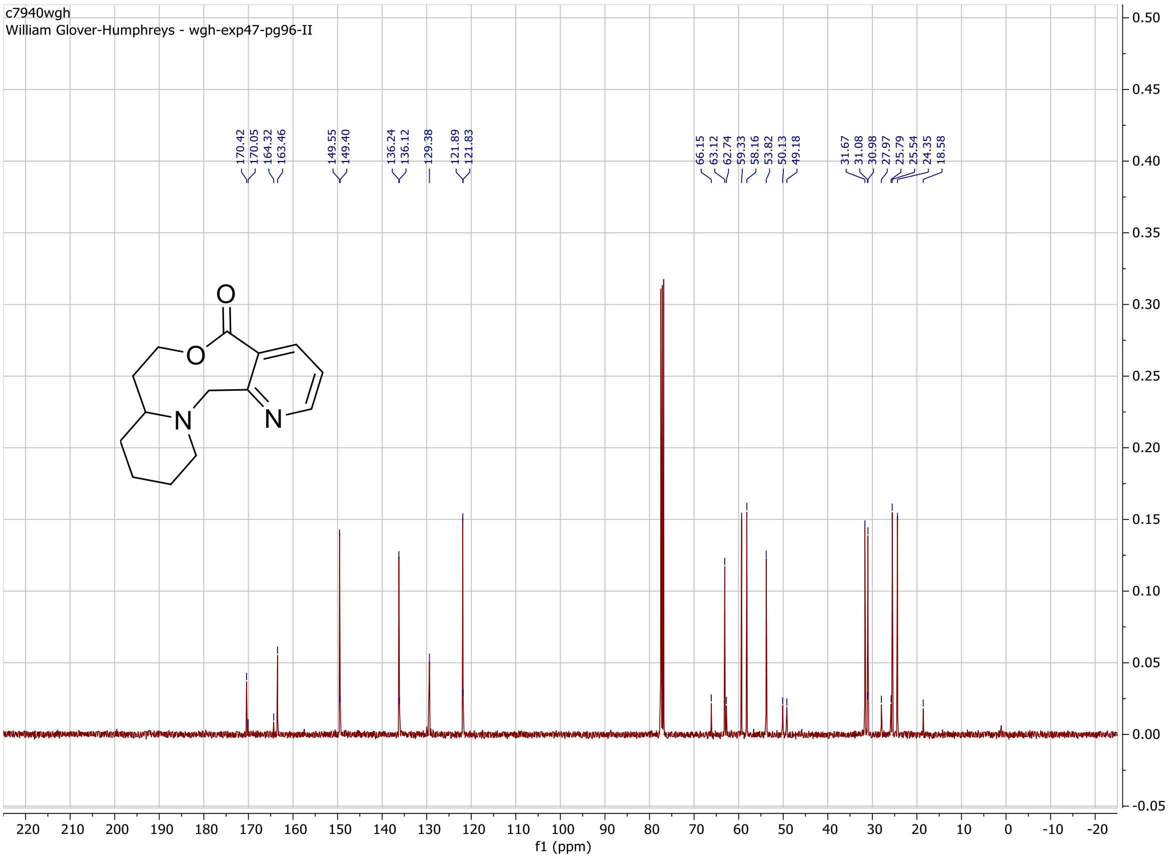

Methyl 3-((2-(2-hydroxyethyl) piperidin-1-yl) methyl) pyrazine-2-carboxylate (S24)

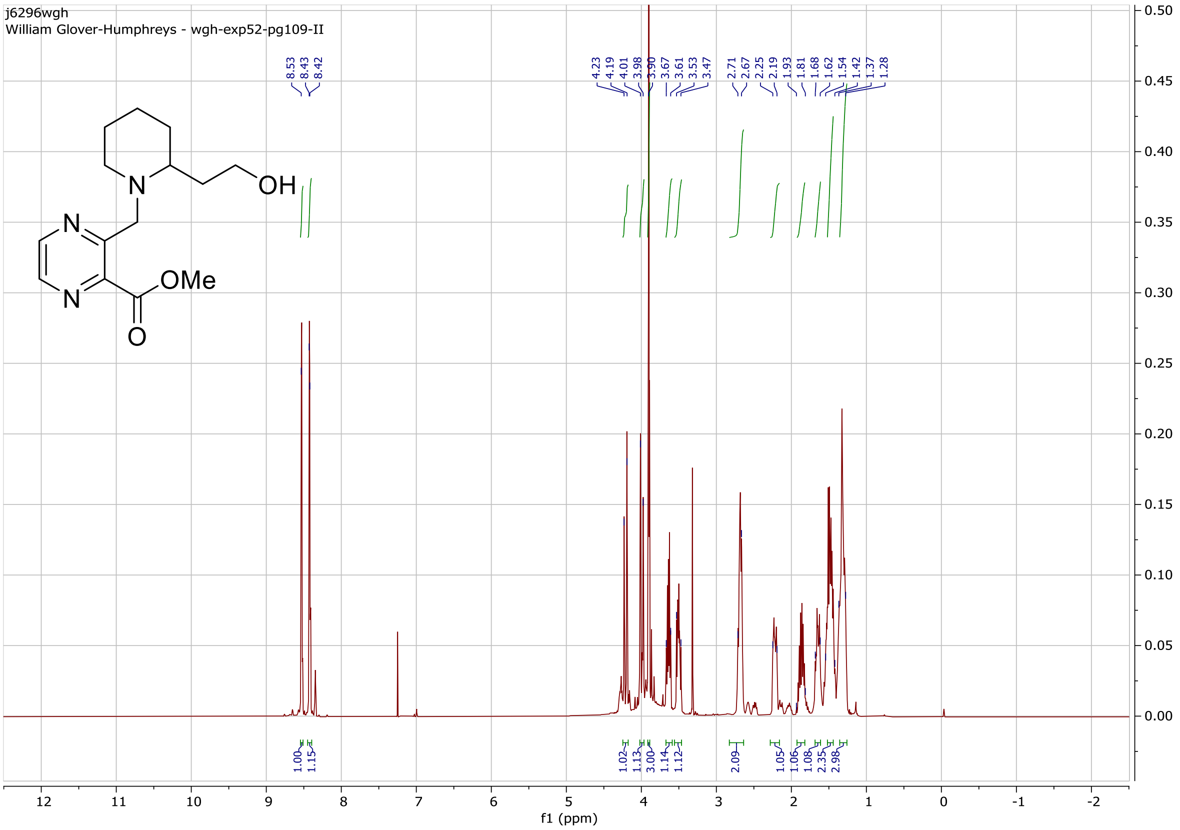

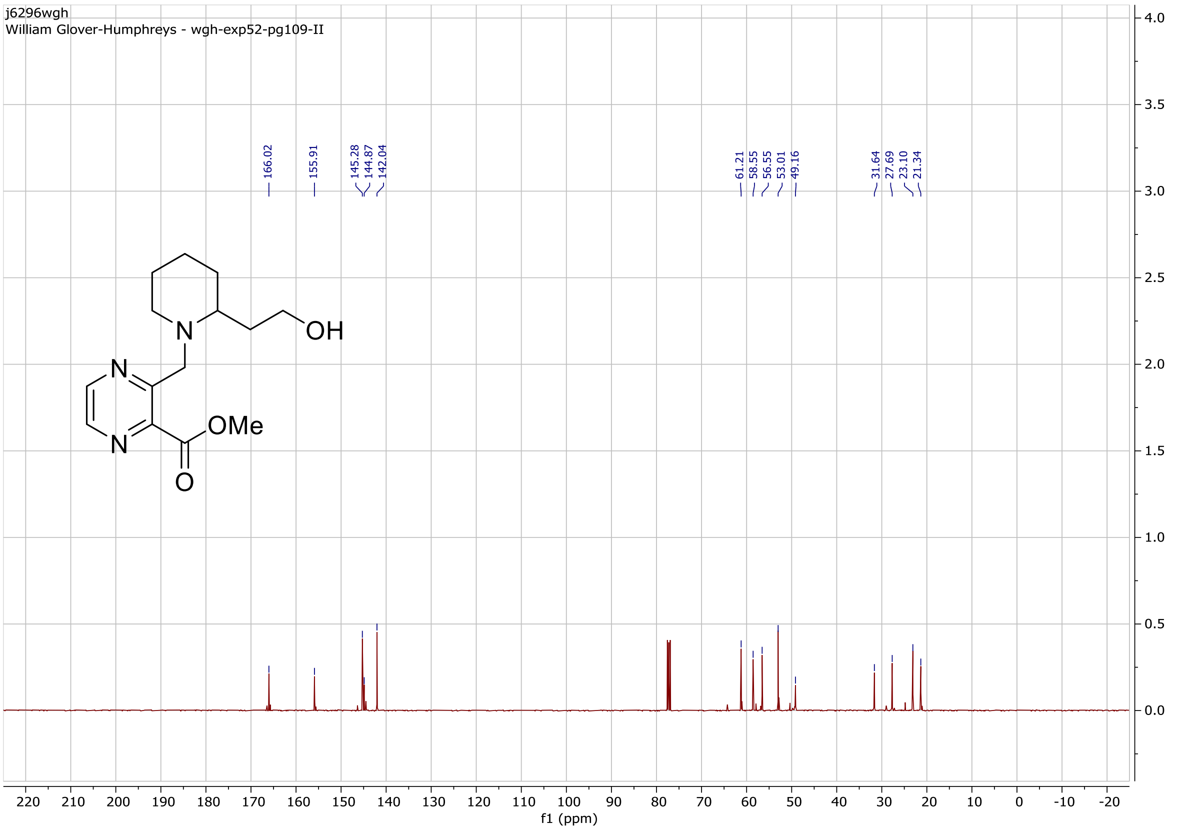

8,8a,9,10,11,12-Hexahydro-7H-pyrazino[2,3-g] pyrido[2,1-d] [1,5] oxazonin-5(14H)-one (46)

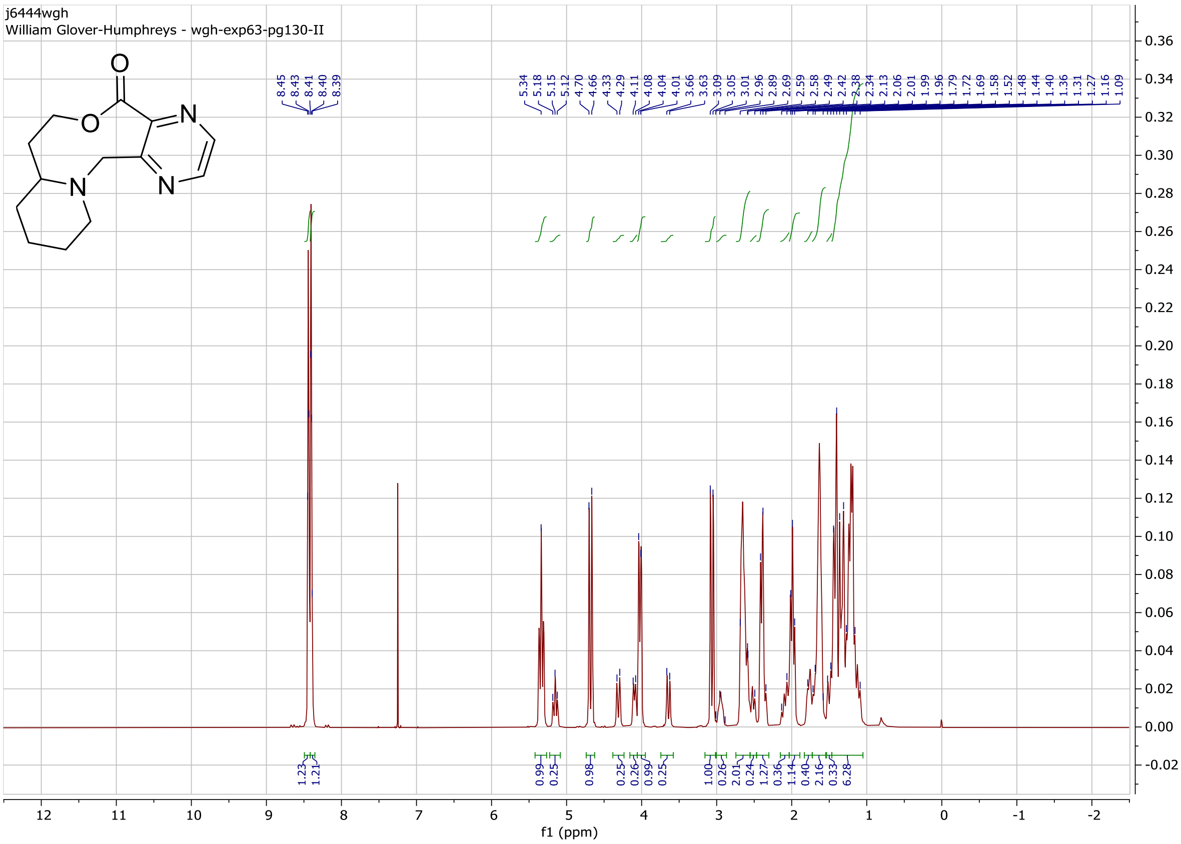

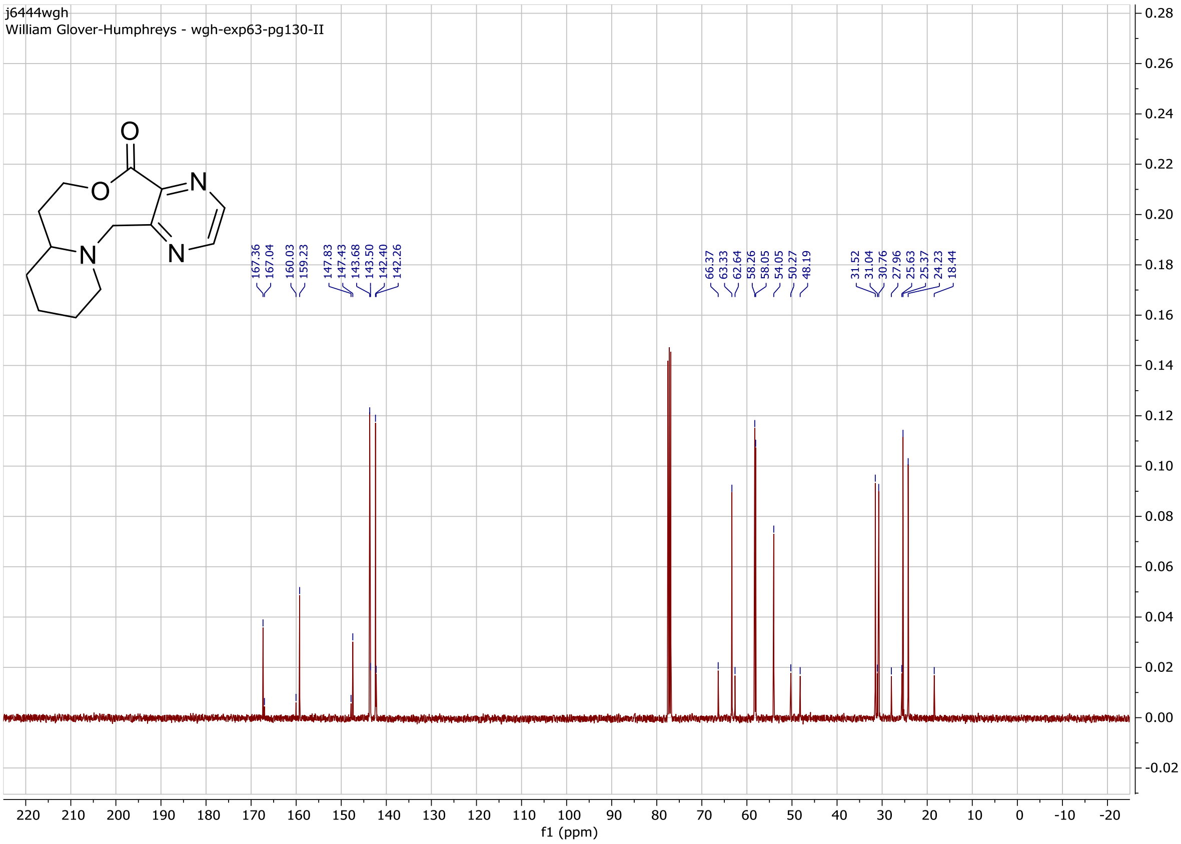

Methyl 2-(((2-hydroxyethyl)thio)methyl)benzoate (S25)

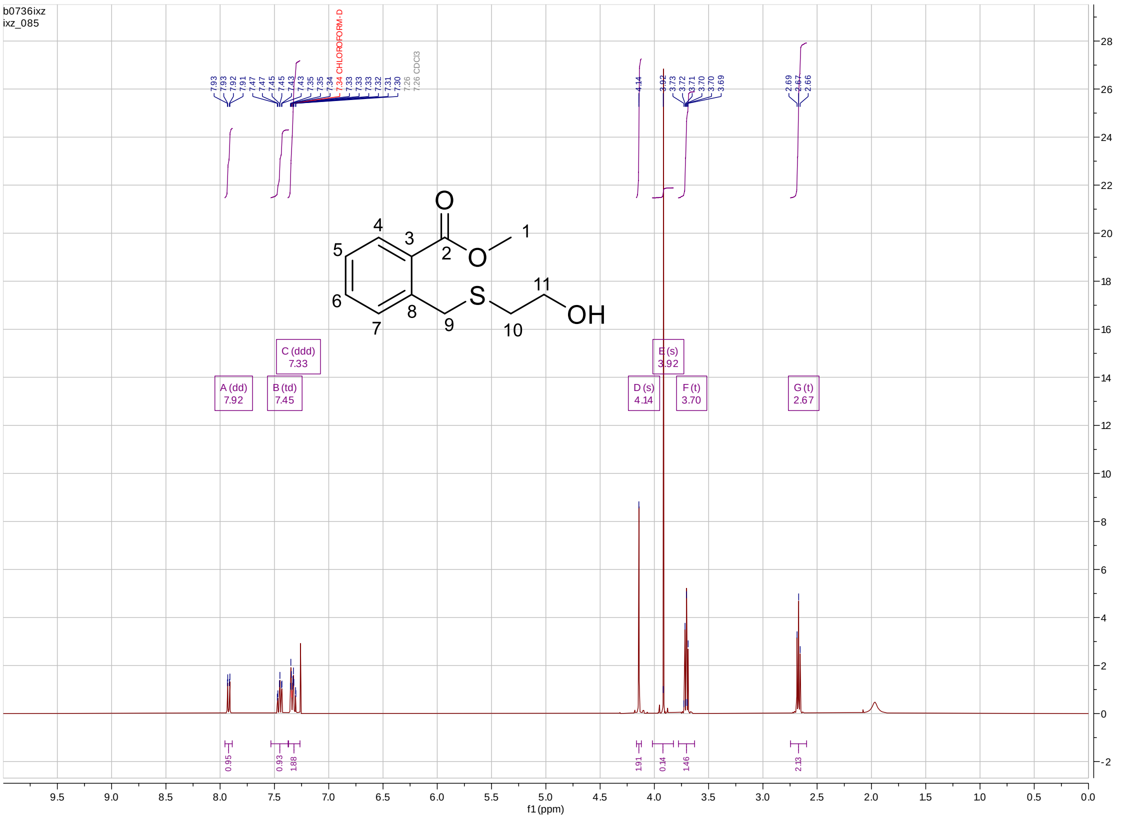

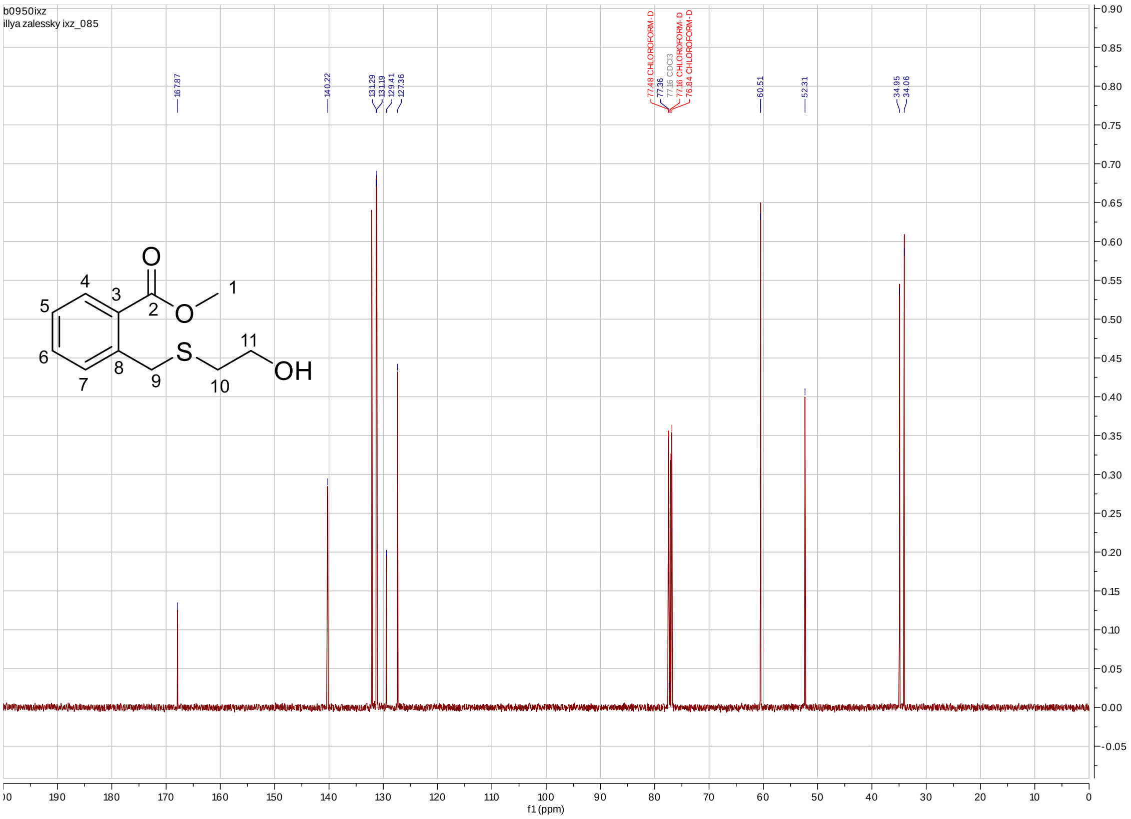

2-(((2-Hydroxyethyl)thio)methyl)benzoic acid (S26)

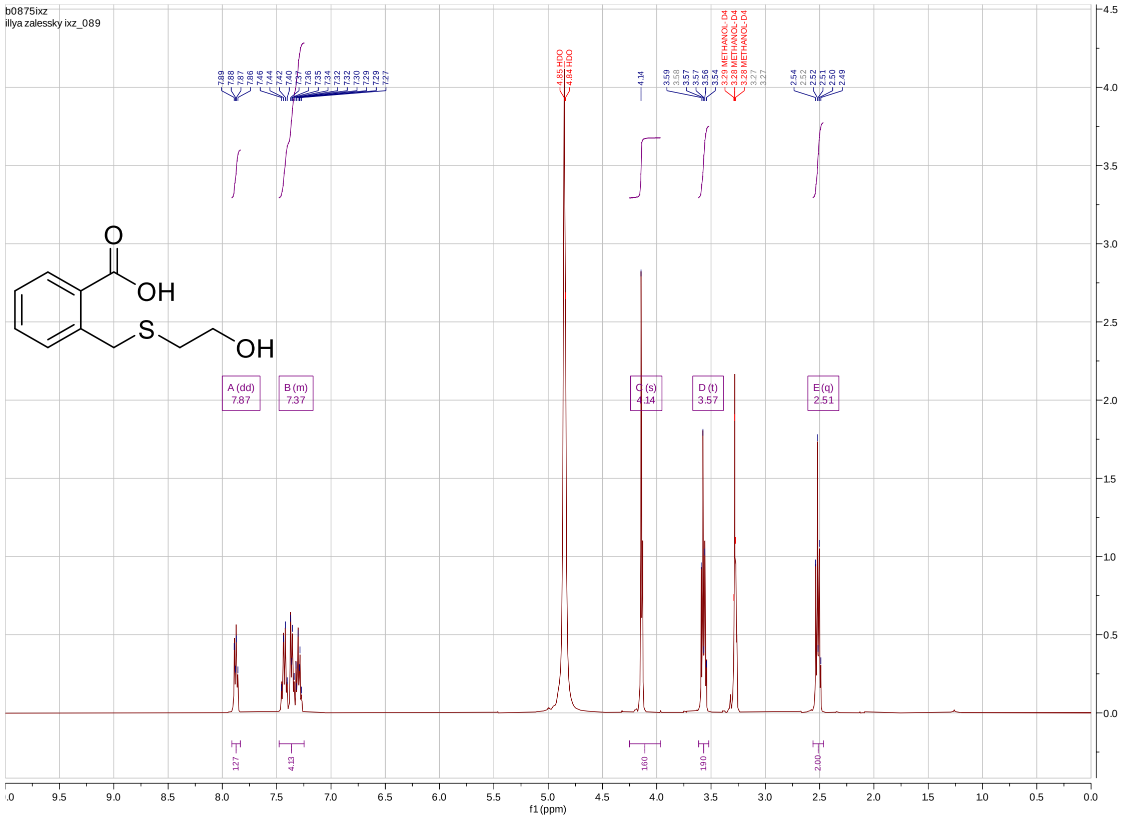

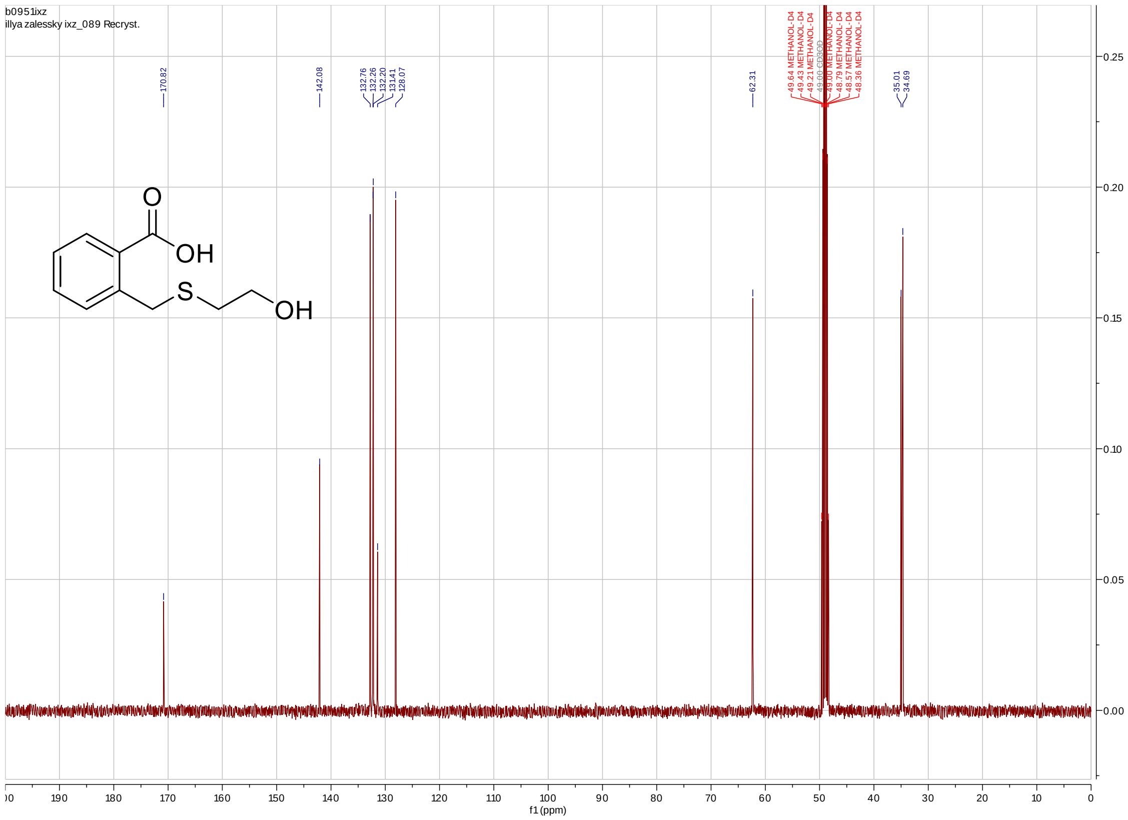

3,4-Dihydrobenzo[*f*][1,4]oxathiocin-1(6*H*)-one (47)

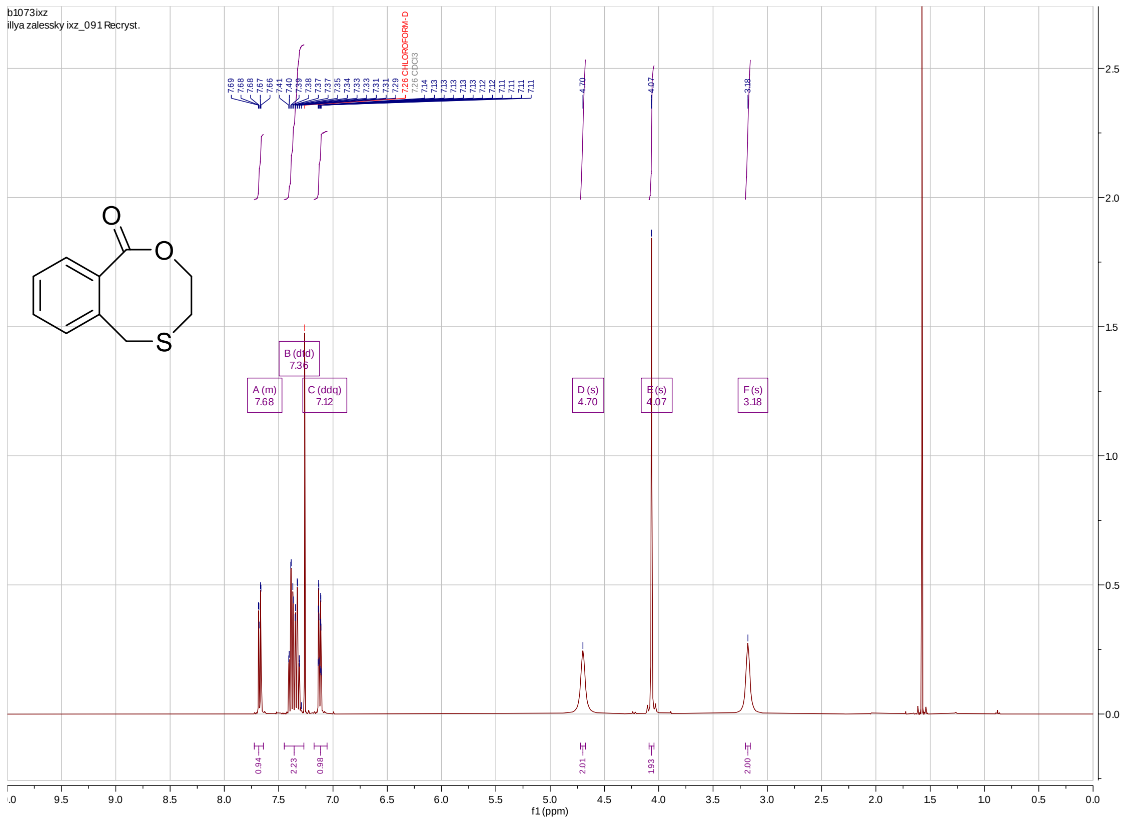

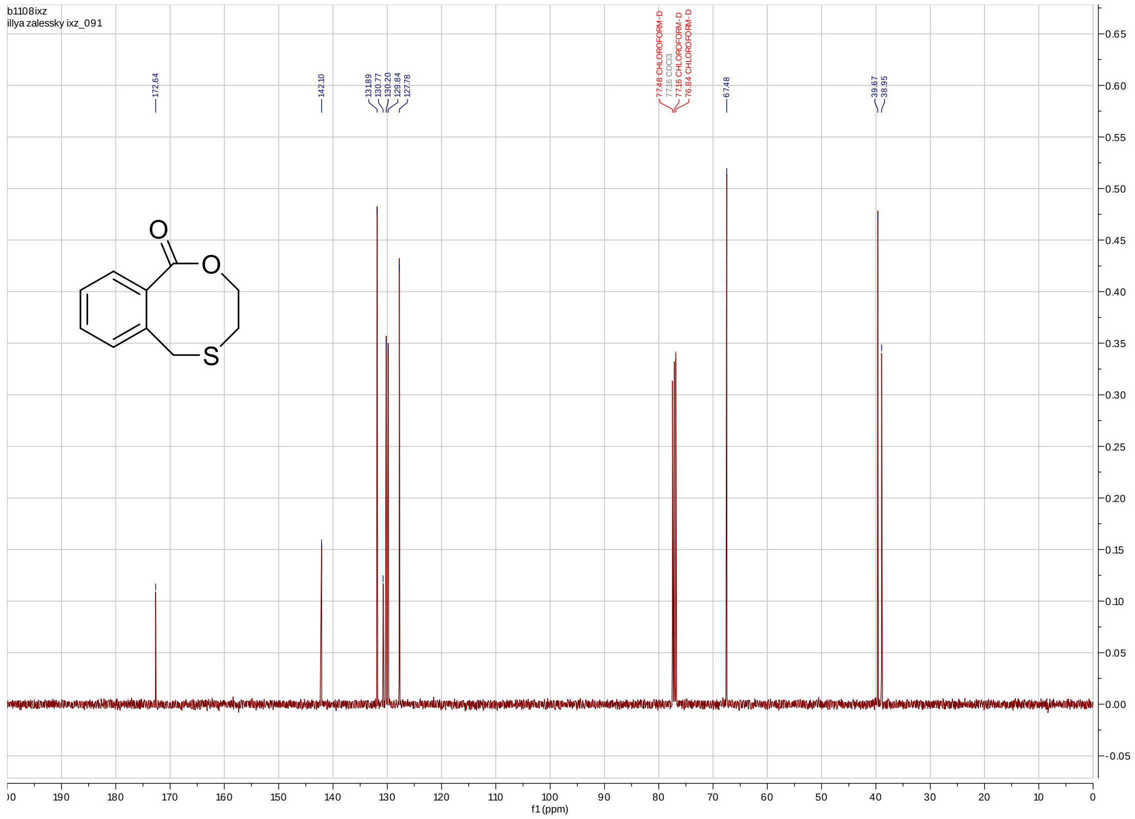

Methyl 5-fluoro-2-(((3-hydroxypropyl)thio)methyl)benzoate (S29)

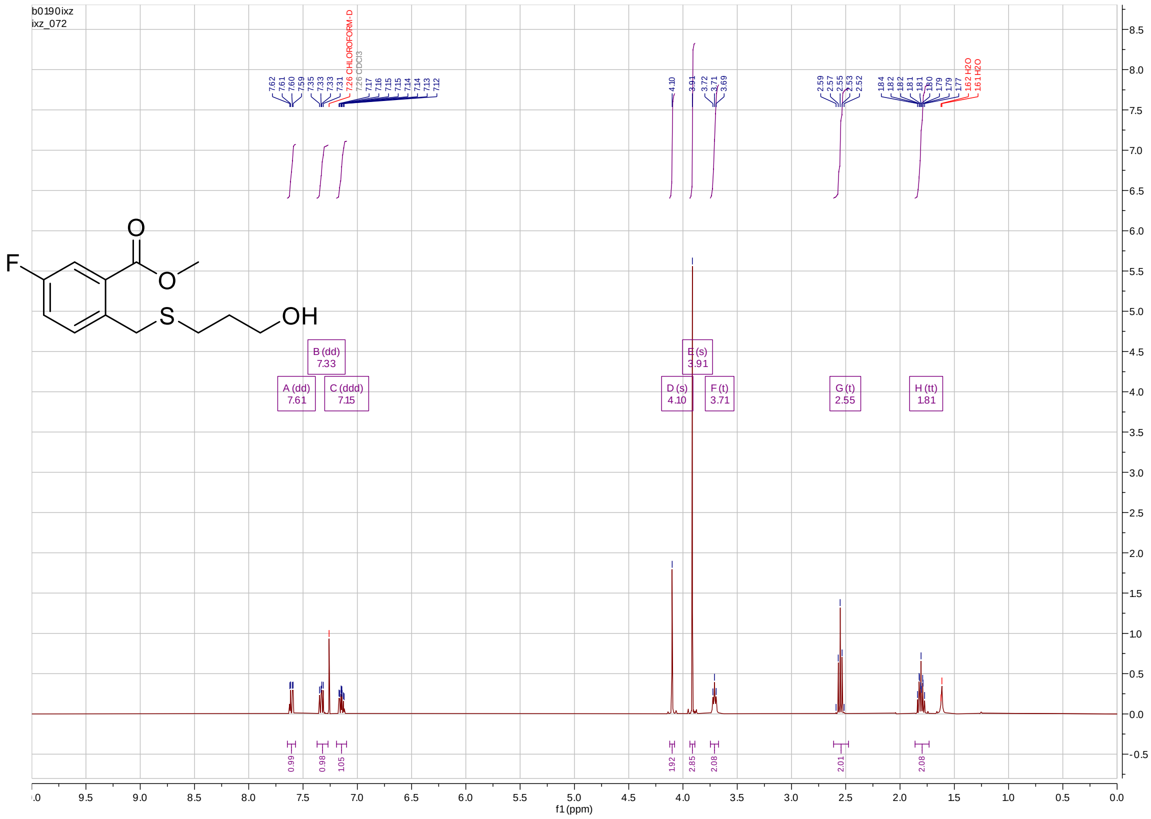

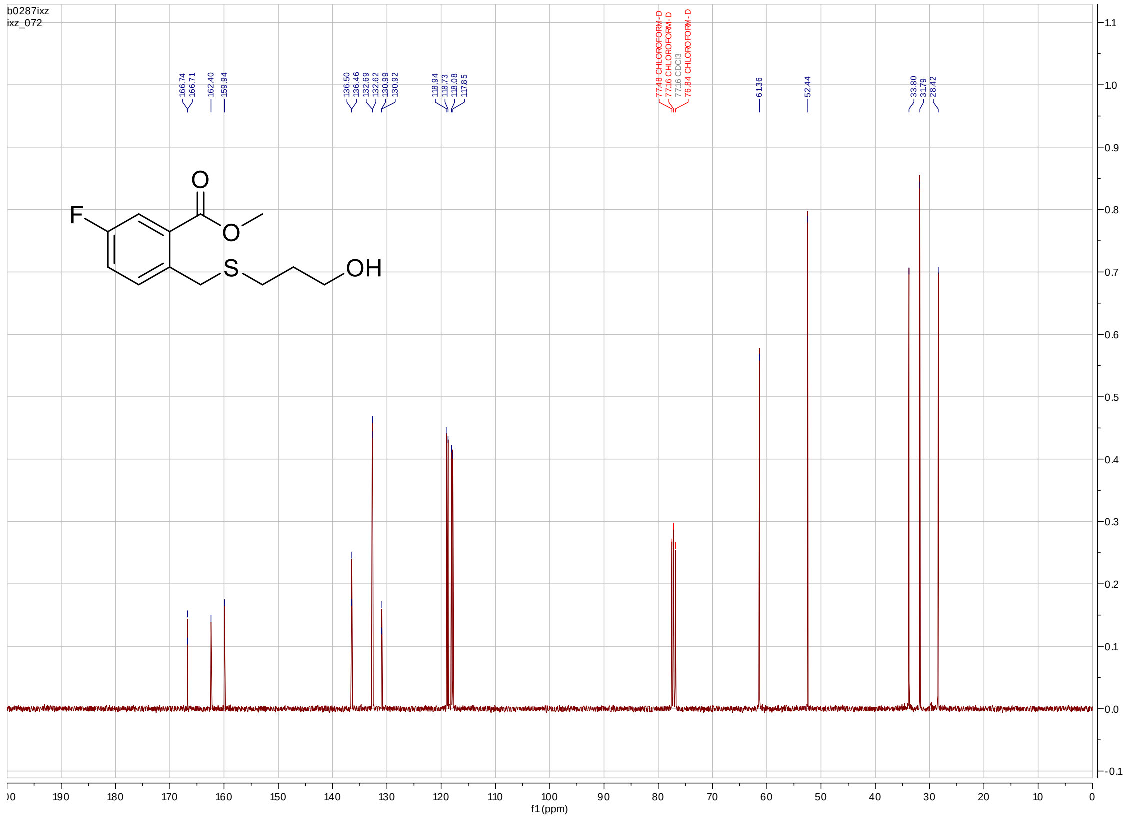

Methyl 5-fluoro-2-(((3-hydroxypropyl)thio)methyl)benzoate (S29)

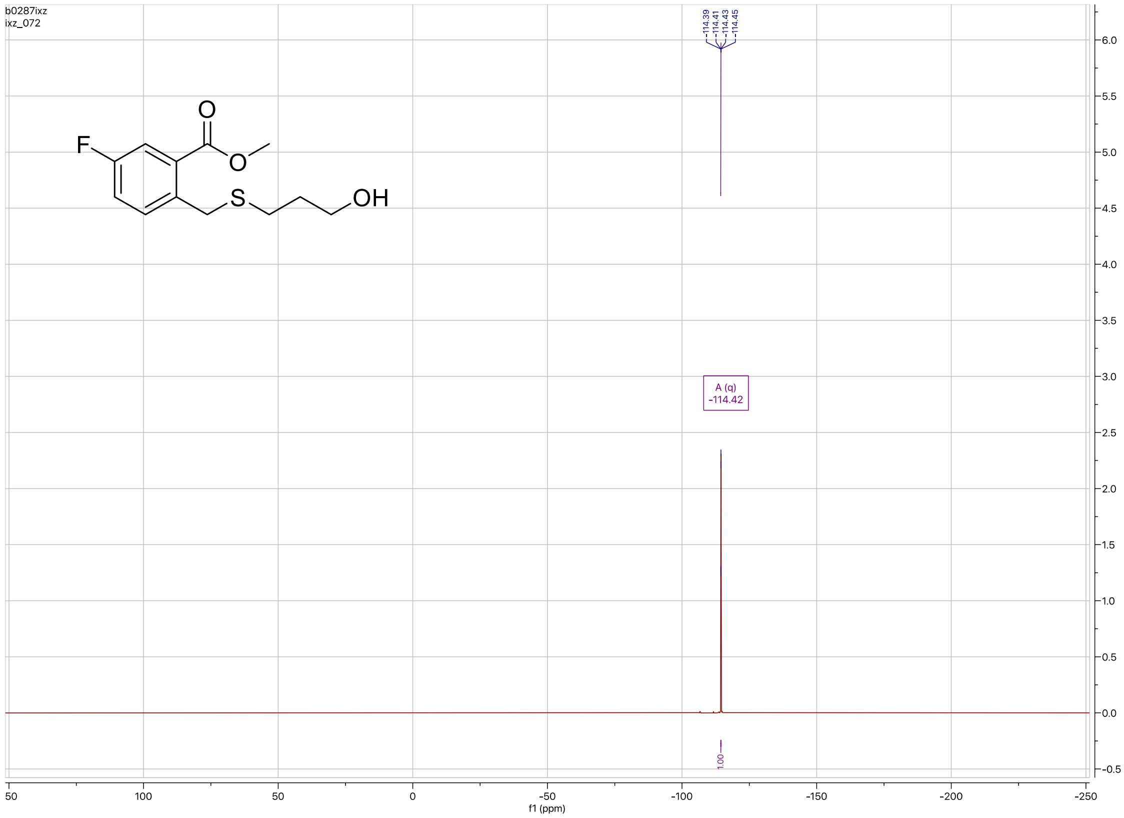

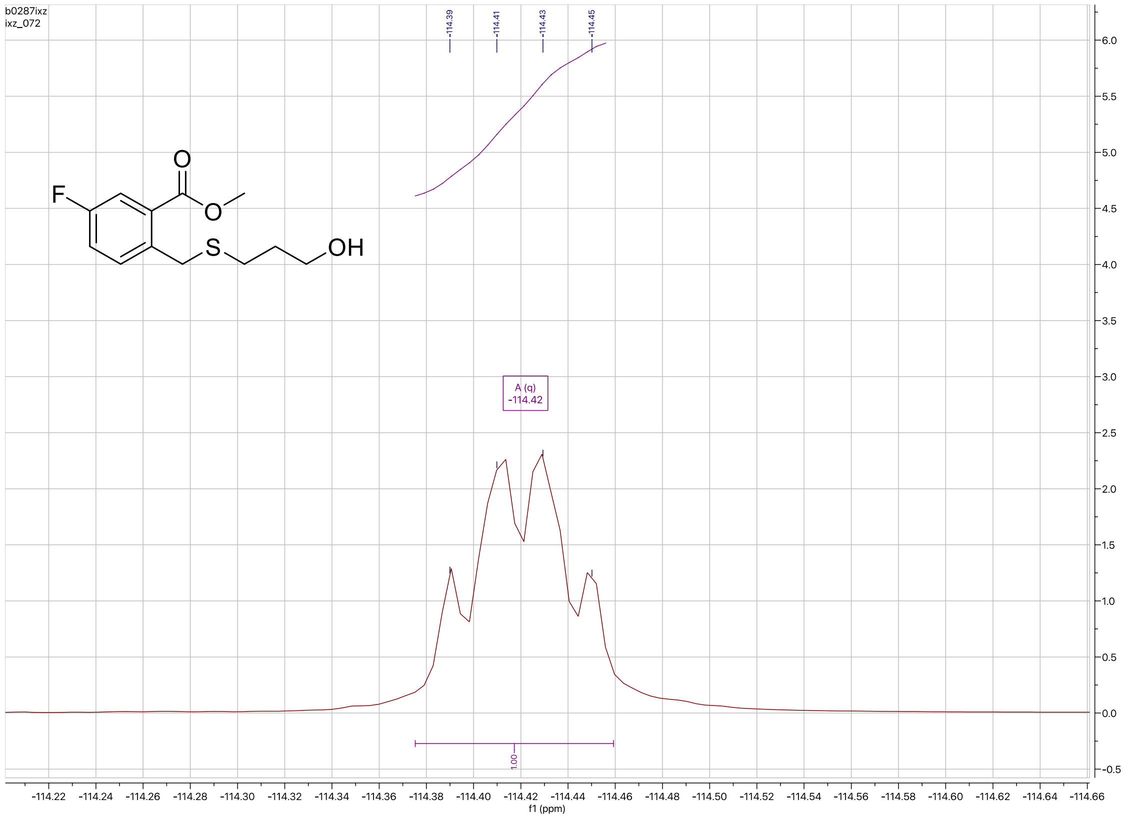

5-Fluoro-2-(3-hydroxypropylsulfanylmethyl)benzoic acid (S30)

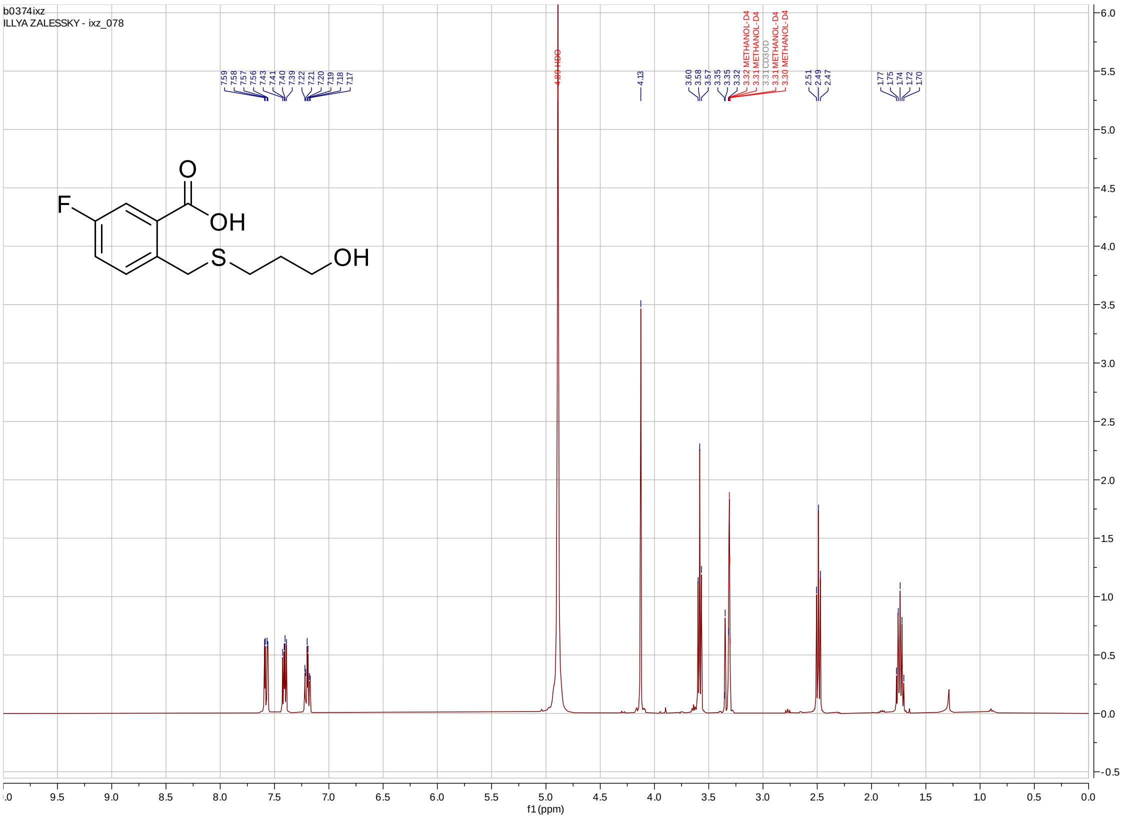

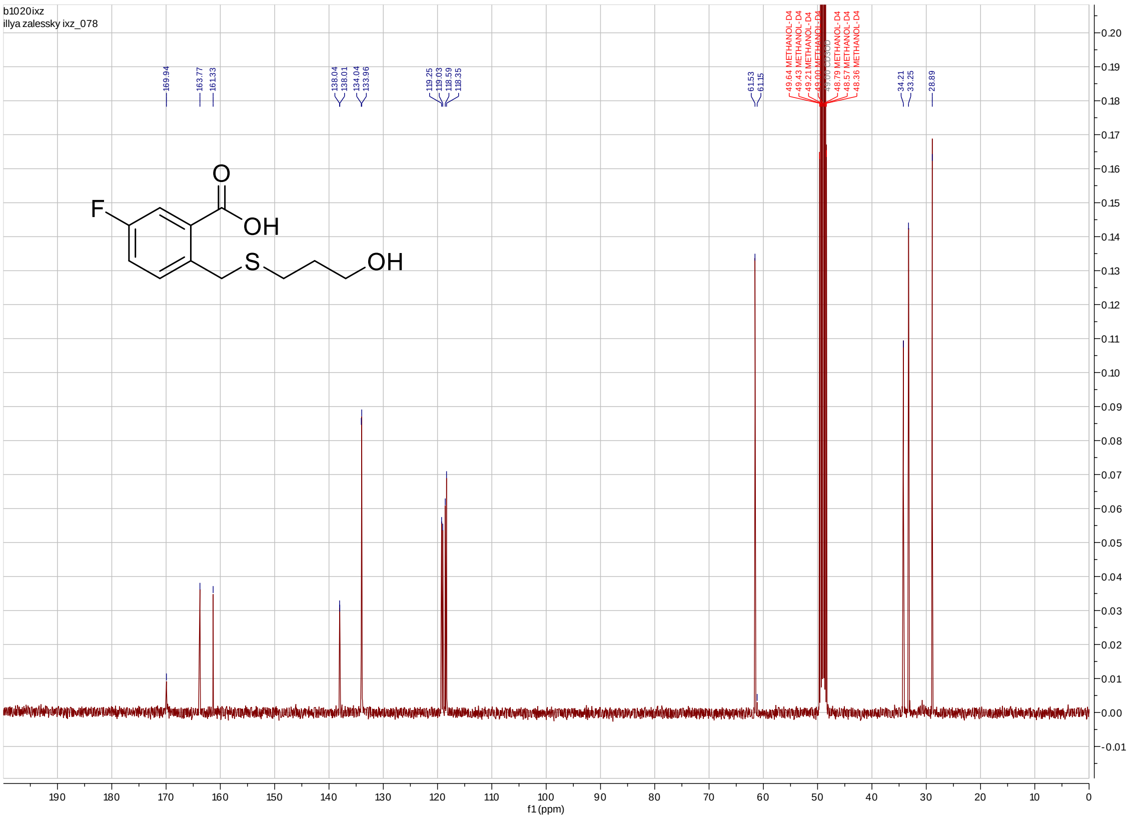

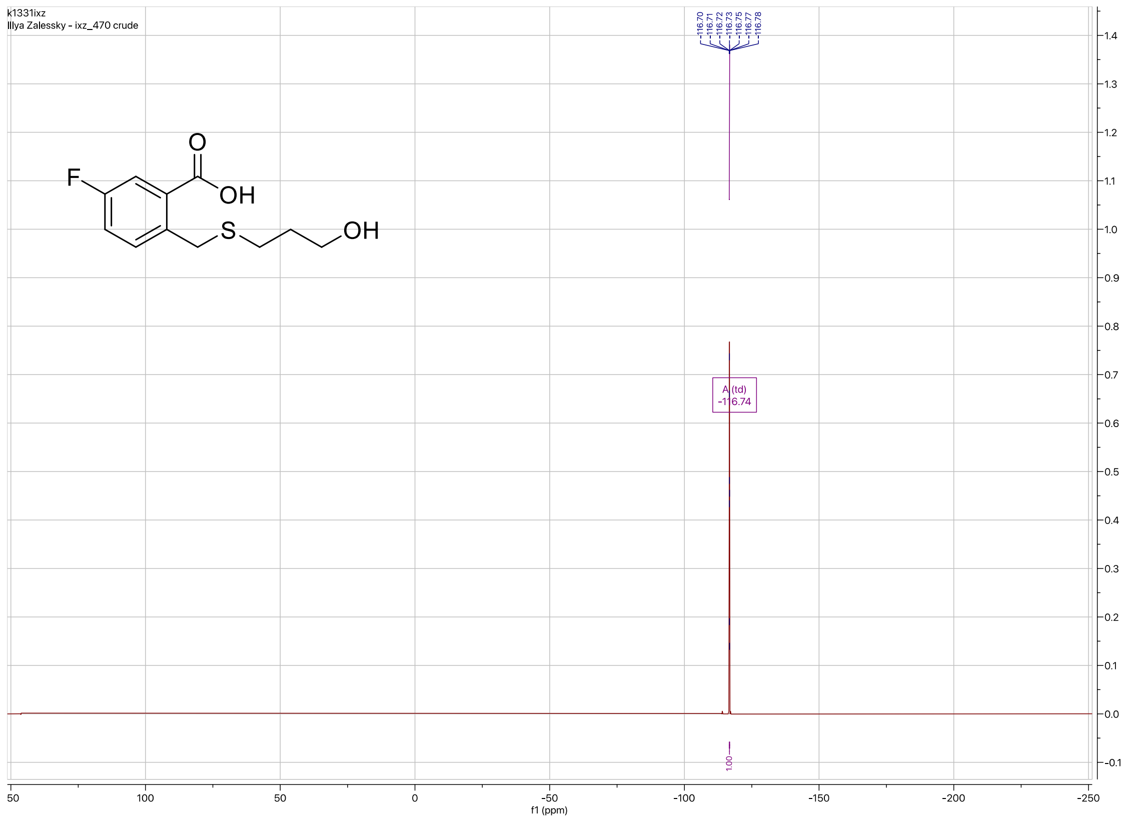
k1331ixz
Illya Zalessky - ixz_470 crude
-116.70
-116.71
-116.72
-116.73
-116.75
-116.77
-116.78
A (td)
-116.74
1.00
f1 (ppm)

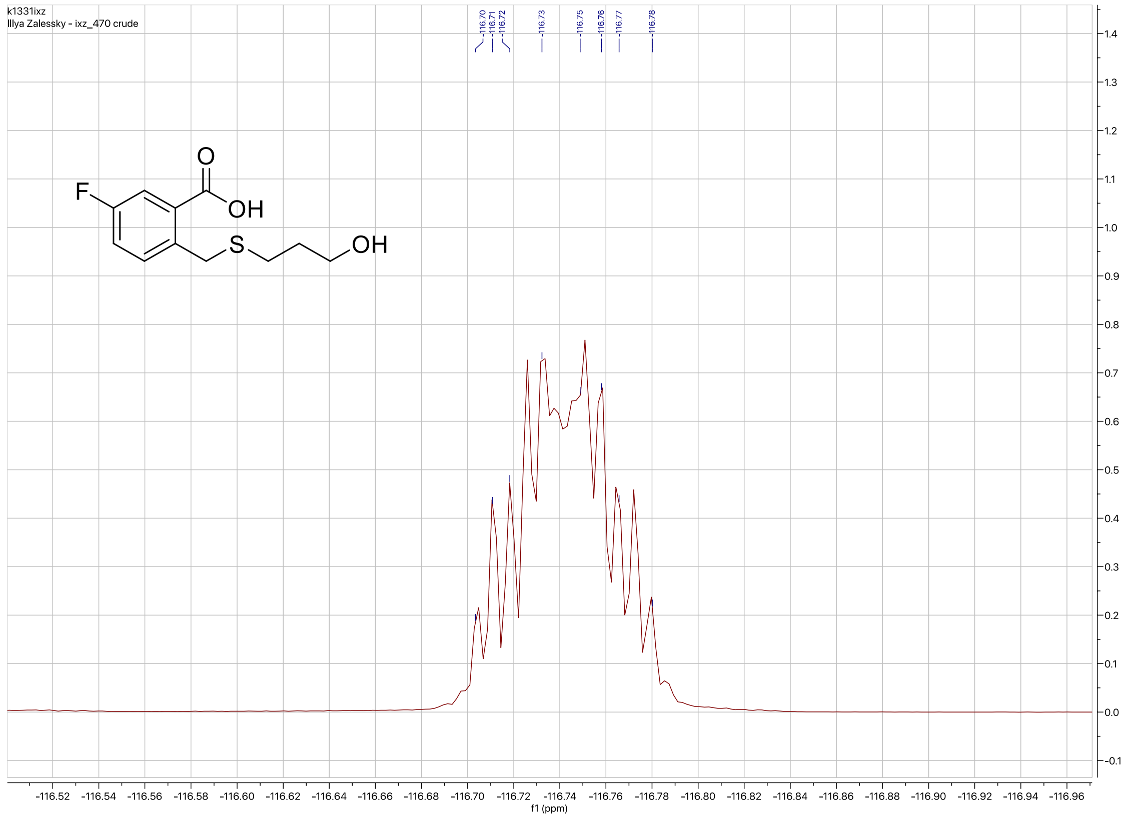
k1331ixz
Illya Zalessky - ixz_470 crude
-116.70
-116.71
-116.72
-116.73
-116.75
-116.76
-116.77
-116.78
f1 (ppm)

10-Fluoro-4,5-dihydro-3*H*-benzo[*g*][1,5]oxathionin-1(7*H*)-one (48)

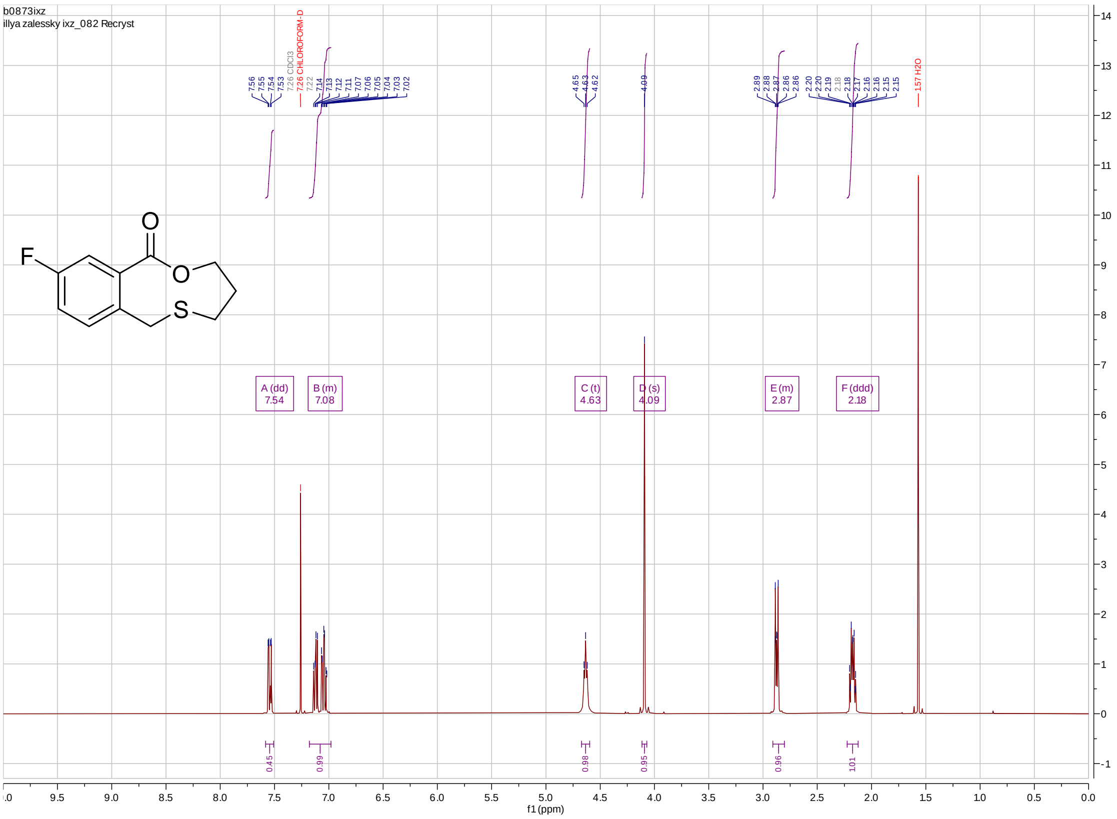

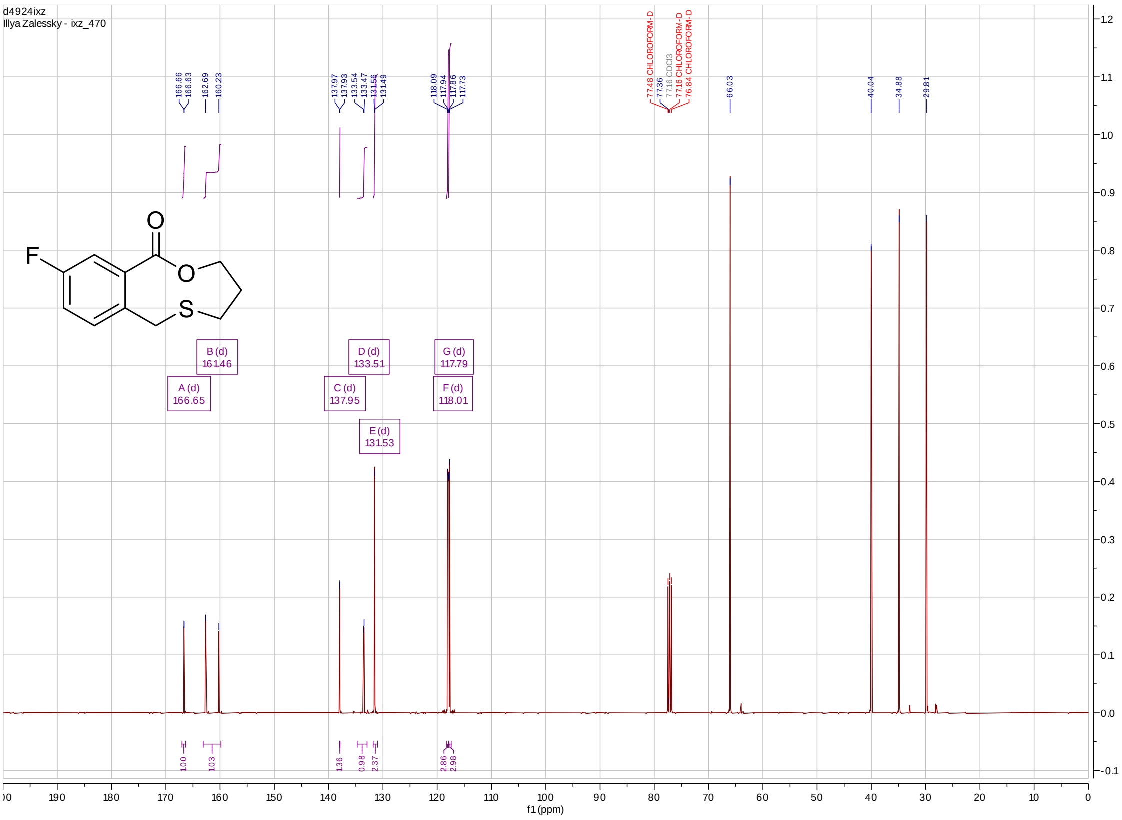

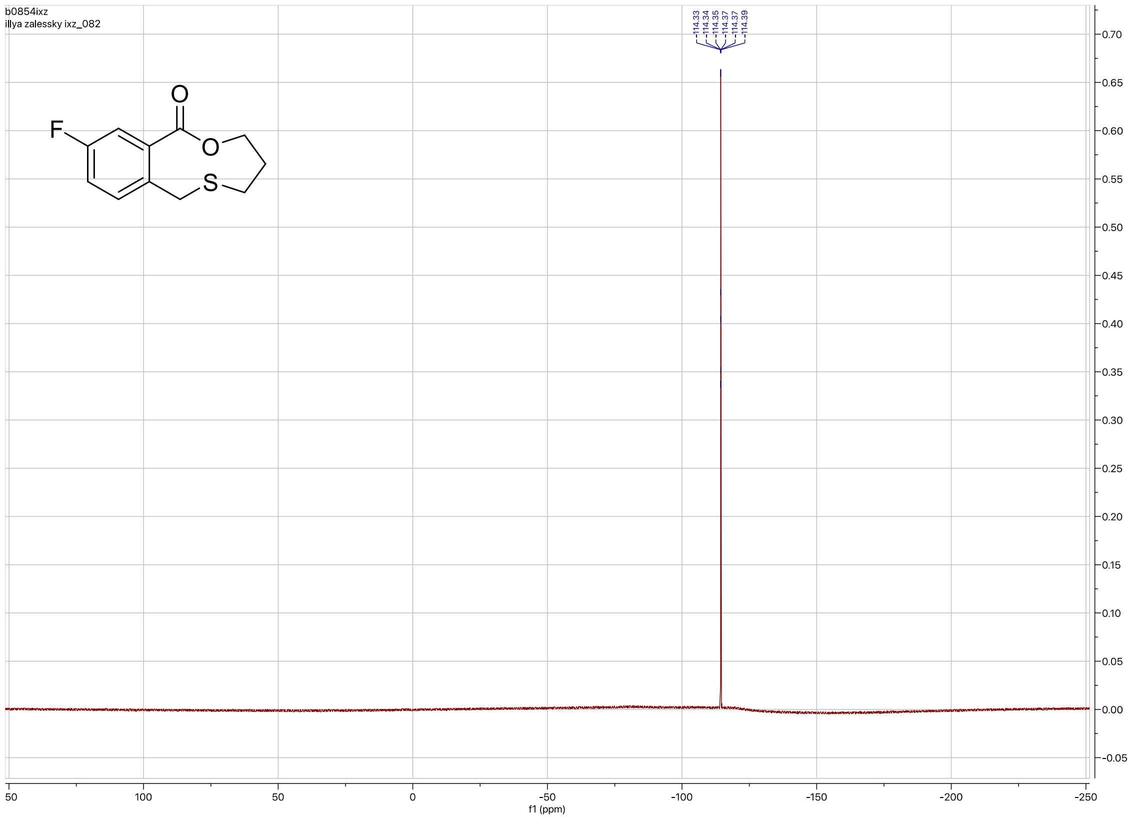
b0854ixz
illya zalessky ixz_082
-114.33
-114.34
-114.35
-114.37
-114.37
-114.39
F
O
O
S
f1 (ppm)

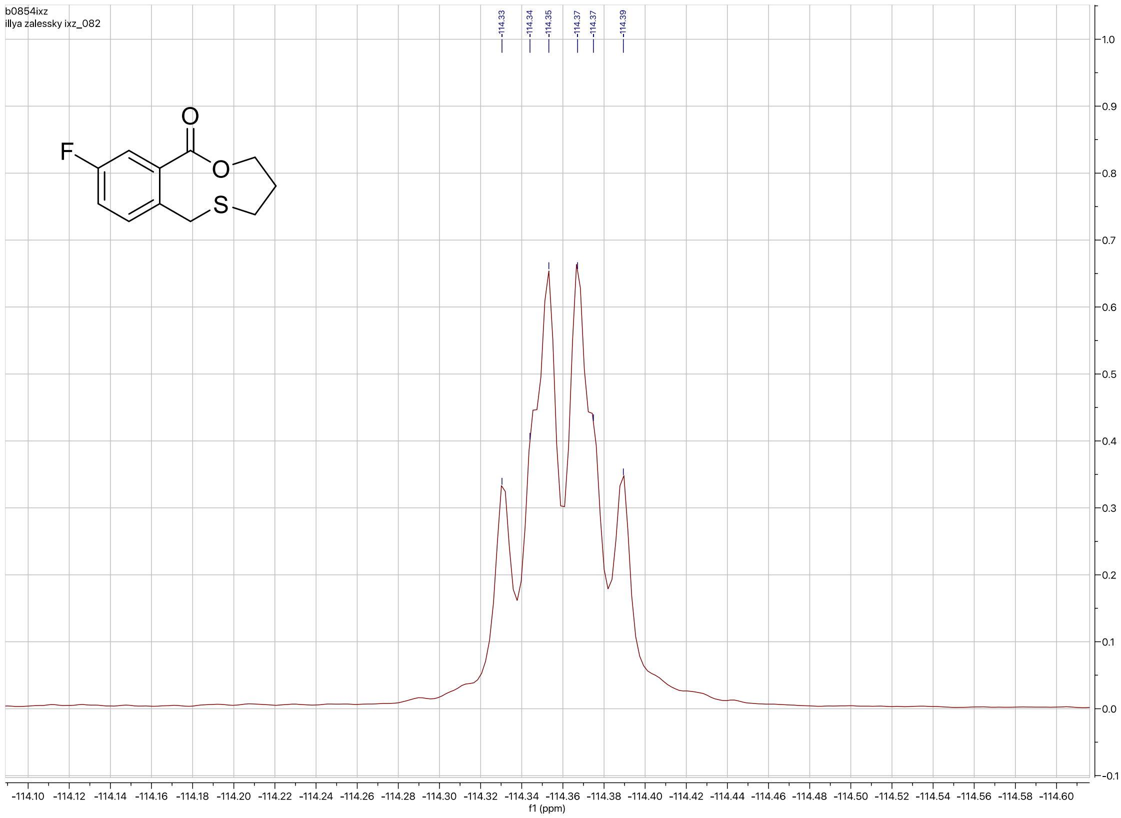
b0854ixz
illya zalessky ixz_082
-114.33
-114.34
-114.35
-114.37
-114.37
-114.39
F
O
O
S
f1 (ppm)

Methyl 2-bromo-6-(bromomethyl)benzoate (S31)

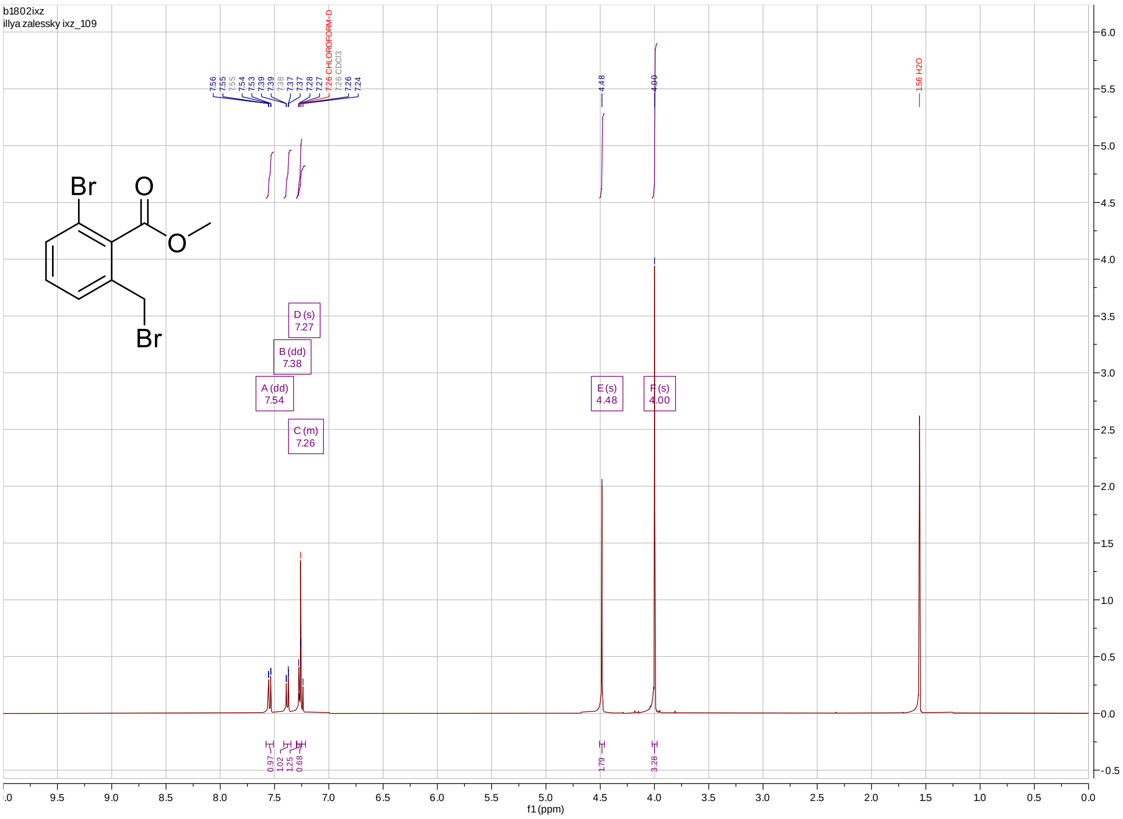

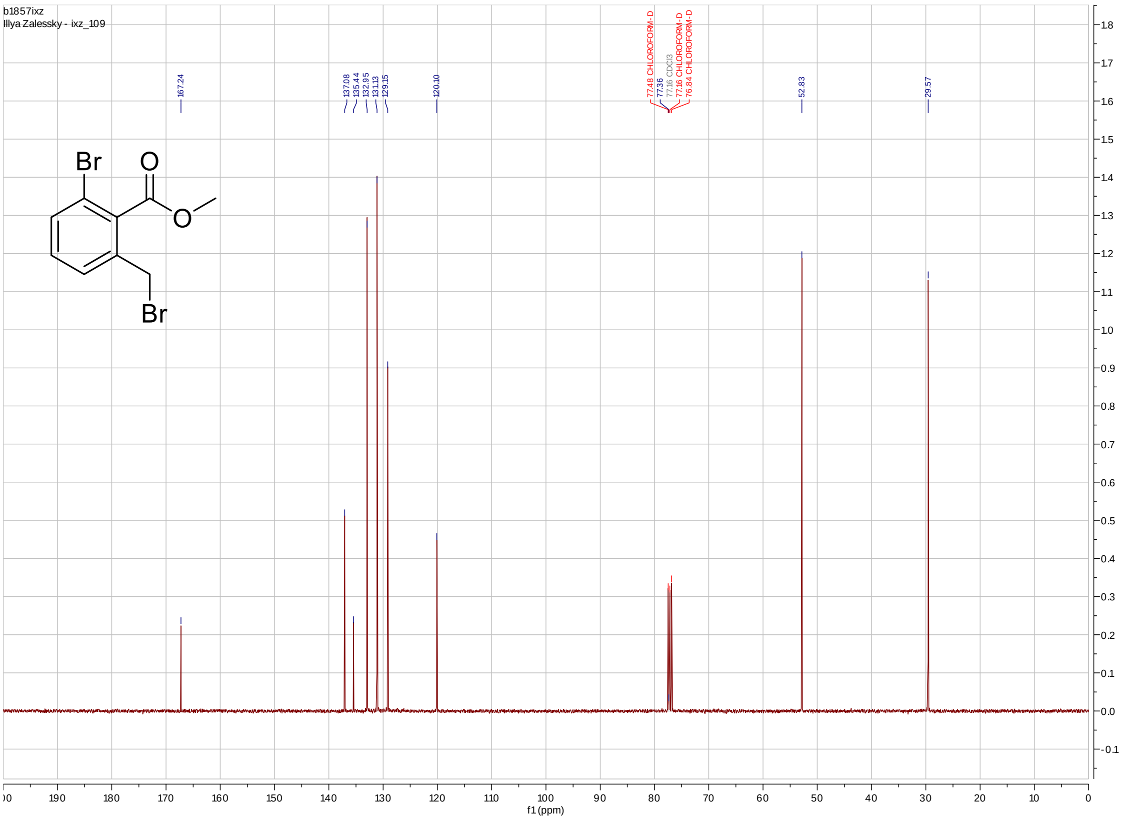

Methyl 2-bromo-6-(((3-hydroxypropyl)thio)methyl)benzoate (S32)

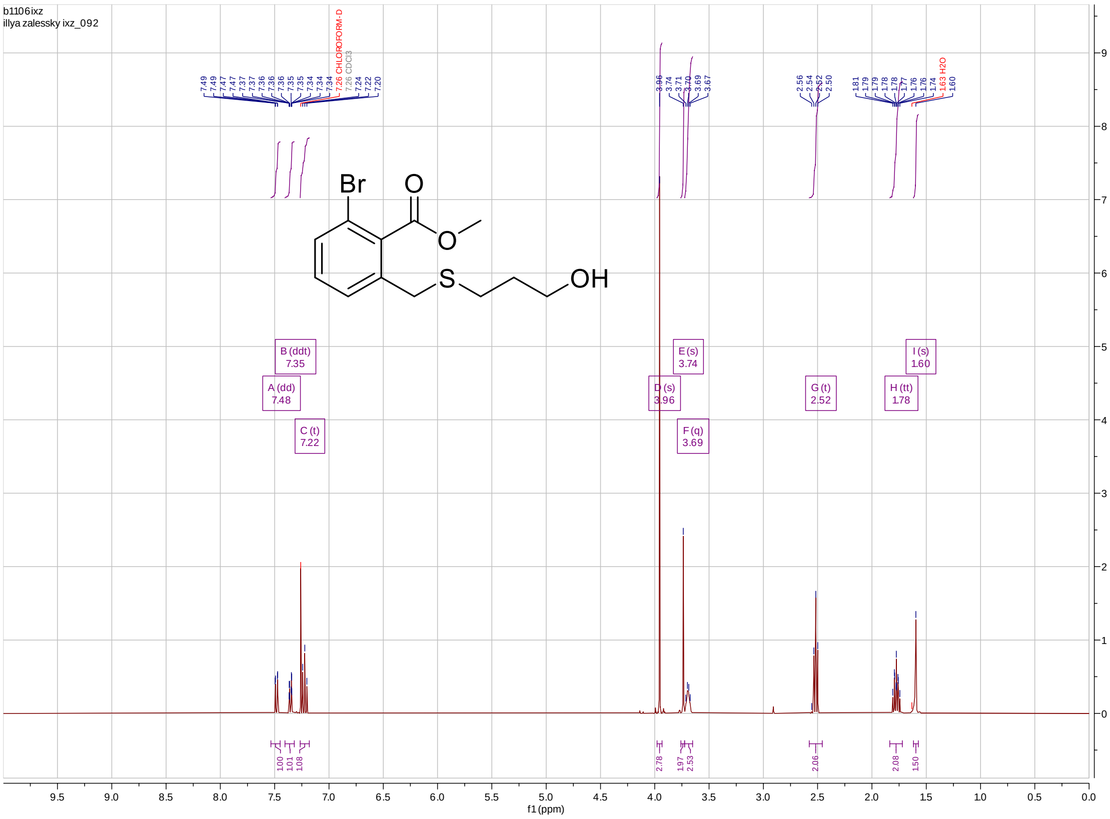

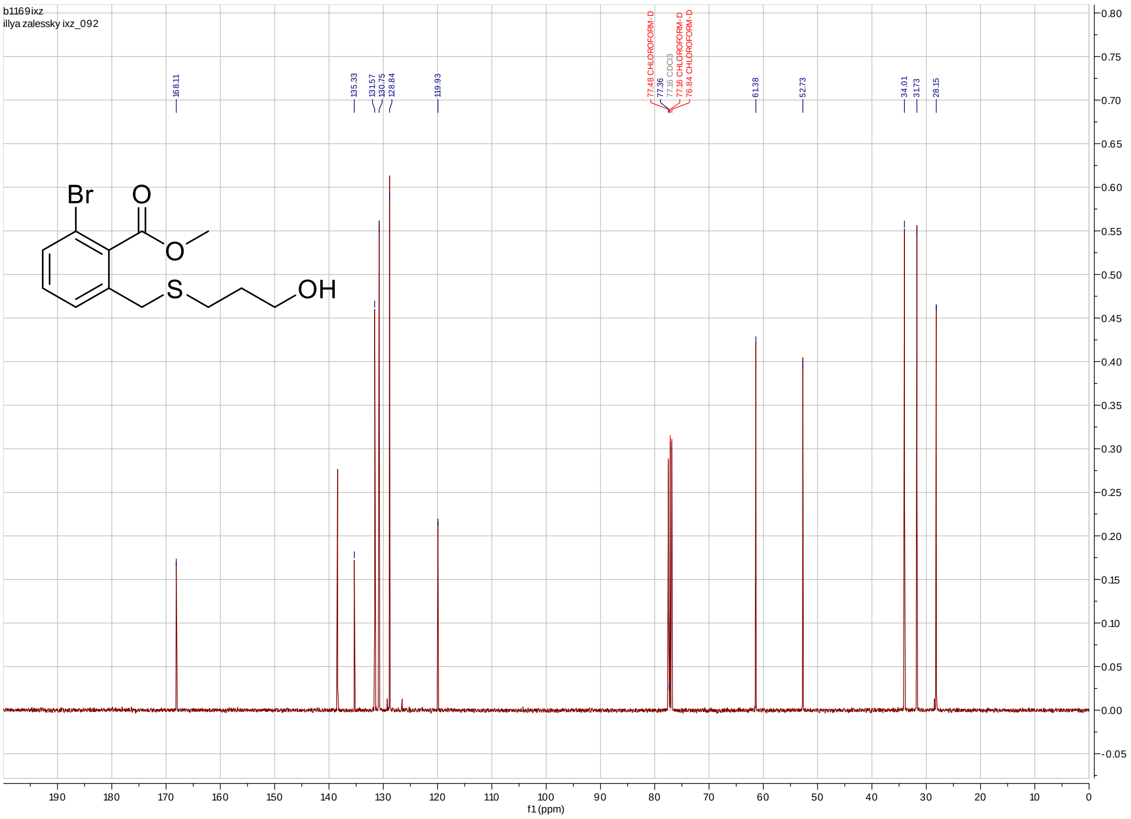

2-Bromo-6-(((3-hydroxypropyl)thio)methyl)benzoic acid (S33)

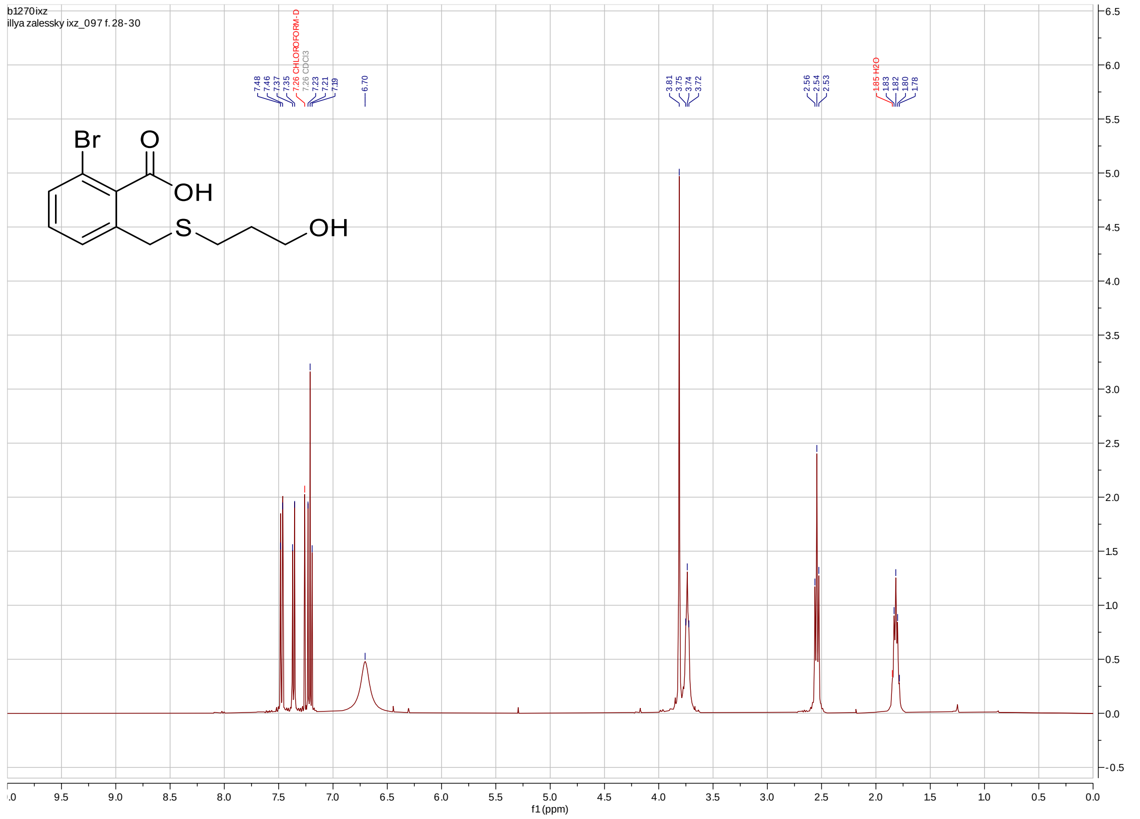

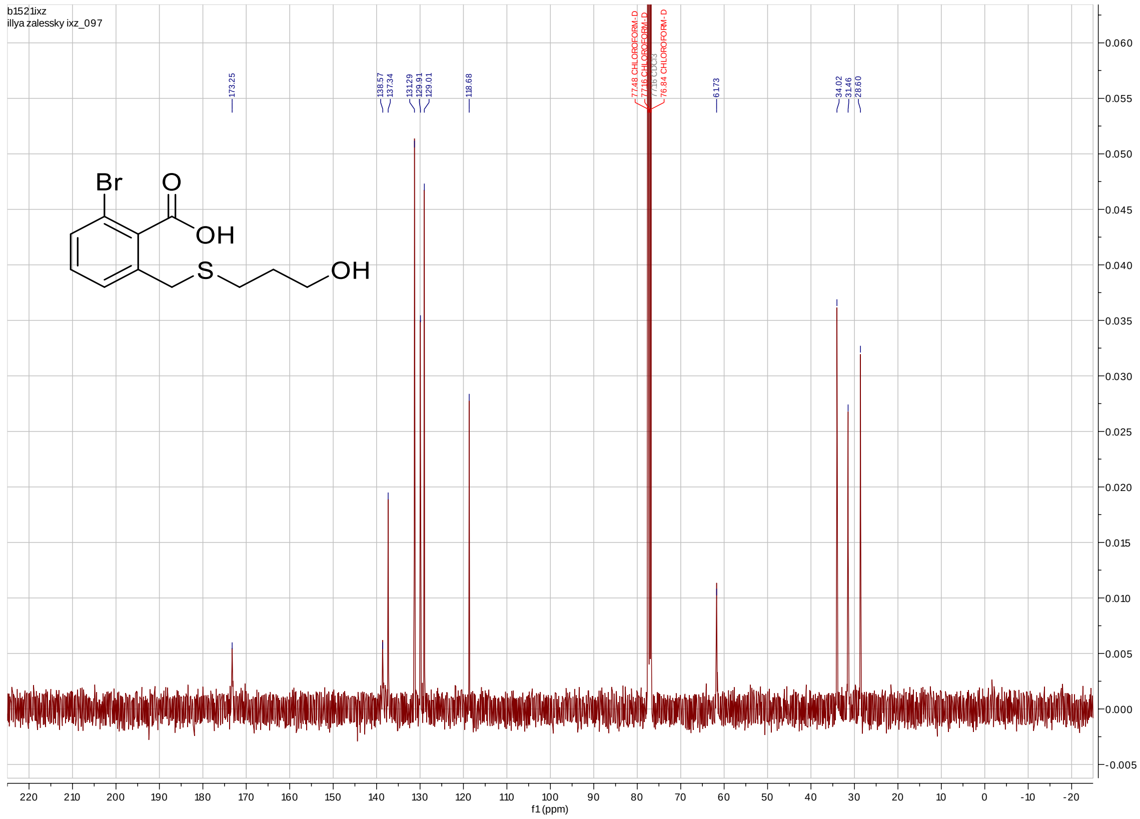

11-Bromo-4,5-dihydro-3*H*-benzo[*g*][1,5]oxathionin-1(7*H*)-one (49a)

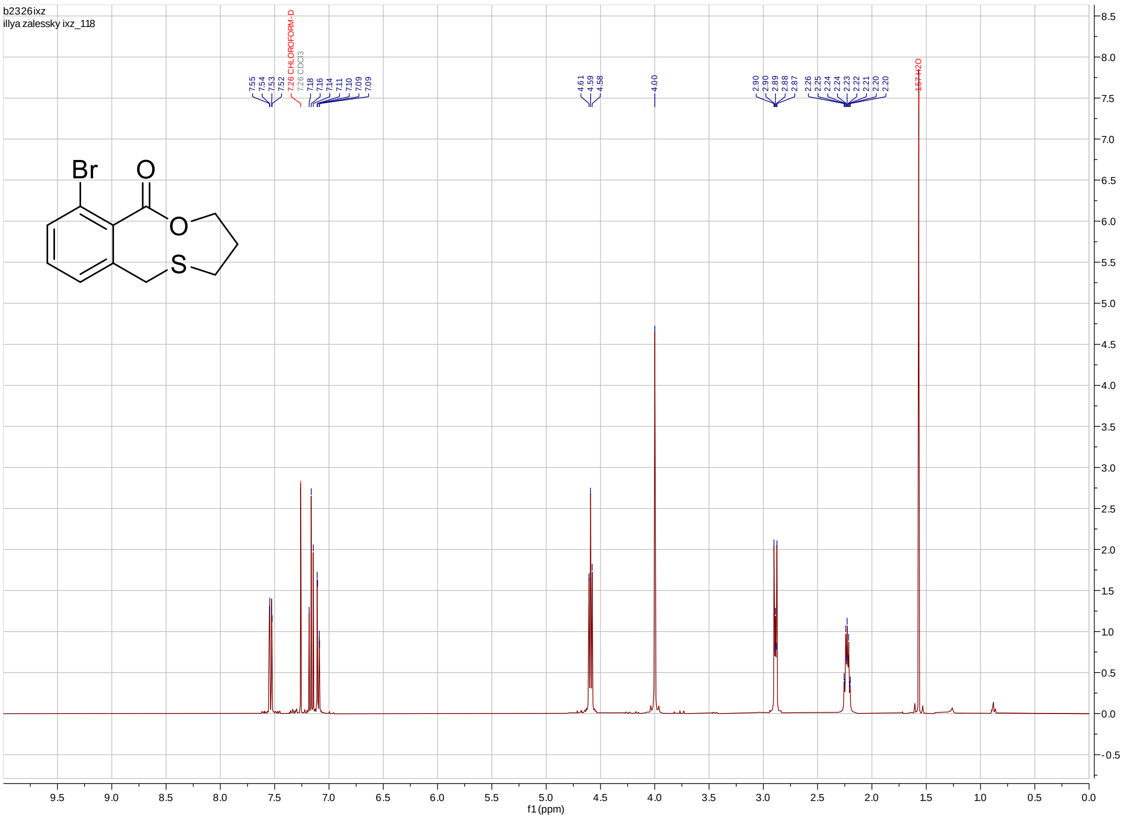

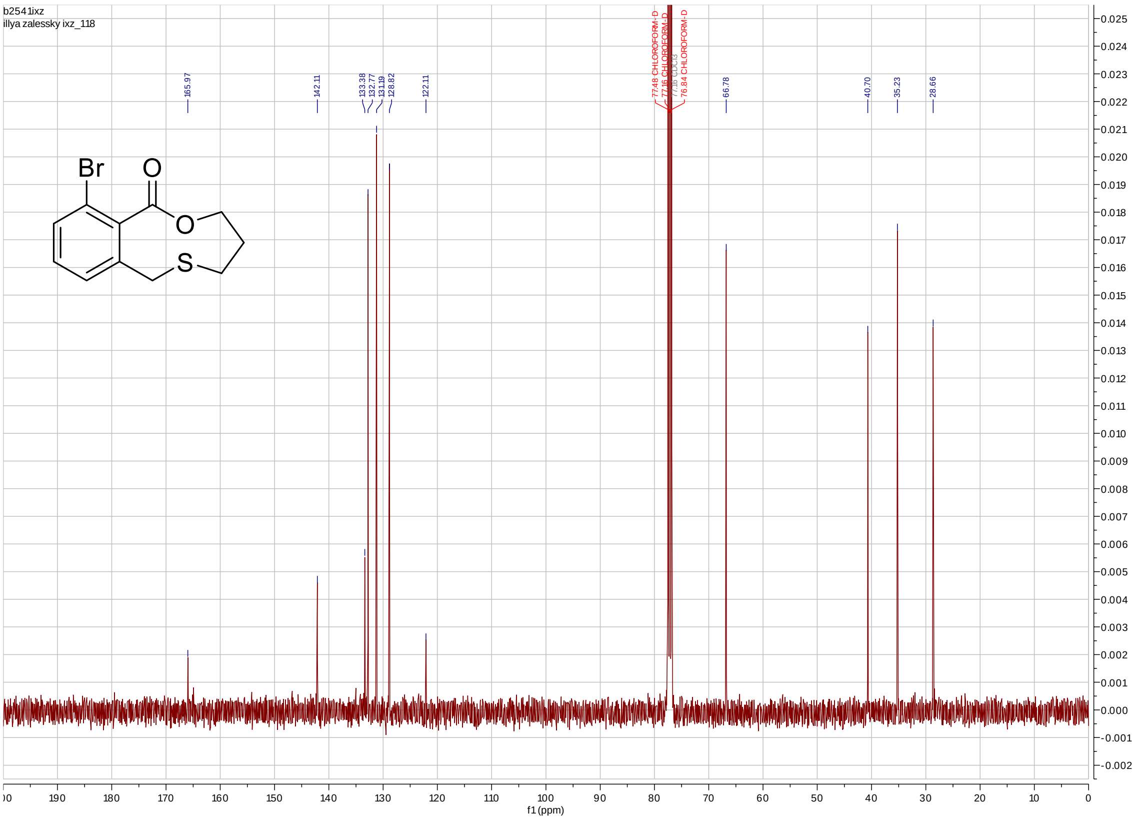

Methyl 4-bromo-2-(((3-hydroxypropyl)thio)methyl)benzoate (S34)

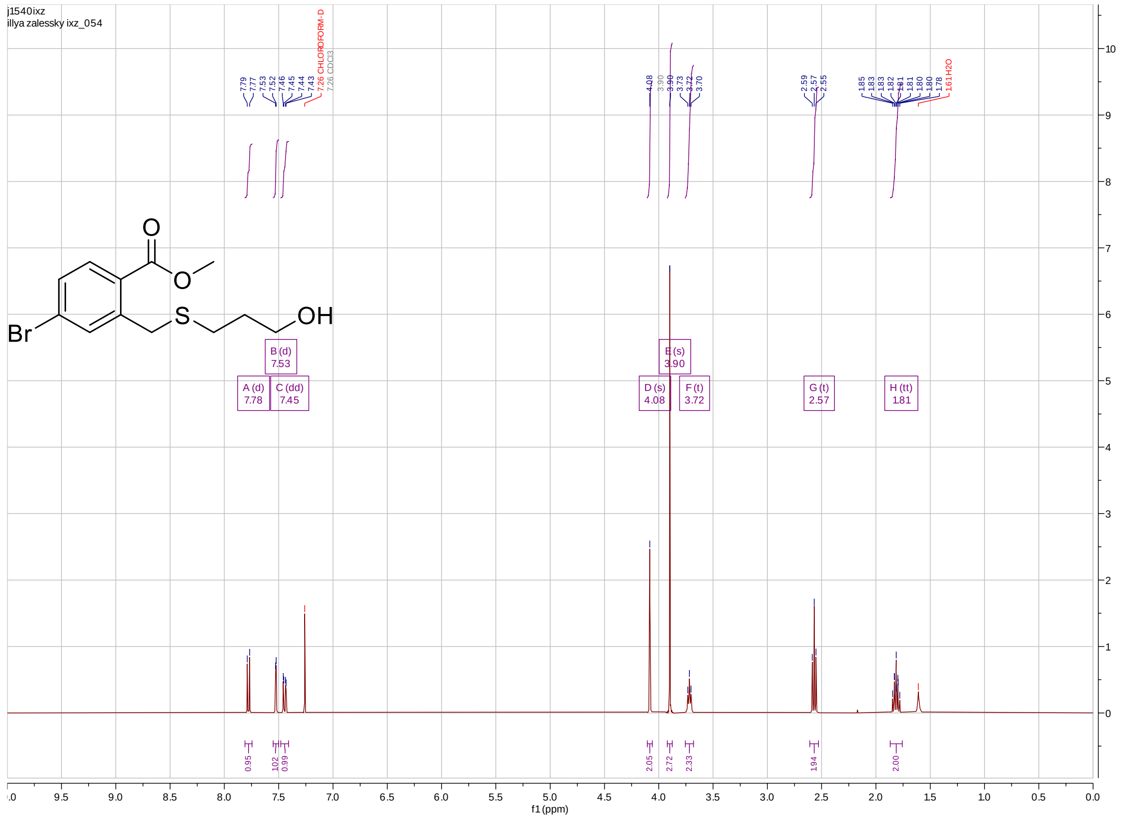

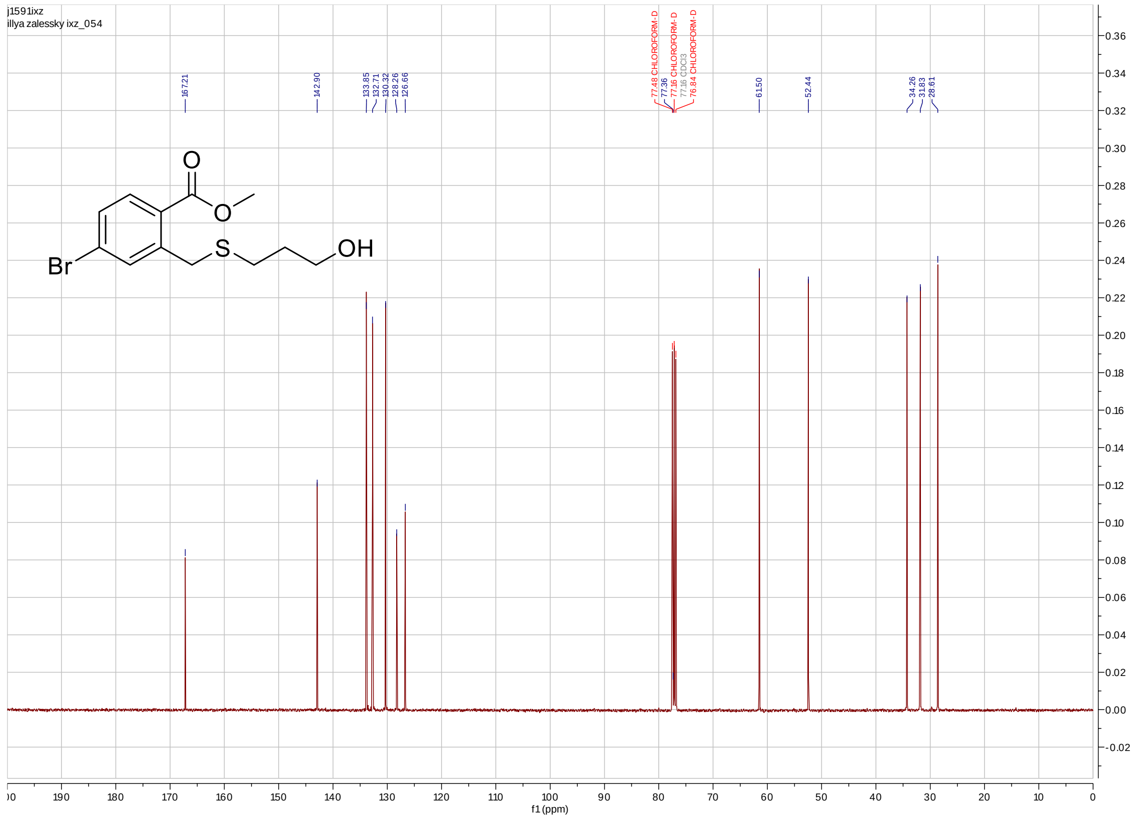

4-Bromo-2-(((3-hydroxypropyl)thio)methyl)benzoic acid (S35)

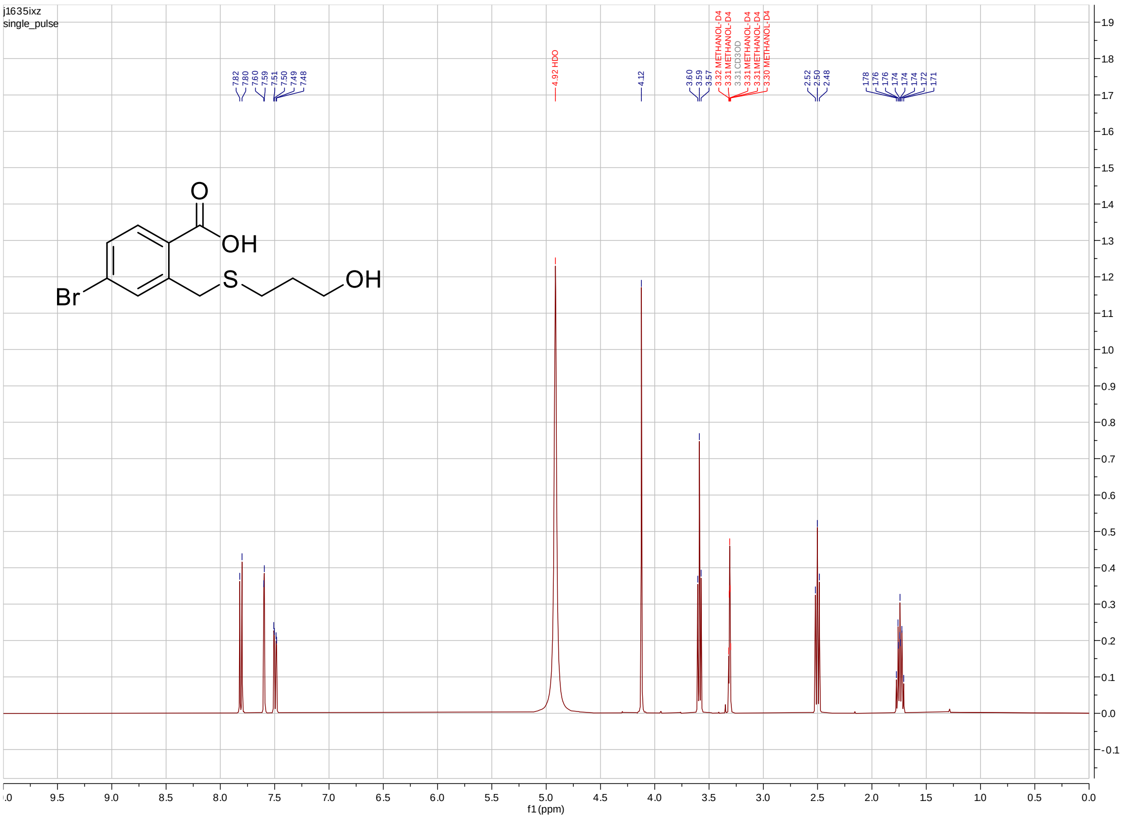

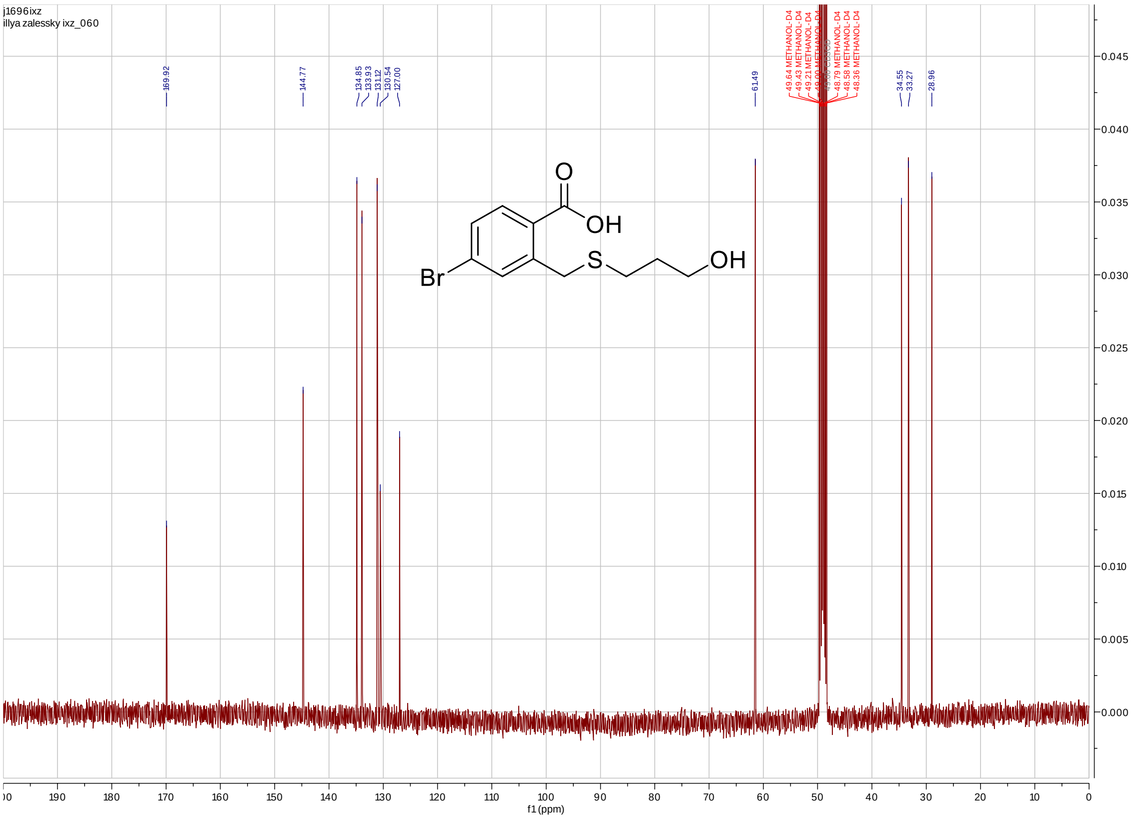

9-Bromo-4,5-dihydro-3*H*-benzo[*g*][1,5]oxathionin-1(7*H*)-one (49b)

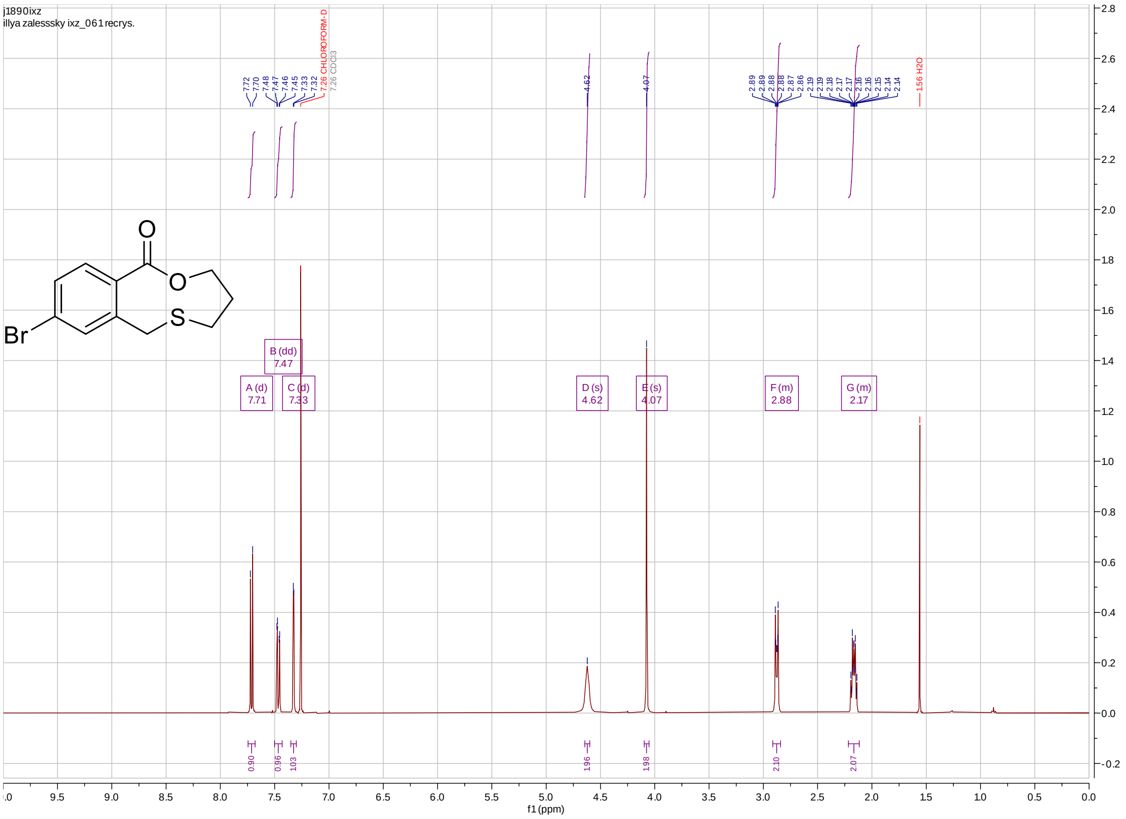

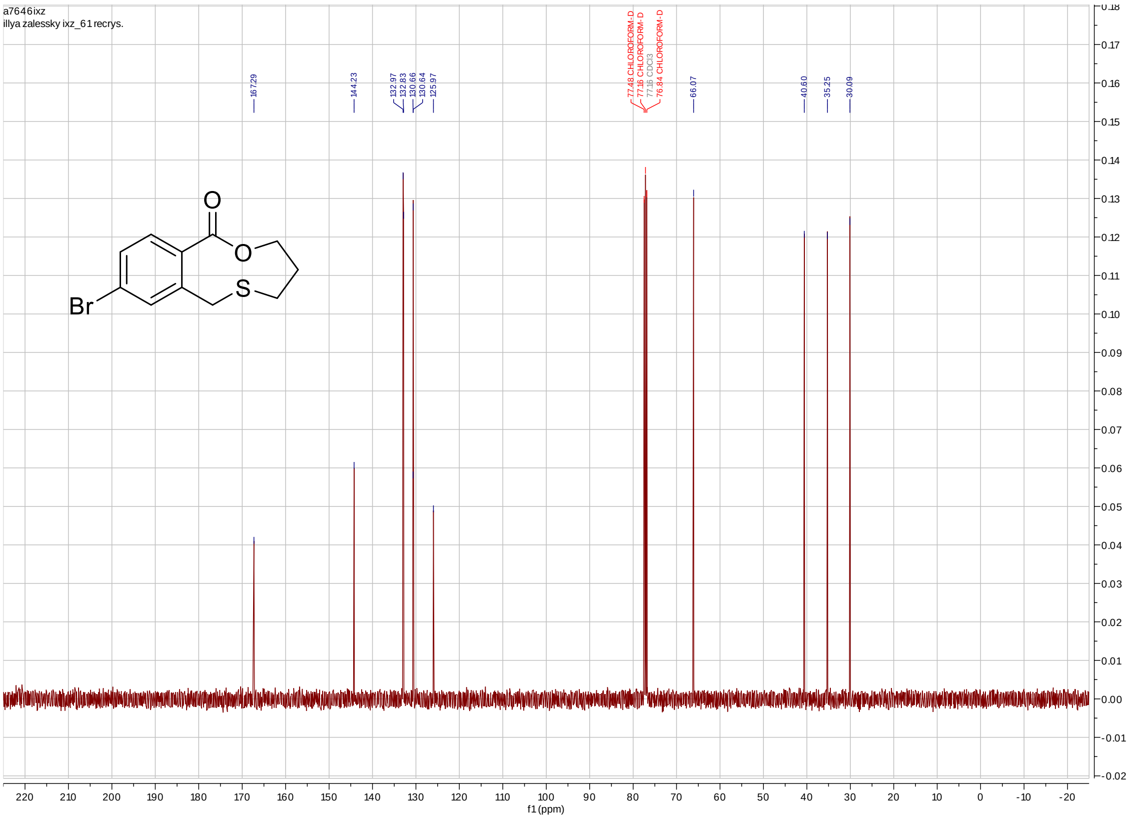

Methyl 3-bromo-2-(bromomethyl)benzoate (S36)

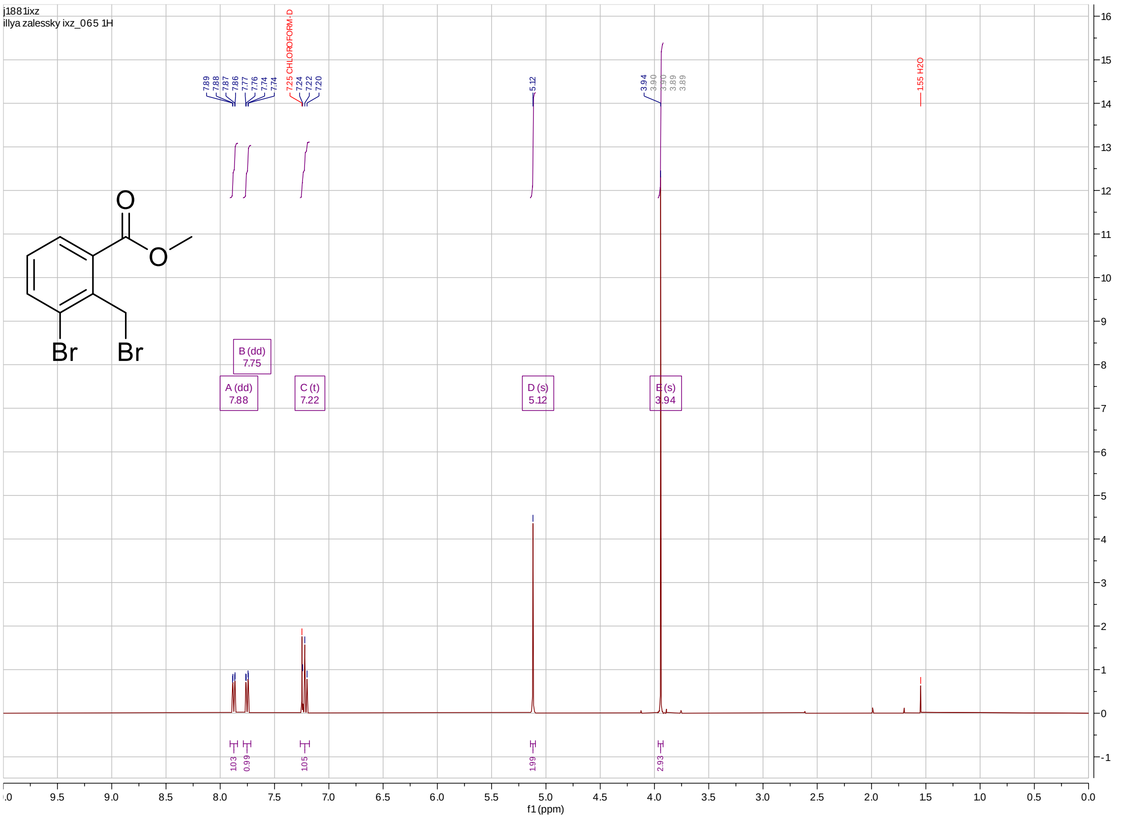

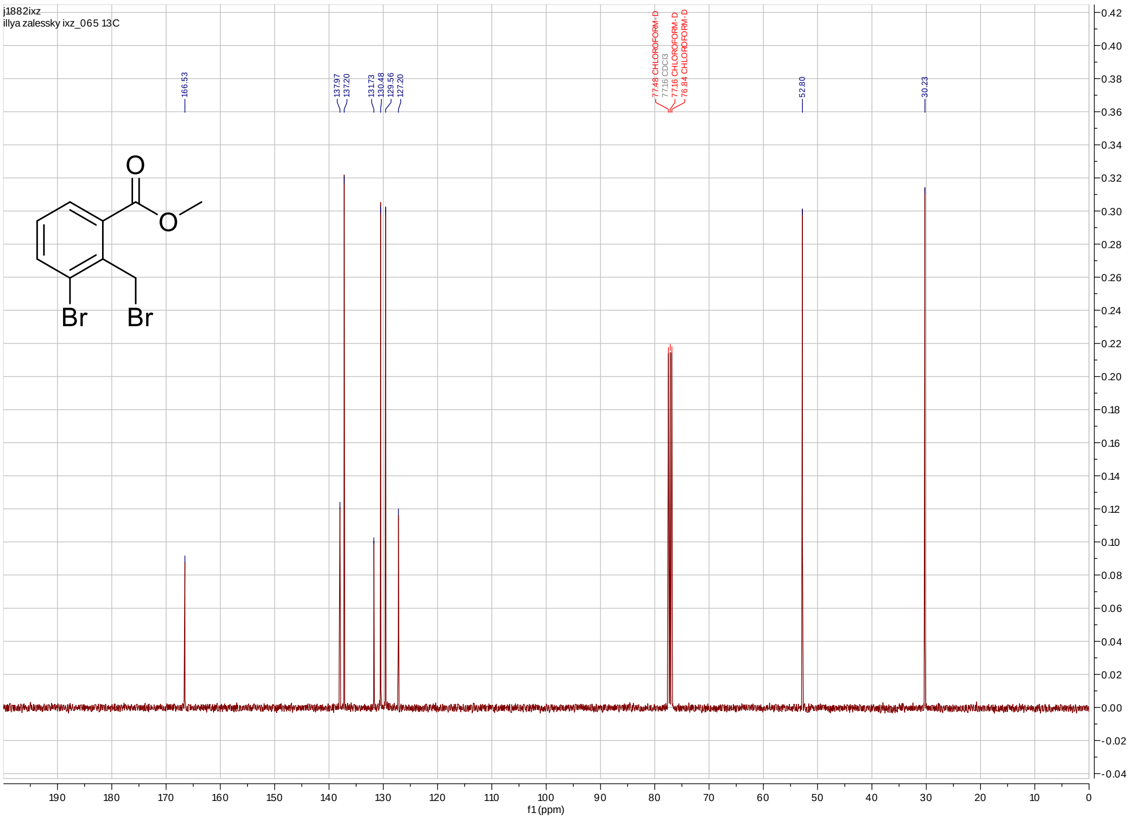

Methyl 3-bromo-2-(((3-hydroxypropyl)thio)methyl)benzoate (S37)

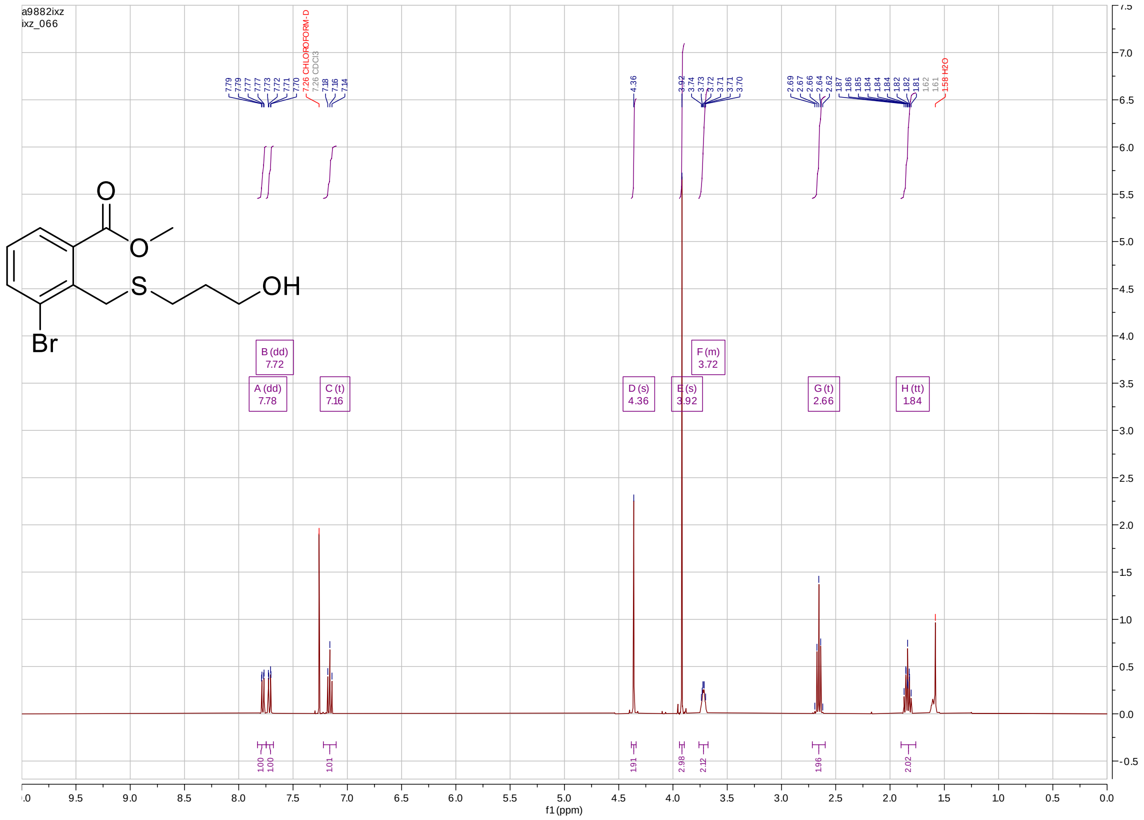

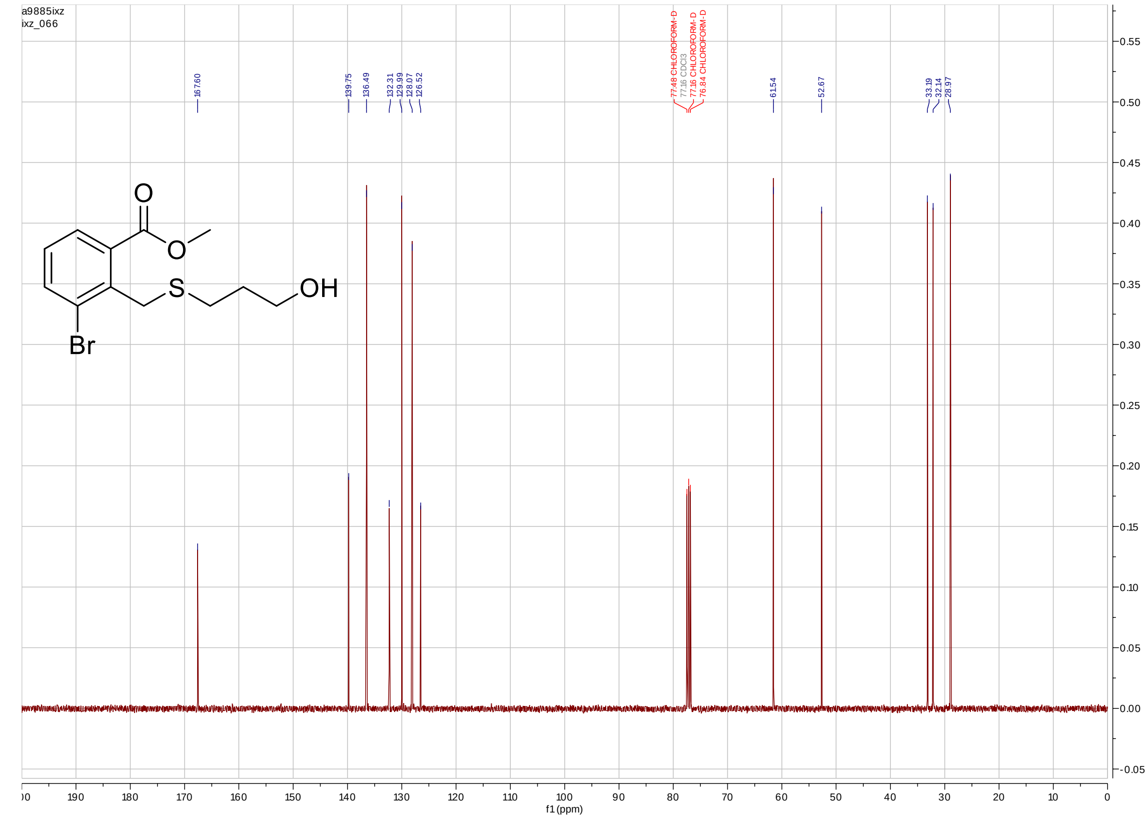

3-Bromo-2-(((3-hydroxypropyl)thio)methyl)benzoic acid (S38)

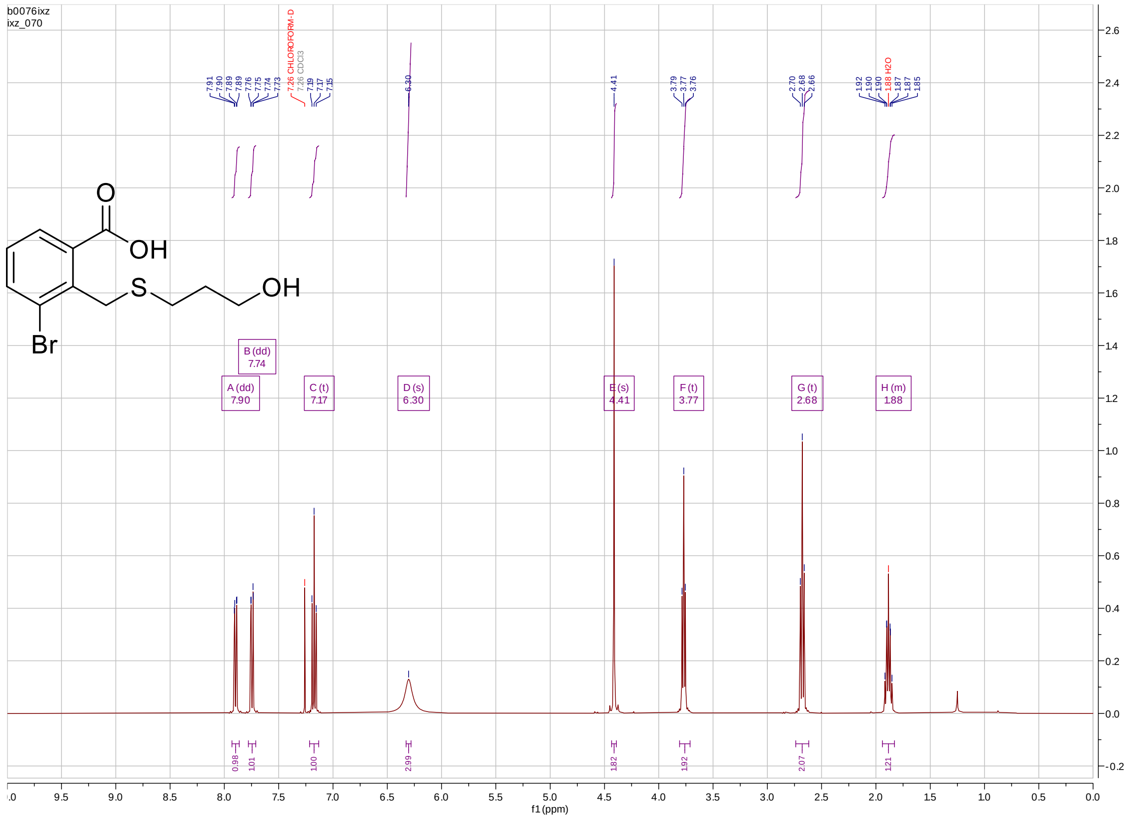

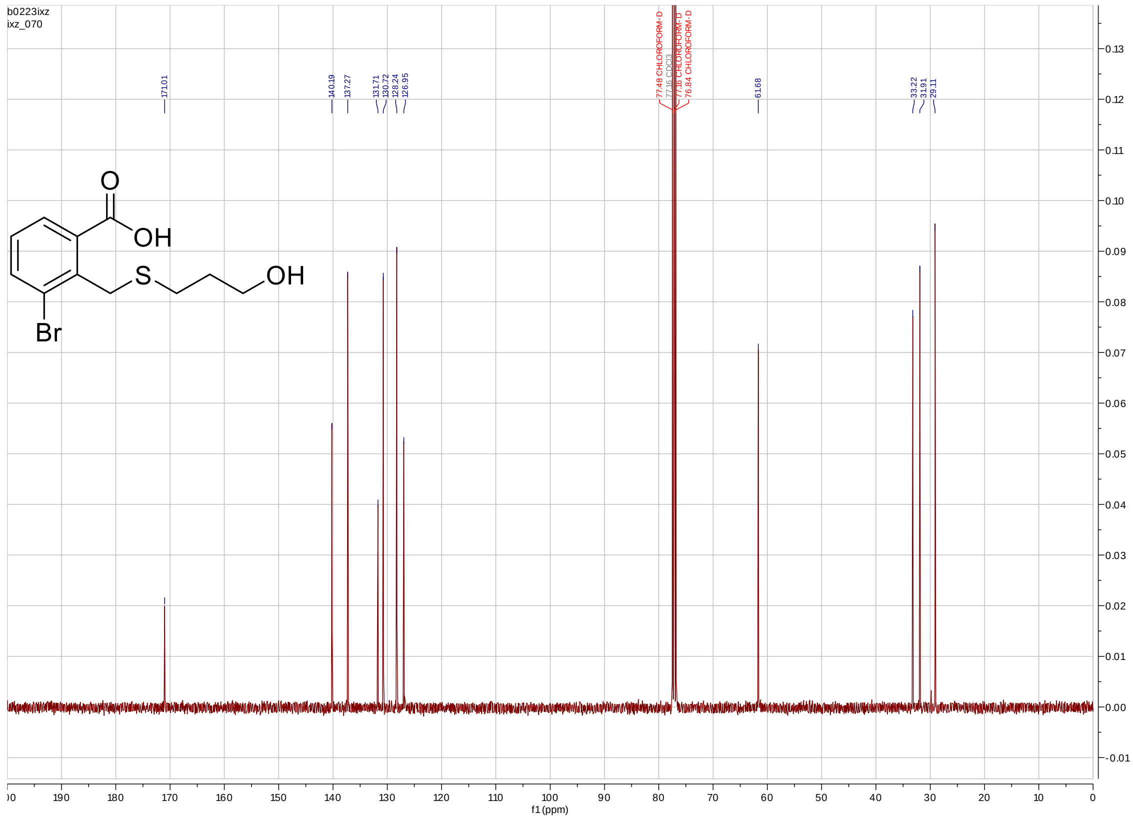

8-Bromo-4,5-dihydro-3*H*-benzo[*g*][*1,5*]oxathionin-1(7*H*)-one (49c)

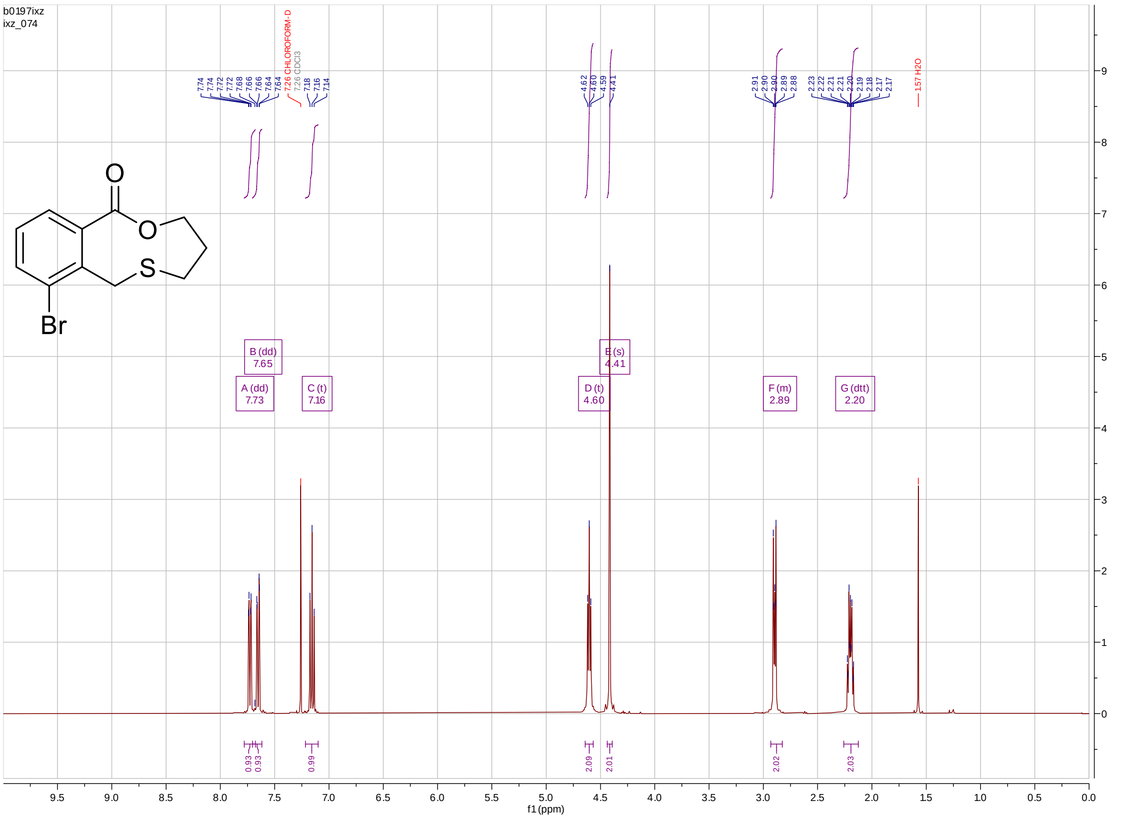

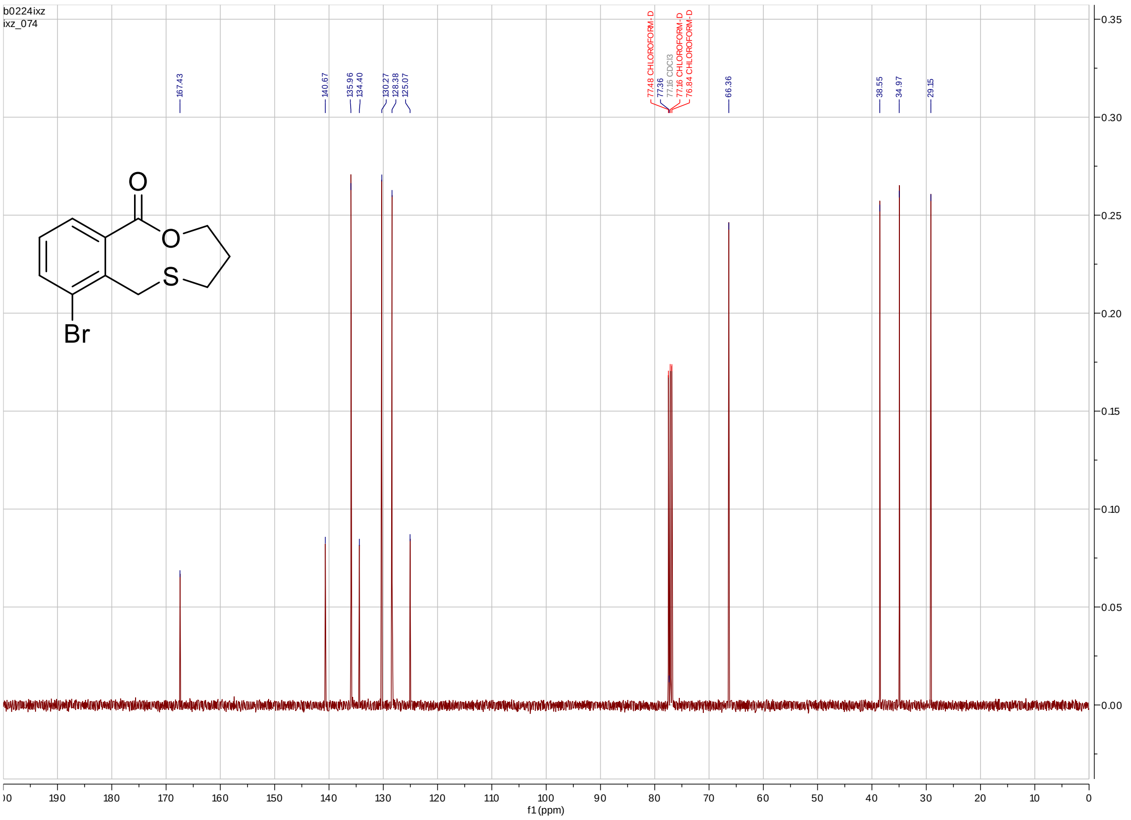

Methyl 2-(((3-hydroxypropyl)thio)methyl)-3-nitrobenzoate (S39)

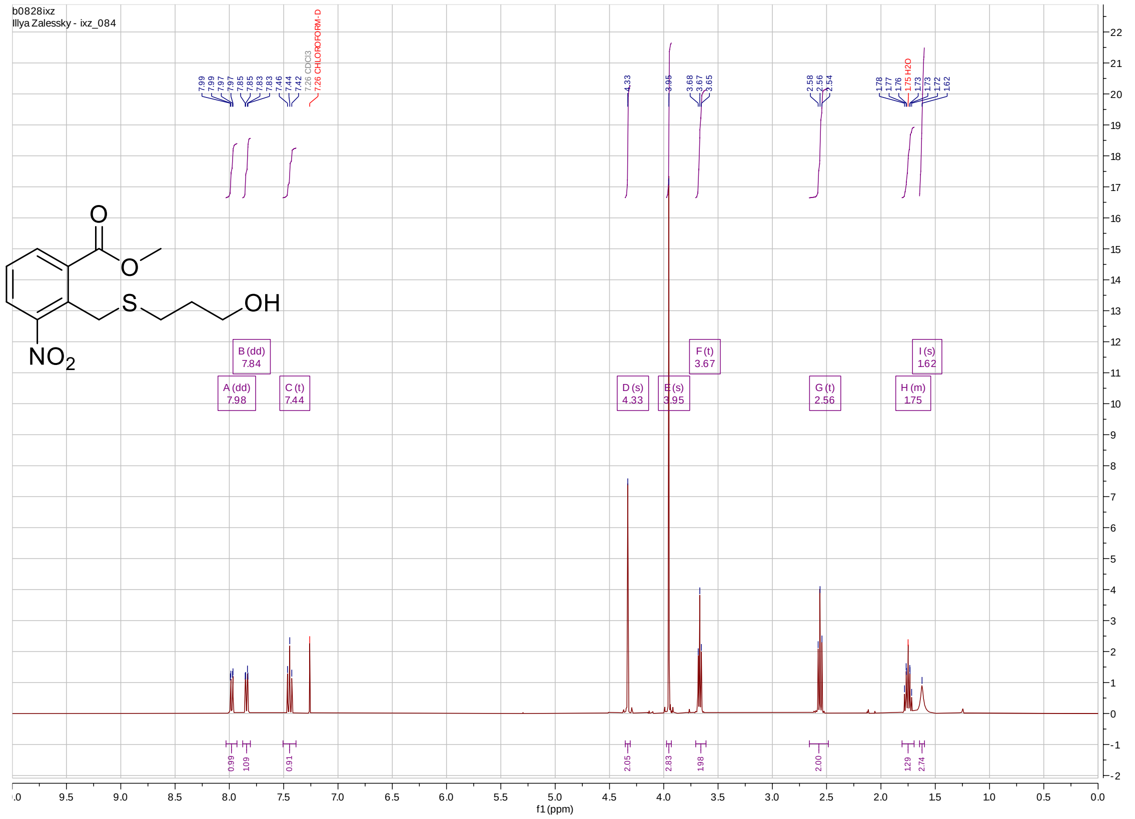

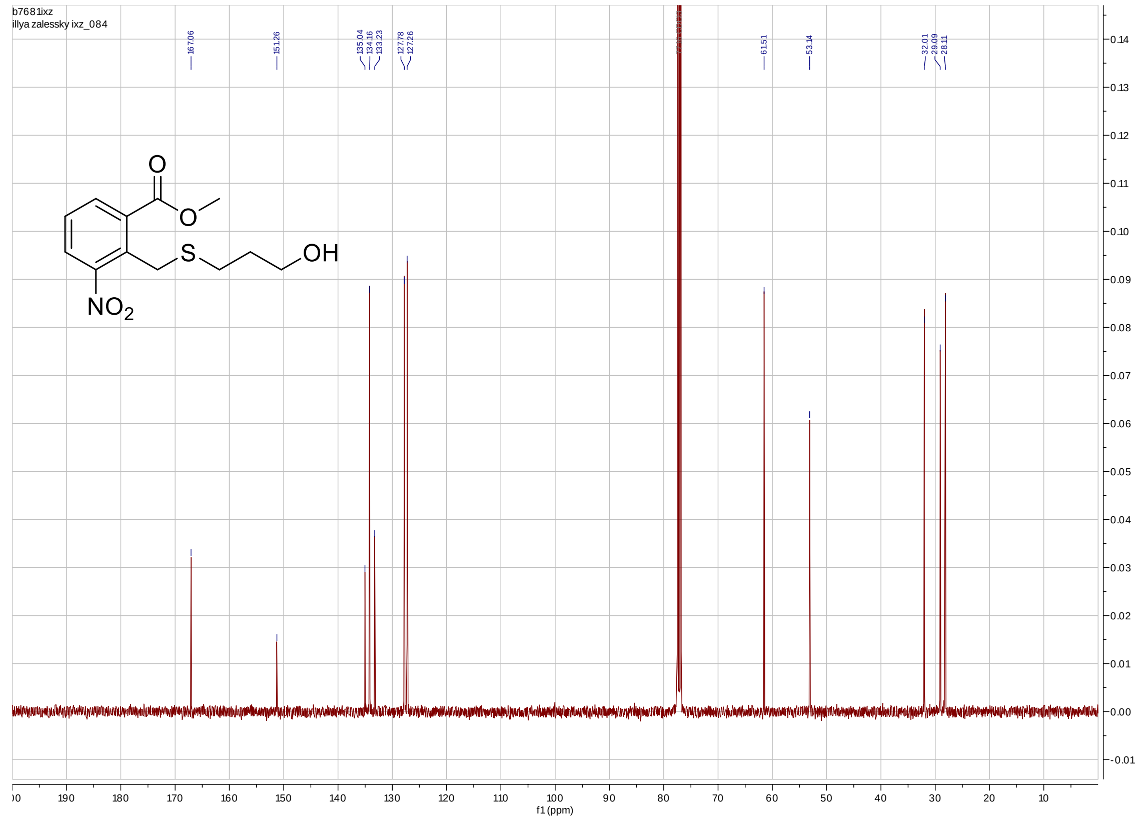

2-(((3-Hydroxypropyl)thio)methyl)-3-nitrobenzoic acid (S40)

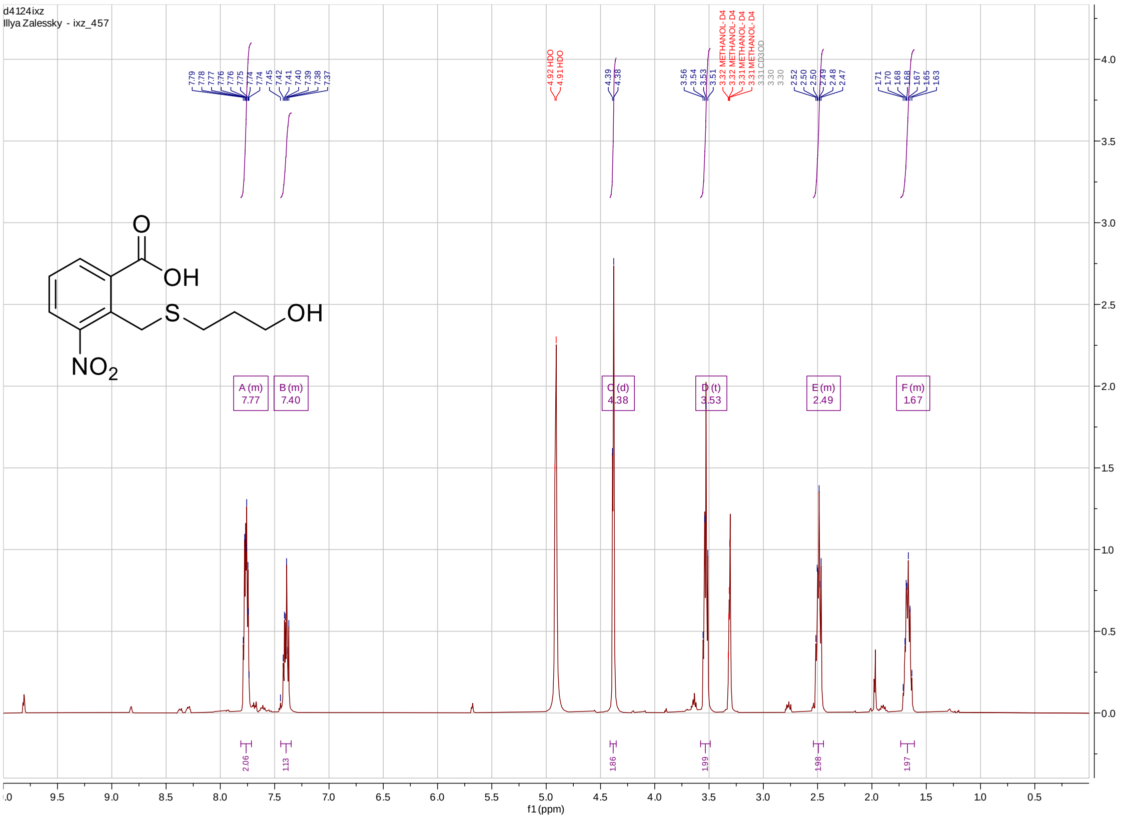

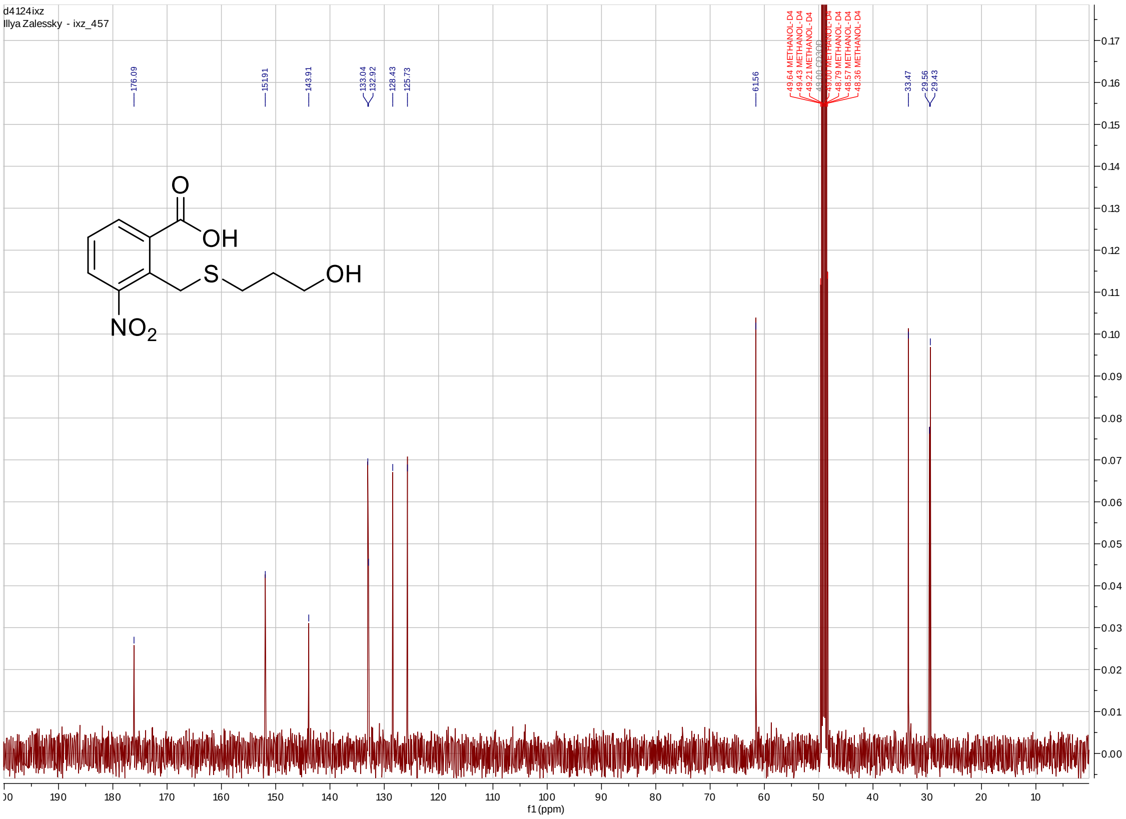

8-Nitro-4,5-dihydro-3H-benzo[g][1,5]oxathionin-1(7H)-one (50)

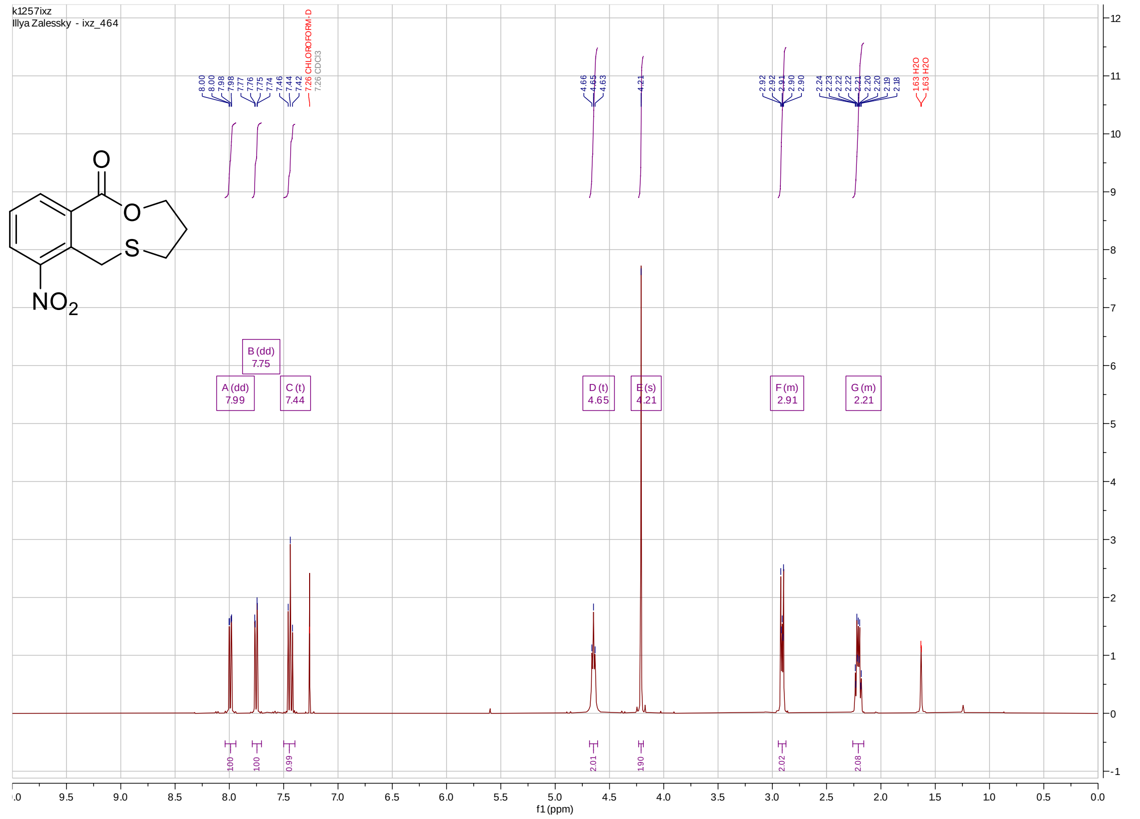

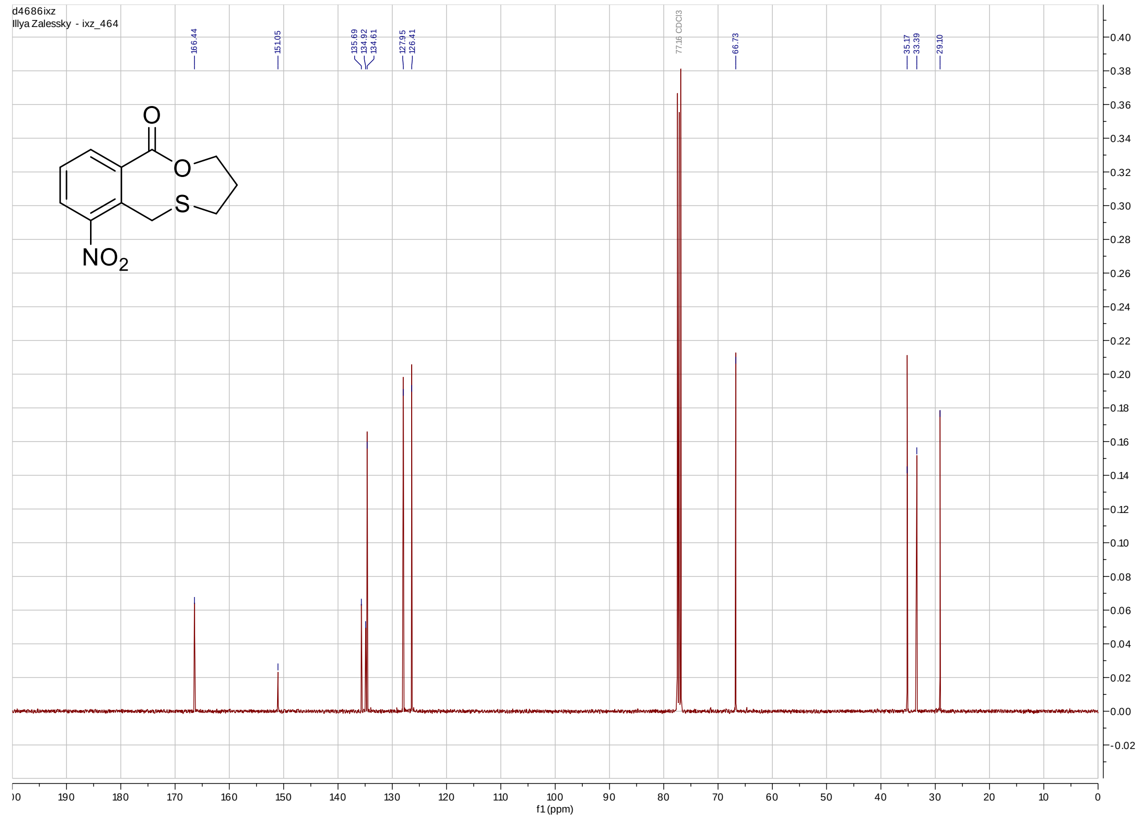

Methyl 2-(((3-oxopropyl)thio)methyl)benzoate (S41)

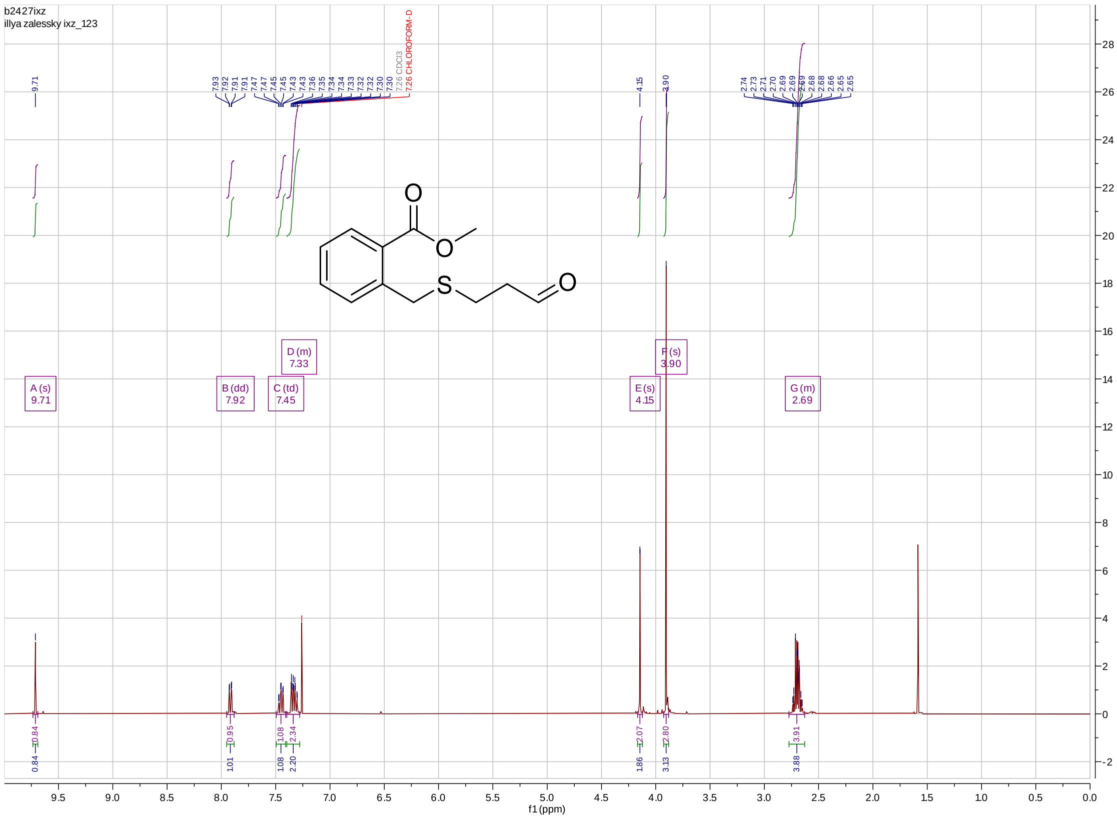

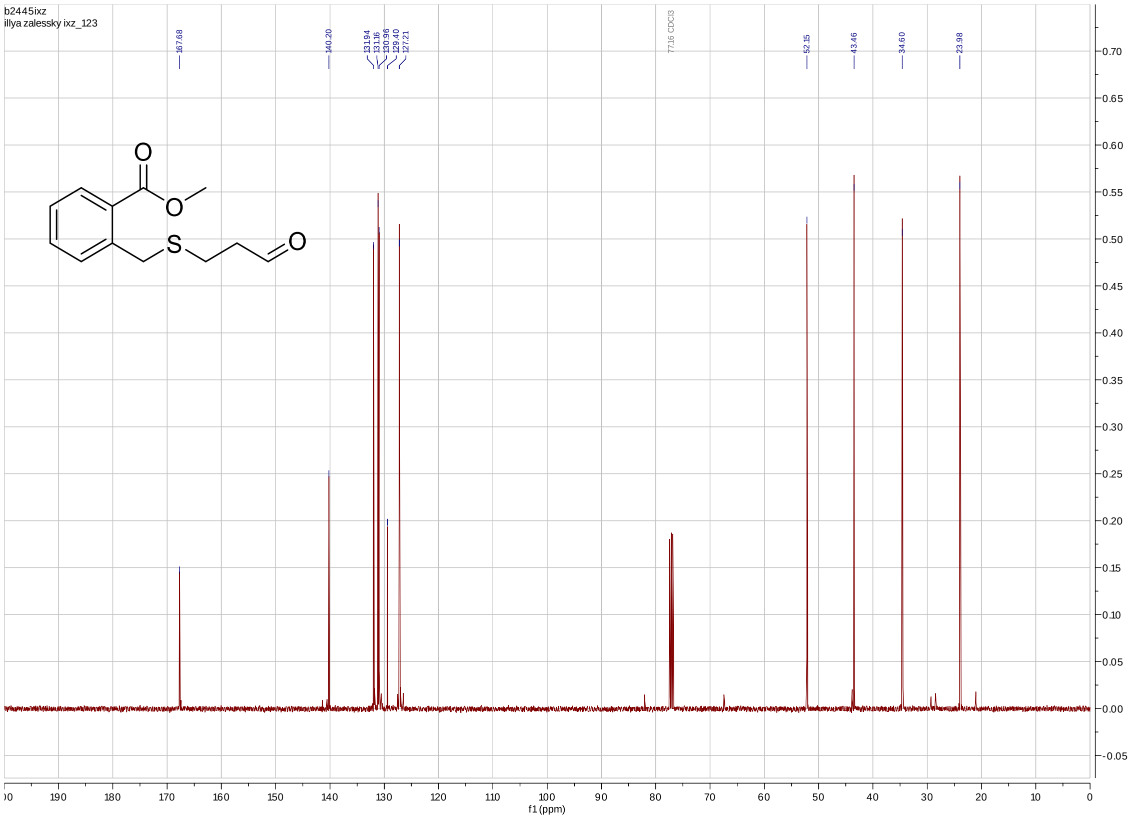

Methyl 2-(((3-hydroxybutyl)thio)methyl)benoate (S42)

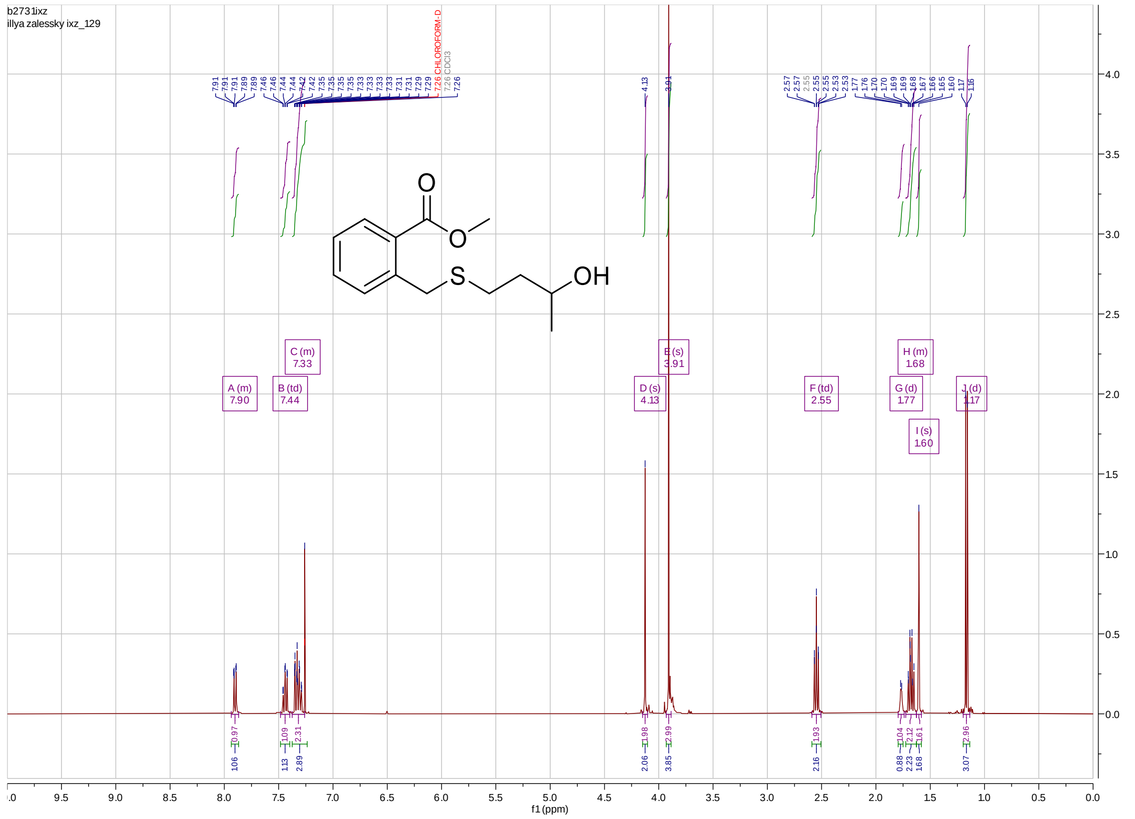

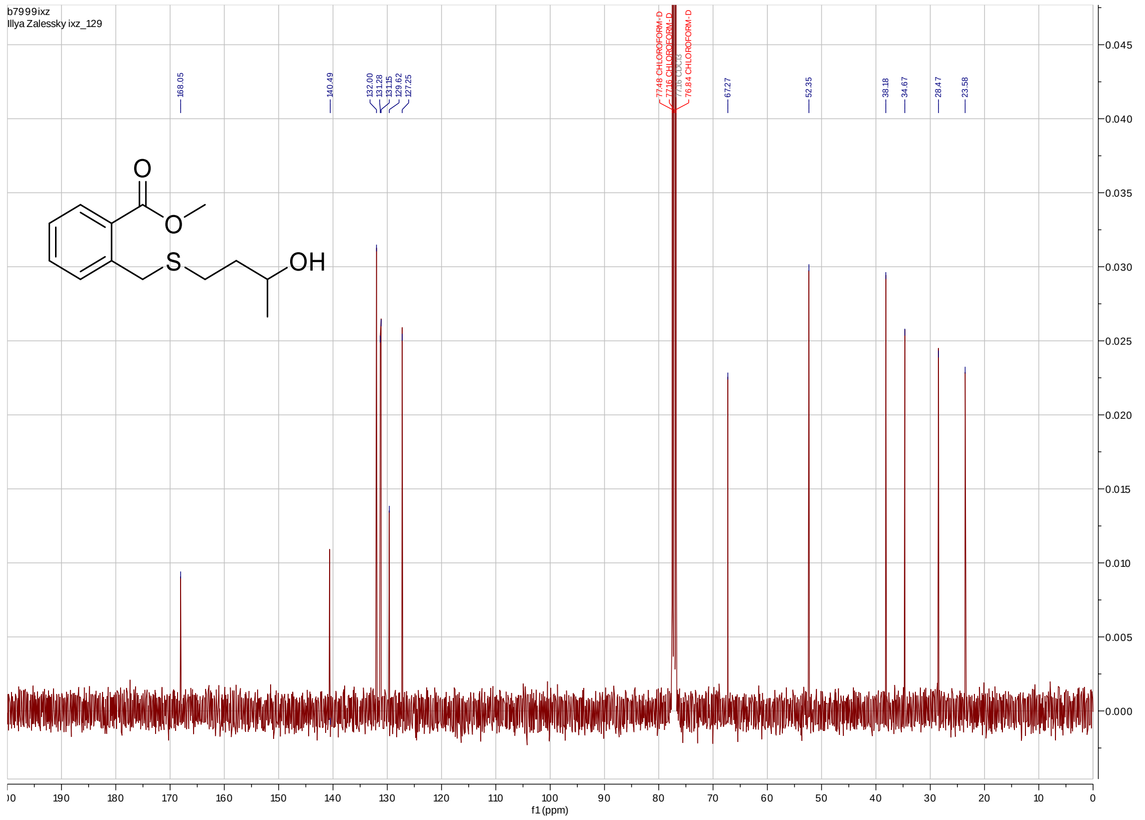

2-(((3-Hydroxybutyl)thio)methyl)benzoic acid (S43)

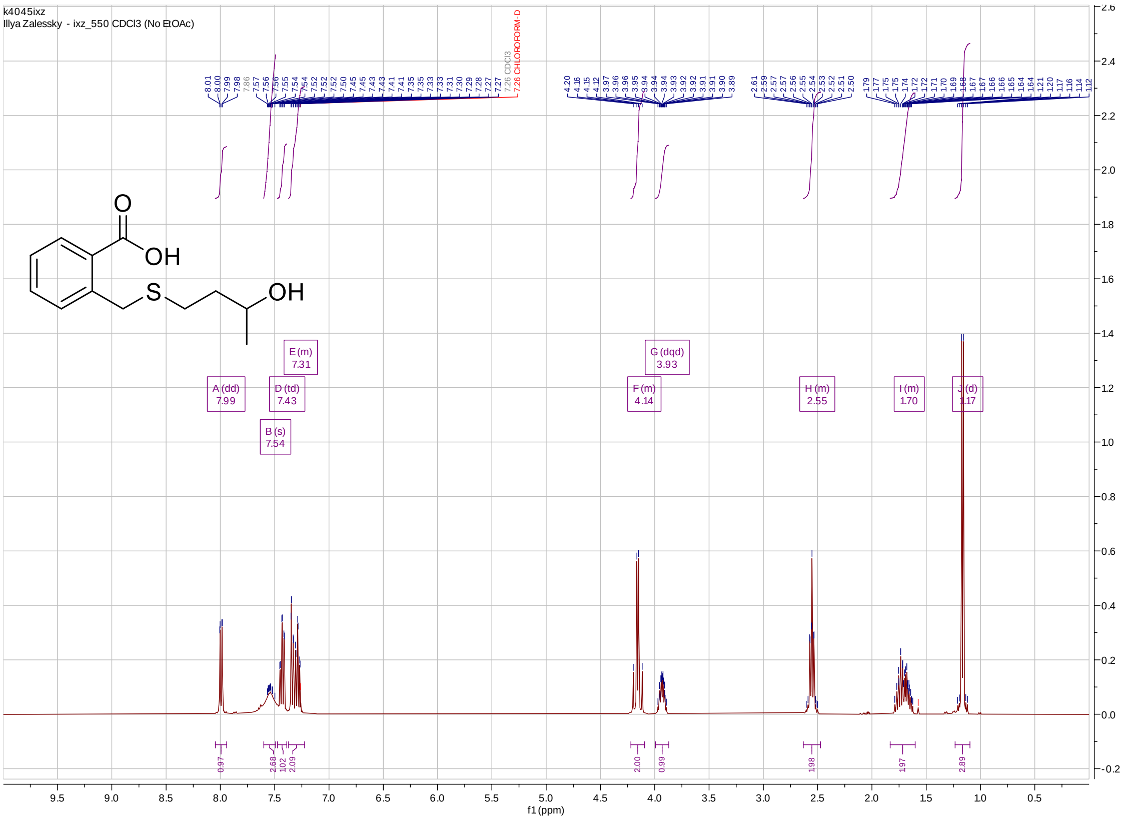

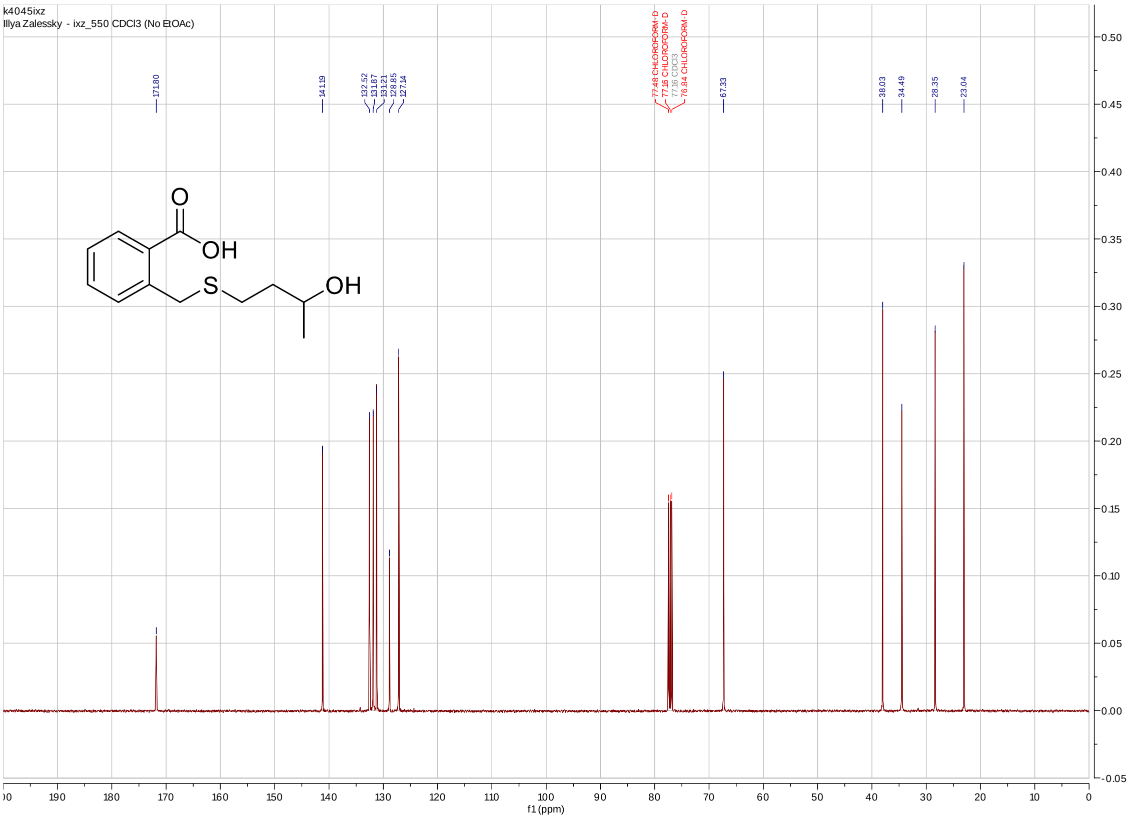

3-Methyl-4,5-dihydro-3*H*-benzo[*g*][1,5]oxathionin-1(7*H*)-one (51)

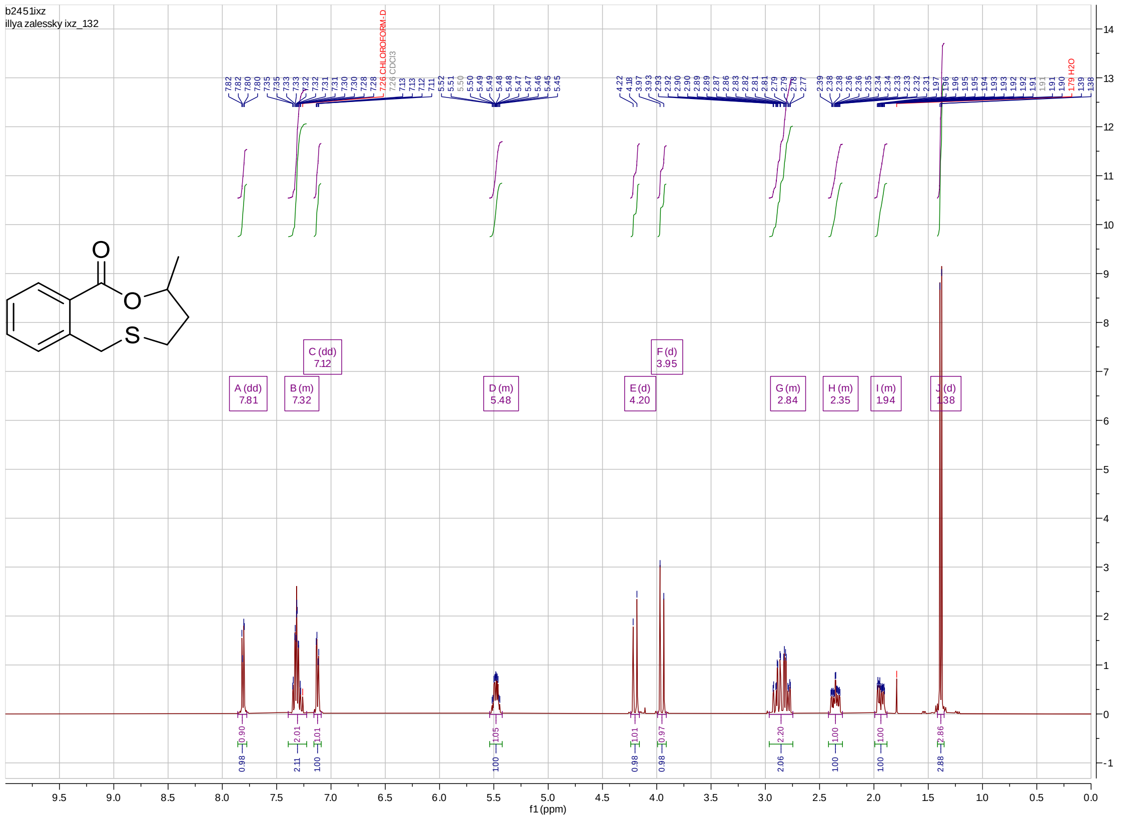

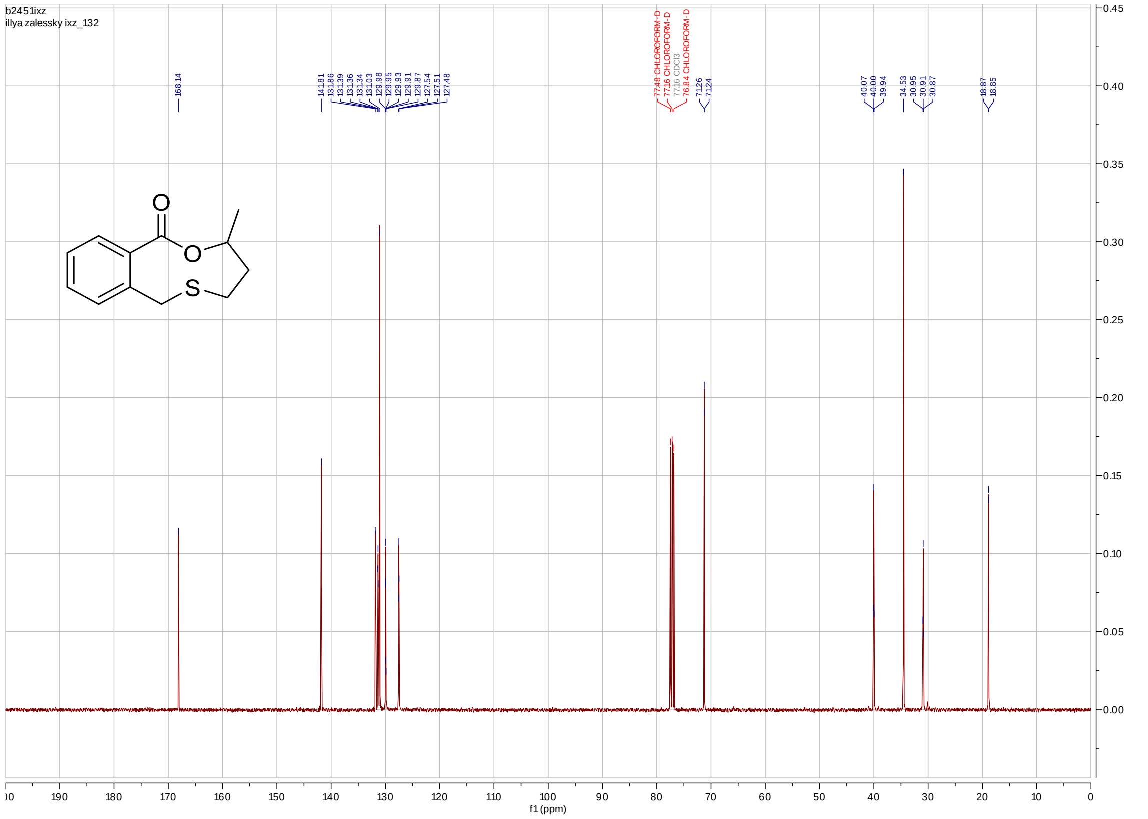

Methyl 2-(((3-hydroxypropyl)selanyl)methyl)benzoate (S45)

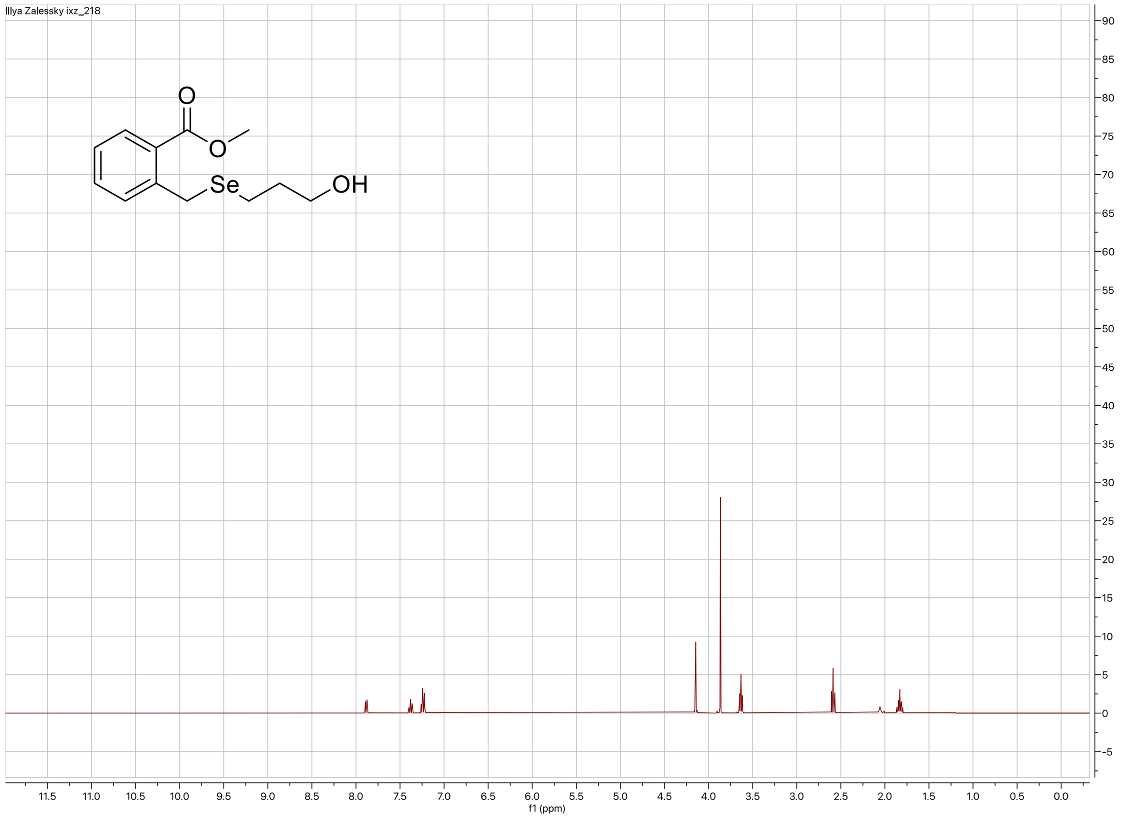

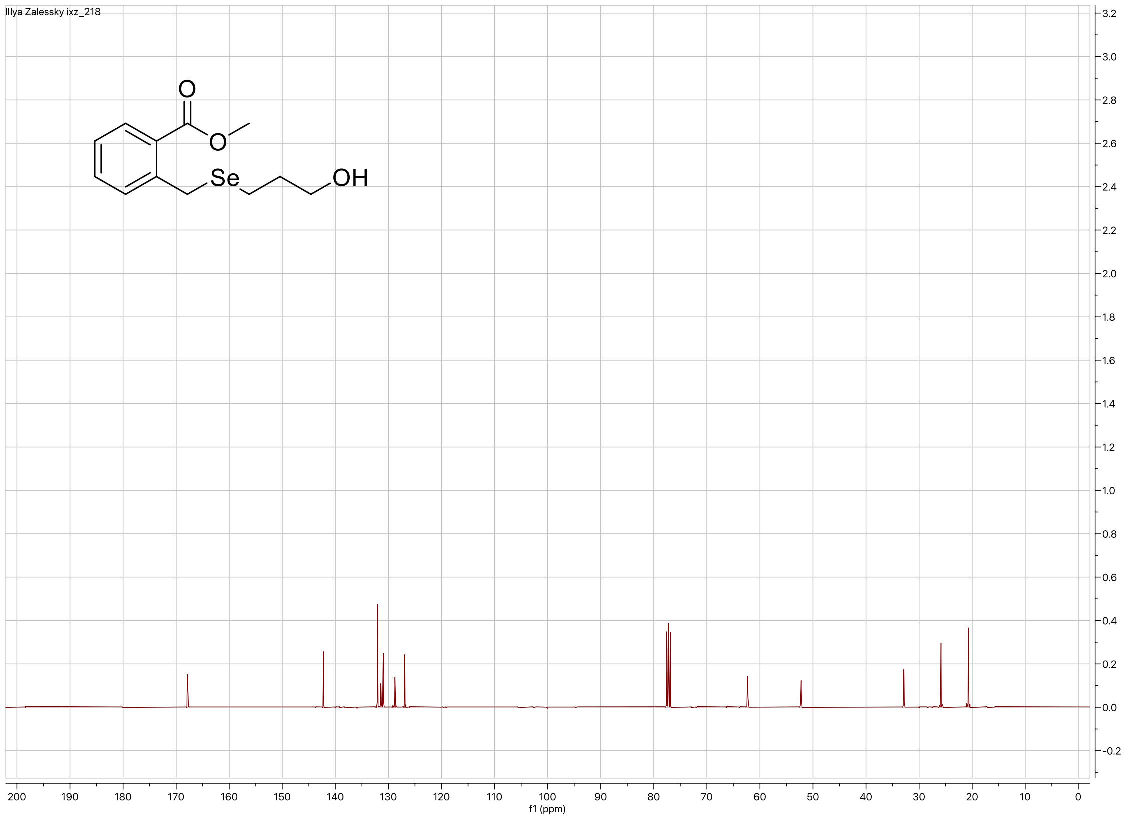

2-(((3-Hydroxypropyl)selanyl)methyl)benzoic acid (S46)

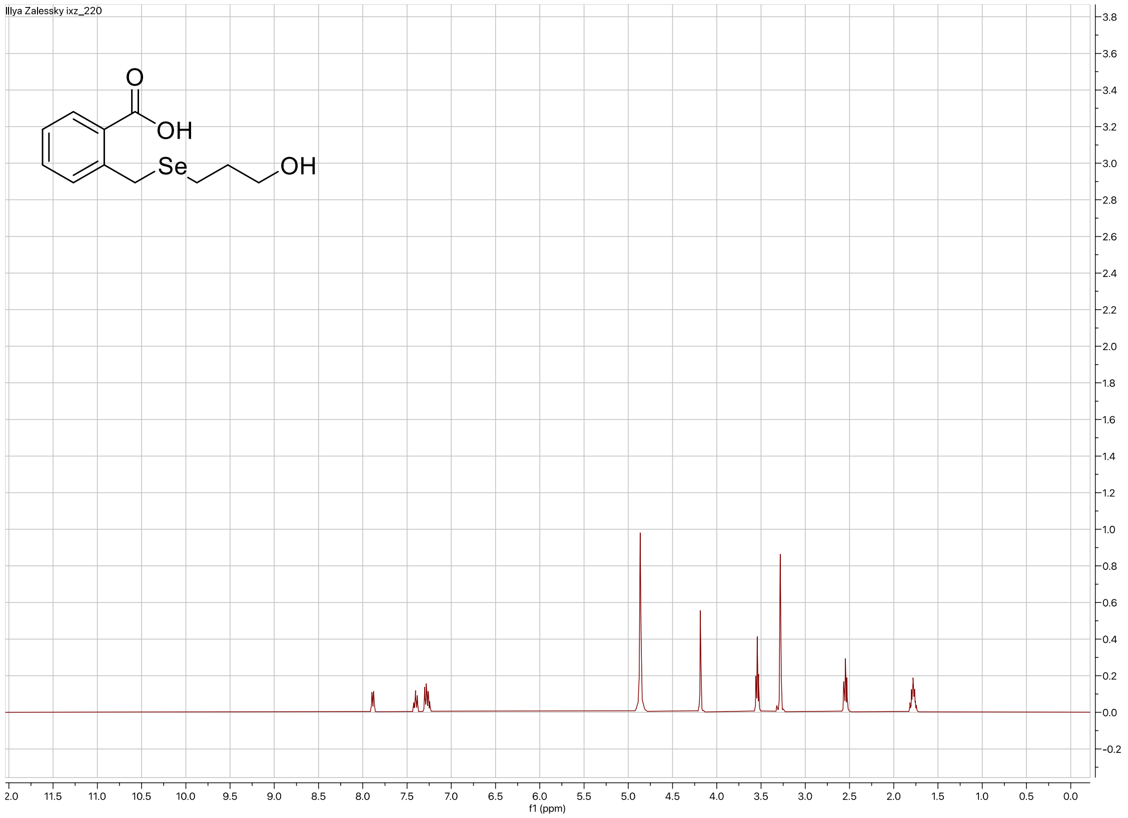

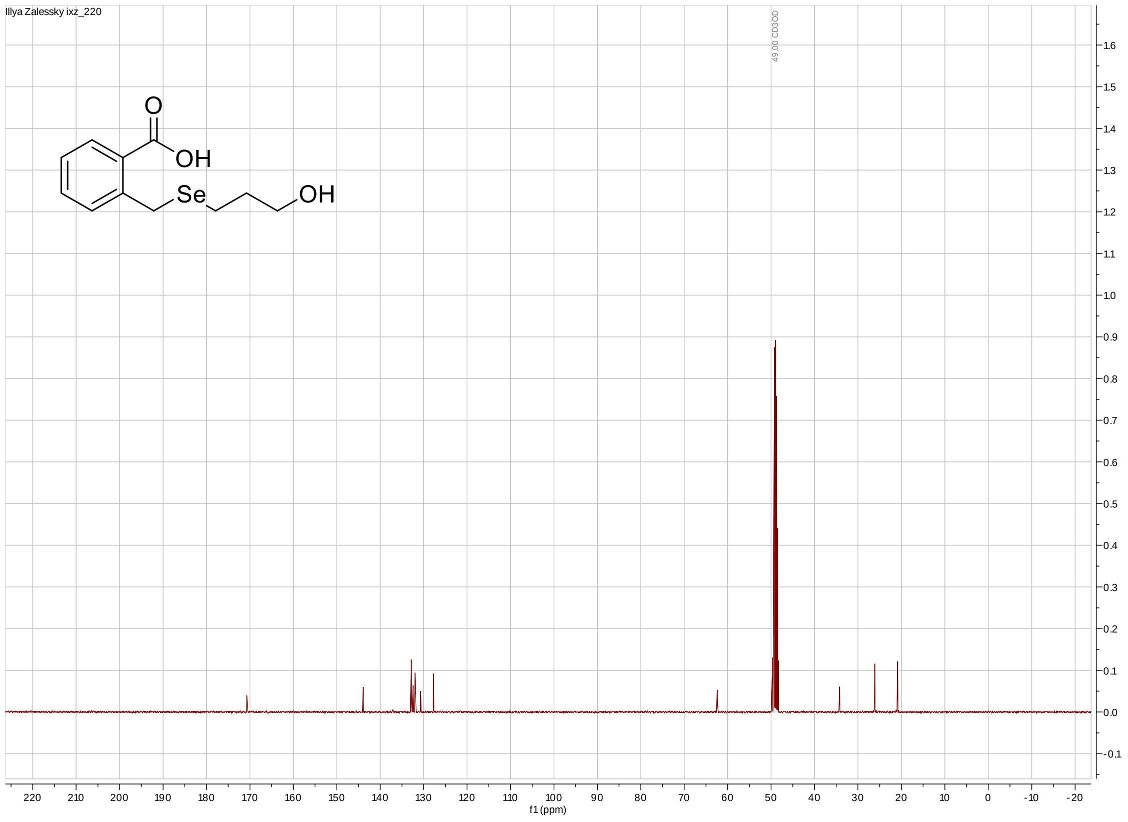

4,5-Dihydro-3*H*-benzo[*g*][1,5]oxaselenonin-1(7*H*)-one (52)

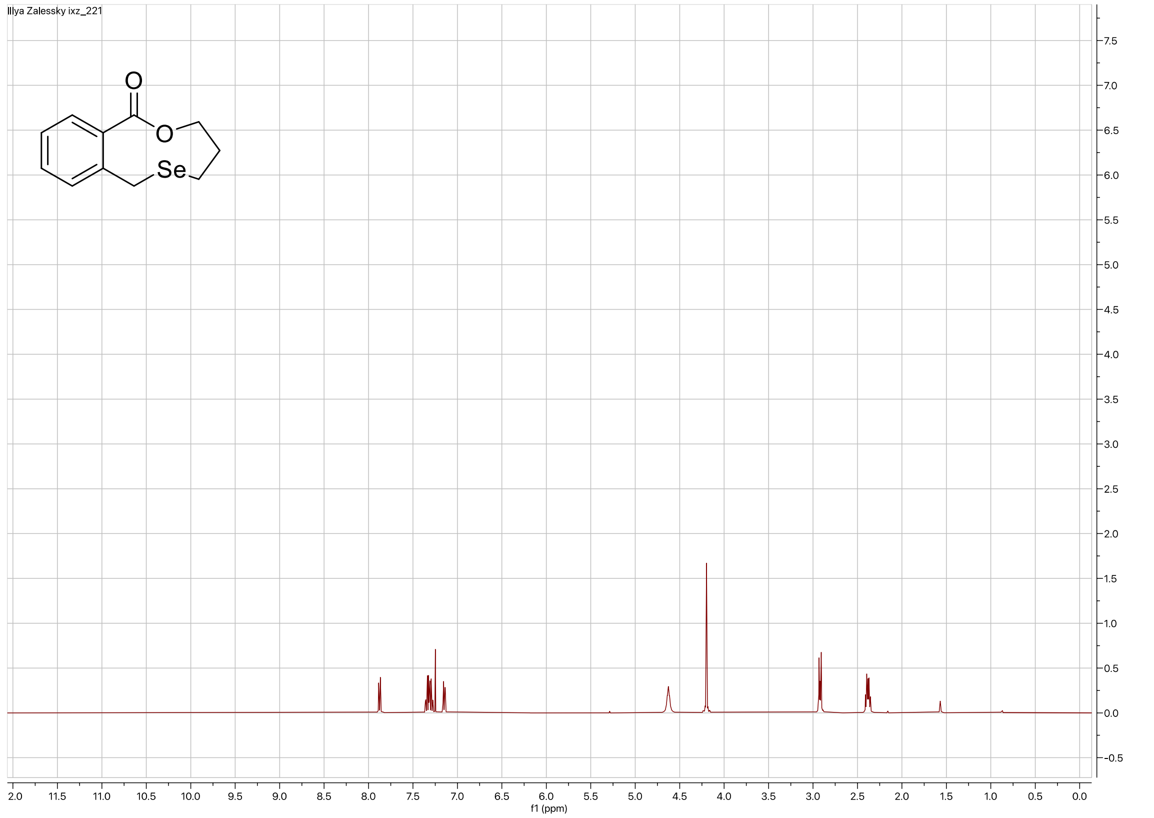

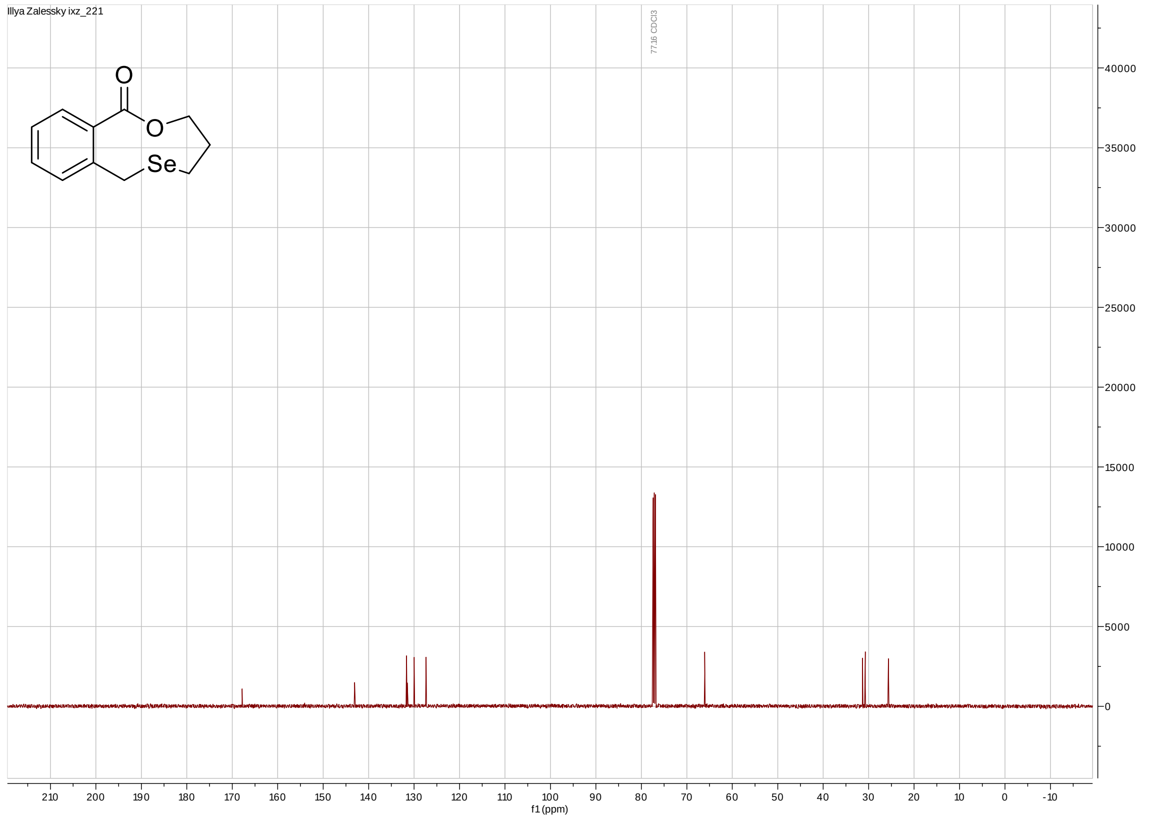

Methyl 2-((benzyl(3-hydroxypropyl) amino) methyl) benzoate (S47)

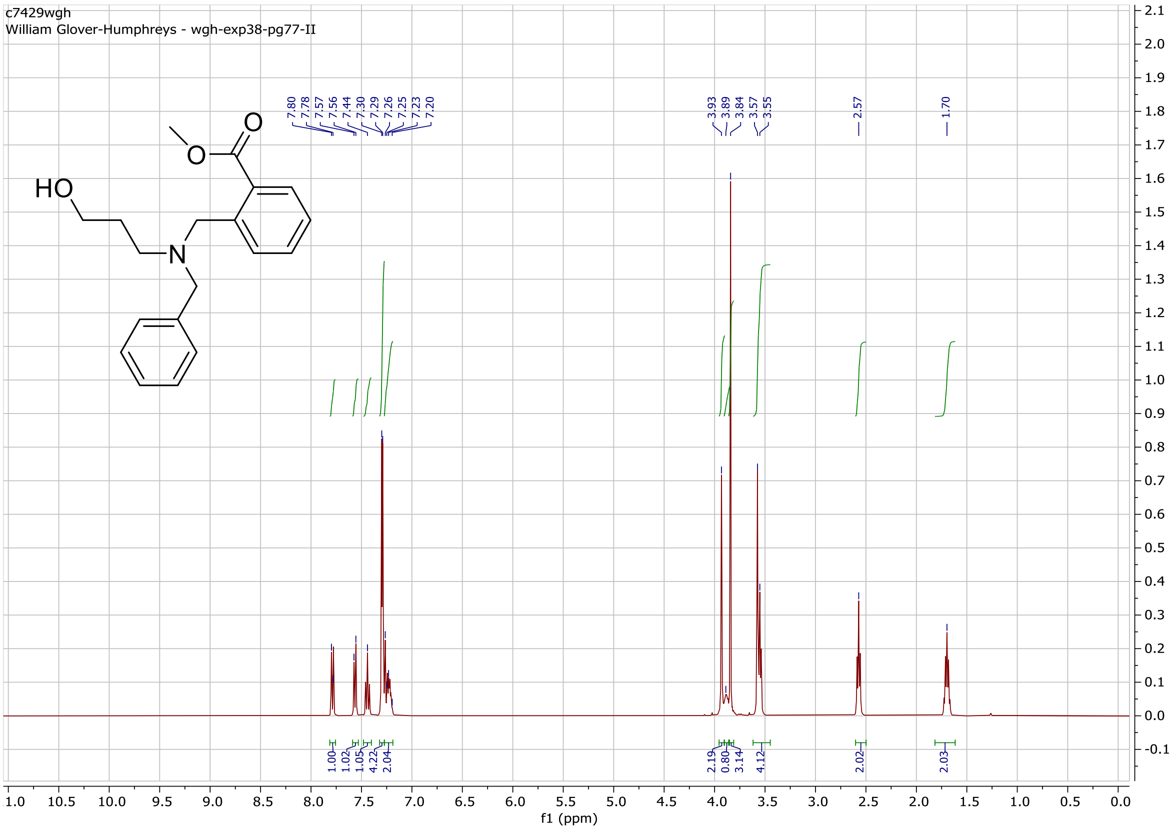

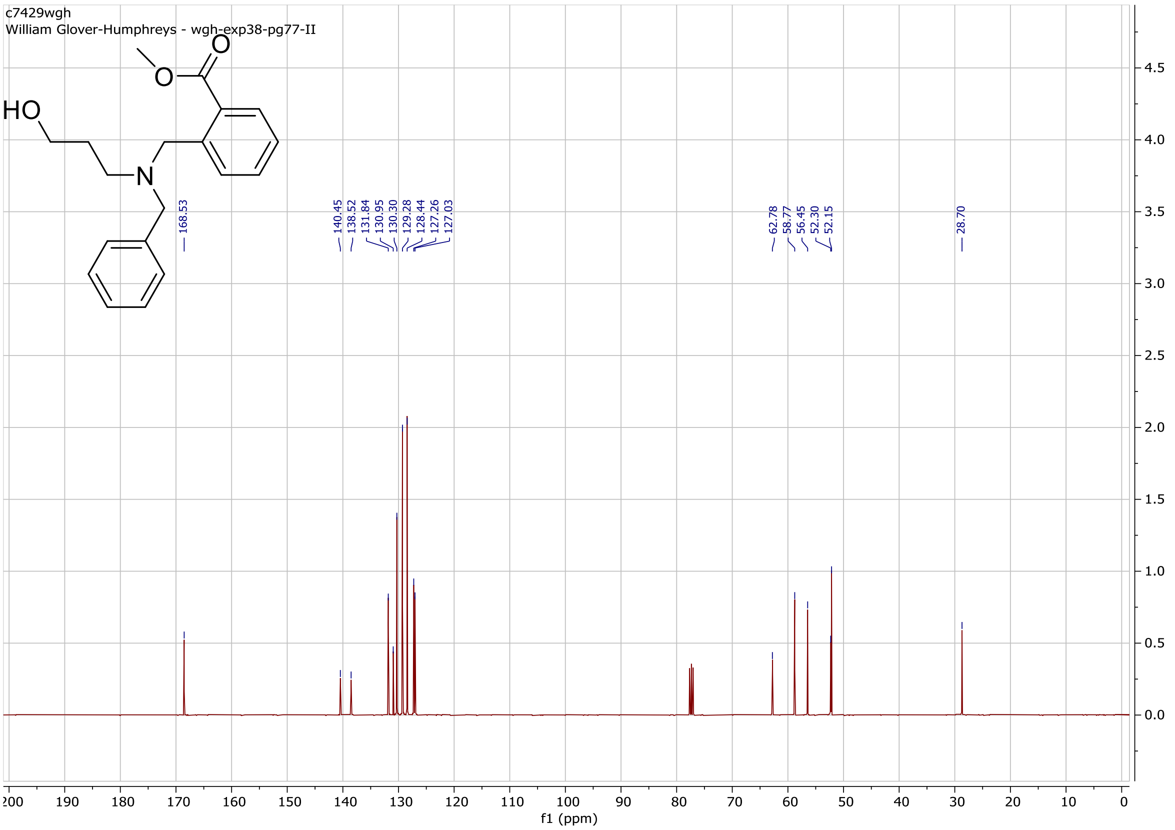

4-Benzyl-4,5,6,7-tetrahydrobenzo[g] [1,5] oxazonin-1(3H)-one (53)

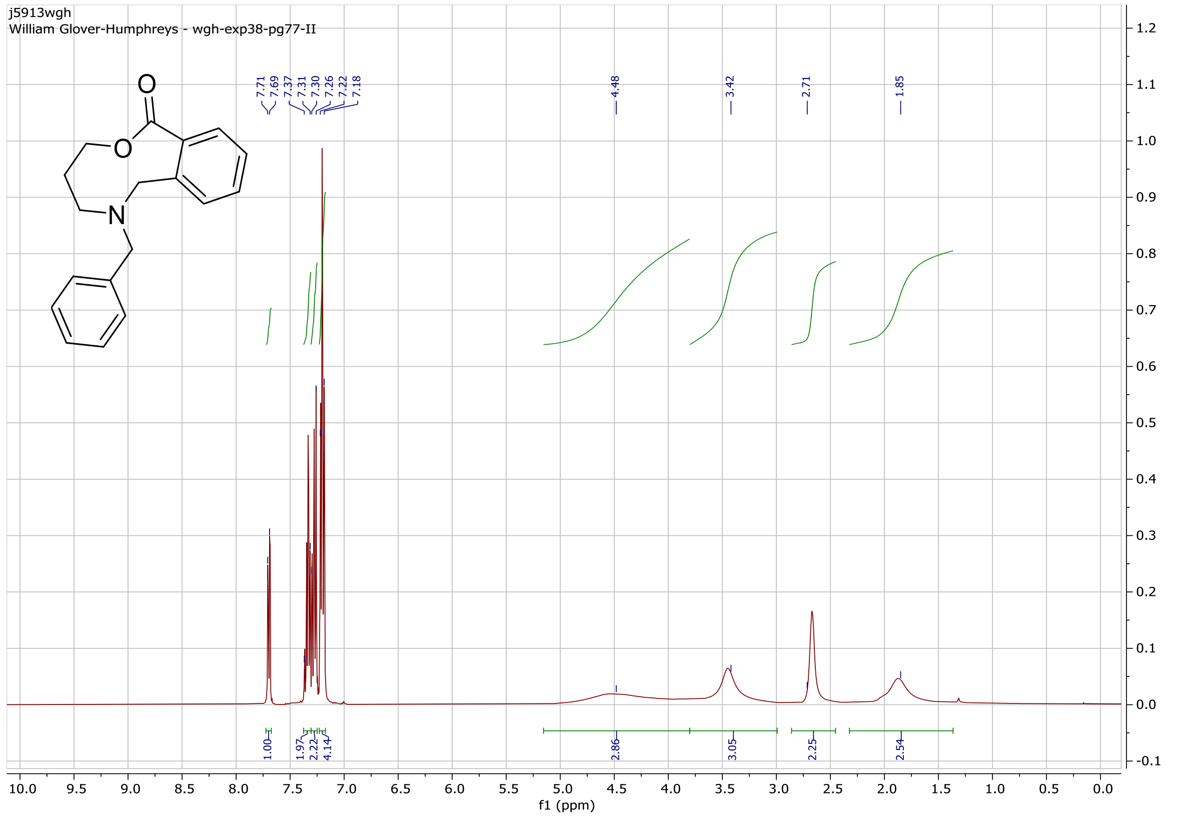

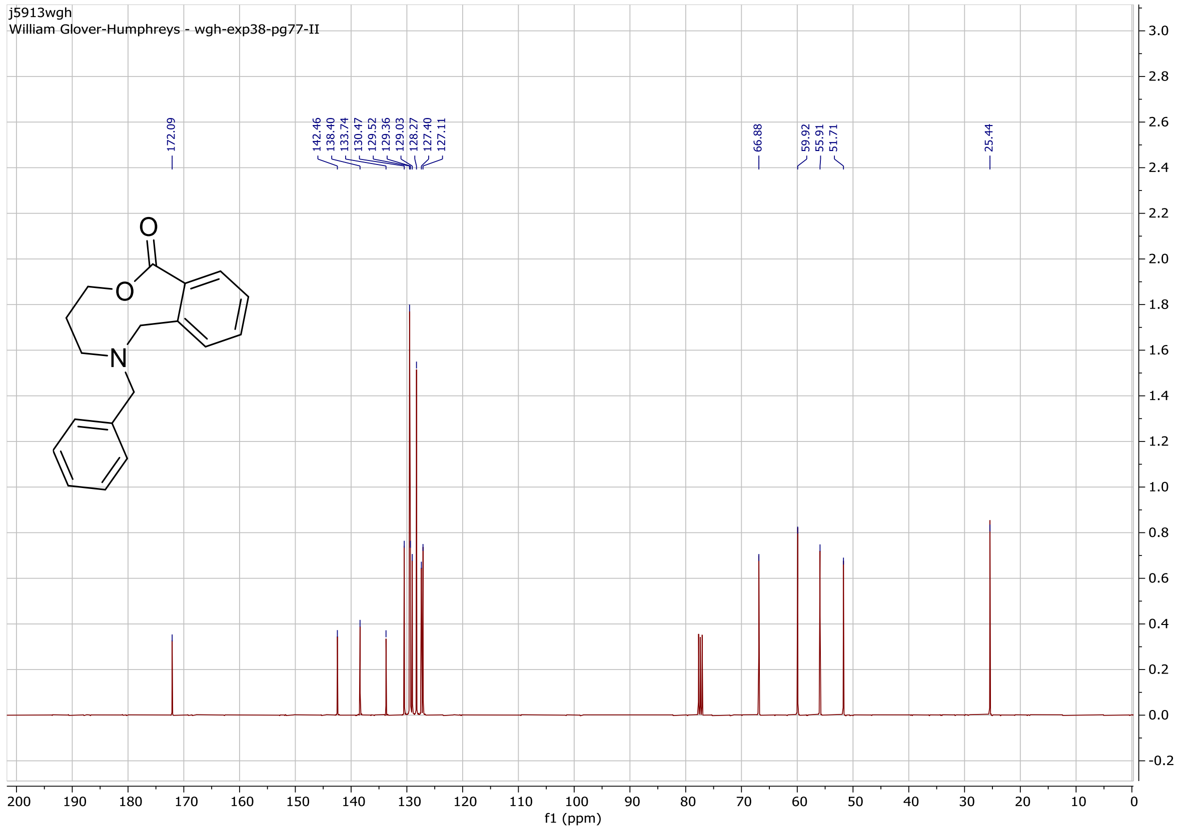

Methyl 2-(2-((2-(2-hydroxyethyl) piperidin-1-yl) methyl) phenyl) acetate (S48)

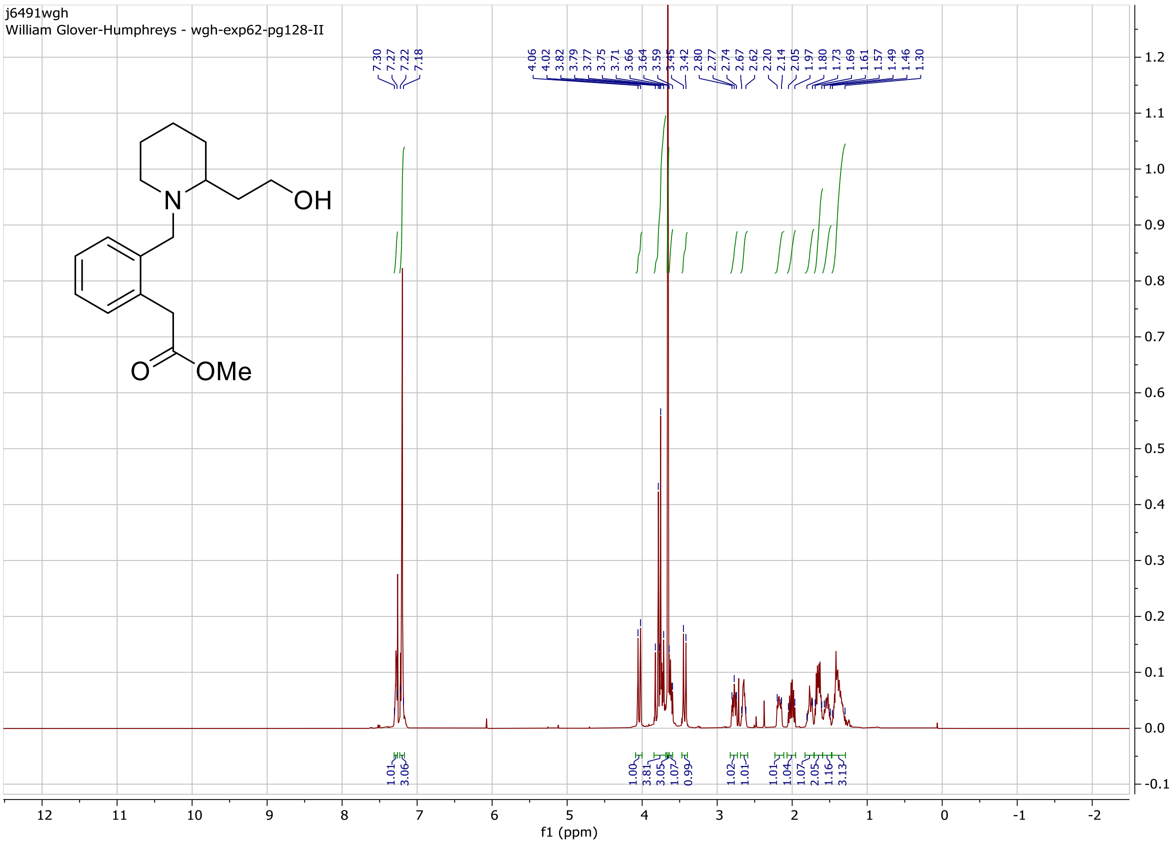

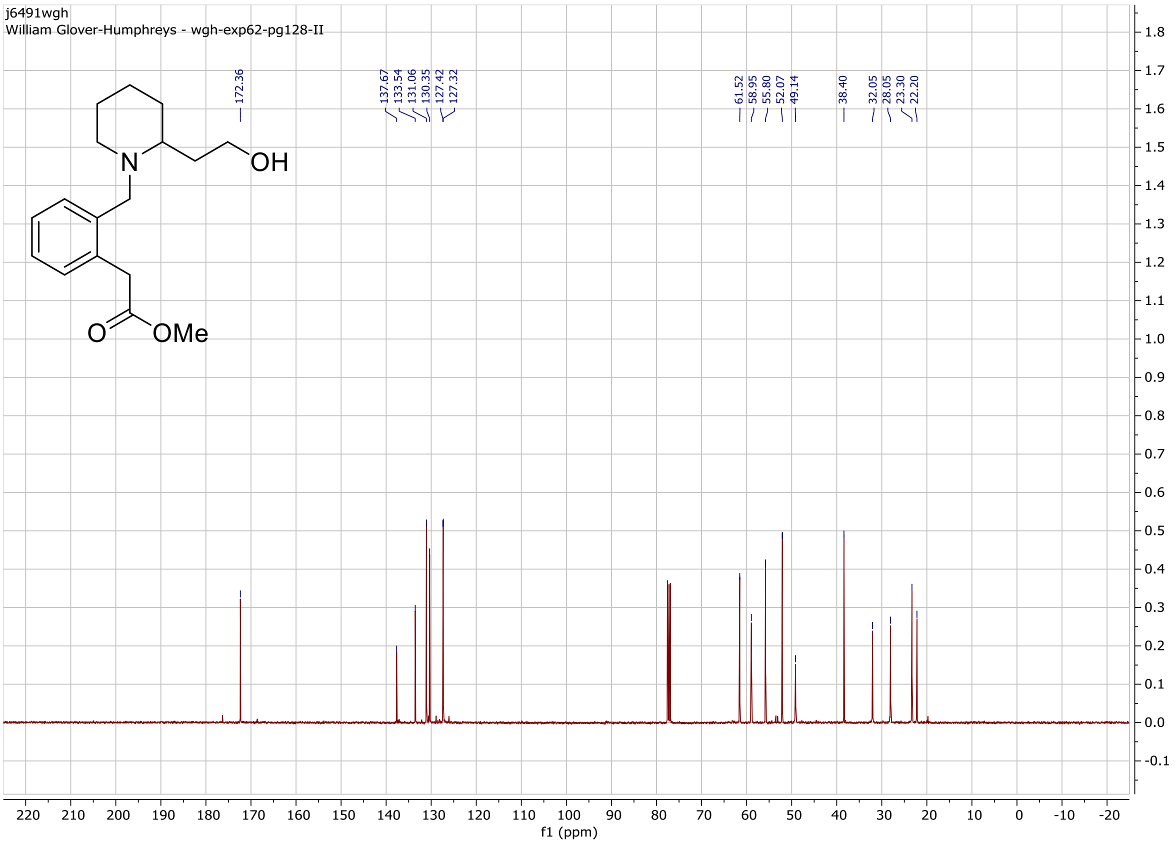

5,9,9a,10,11,12,13,15-Octahydro-6H,8H-benzo[g]pyrido[2,1-d] [1,5] oxazecin-4-one (54)

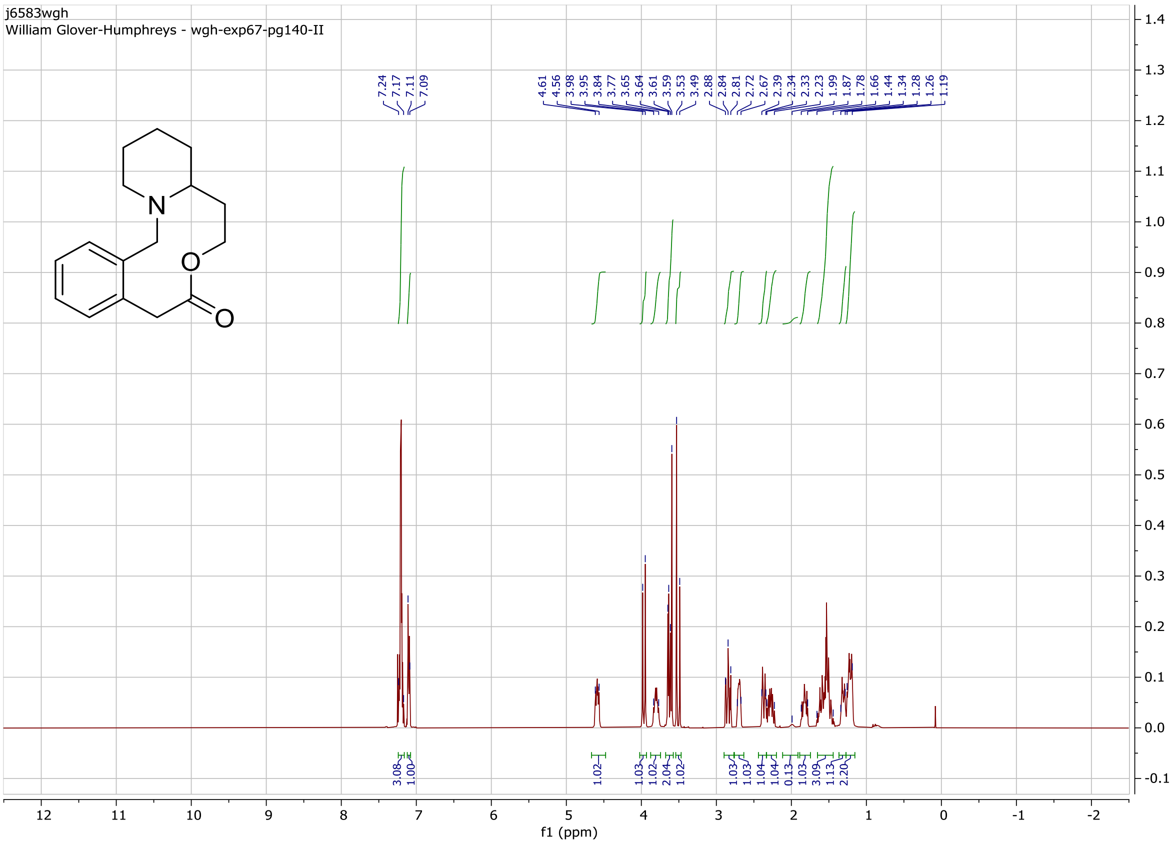

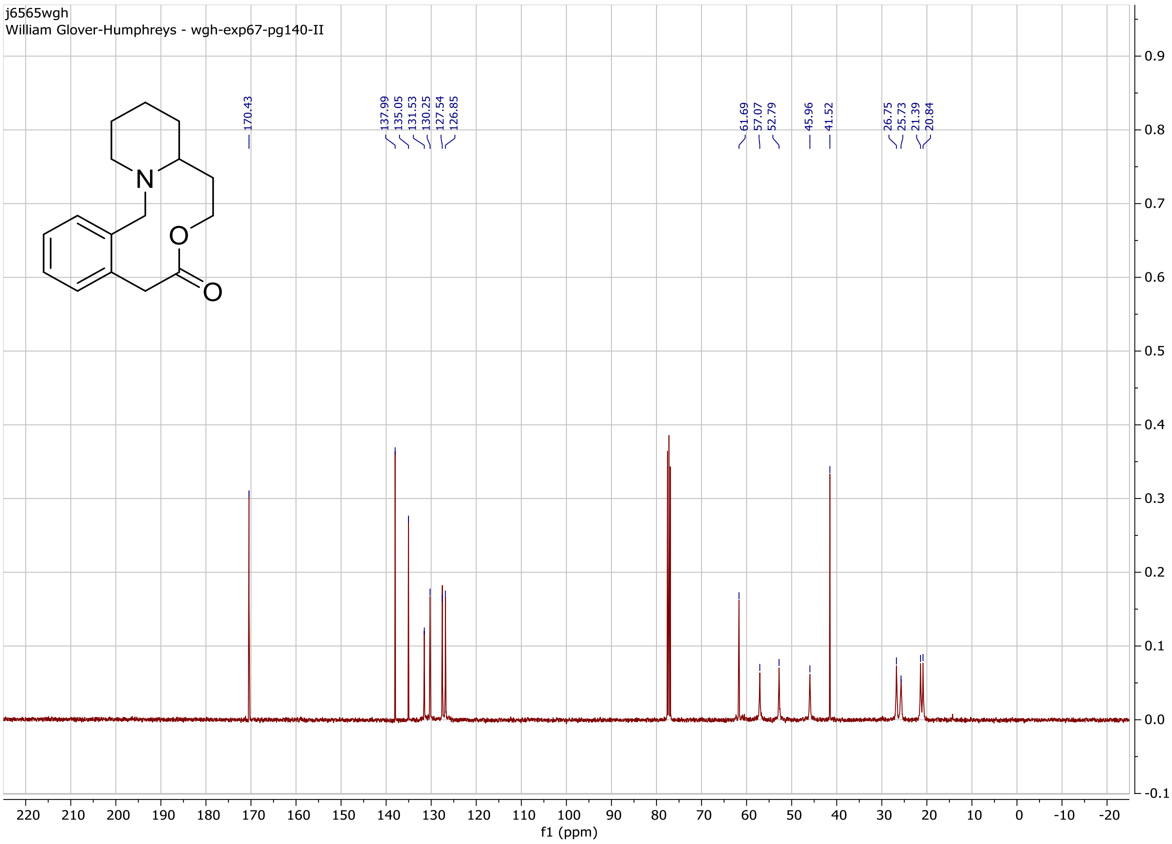

Methyl 2-(2-(6-(2-hydroxypropyl)pyrazin-2-yl)phenyl)acetate (S51)

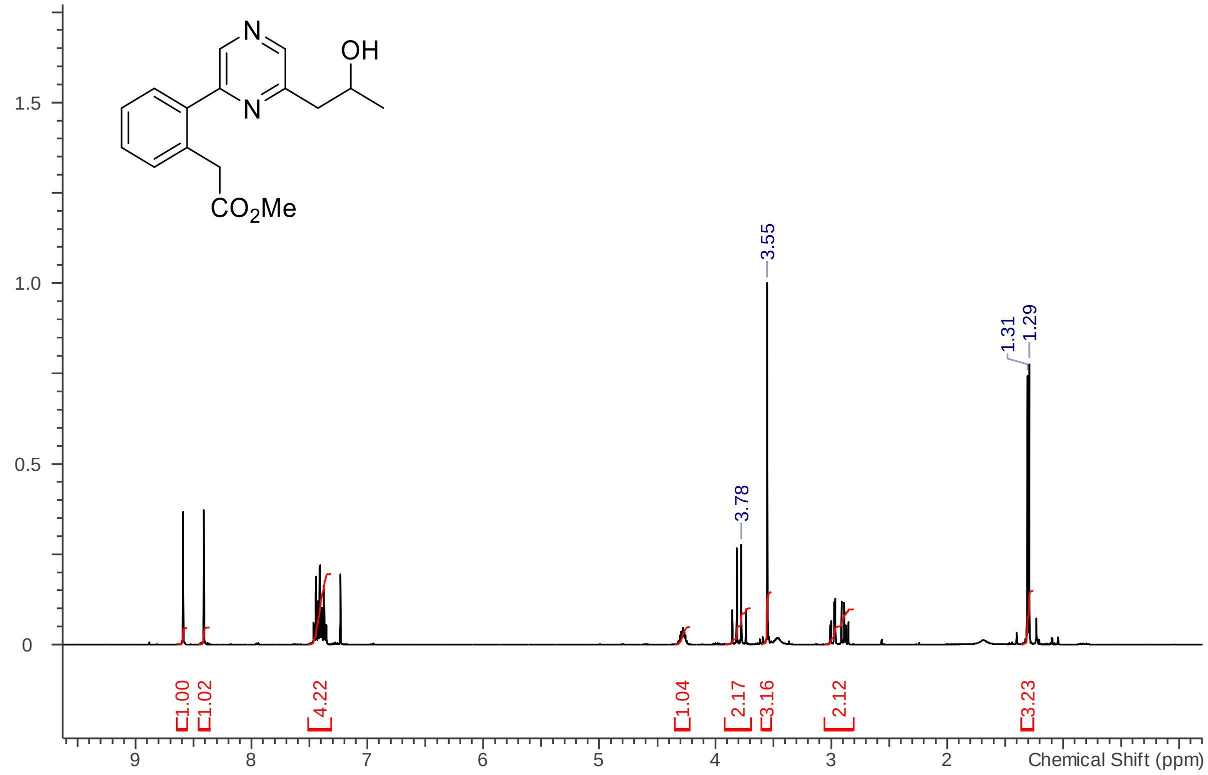

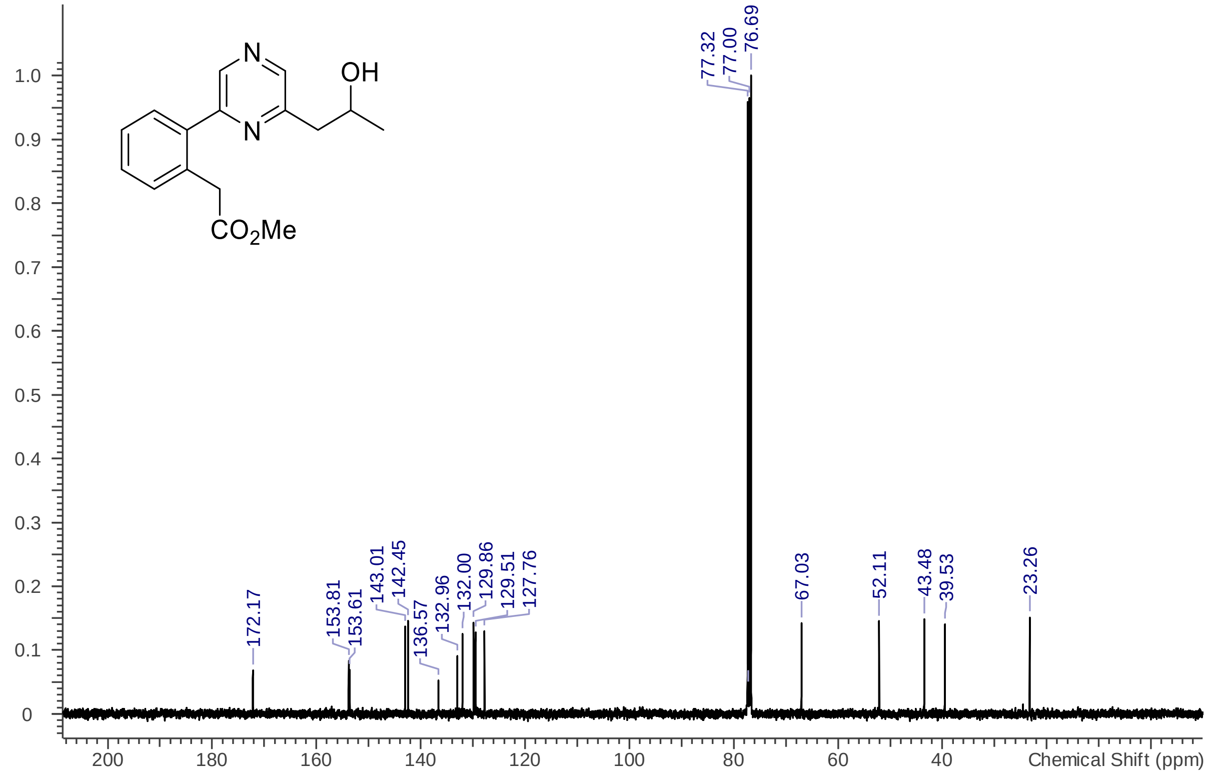

2-(2-(6-(2-Hydroxypropyl)pyrazin-2-yl)phenyl)acetic acid (S52)

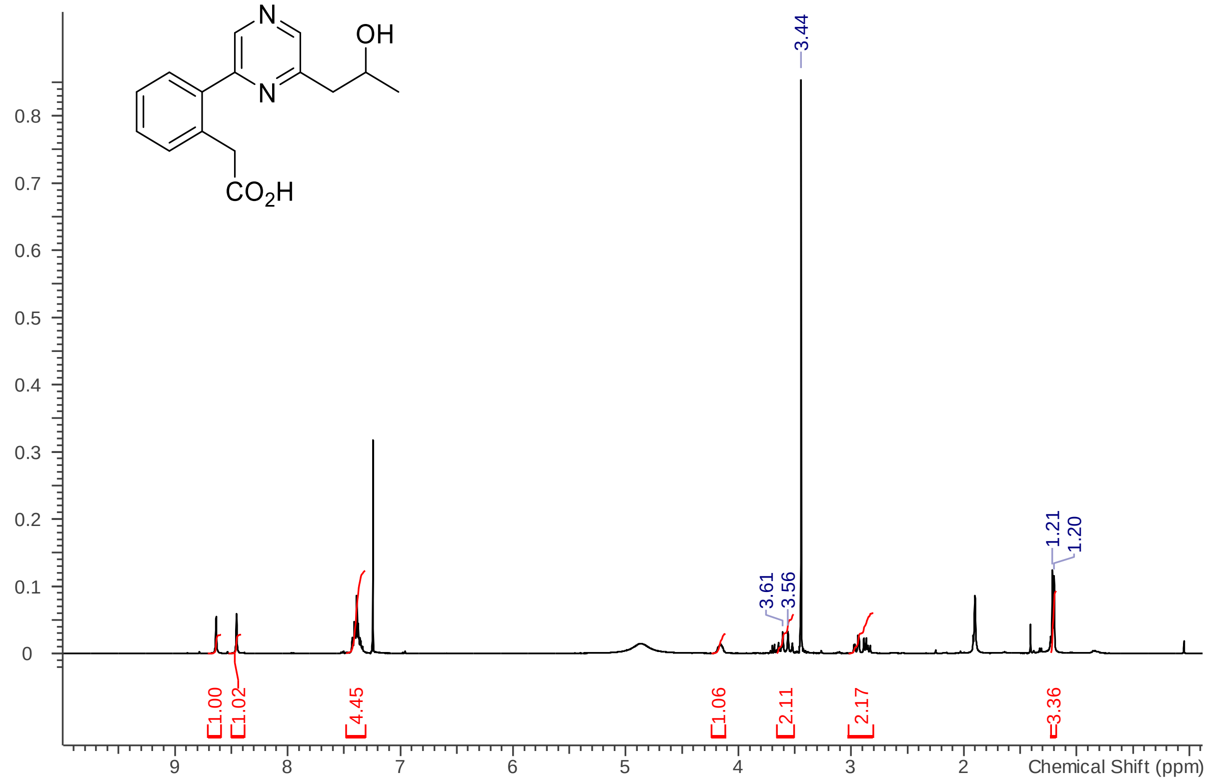

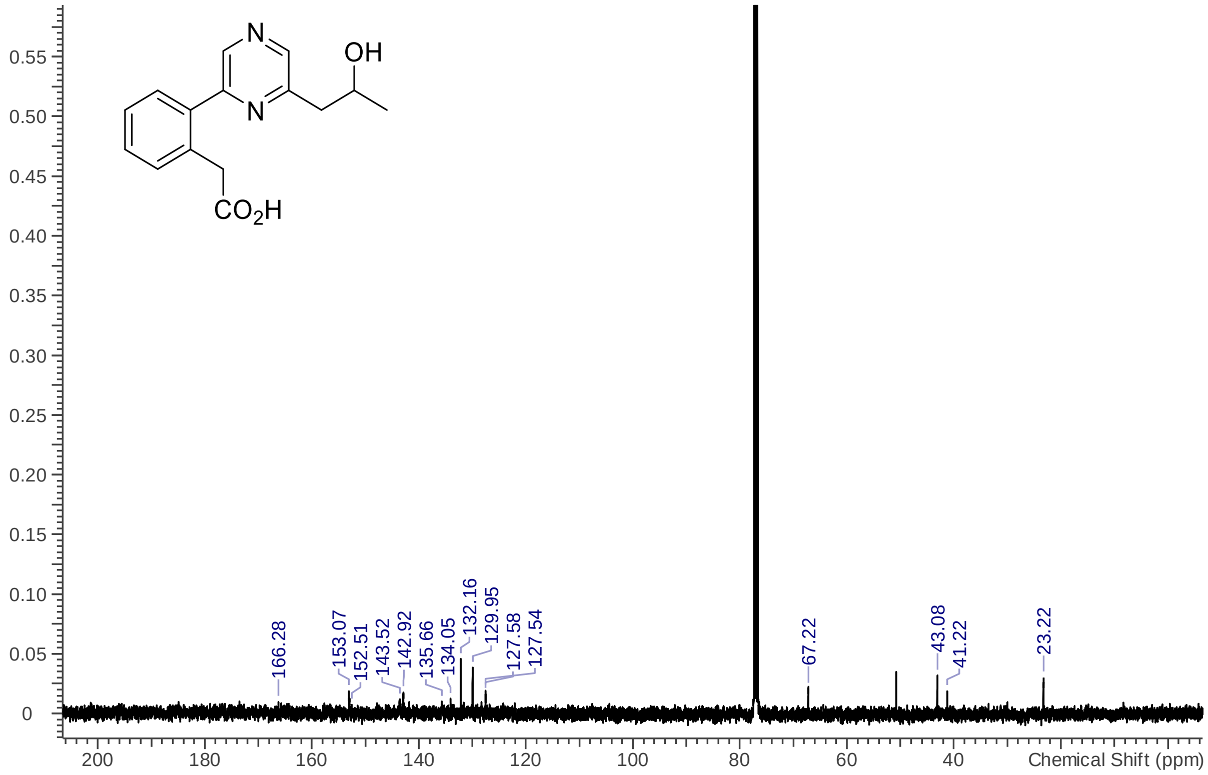

4-Methyl-4,5-dihydro-6,10-(azeno)benzo[*i*][1]oxa[6]azacyclododecin-2(1*H*)-one (55)

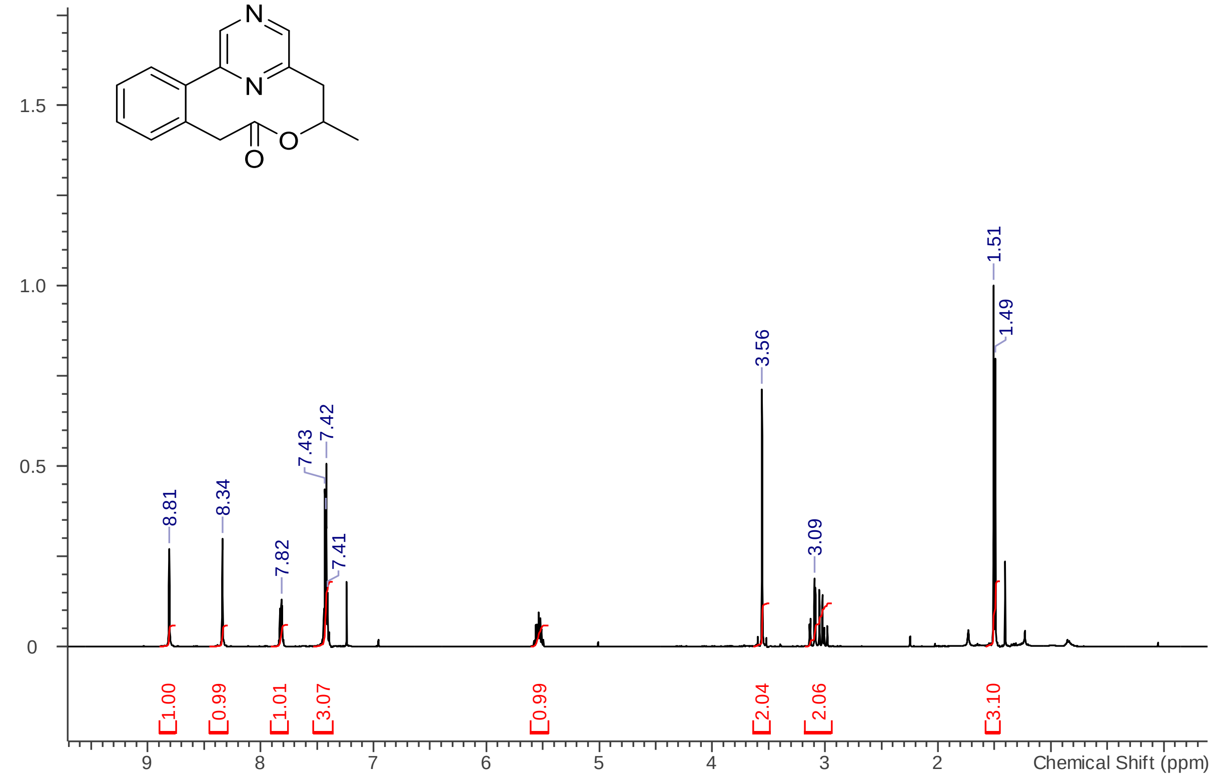

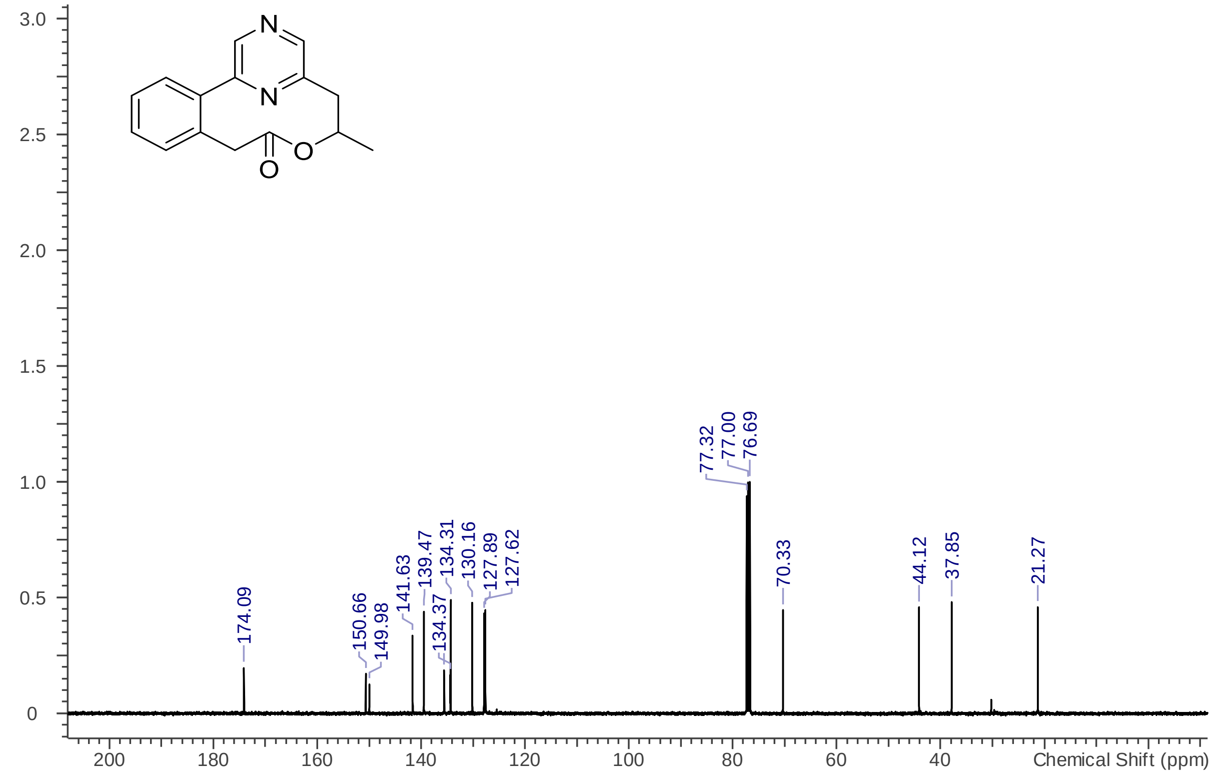

Methyl 3-((benzyl(4-hydroxybutyl) amino) methyl) isoquinoline-4-carboxylate (S53)

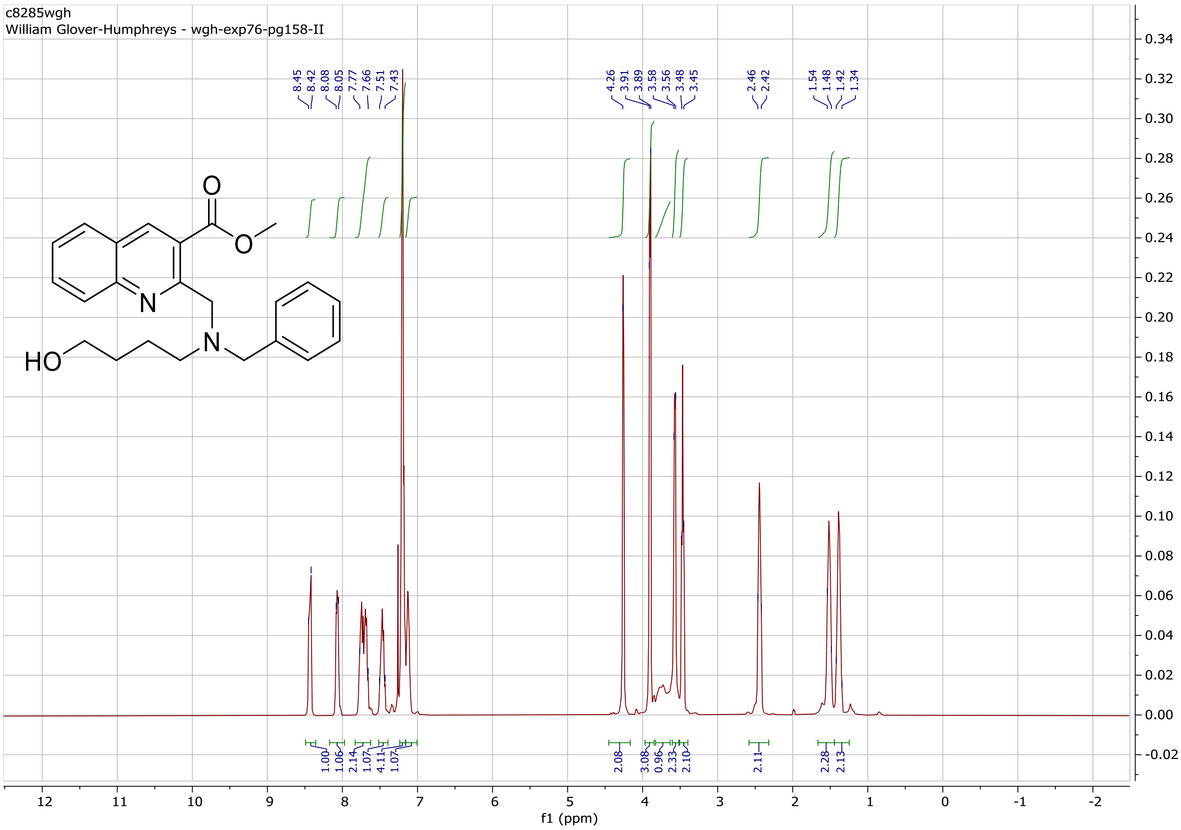

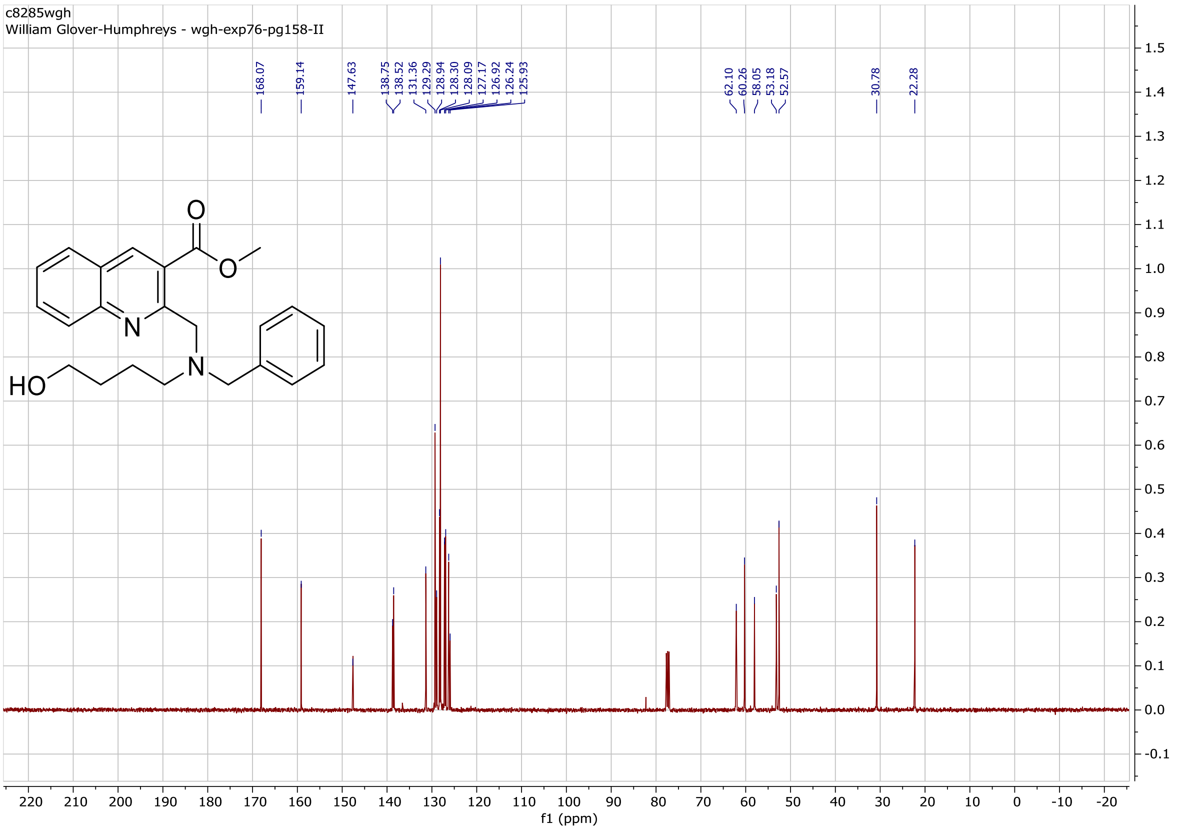

7-Benzyl-3,4,5,6,7,6-hexahydro-1H- [1,6] oxazecino [4,3-b] quinolin-1-one (56)

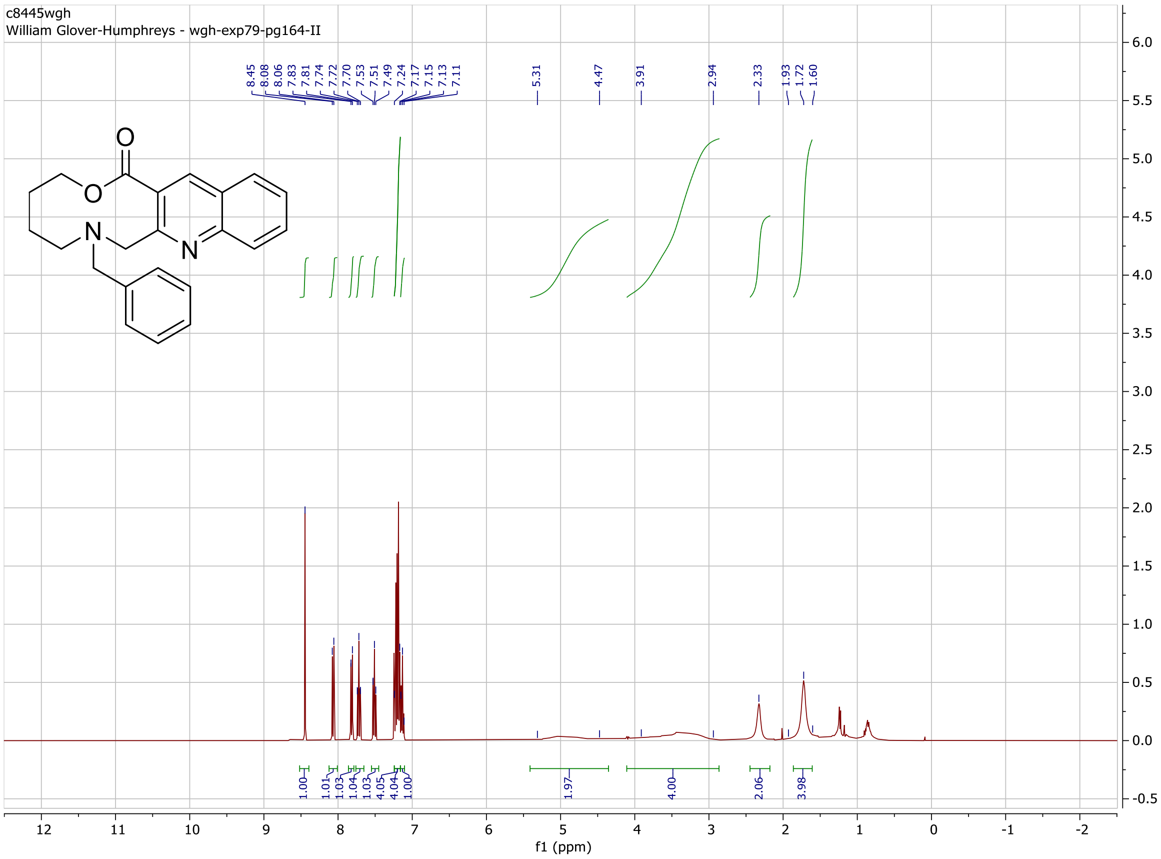

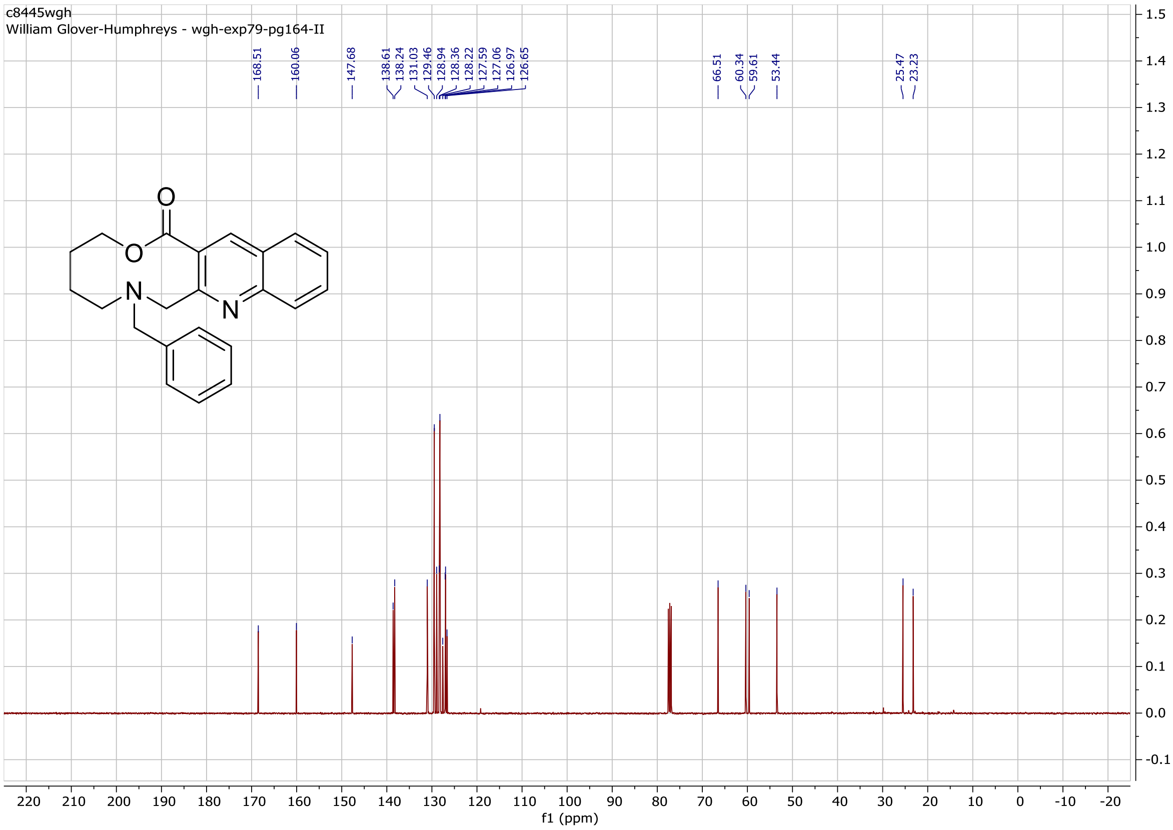

Methyl 2-((butyl(2-hydroxyethyl) amino) methyl) benzoate (S54)

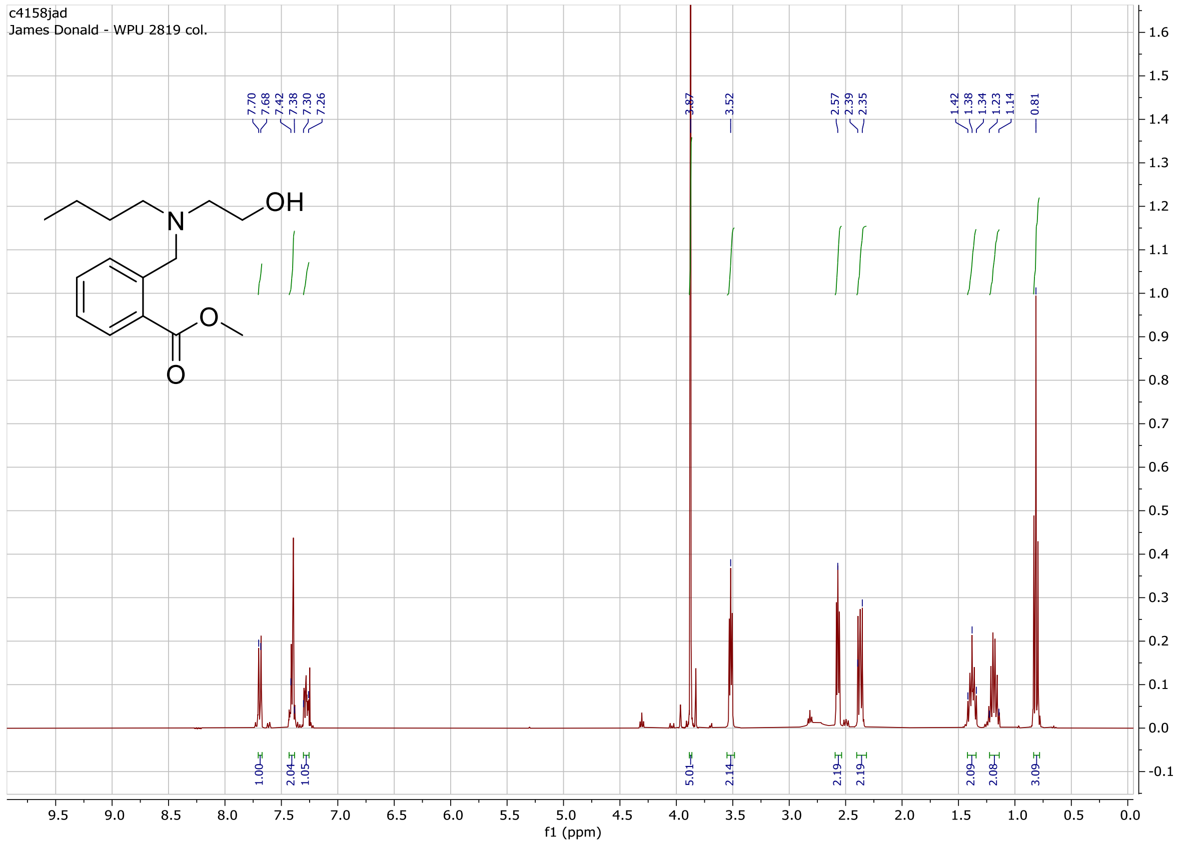

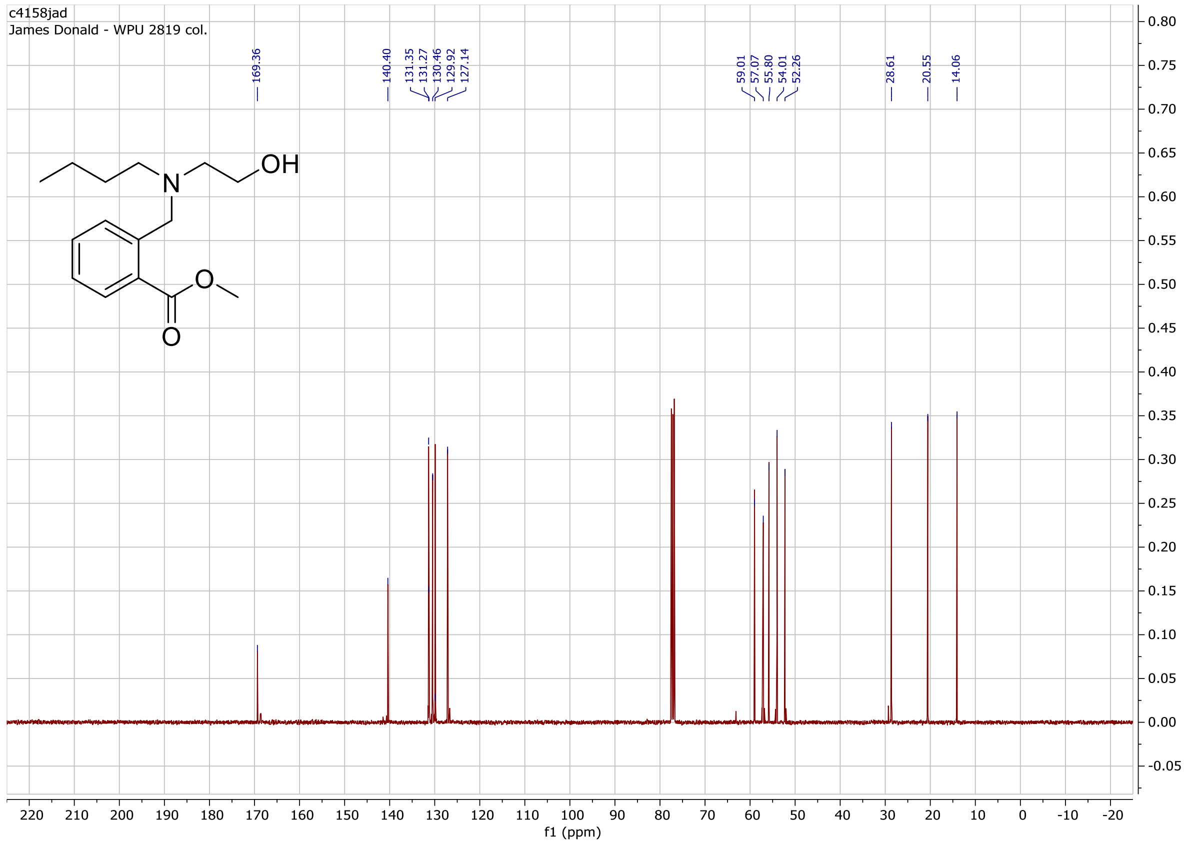

5-Butyl-3,4,5,4-tetrahydro-1H-benzo[f] [1,4] oxazocin-1-one (57)

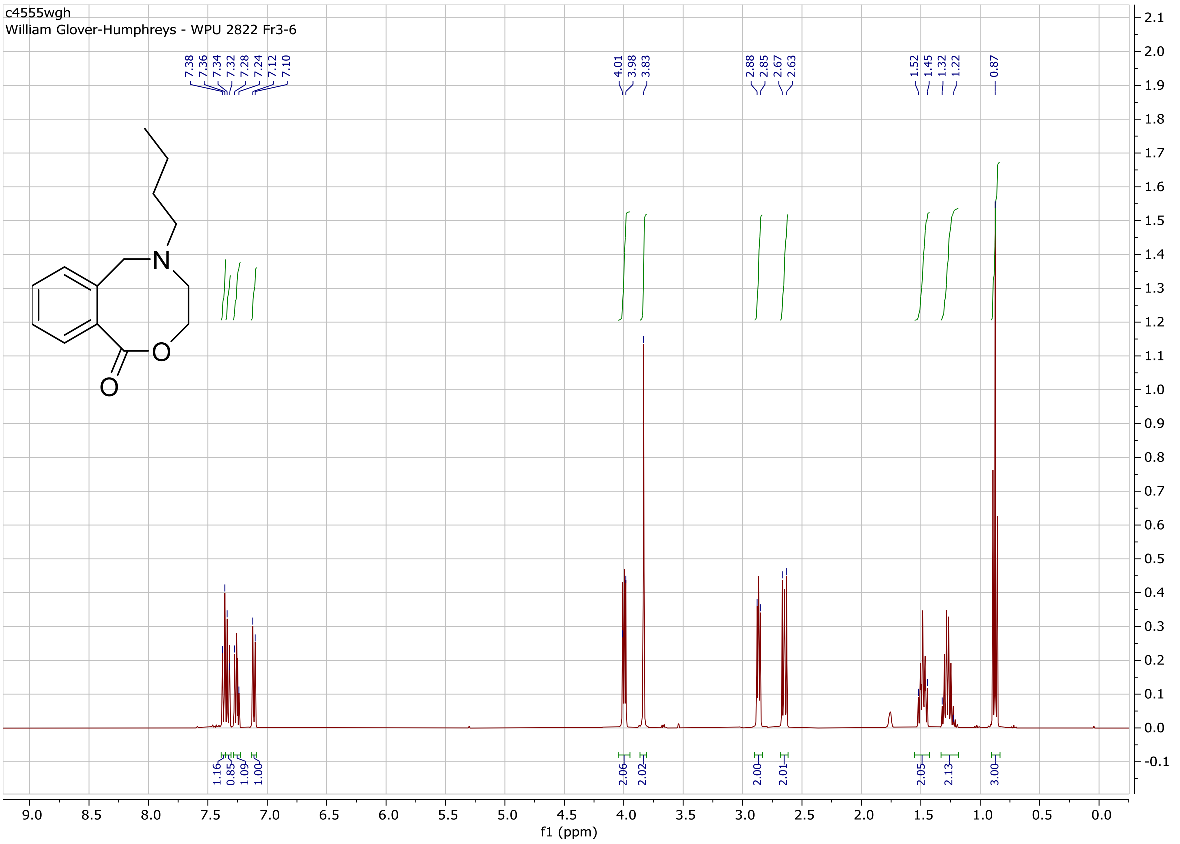

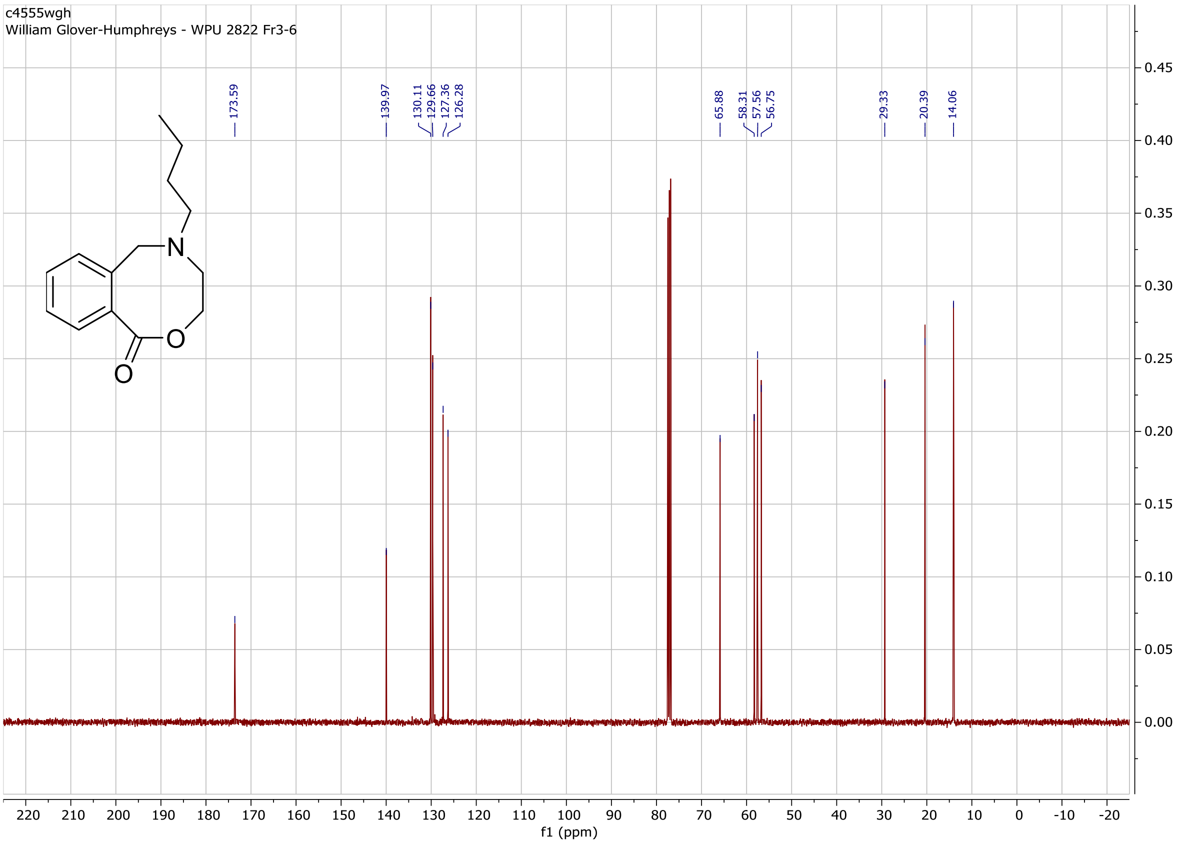

Methyl 2-(bromomethyl) quinoline-3-carboxylate (S55)

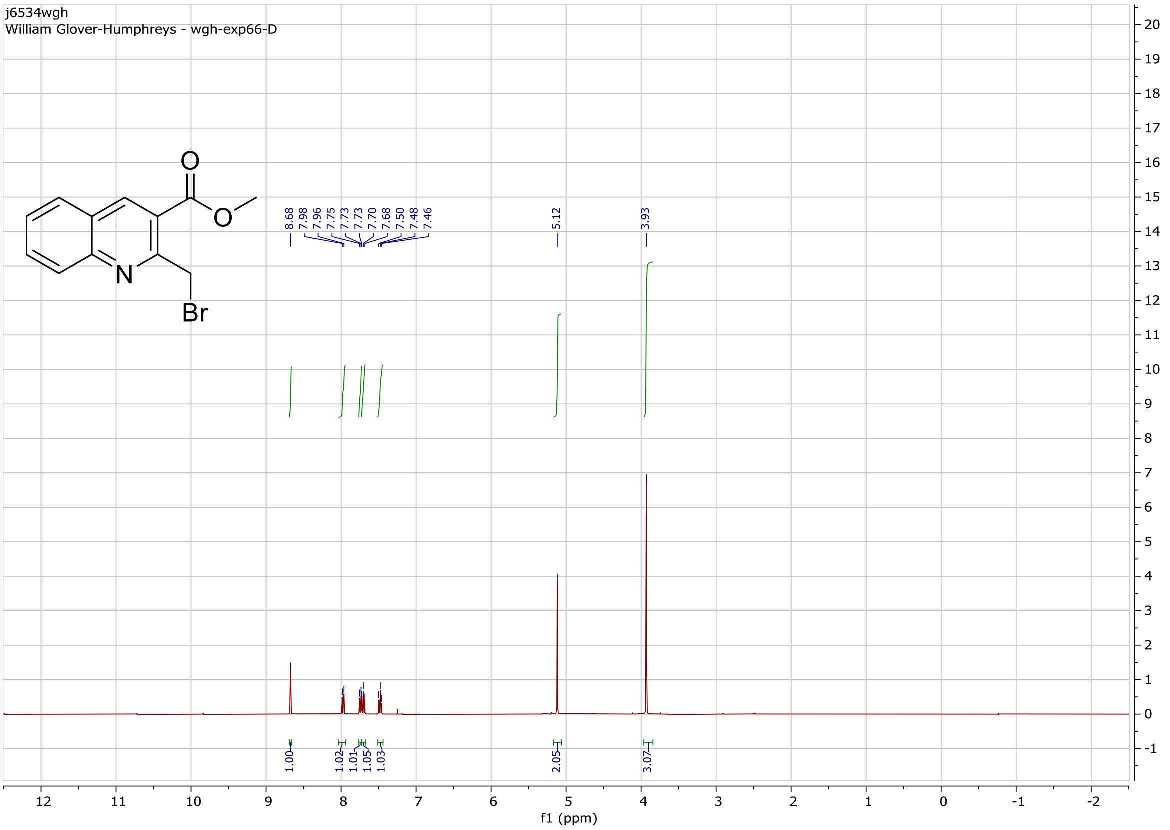

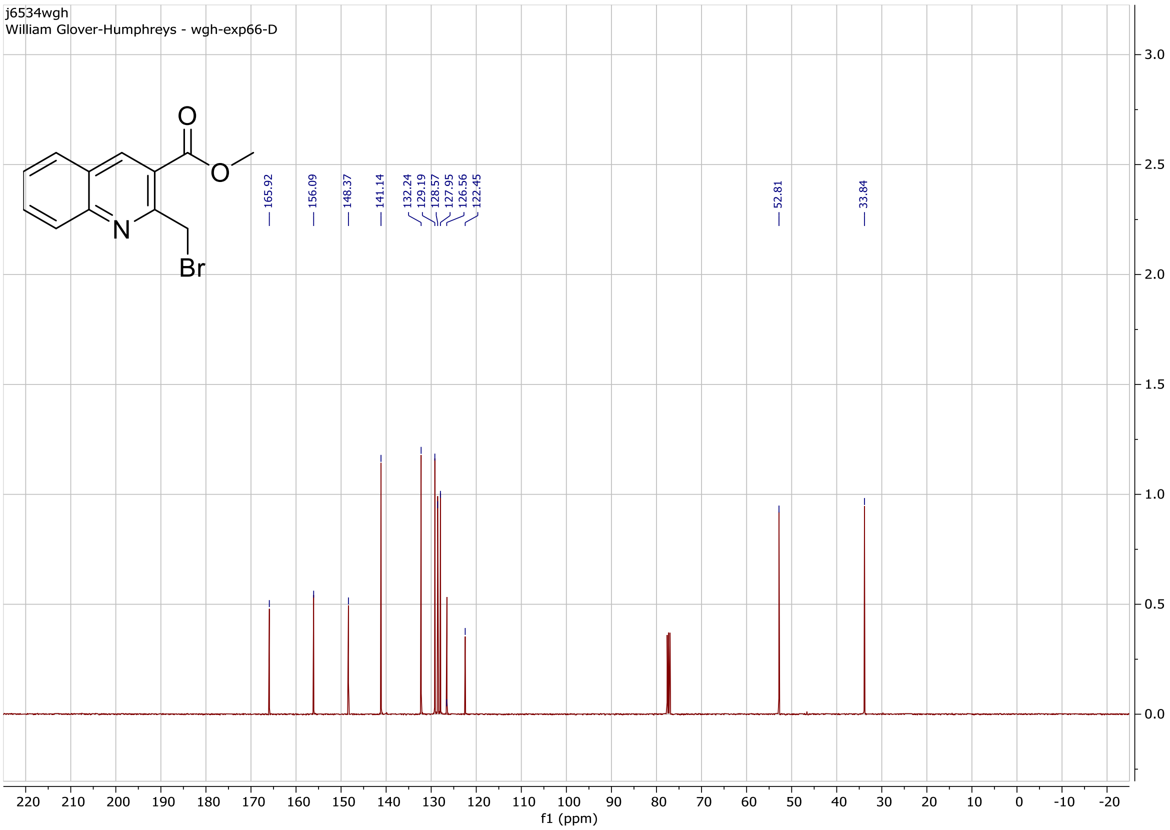

Methyl 2-((2-(2-hydroxyethyl) piperidin-1-yl) methyl) quinoline-3-carboxylate (S56)

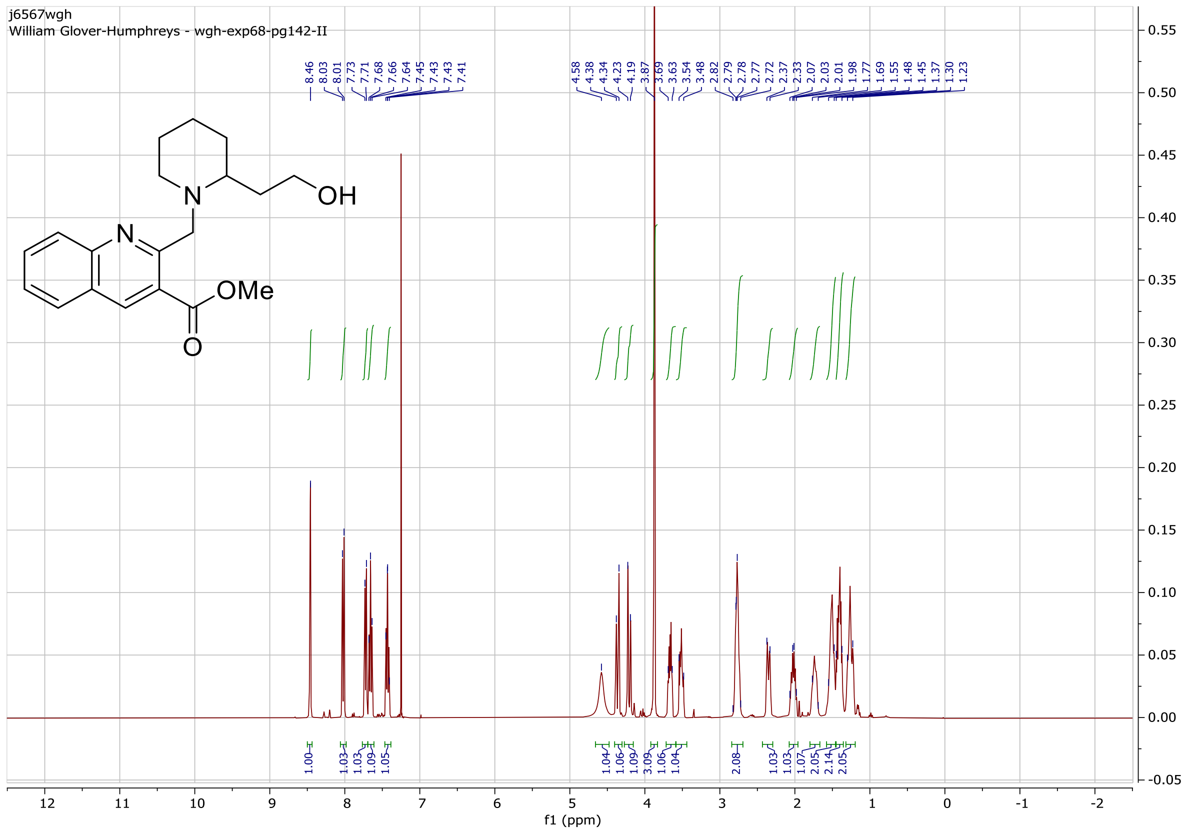

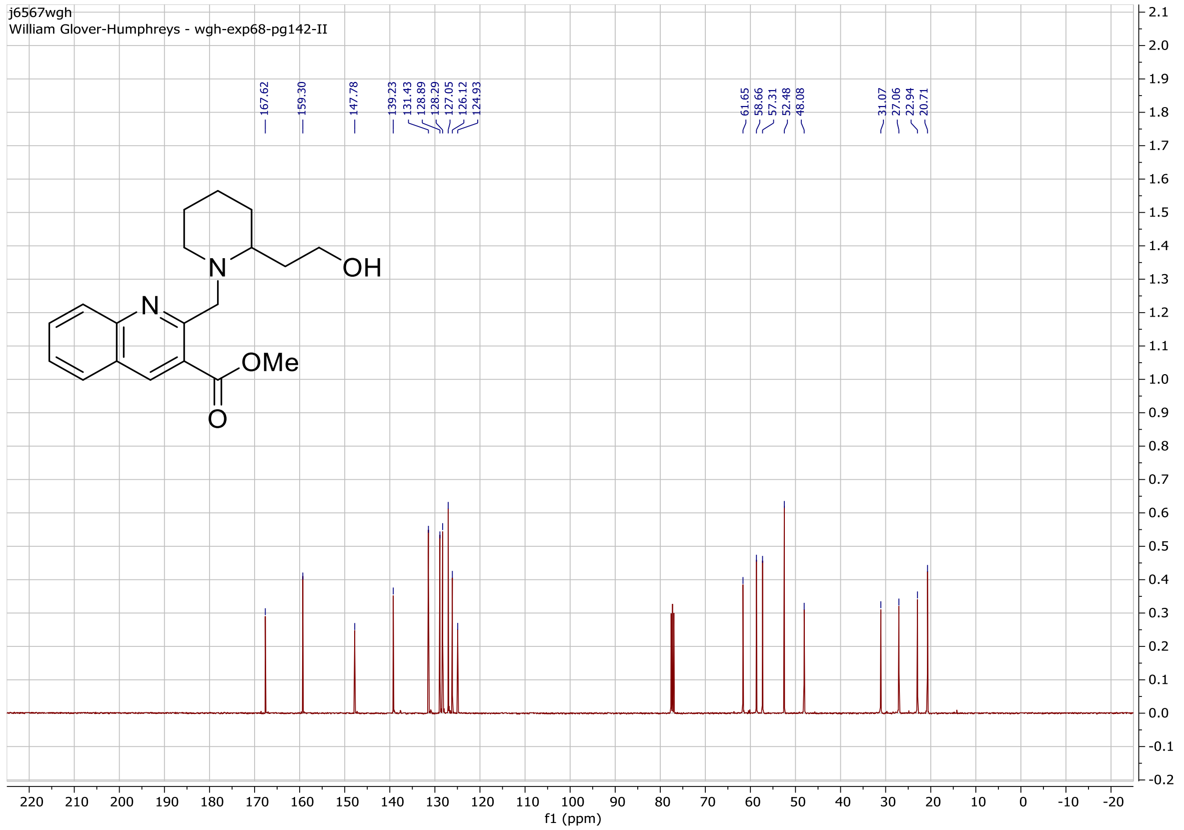

2,3,4,4a,5,4-Hexahydro-1H-pyrido [2',1':4,5] [1,5] oxazonino[7,6-b] quinolin-8(15H)-one (58) In solution in $CDCl_3$, the product exists as a 4:1 mixture of rotamers

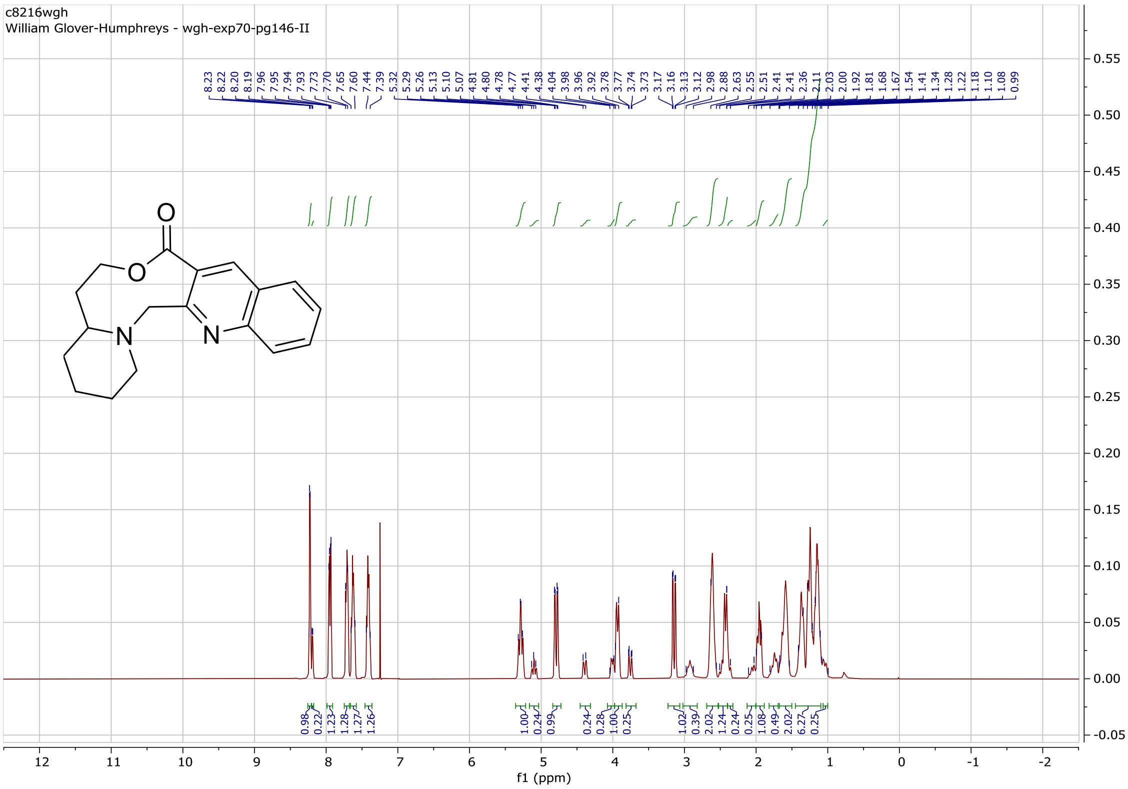

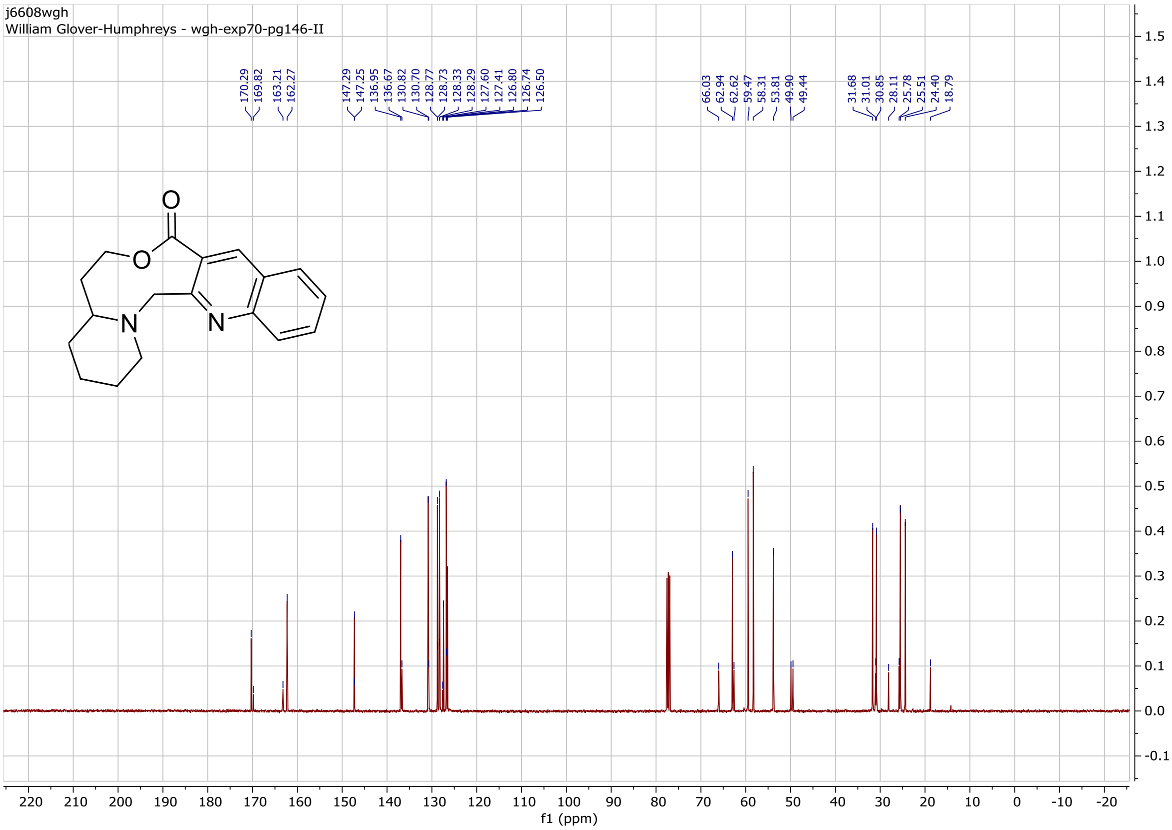

Methyl (*S*)-2-((2-(hydroxymethyl) pyrrolidin-1-yl) methyl) quinoline-3-carboxylate (S57)

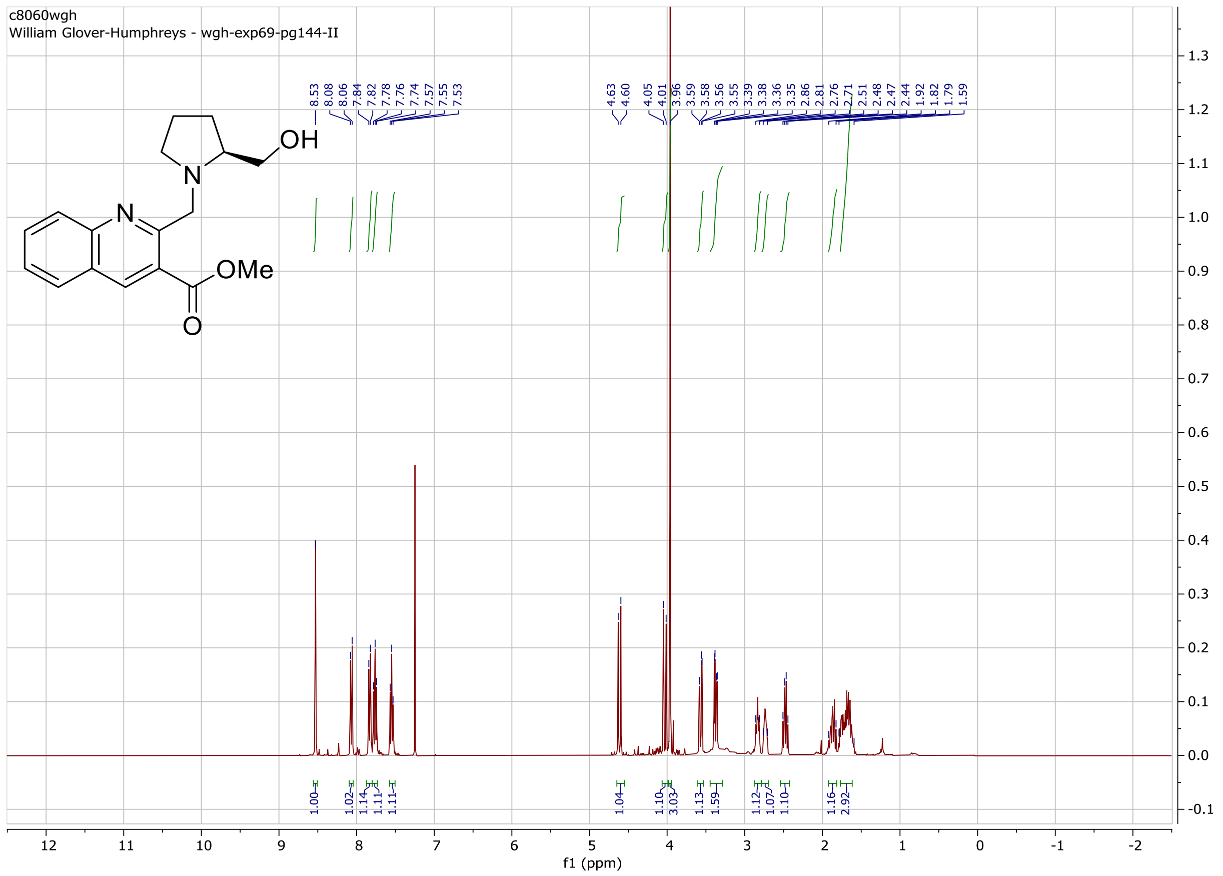
c8060wgh
William Glover-Humphreys - wgh-exp69-pg144-II
OH
N
N
OMe
O
8.53
8.08
8.06
7.84
7.82
7.78
7.76
7.74
7.57
7.55
7.53
4.63
4.60
4.05
4.01
3.96
3.59
3.58
3.56
3.55
3.39
3.38
3.36
3.35
2.86
2.81
2.76
2.71
2.51
2.48
2.47
2.44
1.92
1.82
1.79
1.59
1.00
1.02
1.14
1.11
1.11
1.04
1.10
3.03
1.13
1.59
1.12
1.07
1.10
1.16
2.92
f1 (ppm)

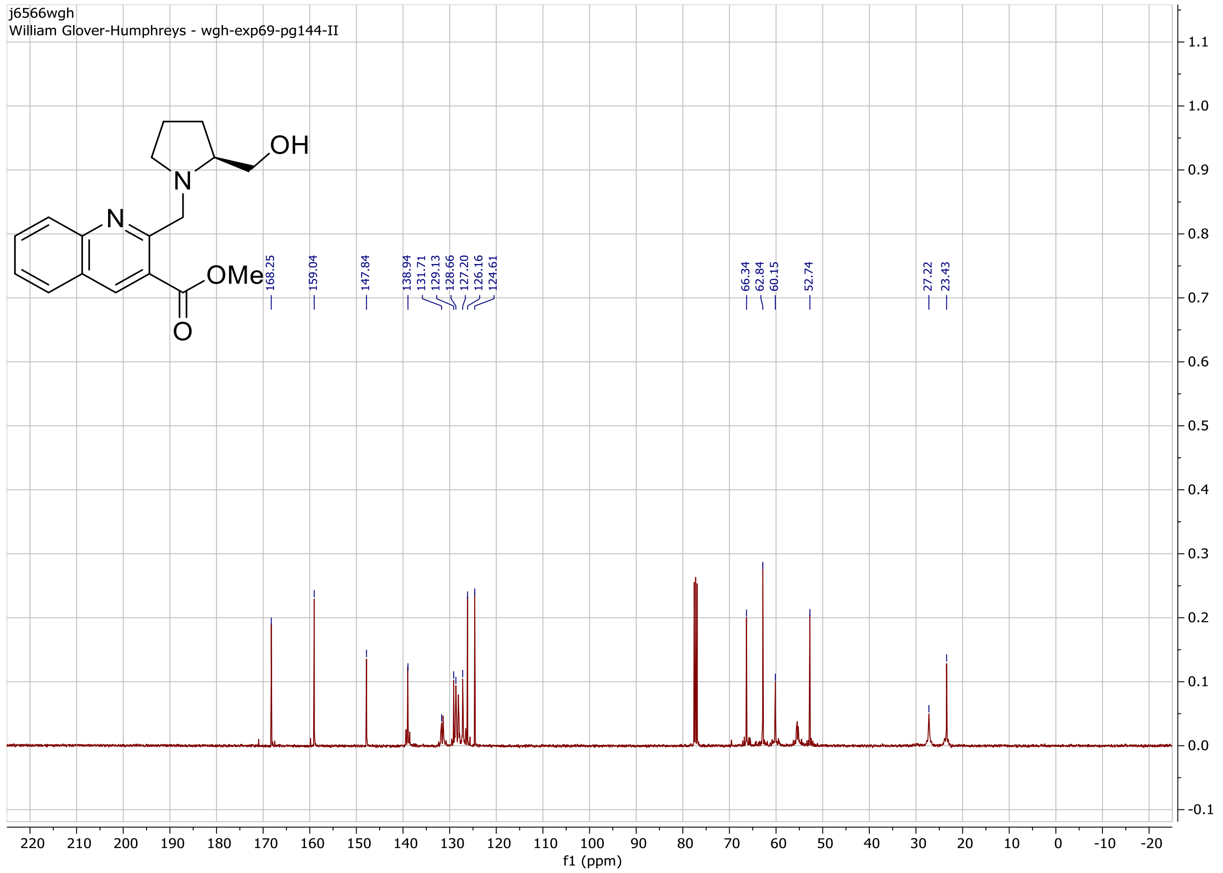
j6566wgh
William Glover-Humphreys - wgh-exp69-pg144-II
OH
N
N
OMe
O
168.25
159.04
147.84
138.94
131.71
129.13
128.66
127.20
126.16
124.61
66.34
62.84
60.15
52.74
27.22
23.43
f1 (ppm)

(S)-2,3,3a,4-Tetrahydro-1H-pyrrolo [2',1':3,4] [1,4] oxazocino [6,7-b] quinolin-6(13H)-one (59)

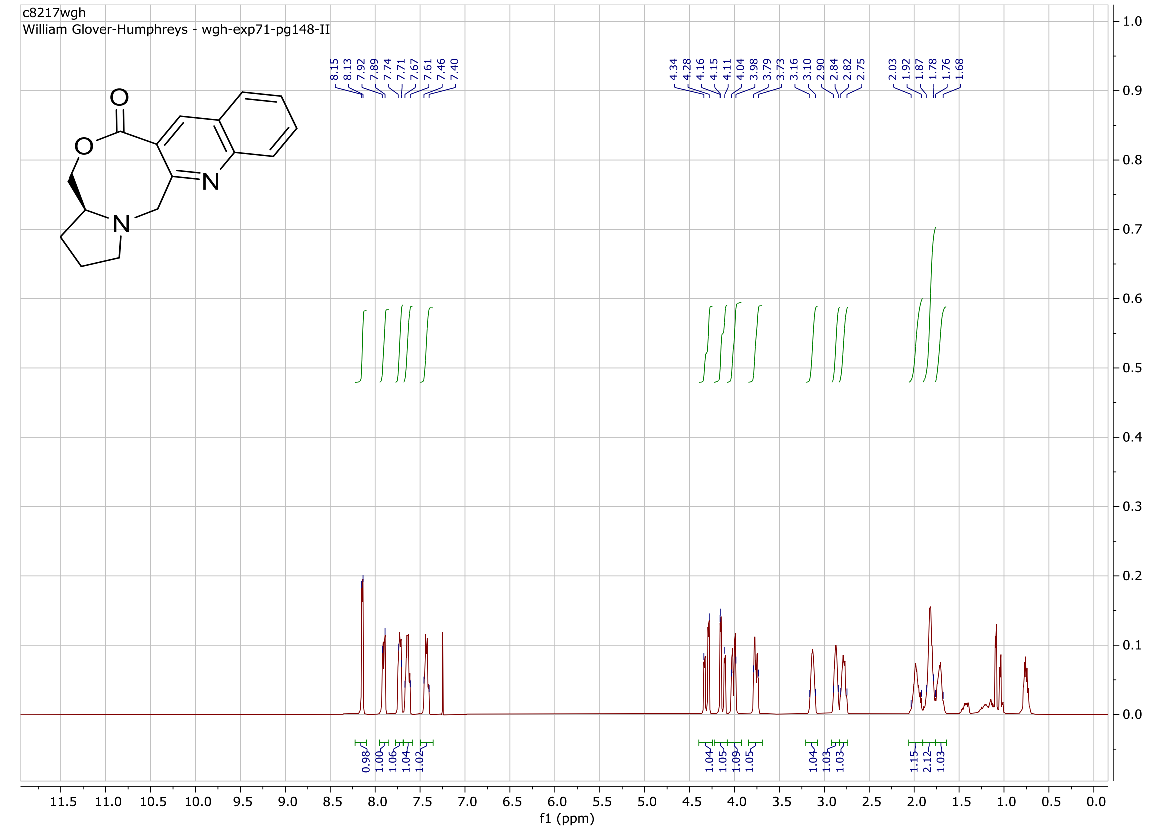

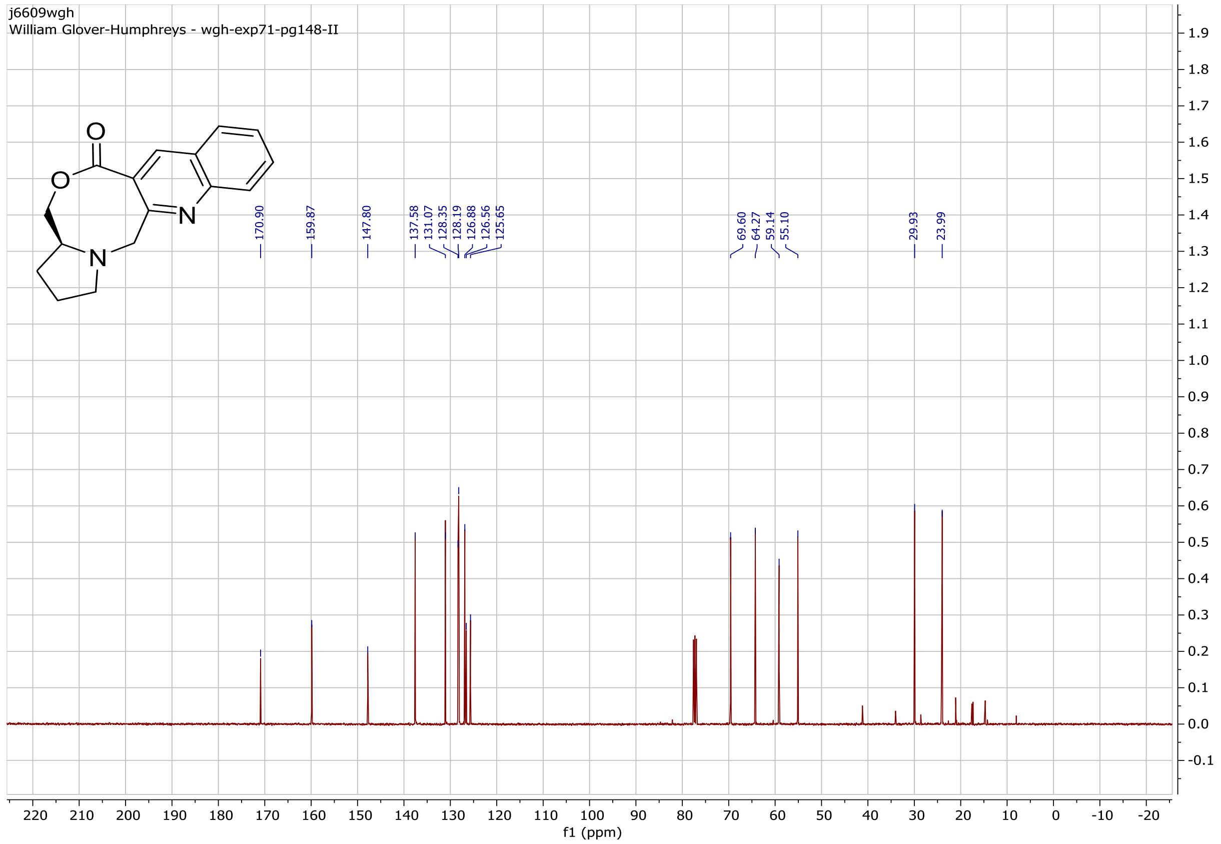

Methyl 2'-((2-hydroxyethyl)(methyl)amino)-[1,1'-biphenyl]-2-carboxylate (S62)

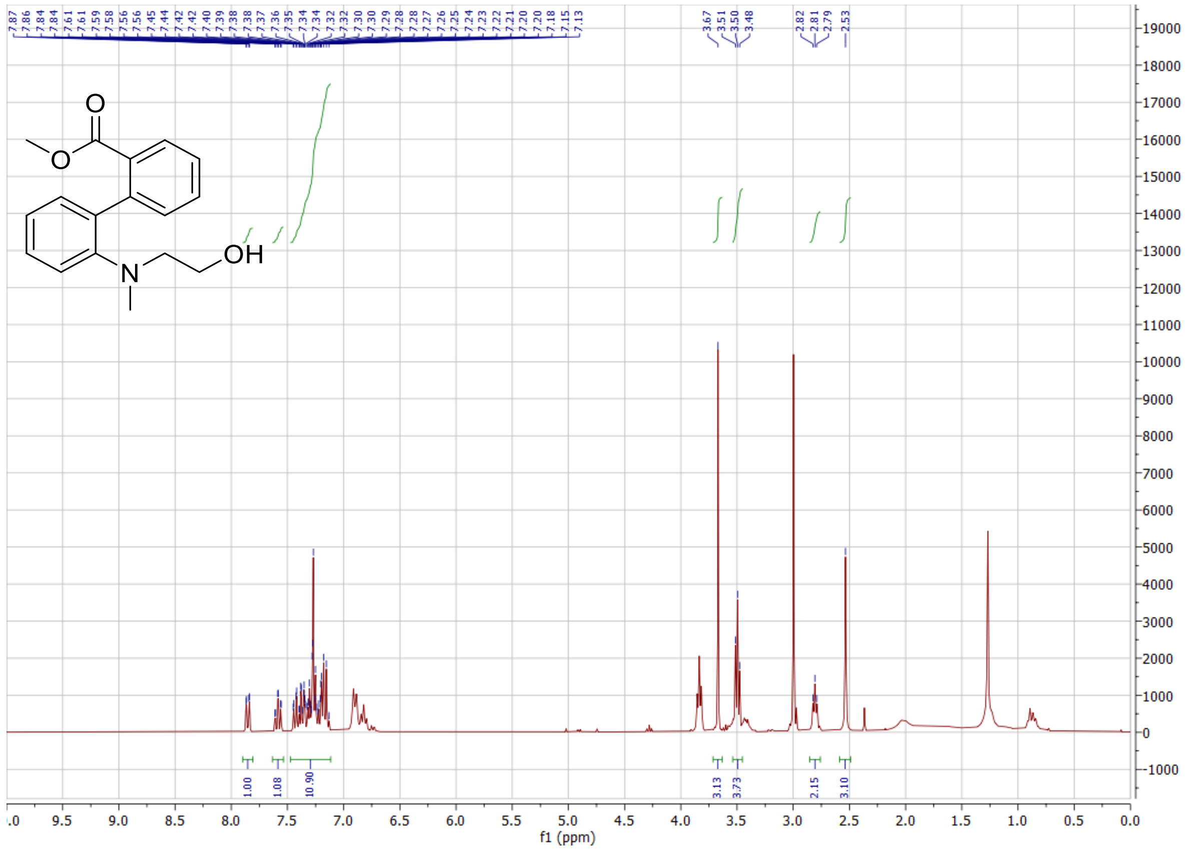

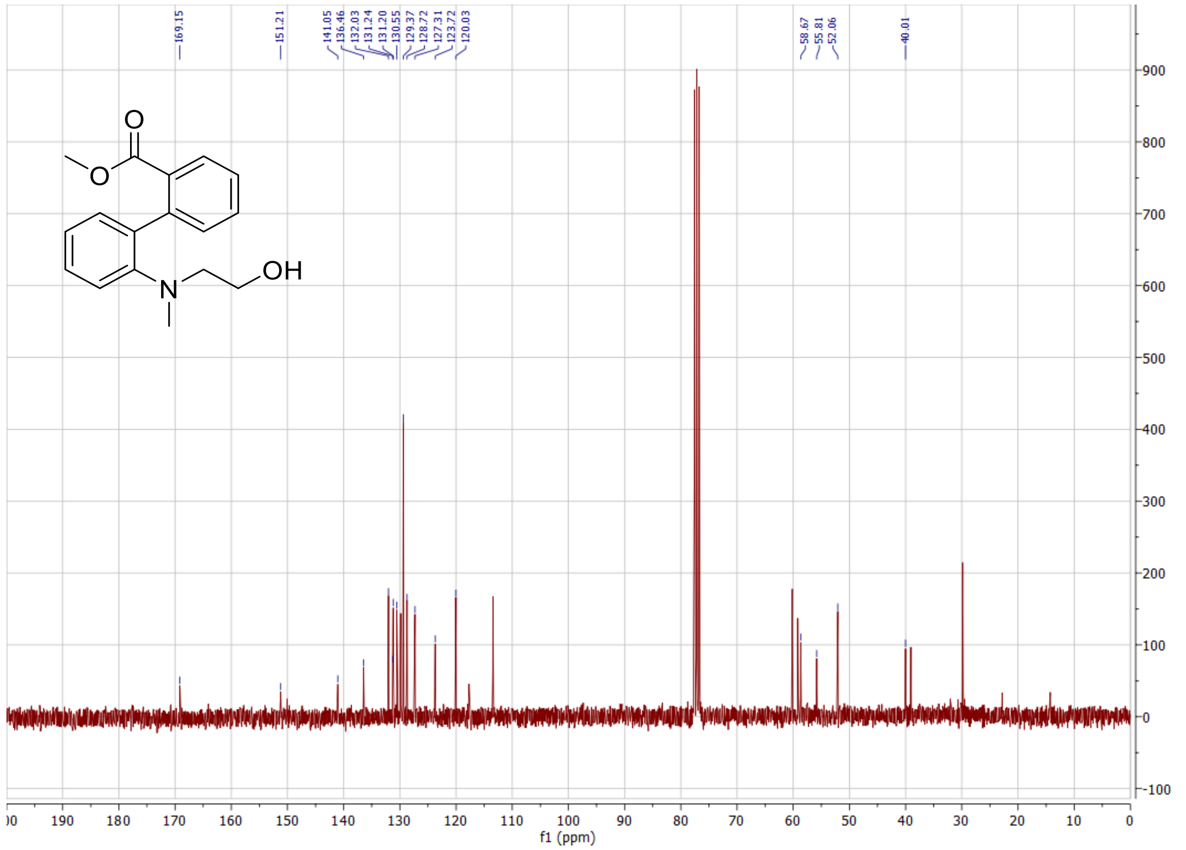

2'-((2-Hydroxyethyl)(methyl)amino)-[1,1'-biphenyl]-2-carboxylic acid (S63)

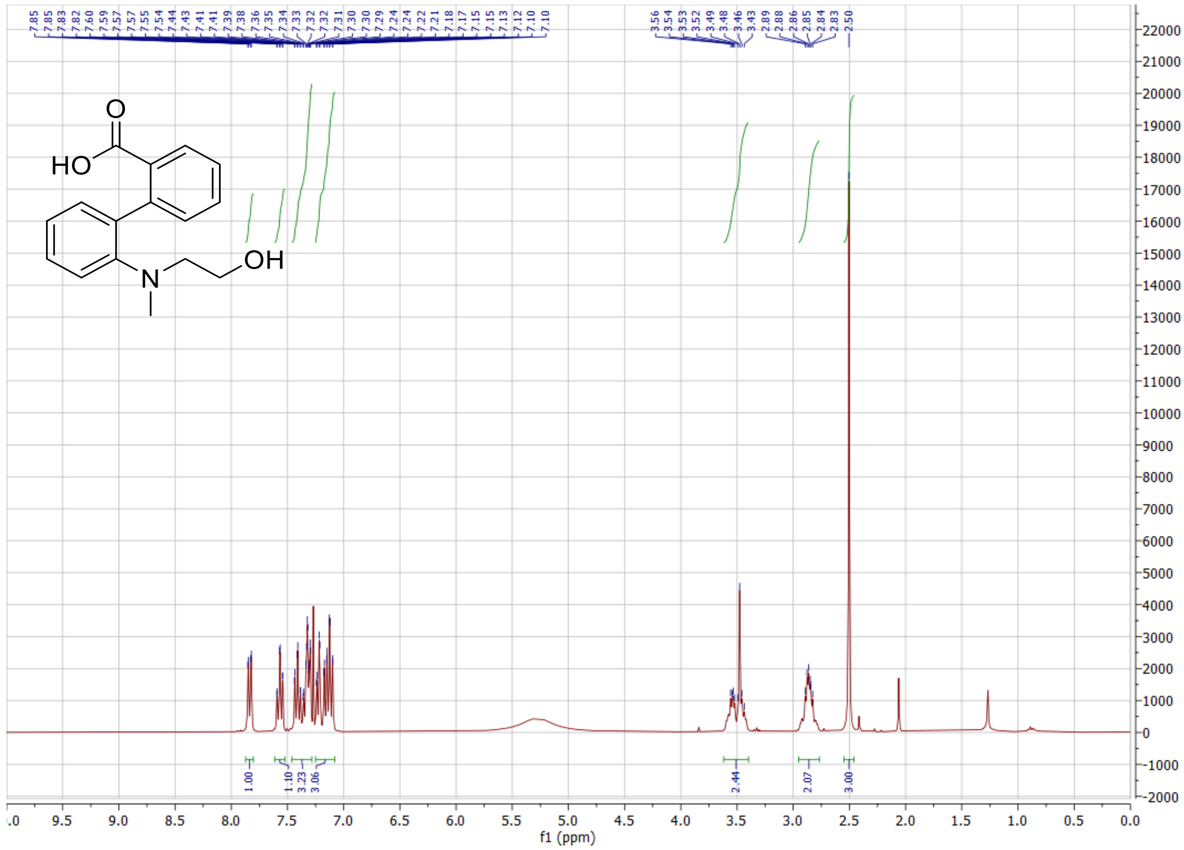

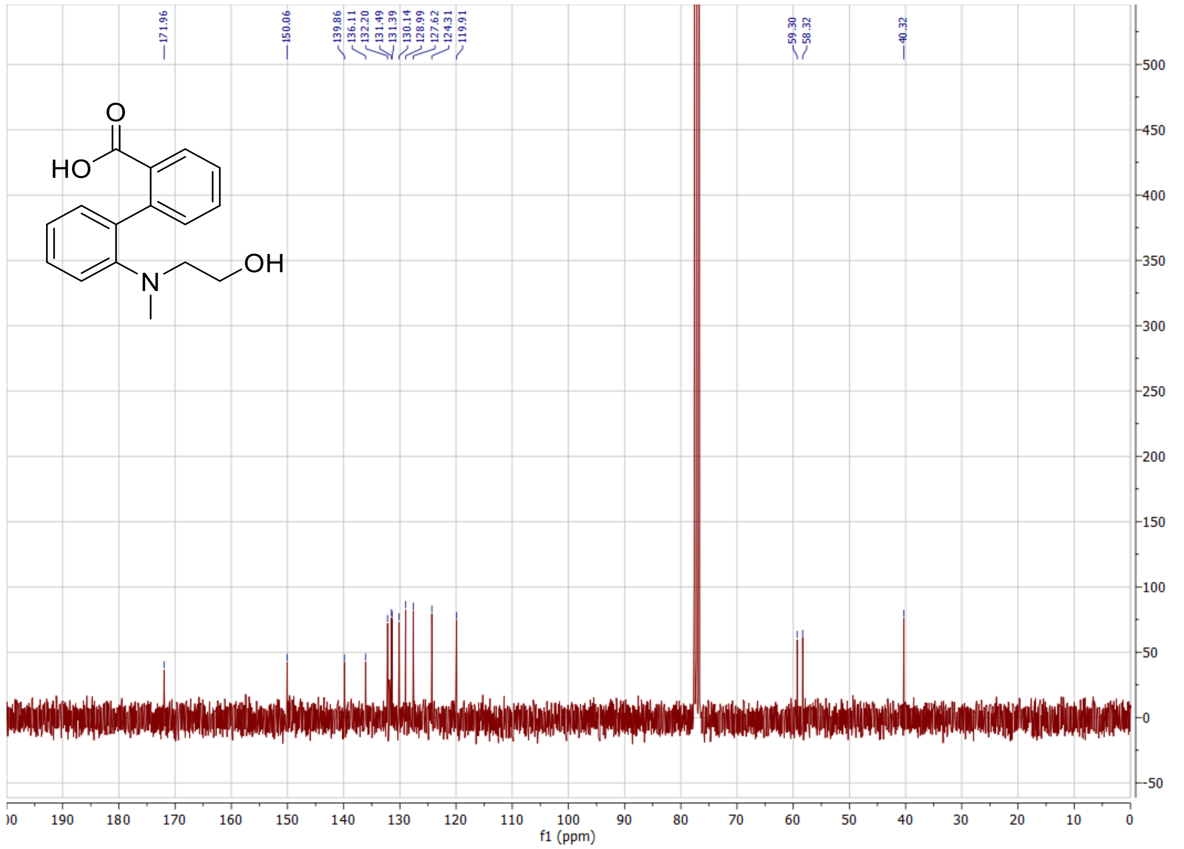

5-Methyl-6,7-dihydrodibenzo[*e*,*g*][1,4]oxazonin-9(5*H*)-one (60)

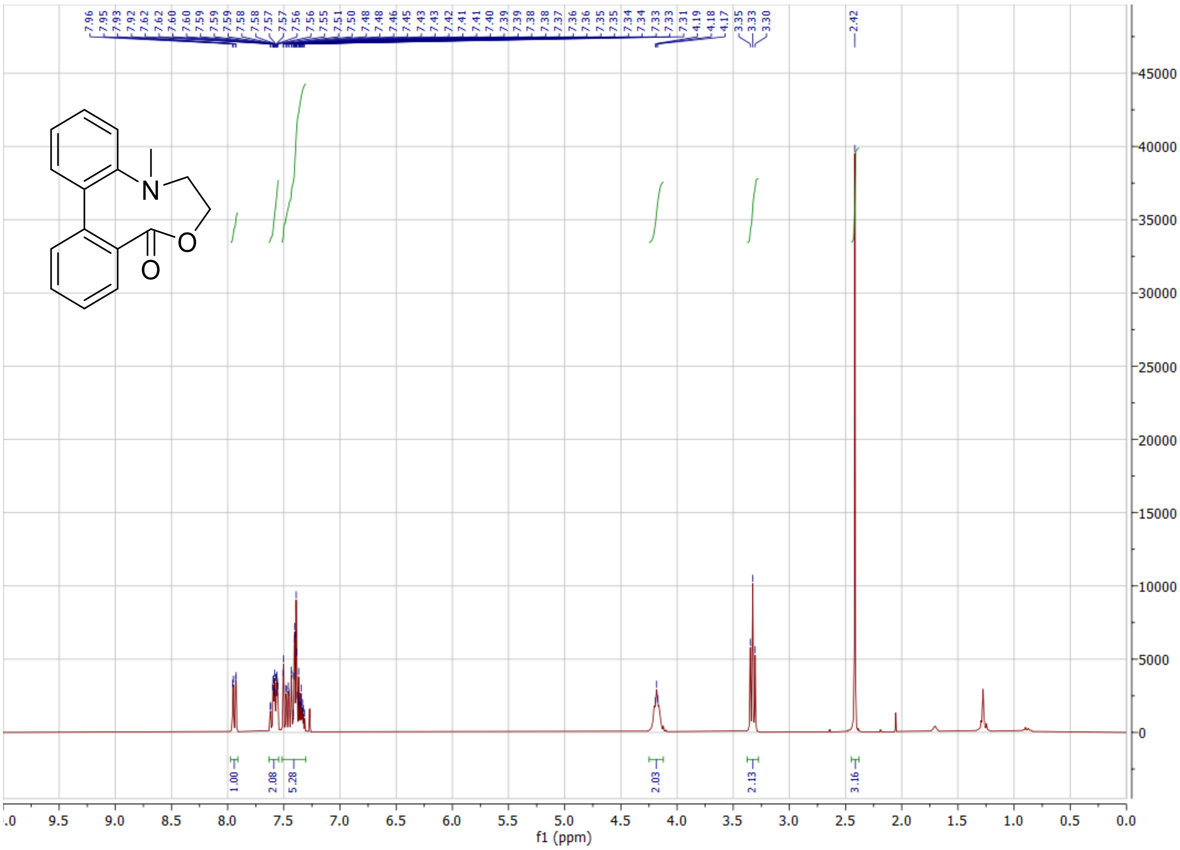

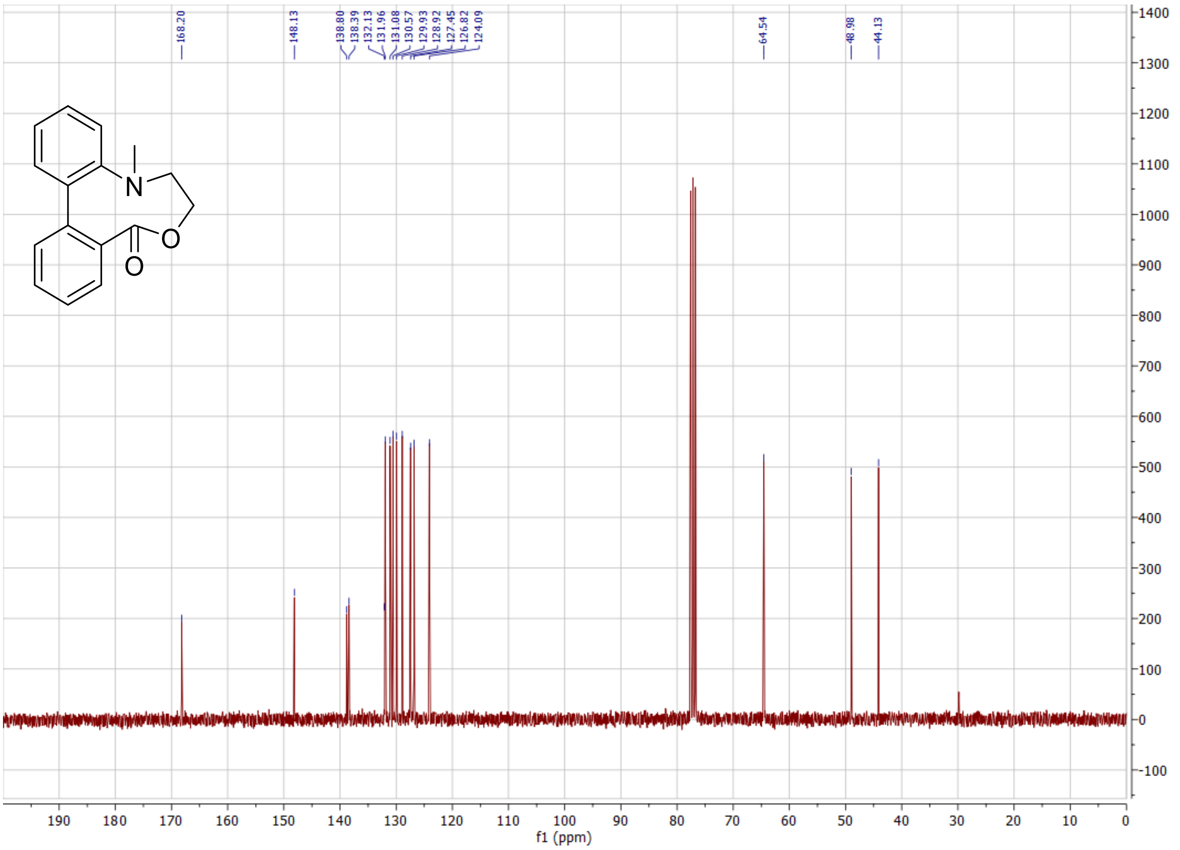

N-(3-Hydroxypropyl)-2-bromoaniline (S64)

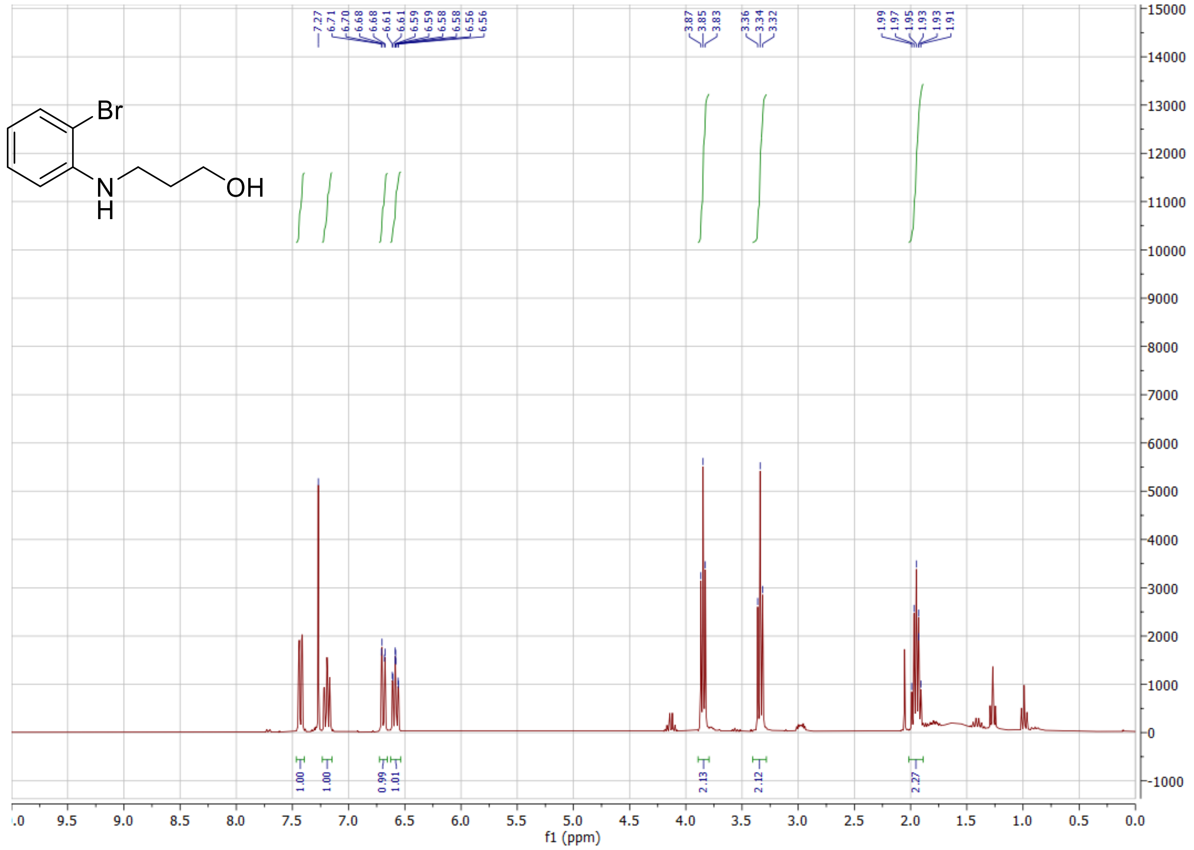

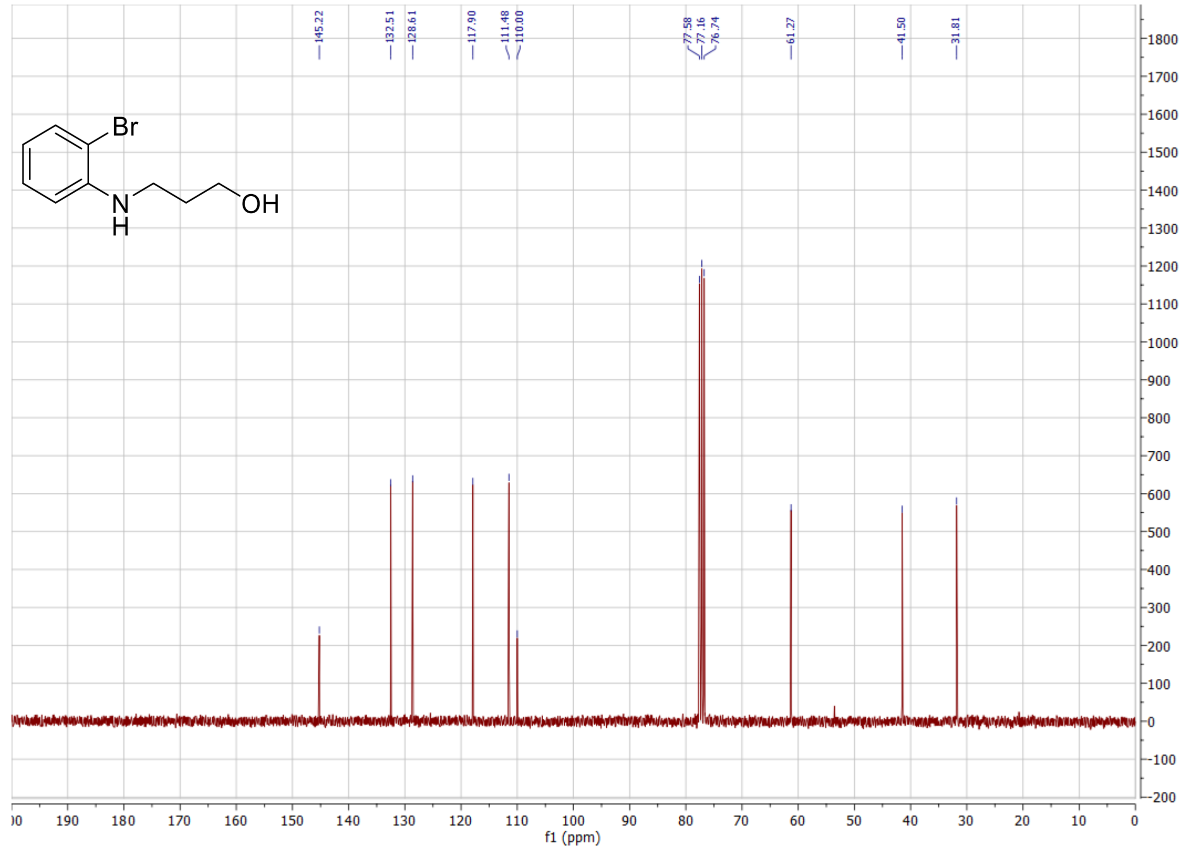

_N_-(3-Hydroxypropyl)-_N_-methyl-2-bromoaniline (S65)

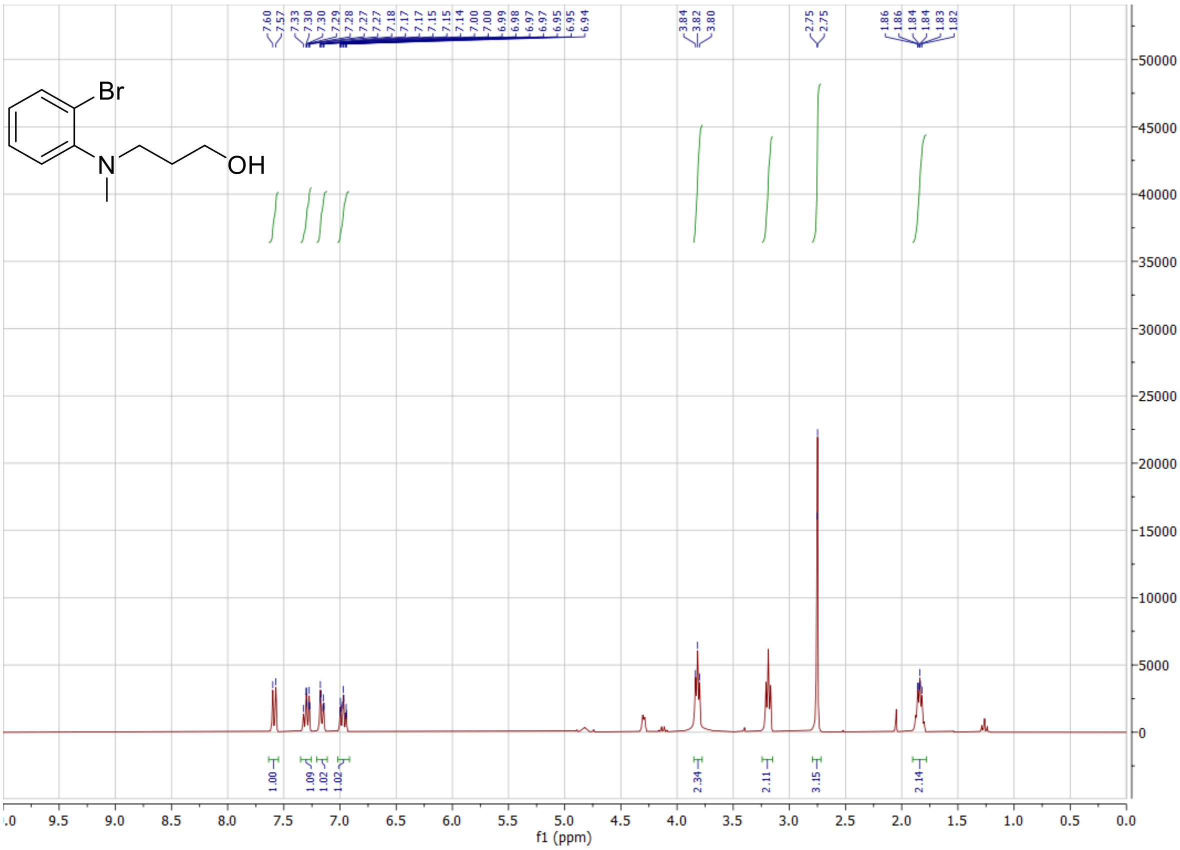

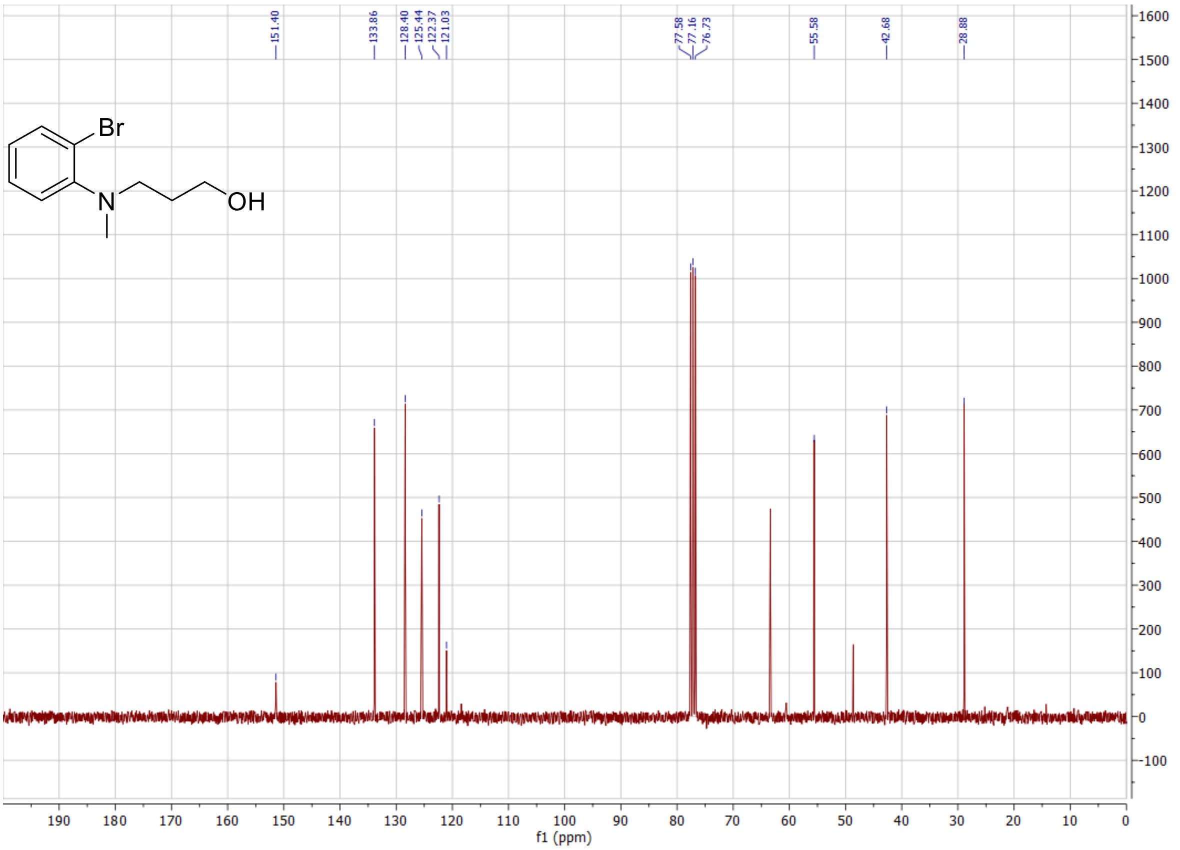

Methyl 2'-((3-hydroxypropyl)(methyl)amino)-[1,1'-biphenyl]-2-carboxylate (S66)

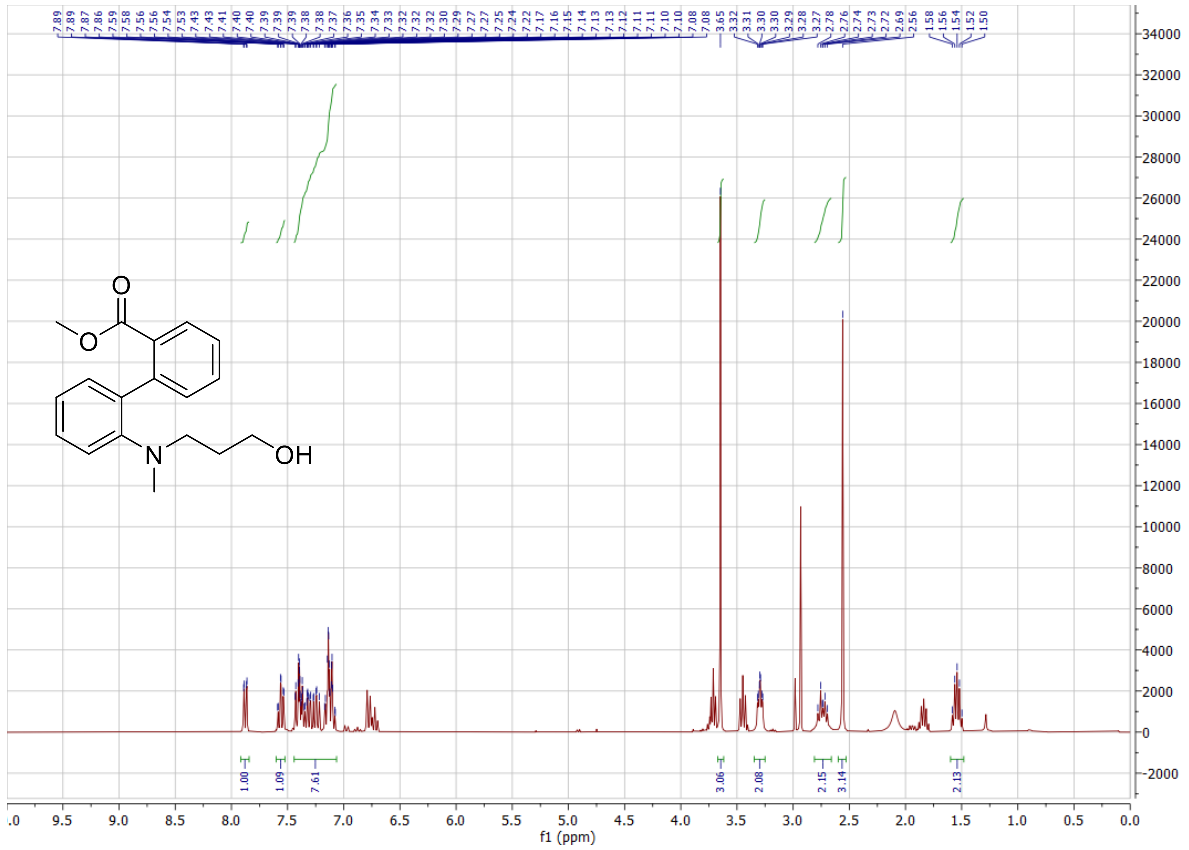

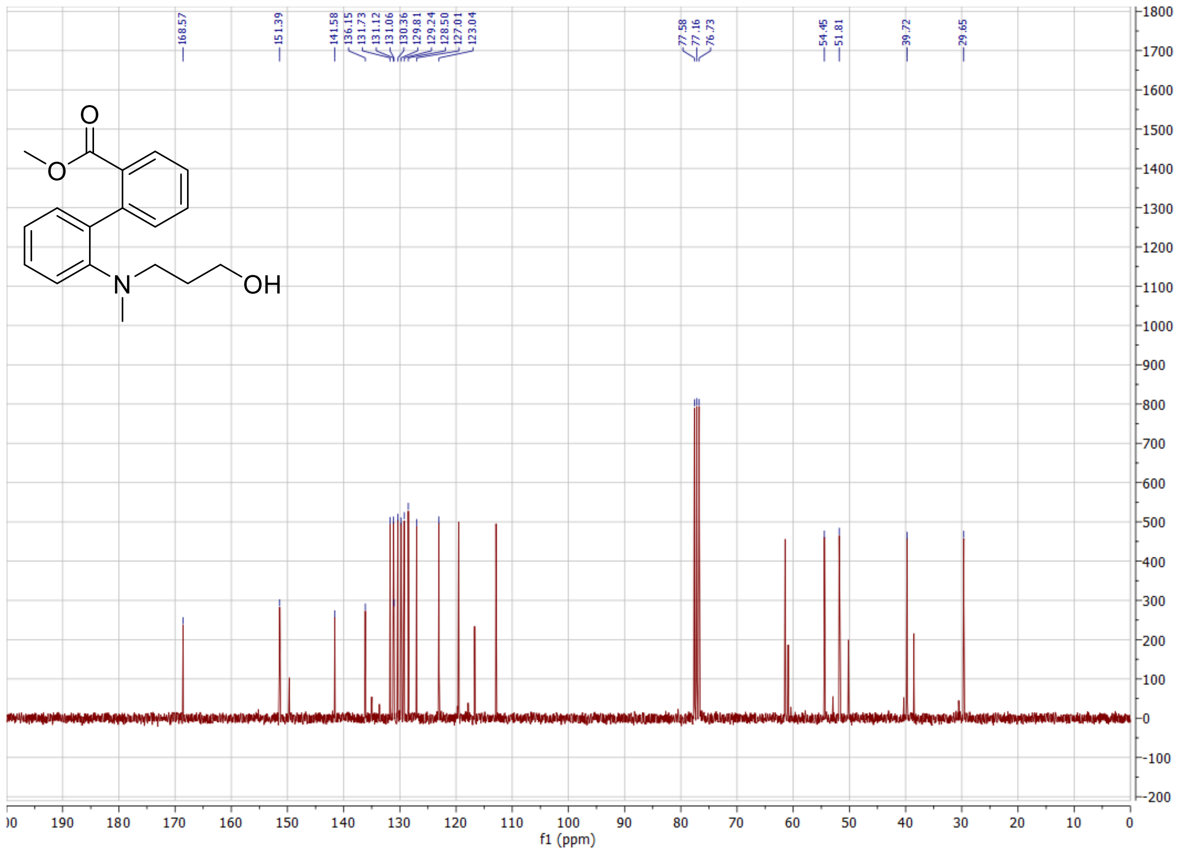

2'-((3-Hydroxypropyl)(methyl)amino)-[1,1'-biphenyl]-2-carboxylic acid (S67)

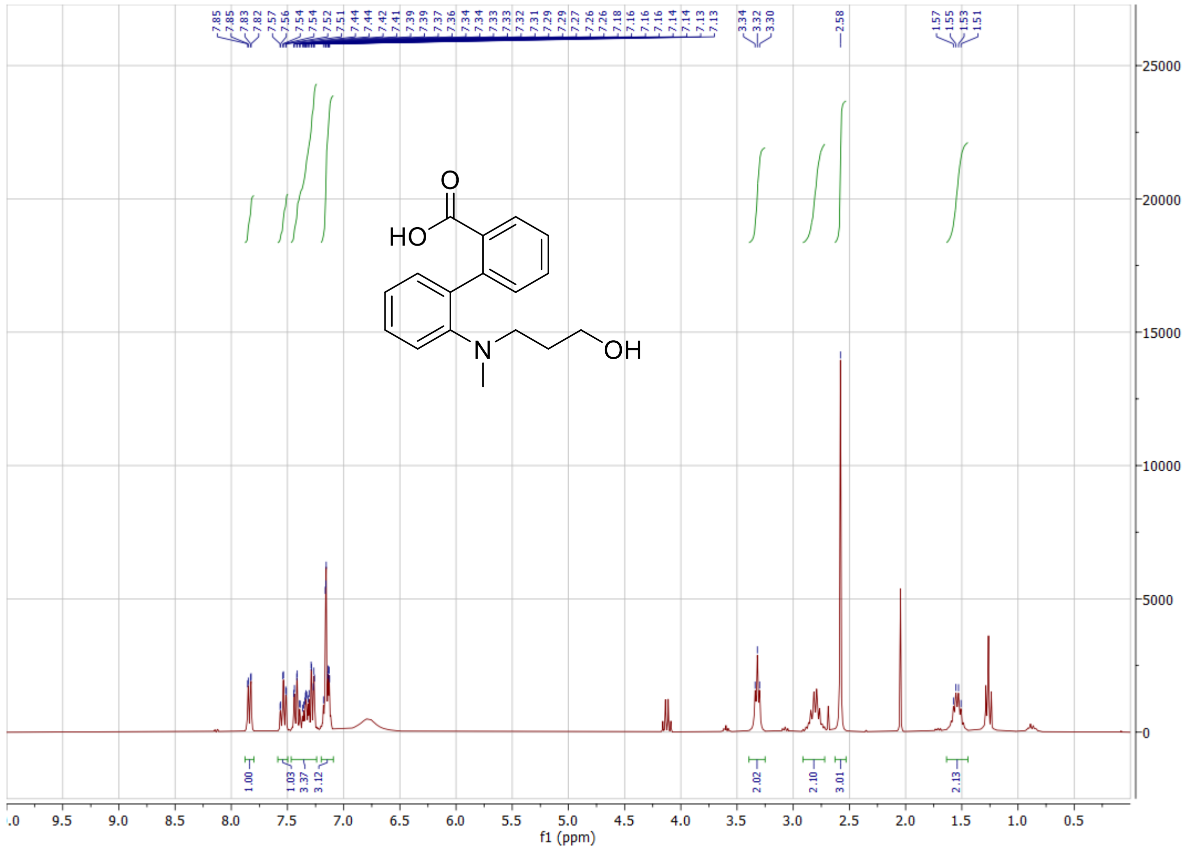

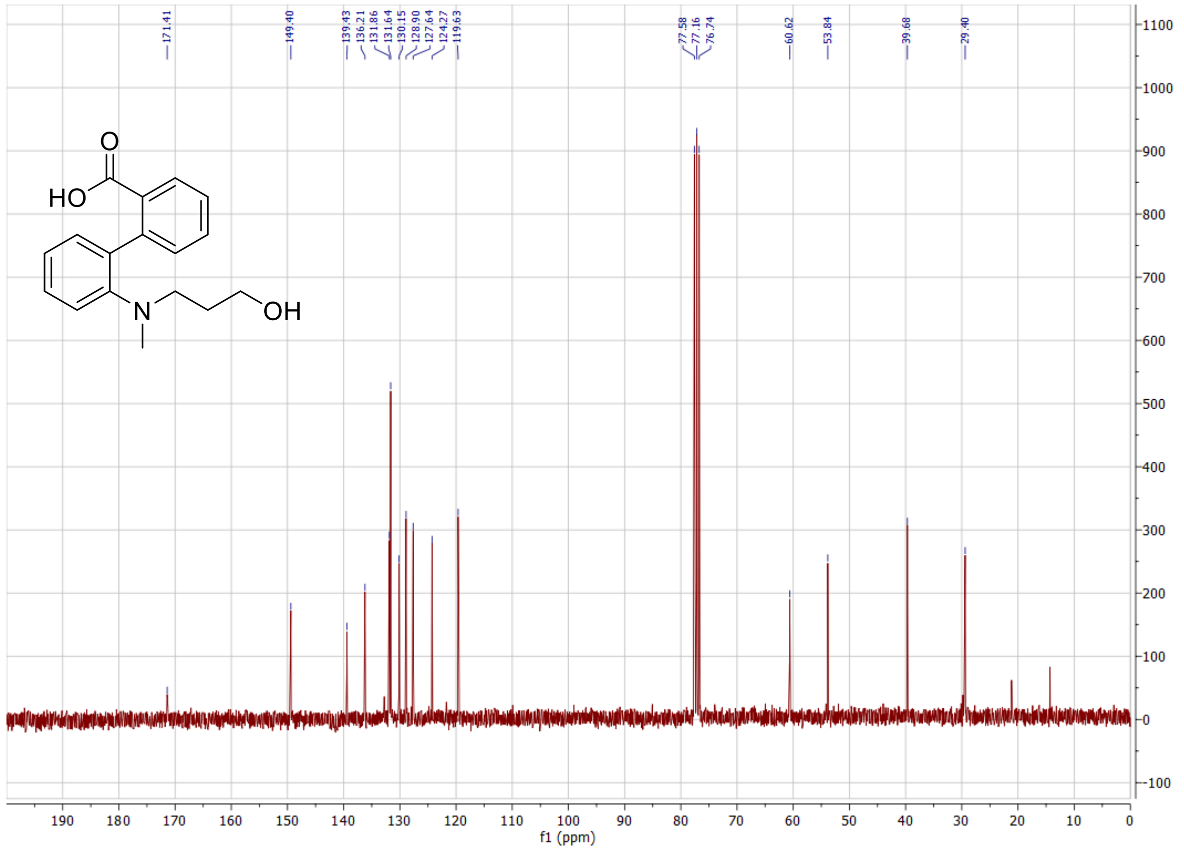

5-Methyl-5,6,7,8-tetrahydro-10*H*-dibenzo[*f*,*h*][1,5]oxazecin-10-one (61)

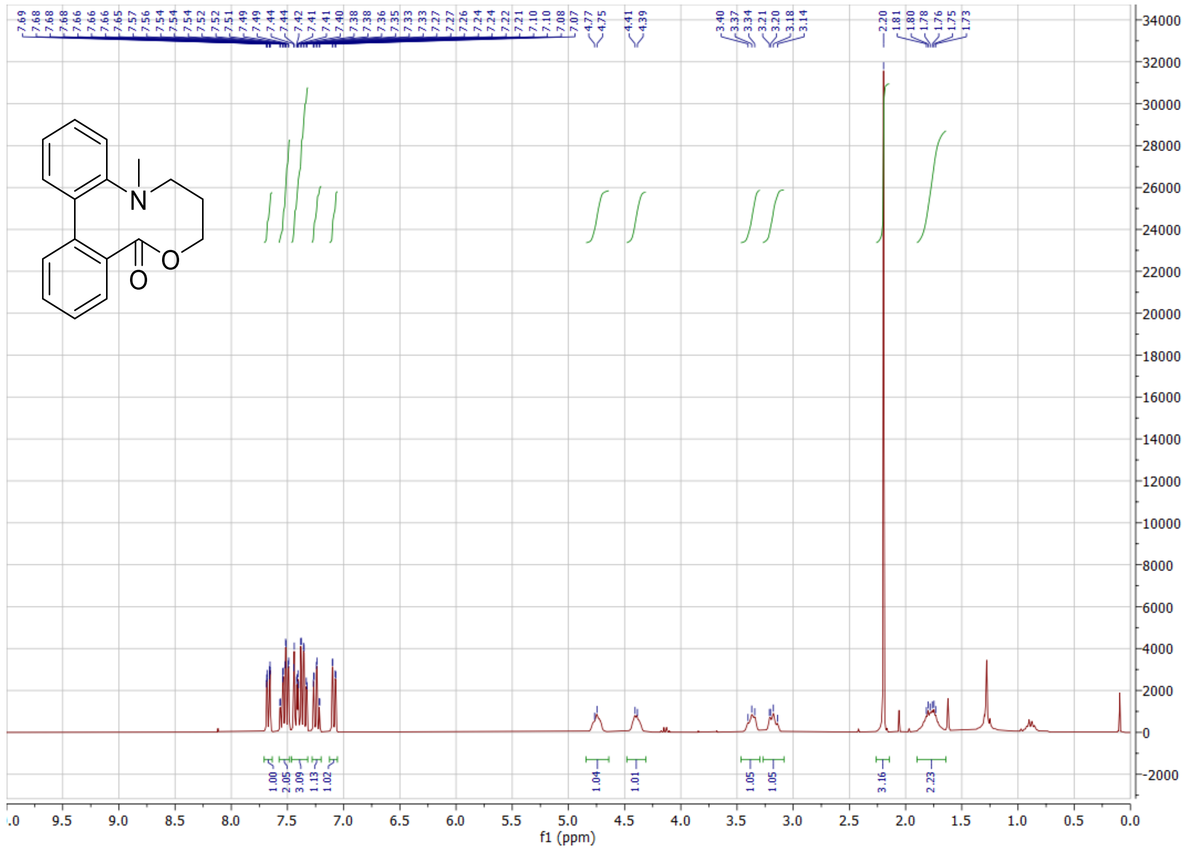

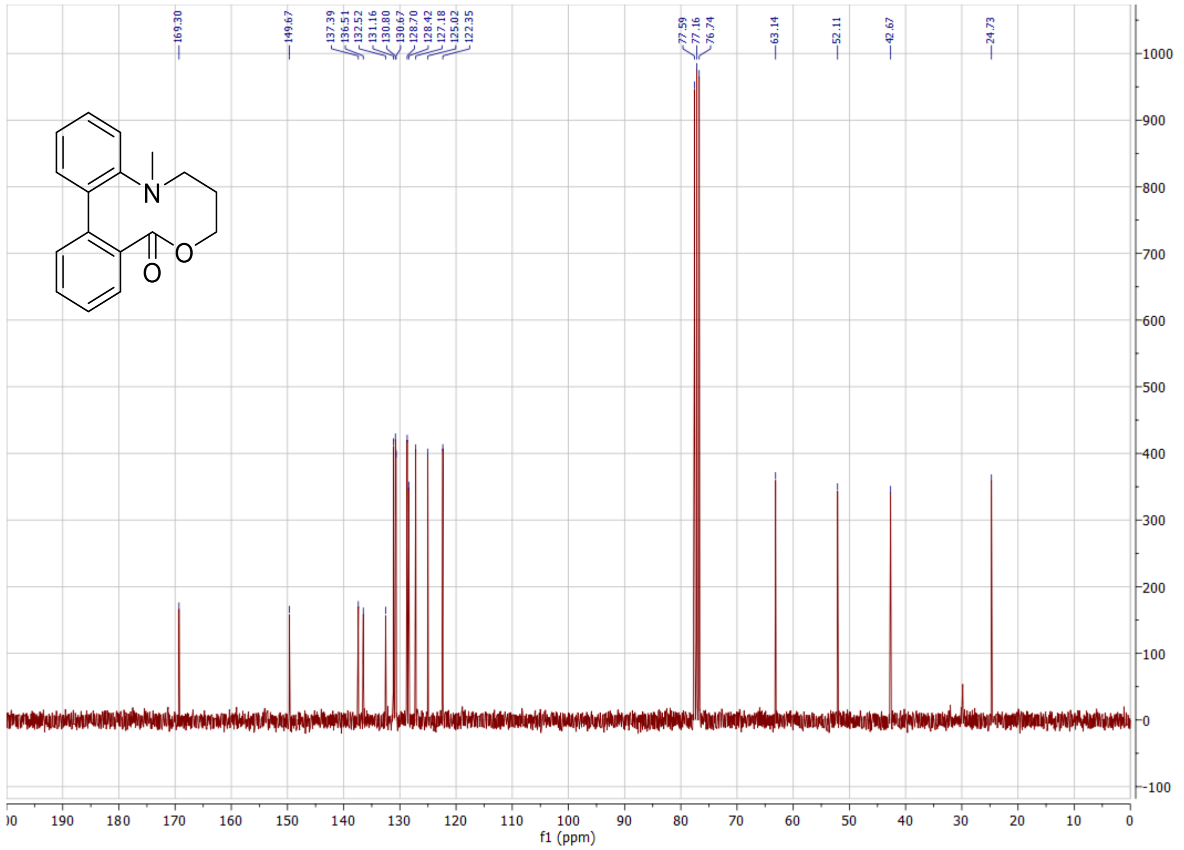

2'-((2-Hydroxypropyl)(methyl)amino)-[1,1'-biphenyl]-2-carboxylic acid (S69)

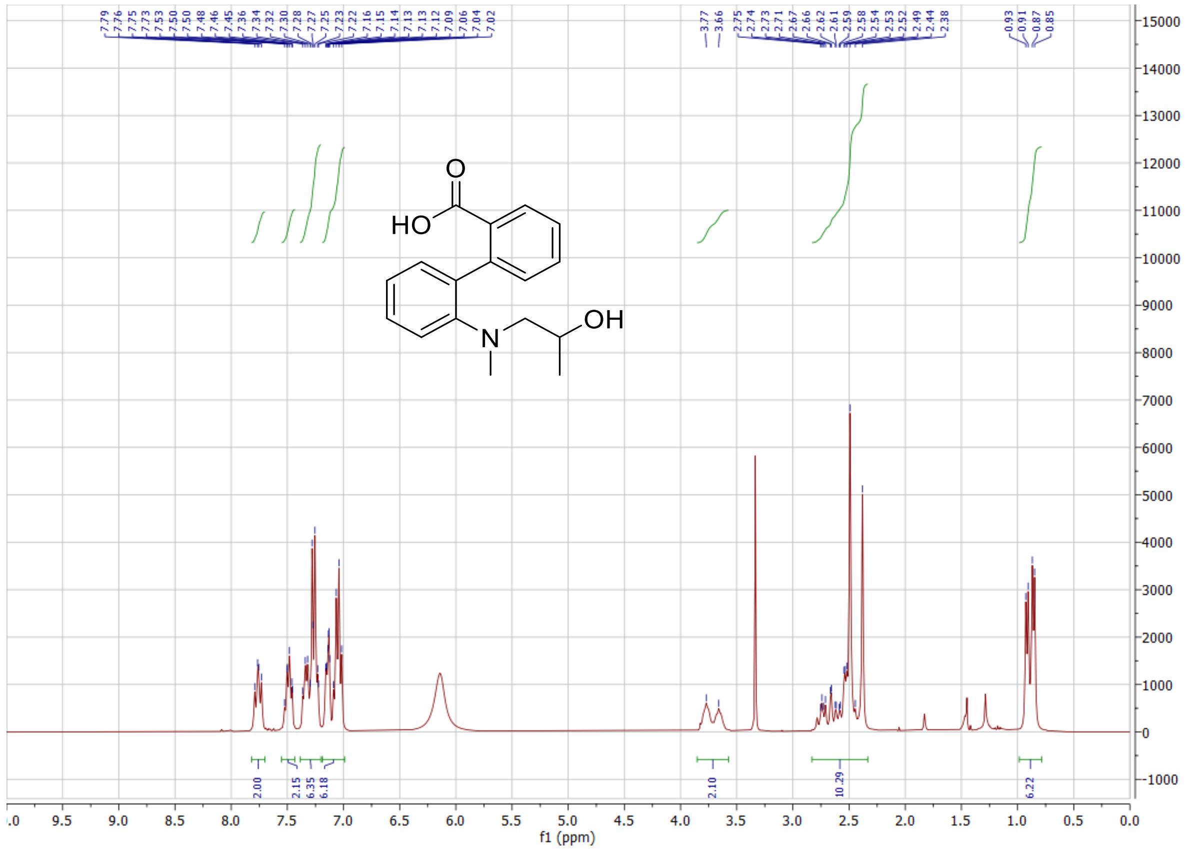

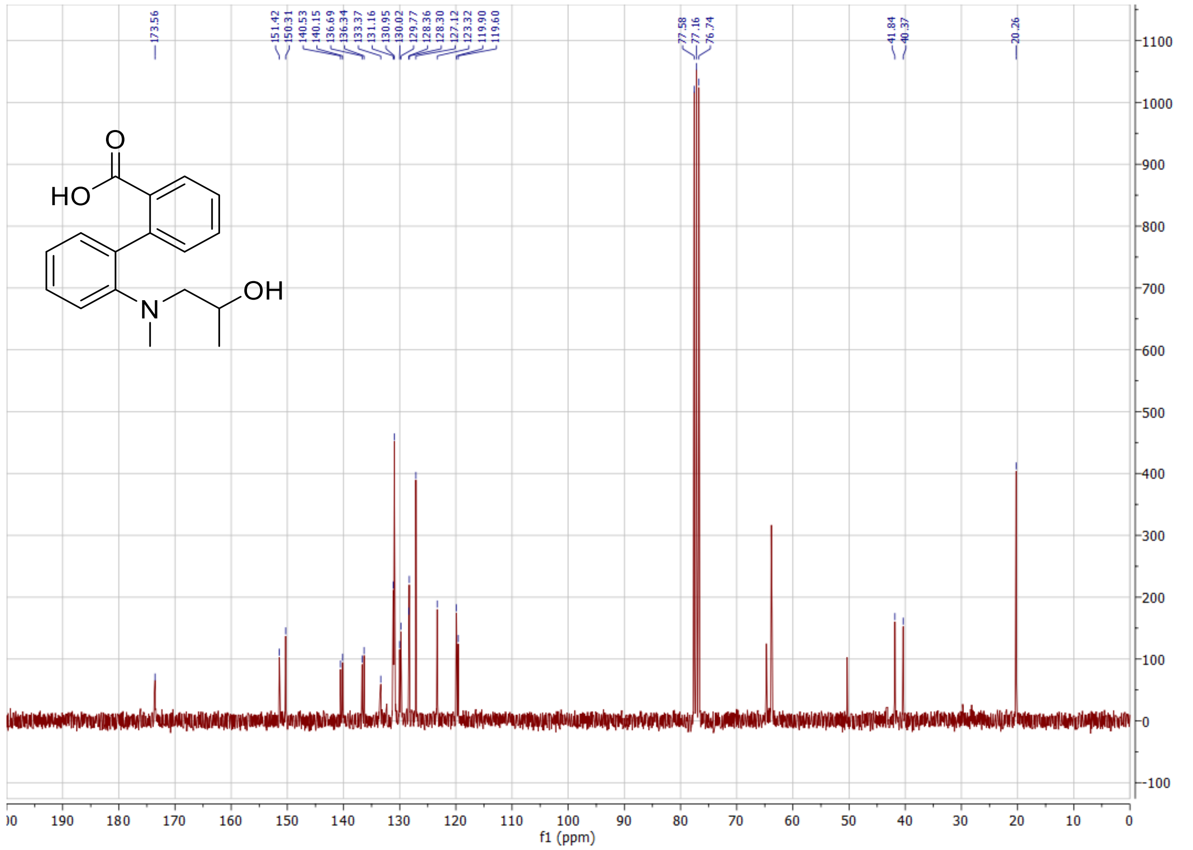

5,7-Dimethyl-6,7-dihydrodibenzo[e,g][1,4]oxazonin-9(5H)-one (62)

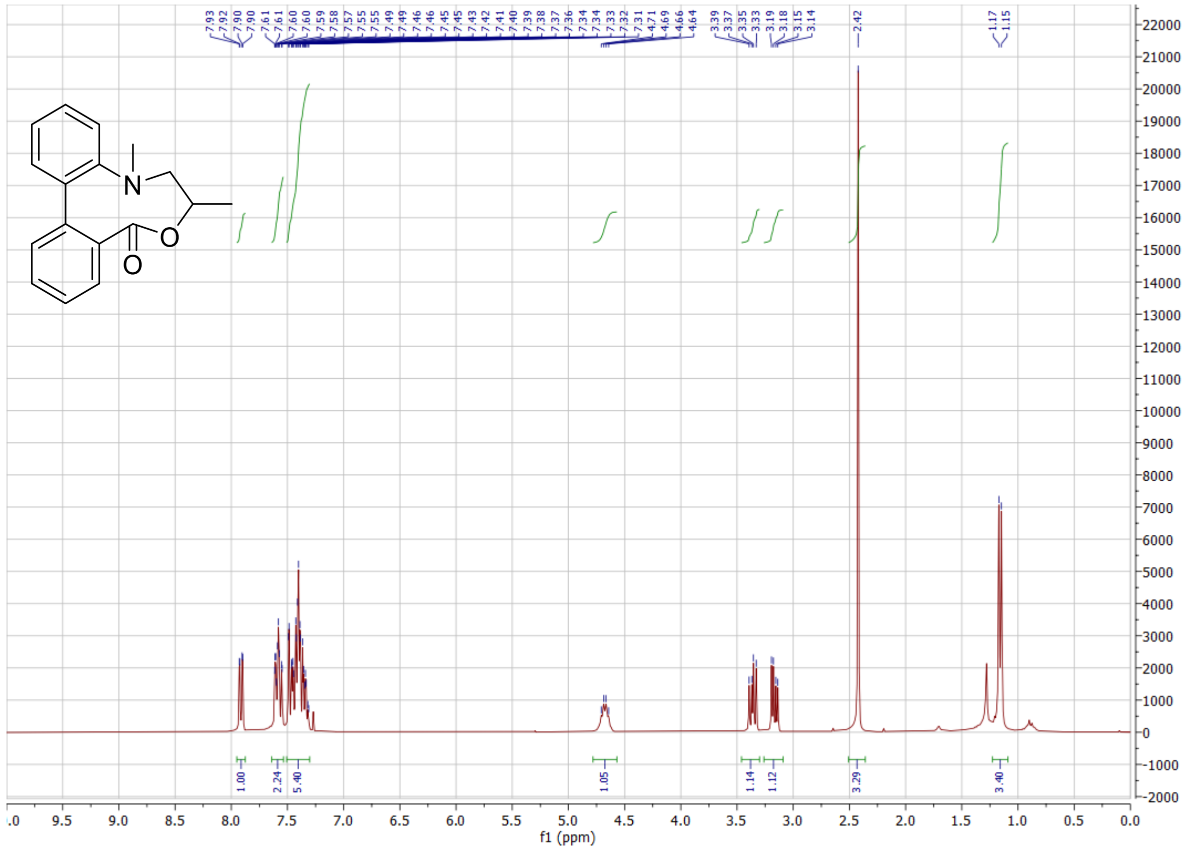

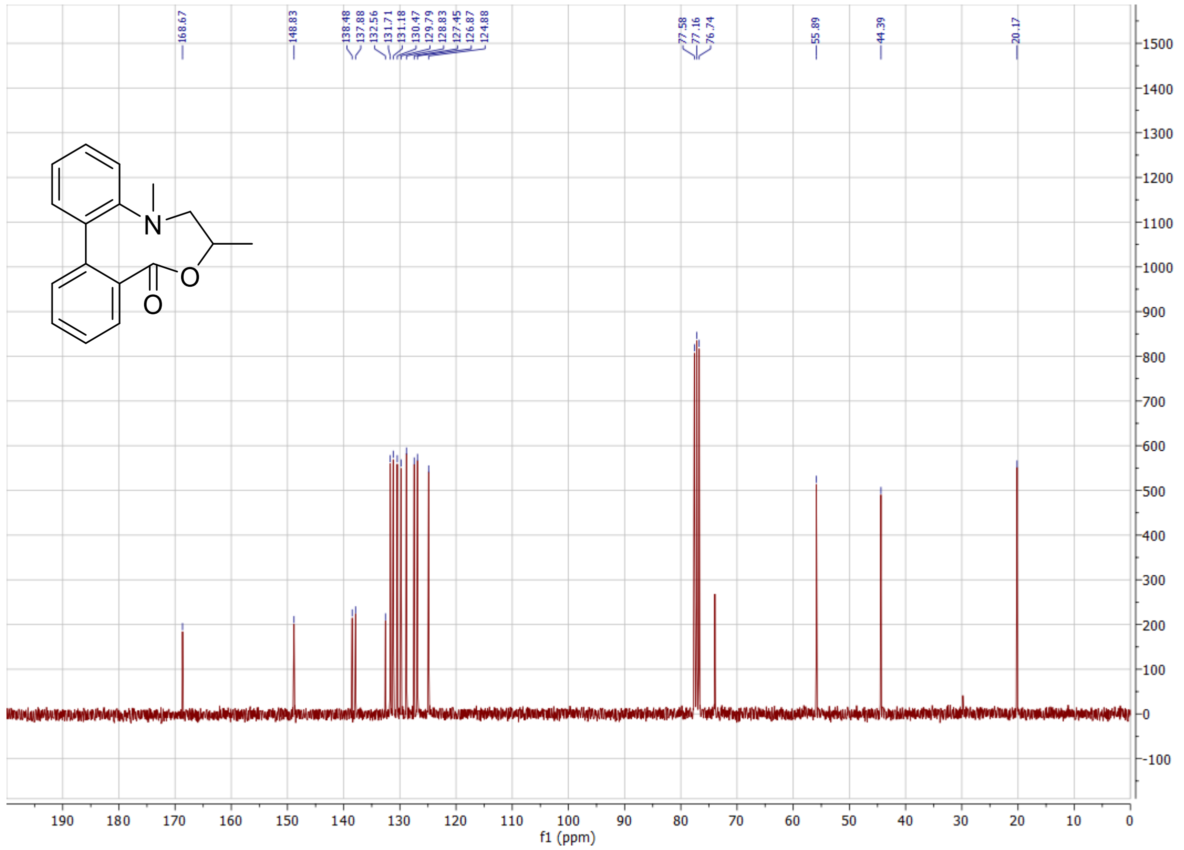

2'-((3-Hydroxybutyl)(methyl)amino)-[1,1'-biphenyl]-2-carboxylic acid (S71)

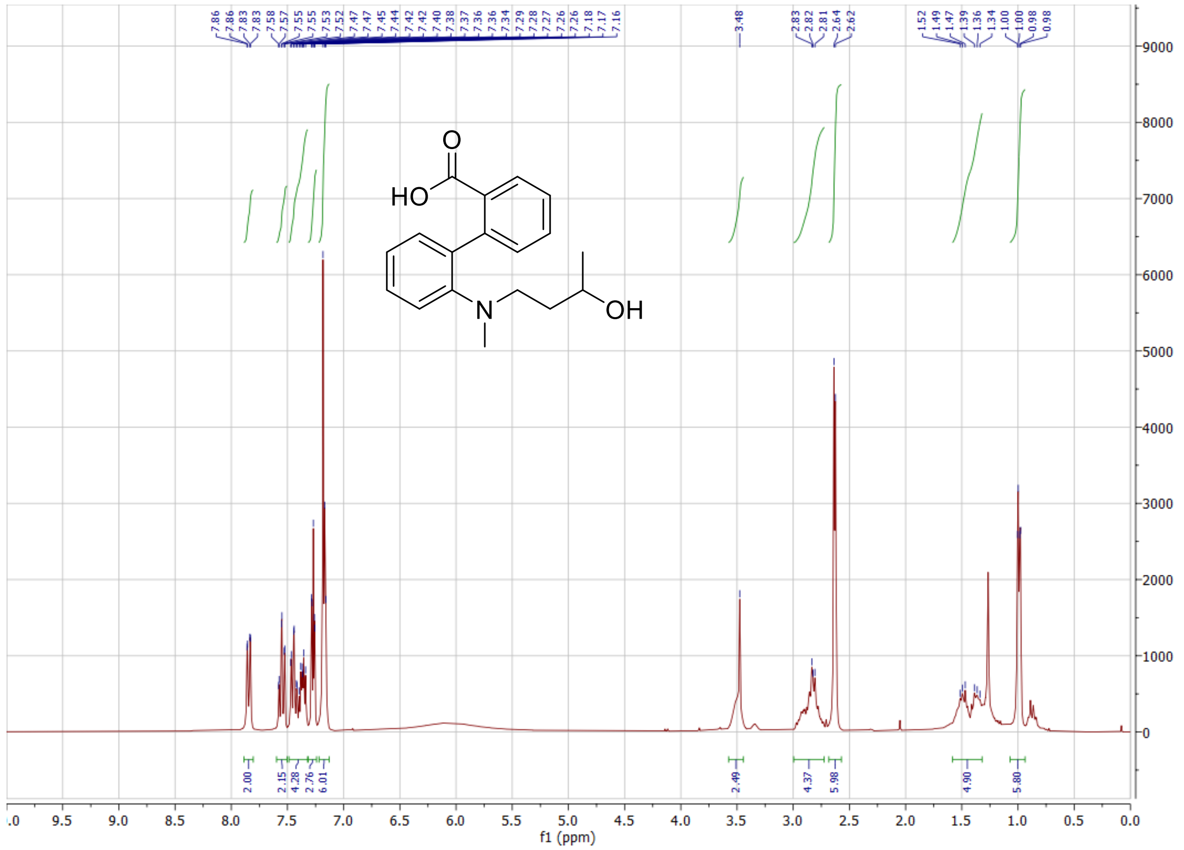

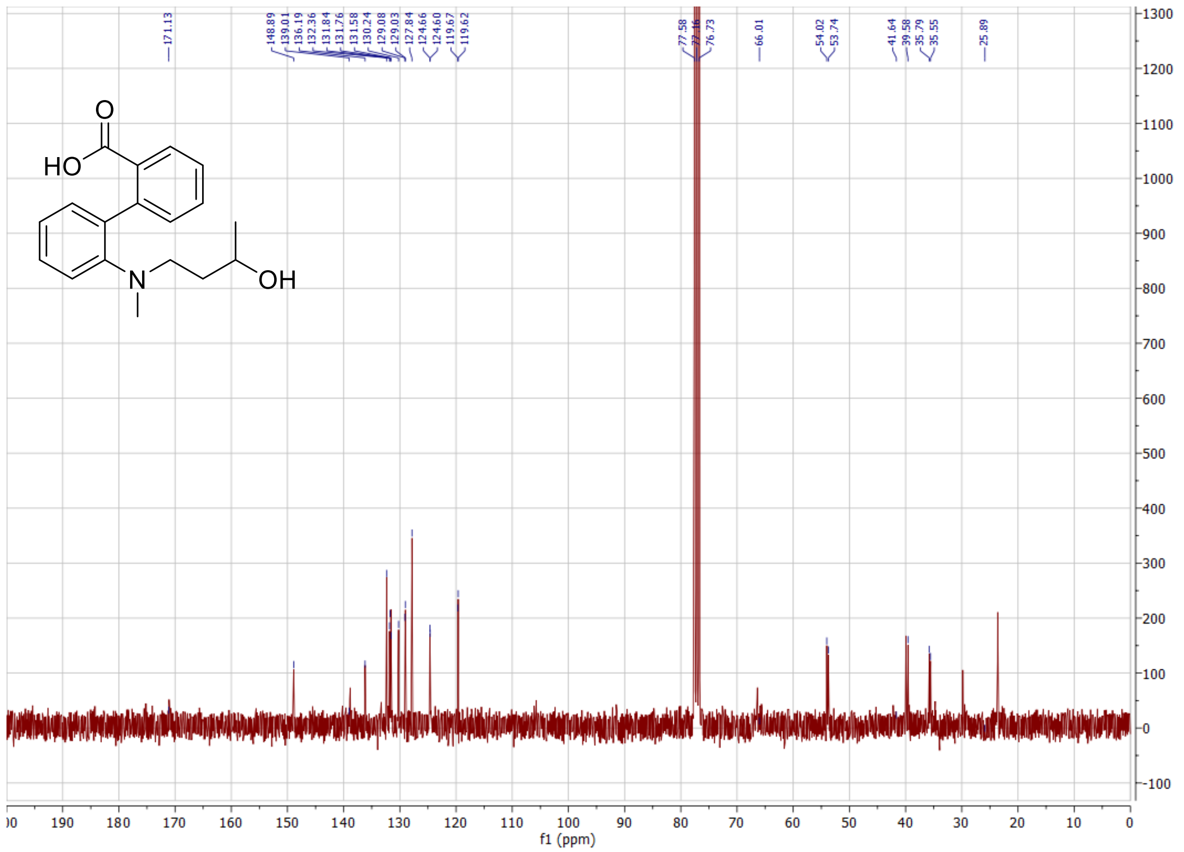

5,8-Dimethyl-5,6,7,8-tetrahydro-10H-dibenzo[f,h][1,5]oxazecin-10-one (63)

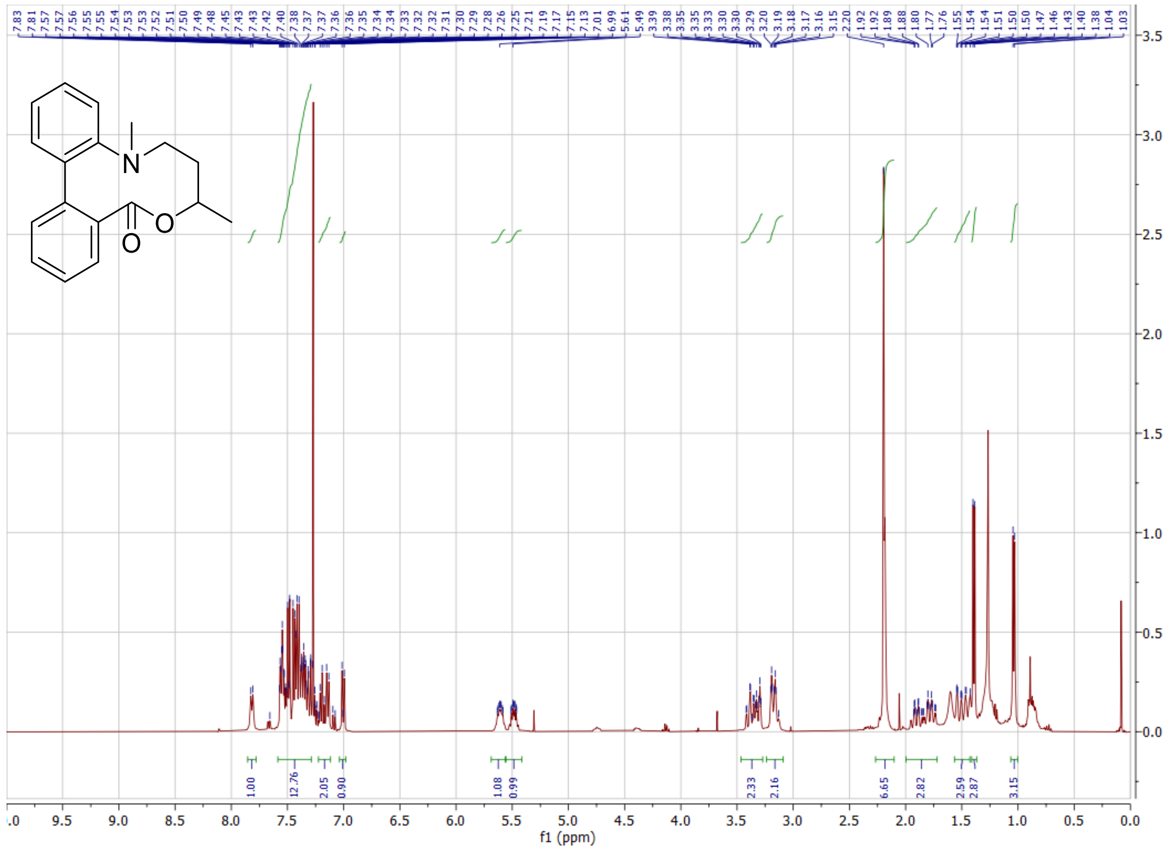

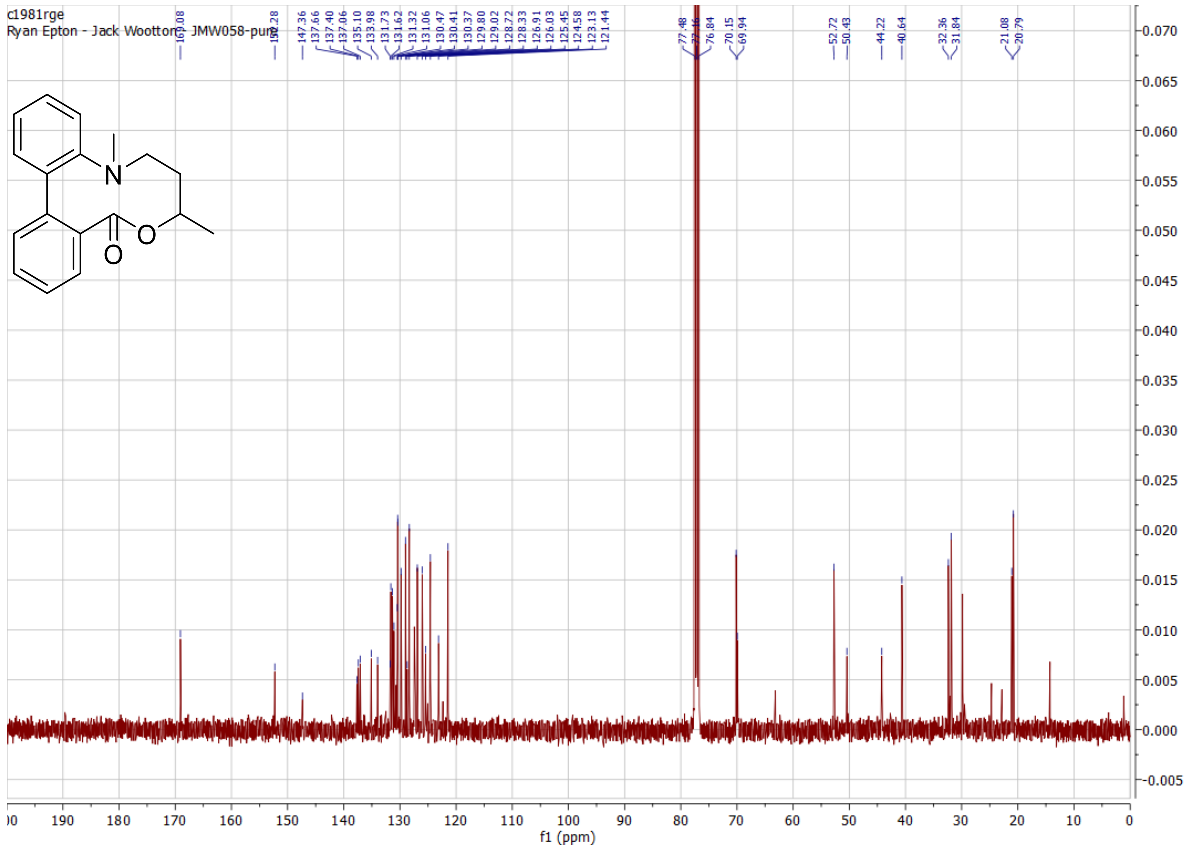

N-(1-(6-bromopyridin-2-yl)propan-2-yl)prop-2-en-1-amine (S72)

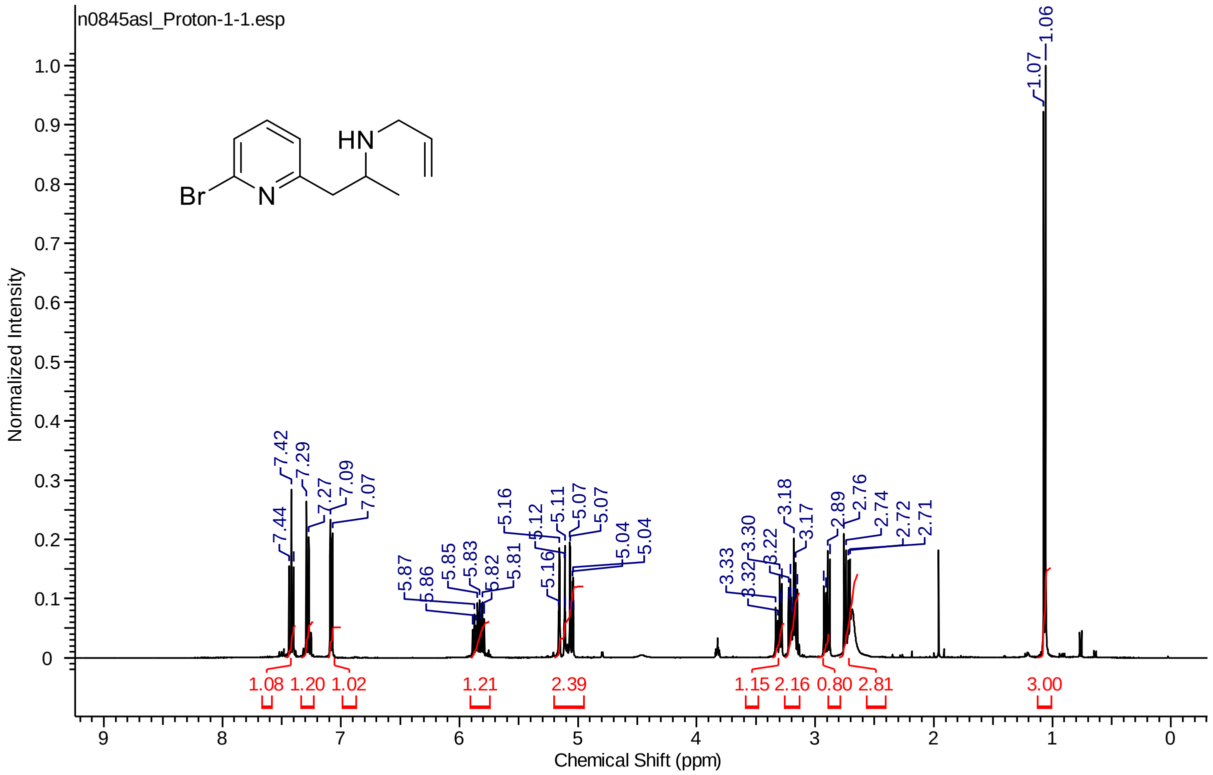

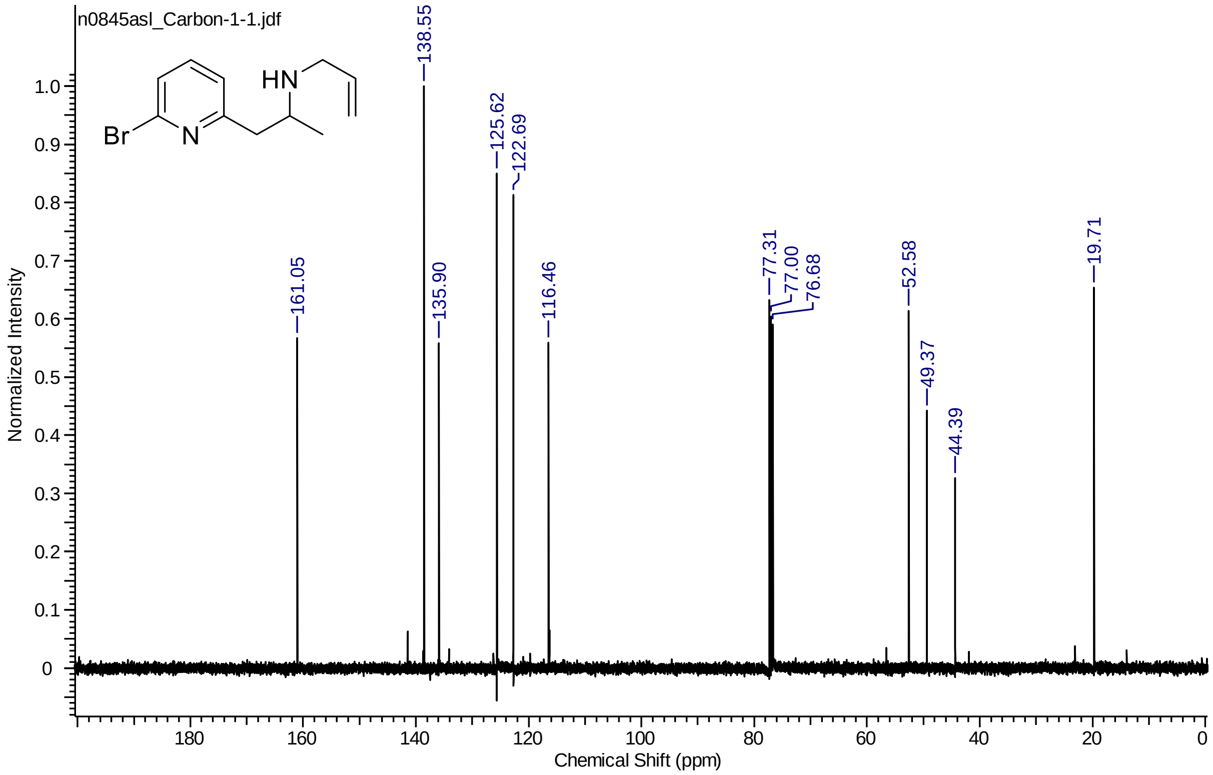

Methyl 2-(2-(6-(2-(allylamino)propyl)pyridin-2-yl)phenyl)acetate (S73)

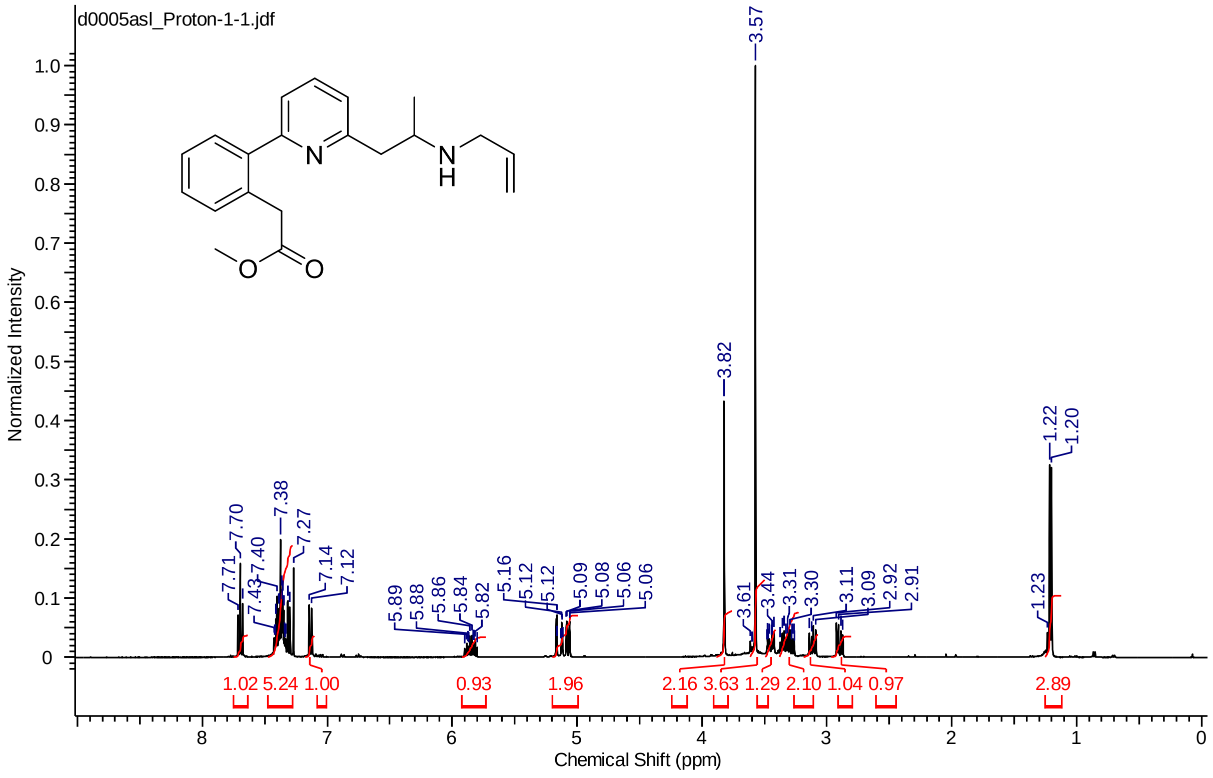

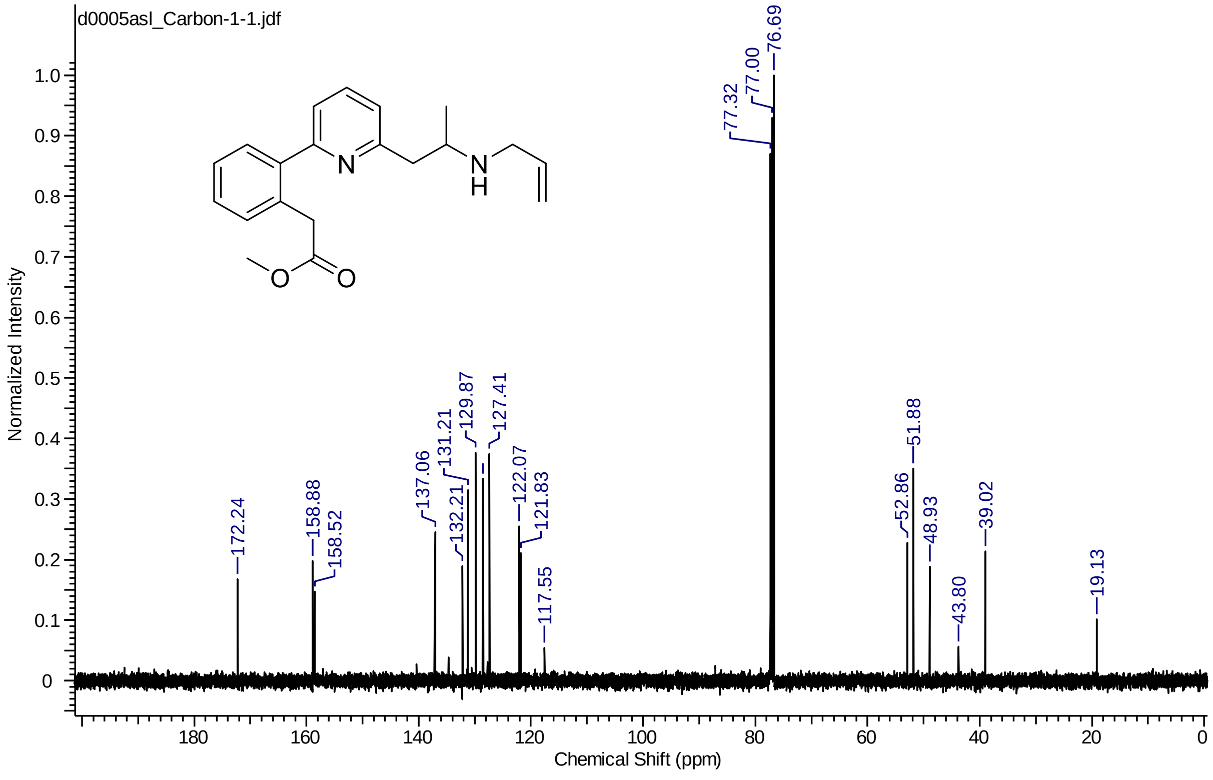

3-Allyl-4-methyl-4,5-dihydro-1*H*-6,10-(azeno)benzo[*d*][1]azacyclododecin-2(3*H*)-one (64)

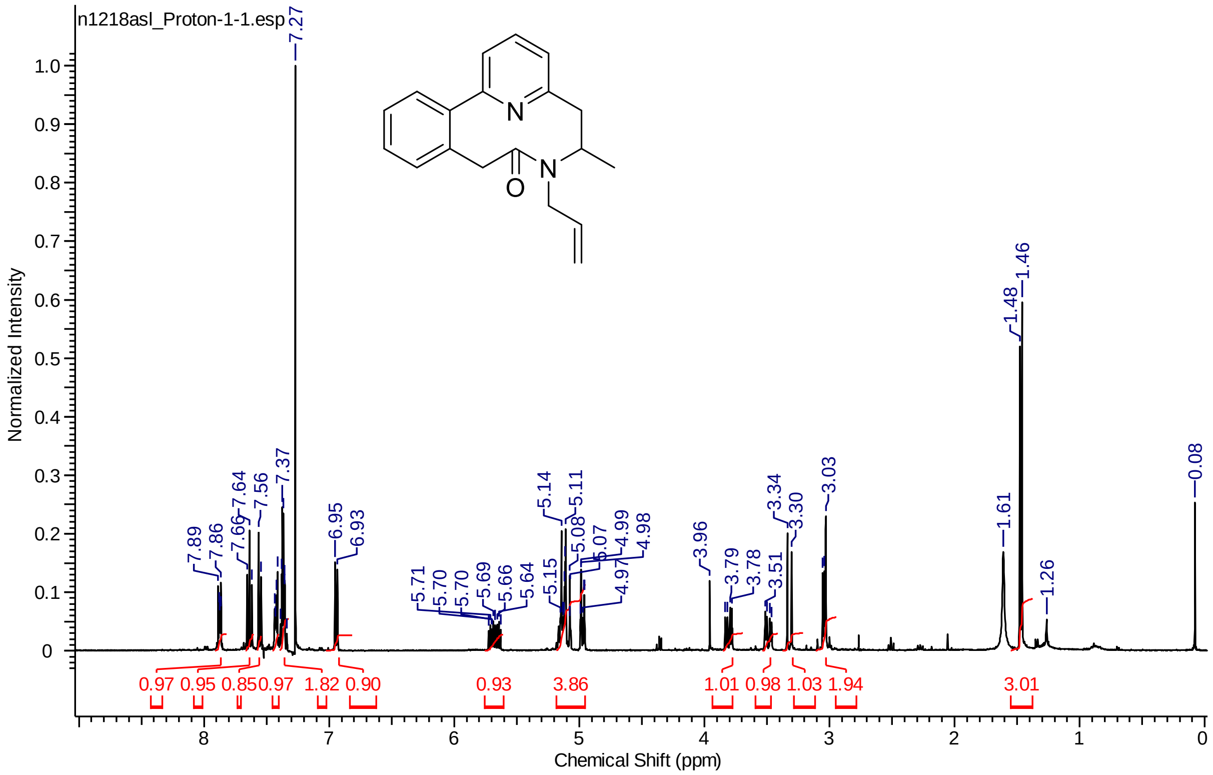

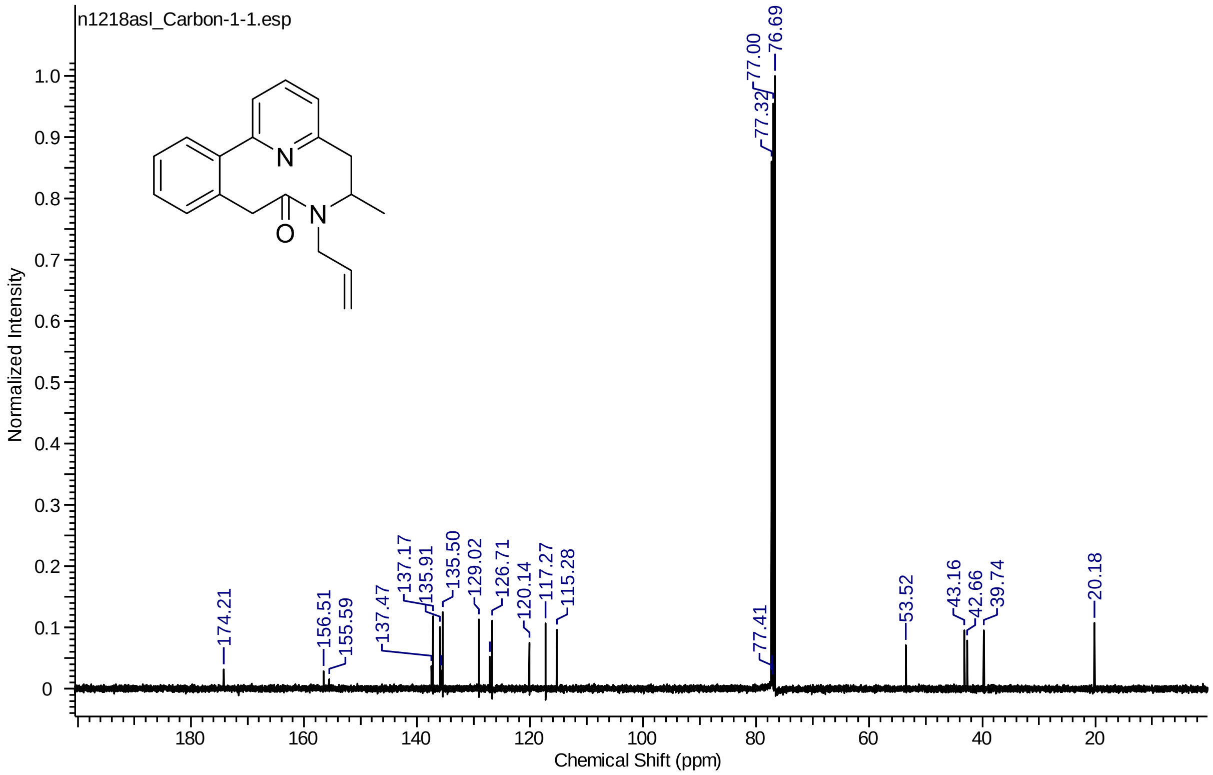

N-(1-(6-bromopyridin-2-yl)propan-2-yl)aniline (S75)

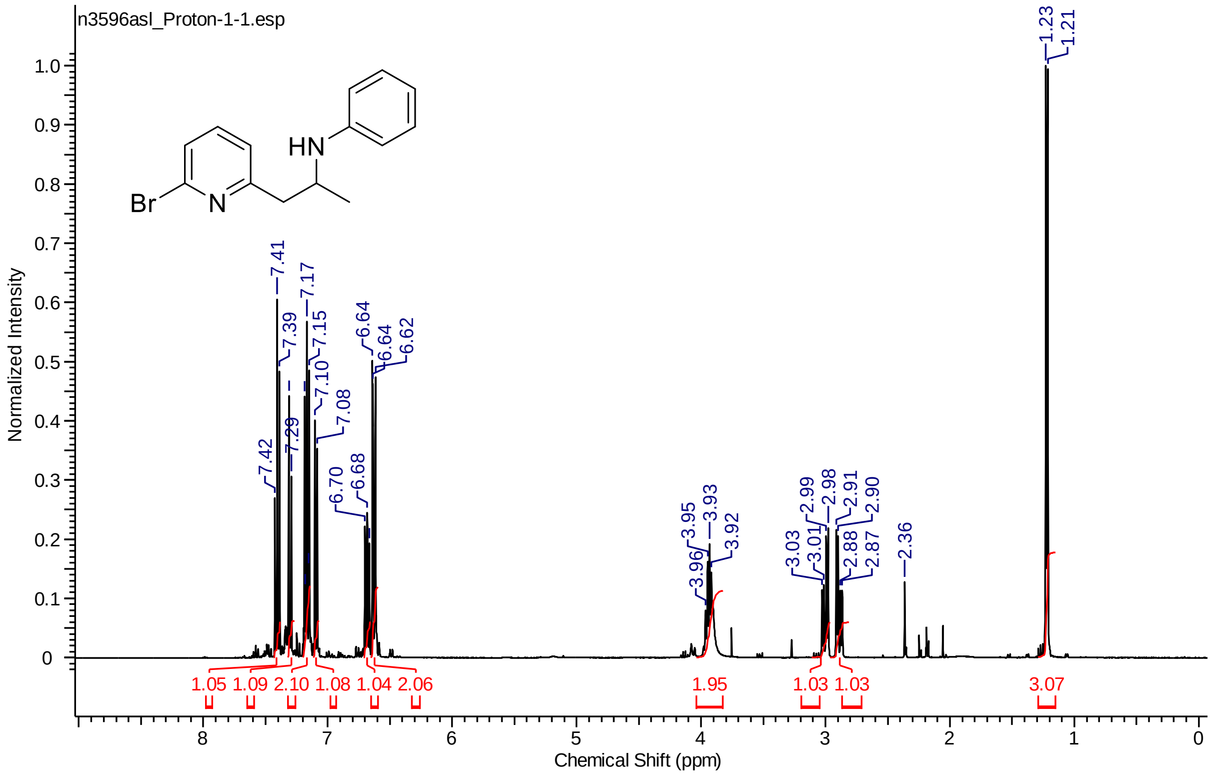

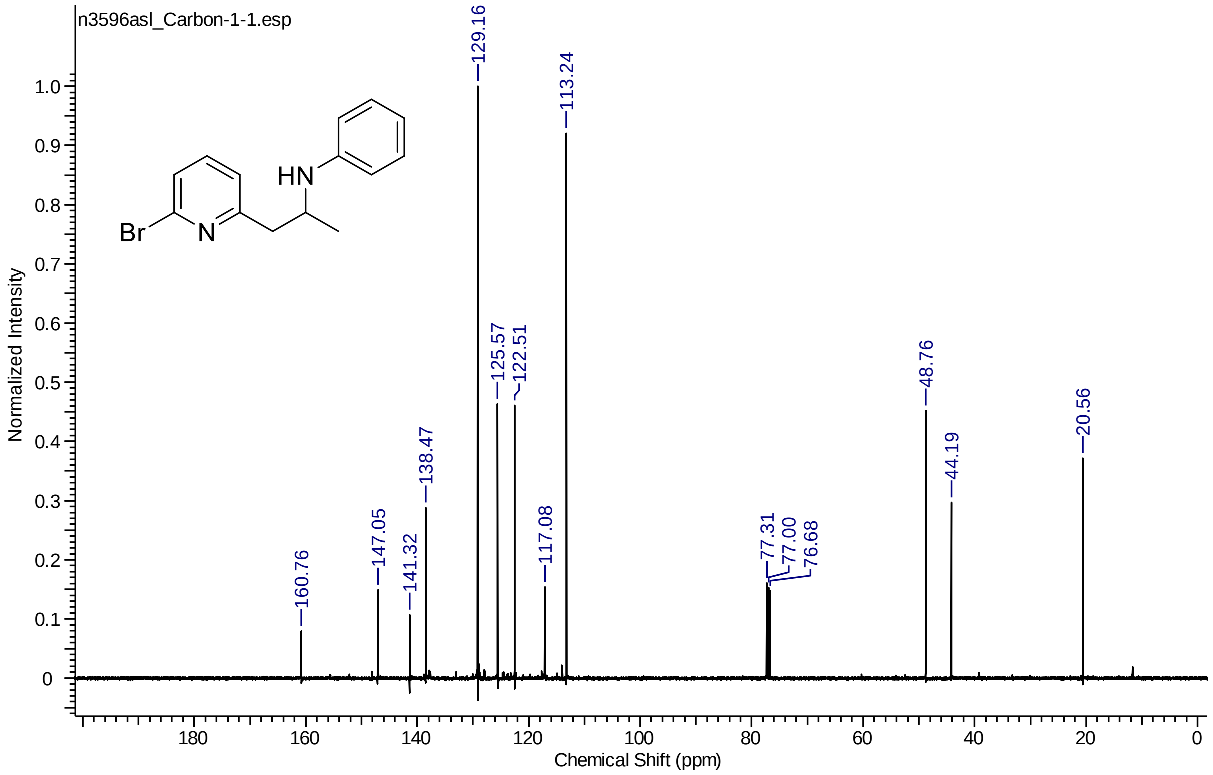

Methyl 2-(2-(6-(2-(phenylamino)propyl)pyridin-2-yl)phenyl)acetate (S76)

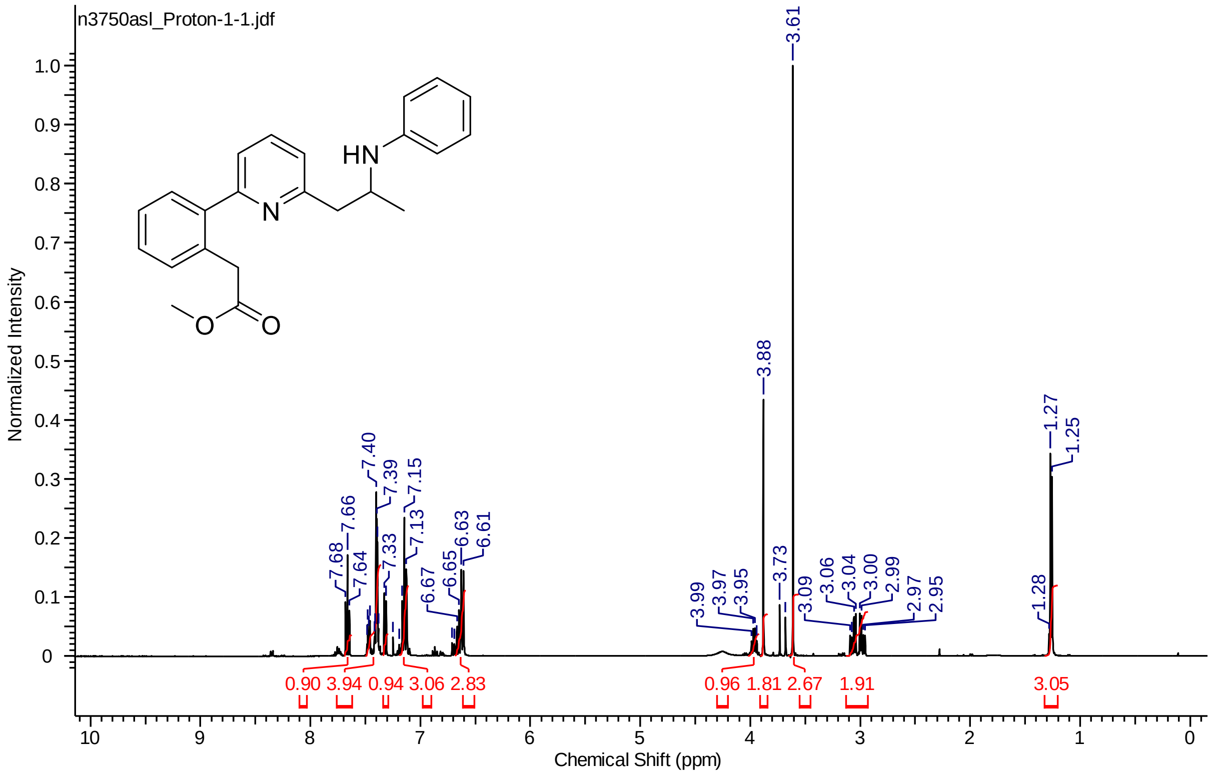

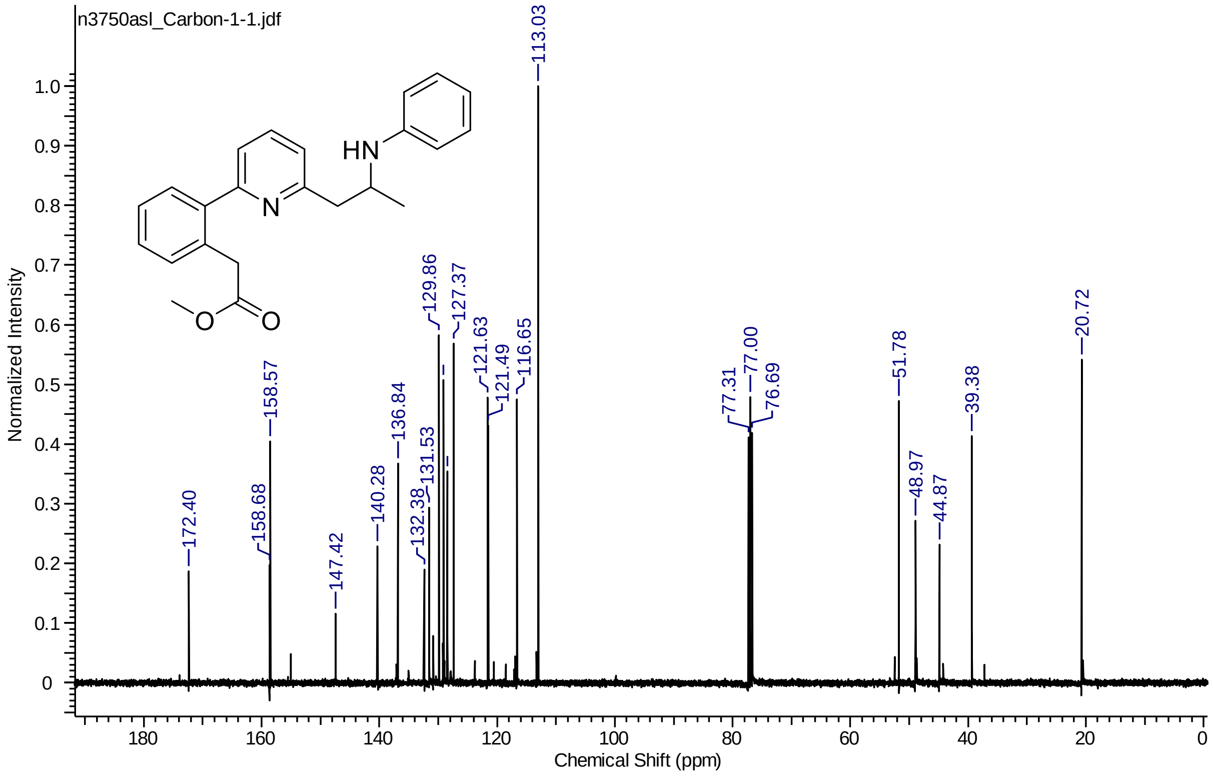

2-(2-(6-(2-(Phenylamino)propyl)pyridin-2-yl)phenyl)acetic acid (S77)

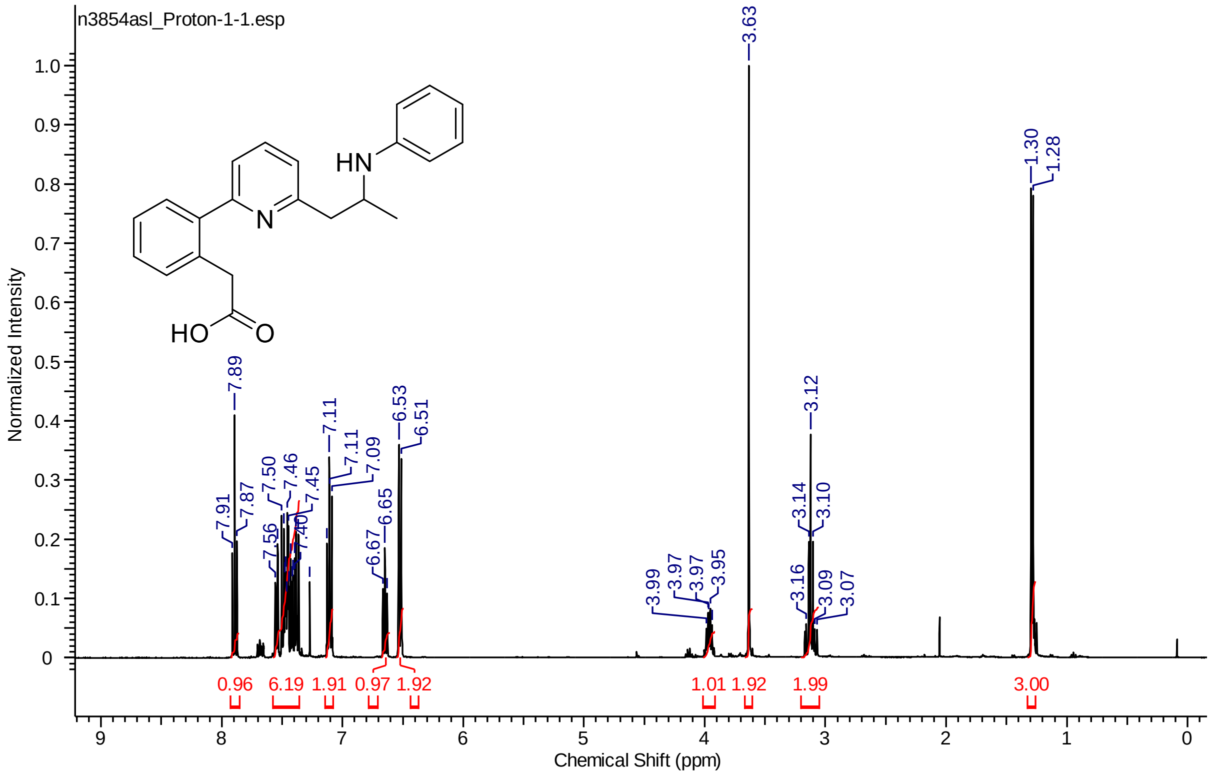

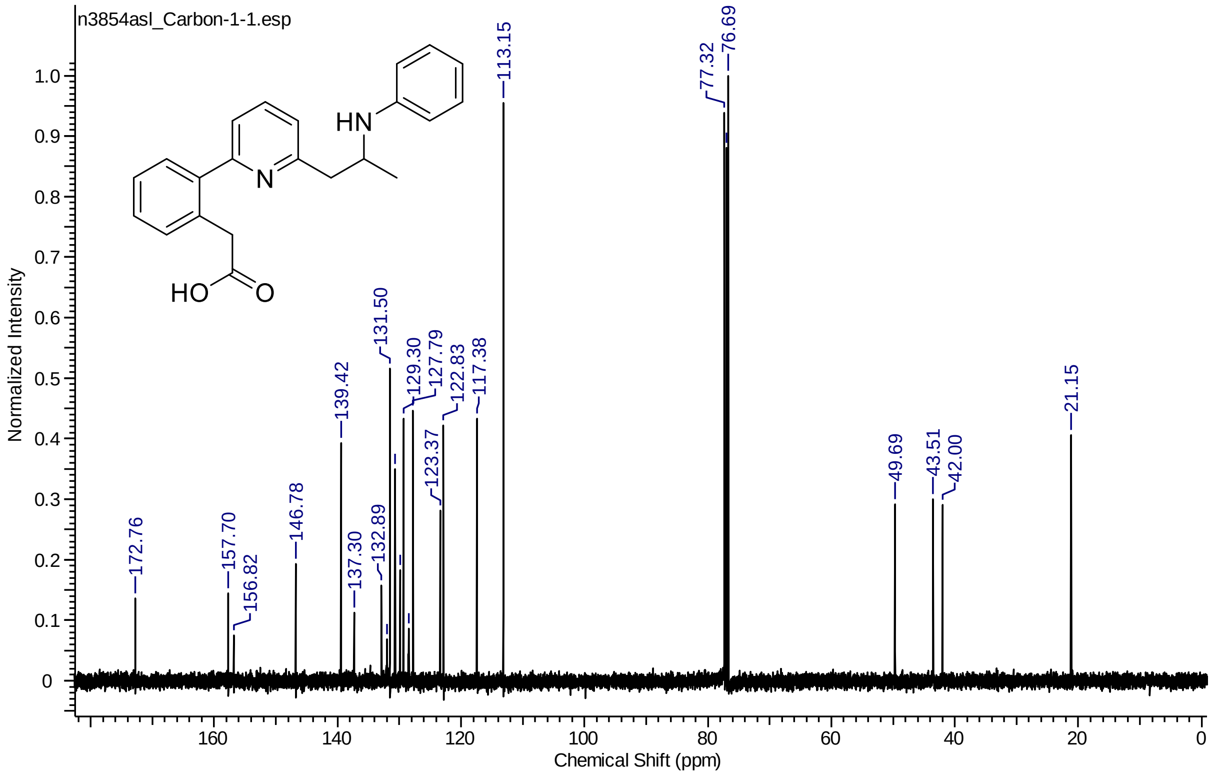

4-Methyl-3-phenyl-4,5-dihydro-1*H*-6,10-(azeno)benzo[*d*][1]azacyclododecin-2(3*H*)-one (65)

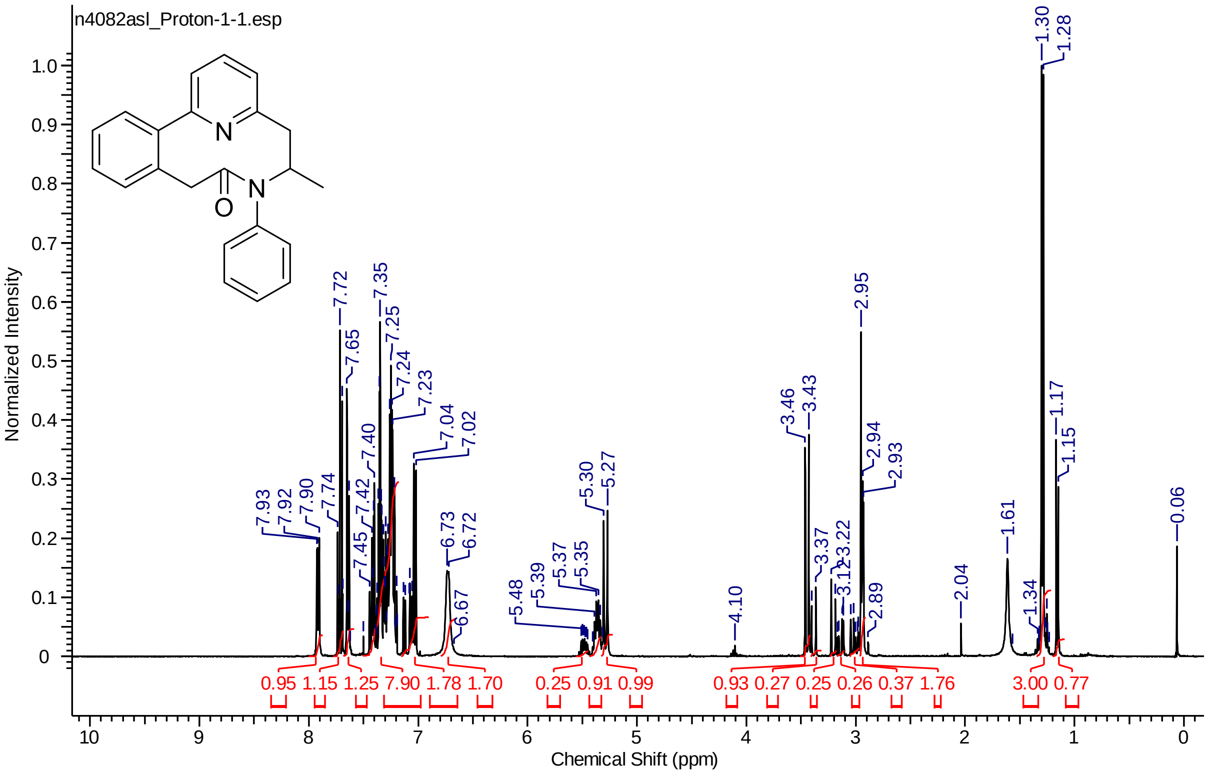

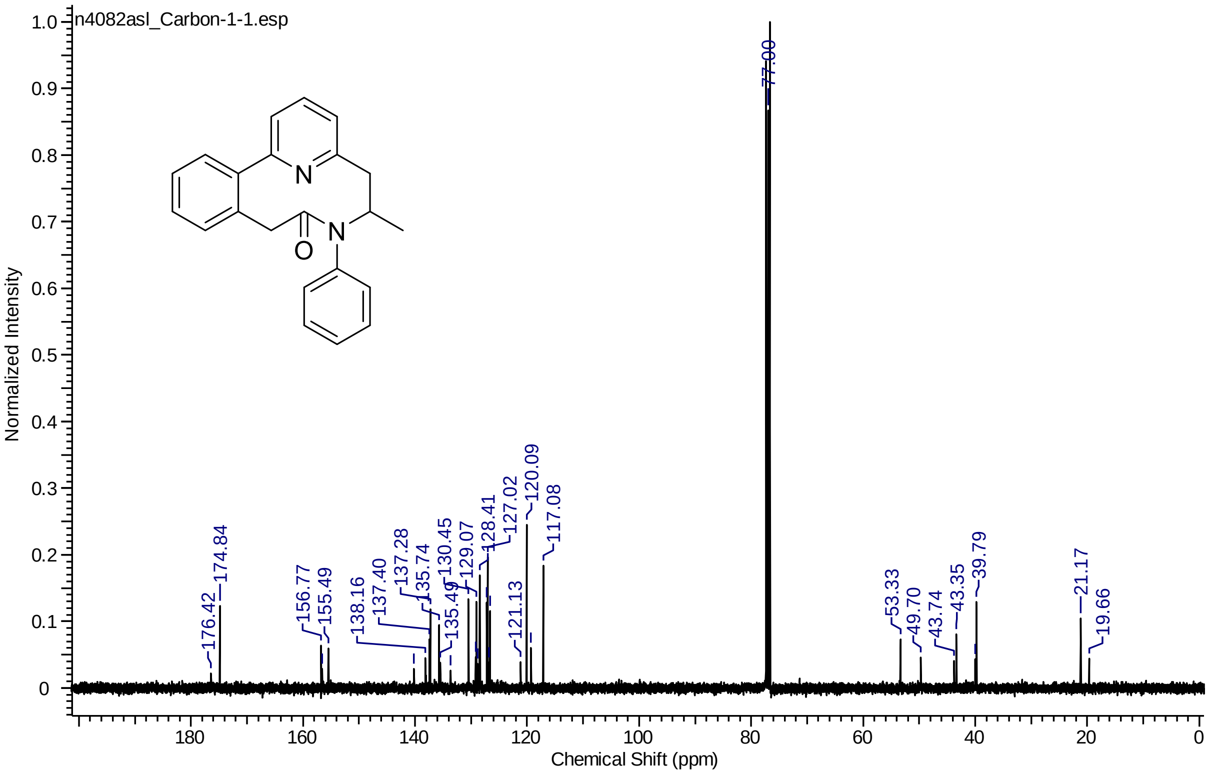

***tert*-Butyl 2-((6-chloropyridin-2-yl)methoxy)acetate (S81)**

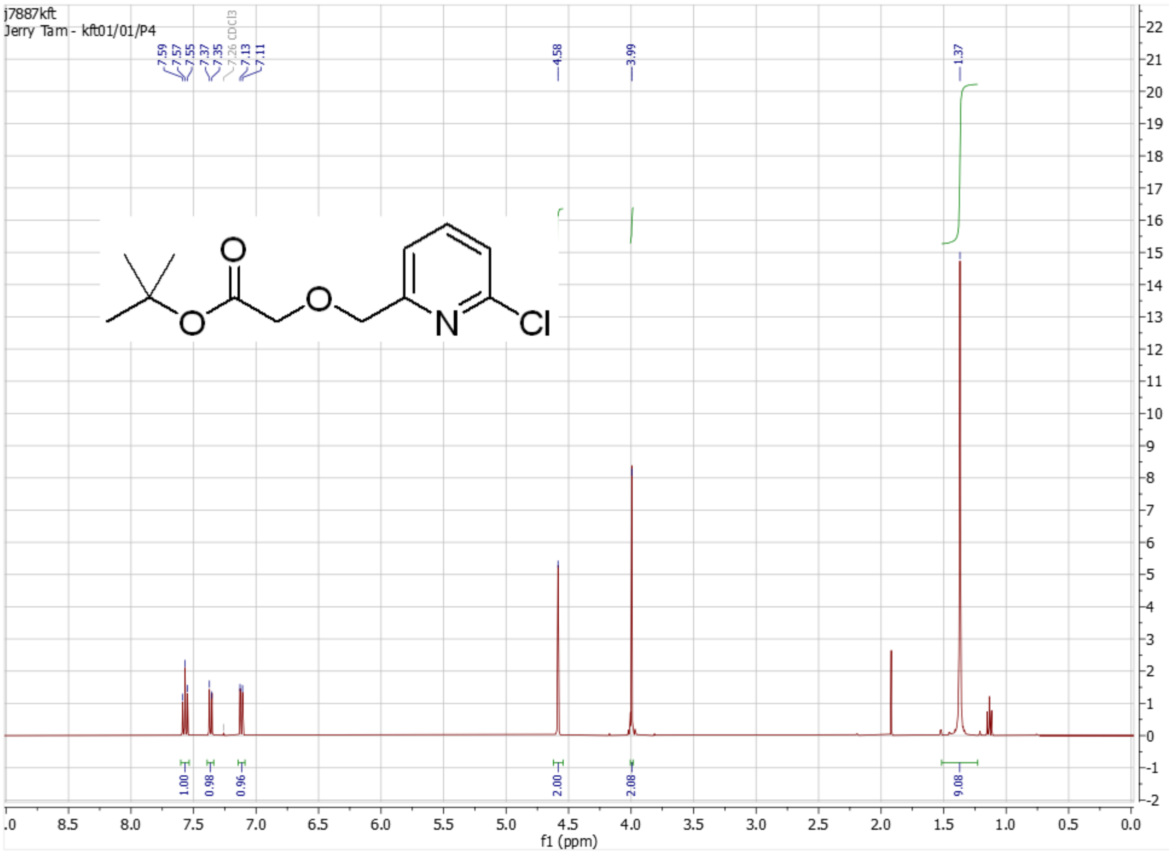

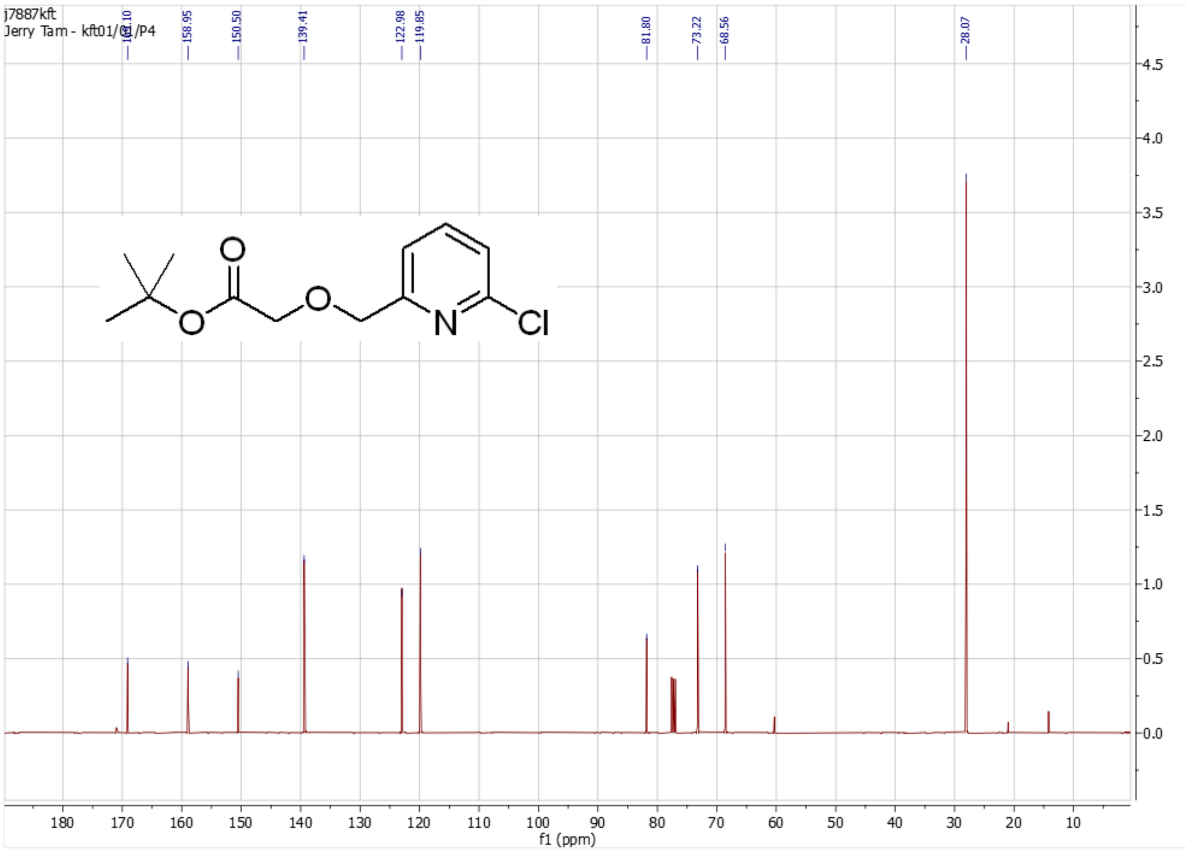

***tert*-Butyl 2-((6-vinylpyridin-2-yl)methoxy)acetate (S82)**

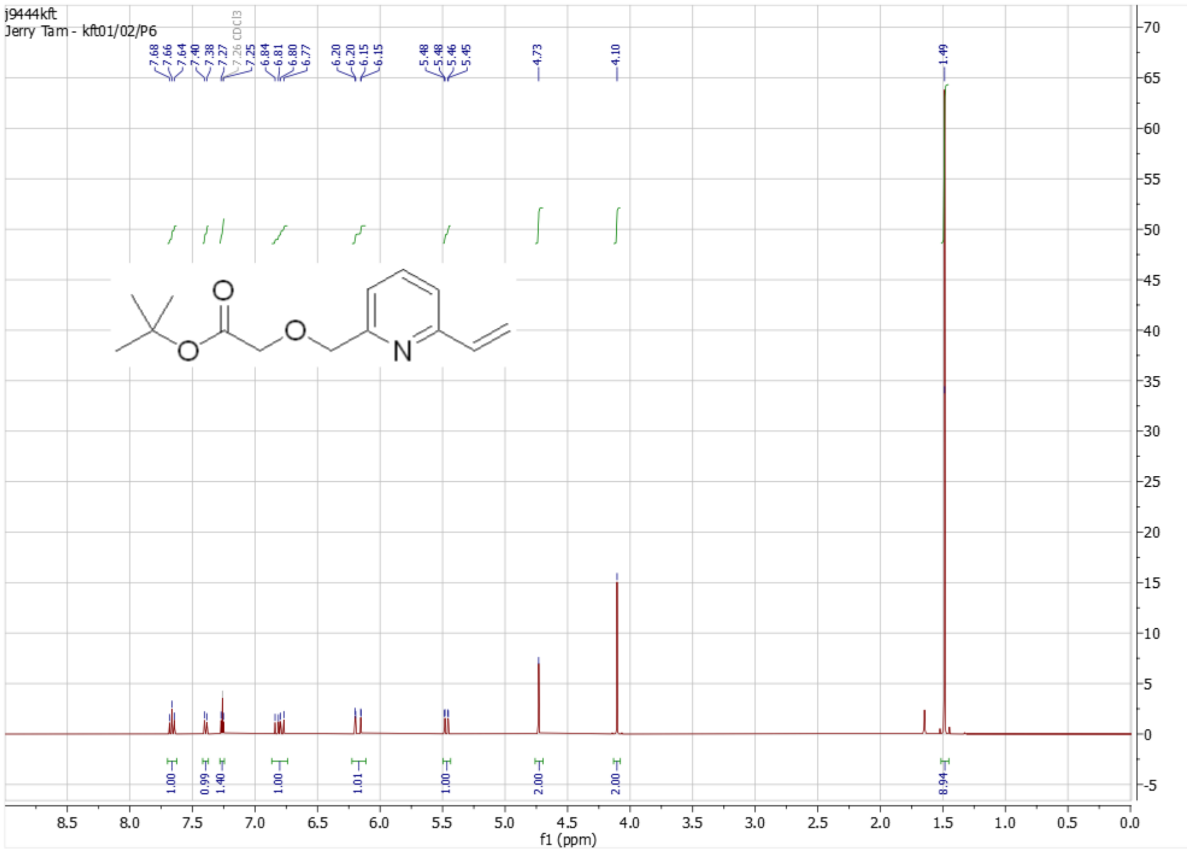

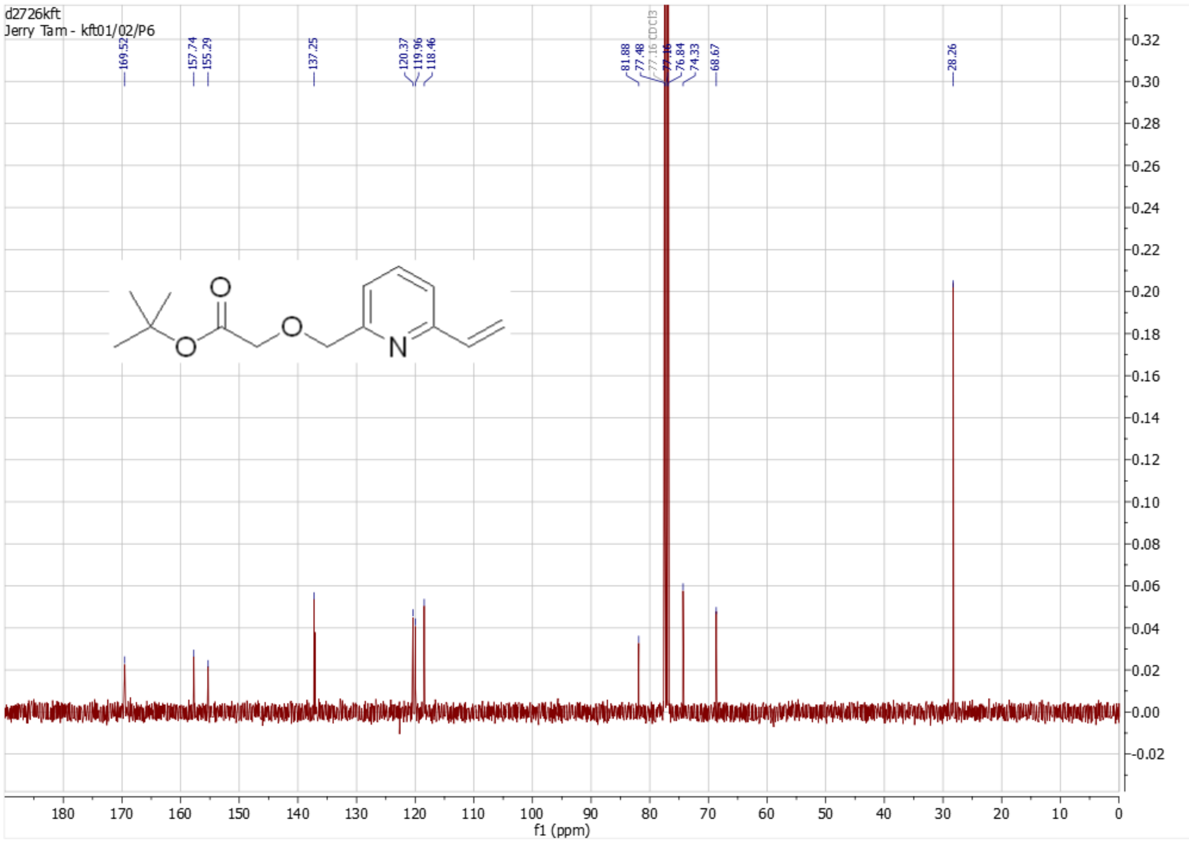

tert-Butyl 2-((6-(2-(phenylamino)ethyl)pyridin-2-yl)methoxy)acetate (S83)

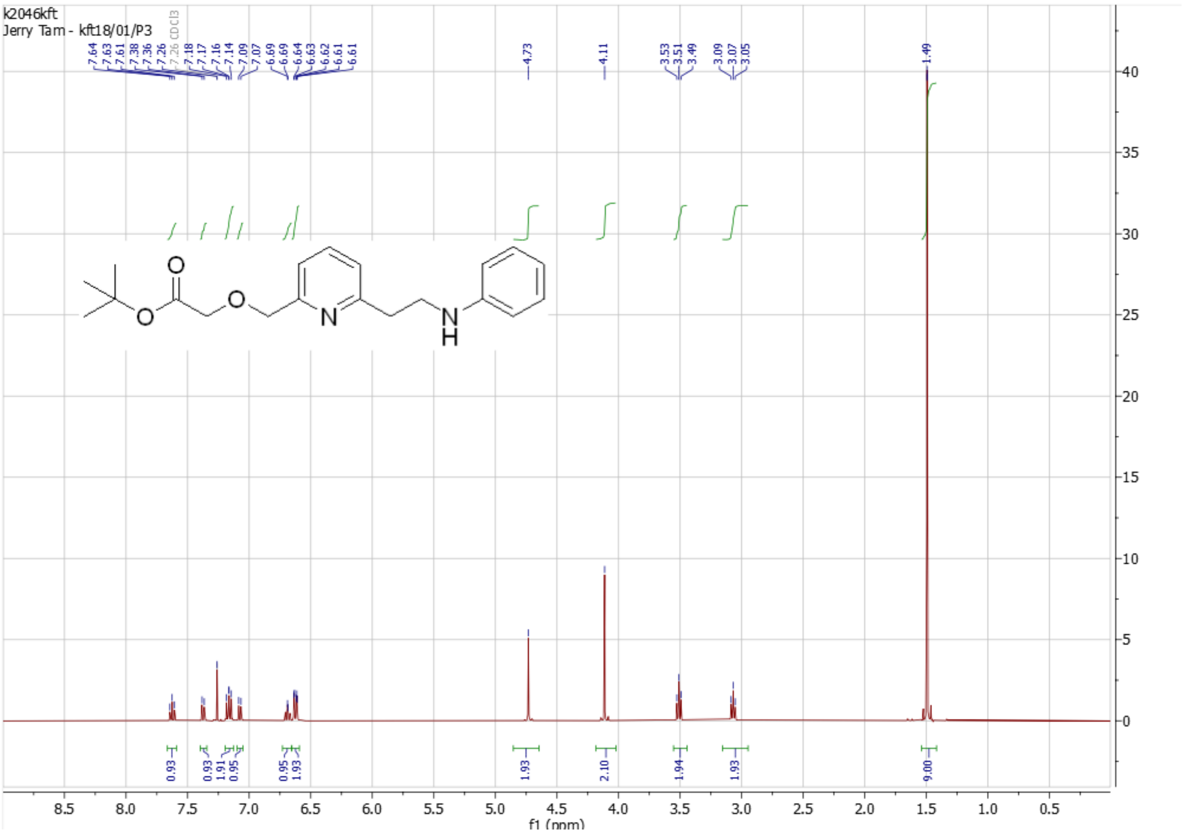

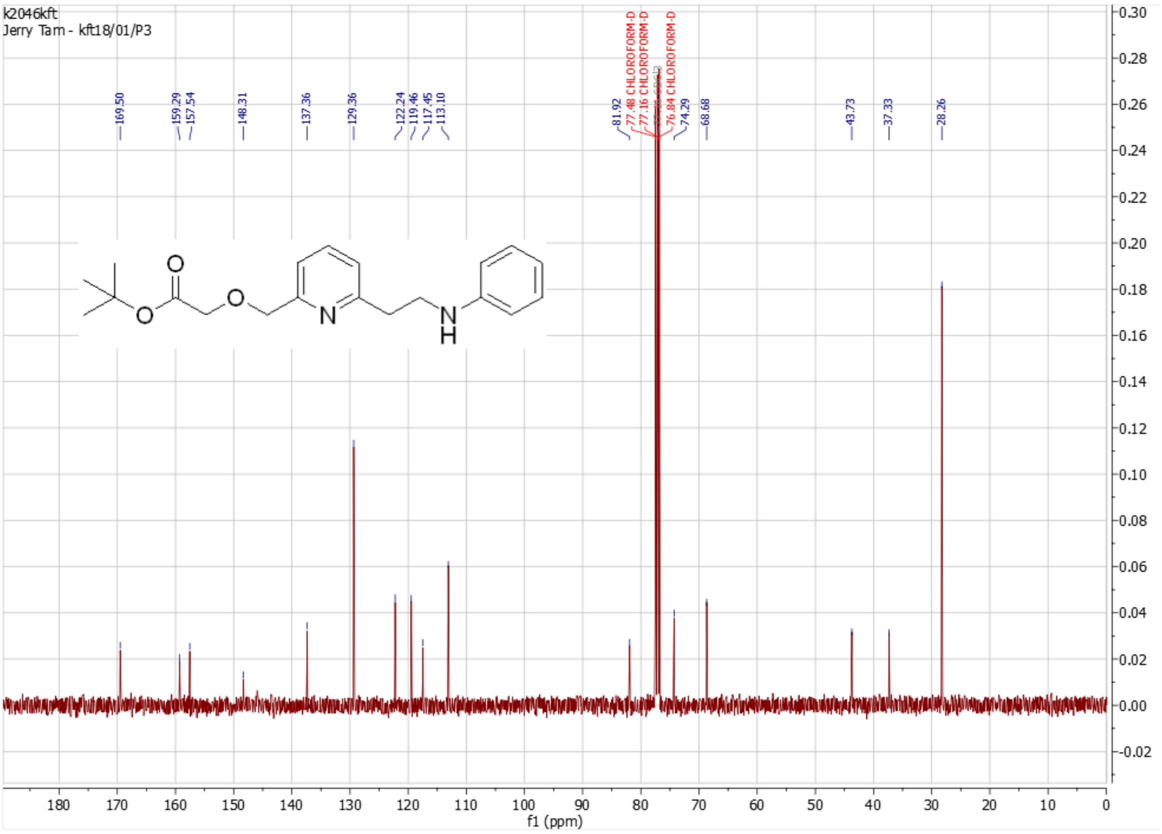

Lactam (67)

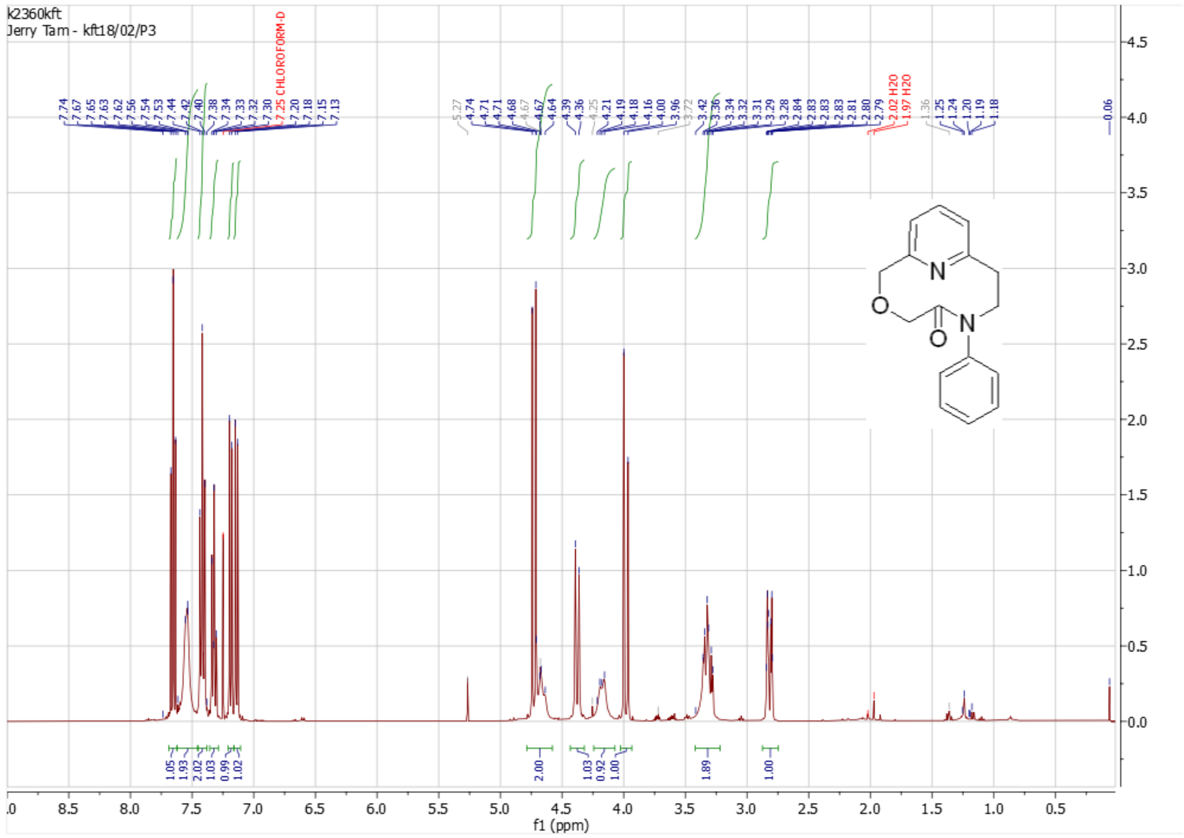

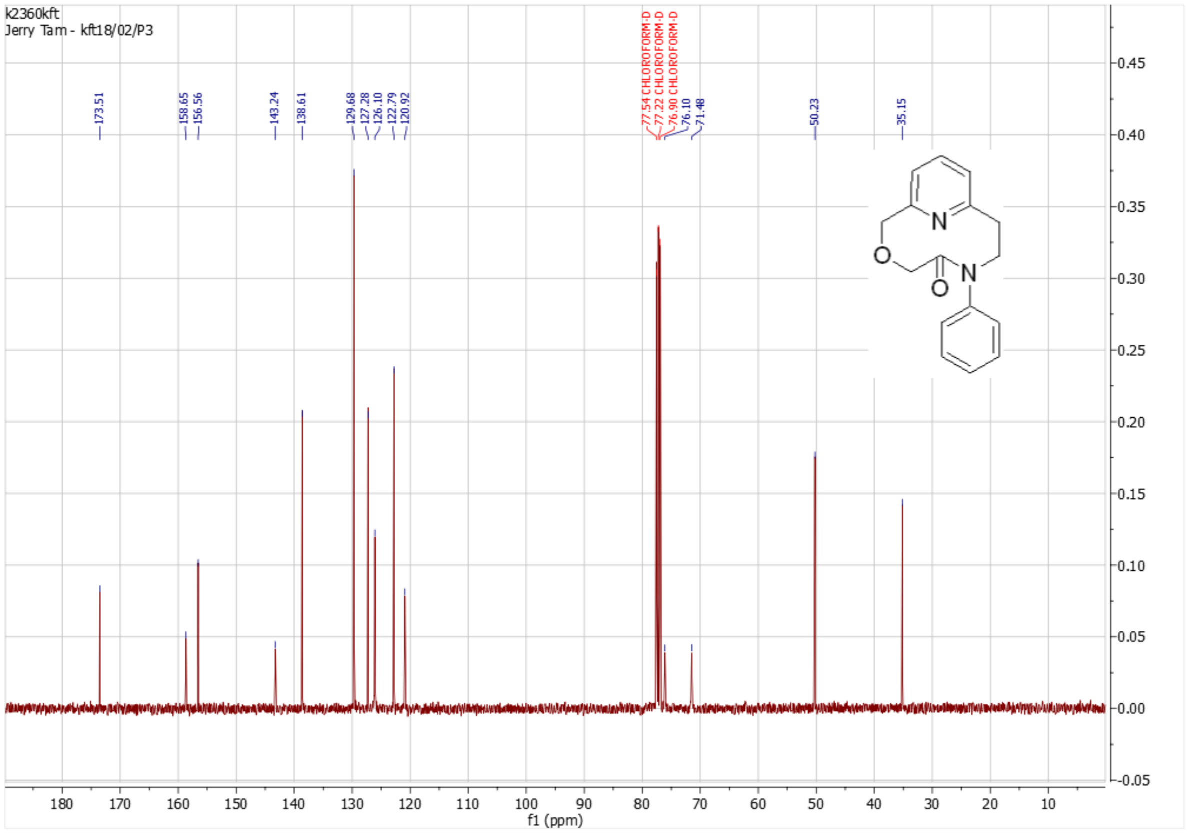

Methyl 2-((ethyl(3-(ethylamino) propyl) amino) methyl) benzoate (S84)

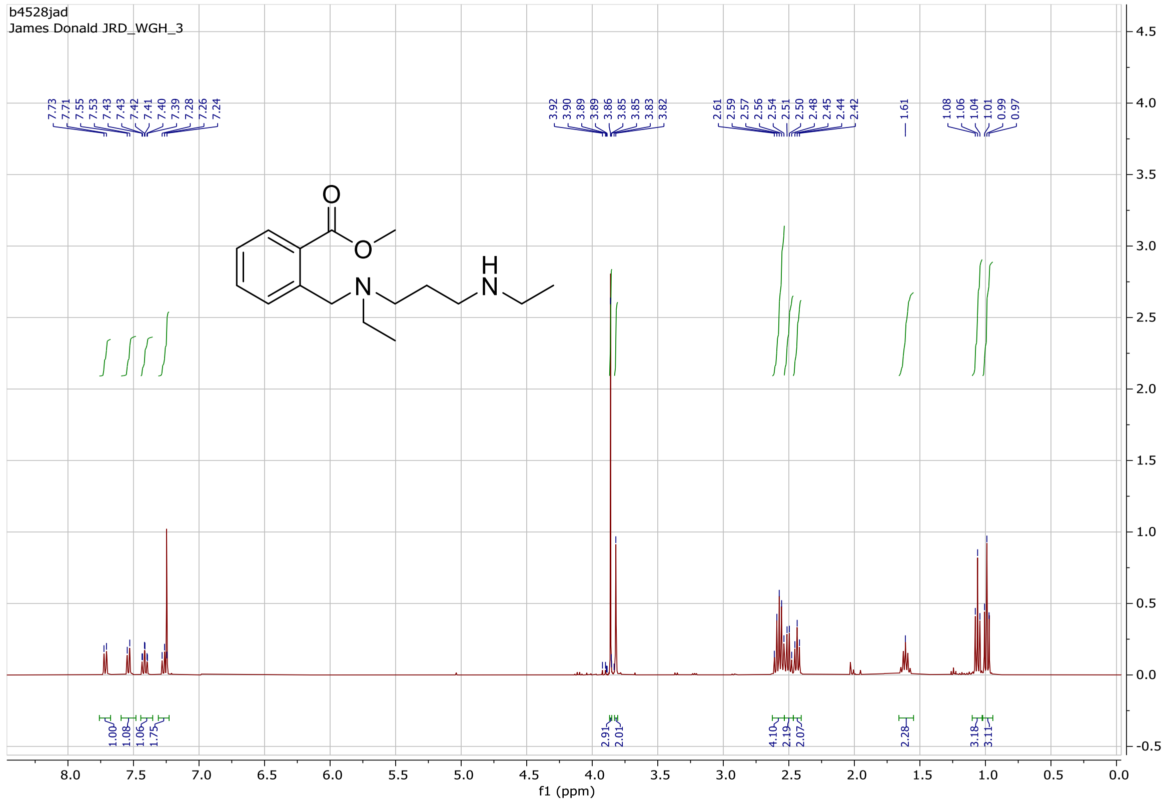

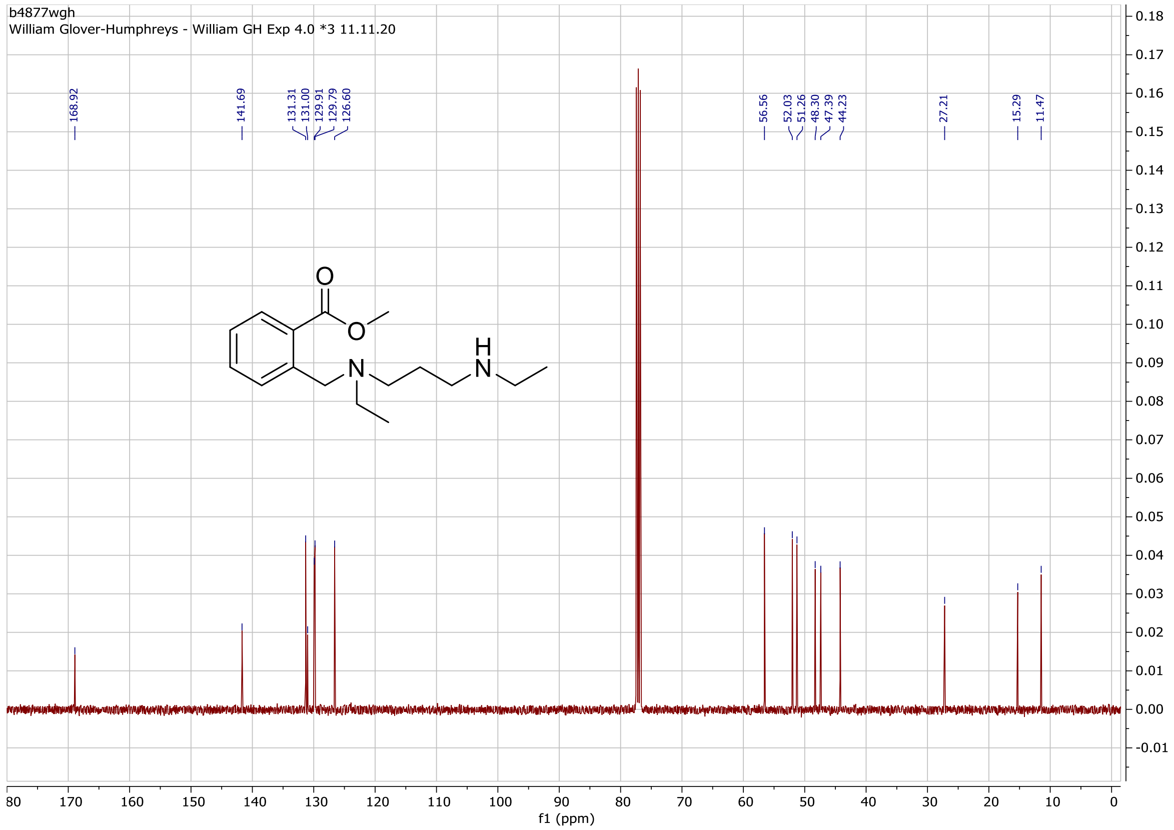

2,4-Diethyl-2,3,4,5,6,7-hexahydro-1H-2,4-benzodiazonin-1-one (68)

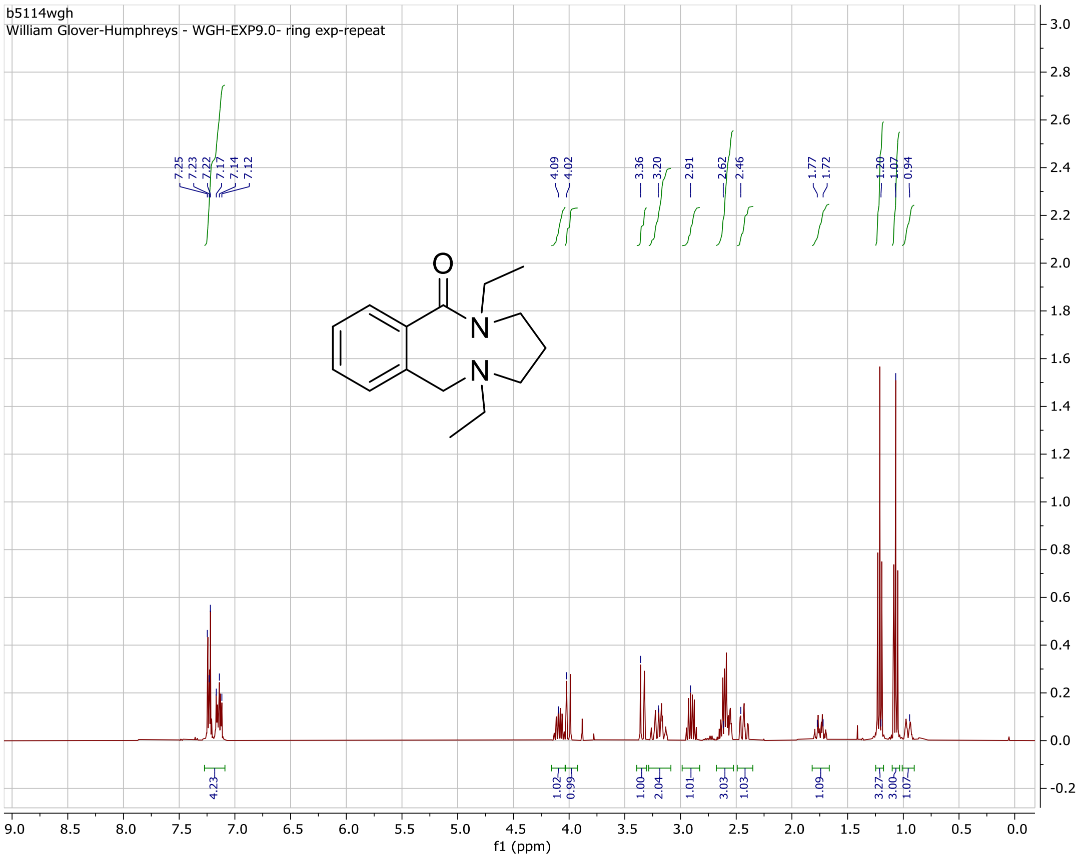

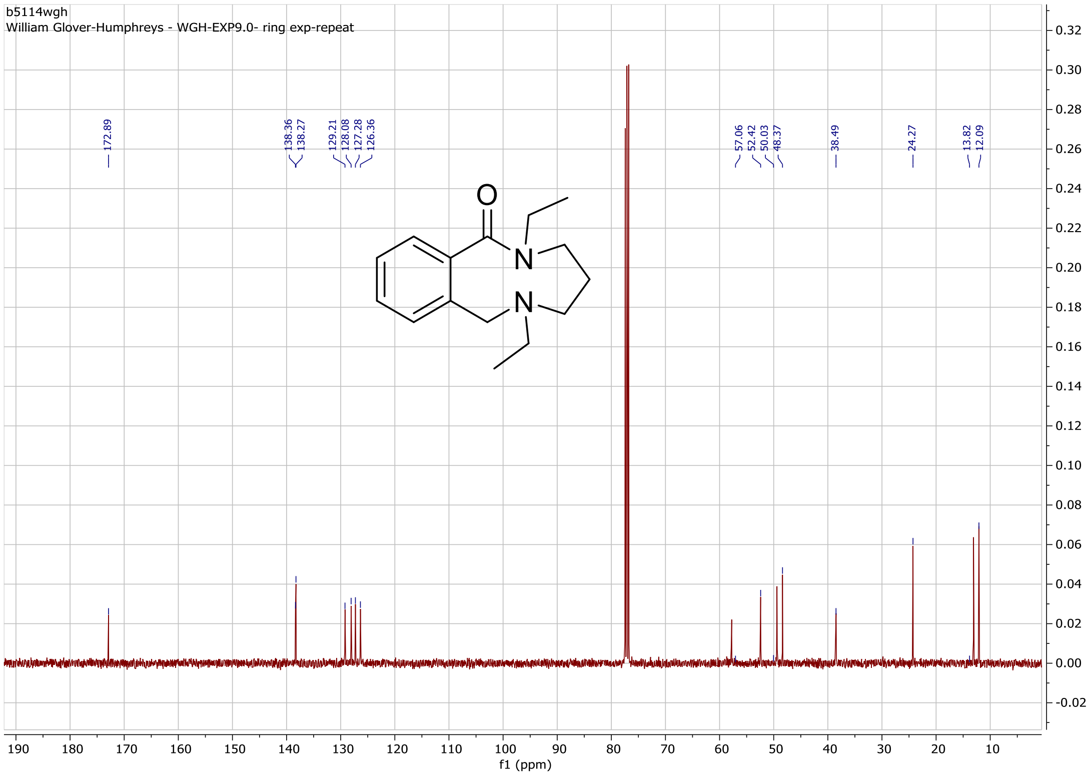

Methyl 2-(((3-mercaptopropyl)thio)methyl)benzoate (S85)

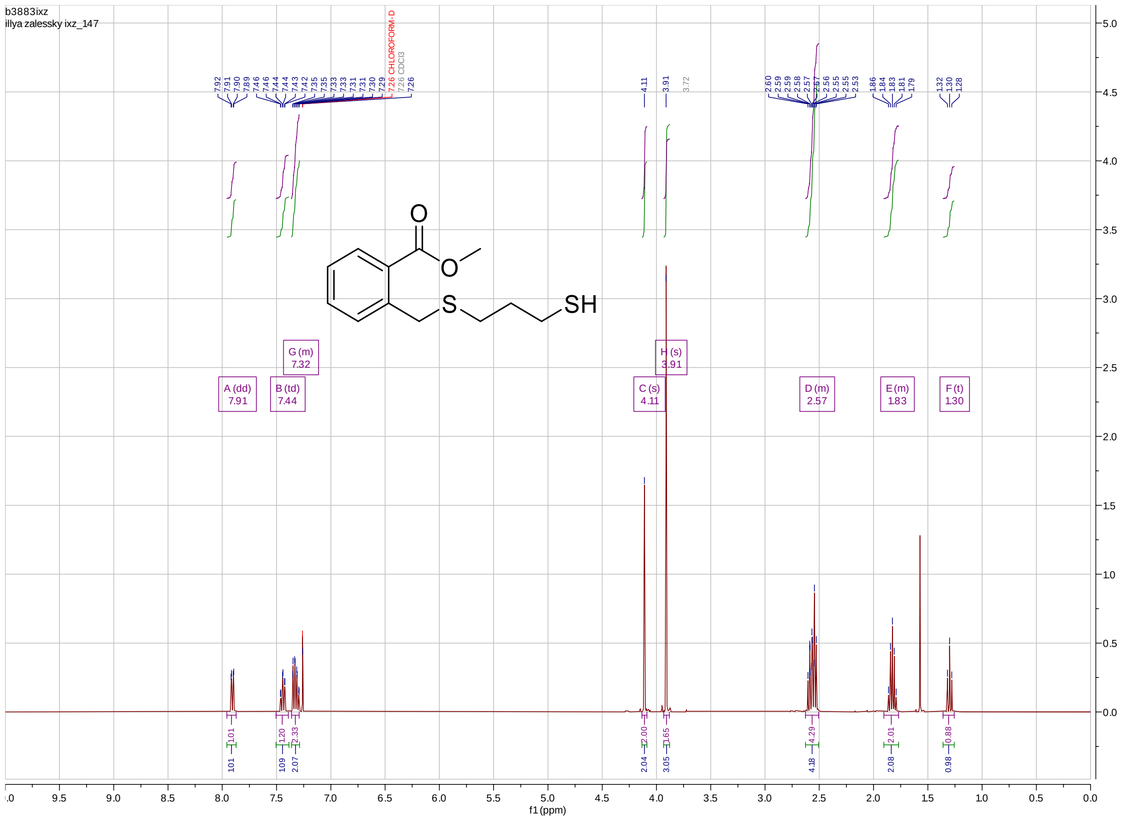

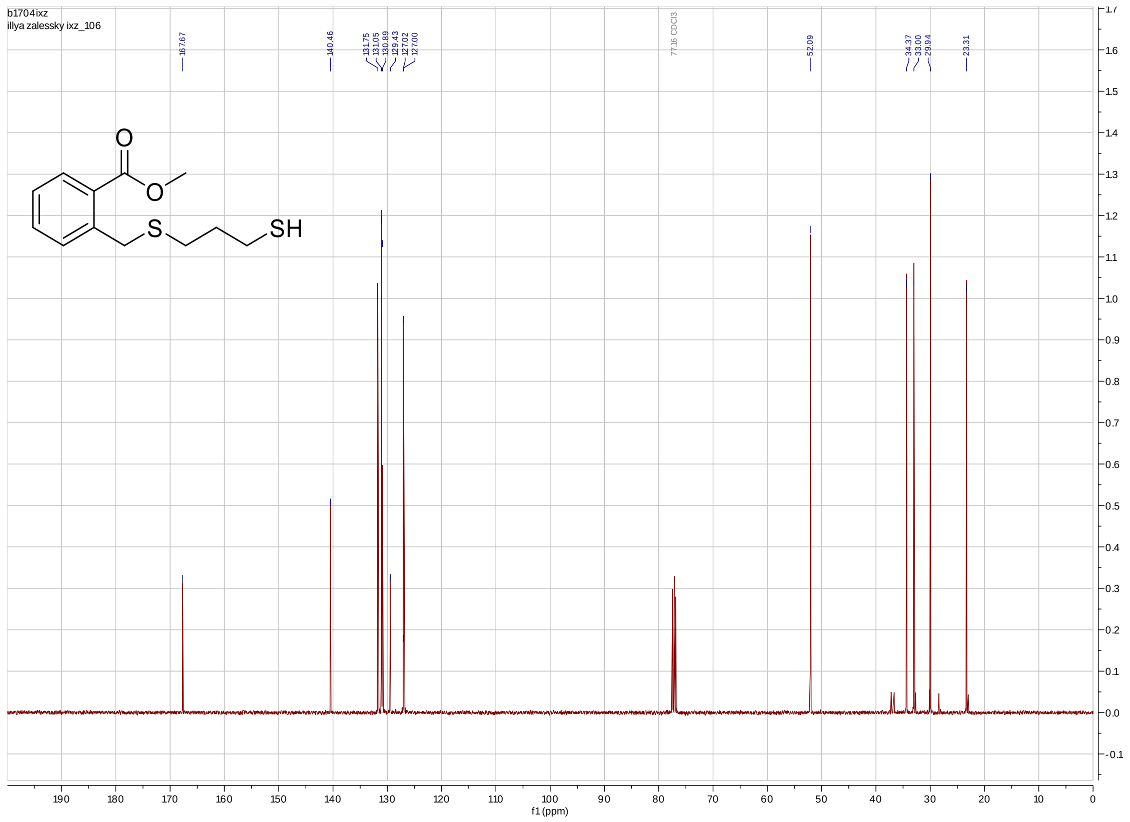

2-(((3-Mercaptopropyl)thio)methyl)benzoic acid (S86)

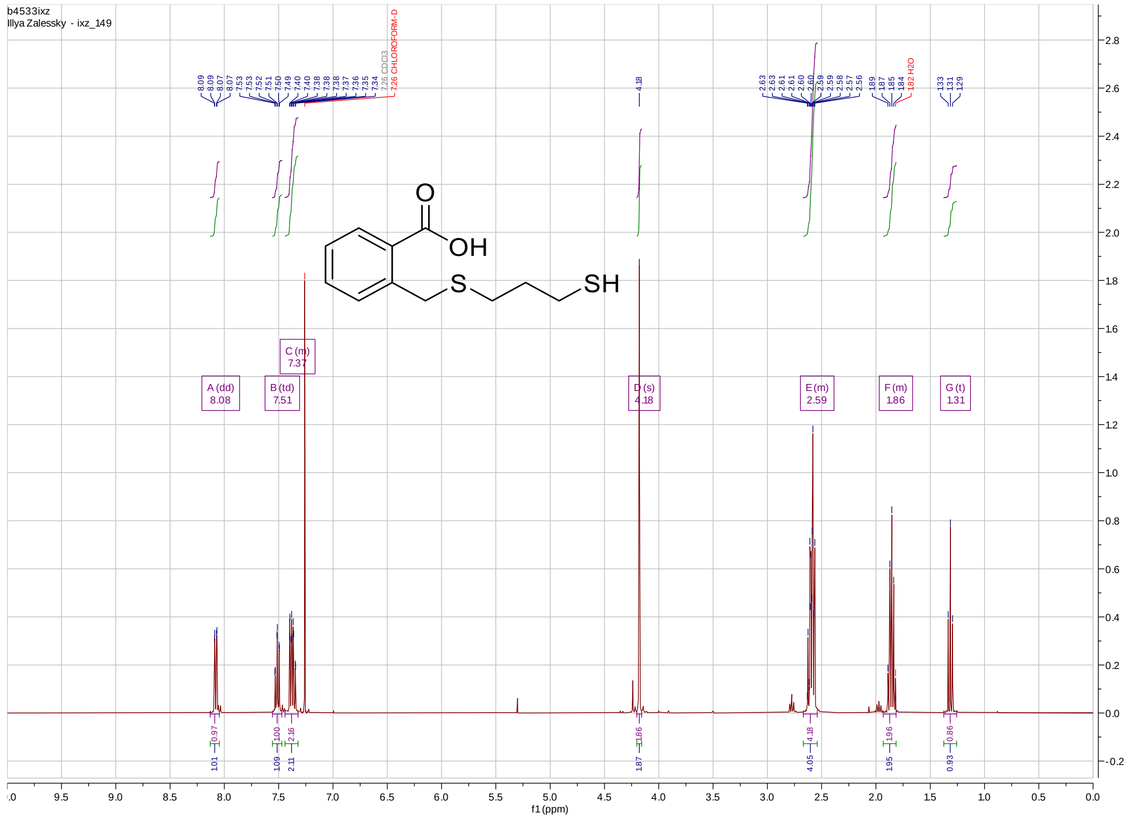

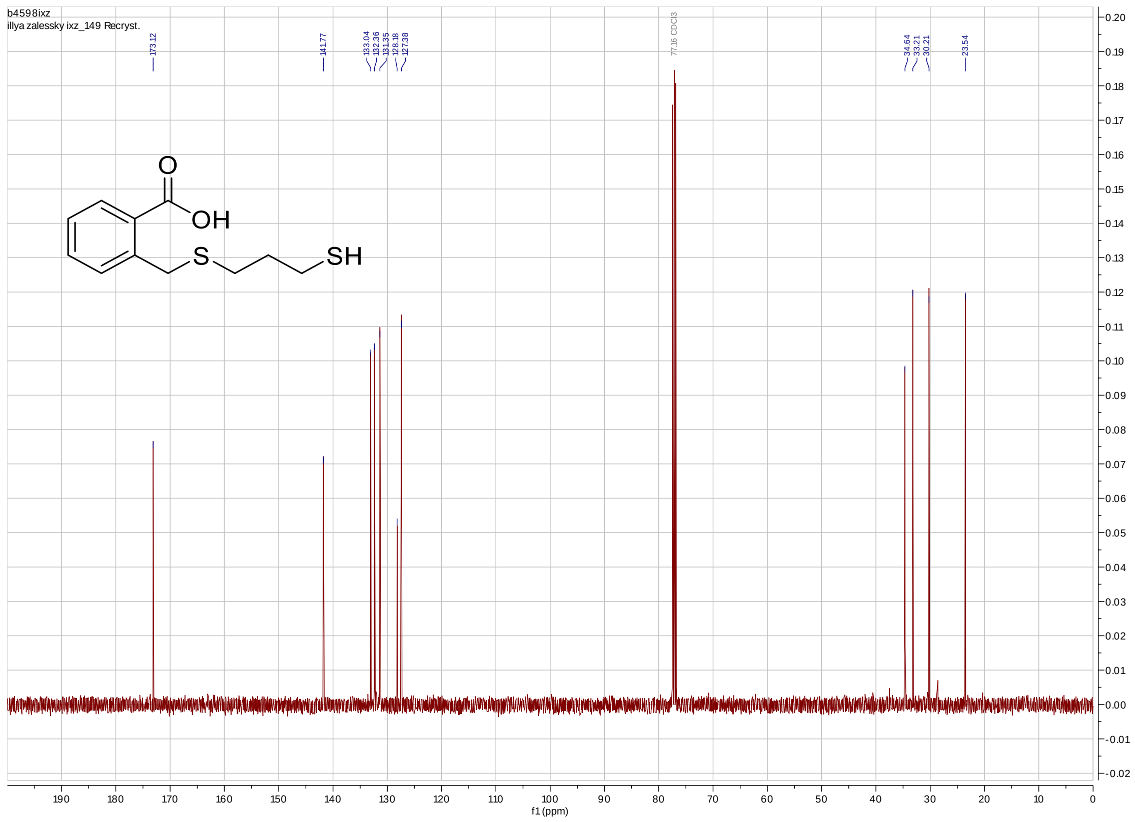

4,5-Dihydro-3H-benzo[*g*][1,5]dithionin-1(7*H*)-one (69)

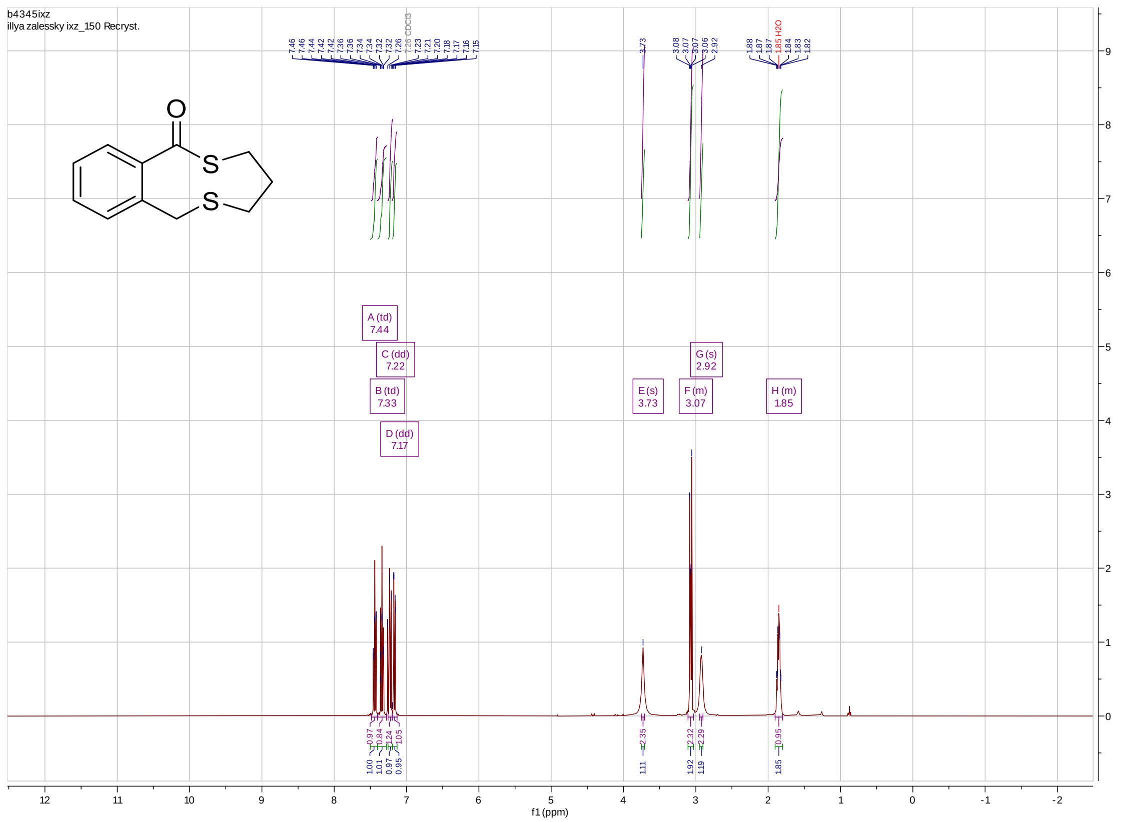

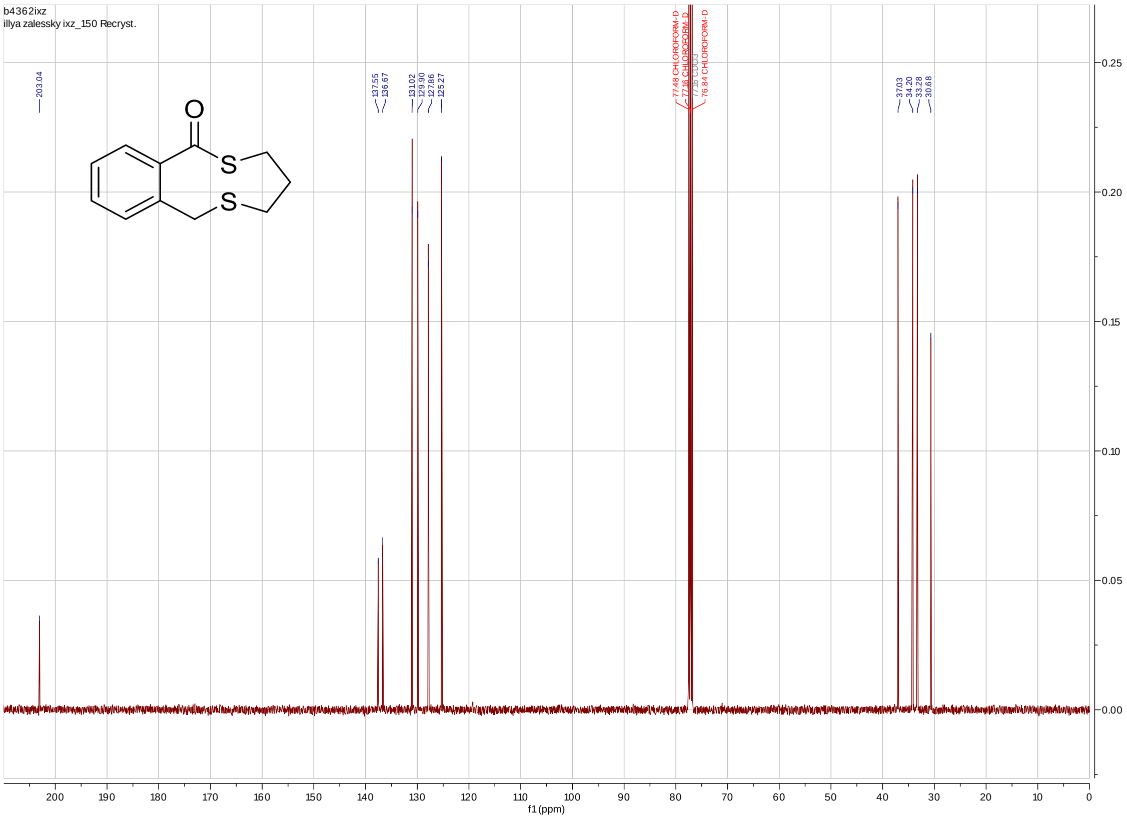

8,9,19,20-Tetrahydro-7*H*,18*H*-dibenzo[*g,p*][1,5,10,14]tetrathiacyclooctadecine-5,16(11*H*,22*H*)-dione (S87)

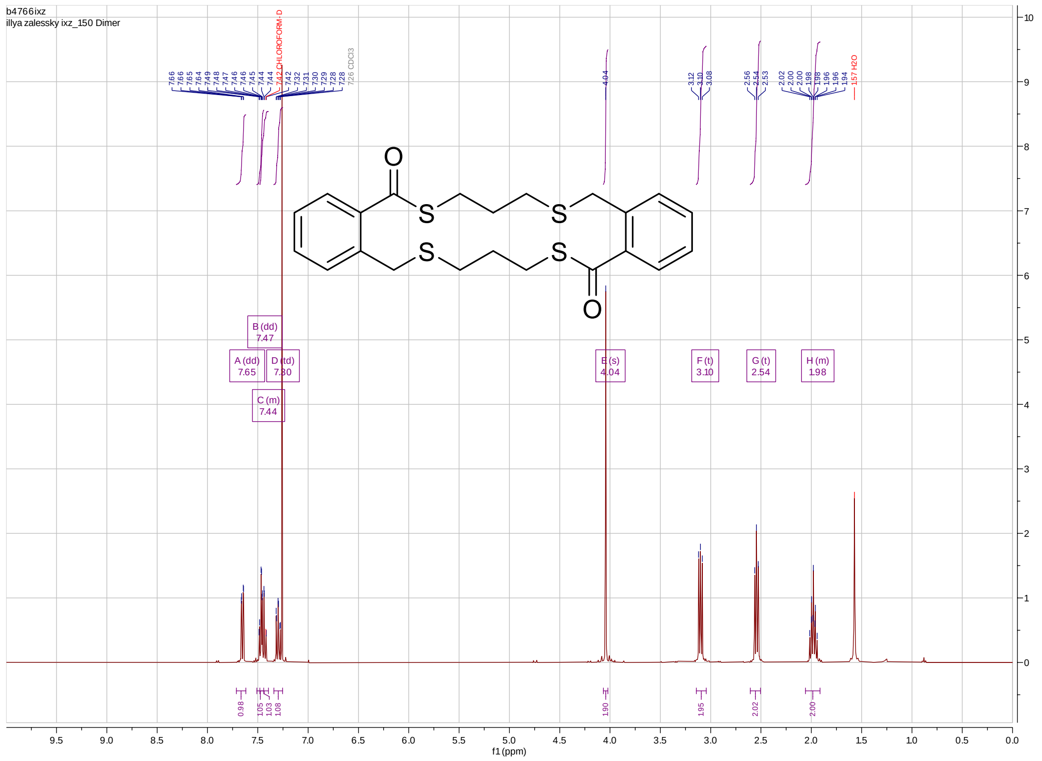

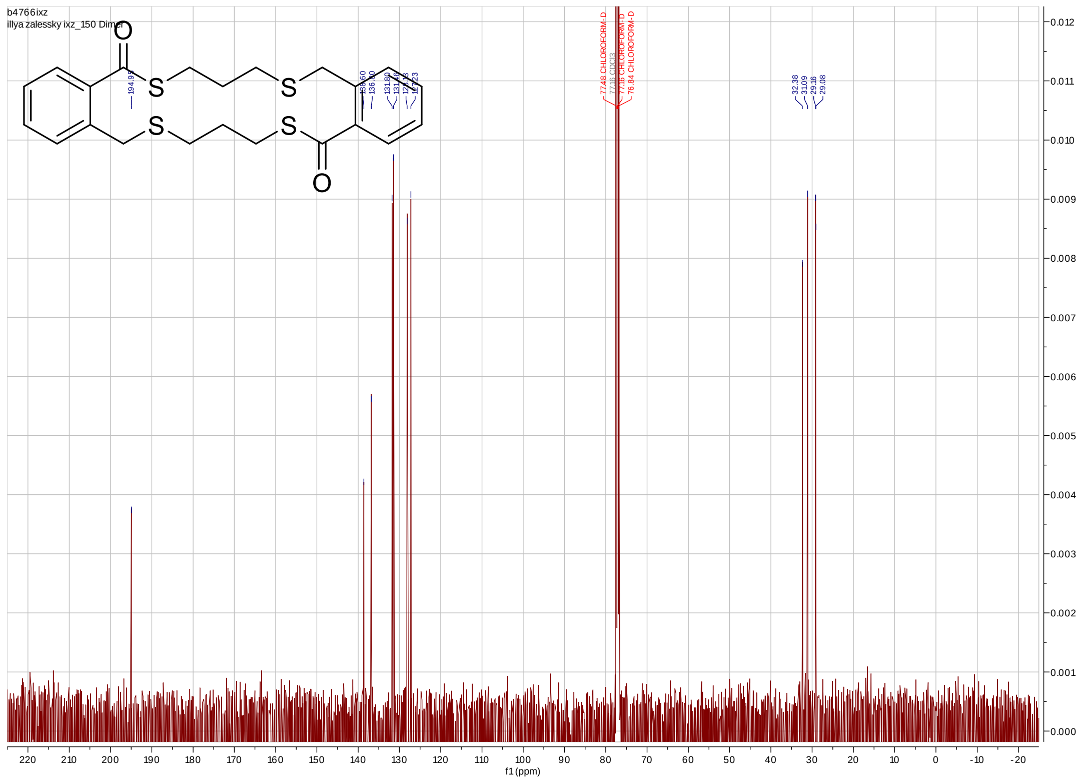

Methyl 2-((methyl(2-(phenylamino) ethyl) amino) methyl) quinoline-3-carboxylate (S68)

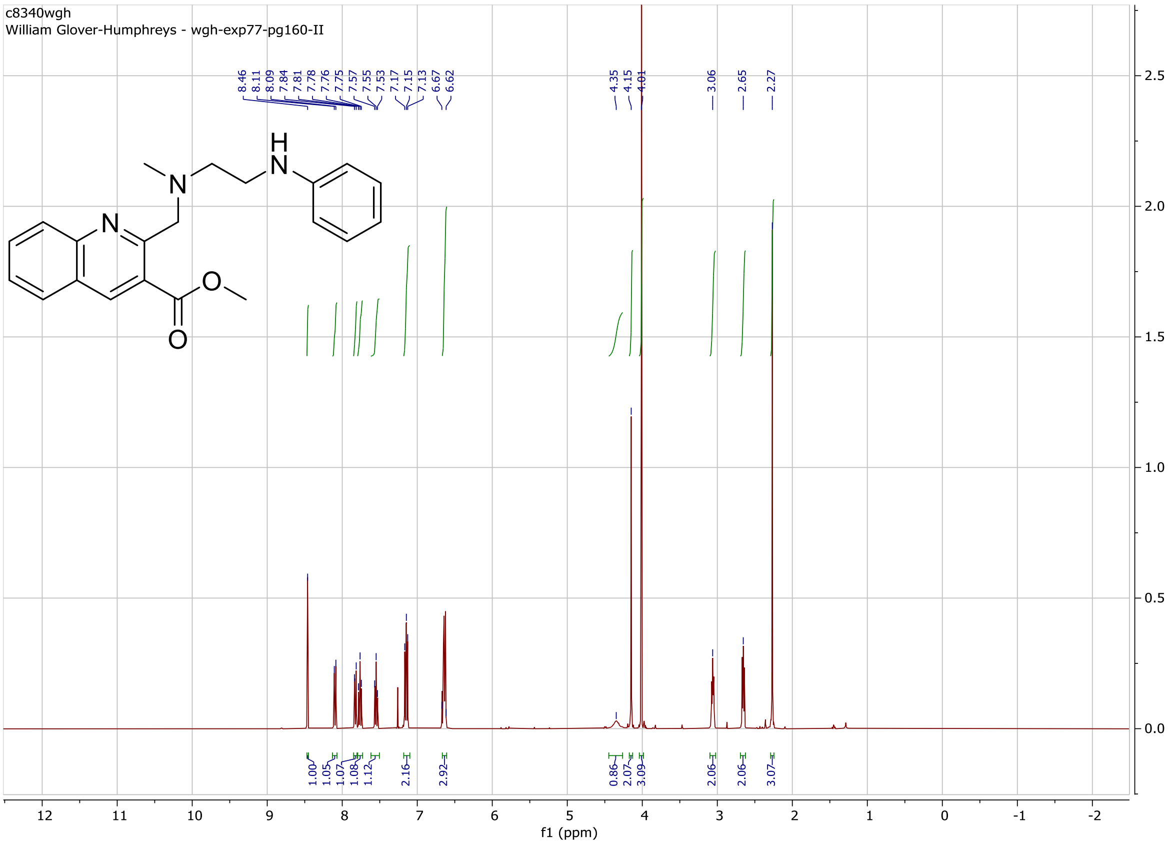

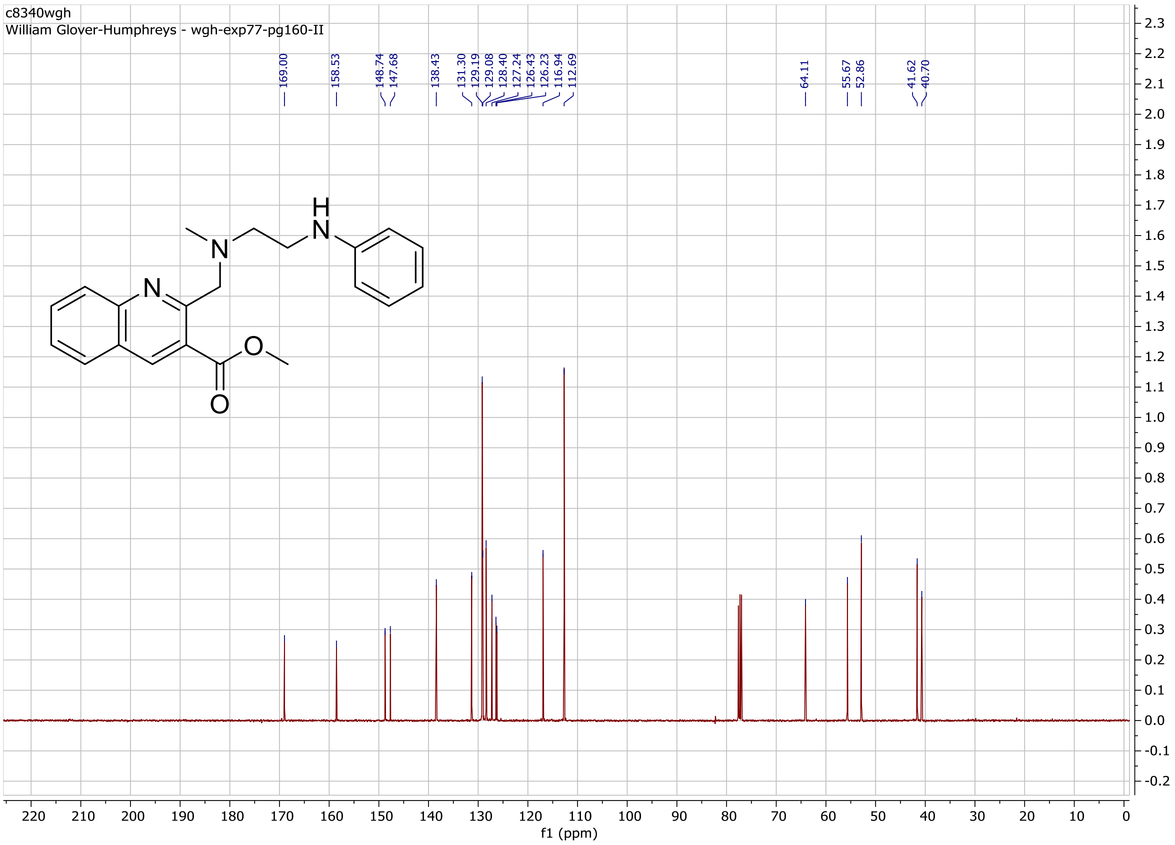

5-Methyl-2-phenyl-3,4,5,4-tetrahydro- [1,4] diazocino[6,7-b] quinolin-1(2H)-one (70)

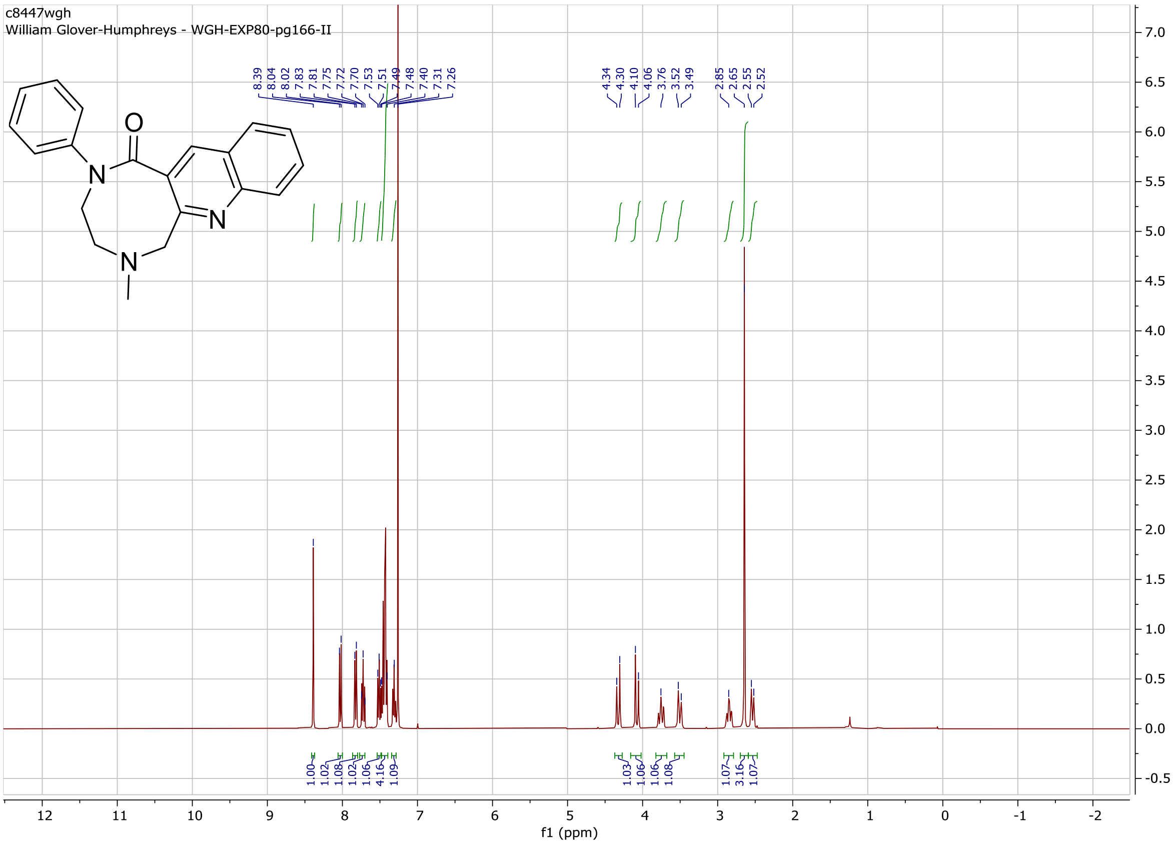

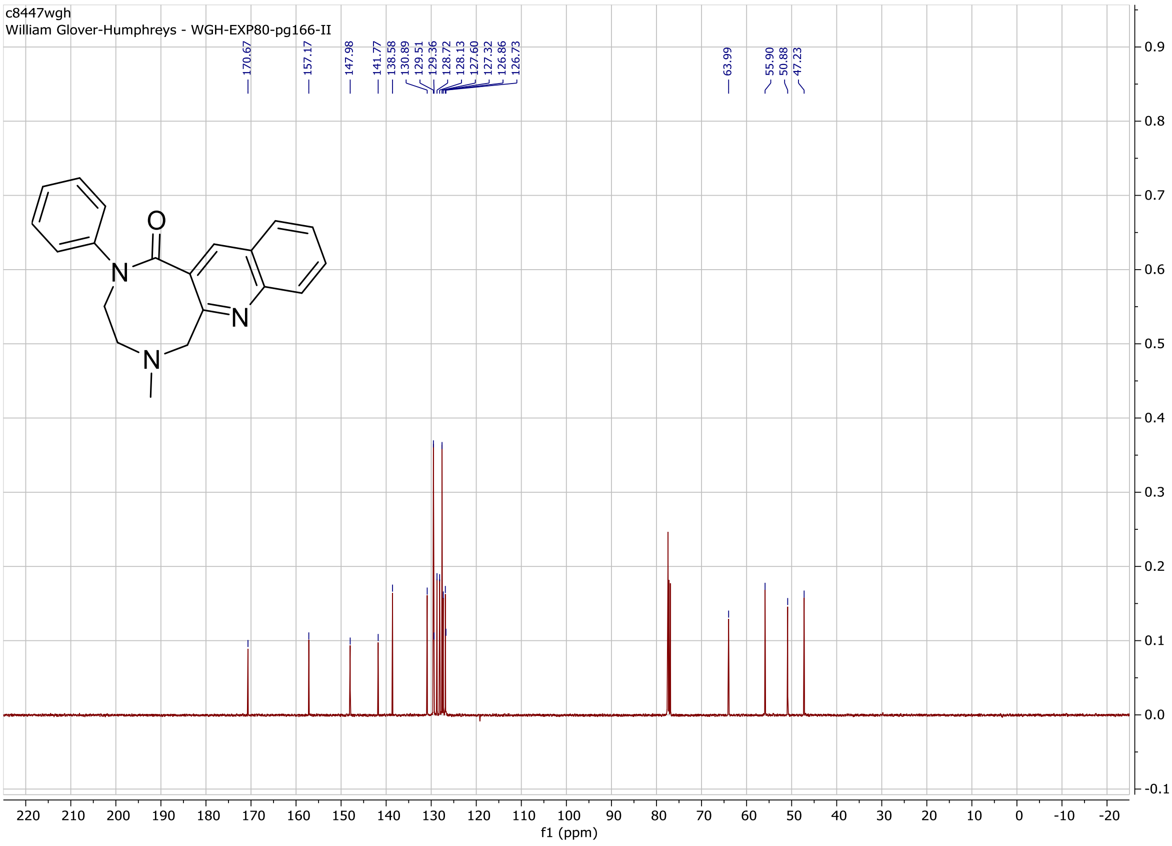

2-((Phenyl(2-(phenylamino)ethyl)amino)methyl)benzoic acid (S89)

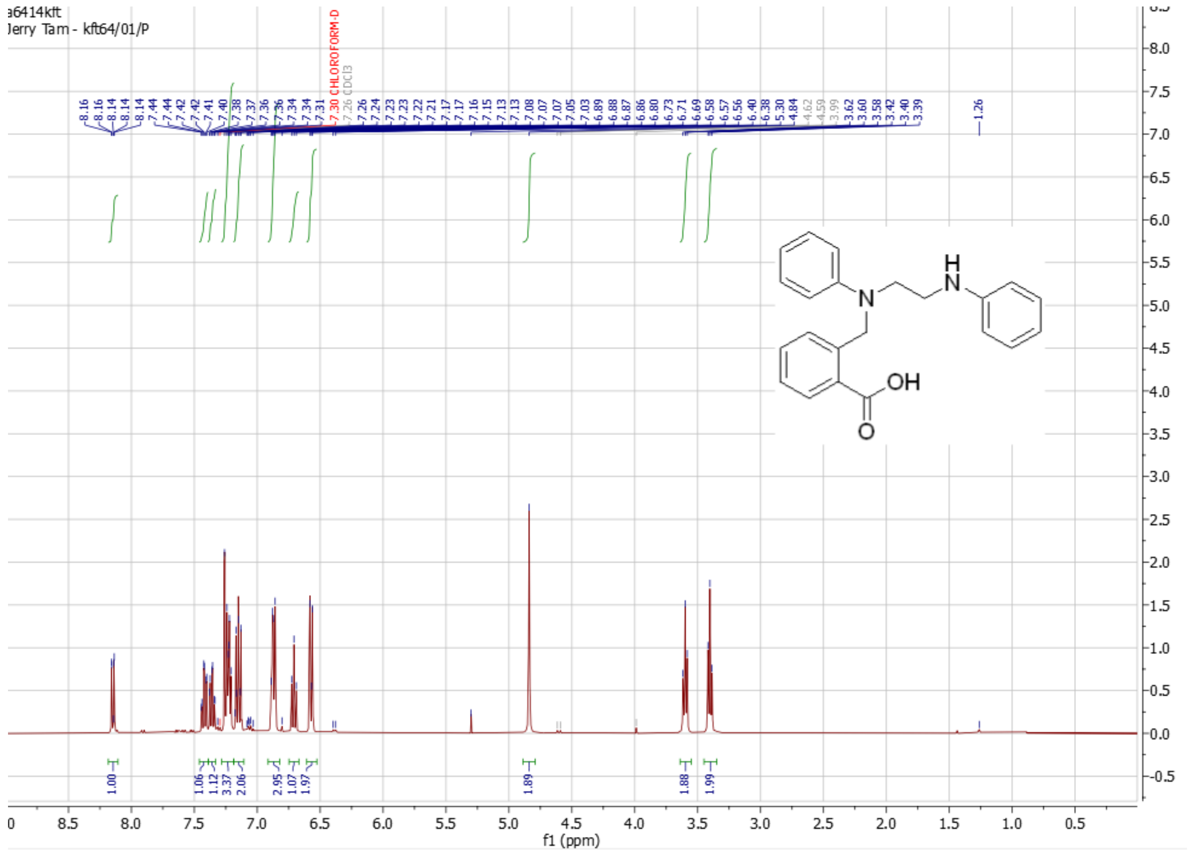

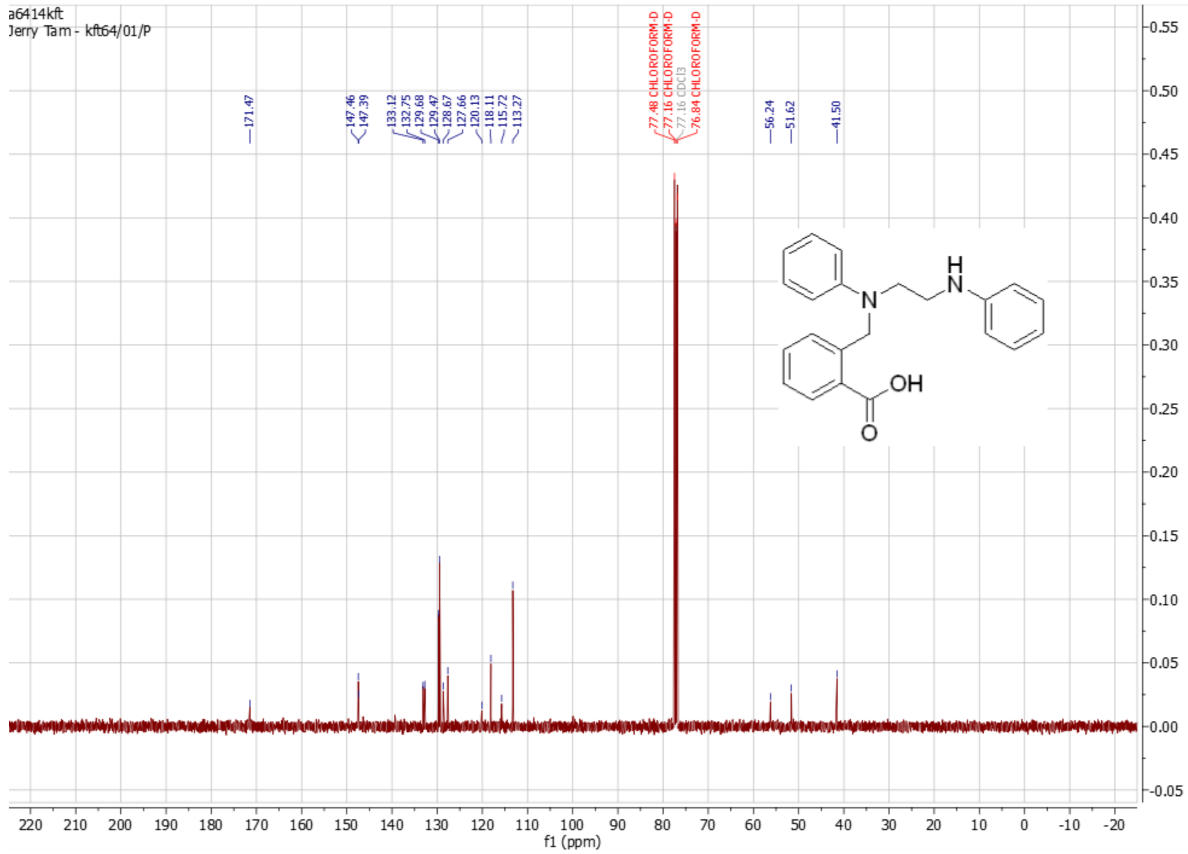

2,5-Diphenyl-3,4,5,6-tetrahydrobenzo[*f*][1,4]diazocin-1(2*H*)-one (71)

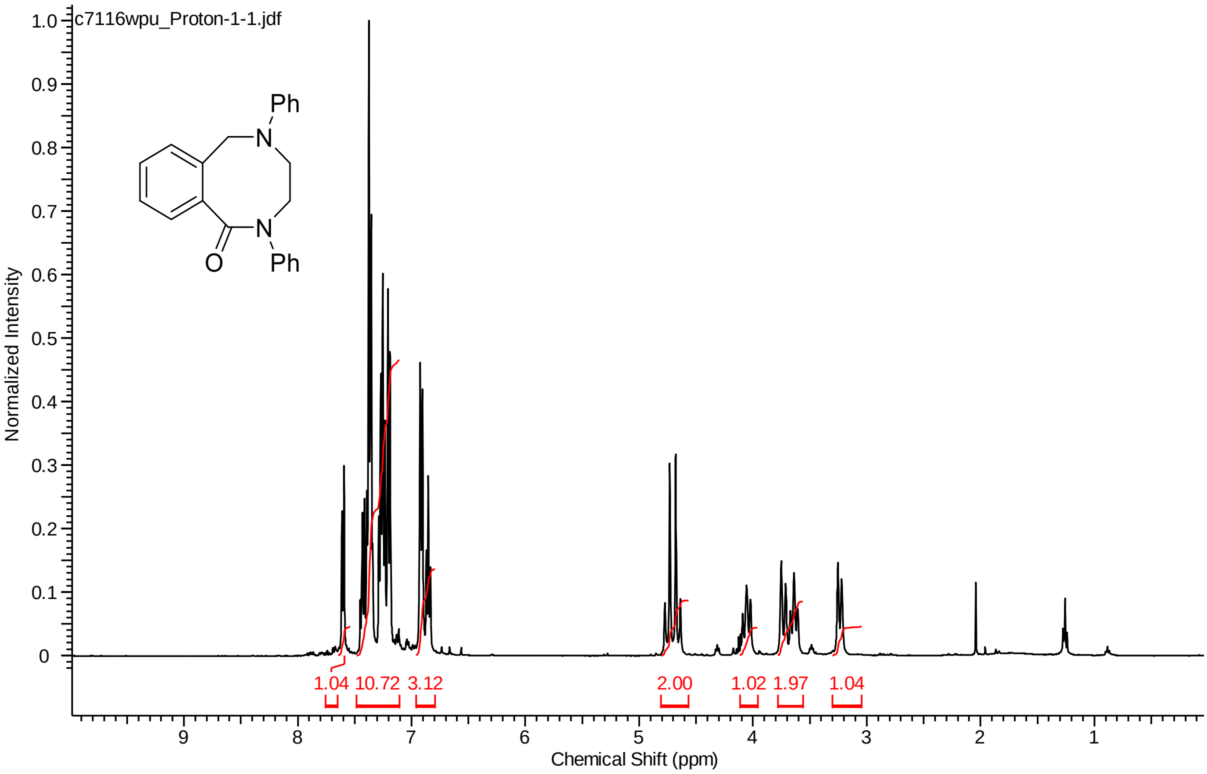

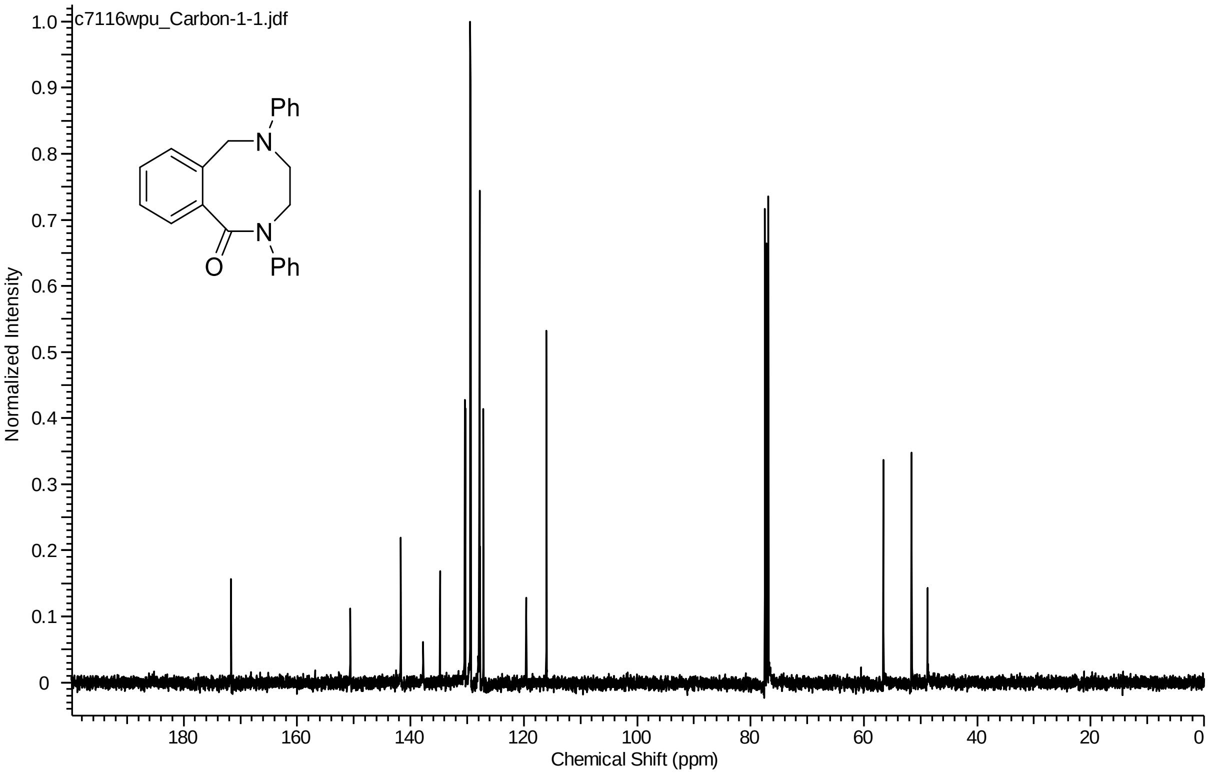

Benzyl 2-(3-(*tert*-butoxy)-3-oxopropyl) pyrrolidine-1-carboxylate (S90)

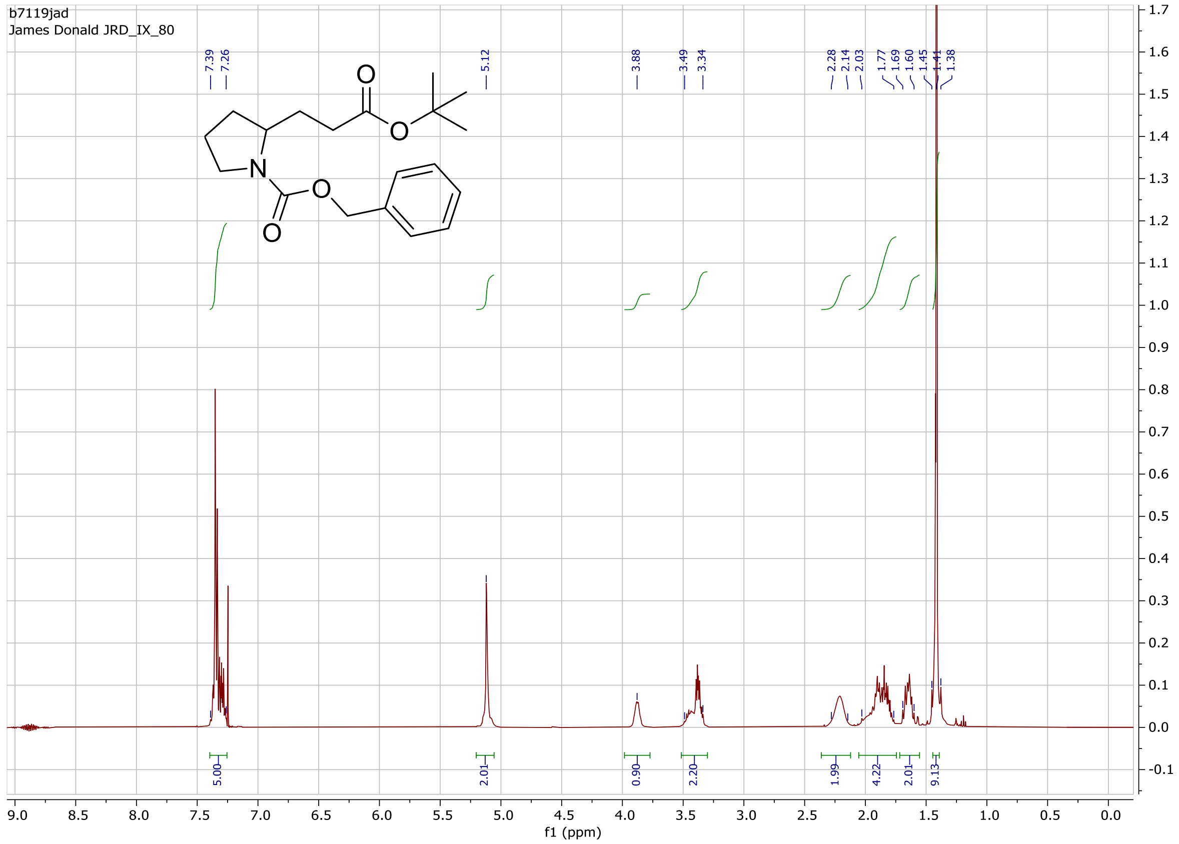

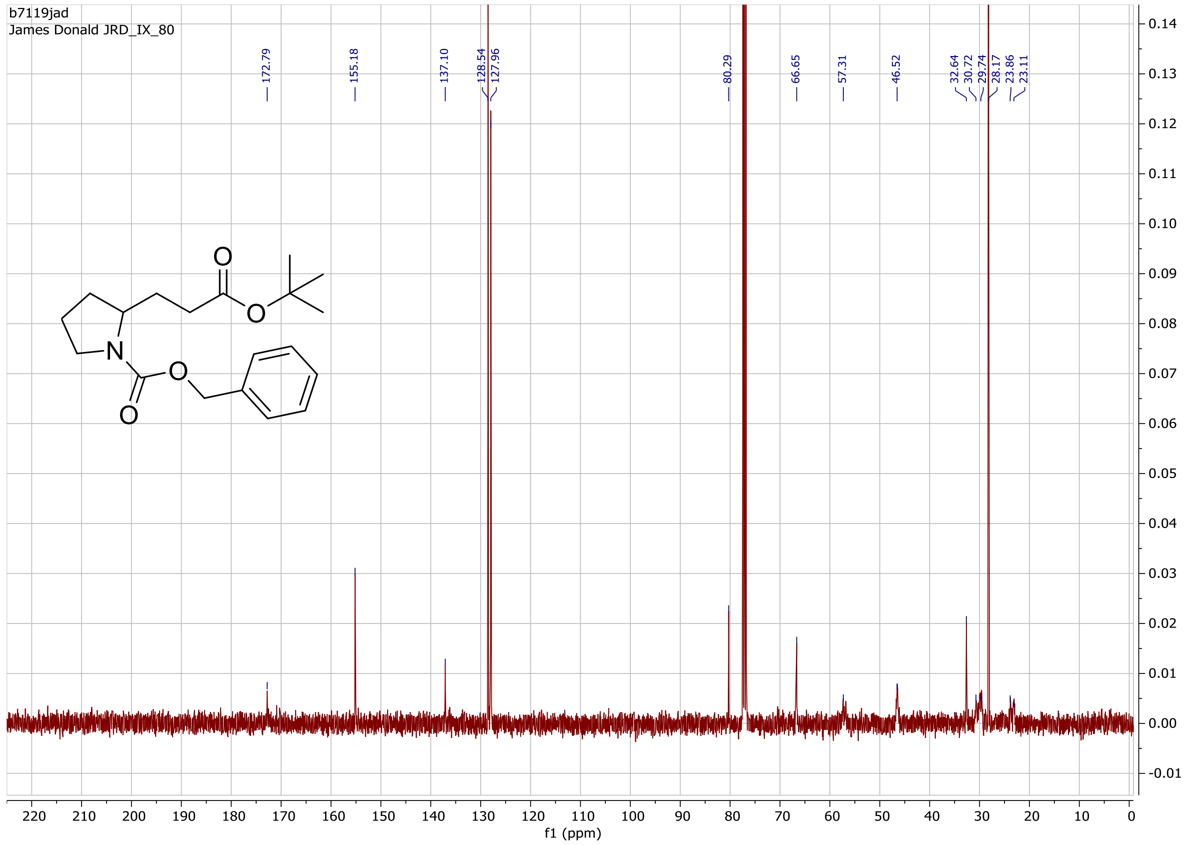

tert-Butyl 3-(1-(3-((*tert*-butoxycarbonyl) amino) propyl) pyrrolidin-2-yl) propanoate (S91)

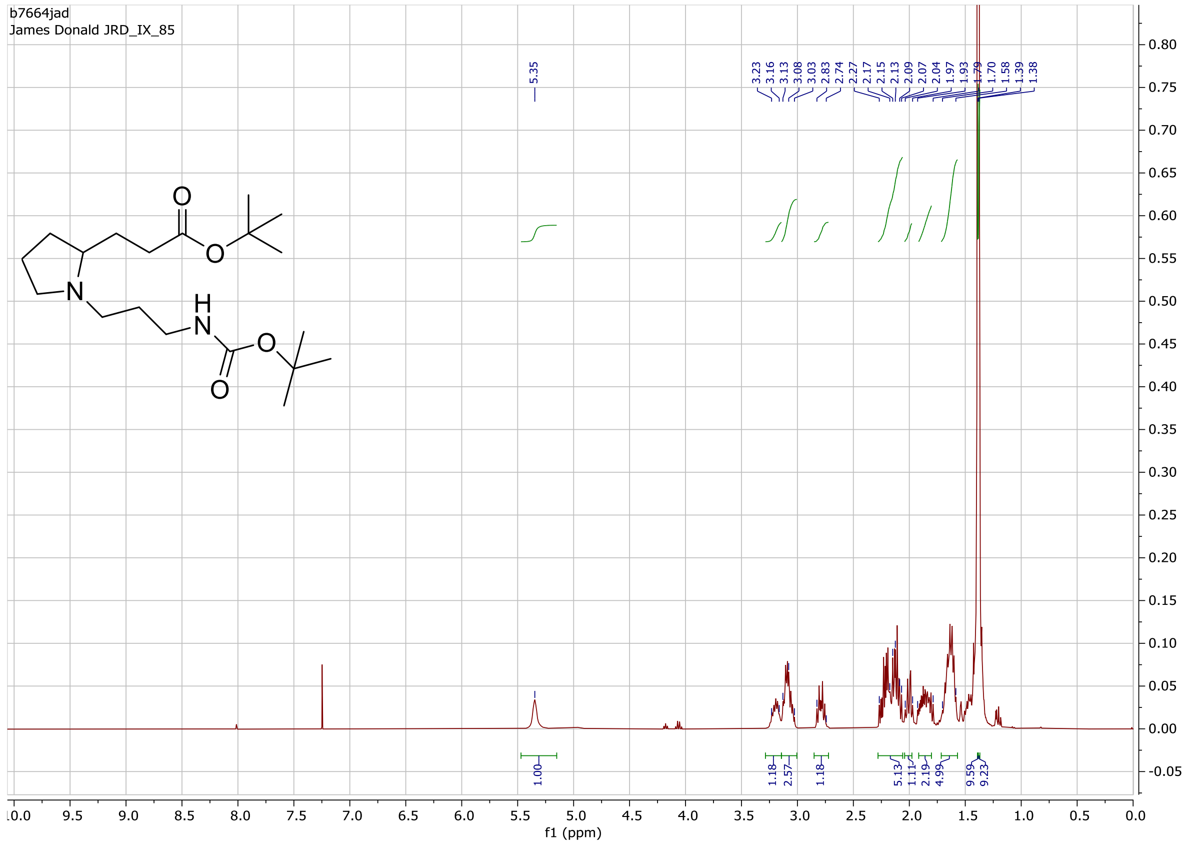

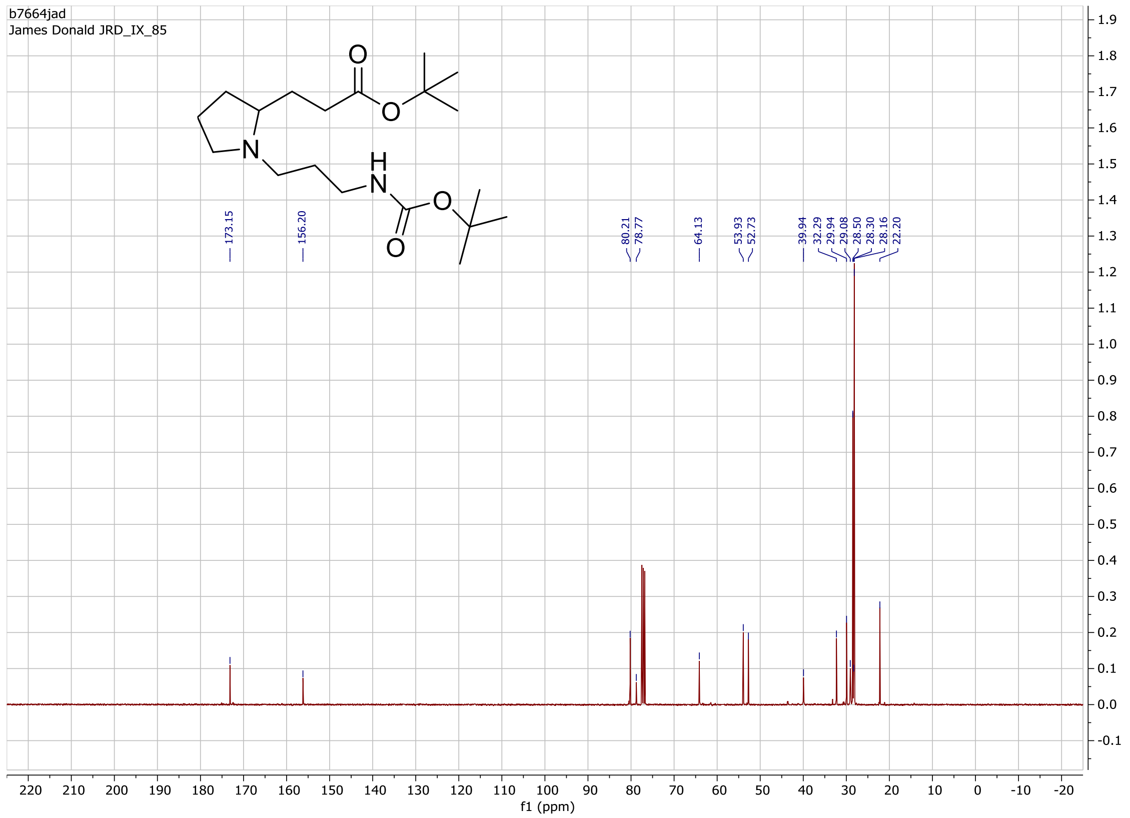

Decahydro-9H-pyrrolo[1,2-e] [1,5] diazonin-9-one (72) In solution in $CDCl_3$, compound **72** esists as a ≈3:1 mixture of rotamers

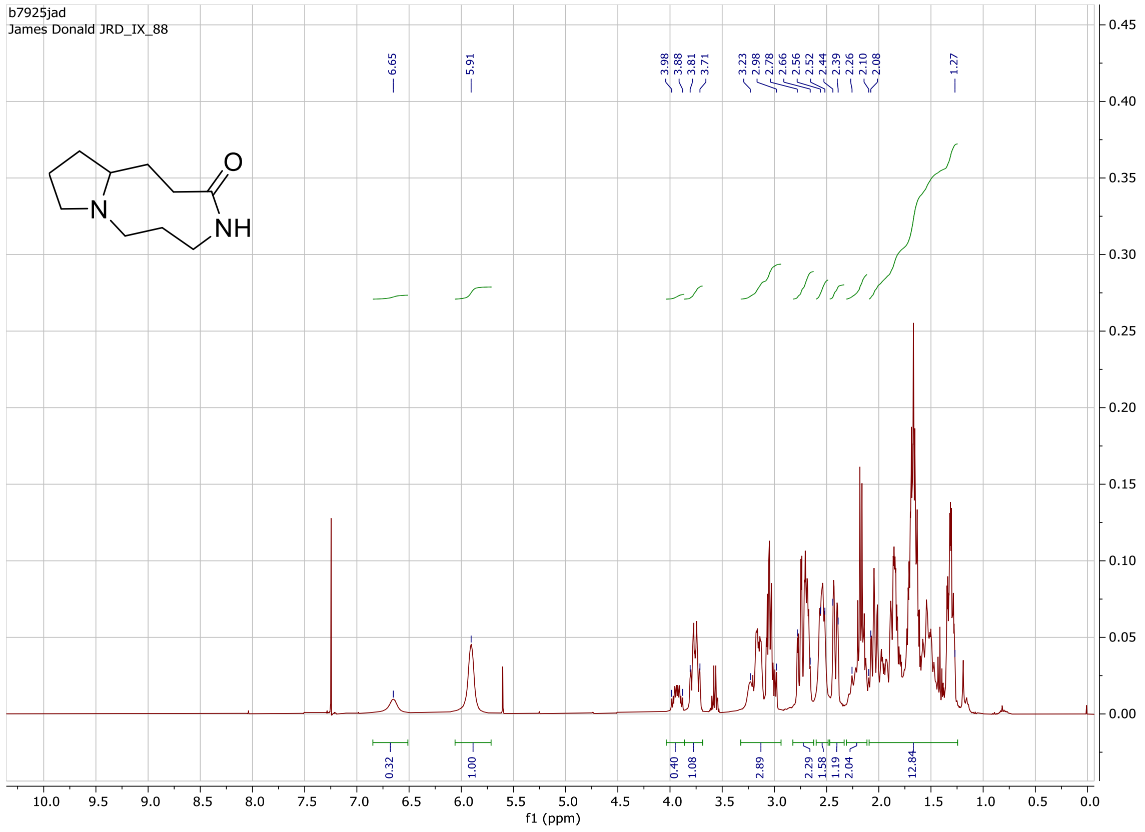

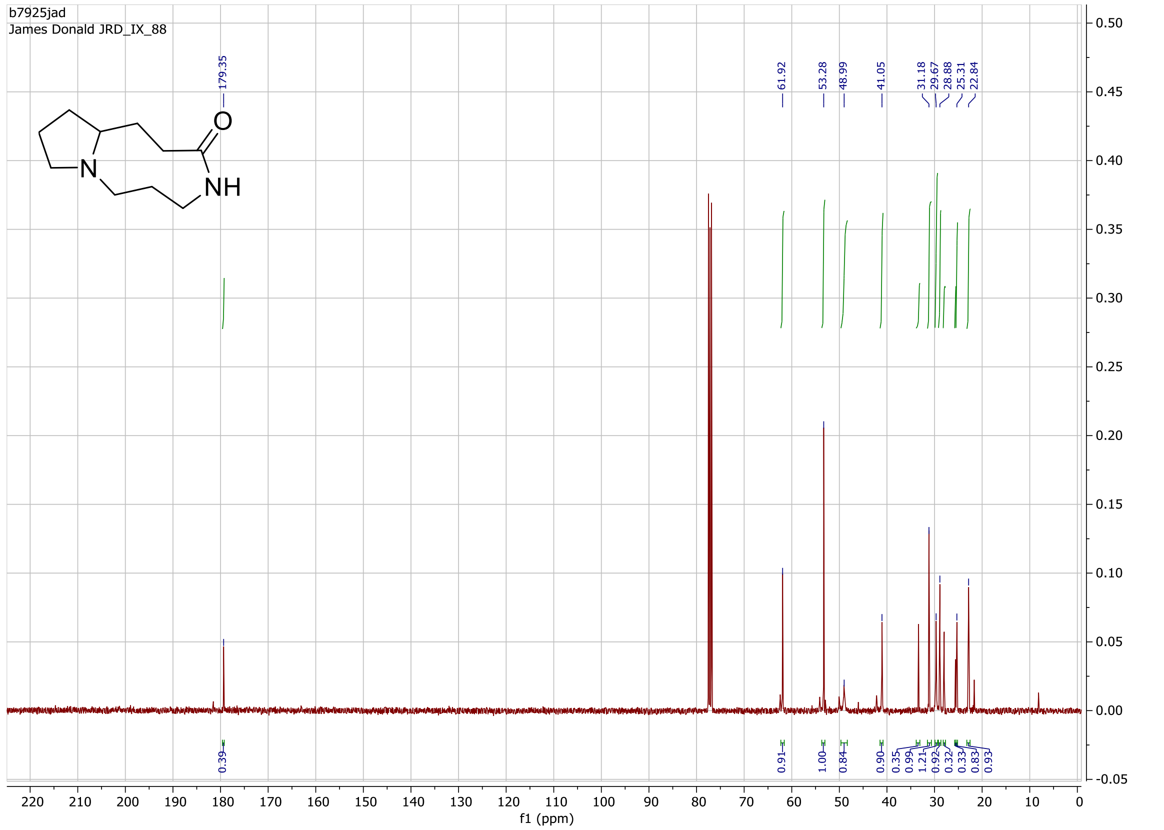

1-[(Benzyloxy) carbonyl] piperidine-2-carboxylic acid (S92)

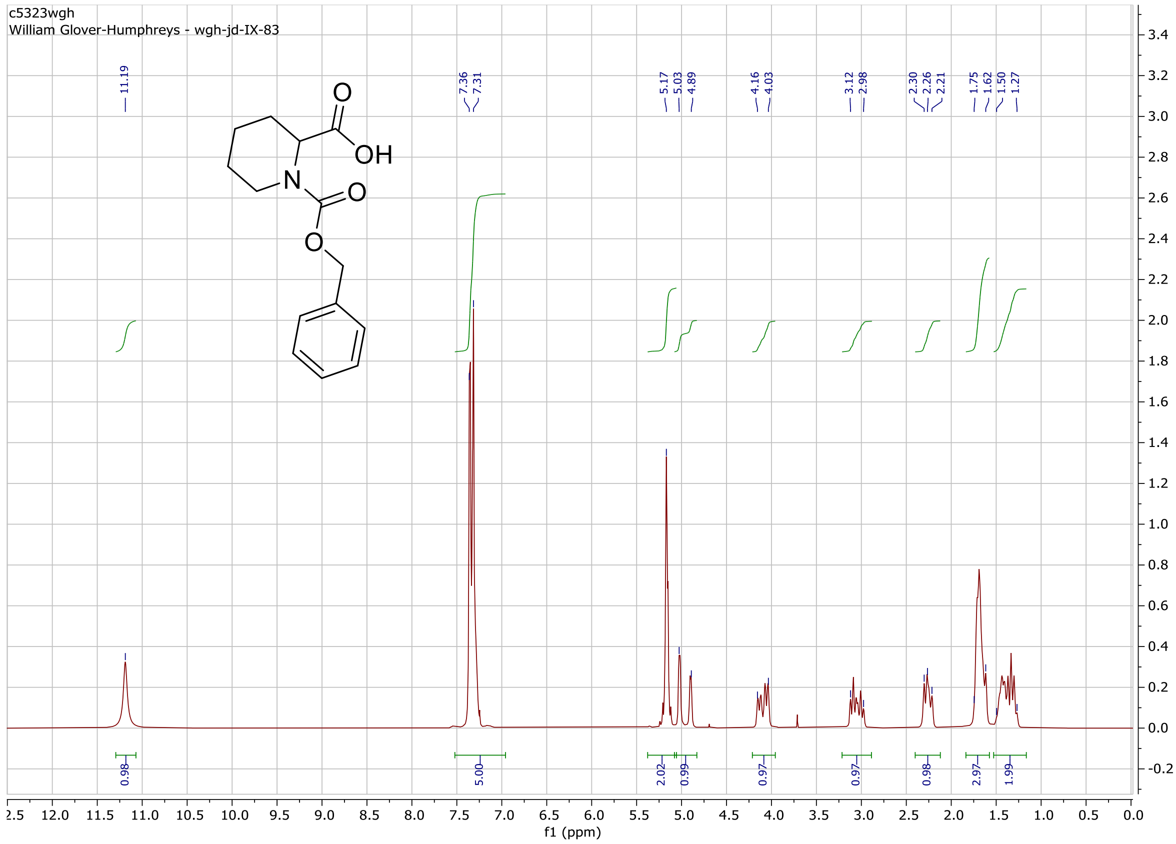

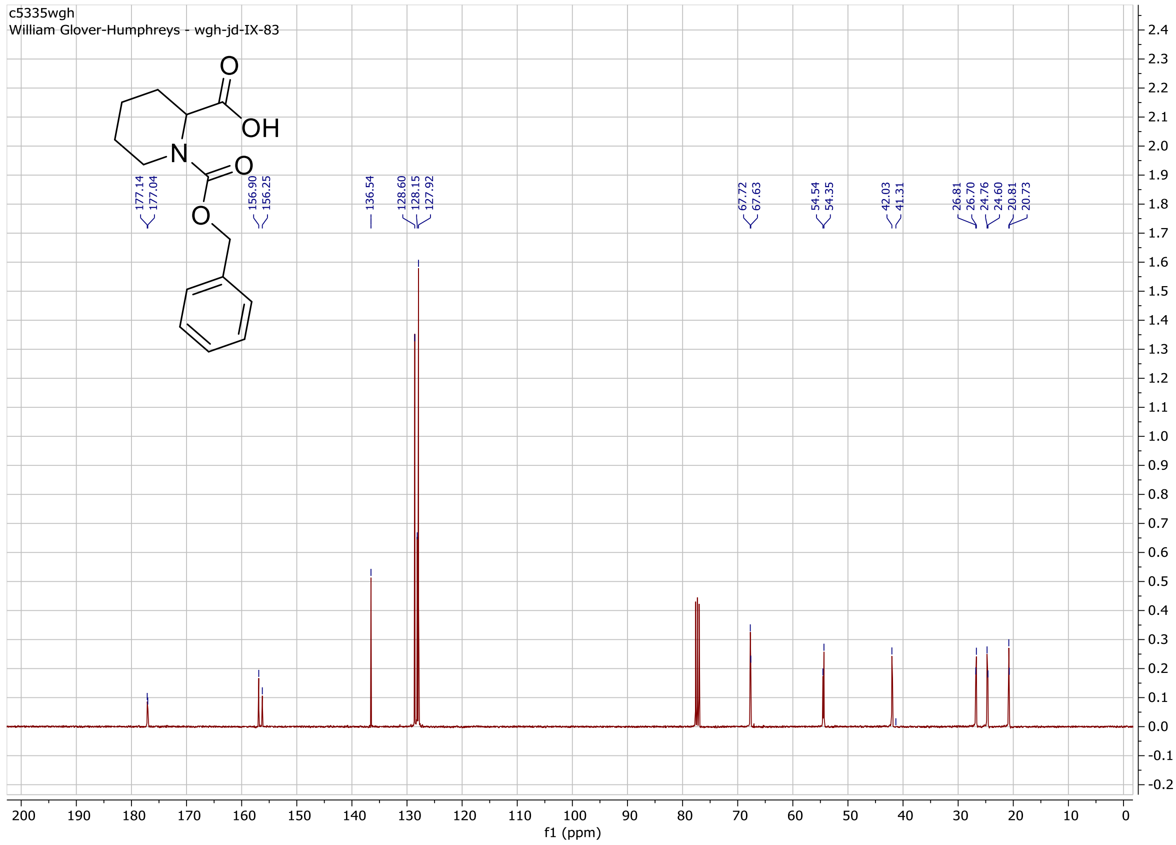

Benzyl 2-(3-*tert*-butoxy-3-oxopropyl) piperidine-1-carboxylate (S93)

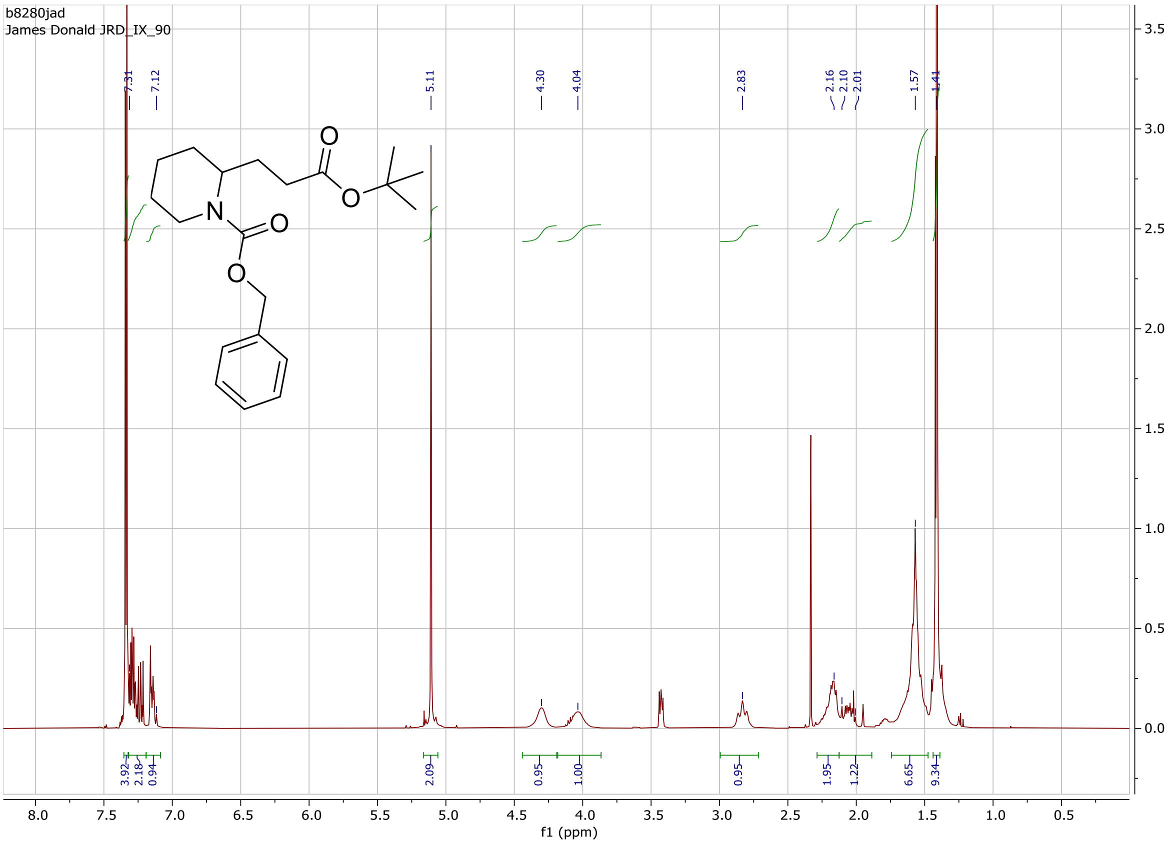

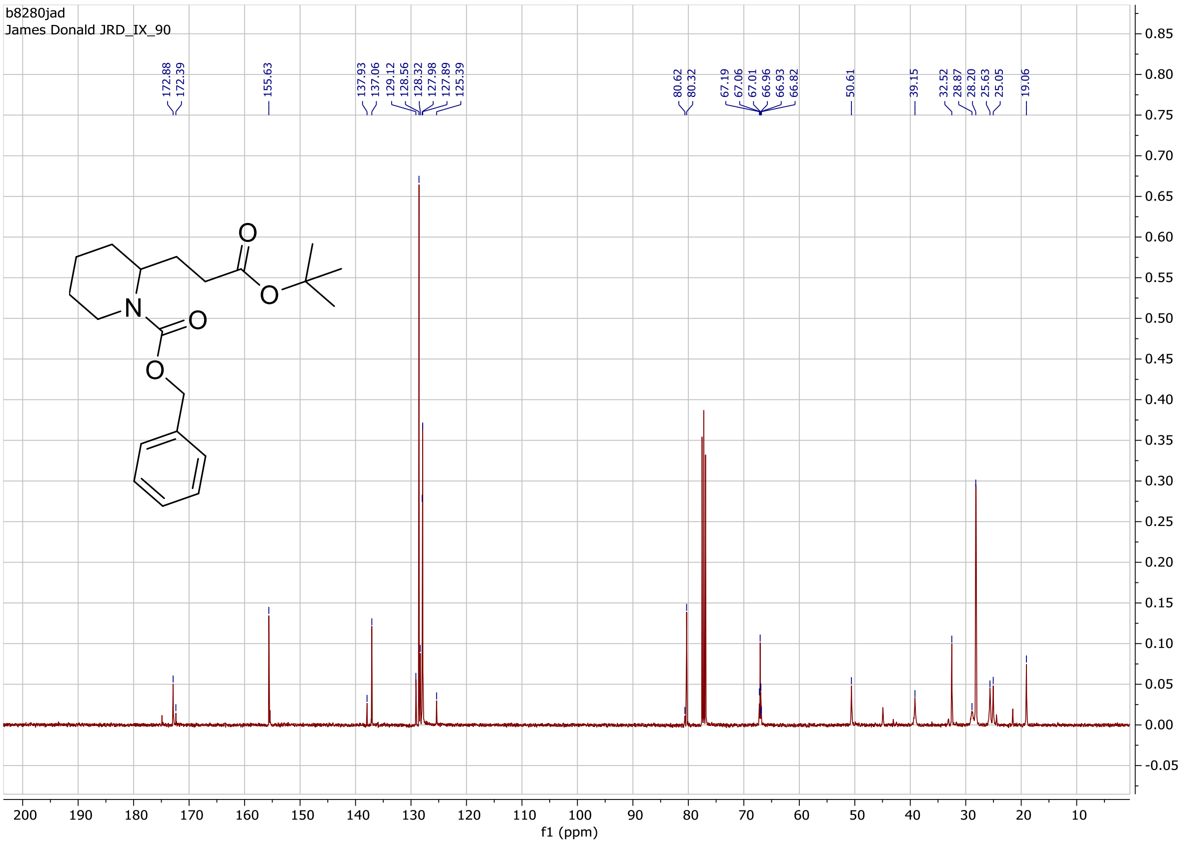

tert-Butyl 3-(1-(3-((tert-butoxycarbonyl) amino) propyl) piperidin-2-yl) propanoate (S94)

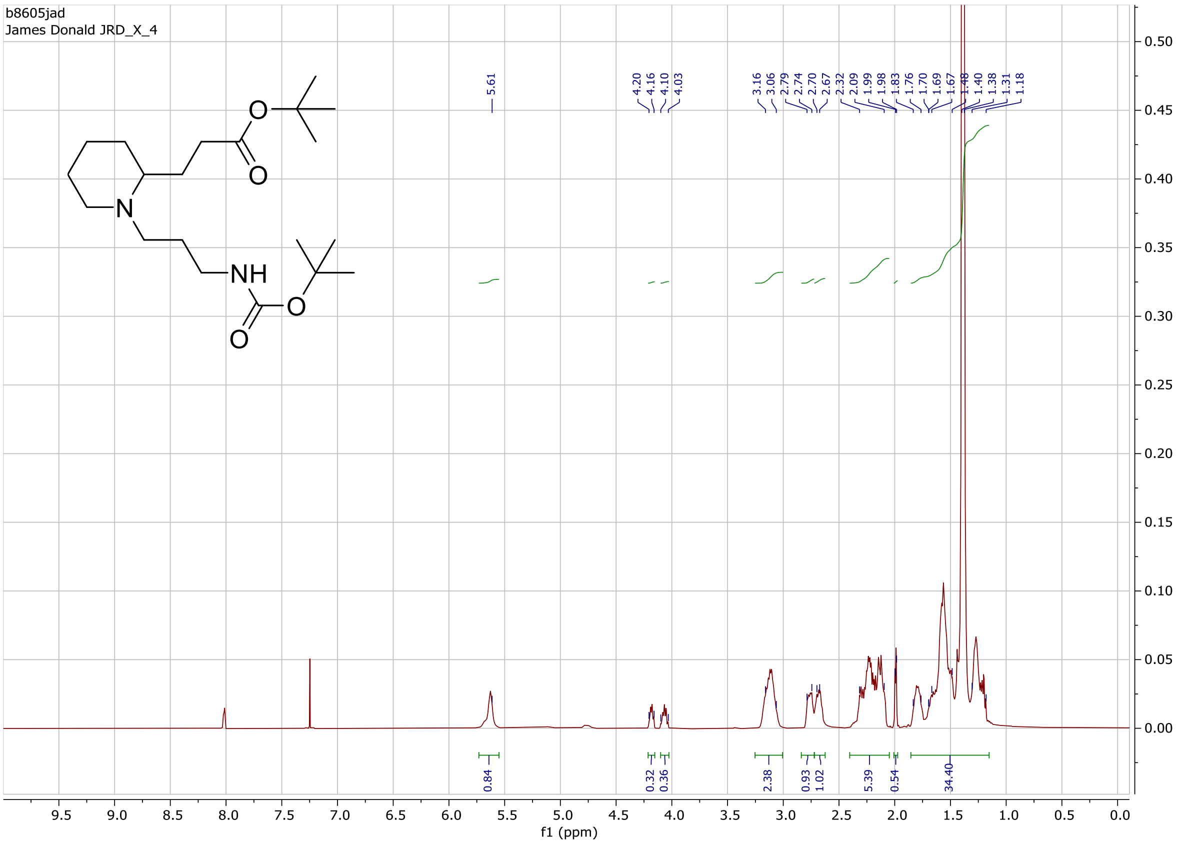

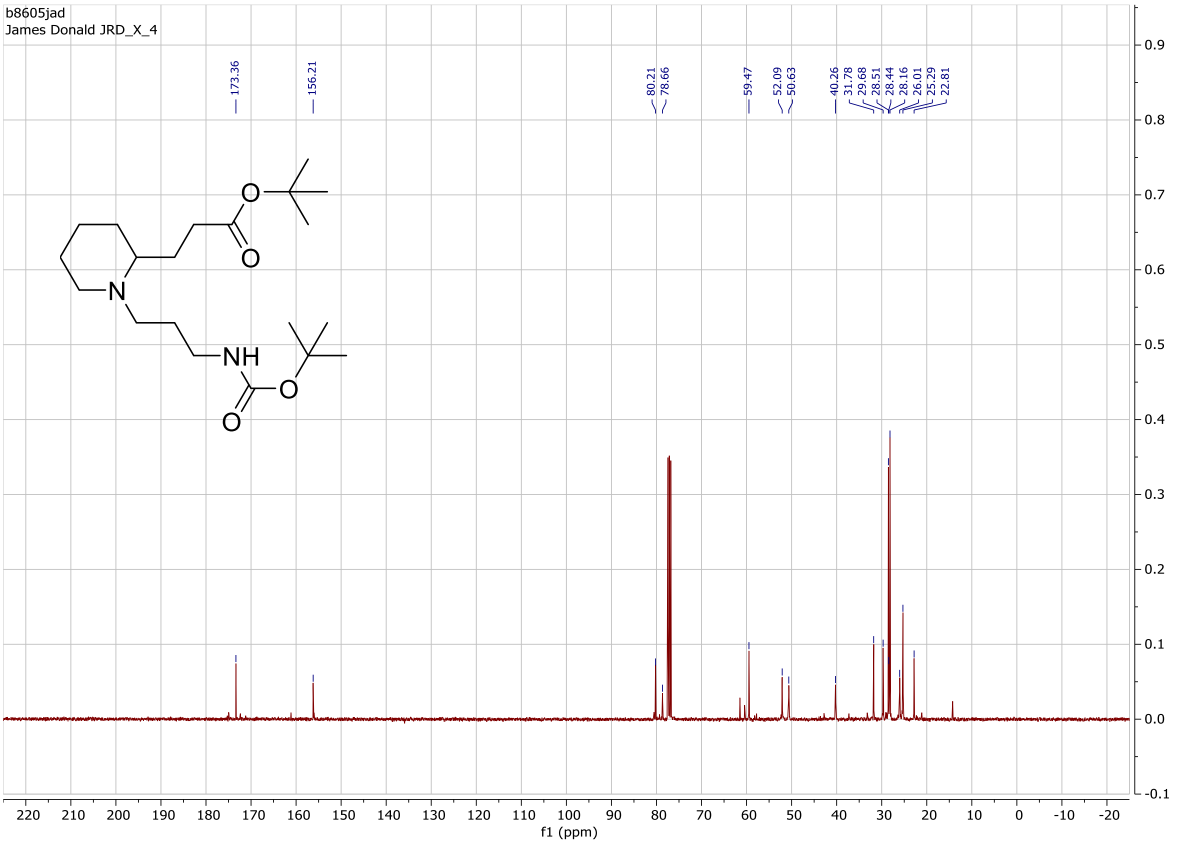

Decahydropyrido[1,2-e] [1,5] diazonin-3(2H)-one (73)

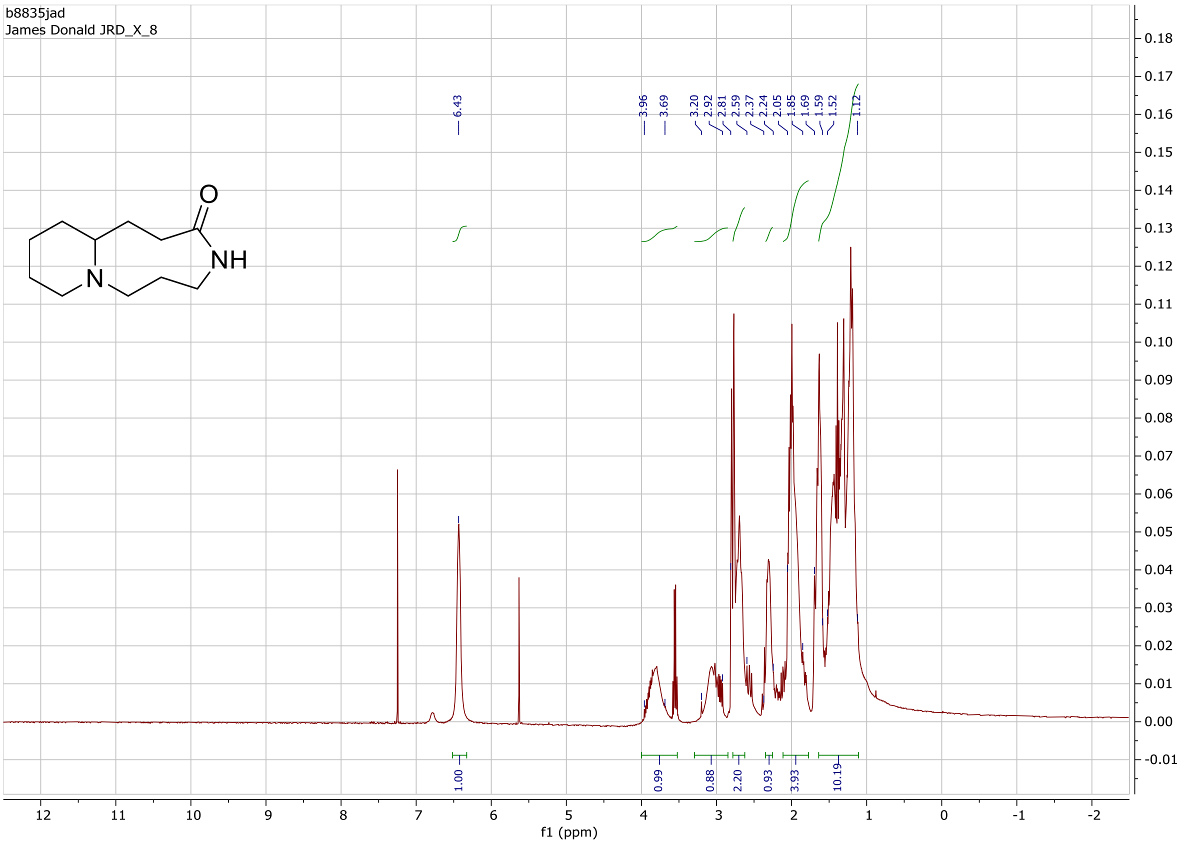

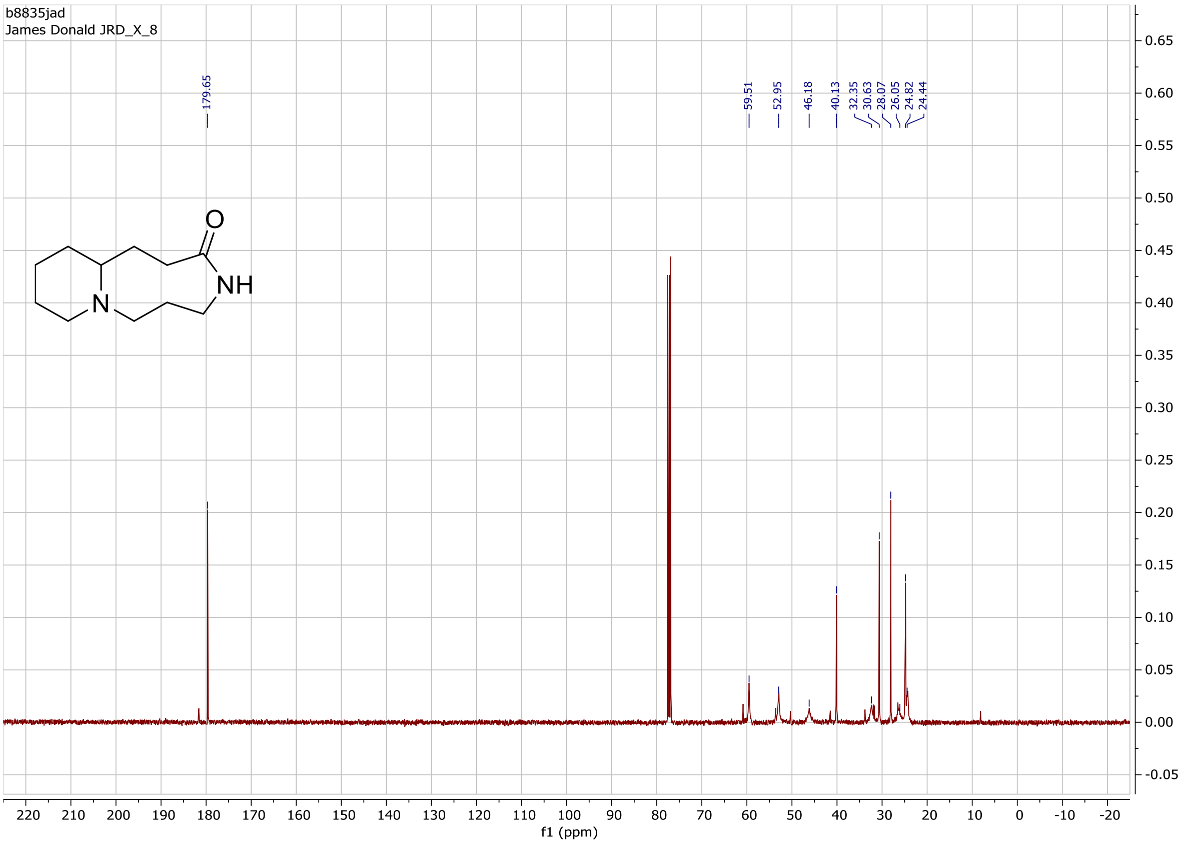

Methyl 2-(((3-azidopropyl)thio)methyl)benzoate (S95)

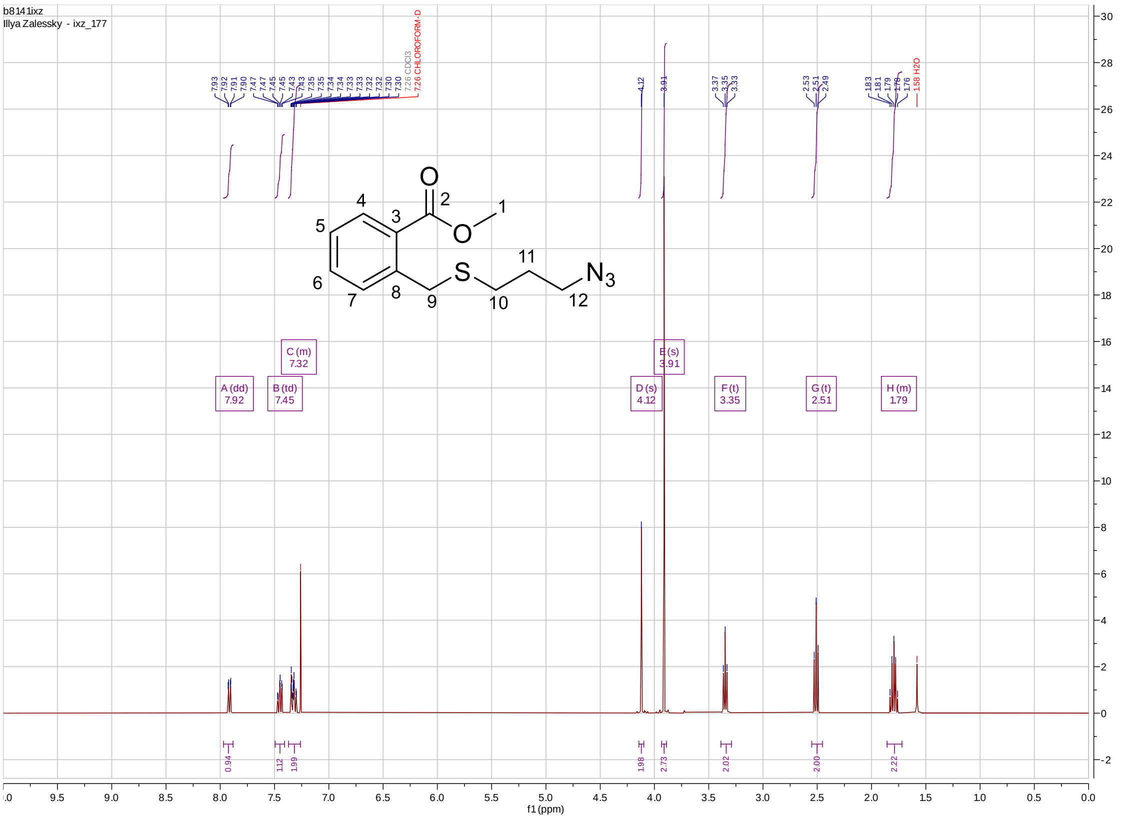

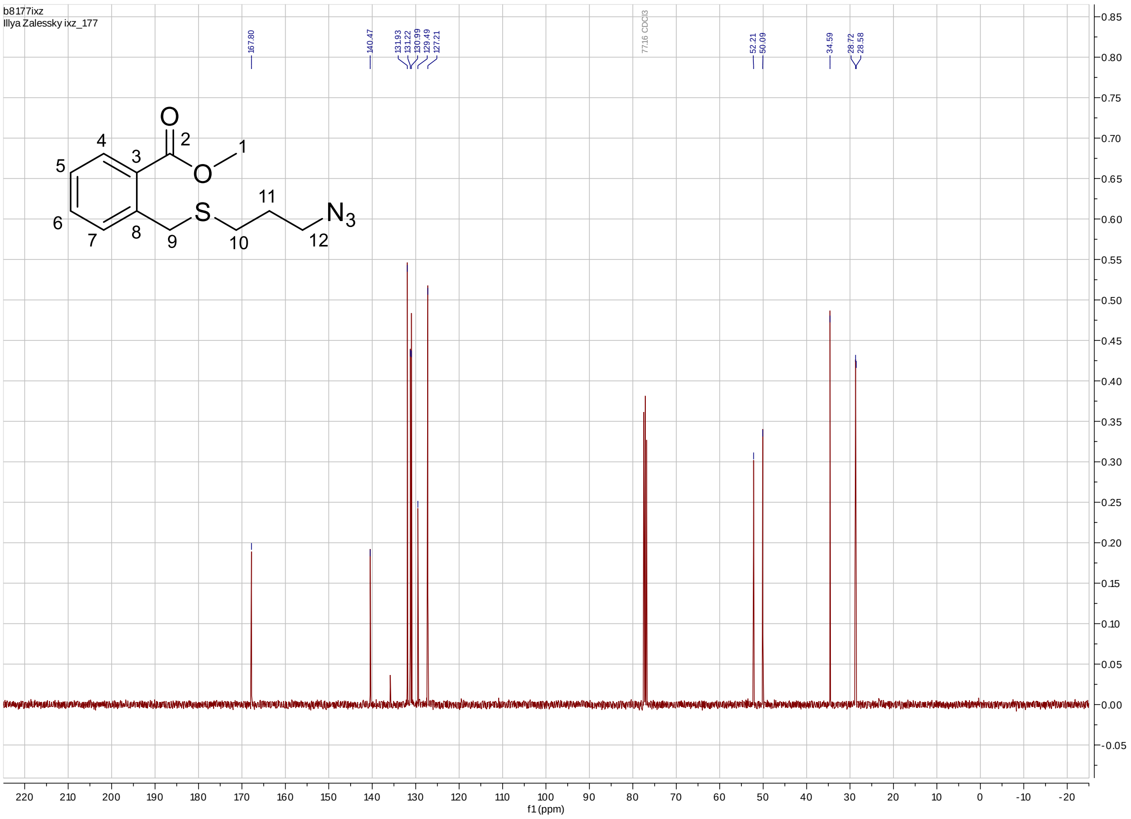

2-(((3-Aminopropyl)thio)methyl)benzoic acid (S97)

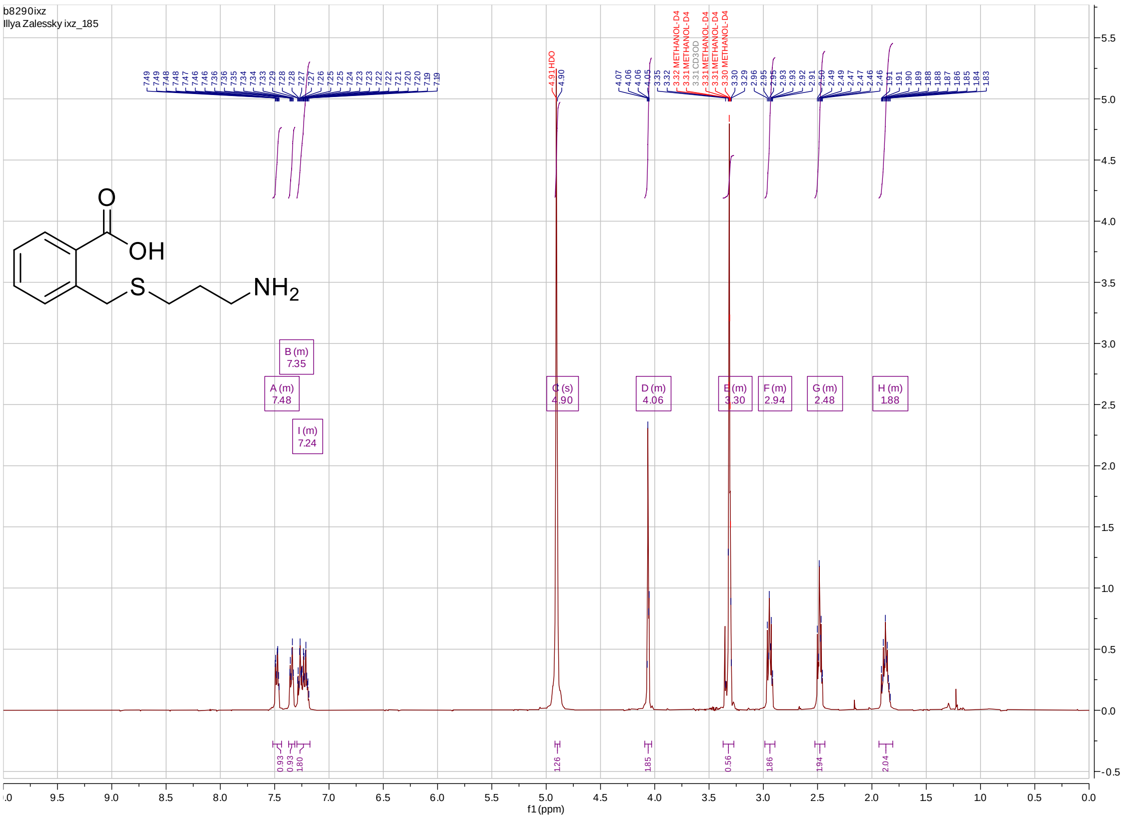

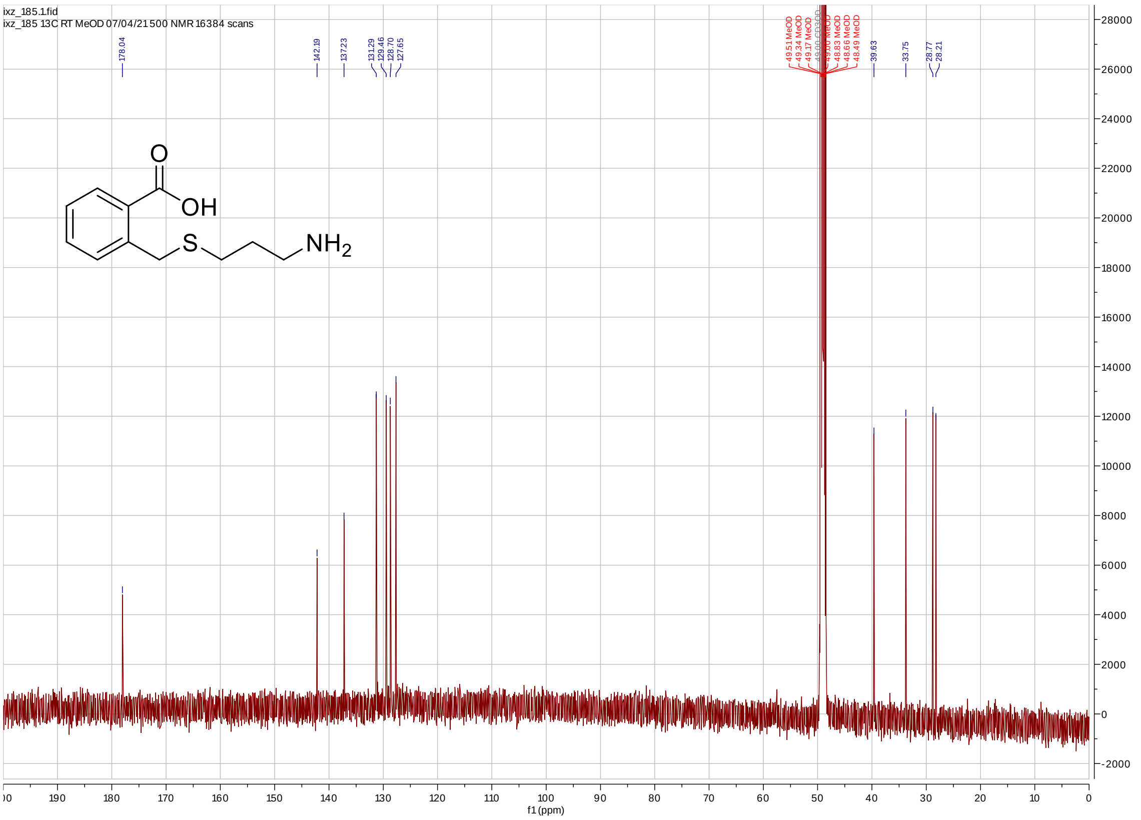

3,4,5,6-Tetrahydrobenzo[g][1,5]thiazonin-7(1H)-one (74)

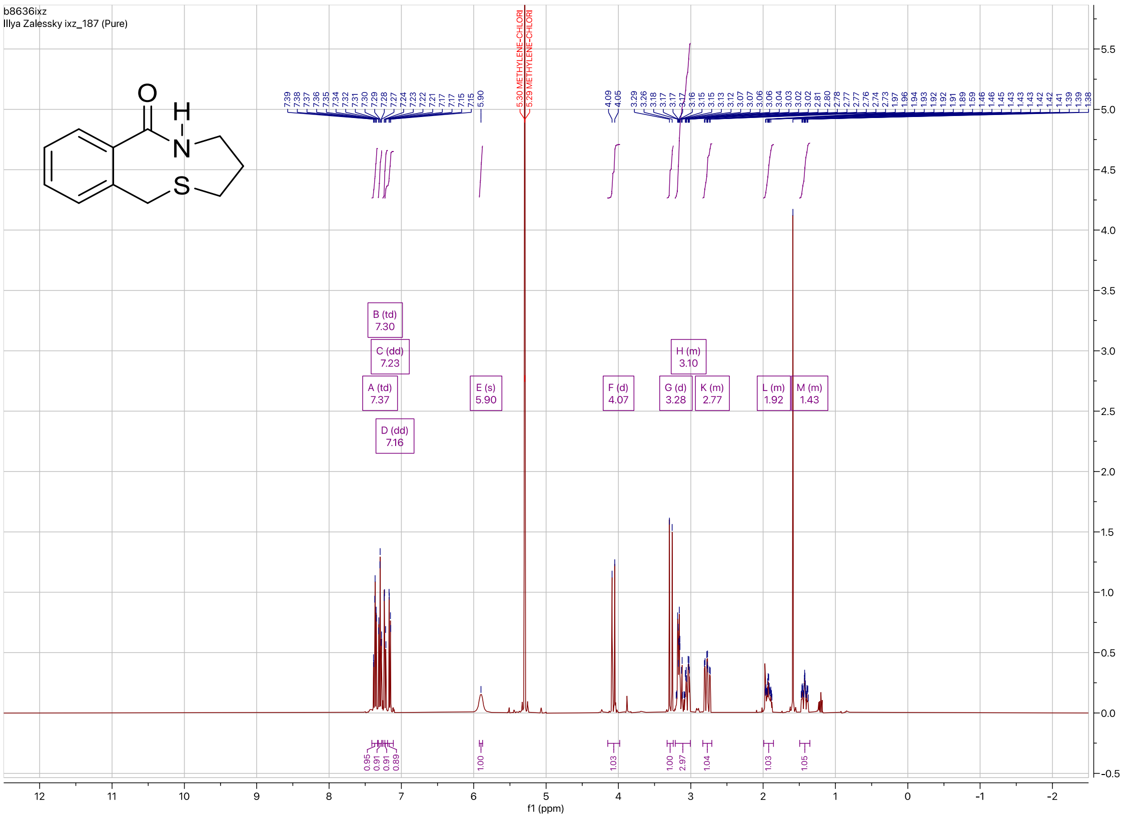

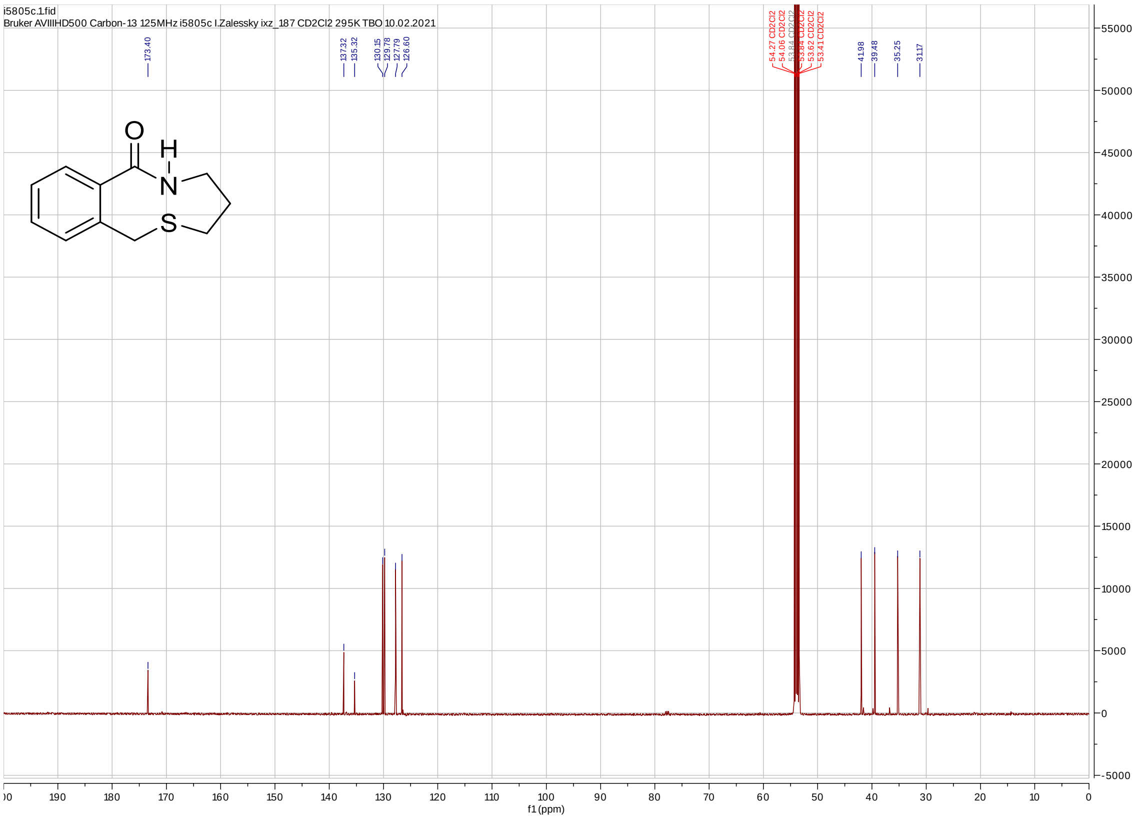

Methyl 2-(((3-bromopropyl)thio)methyl)benzoate (S98)

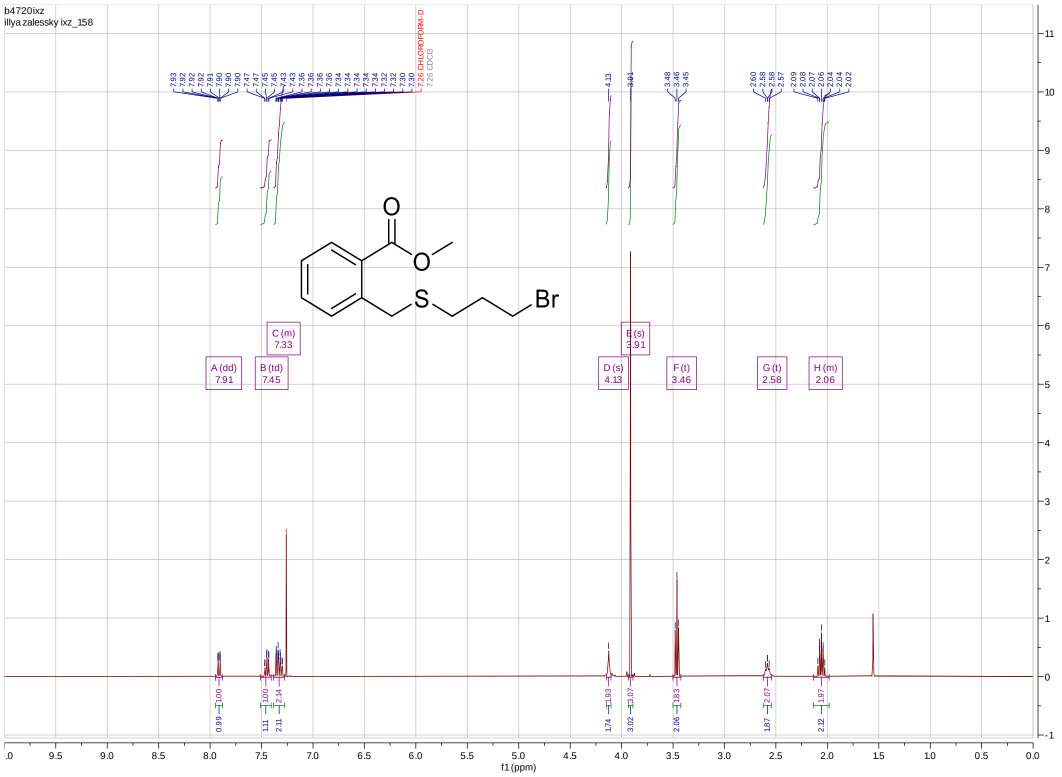

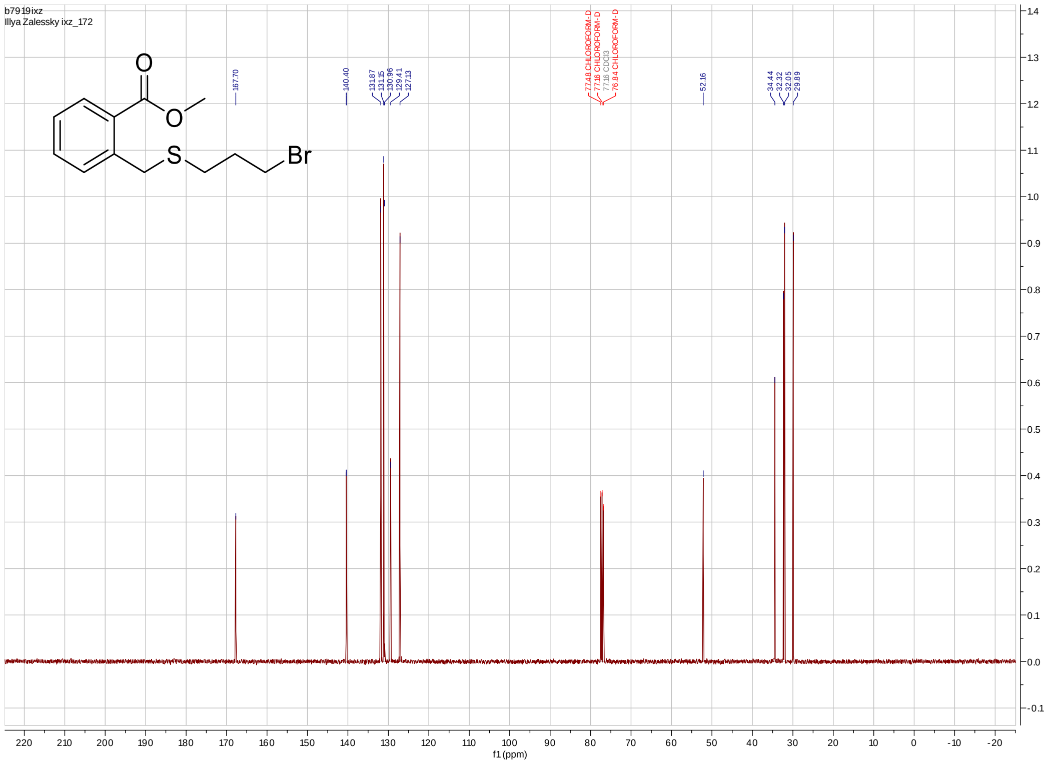

Methyl 2-(((3-(phenylamino)propyl)thio)methyl)benzoate (S99)

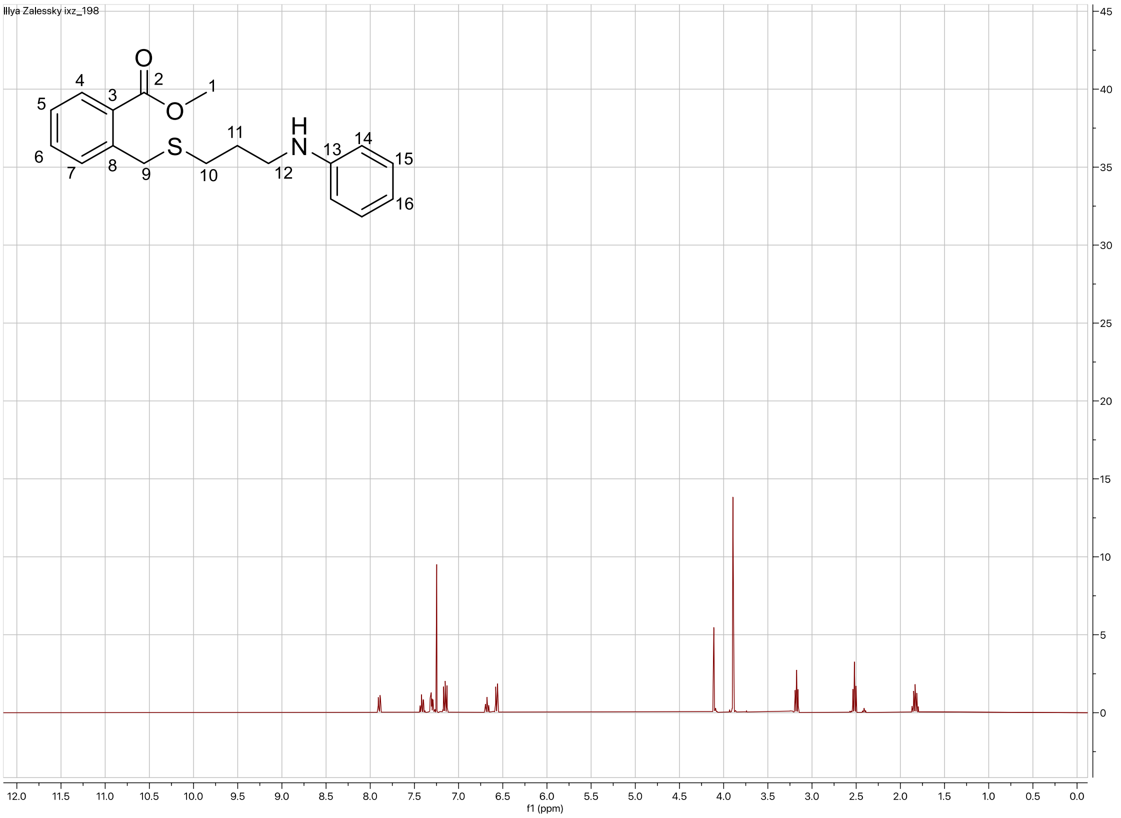

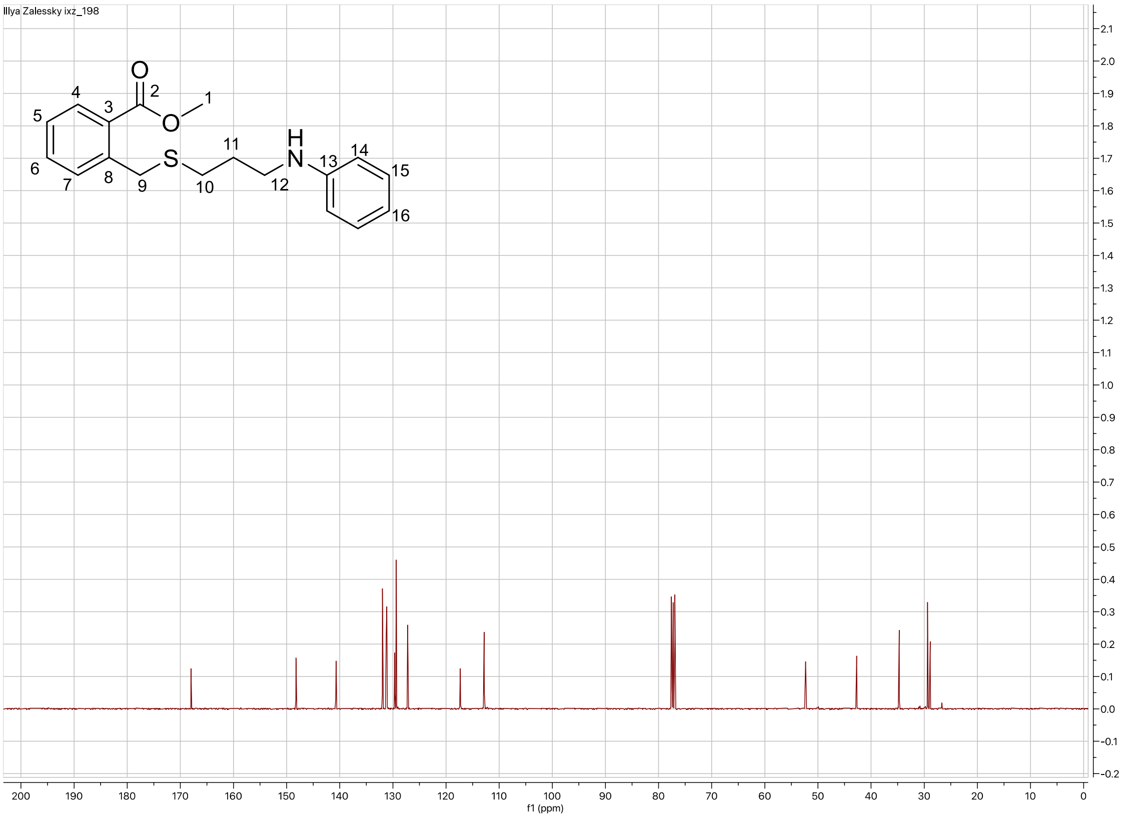

2-(((3-(Phenylamino)propyl)thio)methyl)benzoic acid (S100)

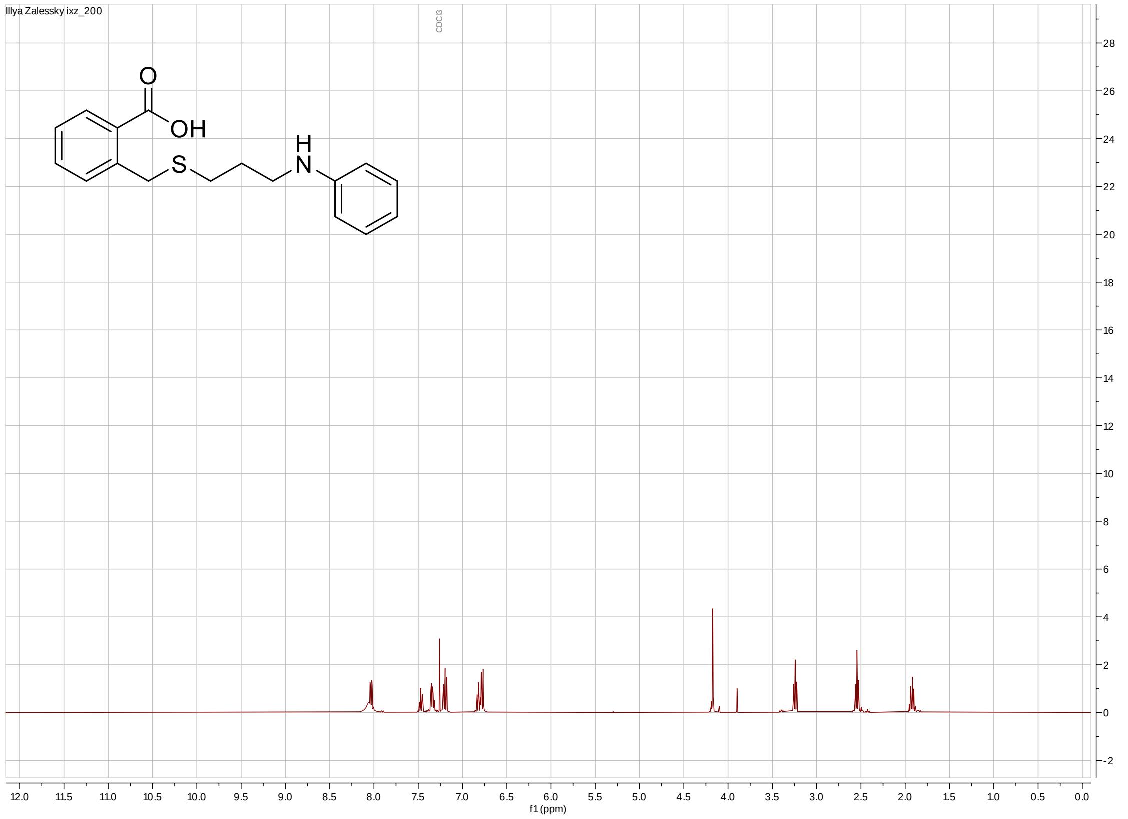

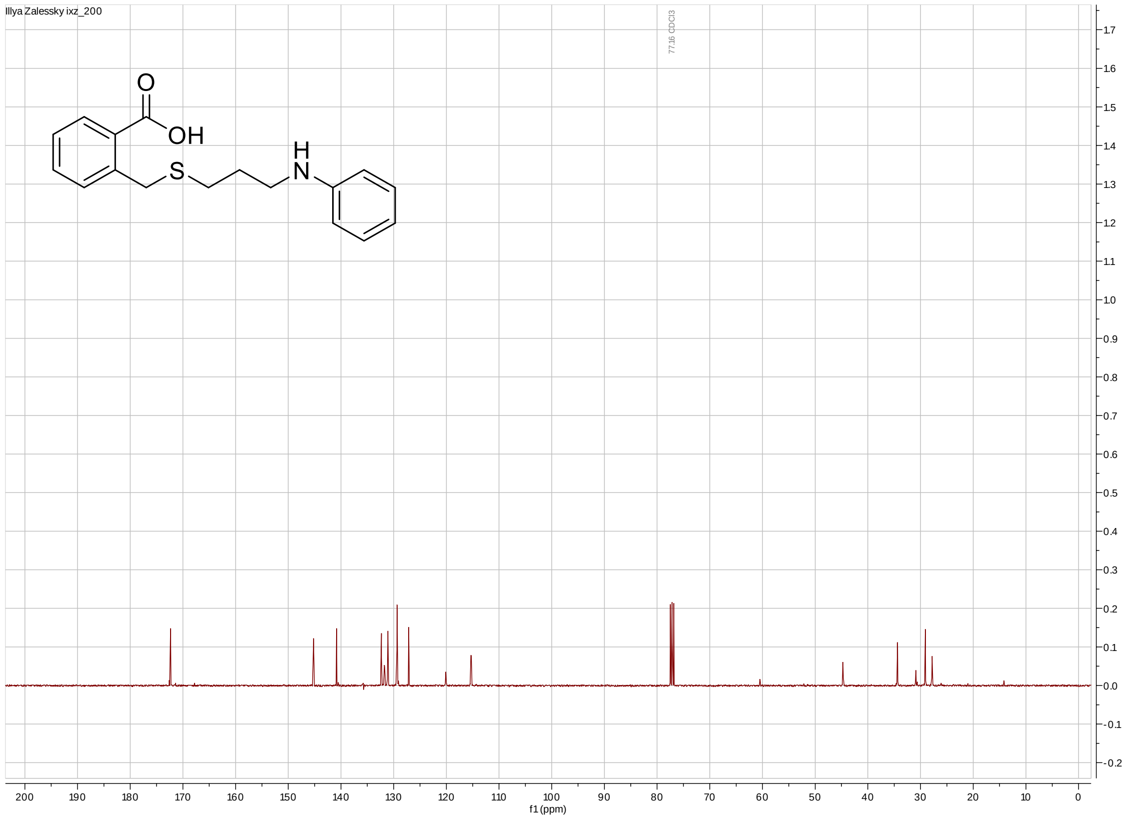

6-Phenyl-3,4,5,6-tetrahydrobenzo[*g*][1,5]thiazonin-7(1*H*)-one (75)

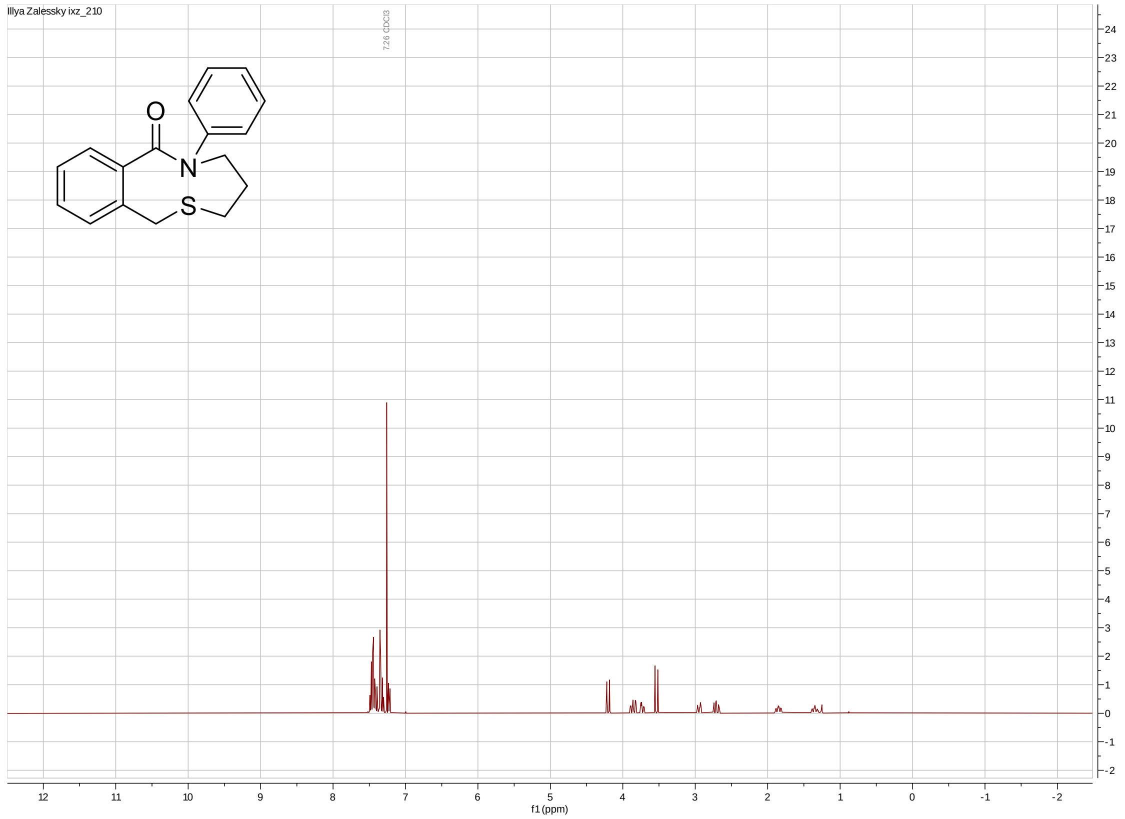

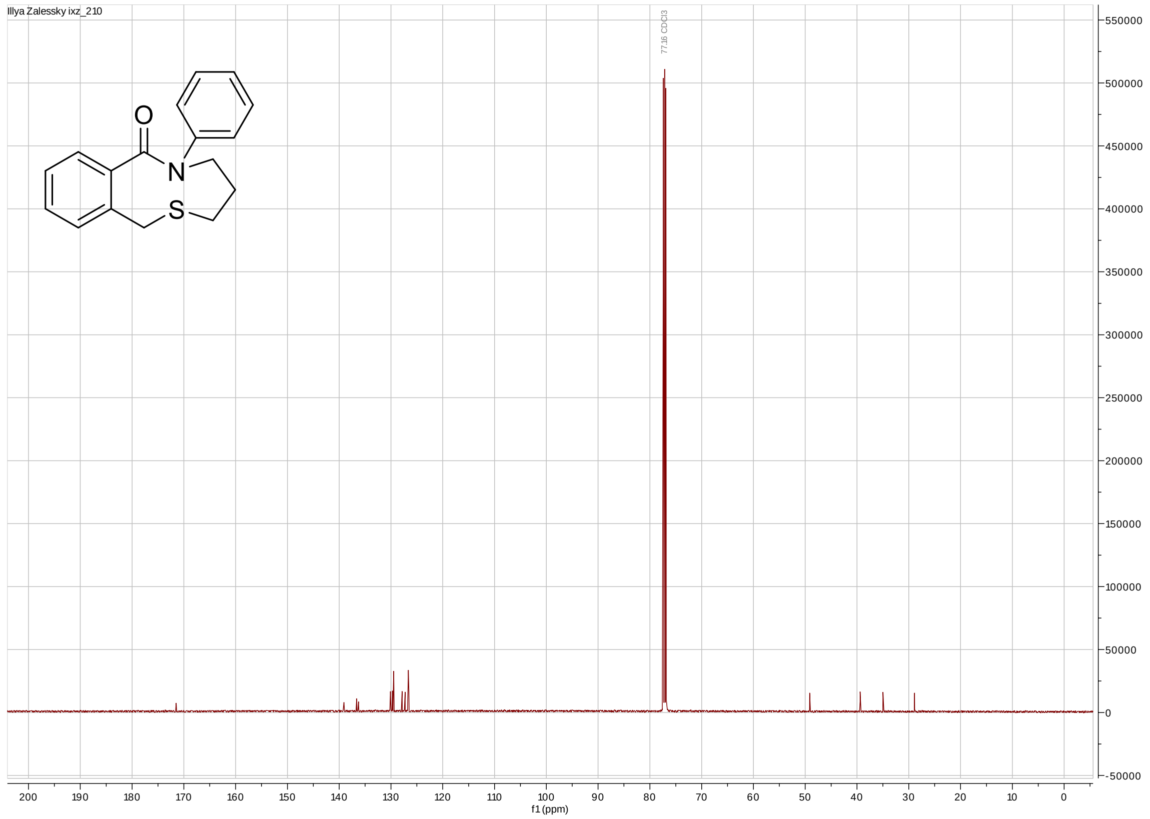

Methyl 2-((methyl(2-(phenylamino) ethyl) amino) methyl) benzoate (S101)

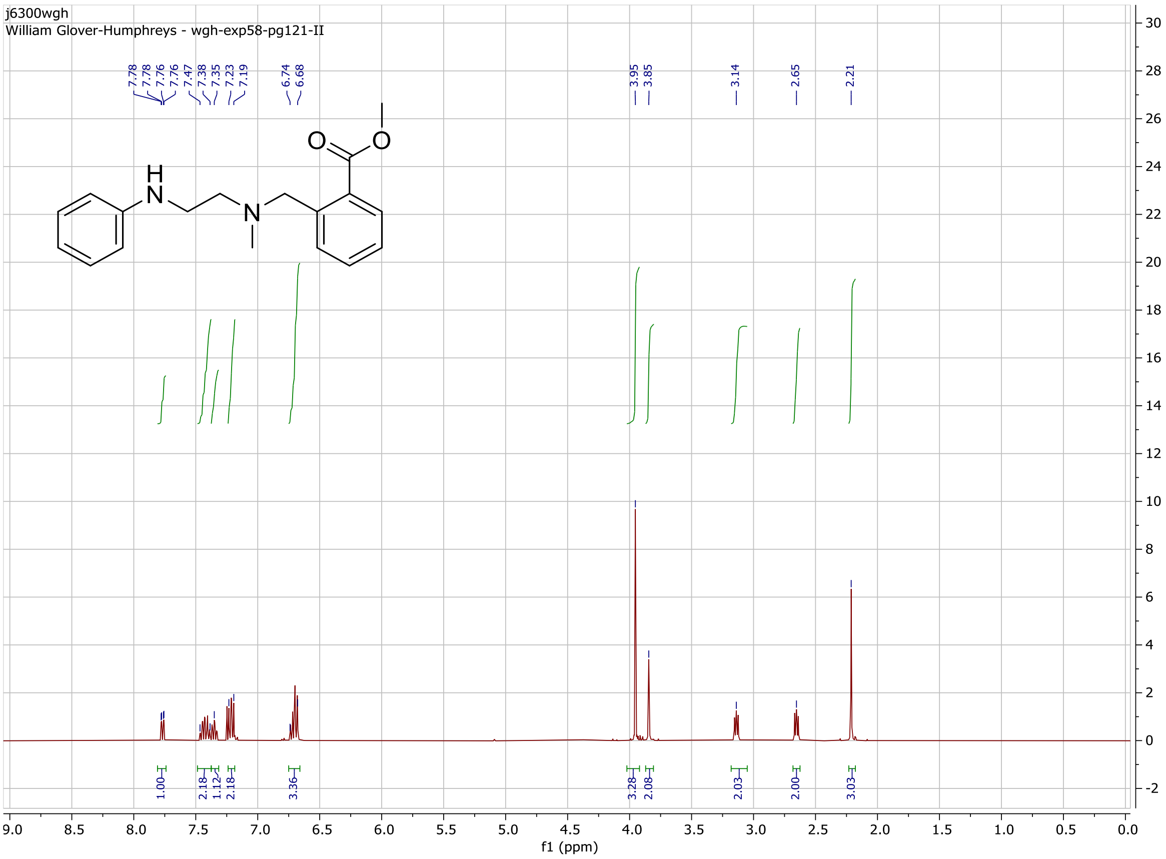

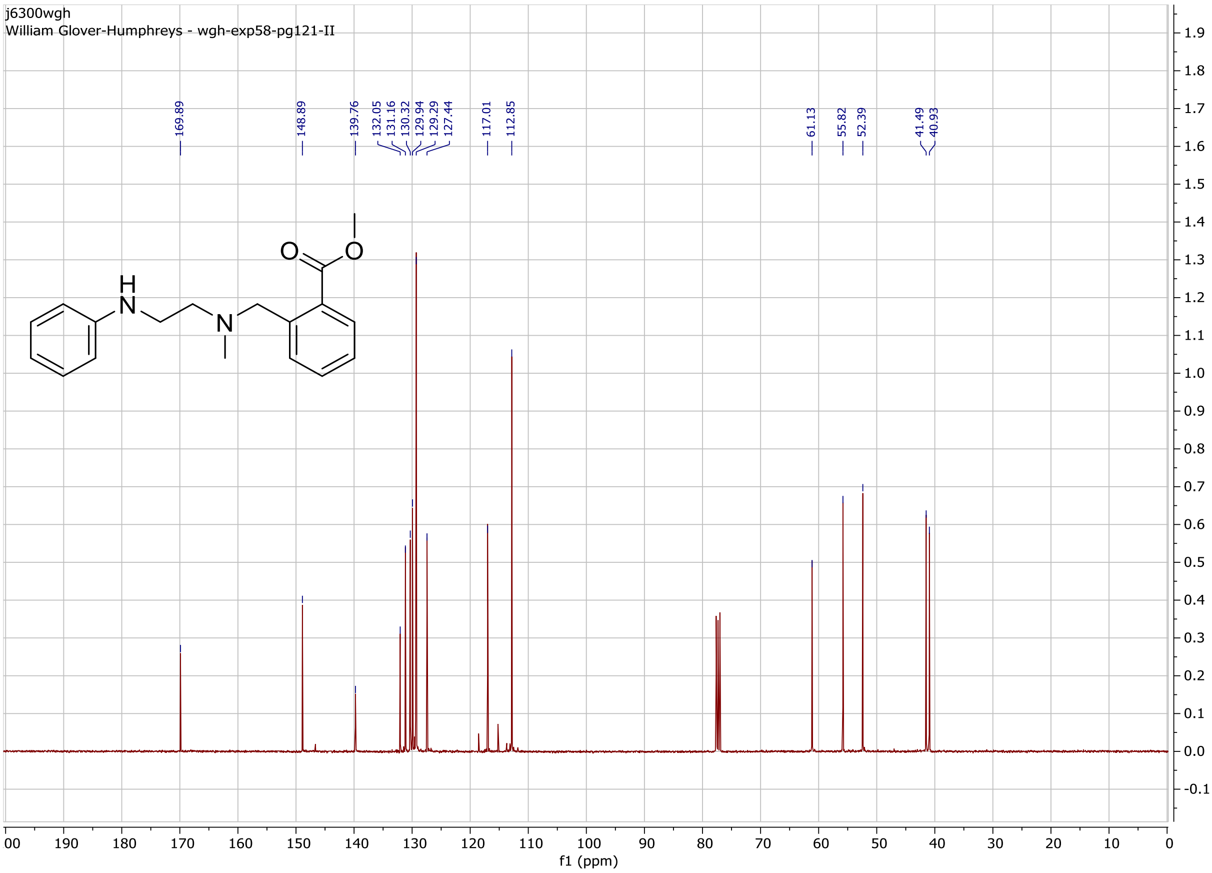

5-Methyl-2-phenyl-3,4,5,4-tetrahydrobenzo[f] [1,4] diazocin-1(2H)-one (76)

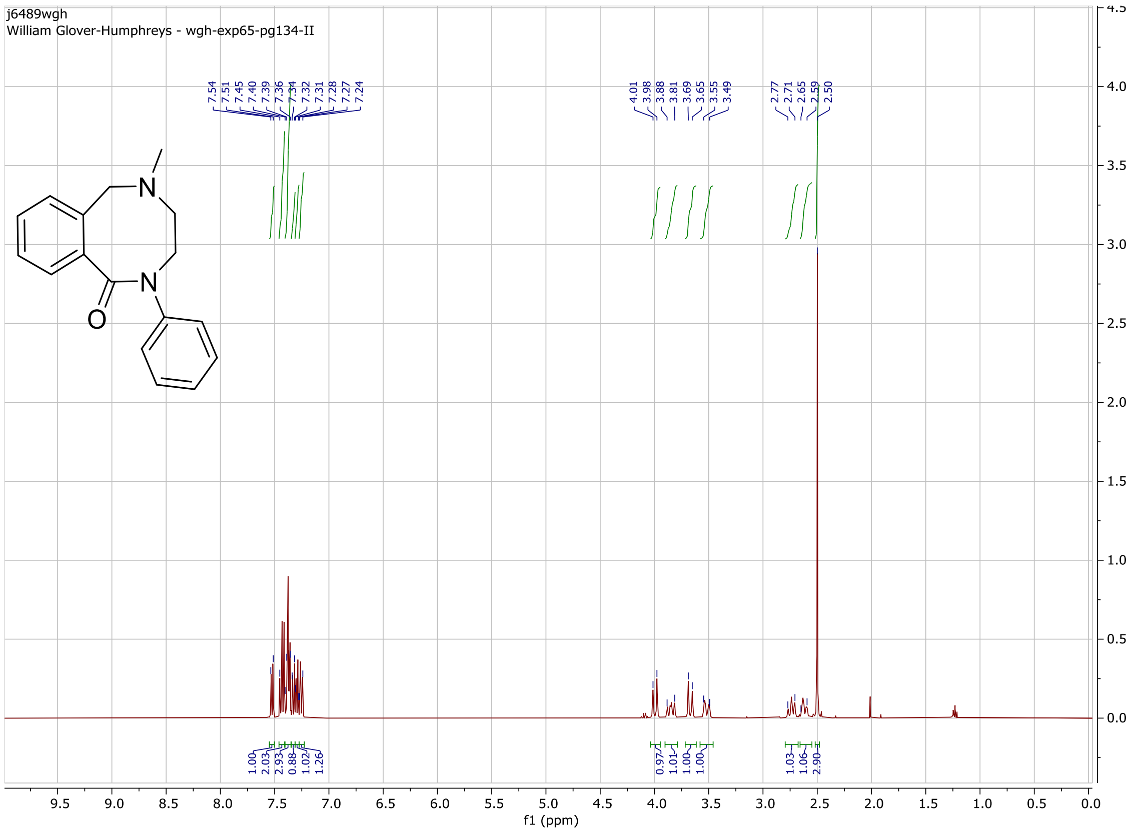

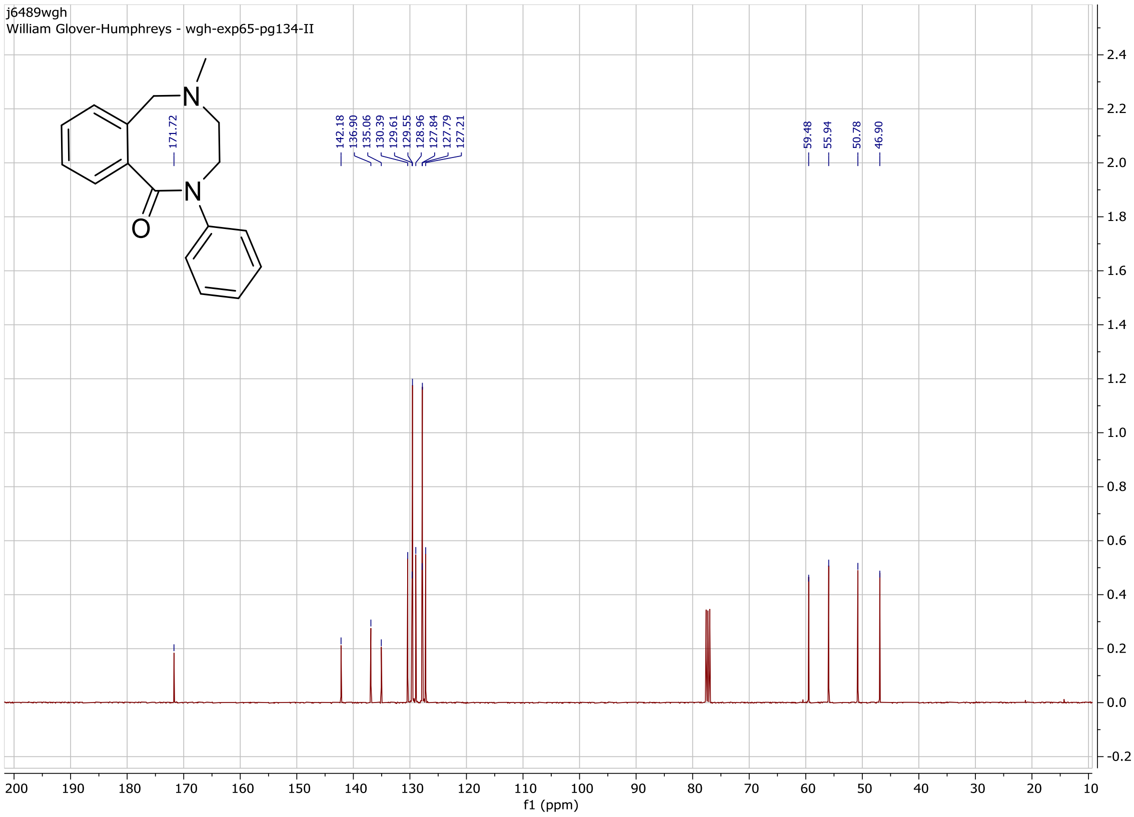

1,5-Diethyl-1,5-diazecan-6-one (77)

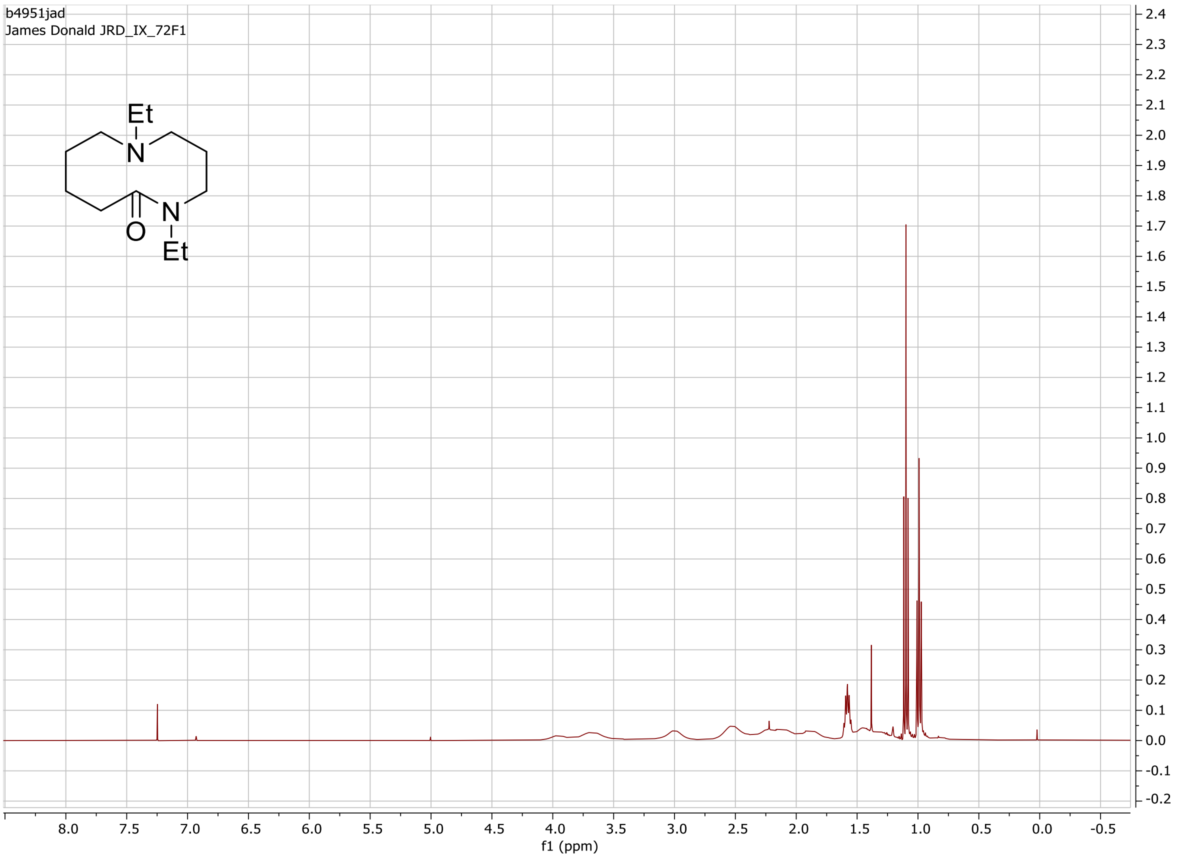

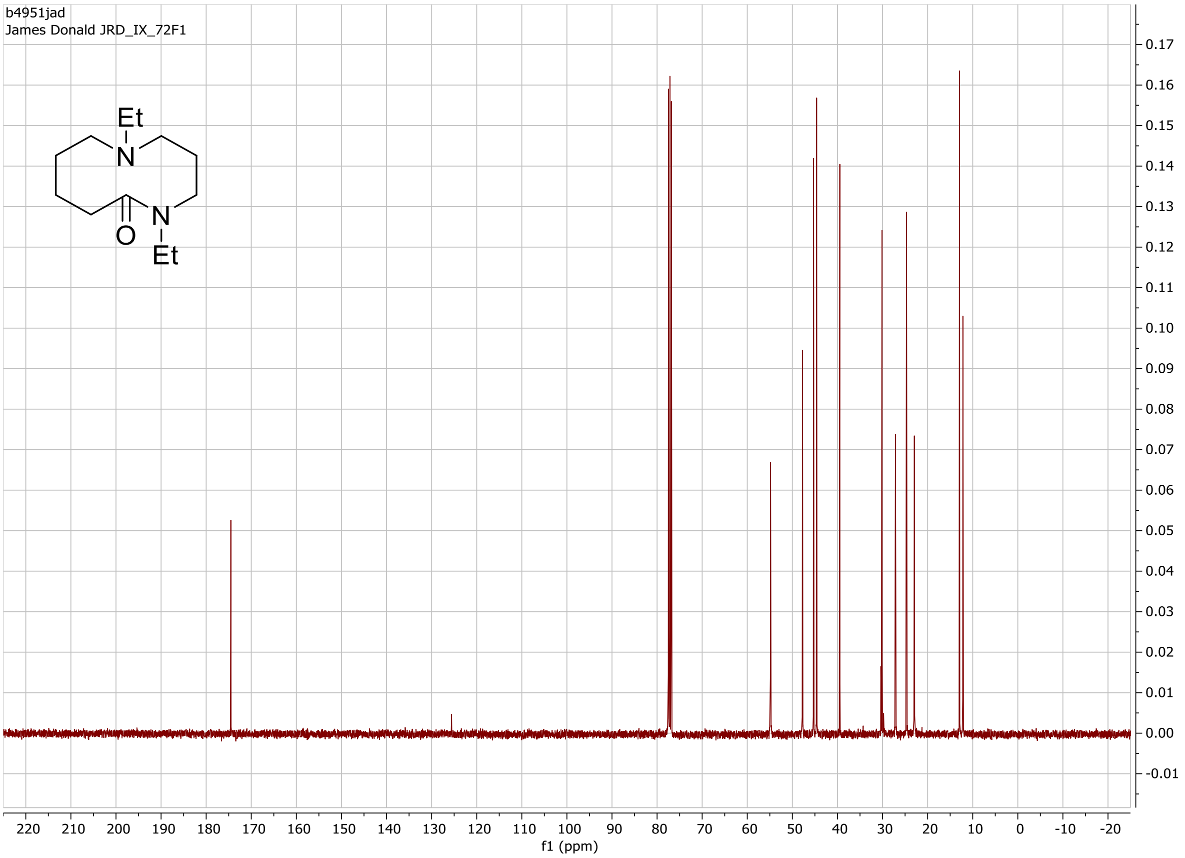

tert-Butyl 4-([(benzyloxy)carbonyl] (methyl)amino) butanoate (S103)

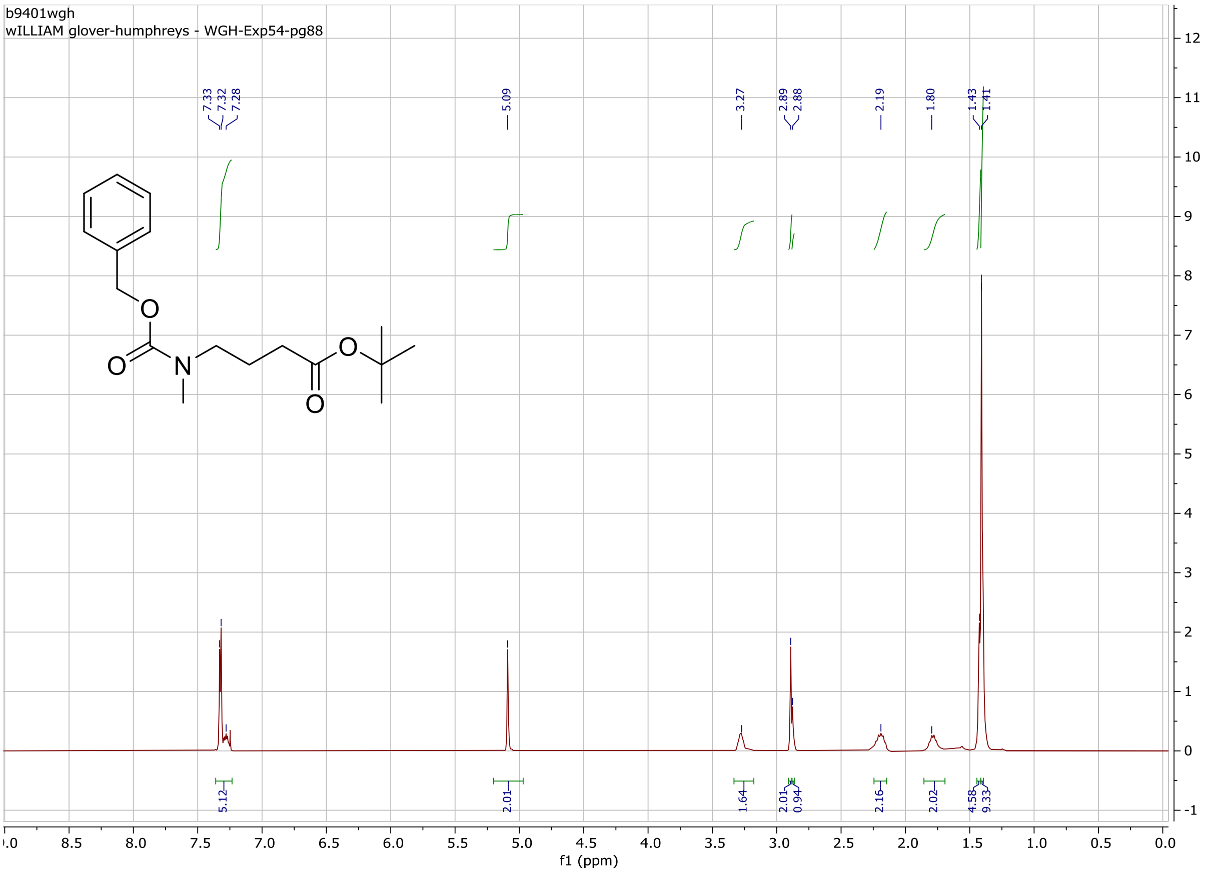

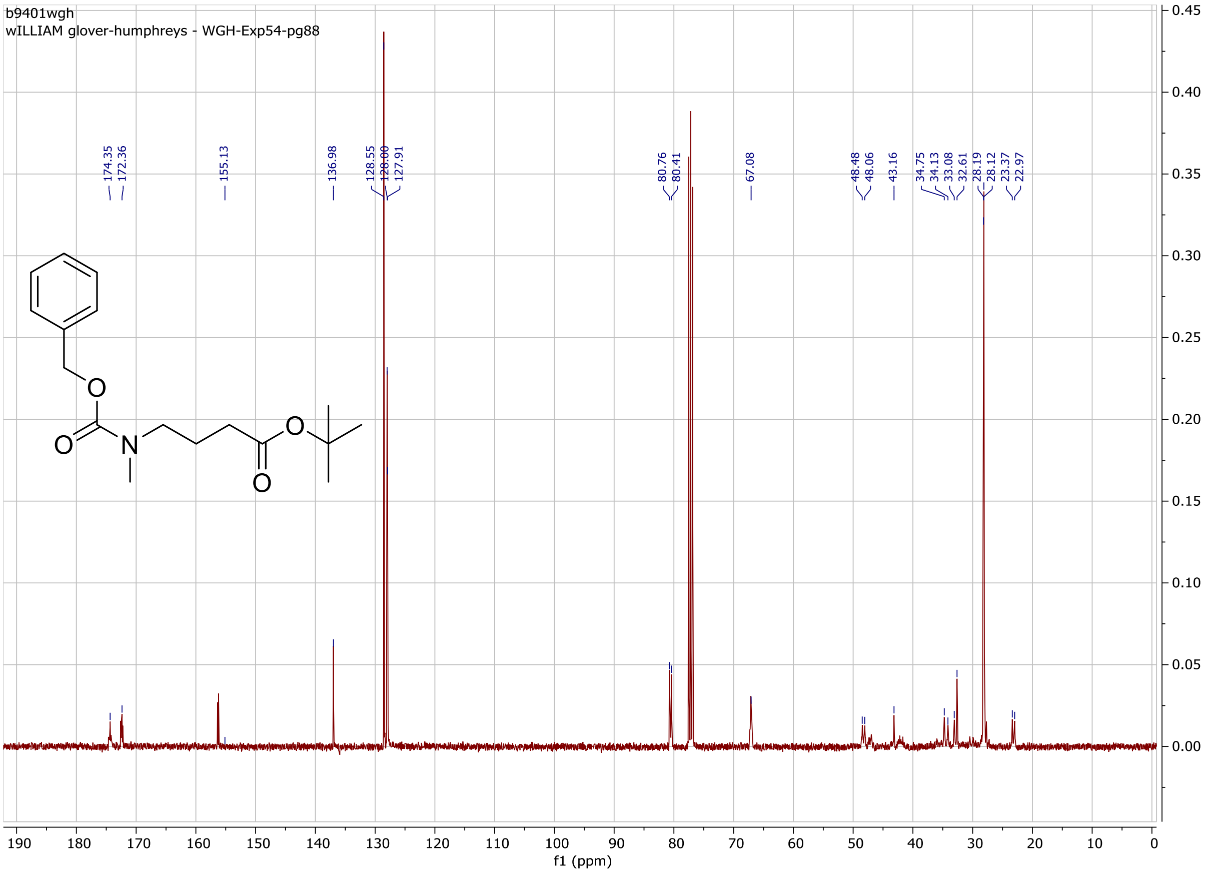

tert-Butyl 4-[(3-[(tert-butoxycarbonyl) amino] propyl) (methyl) amino] butanoate (S104)

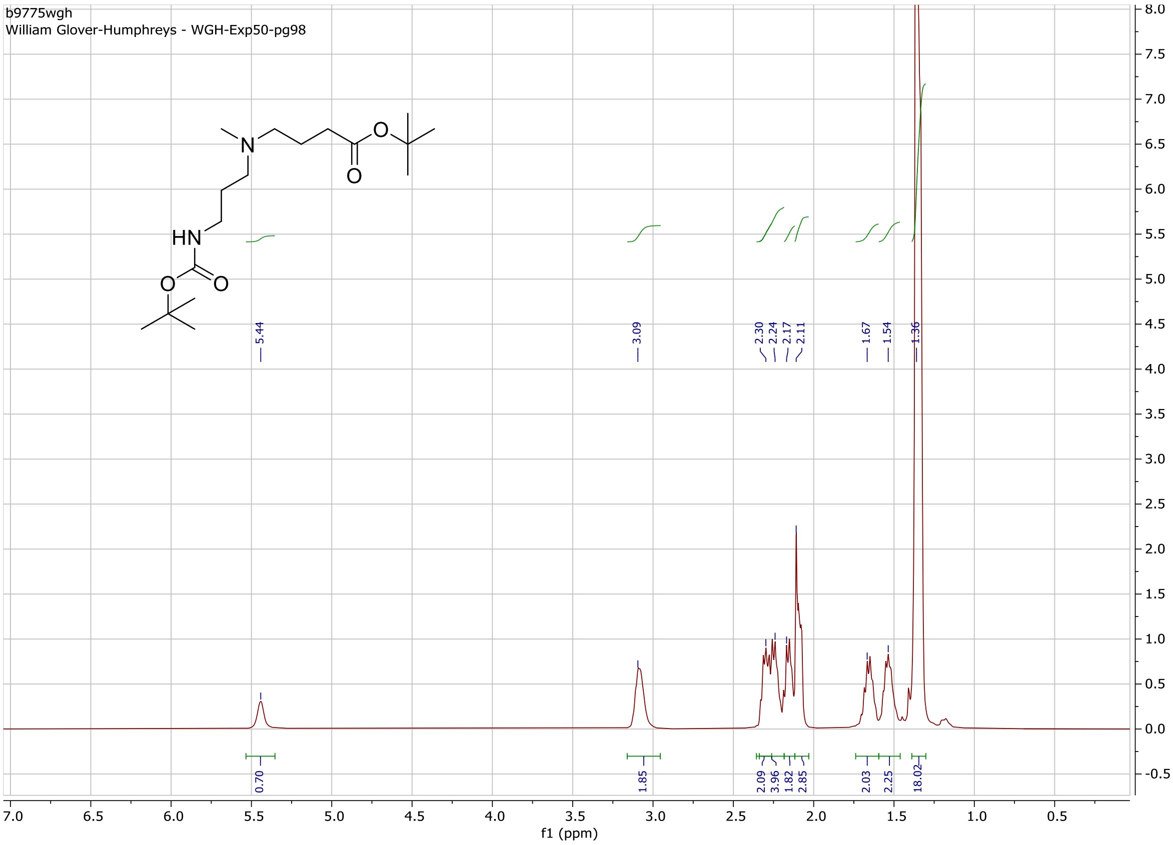

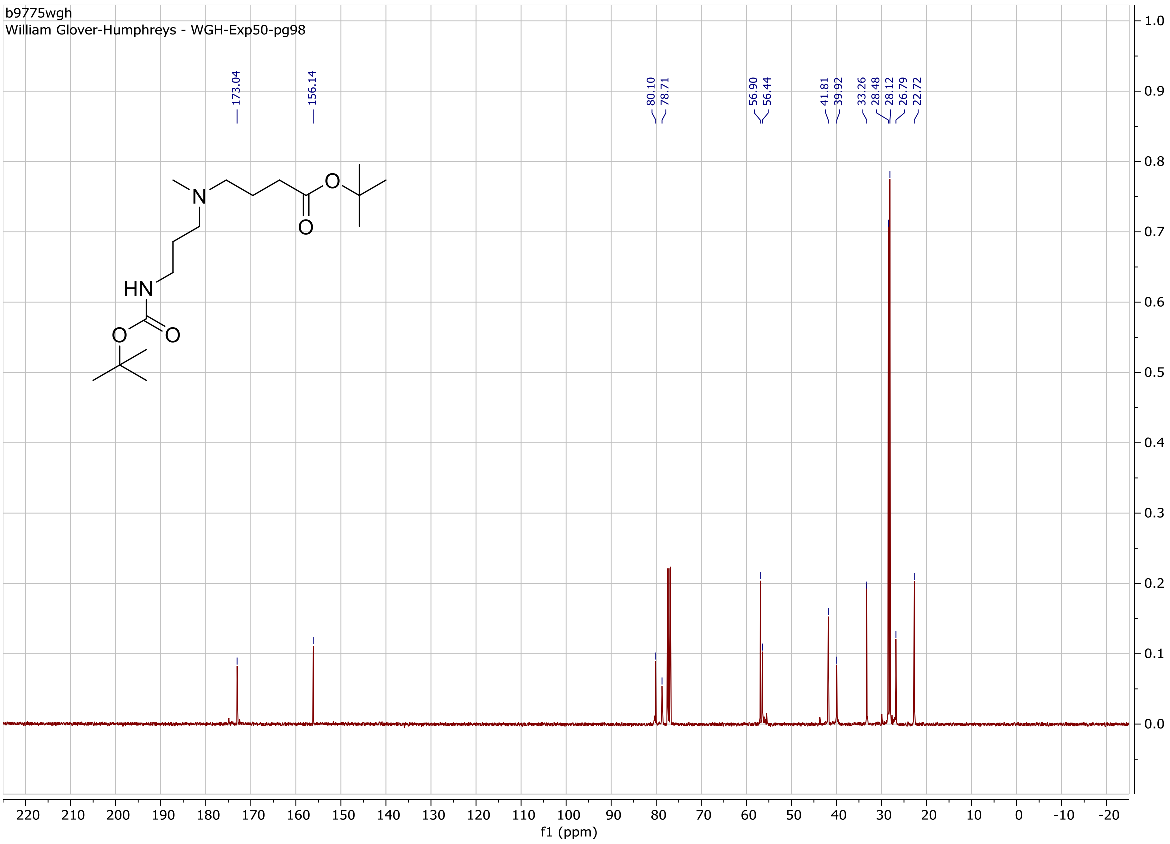

1-Methyl-1,5-diazonan-4-one (78)

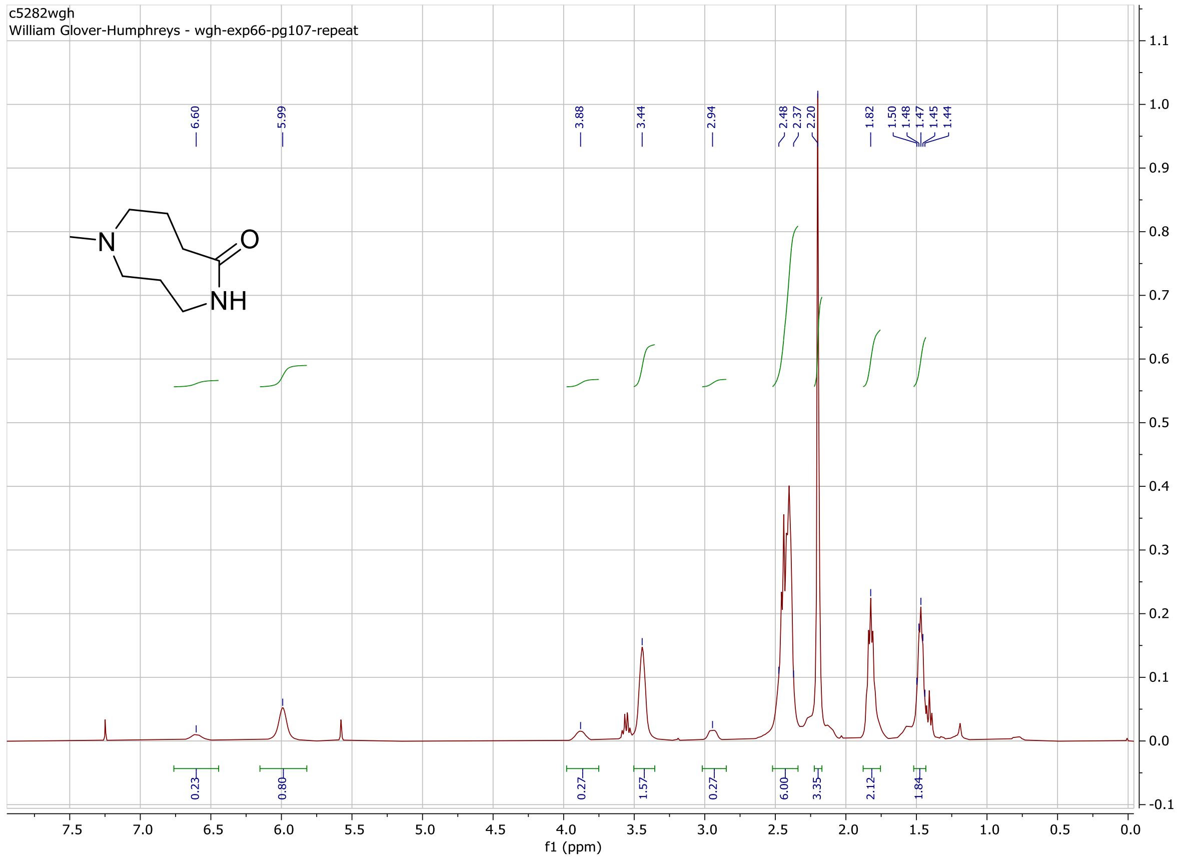

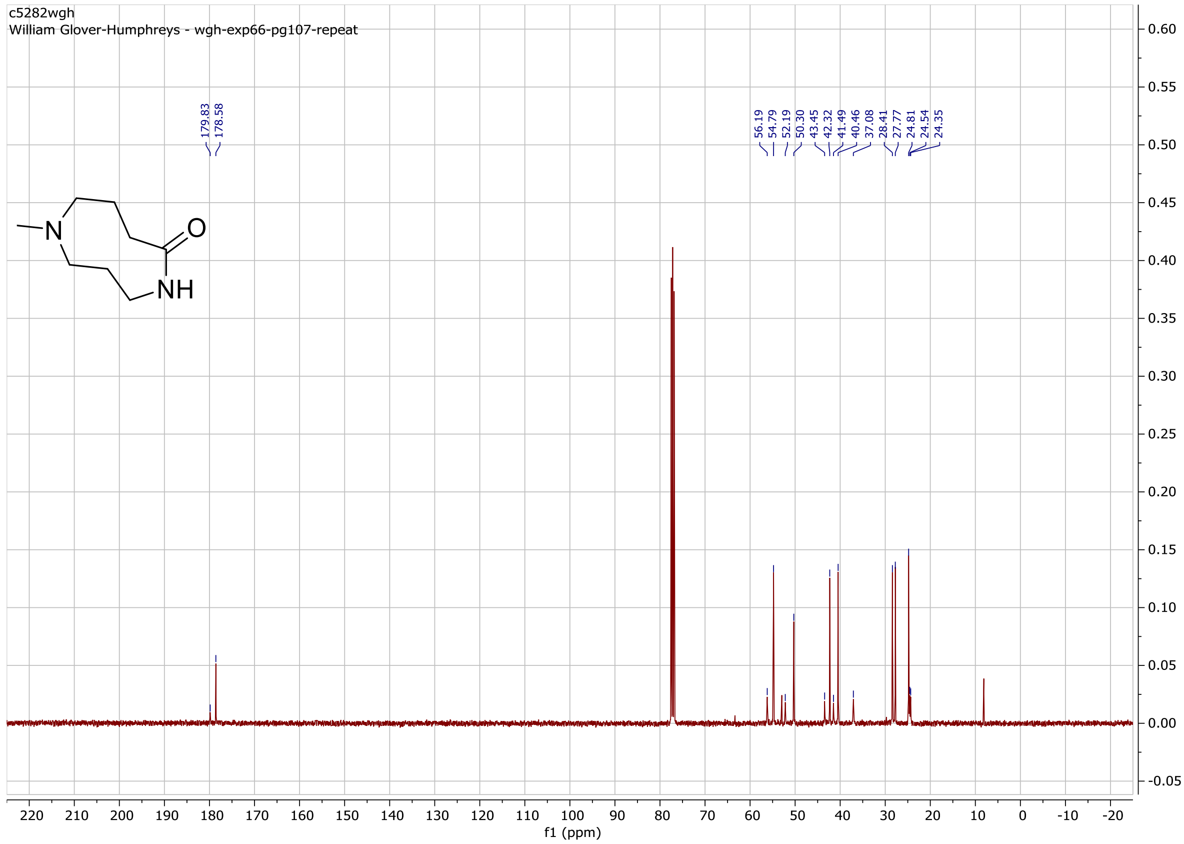

***tert*-Butyl 2-((benzyl(2-(methoxycarbonyl)benzyl)amino)methyl)piperidine-1-carboxylate (S106)**

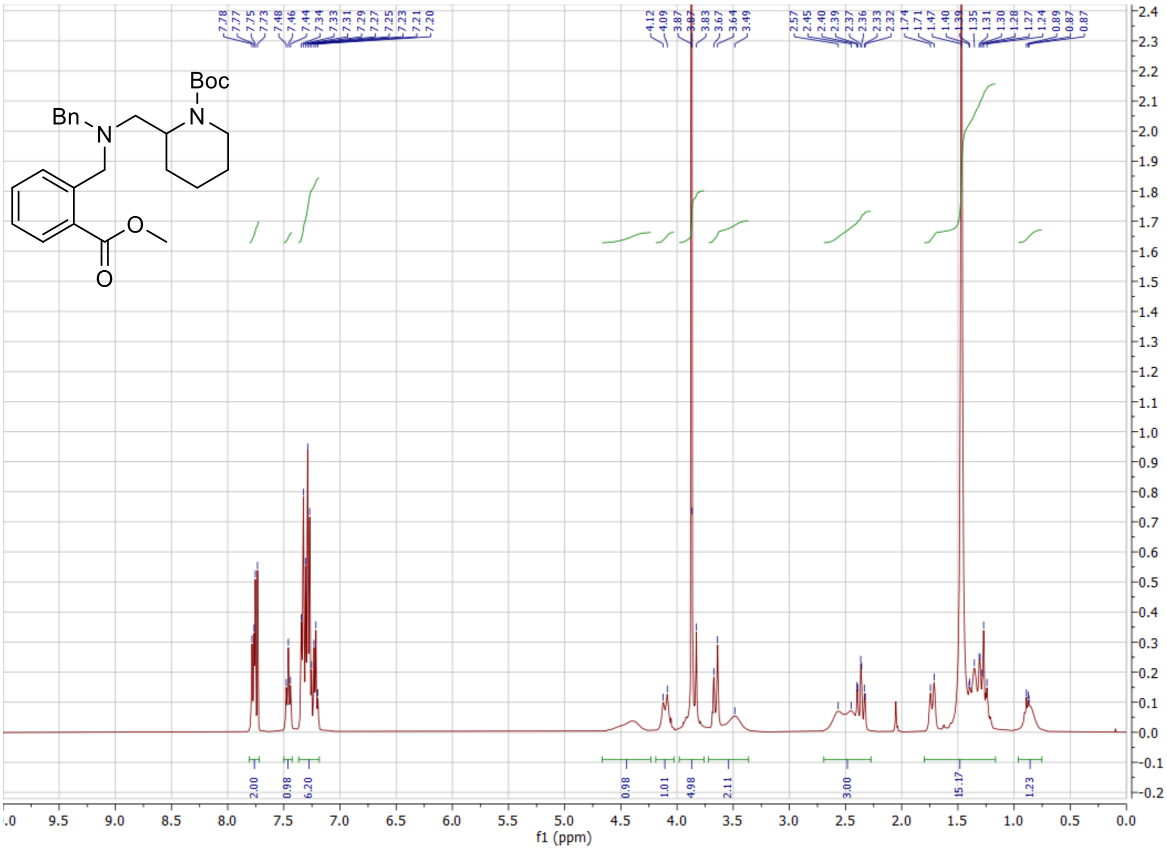

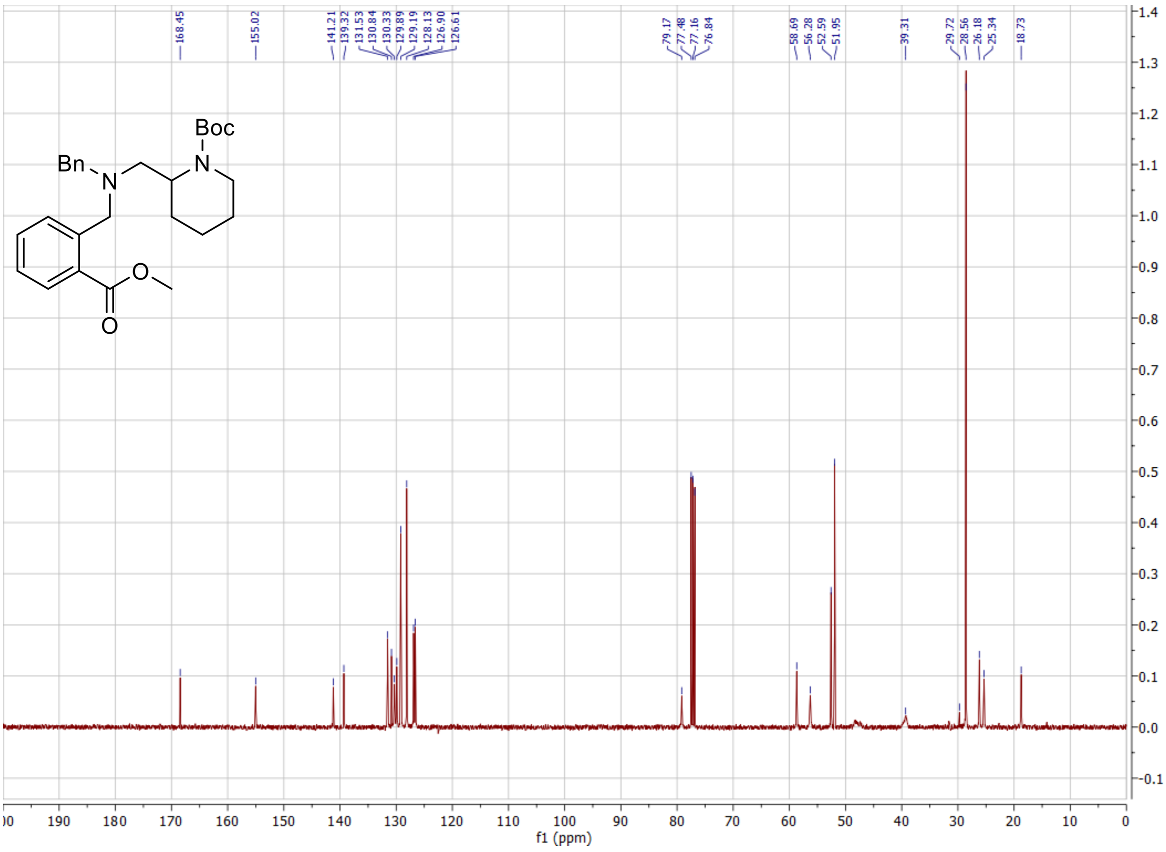

Methyl 2-((benzyl(piperidin-2-ylmethyl)amino)methyl)benzoate (S107)

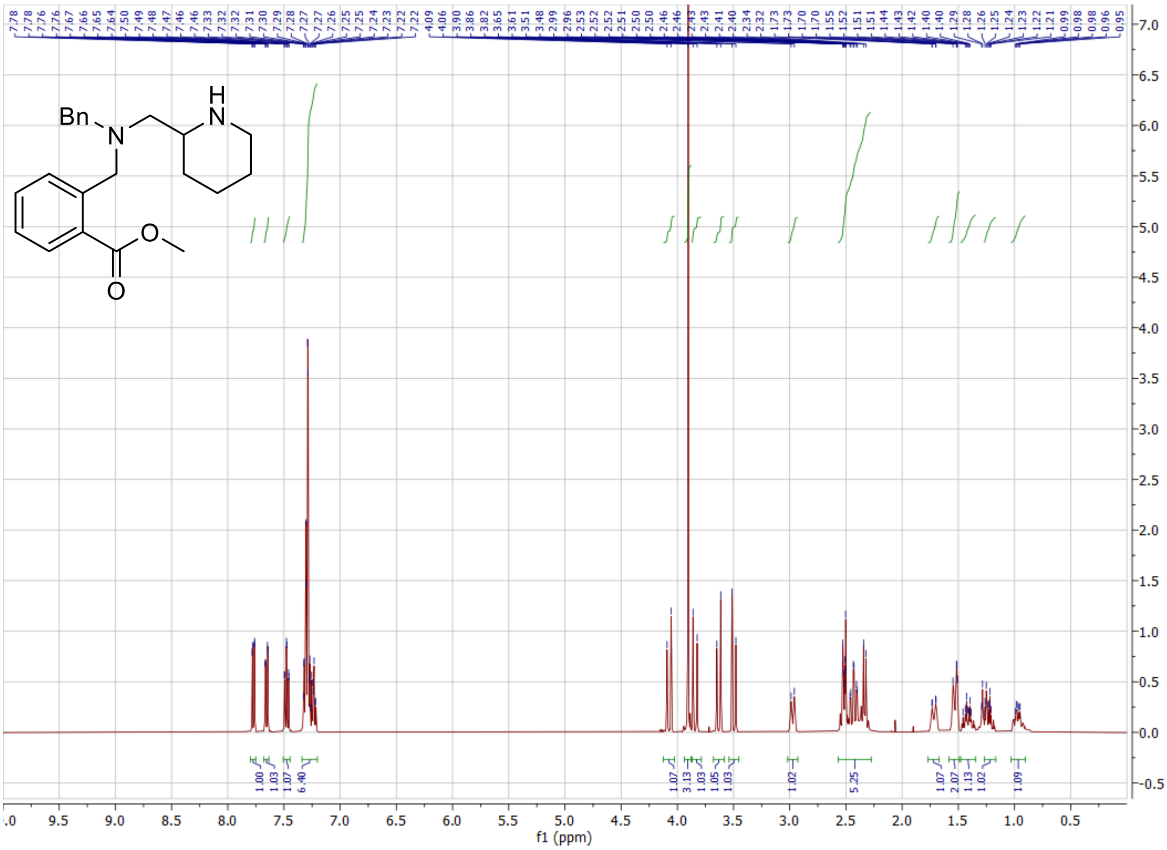

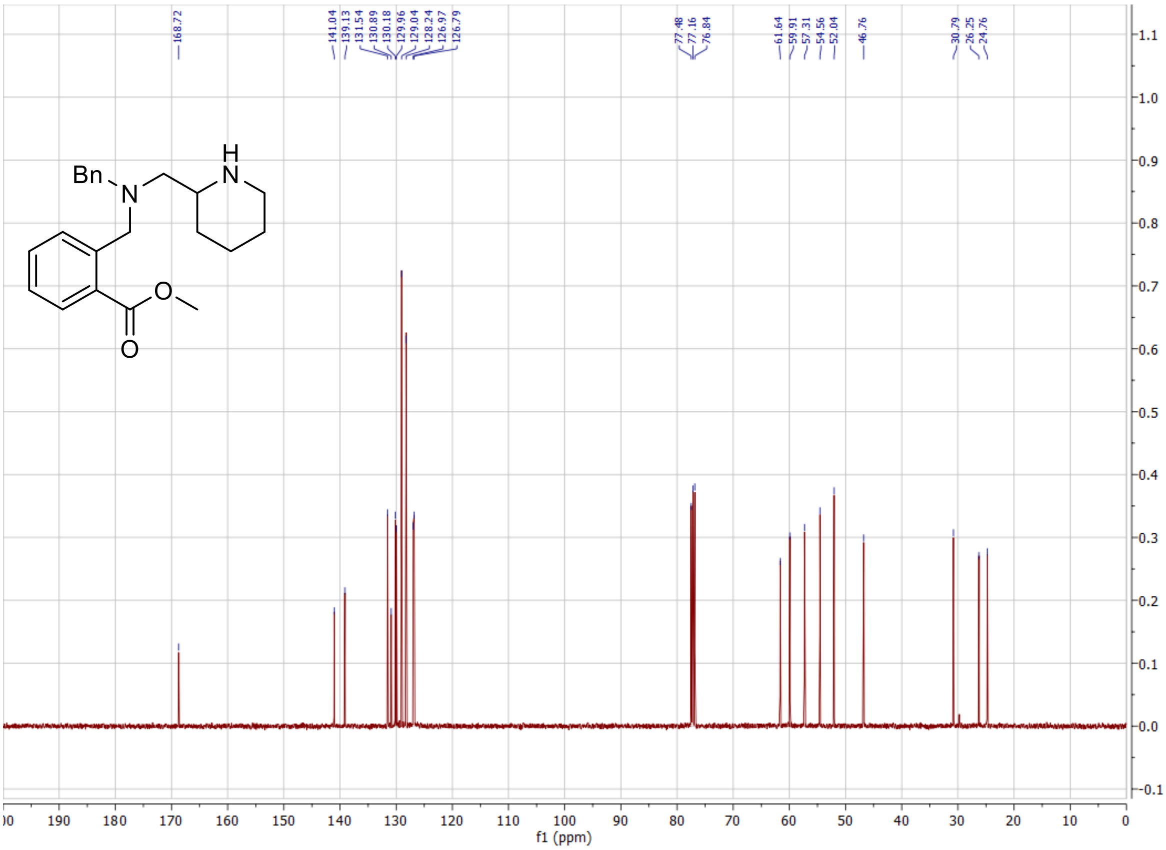

2-((Benzyl(piperidin-2-ylmethyl)amino)methyl)benzoic acid (S108)

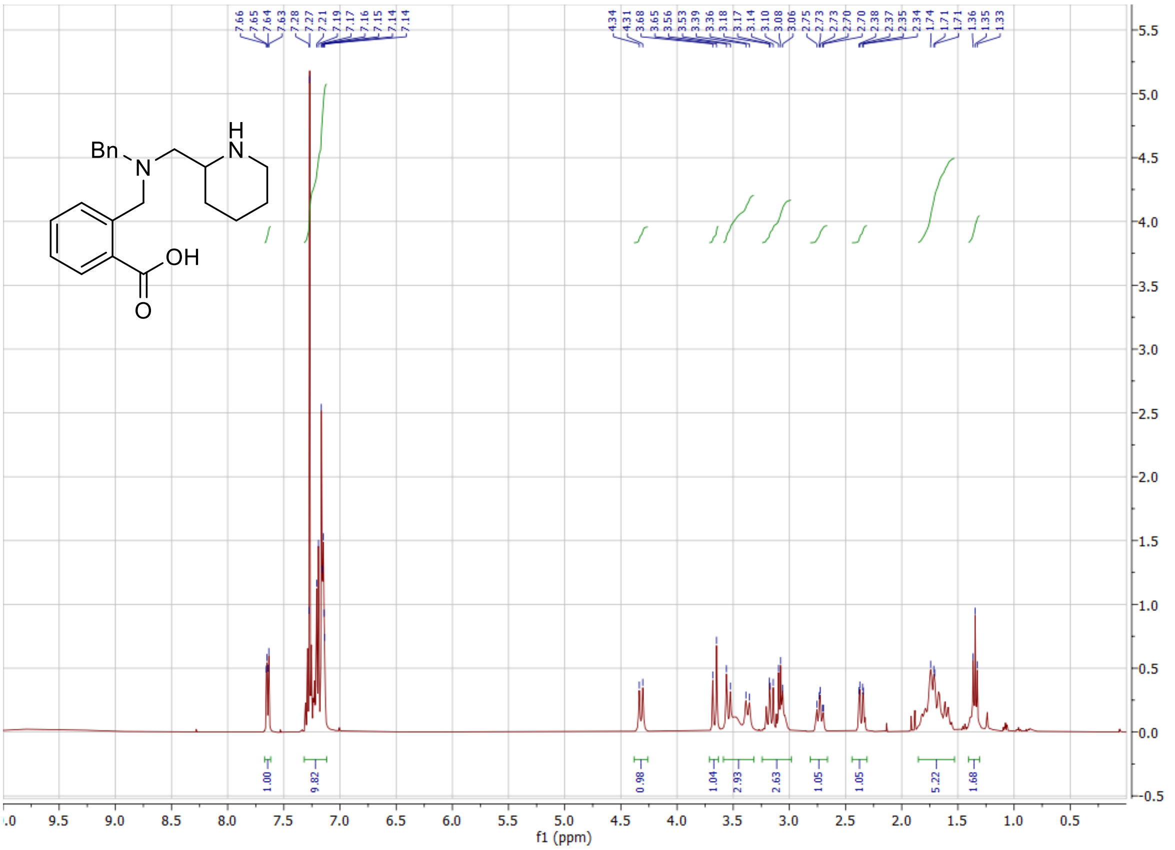

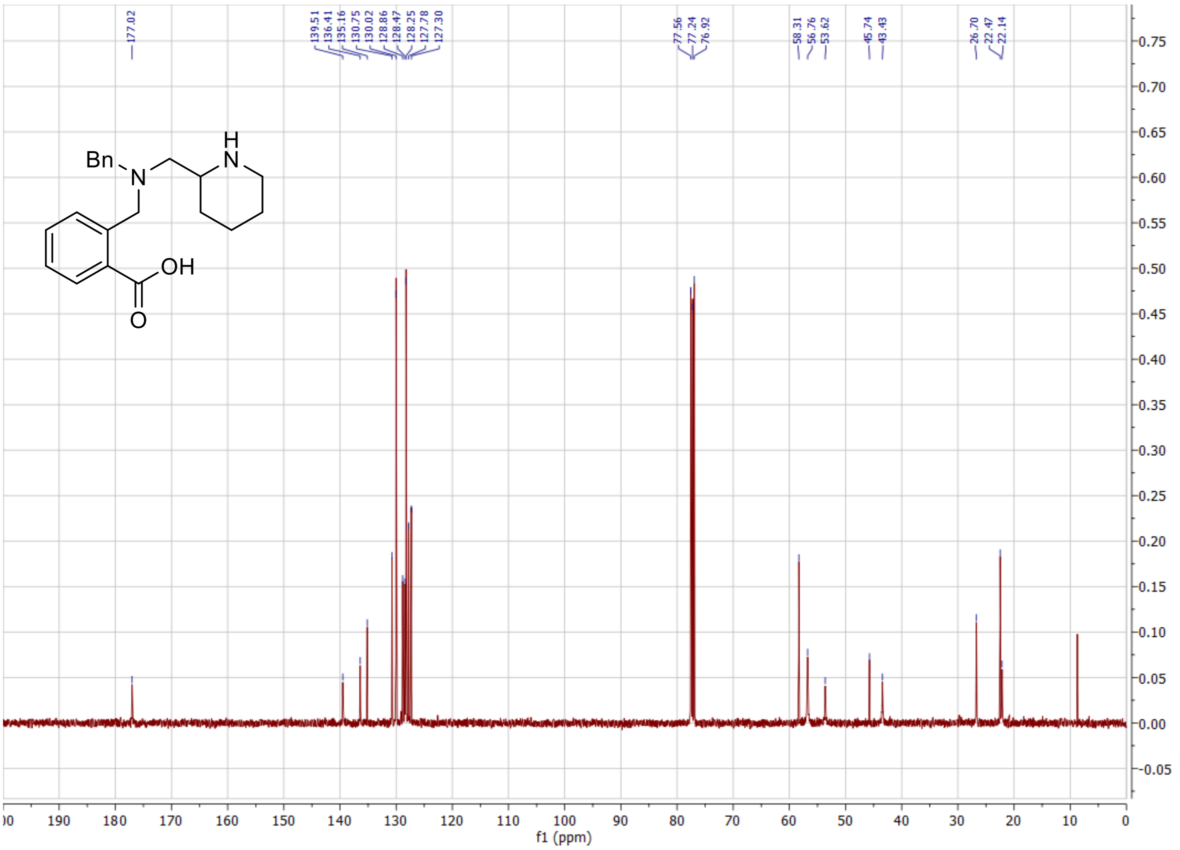

6-Benzyl-5,6,7,7a,8,9,10,11-octahydro-13H-benzo[f]pyrido[1,2-a][1,4]diazocin-13-one (79).

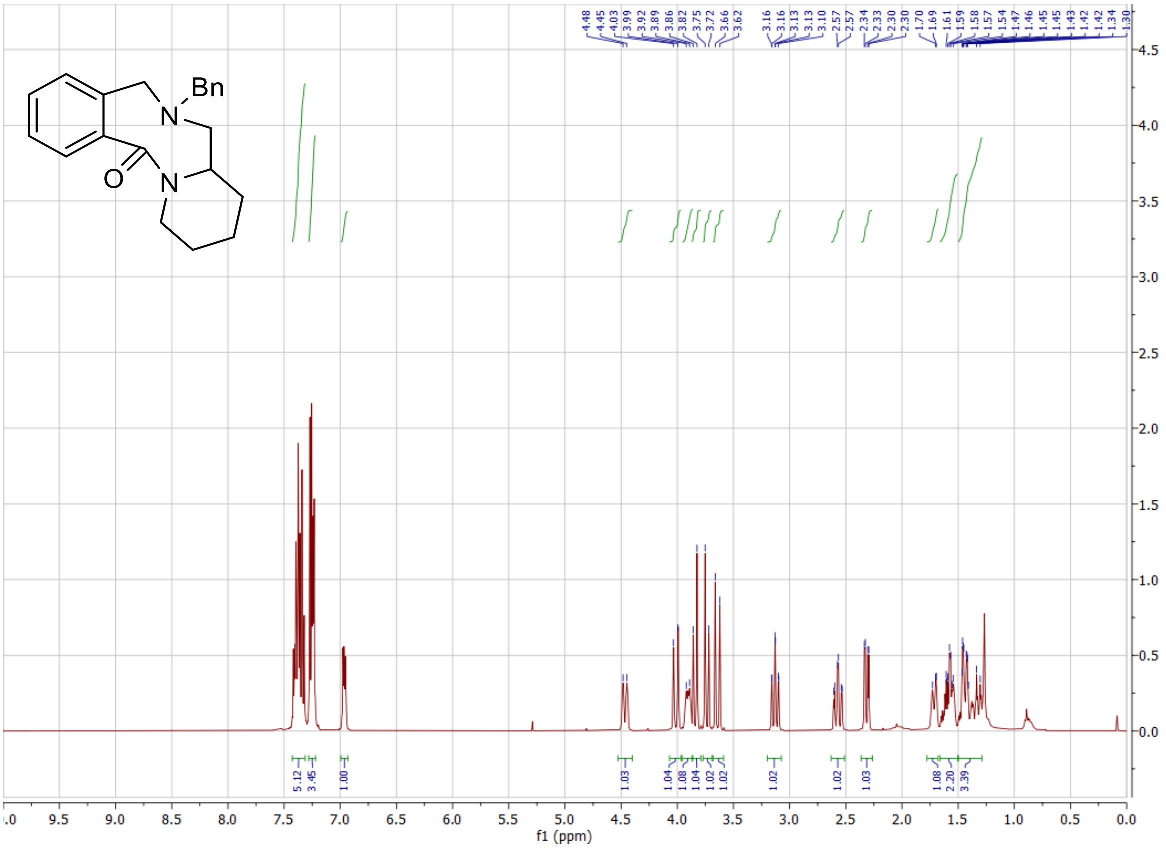

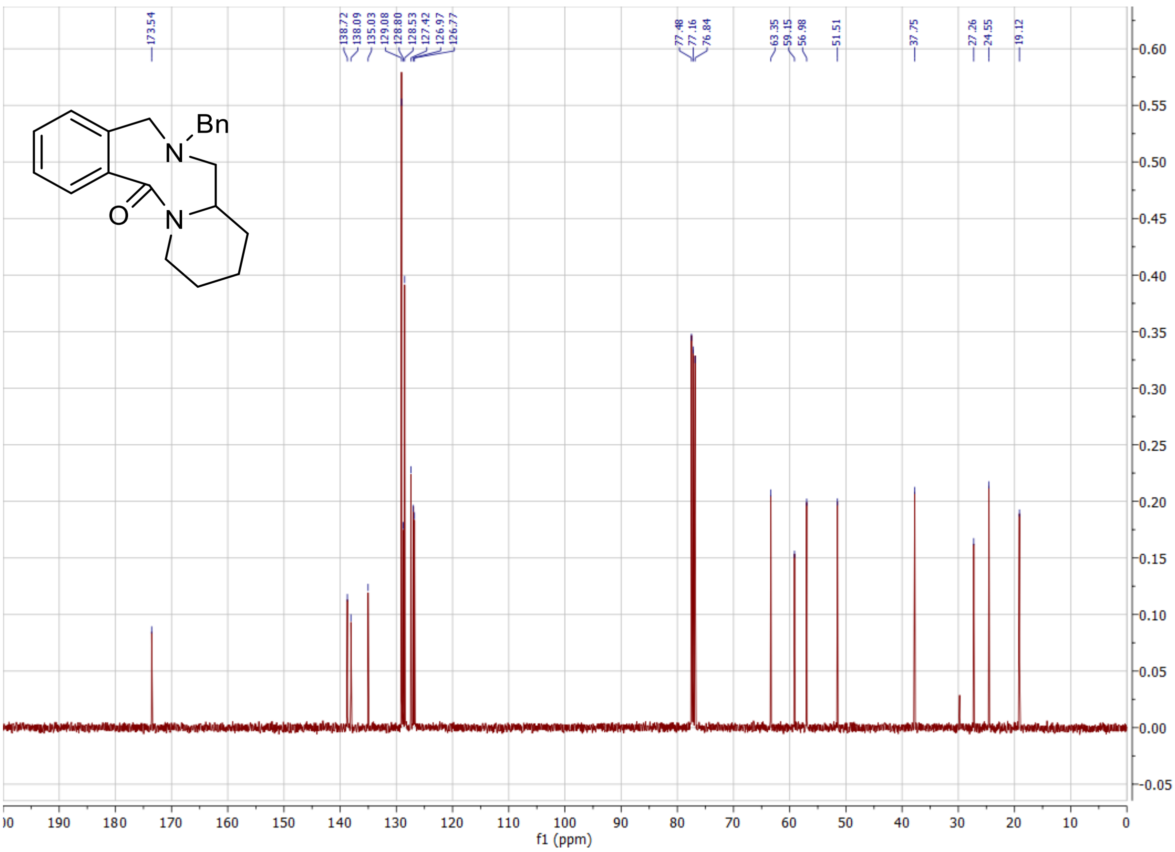

2-[(Benzyloxy)carbonyl]-1,2,3,4-tetrahydroisoquinoline-3-carboxylic acid (S109). In solution in $CDCl_3$, the compound exists as a ≈4:3 mixture, evident in its ^{13}C NMR data.

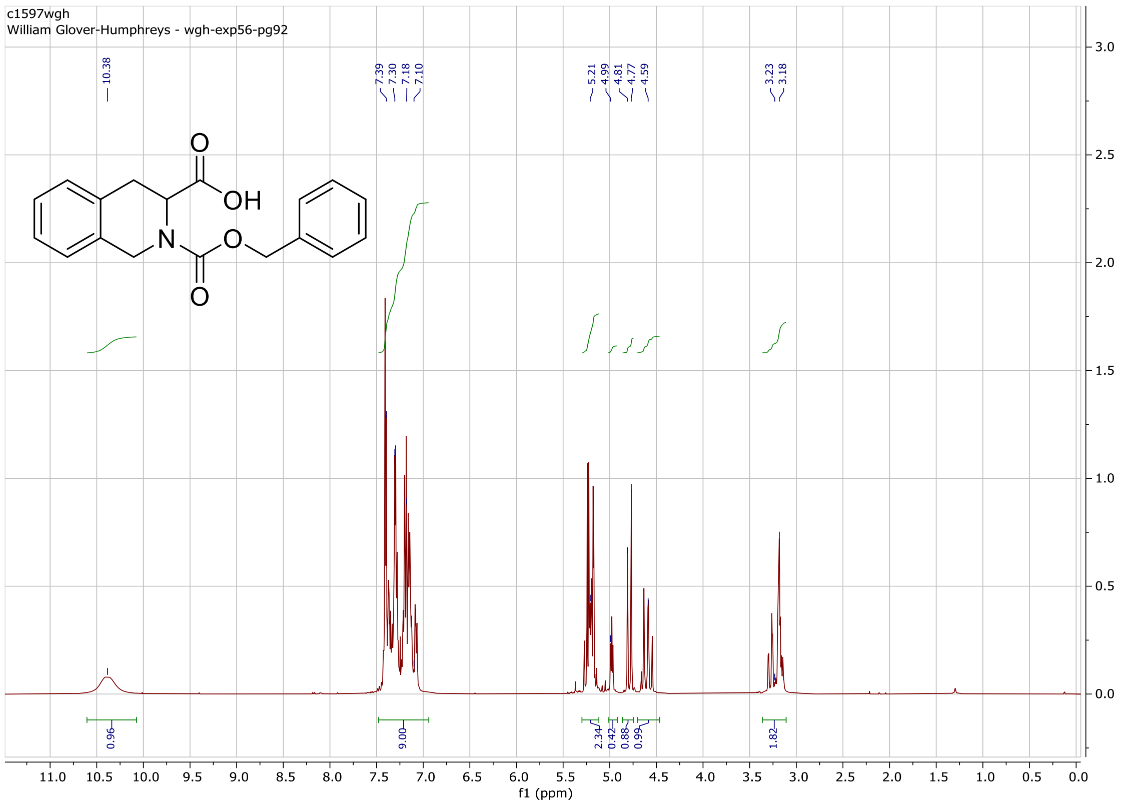

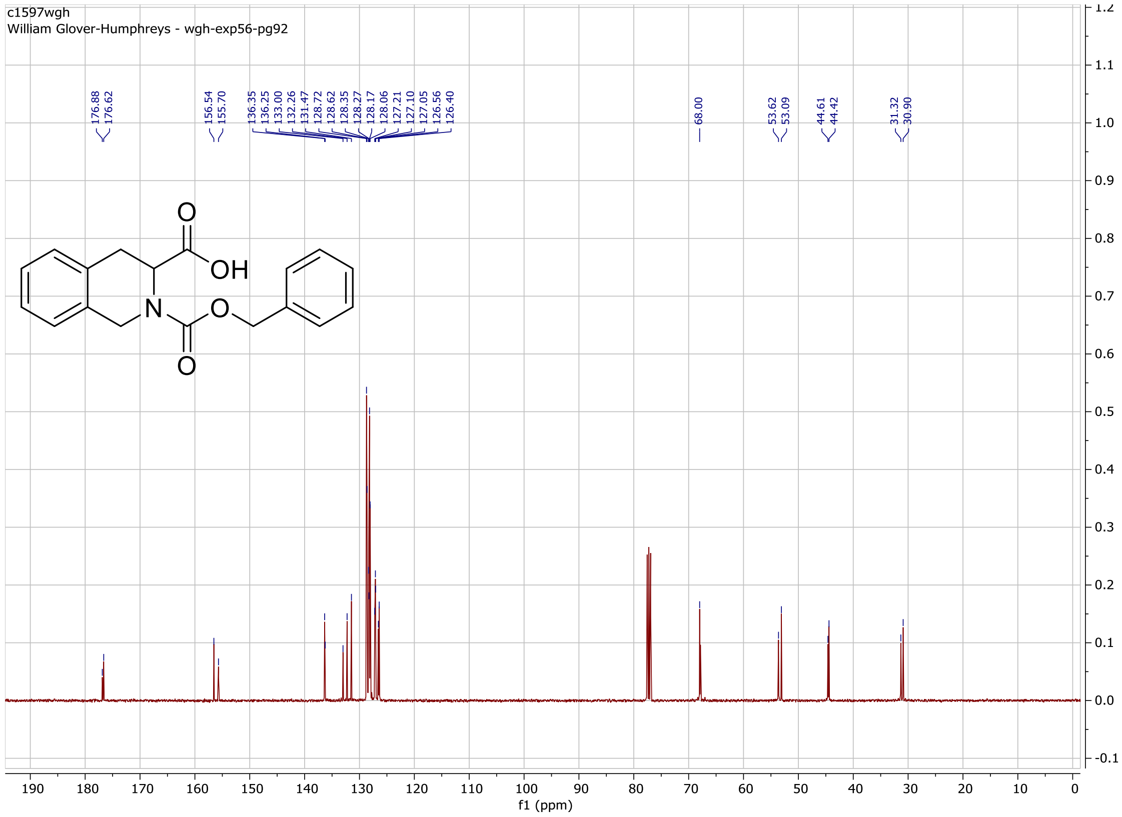

Benzyl 3-(3-*tert*-butoxy-3-oxopropyl)-3,4-dihydroisoquinoline-2(1H)-carboxylate (S110)

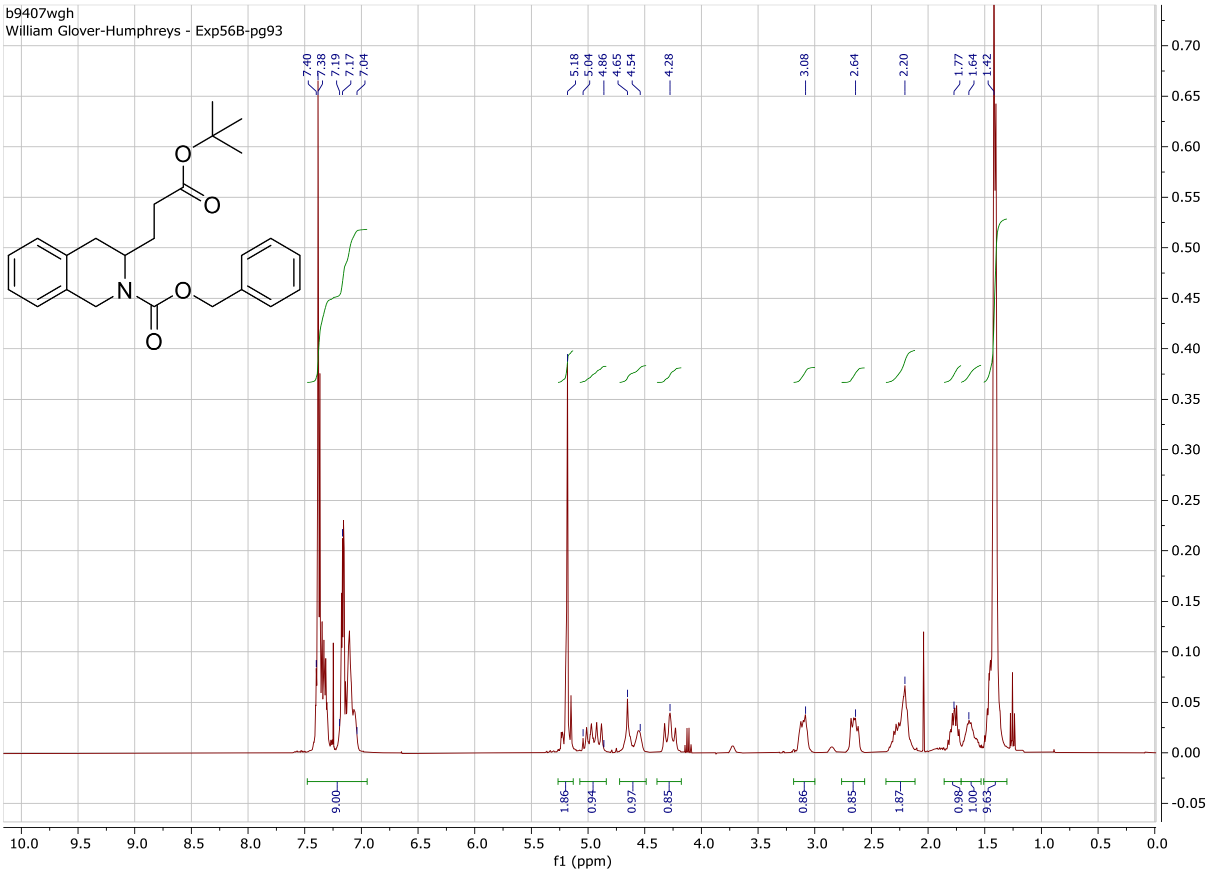

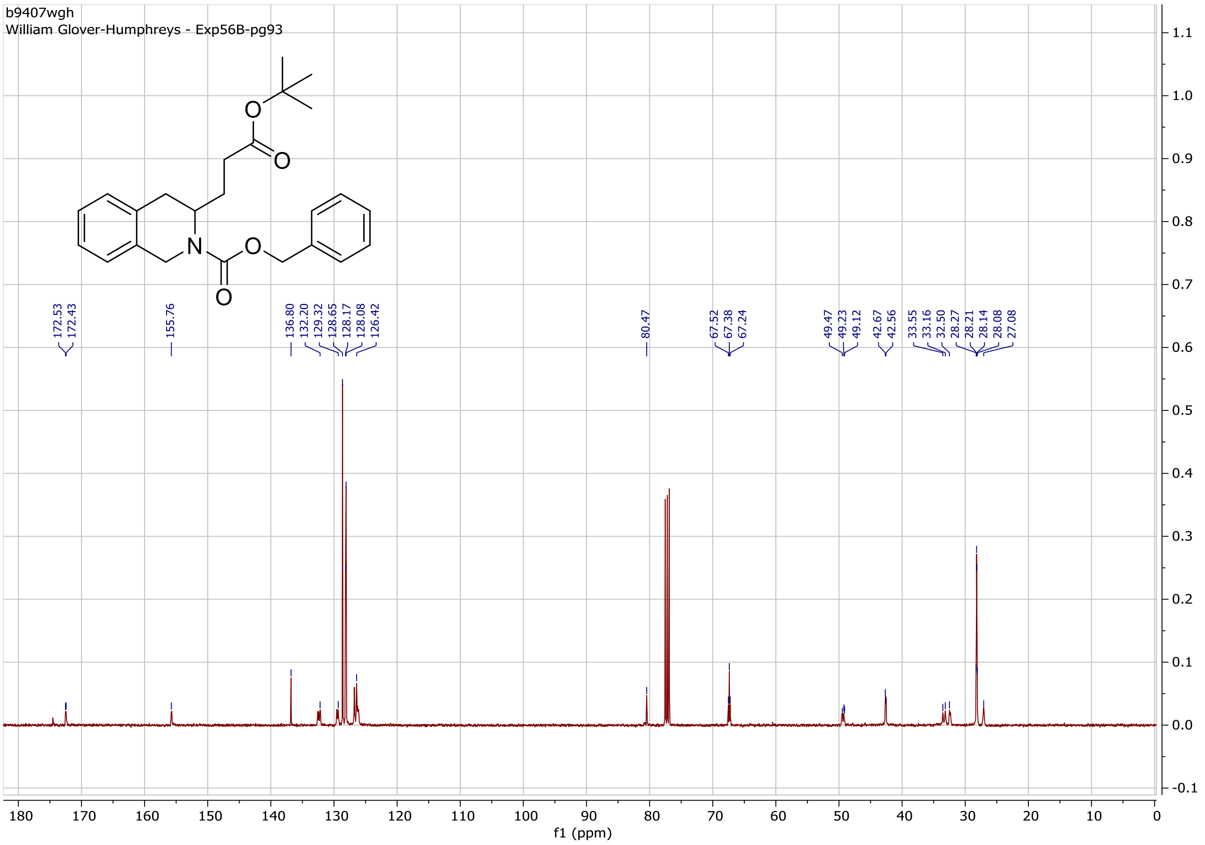

tert-Butyl 3-(2-(3-[(_tert_-butoxycarbonyl) amino] propyl)-1,2,3,4-tetrahydroisoquinolin-3-yl) propanoate (S111)

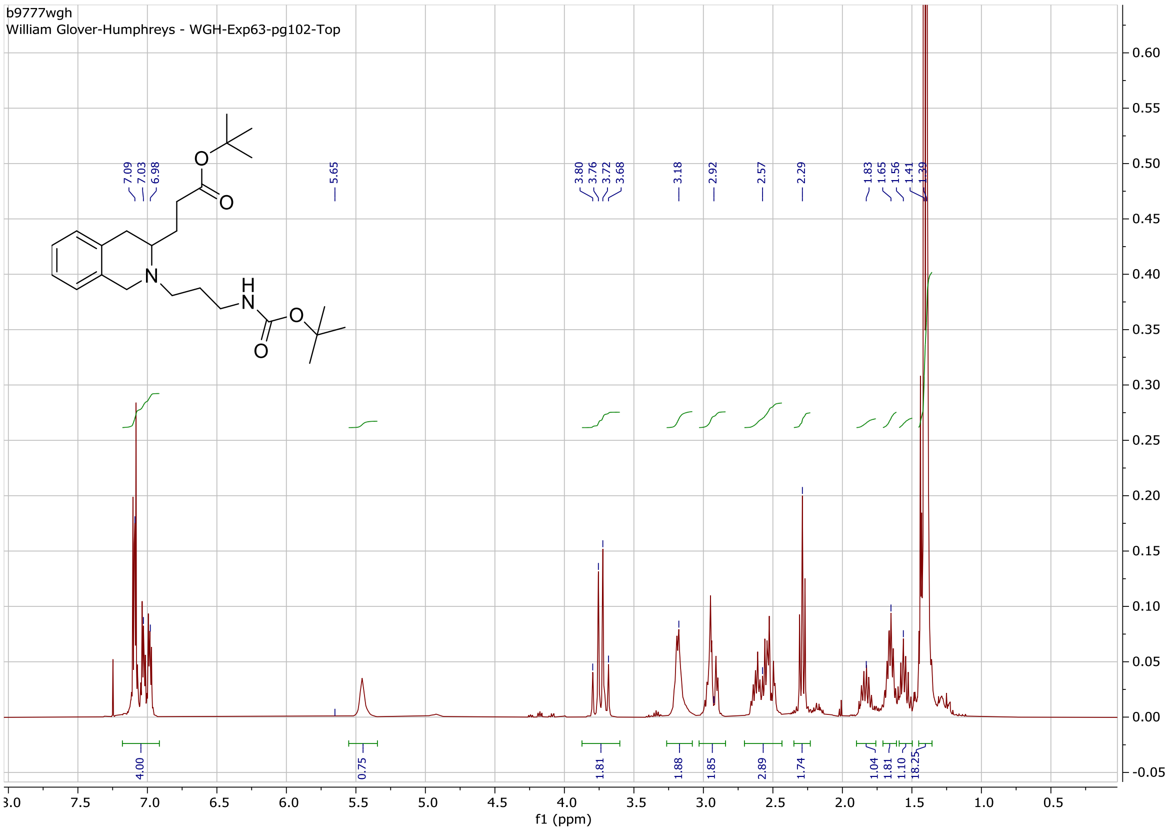

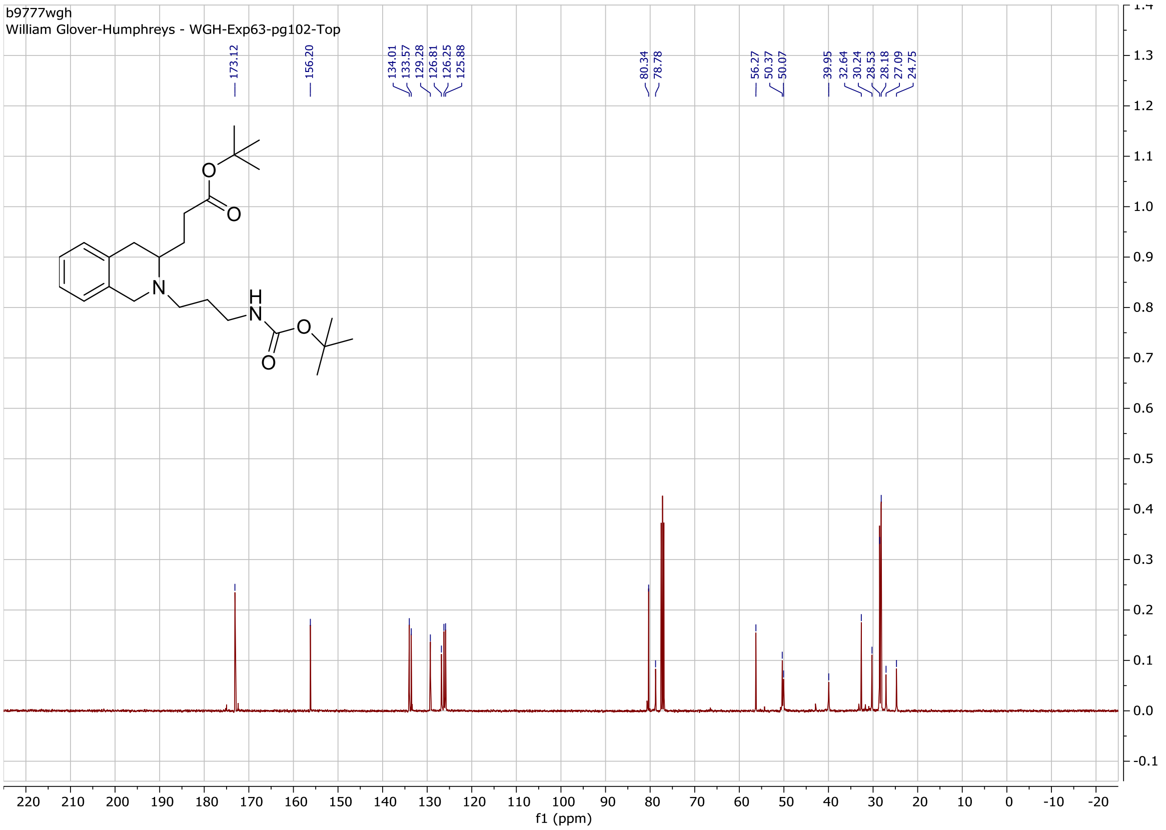

1,4,5,6,7,9,14,14a-octahydro [1,5] diazonino[1,9-b] isoquinolin-3(2H)-one (80). Significant rotameric broadening of NMR signals in was observed.

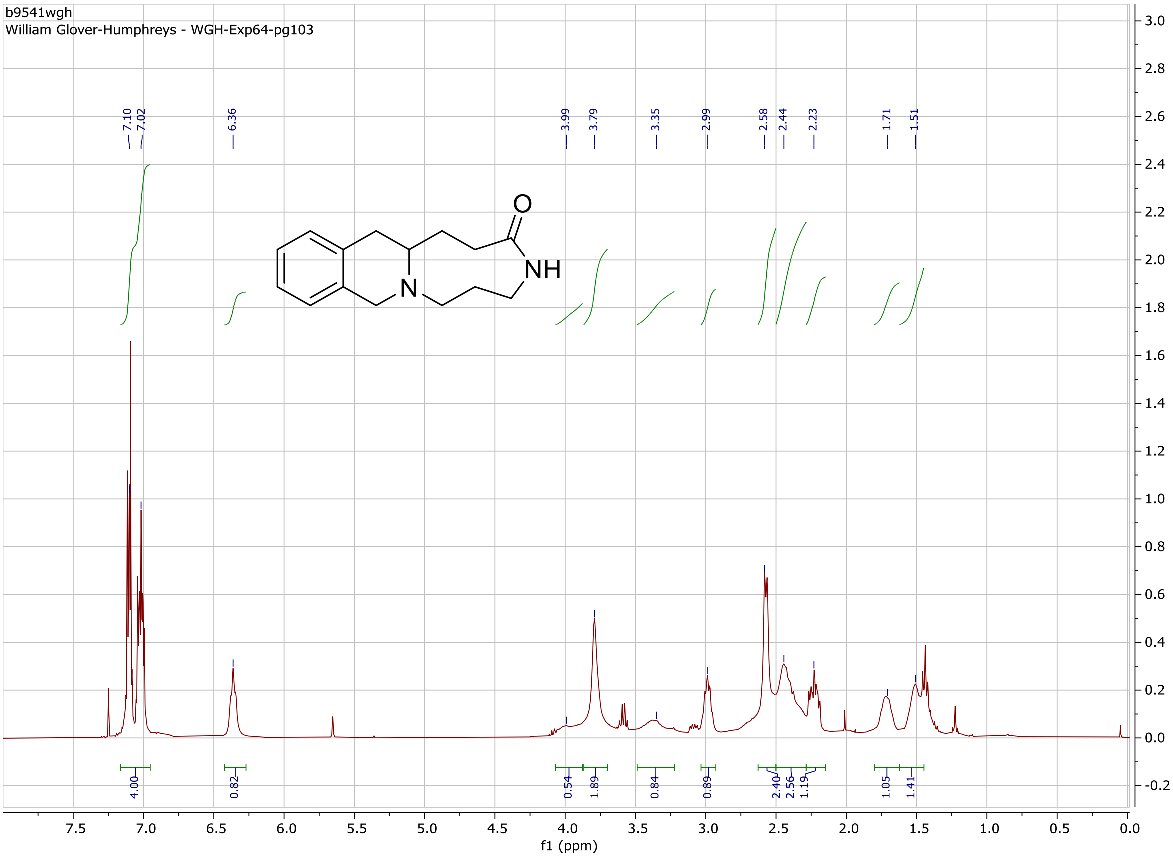

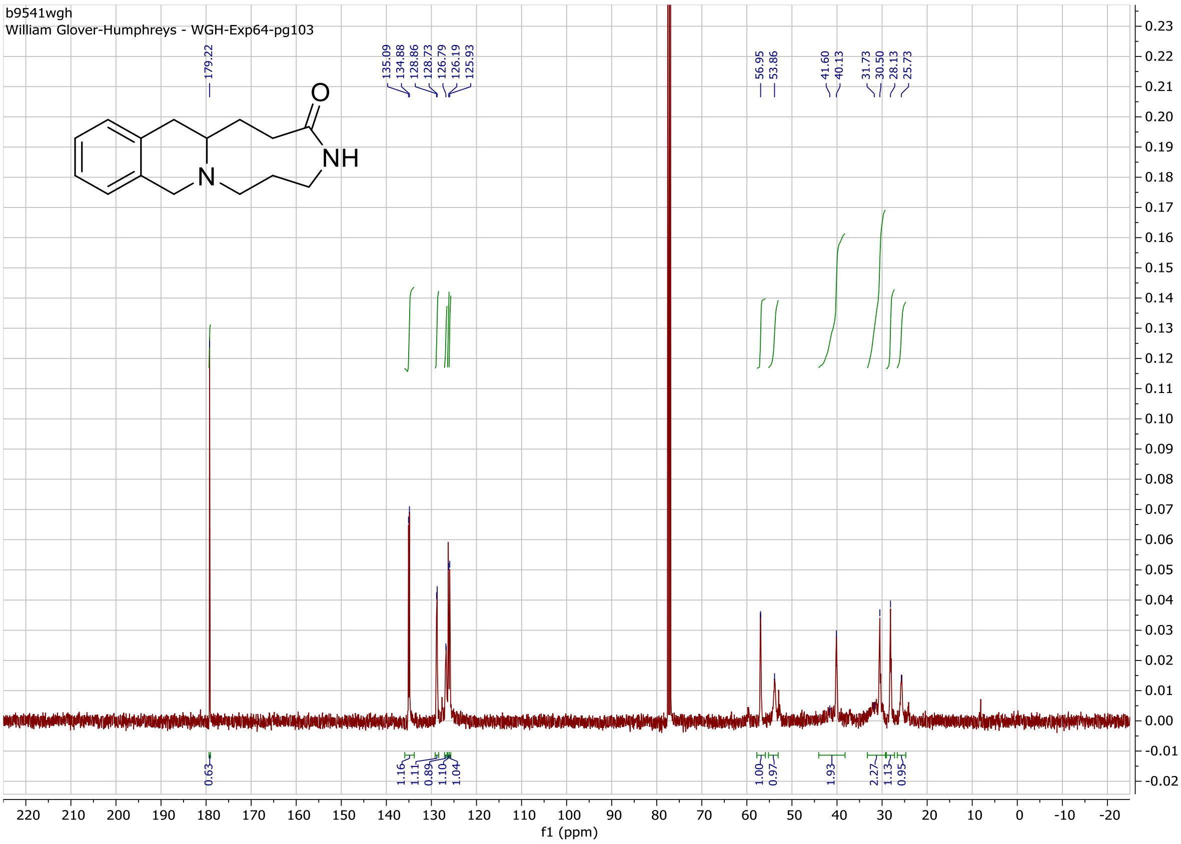

Ethyl 4-((2-methoxyphenyl)amino)butanoate (S112)

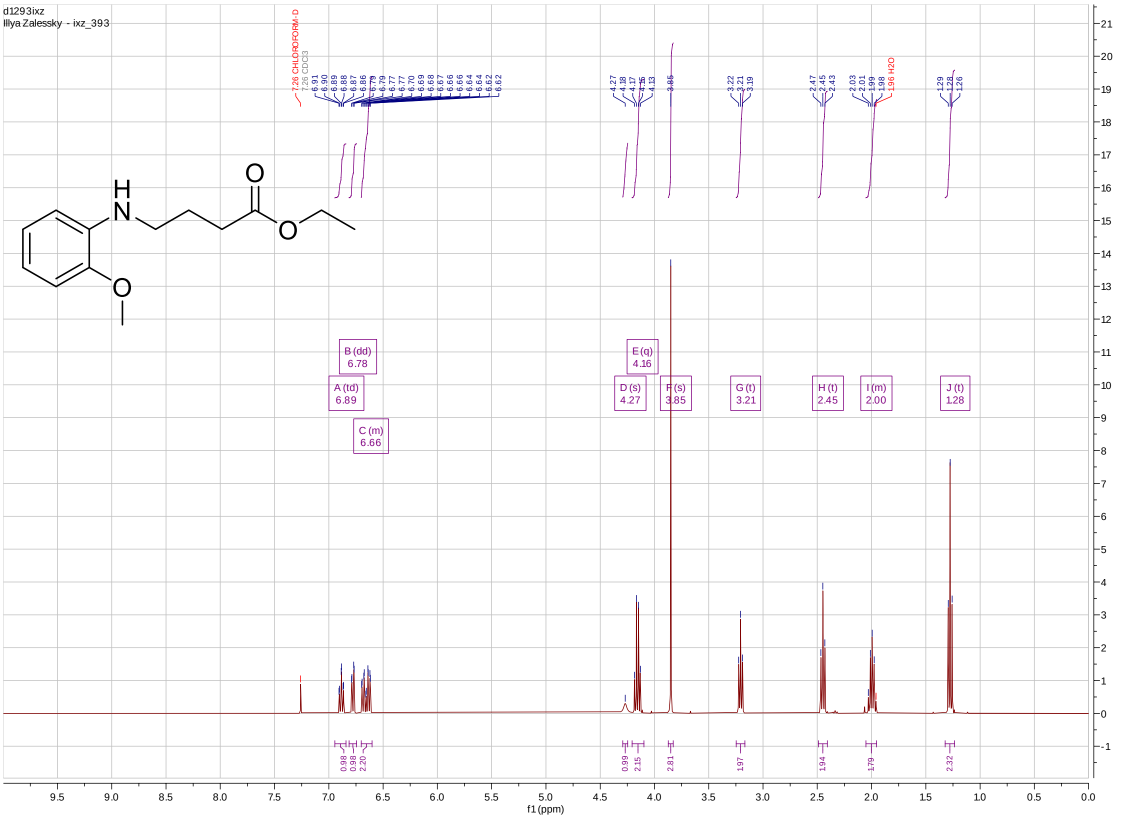

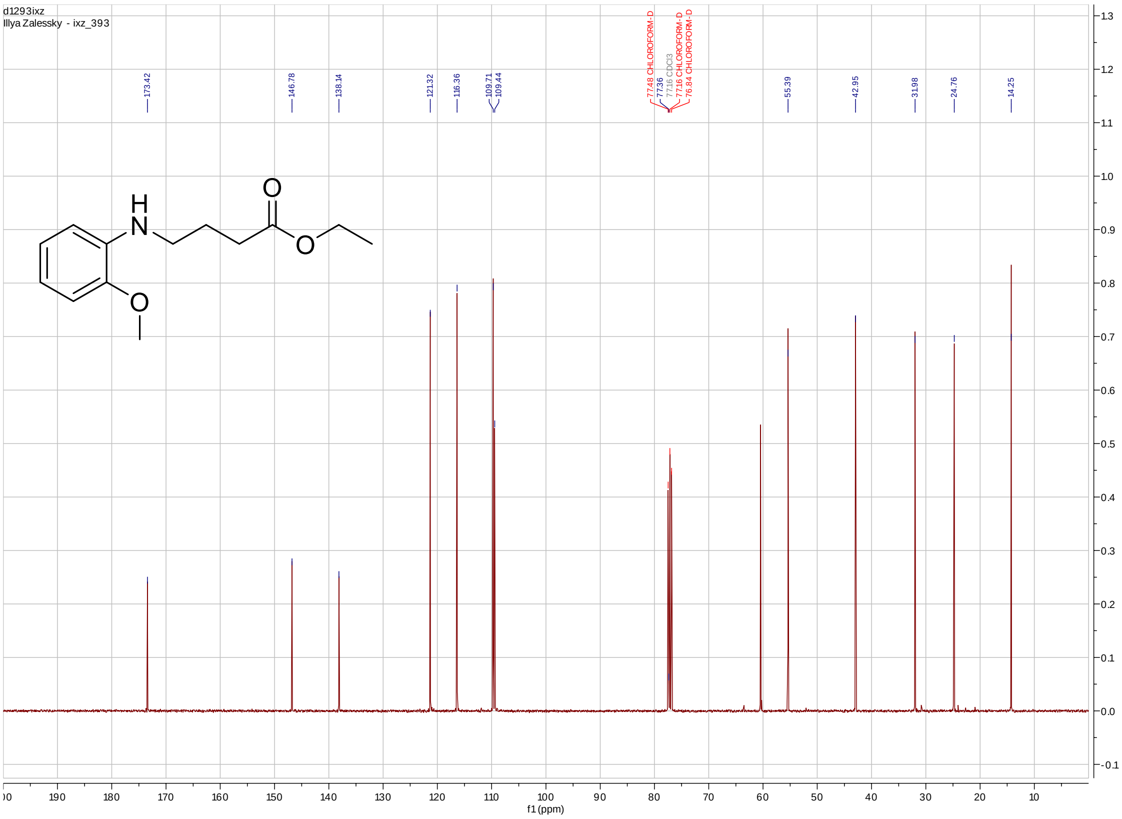

Ethyl 4-((2-methoxyphenyl)(methyl)amino)butanoate (S113)

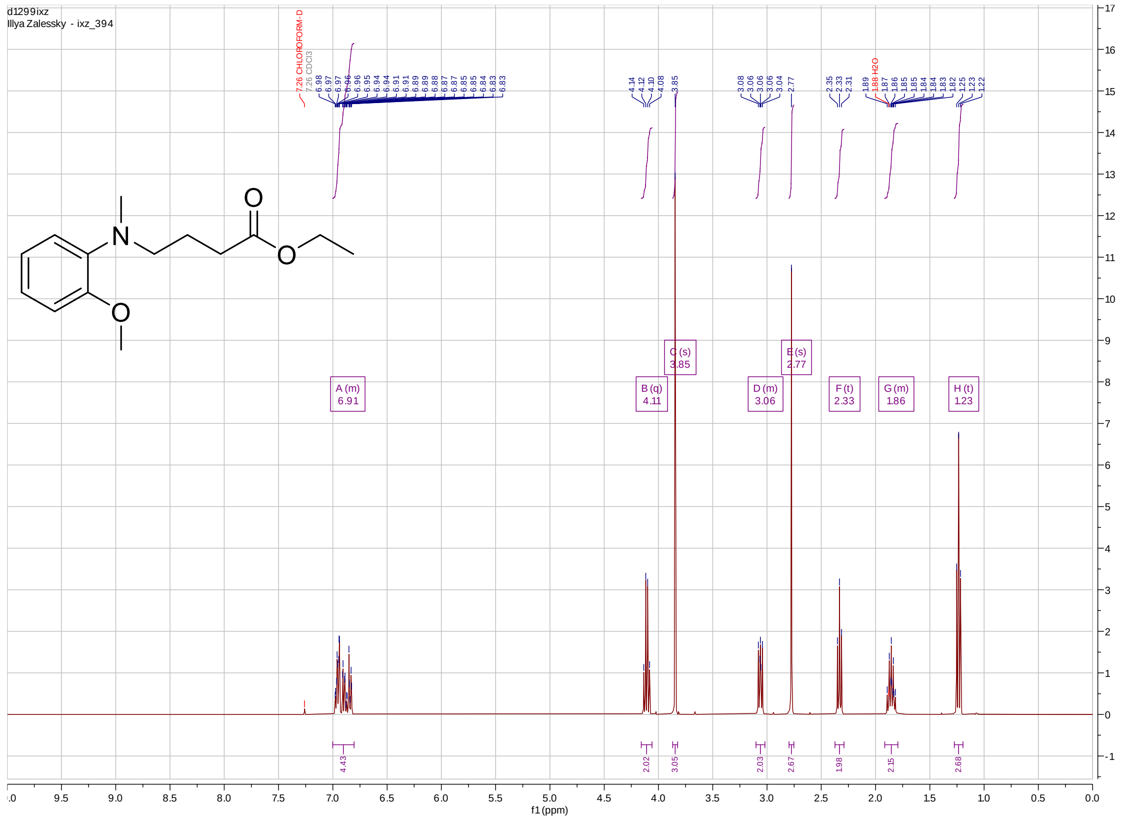

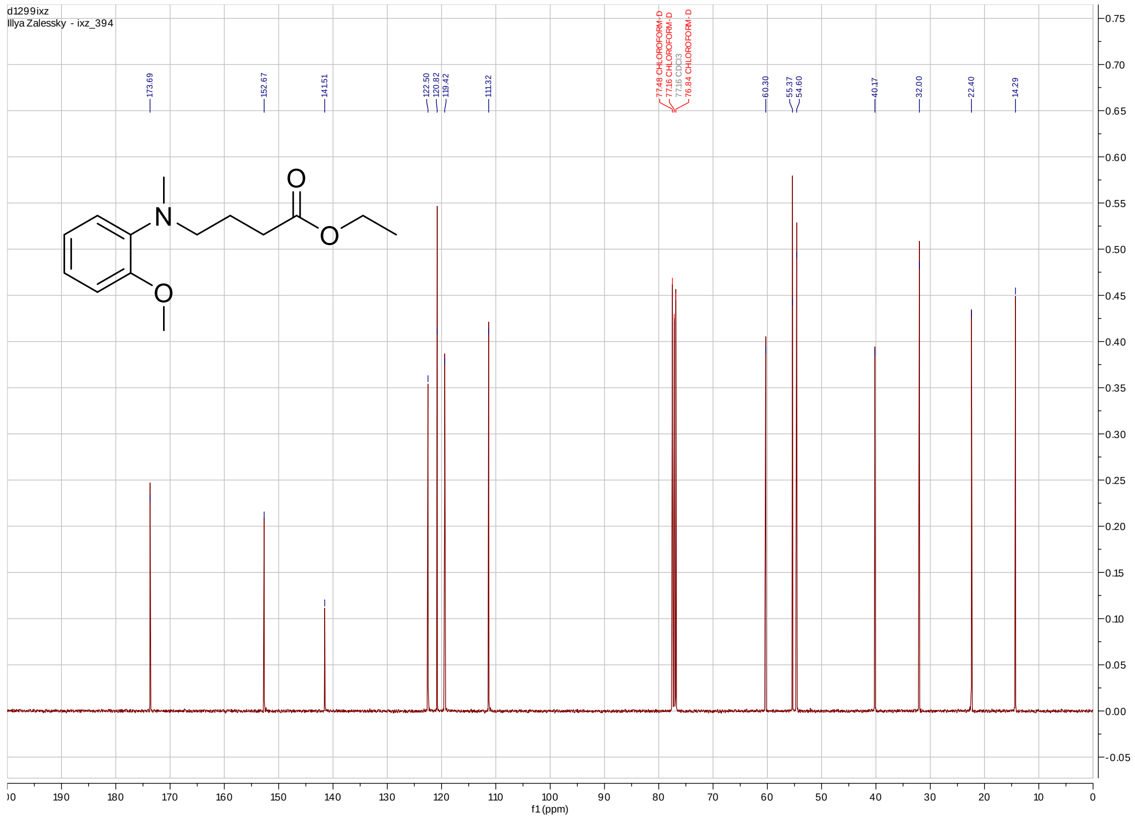

6-Methyl-3,4,5,6-tetrahydro-2H-benzo[b][1,4]oxazocin-2-one (81)

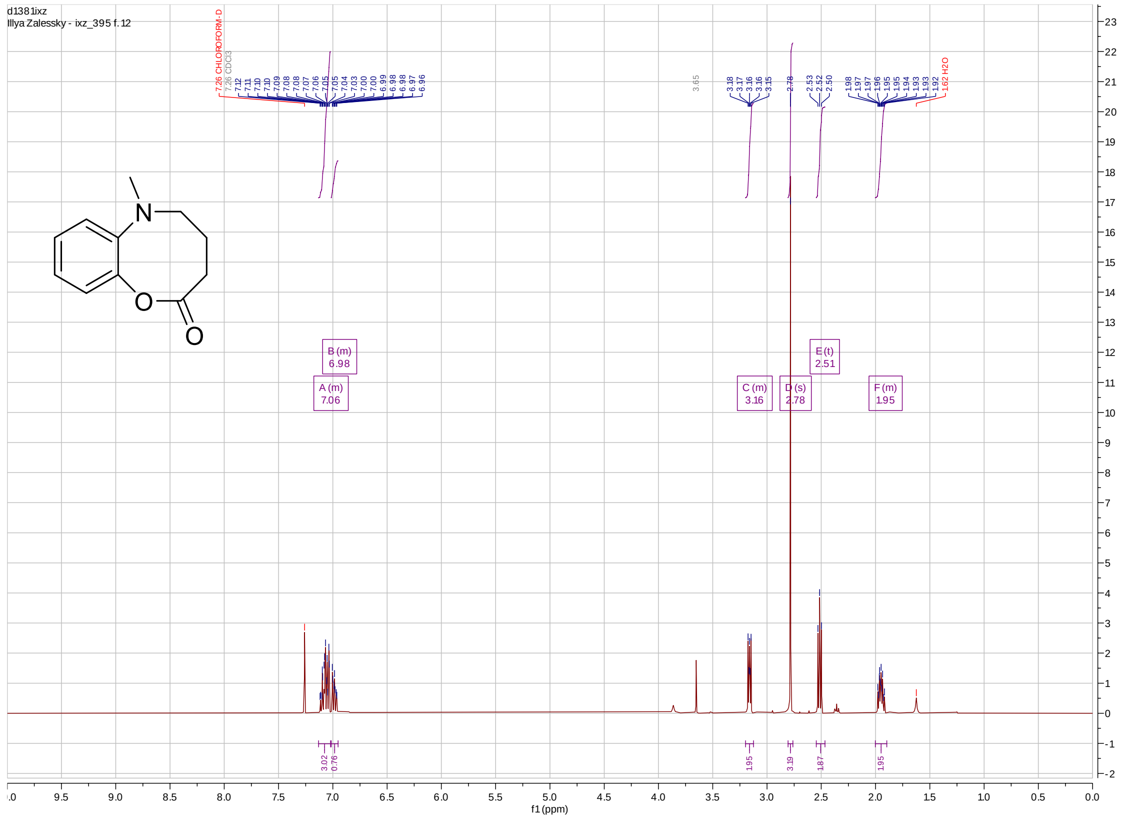

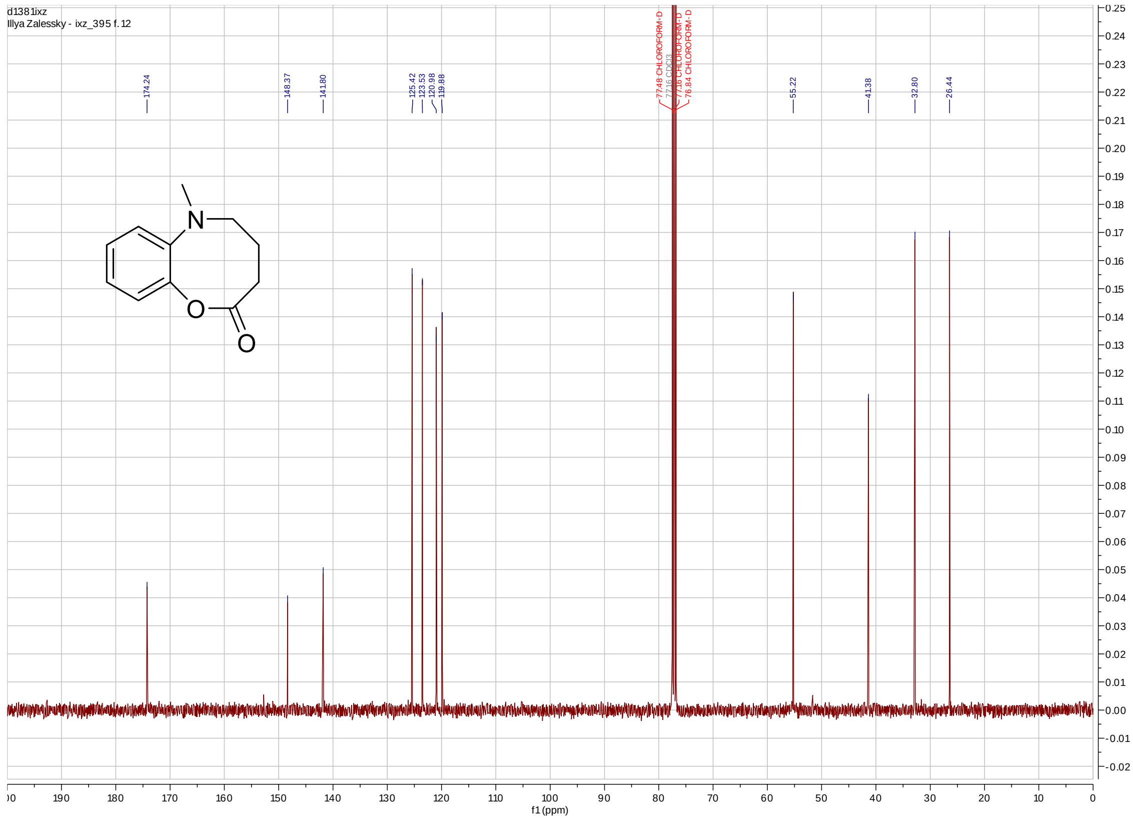

2-[(*tert*-Butyldimethylsilyl)oxy]aniline (S115)

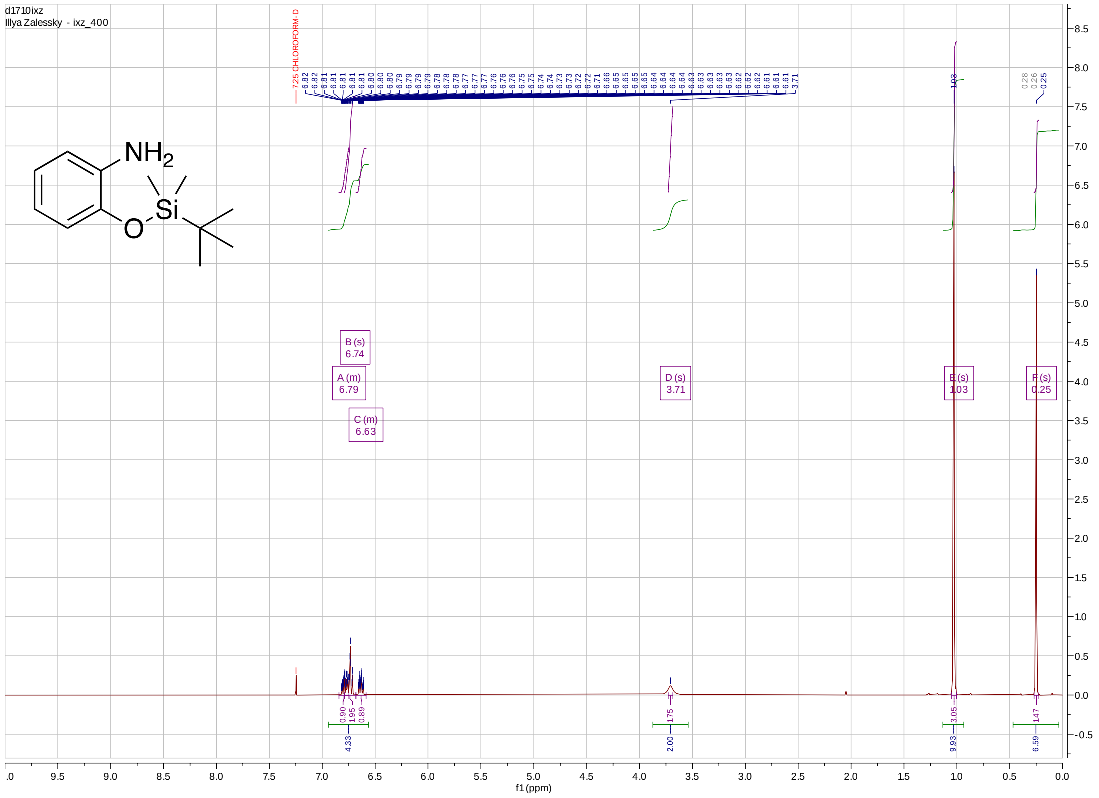

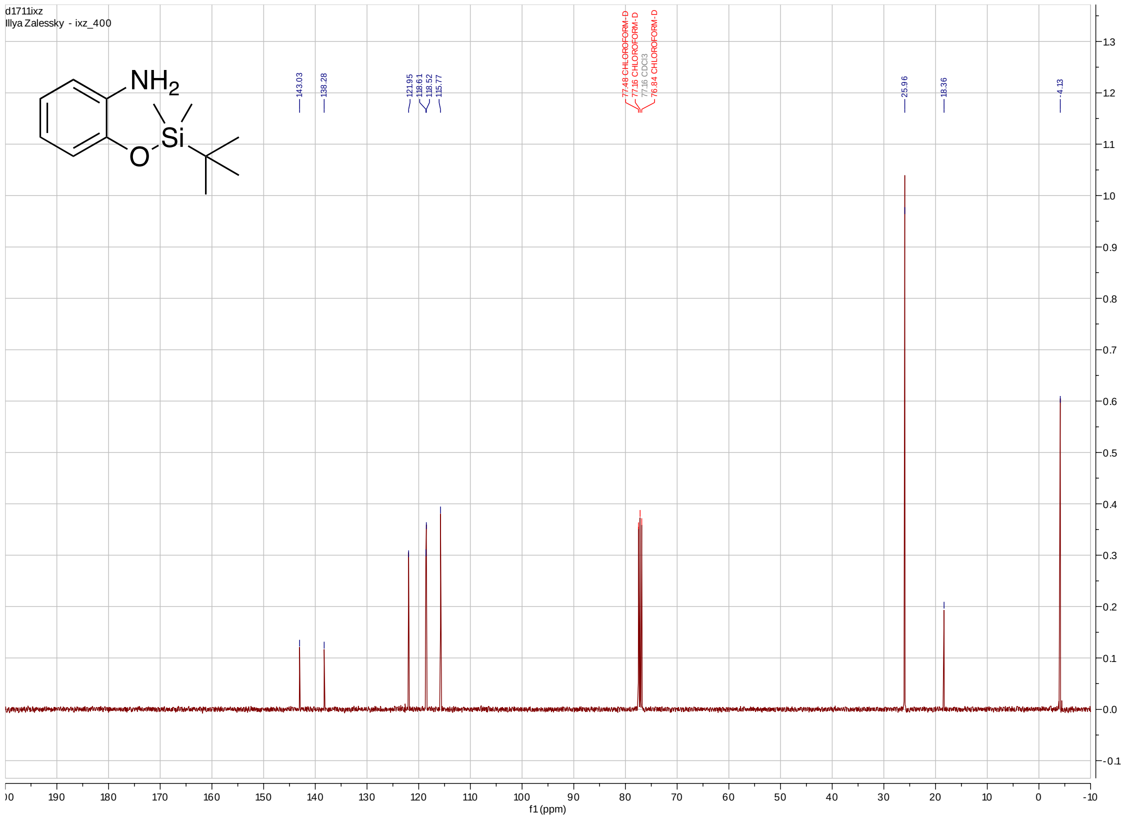

N-Benzyl-2-(tert-butyldimethylsilyloxy)aniline (S116)

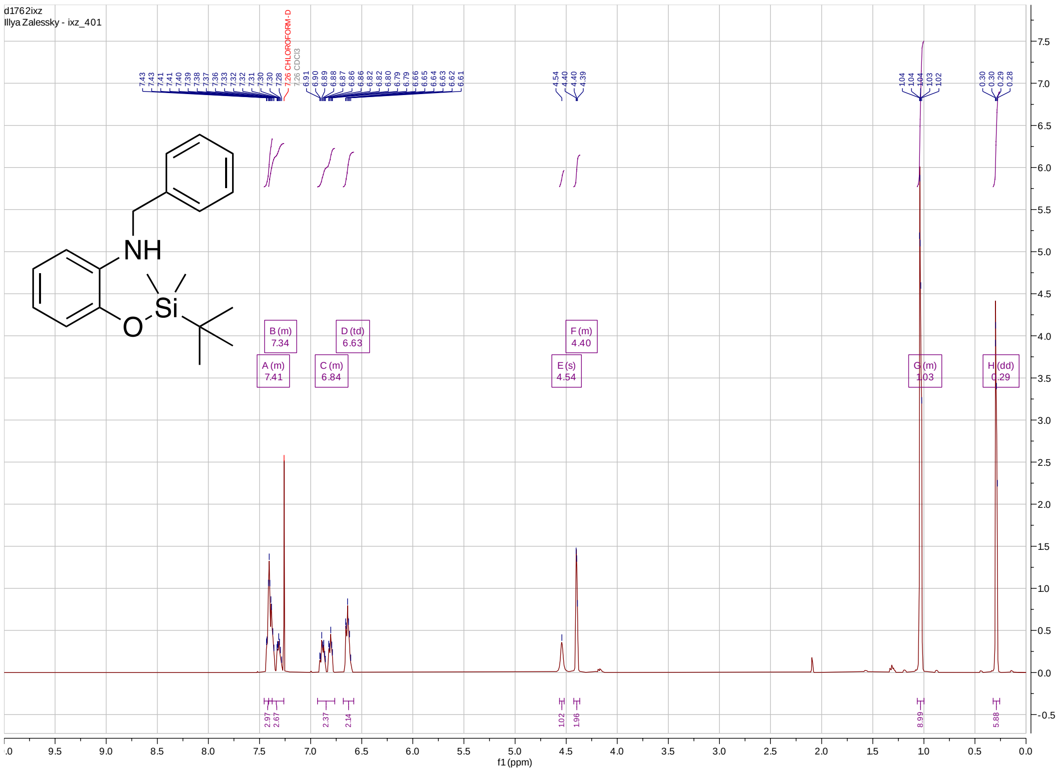

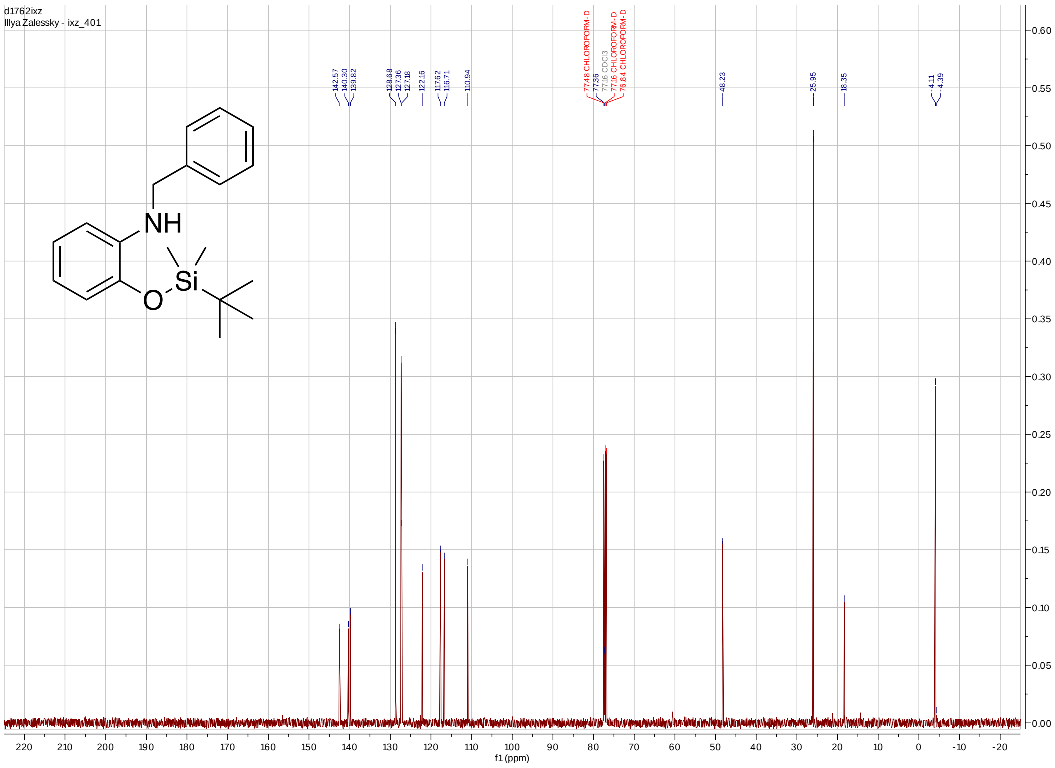

tert-Butyl 3-(benzyl(2-hydroxyphenyl)amino)propanoate (S117)

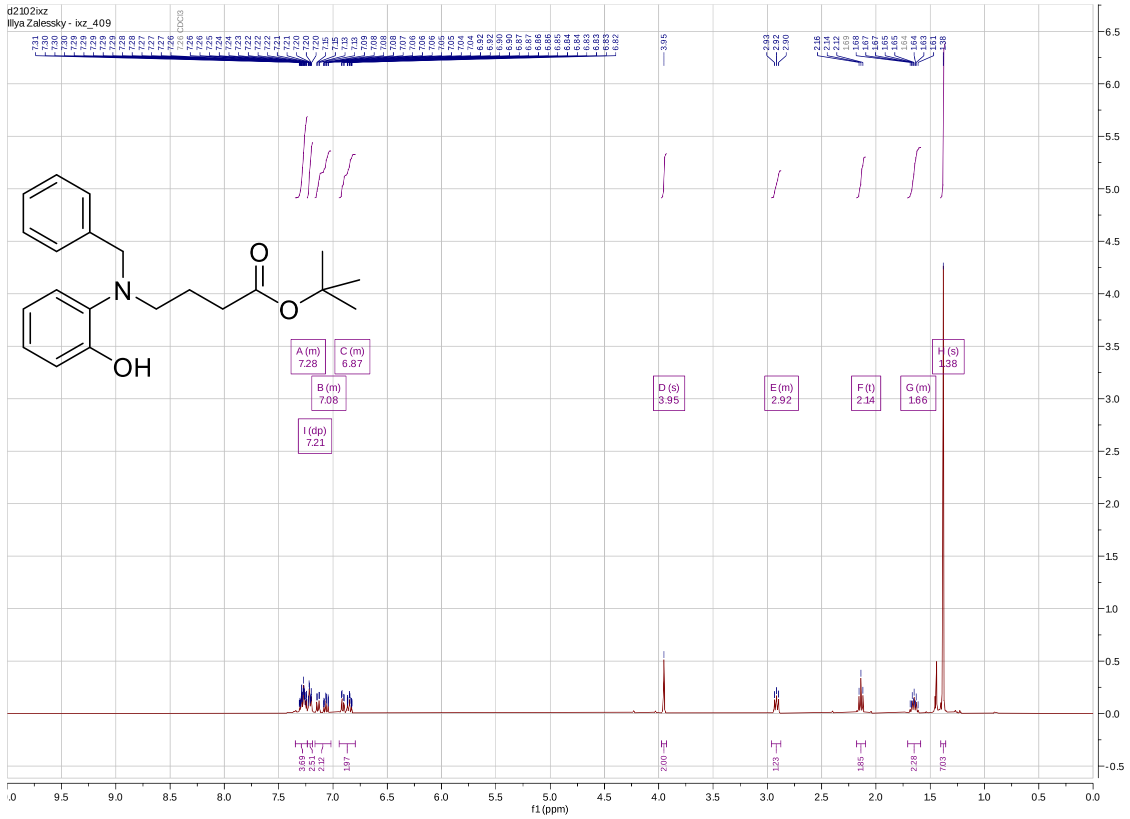

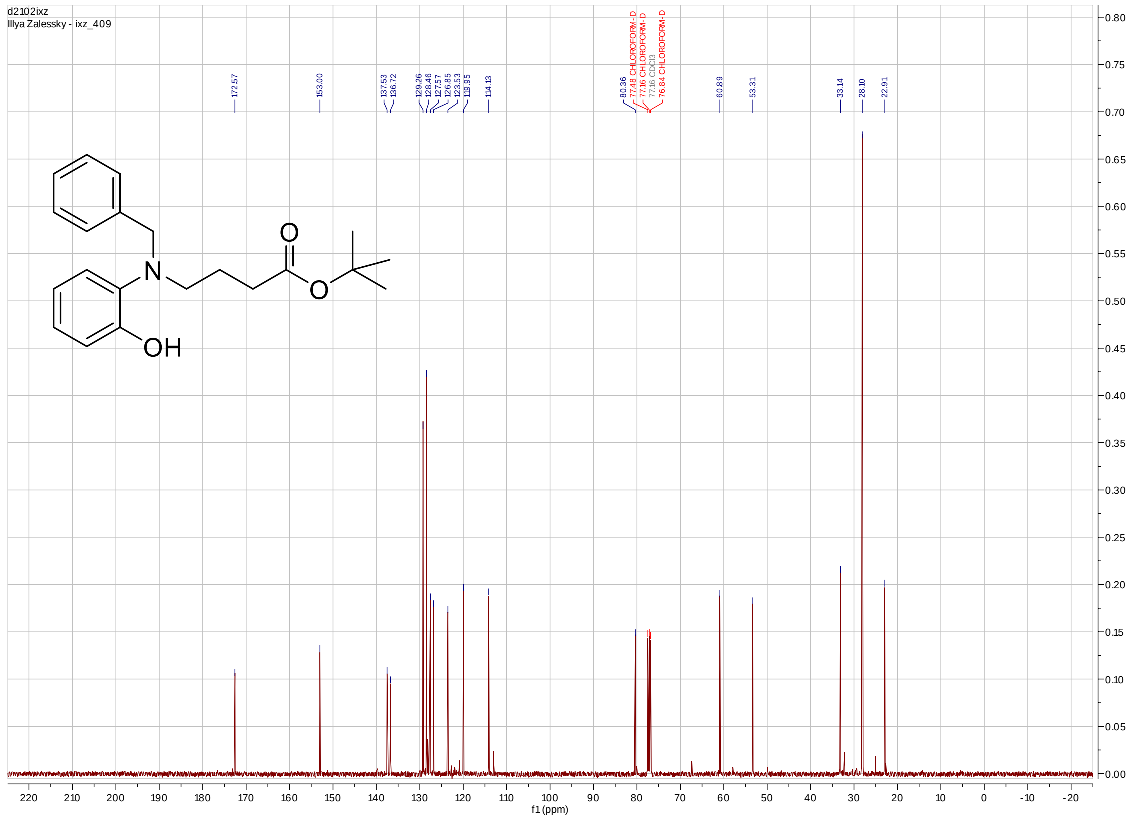

Synthesis of 5-benzyl-4,5-dihydrobenzo[b][1,4]oxazepin-2(3H)-one (82)

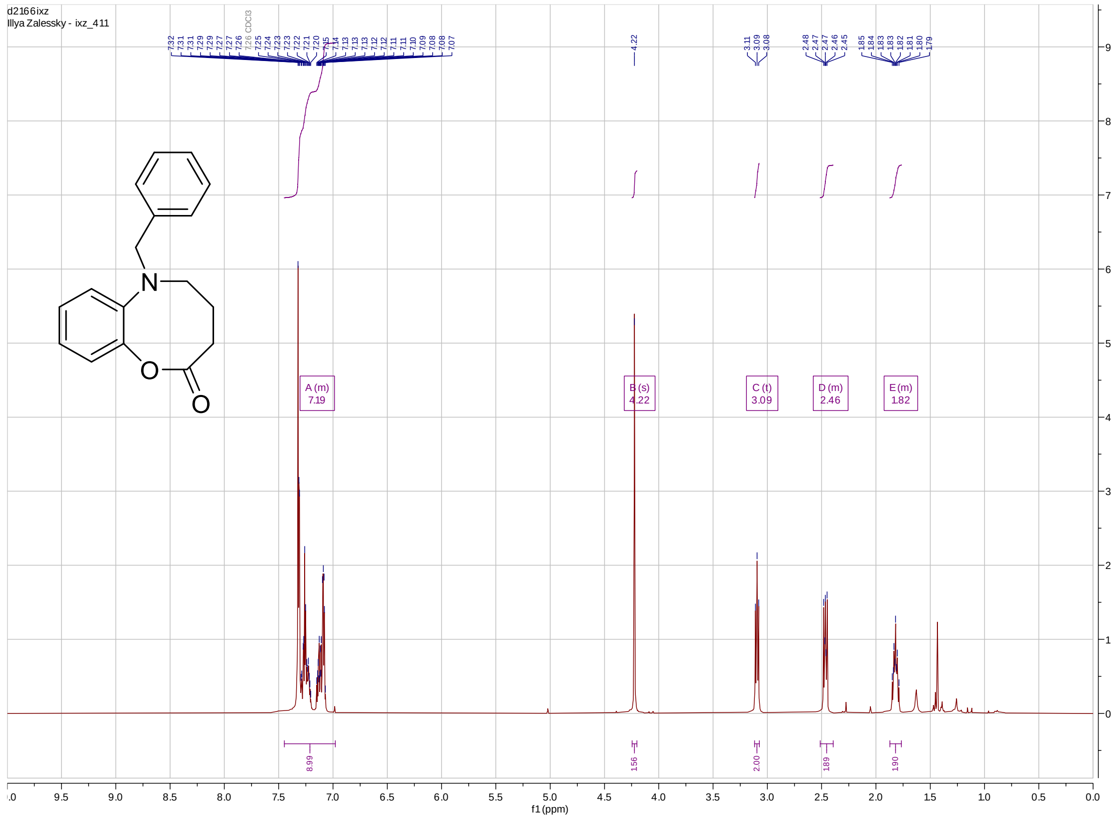

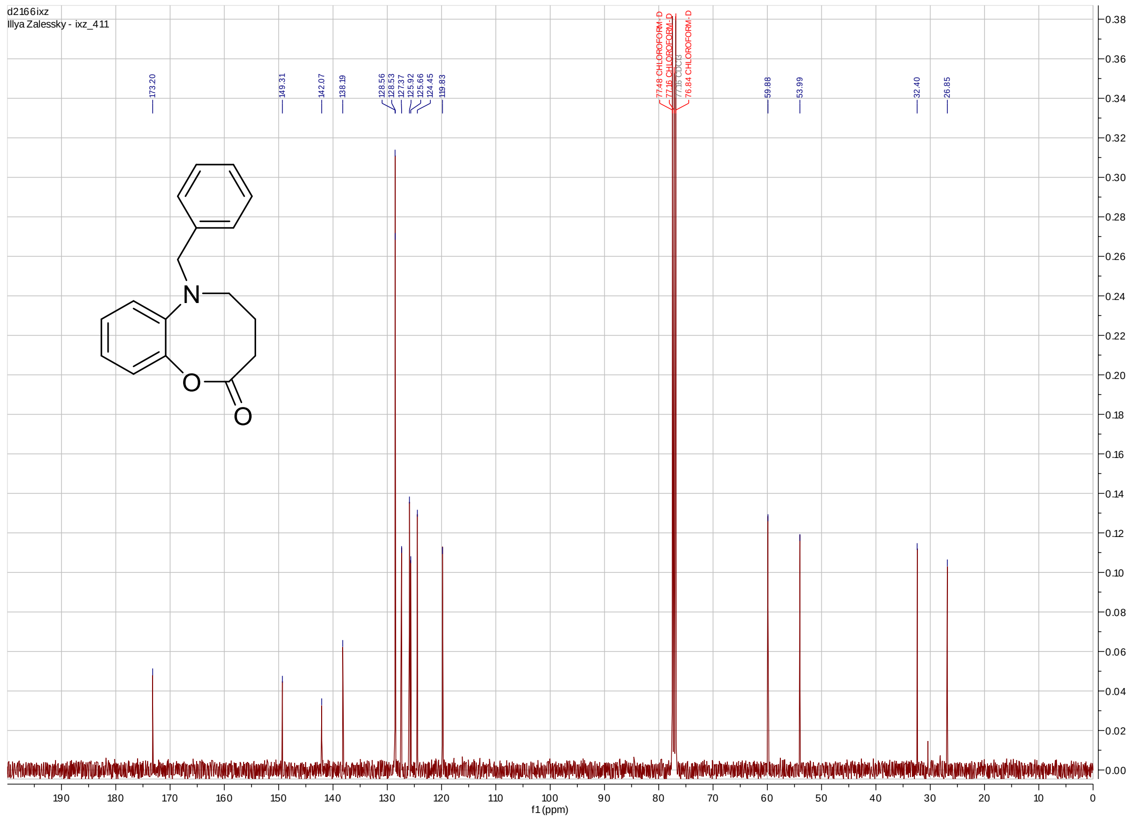

2-(6-Vinylpyridin-2-yl)ethan-1-ol (S119)

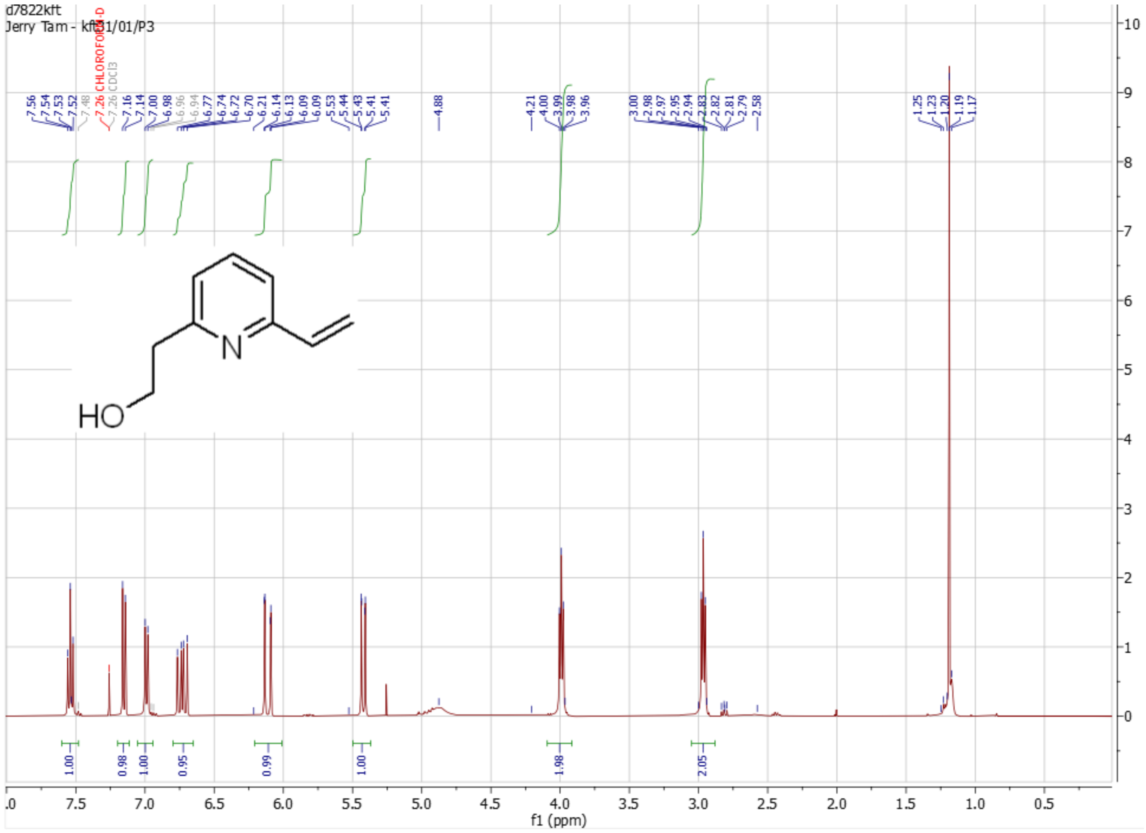

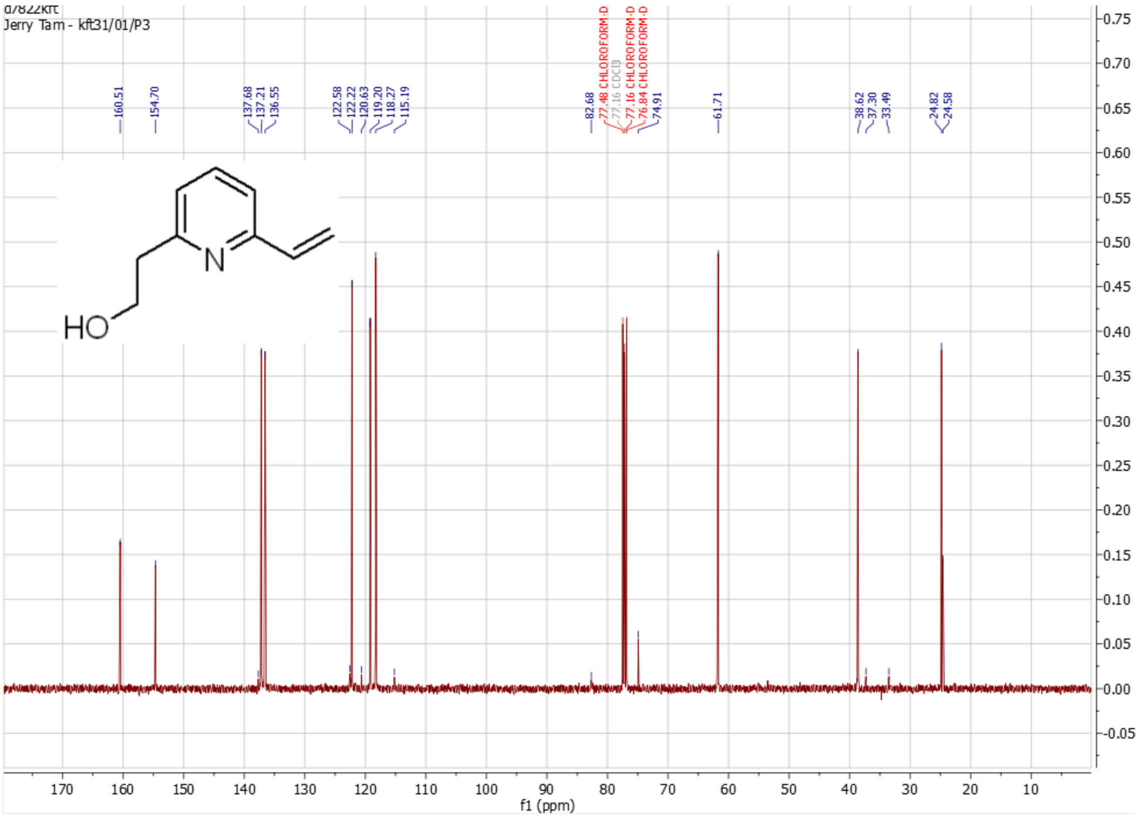

2-(6-(2-(Phenylamino)ethyl)pyridin-2-yl)ethan-1-ol (S120)

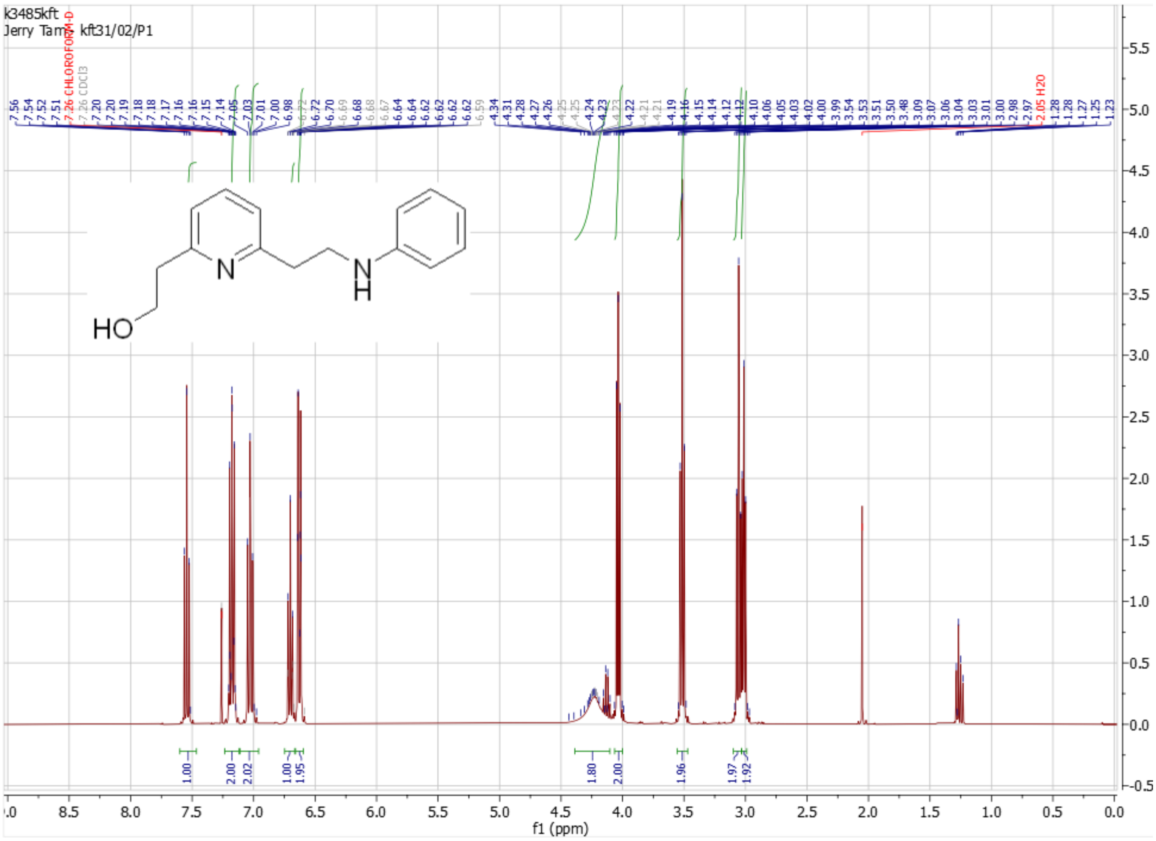

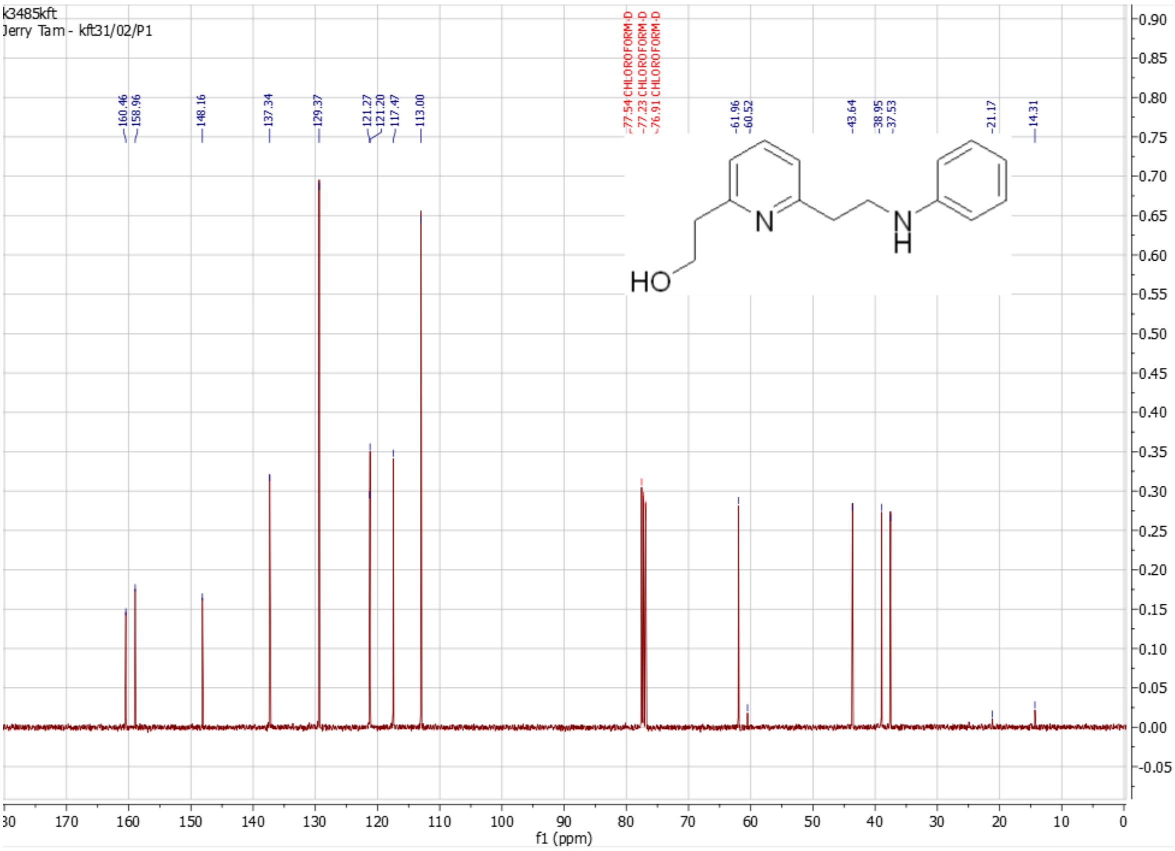

Cyclic carbamate (83)

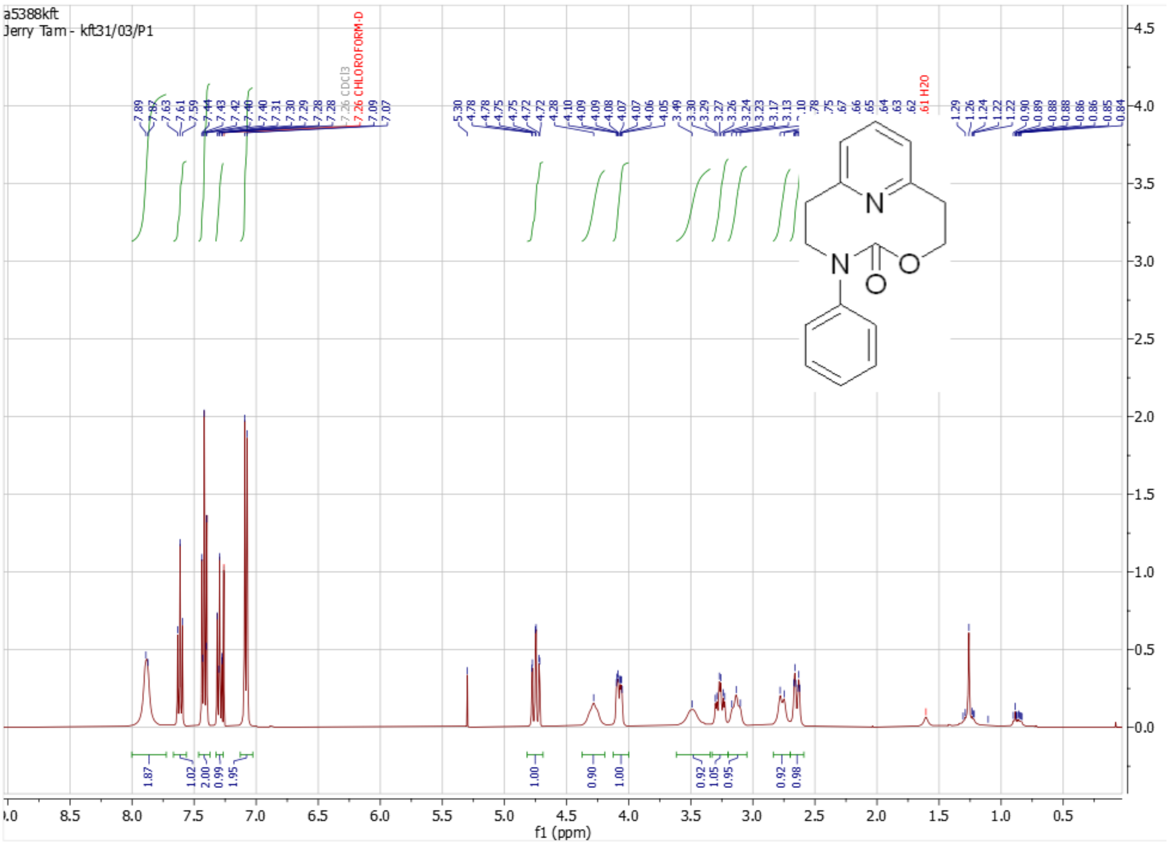

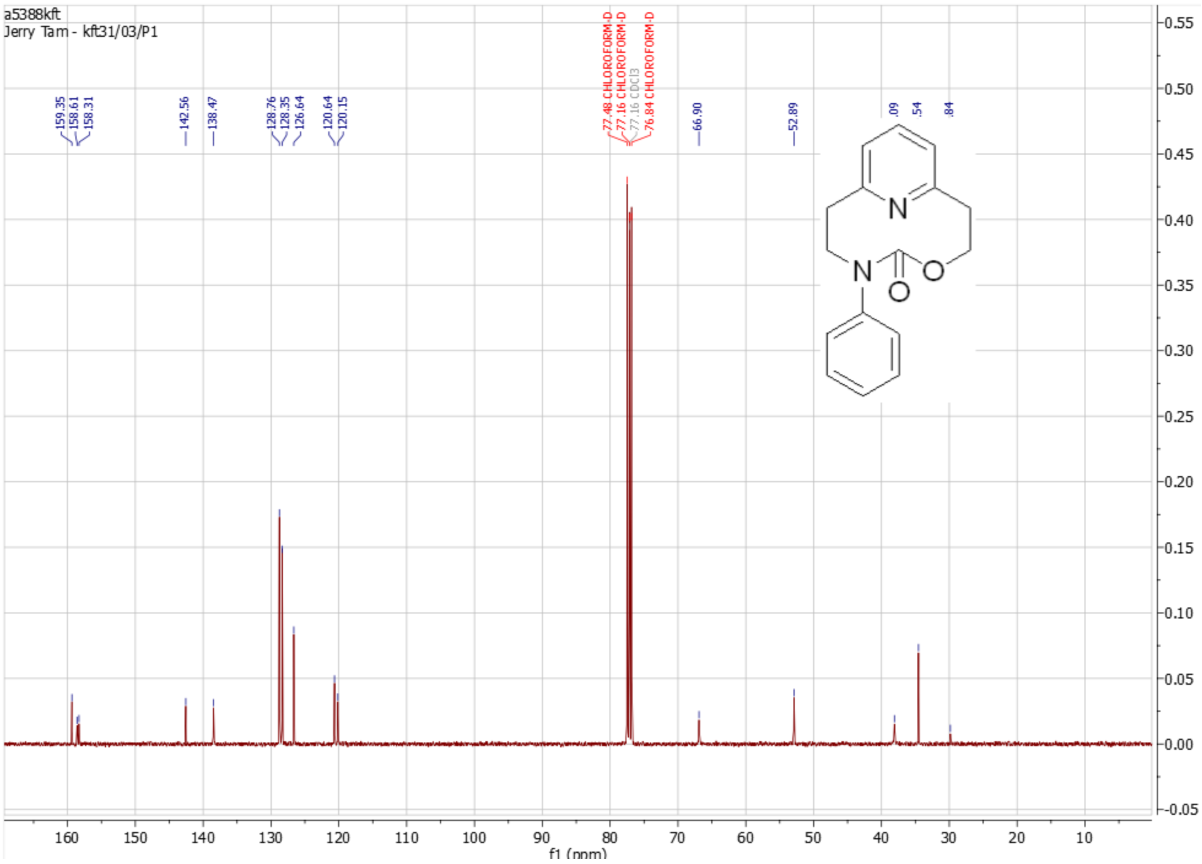

Ethyl 2-(6-bromopyridin-2-yl)acetate (S121)

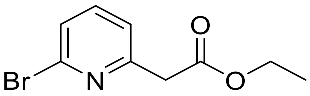

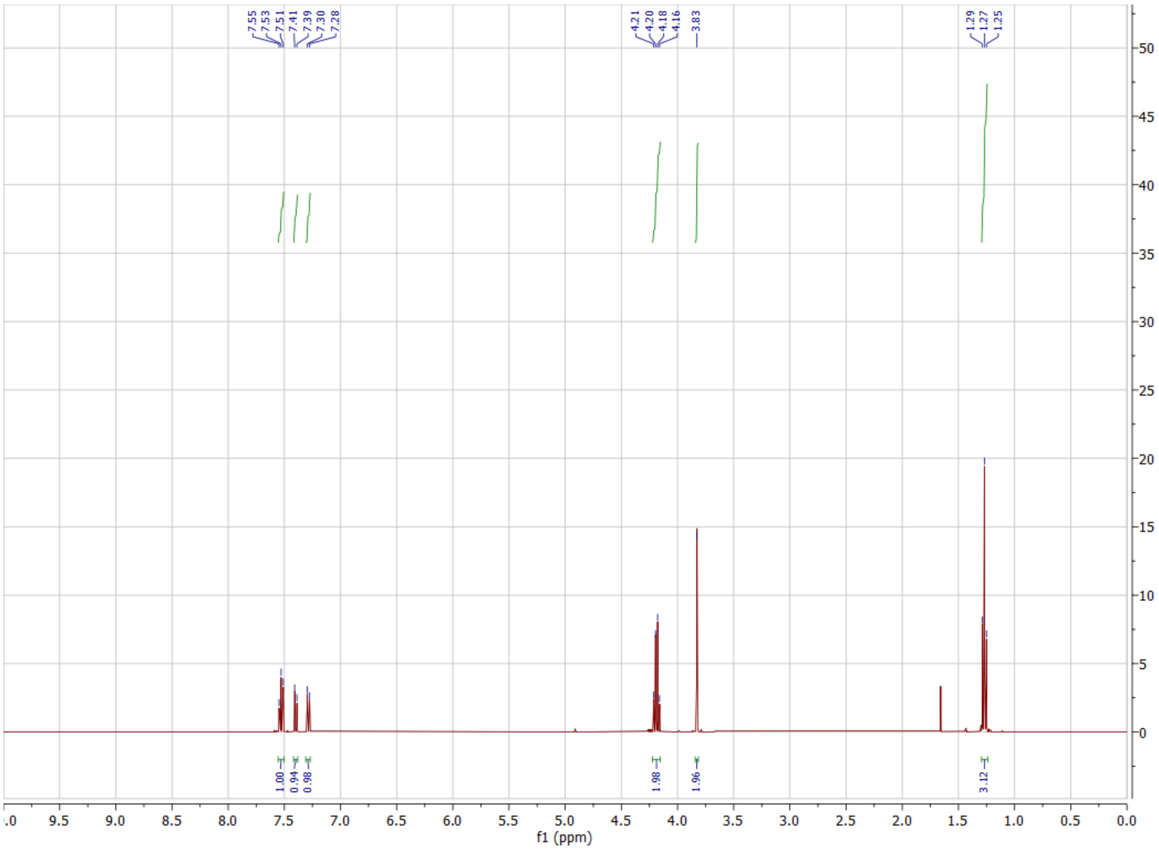

2-(6-Bromopyridin-2-yl)ethan-1-ol (S122)

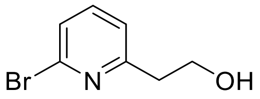

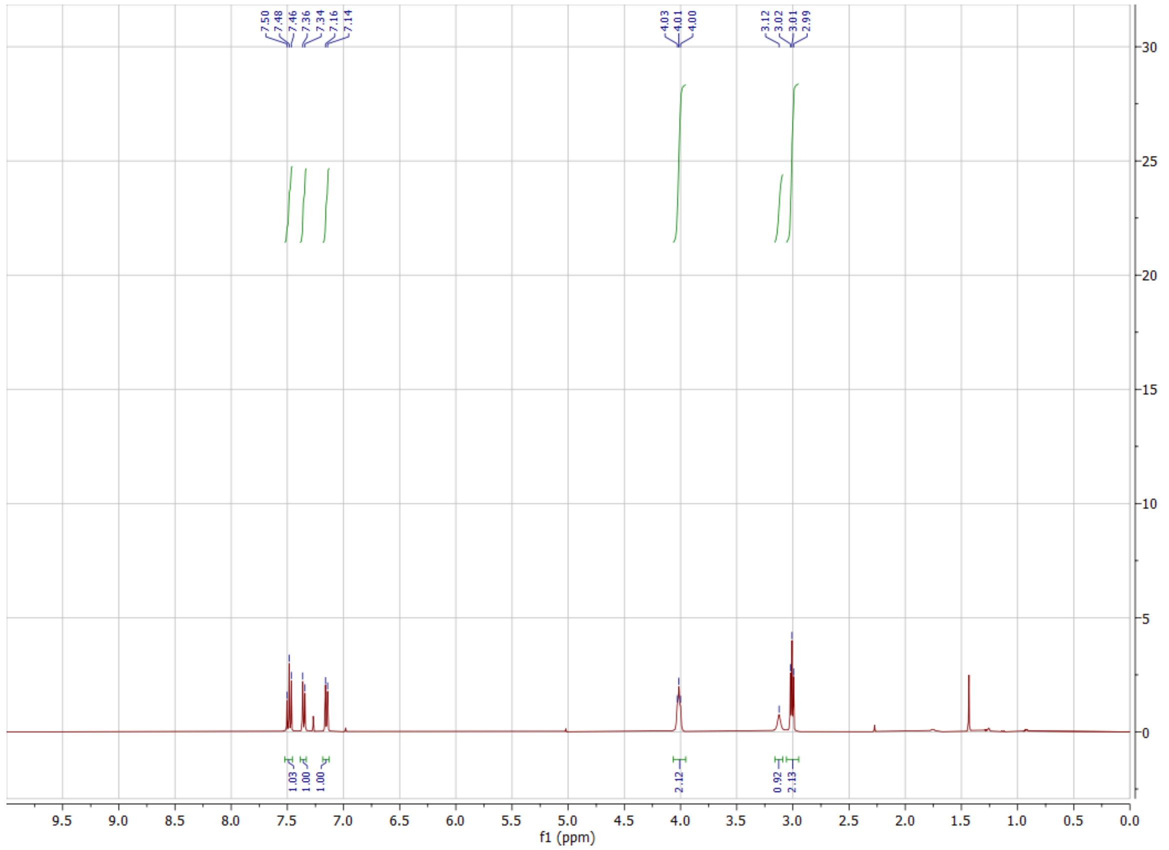

_N_-Methyl-2-(4,4,5,5-tetramethyl-1,3,2-dioxaborolan-2-yl)aniline (S123)

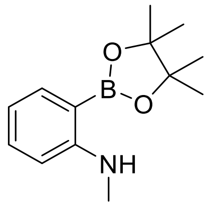

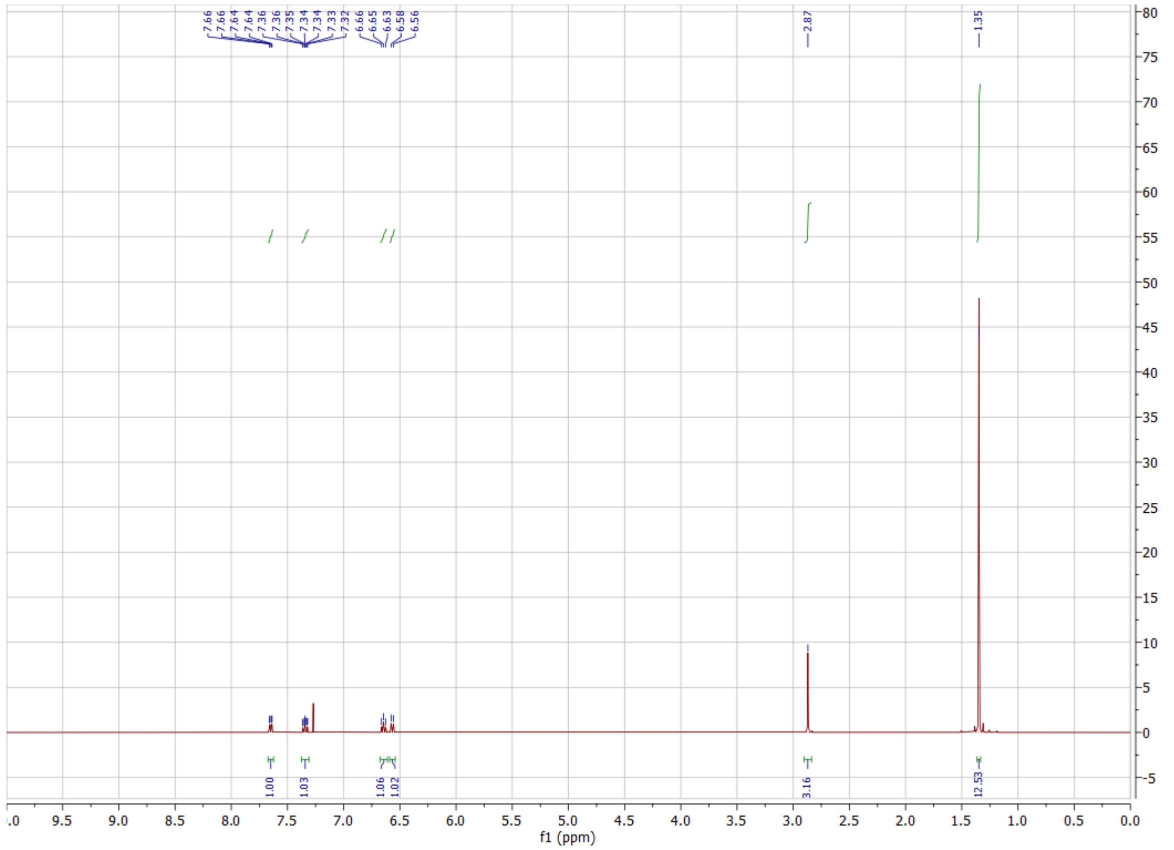

2-(6-(2-(Methylamino)phenyl)pyridin-2-yl)ethan-1-ol (S124)

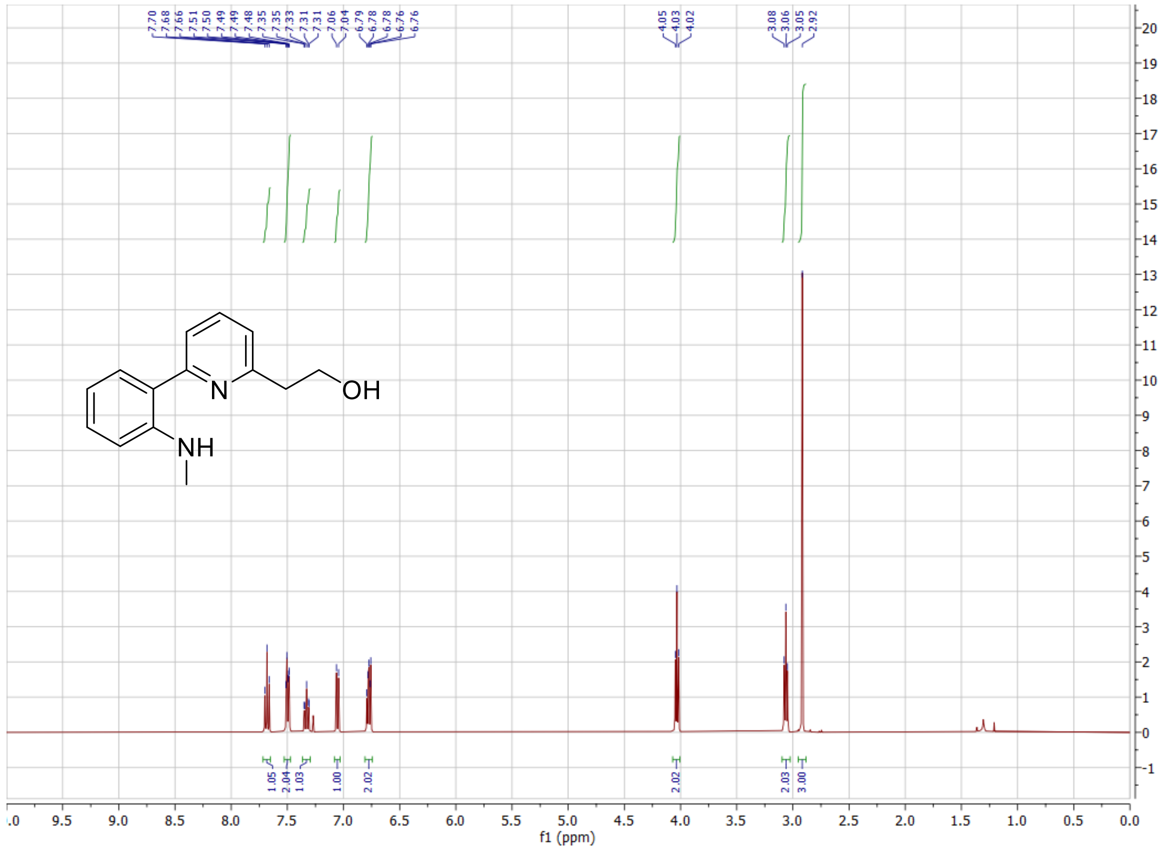

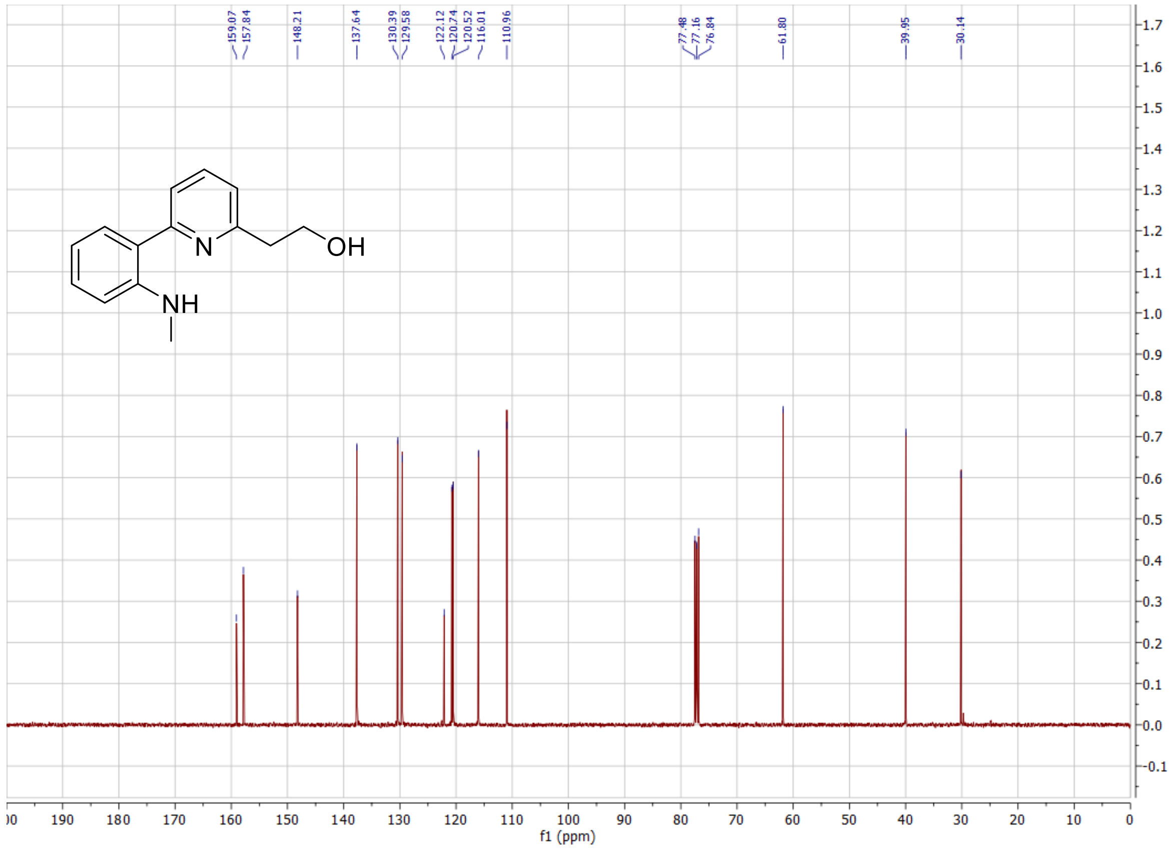

1-Methyl-4,5-dihydro-6,10-(azeno)benzo[d][1]oxa[3]azacyclododecin-2(1H)-one (84)

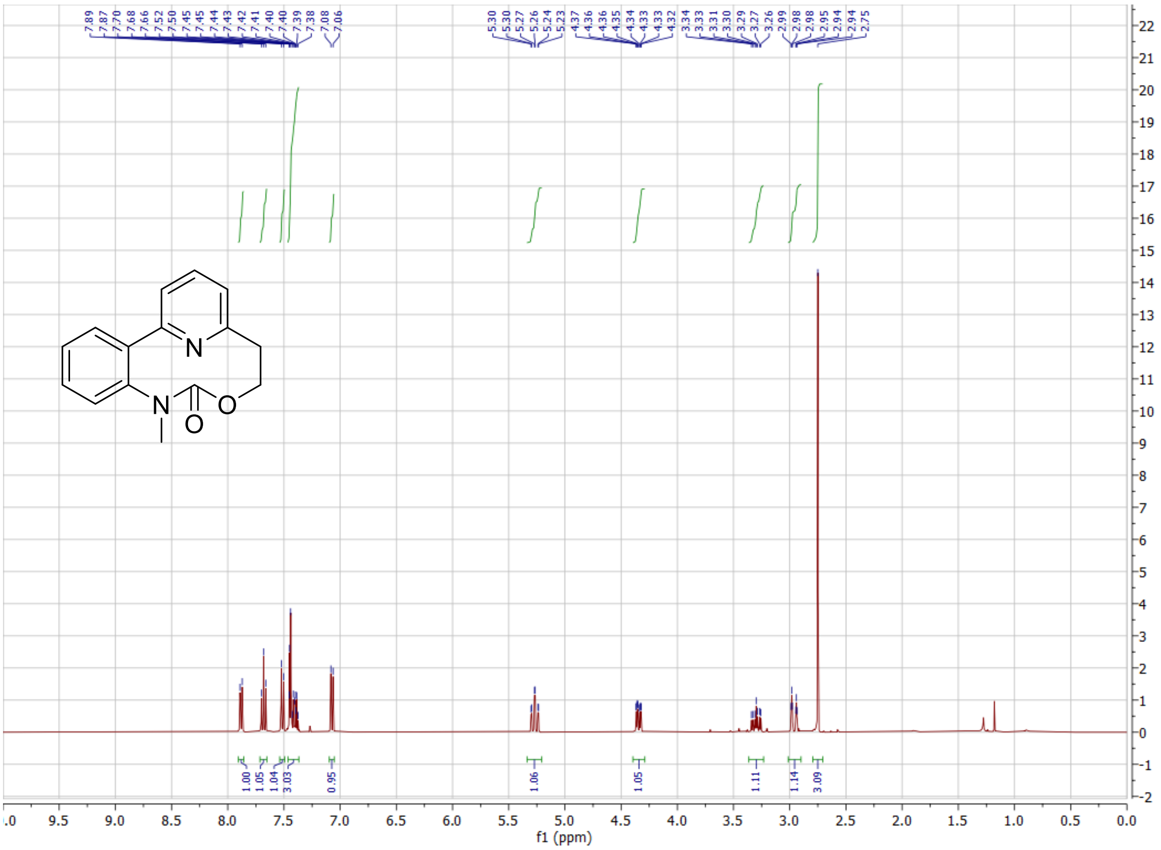

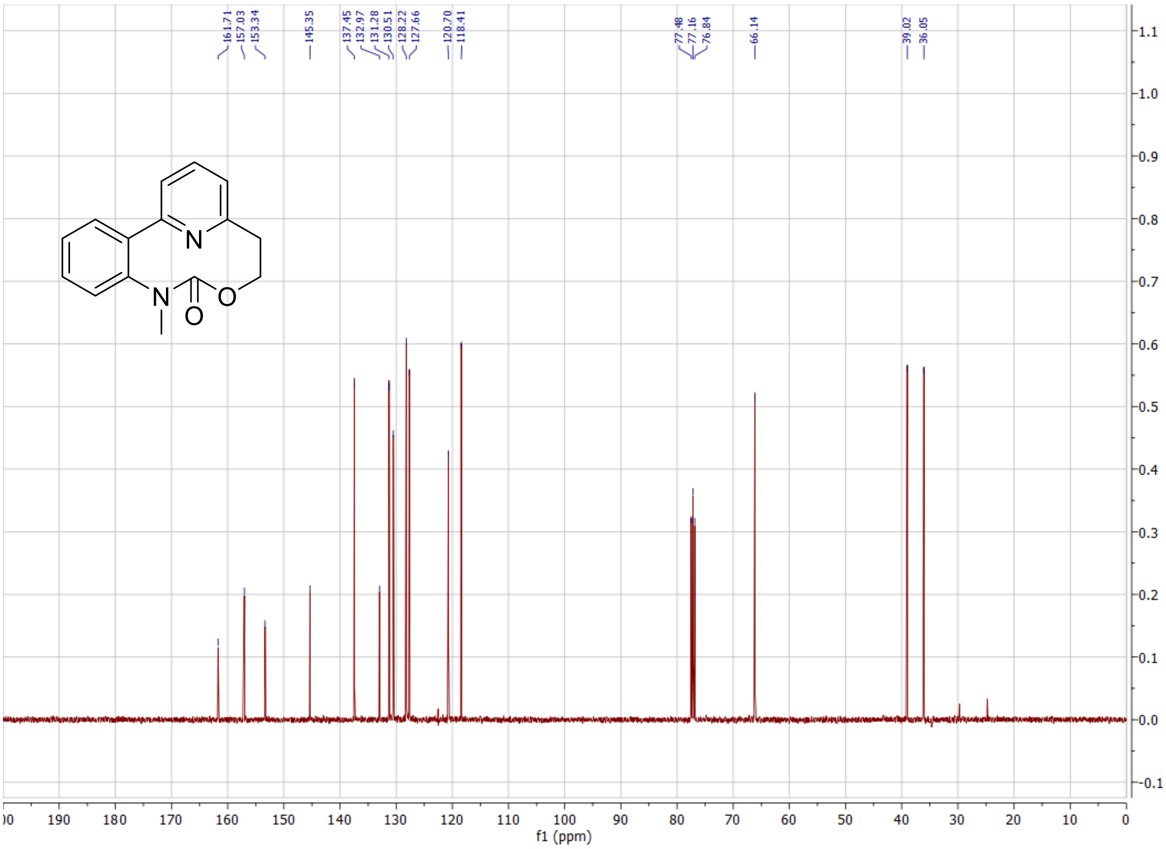

2-(6-(2-(Methylamino)phenyl)pyridin-2-yl)-1-phenylethan-1-ol (S126)

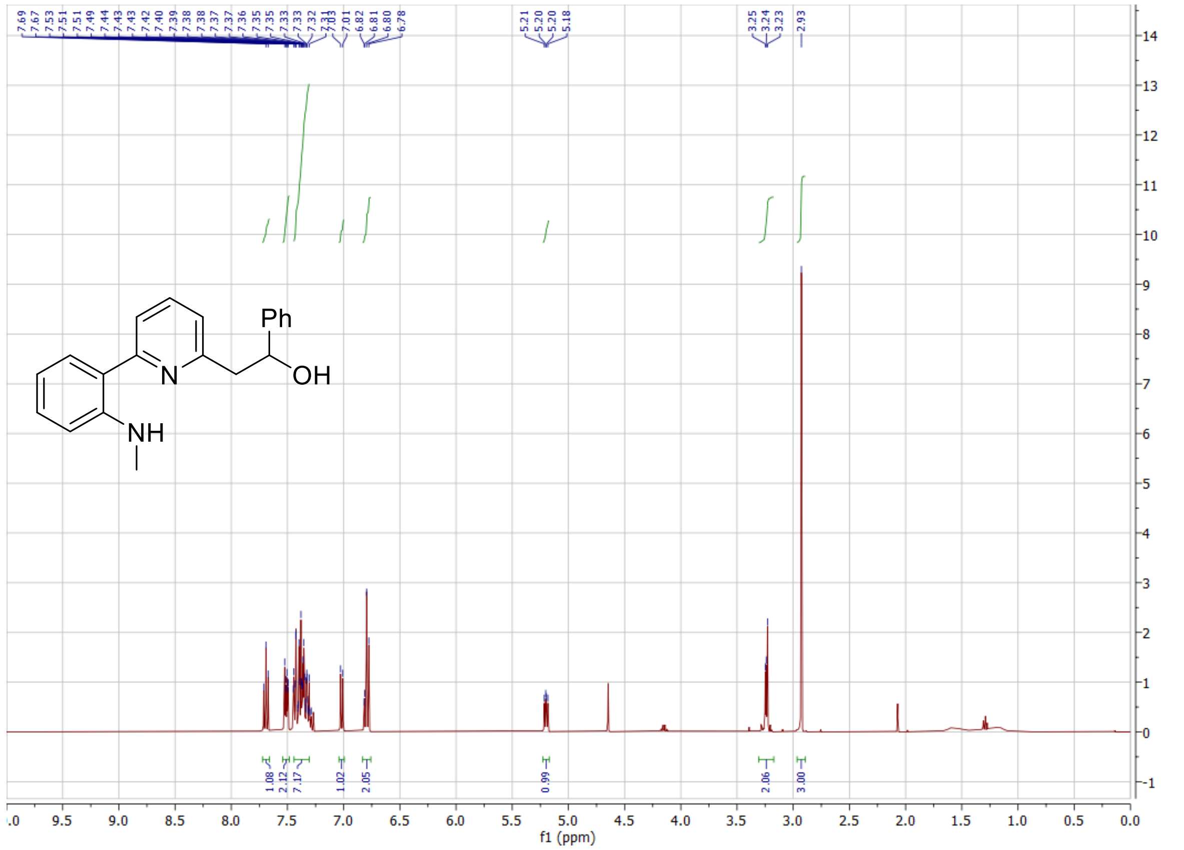

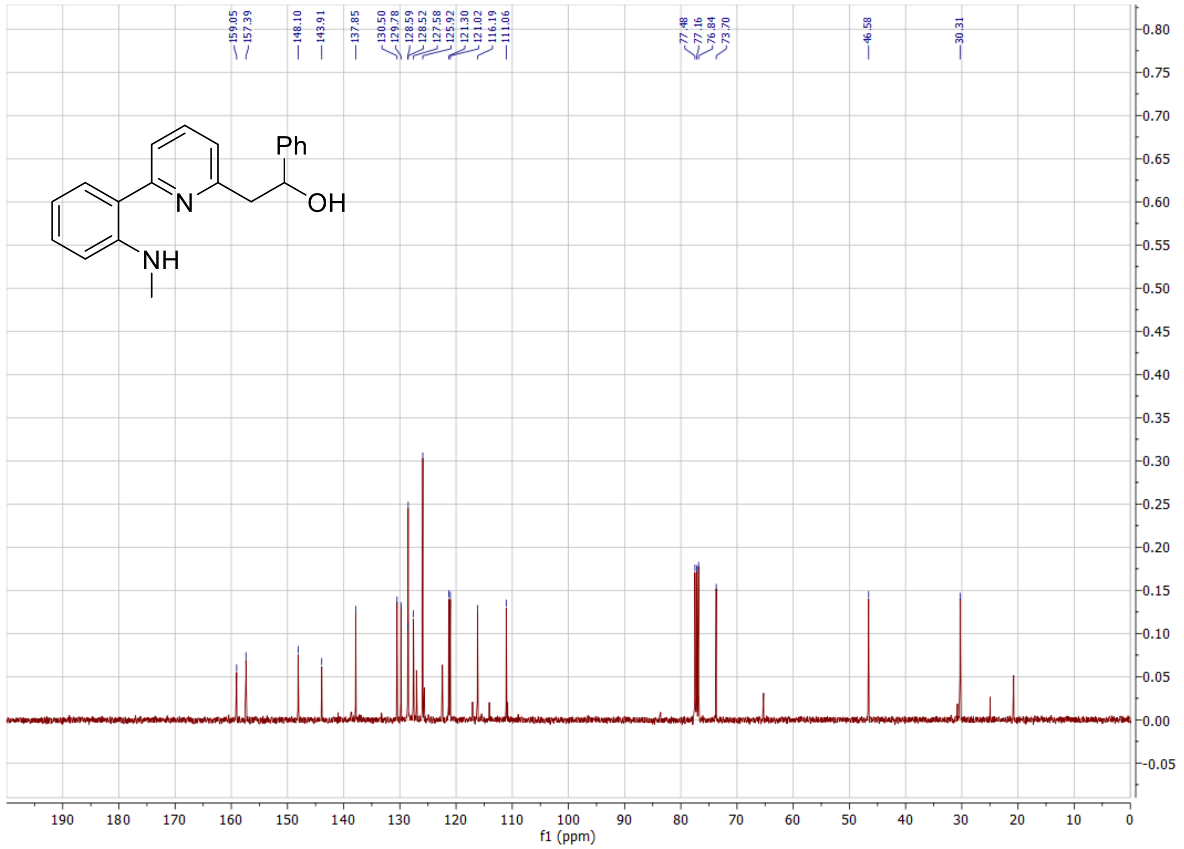

1-Methyl-4,5-dihydro-1H-6,10-(azeno)benzo[d][1]oxa[2]thia[3]azacyclododecine 2-oxide (86)

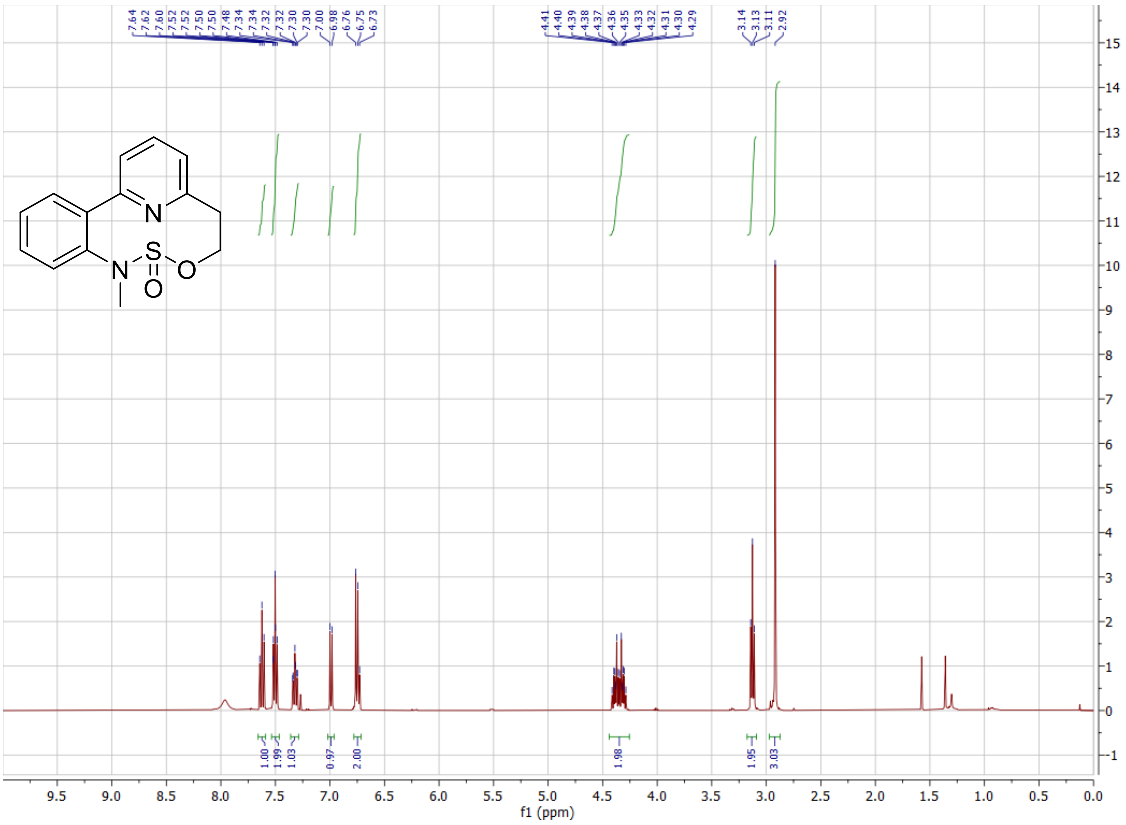

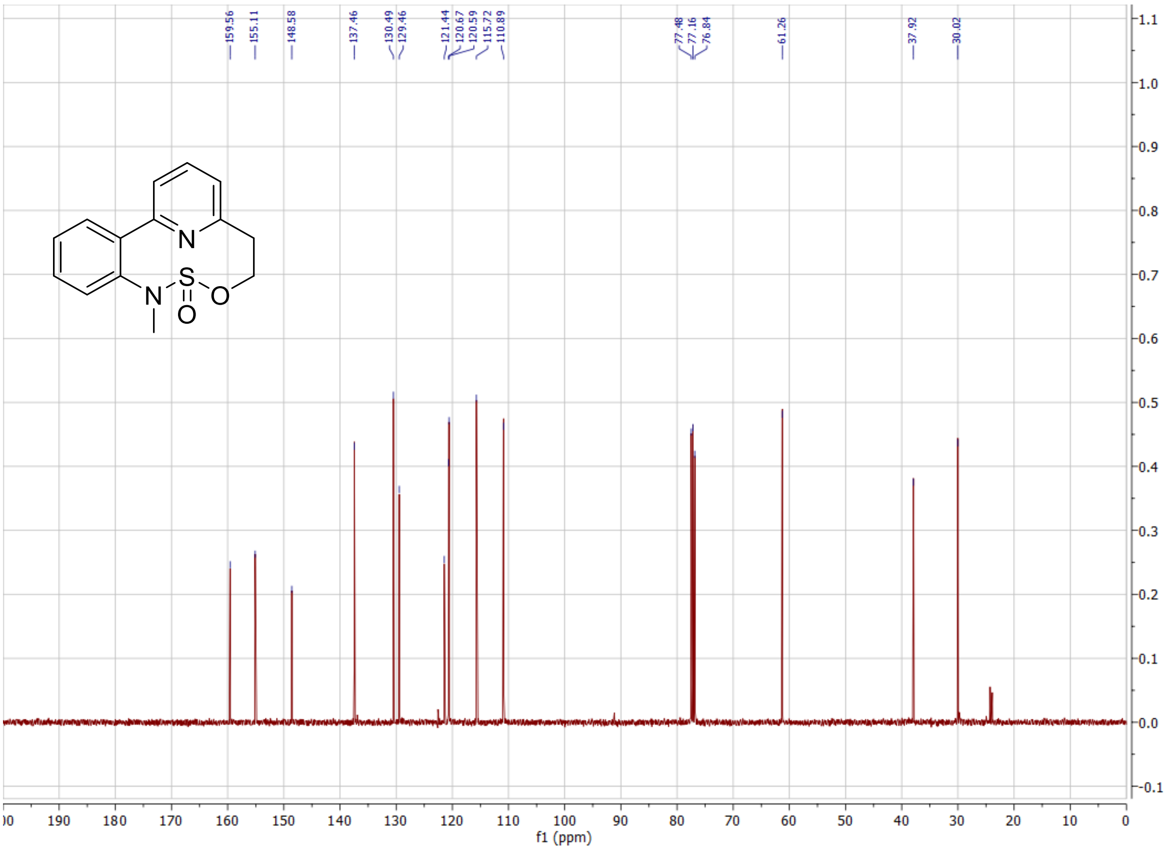

(2-(6-(2-(Phenylamino)propyl)pyridin-2-yl)phenyl)methanol (S127)

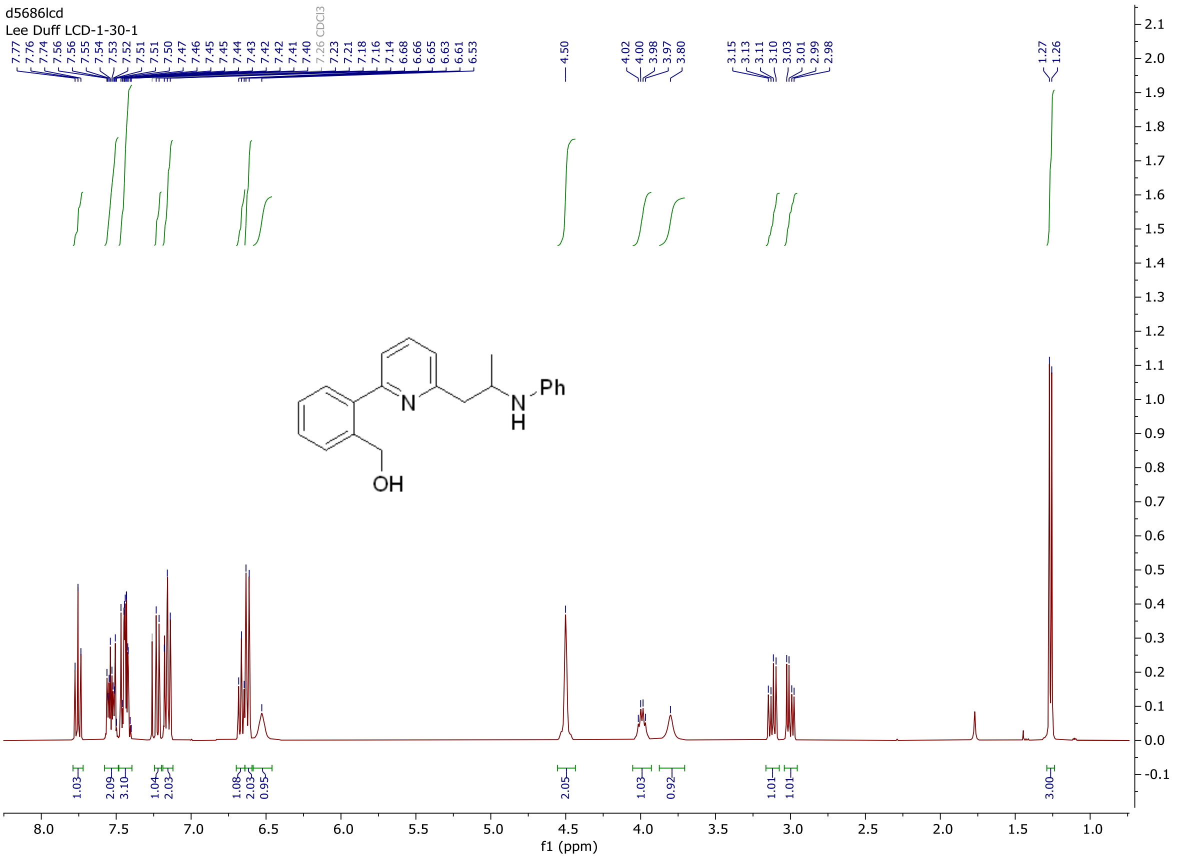

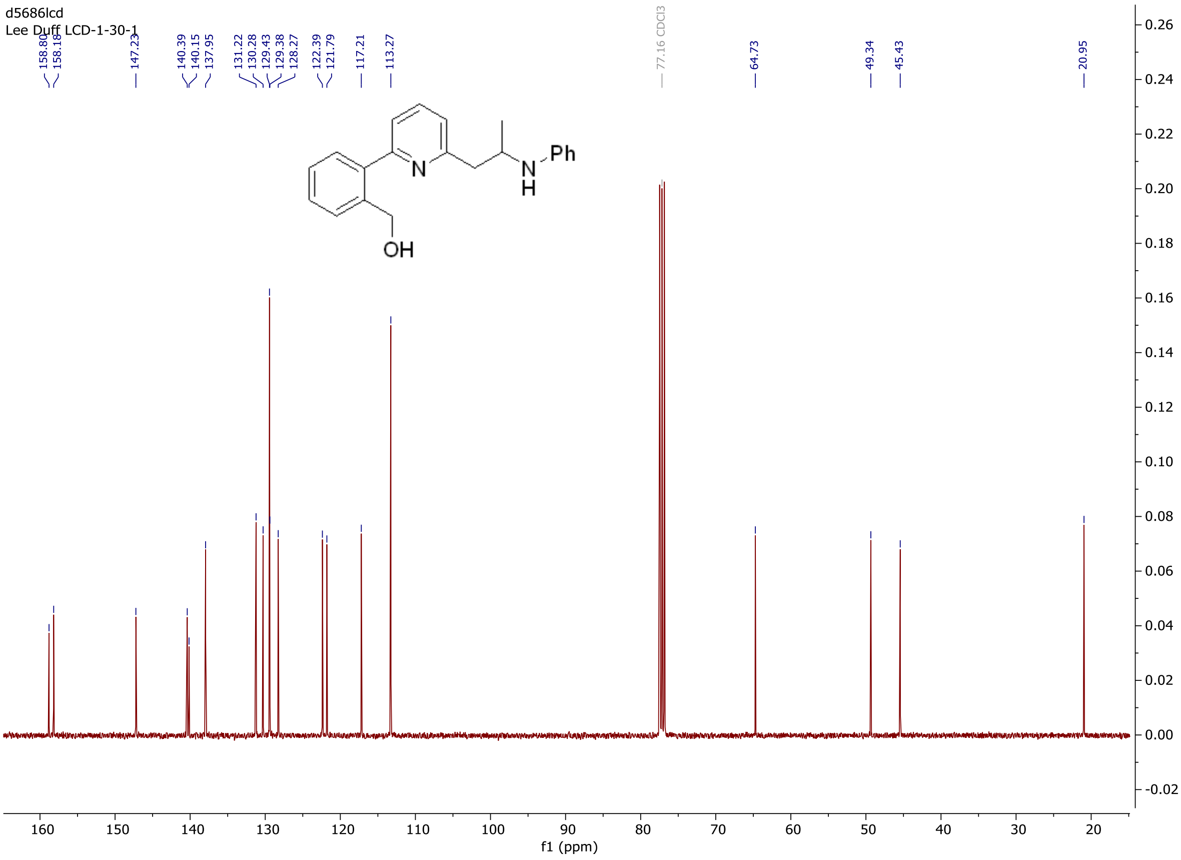

Macrocycle (87)

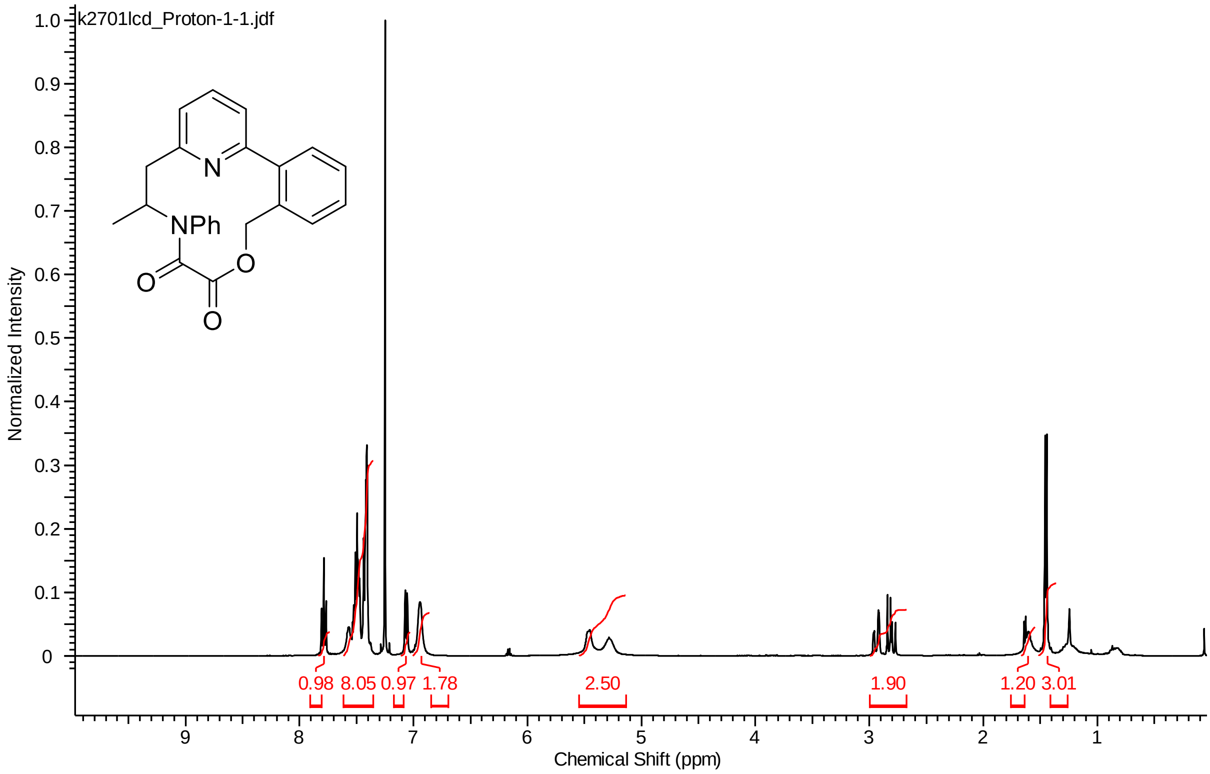

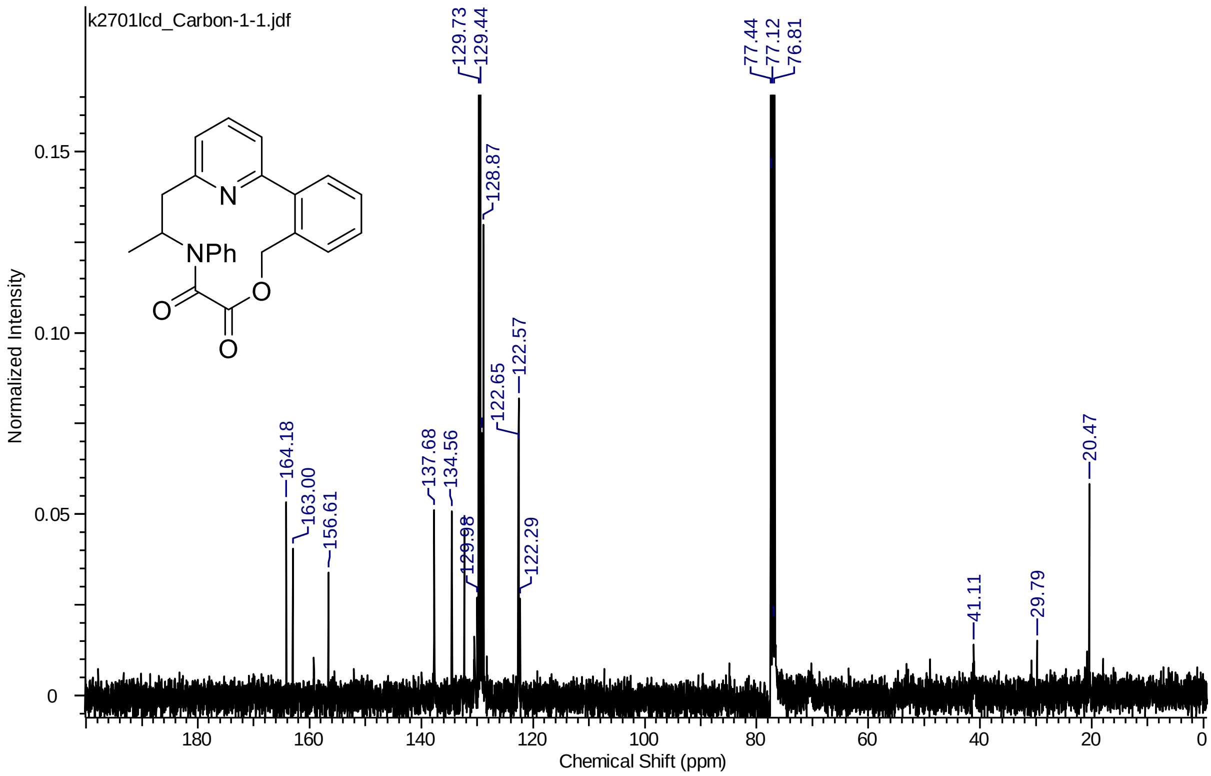

1-(6-(2-(Hydroxymethyl)phenyl)pyridin-2-yl)propan-2-one (S128)

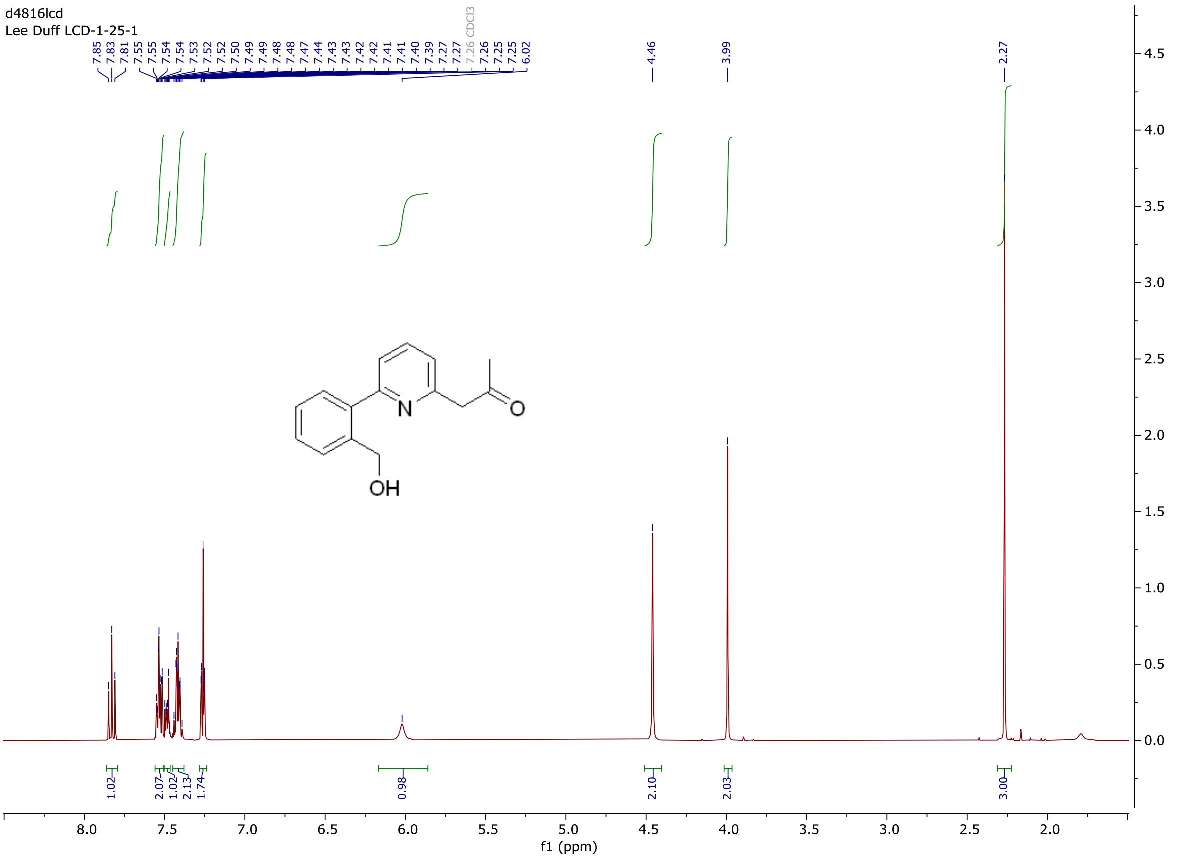

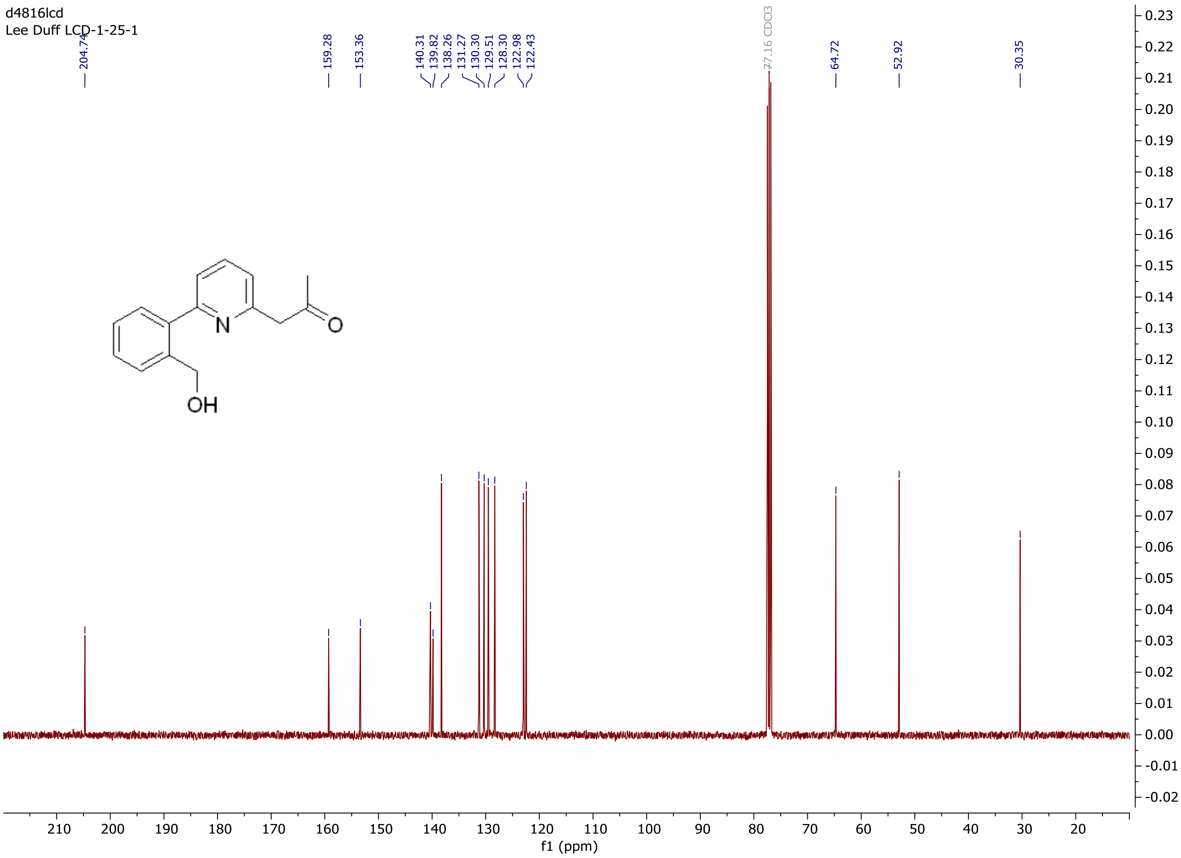

1-(6-(2-(Hydroxymethyl)phenyl)pyridin-2-yl)propan-2-ol (S129)

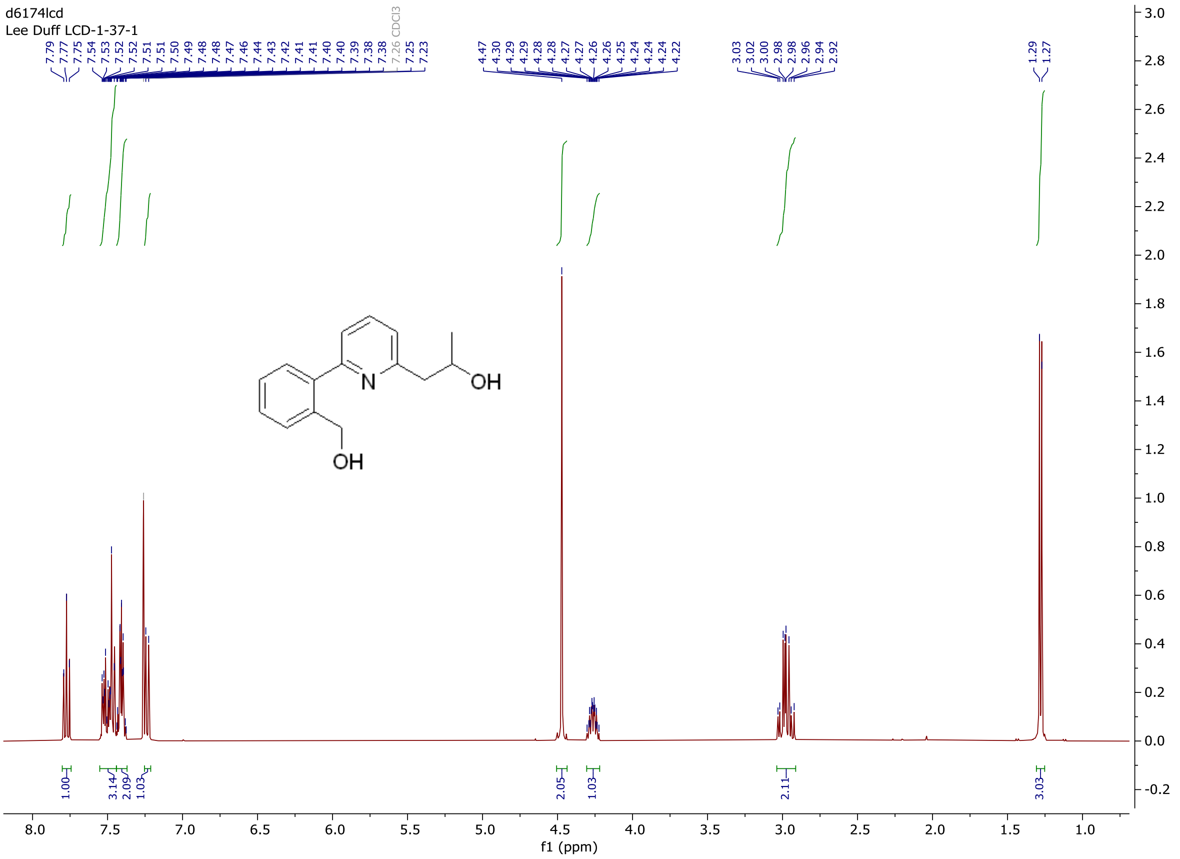

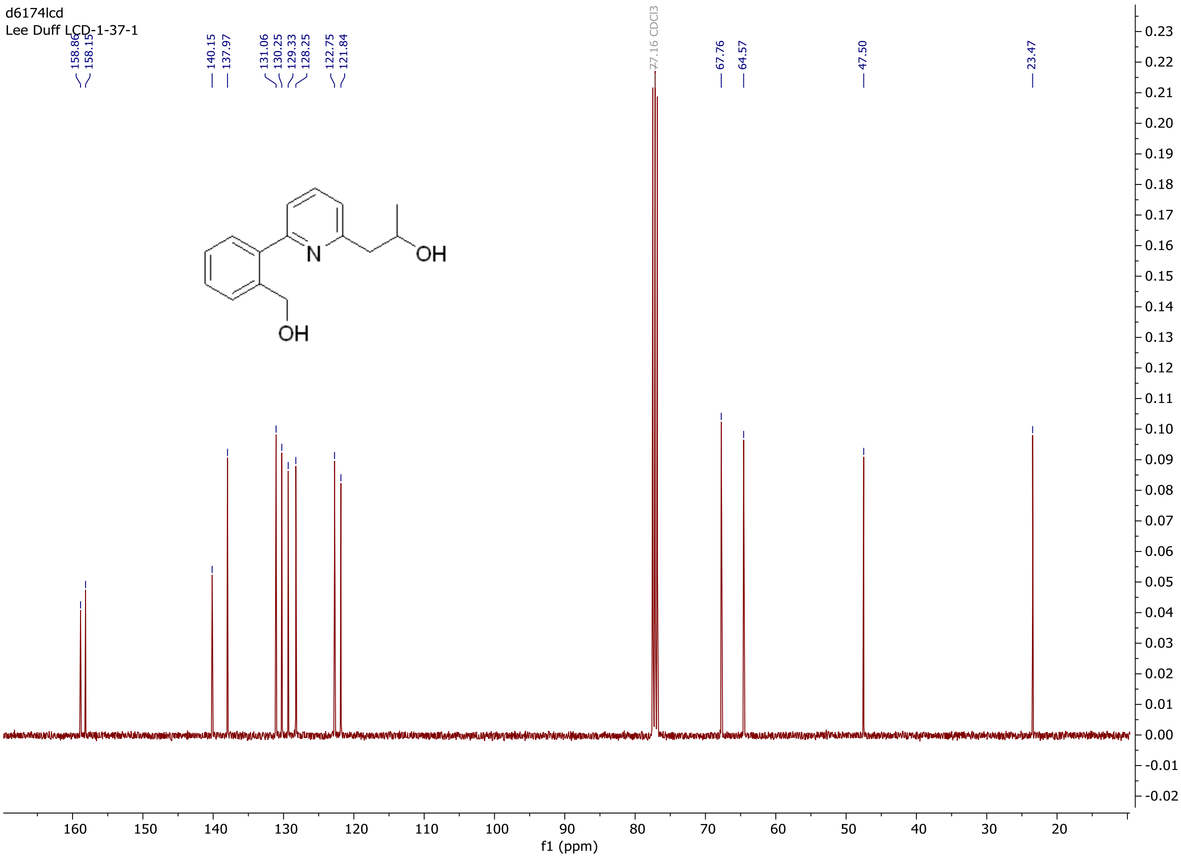

5-Methyl-5,6-dihydro-1*H*-7,11-(azeno)benzo[*e*][1,3]dioxa[2]thiacyclotridecine 3-oxide (88)

5:1 mixture of diastereosiomers

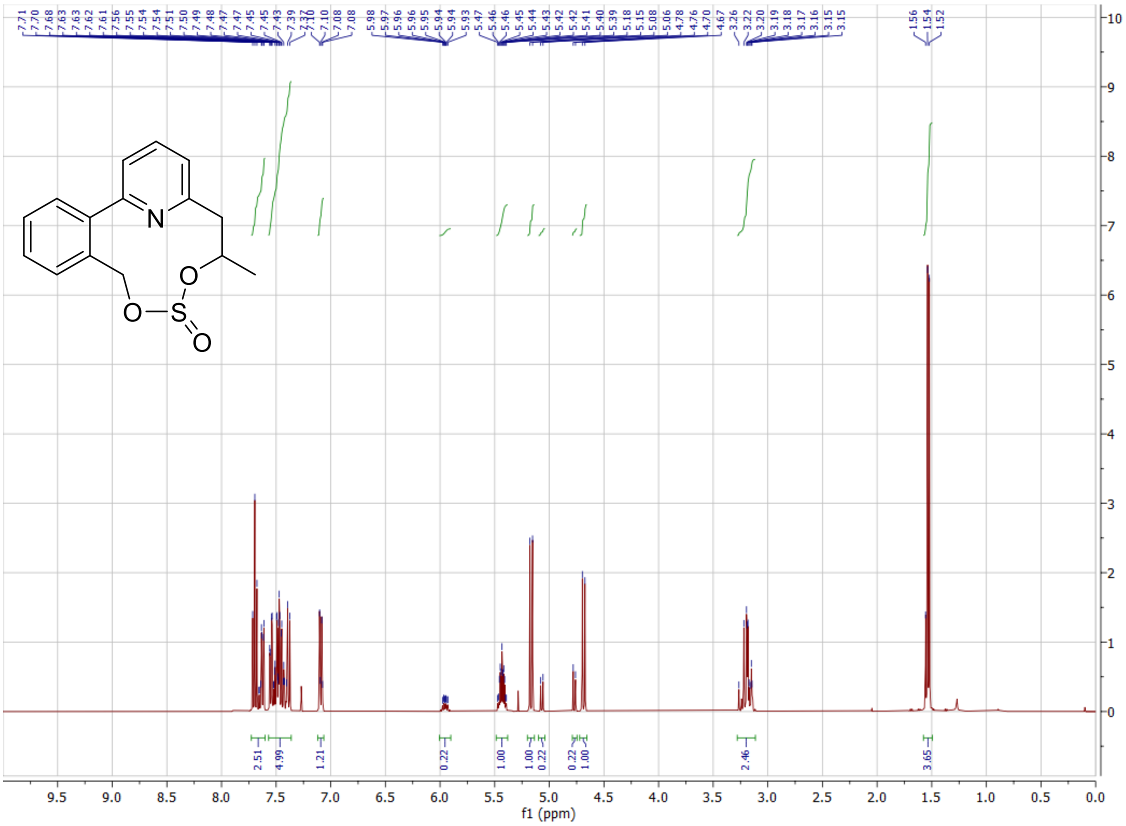

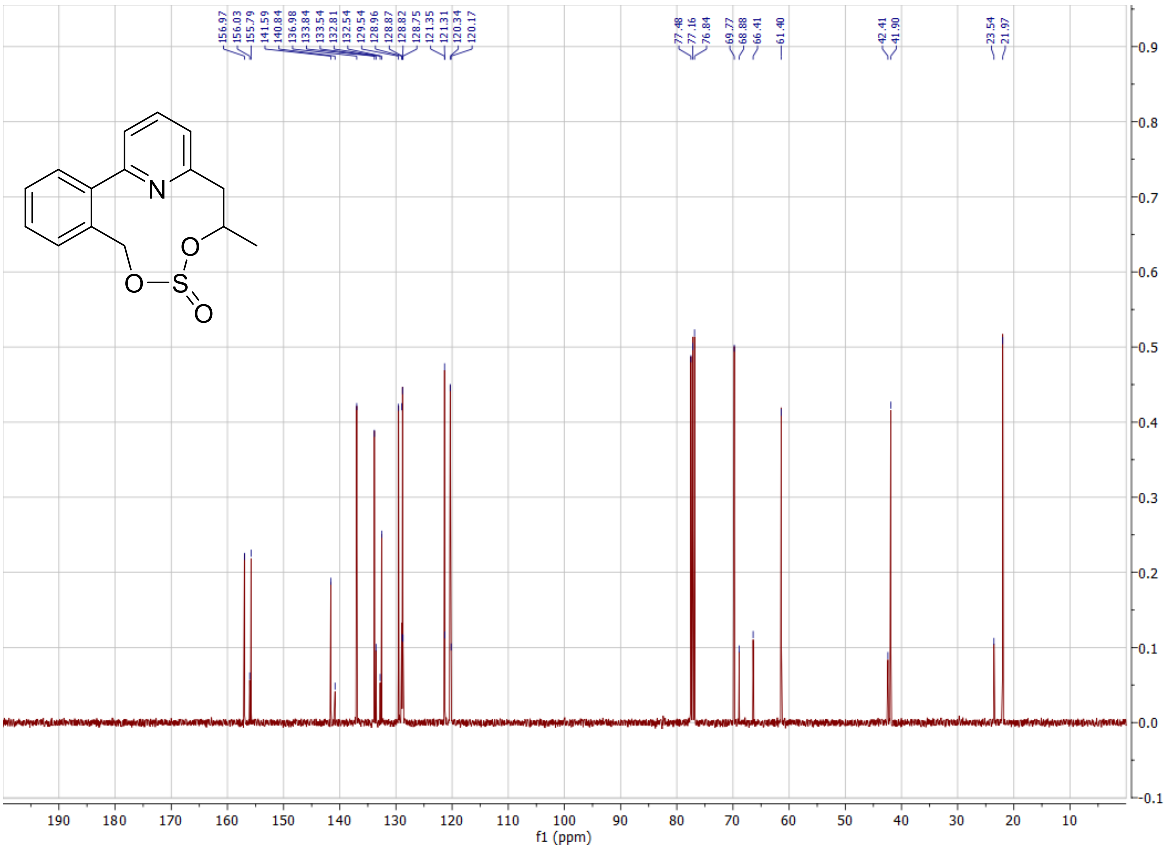

3,3'-(Phenylazanediyl)bis(propan-1-ol) (S130)

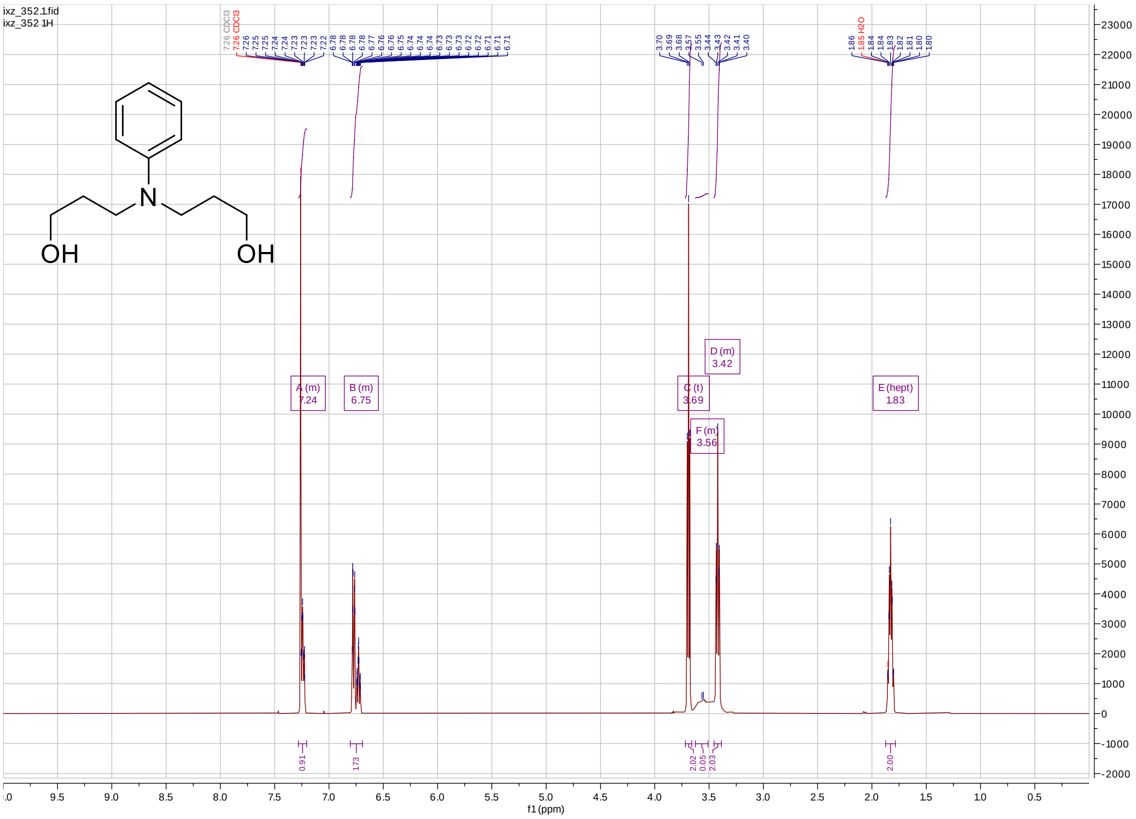

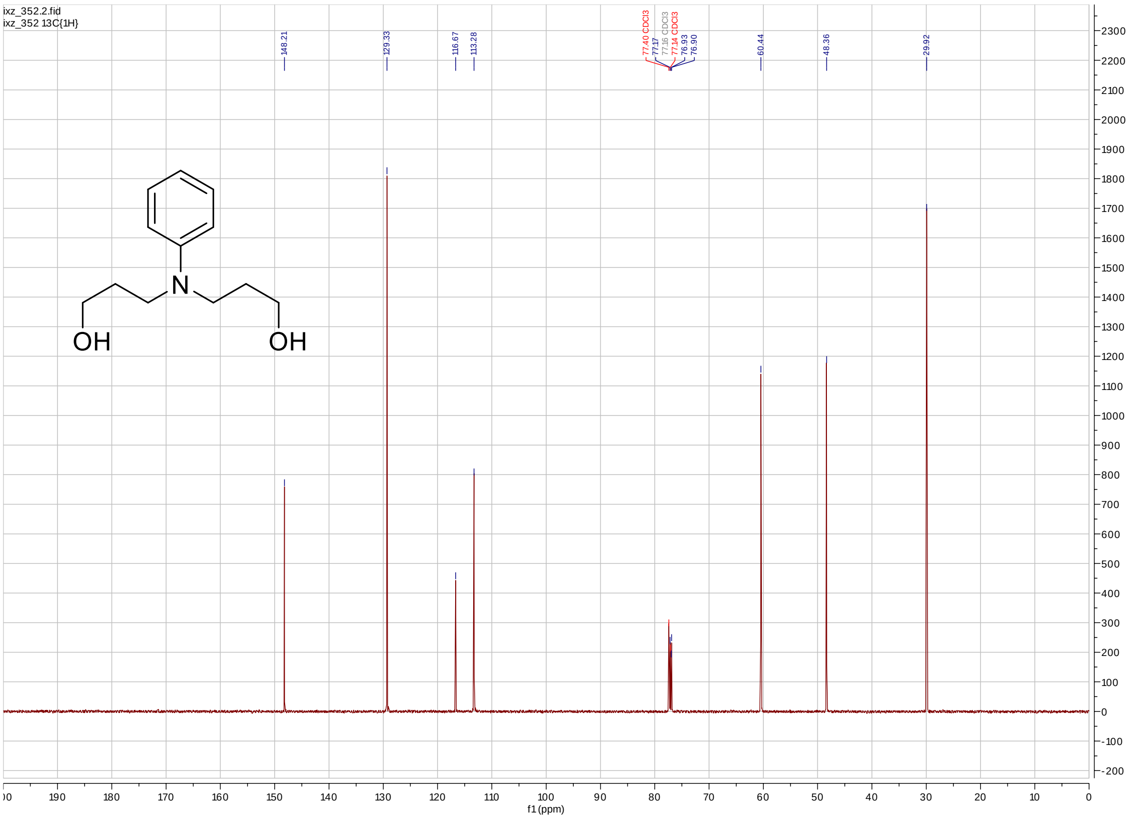

7-Phenyl-1,3,2,7-dioxathiazecane 2-oxide (89)

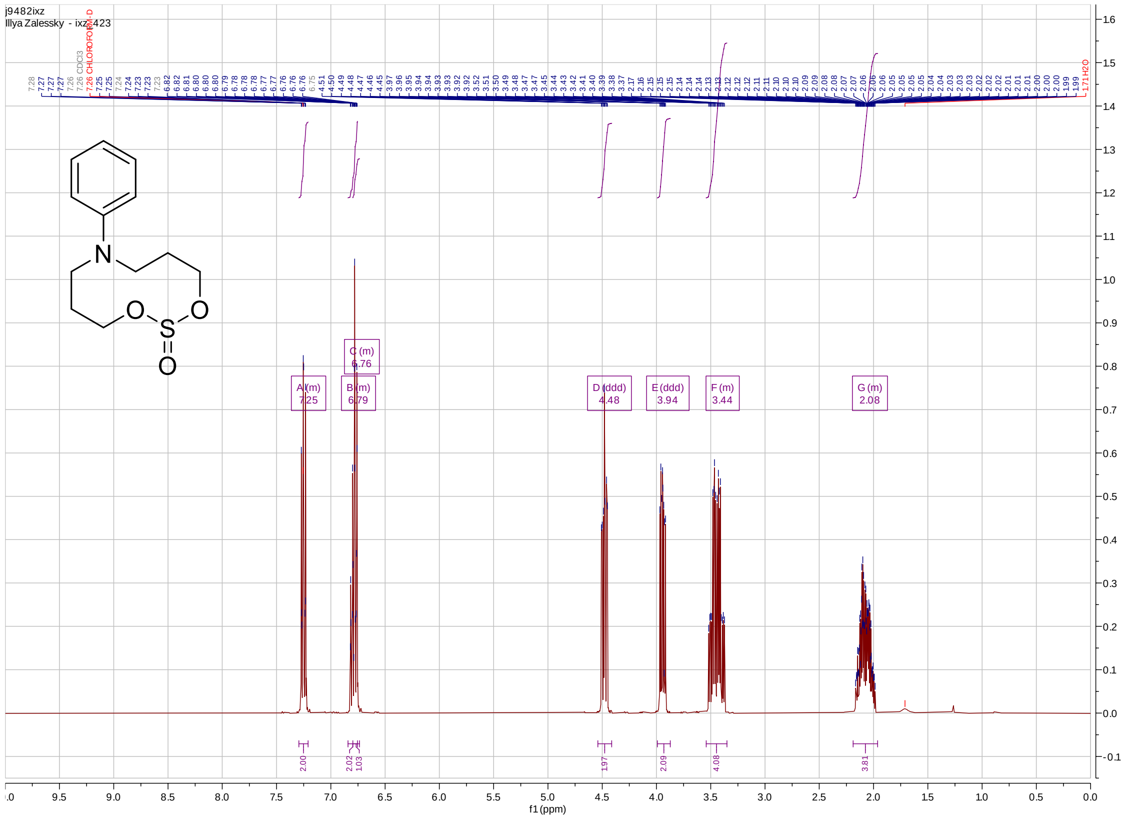

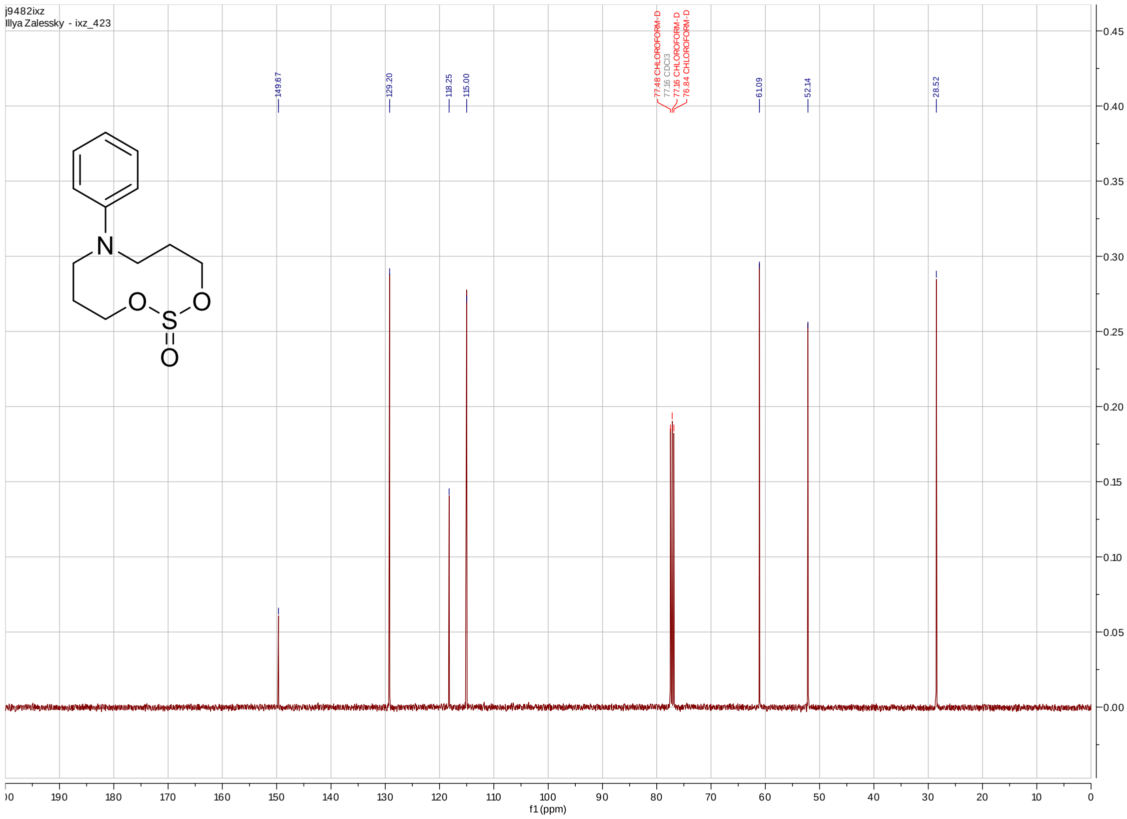

3-(Ethyl(3-(ethylamino) propyl)amino)propan-1-ol (S131)

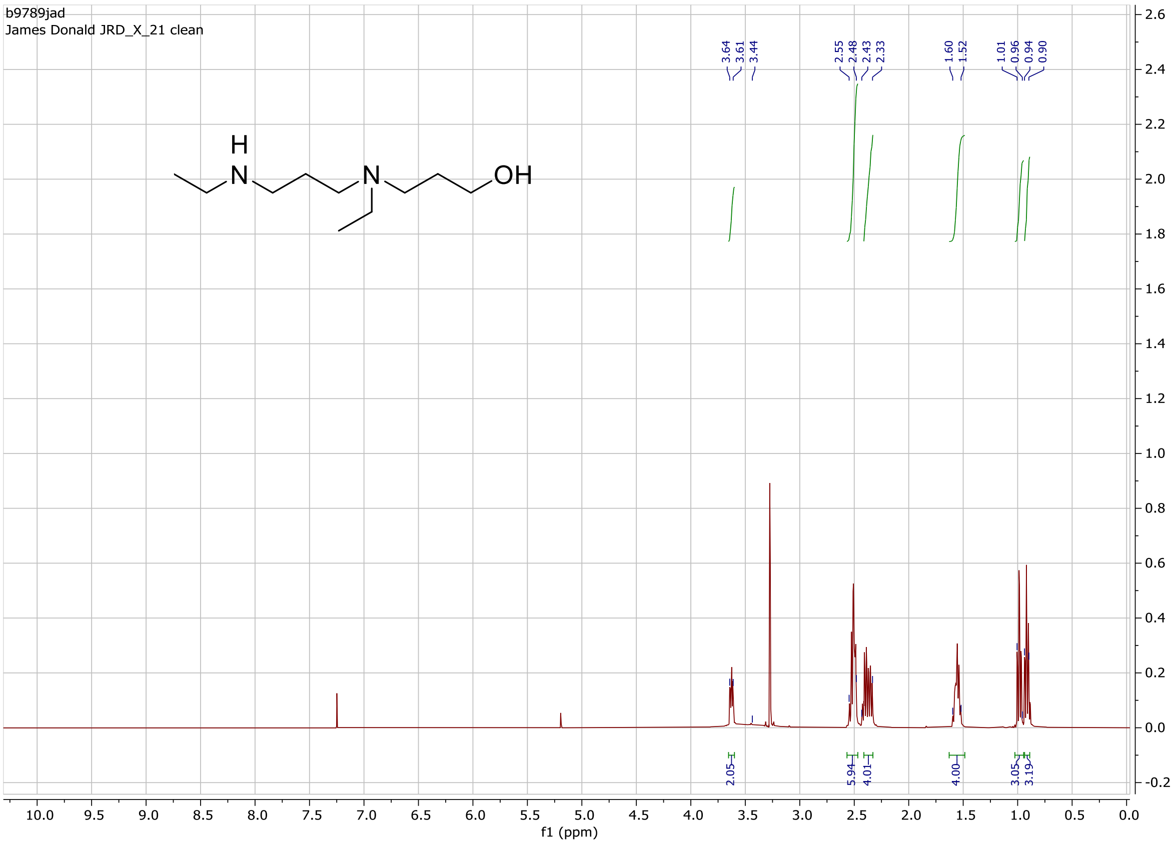

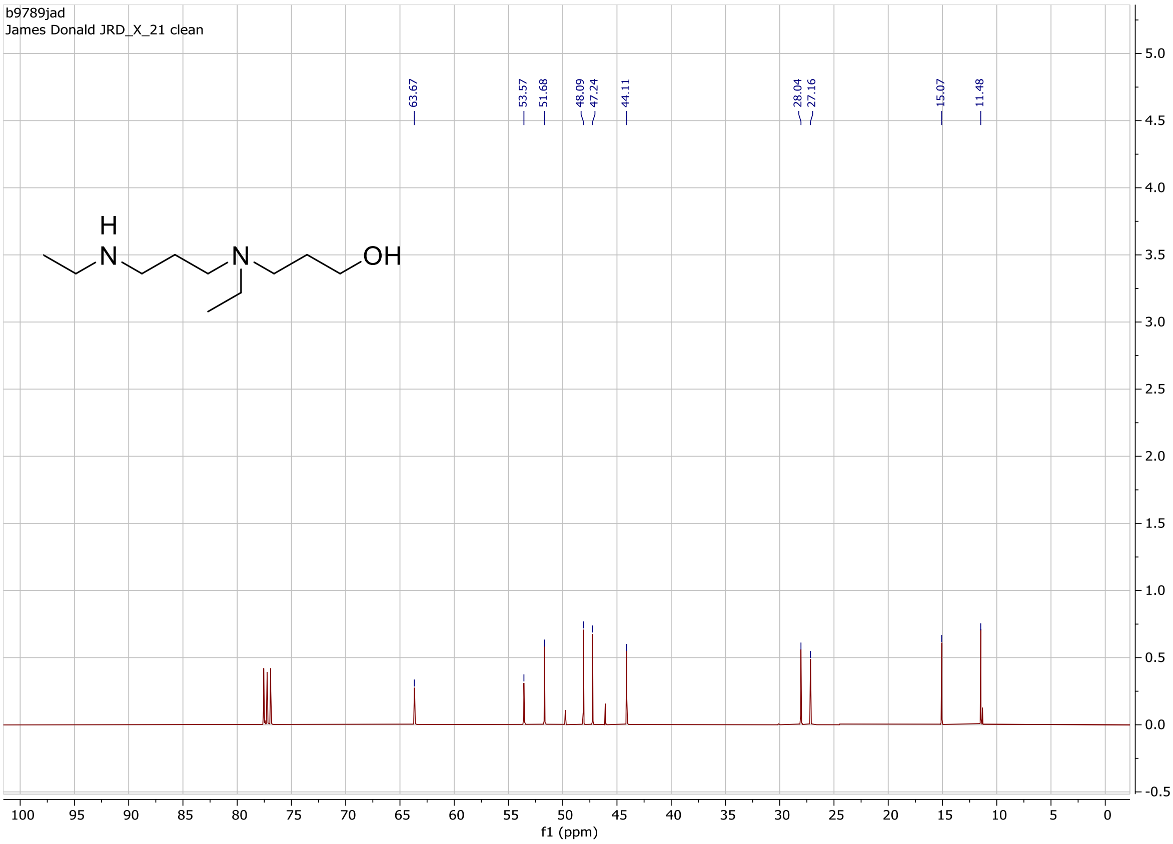

3,7-Diethyl-1,3,7-oxadiazecan-2-one (90)

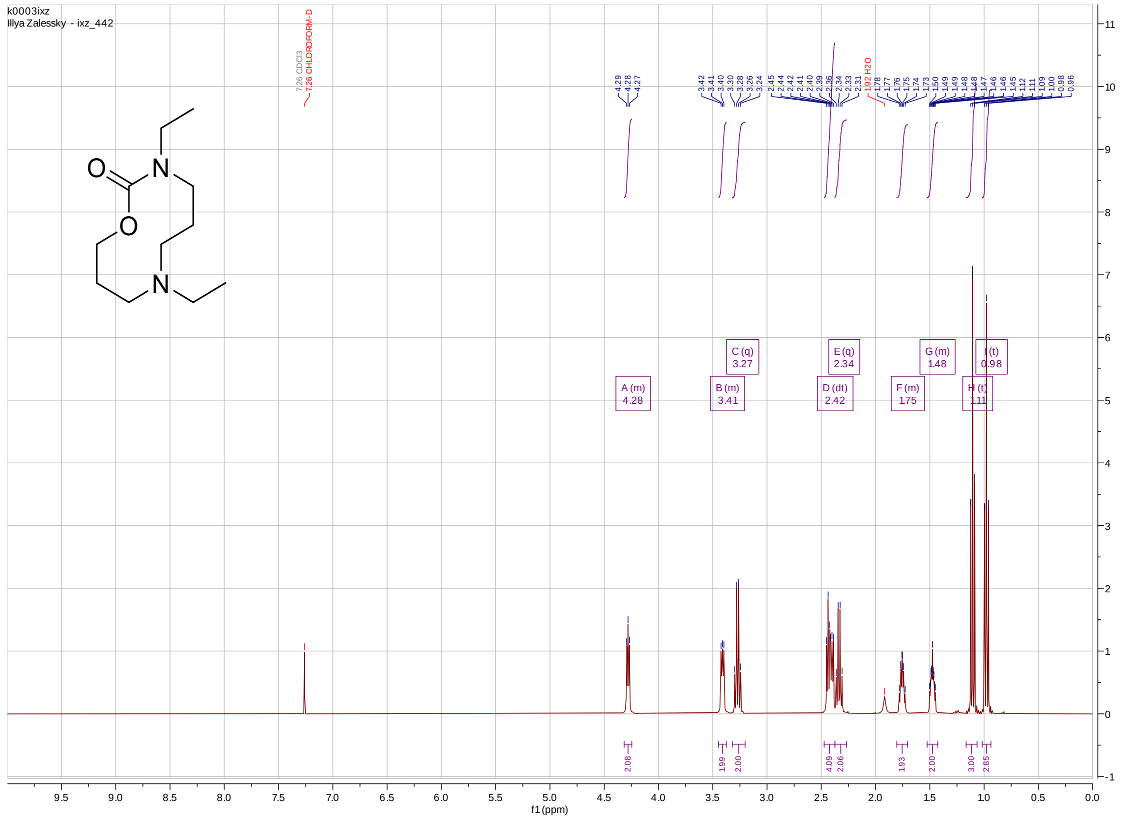

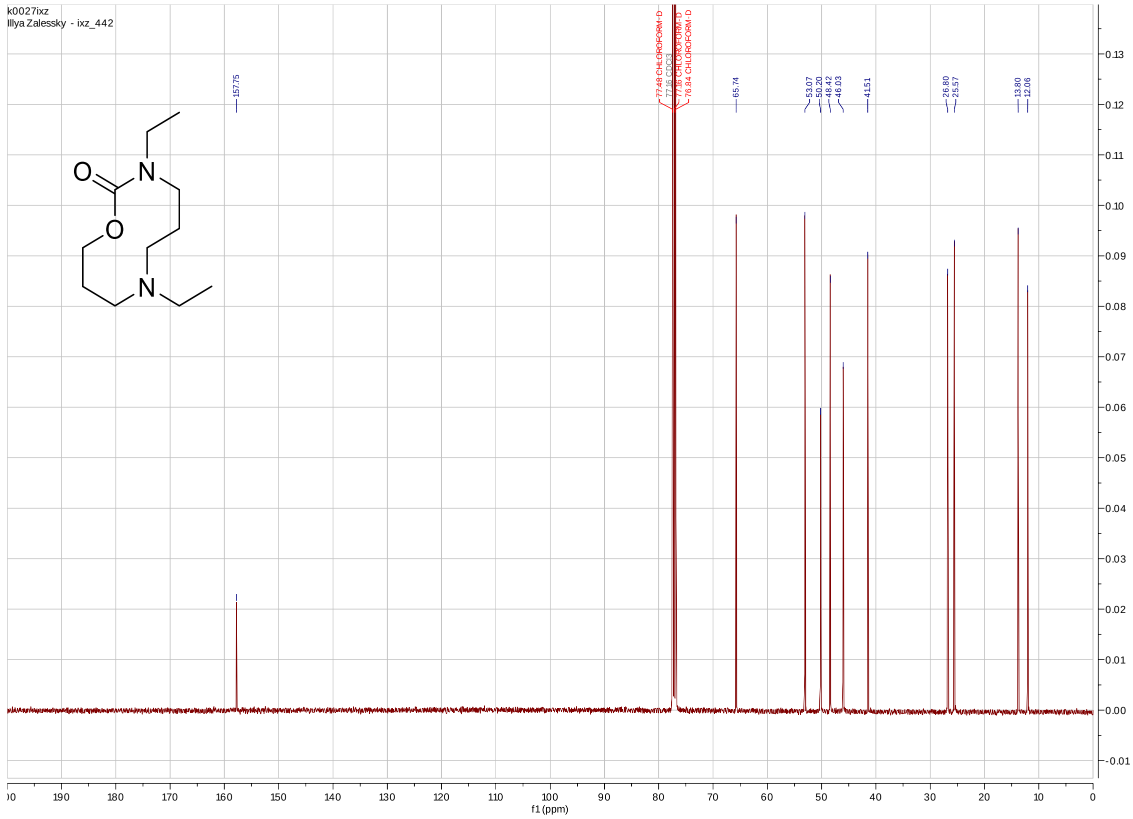

1,7-Dimethyl-4,5,6,7-tetrahydrobenzo[d][1,3,6]oxadiazonin-2(1H)-one (91)

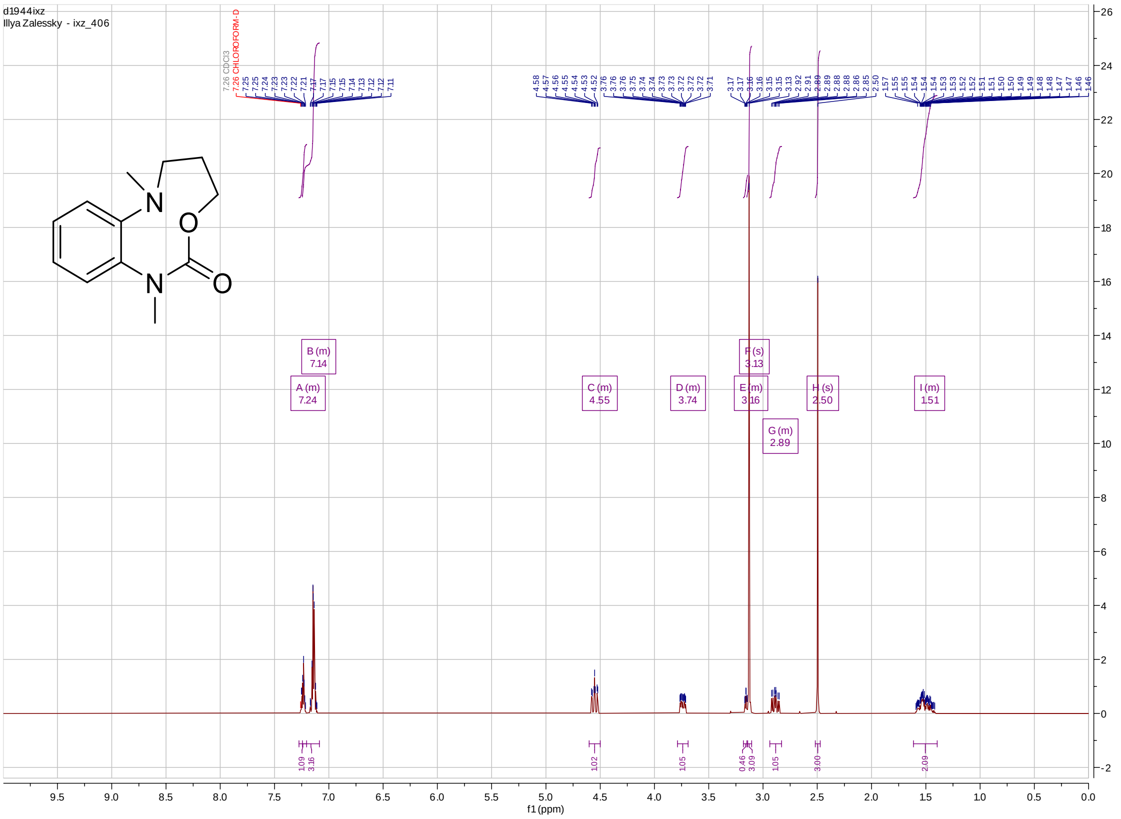

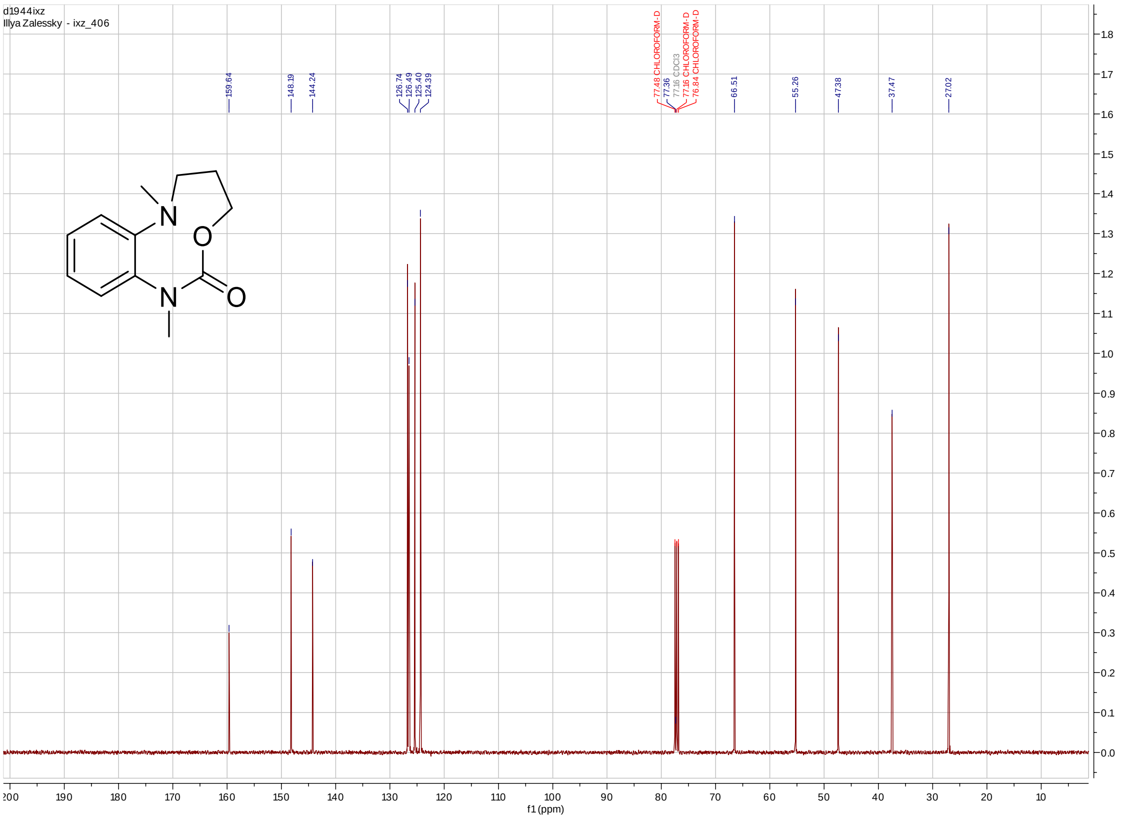

3-(Phenylamino)propan-1-ol (S132)

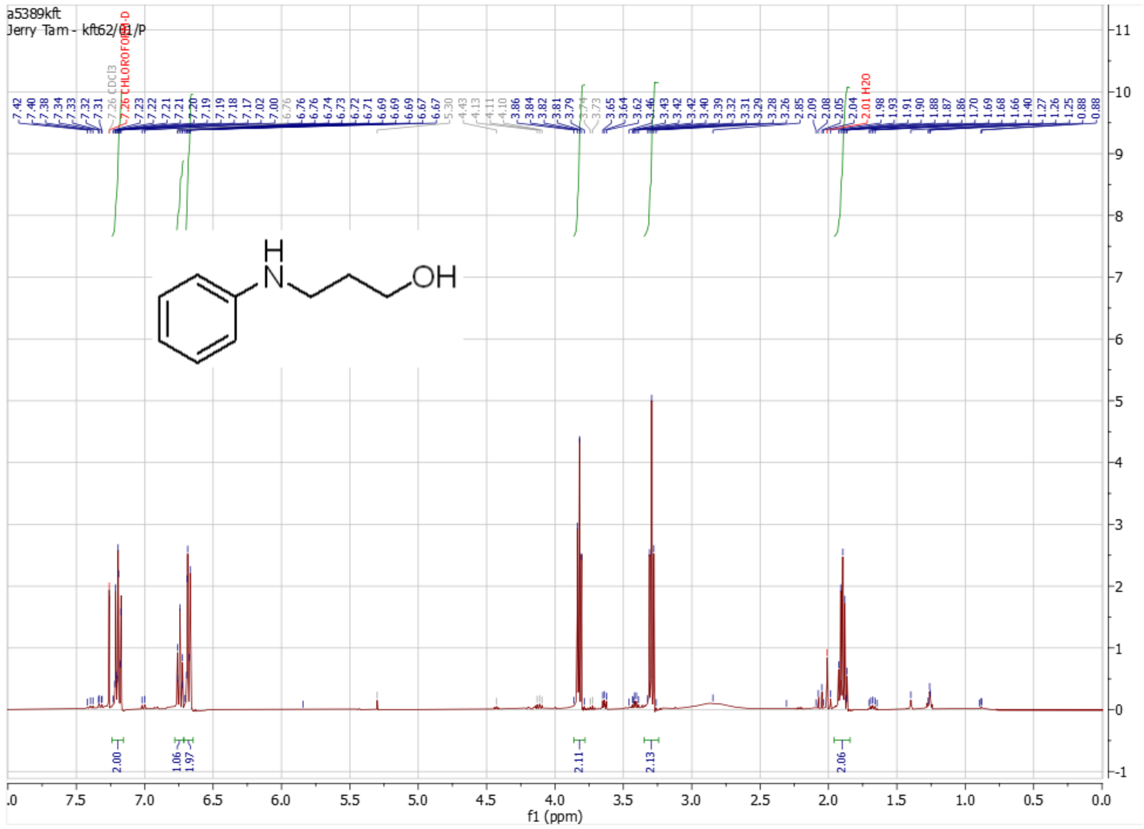

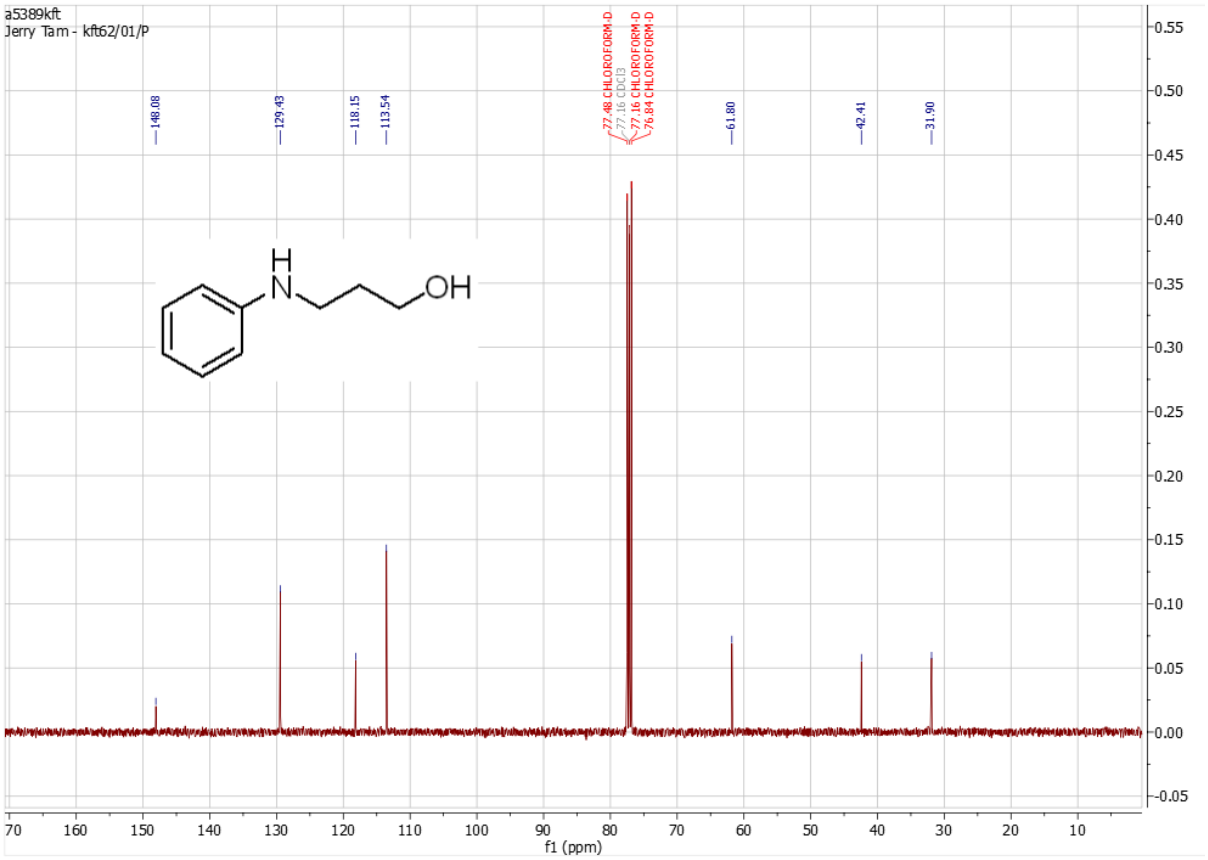

tert-Butyl (3-hydroxypropyl)(phenyl)carbamate (S133)

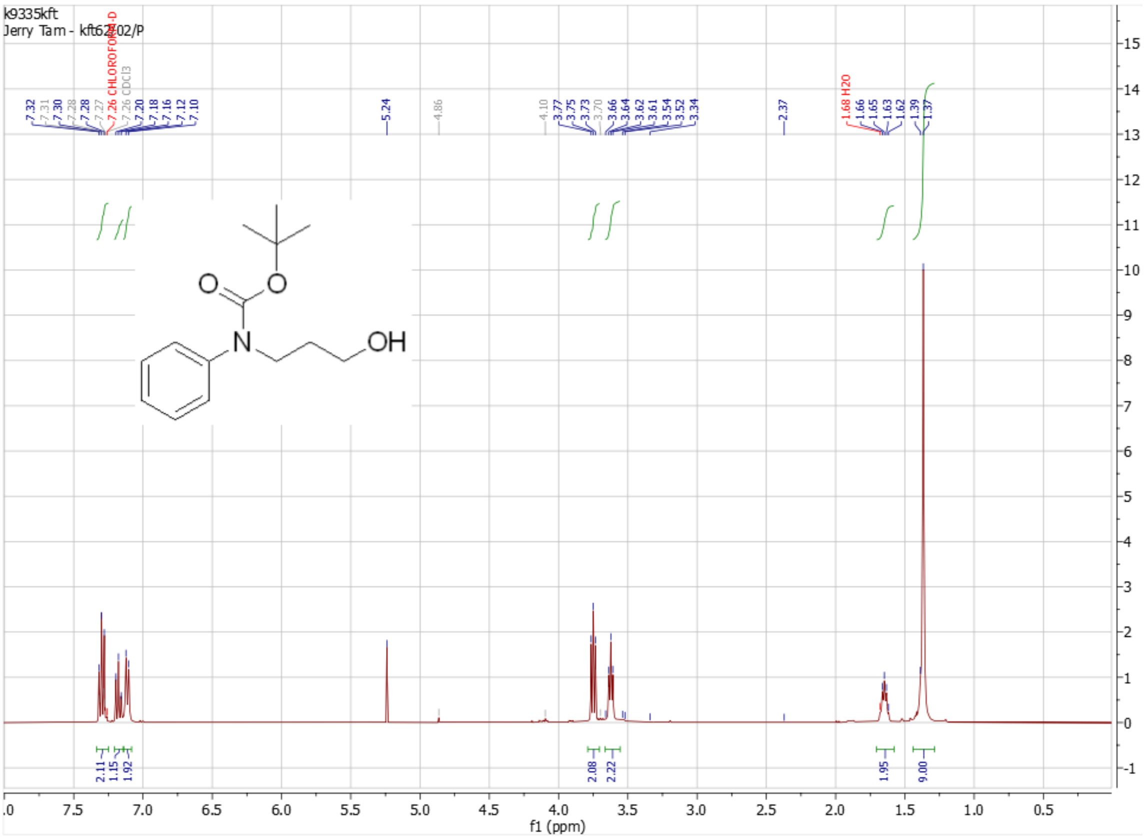

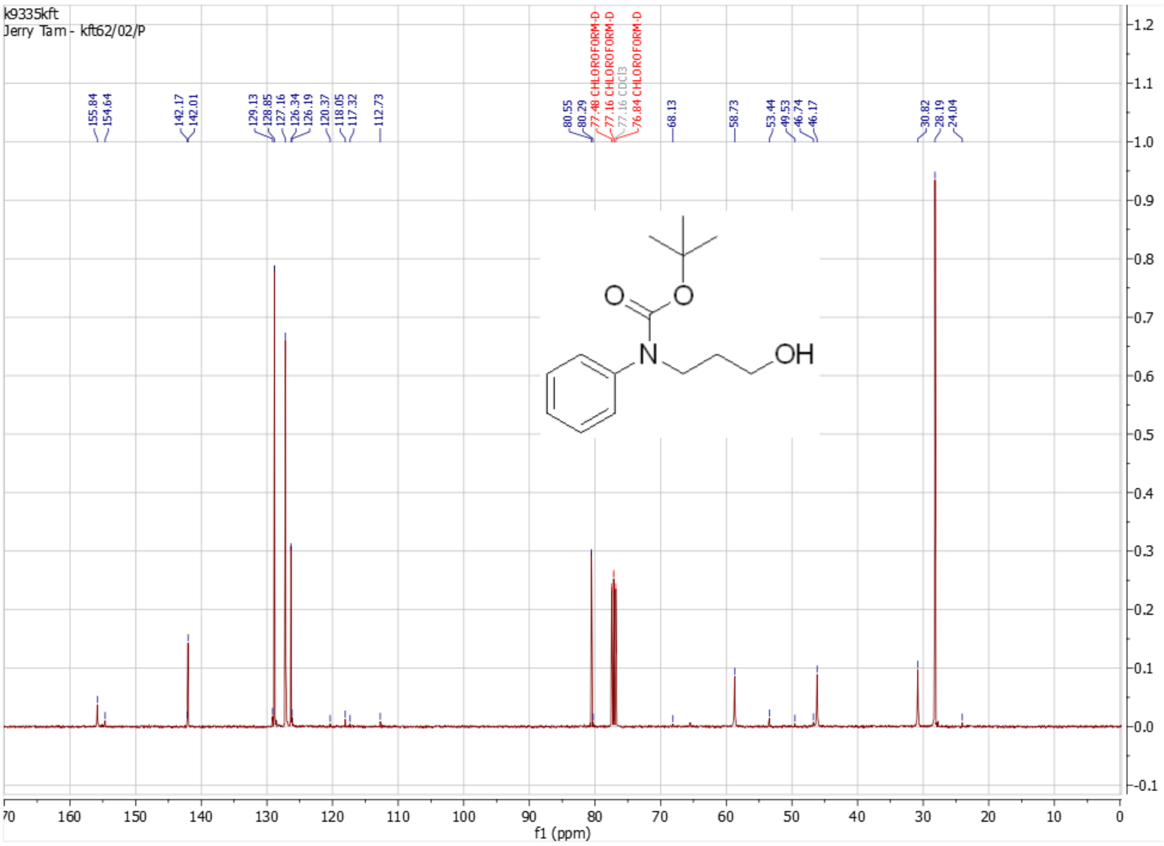

tert-Butyl (3-oxopropyl)(phenyl)carbamate (S134)

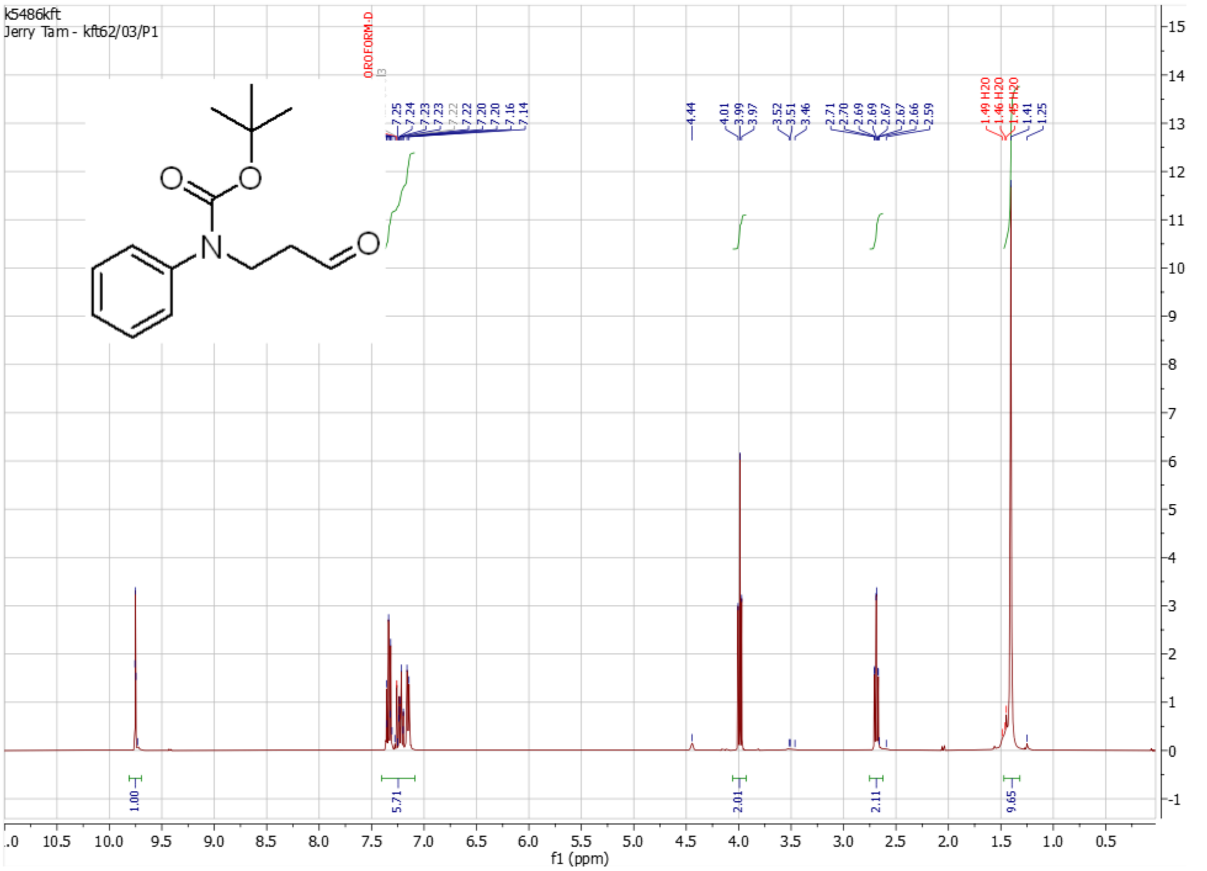

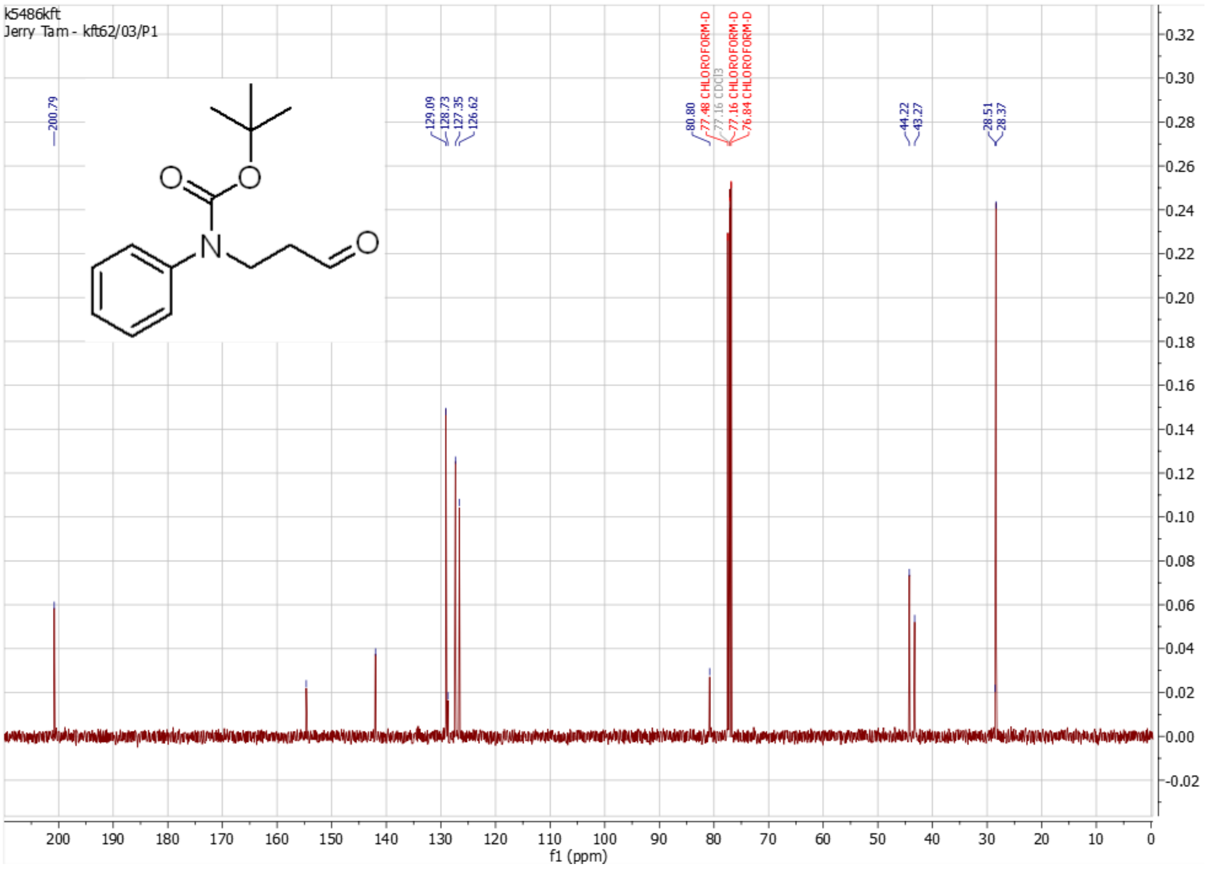

***tert*-Butyl (3-(benzyl(3-hydroxypropyl)amino)propyl)(phenyl)carbamate (S135)**

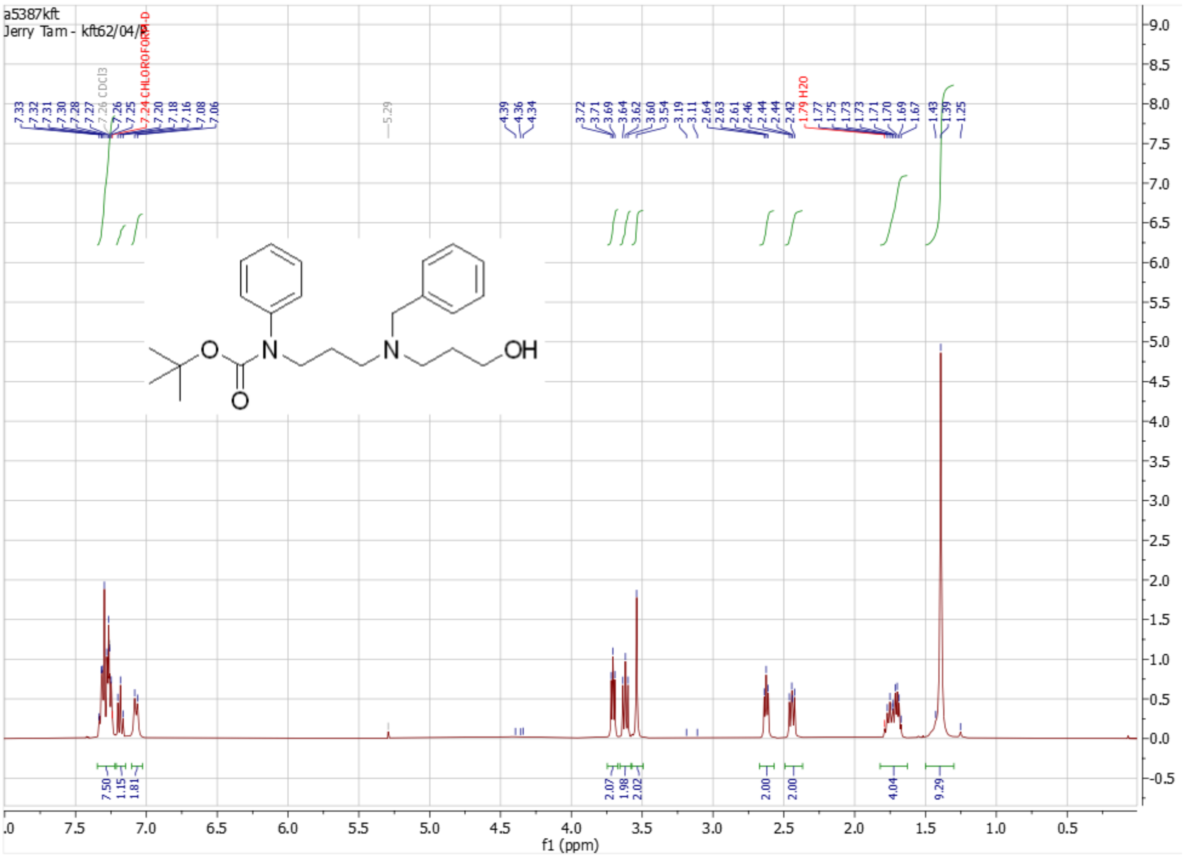

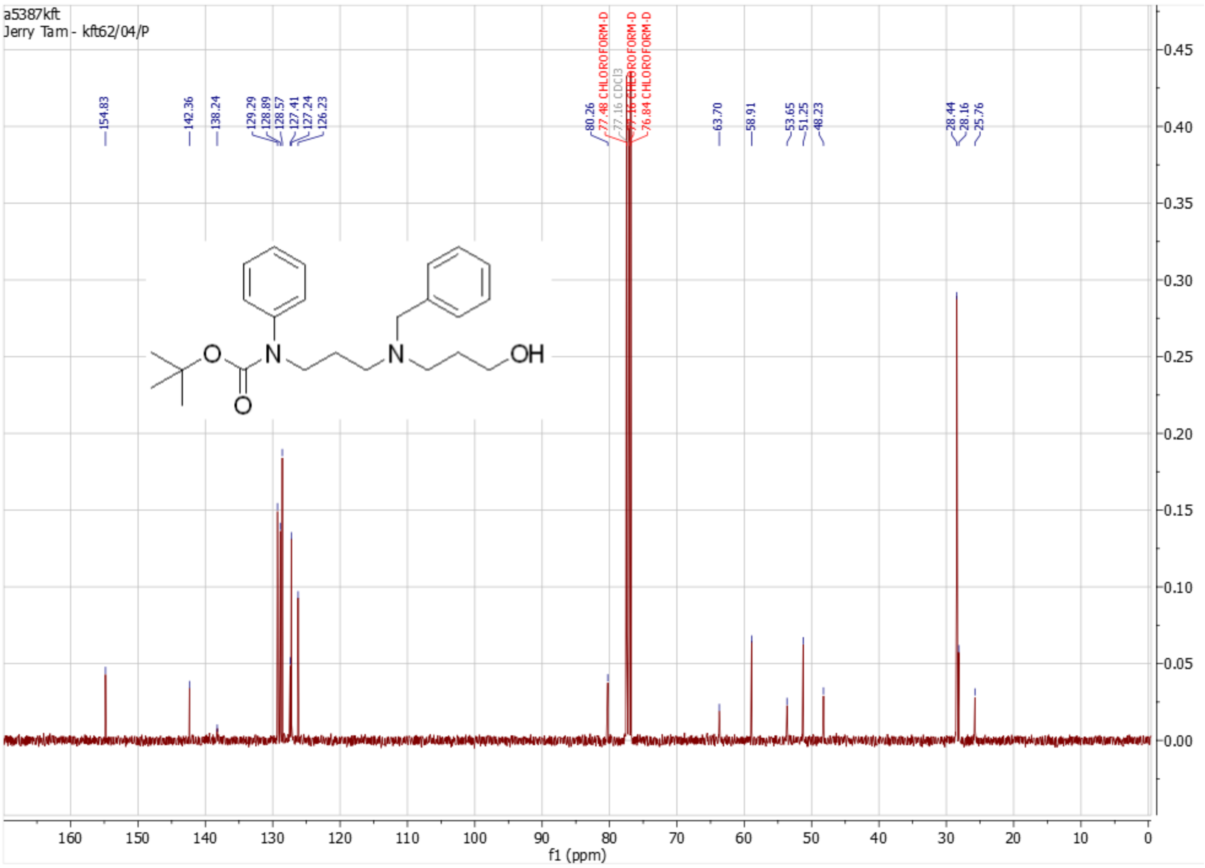

3-(Benzyl(3-(phenylamino)propyl)amino)propan-1-ol (S136)

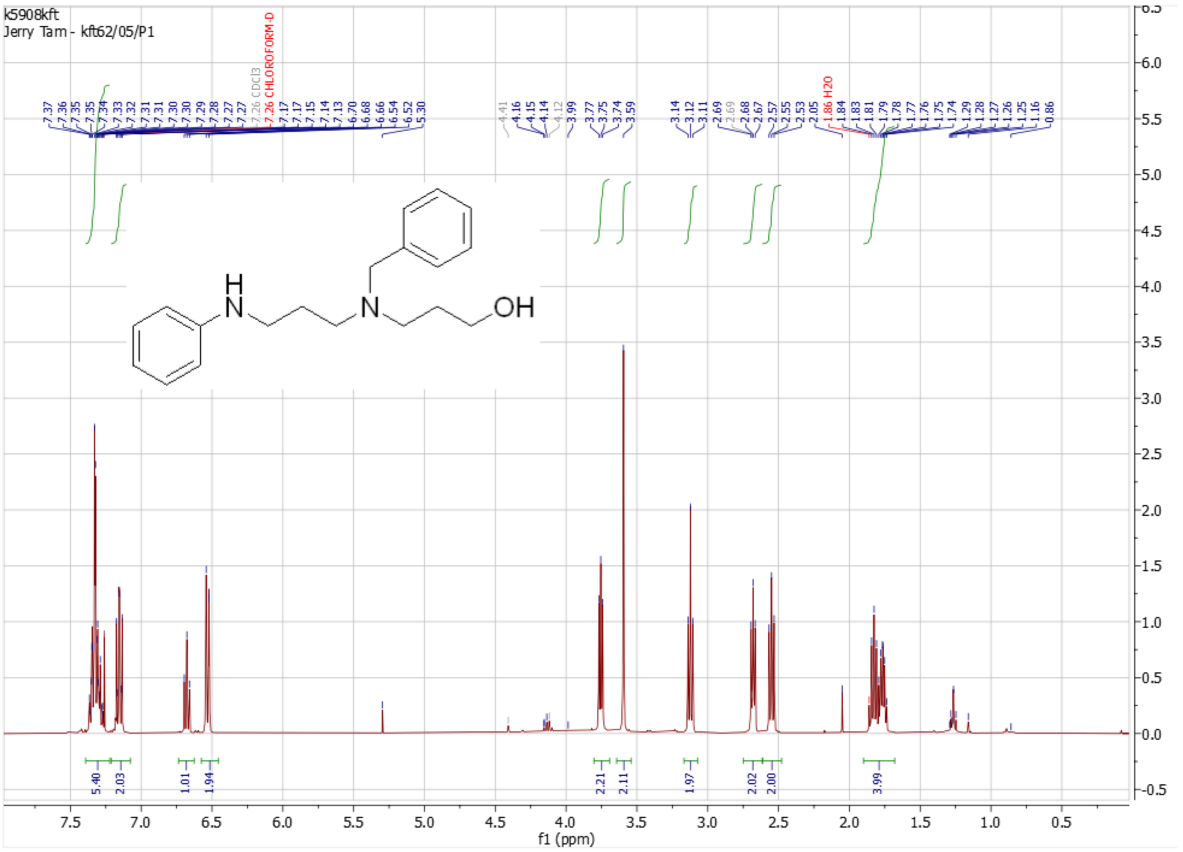

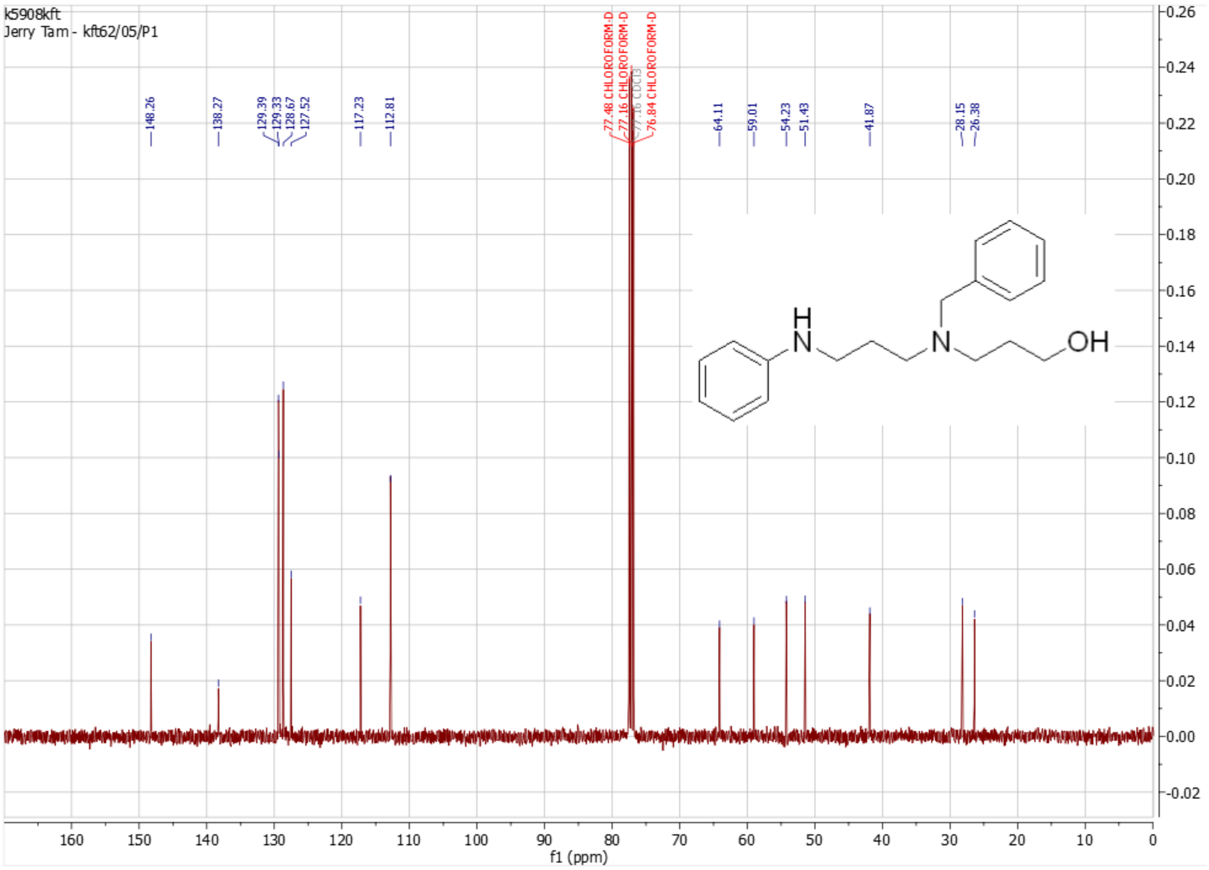

7-Benzyl-3-phenyl-1,3,7-oxadiazecane-2-thione (92)

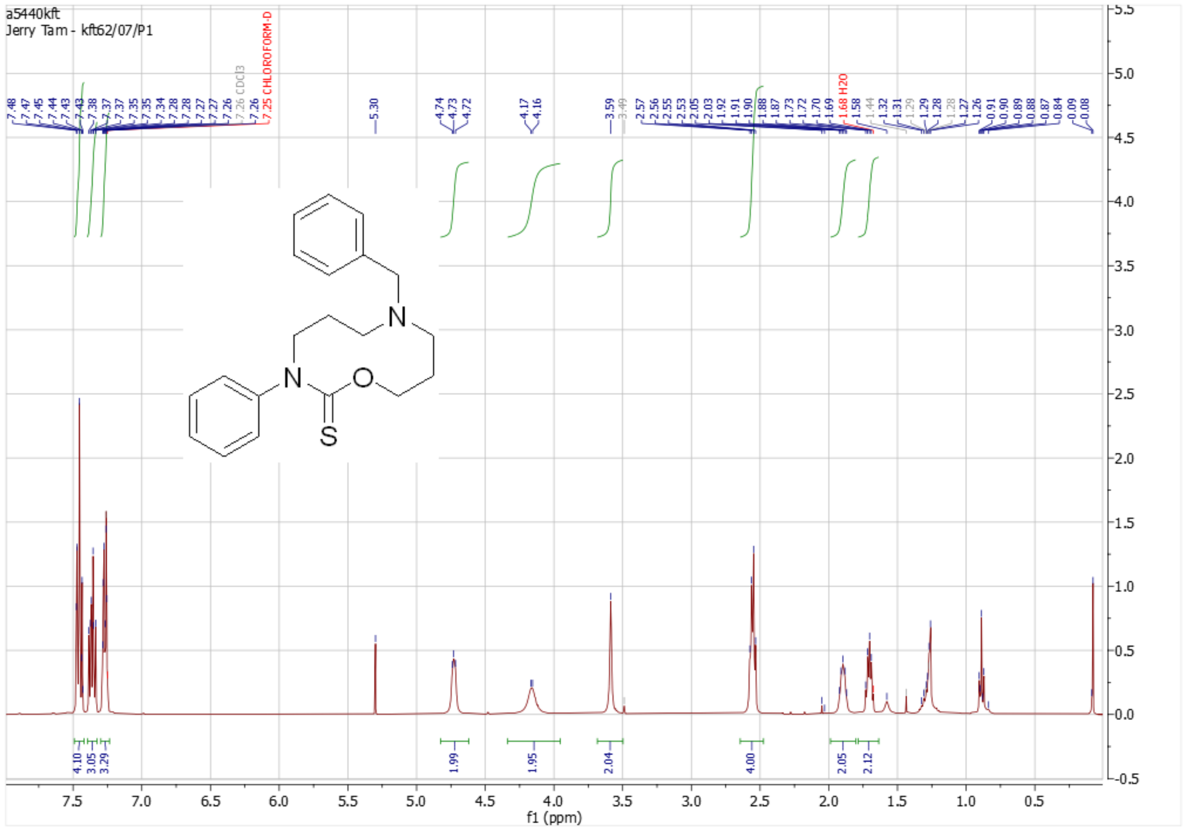

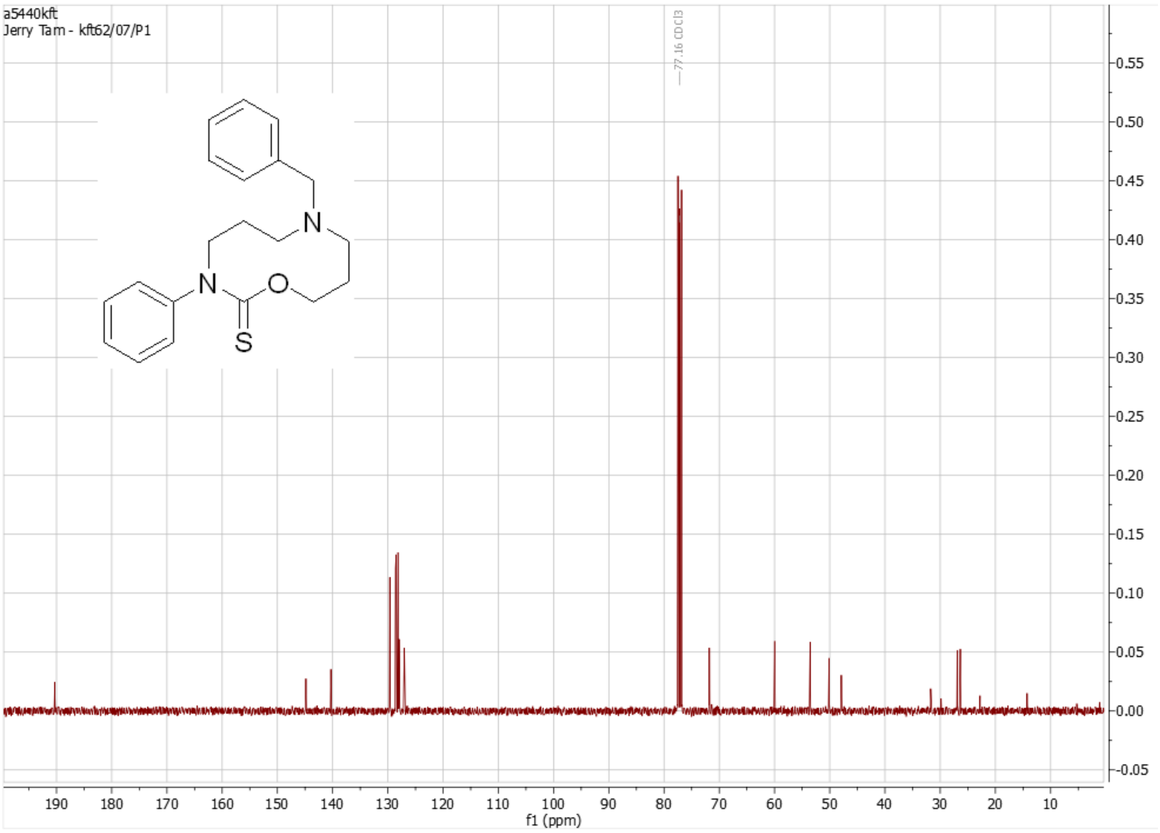

2-(Phenyl(2-(phenylamino)ethyl)amino)ethan-1-ol (S137)

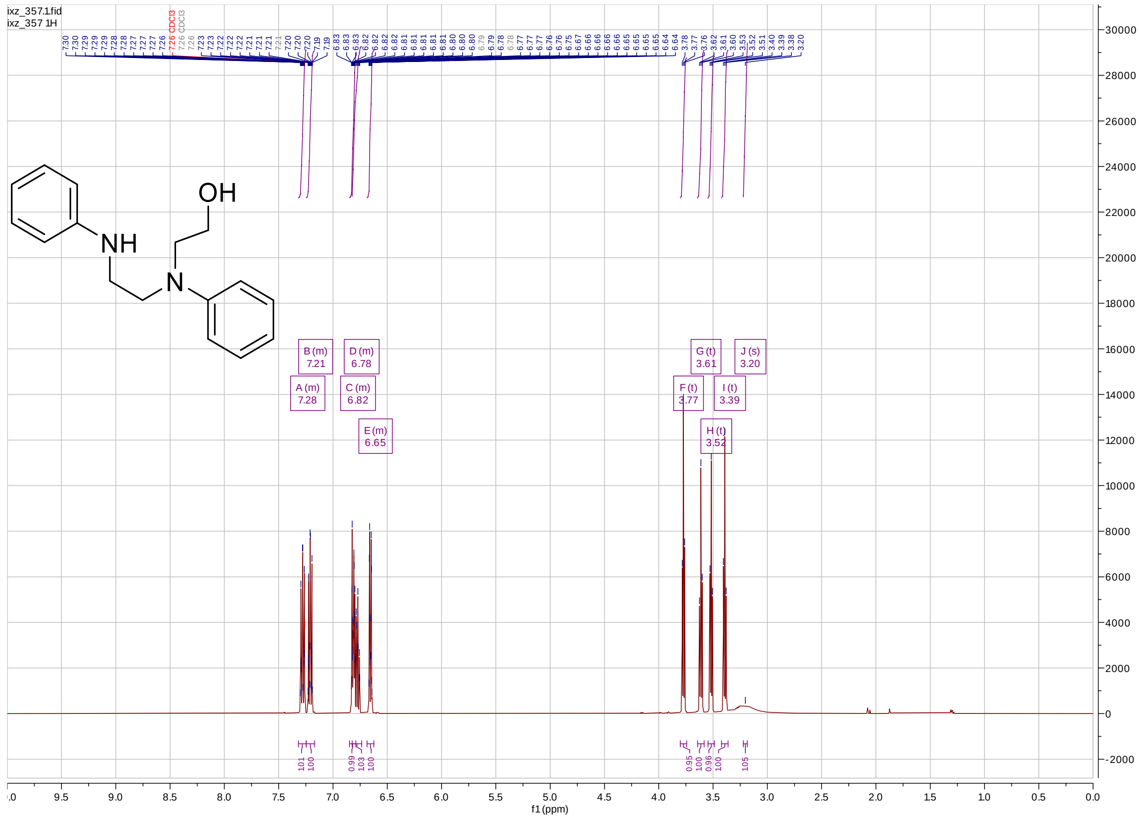

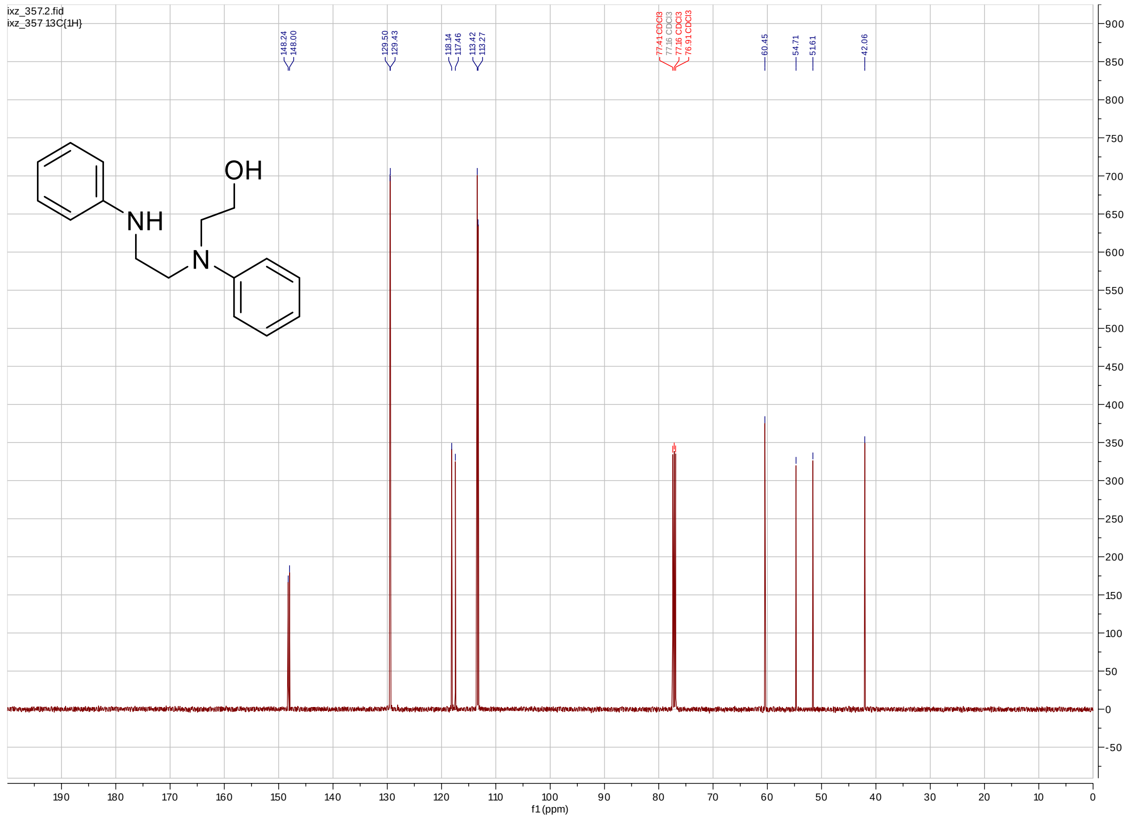

3,6-Diphenyl-1,3,6-oxadiazocane-2-thione (93)

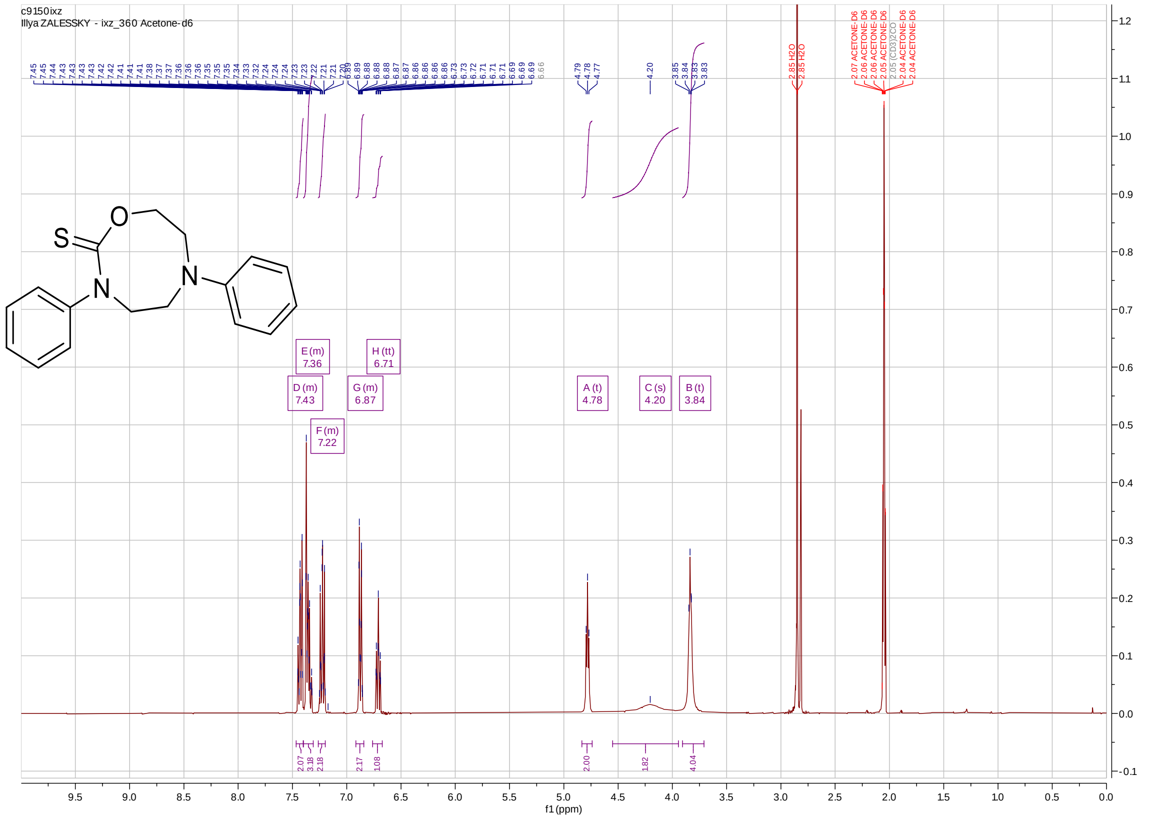

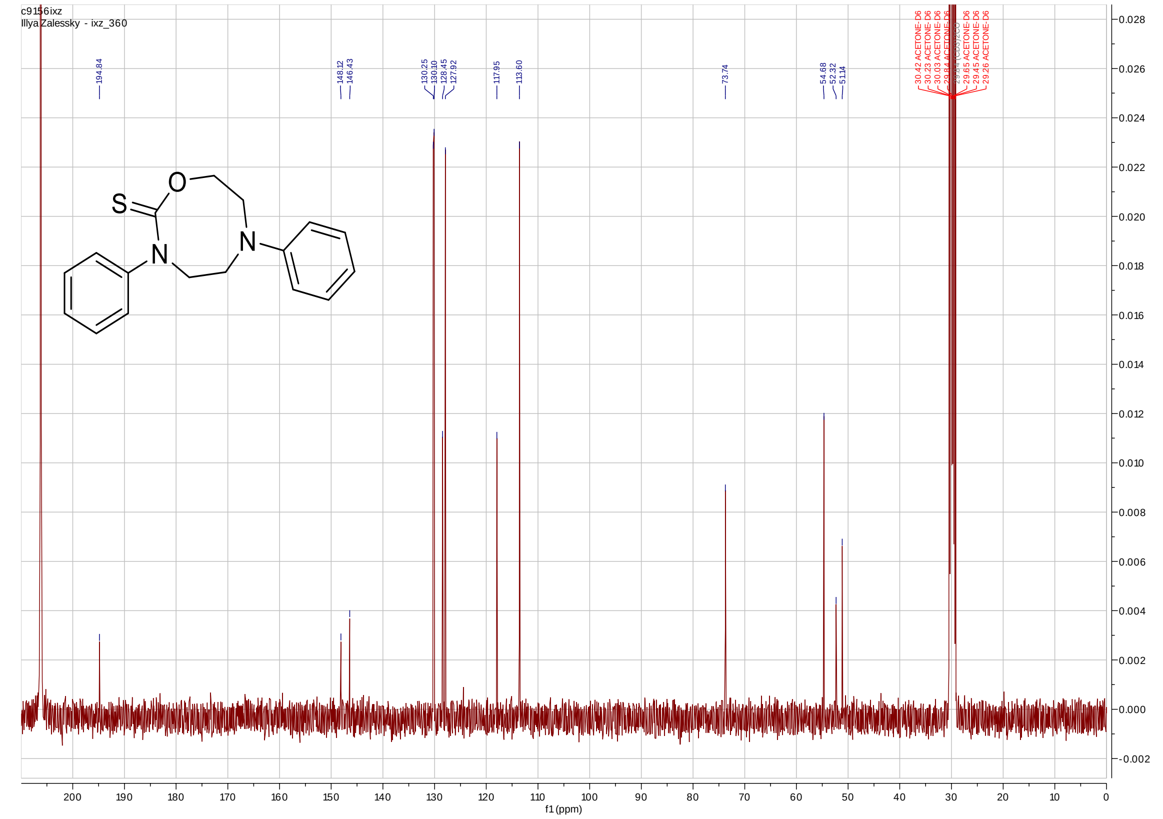

3-(Phenyl(3-(phenylamino)propyl)amino)propan-1-ol (S138)

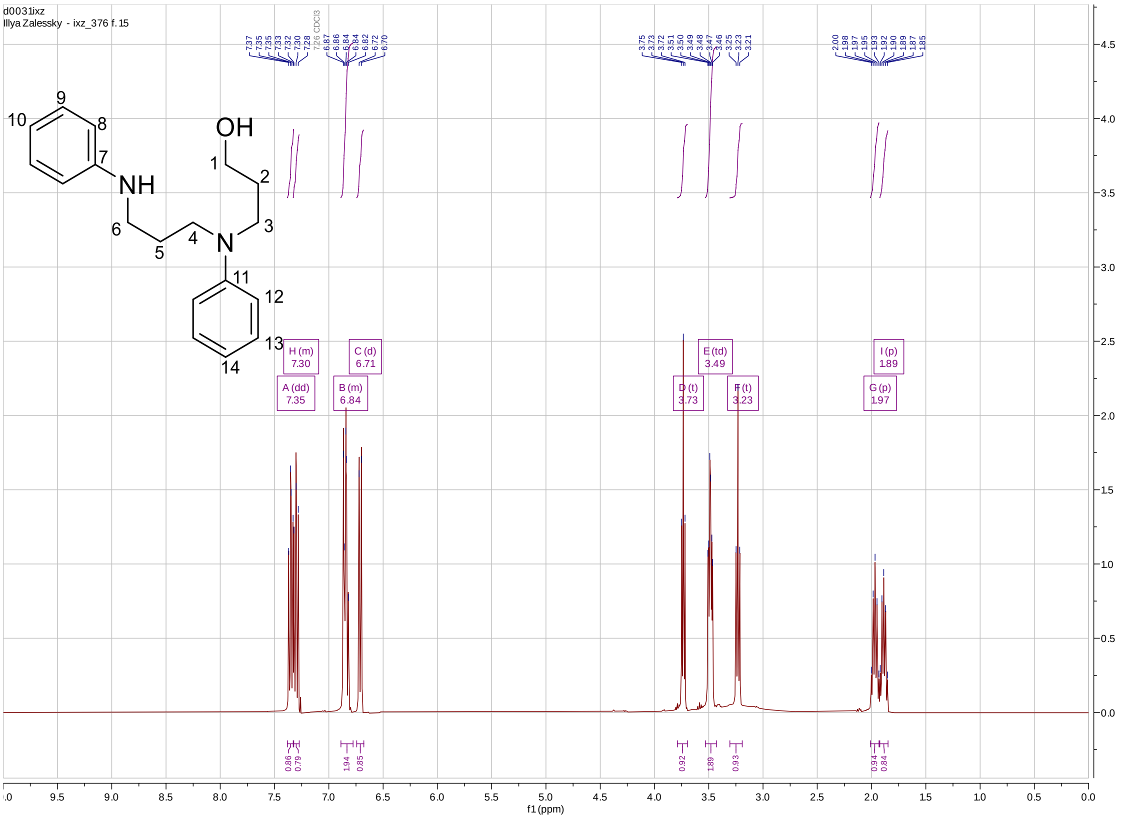

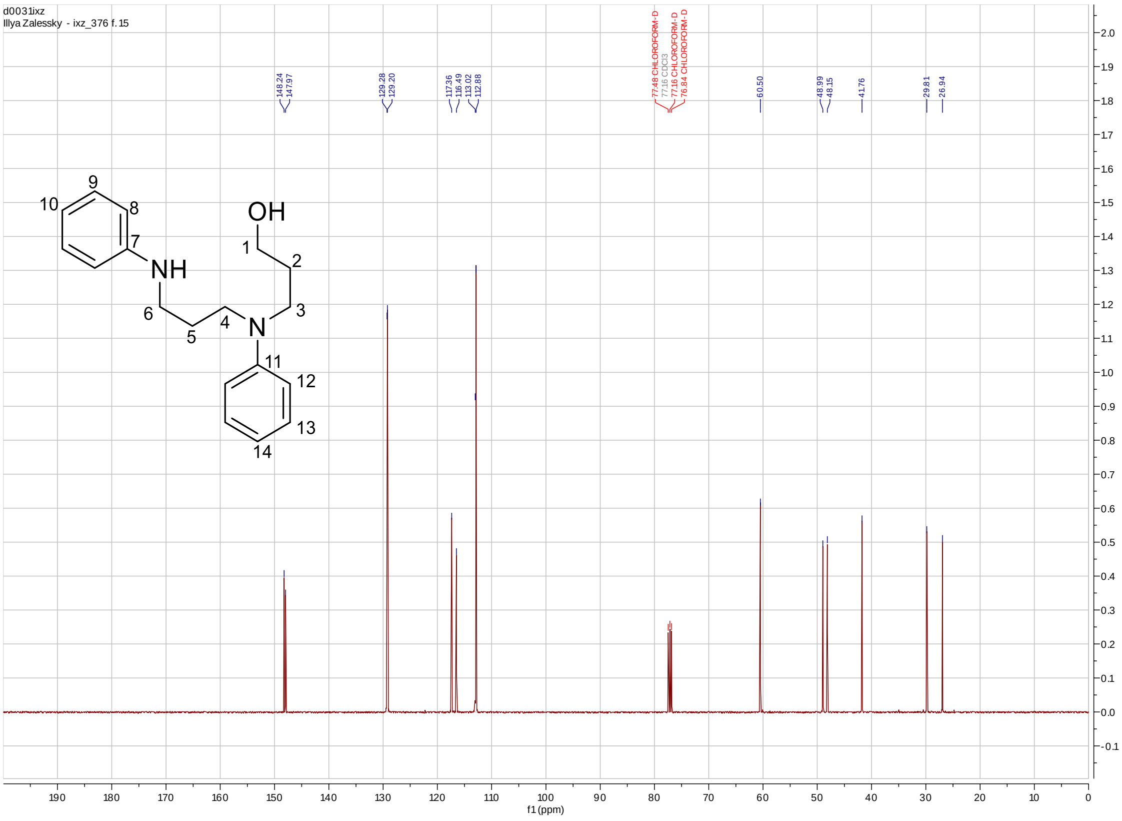

3,7-Diphenyl-1,3,7-oxadiazecan-2-one (94)

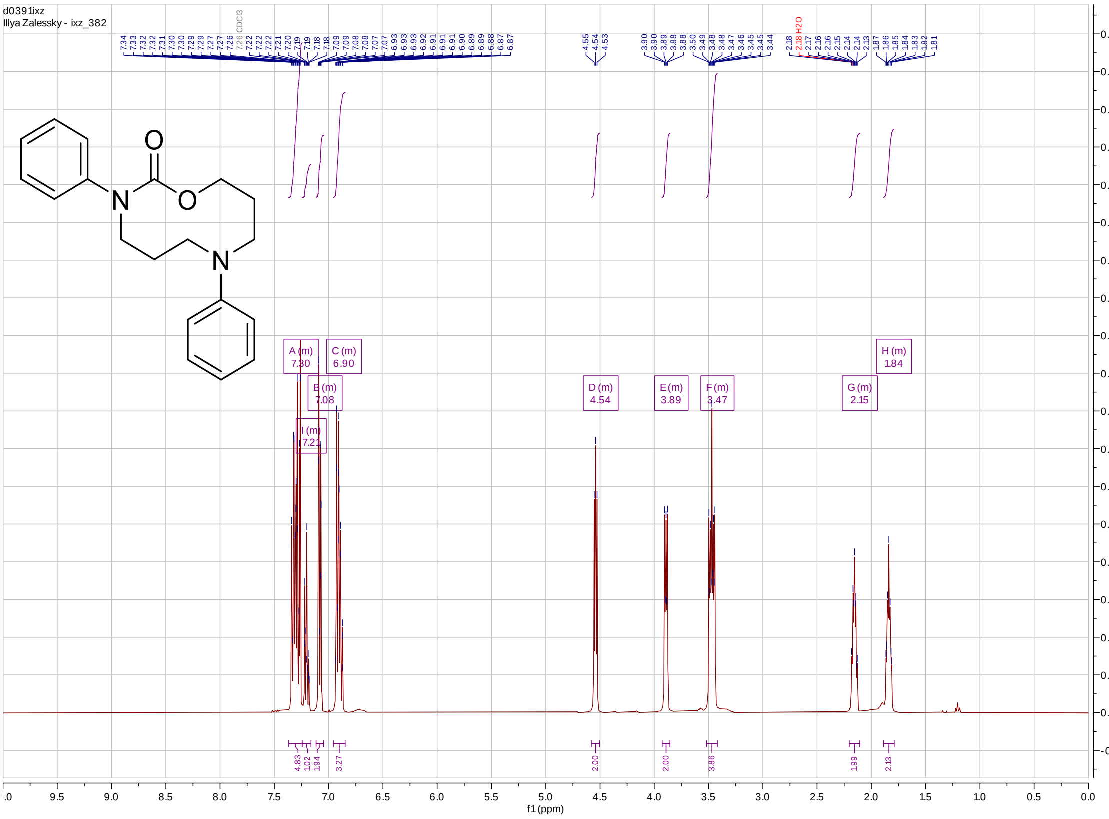

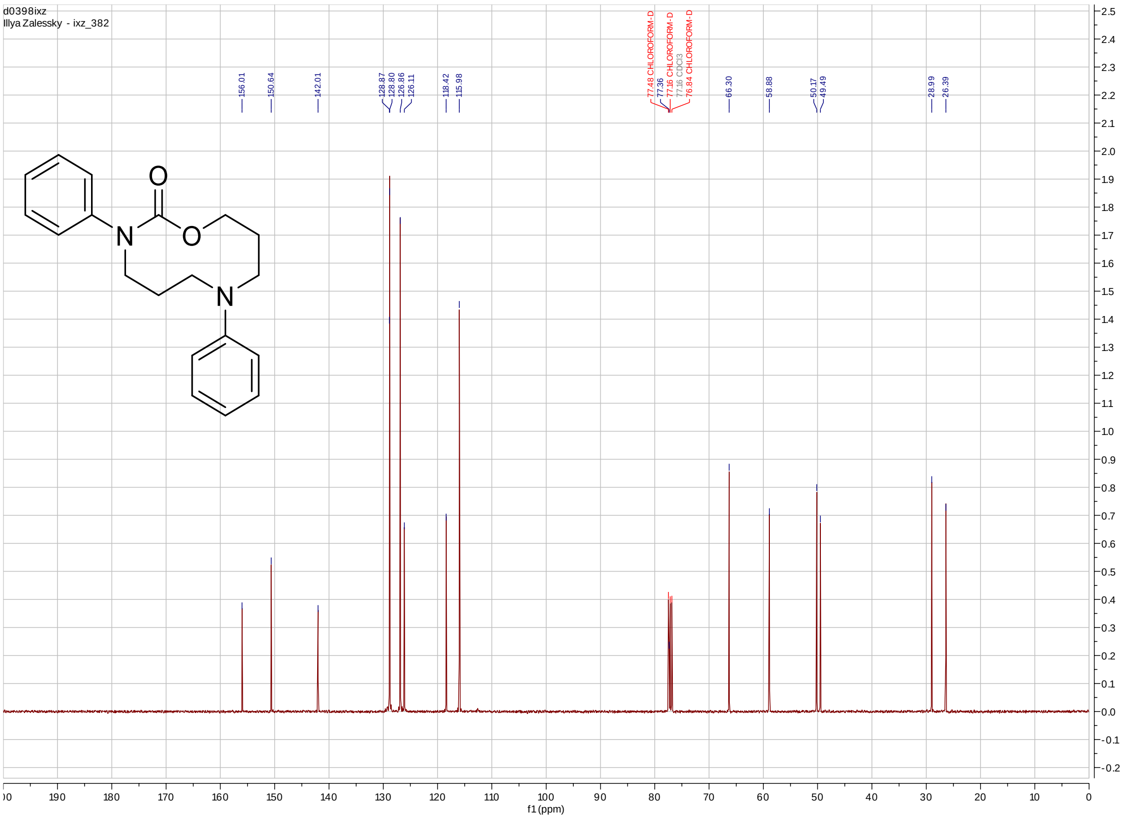

1,7-Dimethyl-4,5,6,7-tetrahydrobenzo[d][1,3,6]oxadiazonine-2(1H)-thione (95)

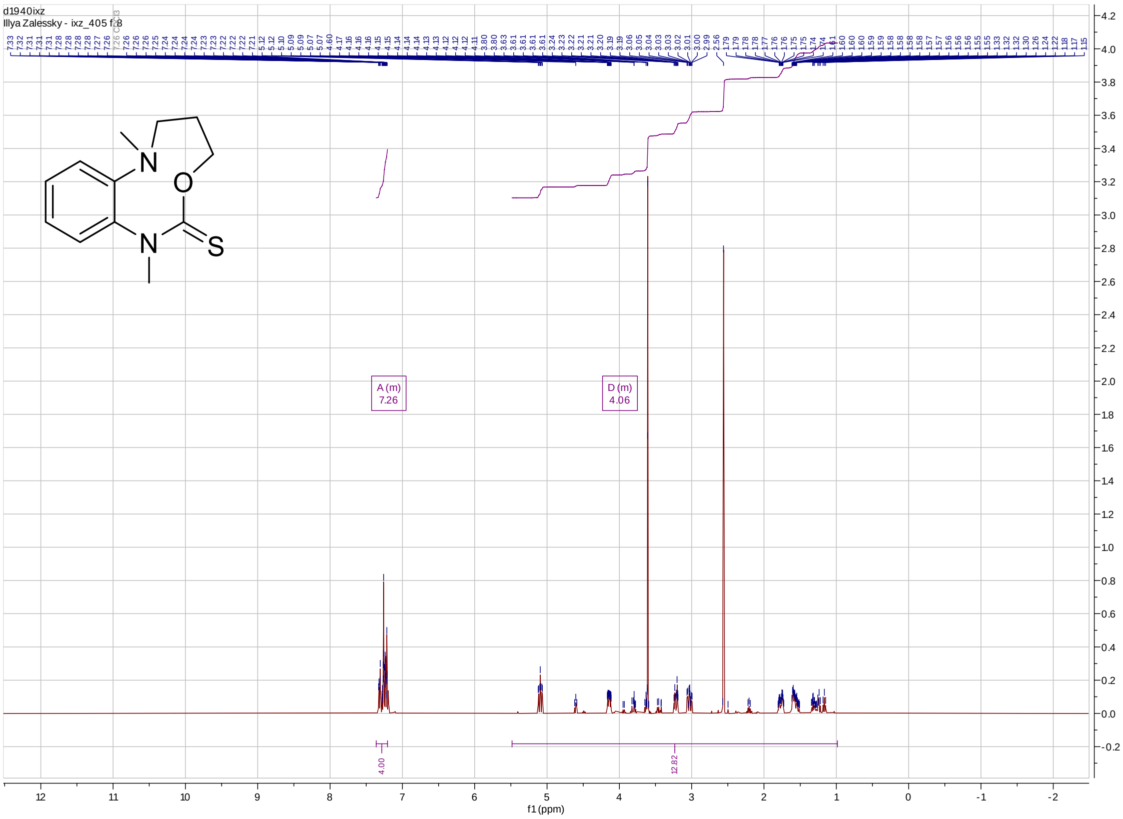

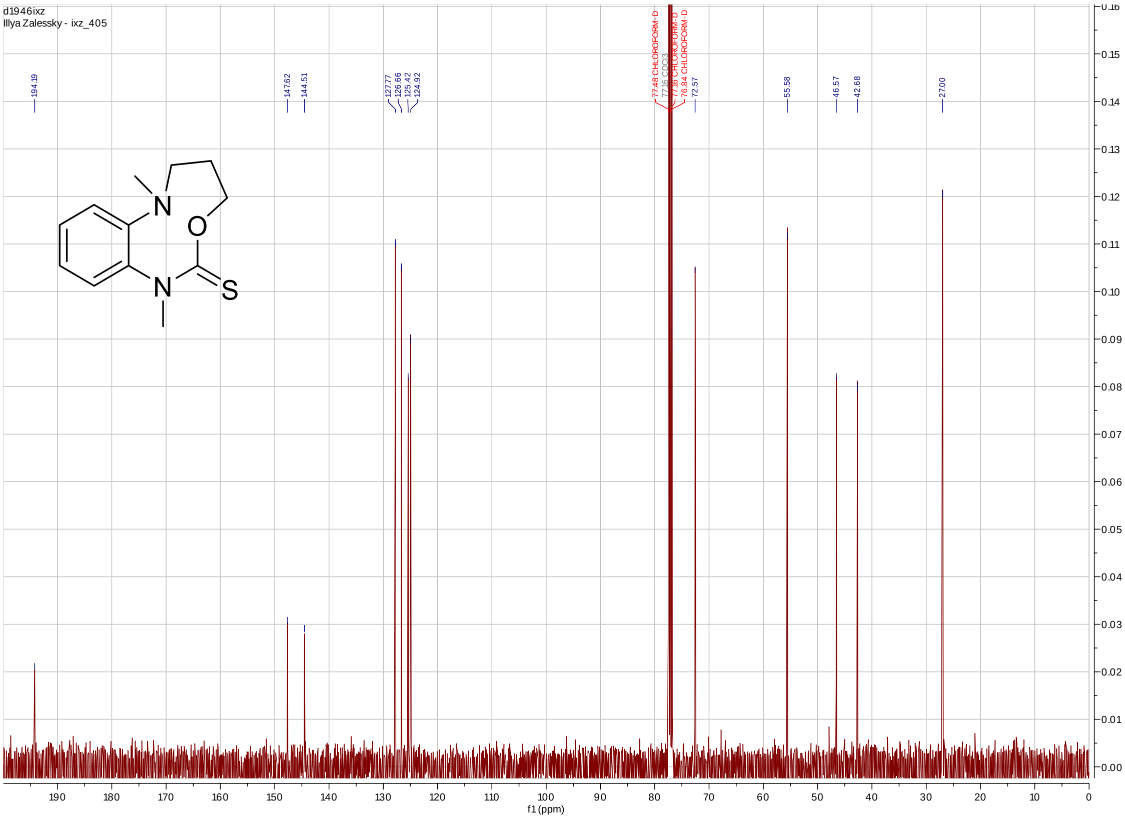

2,6-Divinylpyridine (S139)

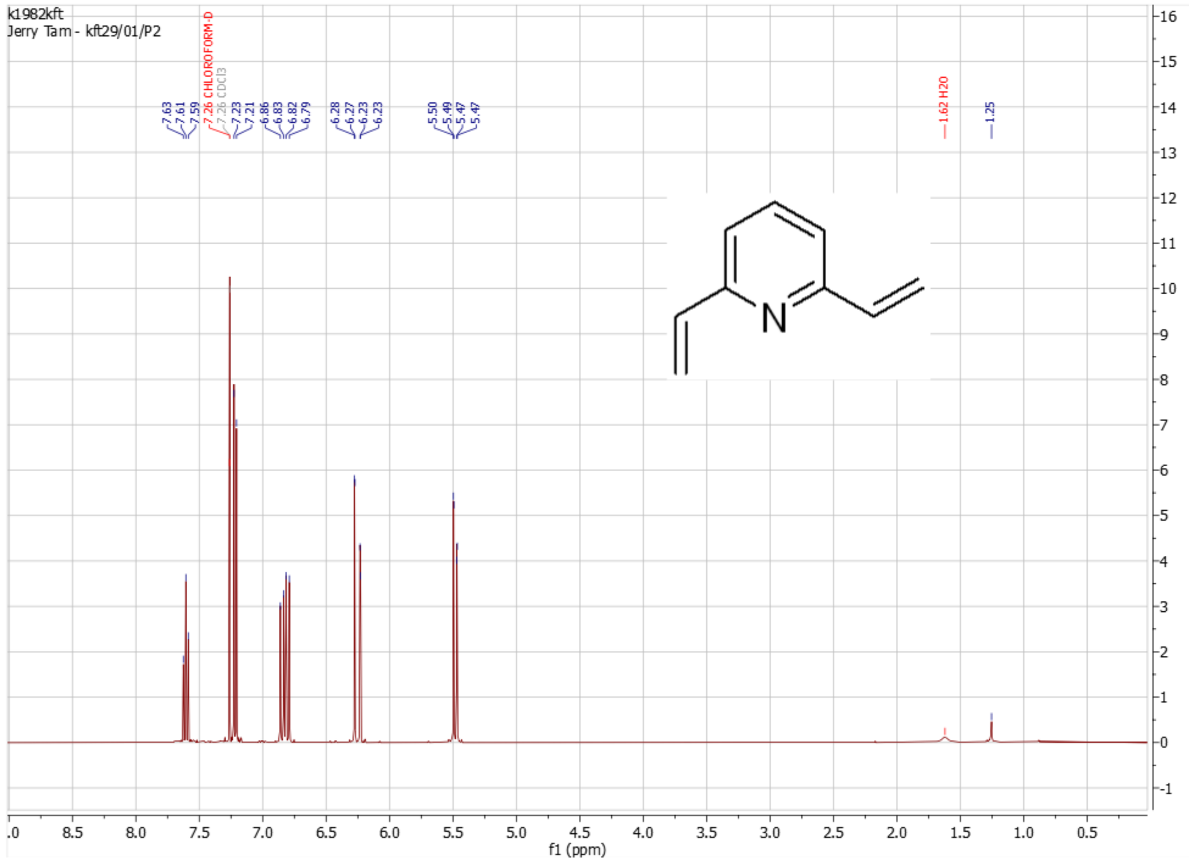

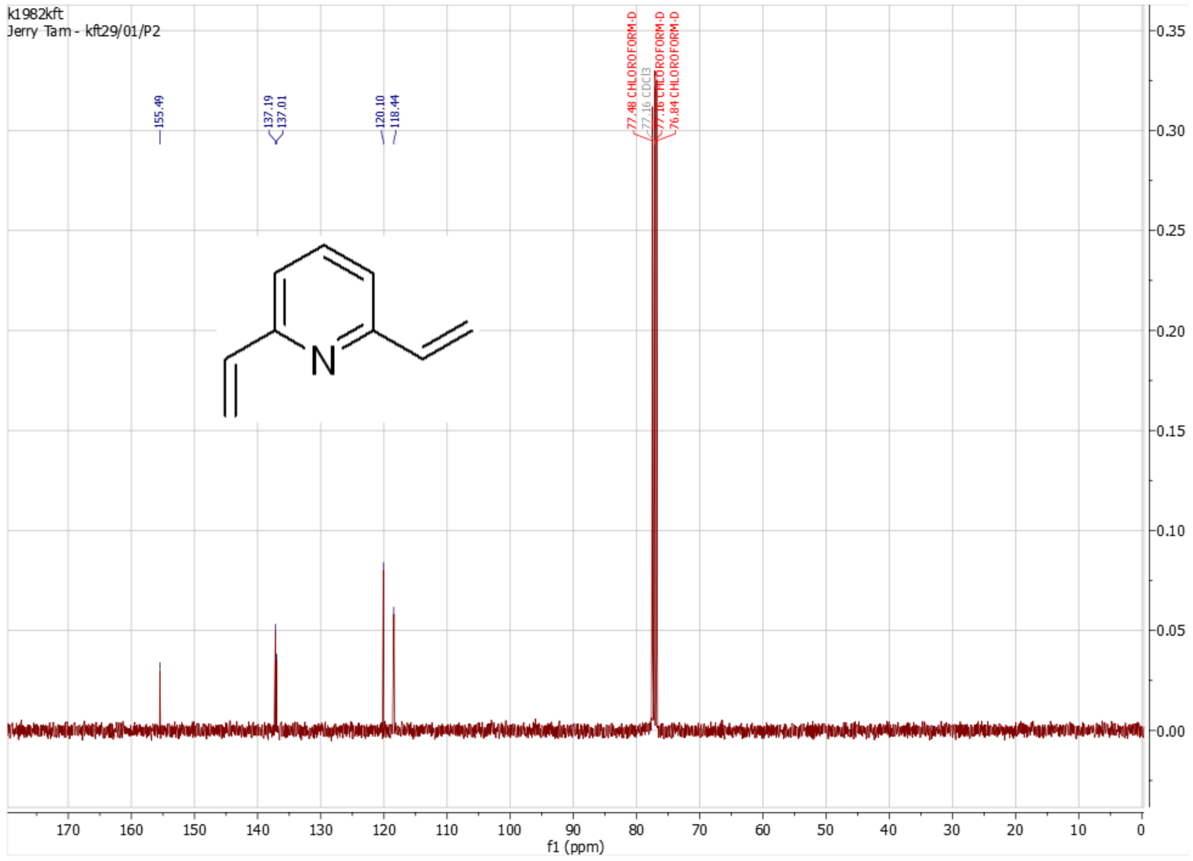

_N_,_N_'-(Pyridine-2,6-diylbis(ethane-2,1-diyl))dianiline (S140)

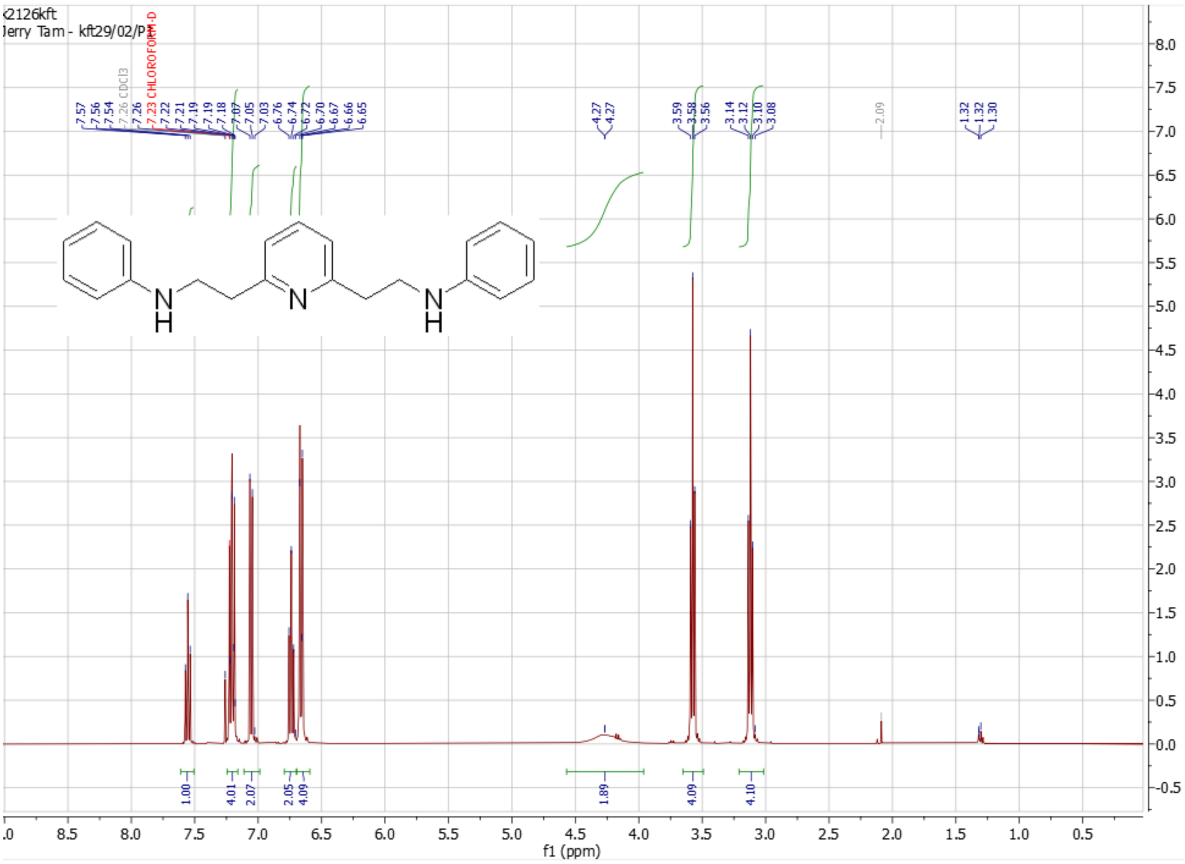

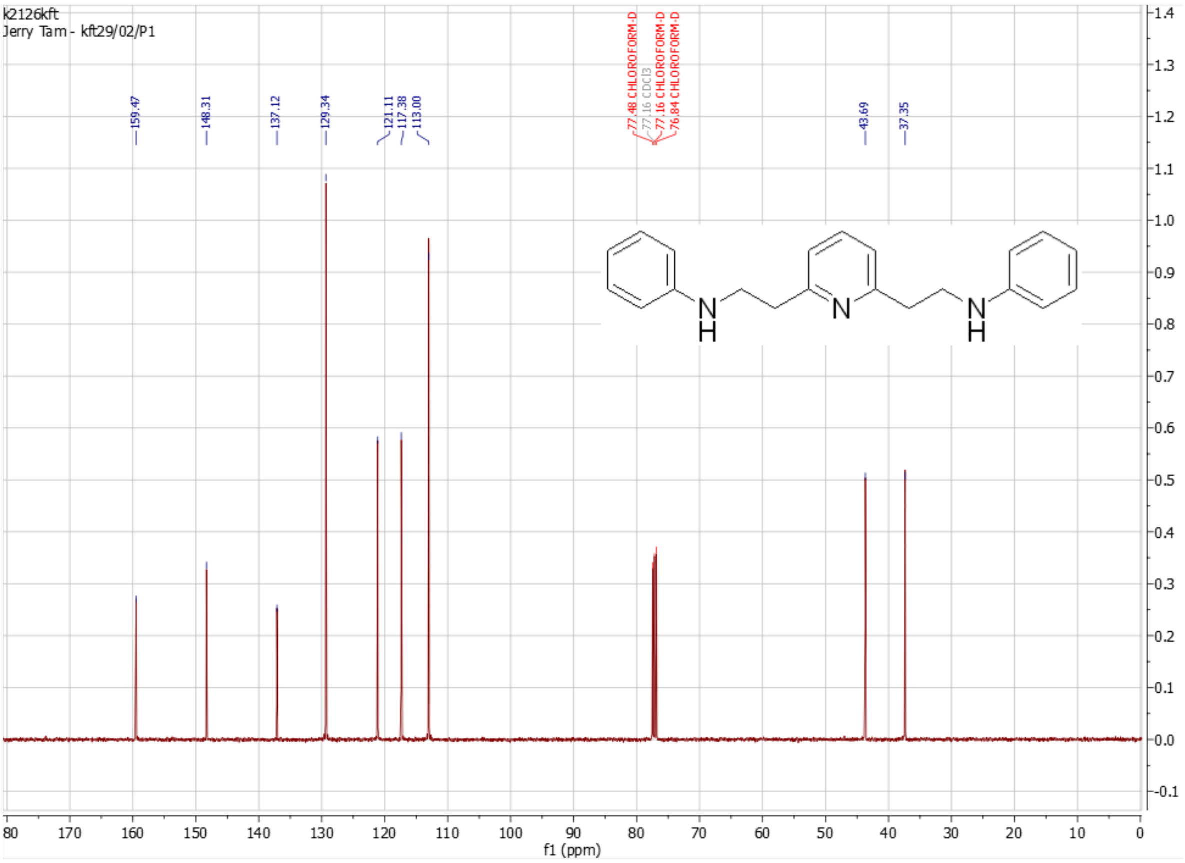

Medium-sized ring (96)

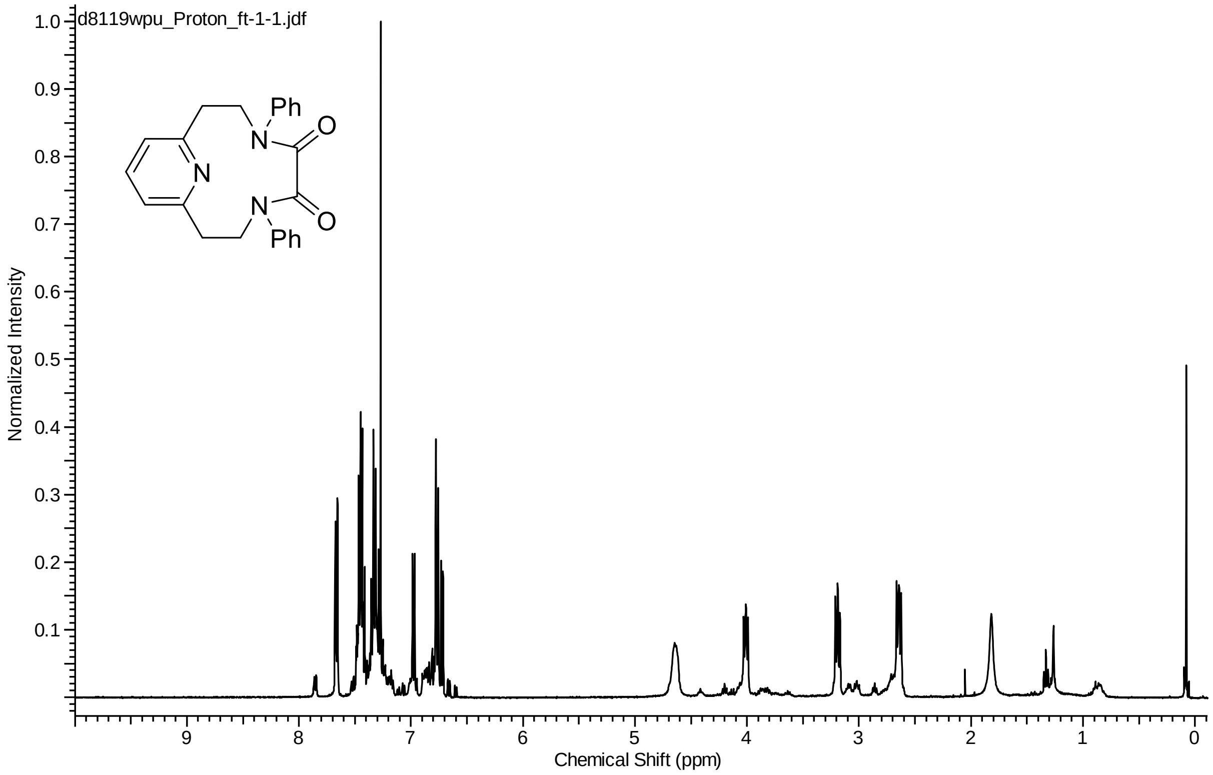

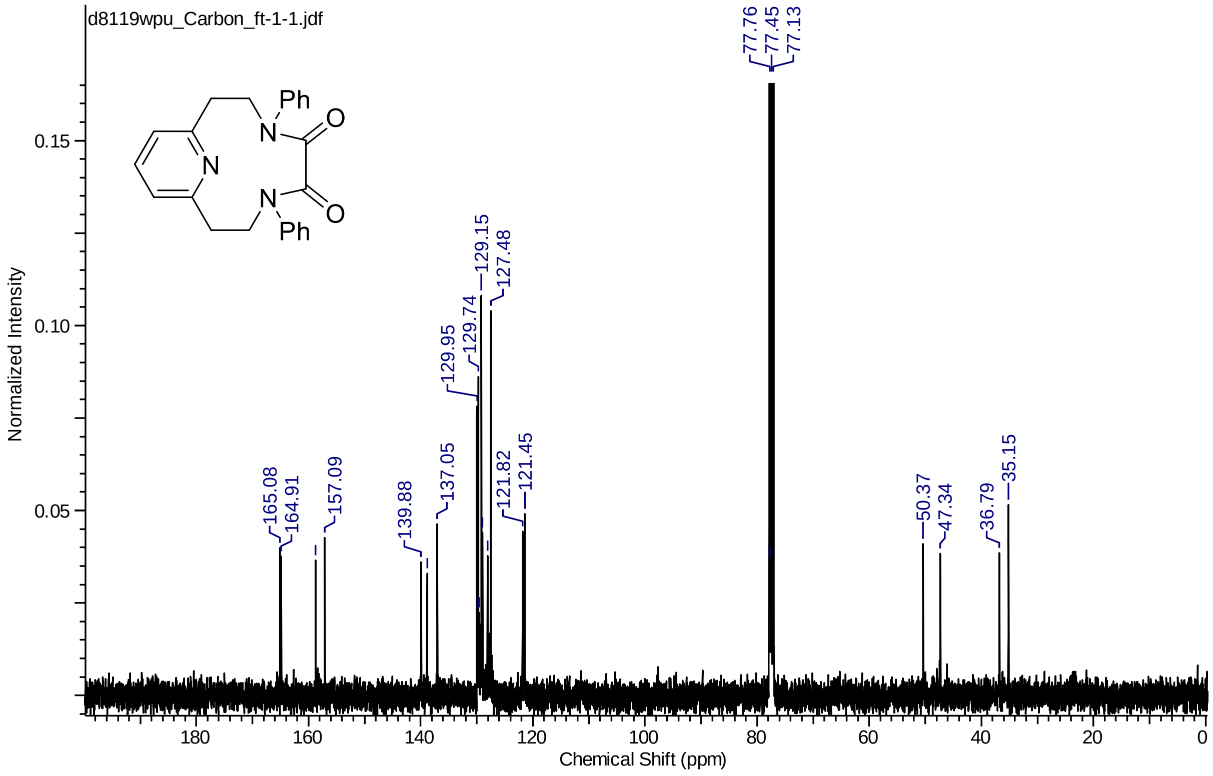

Medium-sized ring (97)

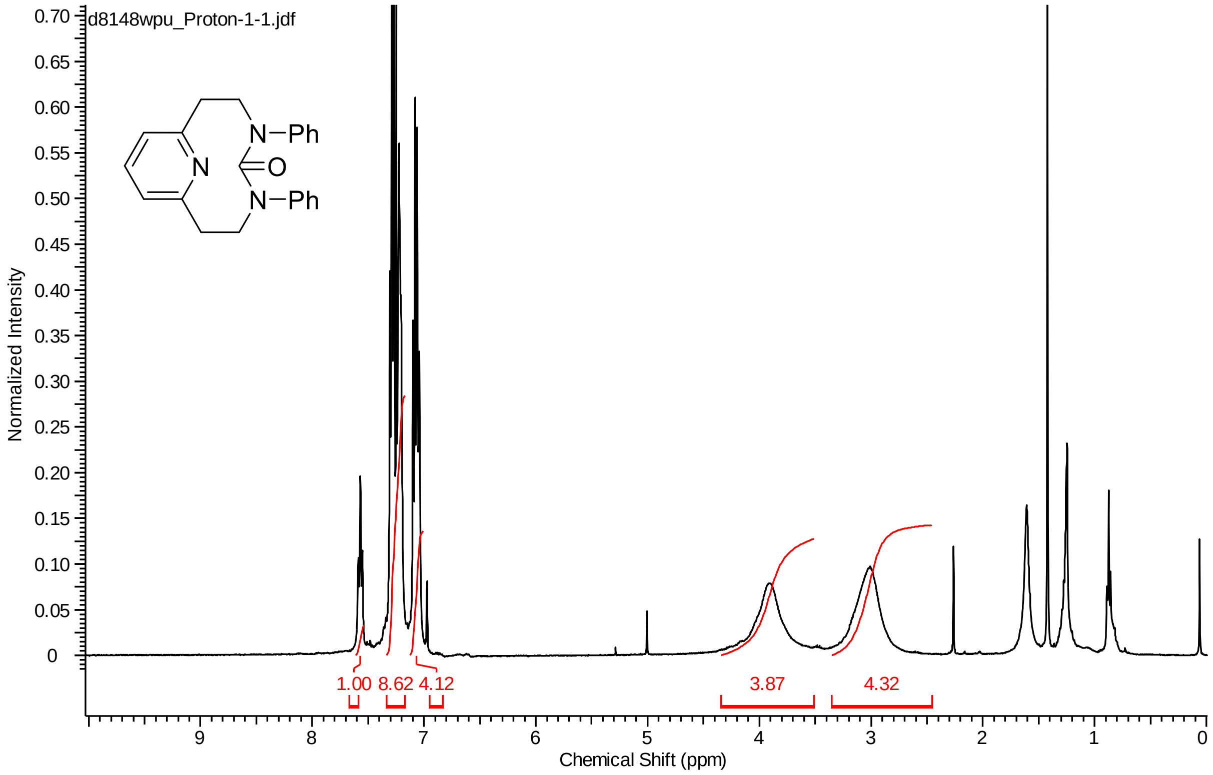

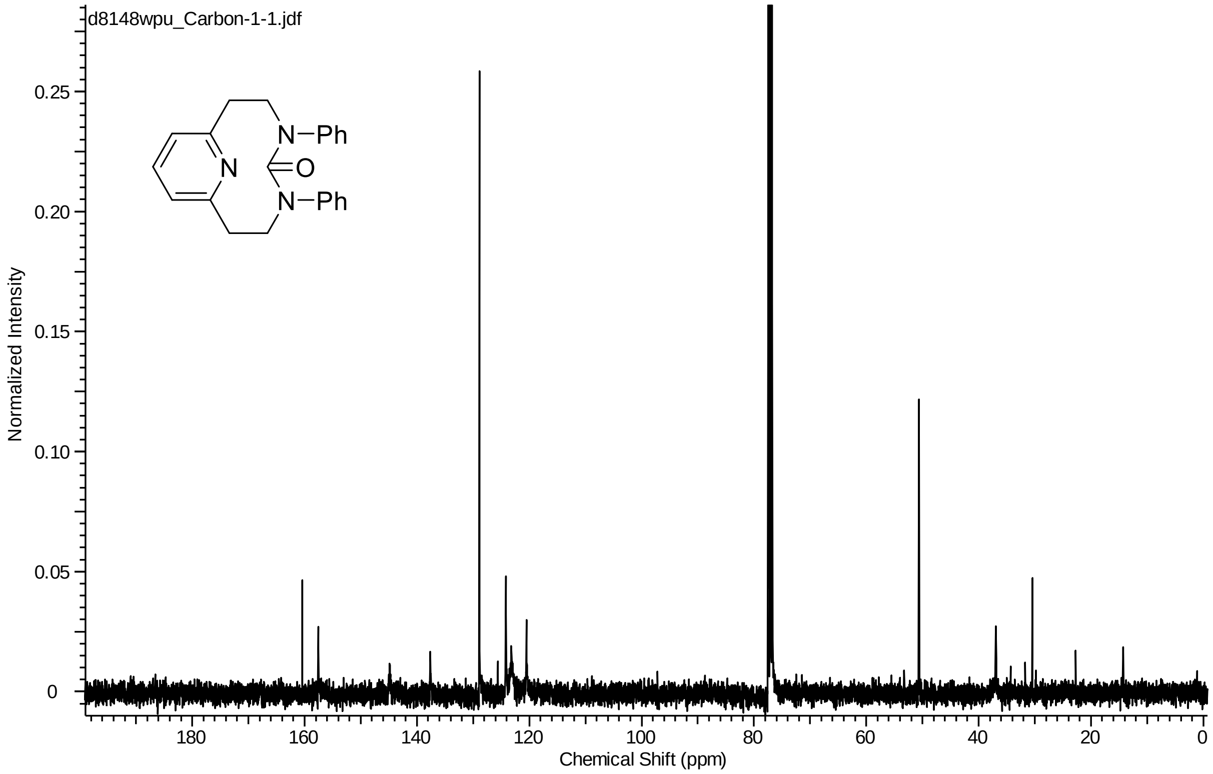

1,4,8-Trimethyl-1,4,8-triazacycloundecane-2,3-dione (98)

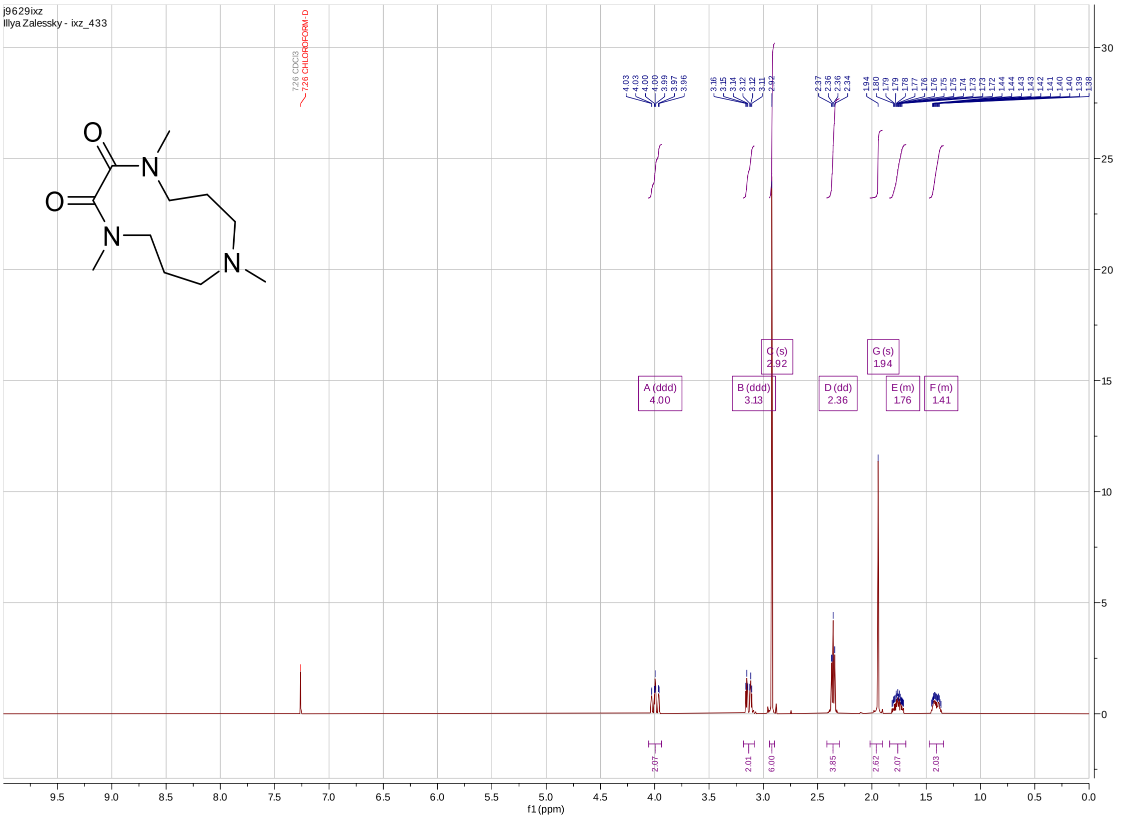

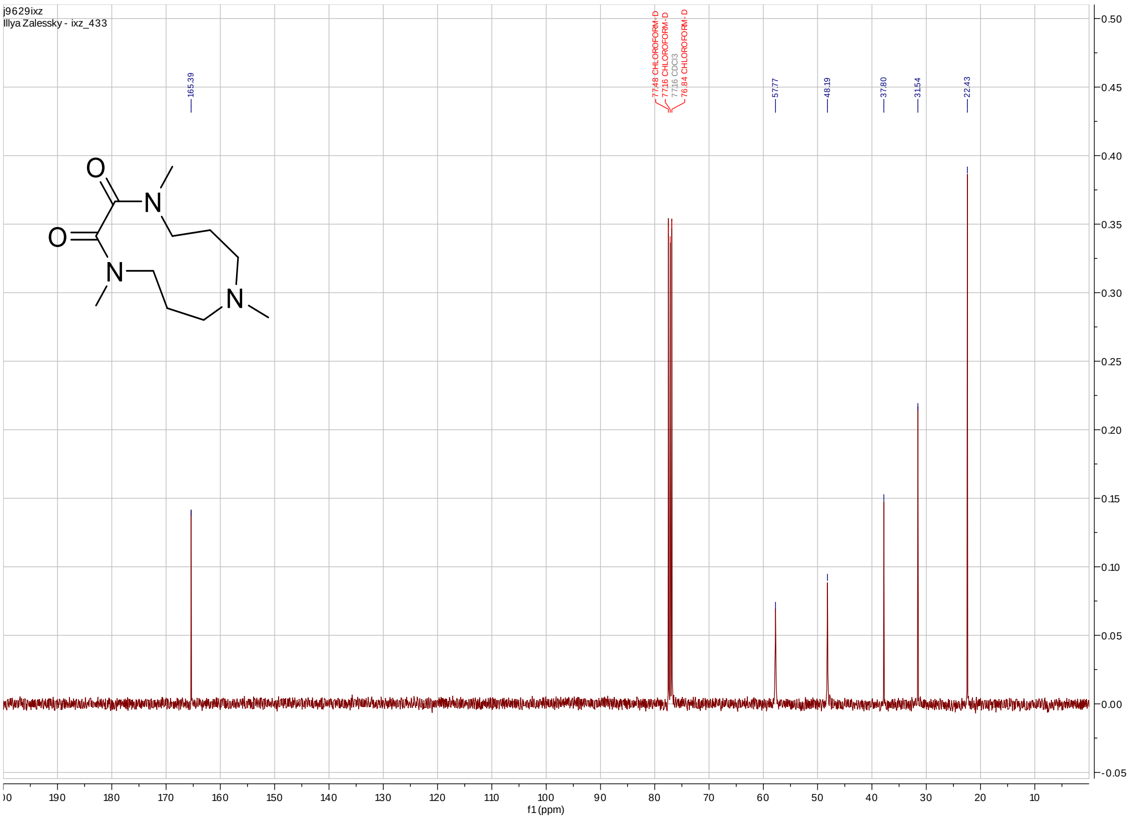

1,4,7-Trimethyl-1,4,7-triazonane-2,3-dione (99)

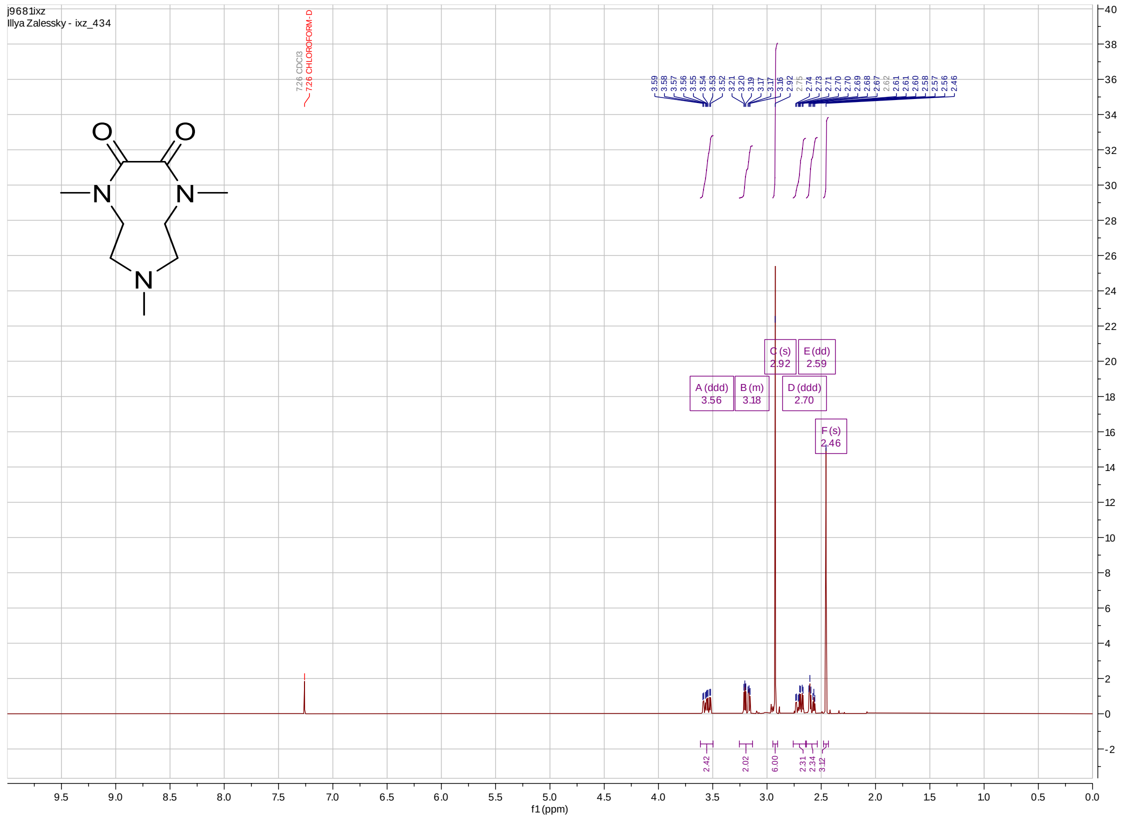

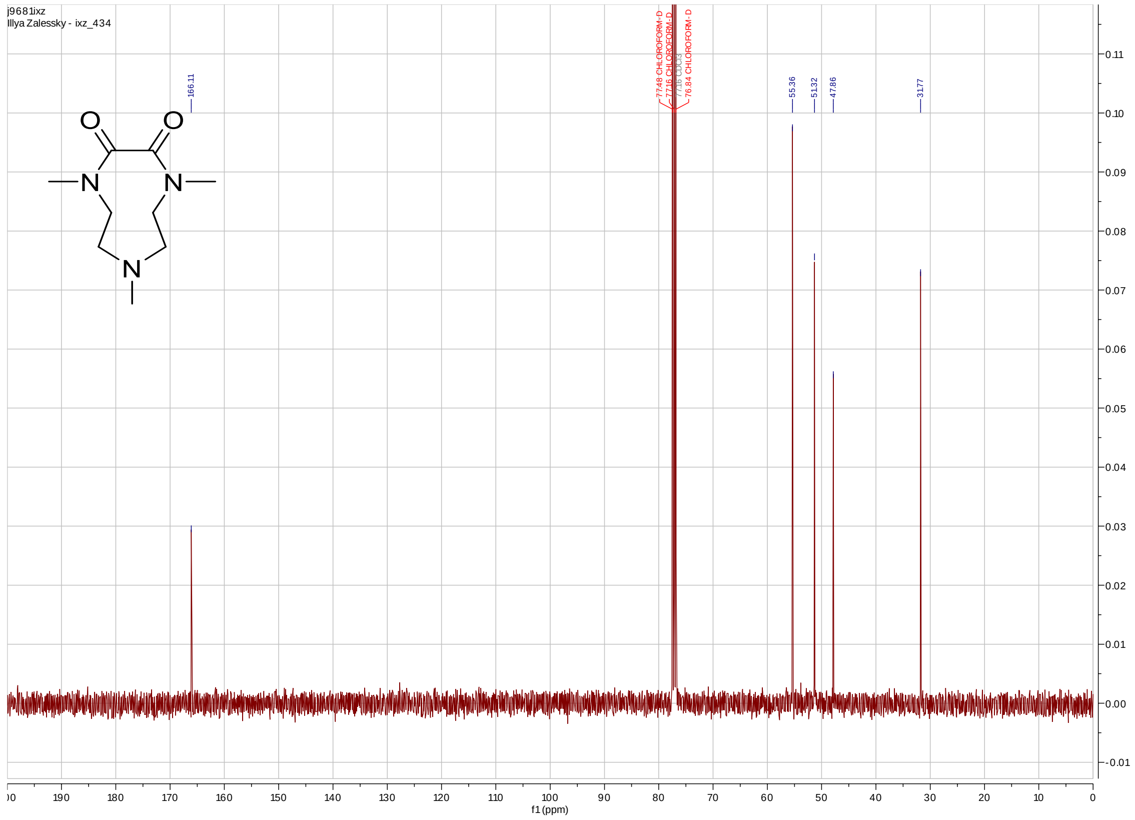

1,3,7-Trimethyl-1,3,7-triazecan-2-one (100)

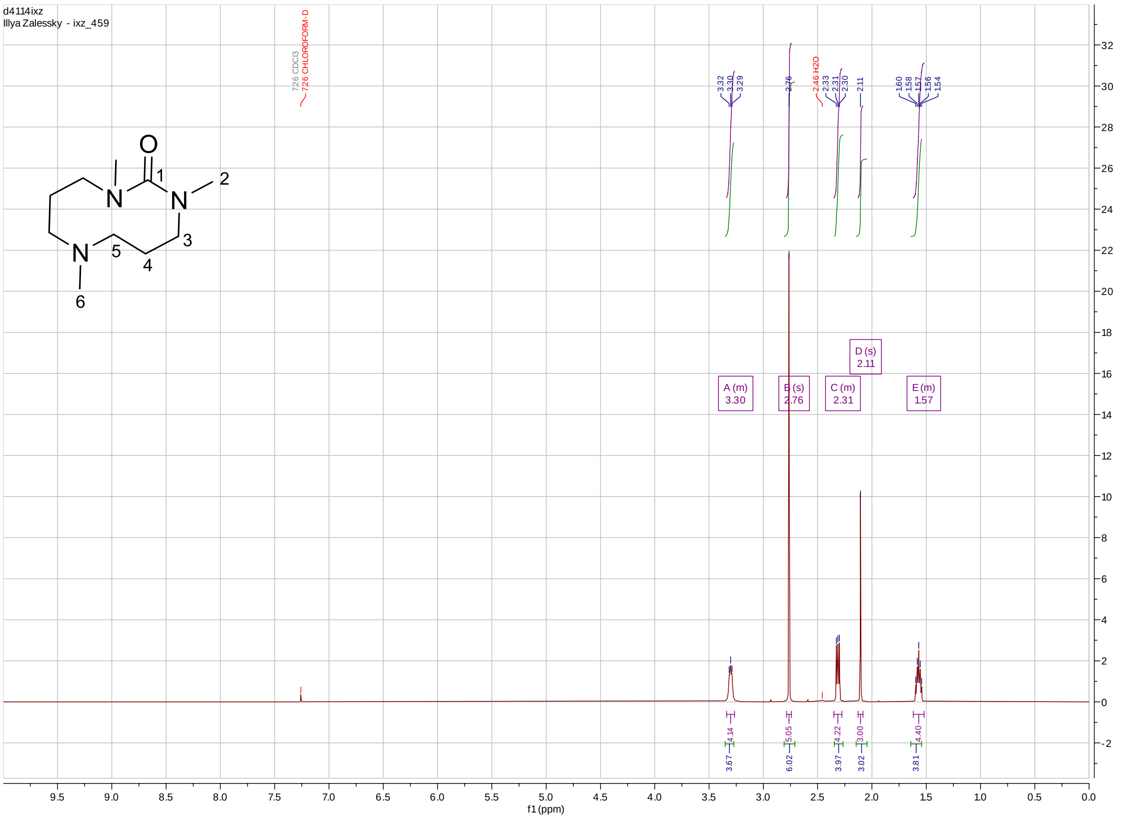

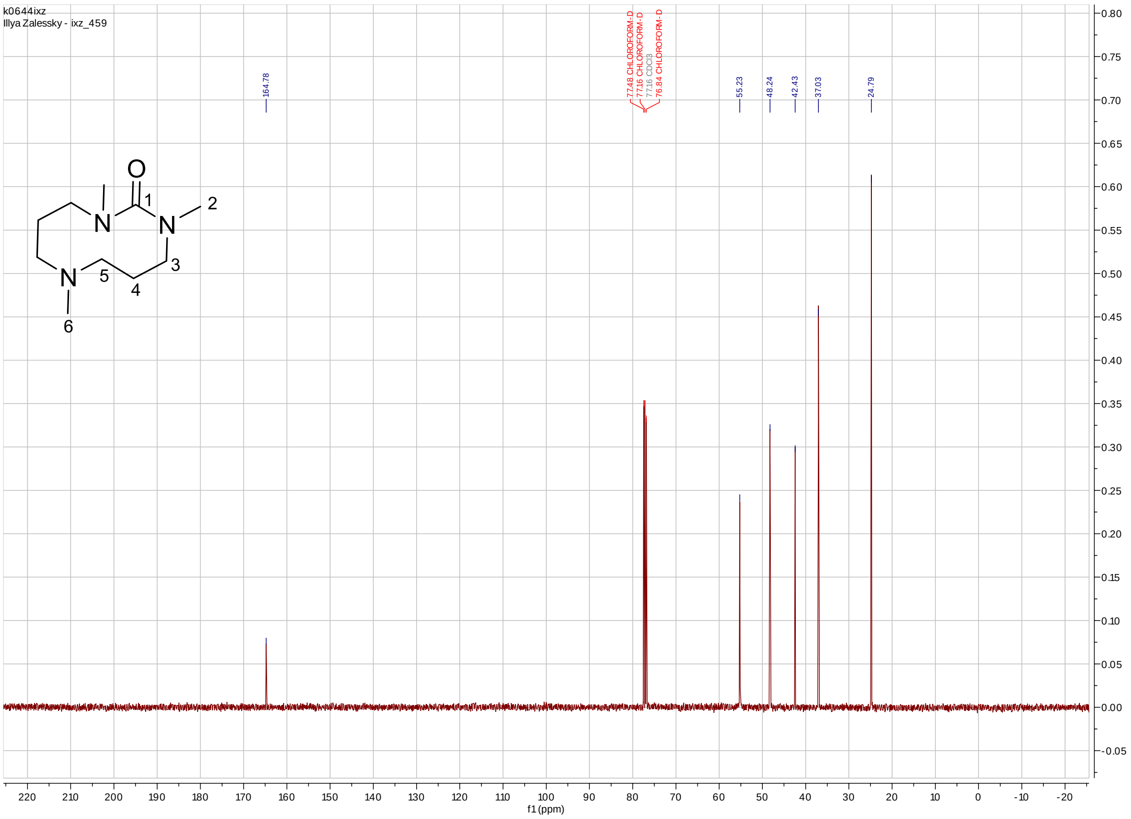

N-phenyl-3-(phenylamino)-N-(2-(phenylamino)ethyl)propenamide (S141)

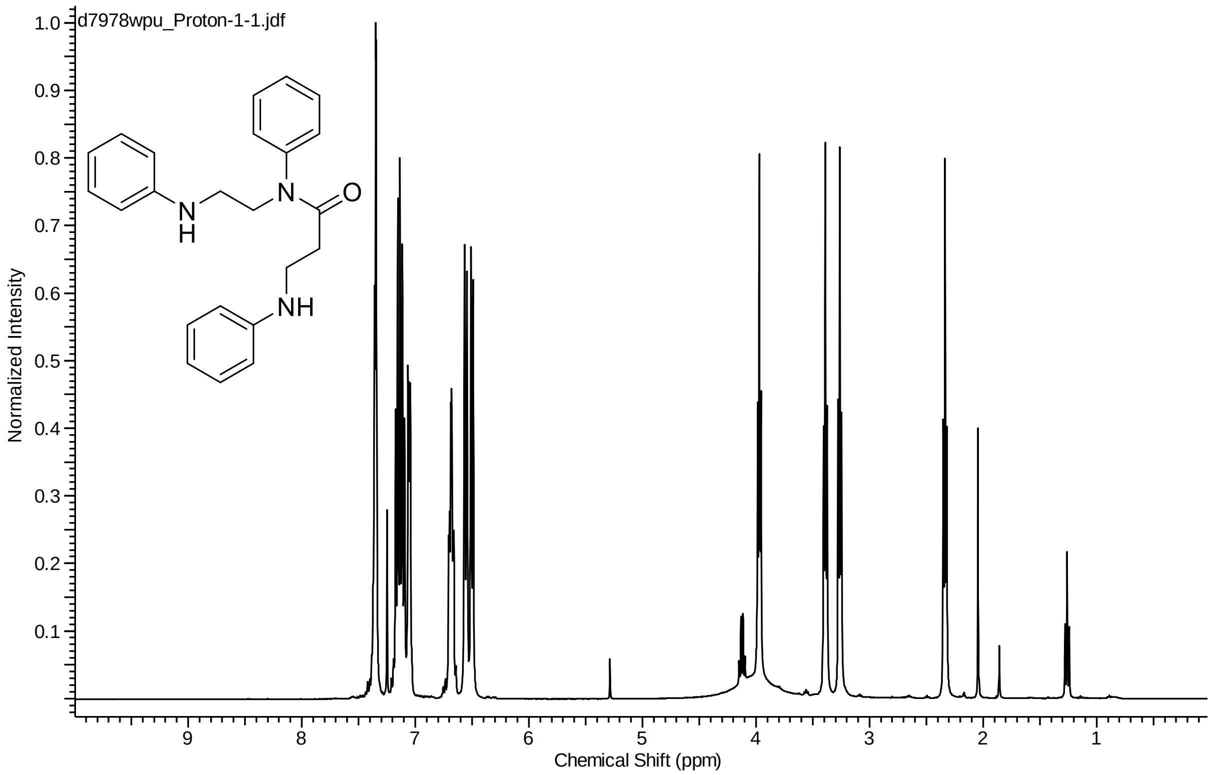

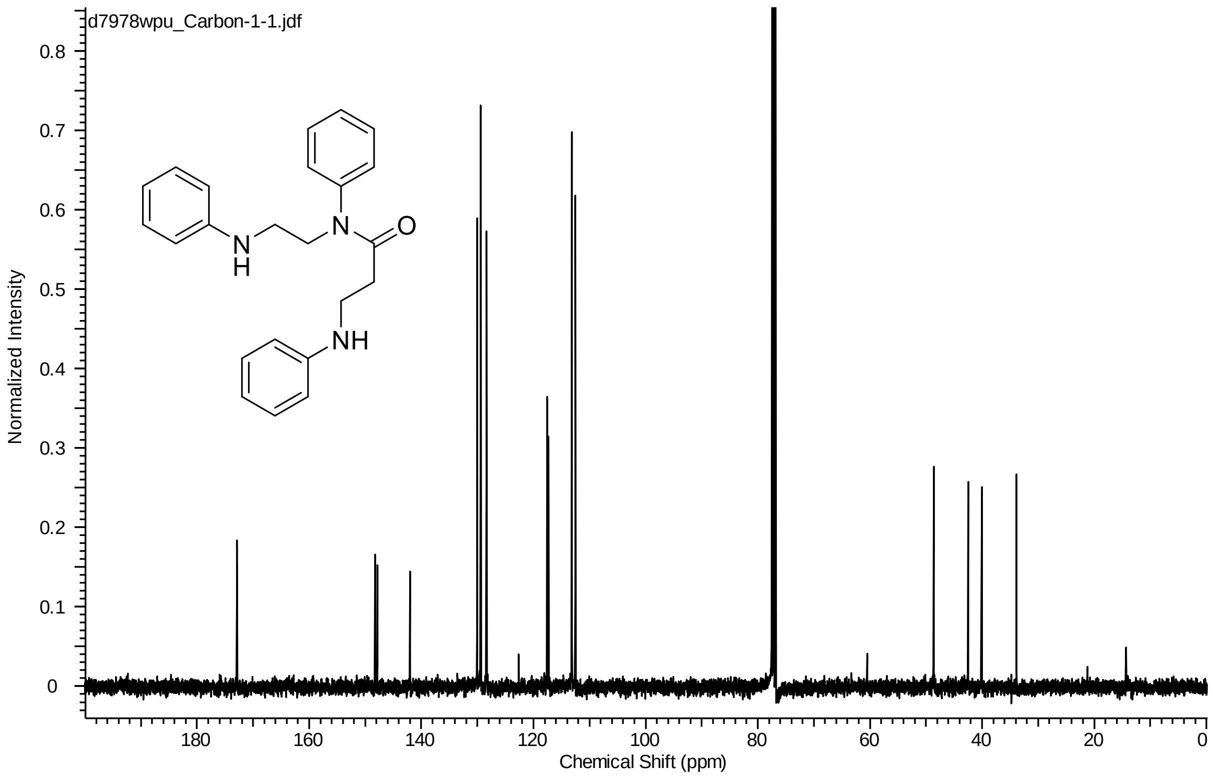

N1,N3-diphenyl-N1-(2-(phenylamino)ethyl)propane-1,3-diamine (S142)

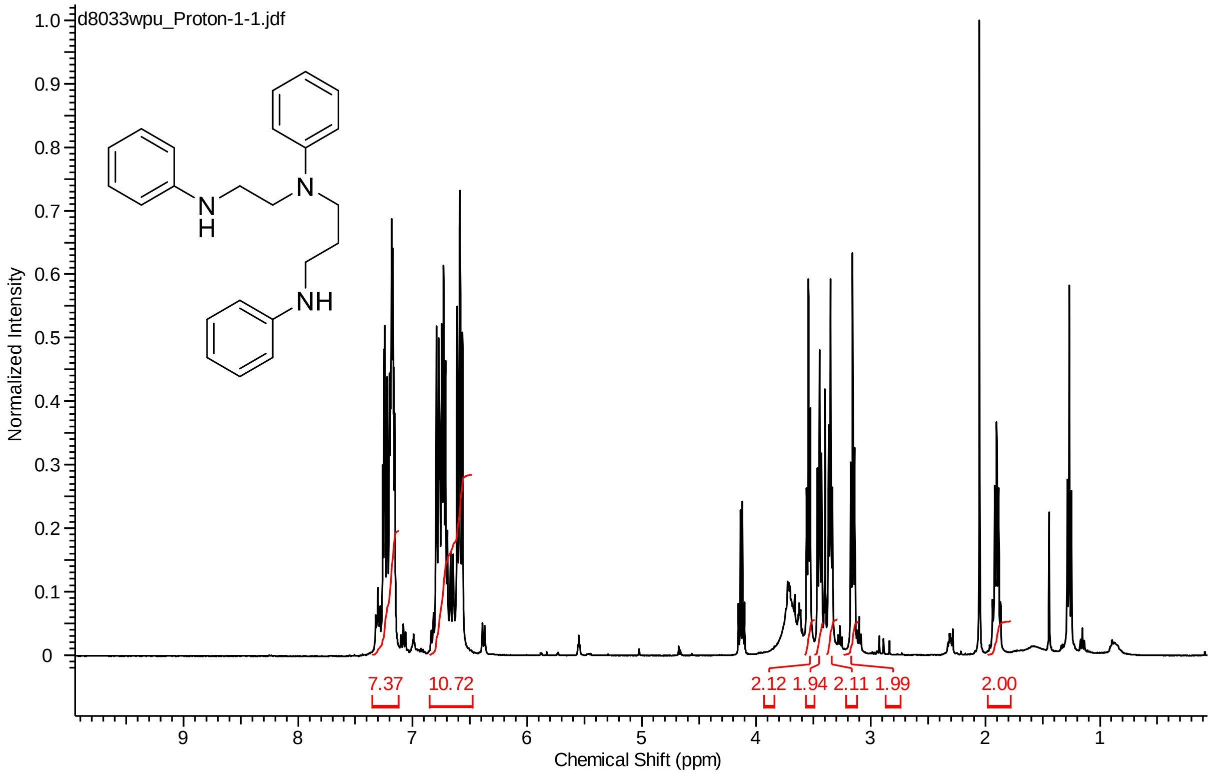

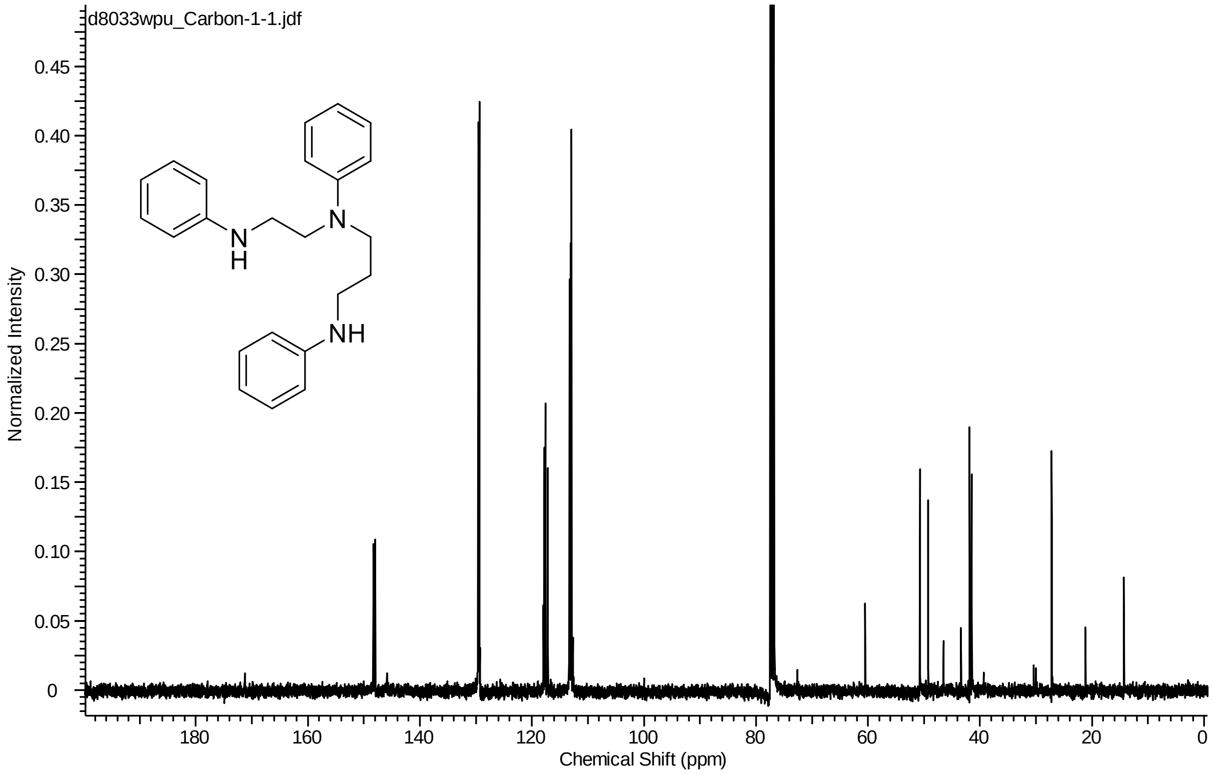

1,3,6-Triphenyl-1,3,6-triazonan-2-one (101)

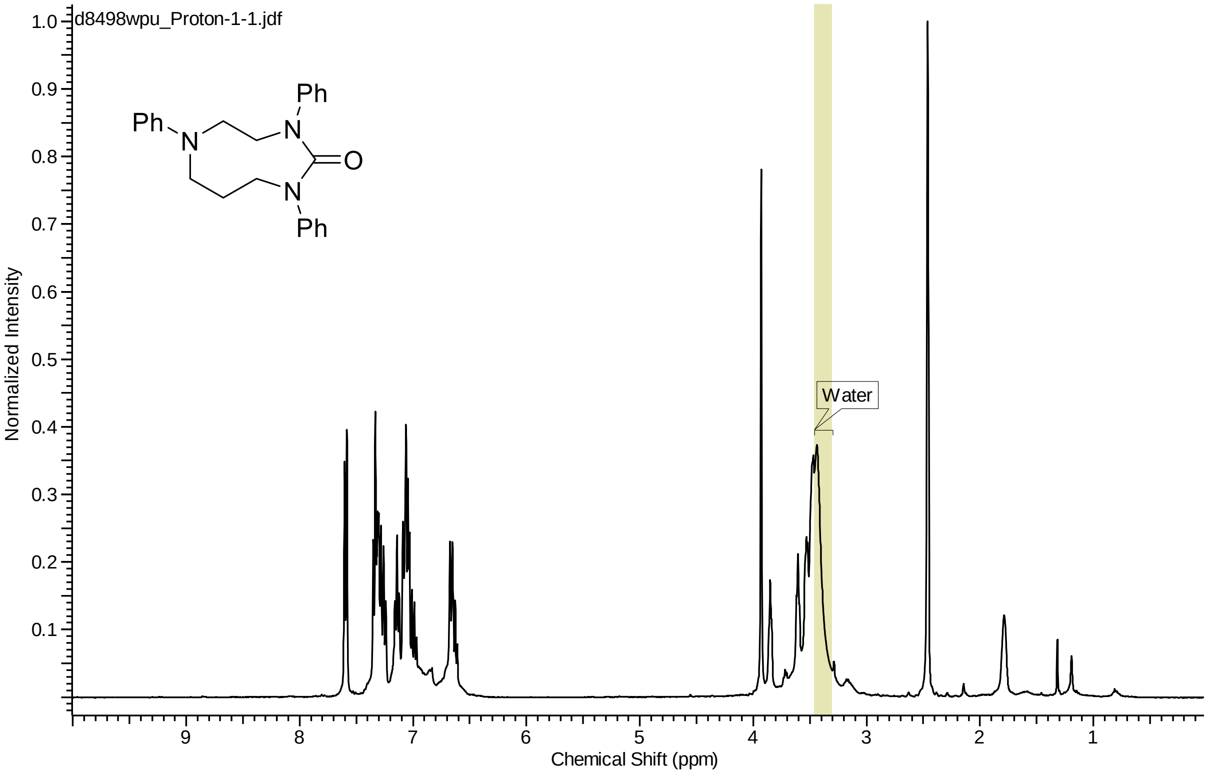

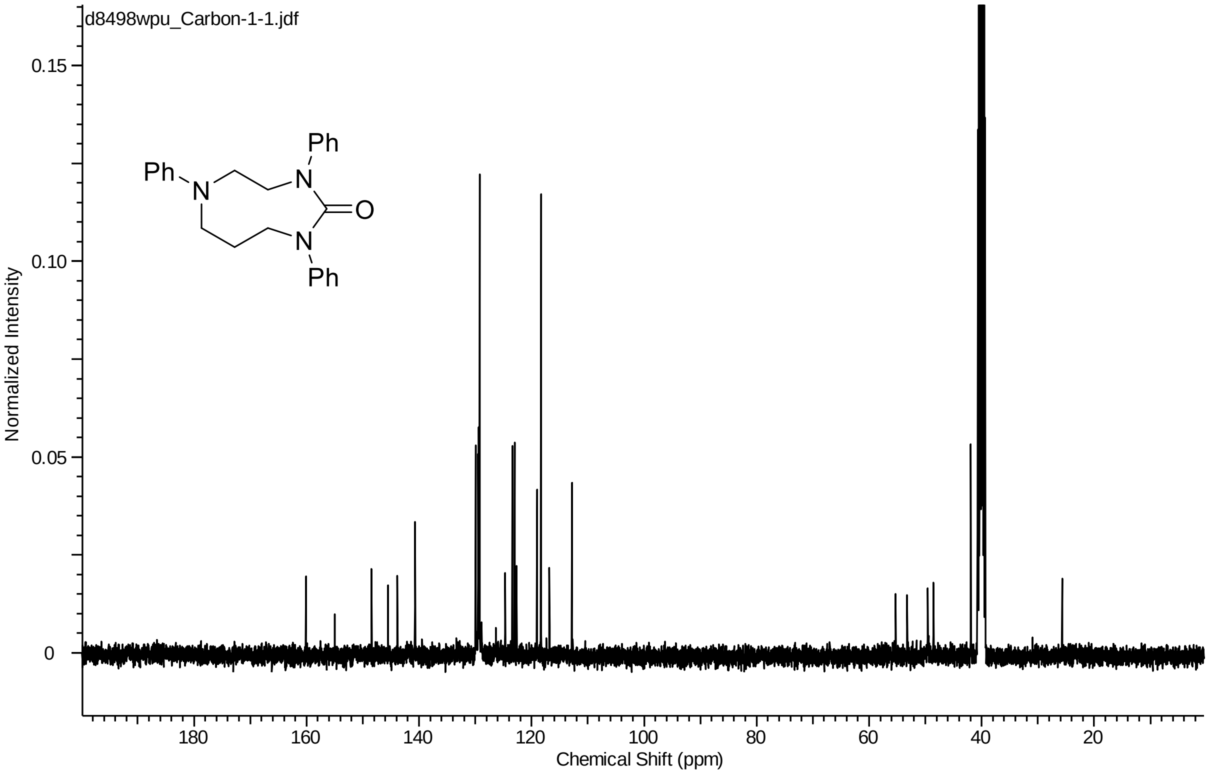

1,3,6-Trimethyl-1,3,6-triazocan-2-one (102)

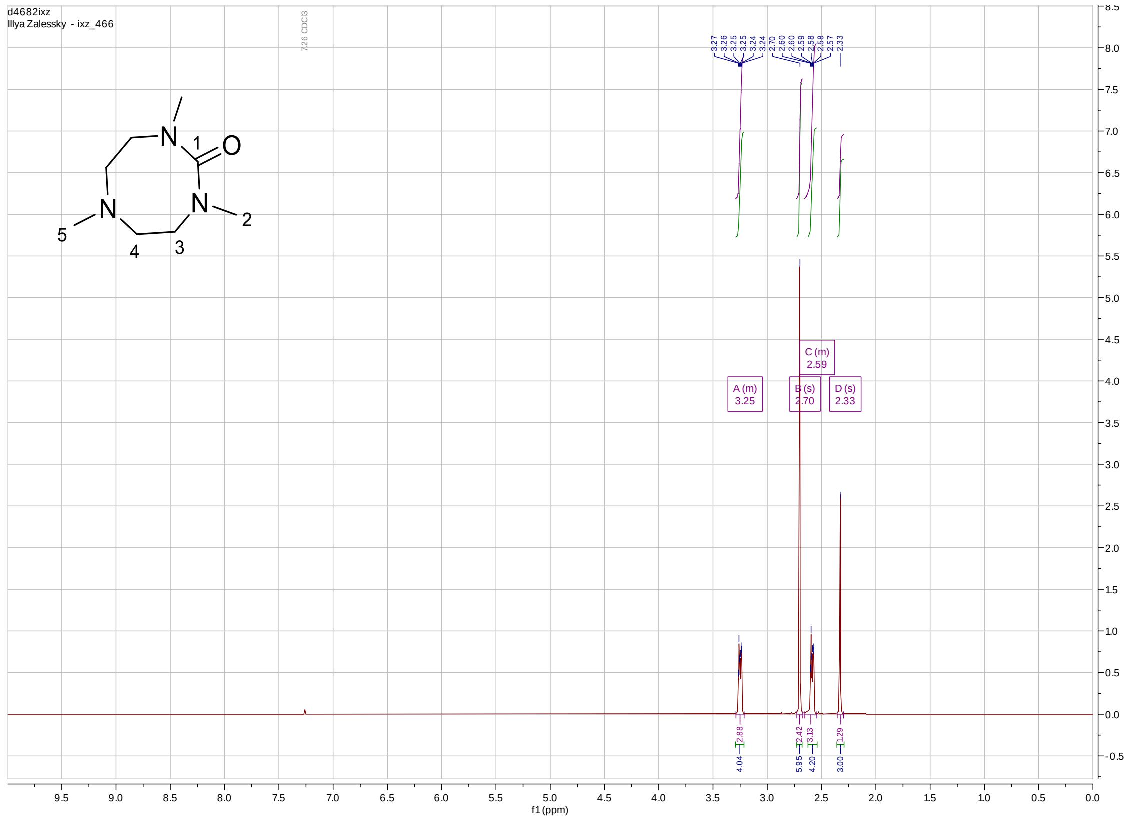

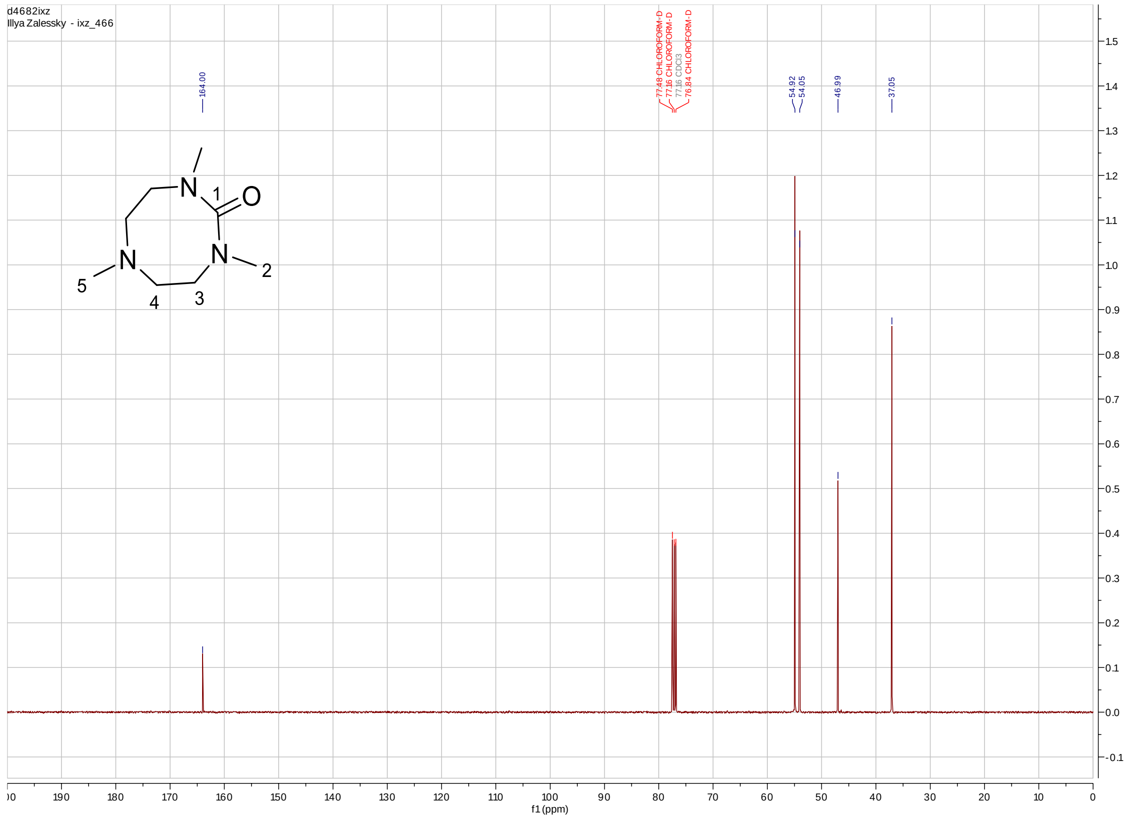

4,7,10-Trimethyl-1,2,4,7,10-thiatetrazecan-3-one 1,1-dioxide (103)

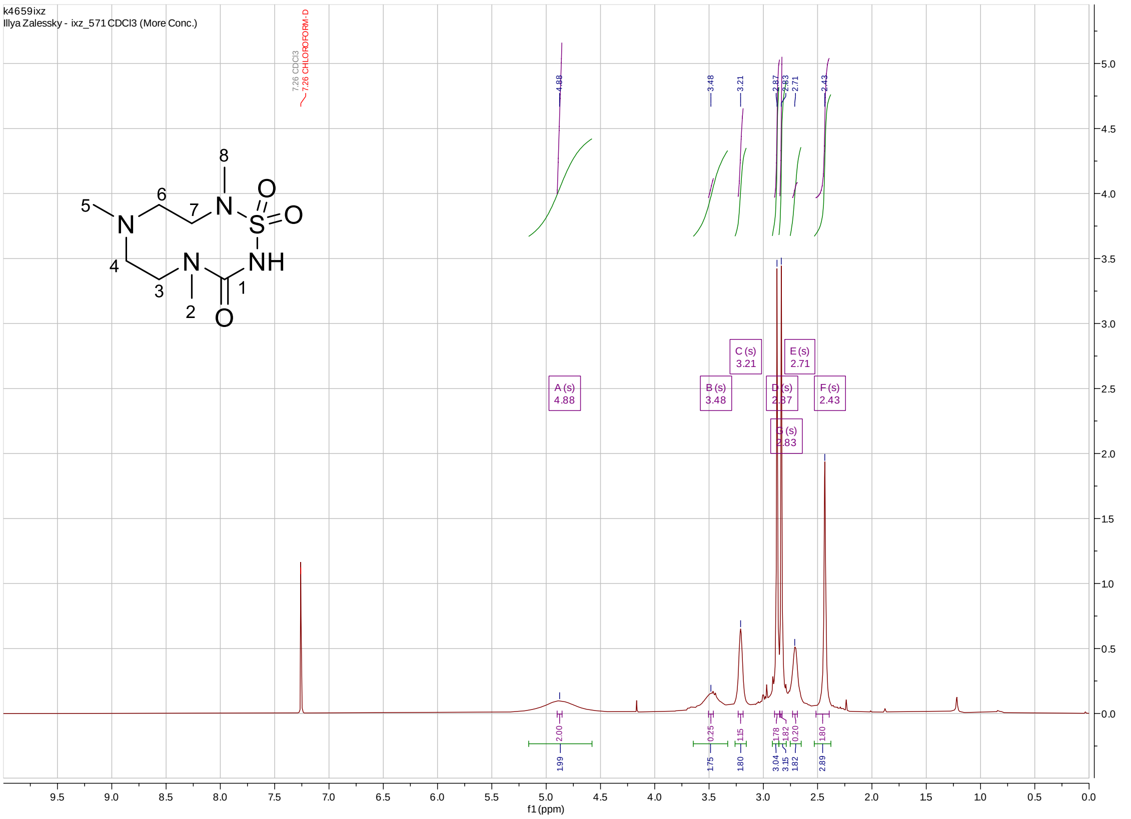

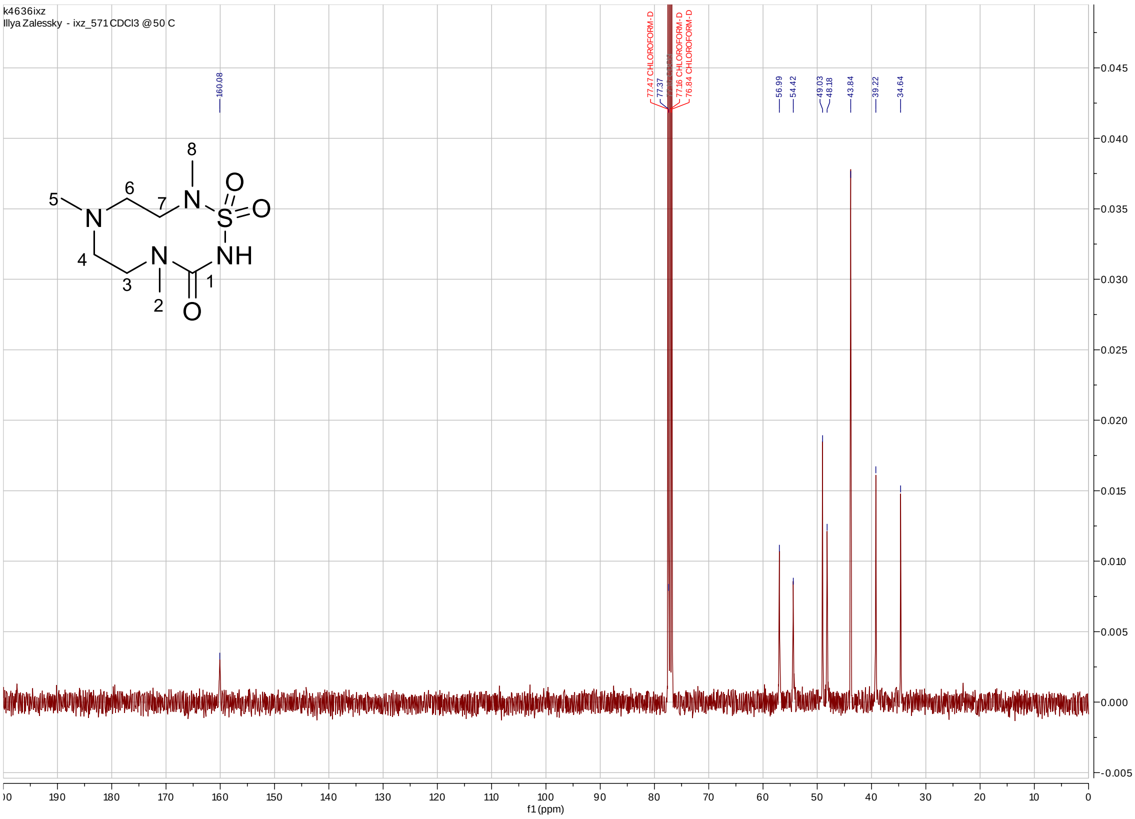

Methyl 2-((benzyl (2-hydroxybenzyl amino) methyl) benzoate (104)

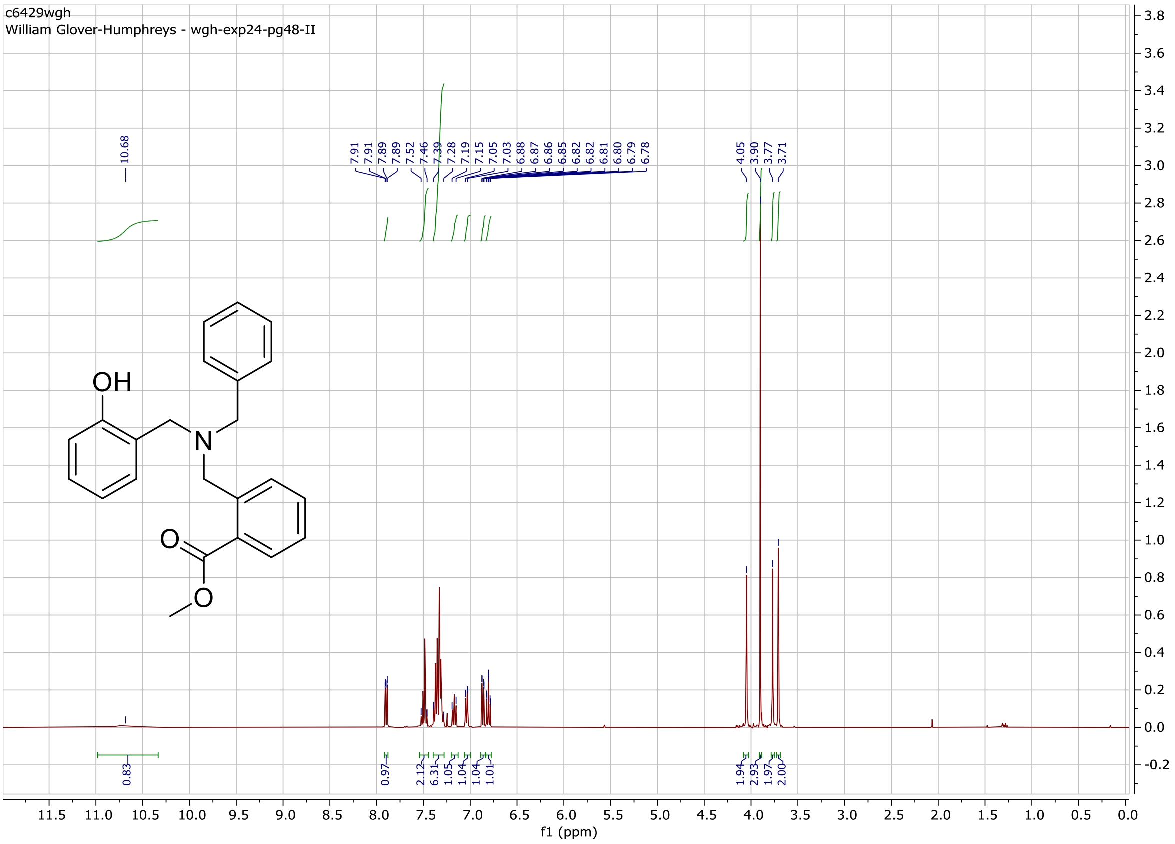

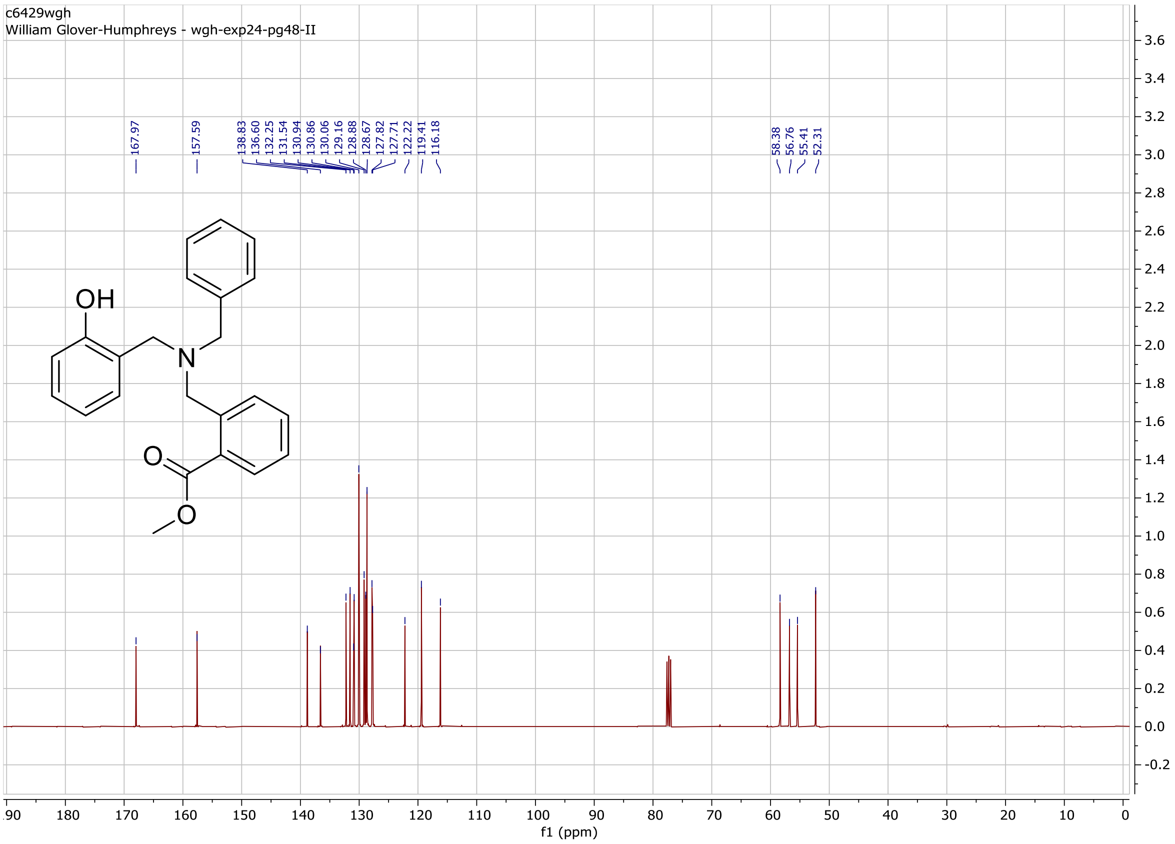

2-Benzylisoindolin-1-one (106)

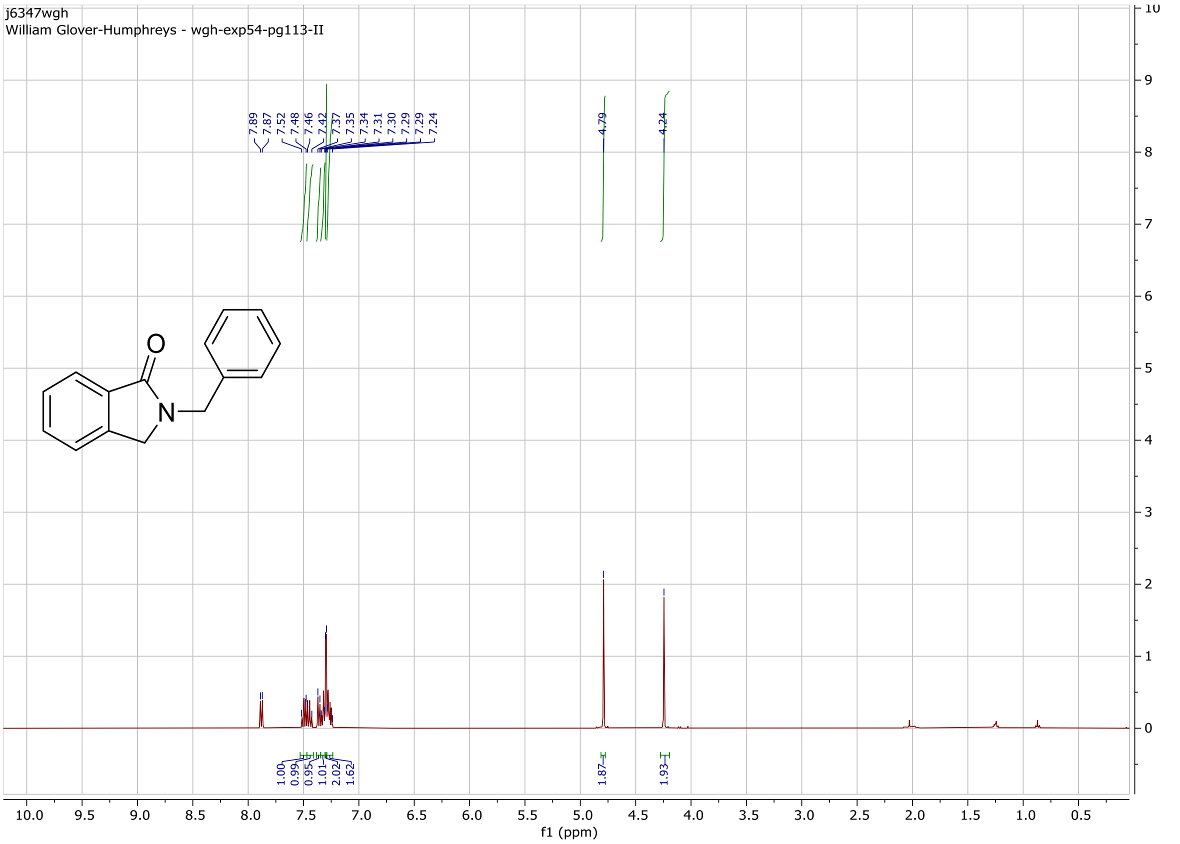

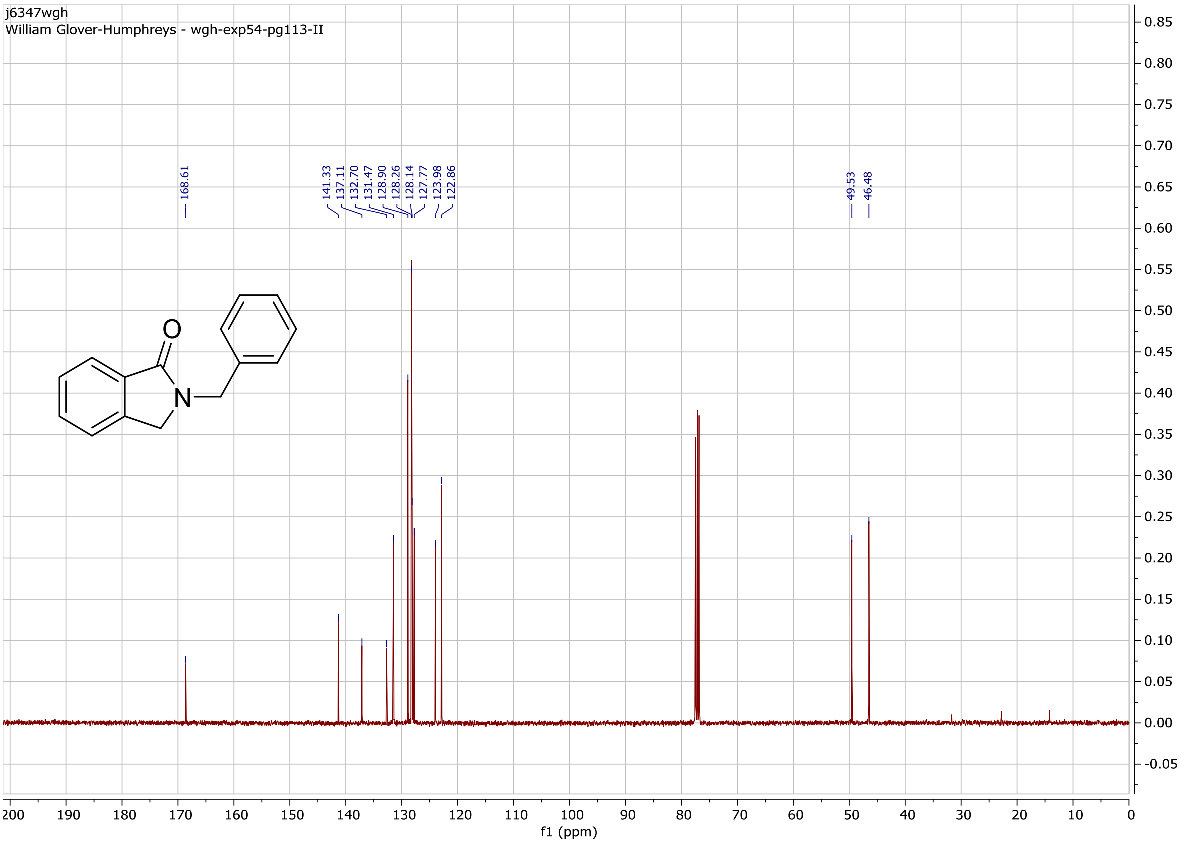

tert-Butyl (3-(phenyl(2-(phenylamino)ethyl)amino)propyl)carbamate (107a)

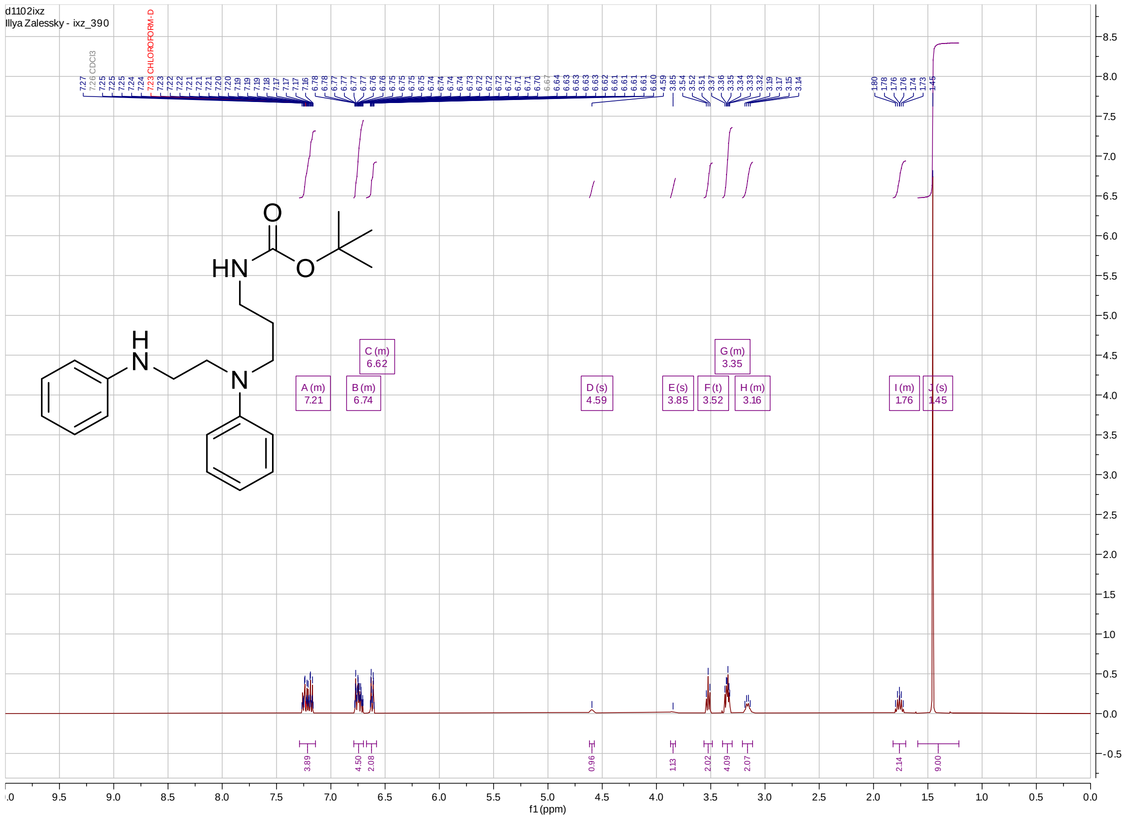

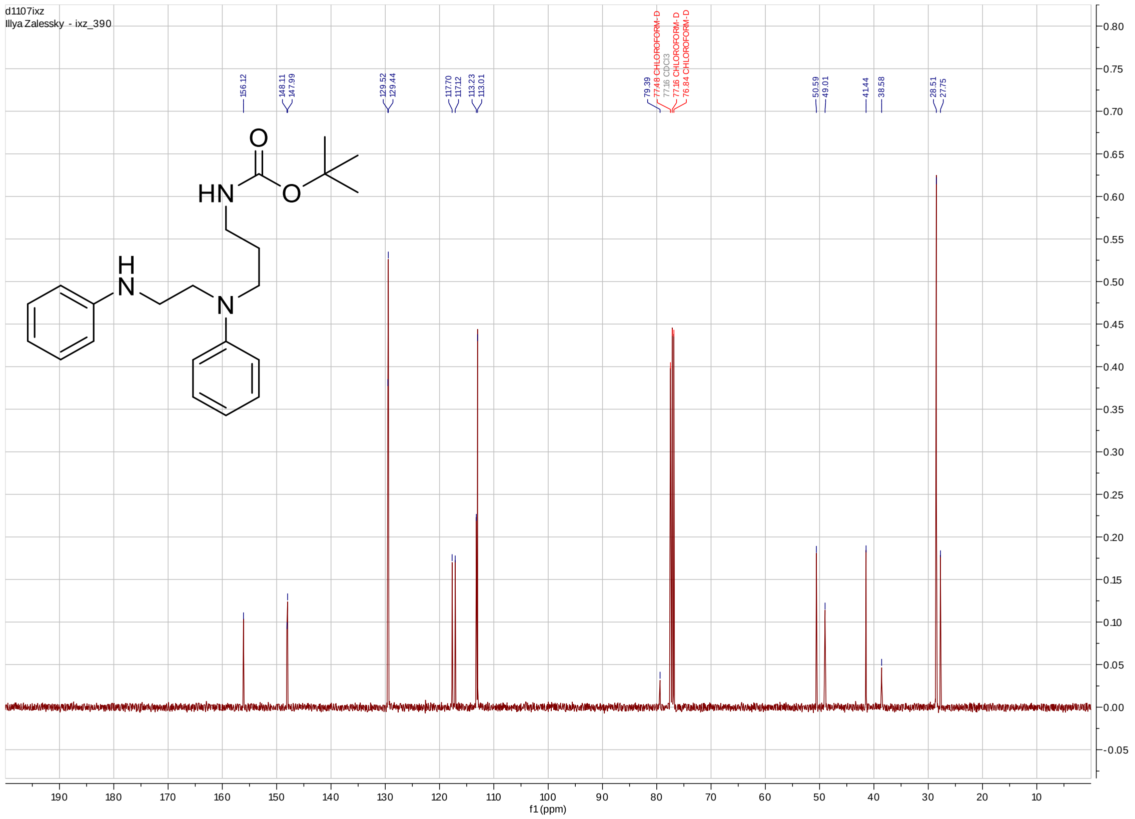

4-(Phenyl(2-(phenylamino)ethyl)amino)butan-1-ol (107b)

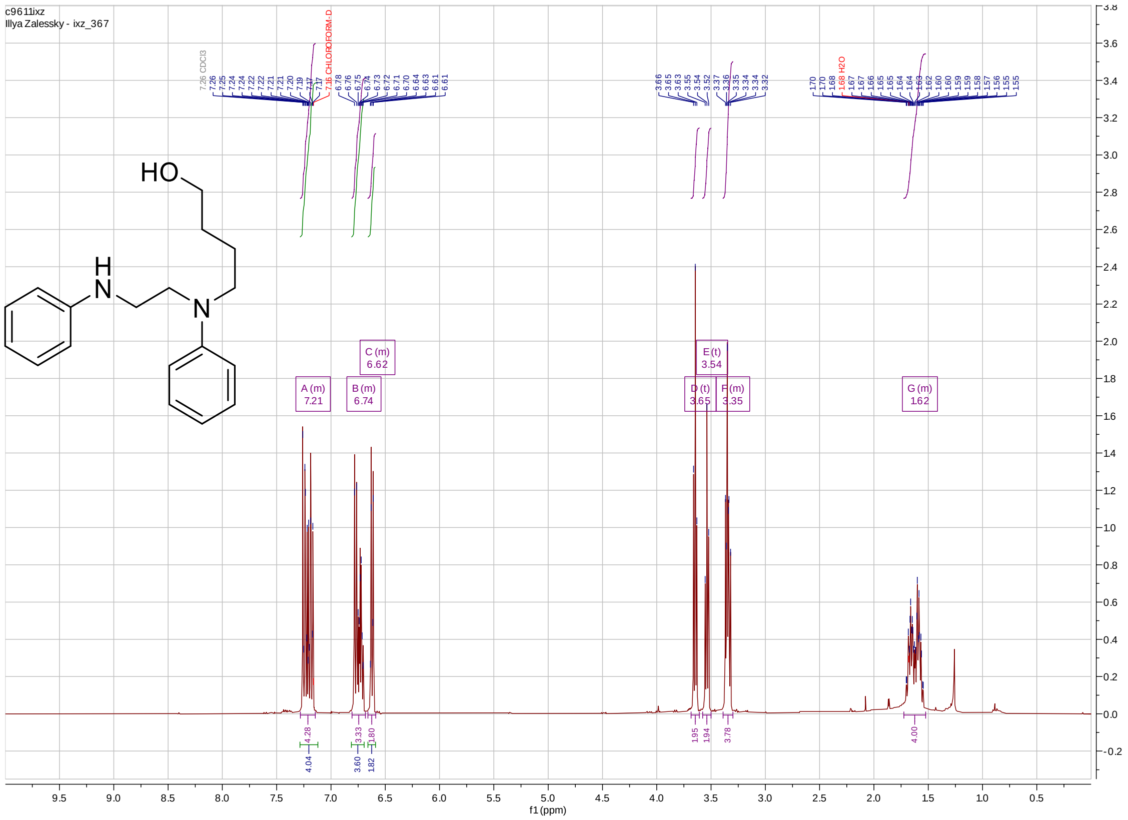

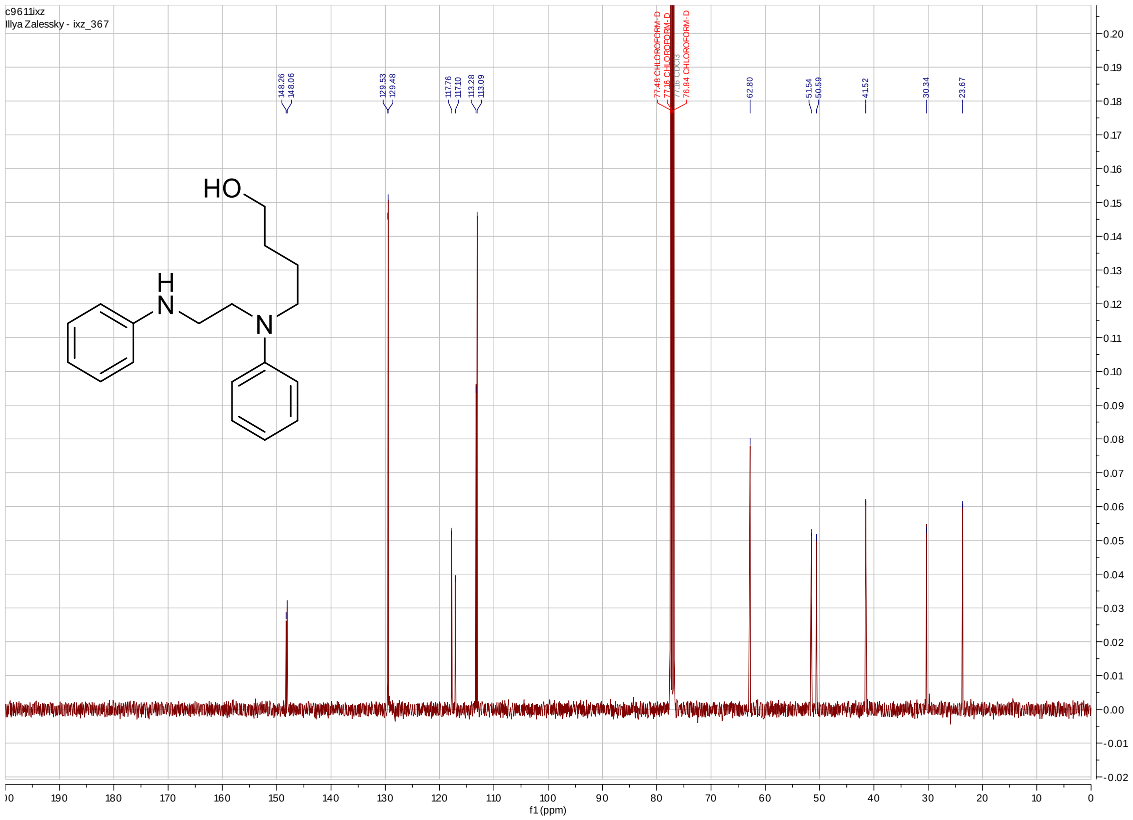

1,3-Diphenylimidazolidine-2-thione (109b)

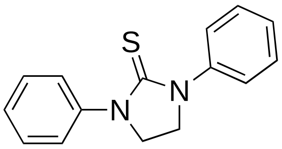

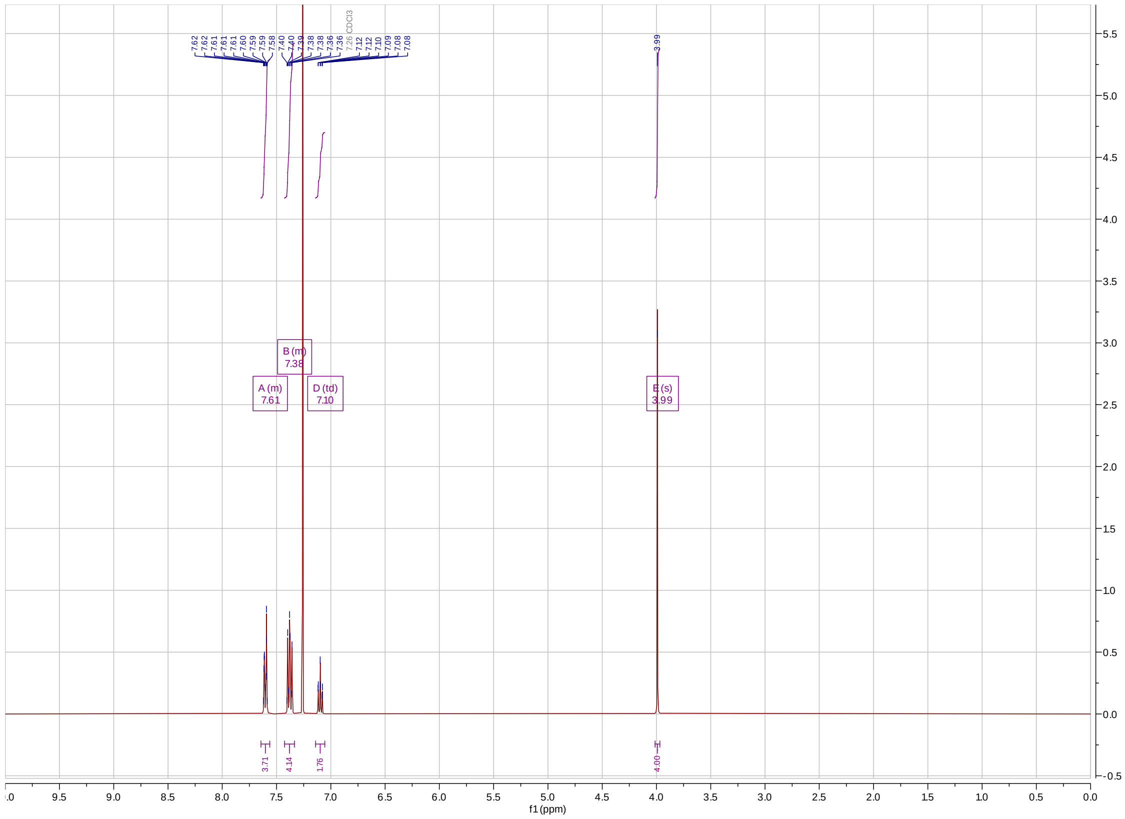

3-{[2-(6-Bromopyridin-2-yl)-1-phenylethyl](methyl)amino}propan-1-ol (S143)

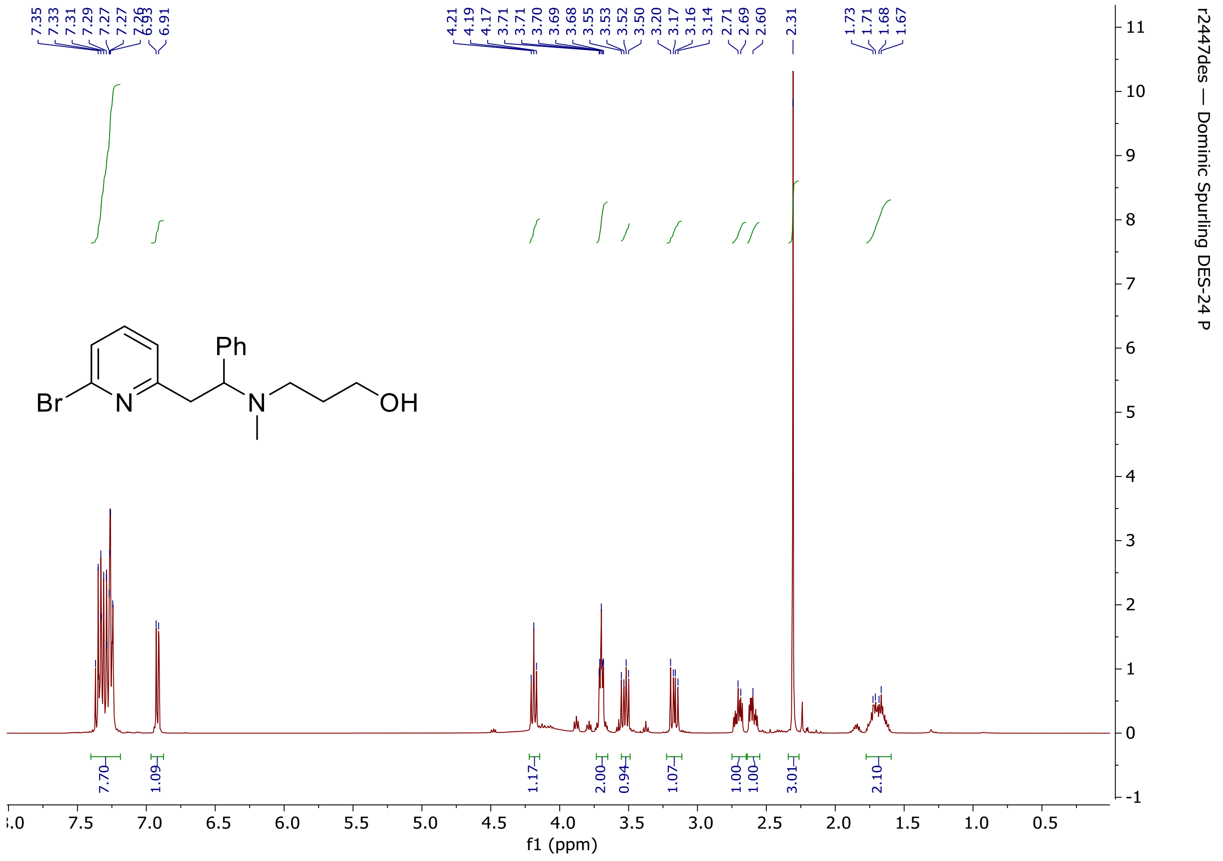

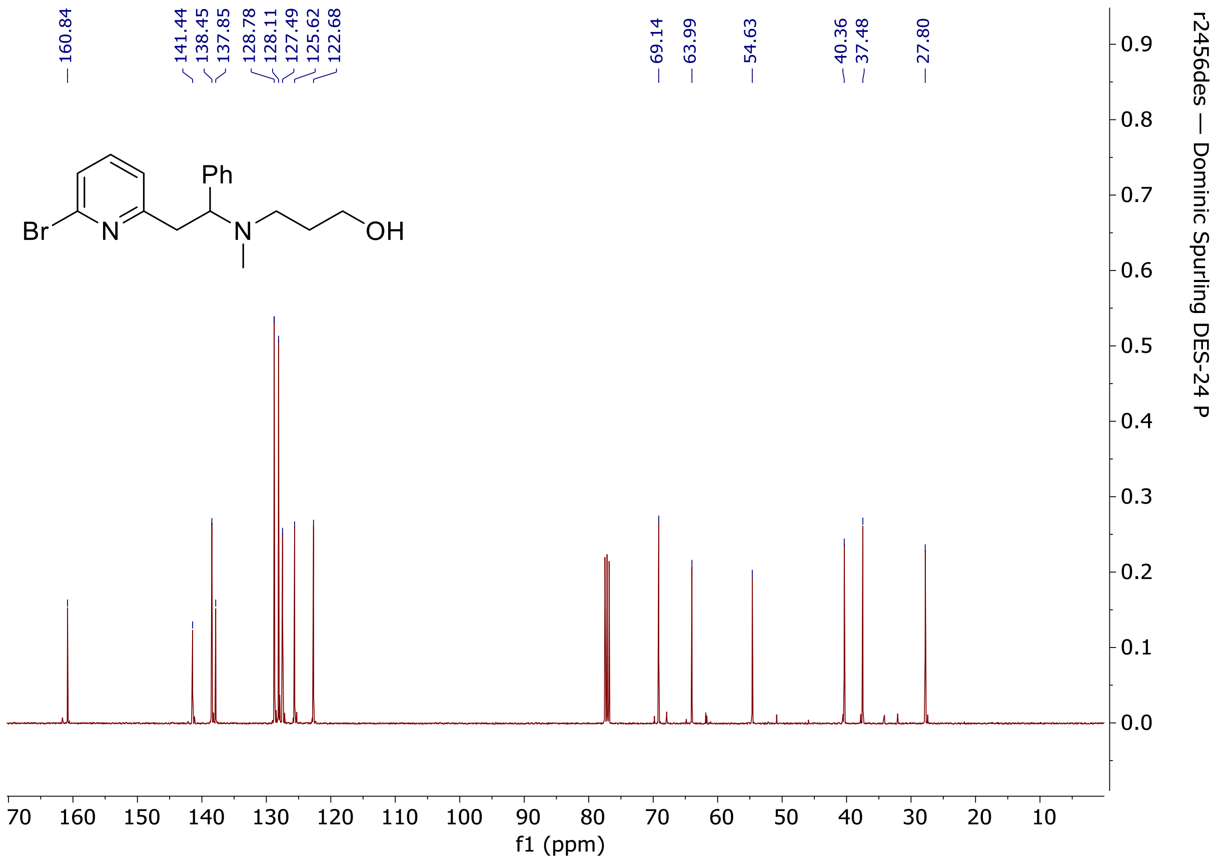

Methyl 2-[2-(6-{2-[(3-hydroxypropyl)(methyl)amino]-2-phenylethyl}pyridin-2-yl)phenyl]acetate (S144)

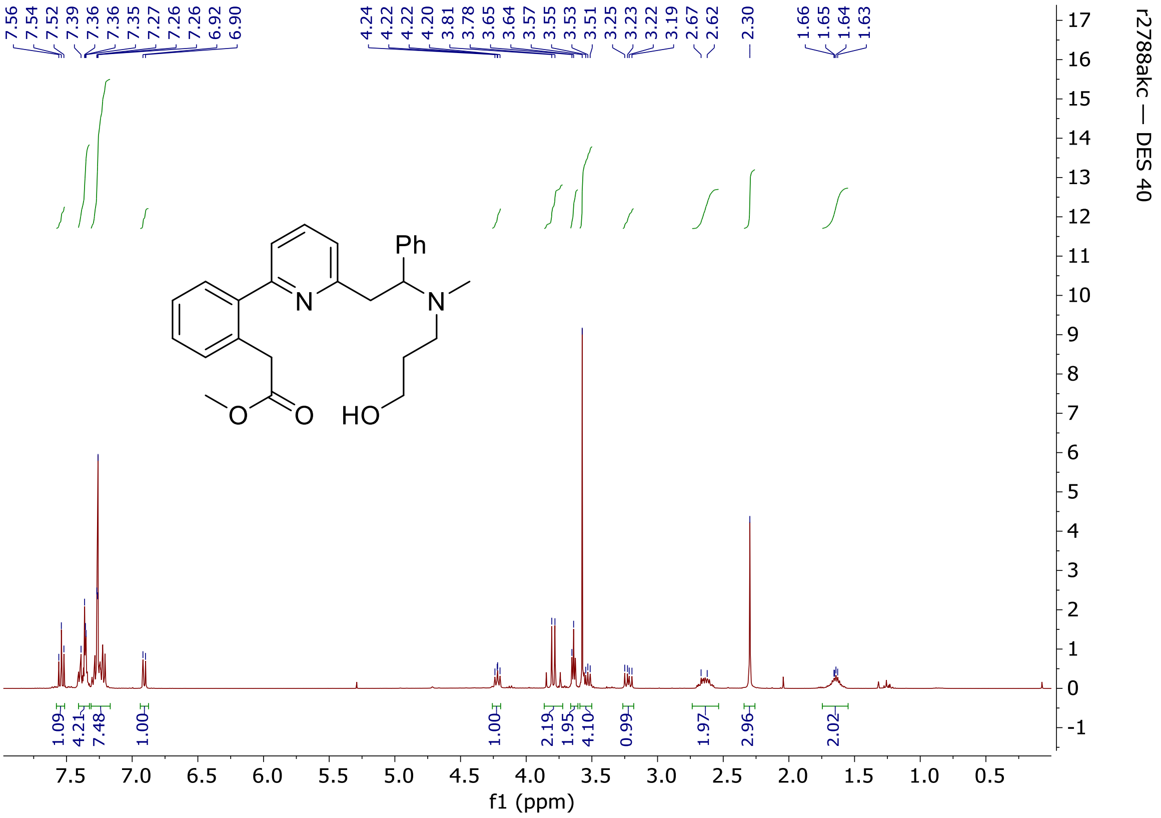

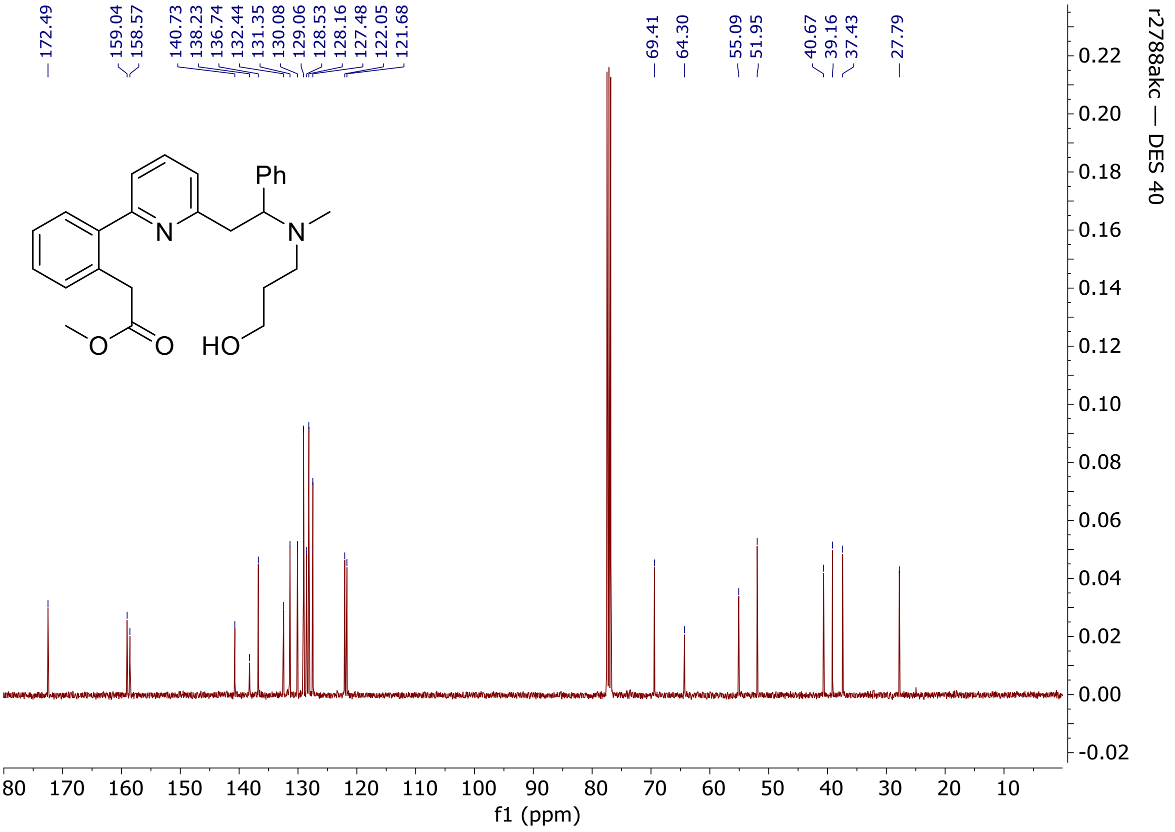

[2-(6-{2-[(3-Hydroxypropyl)(methyl)amino]-2-phenylethyl}pyridin-2-yl)phenyl]acetic acid (114)

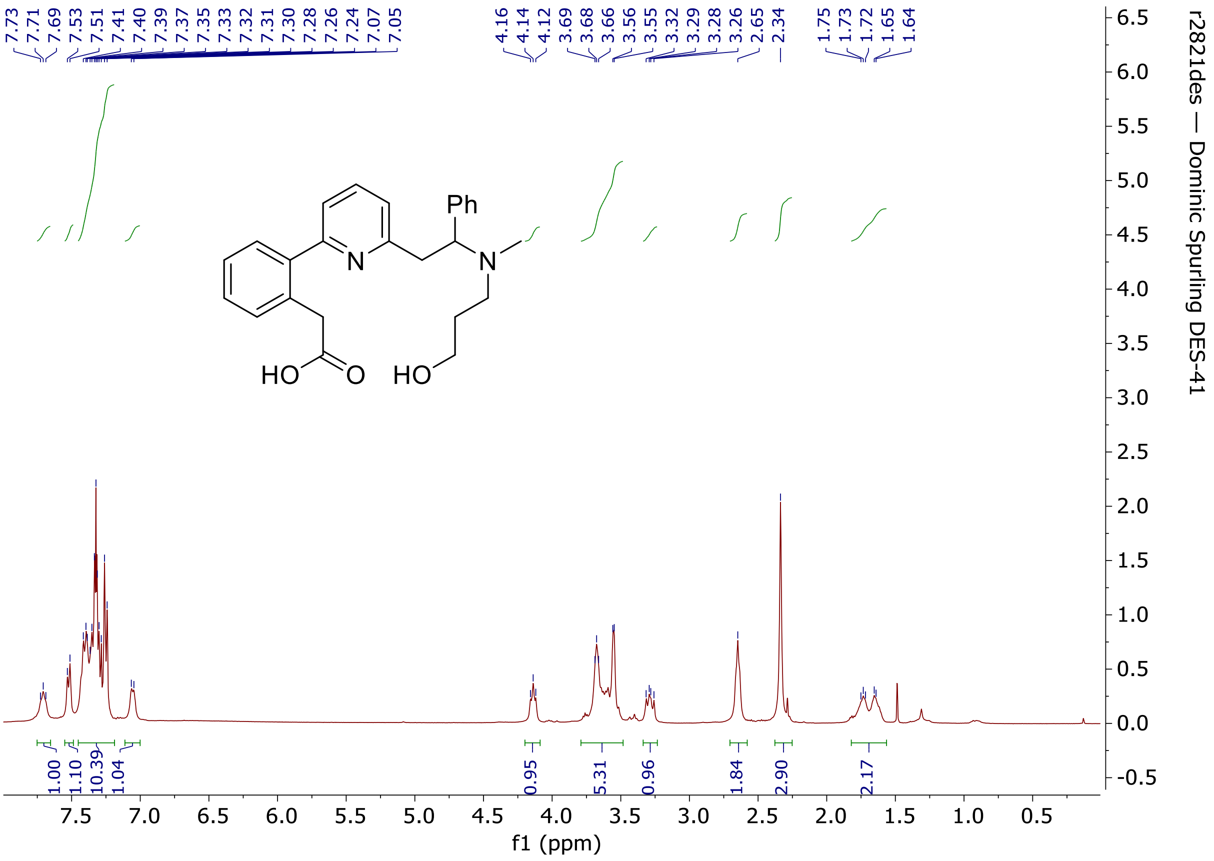

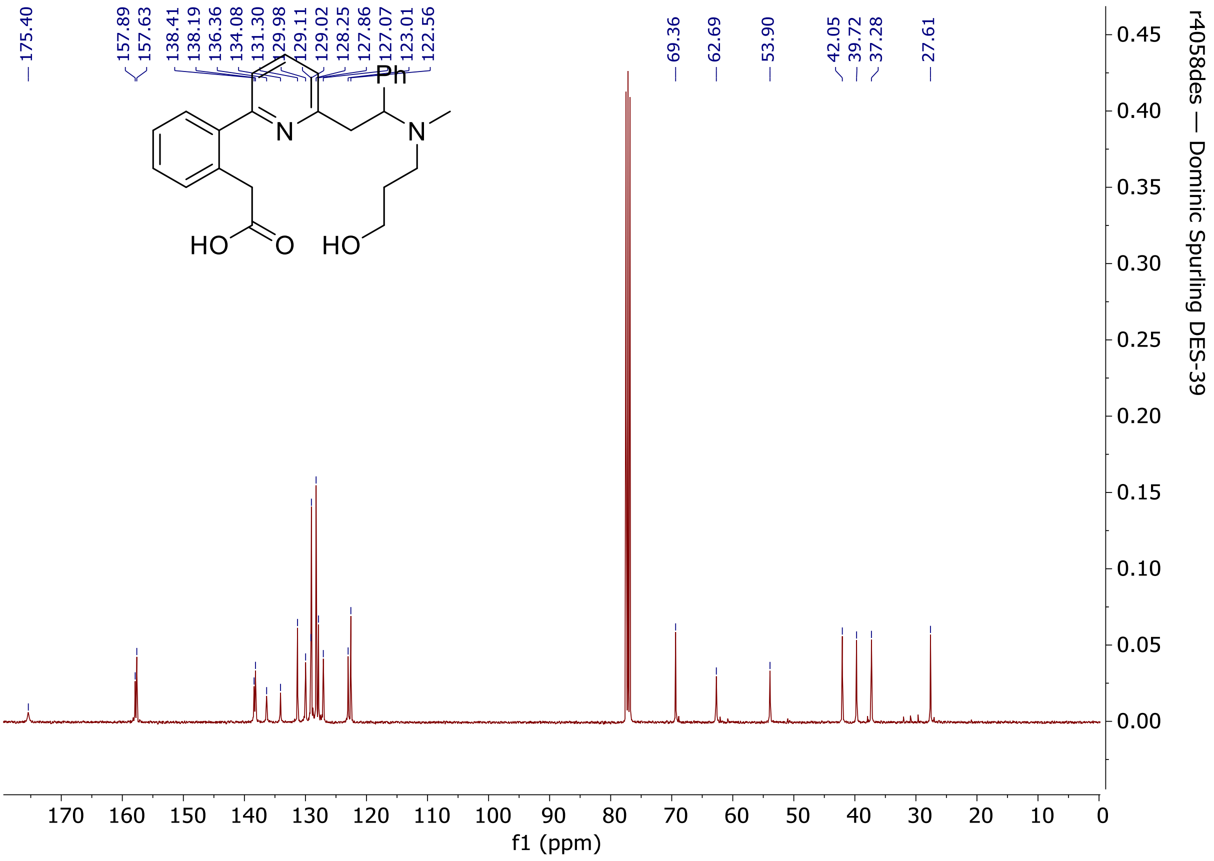

14-Methyl-15-phenyl-10-oxa-14,21-diazatricyclo[15.3.1.0^{2,7}]henicosa-1(21),2,4,6,17,19-hexaen-9-one (117)

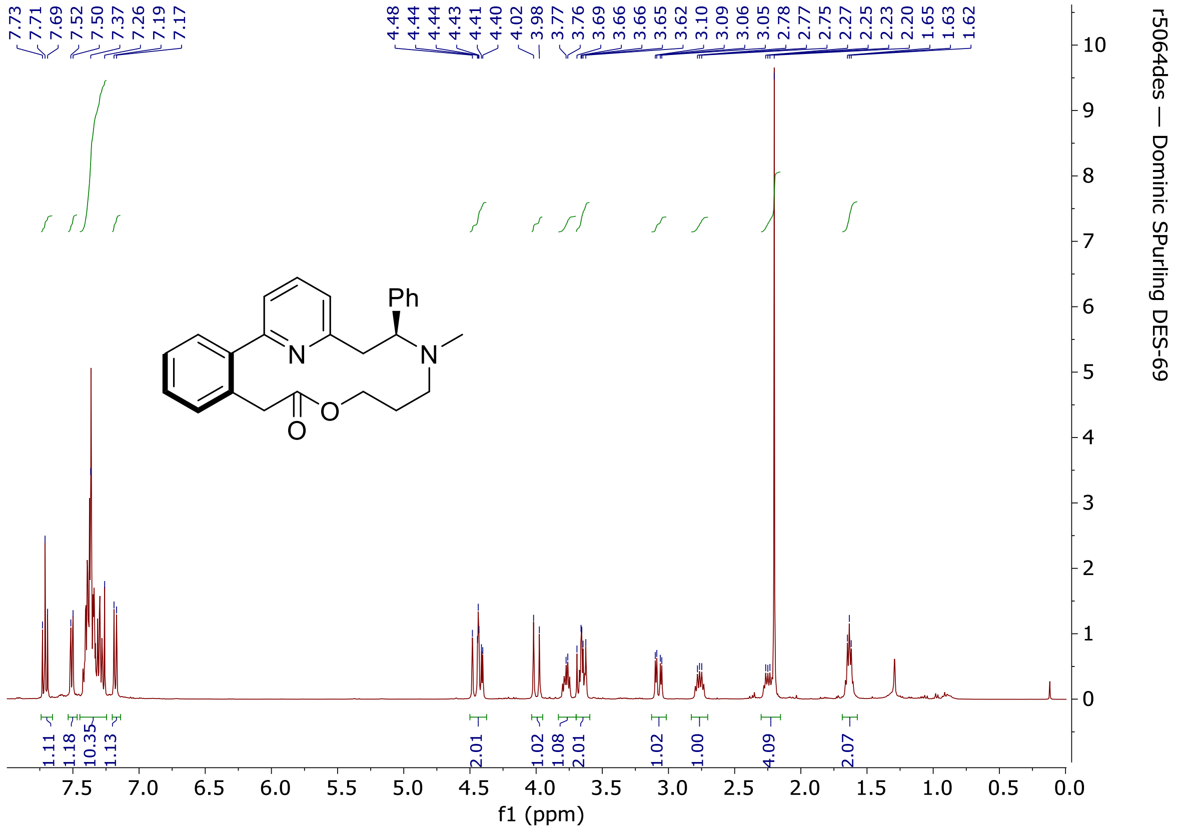

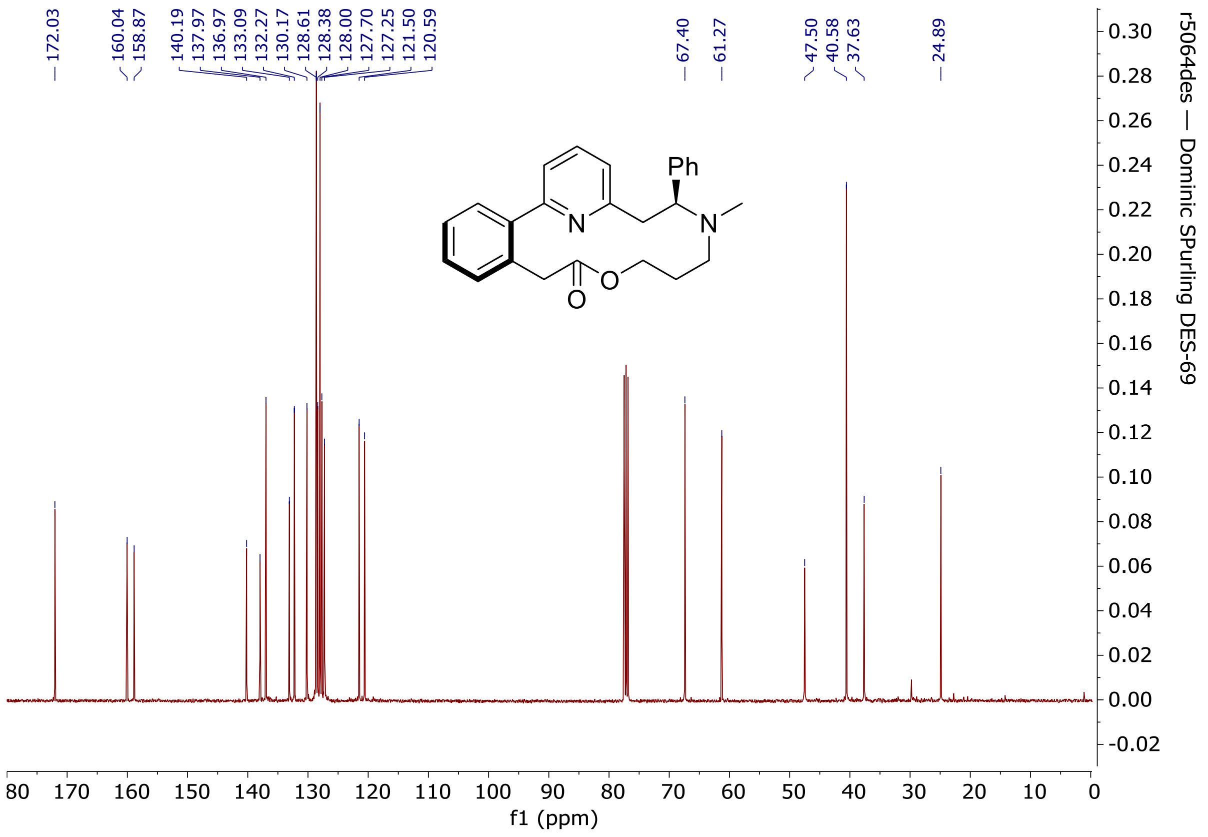

6-(3-Bromophenyl)hex-5-yn-1-ol (S145)

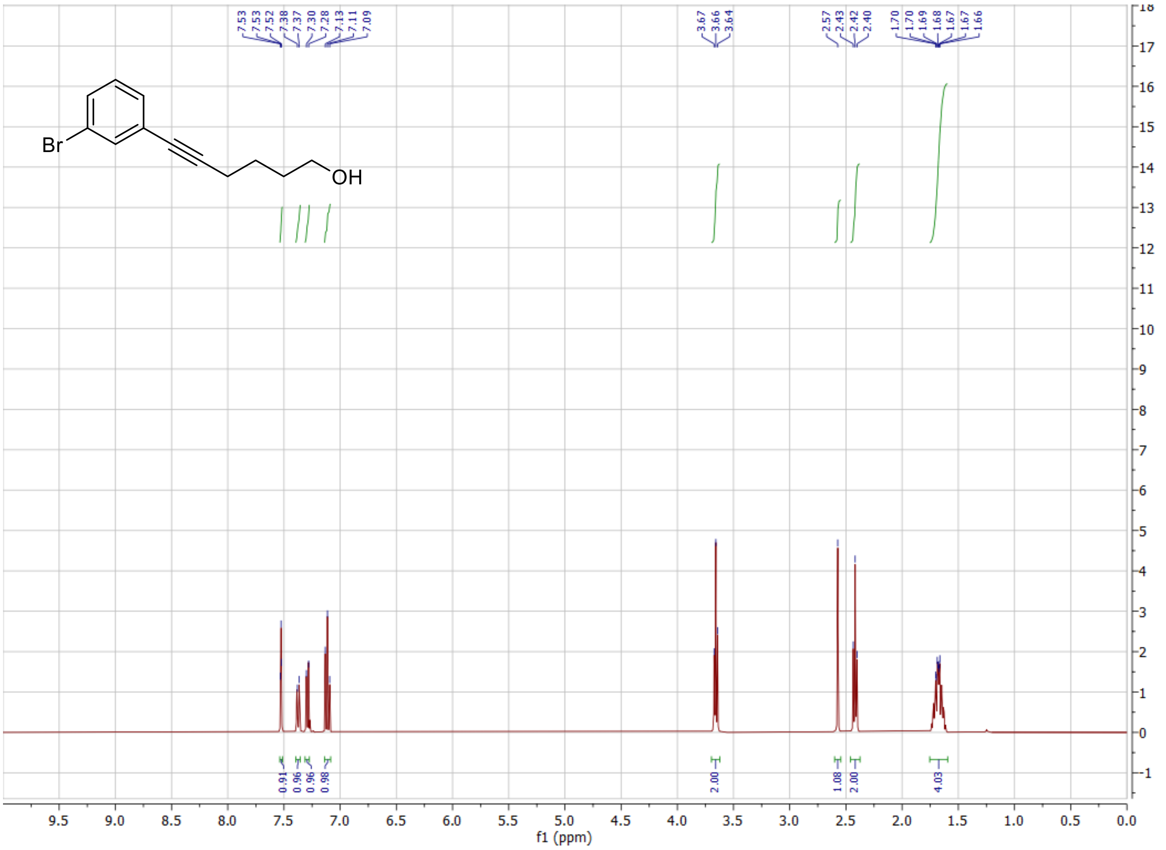

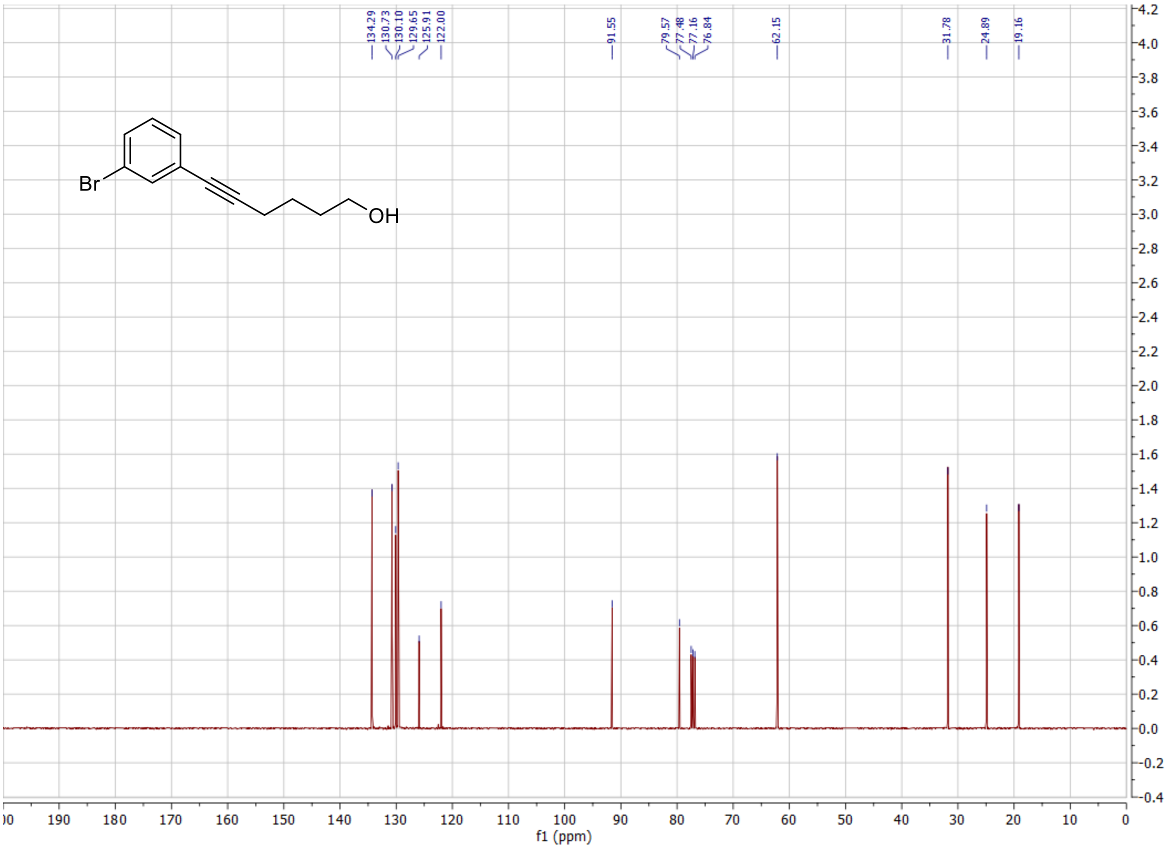

Methyl 2-(3'-(6-hydroxyhex-1-yn-1-yl)-[1,1'-biphenyl]-2-yl)acetate (S146)

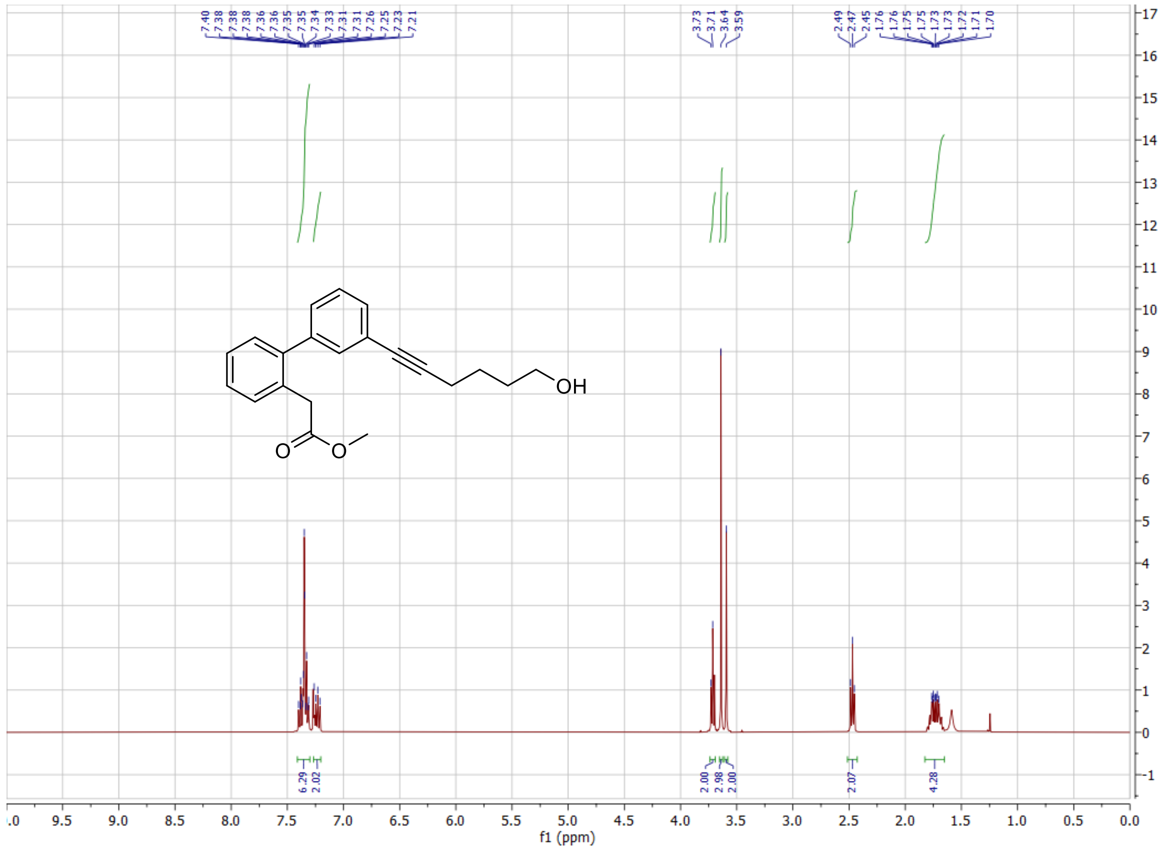

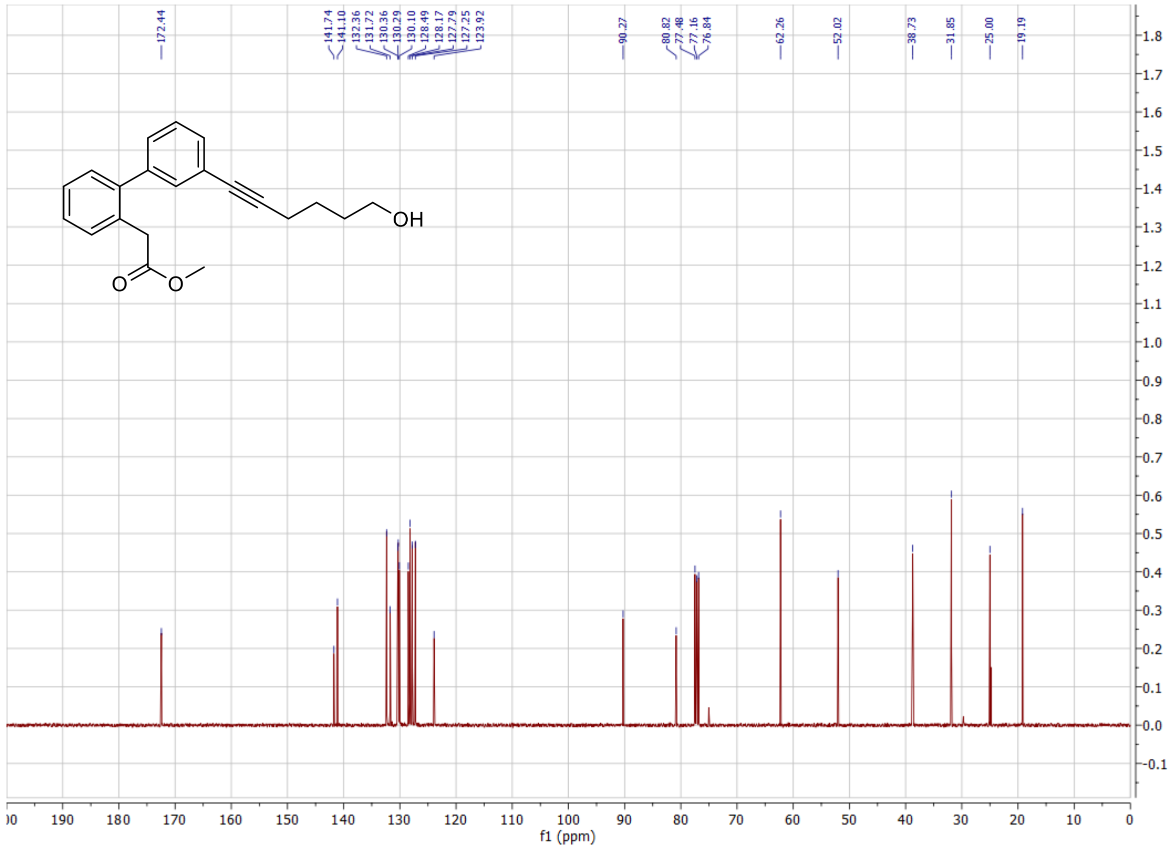

Methyl 2-(3'-(6-hydroxyhexyl)-[1,1'-biphenyl]-2-yl)acetate (S147)

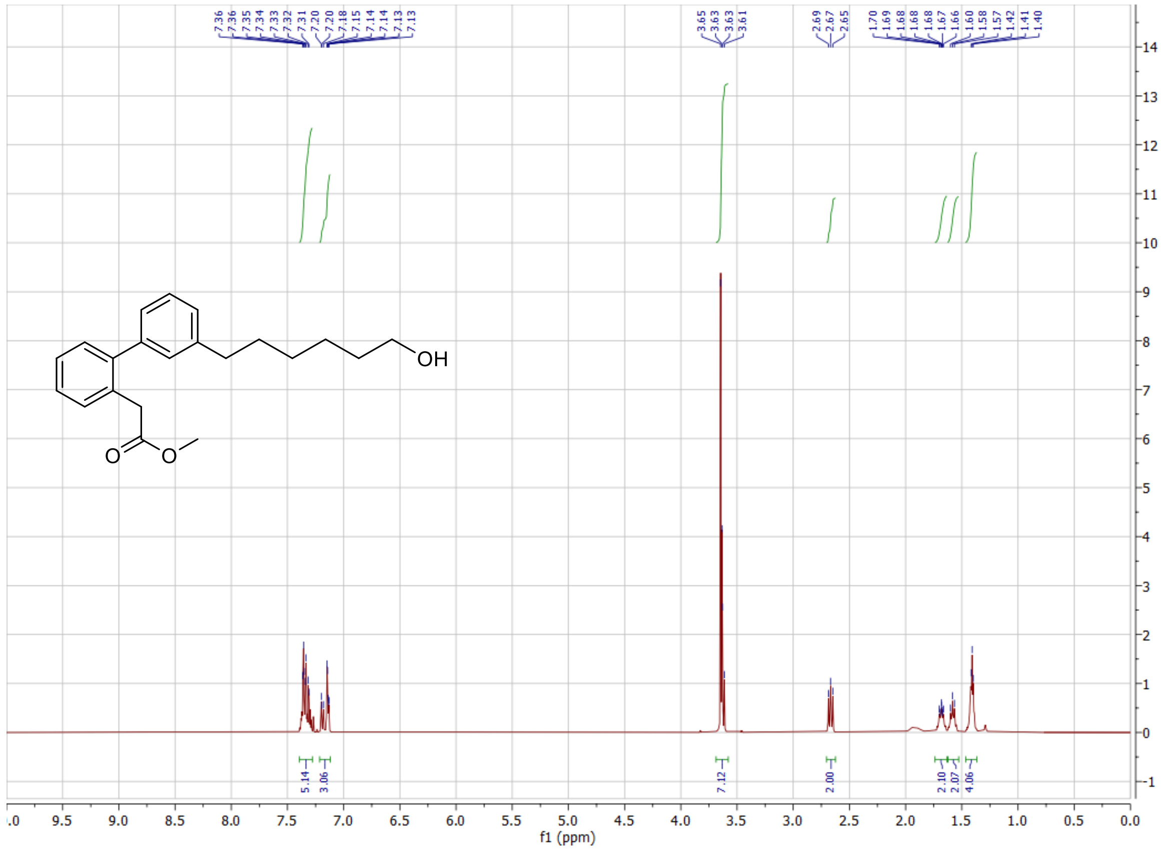

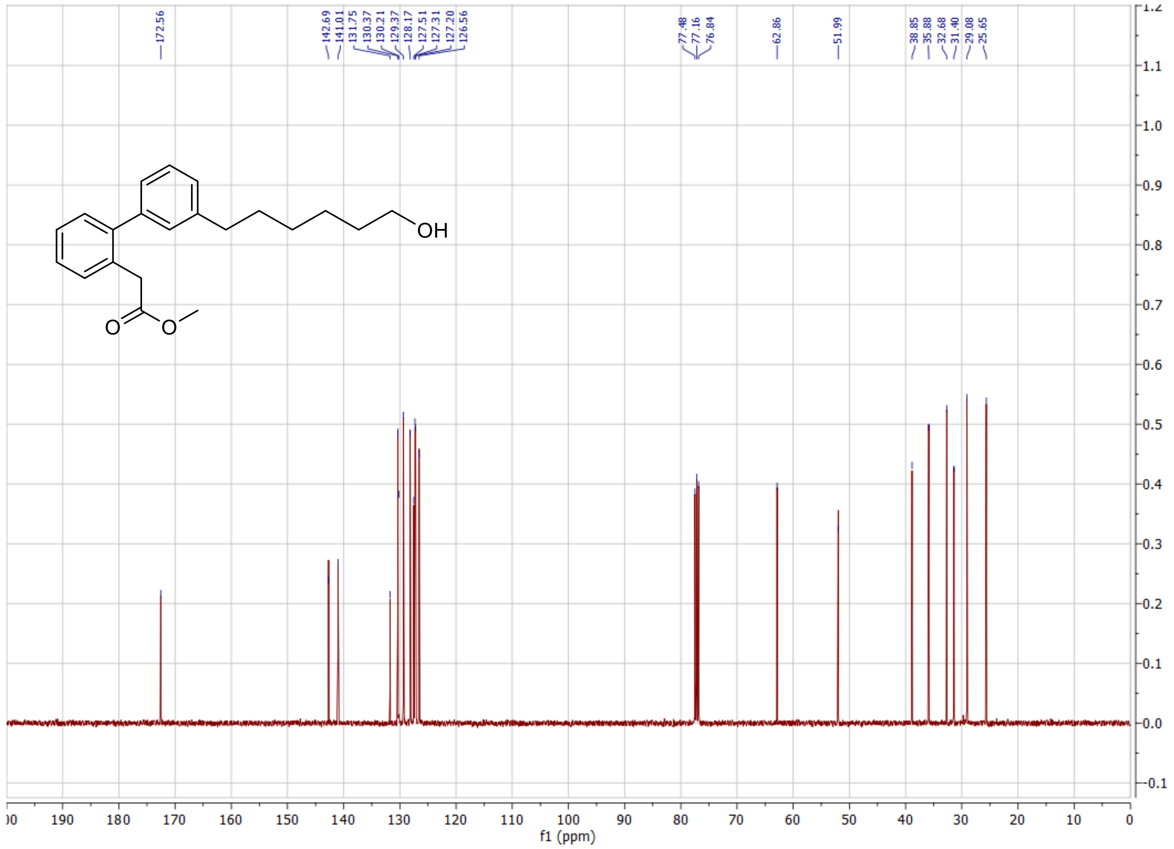

2-(3'-(6-Hydroxyhexyl)-[1,1'-biphenyl]-2-yl)acetic acid (114a)

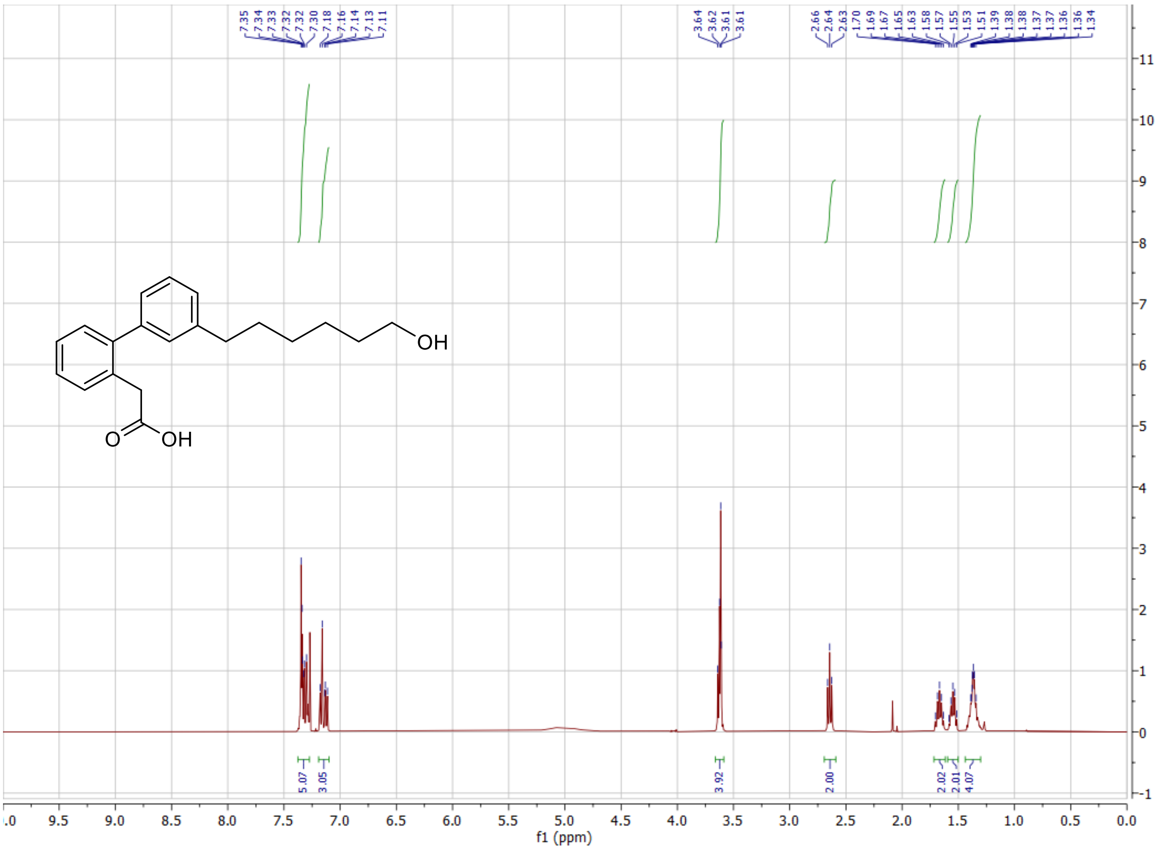

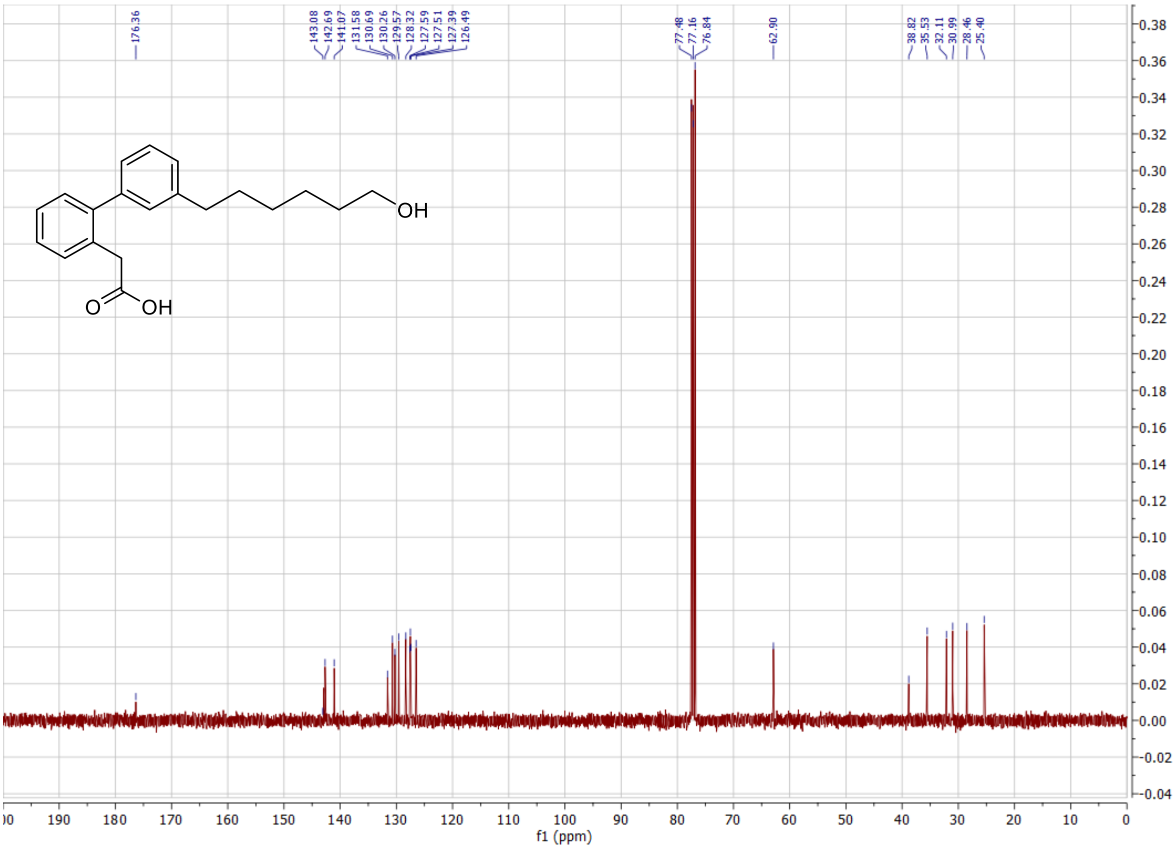

5-Oxa-1(1,3),2(1,2)-dibenzenacycloundecaphan-4-one (117a)

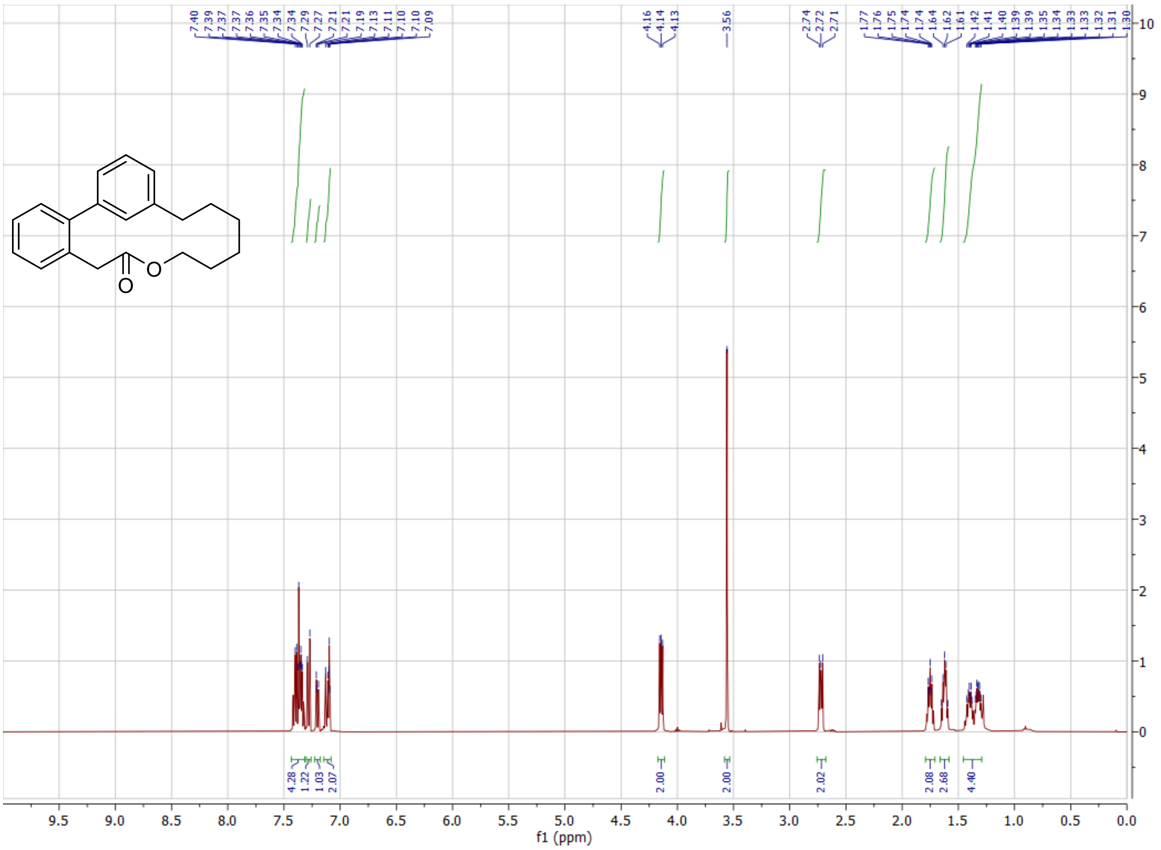

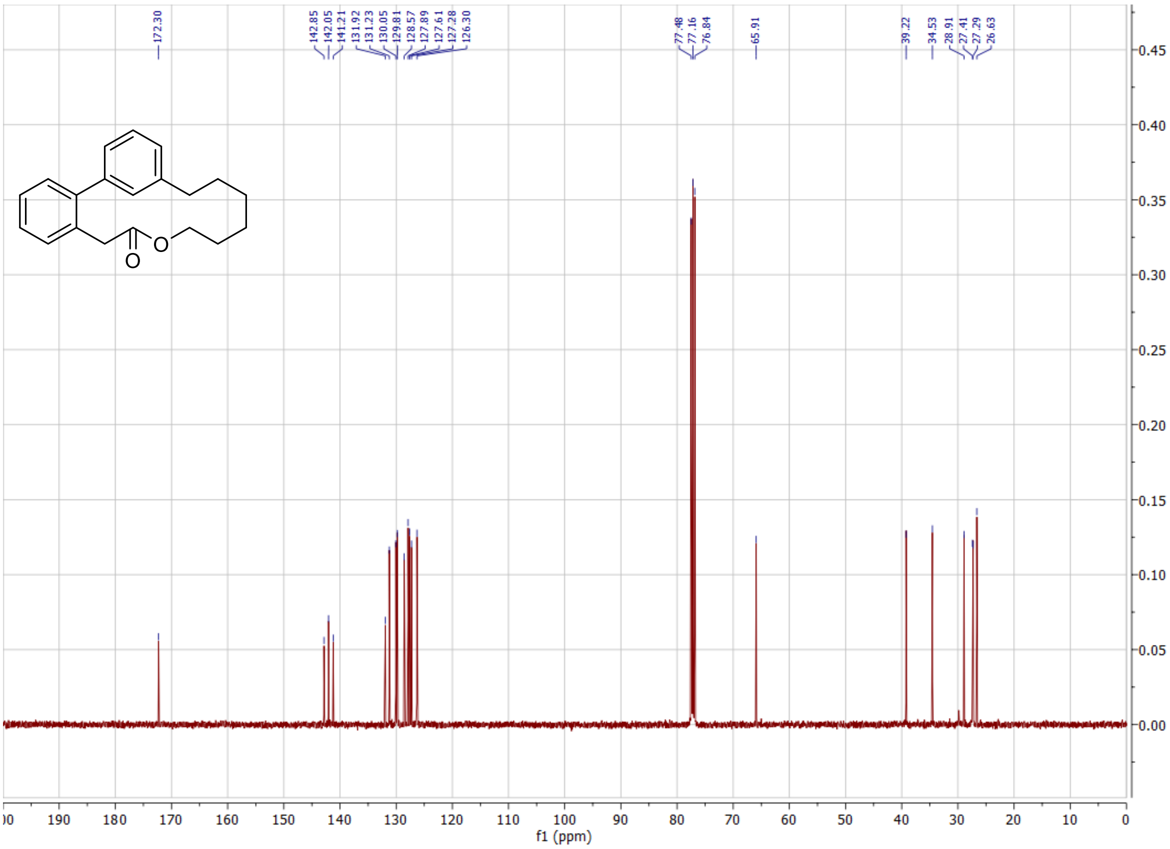

5,16-Dioxa-1,12(1,3),2,13(1,2)-tetrabenzenacyclodocosaphane-4,15-dione (S148)

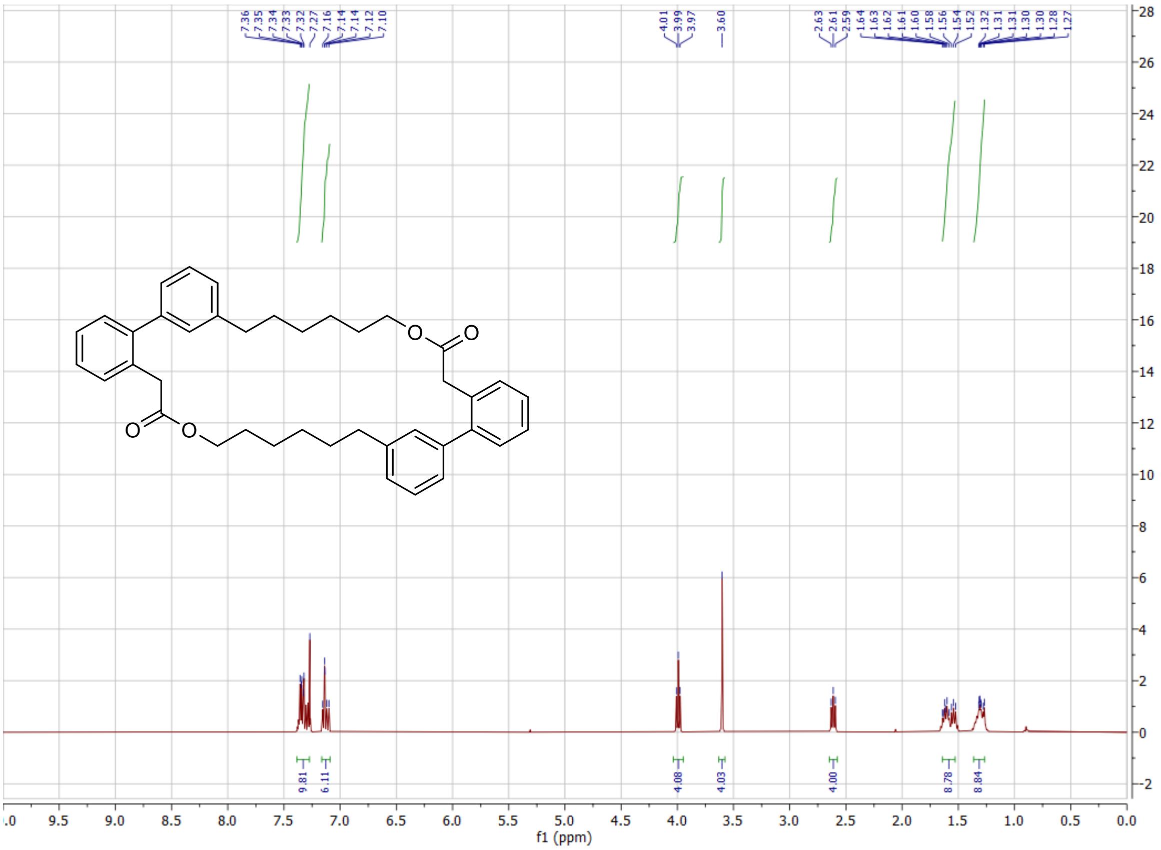

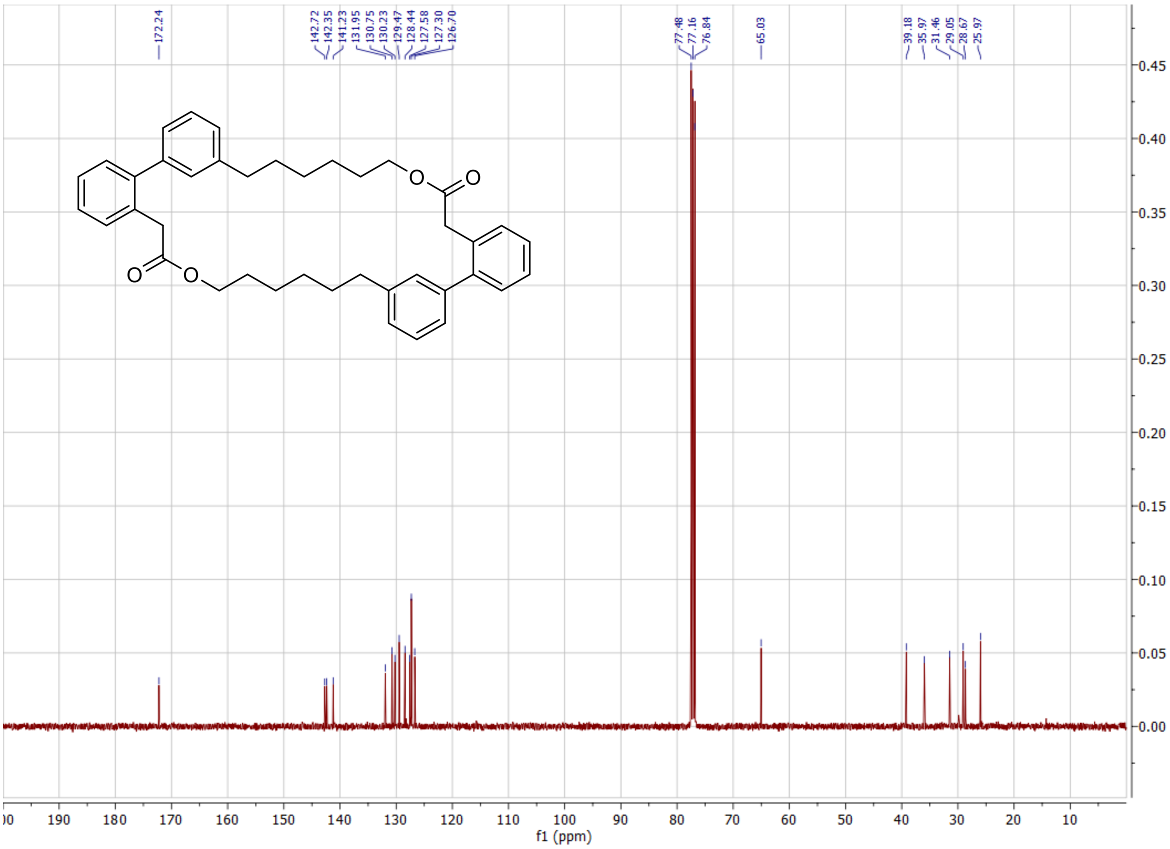

6-(6-Bromopyridin-2-yl)hex-5-yn-1-ol (S149)

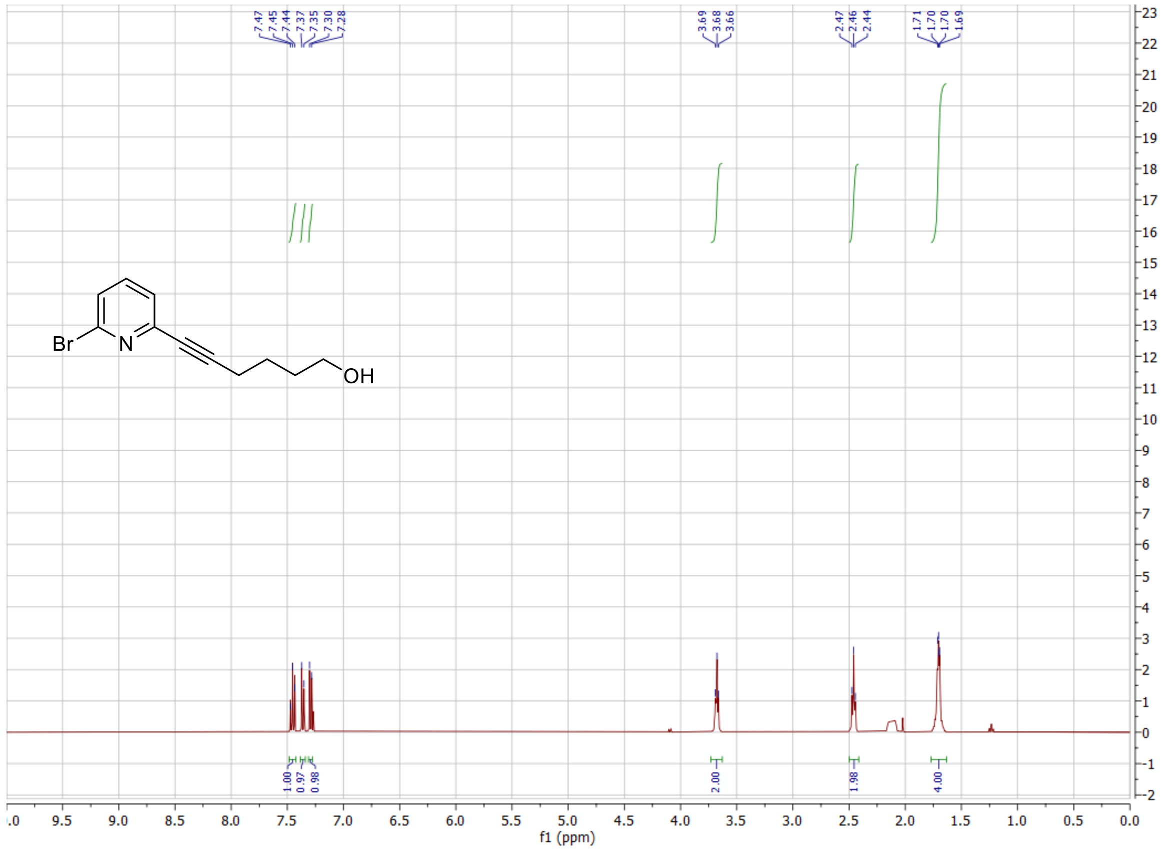

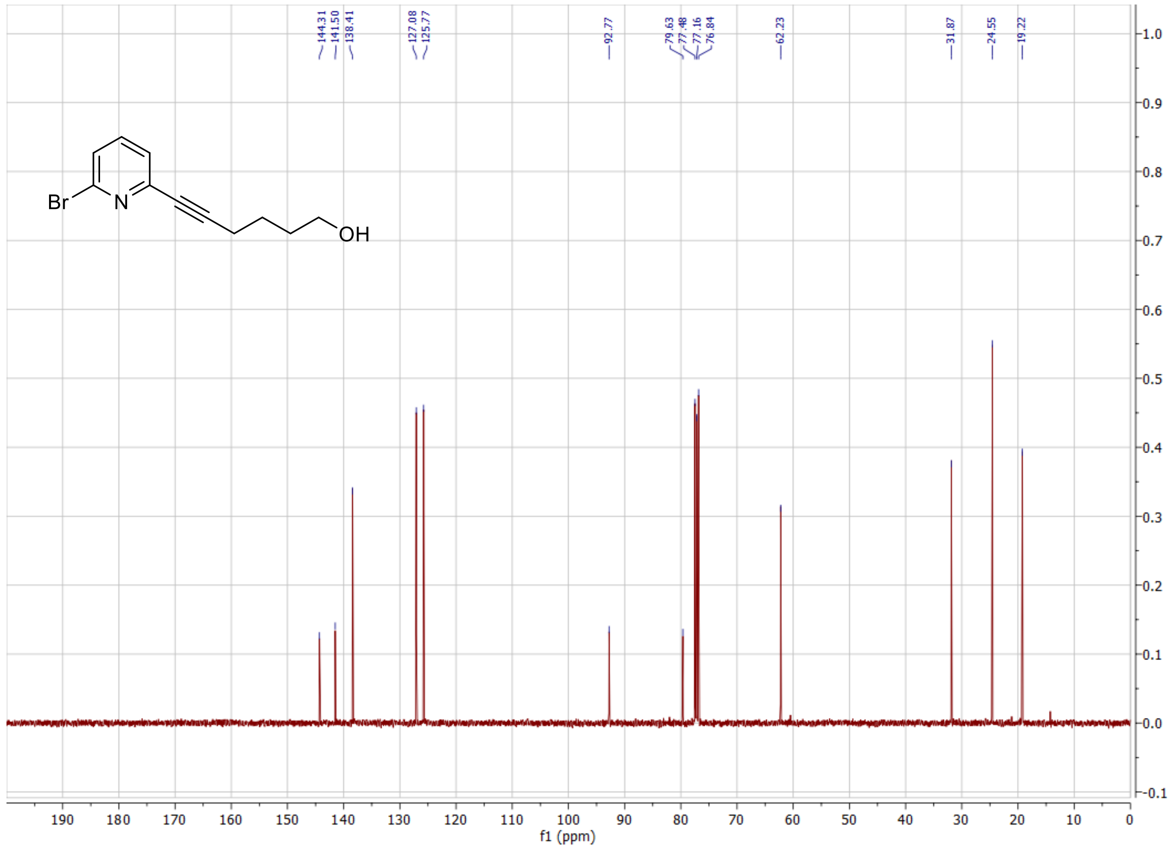

Methyl 2-(2-(6-(6-hydroxyhex-1-yn-1-yl)pyridin-2-yl)phenyl)acetate (S150)

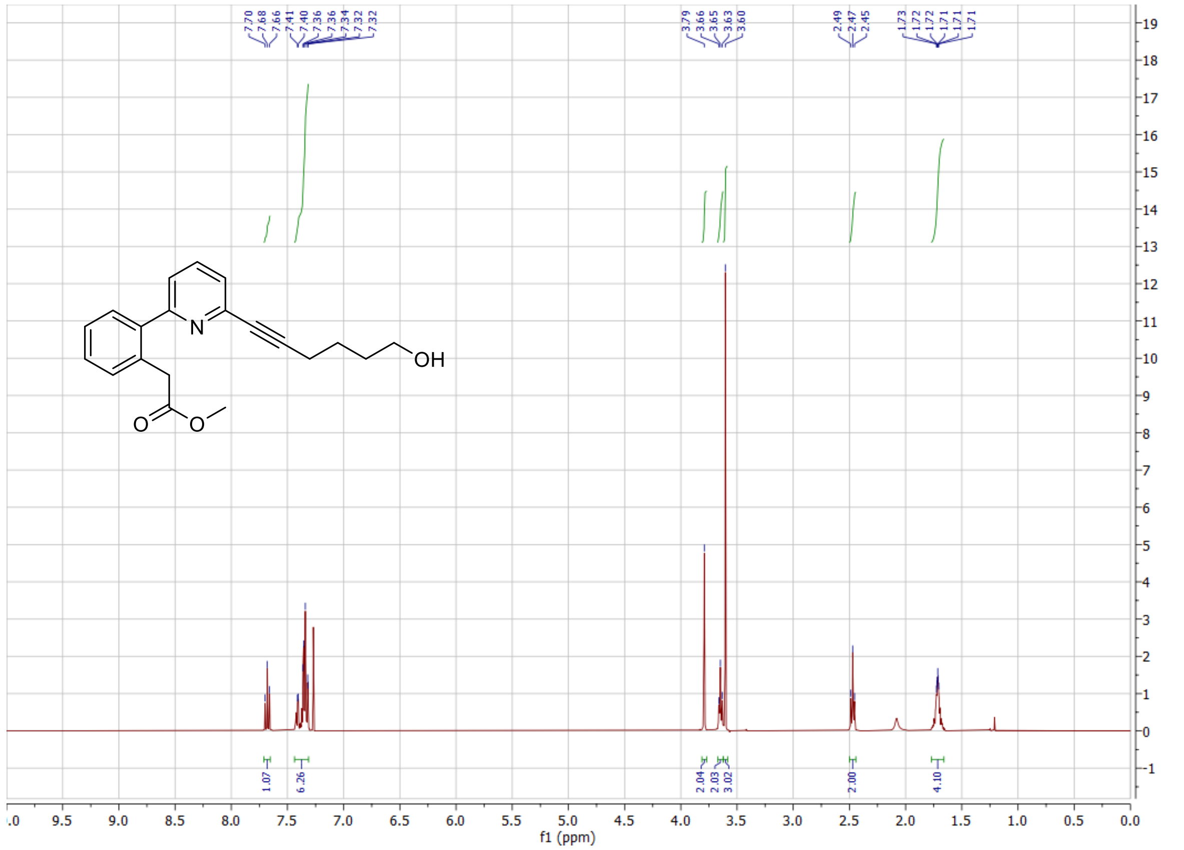

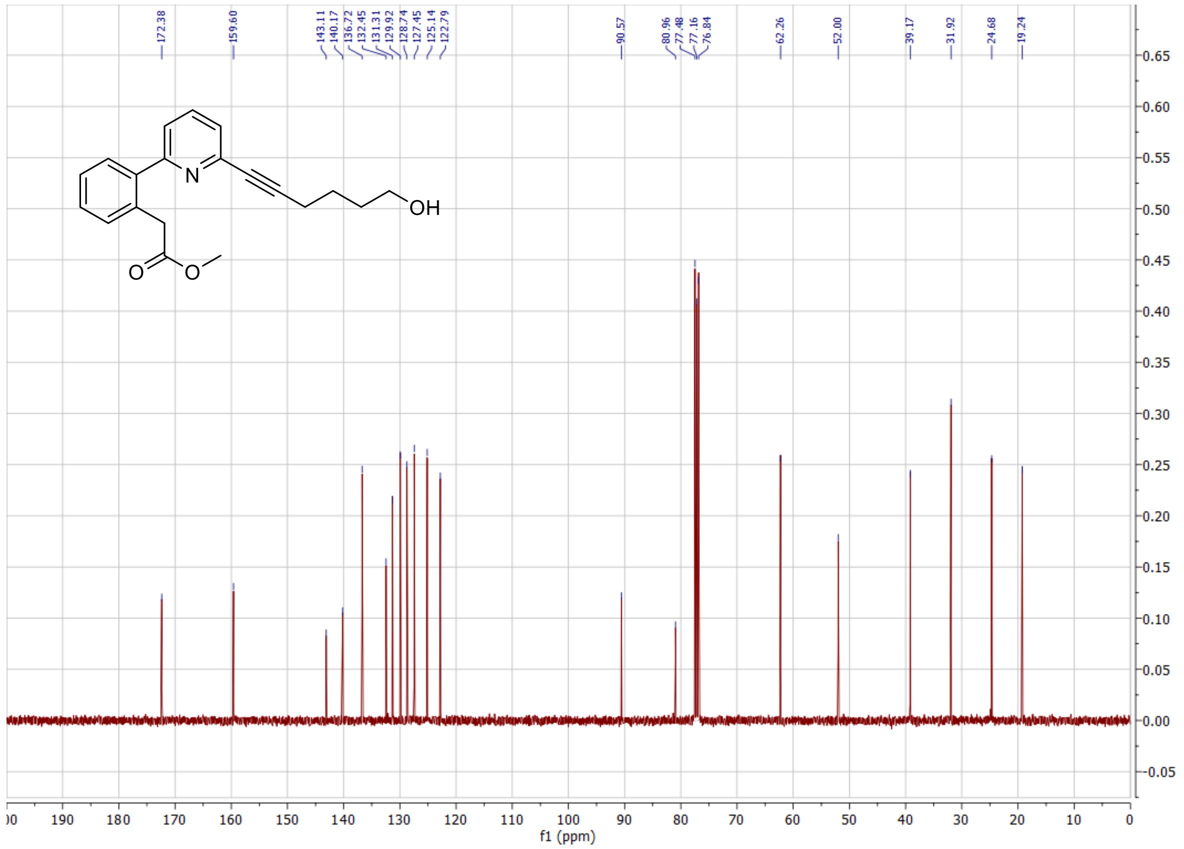

Methyl 2-(2-(6-(6-hydroxyhexyl)pyridin-2-yl)phenyl)acetate (S151)

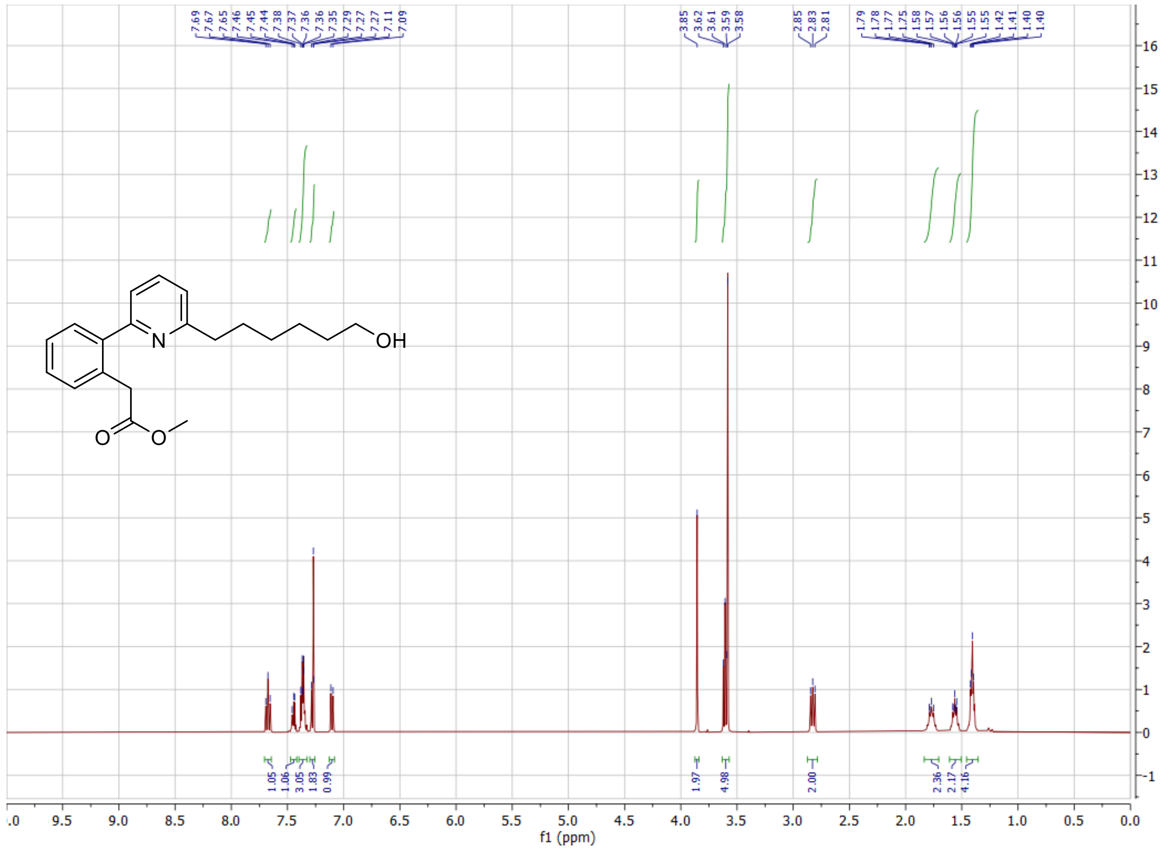

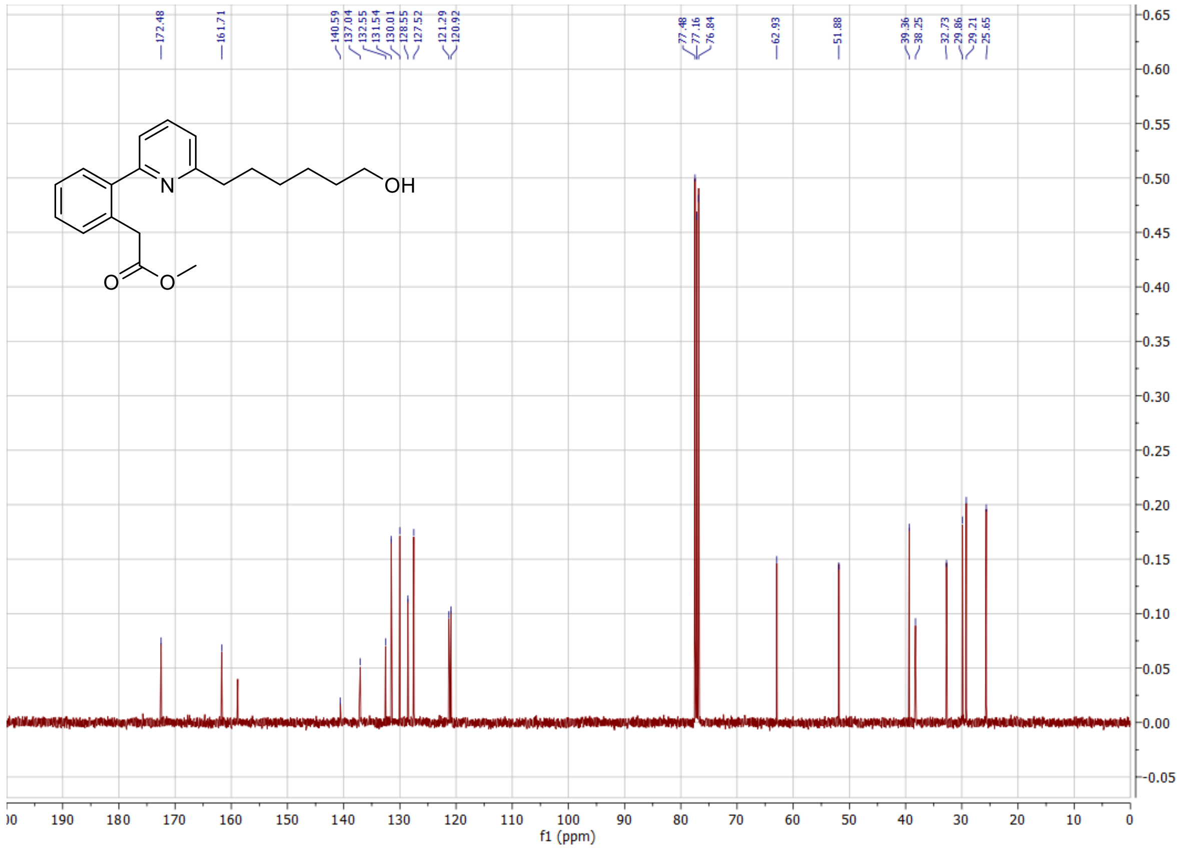

2-(2-(6-(6-Hydroxyhexyl)pyridin-2-yl)phenyl)acetic acid (114b)

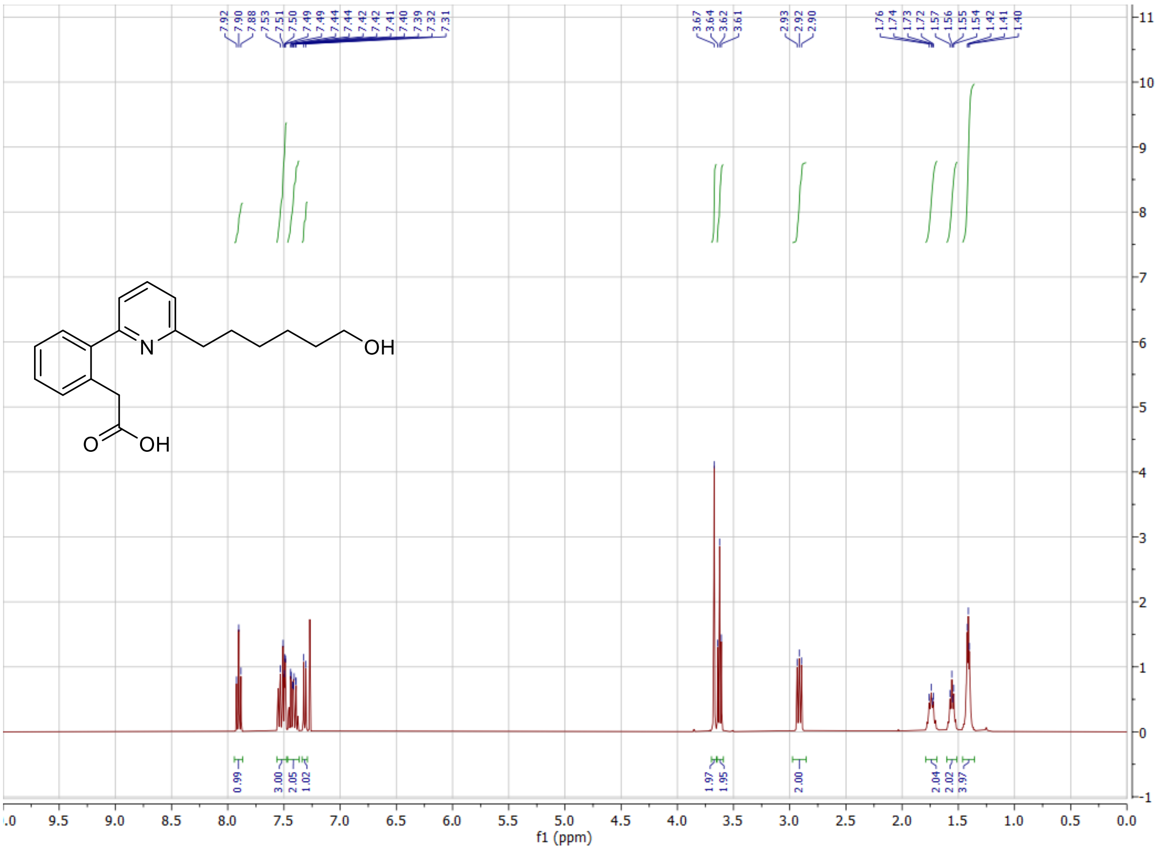

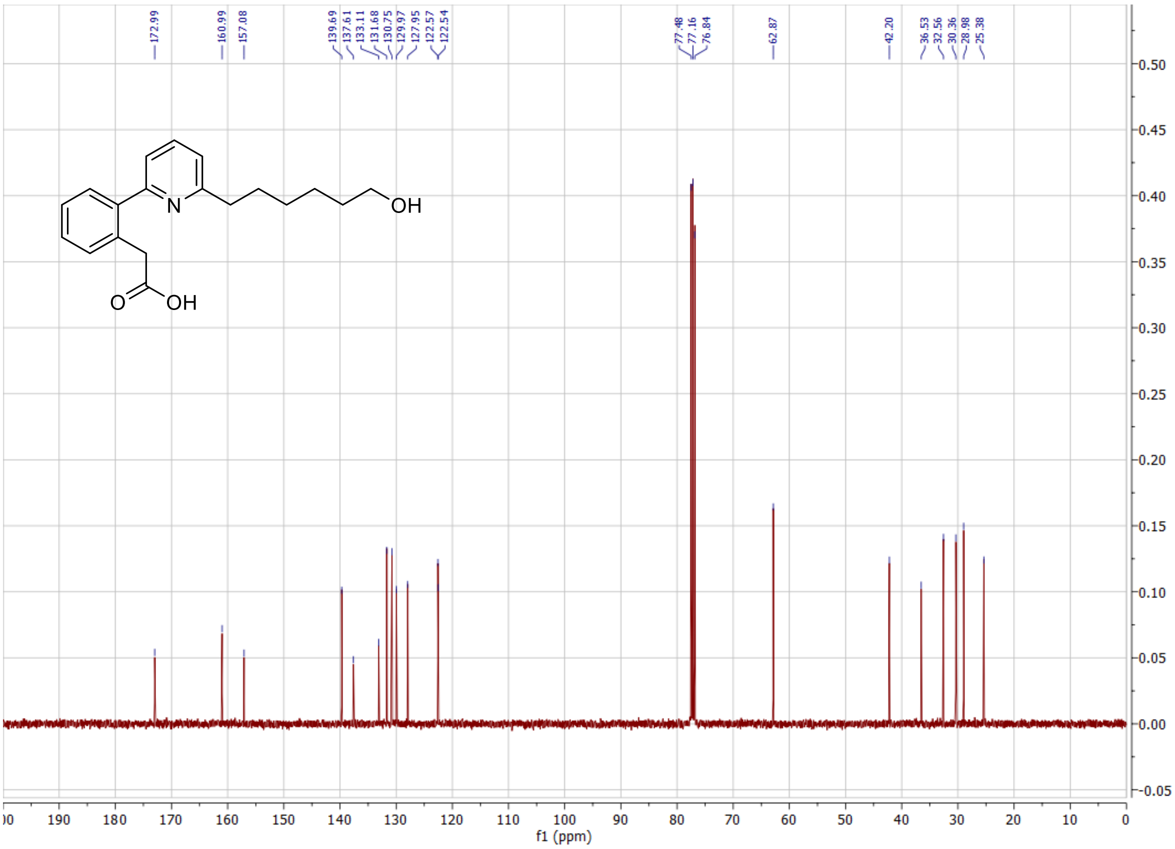

5-Oxa-1(2,6)-pyridina-2(1,2)-benzenacycloundecaphan-4-one (117b)

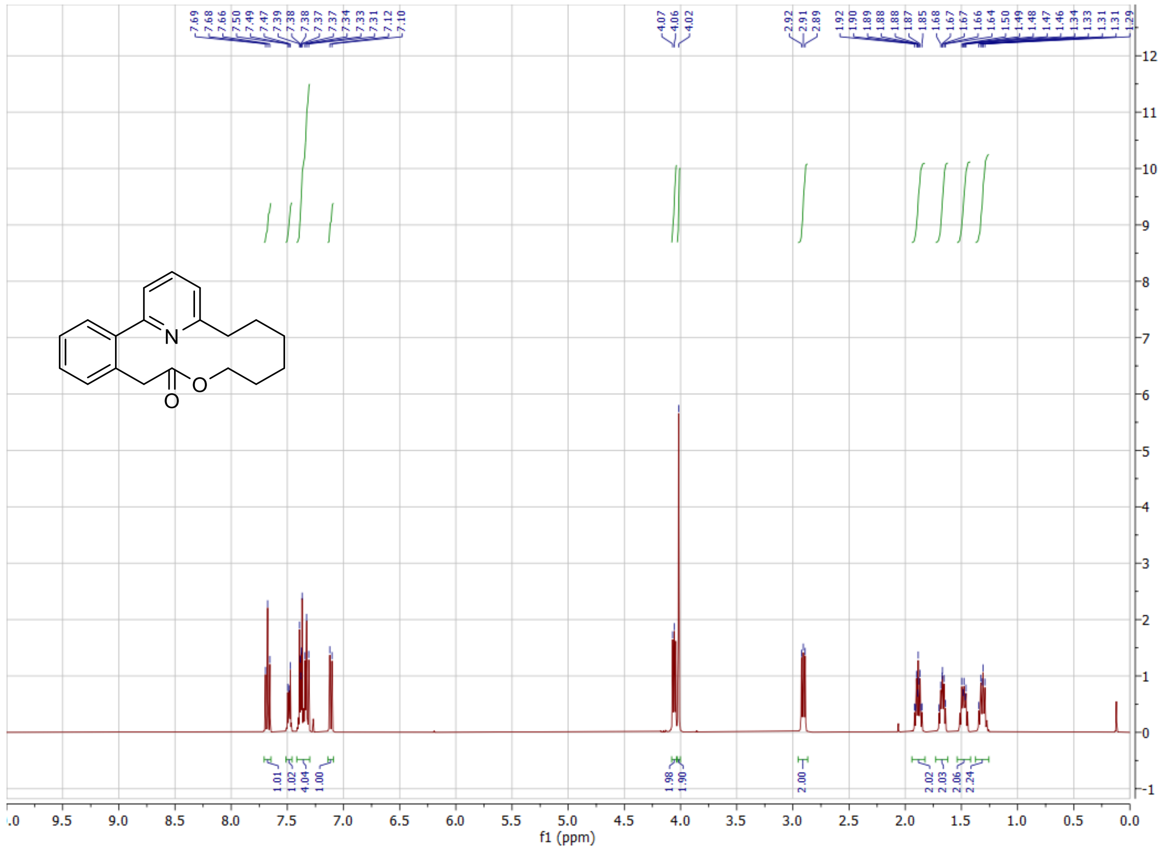

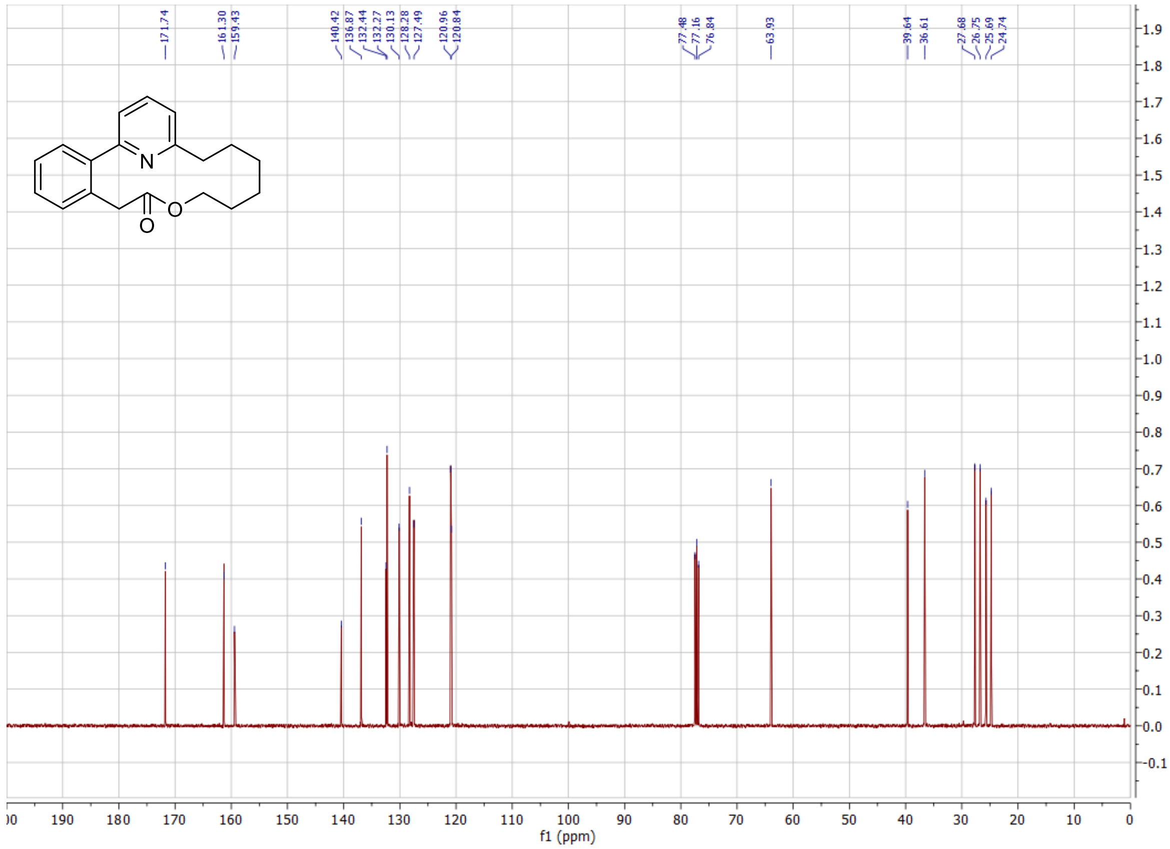

5,16-Dioxa-1,12(2,6)-dipyridina-2,13(1,2)-dibenzenacyclodocosaphane-4,15-dione (S152)

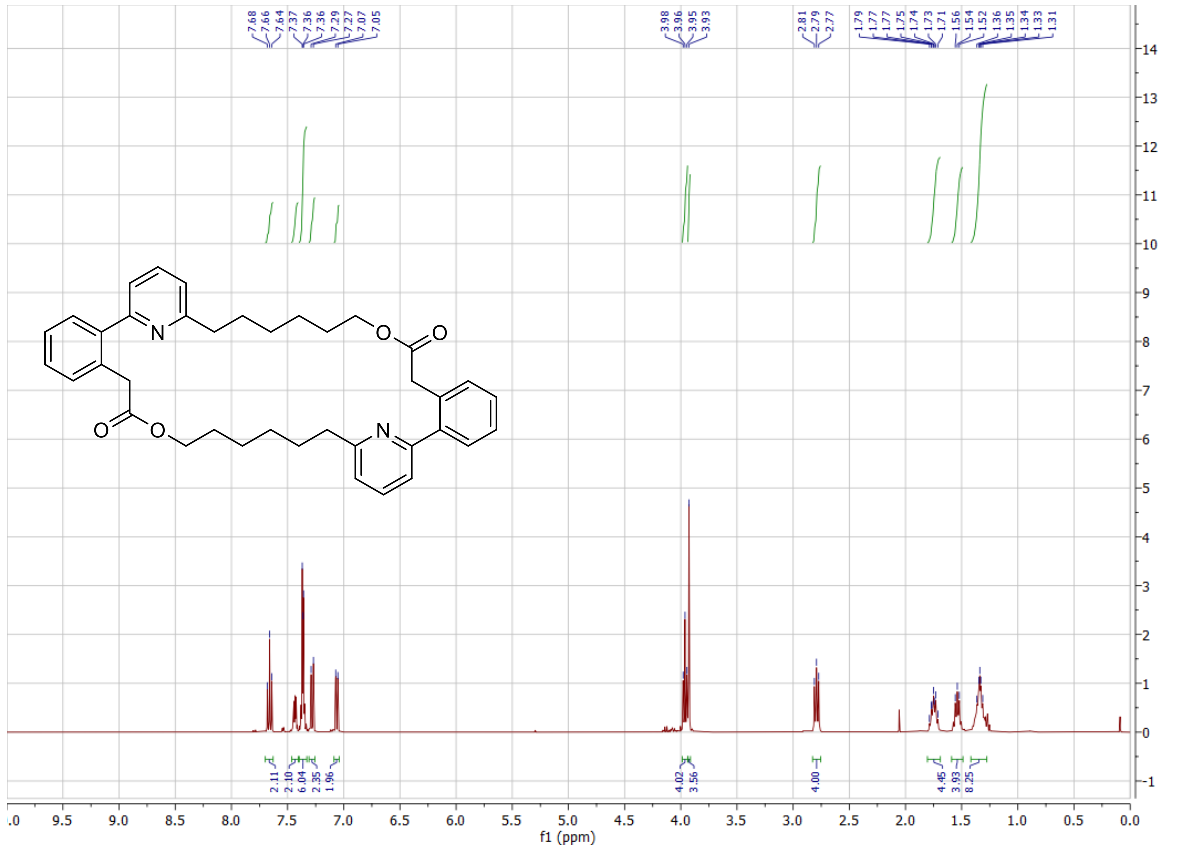

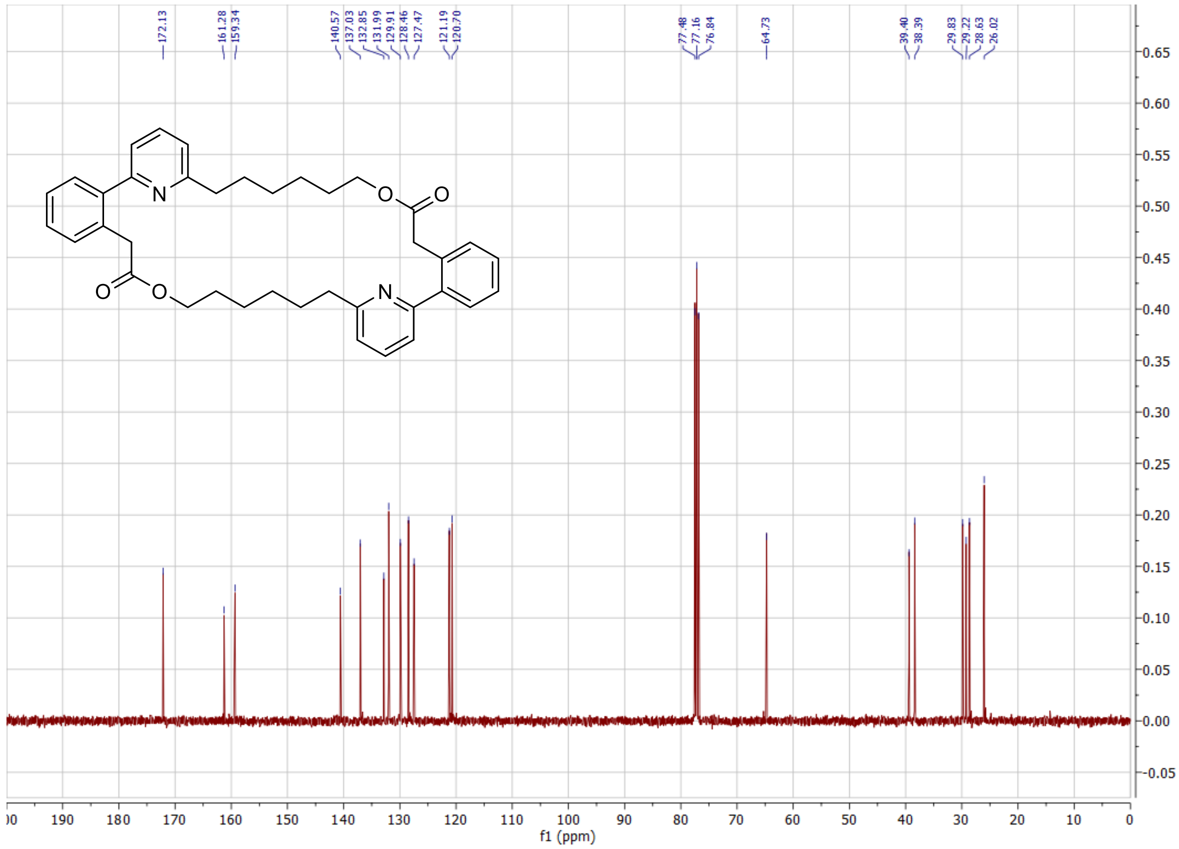

2-(3-Bromophenyl)-*N*-(3-hydroxypropyl)-*N*-methylacetamide (S153)

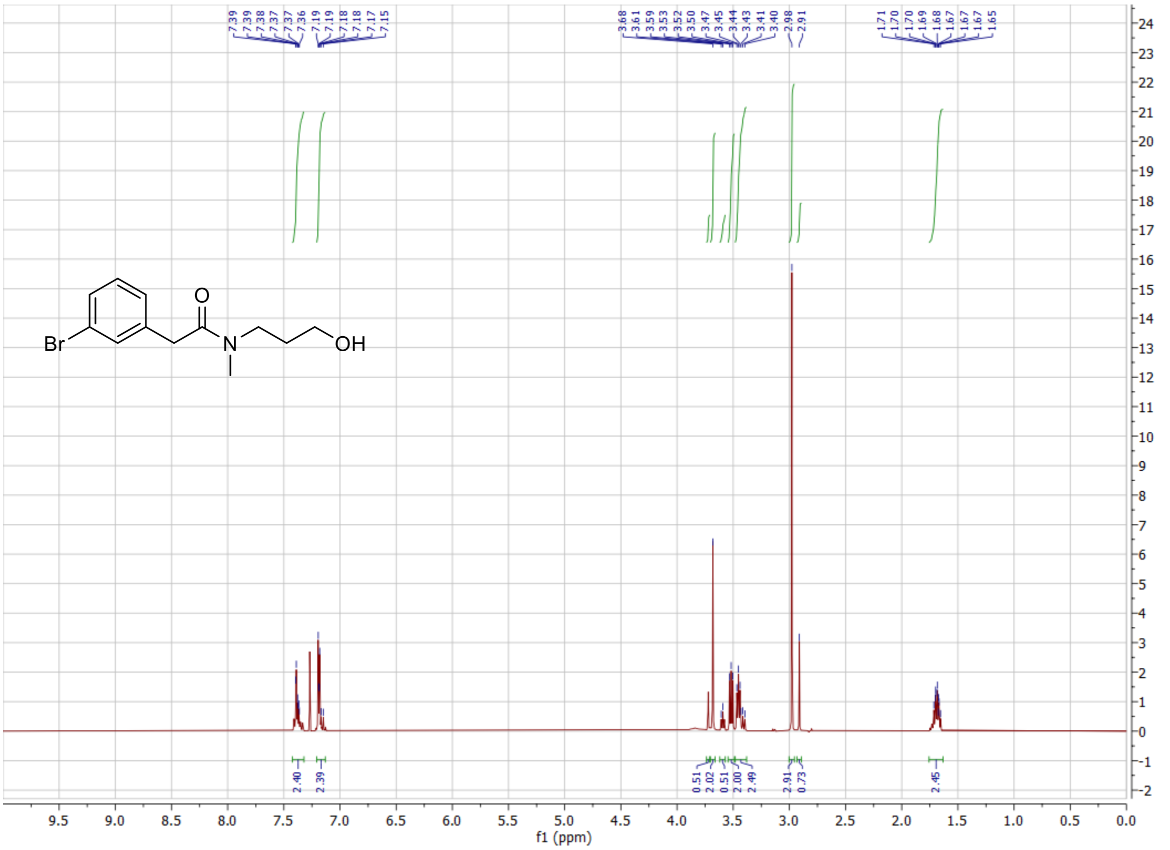

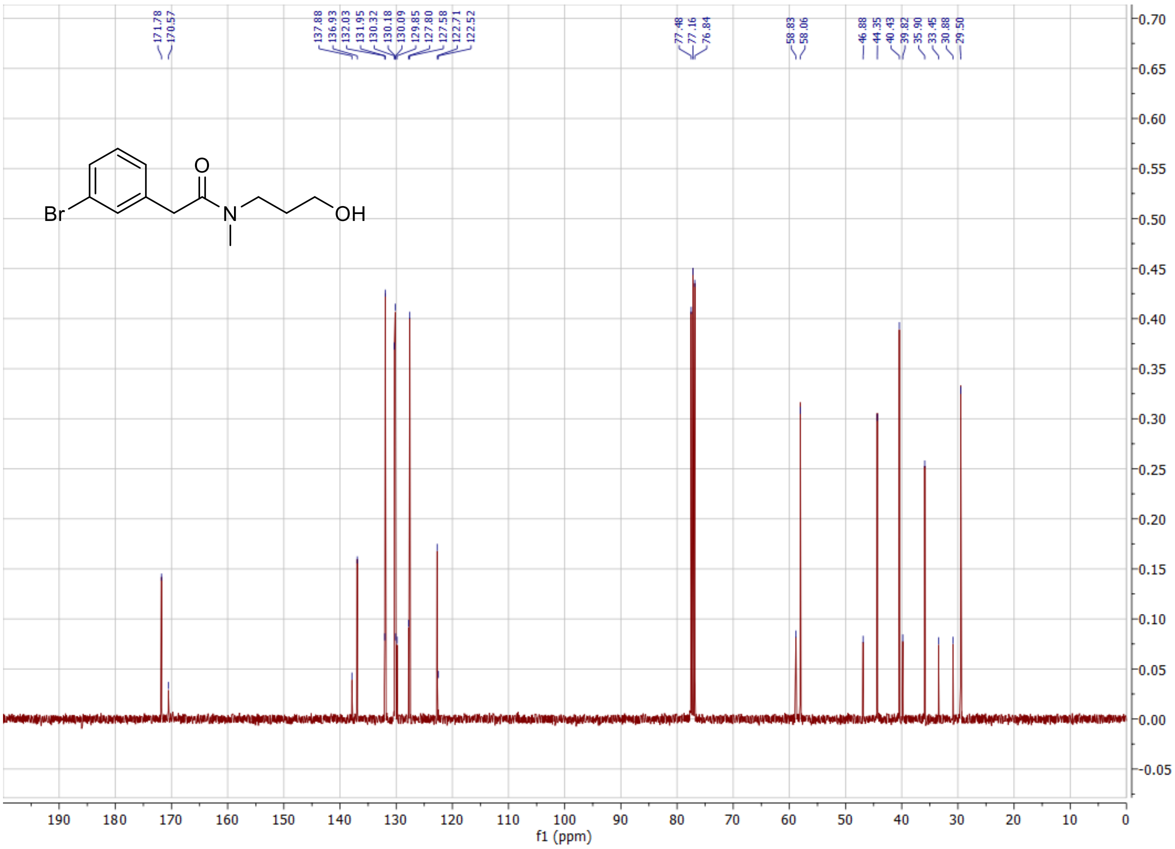

2-(3'-(2-((3-Hydroxypropyl)(methyl)amino)ethyl)-[1,1'-biphenyl]-2-yl)acetic acid (114c)

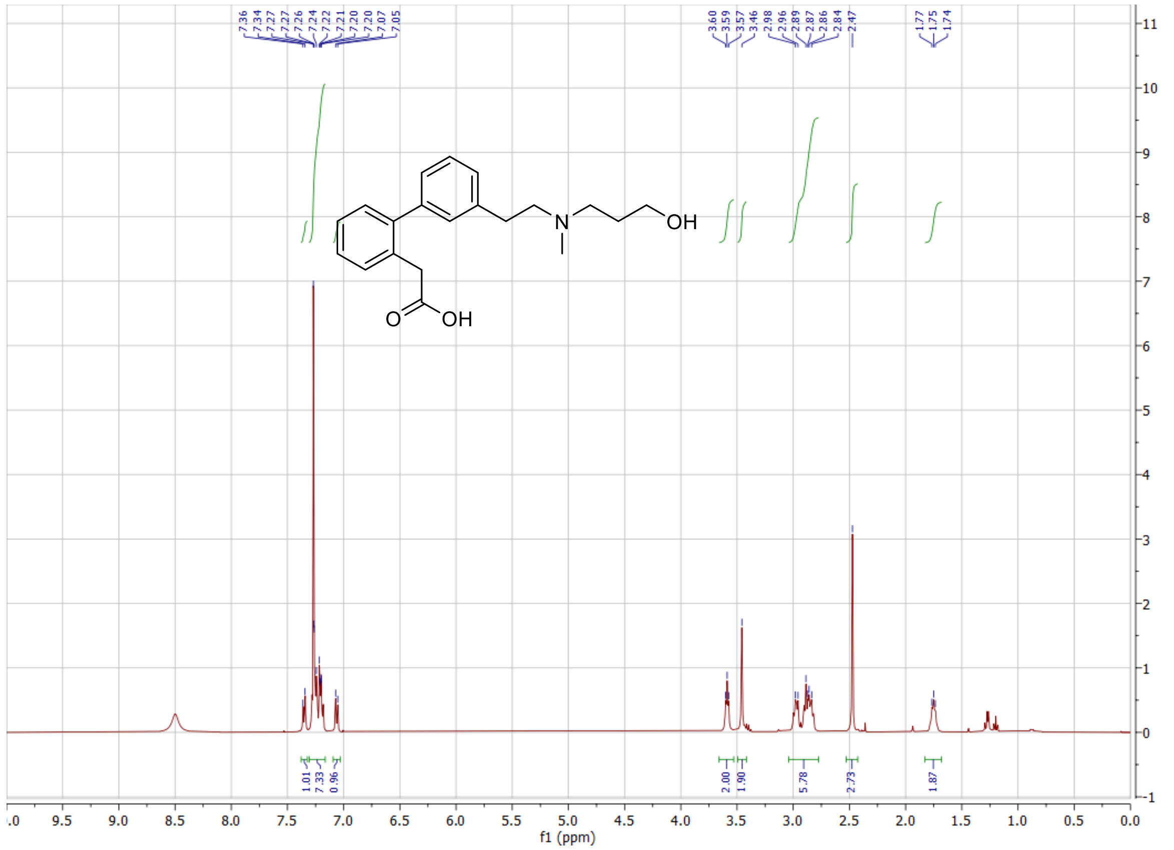

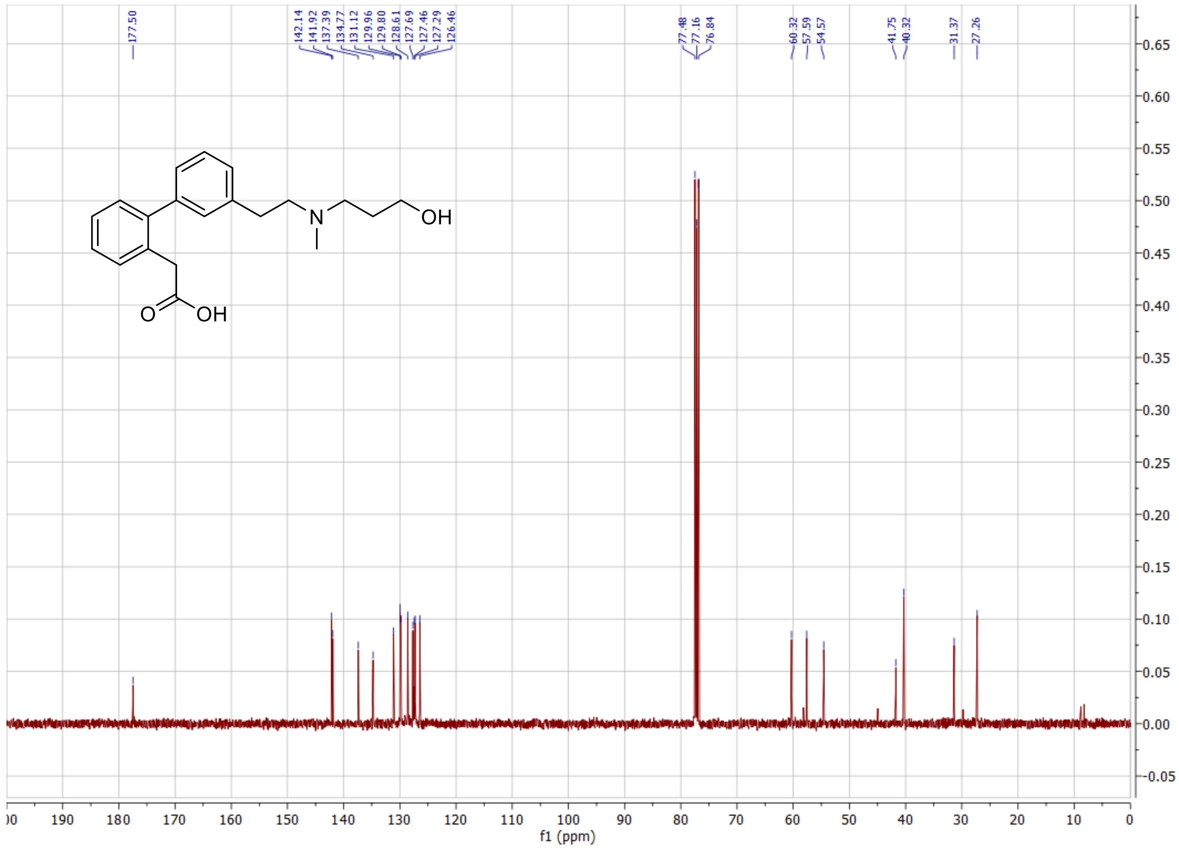

5-Methyl-9-oxa-5-aza-1(1,2),2(1,3)-dibenzenacycloundecaphan-10-one (117c)

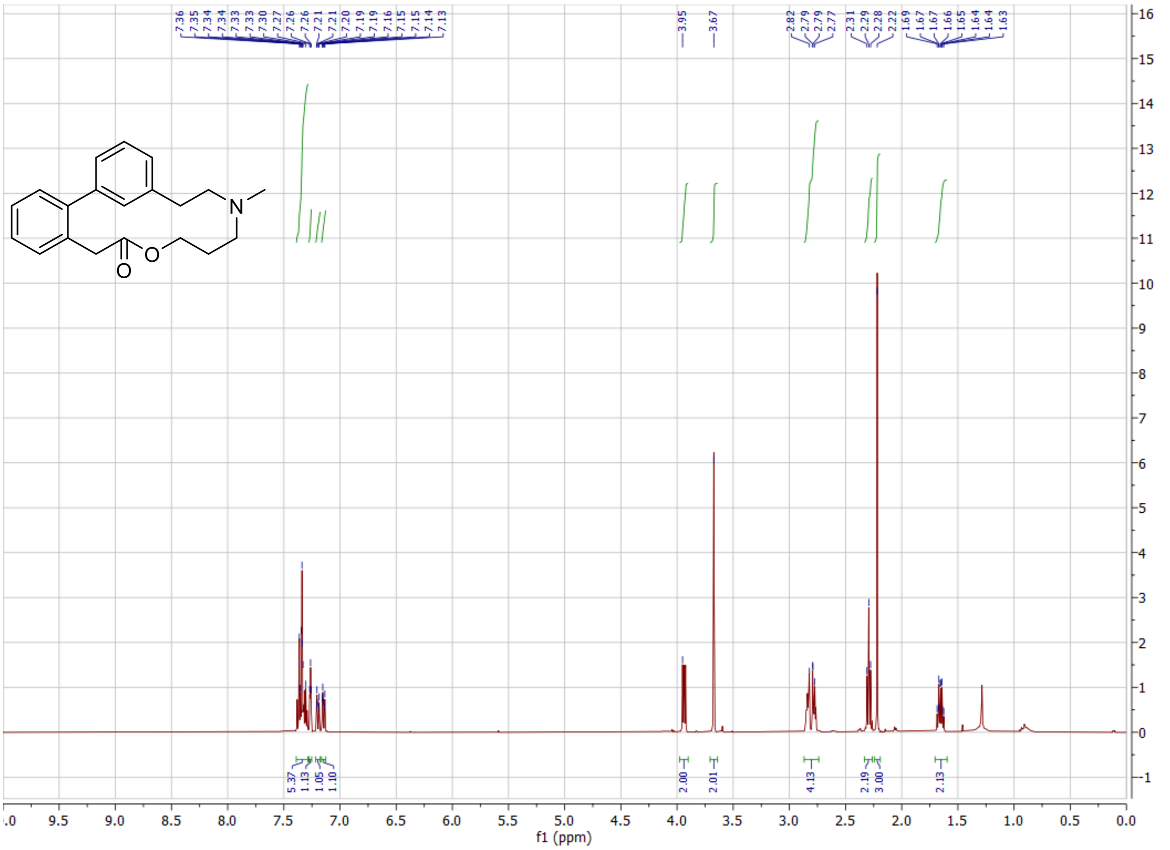

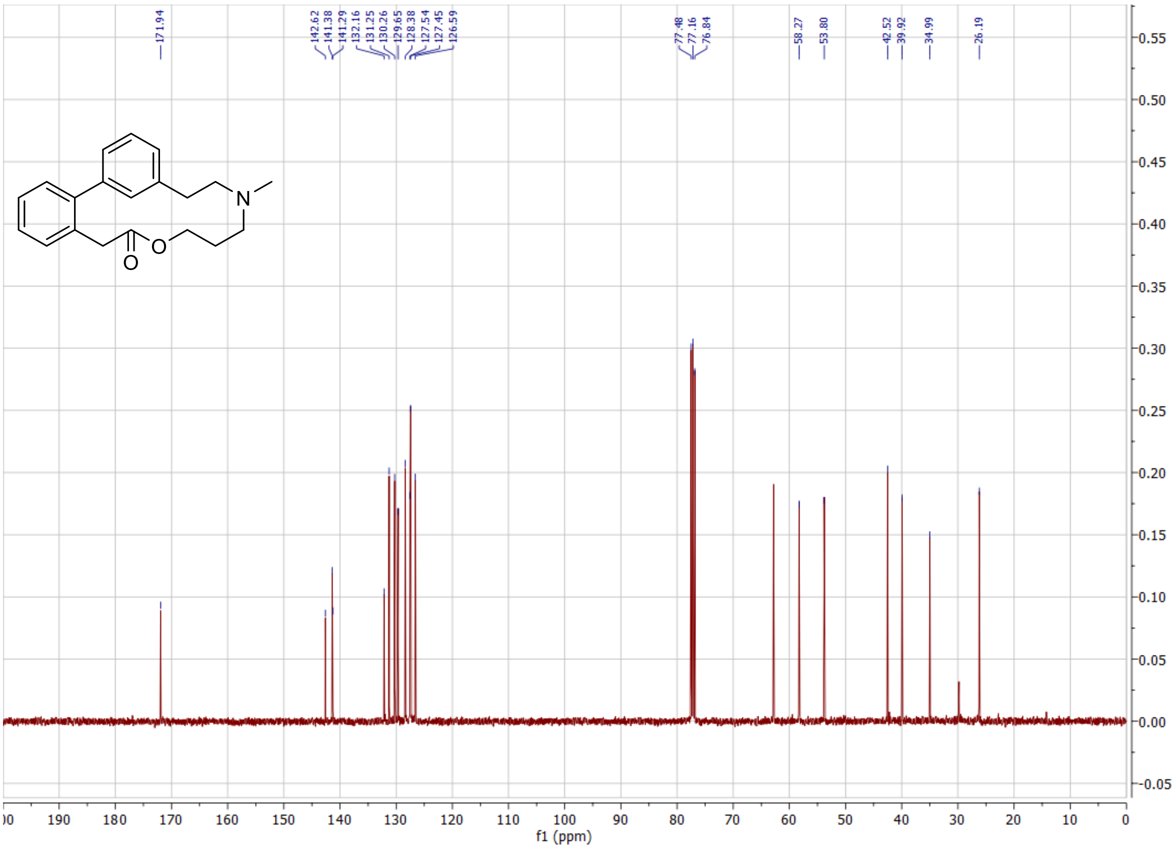

9,20-Dimethyl-5,16-dioxa-9,20-diaza-1,12(1,2),2,13(1,3)-tetrabenzenacyclodocosaphane-4,15-dione (S154)

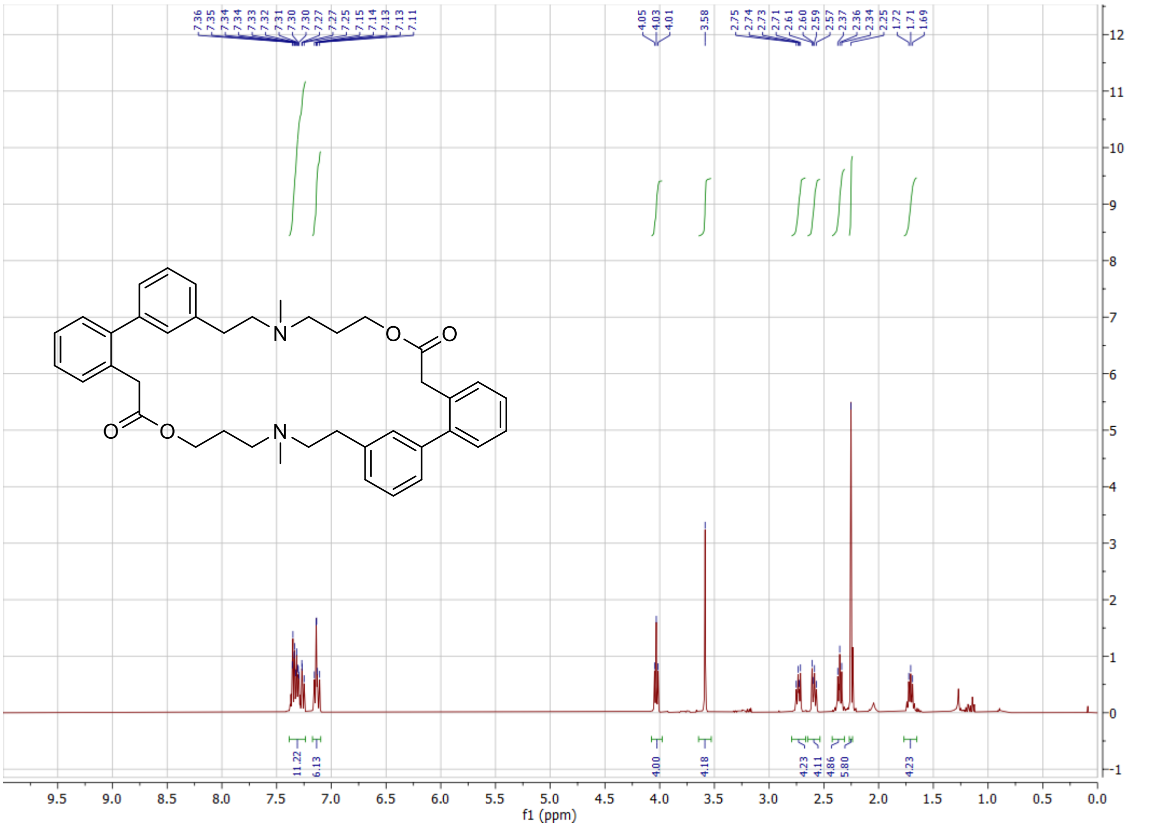

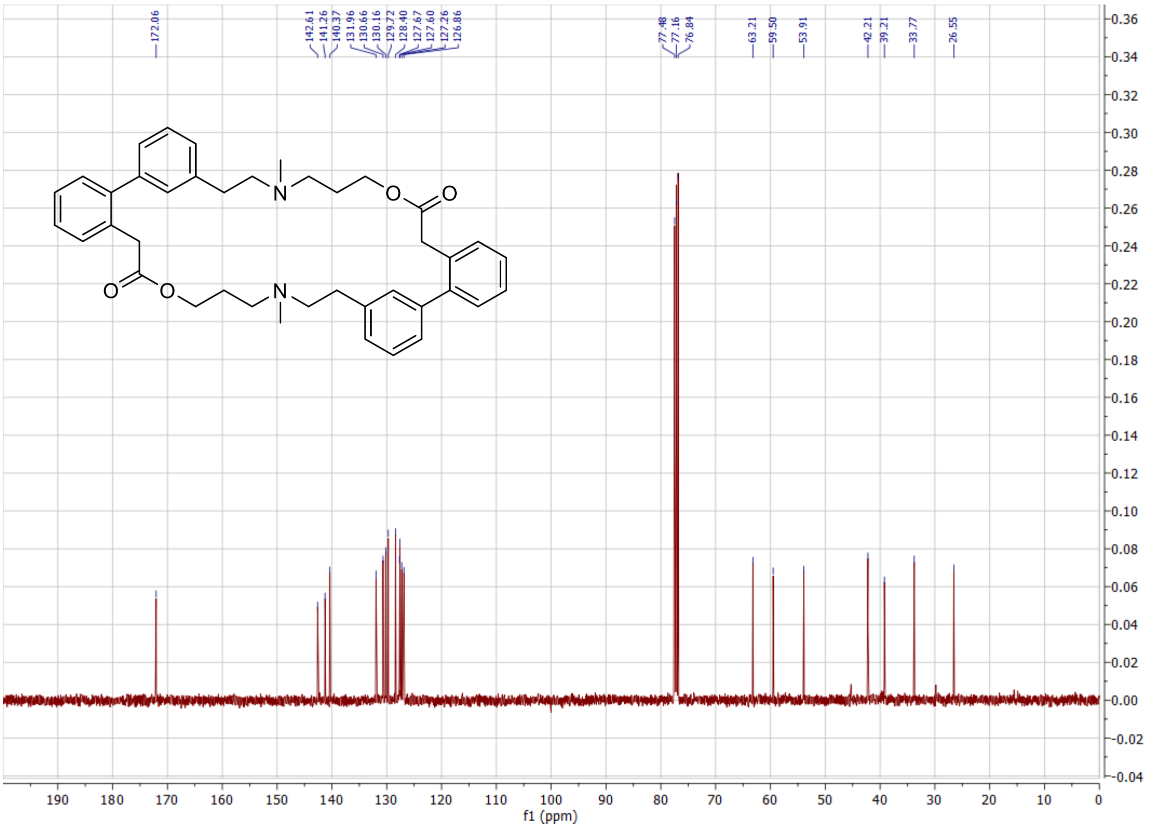

***tert*-Butyl 2-((6-(2-(benzyl(2-hydroxyethyl)amino)ethyl)pyridin-2-yl)methoxy)acetate (S155)**

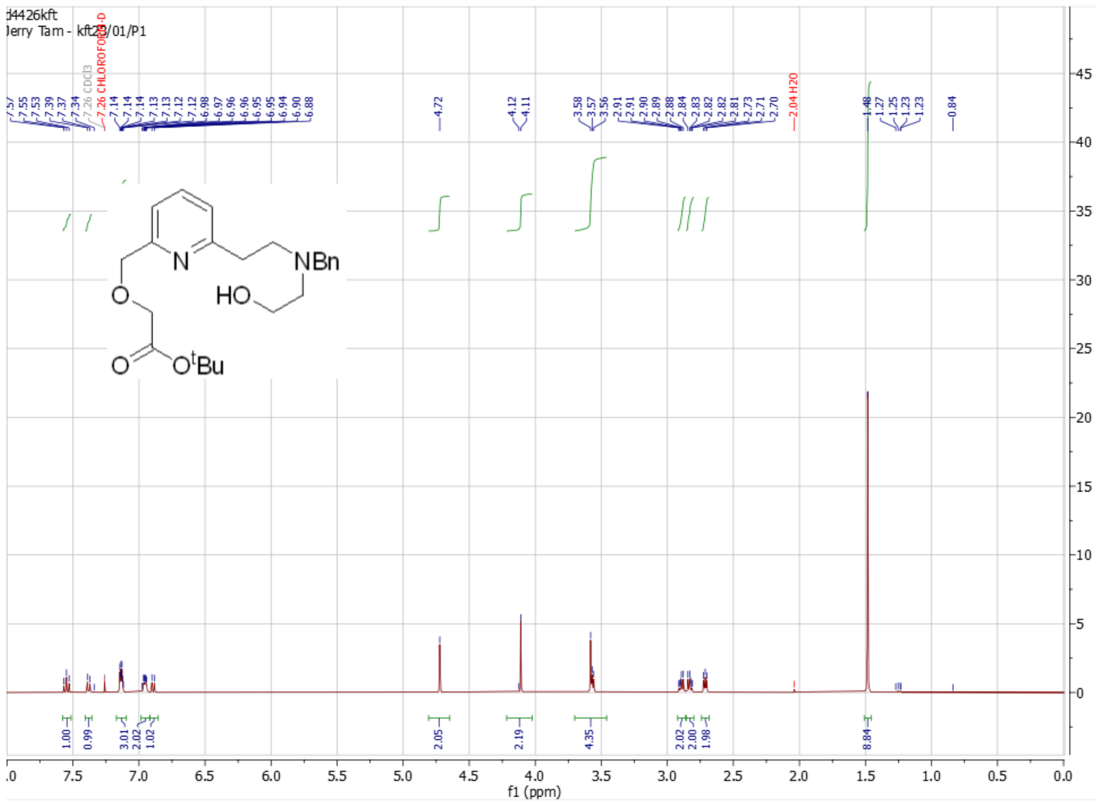

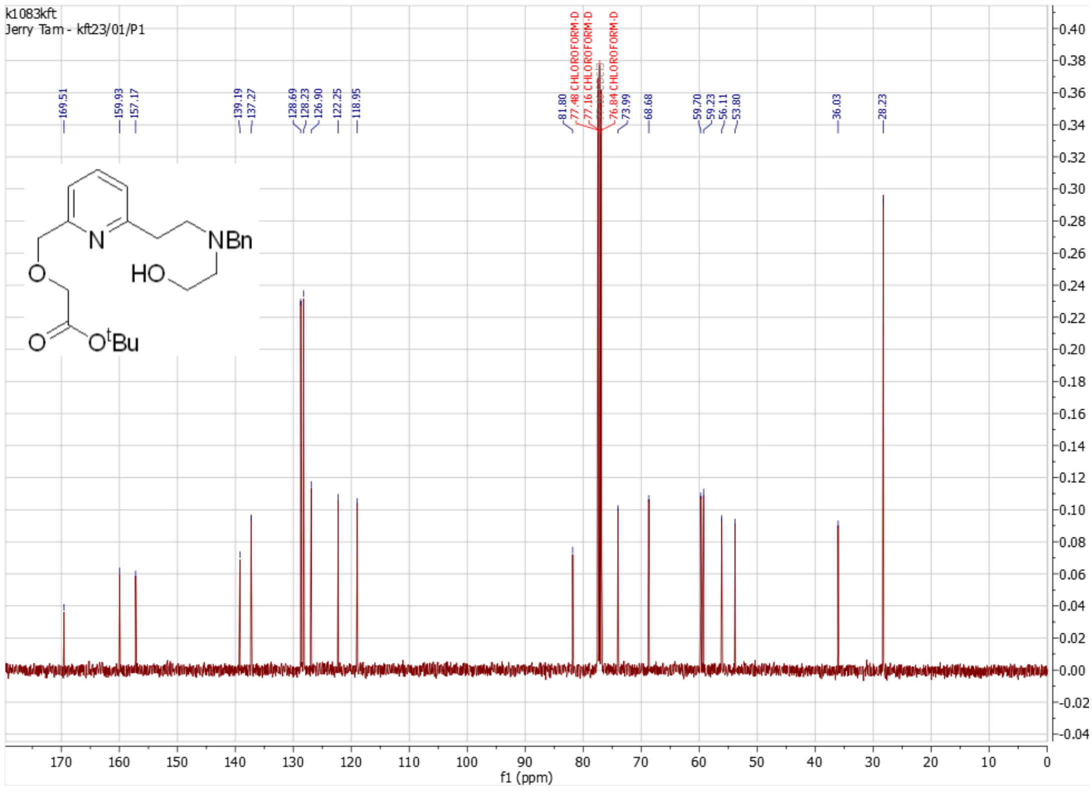

9-Benzyl-3,6-dioxa-9-aza-1(2,6)-pyridinacycloundecaphan-5-one (118)

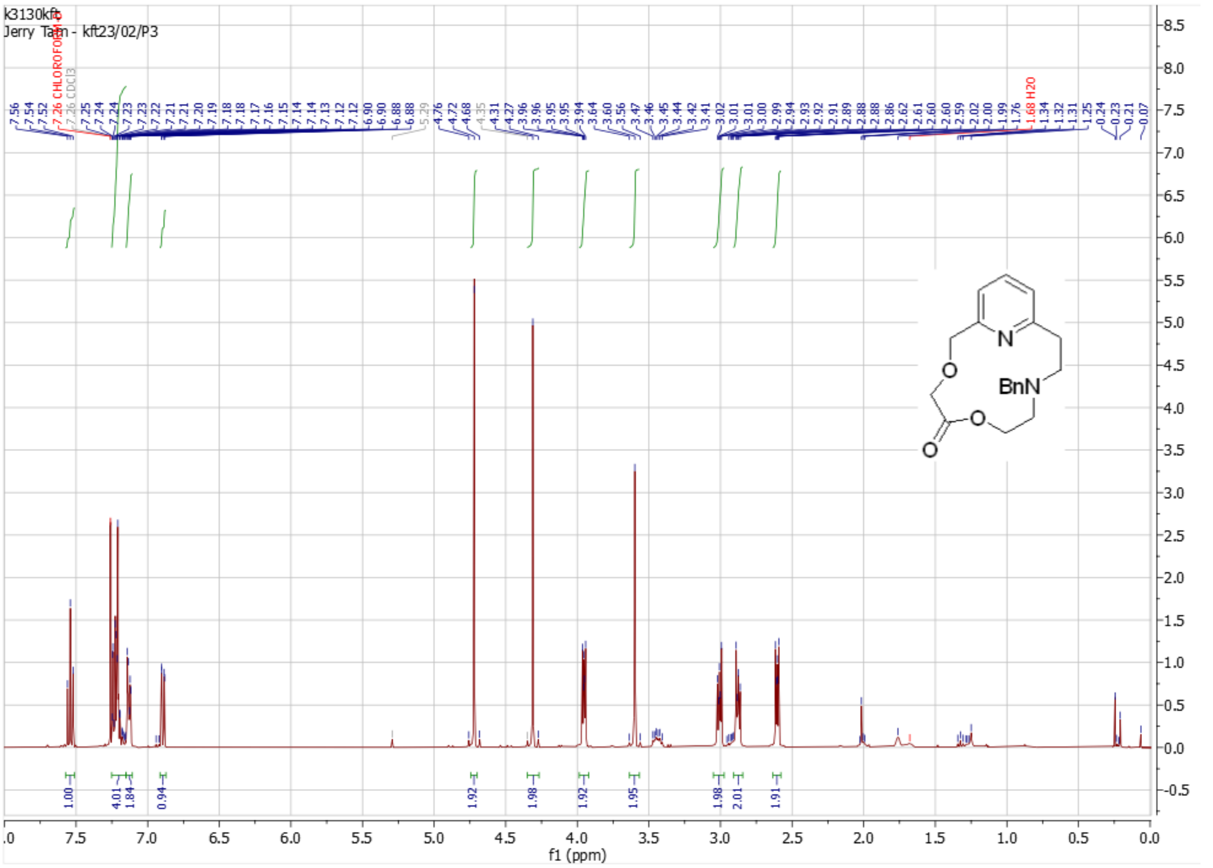

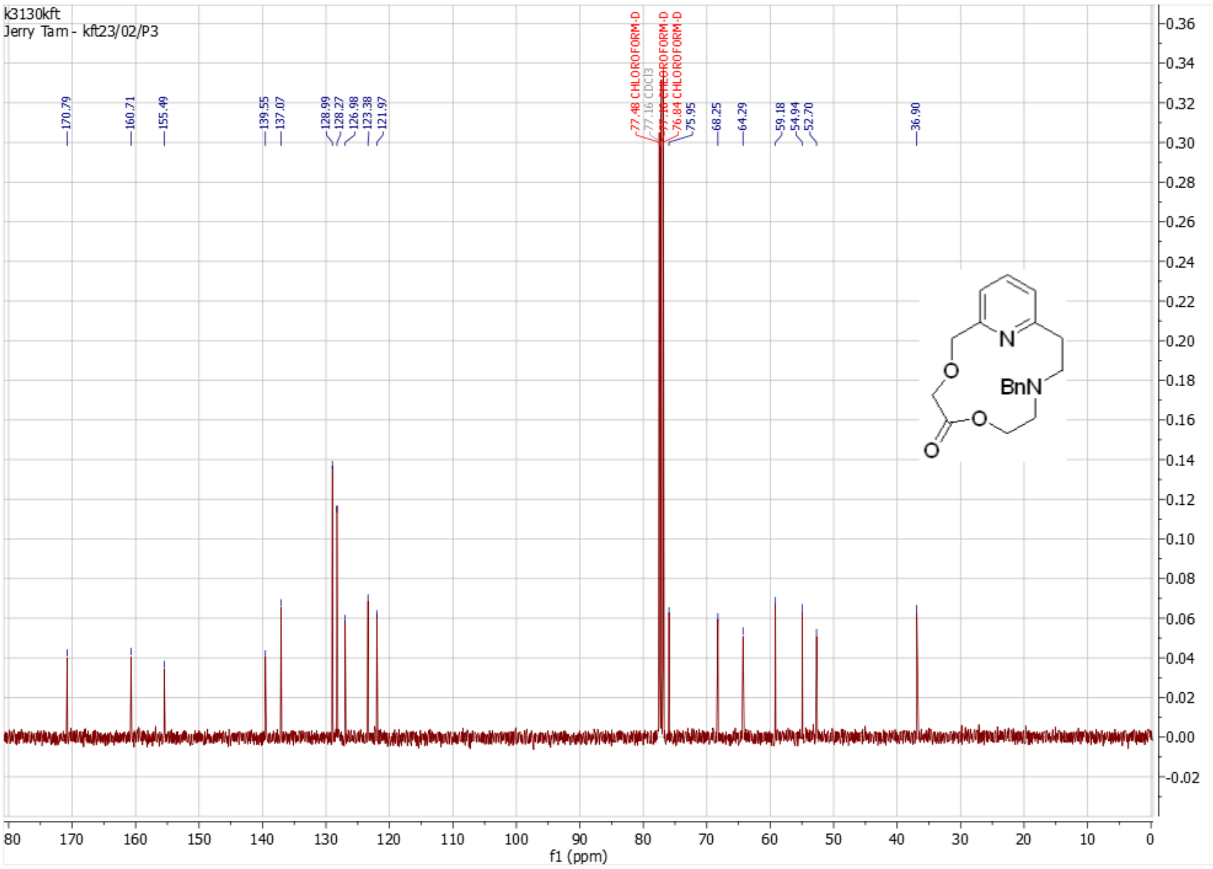

***tert*-Butyl 2-((6-(2-(2-(hydroxymethyl)piperidin-1-yl)ethyl)pyridin-2-yl)methoxy)acetate (S156)**

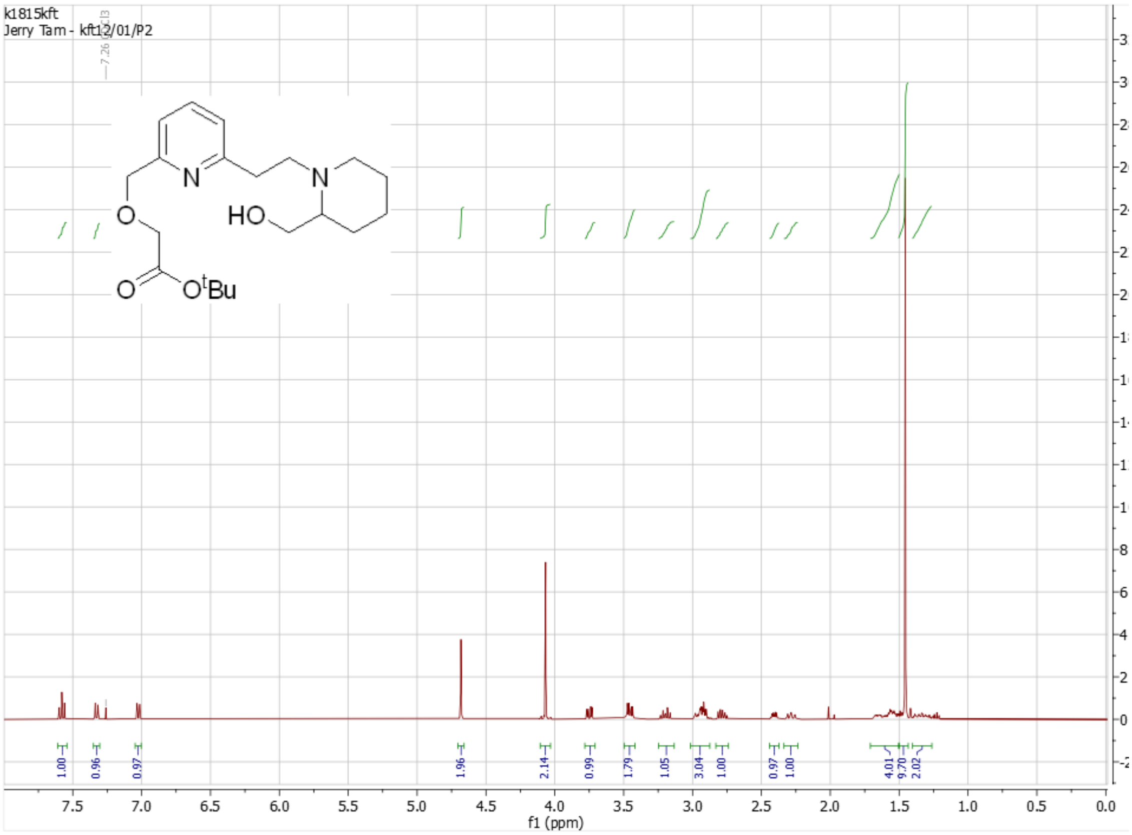

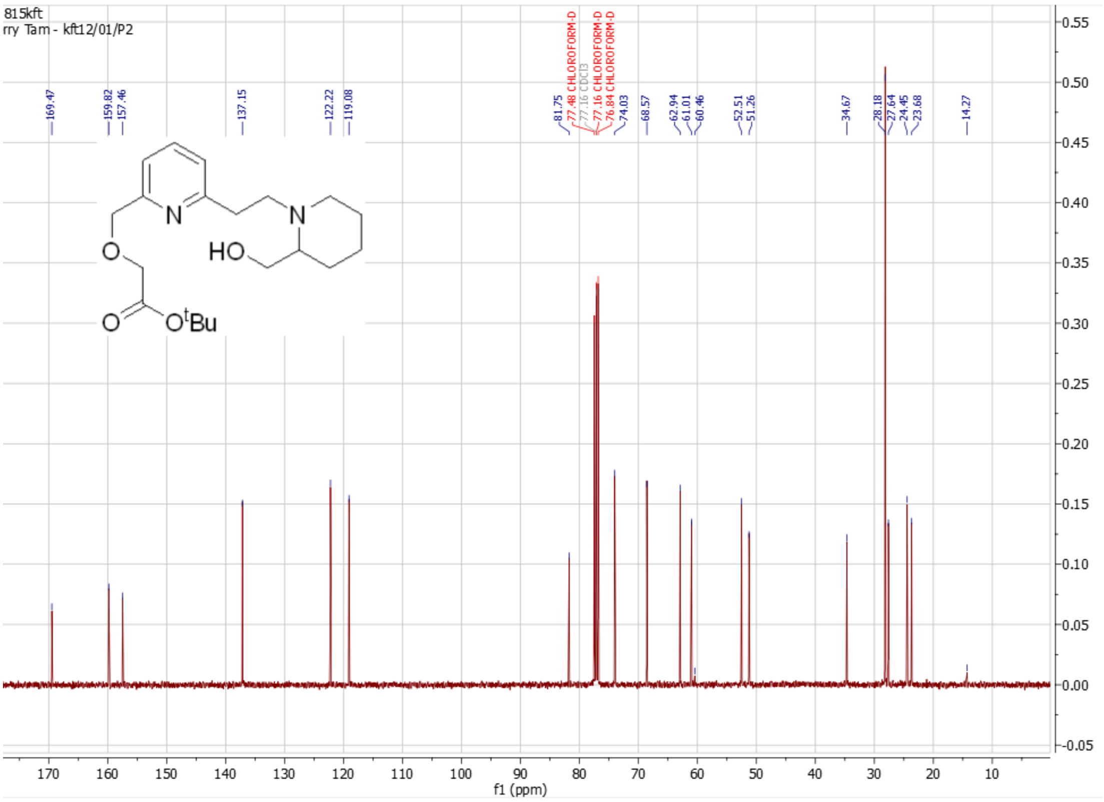

6,9-Dioxa-1(2,6)-pyridina-4(1,2)-piperidinacyclodecaphan-7-one (119)

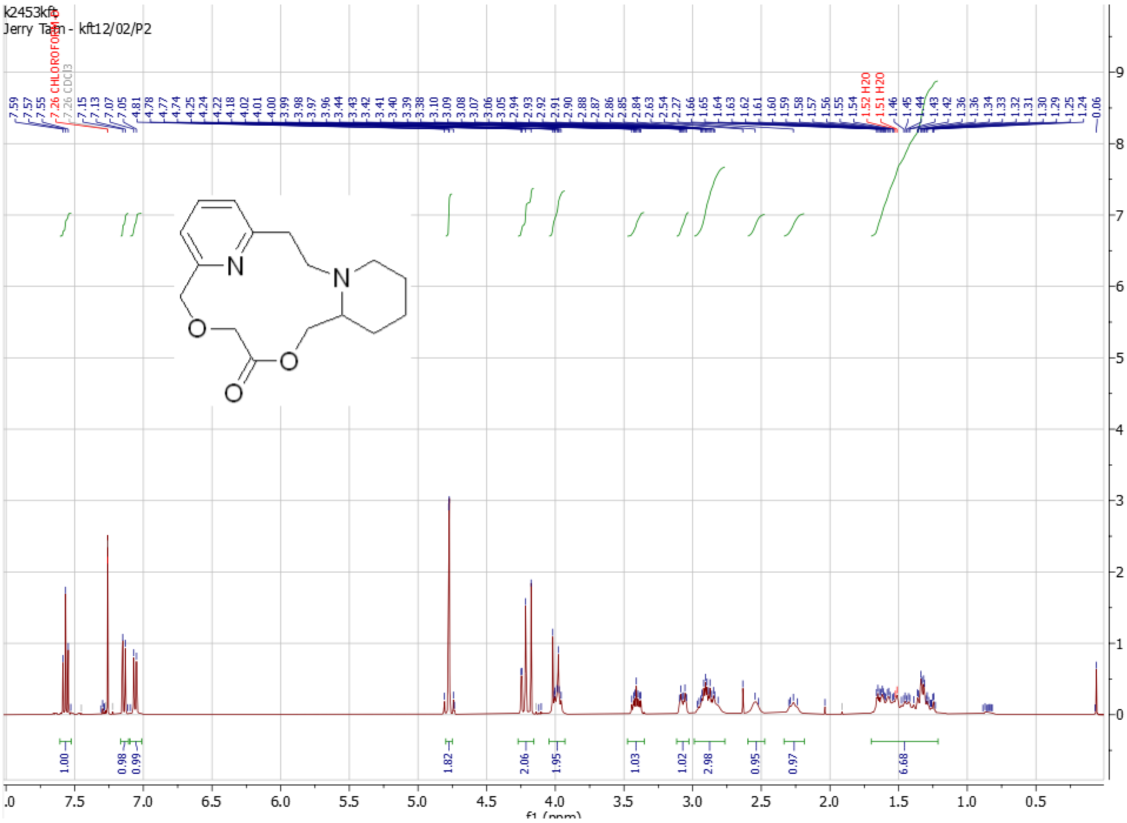

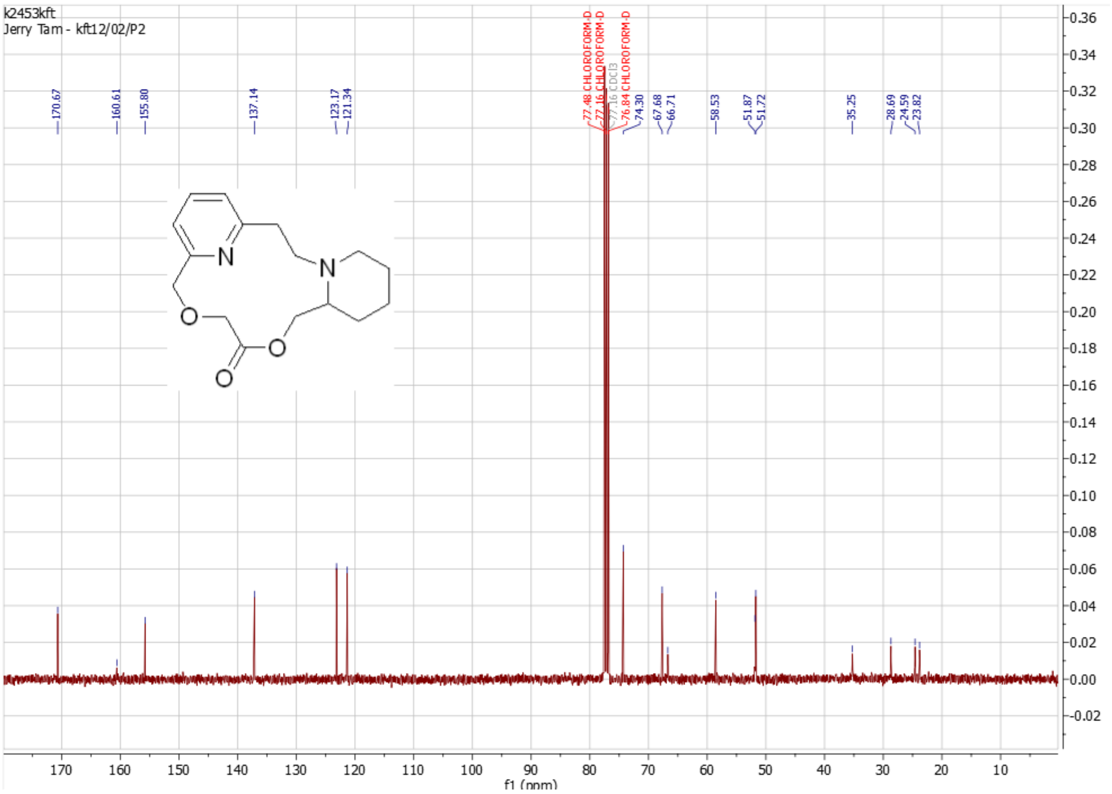

Methyl 4-[benzyl(3-hydroxypropyl)amino]butanoate (S156)

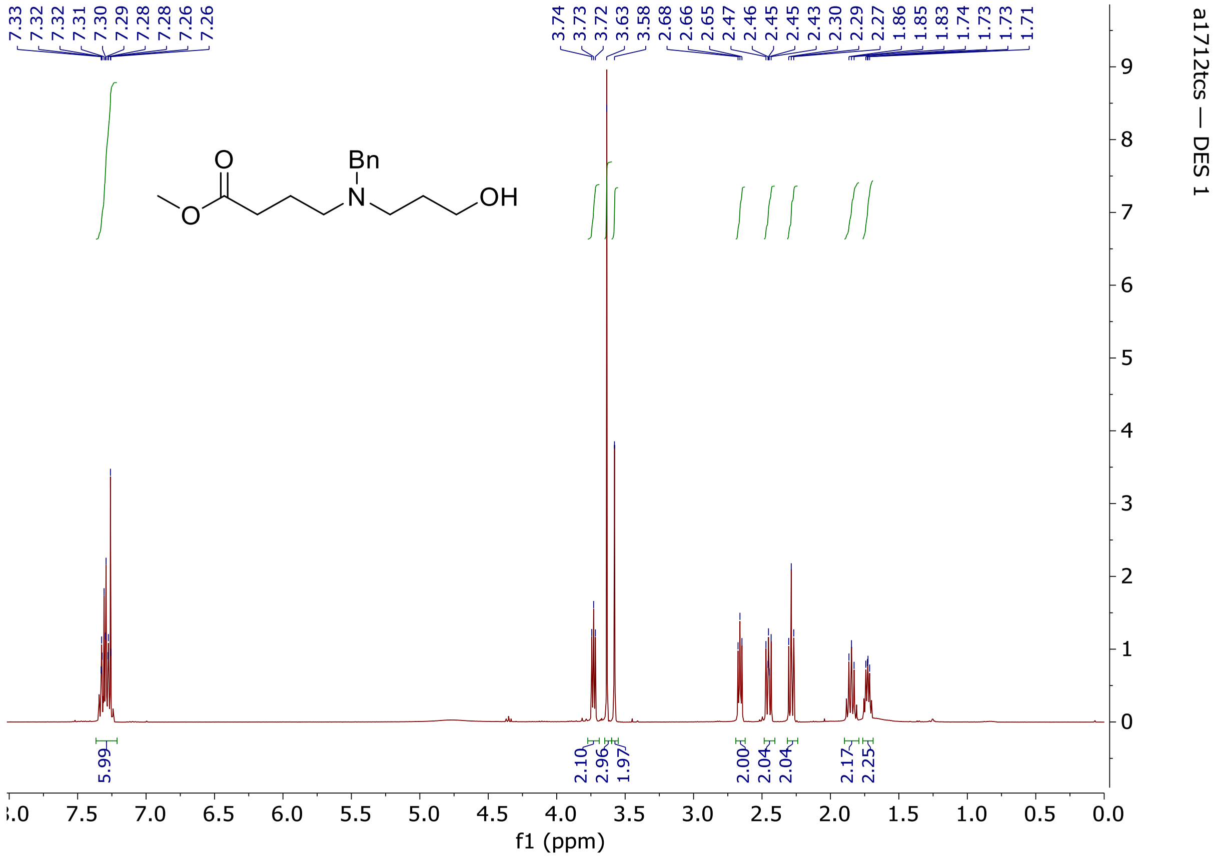

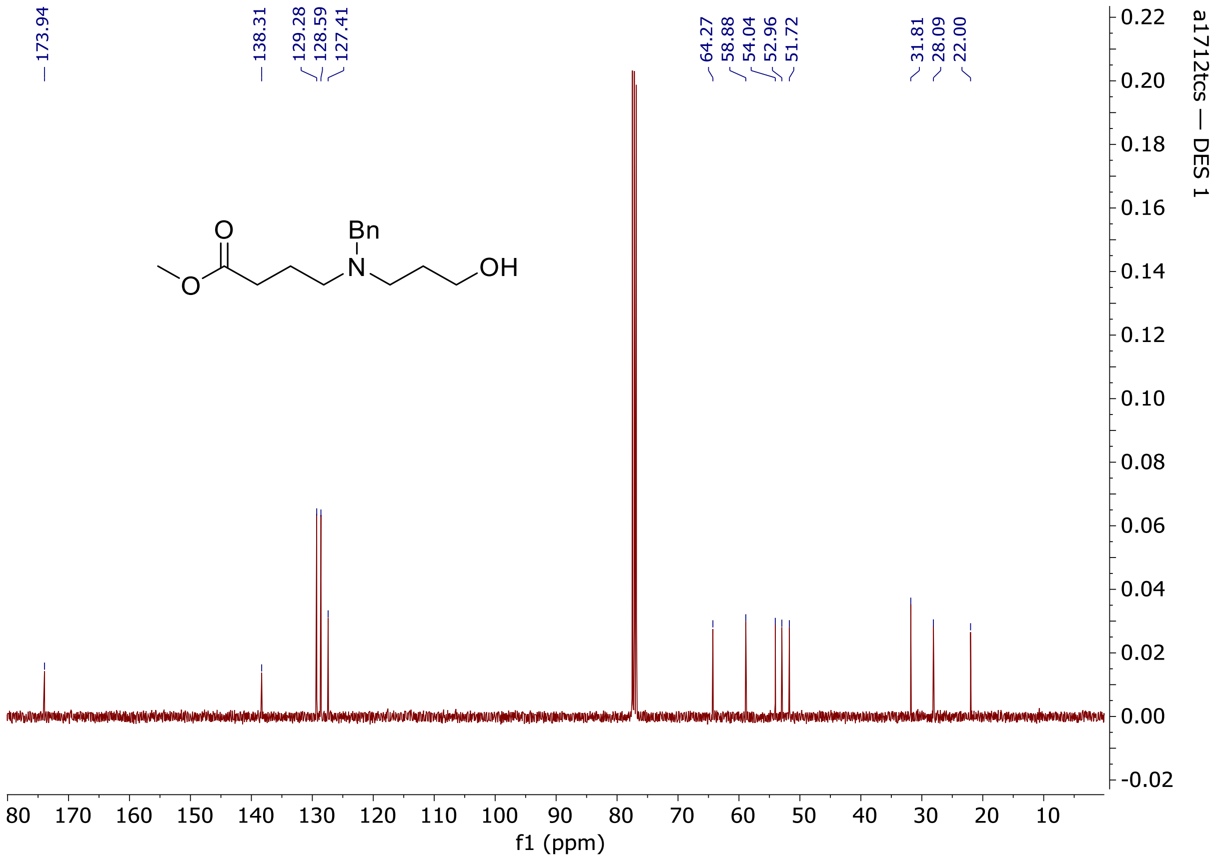

Methyl 4-[benzyl(3-bromopropyl)amino]butanoate (S157)

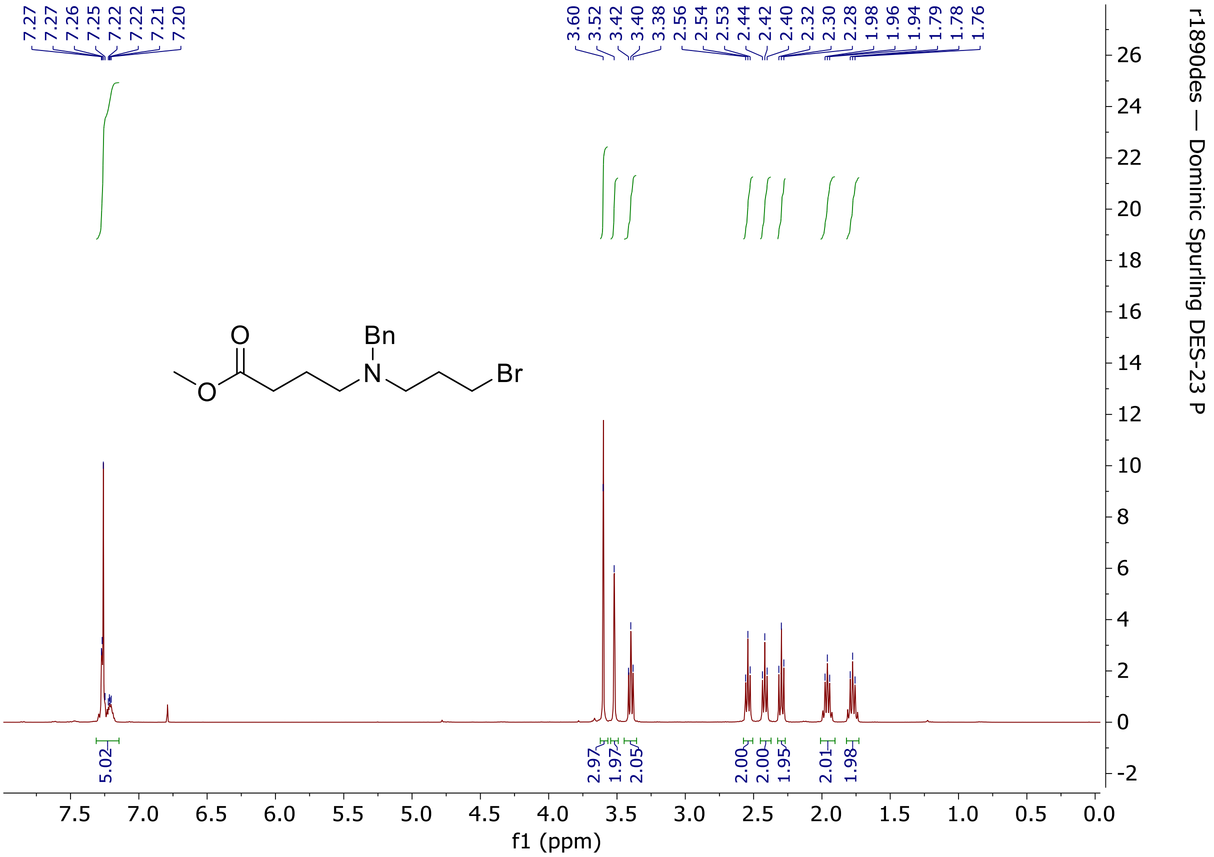

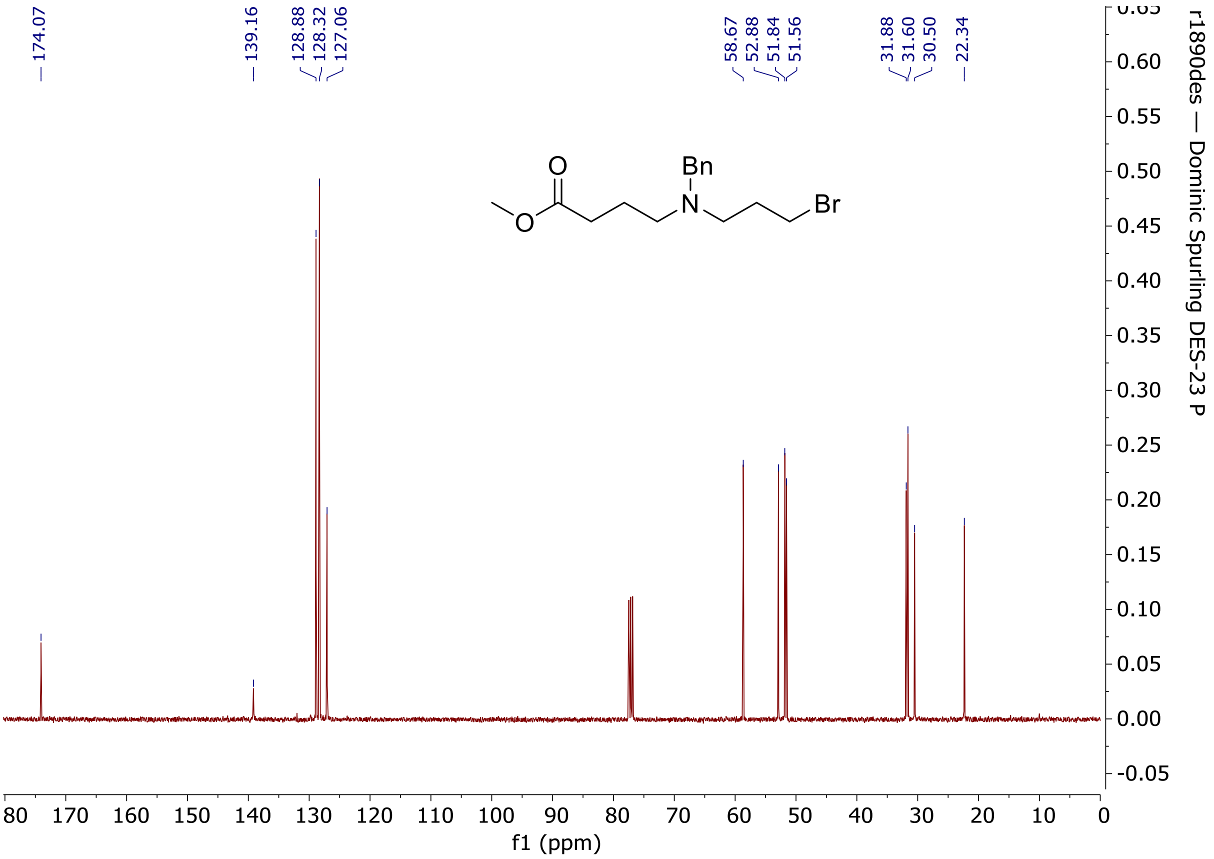

Methyl 4-[benzyl({3-[benzyl(3-hydroxypropyl)amino]propyl})amino]butanoate (S158)

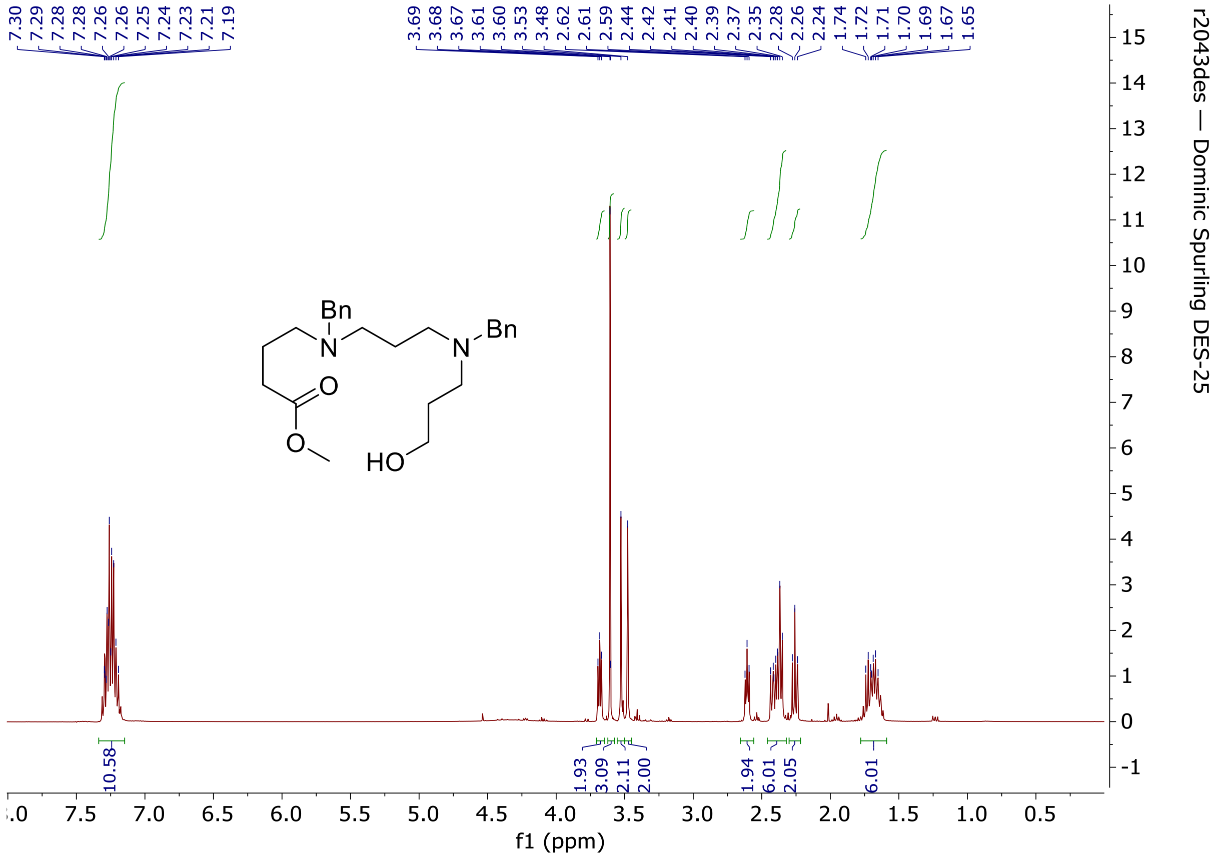

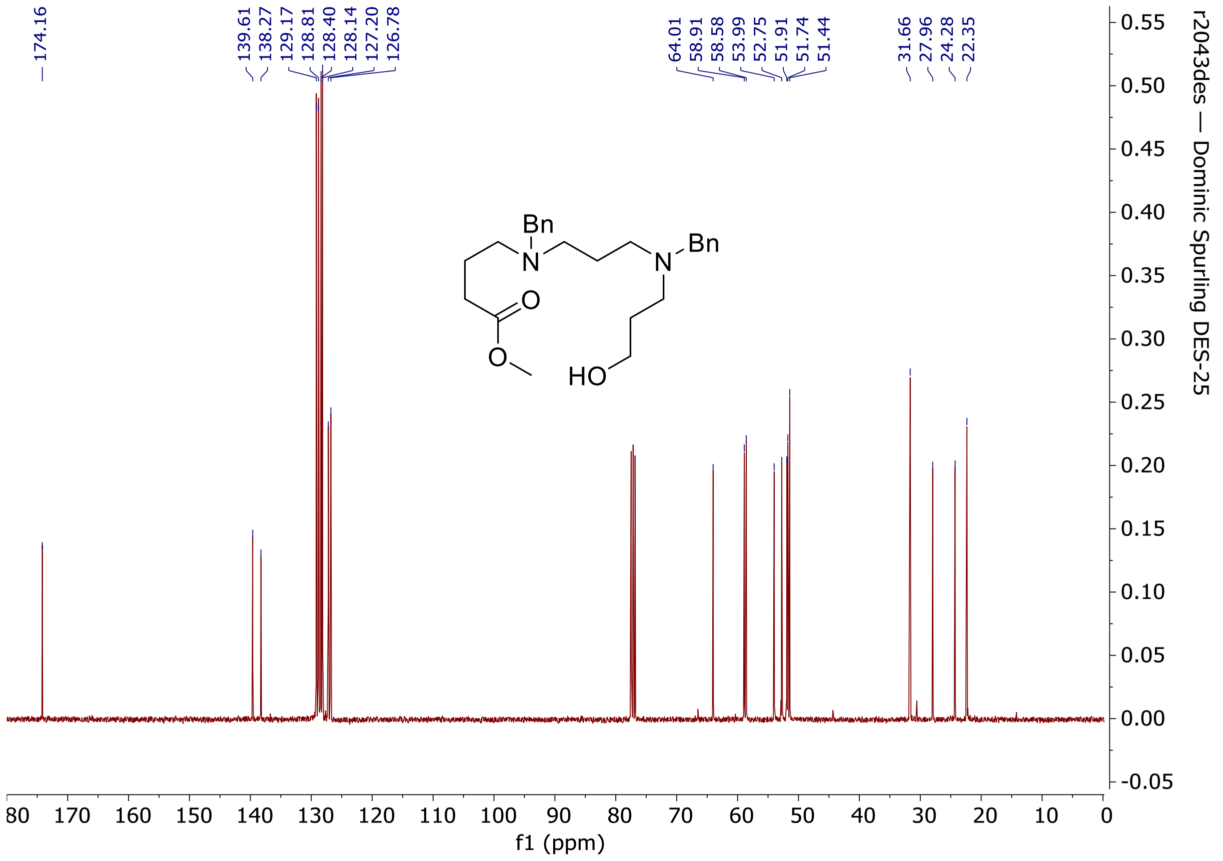

4-[Benzyl({3-[benzyl(3-hydroxypropyl)amino]propyl})amino]butanoic acid (S159)

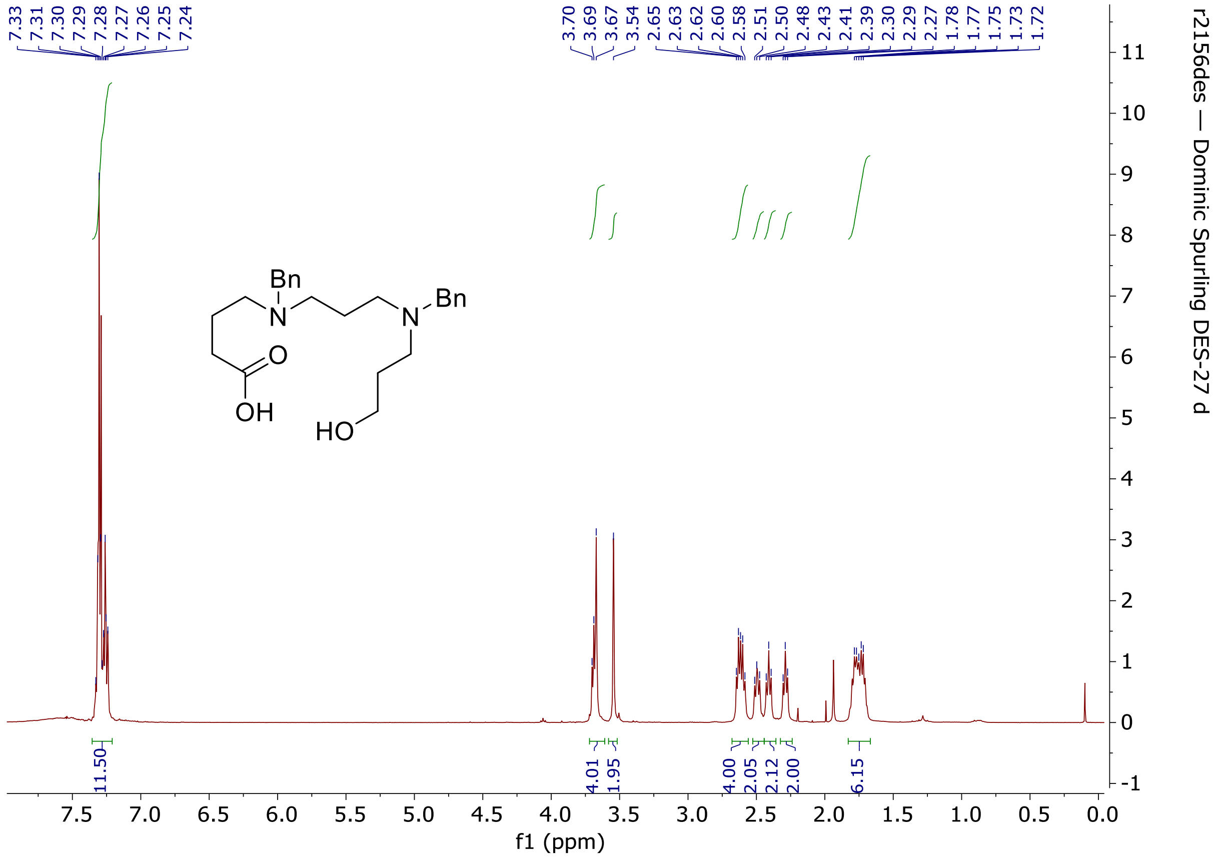

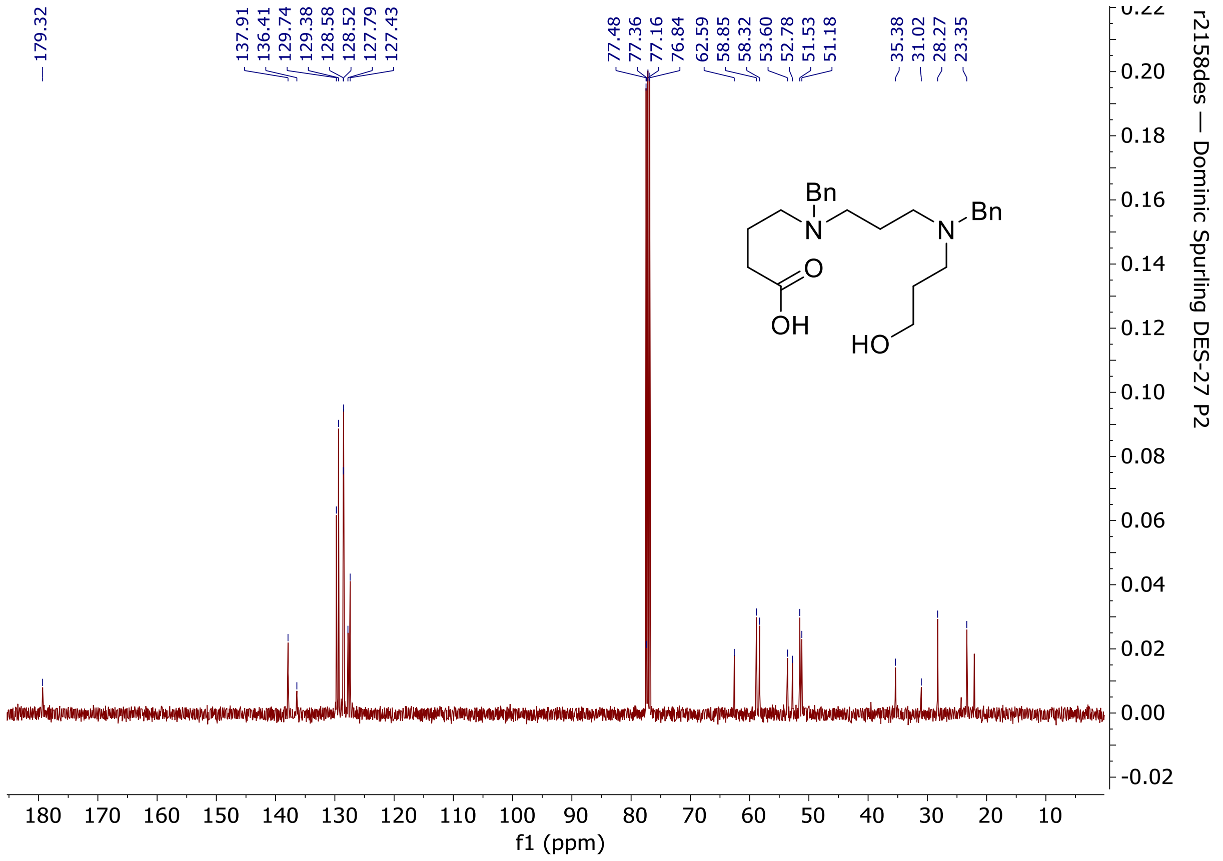

5,9-Dibenzyl-1-oxa-5,9-diazacyclotridecan-13-one (120)

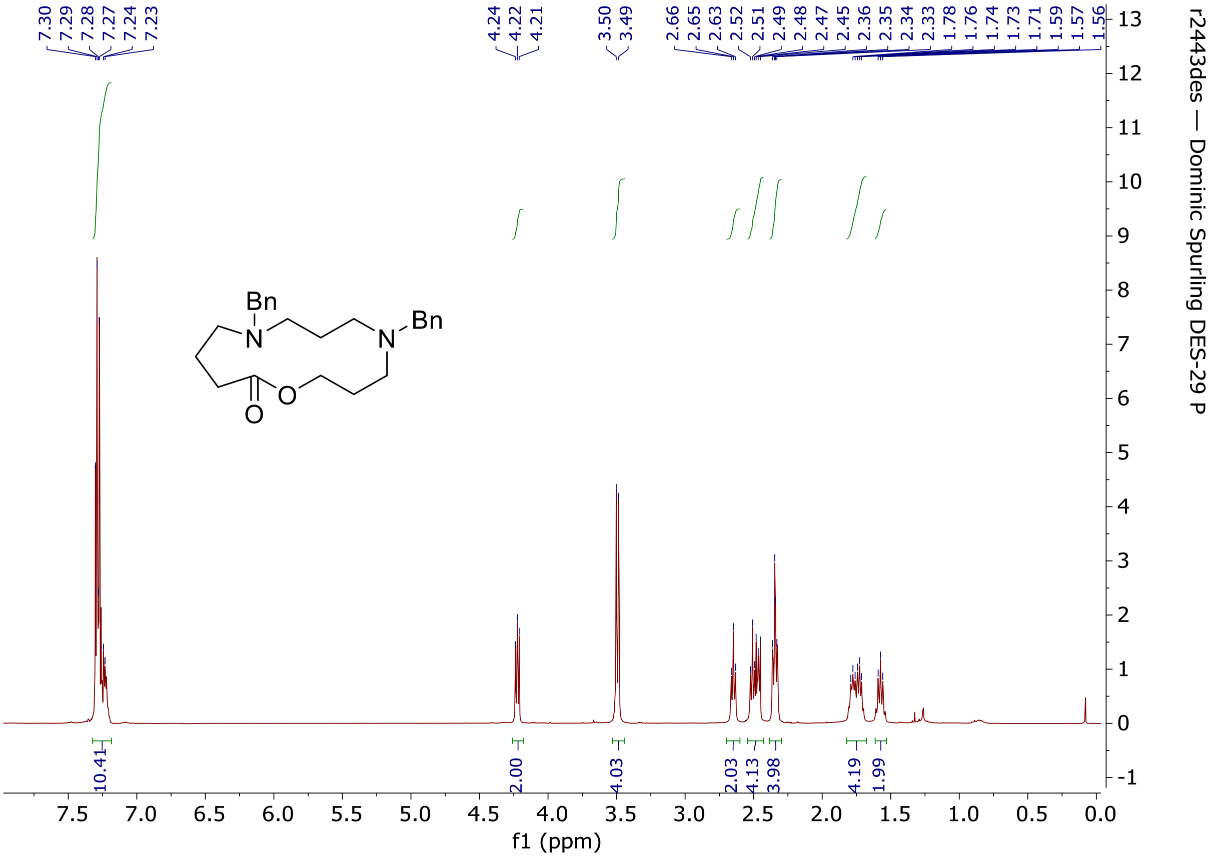

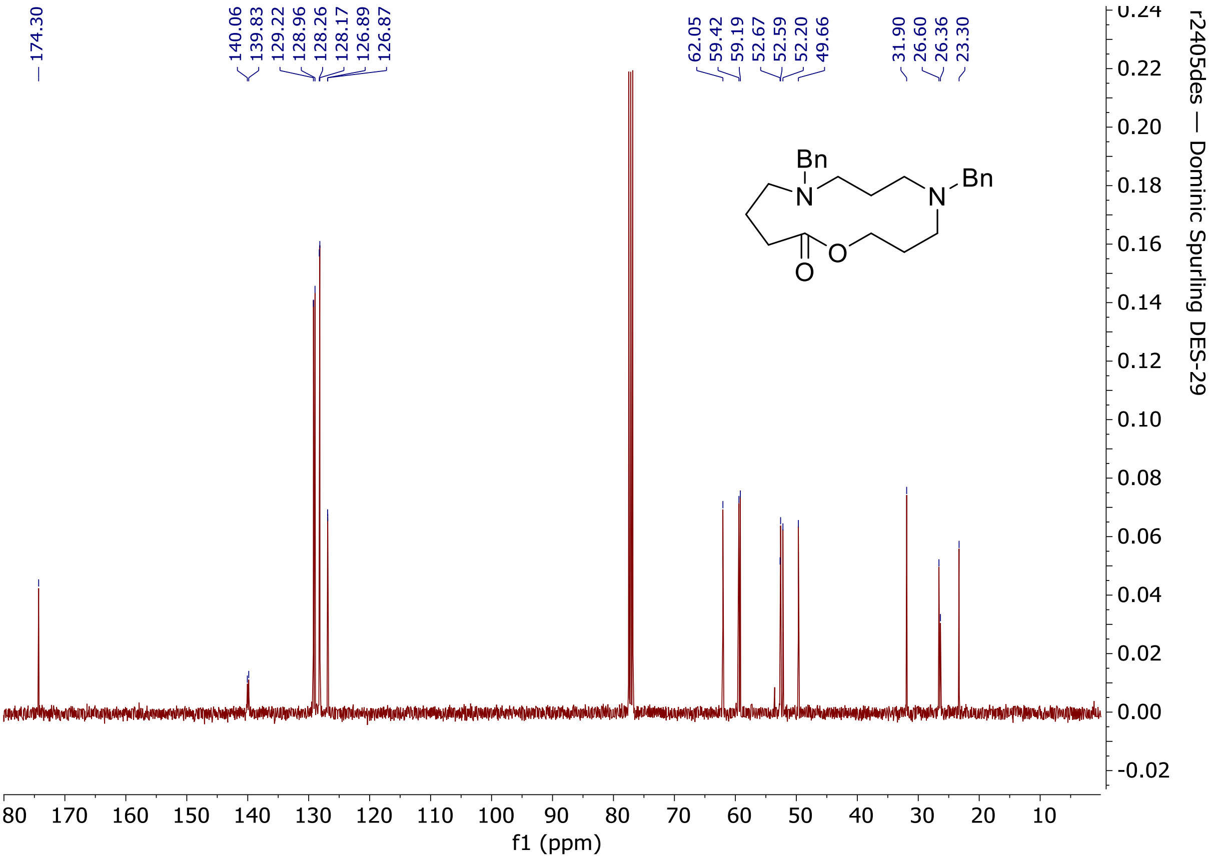

Methyl 2-(((2-((3-hydroxypropyl)(phenyl)amino)ethyl)(phenyl)amino)methyl)benzoate (S161)

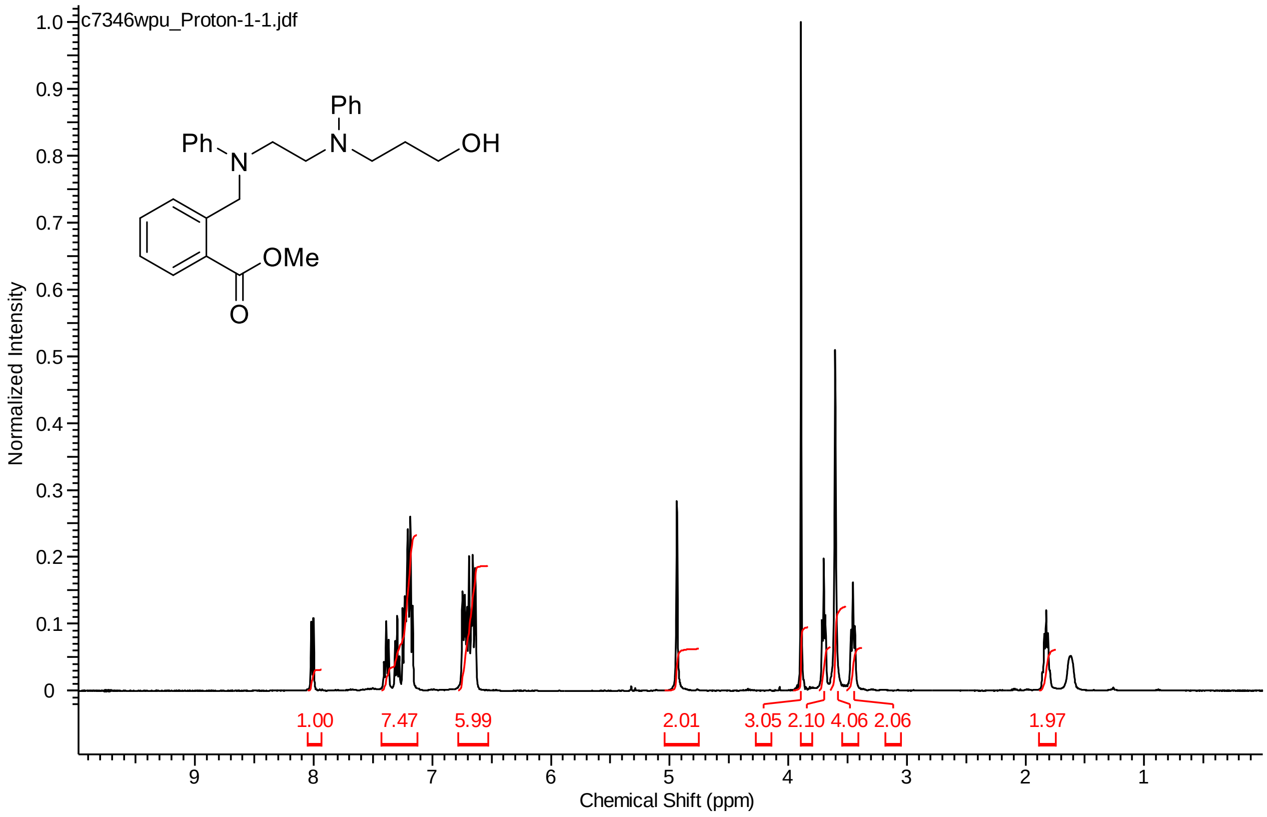

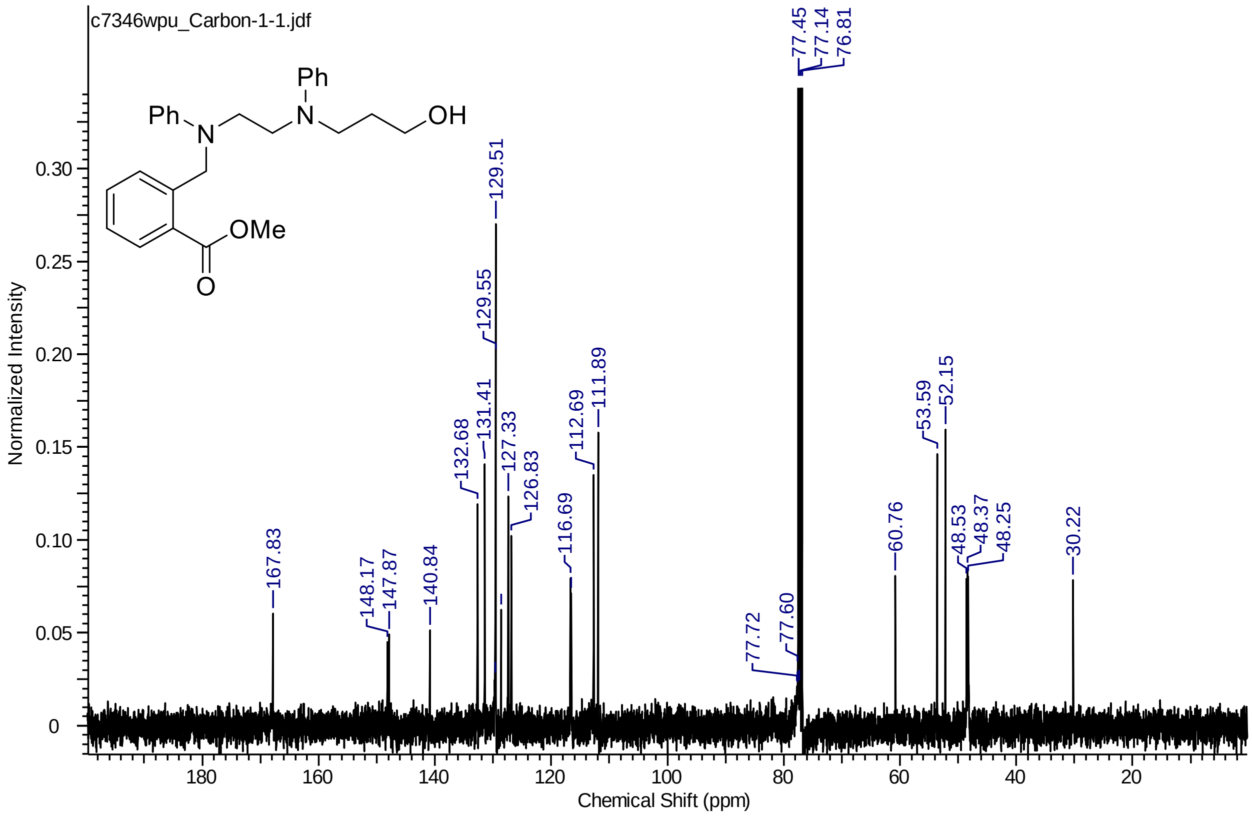

Macrocycle (121). In solution in $CDCl_3$, compound **121** exists as a 10:4:1 (A:B:C) mixture of rotamers

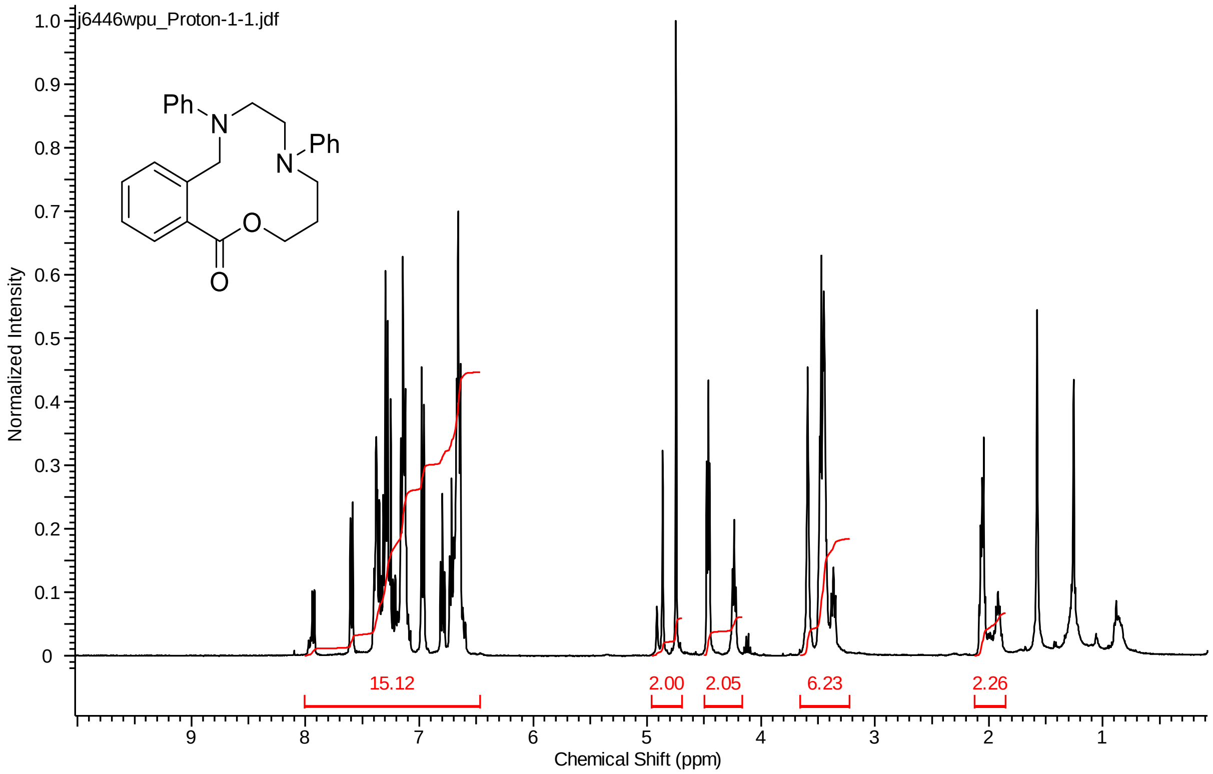

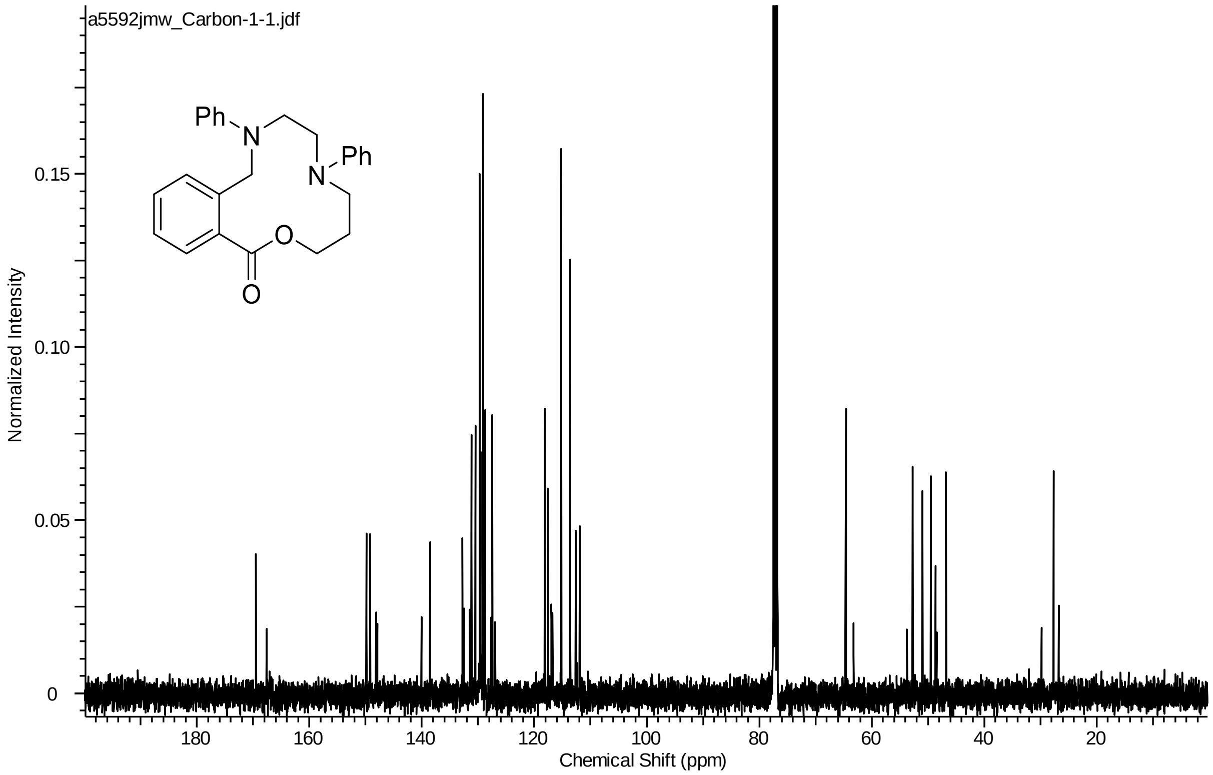

Methyl 2-((benzyl((1-(3-hydroxypropyl)piperidin-2-yl)methyl)amino)methyl)benzoate (S162)

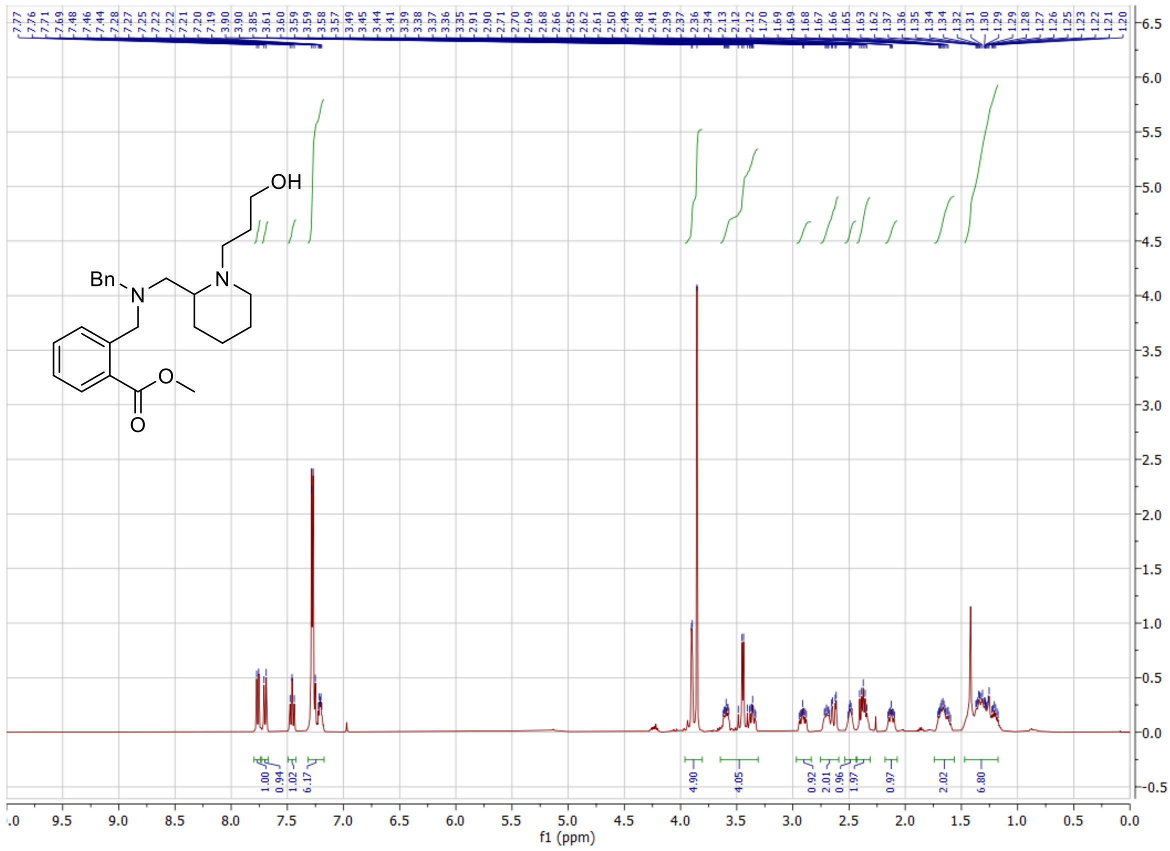

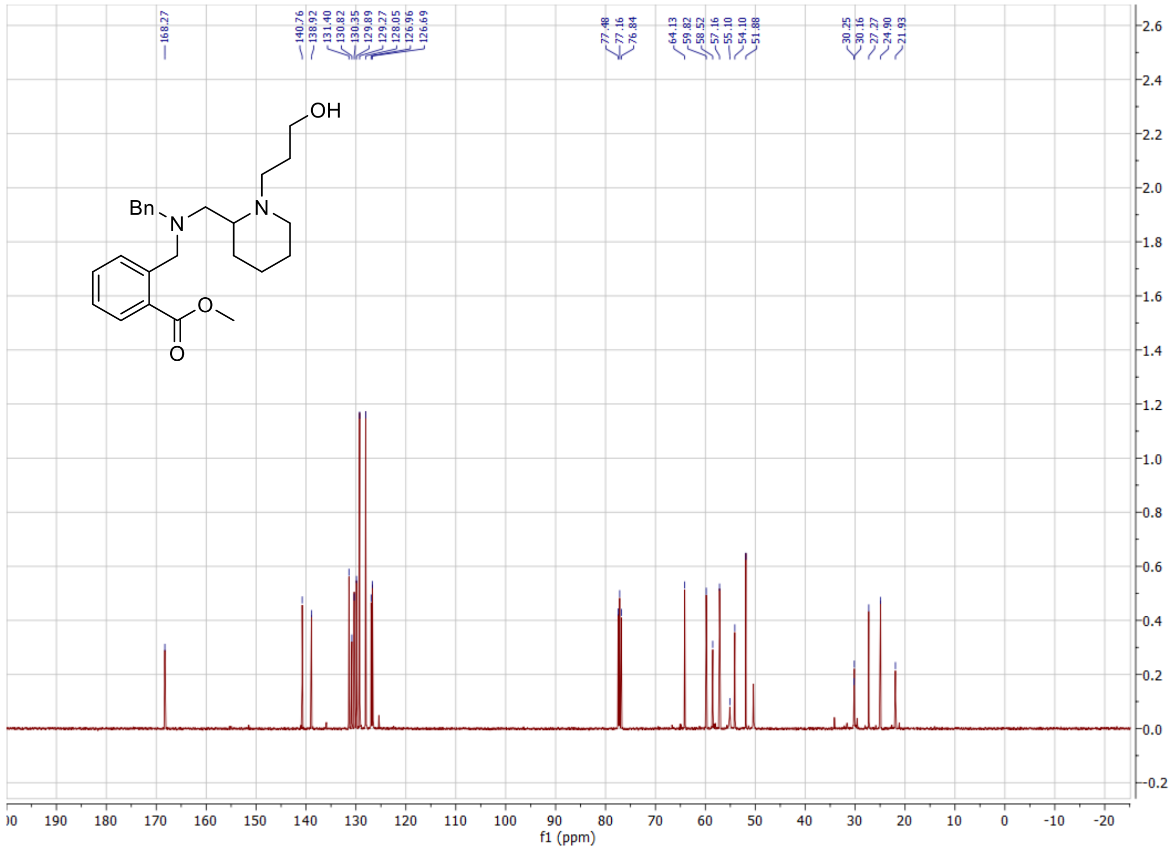

2-((Benzyl((1-(3-hydroxypropyl)piperidin-2-yl)methyl)amino)methyl)benzoic acid (S163)

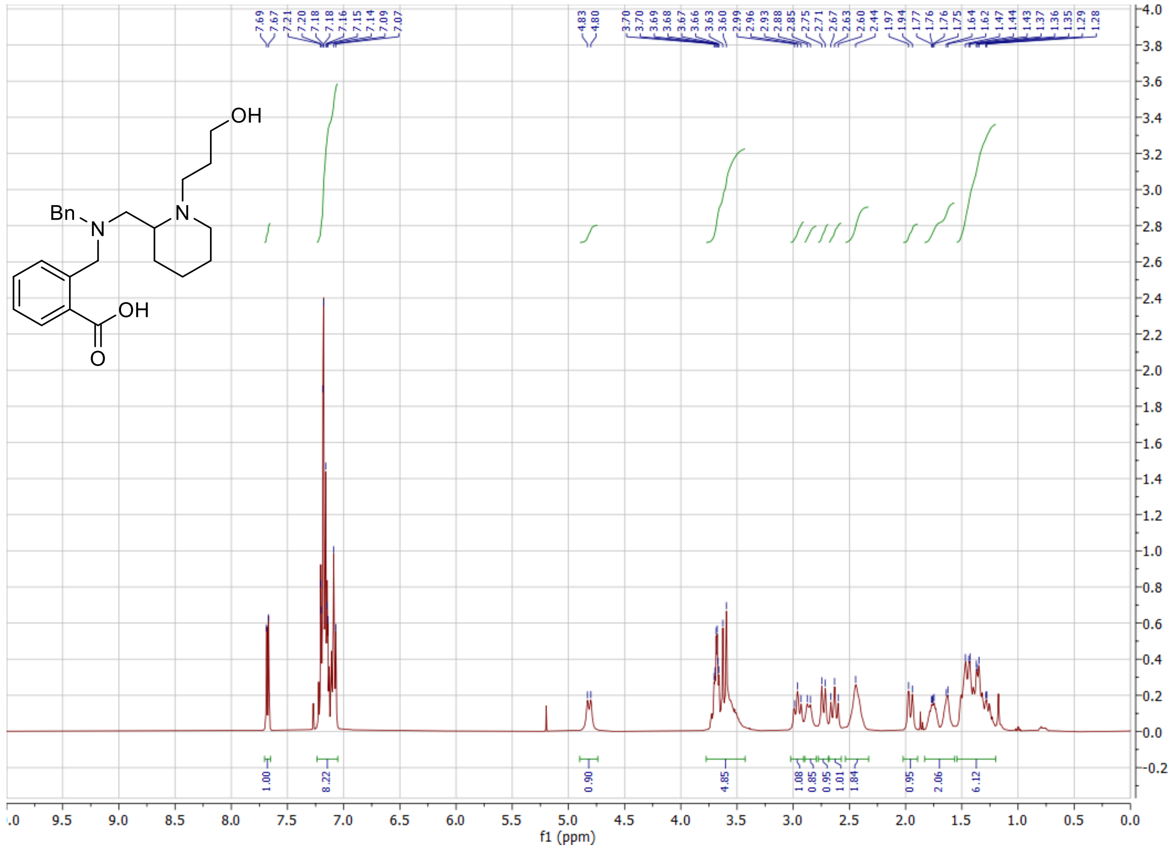

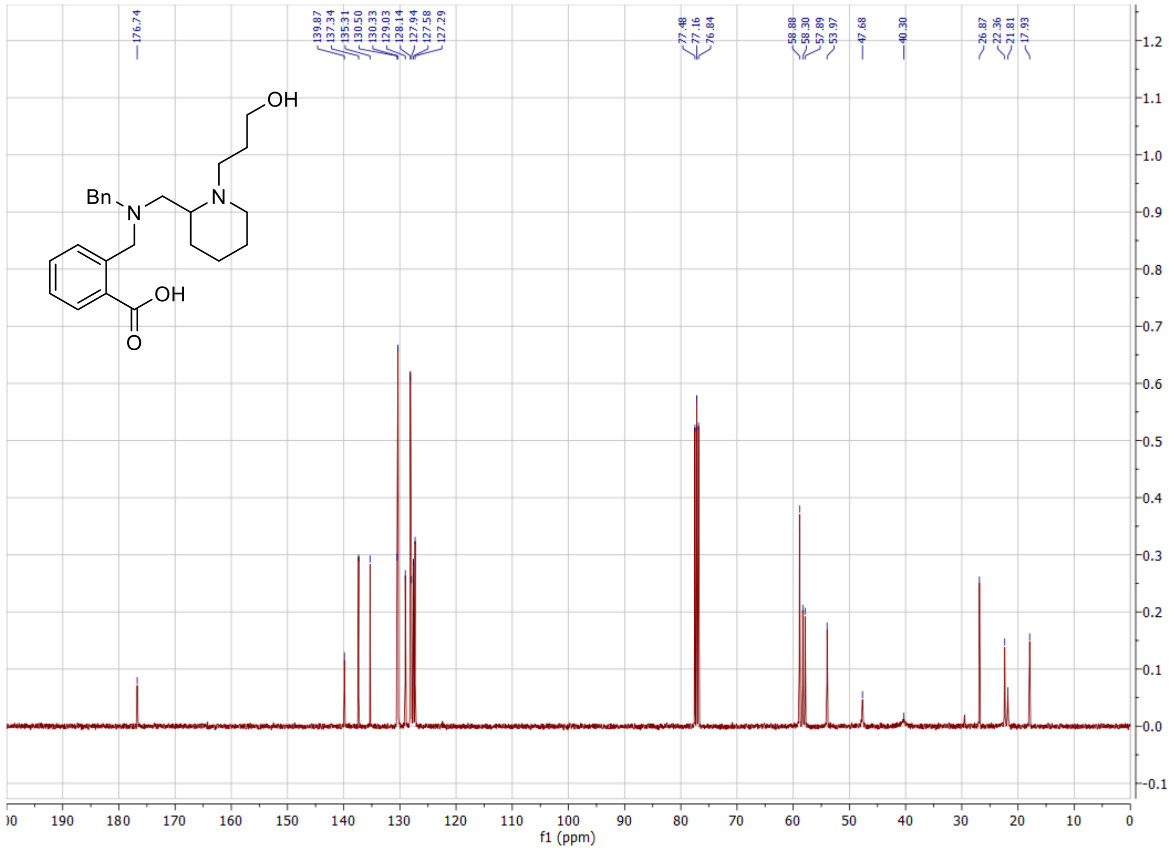

16-Benzyl-1,2,3,4,7,8,15,16,17,17a-decahydro-6H,10H-benzo[j]pyrido[1,2-e][1]oxa[5,8]diazacyclododecin-10-one (122)

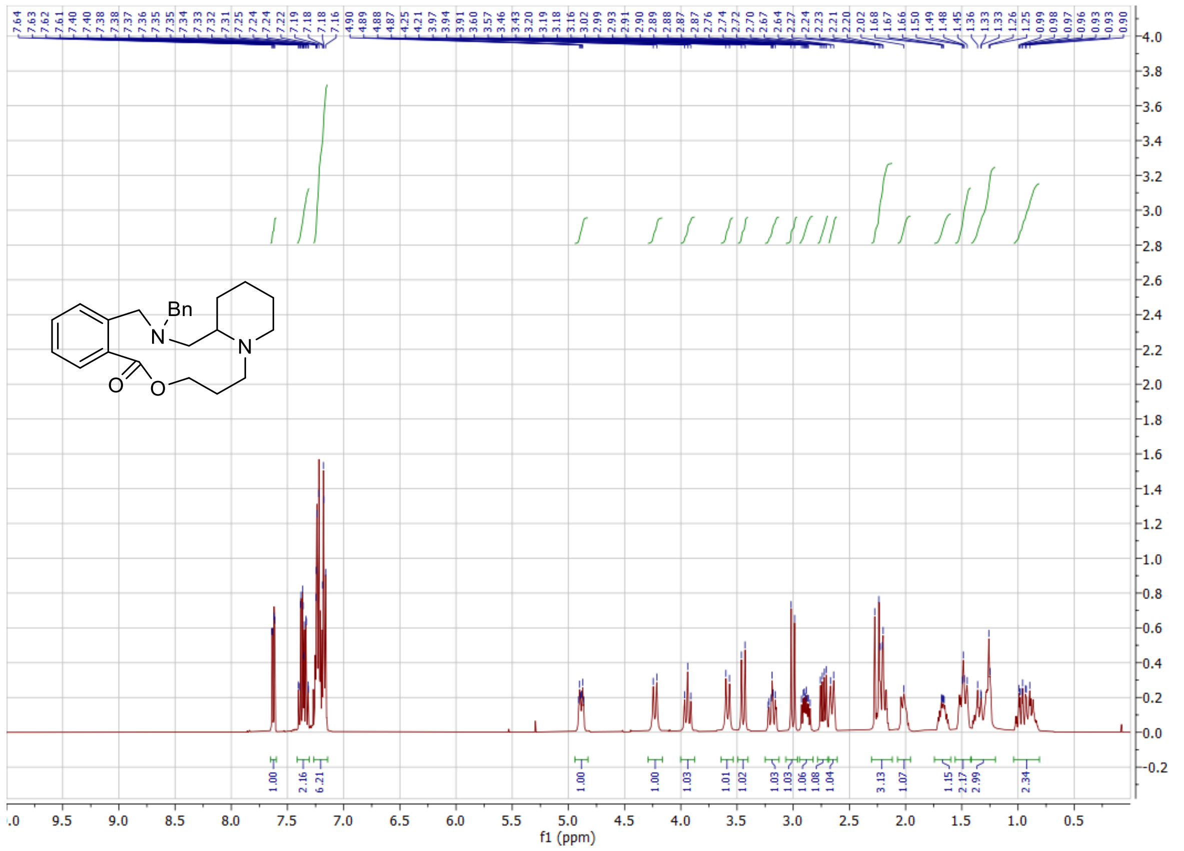

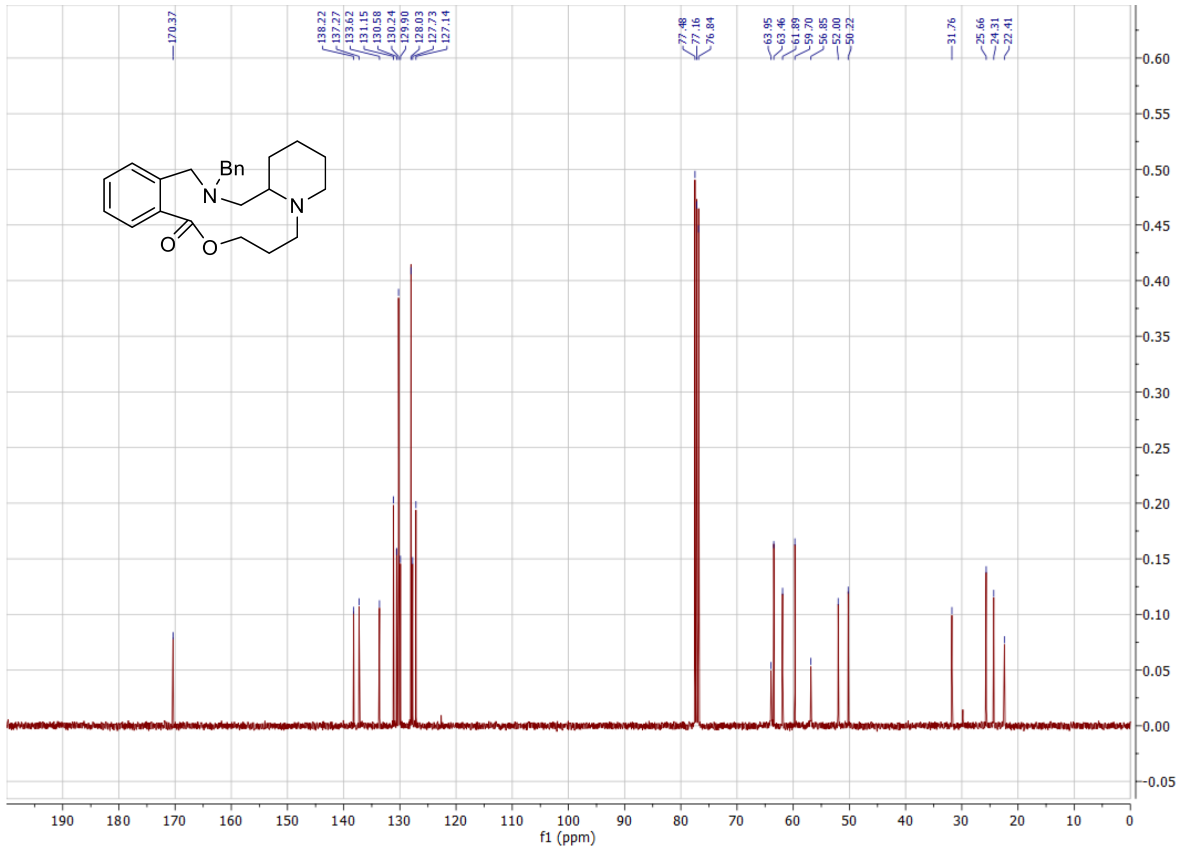

Methyl 2-(((3-bromopropyl)selanyl)methyl)benzoate (S164)

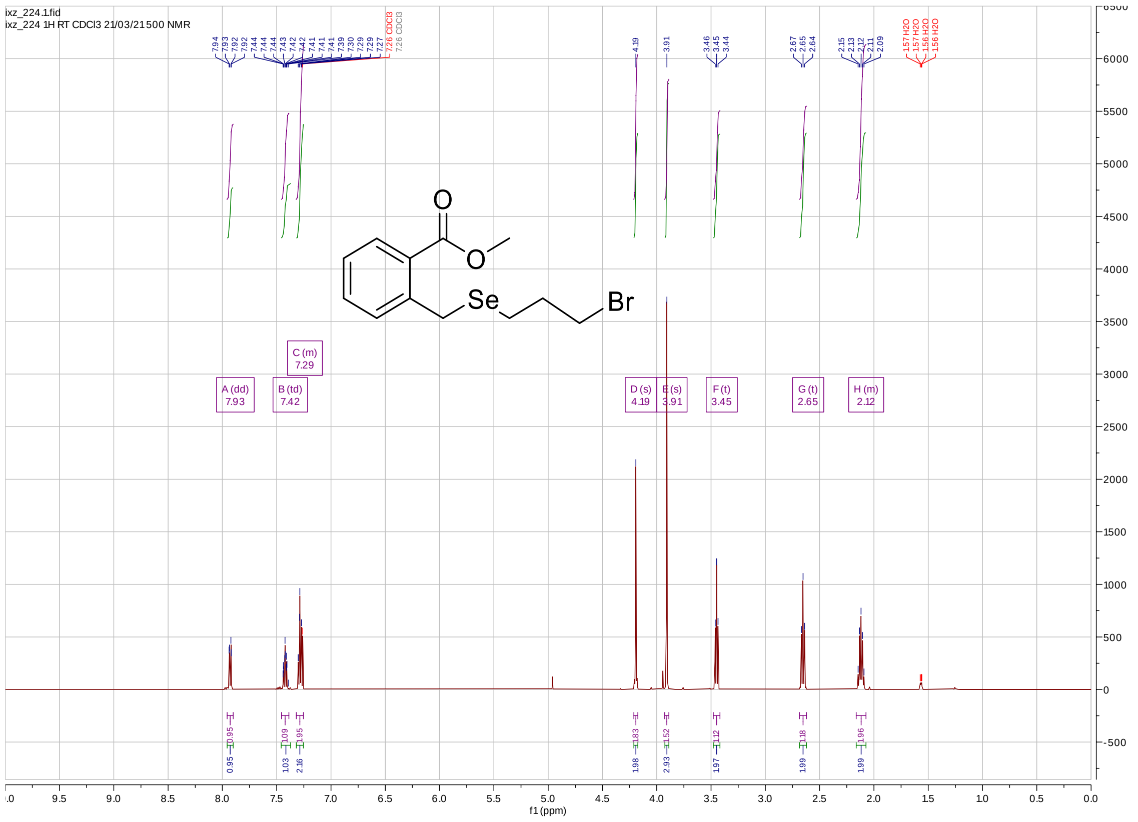

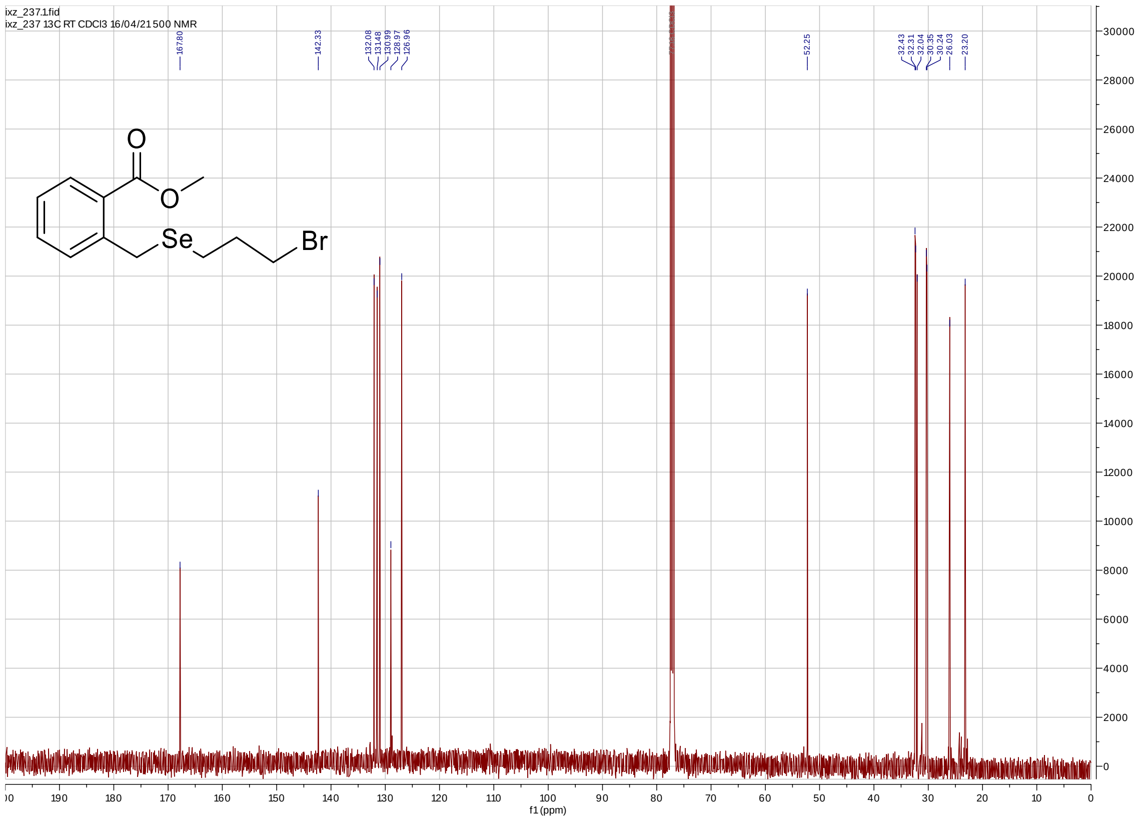

Methyl 2-(((3-(benzyl(3-hydroxypropyl)amino)propyl)selanyl)methyl)benzoate (S165)

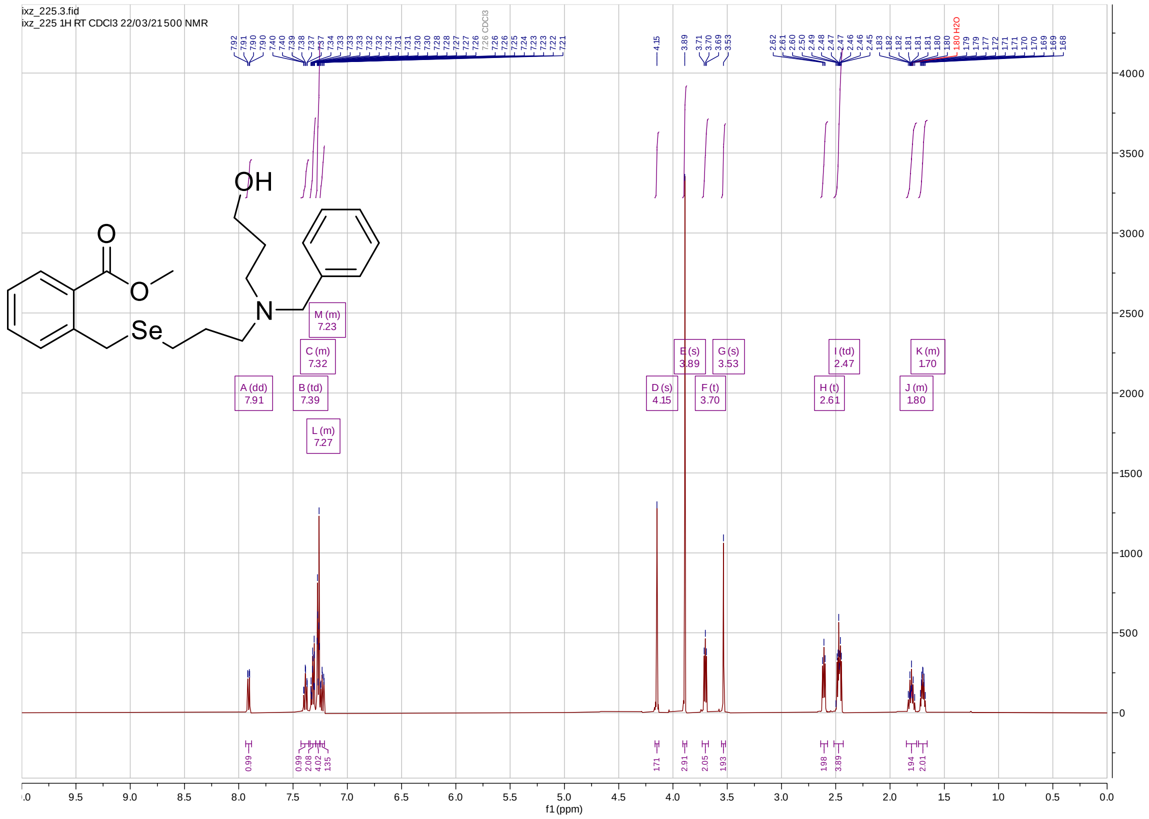

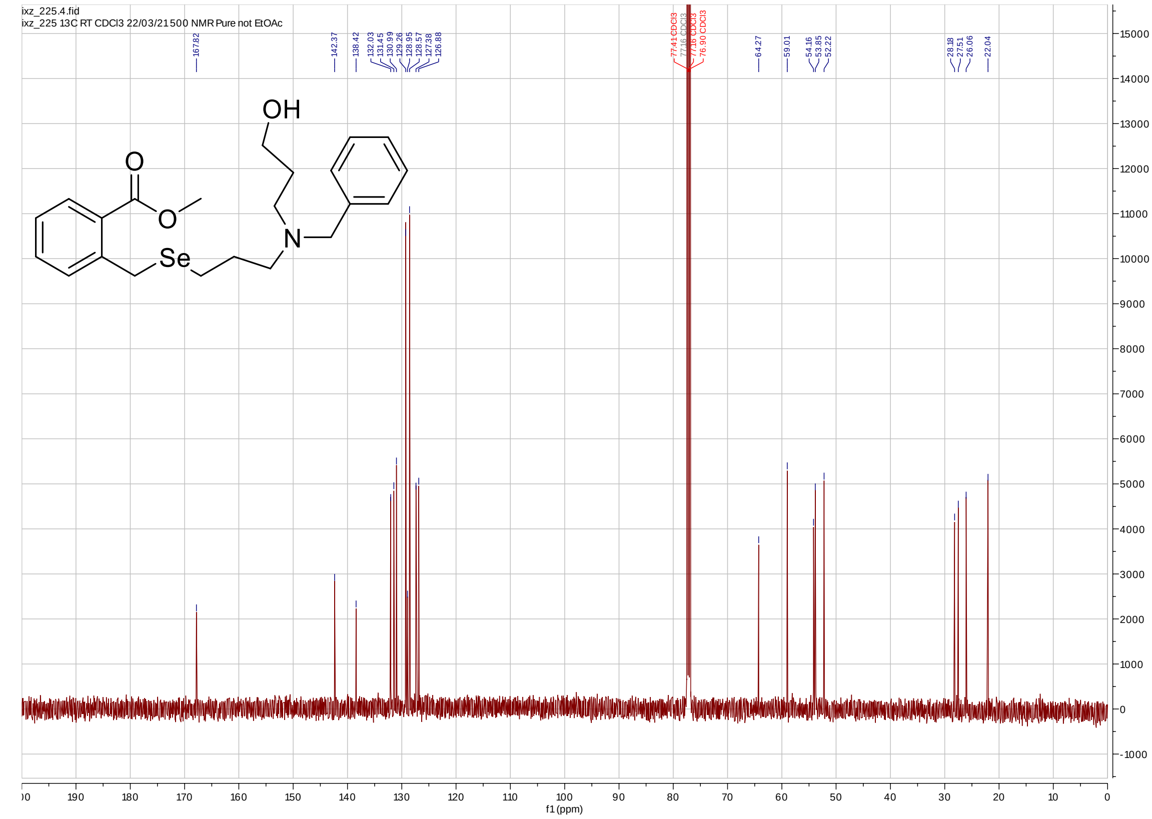

6-Benzyl-4,5,6,7,8,9-hexahydro-3H-benzo[k][1]oxa[9]selena[5]azacyclotridecin-1(11H)-one (123)

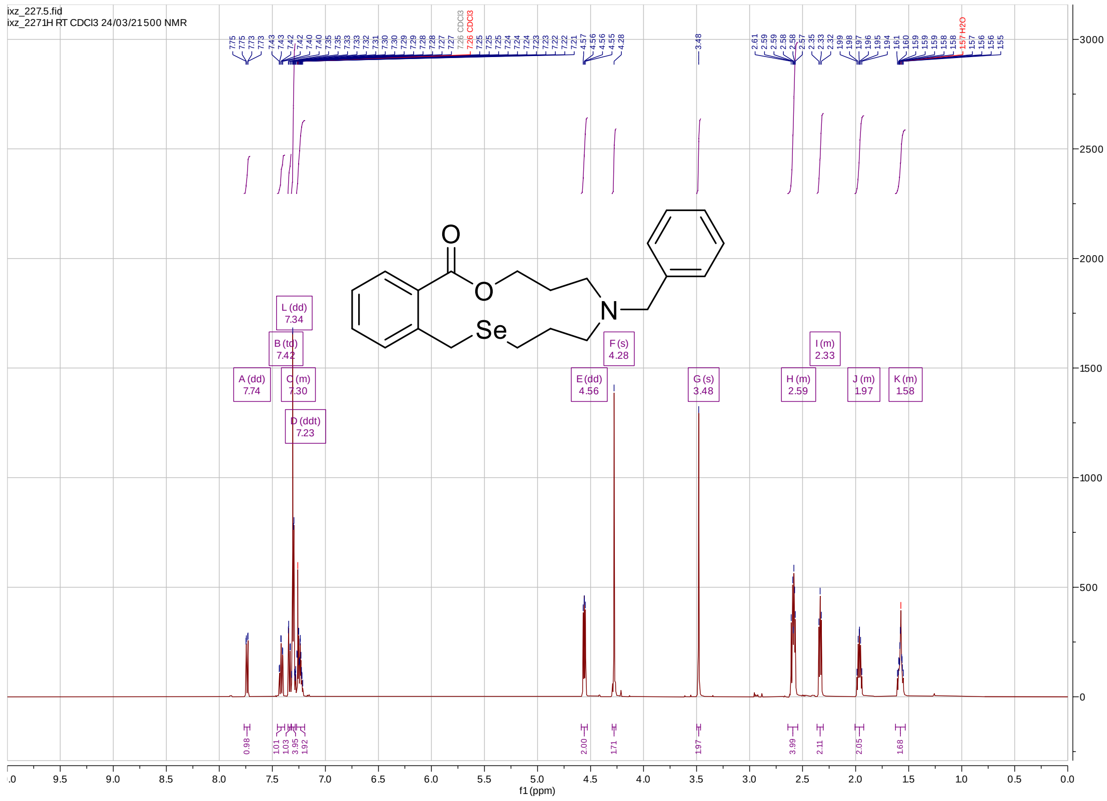

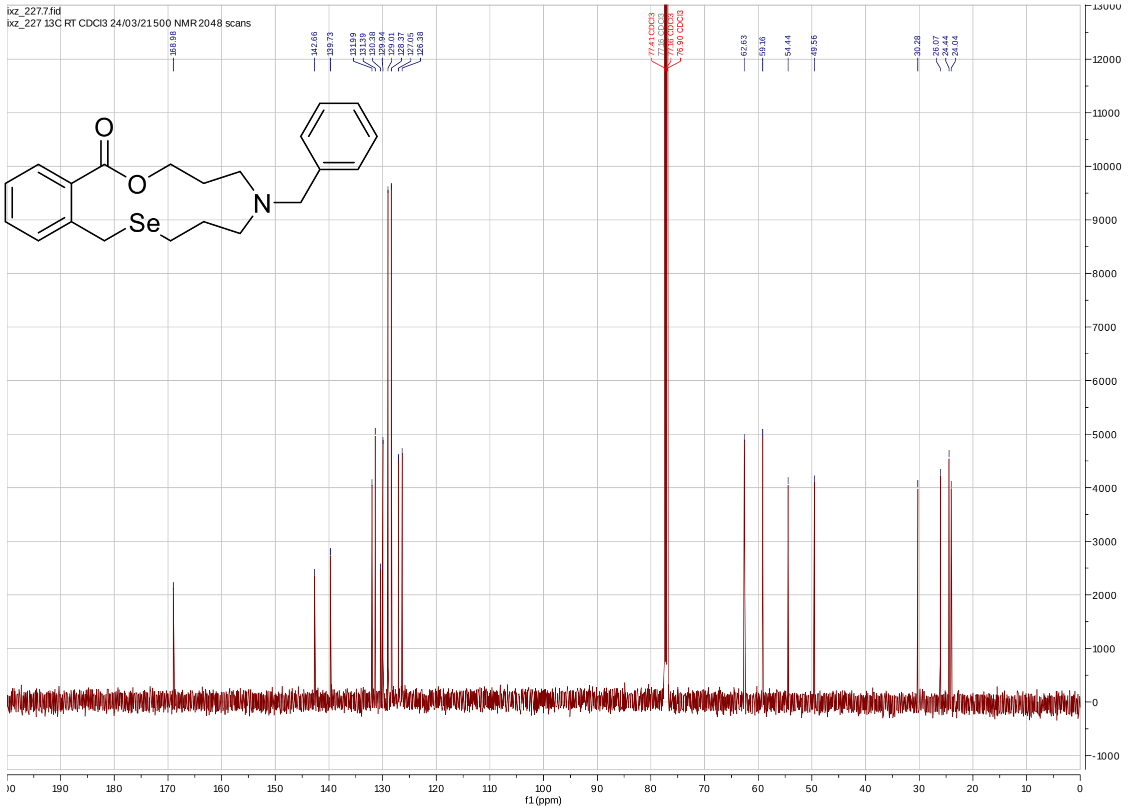

Methyl 2-(((3-((3-hydroxypropyl)thio)propyl)selanyl)methyl)benzoate (S166)

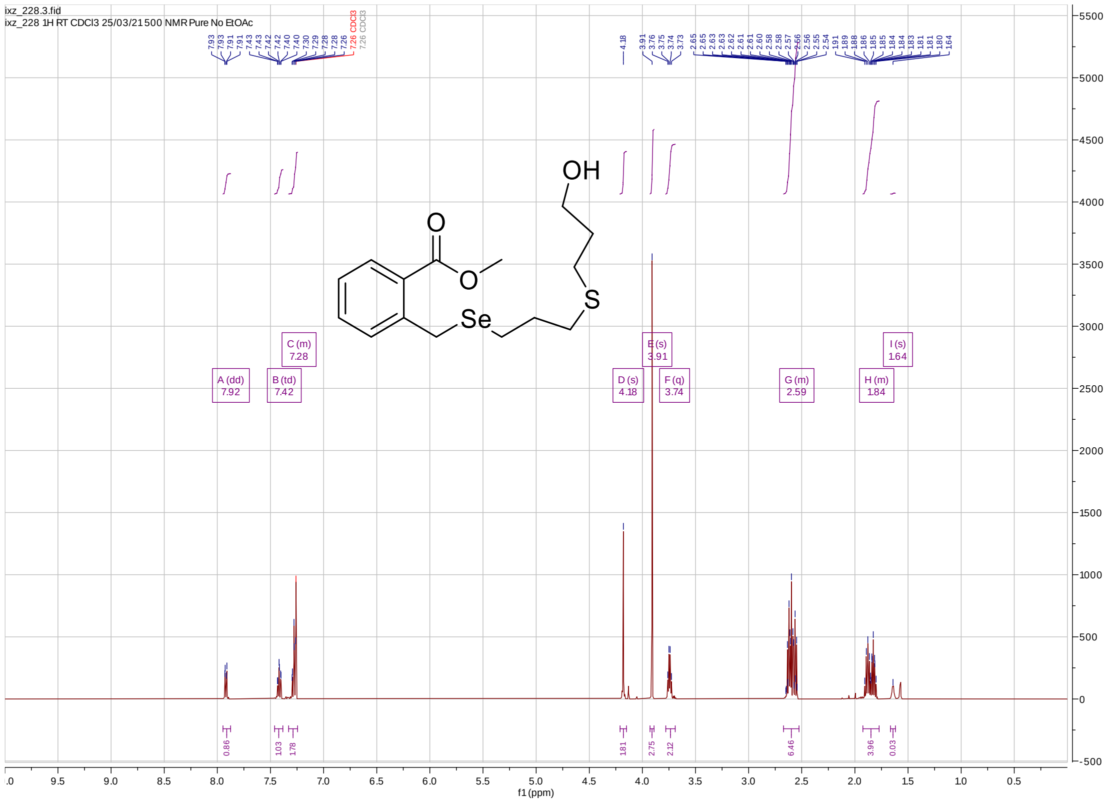

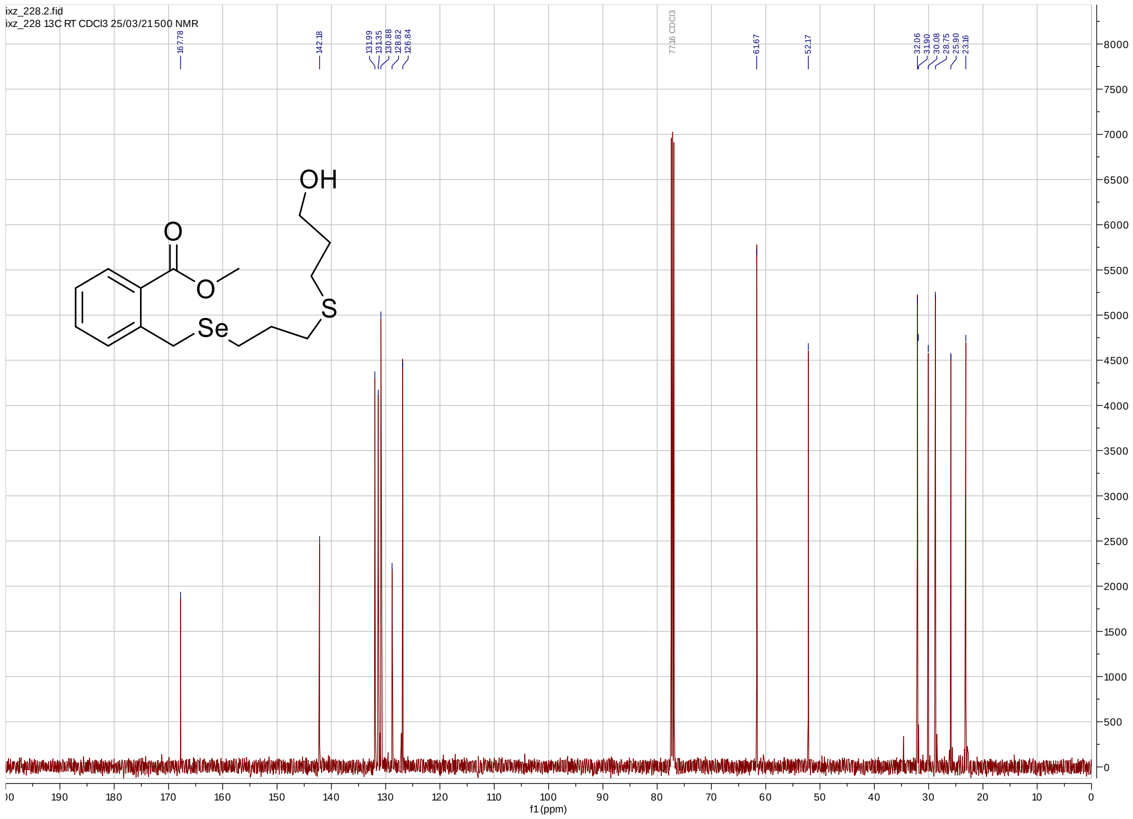

2-(((3-((3-Hydroxypropyl)thio)propyl)selanyl)methyl)benzoic acid (S167)

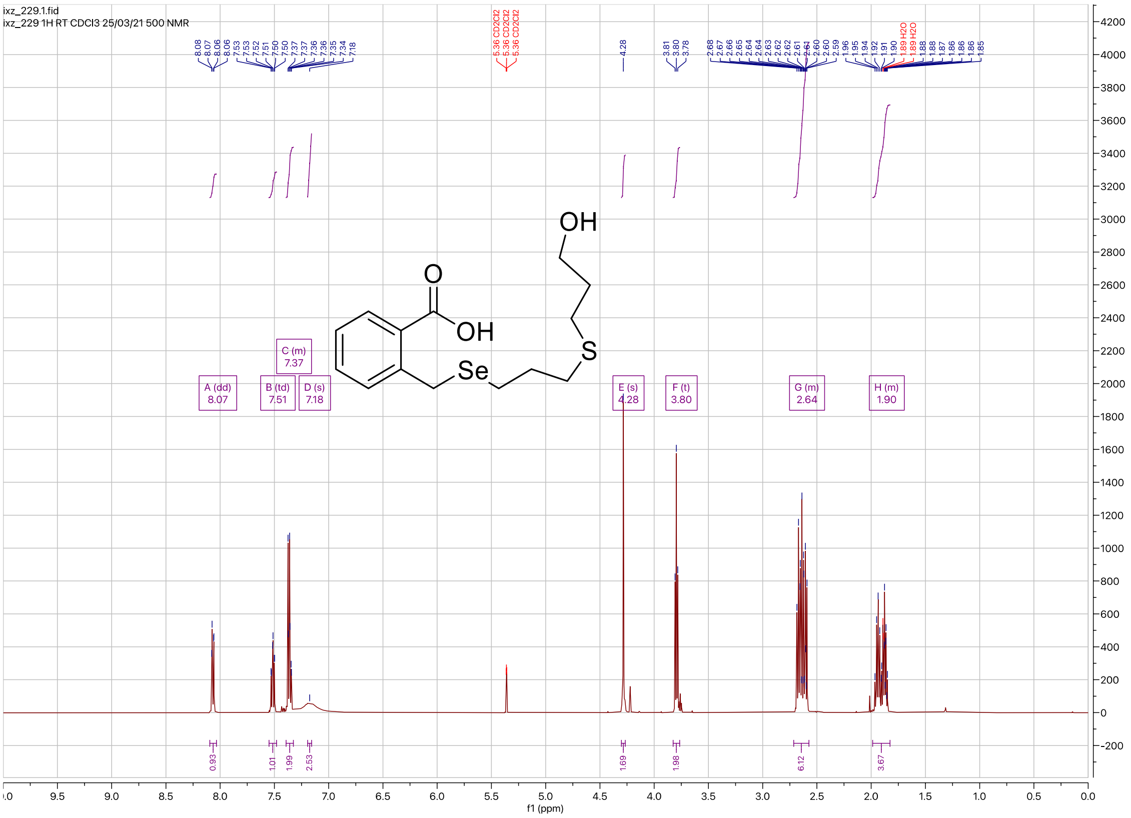

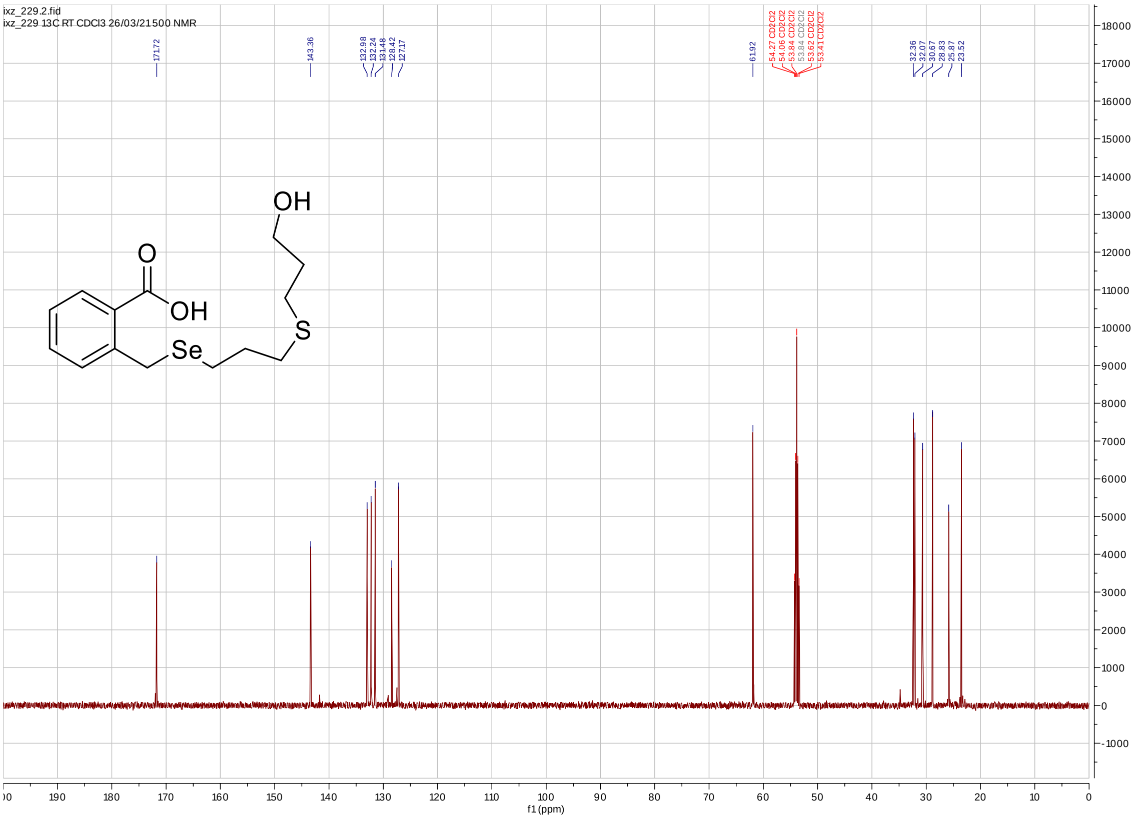

4,5,8,9-Tetrahydro-3H,7H-benzo[k][1]oxa[5]thia[9]selenacyclotridecin-1(11H)-one (124)

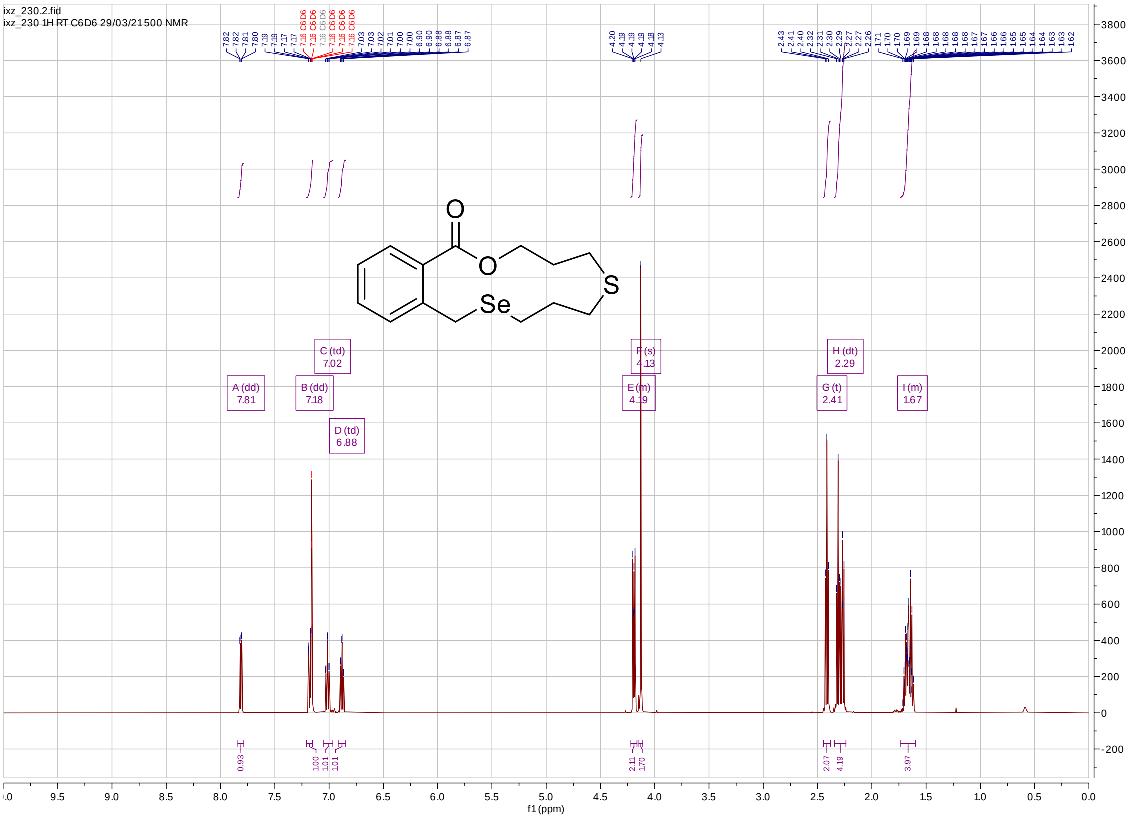

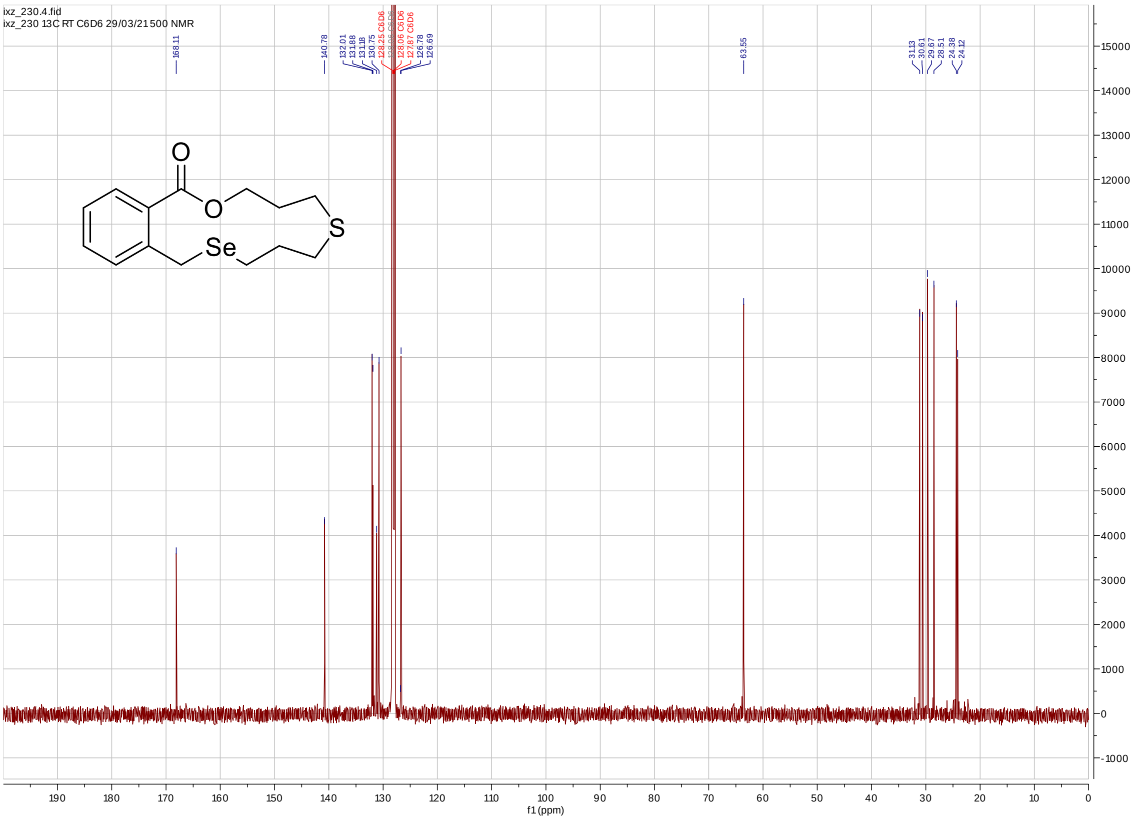

Methyl 2-((ethyl(3-(ethyl(3-hydroxypropyl) amino) propyl) amino) methyl) benzoate (S168)

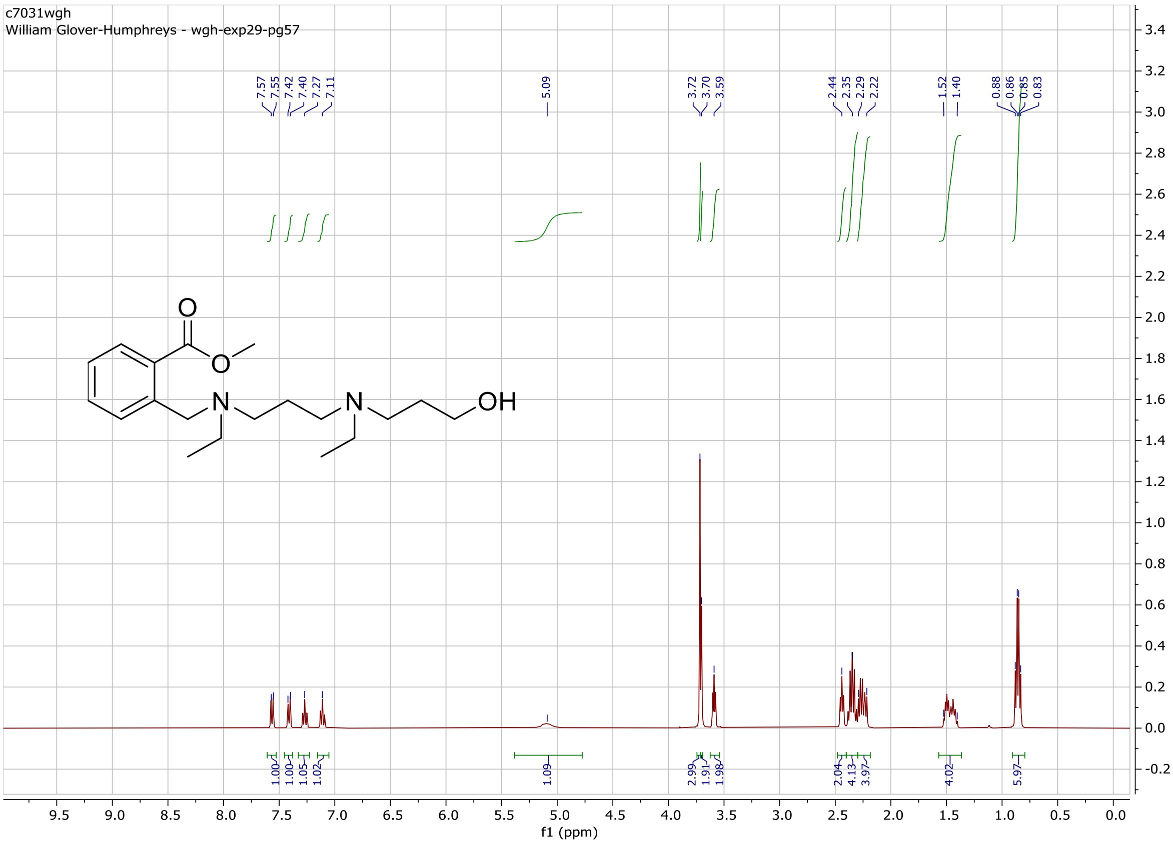

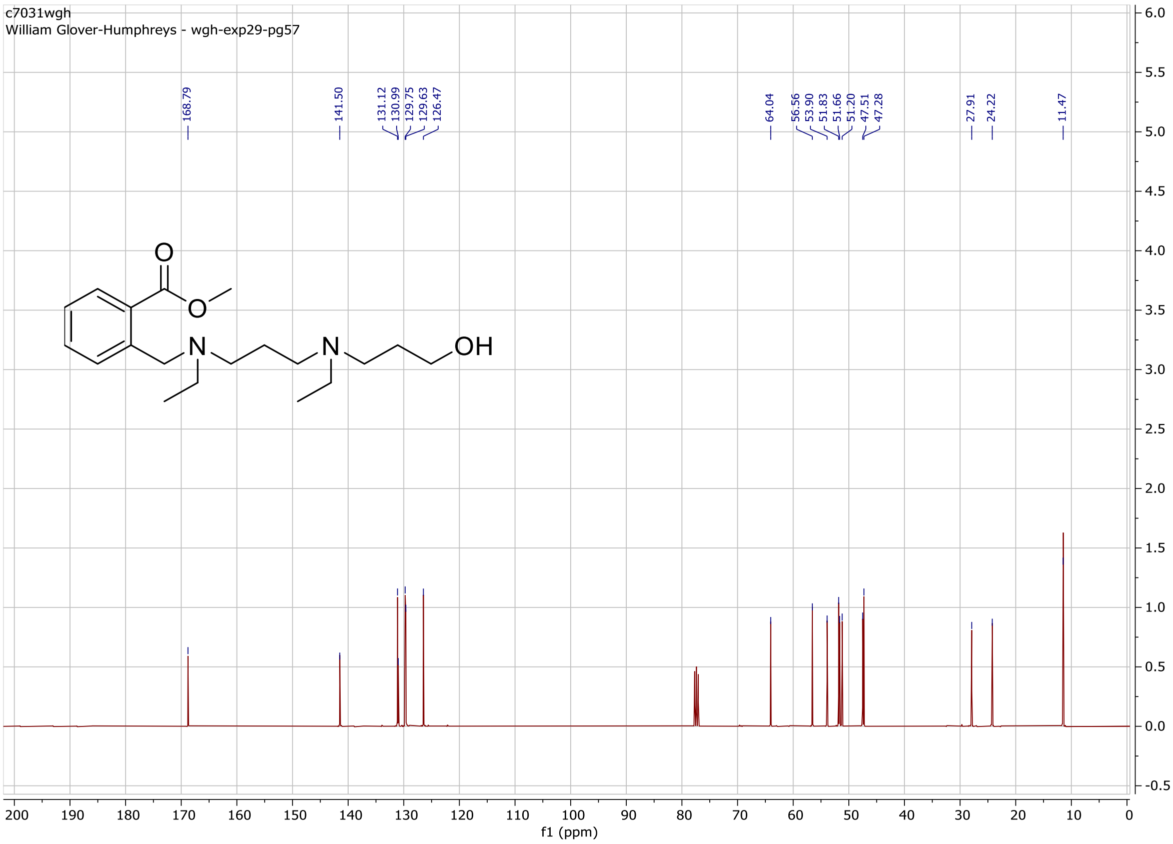

6,10-Diethyl-4,5,6,7,8,9,10,11-octahydrobenzo[k][1] oxa [5,9] diazacyclotridecin-1(3H)-one (125)

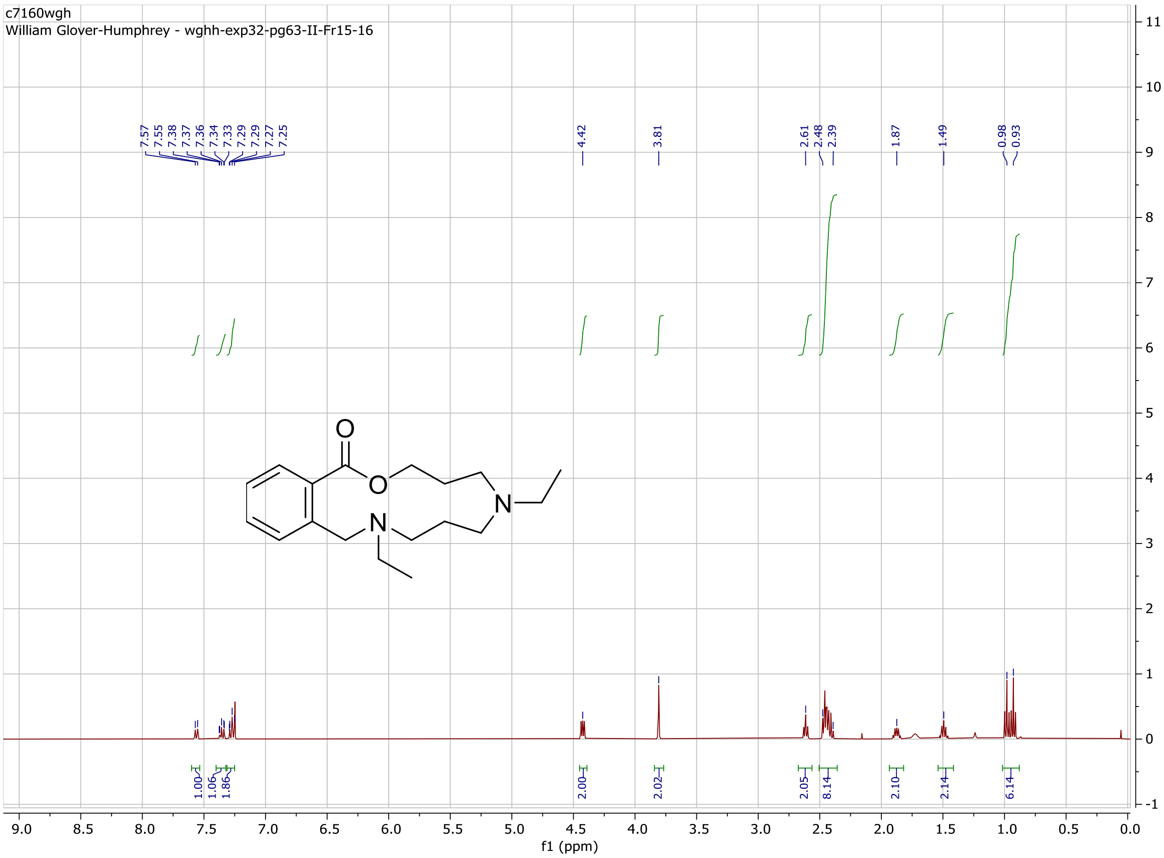

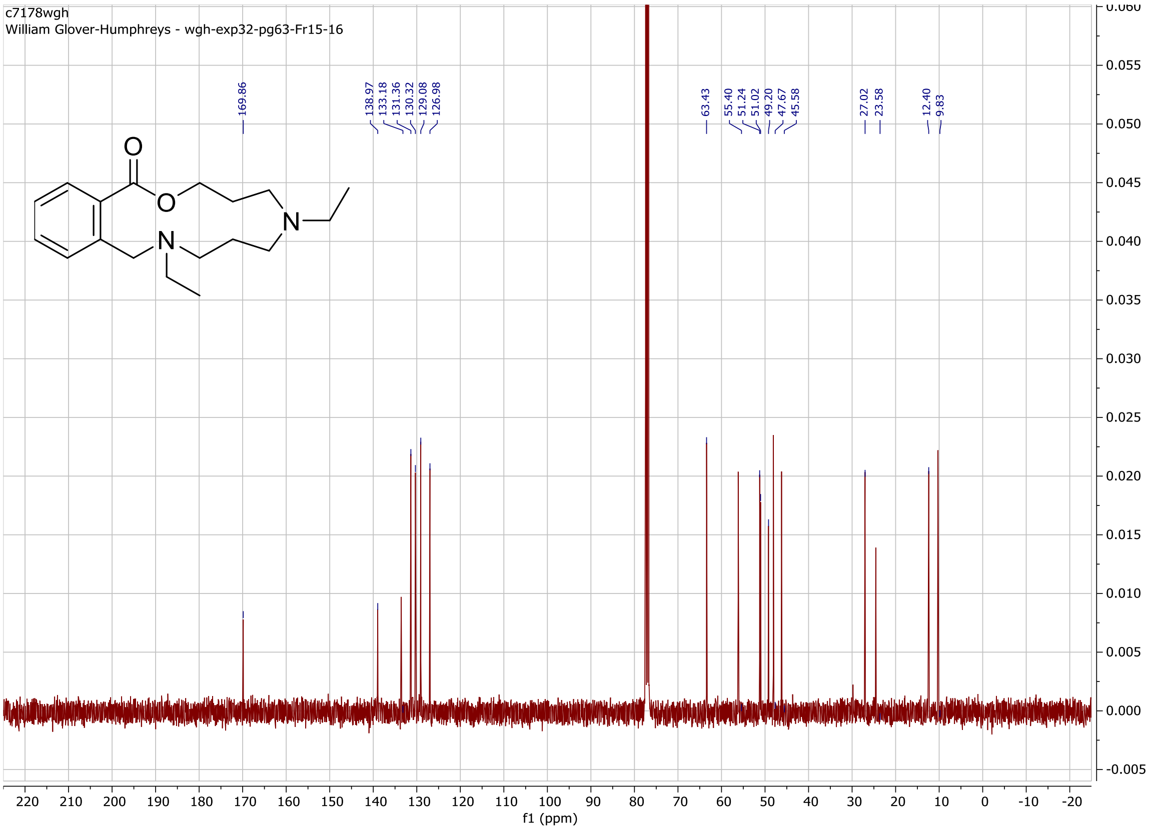

Methyl 2-(((3-((3-mercaptopropyl)thio)propyl)thio)methyl)benzoate (S169)

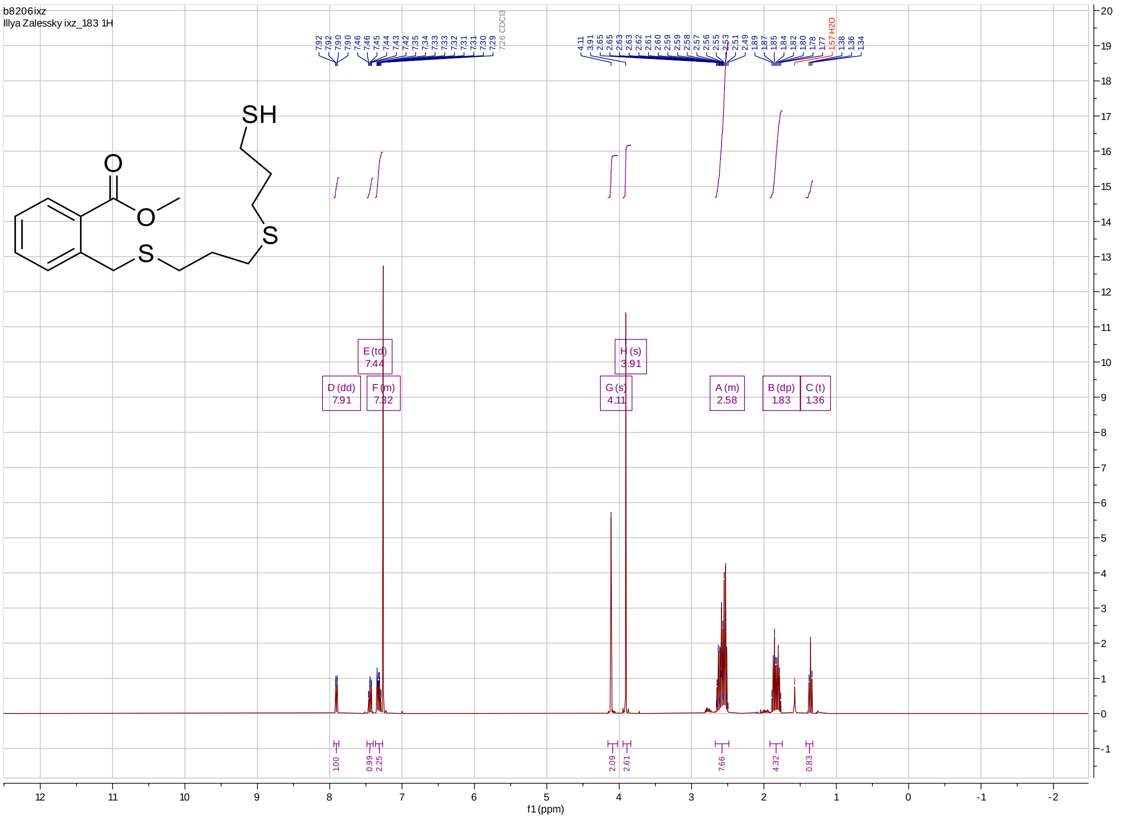

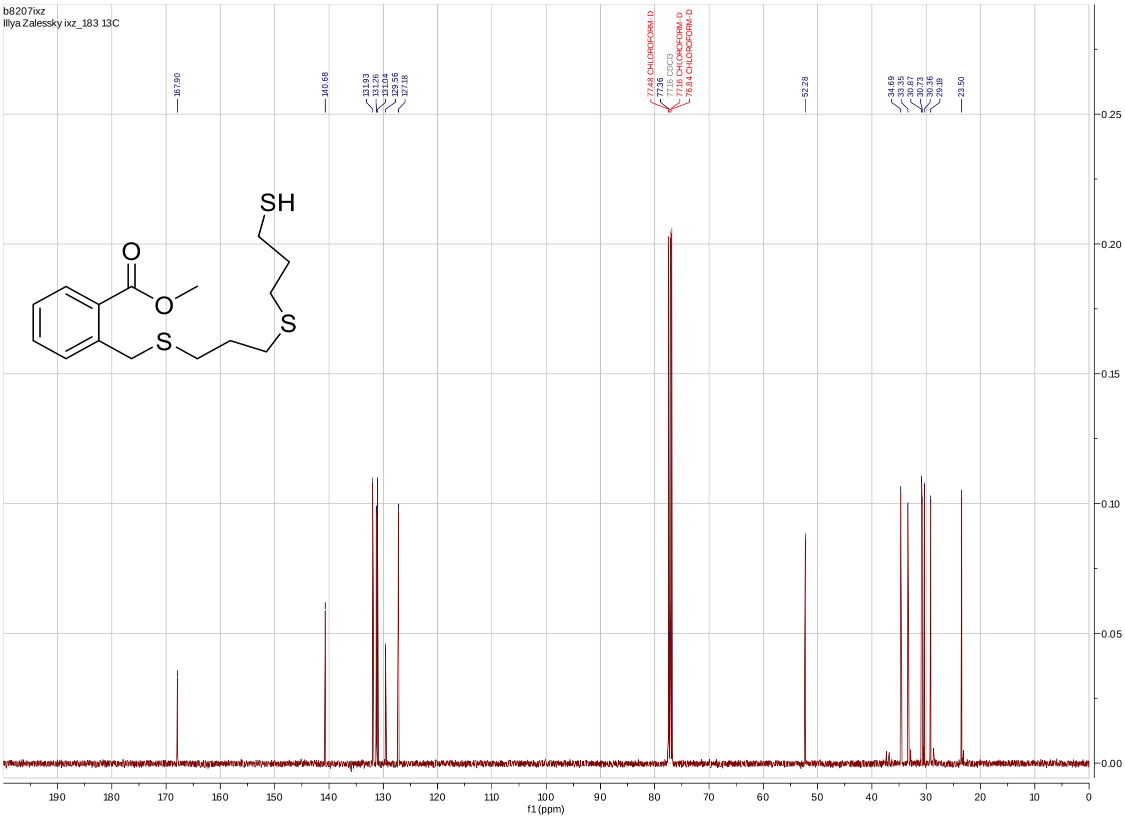

2-(((3-((3-Mercaptopropyl)thio)propyl)thio)methyl)benzoic acid (S170)

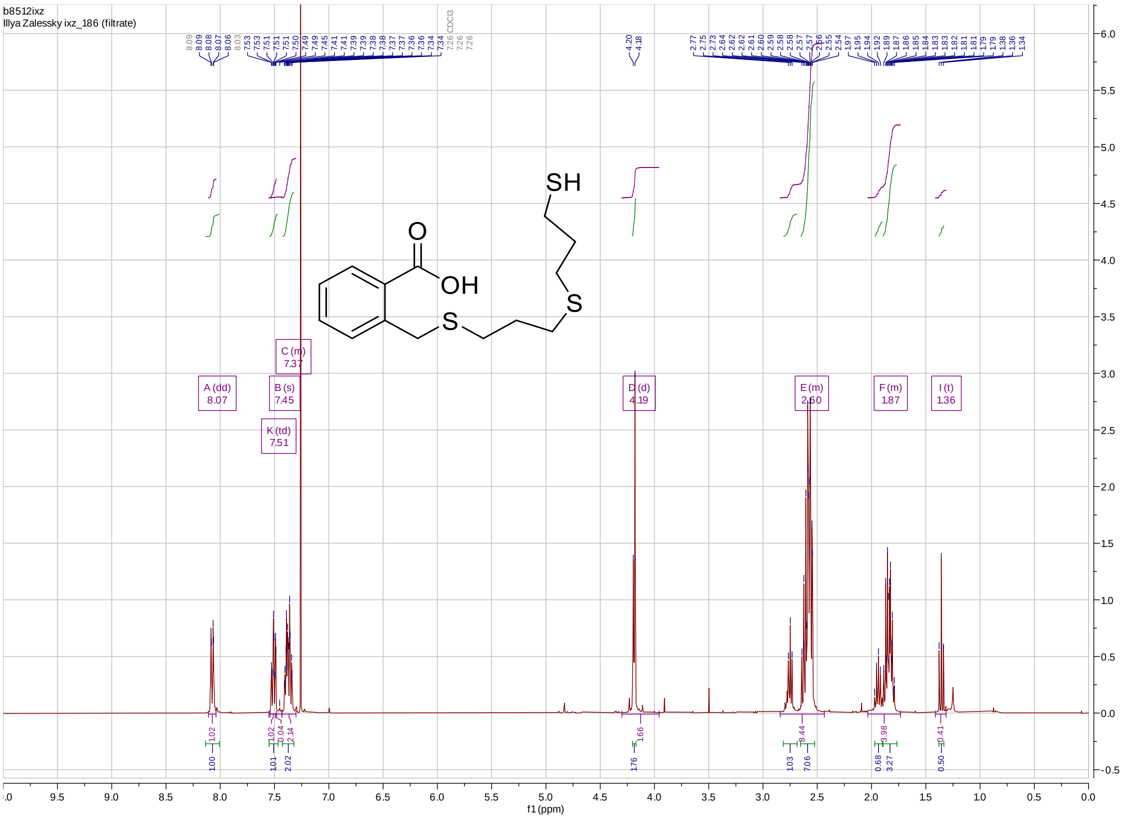

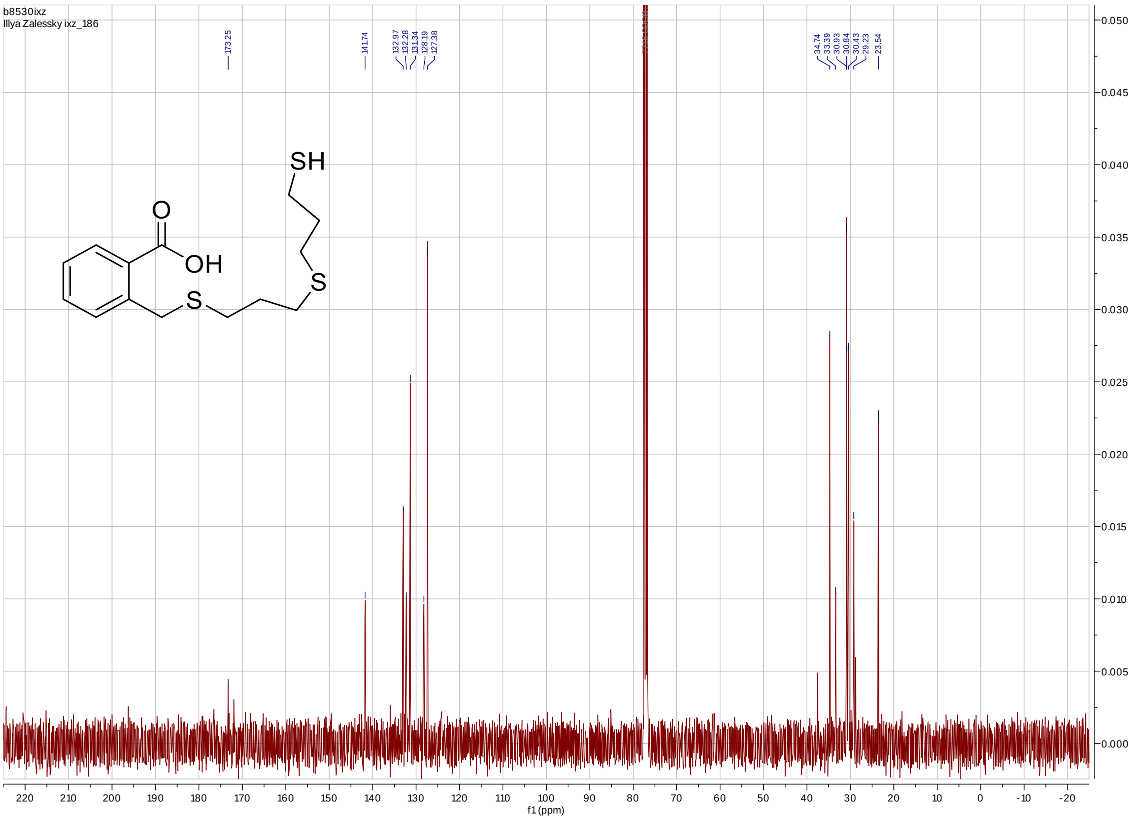

4,5,8,9-Tetrahydro-3H,7H-benzo[k][1,5,9]trithiacyclotridecin-1(11H)-one (126)

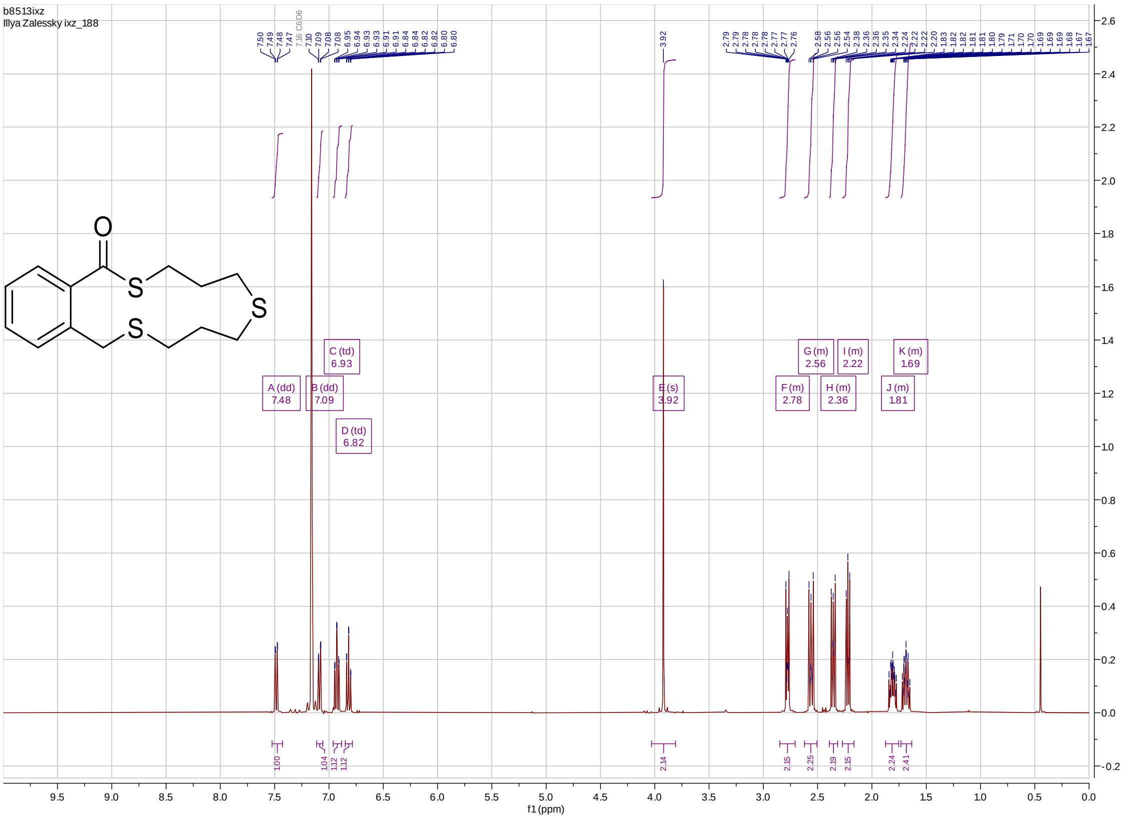

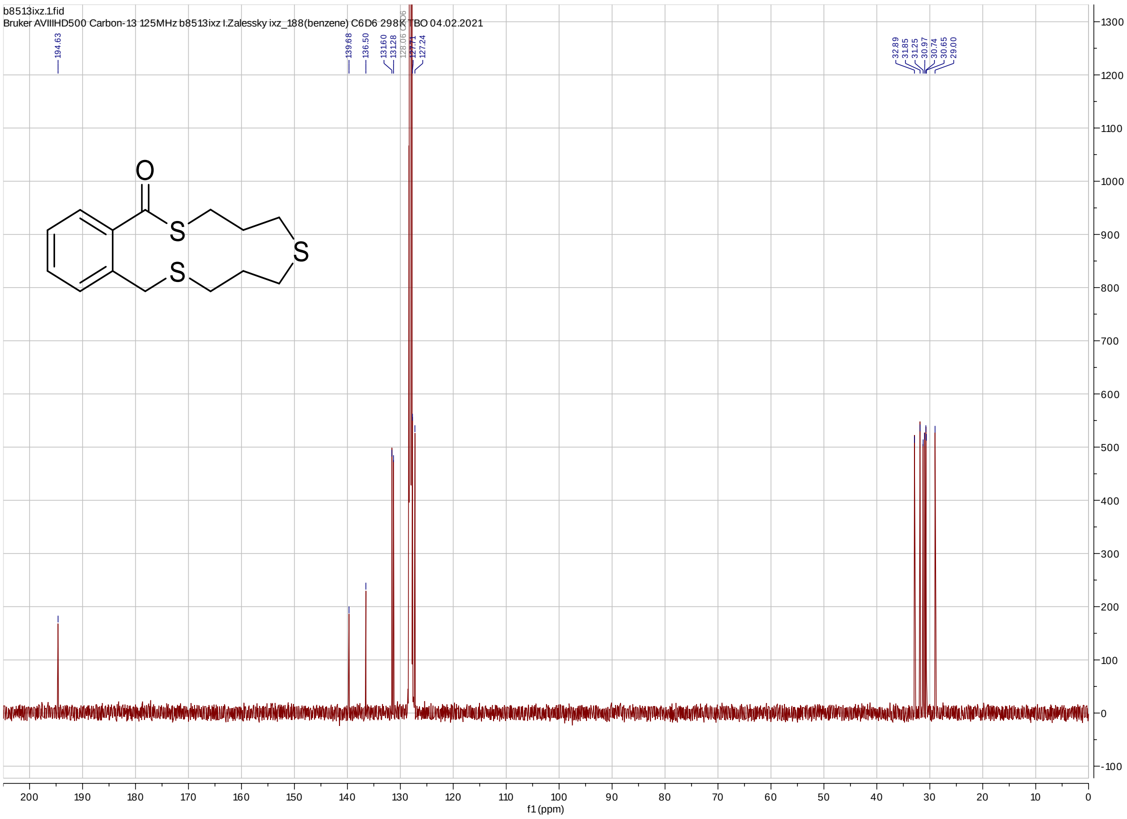

3,3'-(1,2-Phenylenebis(methylazanediyl))bis(propan-1-ol) (S171)

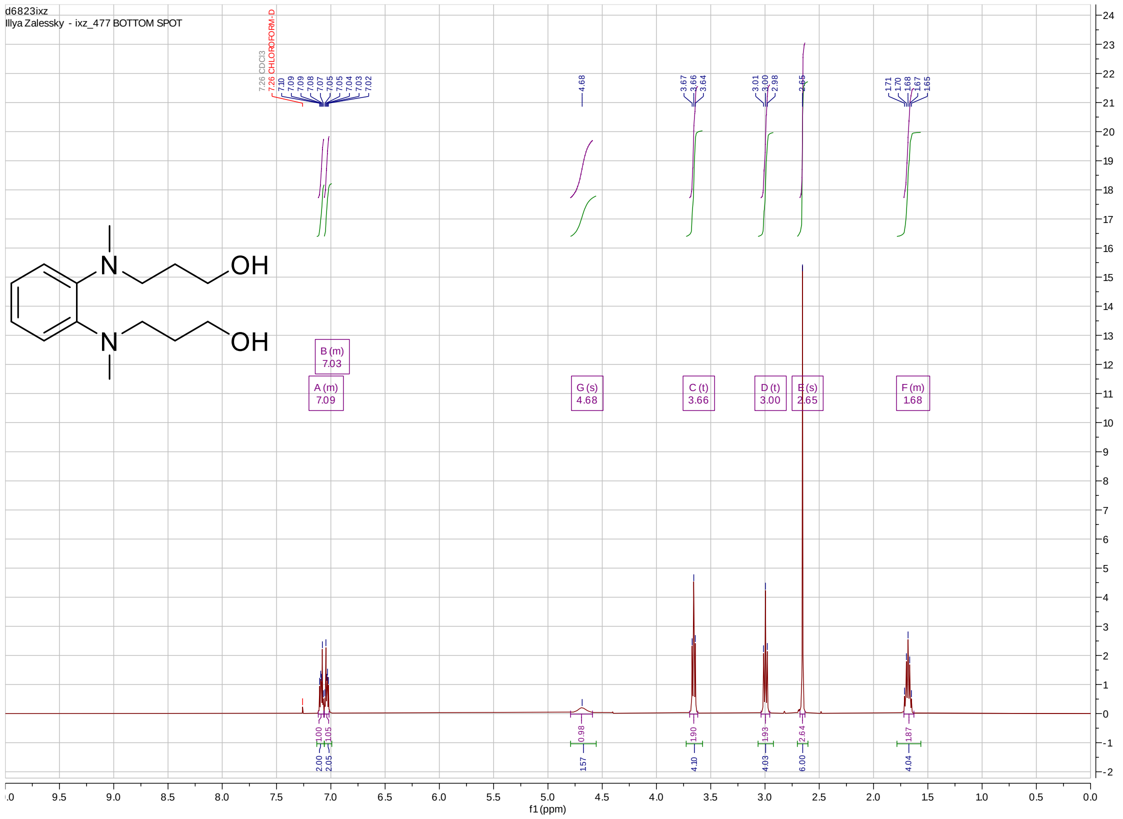

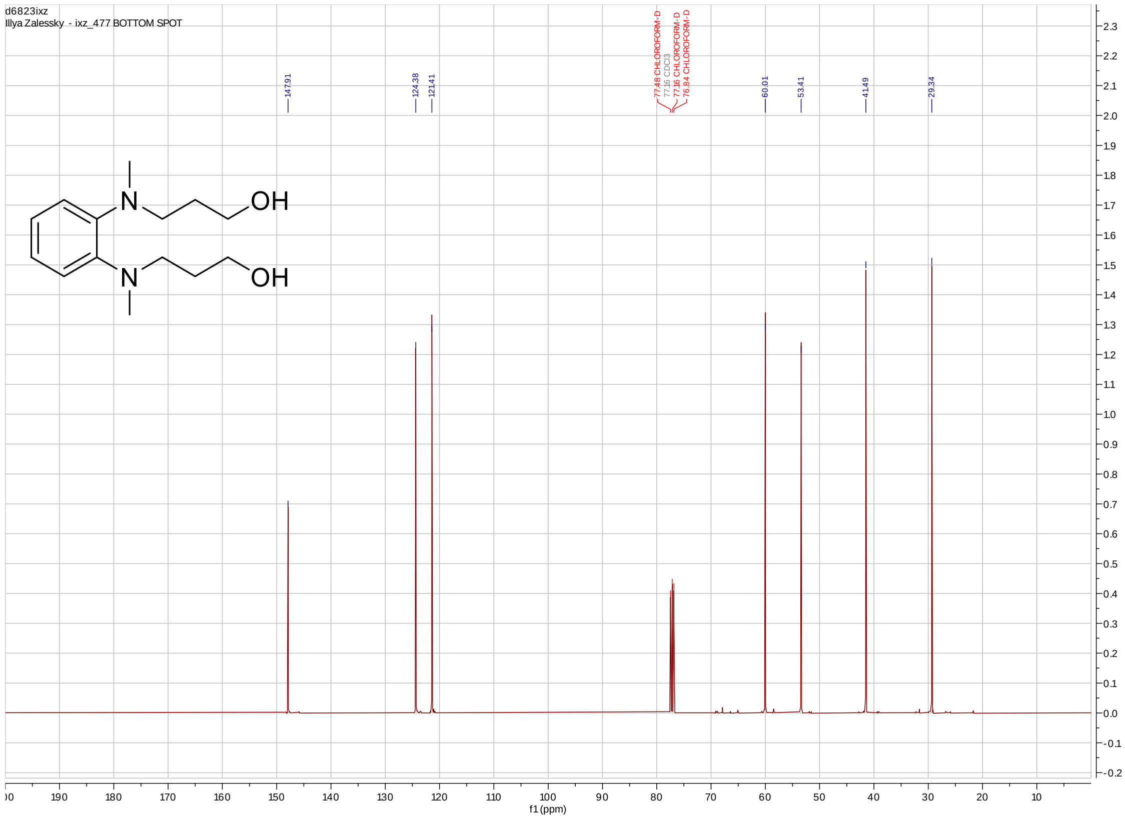

1,11-Dimethyl-6-phenyl-1,2,3,4,8,9,10,11-octahydrobenzo[h][1,3]dioxa[7,10]diaza[2]phosphacyclotridecine 6-oxide (127)

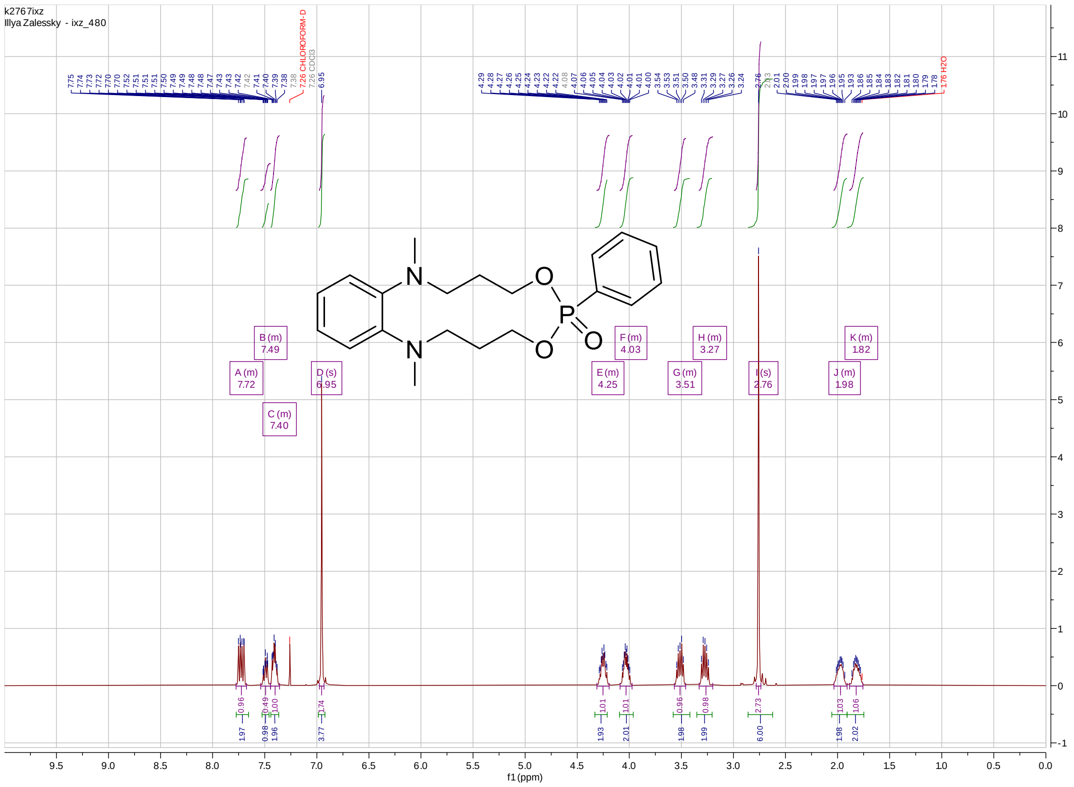

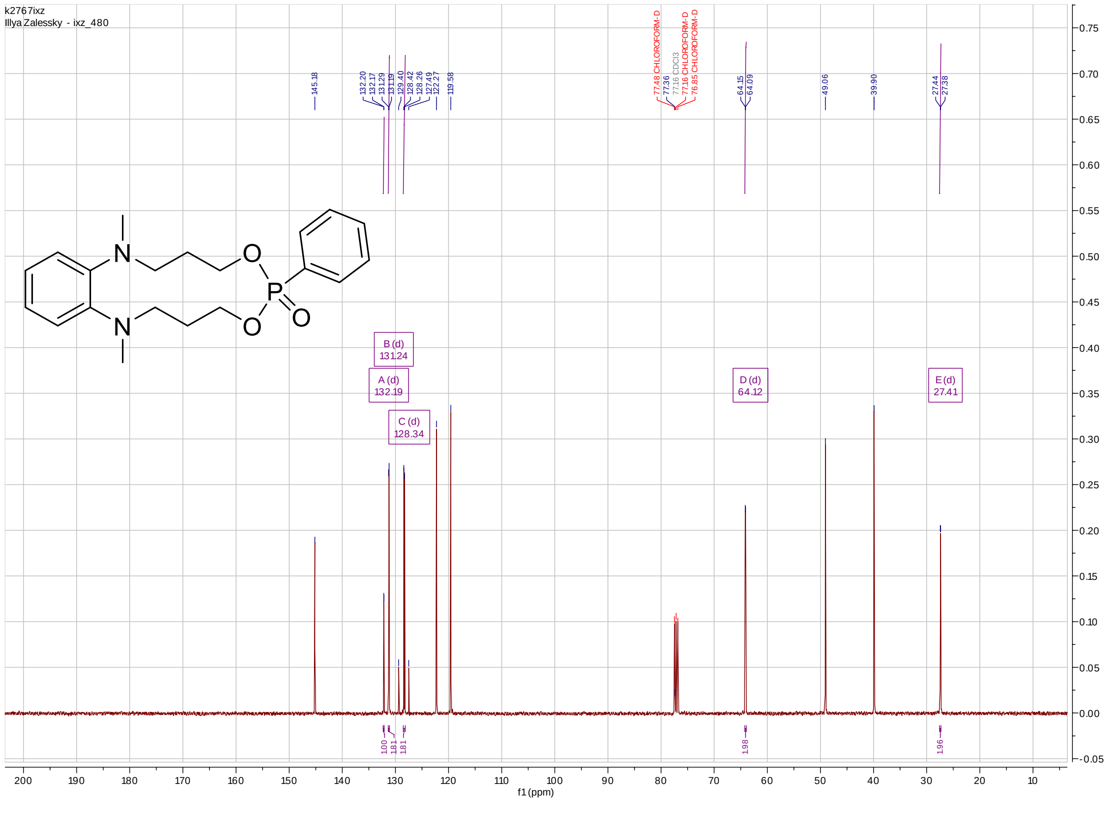

1,11-Dimethyl-6-phenyl-1,2,3,4,8,9,10,11-octahydrobenzo[h][1,3]dioxa[7,10]diaza[2]phosphacyclotridecine 6-oxide (127)- continued

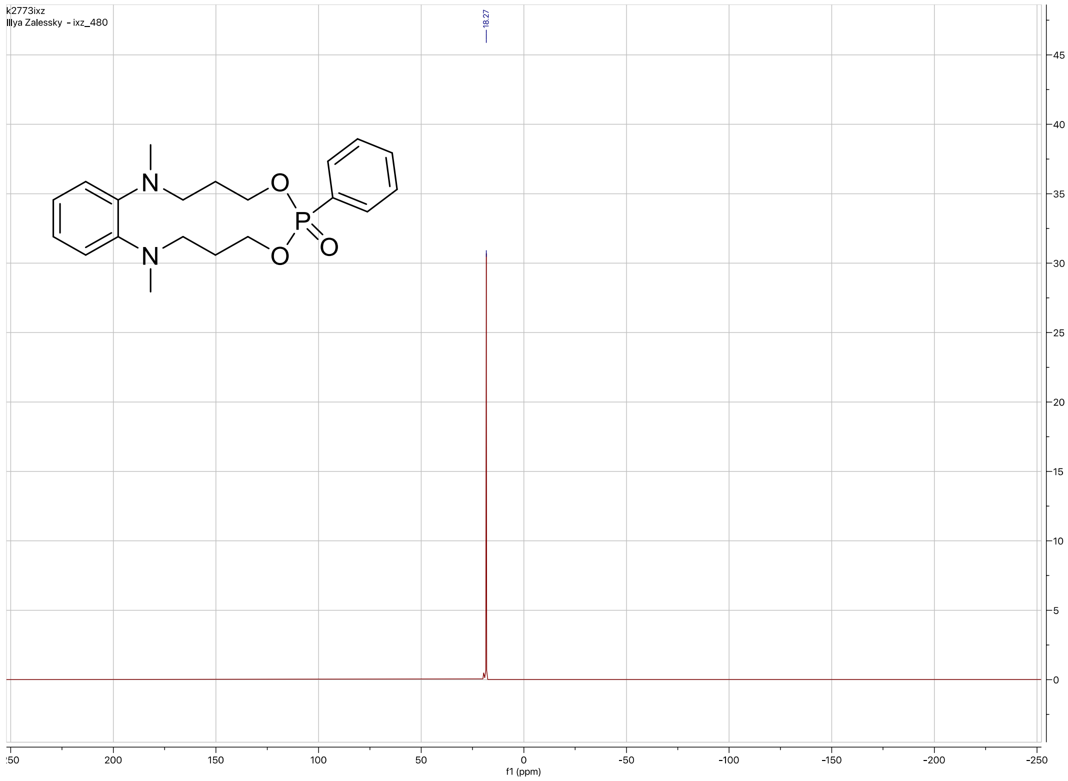

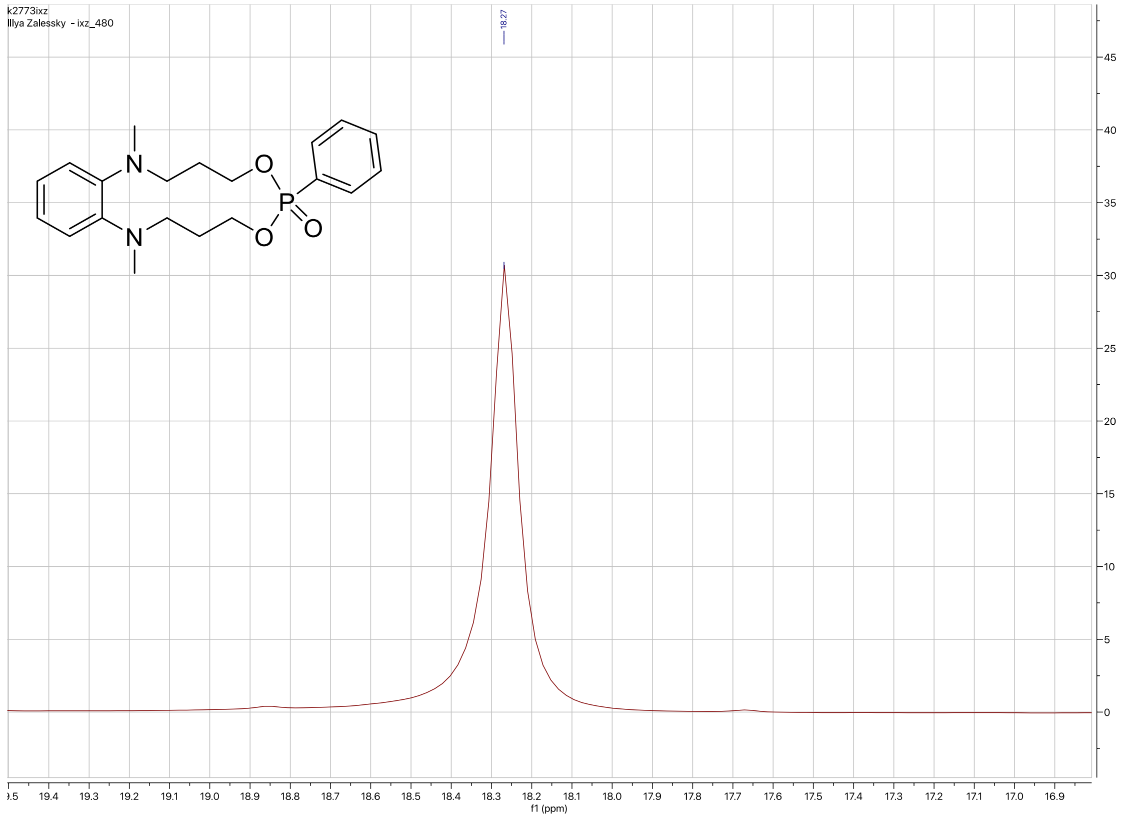

***N*,*N*'-Bis(salicylidene)-*o*-phenylenediamine (S172)**

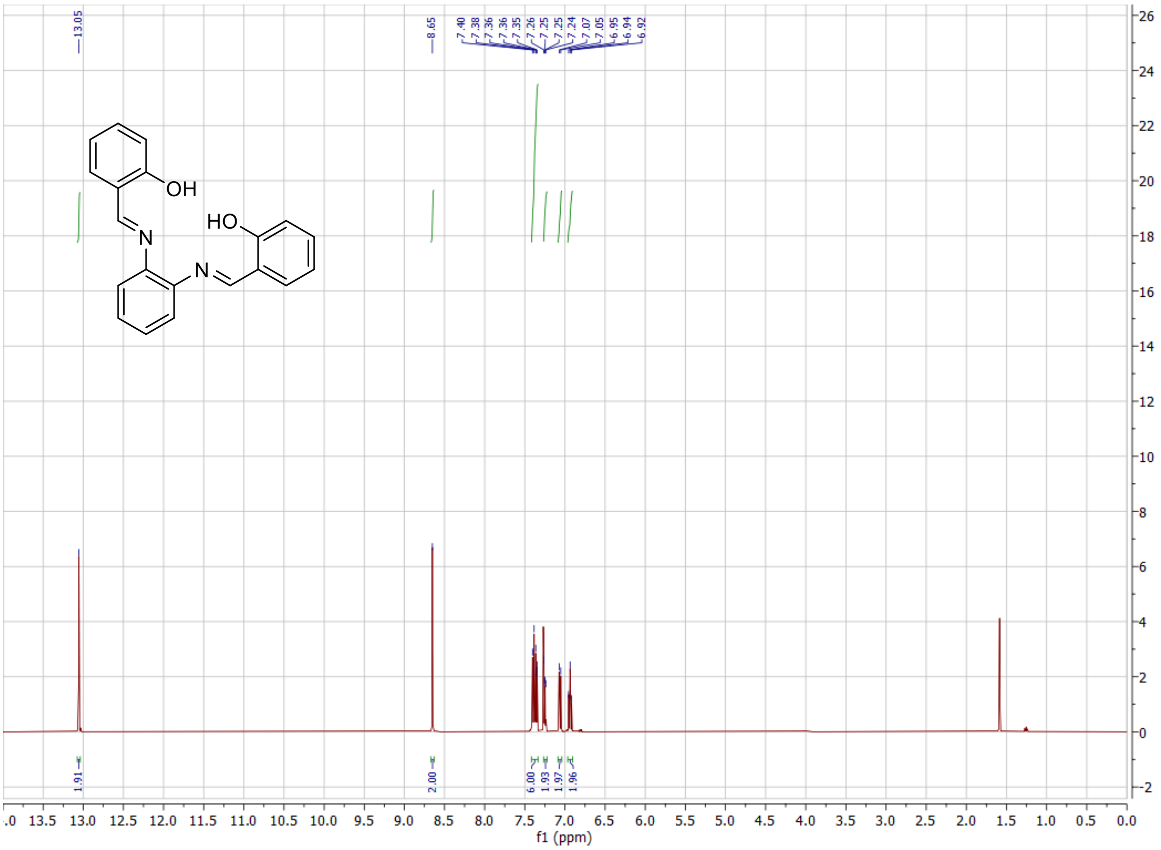

(13E,19E)-Tribenzo[e,i,m][1,4]dioxa[8,11]diazacyclotetradecine-6,7-dione (128)

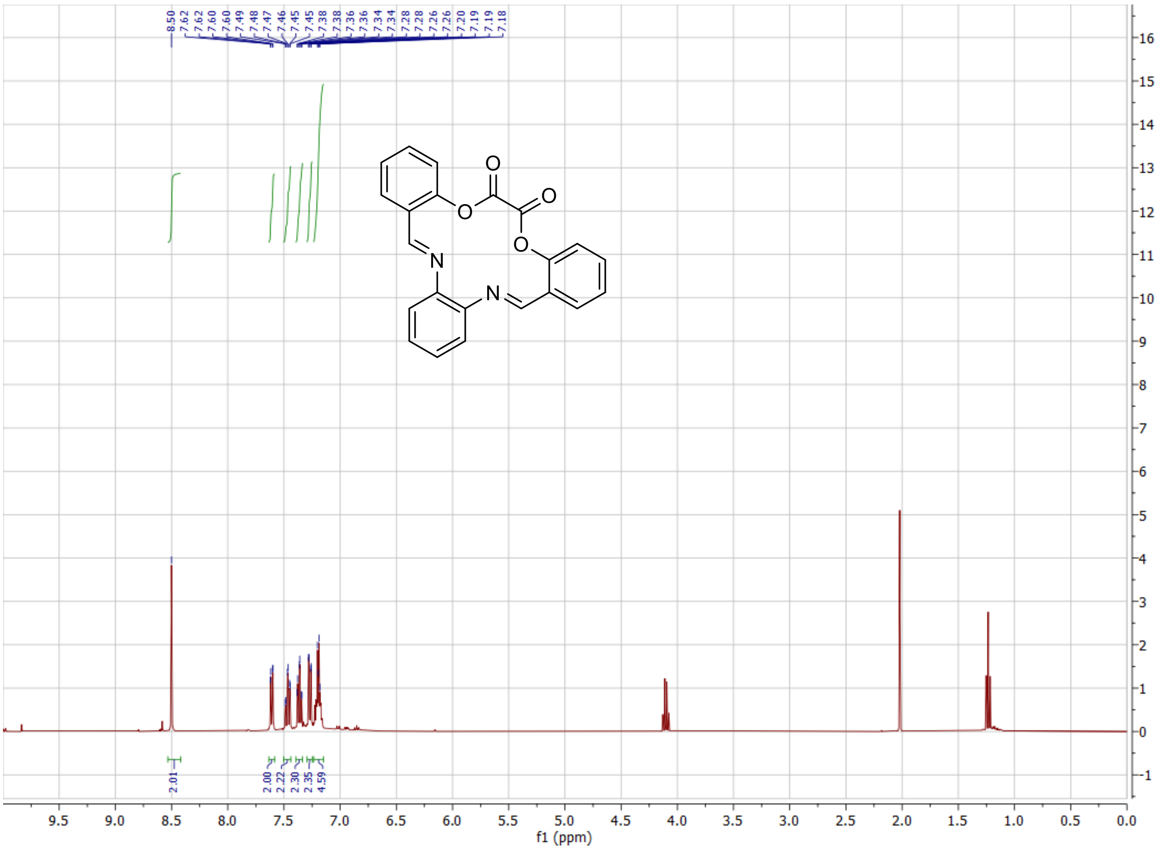

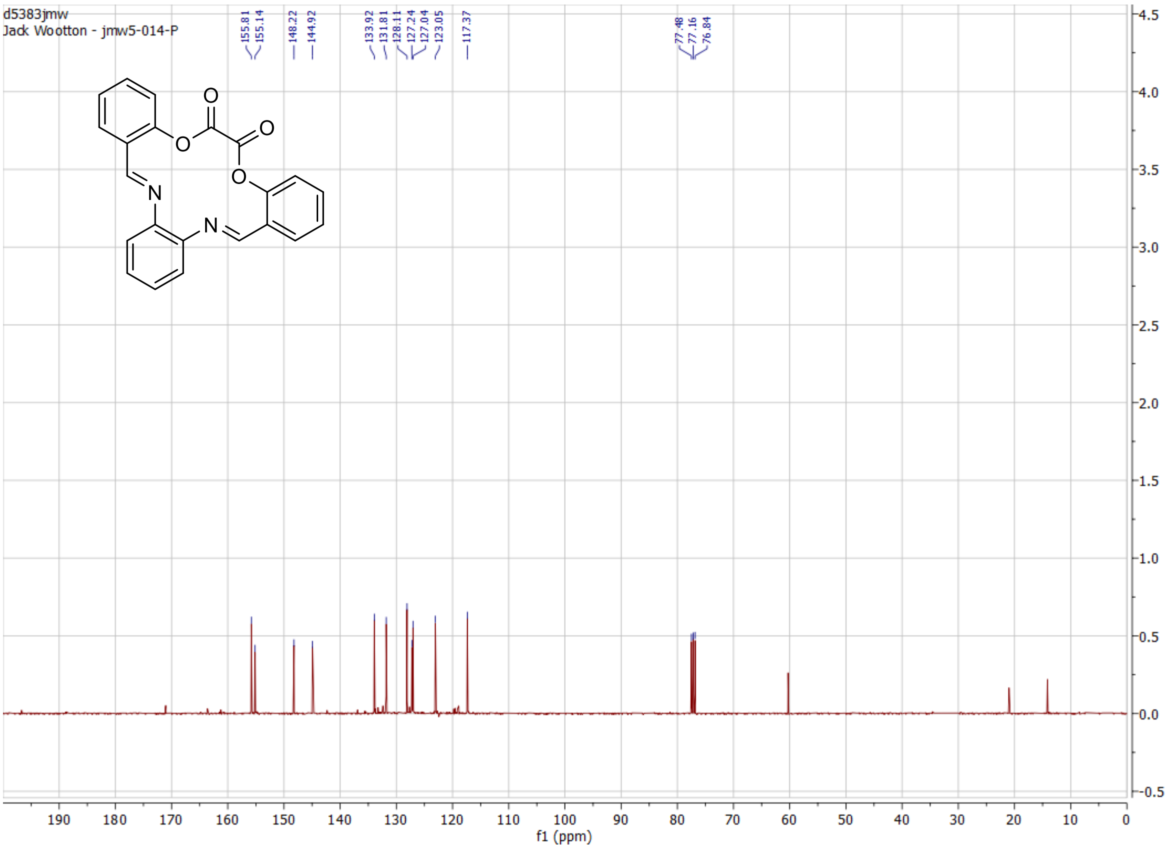

1,11-Dimethyl-1,2,3,4,8,9,10,11-octahydrobenzo[h][1,3]dioxa[2]thia[7,10]diazacyclotridecine 6-oxide (129)

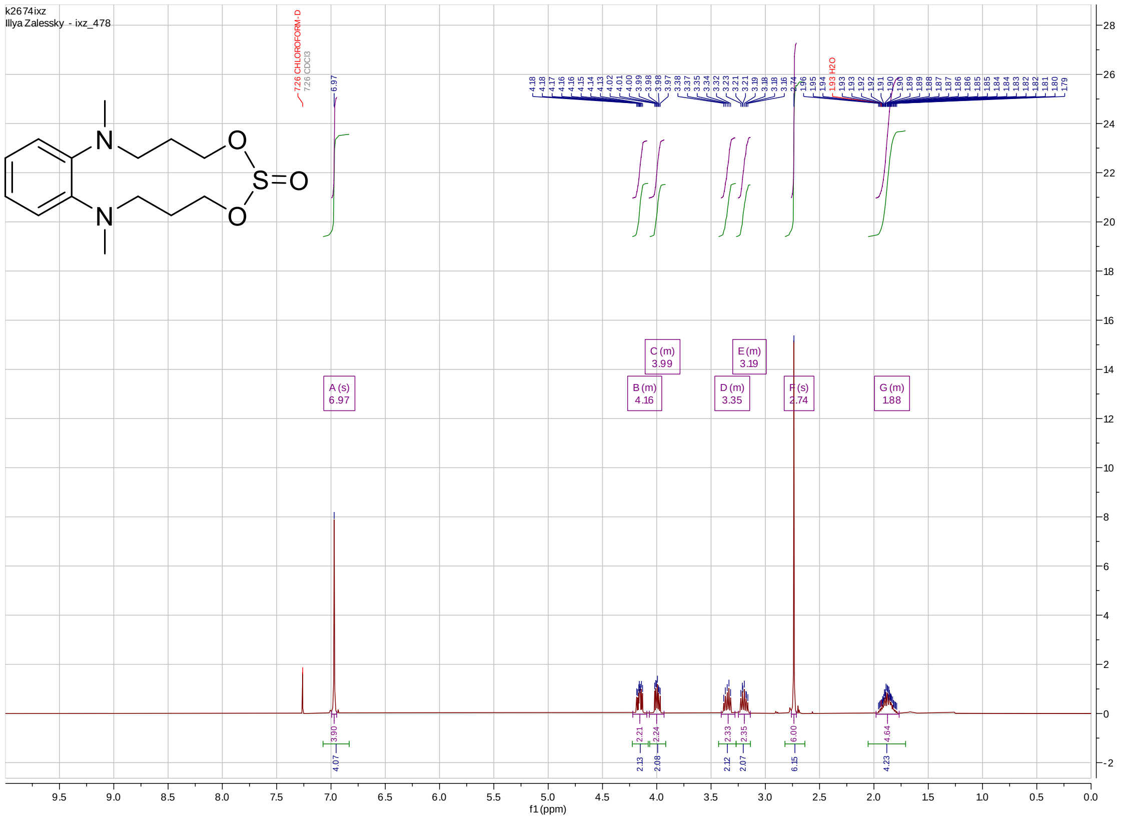

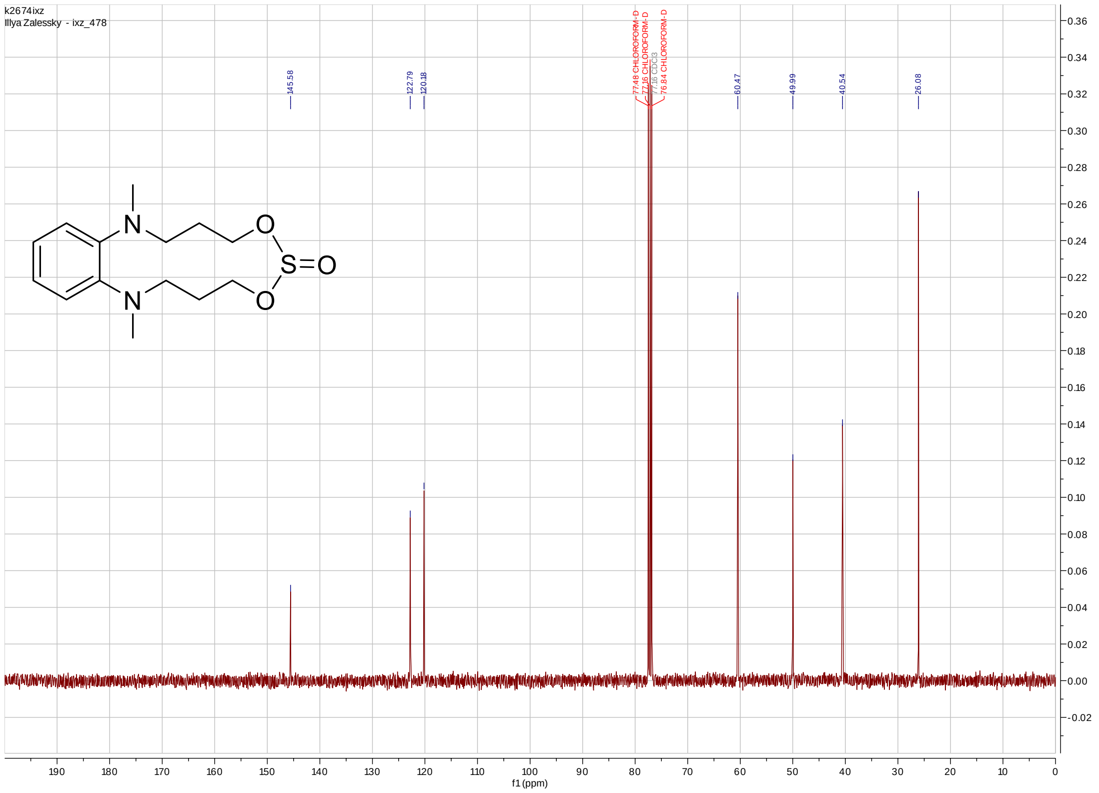

***N*[1],*N*[3]-dibenzylpropane-1,3-diamine (S173)**

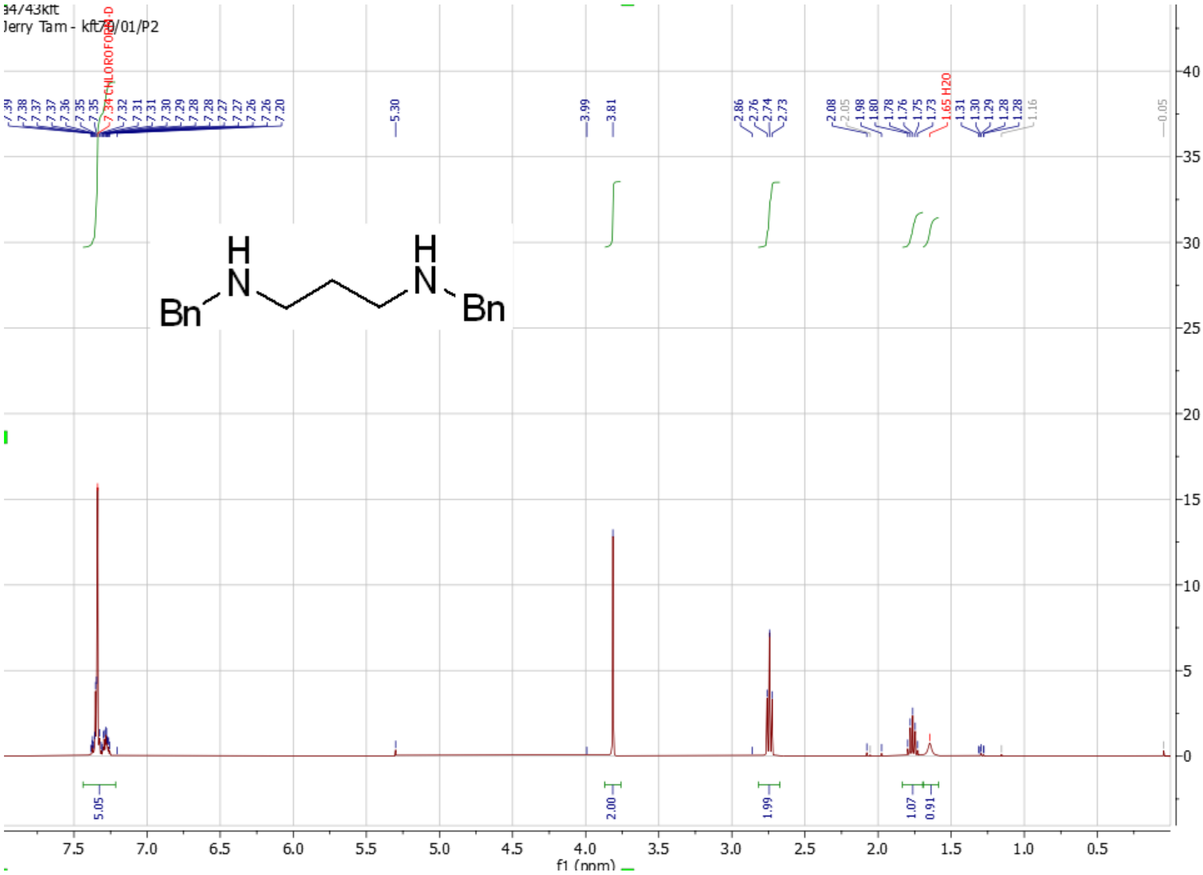

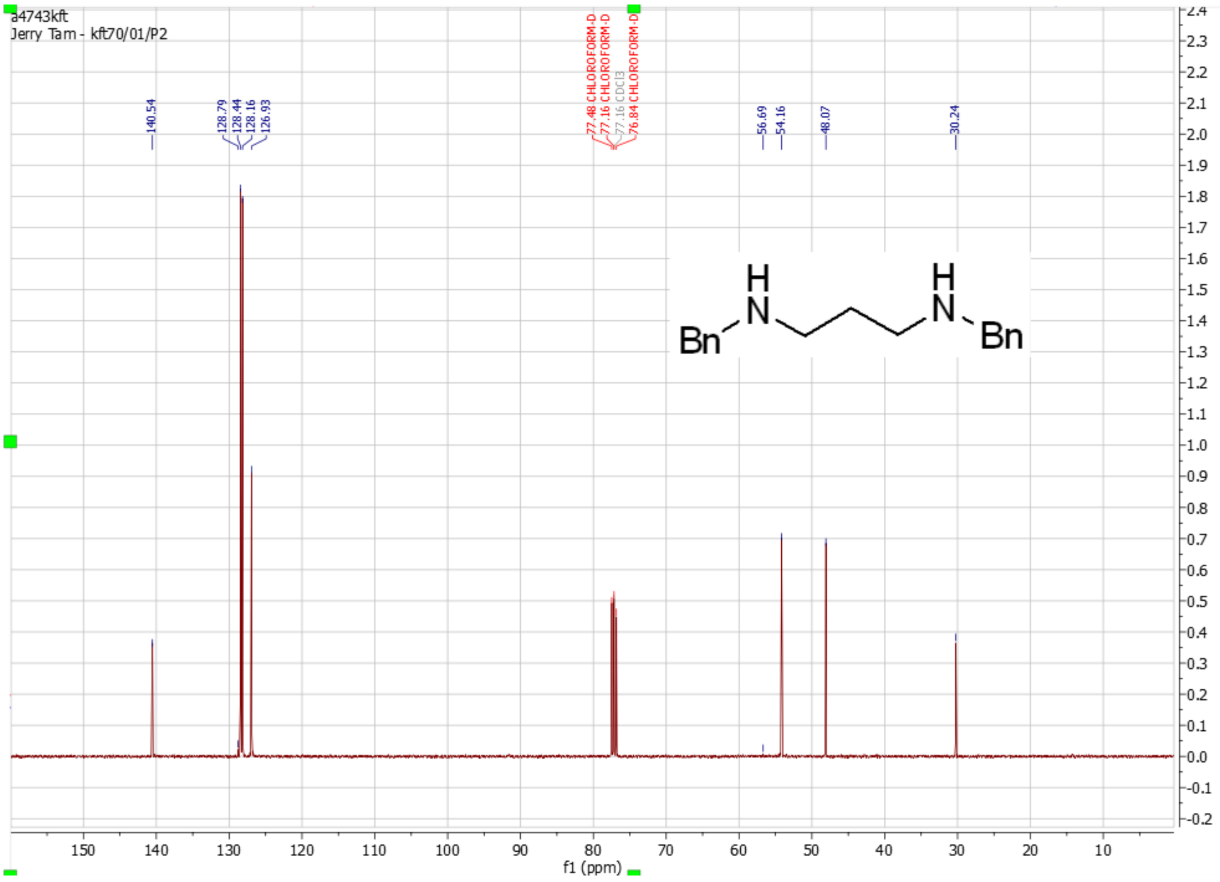

N^1,N^3-dibenzyl-N^1,N^3-bis(3-((*tert*-butyldimethylsilyl)oxy)propyl)propane-1,3-diamine (S174)

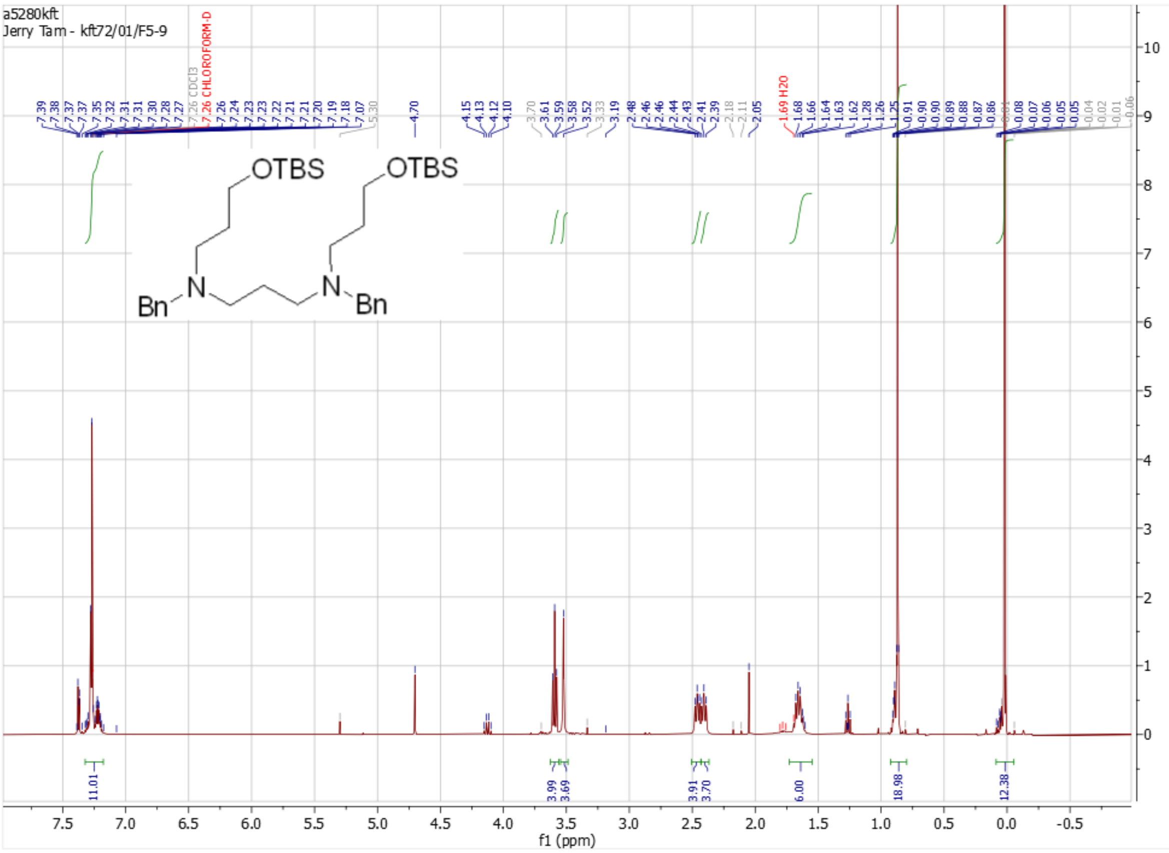

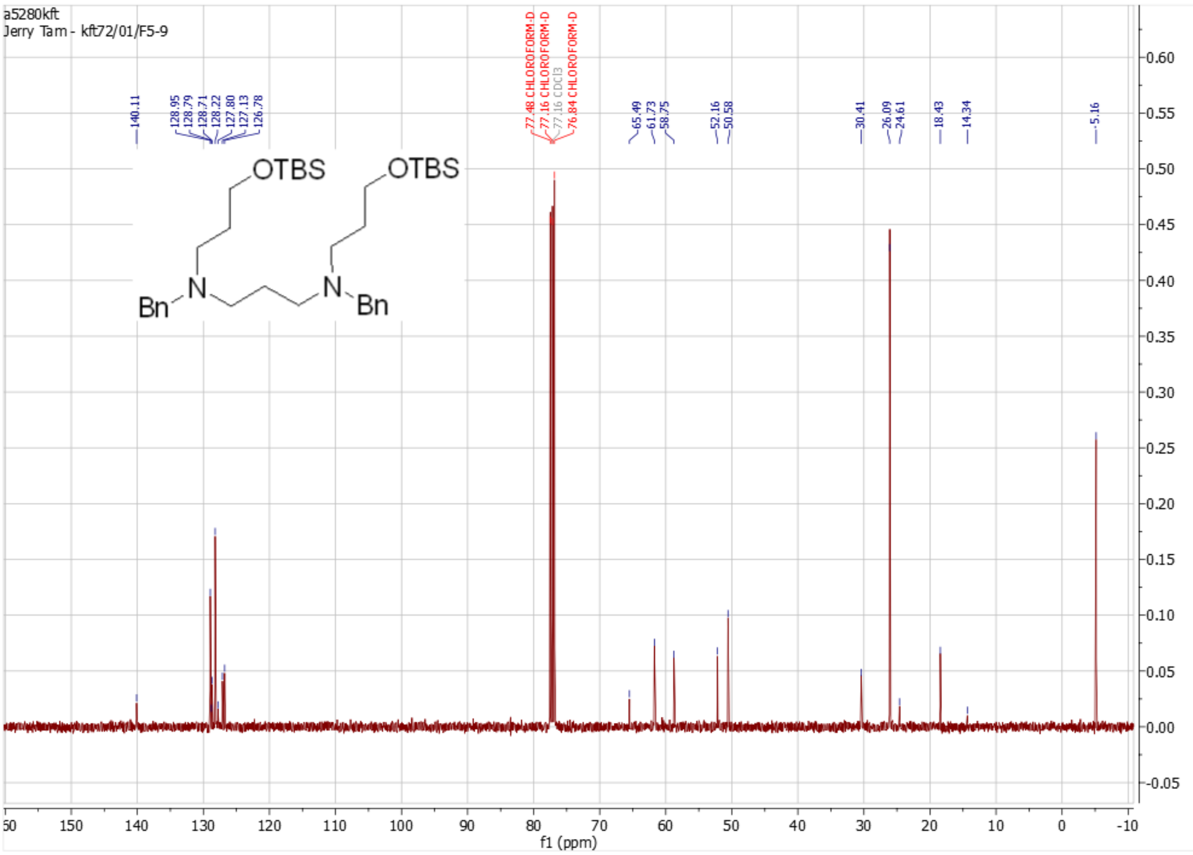

3,3'-(Propane-1,3-diylbis(benzylazanediyl))bis(propan-1-ol) (S175)

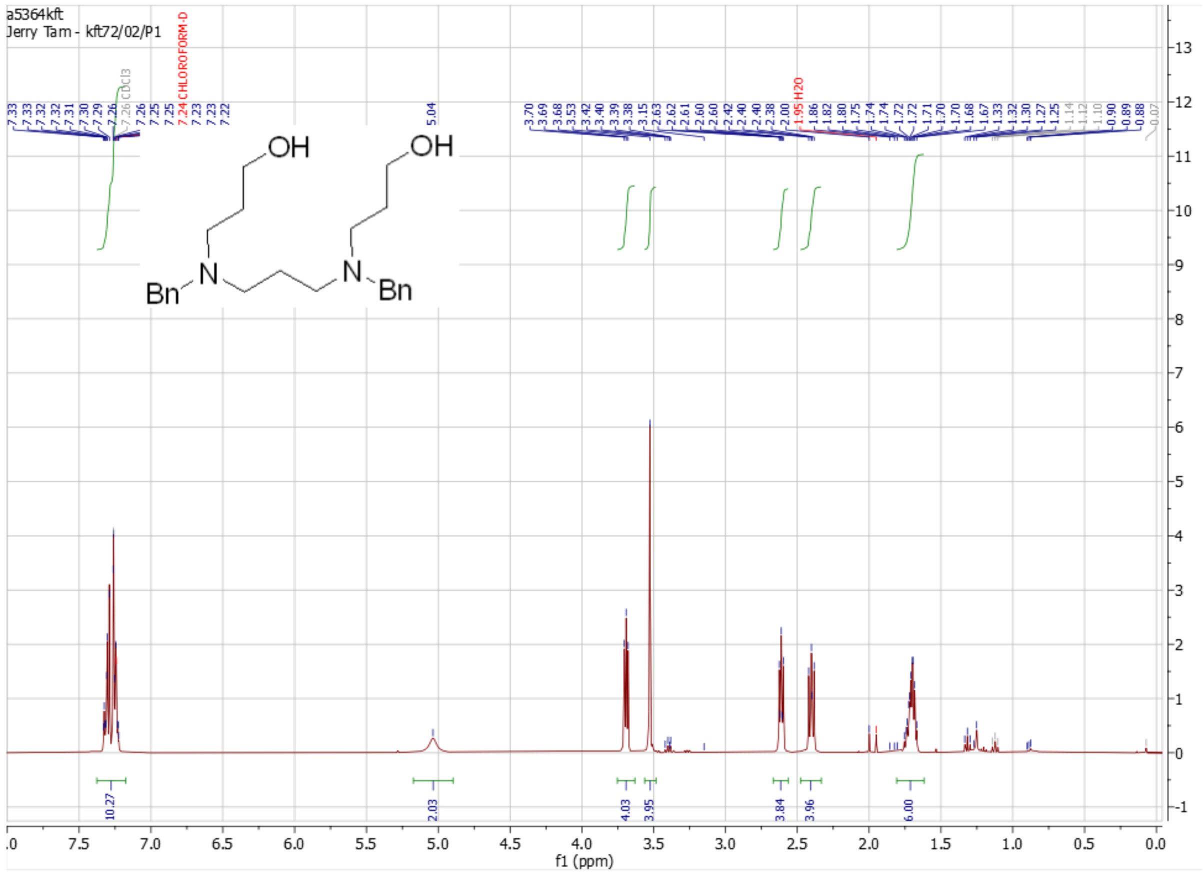

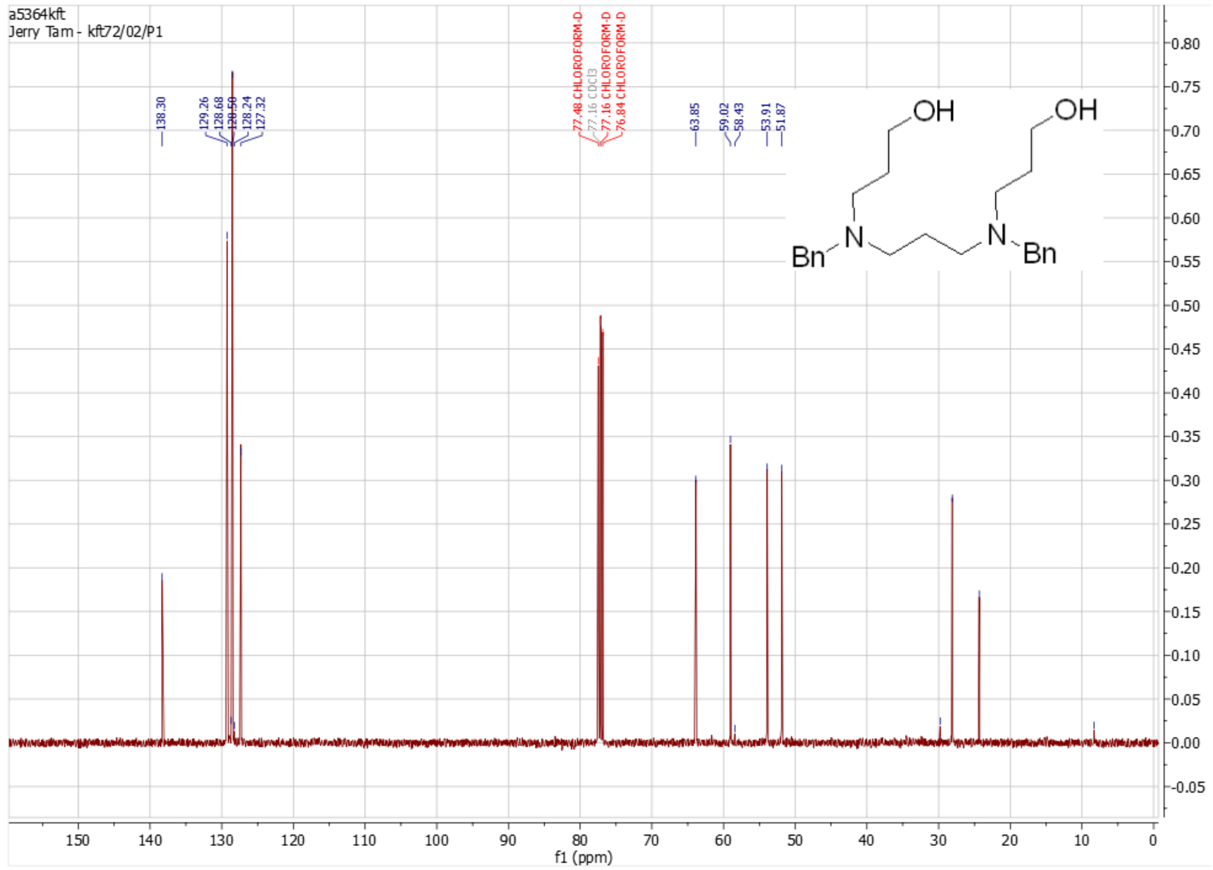

7,11-Dibenzyl-1,3-dioxa-2-thia-7,11-diazacyclotetradecane 2-oxide (130)

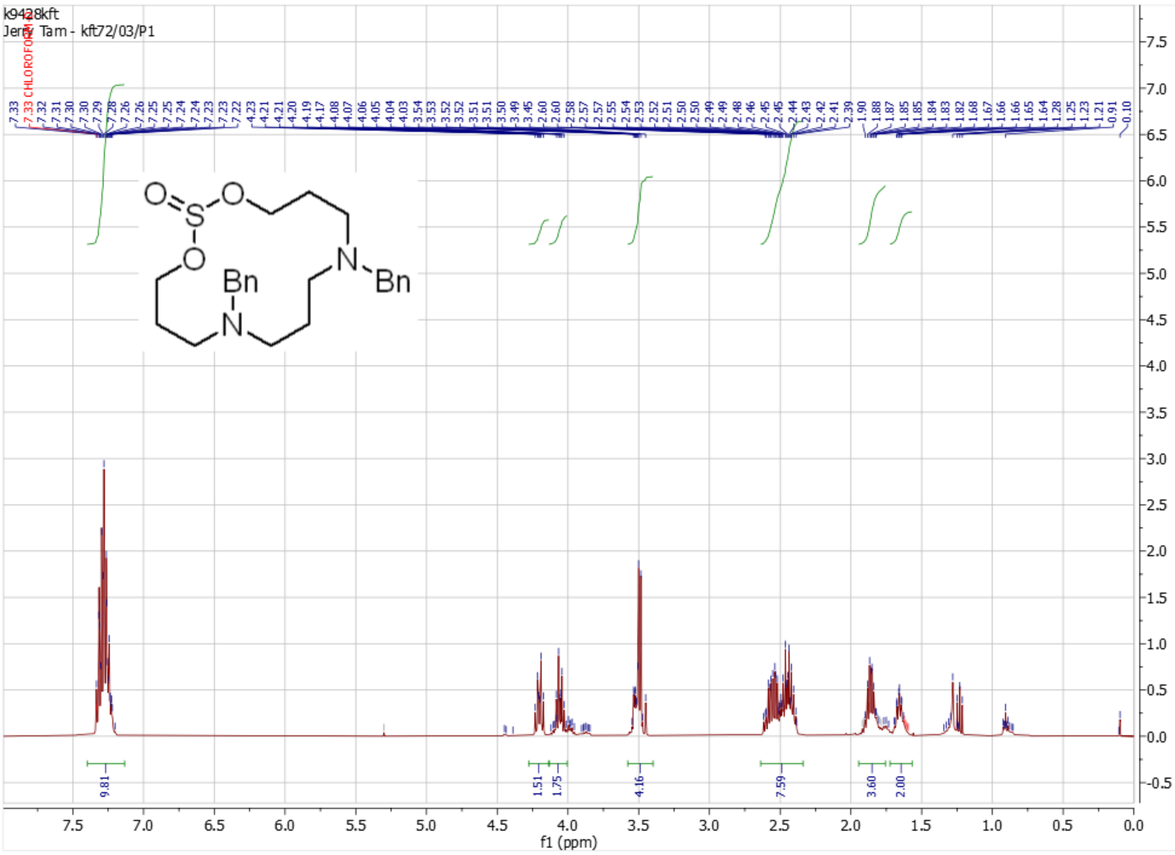

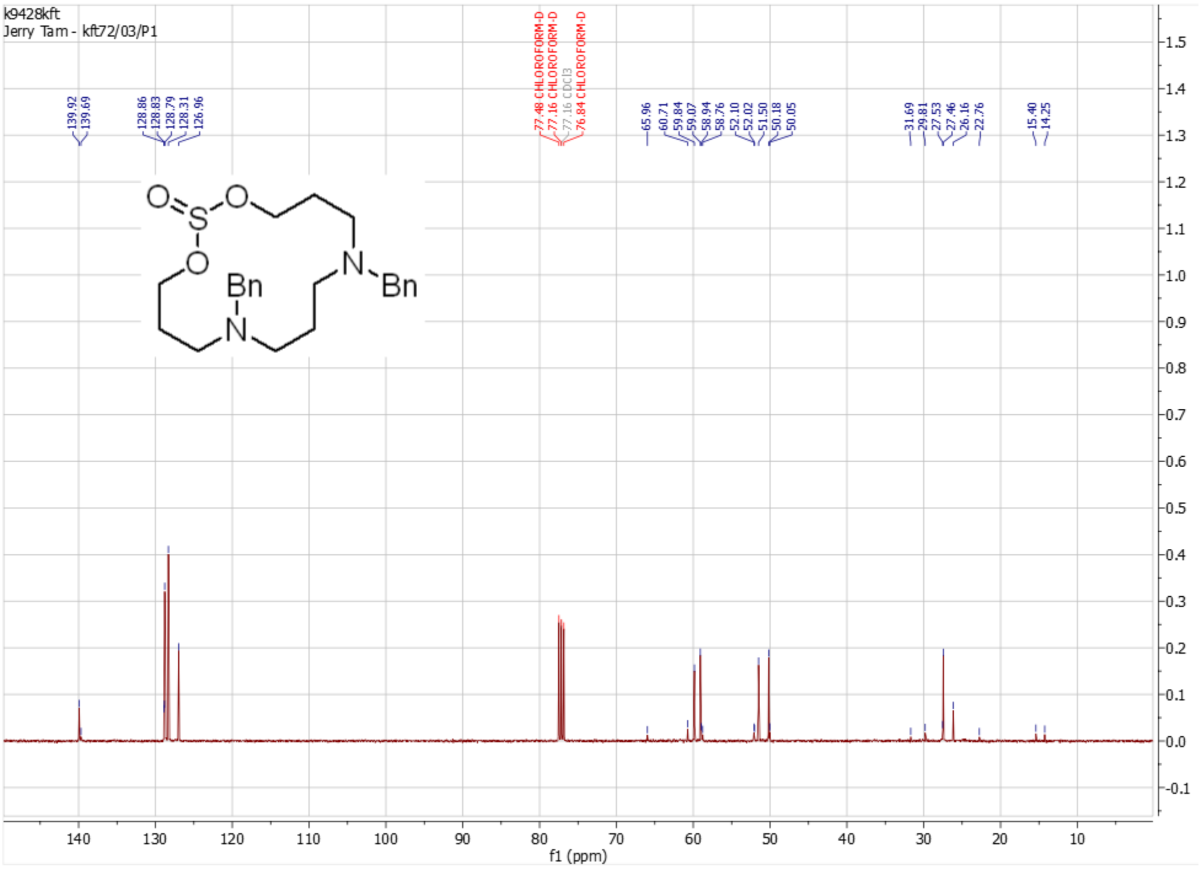

3,3'-(((Methylazanediyl)bis(propane-3,1-diyl))bis(methylazanediyl))bis(propan-1-ol) (S176)

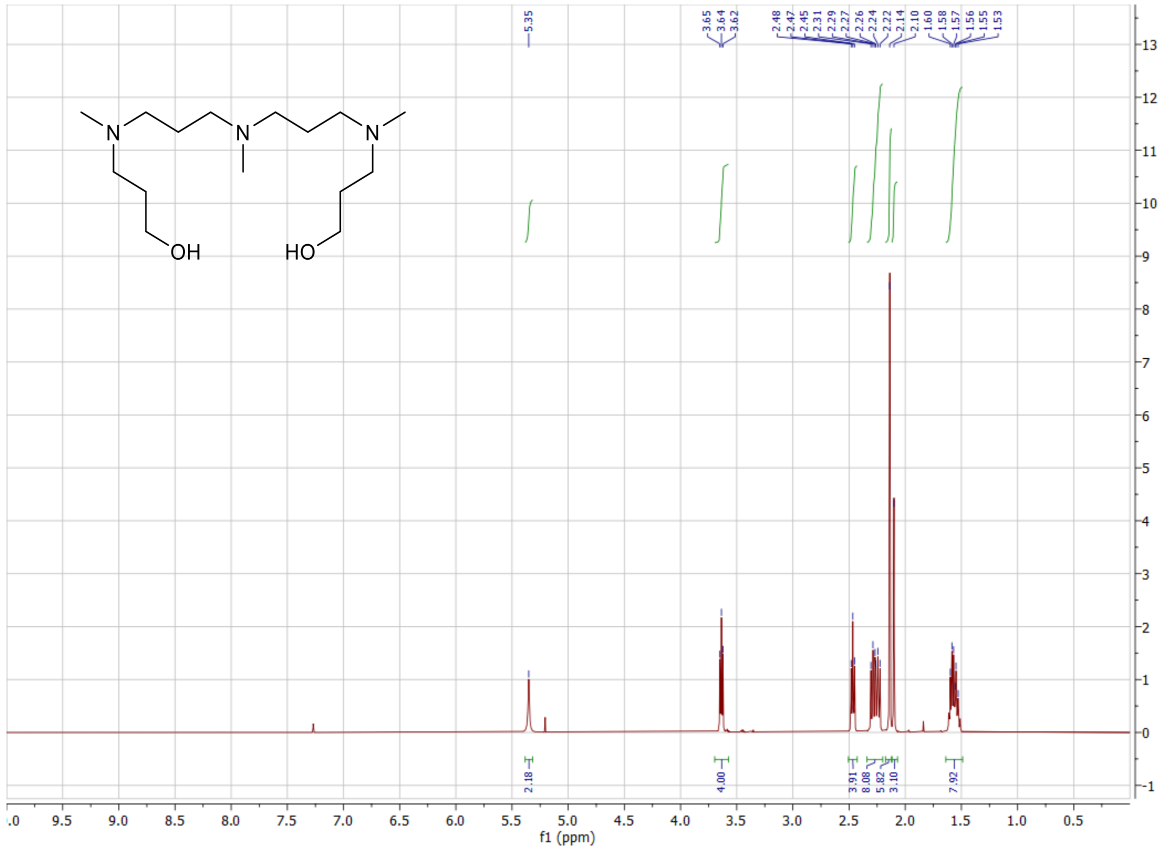

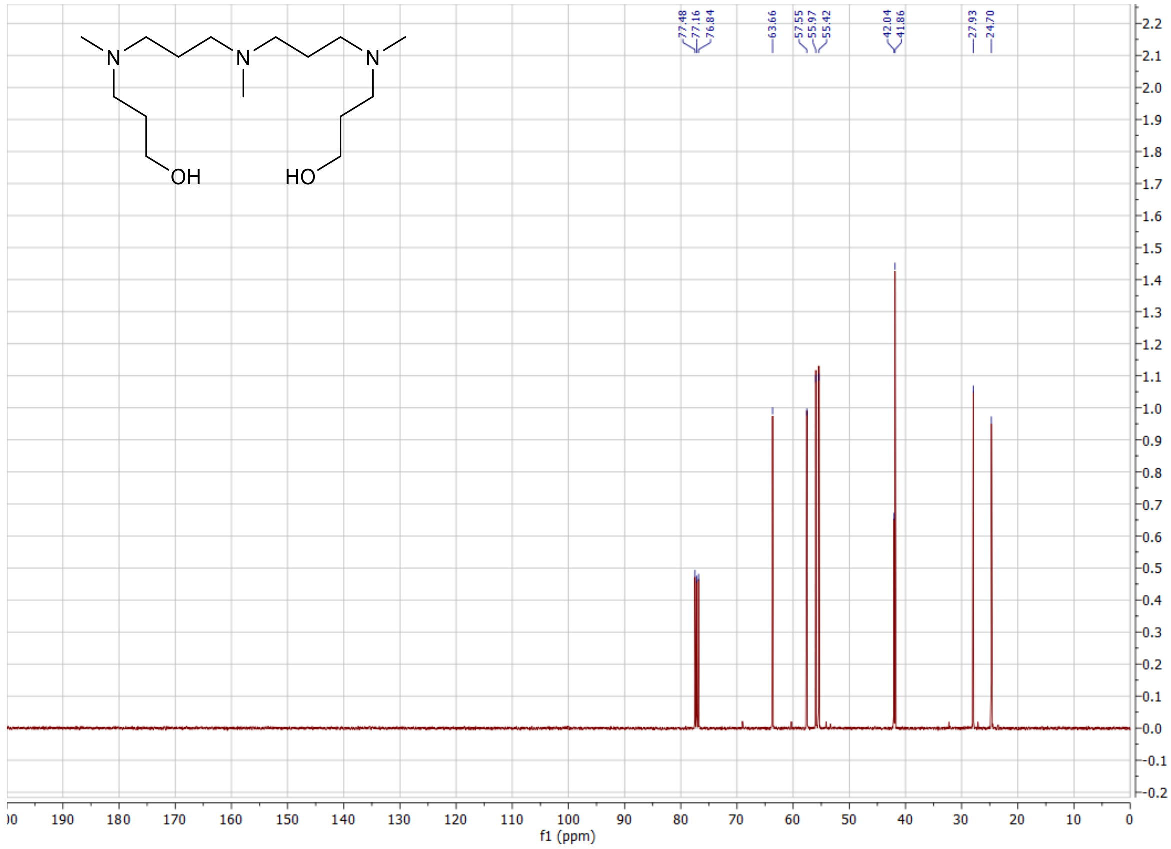

7,11,15-Trimethyl-1,3-dioxa-2-thia-7,11,15-triazacyclooctadecane 2-oxide (131)

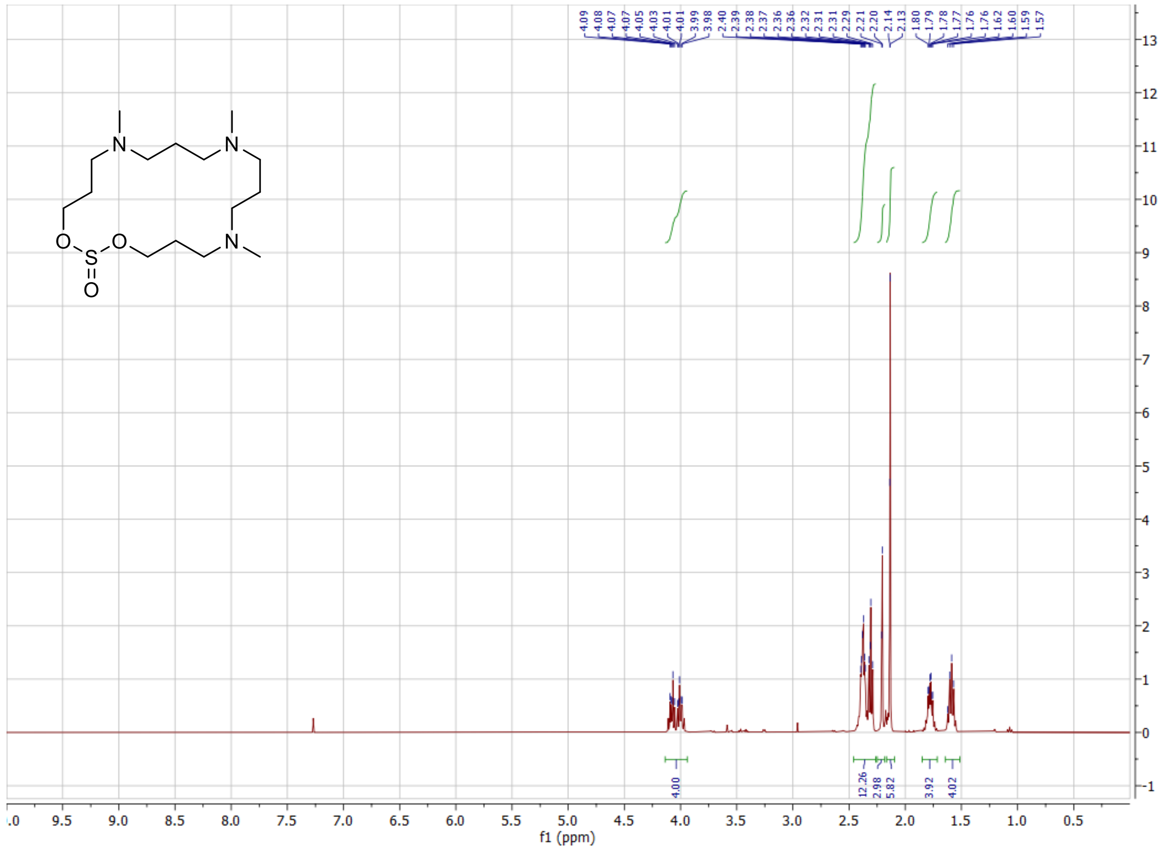

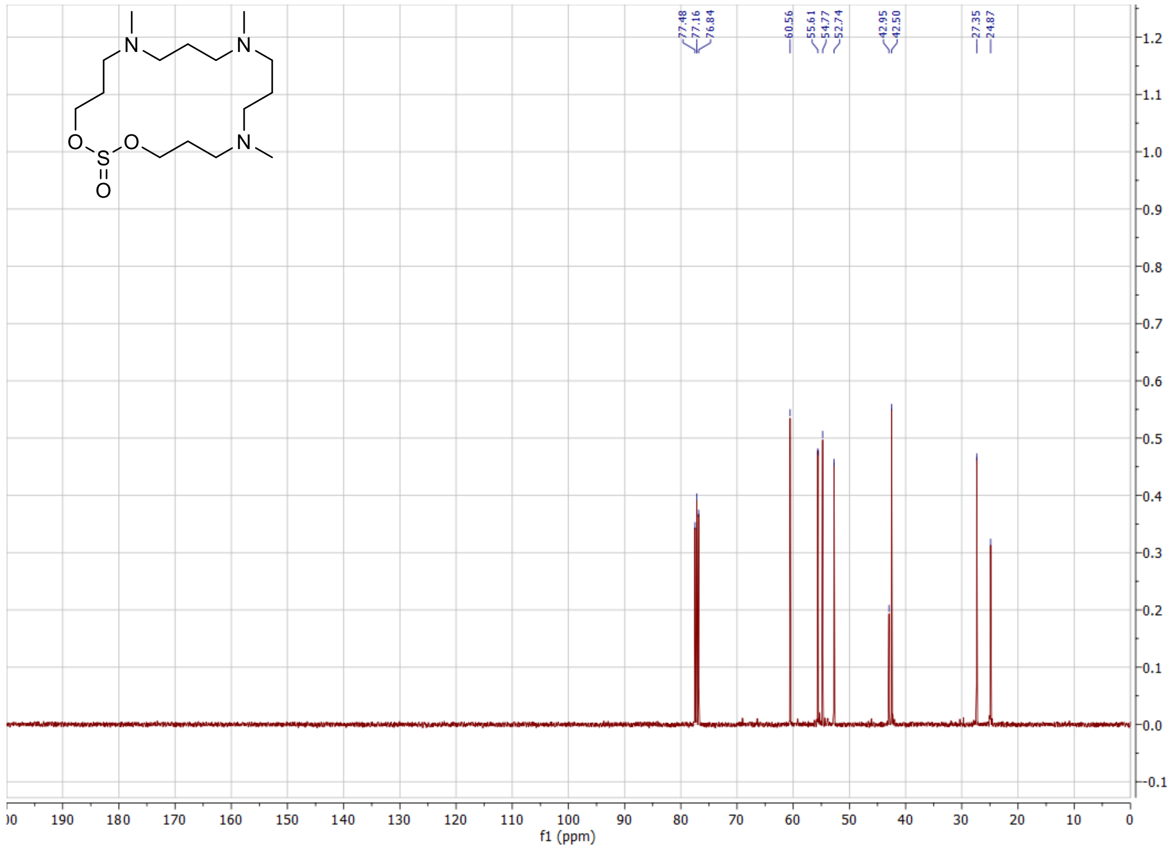

Methyl 2-(((3-((3-bromopropyl)thio)propyl)selanyl)methyl)benzoate (S177)

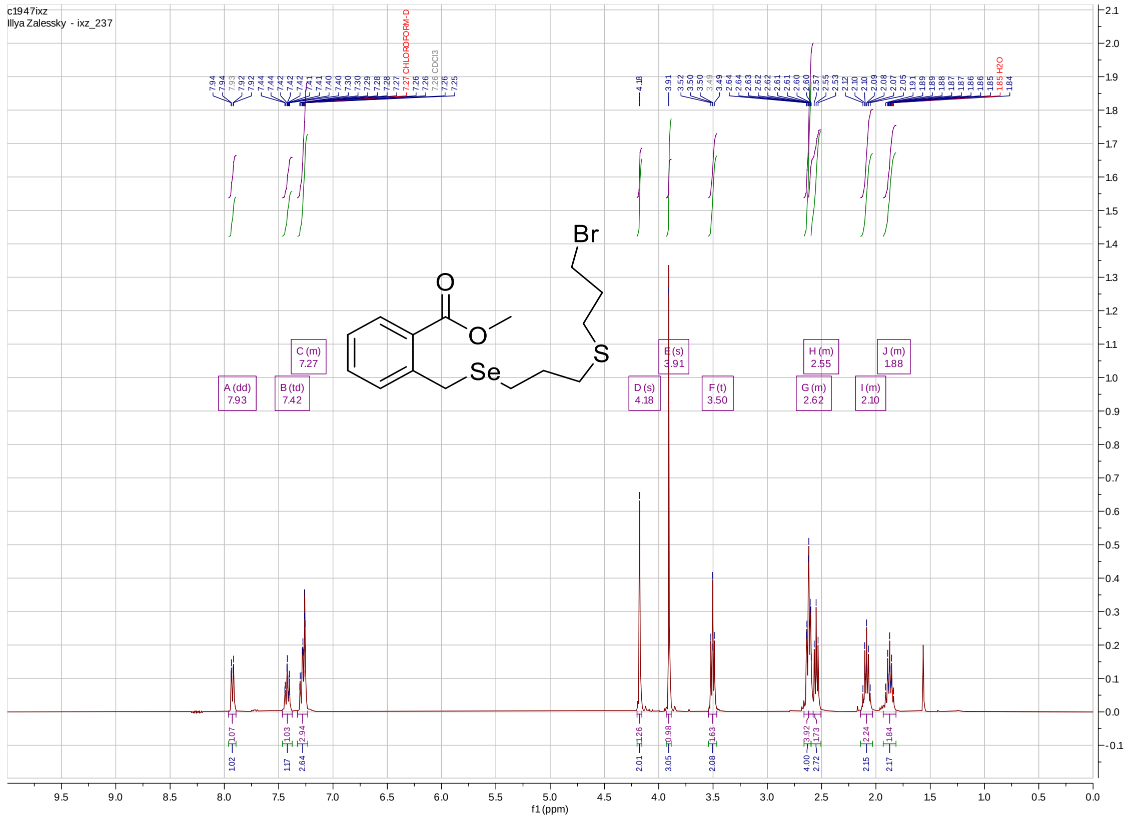

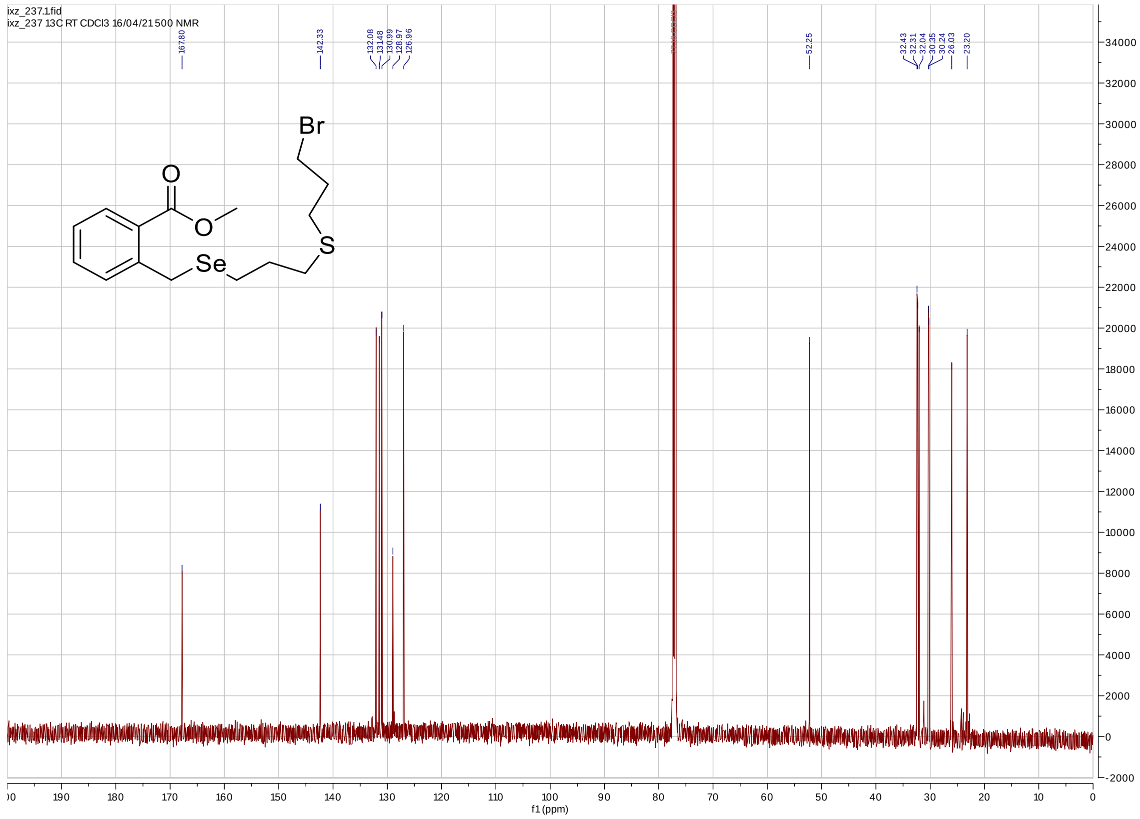

Methyl 2-(((3-((3-(benzyl(3-hydroxypropyl)amino)propyl)thio)propyl)selanyl)methyl)benzoate (S178)

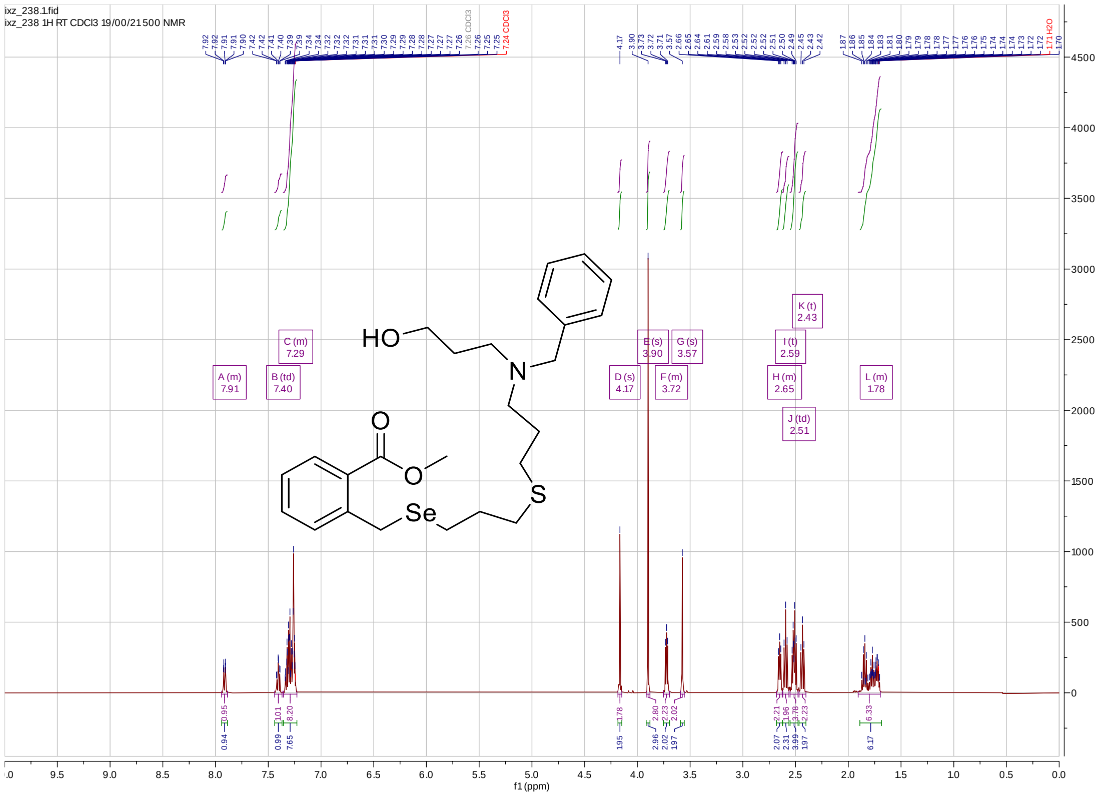

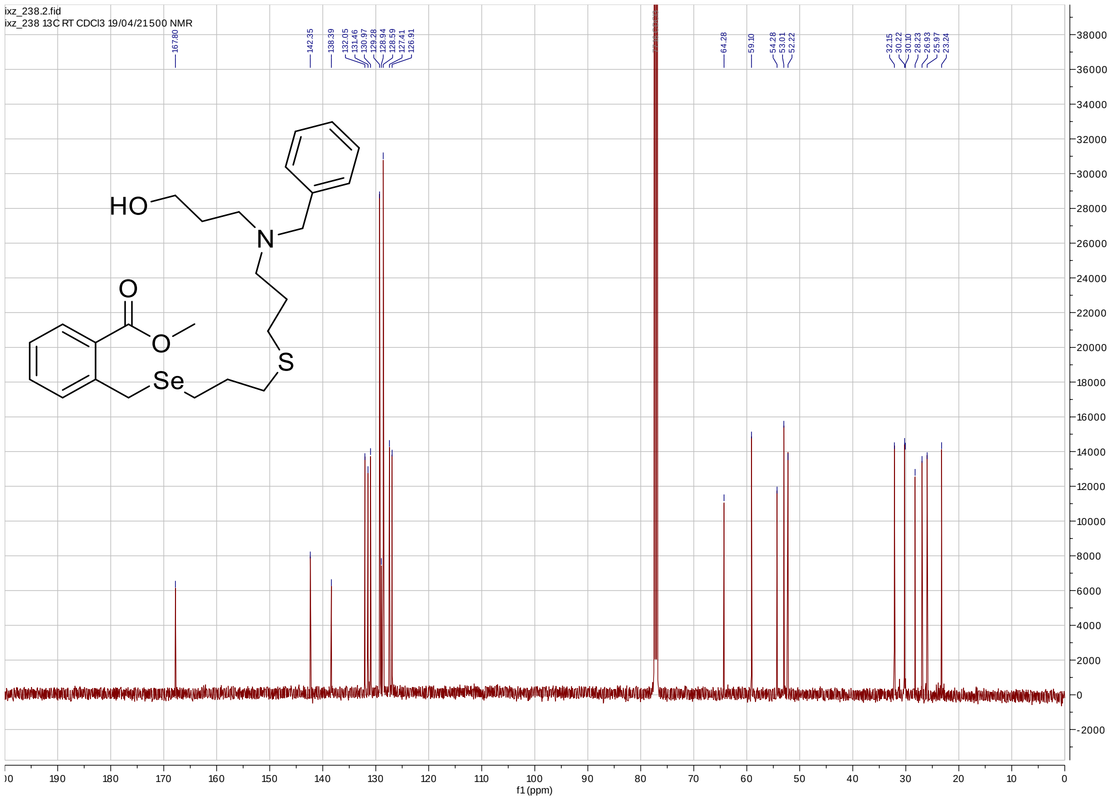

2-(((3-((3-(Benzyl(3-hydroxypropyl)amino)propyl)thio)propyl)selanyl)methyl)benzoic acid (S179)

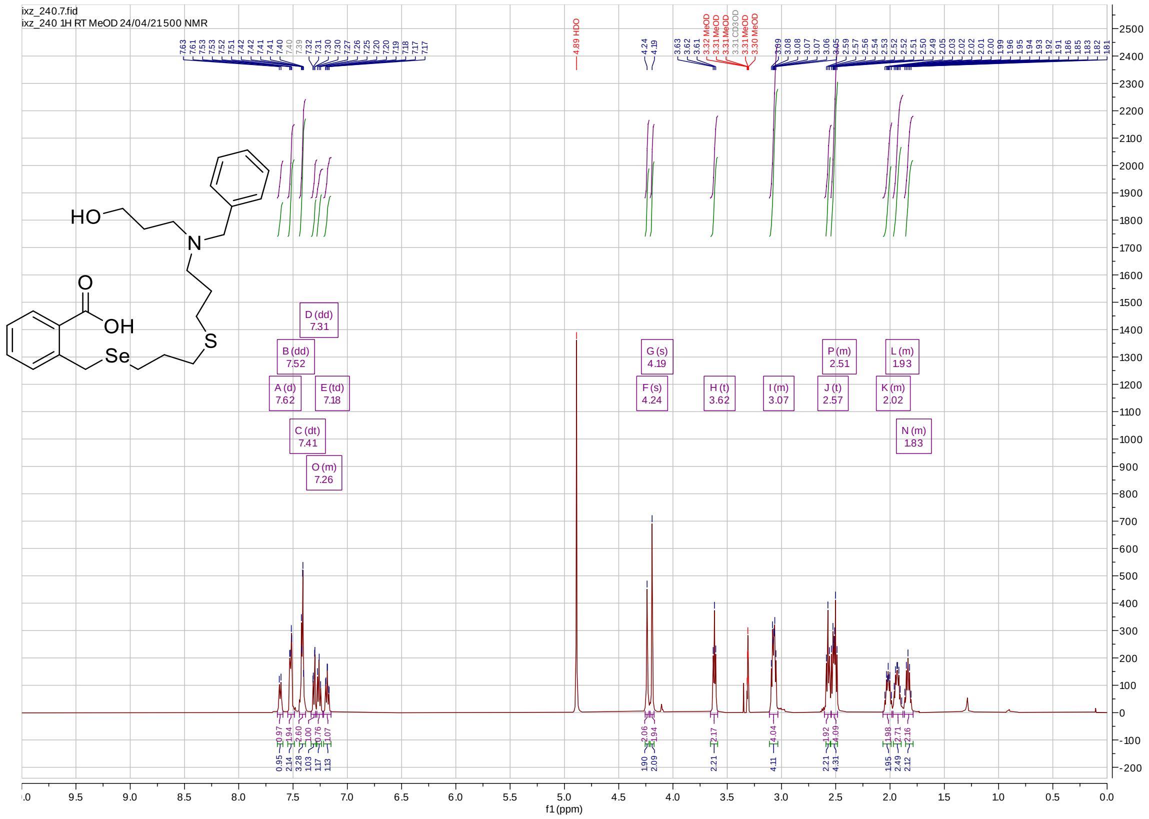

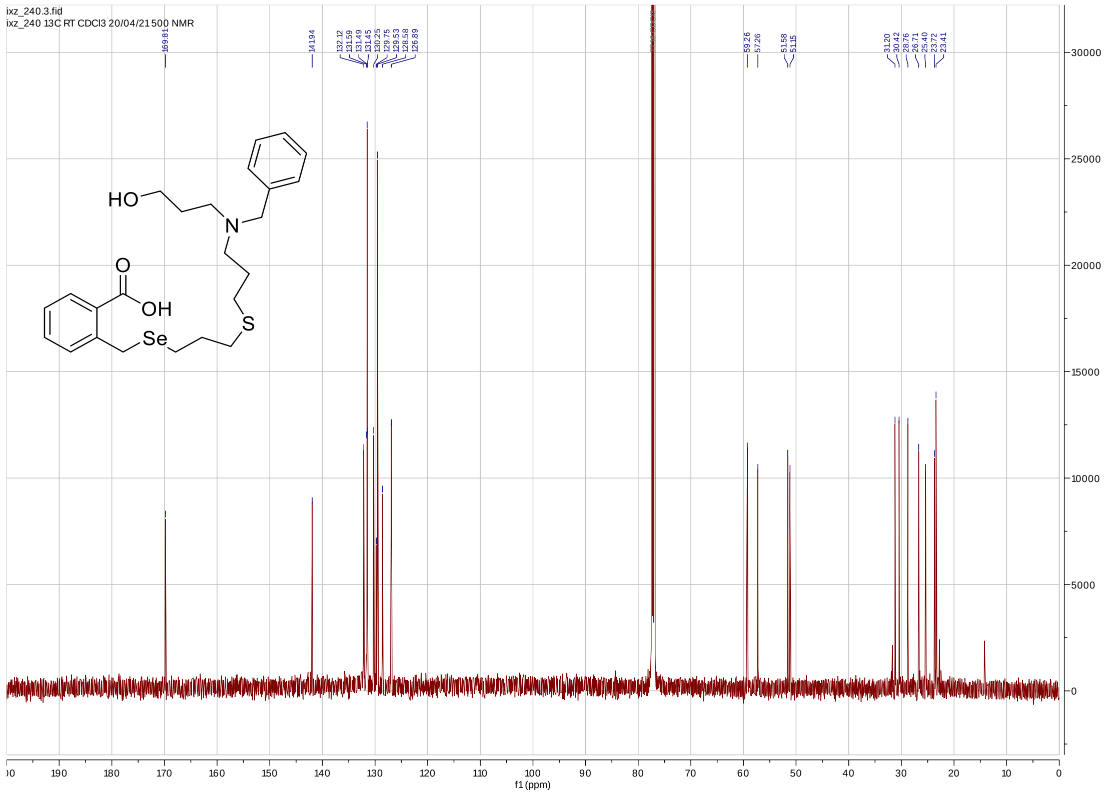

6-Benzyl-4,5,6,7,8,9,12,13-octahydro-3H,11H-benzo[o][1]oxa[9]thia[13]selena[5]azacycloheptadecin-1(15H)-one (132)

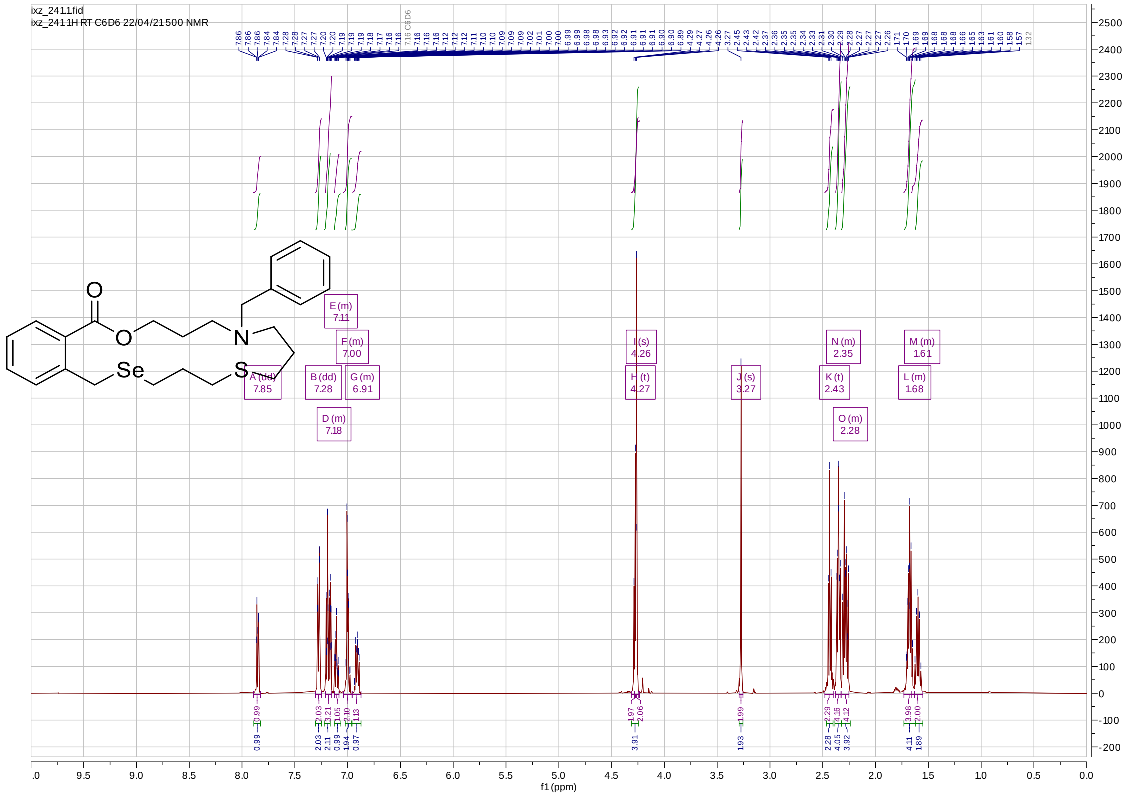

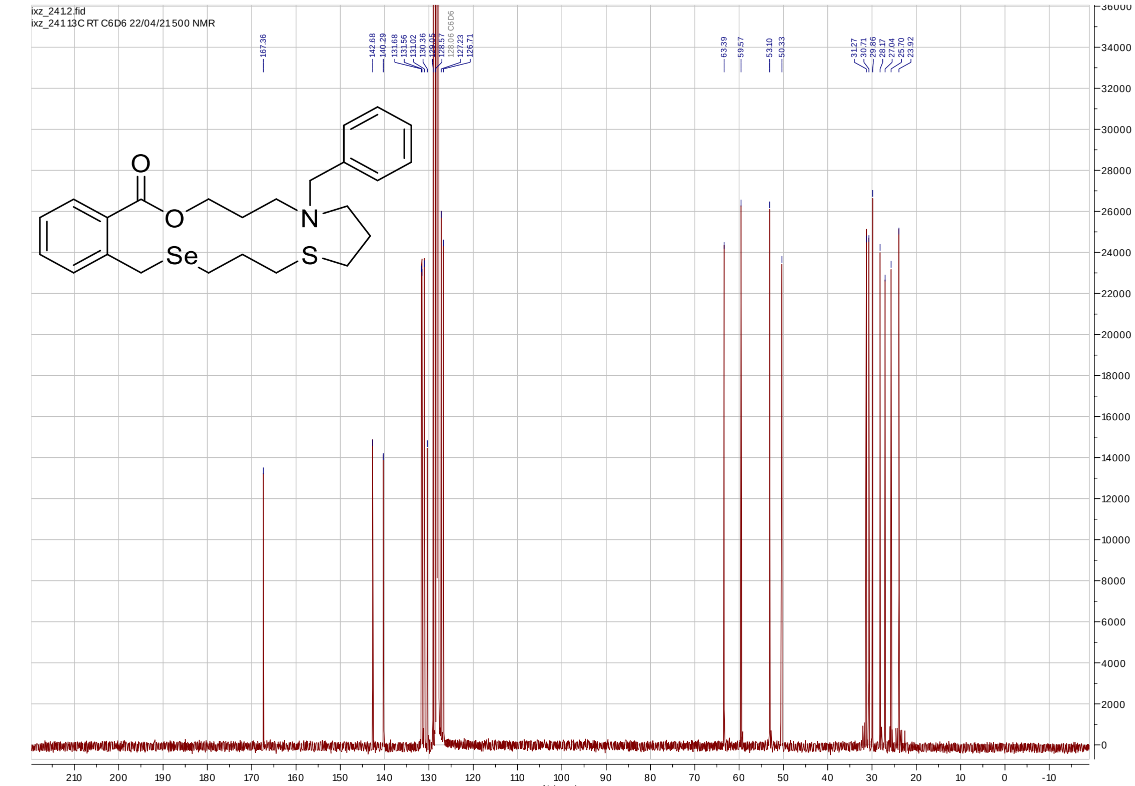

Methyl 2-(8-hydroxyoct-1-yn-1-yl)benzoate (S180)

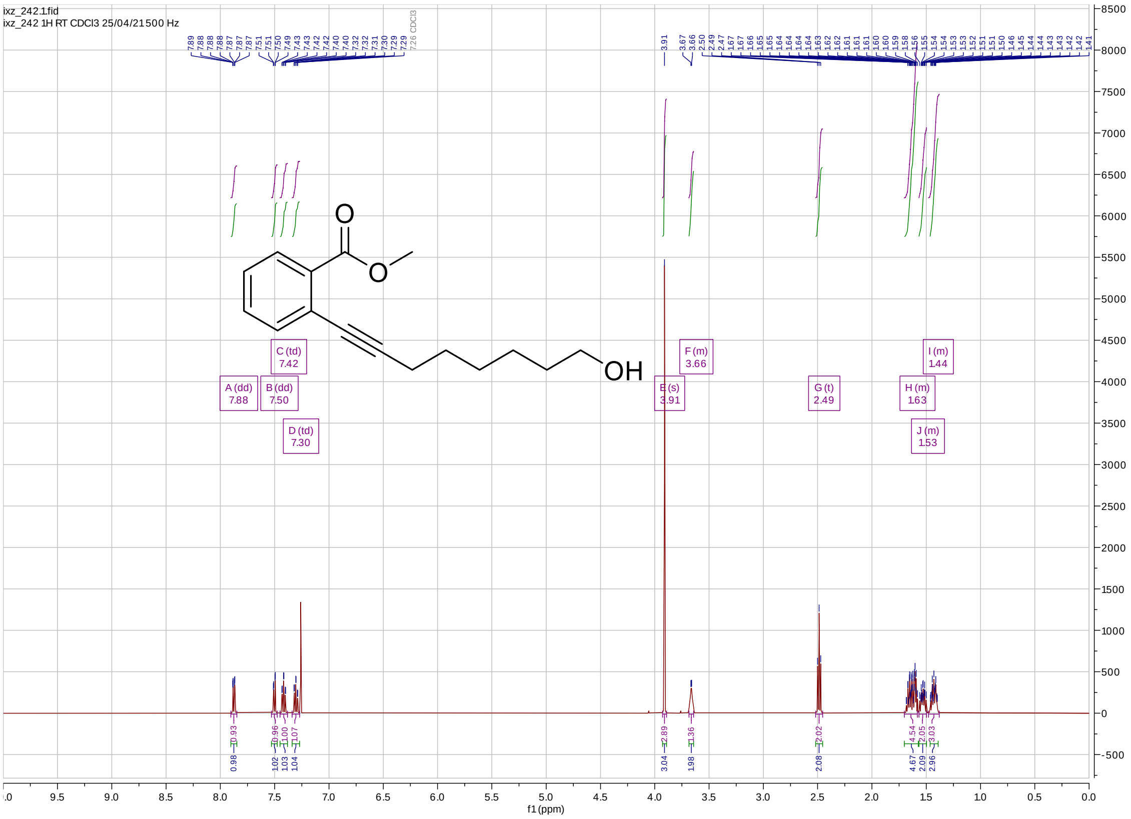

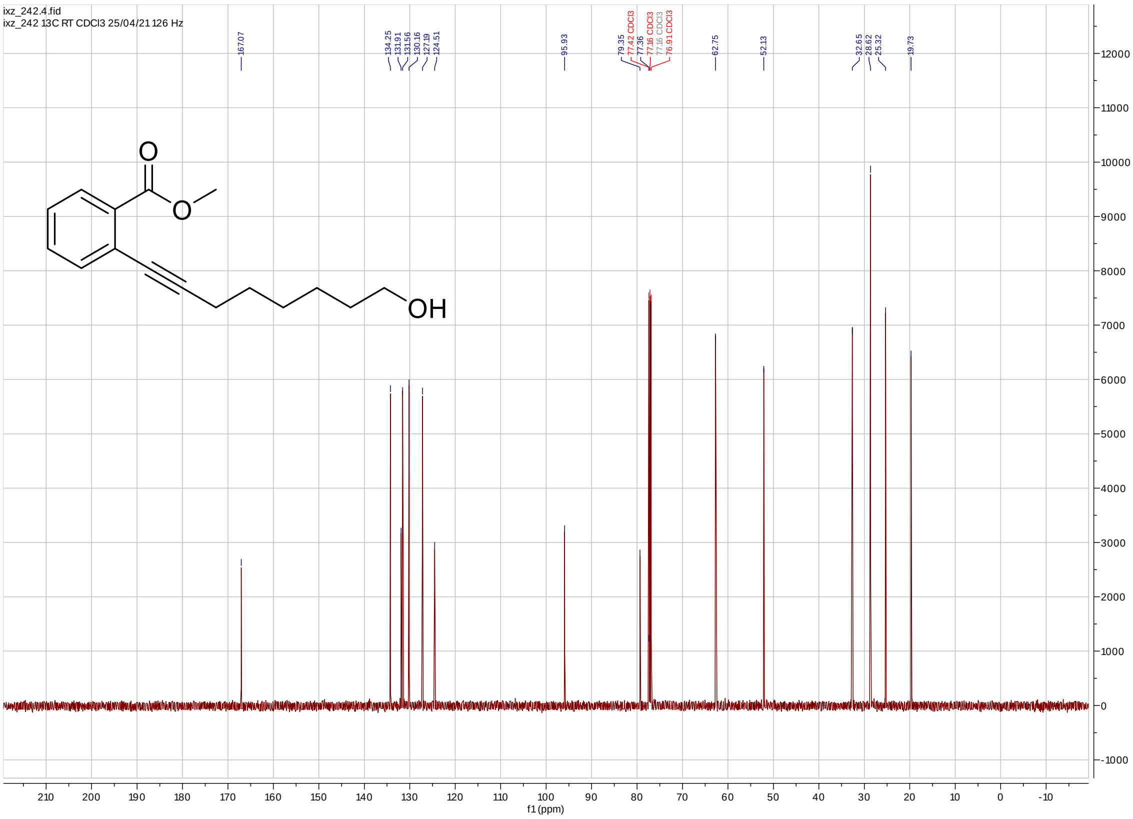

Methyl 2-(8-hydroxyoctyl)benzoate (S181)

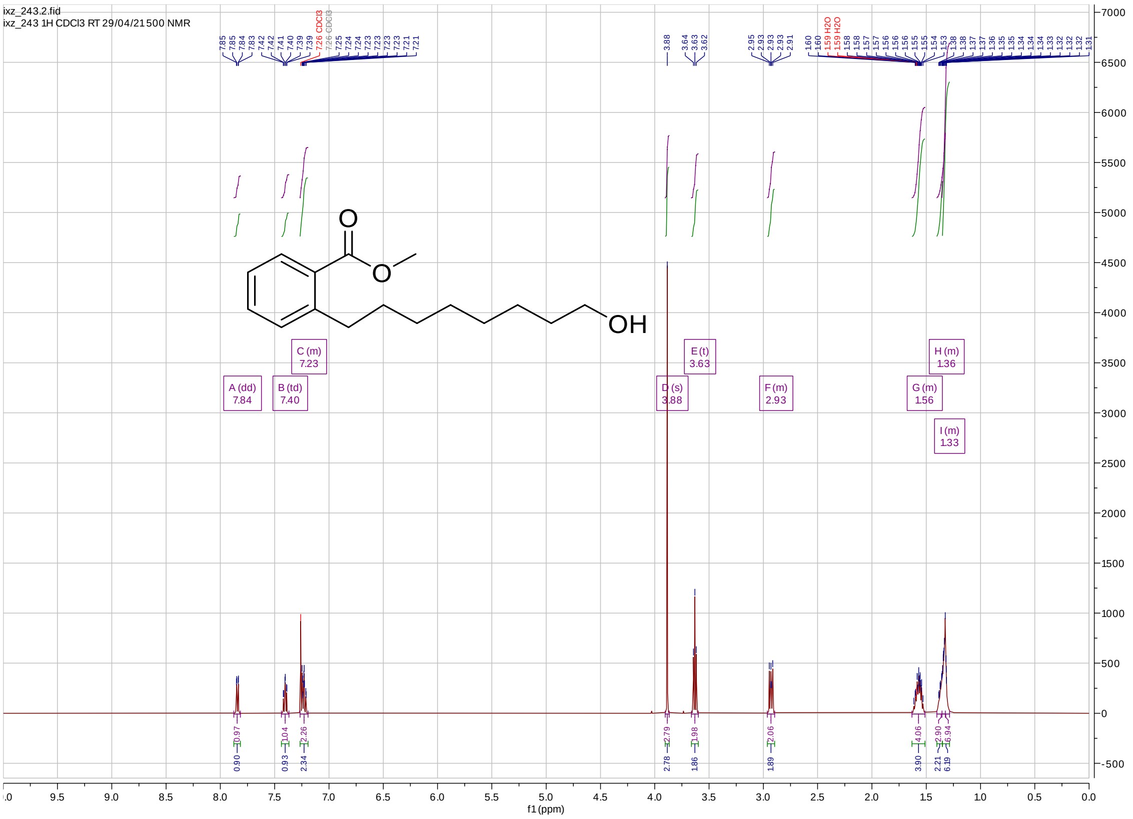

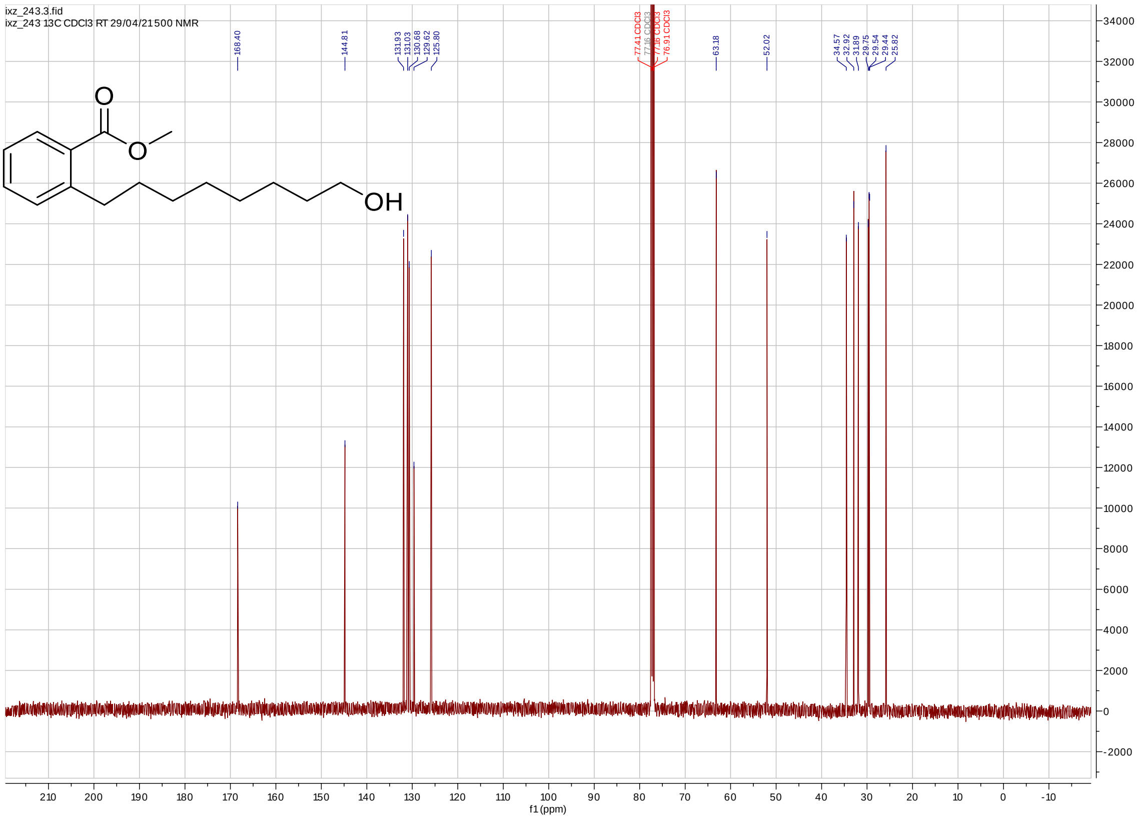

2-(8-Hydroxyoctyl)benzoic acid (133)

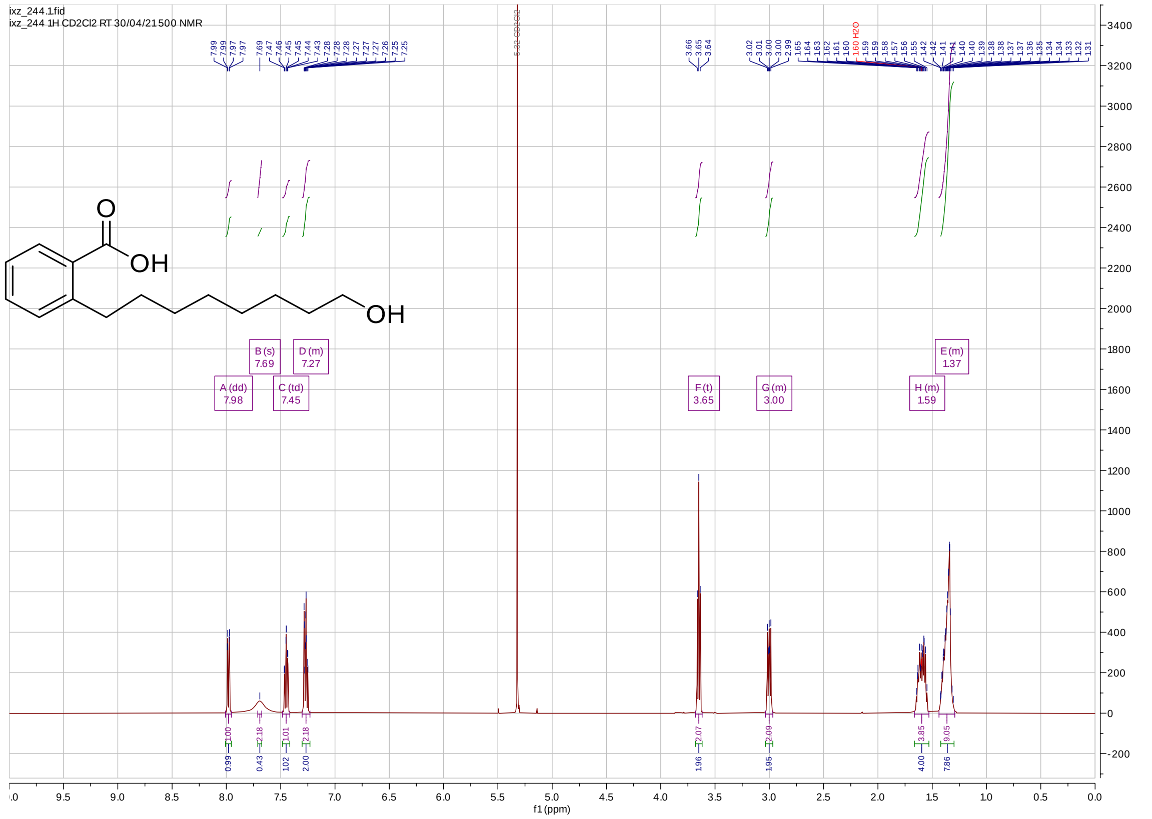

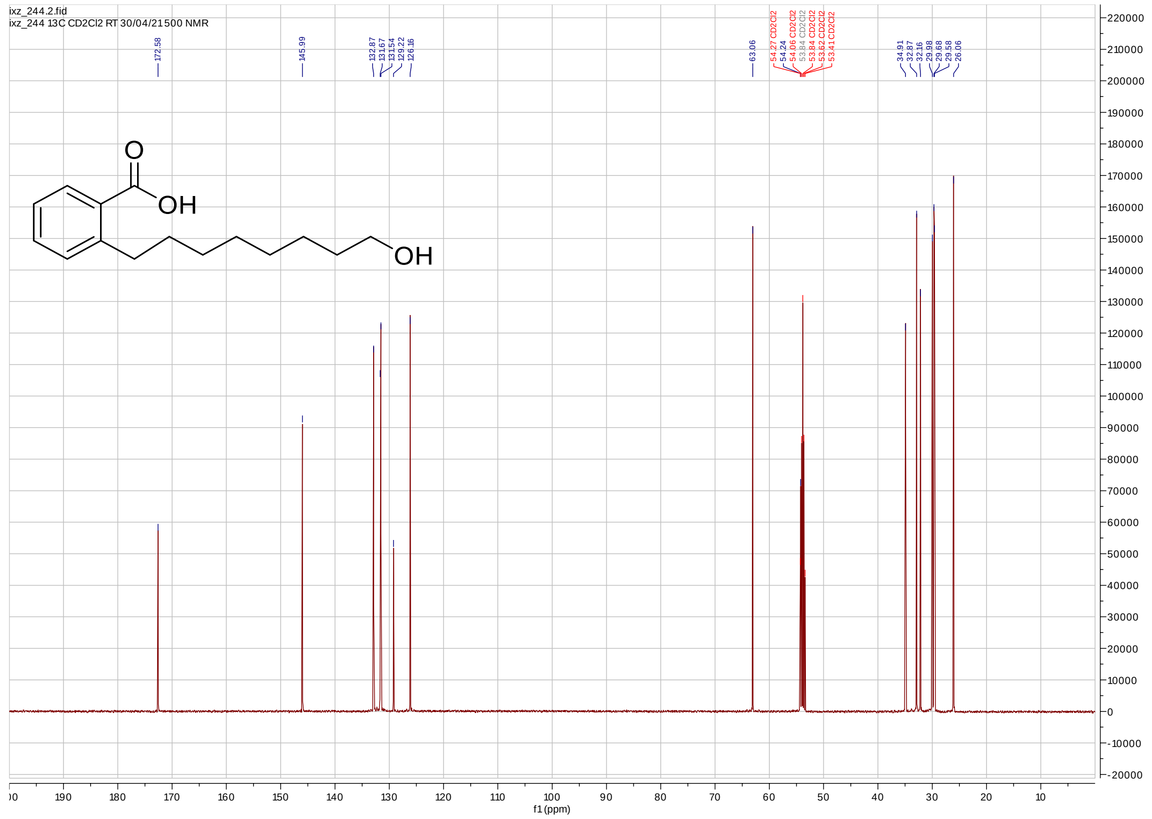

1H-Benzo[d][1,2,3]triazol-1-yl 2-(8-hydroxyoctyl)benzoate (135)

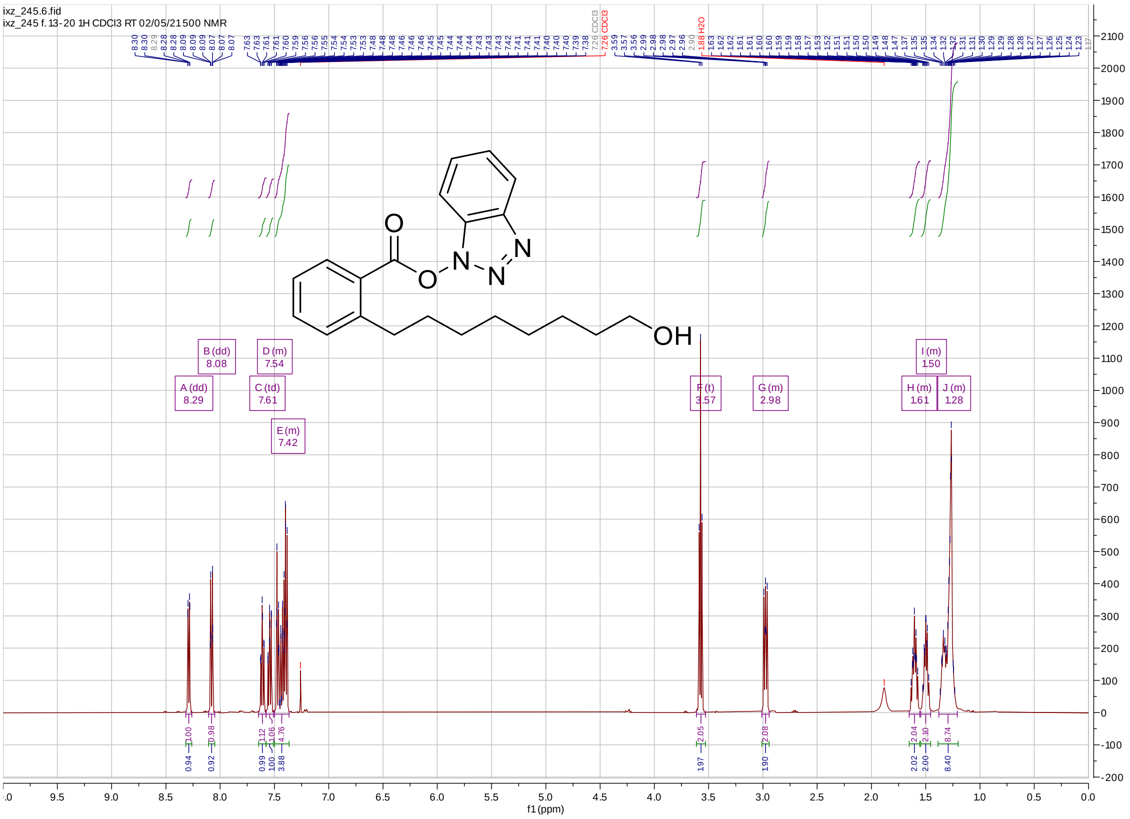

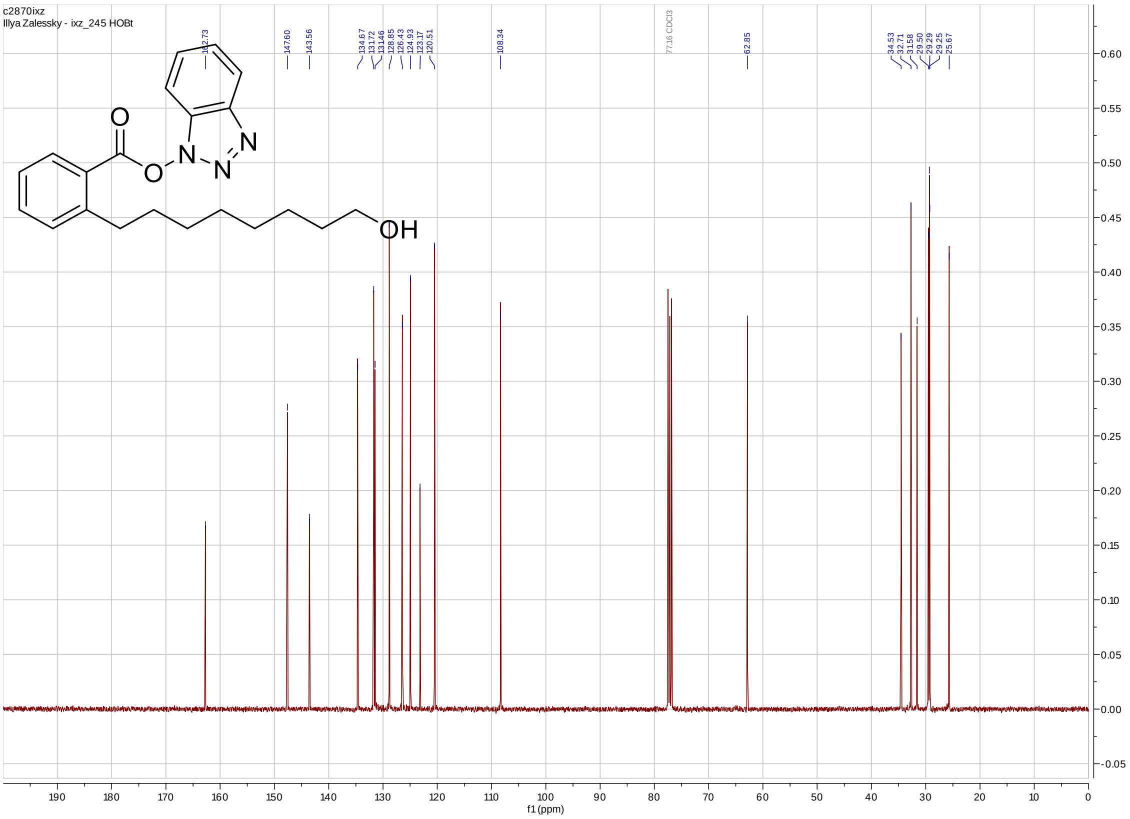

7,8,9,10,11,12,13,14,21,22,23,24,25,26,27,28-hexadecahydro-5H,19H-Dibenzo[c,o][1,13]dioxacyclotetracosine-5,19-dione (136)

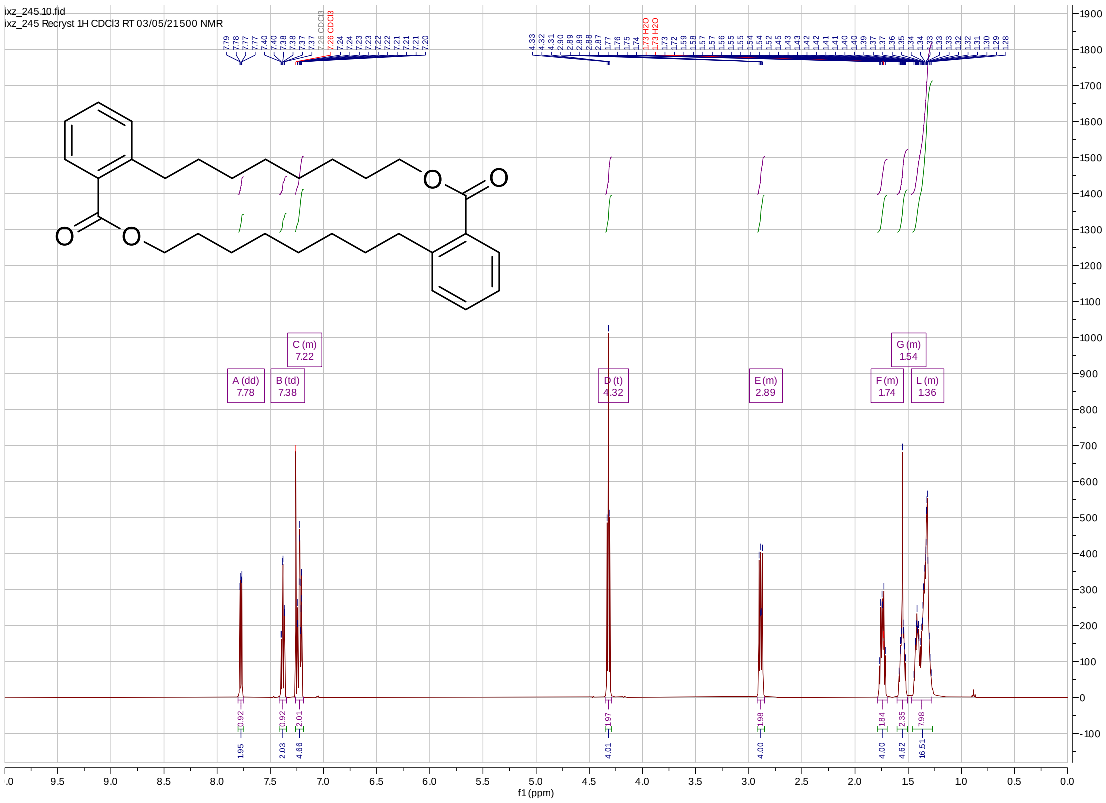

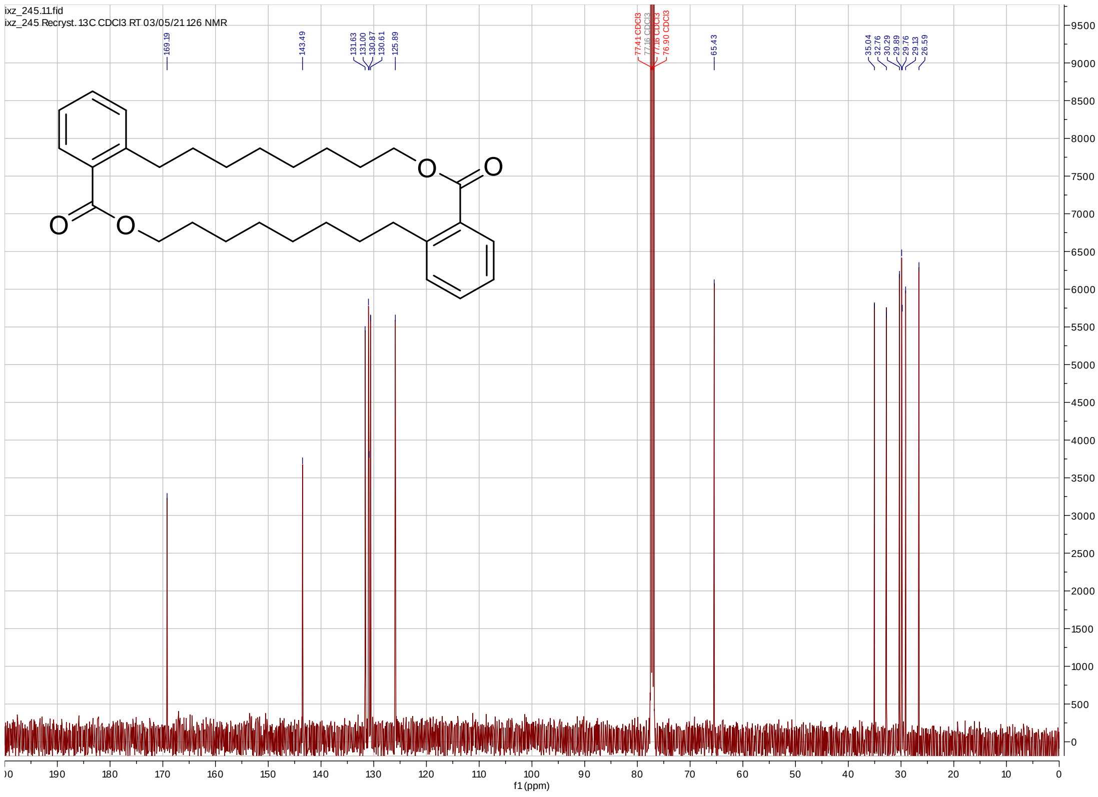

4,4'-(1,2-Phenylene)bis(but-3-yn-1-ol) (S182)

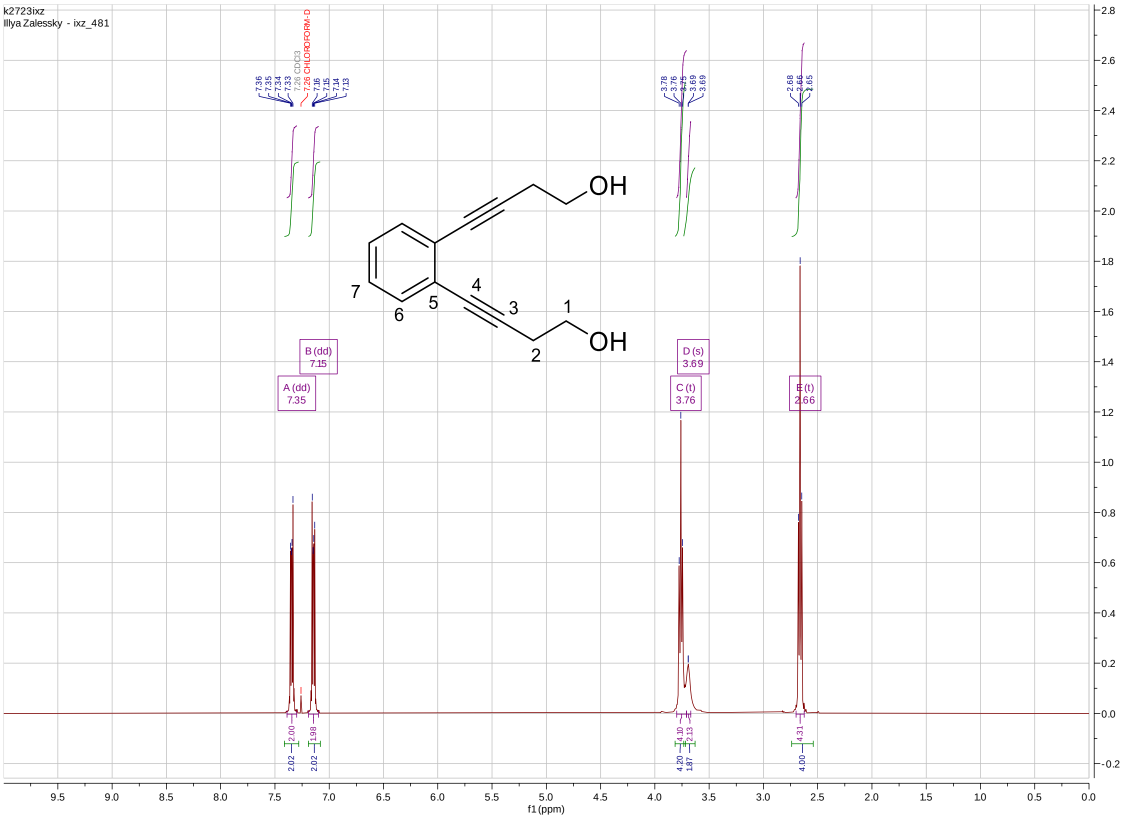

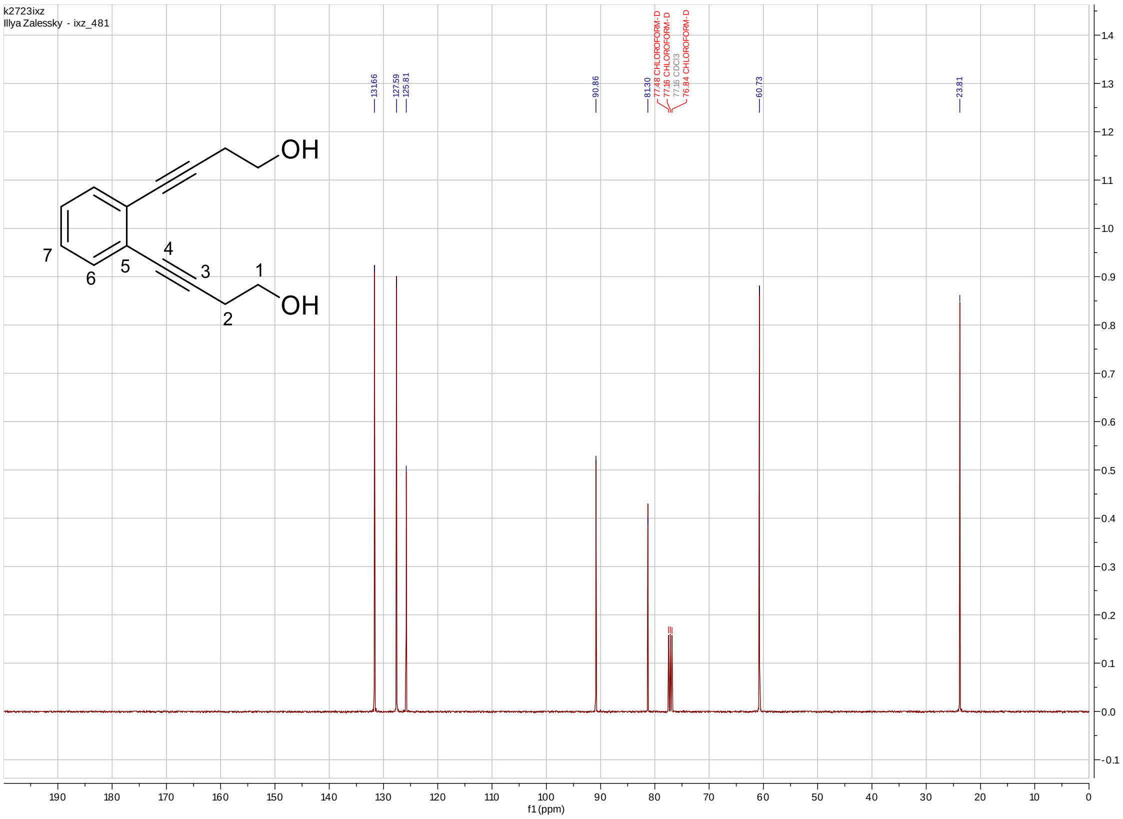

4,4'-(1,2-Phenylene)bis(butan-1-ol) (137)

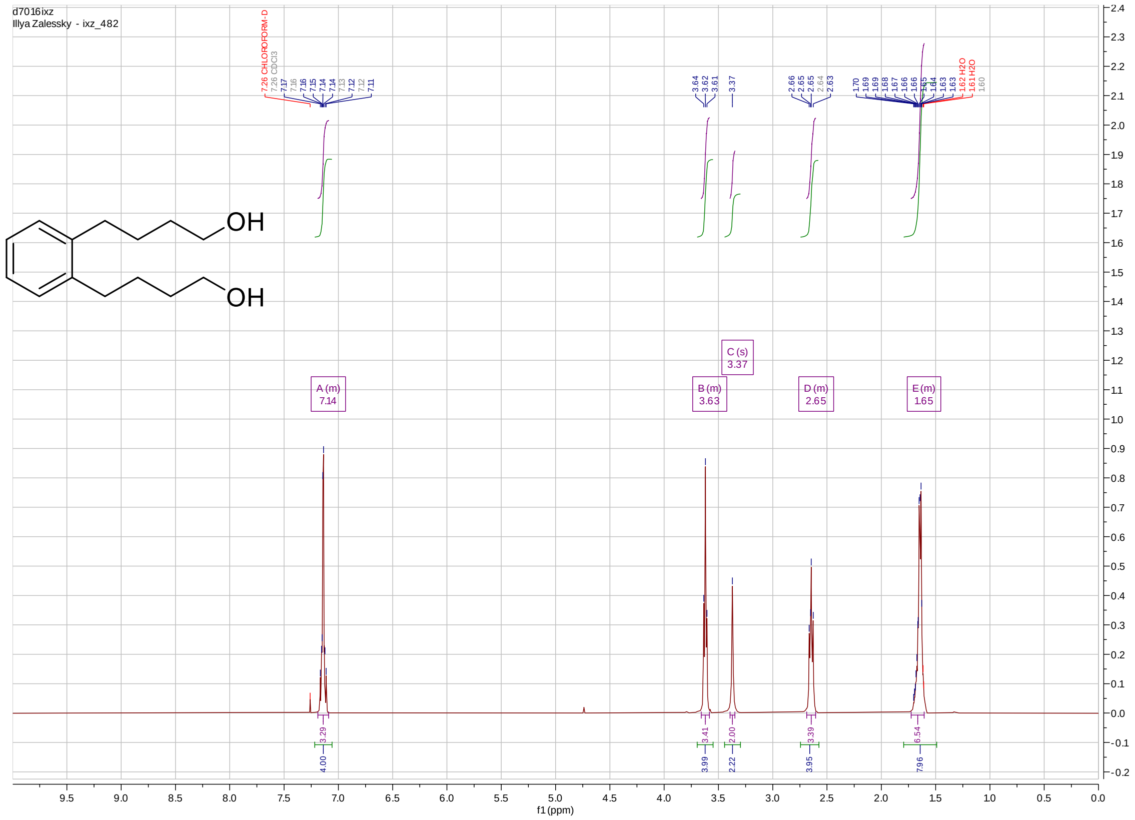

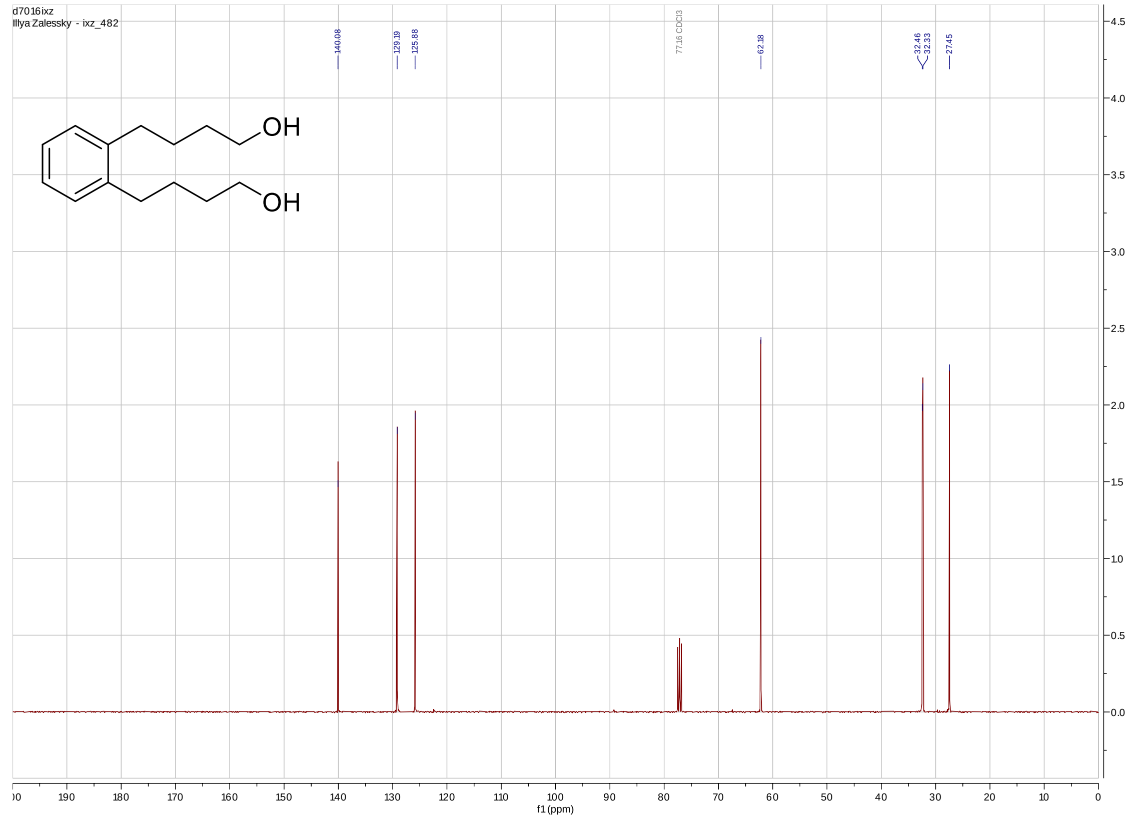

1,2,3,4,8,9,10,11-Octahydrobenzo[h][1,3]dioxa[2]thiacyclotridecine 6-oxide (138)

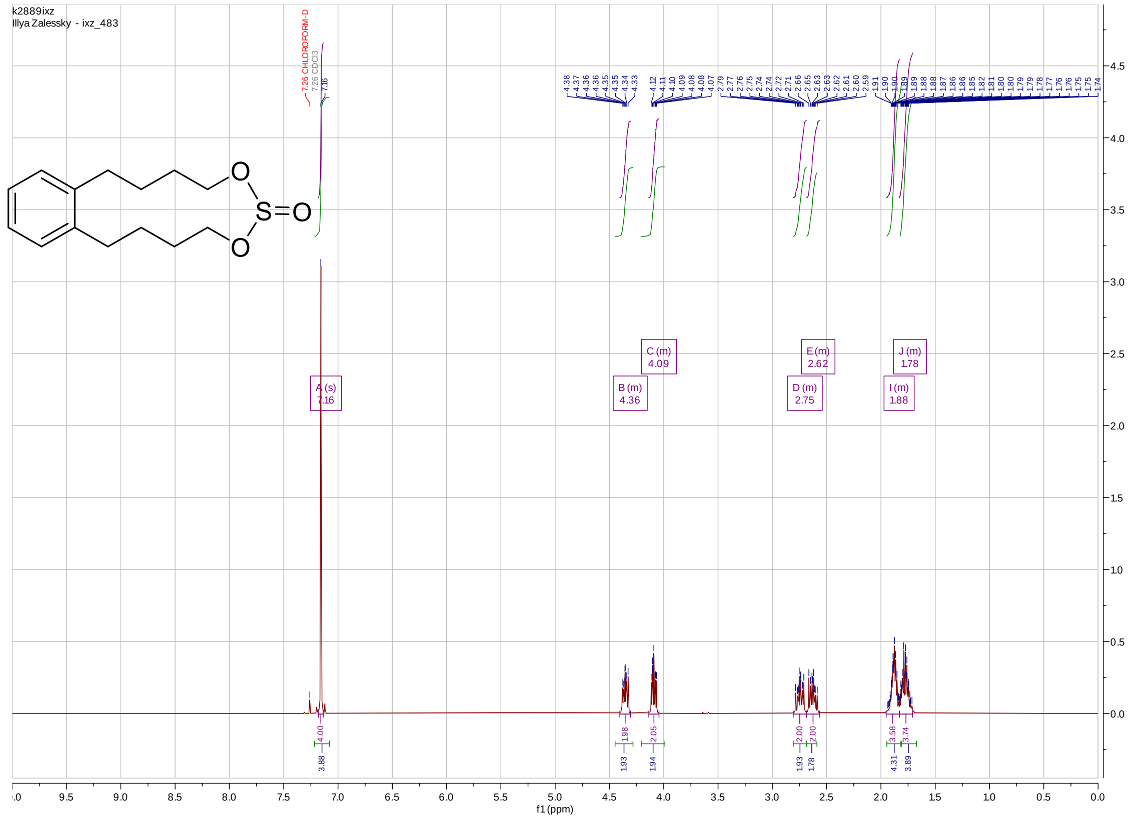

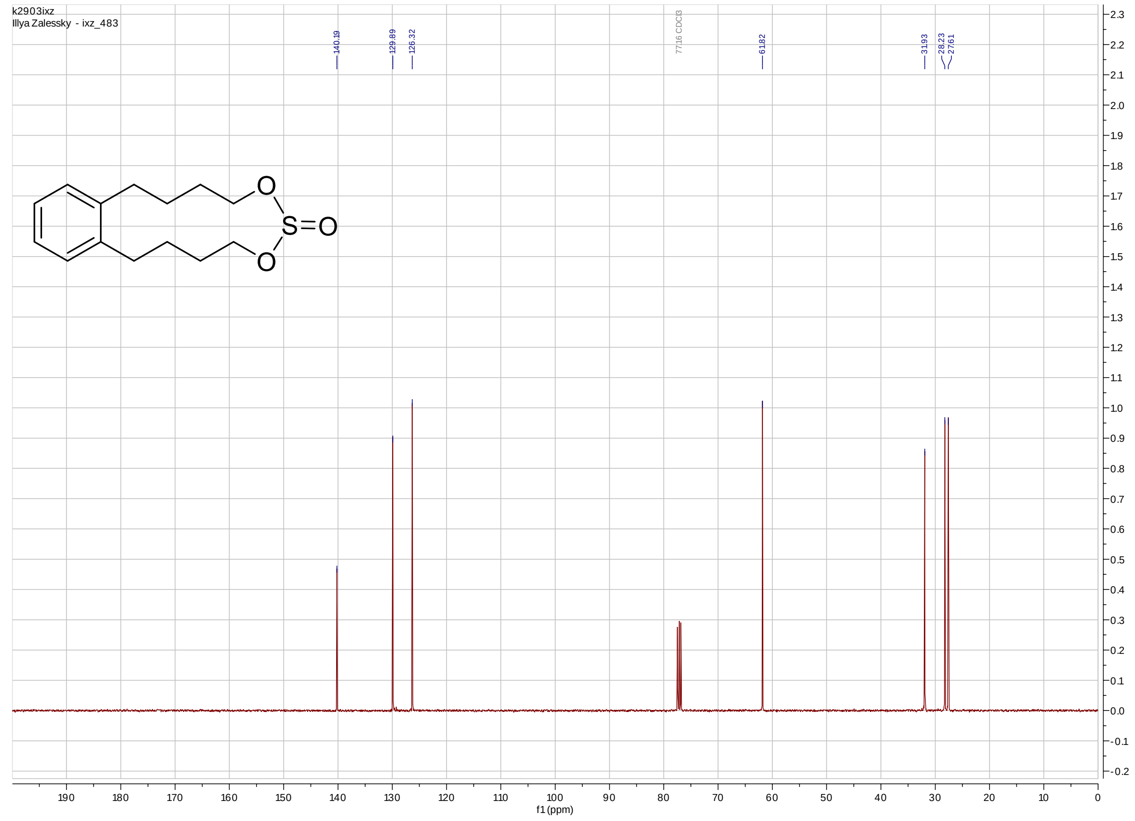

9-Phenyl-4,5-dihydro-3*H*-benzo[*g*][1,5]oxathionin-1(7*H*)-one (139)

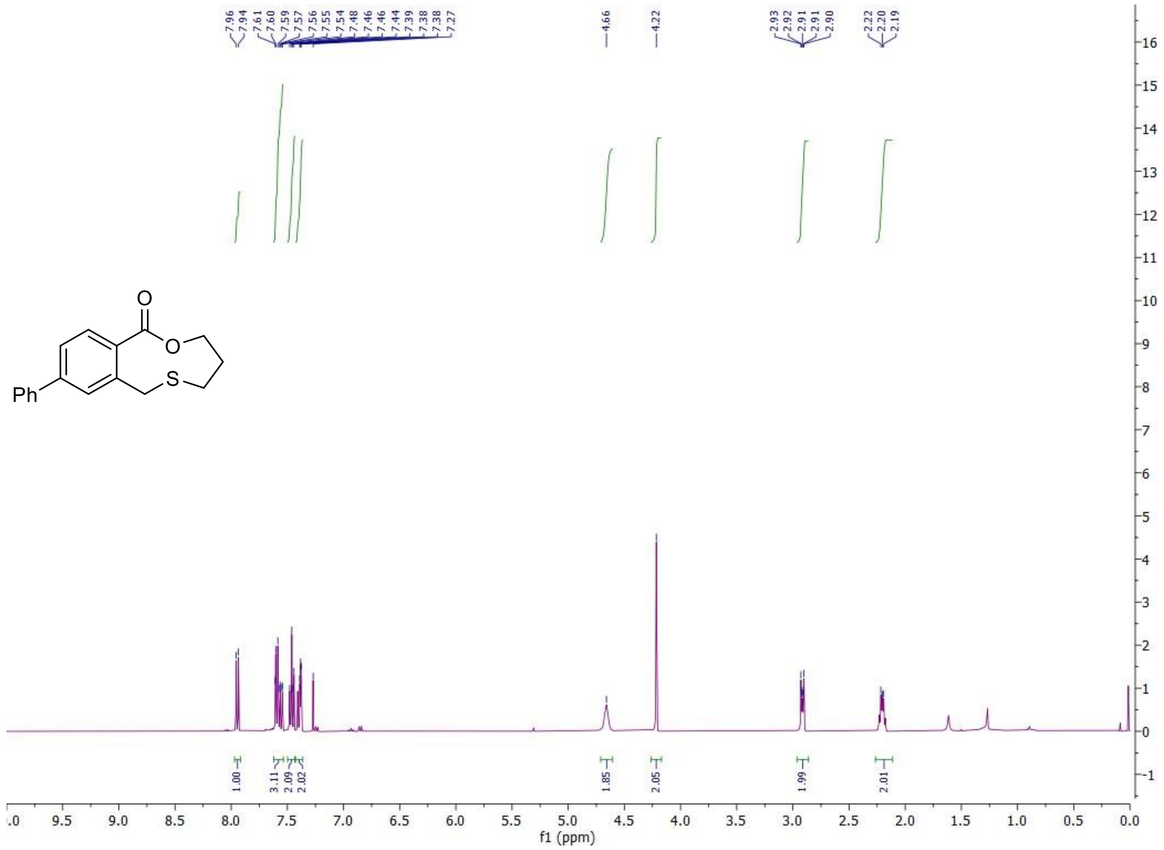

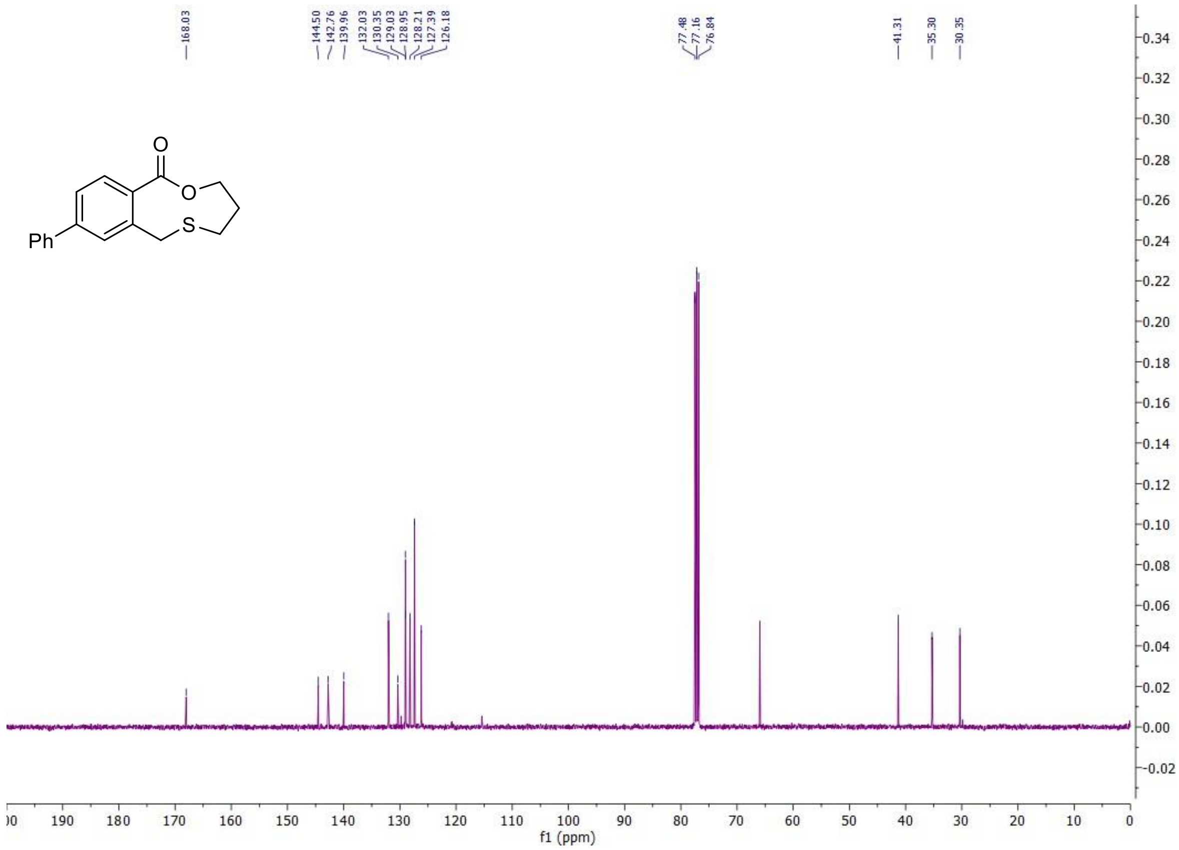

Methyl 2-((butylthio)methyl)benzoate (S183)

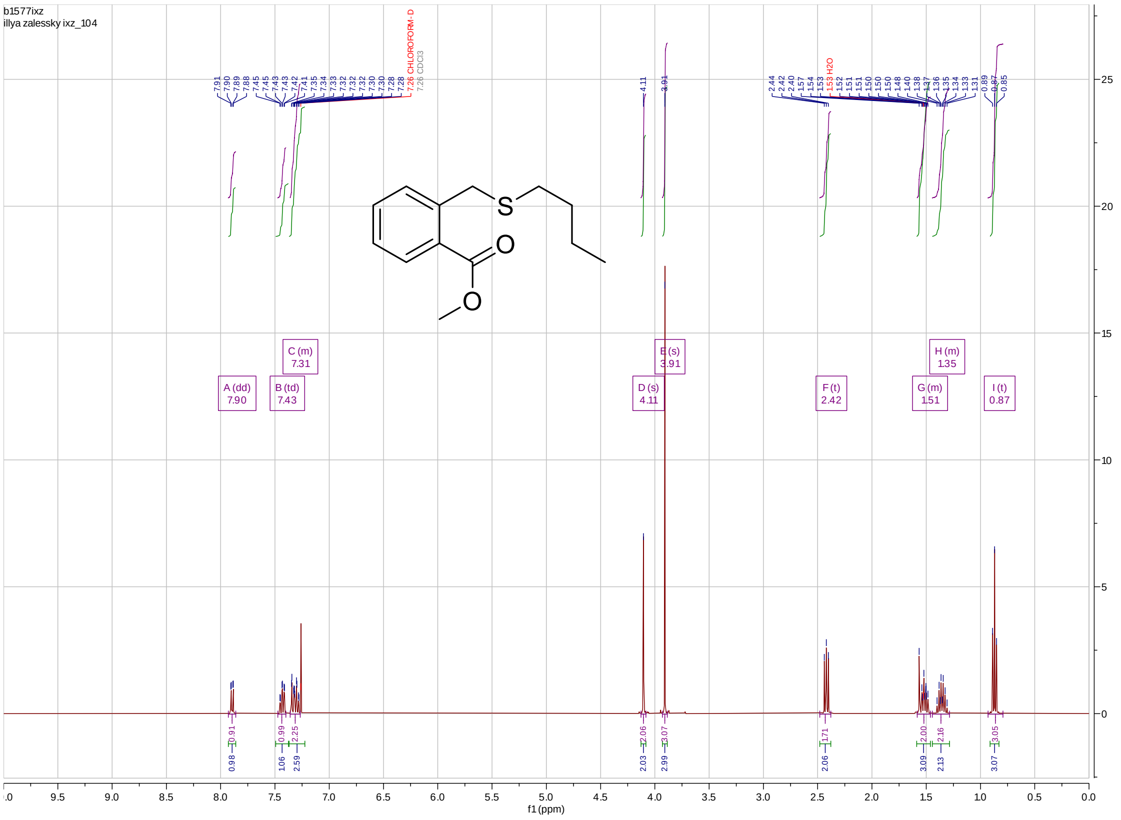

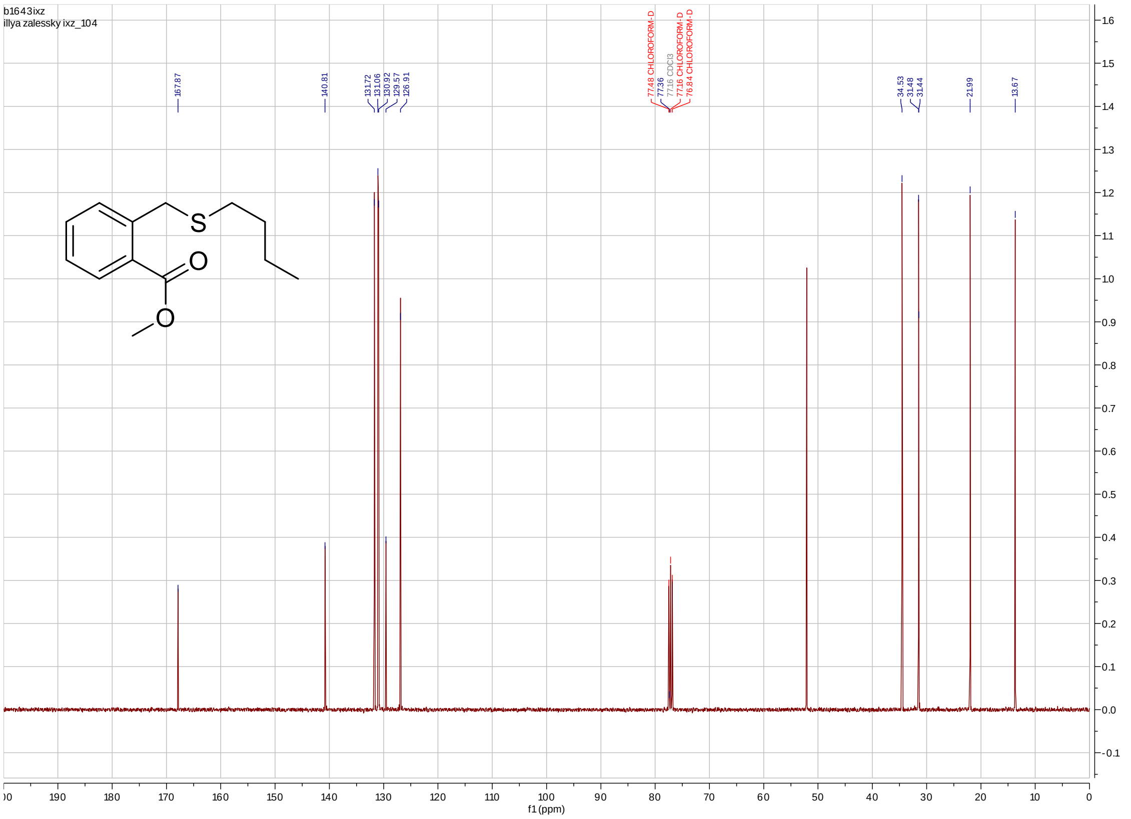

2-((Butylthio)methyl)benzoic acid (S184)

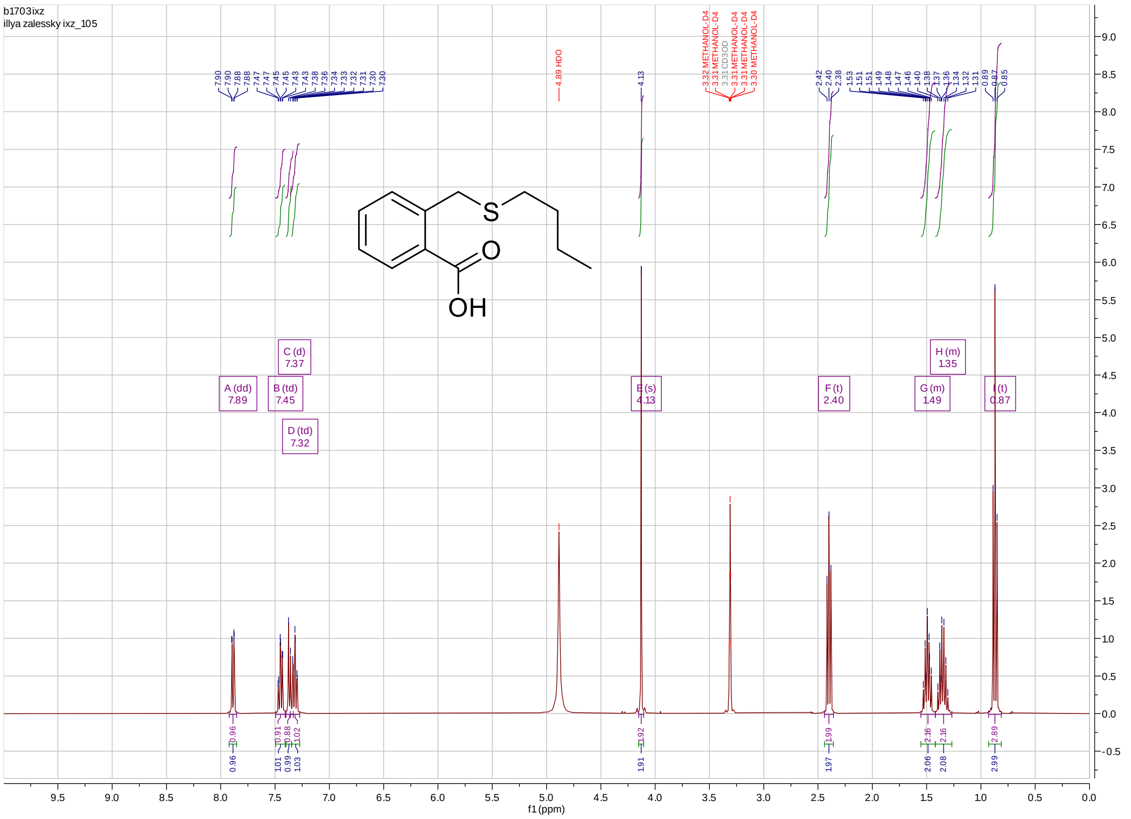

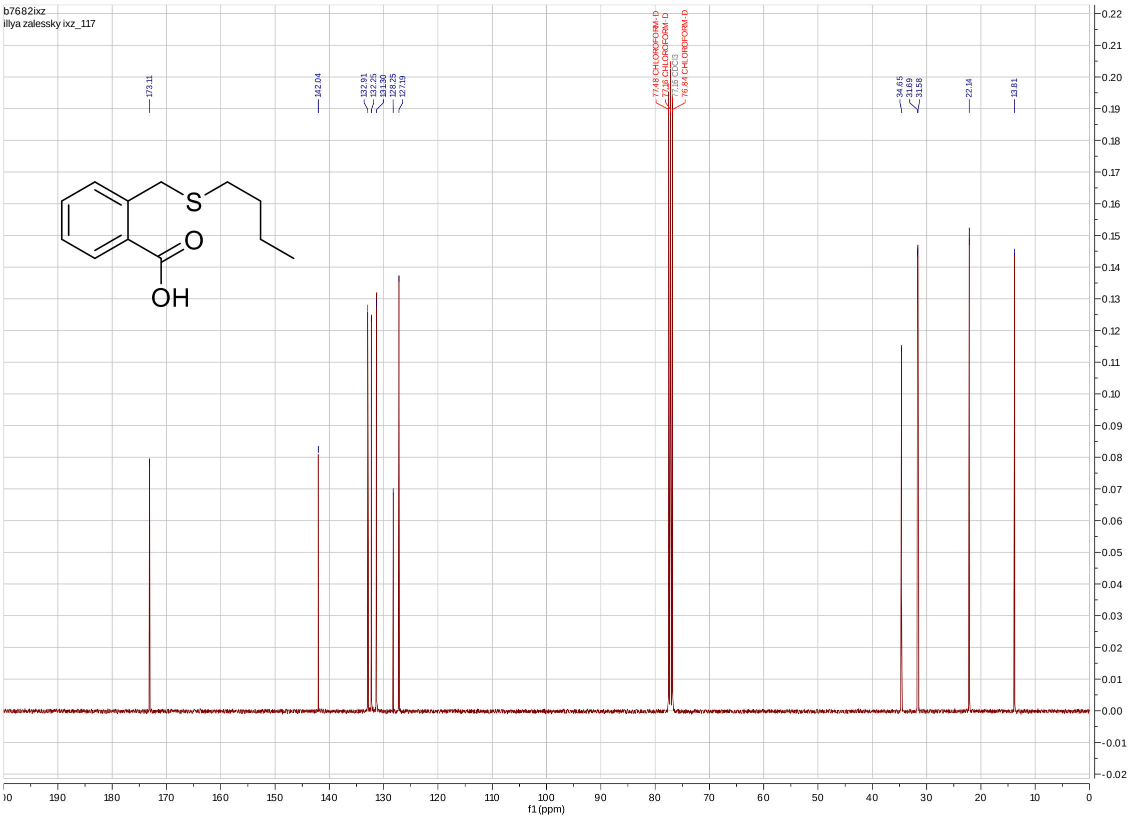

2: *tert*-Butyl (3-(benzylamino)propyl)carbamate (S185)

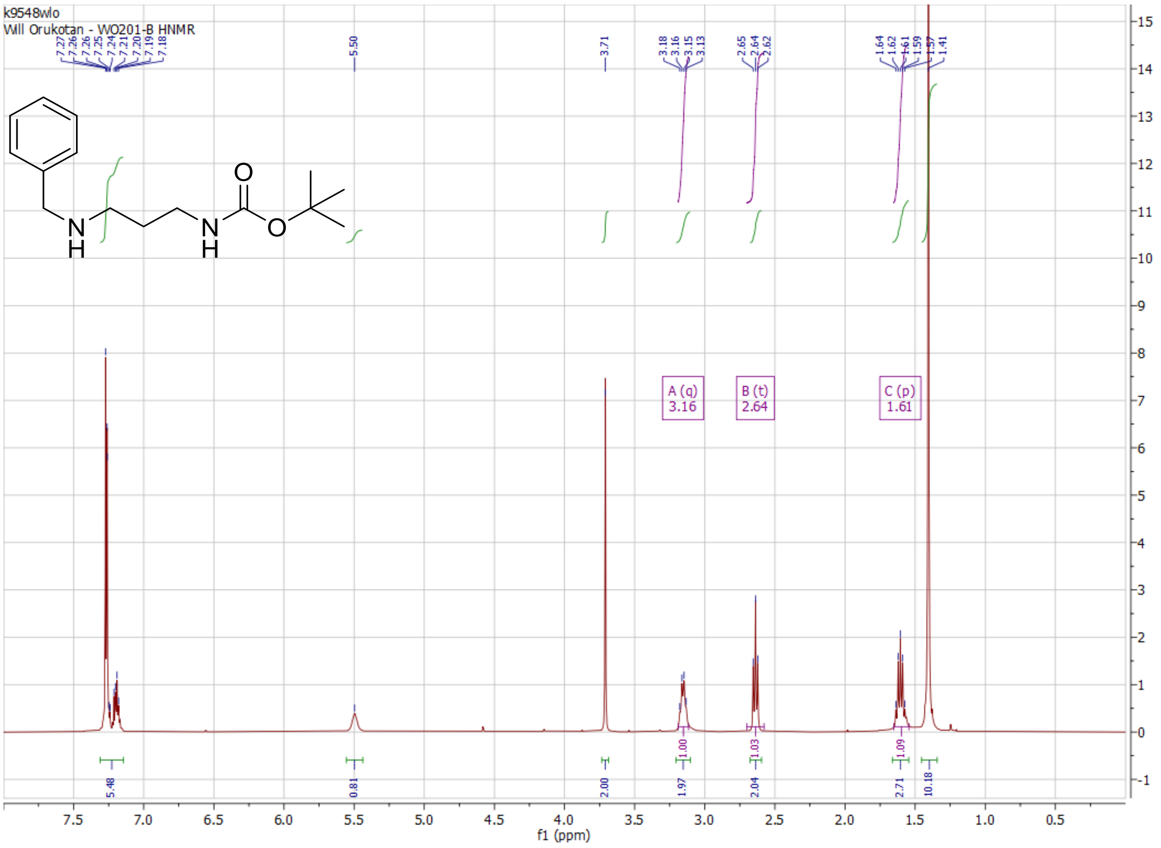

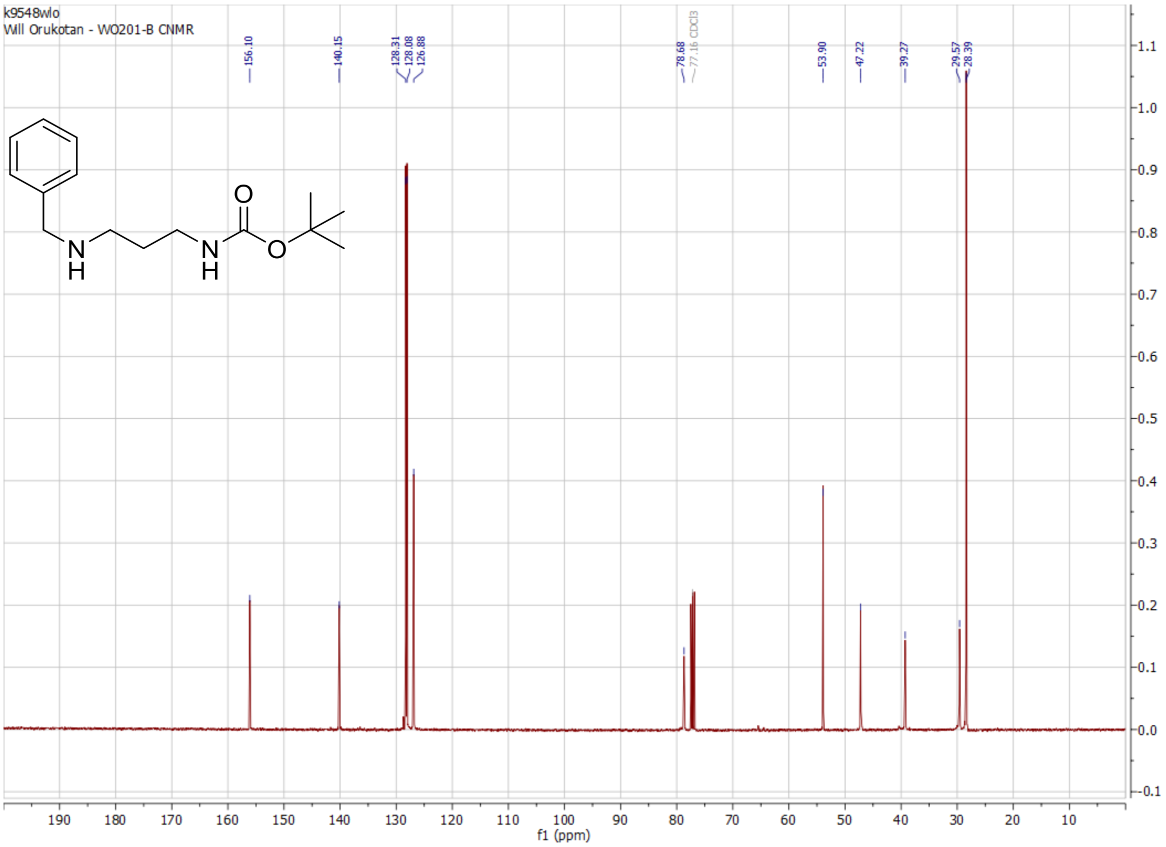

tert-Butyl 4-(benzyl(3-((tert-butoxycarbonyl)amino)propyl)amino)butanoate (S186)

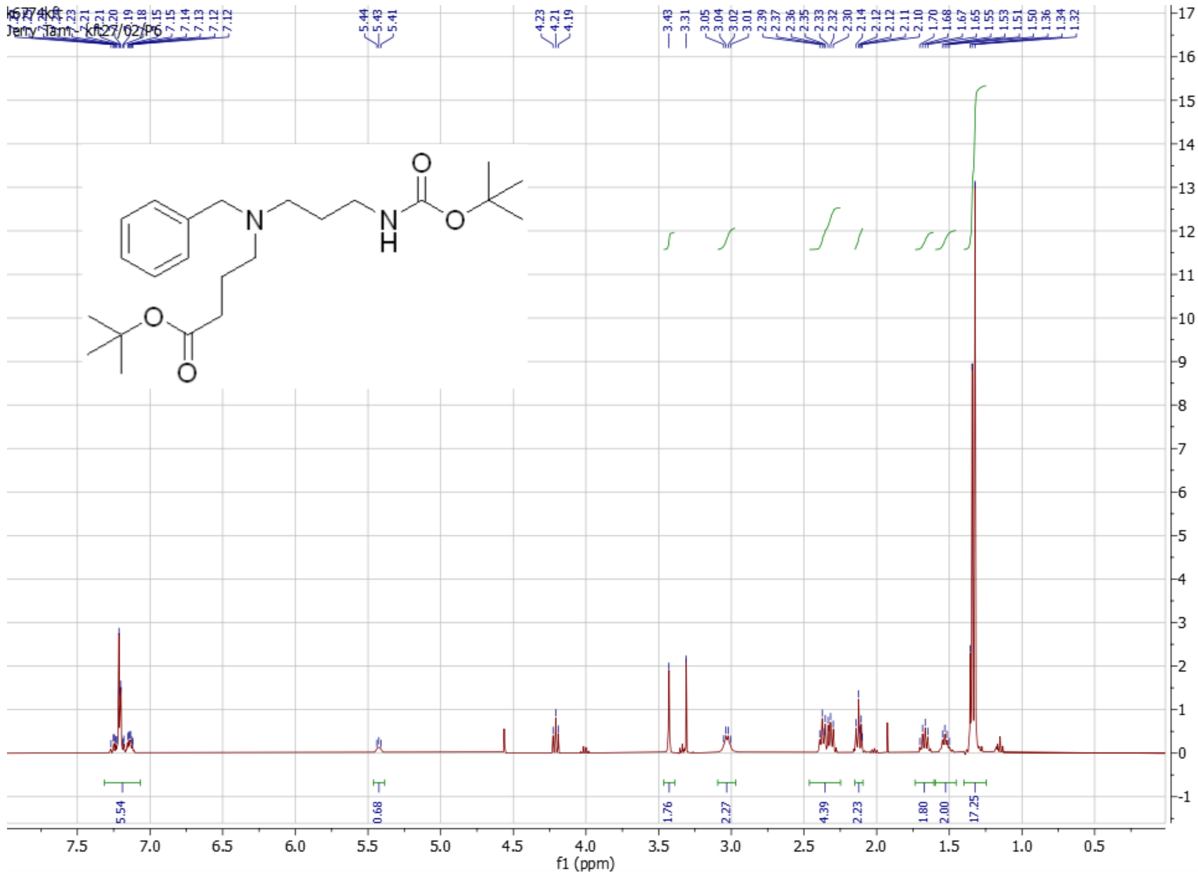

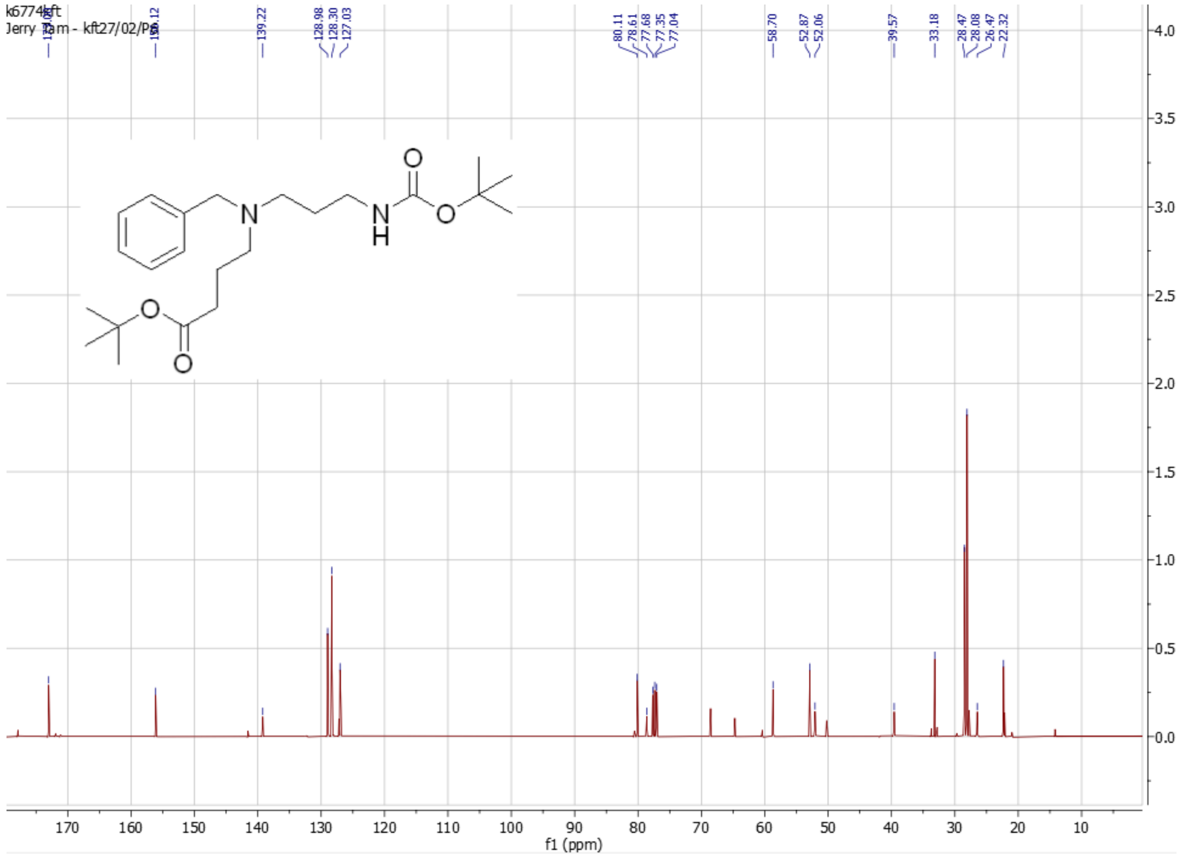

1-Benzyl-1,5-diazonan-6-one (140)

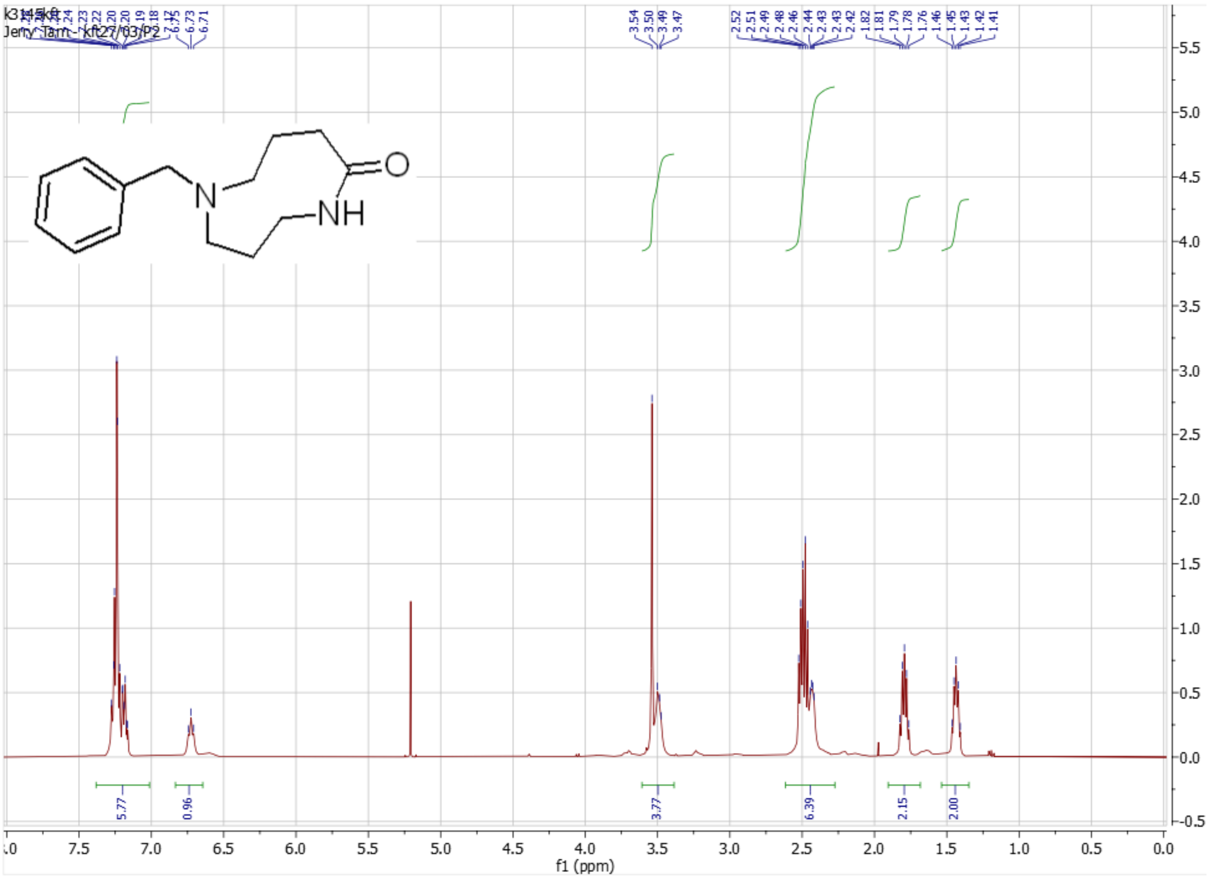

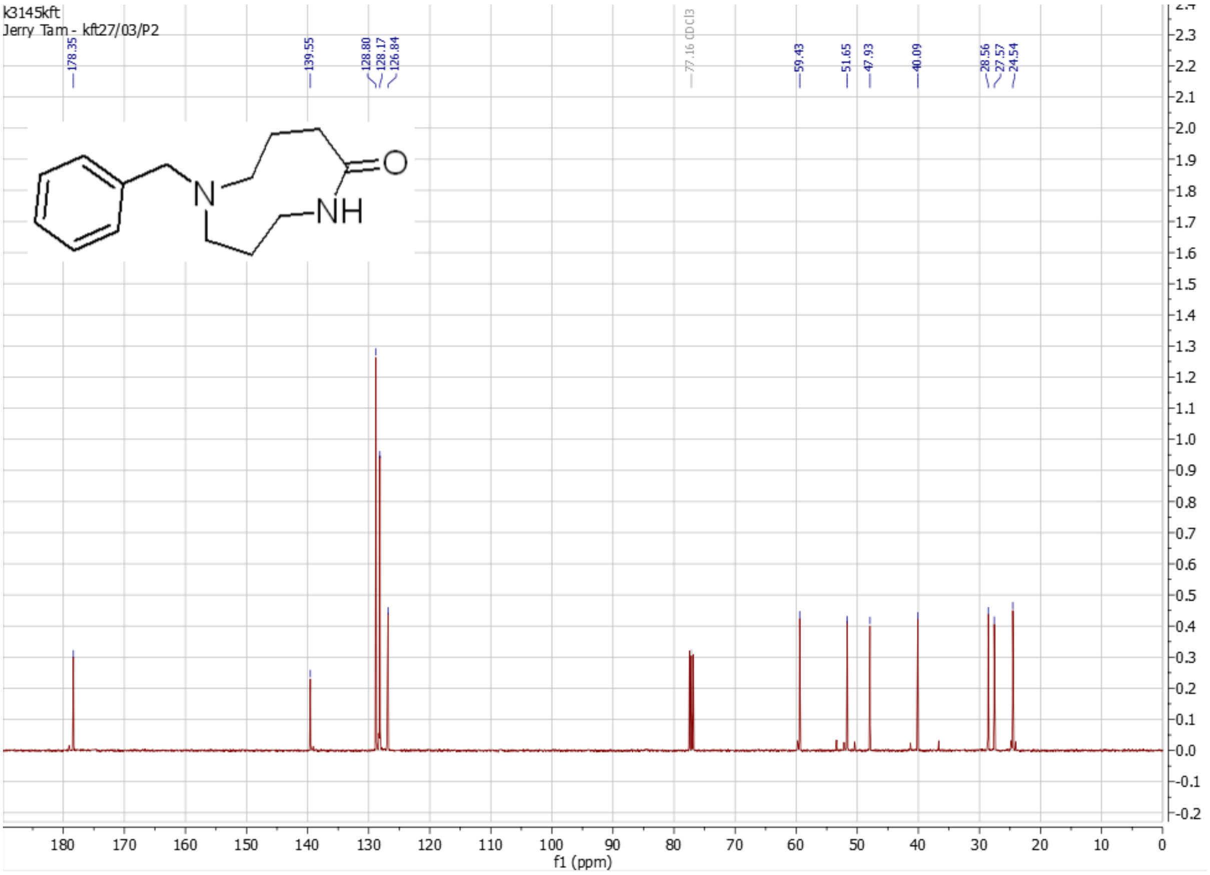

Dodecahydro-1*H*-pyrrolo[1,2-*e*][1,5]diazonine (143)

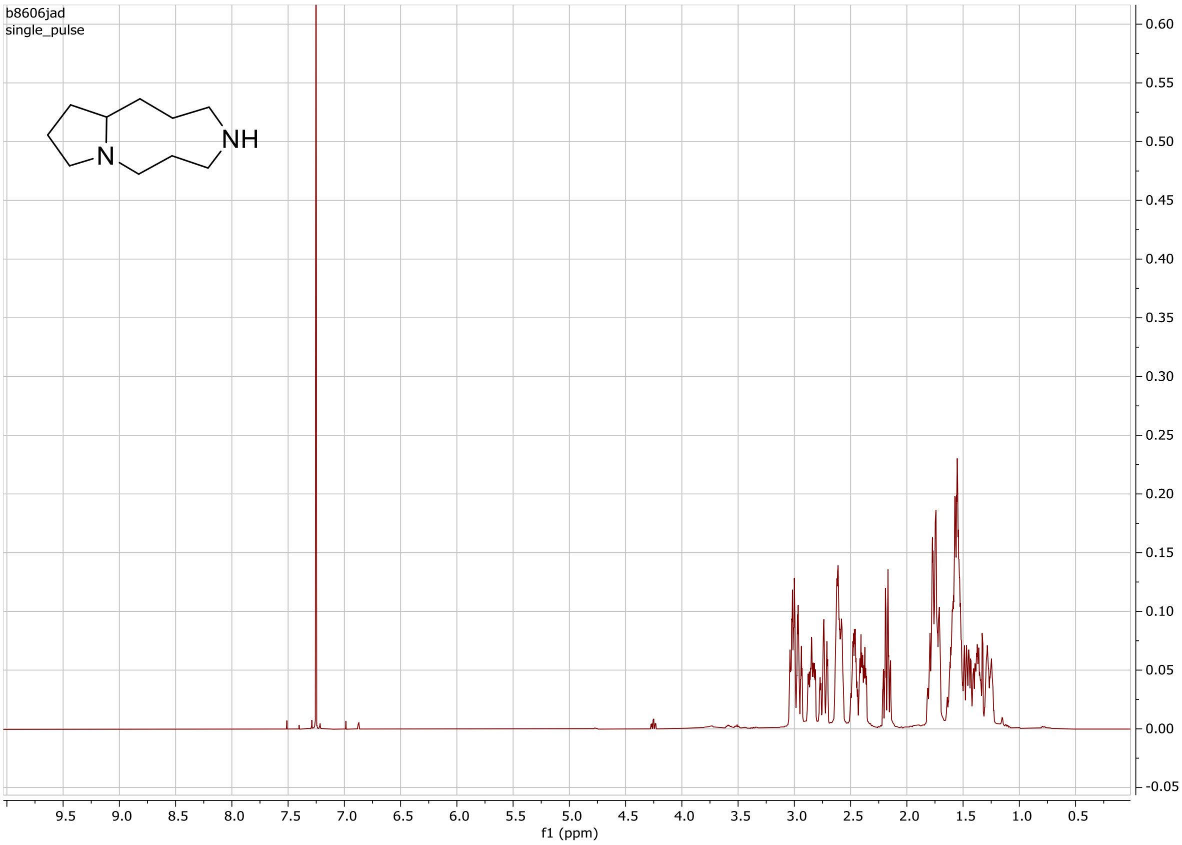

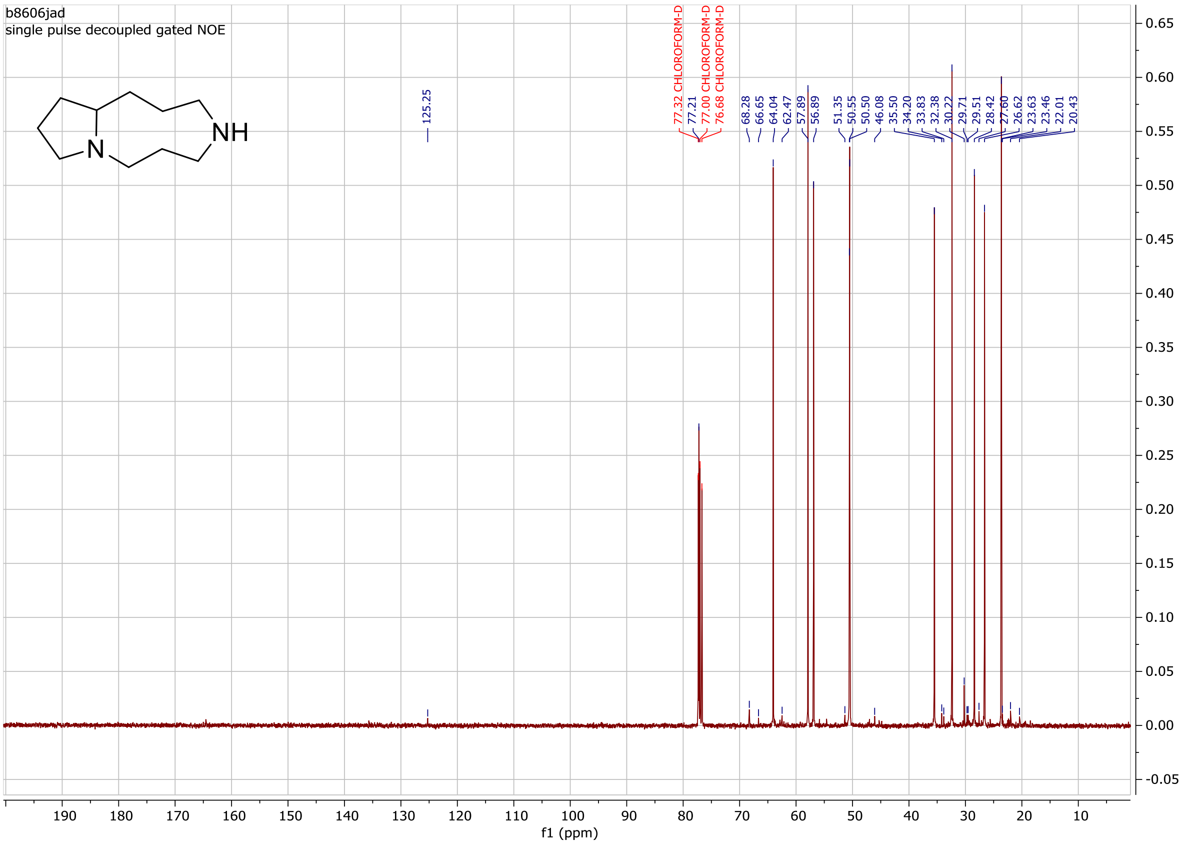

Dodecahydropyrido[1,2-*e*][1,5]diazonine (144)

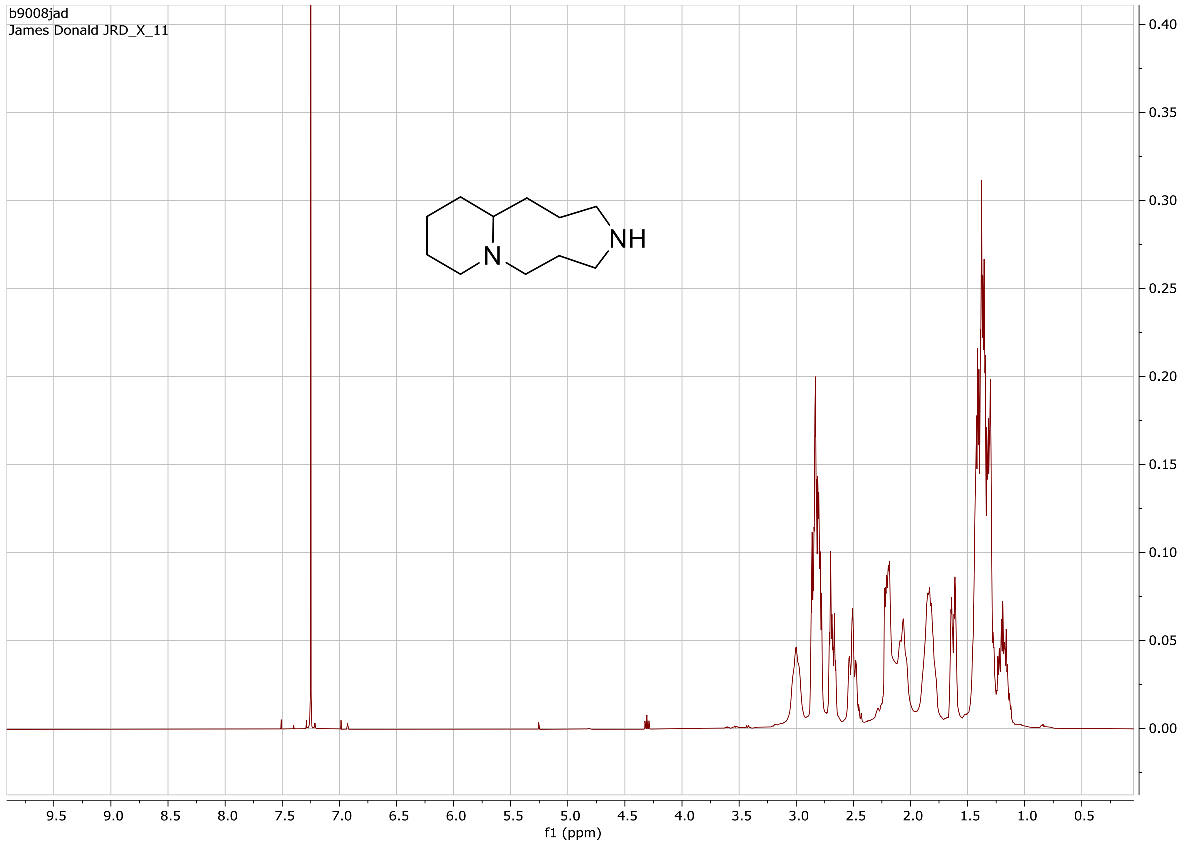

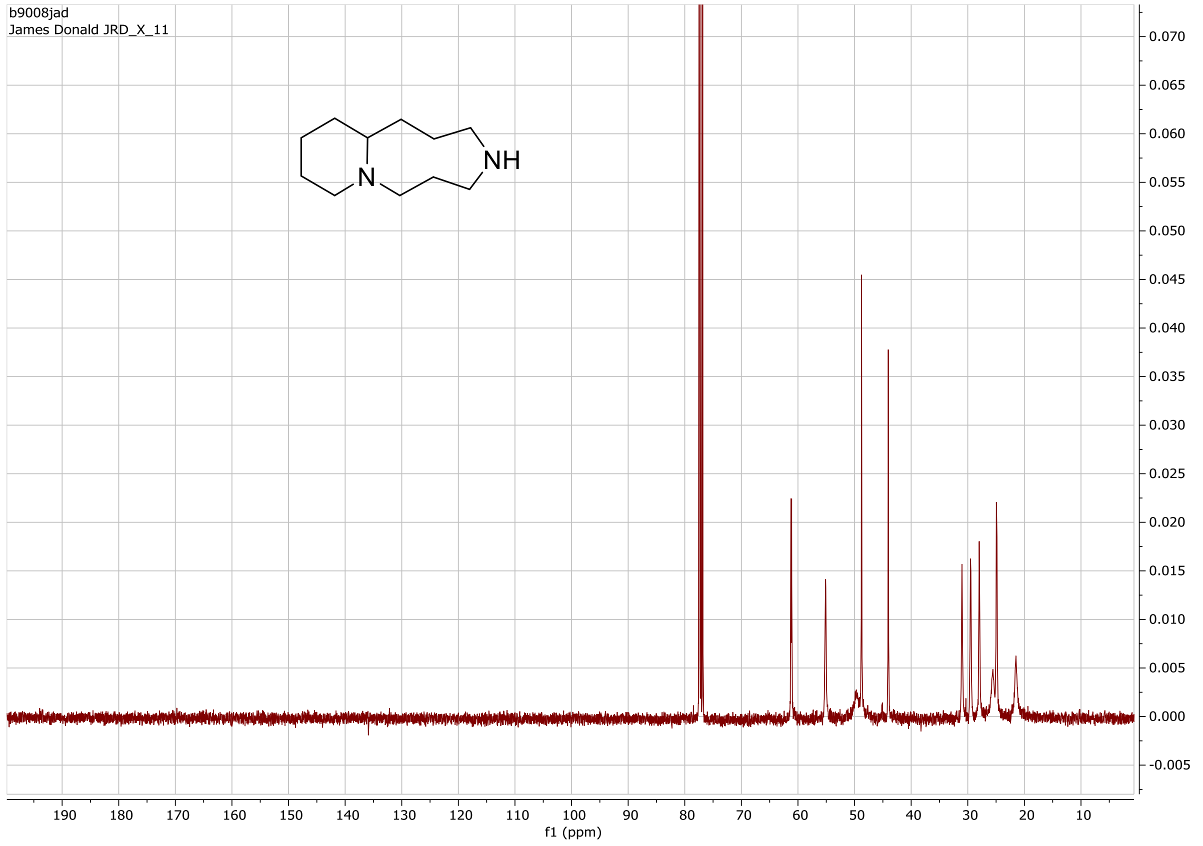

Methyl 4-(benzyl(3-(vinylsulfonamido)propyl)amino)butanoate (S187)

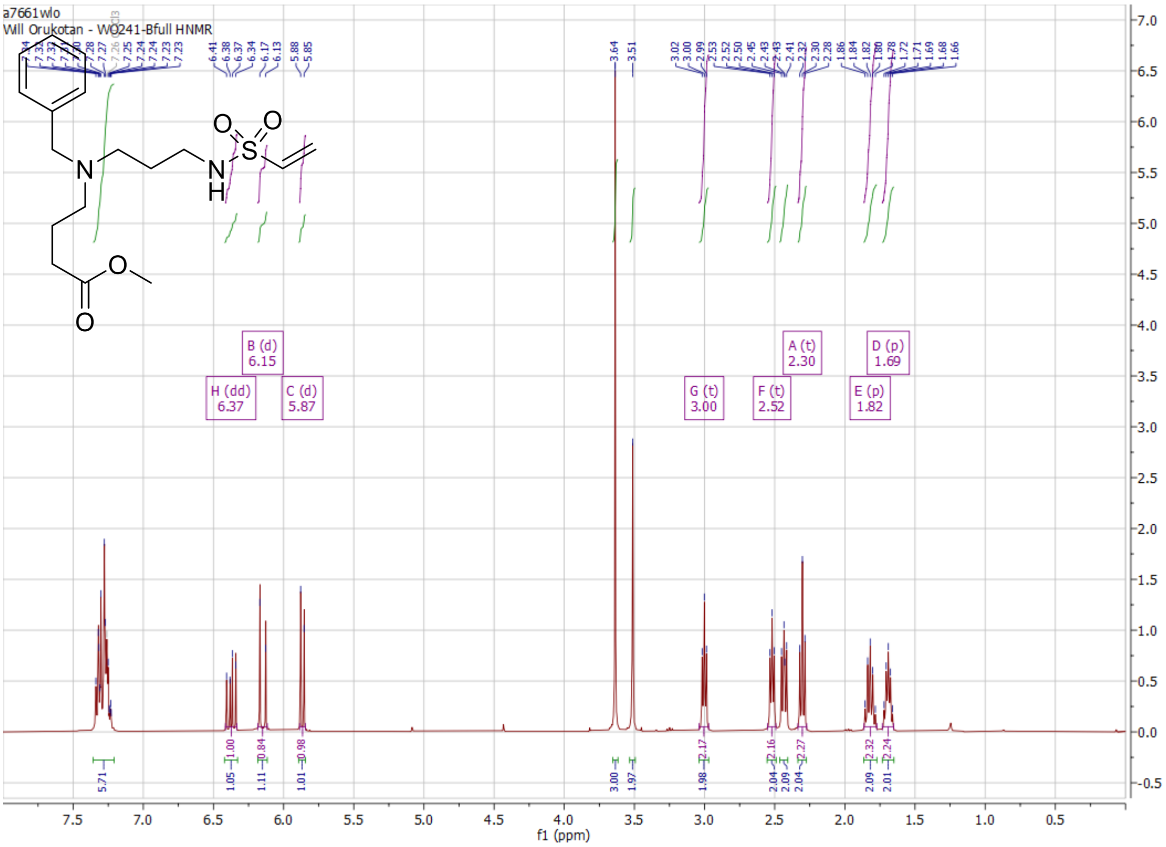

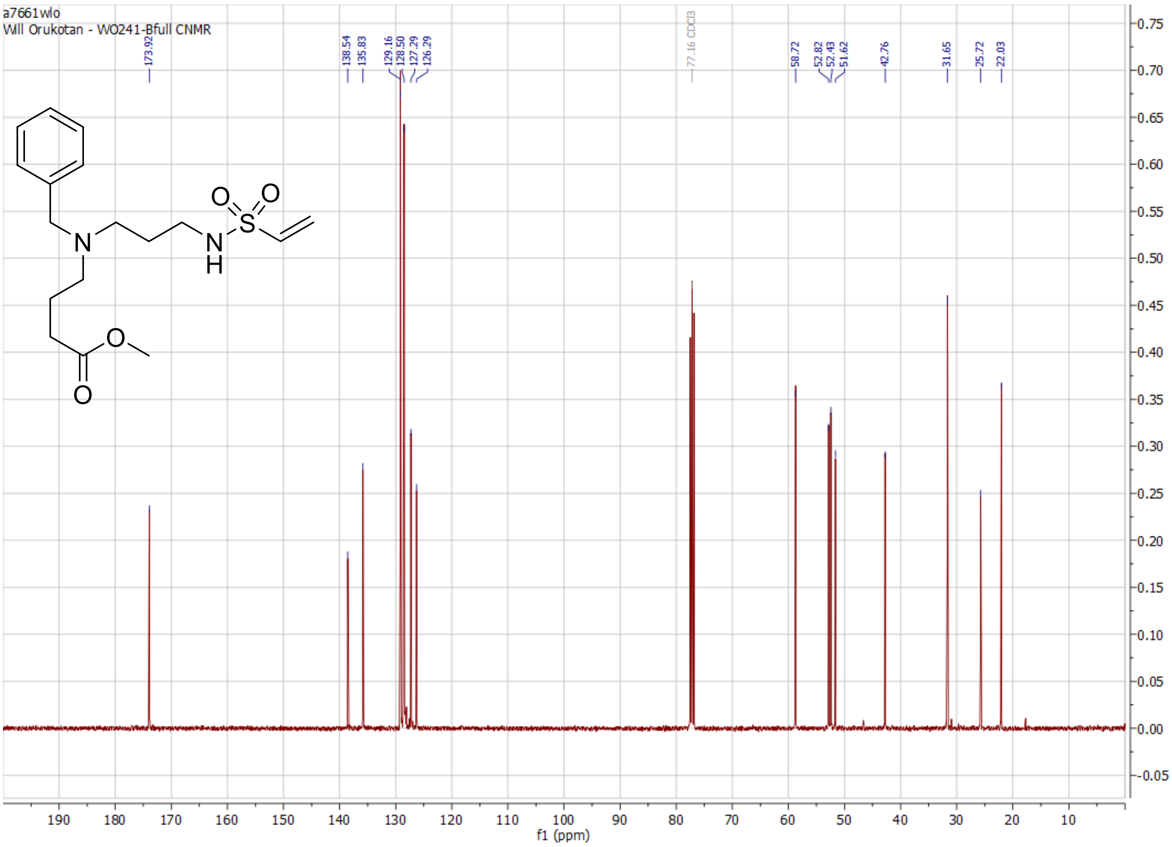

Methyl 4-(benzyl(3-(vinylsulfonamido)propyl)amino)butanoic acid (145)

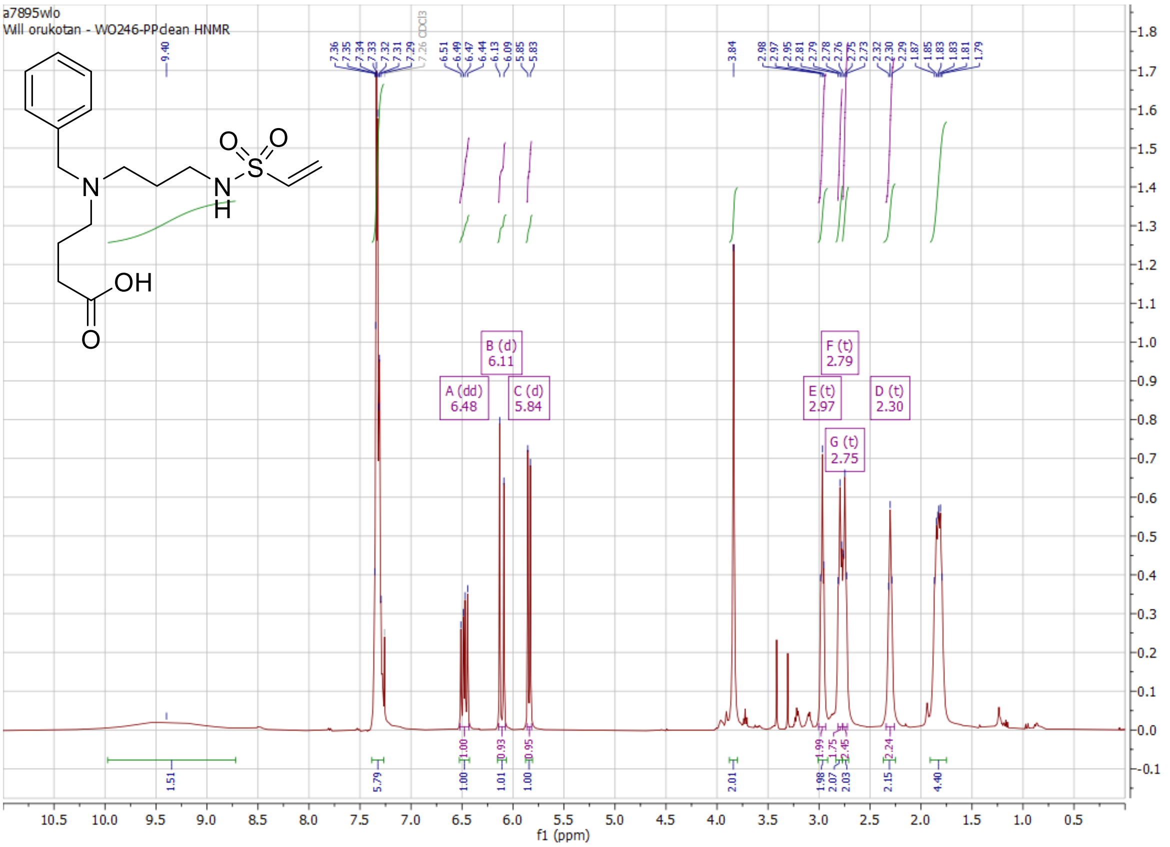

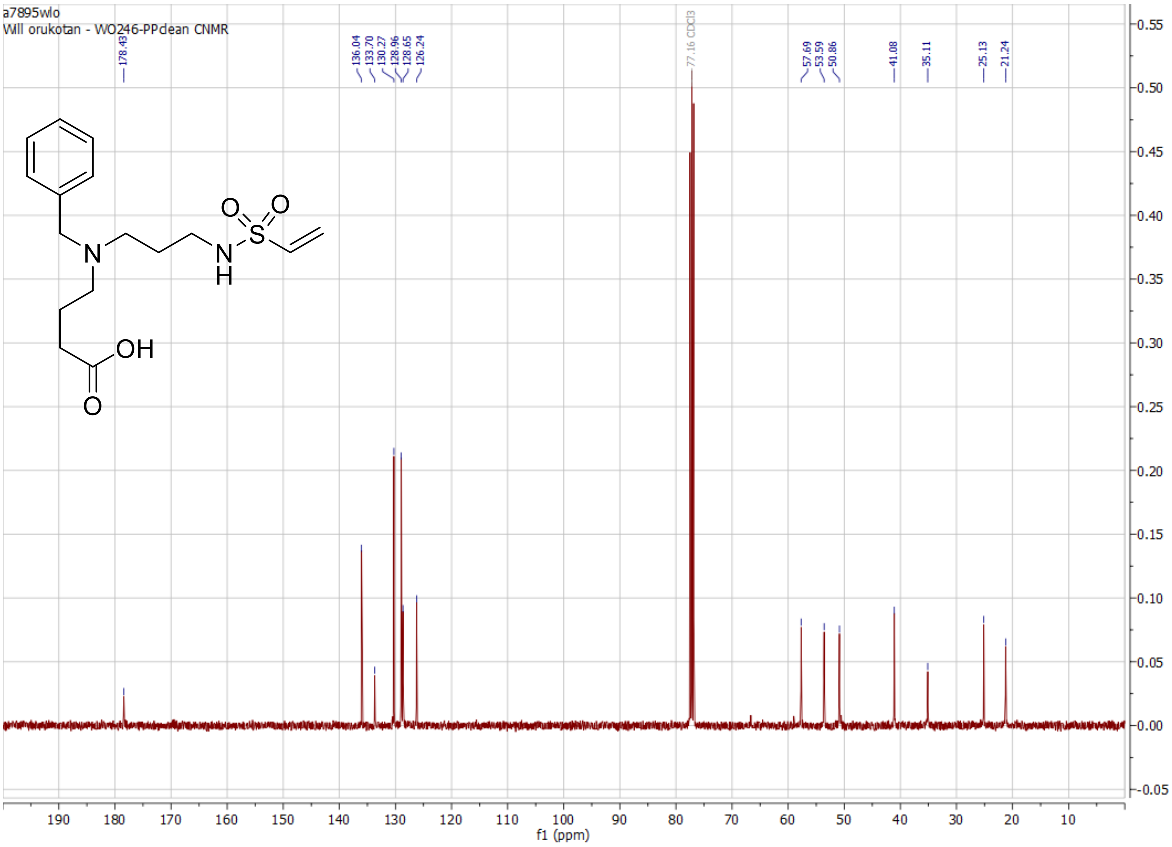

6-Benzyl-11-(4-fluorobenzyl)-1-thia-2,6,11-triazacyclotridecan-10-one 1,1-dioxide (147)

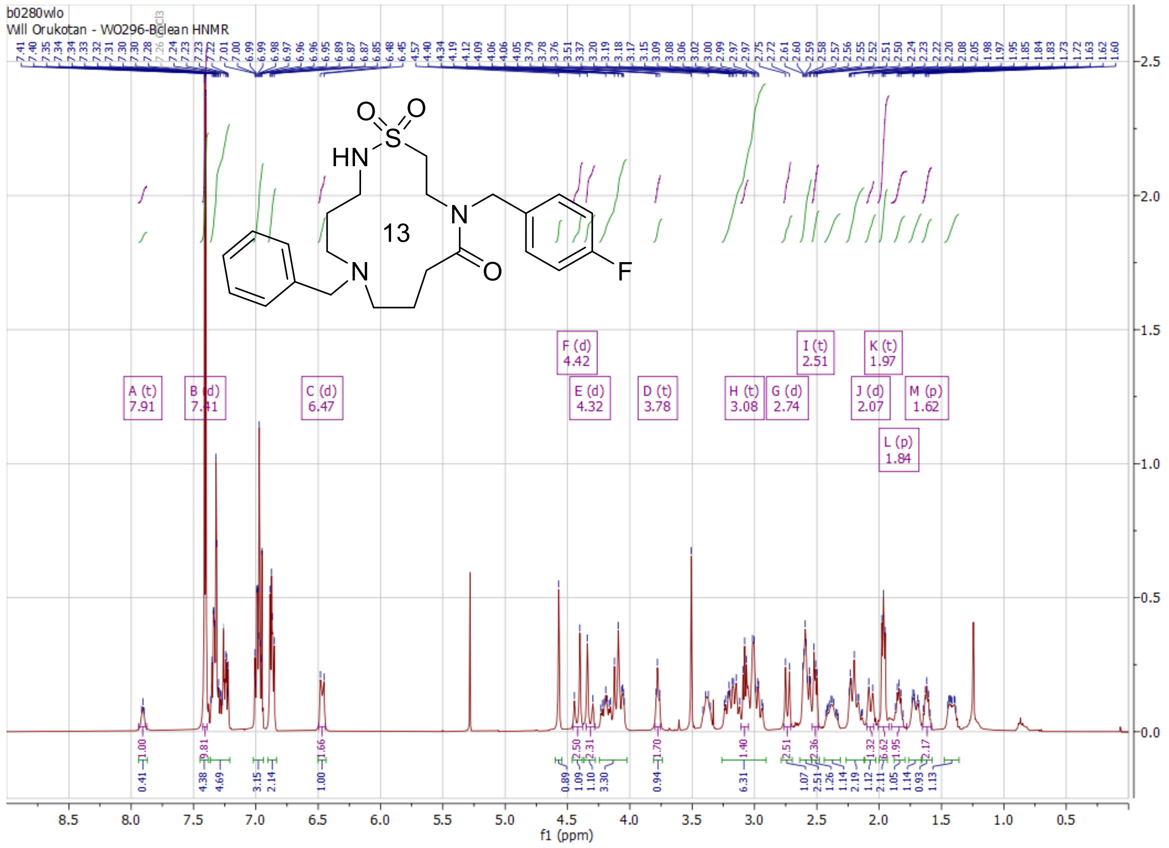

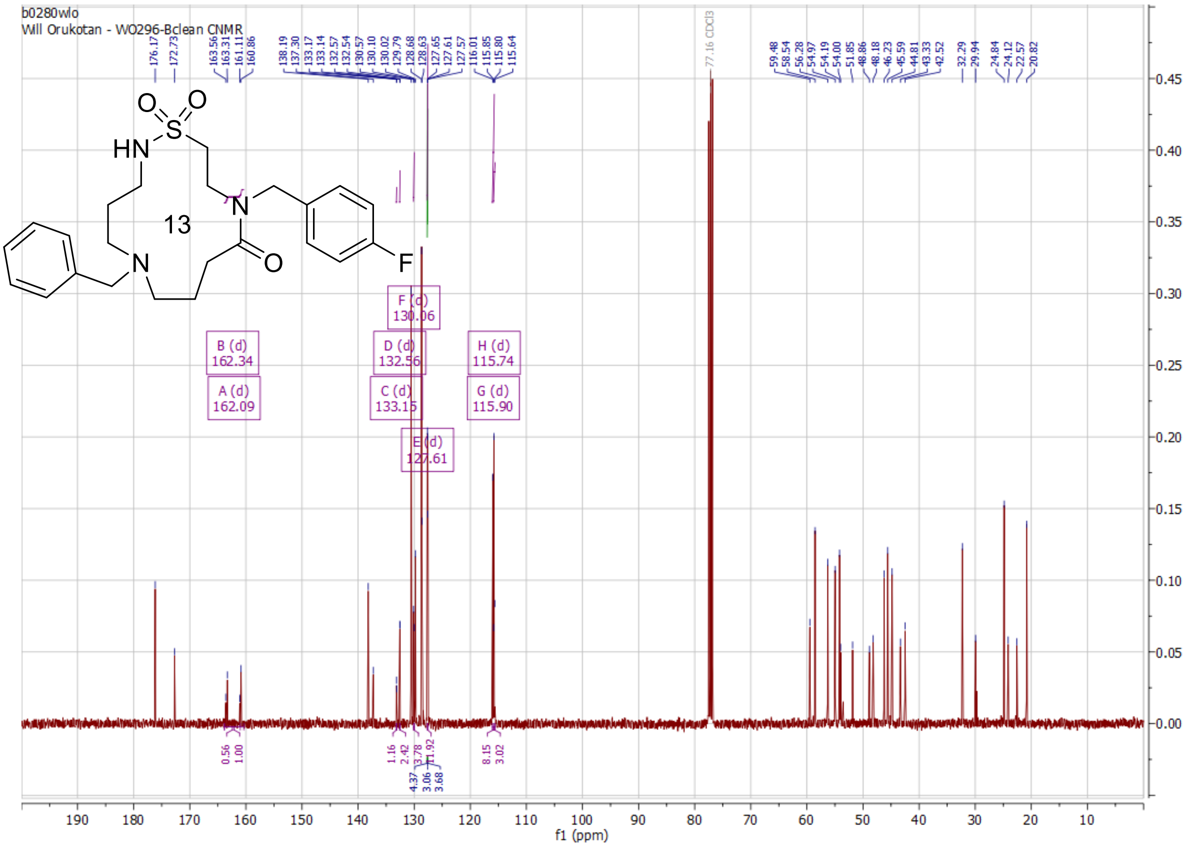

Methyl 2-((2-(hydroxymethyl)azetidin-1-yl)methyl)benzoate (S188)

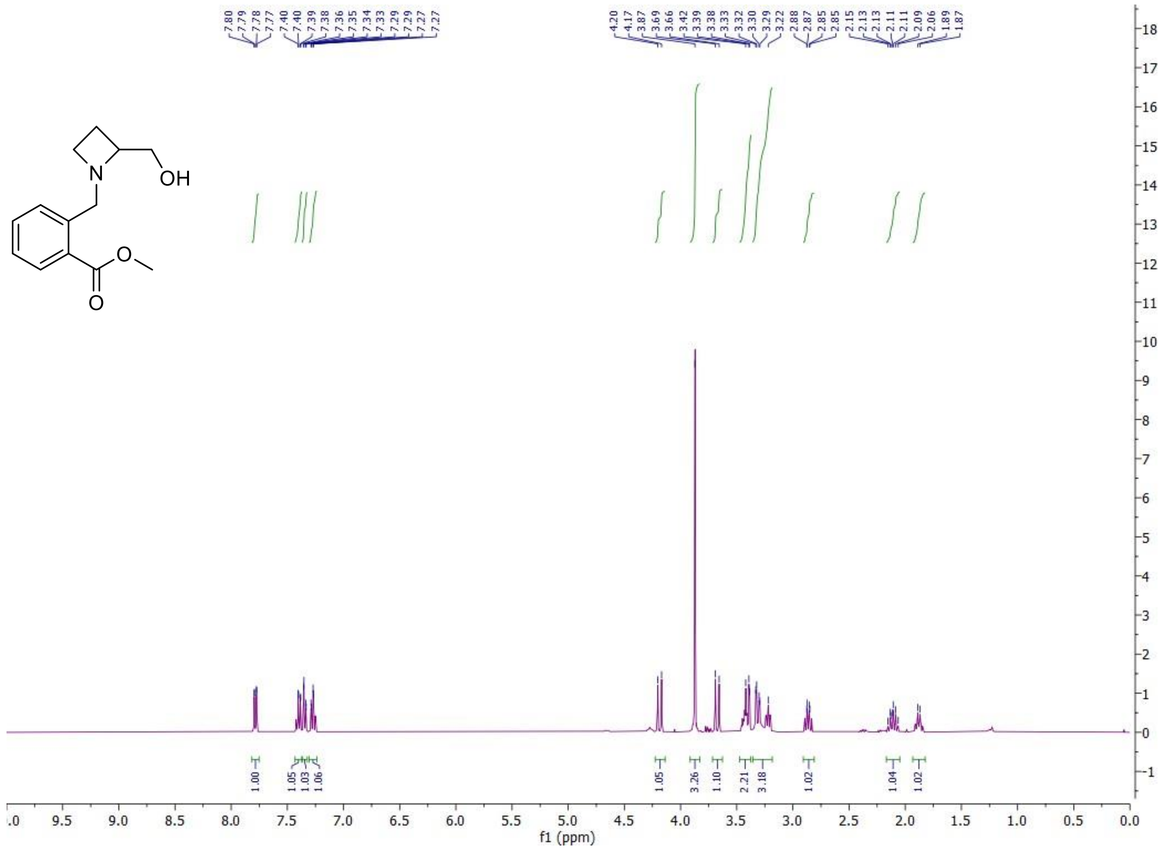

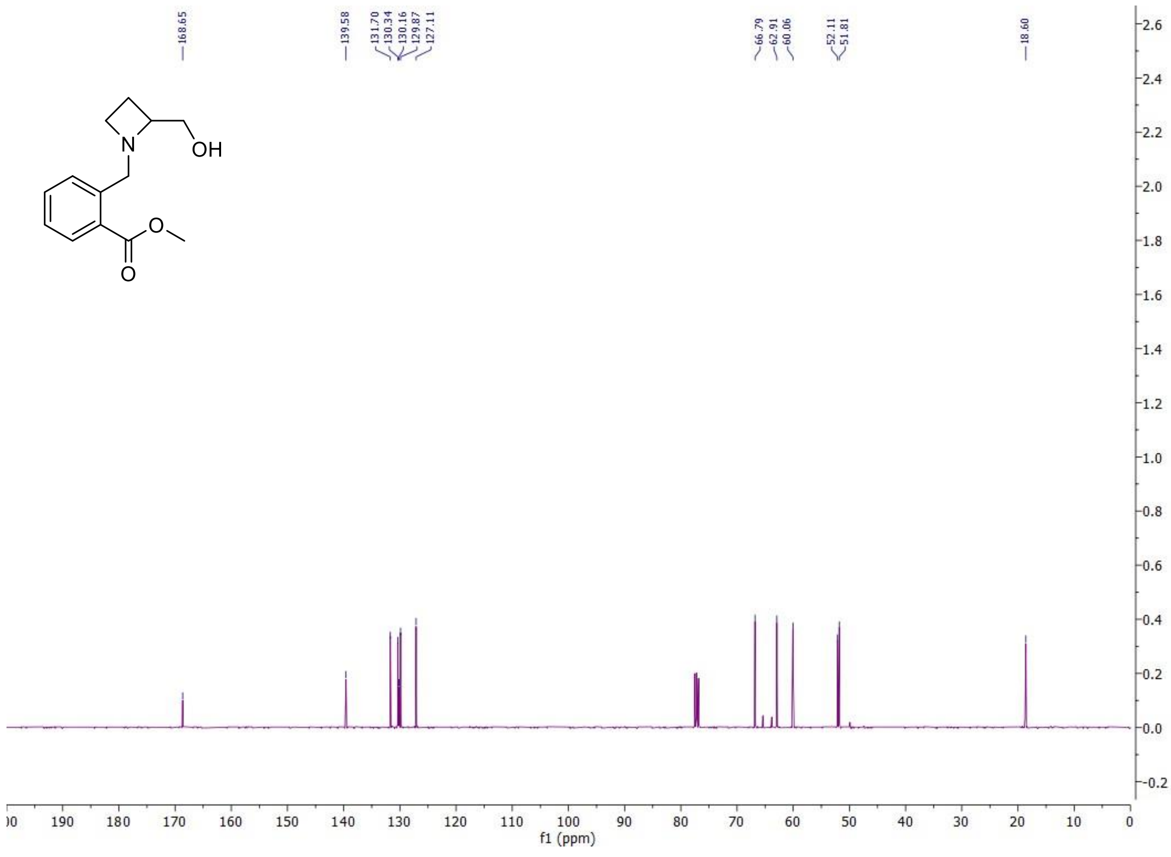

8) References

[1] A. Lawer, J. A. Rossi-Ashton, T. C. Stephens, B. J. Challis, R. G. Epton, J. M. Lynam and W. P. Unsworth *Angew. Chem. Int. Ed.*, **2019**, *58*, 13942–13947

[2] H. Tsukamoto, Y. Kondo *Org. Lett*., **2007**, *9*, 4227–4230.

[3] H. Kroth, N. Sreenivasachary, A. Hamel, P. Benderitter, Y. Varisco, V. Giriens, P. Pagnetti, W. Froestl, A. Pfeifer, A. Muhs *Bioorg. Med. Chem. Lett*. **2016**, *26*, 3330–3335.

[4] M. Vellakkaran, K. Singh, D. Banerjee, *ACS Catal*., 2017, **7**, 8152−8158.

[5] H.Li, S. Shen, C. Zhu, H. Xu, *J. Am. Chem. Soc.*, 2019, **141**, 9415−9421.

[6] S. E. Denmark, S. M. Pham, *J. Org. Chem*., 2003, **68**, 5045–5055.

[7] P. P. Pagare, M. S. Ghatge, Q. Chen, F. N. Musayev, J. Venitz, O. Abdulmalik, Y. Zhang, M. K. Safo, *J. Med. Chem.*, 2020, **63**, 14724–14739.

[8] S. I. Kuwabe, K. E. Torraca and S. L. Buchwald, *J. Am. Chem. Soc.*, 2001, **123**, 12202–12206.

[9] S. I. Kuwabe, K. E. Torraca and S. L. Buchwald, *J. Am. Chem. Soc.*, 2001, **123**, 12202–12206.

[10] Z. Chen and X. Wang, *Org. Biomol. Chem.*, 2017, **15**, 5790–5796.

[11] V. Moas-Héloire, N. Renault, V. Batalha, A. R. Arias, M. Marchivie, S. Yous, N. Deguine, L. Buée, P. Chavatte, D. Blum, L. Lopes, P. Melnyk and L. Agouridas, *Eur. J. Med. Chem*., 2015, **106**, 15–25

[12] J. Zhang, Q. Cai, Q. Gu, X. Shia and S. You, *Chem. Commun.,* 2013, **49**, 7750**–**7752.

[13] K. A. Margrey, A. Levens and D. A. Nicewicz, *Angew. Chem. Int. Ed*., 2017, **56**, 15644 – 15648.

[14] T. Ogiyama, M. Yamaguchi, N. Kurikawa, S. Honzumi, Y. Yamamoto, D. Sugiyama, H. Takakusa and S. Inoue, *Bioorg. Med. Chem*., 2017, **25**, 2234–2243.

[15] P. H. Lee, S. W. Lee, D. Seomoon, *Org. Lett.* 2003, **5**, 4963–4966.

[16] L. Shi, L. Hu, J. Wang, X. Cao and H. Gu, *Org. Lett.*, 2012, **14**, 1876–1879.

[17] L. S. Hamachi, H. Yang, I. Jen-La Plante, N. Saenz, K. Qian, M. P. Campos, G. T. Cleveland, I. Rreza, A. Oza, W. Walravens, E. M. Chan, Z. Hens, A. C. Crowther and J. S. Owen, *Chem. Sci.*, 2019, **10**, 6539–6552.

[18] A. S. Ogunlaja, W. Chidawanyika, E. Antunes, M. A. Fernandes, T. Nyokong, N. Torto and Z. R. Tshentu, *Dalton Trans*., 2012, **41**, 13908–13918.

[19] L. S. Hamachi, H. Yang, I. Jen-La Plante, N. Saenz, K. Qian, M. P. Campos, G. T. Cleveland, I. Rreza, A. Oza, W. Walravens, E. M. Chan, Z. Hens, A. C. Crowther and J. S. Owen, *Chem. Sci.*, 2019, **10**, 6539–6552

[20] K. V. Raghavendra Rao, N. Caiveau, R. David, I. Shalayel, A. Milet and Y. Vallée, *Eur. J. Org. Chem*., 2015, **28**, 6125–6129.

[21] N. Ogawa, S. Furukawa, Y. Kosugi, T. Takazawaa and N. Kanomata, *Chem. Commun*., 2020, **56**, 12917–12920.

[22] PCModel 10.075, K. E. Gilbert, Serena Software, Bloomington IN, 2020.

[23] T. A. Halgren, *J. Comput. Chem.*, 1996, **17**, 490–519.

[24] T. A. Halgren, *J. Comput. Chem.*, 1996, **17**, 520–552.

[25] T. A. Halgren, *J. Comput. Chem.*, 1996, **17**, 553–586.

[26] T. A. Halgren and R. B. Nachbar, *J. Comput. Chem.*, 1996, **17**, 587–615.

[27] T. A. Halgren, *J. Comput. Chem.*, 1996, **17**, 616–641.

[28] Gaussian 16, Revision C.02, M. J. Frisch, G. W. Trucks, H. B. Schlegel, G. E. Scuseria, M. A. Robb, J. R. Cheeseman, G. Scalmani, V. Barone, G. A. Petersson, H. Nakatsuji, X. Li, M. Caricato, A. V. Marenich, J. Bloino, B. G. Janesko, R. Gomperts, B. Mennucci, H. P. Hratchian, J. V. Ortiz, A. F. Izmaylov, J. L. Sonnenberg, D. Williams-Young, F. Ding, F. Lipparini, F. Egidi, J. Goings, B. Peng, A. Petrone, T. Henderson, D. Ranasinghe, V. G. Zakrzewski, J. Gao, N. Rega, G. Zheng, W. Liang, M. Hada, M. Ehara, K. Toyota, R. Fukuda, J. Hasegawa, M. Ishida, T. Nakajima, Y. Honda, O. Kitao, H. Nakai, T. Vreven, K. Throssell, J. A. Montgomery, Jr., J. E. Peralta, F. Ogliaro, M. J. Bearpark, J. J. Heyd, E. N. Brothers, K. N. Kudin, V. N. Staroverov, T. A. Keith, R. Kobayashi, J. Normand, K. Raghavachari, A. P. Rendell, J. C. Burant, S. S. Iyengar, J. Tomasi, M. Cossi, J. M. Millam, M. Klene, C. Adamo, R. Cammi, J. W. Ochterski, R. L. Martin, K. Morokuma, O. Farkas, J. B. Foresman, and D. J. Fox, Gaussian, Inc., Wallingford CT, 2019.

[29] A. D. Becke, *J. Chem. Phys.*, 1993, **98**, 5648–5652.

[30] A. D. Becke, *J. Chem. Phys.*, 1992, **97**, 9173–9177.

[31] P. C. Hariharan and J. A. Pople, *Theor. Chim. Acta*, 1973, **28**, 213–222.

[32] M. M. Francl, W. J. Pietro, W. J. Hehre, J. S. Binkley, M. S. Gordon, D. J. DeFrees and J. A. Pople, *J. Chem. Phys.*, 1982, **77**, 3654–3665.

[33] C. Lee, W. Yang and R. G. Parr, *Phys. Rev. B*, 1988, **37**, 785–789.
